# Variants in the Mitochondrial Genome Sequence of *Rhyzopertha dominica* (Fabricius) (Coleoptera: Bostrycidae)

**DOI:** 10.3390/insects12050387

**Published:** 2021-04-27

**Authors:** Lindsey C. Perkin, Timothy P. L. Smith, Brenda Oppert

**Affiliations:** 1 USDA-ARS, Southern Plains Agricultural Research Center, College Station, TX 77845, USA; lindsey.perkin@usda.gov; 2 USDA-ARS, U.S. Meat Animal Research Center, Clay Center, NE 68933, USA; tim.smith2@usda.gov; 3 USDA-ARS, Center for Grain and Animal Health Research, Manhattan, KS 66502, USA

**Keywords:** insect mitochondria, *Rhyzopertha dominica*, lesser grain borer, phosphine resistance, storage insects, insect control

## Abstract

**Simple Summary:**

The lesser grain borer damages grains in storage worldwide. The major control method for this beetle is phosphine fumigation, but the increase in resistant populations has led to a loss in phosphine efficacy. Insect mitochondria are the major source of energy, and some phosphine-resistant insects have reduced energy production. Therefore, we want to understand whether changes in the mitochondrial genome may promote phosphine resistance in insects, but we need an accurate mitogenome sequence and annotation. We extracted and sequenced genomic DNA from a laboratory colony of the lesser grain borer and assembled and annotated the mitochondrial genome. The mitochondrial genome sequence was similar in structure to other insect mitochondria and encoded typical mitochondrial genes. We compared our predicted mitochondrial genome sequence to that of another lesser grain borer strain from Jingziguan (China). While there was mostly agreement among the two sequences, the data will be used to determine if key differences may suggest mutations in the two populations related to phosphine control pressure. However, differences also could be the result in different genome sequences and interpretations. The data will be useful as a research tool to examine the expression of mitochondrial genes in phosphine susceptible and -resistant insect populations.

**Abstract:**

The lesser grain borer, *Rhyzopertha dominica*, is a coleopteran pest of stored grains and is mainly controlled by phosphine fumigation, but the increase in phosphine-resistant populations threatens efficacy. Some phosphine-resistant insects have reduced respiration, and thus studying the mitochondrial genome may provide additional information regarding resistance. Genomic DNA from an inbred laboratory strain of *R. dominica* was extracted and sequenced with both short (Illumina) and long (Pacific Biosciences) read technologies for whole genome sequence assembly and annotation. Short read sequences were assembled and annotated by open software to identify mitochondrial sequences, and the assembled sequence was manually annotated and verified by long read sequences. The mitochondrial genome sequence for *R. dominica* had a total length of 15,724 bp and encoded 22 *trna* genes, 2 *rRNA* genes, 13 protein coding genes (7 *nad* subunits, 3 *cox*, 2 *atp*, and 1 *cytB*), flanked by a long control region. We compared our predicted mitochondrial genome to that of another from a *R. dominica* strain from Jingziguan (China). While there was mostly agreement between the two assemblies, key differences will be further examined to determine if mutations in populations are related to insecticide control pressure, mainly that of phosphine. Differences in sequence data, assembly, and annotation also may result in different genome interpretations.

## 1. Introduction

The lesser grain borer, *Rhyzopertha dominica* (Fabricius) (Coleoptera: Bostrycidae), is a major pest of stored grains and grain products [1]. Phosphine fumigation is a major control method used in the management of infestations of stored product pests including *R. dominica*. Phosphine is a cost-effective fumigant, and the low cost has increased its usage, resulting in the selection and propagation of resistant populations [2].

Two nuclear genes have been identified in *R. dominica*, *rph1* and *rph2*, that act synergistically to increase phosphine resistance when individuals carry both mutated genes [3,4]. Significant phosphine resistance occurs in insects with mutations in *rph2*, which encodes dihydrolipoamide dehydrogenase (DLD) [5]. The second gene, *rph1*, encodes cytochrome b5 fatty acid desaturase [6], and higher levels of resistance are found in insects with mutations in both genes.

Originally it was hypothesized that phosphine resistance was due to mutations in the mitochondrial genome [7]. The apparent association of altered respiration in phosphine-resistant insects, as has been reported in phosphine-resistant stored product insects including *R. dominica* [8], suggests that mitochondria also may be involved in resistance. A gene expression study indicated that genes related to mitochondrial functions were differentially expressed in phosphine-resistant *Tribolium castaneum* (Herbst) (Coleoptera: Tenebrionidae) [9].

Mitochondria are cellular organelles in eukaryotes that produce energy through oxidative phosphorylation (reviewed in [10]). Insect mitochondria contain a circular double-stranded DNA genome, mtDNA, that ranges in size from 15,000 to 18,000 bp [11]. Mitochondrial genomes are inherited through the female germline and their transcripts are translated using a modified genetic code [12]. Insect mitochondrial genomes are highly conserved, with 37 coding genes (13 protein-coding, two ribosomal RNA, and 22 transfer RNA genes) and a non-coding control region (CR) for initiating transcription and replication [11,13,14]. Non-protein coding RNA genes include *trna* (designated further with the amino acid that it transports), and *rrn* encoding a small mitochondrial ribosome subunit (*rrnS*) and large subunit (*rrnL*) [7]. Protein-coding genes encode polypeptides that integrate into the electron transfer chain, including complex I (6 subunits of NADH dehydrogenase, *nad*), complex III (cytochrome B subunit, *cytB*), complex IV (3 subunits of cytochrome c oxidase, *cox*), and complex V (2 ATP synthase subunits, *atp*).

During the preparation of this manuscript, the mitochondrial genome of a Chinese *R. dominica* strain was published [15]. The genome contained 15,862 bp with 74.36% AT content, including 13 protein-coding genes, 22 tRNA and two rRNA genes. In this study, we assembled and annotated another mitochondrial genome sequence for *R. dominica* from a different geographical origin. We identify differences between the mtDNA of U.S. and Chinese strains and discuss the potential relationship of the mitochondria and resistance in this stored product insect.

## 2. Materials and Methods

### 2.1. Insect Strain

The original *R. dominica* laboratory strain was collected from infested grain in Eastern Kansas in 1972 and has been reared at the Center for Grain and Animal Health Research in Manhattan, KS. An inbred strain from the laboratory colony of *R. dominica* (LGB Inbred D) was used as the source of genetic material. This strain was inbred from a single pair subculture for 20 generations, from December 2009 to March 2012.

### 2.2. Extraction of Nucleic Acids and Sequencing of the Mitochondrial Genome

For short read sequencing, genomic DNA was extracted from 20 mixed-sex *R. dominica* pupae of the LGB Inbred D strain using Quick-DNA™ Tissue/Insect Miniprep Kit (Zymo Research, Irvine, CA, USA). A portion of the DNA was sheared using a Covaris S220 for 400 base fragments as recommended by the manufacturer (Covaris Inc. Woburn, MA, USA). A library was prepared with the TruSeq^®^ PCR-Free library preparation kit (Illumina Inc., San Diego CA, USA) and sequenced on a MiSeq instrument using a 2 × 300 base paired read v3 reagent kit. Size selection of a portion of the gDNA for long read sequencing was performed with a BluePippin instrument (Sage Science Inc., Beverly, MA, USA) using a 15kb lower cutoff value. Libraries for long read sequencing on the RSII platform were constructed using the SMRTbell™ Template Prep Kit 1.0 as recommended by the manufacturer (Pacific Biosciences, Menlo Park, CA, USA, USA). Four libraries were prepared from the same gDNA and sequenced on sixteen SMRT cells of the RSII using P5/C3 and P6/C4 chemistry (eight cells each).

### 2.3. Assembly and Annotation of the Mitochondrial Genome

A complete *R. dominica* mitochondrial genome was assembled from MiSeq short reads (accession #SRR12638565). The open-source software, NOVOPlasty version 2.6.7 [16], was used to separate the mitochondrial sequences from the nuclear sequences. This software requires only shallow sequence depth of genomic DNA and uses a genome skimming approach to extract the high-copy fraction of mitochondrial DNA from the low-copy fraction of nuclear DNA. A de novo assembly was initiated using the *R. dominica* cytochrome oxidase subunit 1 gene (COI; accession #KM450240). The genome was annotated using the web-based program MITOS version 1.0 [17] and visualized with GenomeVx version 1.0 [18]. Long read data were used for verification of assembled sequences (unpublished). Manual annotation of protein-coding gene sequences was done with Expasy Translate [19], BLAST [20], and Clustal Omega v. 1.2.4 [21]. The G+C content spanning the entire mitochondrial genome was assessed by an online tool (webgenetics.com, accessed on 20th September 2020).

## 3. Results

**Figure 1:**
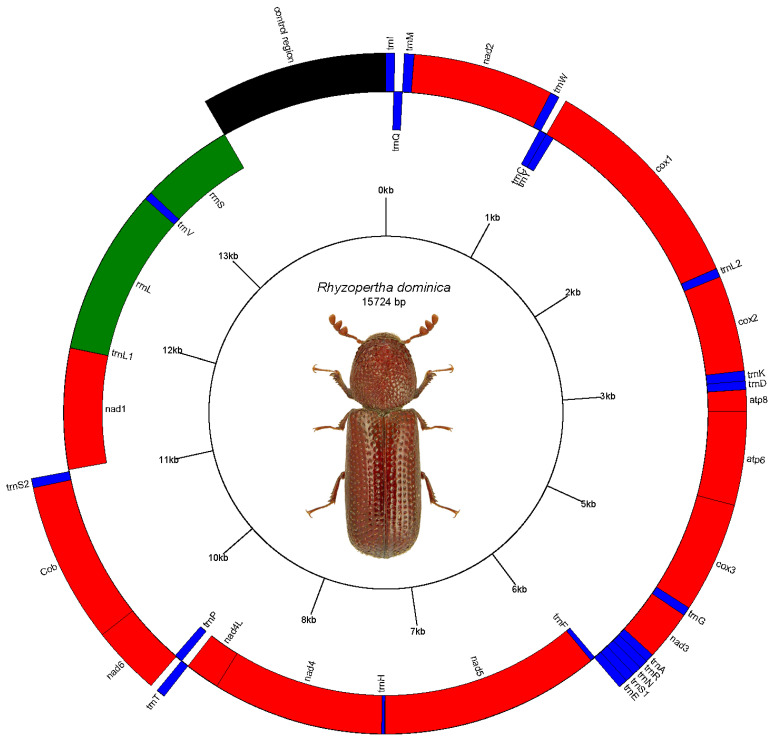
Map of the mitochondrial genome of Rhyzopertha dominica, as predicted by Mitos. Photo used by permission (Lech Borowiec, https://www.coleoptera.org.uk/node/20008, accessed on 24 December 2020).

The short and long read sequencing produced 8.1 and 13.2 billion bases (Gb), respectively, representing approximately 17× and 58× coverage of the beetle genome. MiSeq reads corresponding to the mitochondrial genome were separated from nuclear genomic reads (see Methods), with approximately 0.39% of the 20,951,116 total reads (60,094 reads) representing putative mitochondrial reads. These reads produced a mitochondrial genome assembly (gMT) of the U.S. isolate of R. dominica with a total length of 15,724 bp and G+C content of 25.3% (accession #MW020612). Features of the gMT were annotated to identify 22 tRNA genes, 2 rRNA genes, 13 protein coding genes (7 nad subunits, 3 cox, 2 atp, and 1 cytB), and a non-coding AT-rich control region (1127 bp) (Figure 1). These data were compared to a previously reported gMT sequence of a Chinese isolate of R. dominica [15], which was slightly longer with 15,862 bp and a slightly higher G+C content of 25.6% but containing the same number of tRNA and rRNA genes (Supplemental Figure S1).

### 3.1. RNA Sequences

Genes encoding tRNA were the same in the two R. dominica gMT sequences, although some start codons were variable (Table 1). Single nucleotide variants (SNVs) were found only in trnaM. There was a difference in the directionality of trnaQ, which was on the reverse strand in this assembly but on the forward strand in the assembly from Ouyang et al. [15]. Predictions of tRNA secondary stem loop structures were similar to traditional tRNA (Supplemental Figure S2).

Predicted ribosomal sequences were more variable for rrnL (Table 1). The curated sequence from the Mitos prediction for rrnL had an earlier start (12,379) and stop codon (13,580), resulting in a coding sequence of 1202 vs. 1254 bp in the previous assembly. We used the MITOS prediction for rrnL, as we found that it gave higher BLAST maximum scores and lower e-values (data not shown). The sequences for rrnS only differed by a few nucleotides between the two gMT sequences. Predictions of rRNA secondary structures are provided in Supplemental Figure S3.

### 3.2. Protein Coding Sequences

Comparisons of protein-coding genes and gene products in the two R. dominica gMT sequences identified nonsynonymous substitutions due to SNVs, with the exception of nad4L (Supplemental Figure S4). SNVs in the other nad subunits were A-T and N-D (nad2), M-V (nad3), L-S (nad5), F-C and D-H (nad4), and L-M (nad1) (these reflect the protein predictions from our sequences versus the sequence prediction by Ouyang et al. [15]). SNVs also were found in other coding sequences: E-A (cox1), although we disagreed with the start codon and therefore there is also a difference in the N-terminus; M-T (atp8) and T-A (atp6); and V-I, M-I, M-V (cytB). The validation of these sequences was made by comparing with an alignment of the long-read sequences (Supplemental Figure S5). For every SNV, the long reads supported our predicted sequence by 13–47 long reads.

In some cases, we also found differences in N- and C-termini of predicted protein coding sequences of this assembly compared to that of Ouyang et al. [15] (Supplemental Figure S4). The coding sequences for cox1 were identical, except for a termination codon after lysine. An initiation codon of ATA has been reported for cox1 in Drosophila melanogaster [12]. For cox2, the stop codon was TCA with overlap in adjacent trnk. There also was ovelap with cox3 due to a potential stop codon within the coding sequence for trnG, but the protein coding sequence was highly similar (e^−146^) with cox3 from Dastarcus helophoroides (accession #YP_009049589). For atp8, we agreed with the initiation codon of ATT, but found little support in the NCBI database of other atp8 sequences for the earlier initiation codon, and many atp8 sequences have a MSPL N-terminus (data not shown). The coding sequence for nad5 was longer than the previous sequence, beginning at an earlier start codon but ending at the same stop codon. We retained the isolucene amino acid as the start codon, as well as additional amino acids YLNSL at the C-terminus of other coleopteran nad sequences (i.e., accession #ANJ70442).

The nad1 nucleotide sequences had high variability in a region where the amino acid prediction was YMNLF (Supplemental Figure S4). This region has multiple polyA and polyAT repeats, and therefore the predictions are less certain. However, we found 18 long reads to support our predicted nucleotide sequence for nad1 (Supplemental Figure S5). Our N-terminus sequence for nad1 also was slightly different than that of Ouyang et al. [15], also supported by long read data.

### 3.3. Control Region

The predicted control regions for the two R. dominica mitochondrial sequences were similar in the nucleotide start site (14,412 vs. 14,410) (Table 1). However, our control region length (1312 bp) was shorter than that of the comparative sequence (1449 bp) due to an additional 135 nucleotides after 14,714 bp (numbering from our sequence). There were two long reads that supported the previous sequence (Supplemental Figure S5), but the MITOS prediction did not include those 135 nucleotides, and a BLAST did not identify similar sequences in other organisms. In addition, the control regions in the two mitochondrial genome sequences had seventeen SNVs, with more variability than in other regions.

In evaluating the G+C content of the R. dominica gMT, we noticed a large increase in G+C content at the beginning of the predicted control region around 14,420 bp, followed by a sharp drop to an A+T-rich region (Supplementary Figure S6). The additional 135 nts in the control region of the previous R. dominica gMT sequence is at the transition of G+C- to A+T-rich sequences. The region with high A+T content was about 625 bp (14,850–15,475). A span of 21 polyTs starting at 14,930 nt in the A+T enriched sequence may suggest an origin of replication site at the end of the polyT run.

## 4. Discussion

We assembled and annotated mitochondrial gene sequences from *R. dominica* from short read data and used long read data to further validate sequences. The focus of our study was to understand the *R. dominica* mitochondrial genome in a laboratory strain from a control perspective, whereas the previous mitochondrial genome sequence of a strain from Jingziguan, China, was presented from an evolutionary biology interest [15]. We identified differences among the two assemblies and suggest that key differences from SNVs may result from nonsynonymous mutations in the two populations as a consequence of insecticide control pressure, mainly phosphine. Additionally, differences in sequence data, assembly, and annotation may result in different genome interpretations. We note that our mitochondrial genome sequence was from a highly inbred strain, and the low heterozygosity in our strain was supported by the long-read data. However, this strain has not been evaluated for resistance to phosphine.

In our assembly, *trnaQ* was in the reverse orientation but was forward in the assembly from Ouyang et al. [15]. The transcription orientation of mitochondrial *trnas* are highly conserved, and *trnaQ* generally has a positive orientation, but the orientation of *trnaQ* (-) in *T. castaneum* is an exception [22,23]. Thus, our annotation of the orientation of *trnaQ* (-) in *R. dominica*, as was predicted by MITOS, aligns with that exception.

Differences between the mitochondrial genome sequences of the two *R. dominica* strains occurred in protein-coding sequences, both in SNVs as well as interpretation of N- and C-termini. SNVs were found in all protein coding sequences from the two genome predictions, with the exception of *nad4L* and *nad6*. Differences were nonsynonymous and often resulted in amino acids with different charges or hydrophobicity that could alter the secondary structure and interactions of the subunits. SNVs may represent candidate variants that were selected by phosphine exposure, a hypothesis which could be tested in future studies by low-coverage sequencing of individuals (sufficient for genotyping of mitochondrial genomes) from phosphine-treated and phosphine-naïve locations. We also noted differences in N- and/or C-termini in *cox2*/*cox3*, *atp8*, and *nad3*/*nad5* that were likely due to differences in annotation of the two gMT. High variability in the region of *nad1* encoding YMNLF may suggest that the gene encoding that subunit is undergoing a higher rate of mutation. However, the poly A and AT sequences in that region also may indicate difficulty in assembly and accuracy in the sequence data.

The non-coding control region contains the longest and most variable sequence of the mitochondria [14]. Our annotated control region in the *R. dominica* gMT lacked a 135 nt span that was found in the sequence of Ouyang et al. [15]. The 135 nt sequence bordered the transition of a G+C enriched (>50%) sequence of approximately 250 bp to an A+T enriched sequence of approximately 625 bp. Since both gMT sequences contained this enriched G+C content, it is likely that the sequence is not an artifact, but the functional significance remains to be determined. The invertebrate control region is typified by an A+T-rich region that contains an origin of replication and transcription (Cameron, 2014). The origin of replication in *T. castaneum* was identified starting after a run of 17 polyT nt [24]. In our *R. dominica* gMT sequence, there was a span of 21 polyTs located in the middle of the A+T enriched region which also is likely an origin of replication site. However, there was no overlap in alignment of sequences from the gMT of *R.dominica* and *T. castaneum* due to high sequence variability.

A number of single point mutations in mitochondrial genes were found in phosphine-resistant *Sitophilus oryzae* [25]. In that paper, the authors reflected our earlier observations in *T. castaneum* [9], in that phosphine-resistant insects minimize energy production as a mechanism to avoid phosphine toxicity. In fact, in *R. dominica* adults, many mitochondrial genes are increased in expression when phosphine-susceptible insects are exposed to phosphine, whereas those genes decrease in expression in phosphine-resistant insects during phosphine exposure (unpublished data). It is not clear if the expression of mitochondrial genes is affected by mutations in previously described resistance loci [5,6]. Therefore, understanding mitochondrial genome sequence and structure, as well as gene expression, in phosphine-susceptible and -resistant insects could provide new insights into the control of resistant populations.

## 5. Conclusions

We sequenced, assembled, and annotated the mitochondrial genome of an inbred laboratory strain of a U.S. population of *R. dominica*, a problematic pest of stored grains. We compared this mitochondrial genome sequence to that of a recently published sequence from a strain from Jingziguan, China (Ouyang et al., 2019). Although the two assemblies were similar, key differences will be evaluated in the context of phosphine resistance. Additionally, differences in sequence data, assembly, and annotation may result in different genome interpretations.

## Figures and Tables

**Table 1:** Comparison of Rhyzopertha dominica mitochondrial genome sequence MW020612 (this paper) and NC_042820.1. Start/stop are provided for protein-coding sequences. Bolded entries are differences among the two sequences.

Gene	Strand	Feature	Start, Stop	NC# (This Paper)			NC_042820		
				Start	Stop	Length (bp)	Start	Stop	Length (bp)
*trnI*	+	tRNA		1	63	63	0	63	63
*trnQ*	−	tRNA		61	129	69	60	129	69
*trnM*	+	tRNA		129	197	69	128	197	69
*nad2*	+	CDS	ATA, TAA	198	1190	993	197	1190	993
*trnW*	+	tRNA		1189	1253	65	1188	1253	65
*trnC*	−	tRNA		1246	1306	61	1245	1306	61
*trnY*	−	tRNA		1306	1368	63	1305	1368	63
***cox1***	+	**CDS**	**ATA**, **TAA**	**1370**	**2898**	**1529**	**1369**	**2900**	**1531**
*trnL2*	+	tRNA		2901	2962	62	2900	2962	62
***cox2***	+	**CDS**	**ATA**, **TCA**	**2963**	**3640**	**678**	**2962**	**3638**	**676**
*trnK*	+	tRNA		3639	3709	71	3638	3709	71
*trnD*	+	tRNA		3709	3770	62	3708	3770	62
*atp8*	+	CDS	ATT, TAA	3771	3926	156	3770	3926	156
*atp6*	+	CDS	ATG, TAA	3920	4585	666	3919	4585	666
***cox3***	+	**CDS**	**ATG, TTA**	**4585**	**5373**	**789**	**4584**	**5368**	**784**
*trnG*	+	tRNA		5369	5430	62	5368	5430	62
***nad3***	+	**CDS**	**ATA, TAG**	**5431**	**5784**	**354**	**5430**	**5784**	**354**
*trnA*	+	tRNA		5783	5844	62	5782	5844	62
*trnR*	+	tRNA		5844	5908	65	5843	5908	65
*trnN*	+	tRNA		5908	5971	64	5907	5971	64
*trnS*	+	tRNA		5971	6037	67	5970	6037	67
*trnE*	+	tRNA		6038	6101	64	6037	6101	64
*trnF*	−	tRNA		6100	6163	64	6099	6163	64
***nad5***	−	**CDS**	**ATT, TAA**	**6146**	**7871**	**1726**	**6163**	**7871**	**1708**
*trnH*	−	tRNA		7872	7934	63	7871	7934	63
***nad4***	−	**CDS**	**ATG, TTA**	**7936**	**9252**	**1317**	**7934**	**9252**	**1318**
*nad4L*	−	CDS	ATG, TAA	9246	9518	273	9245	9518	273
*trnT*	+	tRNA		9521	9582	62	9520	9582	62
*trnP*	−	tRNA		9583	9645	63	9582	9645	63
*nad6*	+	CDS	ATT, TAA	9647	10,129	483	9646	10,129	483
*cytb*	+	CDS	ATG, TAG	10,129	11,268	1140	10,128	11,268	1140
*trnS2*	+	tRNA		11,267	11,332	66	11,266	11,332	66
***nad1***	−	**CDS**	**ATA, TAG**	**11,350**	**12,303**	**954**	**11,349**	**12,300**	**951**
*trnL1*	−	tRNA		12,301	12,362	62	12,300	12,362	62
***rrnL***	−	**rRNA**		**12,379**	**13,580**	**1202**	**12,362**	**13,616**	**1254**
***trnV***	−	**tRNA**		**13,618**	**13,679**	**62**	**13,616**	**13,678**	**62**
***rrnS***	−	**rRNA**		**13,680**	**14,411**	**732**	**13,678**	**14,410**	**732**
**control region**	+			**14,412**	**15,724**	**1312**	**14,410**	**15,859**	**1449**

## Data Availability

The data presented in this study are openly available at the National Center for Biotechnology Information under accession numbers SRR12638565 and MW020612.

## Supplementary Materials

The following are available online at https://www.mdpi.com/article/10.3390/insects12050387/s1, Figure S1: Alignment of the predicted sequence (accession #MW020612; top sequence with Rdo_MitoGenome) of the *Rhyzopertha dominica* mitochondrial genome and that predicted by Ouyang et al. [15] (accession #NC_042820), Figure S2: Predicted stem-loop structure for the *trna* genes from the mitochondrial genome of *Rhyzopertha dominica*, Figure S3: Predicted structures of rrna from the mitochondrial genome of *Rhyzopertha dominica*. (a), *rrnL*; (b), *rrnS*, Figure S4: Alignment of protein coding sequences (accession #MW020612; top sequence with Seq# in each alignment) of the *Rhyzopertha dominica* mitochondrial genome and that predicted by Ouyang et al. [15] (accession #NC_042820), Figure S5: Alignment of long reads (PacBio, ml#) to the predicted sequence (accession #MW020612; Rdo_MitoGenome) of the *Rhyzopertha dominica* mitochondrial genome and that predicted by Ouyang et al. [15] (accession #NC_042820), Figure S6: Plot of GC content (%) versus nt in the predicted sequence (accession #MW020612) of the *Rhyzopertha dominica* mitochondrial genome.
