# Supplementary material for: Variants in the Mitochondrial Genome Sequence of Rhyzopertha dominica (Fabricius) (Coleoptera: Bostrycidae)
Source: Insects. 2021 Apr 27;12(5):387. doi: 10.3390/insects12050387 (PMC8146127; doi:10.3390/insects12050387)

Figure S2. Predicted stem-loop structure for the *trna* genes from the mitochondrial genome of *Rhyzopertha dominica.*

a. trnA

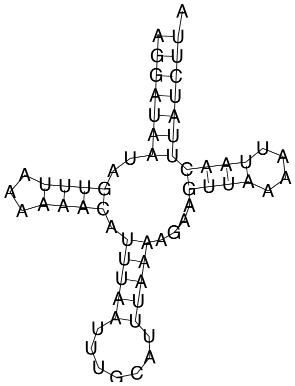

b. trnC

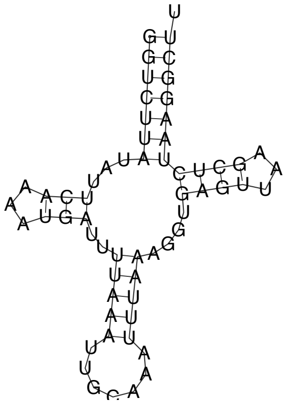

c. trnD

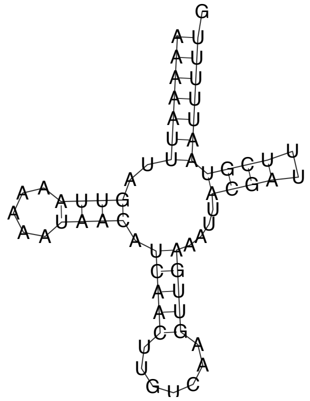

d. trnE

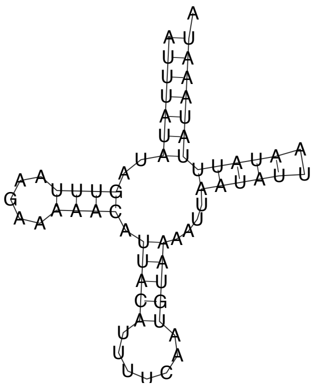

e. trnF

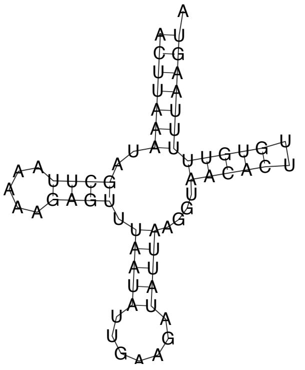

f. trnH

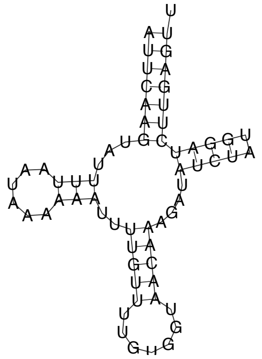

g. trnI

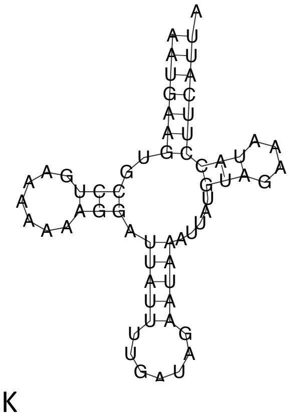

h. trnK

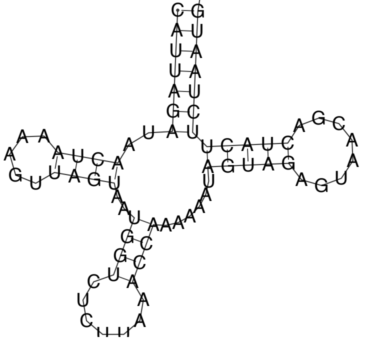

i. trnL1

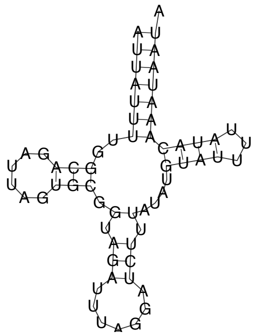

j. trnL2

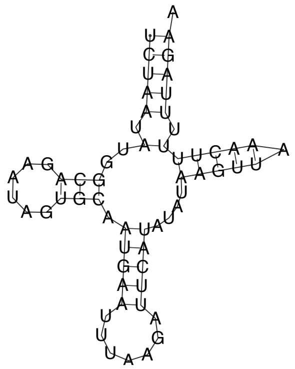

k. trnM

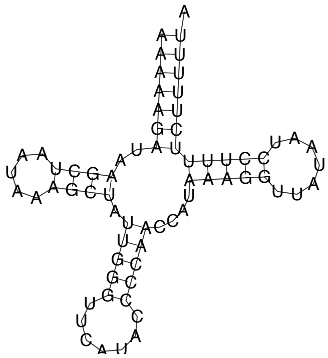

l. trnN

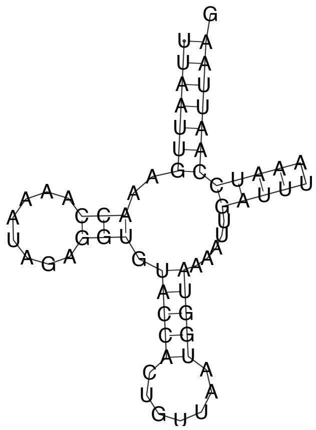

m. trnP

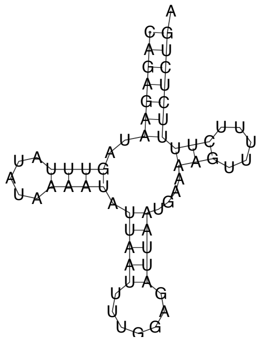

n. trnQ

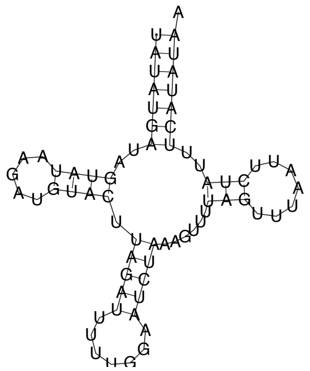

o. trnR

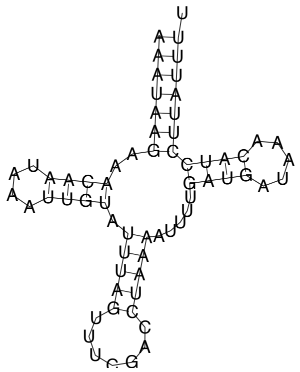

p. trnS1

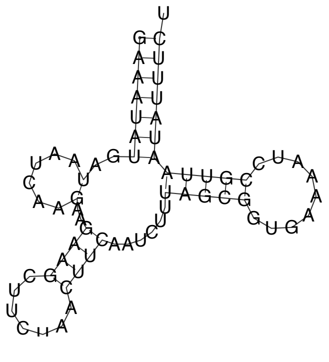

q. trnS2

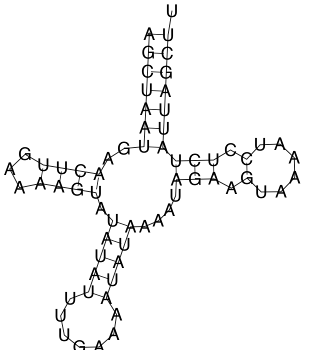

r. trnT

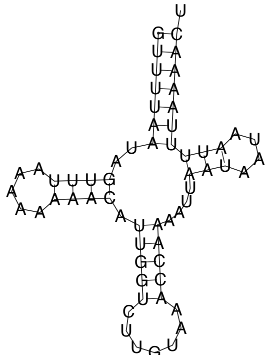

s. trnV

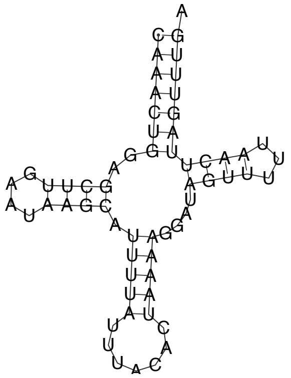

t. trnW

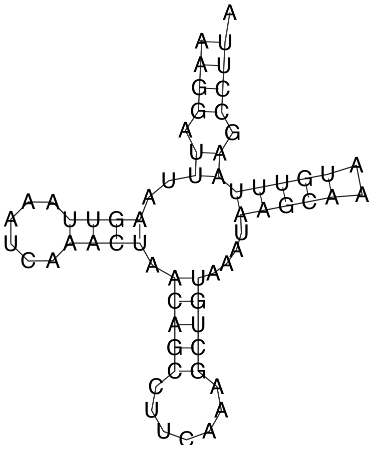

u. trnY

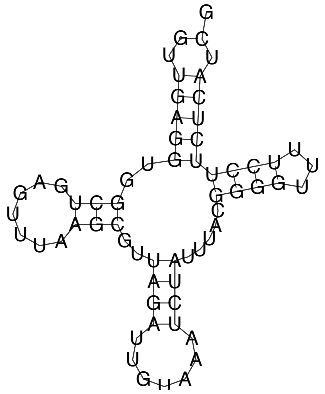

Supplement: Supplementary file 1 [file insects-12-00387-s001.zip › insects-1191702-s/Figure S2.pdf]
