# Supplementary material for: Variants in the Mitochondrial Genome Sequence of Rhyzopertha dominica (Fabricius) (Coleoptera: Bostrycidae)
Source: Insects. 2021 Apr 27;12(5):387. doi: 10.3390/insects12050387 (PMC8146127; doi:10.3390/insects12050387)

Figure S3. Predicted structures of rrna from the mitochondrial genome of *Rhyzopertha dominica*. a), *rrnL*; b), *rrnS*.

a. Predicted structure of *R. dominica rrnL.*

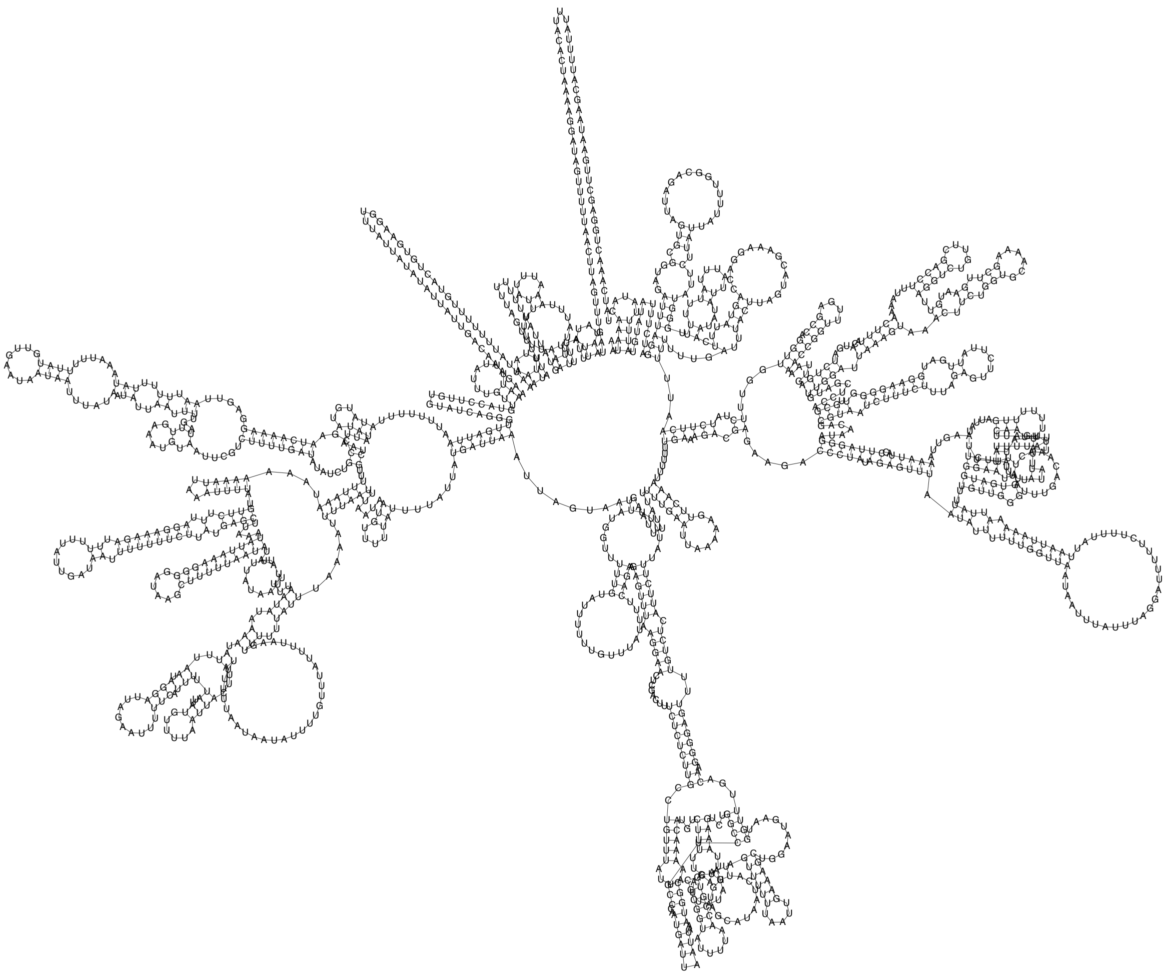

b. Predicted structure of *R. dominica rrnS.*

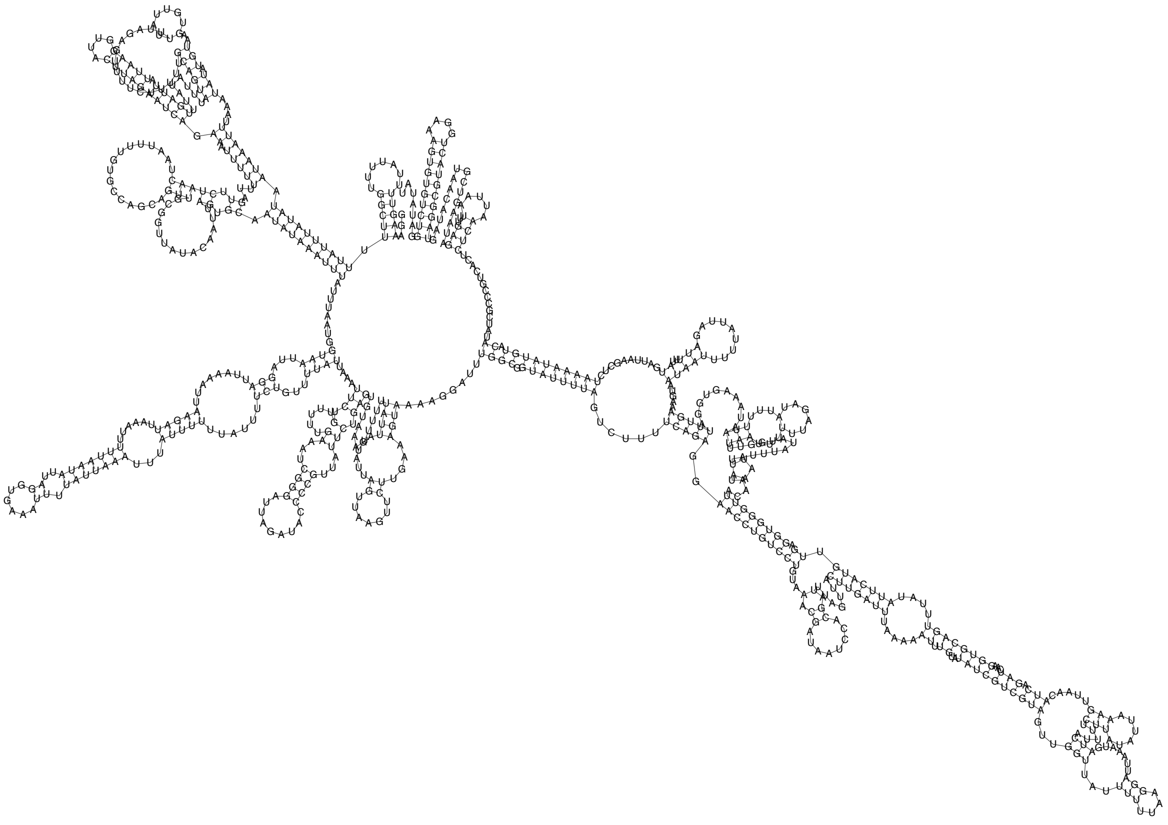

Supplement: Supplementary file 1 [file insects-12-00387-s001.zip › insects-1191702-s/Figure S3.pdf]
