# Supplementary material for: Variants in the Mitochondrial Genome Sequence of Rhyzopertha dominica (Fabricius) (Coleoptera: Bostrycidae)
Source: Insects. 2021 Apr 27;12(5):387. doi: 10.3390/insects12050387 (PMC8146127; doi:10.3390/insects12050387)
Supplement: Supplementary file 1 [file insects-12-00387-s001.zip › insects-1191702-s/Figure S4 edited.pdf]

Figure S4. Alignment of protein coding sequences of the *Rhyzopertha dominica* mitochondrial genome from this paper (top sequence) and that predicted by Ouyang et al. [15] (lower sequence). a), *nad2*; b), *cox1*; c), *cox2*; d), *atp8*; e), *atp6*; f), *cox3*; g), *nad3*; h), *nad5*; i), *nad4*; j), *cytB*; k), *nad1*. Amino acid differences due to SNVs are notated with turquoise shading.

a. nad2

```
Seq4            MIKLSKISFTITMMIGSMITISSYTWMGMWMGLEINLLSIIPLLTTKKNIYSTEAAIKYF 60
QCI56356.1      MIKLSKISFTITMMIGSMITISSYTWMGMWMGLEINLLSIIPLLTTKKNIYSTEAAIKYF 60
                ************************************************************

Seq4            ITQALASSILLMSILILSTNLINQKSAMMILYSSLMTKMGTAPFHFWFPEIIEGLDWMNC 120
QCI56356.1      ITQALTSSILLMSILILSTNLINQKSAMMILYSSLMTKMGTAPFHFWFPEIIEGLDWMNC 120
                *****:******************************************************

Seq4            LIMMTWQKIAPAMILMSSMNMNTFIYITITSSMLISGIMGVNQTSLRKIMAYSSINHMAW 180
QCI56356.1      LIMMTWQKIAPAMILMSSMNMNTFIYITITSSMLISGIMGVNQTSLRKIMAYSSINHMAW 180
                ************************************************************

Seq4            MMASMNNEKIFYIYLSIYITMSSIFIFTLKSNNSLYIYQMMTSNQSPIIKKTFIINLLSM 240
QCI56356.1      MMASMNNEKIFYIYLSIYITMSSIFIFTLKSNNSLYIYQMMTSNQSPIIKKTFIINLLSM 240
                ************************************************************

Seq4            GGLPPLLGFFPKWMVINSMIQSNNLILSTFMVMTTLLTLYFYMQISMPILTMYKMKMKWS 300
QCI56356.1      GGLPPLLGFFPKWMVINSMIQSNDLILSTFMVMTTLLTLYFYMQISMPILTMYKMKMKWS 300
                ***********************:************************************

Seq4            KKNPQQFFMNKMNMINLLSIPVCTIWFNFY  330
QCI56356.1      KKNPQQFFMNKMNMINLLSIPVCTIWFNFY  330
                ******************************
```

b. cox1

```
Seq8                NKWLYSTNHKDIGTLYFIFGIWSGMVGTALSMLIRSELGNPGALIGDDQIYNVIVTAHAF      36
YP_009655010.1      NKWLYSTNHKDIGTLYFIFGIWSGMVGTALSMLIRSELGNPGALIGDDQIYNVIVTAHAF      60
                    ************************************************************

Seq8                IMIFFMVMPMMIGGFGNWLVPLMIGAPDMAFPRMNNMSFWLLPPSLTLLLTSSIVETGAG      96
YP_009655010.1      IMIFFMVMPMMIGGFGNWLVPLMIGAPDMAFPRMNNMSFWLLPPSLTLLLTSSIVETGAG      120
                    ************************************************************

Seq8                TGWTVYPPLSNNTAHSGASVDLAIFSLHLAGISSILGAVNFITTIINMRPKGMTPERIPL      156
YP_009655010.1      TGWTVYPPLSNNTAHSGASVDLAIFSLHLAGISSILGAVNFITTIINMRPKGMTPERIPL      180
                    ************************************************************

Seq8                FVWSVGITALLLLLSLPVLAGAITMLLTDRNLNTSFFDPAGGGDPILYQHLFWFFGHPEV      216
YP_009655010.1      FVWSVGITALLLLLSLPVLAGAITMLLTDRNLNTSFFDPAGGGDPILYQHLFWFFGHPEV      240
                    ************************************************************

Seq8                YILILPGFGMISHIISHESGKKETFGSLGMIYAMMAIGLLGFIVWAHHMFTVGMDVDTRA      276
YP_009655010.1      YILILPGFGMISHIISHESGKKETFGSLGMIYAMMAIGLLGFIVWAHHMFTVGMDVDTRA      300
                    ************************************************************

Seq8                YFTSATMIIAVPTGIKVFSWLATLHGTQMNYSPSMMWSLGFVFLFTVGGLTGVVLANSSI      336
YP_009655010.1      YFTSATMIIAVPTGIKVFSWLATLHGTQMNYSPSMMWSLGFVFLFTVGGLTGVVLANSSI      360
                    ************************************************************
Seq8                DIILHDTYYVVAHFHYVLSMGAVFAIMAGIIQWFPLFTGLTLNEKMLKIQFLIMFMGVNI      396
YP_009655010.1      DIILHDTYYVVAHFHYVLSMGAVFAIMAGIIQWFPLFTGLTLNEKMLKIQFLIMFMGVNI      420
                    ************************************************************

Seq8                TFFPQHFLGLSGMPRRYSDYPDAYLTWNIISSIGSMMSTVSIIFMSFIMWESLSSKRKNI      456
YP_009655010.1      TFFPQHFLGLSGMPRRYSDYPDAYLTWNIISSIGSMMSTVSIIFMSFIMWESLSSKRKNI      480
                    ************************************************************

Seq8                SNNQLSSAIEWLQNSPPEEHSYSELPILAKF       487
YP_009655010.1      SNNQLSSAIEWLQNSPPAEHSYSELPILAK-       510
                    ***************** ************
```

c. cox2

```
Seq10             MATWKMTSMSNSNSPAMEQLTFFHDHTMTVLIMITILVGYLMSTLFTNKLTYRFLLEGQT     60
YP_009655011.1    MATWKMTSMSNSNSPAMEQLTFFHDHTMTVLIMITILVGYLMSTLFTNKLTYRFLLEGQT     60
                  ************************************************************

Seq10             IEMIWTILPAITLIFIALPSLQILYTLDEIINPSMSVKSIGHQWYWSYEYSDFKKTEFDS     120
YP_009655011.1    IEMIWTILPAITLIFIALPSLQILYTLDEIINPSMSVKSIGHQWYWSYEYSDFKKTEFDS     120
                  ************************************************************

Seq10             YMKSPNEIKNQEFRLLDVDNRMTLPMKTKIRLLVSSTDVIHSWTIPSLGVKIDATPGRLN     180
YP_009655011.1    YMKSPNEIKNQEFRLLDVDNRMTLPMKTKIRLLVSSTDVIHSWTIPSLGVKIDATPGRLN     180
                  ************************************************************

Seq10             QASMFIINPGLMYGQCSEICGANHSFMPIVVESITPNKFIEWIKNS 229
YP_009655011.1    QASMFIINPGLMYGQCSEICGANHSFMPIVVESITPNKFIEWIKN-          225
                  *********************************************
```

d. atp8

```
Seq13             ---MSPLNWMTLFIFFTITLVIMSTMNFYNYKPEPLKGEKTISMSKKNWKW     48
YP_009655012.1    MPQMSPLNWTTLFIFFTITLVIMSTMNFYNYKPEPLKGEKTISMSKKNWKW     51
                     ****** *****************************************
```

e. atp6

```
Seq14             MMSNLFSSFDPSTQILSLNWLSSLIILMIMPMNFWLIPSRMSLLWMKISKKLHNEFKILI     60
YP_009655013.1    MMSNLFSSFDPSTQILSLNWLSSLIILMIMPMNFWLIPSRMSLLWMKISKKLHNEFKILI     60
                  ************************************************************

Seq14             GKNNGSSLMFTSLLLLIMINNFMGLFPYIFTSTSHLTMTLTLALPLWTSFMIYGWFNNTI     120
YP_009655013.1    GKNNGSSLMFTSLLLLIMINNFMGLFPYIFTSTSHLTMTLTLALPLWTSFMIYGWFNNTI     120
                  ************************************************************

Seq14             SMLAHLVPQGTPPILMPFMVCIETISNIIRPGTLAIRLTANMIAGHLLLTLLGNTGAGIS     180
YP_009655013.1    SMLAHLVPQGTPPILMPFMVCIETISNIIRPGALAIRLTANMIAGHLLLTLLGNTGAGIS     180
                  ********************************:***************************

Seq14             SILVSILILTQILLLVLESAVAIIQSYVFAILSTLYSSEVN     221
YP_009655013.1    SILVSILILTQILLLVLESAVAIIQSYVFAILSTLYSSEVN     221
                  *****************************************
```

f. cox3

```
Seq15             MTKKNHPFHLVDVSPWPILGALSAMSTMVGLIKWFHMYQVNLFLVGLLSTSLIMYQWWRD  60
YP_009655014.1    MTKKNHPFHLVDVSPWPILGALSAMSTMVGLIKWFHMYQVNLFLVGLLSTSLIMYQWWRD  60
                  ************************************************************

Seq15             ITREGSFQGHHTFIVTMGLRWGMILFITSEVFFFISFFWGFFHSSLSPSIEIGMNWPPLG  120
YP_009655014.1    ITREGSFQGHHTFIVTMGLRWGMILFITSEVFFFISFFWGFFHSSLSPSIEIGMNWPPLG  120
                  ************************************************************

Seq15             ILTFNPLSIPLLNTLILLTSGLTVTWAHHSLMENNWKQANQGLTLTIILGLYFTILQAYE  180
YP_009655014.1    ILTFNPLSIPLLNTLILLTSGLTVTWAHHSLMENNWKQANQGLTLTIILGLYFTILQAYE  180
                  ************************************************************

Seq15             YIEAPFTISDSVYGSSFFMATGFHGLHVIIGTTFLSVCLLRHLMNHFSMIHHFGFEAAAW  240
YP_009655014.1    YIEAPFTISDSVYGSSFFMATGFHGLHVIIGTTFLSVCLLRHLMNHFSMIHHFGFEAAAW  240
                  ************************************************************

Seq15             YWHFVDVVWLFLYLSIYWWGK    280
YP_009655014.1    YWHFVDVVWLFLYLSIYWWGS    261
                  ********************.
```

g. nad3

```
Seq17               MKIILISSMIIFSISLILMMMNQVISKKTFKDREKMSPYECGFDPKSHARIPLSIRFFLI      60
YP_009655015.1      MKIILISSMIIFSISLILMMMNQVISKKTFKDREKMSPYECGFDPKSHARIPLSIRFFLI      60
                    ************************************************************

Seq17               TMIFLIFDVEITLLLPAINNLKTTNPLEFLITFIFFISILTLGTIHEWKQGALNWK-        117
YP_009655015.1      TVIFLIFDVEITLLLPAINNLKTTNPLEFLITFIFFISILTLGTIHEWKQGALNWKN        117
                    *:*******************************************************
```

h. nad5

```
Seq24               MSLTFVFFSLFLFCSFFFFFLSLYFMILDFSLFVEYELFSLNSSLINFSLYFDFMSLIFM      60
YP_009655016.1      MSLTFVFFSLFLFCSFFFFFLSLYFMILDFSLFVEYELFSLNSSLINFSLYFDFMSLIFM      60
                    ************************************************************

Seq24               SFVFFISSMVVFYSEDYMHGDLMLNRFILLVVLFVLSMMMMILSPNIISILLGWDGLGLV      120
YP_009655016.1      SFVFFISSMVVFYSEDYMHGDLMLNRFILLVVLFVLSMMMMILSPNIISILLGWDGLGLV      120
                    ************************************************************

Seq24               SYSLVIYYQNFKSFSAGMLTALSNRIGDVALLMSIAWMLNYGSWNYLFYVFFIDDNYLFI      180
YP_009655016.1      SYSLVIYYQNFKSFSAGMLTALSNRIGDVALLMSIAWMLNYGSWNYLFYVFFIDDNYLFI      180
                    ************************************************************

Seq24               ISLFIMLAAFTKSAQIPFSAWLPAAMAAPTPVSSLVHSSTLVTAGVYLMIRFSYCFCDKV      240
YP_009655016.1      ISLFIMLAAFTKSAQIPFSAWLPAAMAAPTPVSSLVHSSTLVTAGVYLMIRFSYCFCDKV      240
                    ************************************************************

Seq24               LILGLLLSCLTMFMAGLGANFEFDLKKIIALSTLSQLGLMMSILFSGGEYLAFFHLLSHA      300
YP_009655016.1      LILGLLLSCLTMFMAGLGANFEFDLKKIIALSTLSQLGLMMSILFSGGEYLAFFHLLSHA      300
                    ************************************************************

Seq24               LFKALLFMCAGSFIHSFMNFQDIRFMGSALNFMPLTSCFFIICNFSLCGLPFLSGFYSKD      360
YP_009655016.1      LFKALLFMCAGSFIHSFMNFQDIRFMGSALNFMPLTSCFFIICNFSLCGLPFLSGFYSKD      360
                    ************************************************************

Seq24               LILEFYSMKFVNMFSYILFFVSTGLTVSYTVRLMYYLFFGYLNFSPLFLISEGGNFMLFG      420
YP_009655016.1      LISEFYSMKFVNMFSYILFFVSTGLTVSYTVRLMYYLFFGYLNFSPLFLISEGGNFMLFG      420
                    ** *********************************************************

Seq24               MLGLIFPVIVAGSVLSWLMFSTPYYICLPLYMKFMTLFVIFSGLLIGVEVSRFSLNYHSK      480
YP_009655016.1      MLGLIFPVIVAGSVLSWLMFSTPYYICLPLYMKFMTLFVIFSGLLIGVEVSRFSLNYHSK      480
                    ************************************************************

Seq24               SLNFMNFSWFSSLMWGLPLISSLGMSIFPLTLGRLFISSFDQGWLEHLGAQGFMMNILKL      540
YP_009655016.1      SLNFMNFSWFSSLMWGLPLISSLGMSIFPLTLGRLFISSFDQGWLEHLGAQGFMMNILKL      540
                    ************************************************************

Seq24               TRSLHLIFSNQLKLFLIMLIFWVSFLILFYLNSL   577
YP_009655016.1      TRSLHLIFSNQLKLFLIMLIFWVSFLILF-----   569
                    *****************************
```

i. nad4

```
Seq26               MMKFILFVFFMIPLSFLDFYLVFLFLGLMMFIFLKIAPVSTILLGLGFGCDLFSGPLILL     60
YP_009655017.1      MMKFILFVFFMIPLSFLDFYLVFLFLGLMMFIFLKIAPVSTILLGLGFGCDLFSGPLILL     60
                    ************************************************************

Seq26               SFFICLLMFIASSKIYMDNNFHFYFSLNVIMLLFFLICAFSSLNFILFYVFFESSLIPTL     120
YP_009655017.1      SFFICLLMFIASSKIYMDNNFHFYFSLNVIMLLFFLICAFSSLNFILFYVFFESSLIPTL     120
                    ************************************************************

Seq26               FLILGWGYQPERIQAGFYLLFYTLFASLPMFLGLMHYYLSFGSLSMFLMFQVNSMIFFNM     180
YP_009655017.1      FLILGWGYQPERIQAGFYLLFYTLFASLPMFLGLMHYYLSFGSLSMFLMFQVNSMIFFNM     180
                    ************************************************************

Seq26               MIFVFLVKIPMFVVHLWLPKAHVEAPVSGSMILAGVMLKLGGYGLLRVFPLFKAEVSFFS     240
YP_009655017.1      MIFVFLVKIPMFVVHLWLPKAHVEAPVSGSMILAGVMLKLGGYGLLRVFPLFKAEVSFFS     240
                    ************************************************************

Seq26               TFFVSISVFGGLIVSLICLRQSDIKSLIAYSSVAHMSMVISGCFTLTTWGVIGSLIMMIS     300
YP_009655017.1      TFFVSISVFGGLIVSLICLRQSDIKSLIAYSSVAHMSMVISGCFTLTTWGVIGSLIMMIS     300
                    ************************************************************

Seq26               HGICSSGLFCLANITYERSLSRSLYLNKGYINILPNLSLWWFLLCSSNMAAPPSLNLVSE     360
YP_009655017.1      HGICSSGLFCLANITYERSLSRSLYLNKGYINILPNLSLWWFLLCSSNMAAPPSLNLVSE     360
                    ************************************************************

Seq26               ILILSSLVSWSSLMILILSFLSFLSAAYSLFLYSFTQHGMVSSGLFSFWSVNVREYLLLF     420
YP_009655017.1      ILILSSLVSWSSLMILILSFLSFLSAAYSLFLYSFTQHGMVSSGLFSFWSVNVREYLLLF     420
                    ************************************************************

Seq26               FHWIPLNLIFLFMDNLFSL         451
YP_009655017.1      CHWIPLNLIFLFMHNLFSL         439
                     ************.*****
```

j. cytB

```
Seq31               MKKQLMKISPVTKIINNALIDLPTPSNISIWWNMGSILGLCLIIQIITGMFLTMHYTPNT     60
YP_009655020.1      MKKQLMKISPVTKIINNALIDLPTPSNISIWWNMGSILGLCLIIQIITGLFLTMHYTPNT     60
                    *************************************************:**********

Seq31               EMAFNSVVHICRDVNYGWMIRTIHANGASMFFICIYMHIGRGLYYGSYKLIHTWMVGVII     120
YP_009655020.1      EMAFNSVVHICRDVNYGWMIRTIHANGASMFFICIYMHIGRGLYYGSYKLIHTWMVGVII     120
                    ************************************************************

Seq31               LFLVMATAFLGYVLPWGQMSFWGATVITNLLSAIPYVGKMIVEWLWGGFAVDNATLNRFF     180
YP_009655020.1      LFLIMATAFLGYVLPWGQMSFWGATVITNLLSAIPYVGKMIVEWLWGGFAVDNATLNRFF     180
                    ***:********************************************************

Seq31               AFHFLLPFIVSAMVMIHLLFLHQTGSNNPLGTNSNIDKIPFHPYFSTKDMMGFIVTTMML     240
YP_009655020.1      AFHFLLPFIVSAMVMIHLLFLHQTGSNNPLGTNSNIDKIPFHPYFSTKDIMGFIVTTMML     240
                    *************************************************:**********

Seq31               MTISLWNPYLLGDPDNFIPANPLVTPVHIQPEWYFLFAYAILRSIPNKLGGVIALIMSIA     300
YP_009655020.1      MTISLWNPYLLGDPDNFIPANPLVTPVHIQPEWYFLFAYAILRSIPNKLGGVIALIMSIA     300
                    ************************************************************

Seq31               ILFIIPLTNKSKFQSMNFYPMNKFLFWSMVSTVILLTWIGARPVQDPYITTGQVLTIIYF     360
YP_009655020.1      ILFIIPLTNKSKFQSMNFYPVNKFLFWSMVSTVILLTWIGARPVQDPYITTGQVLTIIYF     360
                    ********************:***************************************

Seq31               LYYMITPLTLKIWDKILIK         379
YP_009655020.1      LYYMITPLTLKIWDKILIK         379
                    *******************
```

k. nad1

```
Seq33                 MLFIFDFILVFFSLLVLIIGVLVGVAFLTLMERKVLGYIQLRKGPNKVGFMGLLQPFSDA      60
YP_009655021.1        -MFIFDFILVFFSLLVLIIGVLVGVAFLTLMERKVLGYIQLRKGPNKVGFMGLLQPFSDA      59
                       :**********************************************************

Seq33                 IKLFSKEQCLPLVSNYVFYYFSPVMNFFISLMMWLSFPFFSYFLSFNLGMLFFLCFSSLS      120
YP_009655021.1        IKLFSKEQCLPLVSNYVFYYFSPVMNFFISLMMWLSFPFFSYFLSFNLGMLFFLCFSSLS      119
                      ************************************************************

Seq33                 VYAVMISGWSSNSNYSMIGSLRSMAQTISYEVSLVIIIISFLVMTSSLCFYDFFKYQKYL      180
YP_009655021.1        VYAVMISGWSSNSNYSMIGSLRSMAQTISYEVSLVIIIISFLVMTSSLCFYDFFKYQKYL      179
                      ************************************************************

Seq33                 WFVFLMFPLSLILFVSMLAETNRTPFDFAEGESELVSGFNVEYSSGGFALIFLAEYSSIL      240
YP_009655021.1        WFVFLMFPLSLILFVSMLAETNRTPFDFAEGESELVSGFNVEYSSGGFALIFLAEYSSIL      239
                      ************************************************************
Seq33                 FMSMICSLMFFGGDYMNLFFFIKIVFFSFLWVWVRGTLPRYRYDKLMYLAWKIYLPVSLC      300
YP_009655021.1        FMSMICSLMFFGGDLYKFIFFIKIVFFSFLWVWVRGTLPRYRYDKLMYLAWKIYLPVSLC      299
                      **************  :::*****************************************

Seq33                 FLLFYLGLKTLCWVFII 317
YP_009655021.1        FLLFYLGLKTLCWVFII 316
                      *****************
```
