# Supplementary material for: Variants in the Mitochondrial Genome Sequence of Rhyzopertha dominica (Fabricius) (Coleoptera: Bostrycidae)
Source: Insects. 2021 Apr 27;12(5):387. doi: 10.3390/insects12050387 (PMC8146127; doi:10.3390/insects12050387)
Supplement: Supplementary file 1 [file insects-12-00387-s001.zip › insects-1191702-s/Figure S5.pdf]

Figure S5. Alignment of long reads (PacBio, ml#) to the predicted sequence (accession #MW020612; Rdo_MitoGenome) of the *Rhyzopertha dominica* mitochondrial genome and that predicted by Ouyang et al. [15] (accession #NC_042820). Protein-coding sequences are grayed and annotated below.

```
m141031_iid=347546_0       ------------------------------------------------------------   0
m150506_iid=3127414_0      ------------------------------------------------------------   0
m141102_iid=1245517_0      ------------------------------------------------------------   0
m141107_iid=1405727_0      ------------------------------------------------------------   0
m141102_iid=987614_0       ------------------------------------------------------------   0
m141108_iid=1701429_0      ------------------------------------------------------------   0
m141102_iid=987286_0       ------------------------------------------------------------   0
m150430_iid=3272125_0      ------------------------------------------------------------   0
m141102_iid=754225_0       ------------------------------------------------------------   0
m141107_iid=1409934_0      ------------------------------------------------------------   0
m150503_iid=3416920_0      ------------------------------------------------------------   0
m141108_iid=1800121_0      ------------------------------------------------------------   0
m141031_iid=147540_0       ------------------------------------------------------------   0
m150507_iid=3674924_0      ------------------------------------------------------------   0
m150503_iid=2923751_0      ------------------------------------------------------------   0
m141031_iid=115118_0       ------------------------------------------------------------   0
m150430_iid=2722448_0      ------------------------------------------------------------   0
m150507_iid=3102647_0      ------------------------------------------------------------   0
m141108_iid=1811866_0      ------------------------------------------------------------   0
m150501_iid=2948495_0      ------------------------------------------------------------   0
m150504_iid=3238195_0      ------------------------------------------------------------   0
m150503_iid=3388511_0      ------------------------------------------------------------   0
m150505_iid=3777611_0      ------------------------------------------------------------   0
m150430_iid=2438833_0      ------------------------------------------------------------   0
m141108_iid=1698969_0      ------------------------------------------------------------   0
m150430_iid=3059190_0      ------------------------------------------------------------   0
m141107_iid=1383067_0      ------------------------------------------------------------   0
m141101_iid=597008_0       ------------------------------------------------------------   0
m150506_iid=2596496_0      ------GTGCCTGAAAAAAAGGATTATTTTGATAGAATAAATTATGTAGAAATACCTTCA   54
m141102_iid=910051_0       ------------------------------------------------------------   0
m150505_iid=3784017_0      ------------------------------------------------------------   0
m141101_iid=682151_0       ------------------------------------------------------------   0
m150501_iid=3748029_0      ------------------------------------------------------------   0
m150504_iid=2876272_0      ------------------------------------------------------------   0
NC_042820.1                AATGAAGTGCCTGAAAAAAAGGATTATTTTGATAGAATAAATTATGTAGAAATACCTTCA   60
m141102_iid=1228825_0      ------------------------------------------------------------   0
m150504_iid=2876274_0      ------------------------------------------------------------   0
m141030_iid=80036_0        ------------------------------------------------------------   0
m150506_iid=2596494_0      --TGAAGTGCCTGAAAA-AAGGATTATTTTGATAGAATAAATTATGTAGAAATACCTTCA   57
m150503_iid=2526862_0      ------------------------------------------------------------   0
m150506_iid=2567000_0      ------------------------------------------------------------   0
m150507_iid=3073271_0      ------------------------------------------------------------   0
m150504_iid=3156087_0      ------------------------------------------------------------   0
m150505_iid=2498502_0      ------------------------------------------------------------   0
m141102_iid=754227_0       ------------------------------------------------------------   0
m150507_iid=3469514_0      ------------------------------------------------------------   0
Rdo_MitoGenome             AATGAAGTGCCTGAAAAAAAGGATTATTTTGATAGAATAAATTATGTAGAAATACCTTCA   60
m141101_iid=614933_0       ------------------------------------------------------------   0


m141031_iid=347546_0       ------------------------------------------------------------   0
m150506_iid=3127414_0      ------------------------------------------------------------   0
m141102_iid=1245517_0      ------------------------------------------------------------   0
m141107_iid=1405727_0      ------------------------------------------------------------   0
m141102_iid=987614_0       ------------------------------------------------------------   0
m141108_iid=1701429_0      ------------------------------------------------------------   0
m141102_iid=987286_0       ------------------------------------------------------------   0
m150430_iid=3272125_0      ------------------------------------------------------------   0
m141102_iid=754225_0       ------------------------------------------------------------   0
m141107_iid=1409934_0      ------------------------------------------------------------   0
m150503_iid=3416920_0      ------------------------------------------------------------   0
m141108_iid=1800121_0      ------------------------------------------------------------   0
m141031_iid=147540_0       ------------------------------------------------------------   0
m150507_iid=3674924_0      ------------------------------------------------------------   0
m150503_iid=2923751_0      ------------------------------------------------------------   0
```

m141031_iid=115118_0	--	0
m150430_iid=2722448_0	--	0
m150507_iid=3102647_0	--	0
m141108_iid=1811866_0	--	0
m150501_iid=2948495_0	--	0
m150504_iid=3238195_0	--	0
m150503_iid=3388511_0	--	0
m150505_iid=3777611_0	--	0
m150430_iid=2438833_0	--	0
m141108_iid=1698969_0	--	0
m150430_iid=3059190_0	--	0
m141107_iid=1383067_0	--	0
m141101_iid=597008_0	--	0
m150506_iid=2596496_0	TTATATGAAATAGAATTAAACTAAAACTTTAGATTCCAA-AATCTAAGTACATCTTATAC	113
m141102_iid=910051_0	--	0
m150505_iid=3784017_0	--	0
m141101_iid=682151_0	--	0
m150501_iid=3748029_0	--	0
m150504_iid=2876272_0	--	0
NC_042820.1	TTATATGAAATAGAATTAAACTAAAACTTTAGATTCCAAAAATCTAAGTACATCTTATAC	120
m141102_iid=1228825_0	--	0
m150504_iid=2876274_0	--	0
m141030_iid=80036_0	--	0
m150506_iid=2596494_0	TTATATGAAATAGAATTAAACTAAAACTTTAGATTCCAAAAATCTAAGTACATCTTATAC	117
m150503_iid=2526862_0	--	0
m150506_iid=2567000_0	--	0
m150507_iid=3073271_0	--	0
m150504_iid=3156087_0	--	0
m150505_iid=2498502_0	--	0
m141102_iid=754227_0	--	0
m150507_iid=3469514_0	--	0
Rdo_MitoGenome	TTATATGAAATAGAATTAAACTAAAACTTTAGATTCCAAAAATCTAAGTACATCTTATAC	120
m141101_iid=614933_0	--	0

m141031_iid=347546_0	--	0
m150506_iid=3127414_0	--	0
m141102_iid=1245517_0	--	0
m141107_iid=1405727_0	--	0
m141102_iid=987614_0	--	0
m141108_iid=1701429_0	--	0
m141102_iid=987286_0	--	0
m150430_iid=3272125_0	--	0
m141102_iid=754225_0	--	0
m141107_iid=1409934_0	--	0
m150503_iid=3416920_0	--	0
m141108_iid=1800121_0	--	0
m141031_iid=147540_0	--	0
m150507_iid=3674924_0	--	0
m150503_iid=2923751_0	--	0
m141031_iid=115118_0	--	0
m150430_iid=2722448_0	--	0
m150507_iid=3102647_0	--	0
m141108_iid=1811866_0	--	0
m150501_iid=2948495_0	--	0
m150504_iid=3238195_0	--	0
m150503_iid=3388511_0	--	0
m150505_iid=3777611_0	--	0
m150430_iid=2438833_0	--	0
m141108_iid=1698969_0	--	0
m150430_iid=3059190_0	--	0
m141107_iid=1383067_0	--	0
m141101_iid=597008_0	--	0
m150506_iid=2596496_0	TATCATATAAAAAGATAAGCTAATAAAGCTATTGGGTTCATACCCCAACCATAAAGGTTA	173
m141102_iid=910051_0	--	0
m150505_iid=3784017_0	--	0
m141101_iid=682151_0	--	0
m150501_iid=3748029_0	--	0
m150504_iid=2876272_0	--	0
NC_042820.1	TATCATATAAAAAGATAAGCTAATAAAGCTATTGGGTTCATACCCCGACCATAAAGGTTA	180
m141102_iid=1228825_0	--CCAACCATAAAGGTTA	16

```
m150504_iid=2876274_0      -------------------------------------------------CATAAAGGTTA   11
m141030_iid=80036_0        ------------------------------------------------------------   0
m150506_iid=2596494_0      TATCATATAAAAAGATAAGCTAATAAAGCTATTGGGTTCATACCCCAACCATAAAGGTTA   177
m150503_iid=2526862_0      ------------------------------------------------------------   0
m150506_iid=2567000_0      ------------------------------------------------------------   0
m150507_iid=3073271_0      ------------------------------------------------------------   0
m150504_iid=3156087_0      ------------------------------------------------------------   0
m150505_iid=2498502_0      ------------------------------------------------------------   0
m141102_iid=754227_0       ------------------------------------------------------------   0
m150507_iid=3469514_0      ------------------------------------------------------------   0
Rdo_MitoGenome             TATCATATAAAAAGATAAGCTAATAAAGCTATTGGGTTCATACCCCAACCATAAAGGTTA   180
m141101_iid=614933_0       ------------------------------------------------------------   0


m141031_iid=347546_0       ------------------------------------------------------------   0
m150506_iid=3127414_0      ------------------------------------------------------------   0
m141102_iid=1245517_0      ------------------------------------------------------------   0
m141107_iid=1405727_0      ------------------------------------------------------------   0
m141102_iid=987614_0       ------------------------------------------------------------   0
m141108_iid=1701429_0      ------------------------------------------------------------   0
m141102_iid=987286_0       ------------------------------------------------------------   0
m150430_iid=3272125_0      ------------------------------------------------------------   0
m141102_iid=754225_0       ------------------------------------------------------------   0
m141107_iid=1409934_0      ------------------------------------------------------------   0
m150503_iid=3416920_0      ------------------------------------------------------------   0
m141108_iid=1800121_0      ------------------------------------------------------------   0
m141031_iid=147540_0       ------------------------------------------------------------   0
m150507_iid=3674924_0      ------------------------------------------------------------   0
m150503_iid=2923751_0      ------------------------------------------------------------   0
m141031_iid=115118_0       ------------------------------------------------------------   0
m150430_iid=2722448_0      ------------------------------------------------------------   0
m150507_iid=3102647_0      ------------------------------------------------------------   0
m141108_iid=1811866_0      ------------------------------------------------------------   0
m150501_iid=2948495_0      ------------------------------------------------------------   0
m150504_iid=3238195_0      ------------------------------------------------------------   0
m150503_iid=3388511_0      ------------------------------------------------------------   0
m150505_iid=3777611_0      ------------------------------------------------------------   0
m150430_iid=2438833_0      ------------------------------------------------------------   0
m141108_iid=1698969_0      ------------------------------------------------------------   0
m150430_iid=3059190_0      ------------------------------------------------------------   0
m141107_iid=1383067_0      ------------------------------------------------------------   0
m141101_iid=597008_0       ------------------------------------------------------------   0
m150506_iid=2596496_0      T-AATCCTTTTCTTTTTAATAATCAAATTATCAAAAATCTCATTTACAATTACAATAATA   232
m141102_iid=910051_0       ------------------------------------------------------------   0
m150505_iid=3784017_0      ------------------------------------------------------------   0
m141101_iid=682151_0       ------------------------------------------------------------   0
m150501_iid=3748029_0      ------------------------------------------------------------   0
m150504_iid=2876272_0      ------------------------------------------------------------   0
NC_042820.1                T-AATCCTTTTCTTTTTAATAATCAAATTATCAAAAATCTCATTTACAATTACAATAATA   239
m141102_iid=1228825_0      TAAATCCTTTTCTTTTTAATAATCAAATTATCAAAAATCTCATTTACAATTACAATAA-A   75
m150504_iid=2876274_0      T-AATCCTTTTCTTTTTAATAATCAAATTATCAAAAATCTCATTTACAATTACAATAATA   70
m141030_iid=80036_0        ------------------------------------------------------------   0
m150506_iid=2596494_0      T-AATCCTTTTCTTTTTAATAATCAAATTATCAAAAATCTCATTTACAATTACAATAATA   236
m150503_iid=2526862_0      ------------------------------------------------------------   0
m150506_iid=2567000_0      ------------------------------------------------------------   0
m150507_iid=3073271_0      ------------------------------------------------------------   0
m150504_iid=3156087_0      ------------------------------------------------------------   0
m150505_iid=2498502_0      ------------------------------------------------------------   0
m141102_iid=754227_0       ------------------------------------------------------------   0
m150507_iid=3469514_0      ------------------------------------------------------------   0
Rdo_MitoGenome             T-AATCCTTTTCTTTTTAATAATCAAATTATCAAAAATCTCATTTACAATTACAATAATA   239
m141101_iid=614933_0       ------------------------------------------------------------   0
                                             nad2 ---------------------------------->

m141031_iid=347546_0       ------------------------------------------------------------   0
m150506_iid=3127414_0      ------------------------------------------------------------   0
m141102_iid=1245517_0      ------------------------------------------------------------   0
m141107_iid=1405727_0      ------------------------------------------------------------   0
m141102_iid=987614_0       ------------------------------------------------------------   0
m141108_iid=1701429_0      ------------------------------------------------------------   0
m141102_iid=987286_0       ------------------------------------------------------------   0
```

```
m150430_iid=3272125_0      ------------------------------------------------------------  0
m141102_iid=754225_0       ------------------------------------------------------------  0
m141107_iid=1409934_0      ------------------------------------------------------------  0
m150503_iid=3416920_0      ------------------------------------------------------------  0
m141108_iid=1800121_0      ------------------------------------------------------------  0
m141031_iid=147540_0       ------------------------------------------------------------  0
m150507_iid=3674924_0      ------------------------------------------------------------  0
m150503_iid=2923751_0      ------------------------------------------------------------  0
m141031_iid=115118_0       ------------------------------------------------------------  0
m150430_iid=2722448_0      ------------------------------------------------------------  0
m150507_iid=3102647_0      ------------------------------------------------------------  0
m141108_iid=1811866_0      ------------------------------------------------------------  0
m150501_iid=2948495_0      ------------------------------------------------------------  0
m150504_iid=3238195_0      ------------------------------------------------------------  0
m150503_iid=3388511_0      ------------------------------------------------------------  0
m150505_iid=3777611_0      ------------------------------------------------------------  0
m150430_iid=2438833_0      ------------------------------------------------------------  0
m141108_iid=1698969_0      ------------------------------------------------------------  0
m150430_iid=3059190_0      ------------------------------------------------------------  0
m141107_iid=1383067_0      ------------------------------------------------------------  0
m141101_iid=597008_0       ------------------------------------------------------------  0
m150506_iid=2596496_0      ATCGGATCAATAATTACAATTTCATCGTATACATGAATAGGAATATGAATAGGCTTAGAA  292
m141102_iid=910051_0       ------------------------------------------------------------  0
m150505_iid=3784017_0      ------------------------------------------------------------  0
m141101_iid=682151_0       ------------------------------------------------------------  0
m150501_iid=3748029_0      ------------------------------------------------------------  0
m150504_iid=2876272_0      -TCGGATCAATAATTACAATTTCATCGTATACATGAATAGGAATATGAATAGGCTTAGAA  59
NC_042820.1                ATCGGATCAATAATTACAATTTCATCGTATACATGAATAGGAATATGAATAGGCTTAGAA  299
m141102_iid=1228825_0      ATCGGATCAATAATTACAATTTCATCGTATACATGAATAGGAATATGAATAGGCTTAGAA  135
m150504_iid=2876274_0      ATCGGATCAATAATTACAATTTCATCGTATACATGAATAGGAATATGAATAGGCTTAGAA  130
m141030_iid=80036_0        ------------------------------------------------------------  0
m150506_iid=2596494_0      ATCGGATCAATAATTACAATTTCATCGTATACATGAATAGGAATATGAATAGGCTTAGAA  296
m150503_iid=2526862_0      ------------------------------------------------------------  0
m150506_iid=2567000_0      ------------------------------------------------------------  0
m150507_iid=3073271_0      ------------------------------------------------------------  0
m150504_iid=3156087_0      ------------------------------------------------------------  0
m150505_iid=2498502_0      ------------------------------------------------------------  0
m141102_iid=754227_0       ------------------------------------------------------------  0
m150507_iid=3469514_0      ------------------------------------------------------------  0
Rdo_MitoGenome             ATCGGATCAATAATTACAATTTCATCGTATACATGAATAGGAATATGAATAGGCTTAGAA  299
m141101_iid=614933_0       ------------------------------------------------------------  0
```

nad2 →

```
m141031_iid=347546_0       ------------------------------------------------------------  0
m150506_iid=3127414_0      ------------------------------------------------------------  0
m141102_iid=1245517_0      ------------------------------------------------------------  0
m141107_iid=1405727_0      ------------------------------------------------------------  0
m141102_iid=987614_0       ------------------------------------------------------------  0
m141108_iid=1701429_0      ------------------------------------------------------------  0
m141102_iid=987286_0       ------------------------------------------------------------  0
m150430_iid=3272125_0      ------------------------------------------------------------  0
m141102_iid=754225_0       ------------------------------------------------------------  0
m141107_iid=1409934_0      ------------------------------------------------------------  0
m150503_iid=3416920_0      ------------------------------------------------------------  0
m141108_iid=1800121_0      ------------------------------------------------------------  0
m141031_iid=147540_0       ------------------------------------------------------------  0
m150507_iid=3674924_0      ------------------------------------------------------------  0
m150503_iid=2923751_0      ------------------------------------------------------------  0
m141031_iid=115118_0       ------------------------------------------------------------  0
m150430_iid=2722448_0      ------------------------------------------------------------  0
m150507_iid=3102647_0      ------------------------------------------------------------  0
m141108_iid=1811866_0      ------------------------------------------------------------  0
m150501_iid=2948495_0      ------------------------------------------------------------  0
m150504_iid=3238195_0      ------------------------------------------------------------  0
m150503_iid=3388511_0      ------------------------------------------------------------  0
m150505_iid=3777611_0      ------------------------------------------------------------  0
m150430_iid=2438833_0      ------------------------------------------------------------  0
m141108_iid=1698969_0      ---------------------------------------AAAAACATTTATTCAAC-GAA  20
m150430_iid=3059190_0      ------------------------------------------------------------  0
m141107_iid=1383067_0      ------------------------------------------------------------  0
m141101_iid=597008_0       ------------------------------------------------------------  0
```

```
m150506_iid=2596496_0      ATTAACCTTCTTTCAATTATTCCACTATTAACAACTAAAAAAAACATTTATTCAACCGAA   352
m141102_iid=910051_0       ------------------------------------------------------------   0
m150505_iid=3784017_0      ------------------------------------------------------------   0
m141101_iid=682151_0       ------------------------------------------------------------   0
m150501_iid=3748029_0      ------------------------------------------------------------   0
m150504_iid=2876272_0      ATTAACCTTCTTTCAATTATTCCACTATTAACAACTAAAAAAAACATTTATTCAACCGAA   119
NC_042820.1                ATTAACCTTCTTTCAATTATTCCACTATTAACAACTAAAAAAAACATTTATTCAACCGAA   359
m141102_iid=1228825_0      ATTAACCTTCTTTCAATTATTCCACTATTAACAACTAAAAAAAACATTTATTCAACCGAA   195
m150504_iid=2876274_0      ATTAACCTTCTTTCAATTATTCCACTATTAACAACTAAAAAAAACATTTATTCAACCGAA   190
m141030_iid=80036_0        ------------------------------------------------------------   0
m150506_iid=2596494_0      ATTAACCTTCTTTCAATTATTCCACTATTAACAACTAAA-AAAACATTTATTCAACCGAA   355
m150503_iid=2526862_0      -------------------TTCCACTATTAACAACTAAAAAAAACATTTATTCAACCGAA   41
m150506_iid=2567000_0      ------------------------------------------------------------   0
m150507_iid=3073271_0      ------------------------------------------------------------   0
m150504_iid=3156087_0      ------------------------------------------------------------   0
m150505_iid=2498502_0      ------------------------------------------------------------   0
m141102_iid=754227_0       ------------------------------------------------------------   0
m150507_iid=3469514_0      ------------------------------------------------------------   0
Rdo_MitoGenome             ATTAACCTTCTTTCAATTATTCCACTATTAACAACTAAAAAAAACATTTATTCAACCGAA   359
m141101_iid=614933_0       ------------------------------------------------------------   0
```

nad2

```
m141031_iid=347546_0       ------------------------------------------------------------   0
m150506_iid=3127414_0      ------------------------------------------------------------   0
m141102_iid=1245517_0      ------------------------------------------------------------   0
m141107_iid=1405727_0      ------------------------------------------------------------   0
m141102_iid=987614_0       ------------------------------------------------------------   0
m141108_iid=1701429_0      ------------------------------------------------------------   0
m141102_iid=987286_0       ------------------------------------------------------------   0
m150430_iid=3272125_0      ------------------------------------------------------------   0
m141102_iid=754225_0       ------------------------------------------------------------   0
m141107_iid=1409934_0      ------------------------------------------------------------   0
m150503_iid=3416920_0      ------------------------------------------------------------   0
m141108_iid=1800121_0      ------------------------------------------------------------   0
m141031_iid=147540_0       ------------------------------------------------------------   0
m150507_iid=3674924_0      ------------------------------------------------------------   0
m150503_iid=2923751_0      ------------------------------------------------------------   0
m141031_iid=115118_0       ------------------------------------------------------------   0
m150430_iid=2722448_0      ------------------------------------------------------------   0
m150507_iid=3102647_0      ------------------------------------------------------------   0
m141108_iid=1811866_0      ------------------------------------------------------------   0
m150501_iid=2948495_0      ------------------------------------------------------------   0
m150504_iid=3238195_0      ------------------------------------------------------------   0
m150503_iid=3388511_0      ------------------------------------------------------------   0
m150505_iid=3777611_0      ------------------------------------------------------------   0
m150430_iid=2438833_0      ------------------------------------------------------------   0
m141108_iid=1698969_0      GCAGCAATCAAATACTTCATTACACAAGCATTGGCATCATCAATCCTATTAATATCAATC   80
m150430_iid=3059190_0      ------------------------------------------------------------   0
m141107_iid=1383067_0      ------------------------------------------------------------   0
m141101_iid=597008_0       ------------------------------------------------------------   0
m150506_iid=2596496_0      GCAGCAATCAAATACTTCATTACACAAGCATTGGCATCATCAATCCTATTAATATCAATC   412
m141102_iid=910051_0       ------------------------------------------------------------   0
m150505_iid=3784017_0      ------------------------------------------------------------   0
m141101_iid=682151_0       ------------------------------------------------------------   0
m150501_iid=3748029_0      ------------------------------------------------------------   0
m150504_iid=2876272_0      GCAGCAATCAAATACTTCATTACACAAGCATTGGCATCATCAATCCTATTAATATCAATC   179
NC_042820.1                GCAGCAATCAAATACTTCATTACACAAGCATTGACATCATCAATCCTATTAATATCAATC   419
m141102_iid=1228825_0      GCAGCAATCAAATACTTCATTACACAAGCATTGGCATCATCAATCCTATTAATATCAATC   255
m150504_iid=2876274_0      GCAGCAATCAAATACTTCATTACACAAGCATTGGCATCATCAATCCTATTAATATCAATC   250
m141030_iid=80036_0        ------------------------------TTGGCATCATCAATCCTATTAATATCAATC   30
m150506_iid=2596494_0      GCAGCAATCAAATACTTCATTACACAAGCATTGGCATCATCAATCCTATTAATATCAATC   415
m150503_iid=2526862_0      GCAGCAATCAAATACTTCATTACACAAGCATTGGCATCATCAATCCTATTAATATCAATC   101
m150506_iid=2567000_0      ------------------------------------------------------------   0
m150507_iid=3073271_0      ------------------------------------------------------------   0
m150504_iid=3156087_0      ------------------------------------------------------------   0
m150505_iid=2498502_0      ------------------------------------------------------------   0
m141102_iid=754227_0       ------------------------------------------------------------   0
m150507_iid=3469514_0      ------------------------------------------------------------   0
Rdo_MitoGenome             GCAGCAATCAAATACTTCATTACACAAGCATTGGCATCATCAATCCTATTAATATCAATC   419
m141101_iid=614933_0       ------------------------------------------------------------   0
```

nad2

```
m141031_iid=347546_0       ------------------------------------------------------------   0
m150506_iid=3127414_0      ------------------------------------------------------------   0
m141102_iid=1245517_0      ------------------------------------------------------------   0
m141107_iid=1405727_0      ------------------------------------------------------------   0
m141102_iid=987614_0       ------------------------------------------------------------   0
m141108_iid=1701429_0      ------------------------------------------------------------   0
m141102_iid=987286_0       ------------------------------------------------------------   0
m150430_iid=3272125_0      ------------------------------------------------------------   0
m141102_iid=754225_0       ------------------------------------------------------------   0
m141107_iid=1409934_0      ------------------------------------------------------------   0
m150503_iid=3416920_0      ------------------------------------------------------------   0
m141108_iid=1800121_0      ------------------------------------------------------------   0
m141031_iid=147540_0       ------------------------------------------------------------   0
m150507_iid=3674924_0      ------------------------------------------------------------   0
m150503_iid=2923751_0      ------------------------------------------------------------   0
m141031_iid=115118_0       ------------------------------------------------------------   0
m150430_iid=2722448_0      ------------------------------------------------------------   0
m150507_iid=3102647_0      ------------------------------------------------------------   0
m141108_iid=1811866_0      ------------------------------------------------------------   0
m150501_iid=2948495_0      ------------------------------------------------------------   0
m150504_iid=3238195_0      ------------------------------------------------------------   0
m150503_iid=3388511_0      ------------------------------------------------------------   0
m150505_iid=3777611_0      ------------------------------------------------------------   0
m150430_iid=2438833_0      ------------------------------------------------------------   0
m141108_iid=1698969_0      CTAATTTTTATTCAACAAATCTAATTAACCAAAAGTCCGC-ATAATAATTCTTTATTCAT   139
m150430_iid=3059190_0      ------------------------------------------------------------   0
m141107_iid=1383067_0      ------------------------------------------------------------   0
m141101_iid=597008_0       ------------------------------------------------------------   0
m150506_iid=2596496_0      CTAATTTT--ATCAACAAATCTAATTAACCAAAAGTCCGCAATAATAATTCTTTATTCAT   470
m141102_iid=910051_0       ------------------------------------------------------------   0
m150505_iid=3784017_0      ------------------------------------------------------------   0
m141101_iid=682151_0       ------------------------------------------------------------   0
m150501_iid=3748029_0      ------------------------------------------------------------   0
m150504_iid=2876272_0      CTAATTTT--ATCAACAAATCTAATTAACCAAAAGTCCGCAATAATAATTCTTTATTCAT   237
NC_042820.1                CTAATTTT--ATCAACAAATCTAATTAACCAAAAGTCCGCAATAATAATTCTTTATTCAT   477
m141102_iid=1228825_0      CTAATTTT--ATCAACAAATCTAATTAACCAAAAGTCCGCAATAATAATTCTTTATTCAT   313
m150504_iid=2876274_0      CTAATTTT--ATCAACAAATCTAATTAA-CAAAAGTCCGCAATAATAATTCTTTATTCAT   307
m141030_iid=80036_0        CTAATTT---ATCAACAAATCTAATTAACCAAA---CCGCAATAATAATTCTTTATTCAT   84
m150506_iid=2596494_0      CTAATTTT--ATCAACAAATCTAATTAACCAAAAGTCCGCAATAATAATTCTTTATTCAT   473
m150503_iid=2526862_0      CTAATTTT--ATCAACAAATCTAATTAACCAAAAGTCCGCAATAATAATTCTTTATTCAT   159
m150506_iid=2567000_0      ------------------------------------------------------------   0
m150507_iid=3073271_0      ------------------------------------------------------------   0
m150504_iid=3156087_0      ------------------------------------------------------------   0
m150505_iid=2498502_0      ------------------------------------------------------------   0
m141102_iid=754227_0       ------------------------------------------------------------   0
m150507_iid=3469514_0      ----------------------------------------------------------AT   2
Rdo_MitoGenome             CTAATTTT--ATCAACAAATCTAATTAACCAAAAGTCCGCAATAATAATTCTTTATTCAT   477
m141101_iid=614933_0       ------------------------------------------------------------   0
```

nad2

```
m141031_iid=347546_0       ------------------------------------------------------------   0
m150506_iid=3127414_0      ------------------------------------------------------------   0
m141102_iid=1245517_0      ------------------------------------------------------------   0
m141107_iid=1405727_0      ------------------------------------------------------------   0
m141102_iid=987614_0       ------------------------------------------------------------   0
m141108_iid=1701429_0      ------------------------------------------------------------   0
m141102_iid=987286_0       ------------------------------------------------------------   0
m150430_iid=3272125_0      ------------------------------------------------------------   0
m141102_iid=754225_0       ------------------------------------------------------------   0
m141107_iid=1409934_0      ------------------------------------------------------------   0
m150503_iid=3416920_0      ------------------------------------------------------------   0
m141108_iid=1800121_0      ------------------------------------------------------------   0
m141031_iid=147540_0       ------------------------------------------------------------   0
m150507_iid=3674924_0      ------------------------------------------------------------   0
m150503_iid=2923751_0      ------------------------------------------------------------   0
m141031_iid=115118_0       ------------------------------------------------------------   0
m150430_iid=2722448_0      ------------------------------------------------------------   0
m150507_iid=3102647_0      ------------------------------------------------------------   0
m141108_iid=1811866_0      ------------------------------------------------------------   0
m150501_iid=2948495_0      ------------------------------------------------------------   0
```

```
m150504_iid=3238195_0      ------------------------------------------------------------   0
m150503_iid=3388511_0      ------------------------------------------------------------   0
m150505_iid=3777611_0      ------------------------------------------------------------   0
m150430_iid=2438833_0      ------------------------------------------------------------   0
m141108_iid=1698969_0      CTTTAATAAC-AAAATAGGGACAGCCCCATTCCATTTTTGATTTCCTGAAATCATTGAAG   198
m150430_iid=3059190_0      ------------------------------------------------------------   0
m141107_iid=1383067_0      ------------------------------------------------------------   0
m141101_iid=597008_0       ------------------------------------------------------------   0
m150506_iid=2596496_0      CTTTAATAACAAAAATAGGAACAGCCCCATTCCATTTTTGATTTCCTGAAATCATTGAAG   530
m141102_iid=910051_0       ----------------------------------------------TGAAATCATTGAAG   14
m150505_iid=3784017_0      ------------------------------------------------------------   0
m141101_iid=682151_0       ------------------------------------------------------------   0
m150501_iid=3748029_0      ------------------------------------------------------------   0
m150504_iid=2876272_0      CTTTAATAACAAAAATAGGAACAGCCCCATTCCATTTTTGATTTCCTGAAATCATTGAAG   297
NC_042820.1                CTTTAATAACAAAAATAGGAACAGCCCCATTCCATTTTTGATTTCCTGAAATCATTGAAG   537
m141102_iid=1228825_0      CTTTAATAACAAAAATAGGAACAGCCCCATTCCATTTTTGATTTCCTGAAATCATTGAAG   373
m150504_iid=2876274_0      CTTTAATAACAAAAATAGGAACAGCCCCATTCCATTTTTGATTTCCTGAAATCATTGAAG   367
m141030_iid=80036_0        CTTTAATAAACAAAAATAGAACAGCC-----CCATTATTTATTTCCTGAAAT--TTGAGG   137
m150506_iid=2596494_0      CTTTAATAACAAAAATAGGAACAGCCCCATTCCATTTTTGATTTCCTGAAATCATTGAAG   533
m150503_iid=2526862_0      CTTTAATAACAAAAATAGGAACAGCCCCATTCCATTTTTGATTTCCTGAAATCATTGAAG   219
m150506_iid=2567000_0      ------------------------------------------------------------   0
m150507_iid=3073271_0      ------------------------------------------------------------   0
m150504_iid=3156087_0      ------------------------------------------------------------   0
m150505_iid=2498502_0      ------------------------------------------------------------   0
m141102_iid=754227_0       ------------------------------------------------------------   0
m150507_iid=3469514_0      CTTTAATAACAAAAATAGGAACAGCCCCATTCCATTTTTGATTTCCTGAAATCATTGAAG   62
Rdo_MitoGenome             CTTTAATAACAAAAATAGGAACAGCCCCATTCCATTTTTGATTTCCTGAAATCATTGAAG   537
m141101_iid=614933_0       ------------------------------------------------------------   0
```

nad2 →

```
m141031_iid=347546_0       ------------------------------------------------------------   0
m150506_iid=3127414_0      ------------------------------------------------------------   0
m141102_iid=1245517_0      ------------------------------------------------------------   0
m141107_iid=1405727_0      ------------------------------------------------------------   0
m141102_iid=987614_0       ------------------------------------------------------------   0
m141108_iid=1701429_0      ------------------------------------------------------------   0
m141102_iid=987286_0       ------------------------------------------------------------   0
m150430_iid=3272125_0      ------------------------------------------------------------   0
m141102_iid=754225_0       ------------------------------------------------------------   0
m141107_iid=1409934_0      ------------------TGCTTAATT-ATAATAACCTGACAAAAAATTGC-CCTGCAAT   40
m150503_iid=3416920_0      ------------------------------------------------------------   0
m141108_iid=1800121_0      ------------------------------------------------------------   0
m141031_iid=147540_0       ------------------------------------------------------------   0
m150507_iid=3674924_0      ------------------------------------------------------------   0
m150503_iid=2923751_0      ------------------------------------------------------------   0
m141031_iid=115118_0       ------------------------------------------------------------   0
m150430_iid=2722448_0      ------------------------------------------------------------   0
m150507_iid=3102647_0      ------------------------------------------------------------   0
m141108_iid=1811866_0      ------------------------------------------------------------   0
m150501_iid=2948495_0      ------------------------------------------------------------   0
m150504_iid=3238195_0      ------------------------------------------------------------   0
m150503_iid=3388511_0      ------------------------------------------------------------   0
m150505_iid=3777611_0      ------------------------------------------------------------   0
m150430_iid=2438833_0      ------------------------------------------------------------   0
m141108_iid=1698969_0      GATTAGATTGAATAAA-TTGCTTAATTAT-AATAACCTGACAAAAAATTGCCCCTGCAAT   256
m150430_iid=3059190_0      ------------------------------------------------------------   0
m141107_iid=1383067_0      ------------------------------------------------------------   0
m141101_iid=597008_0       ------------------------------------------------------------   0
m150506_iid=2596496_0      GATTAGATTGAATAAA-TTGCTTAATTA-TAATAACCTGACAAAAAATTGCCCCTGCAAT   588
m141102_iid=910051_0       GATTAGATTGAATAAATTTGCTTAATTATAATATACCTGACAAAAAATTGCCCCTGCAAT   74
m150505_iid=3784017_0      ------------------------------------------------------------   0
m141101_iid=682151_0       ------------------------------------------------------------   0
m150501_iid=3748029_0      ------------------------------------------------------------   0
m150504_iid=2876272_0      GATTAGATTGAATAAA-TTGCTTAATTAT-AATAACCTGACAAAAAATTGCCCCTGCAAT   355
NC_042820.1                GATTAGATTGAATAAA-TTGCTTAATTAT-AATAACCTGACAAAAAATTGCCCCTGCAAT   595
m141102_iid=1228825_0      GATTAGATTGAATAAA-TTGCTTAATTAT-AATAACCTGACAAAAAATTGCCCCTGCAAT   431
m150504_iid=2876274_0      GATTAGATTGAATAAA-TTGCTTAATTAT-AATAACCTGACAAAAAATTGCCCCTGCAAT   425
m141030_iid=80036_0        ATTAGGATTAATTAAA-TTGCTTAATTA--AATAACCTGACAAAAAATTGCCCCTGCAAT   194
m150506_iid=2596494_0      GATTAGATTGAATAAA-TTGCTTAATTAT-AATAACCTGACAAAAAATTGCCCCTGCAAT   591
m150503_iid=2526862_0      GATTAGATTGAATAAA-TTGCTTAATTAT-AATAACCTGACAAAAAATTGCCCCTGCAAT   277
m150506_iid=2567000_0      ------------------------------------------------------------   0
```

```
m150507_iid=3073271_0      ------------------------------------------------------------   0
m150504_iid=3156087_0      ------------------------------------------------------------   0
m150505_iid=2498502_0      ------------------------------------------------------------   0
m141102_iid=754227_0       ------------------------------------------------------------   0
m150507_iid=3469514_0      GATTAGATTGAATAAA-TTGCTTAATTAT-AATAACCTGACAAAAAATTGCCCCTGCAAT   120
Rdo_MitoGenome             GATTAGATTGAATAAA-TTGCTTAATTAT-AATAACCTGACAAAAAATTGCCCCTGCAAT   595
m141101_iid=614933_0       ------------------------------------------------------------   0
```

nad2

```
m141031_iid=347546_0       ------------------------------------------------------------   0
m150506_iid=3127414_0      ------------------------------------------------------------   0
m141102_iid=1245517_0      ------------------------------------------------------------   0
m141107_iid=1405727_0      ------------------------------------------------------------   0
m141102_iid=987614_0       ------------------------------------------------------------   0
m141108_iid=1701429_0      ------------------------------------------------------------   0
m141102_iid=987286_0       ------------------------------------------------------------   0
m150430_iid=3272125_0      ------------------------------------------------------------   0
m141102_iid=754225_0       ------------------------------------------------------------   0
m141107_iid=1409934_0      AATTCTAATATCATCAATAAATATAAACATATAACACATTTATTTACATTAC-ATTACGT   99
m150503_iid=3416920_0      ------------------------------------------------------------   0
m141108_iid=1800121_0      ------------------------------------------------------------   0
m141031_iid=147540_0       ------------------------------------------------------------   0
m150507_iid=3674924_0      ------------------------------------------------------------   0
m150503_iid=2923751_0      ------------------------------------------------------------   0
m141031_iid=115118_0       ------------------------------------------------------------   0
m150430_iid=2722448_0      ------------------------------------------------------------   0
m150507_iid=3102647_0      ------------------------------------------------------------   0
m141108_iid=1811866_0      ------------------------------------------------------------   0
m150501_iid=2948495_0      ------------------------------------------------------------   0
m150504_iid=3238195_0      ------------------------------------------------------------   0
m150503_iid=3388511_0      ------------------------------------------------------------   0
m150505_iid=3777611_0      ------------------------------------------------------------   0
m150430_iid=2438833_0      ------------------------------------------------------------   0
m141108_iid=1698969_0      AATTCTAATATCATCAATAAATATAA-------ACACATTTATTTACATTACAATTACGT   309
m150430_iid=3059190_0      ------------------------------------------------------------   0
m141107_iid=1383067_0      ------------------------------------------------------------   0
m141101_iid=597008_0       ------------------------------------------------------------   0
m150506_iid=2596496_0      AATTCTAATATCATCAATAAATATAA-------ACACATTTATTTACATTACAATTACGT   641
m141102_iid=910051_0       AATTCTAATATCATCAATAAATATAA-------ACACATTTATTTACATTACAATTACGT   127
m150505_iid=3784017_0      ------------------------------------------------------------   0
m141101_iid=682151_0       ------------------------------------------------------------   0
m150501_iid=3748029_0      ------------------------------------------------------------   0
m150504_iid=2876272_0      AATTCTAATATCATCAATAAATATAA-------ACACATTTATTTACATTACAATTACGT   408
NC_042820.1                AATTCTAATATCATCAATAAATATAA-------ACACATTTATTTACATTACAATTACGT   648
m141102_iid=1228825_0      AATTCTAATATCATCAATAAATATAA-------ACACATTTATTTACATTACAATTACGT   484
m150504_iid=2876274_0      AATTCTAATATCATCAATAAATATAA-------ACACATTTATTTACATTACAATTACGT   478
m141030_iid=80036_0        AATTCTAATATCATCAAT-AATATAA-------ACACATTTATTTACATTACAATTACGT   246
m150506_iid=2596494_0      AATTCTAATATCATCAATAAATATAA-------ACACATTTATTTACATTACAATTACGT   644
m150503_iid=2526862_0      AATTCTAATATCATCAATAAATATAA-------ACACATTTATTTACATTACAATTACGT   330
m150506_iid=2567000_0      ------------------------------------------------------------   0
m150507_iid=3073271_0      ------------------------------------------------------------   0
m150504_iid=3156087_0      ------------------------------------------------------------   0
m150505_iid=2498502_0      ------------------------------------------------------------   0
m141102_iid=754227_0       ------------------------------------------------------------   0
m150507_iid=3469514_0      AATTCTAATATCATCAATAAATATAA-------ACACATTTATTTACATTACAATTACGT   173
Rdo_MitoGenome             AATTCTAATATCATCAATAAATATAA-------ACACATTTATTTACATTACAATTACGT   648
m141101_iid=614933_0       ------------------------------------------------------------   0
```

nad2

```
m141031_iid=347546_0       ------------------------------------------------------------   0
m150506_iid=3127414_0      ------------------------------------------------------------   0
m141102_iid=1245517_0      ------------------------------------------------------------   0
m141107_iid=1405727_0      ------------------------------------------------------------   0
m141102_iid=987614_0       ------------------------------------------------------------   0
m141108_iid=1701429_0      ------------------------------------------------------------   0
m141102_iid=987286_0       ------------------------------------------------------------   0
m150430_iid=3272125_0      ------------------------------------------------------------   0
m141102_iid=754225_0       ------------------------------------------------------------   0
m141107_iid=1409934_0      CATCAATATTAATCAGAGGAATTATAGGAGTA-AACCAGACAAGACTACGAAAAATCATA   158
m150503_iid=3416920_0      ------------------------------------------------------------   0
m141108_iid=1800121_0      ------------------------------------------------------------   0
```

```
m141031_iid=147540_0        ------------------------------------------------------------   0
m150507_iid=3674924_0       ------------------------------------------------------------   0
m150503_iid=2923751_0       ------------------------------------------------------------   0
m141031_iid=115118_0        ------------------------------------------------------------   0
m150430_iid=2722448_0       ------------------------------------------------------------   0
m150507_iid=3102647_0       ------------------------------------------------------------   0
m141108_iid=1811866_0       ------------------------------------------------------------   0
m150501_iid=2948495_0       ------------------------------------------------------------   0
m150504_iid=3238195_0       ------------------------------------------------------------   0
m150503_iid=3388511_0       ------------------------------------------------------------   0
m150505_iid=3777611_0       ------------------------------------------------------------   0
m150430_iid=2438833_0       ------------------------------------------------------------   0
m141108_iid=1698969_0       CATCAATATTAATCAGA--------TGGAGTA-AACCAGACAAGACTACGAAAAAA-ATA   359
m150430_iid=3059190_0       ------------------------------------------------------------   0
m141107_iid=1383067_0       ------------------------------------------------------------   0
m141101_iid=597008_0        ------------------------------------------------------------   0
m150506_iid=2596496_0       CATCAATATTAATCAGAGGAATTATAGGAGTA-AACCAGACAAGACTACGAAAAATCATA   700
m141102_iid=910051_0        CATCAATATTAATCAGAGGAATTATAGGAGTA-AACCAGACAAGACTACAAAAAATCATA   186
m150505_iid=3784017_0       ------------------------------------------------------------   0
m141101_iid=682151_0        ------------------------------------------------------------   0
m150501_iid=3748029_0       ------------------------------------------------------------   0
m150504_iid=2876272_0       CATCAATATTAATCAGAGGAATTATAGGAGTA-AACCAGACAAGACTACGAAAAATCATA   467
NC_042820.1                 CATCAATATTAATCAGAGGAATTATAGGAGTA-AACCAGACAAGACTACGAAAAATCATA   707
m141102_iid=1228825_0       CATCAATATTAATCAGAGGAATTATAGGAGTA-AACCAGACAAGACTACGAAAAATCATA   543
m150504_iid=2876274_0       CATCAATATTAATCAGAGGAATTATAGGAGTA-AACCAGACAAGACTACGAAAAATCATA   537
m141030_iid=80036_0         CATCAATATTAATCAGAGGAATTATAGGAGTA-AACCAGACAAGACTACGAAAAATCATA   305
m150506_iid=2596494_0       CATCAATATTAATCAGAGGAATTATAGGAGTA-AACCAGACAAGACTACGAAAAATCATA   703
m150503_iid=2526862_0       CATCAATATTAATCAGAGGAATTATAGGAGTA-AACCAGACAAGACTACGAAAAATCATA   389
m150506_iid=2567000_0       ------------------------------------------------------------   0
m150507_iid=3073271_0       ------------------------------------------------------------   0
m150504_iid=3156087_0       ------------------------------------------------------------   0
m150505_iid=2498502_0       ------------------------------------------------------------   0
m141102_iid=754227_0        ------------------------------------------------------------   0
m150507_iid=3469514_0       CATCAATATTAATCAGAGGAATTATAGGAGTAAAACCAGACAAGACTACGAAAAATCATA   233
Rdo_MitoGenome              CATCAATATTAATCAGAGGAATTATAGGAGTA-AACCAGACAAGACTACGAAAAATCATA   707
m141101_iid=614933_0        ------------------------------------------------------------   0
```

nad2

```
m141031_iid=347546_0        ------------------------------------------------------------   0
m150506_iid=3127414_0       ------------------------------------------------------------   0
m141102_iid=1245517_0       ------------------------------------------------------------   0
m141107_iid=1405727_0       ------------------------------------------------------------   0
m141102_iid=987614_0        ------------------------------------------------------------   0
m141108_iid=1701429_0       ------------------------------------------------------------   0
m141102_iid=987286_0        ------------------------------------------------------------   0
m150430_iid=3272125_0       ------------------------------------------------------------   0
m141102_iid=754225_0        ------------------------------------------------------------   0
m141107_iid=1409934_0       GCATATTCATCTATTAACCATATAGCATGAATAATAGCATCAATAAACAATGAAAAAATC   218
m150503_iid=3416920_0       ------------------------------------------------------------   0
m141108_iid=1800121_0       ------------------------------------------------------------   0
m141031_iid=147540_0        ------------------------------------------------------------   0
m150507_iid=3674924_0       ------------------------------------------------------------   0
m150503_iid=2923751_0       ------------------------------------------------------------   0
m141031_iid=115118_0        ------------------------------------------------------------   0
m150430_iid=2722448_0       ------------------------------------------------------------   0
m150507_iid=3102647_0       ------------------------------------------------------------   0
m141108_iid=1811866_0       ------------------------------------------------------------   0
m150501_iid=2948495_0       ------------------------------------------------------------   0
m150504_iid=3238195_0       ------------------------------------------------------------   0
m150503_iid=3388511_0       ------------------------------------------------------------   0
m150505_iid=3777611_0       ------------------------------------------------------------   0
m150430_iid=2438833_0       ------------------------------------------------------------   0
m141108_iid=1698969_0       GCATATTCATCTATTAACCATATAGCATGAATAATAGCATCAATAAACAATGAAAAAATC   419
m150430_iid=3059190_0       ------------------------------------------------------------   0
m141107_iid=1383067_0       ------------------------------------------------------------   0
m141101_iid=597008_0        ------------------------------------------------------------   0
m150506_iid=2596496_0       GCATATTCATCTATTAACCATATAGCATGAATAATAGCATCAATAAACAATGAAAAAATC   760
m141102_iid=910051_0        GCATATTCATCTATTAACCATATAGCATGAATAATAGCATCAATAAACAATGAAAAAATC   246
m150505_iid=3784017_0       ------------------------------------------------------------   0
m141101_iid=682151_0        ------------------------------------------------------------   0
m150501_iid=3748029_0       ------------------------------------------------------------   0
```

```
m150504_iid=2876272_0      GCATATTCATCTATTAACCATATAGCATGAATAATAGCATCAATAAACAATGAAAAAATC   527
NC_042820.1                GCATATTCATCTATTAACCATATAGCATGAATAATAGCATCAATAAACAATGAAAAAATC   767
m141102_iid=1228825_0      GCATATTCATCTATTAACCATATAGCATGAATAATAGCATCAATAAACAATGAAAAAATC   603
m150504_iid=2876274_0      GCATATTCATCTATTAACCATATAGCATGAATAATAGCATCAATAAACAATGAAAAAATC   597
m141030_iid=80036_0        GCATATTCATCTATTAACCATATAGCATGAATAATAGCATCAATAAACAATGAAAAAATC   365
m150506_iid=2596494_0      GCATATTCATCTATTAACCATATAGCATGAATAATAGCATCAATAAACAATGAAAAAATC   763
m150503_iid=2526862_0      GCATATTCATCTATTAACCATATAGCATGAATAATAGCATCAATAAACAATGAAAAAATC   449
m150506_iid=2567000_0      ------------------------------------------------------------   0
m150507_iid=3073271_0      ------------------------------------------------------------   0
m150504_iid=3156087_0      ------------------------------------------------------------   0
m150505_iid=2498502_0      ------------------------------------------------------------   0
m141102_iid=754227_0       ------------------------------------------------------------   0
m150507_iid=3469514_0      GCATAT--TTCTATTAACCATATAGCATGAATAATAGCATCAATAAACAATGAAAAAATC   291
Rdo_MitoGenome             GCATATTCATCTATTAACCATATAGCATGAATAATAGCATCAATAAACAATGAAAAAATC   767
m141101_iid=614933_0       ------------------------------------------------------------   0
```

nad2

```
m141031_iid=347546_0       ------------------------------------------------------------   0
m150506_iid=3127414_0      ------------------------------------------------------------   0
m141102_iid=1245517_0      ------------------------------------------------------------   0
m141107_iid=1405727_0      ------------------------------------------------------------   0
m141102_iid=987614_0       ------------------------------------------------------------   0
m141108_iid=1701429_0      ------------------------------------------------------------   0
m141102_iid=987286_0       ------------------------------------------------------------   0
m150430_iid=3272125_0      ------------------------------------------------------------   0
m141102_iid=754225_0       ------------------------------------------------------------   0
m141107_iid=1409934_0      TTTTACATTTACTTATCAATCTATATTACAATATCATCAATCTTCATTTTTACATTAAAA   278
m150503_iid=3416920_0      ------------------------------------------------------------   0
m141108_iid=1800121_0      ------------------------------------------------------------   0
m141031_iid=147540_0       ------------------------------------------------------------   0
m150507_iid=3674924_0      ------------------------------------------------------------   0
m150503_iid=2923751_0      ------------------------------------------------------------   0
m141031_iid=115118_0       ------------------------------------------------------------   0
m150430_iid=2722448_0      ------------------------------------------------------------   0
m150507_iid=3102647_0      ------------------------------------------------------------   0
m141108_iid=1811866_0      ------------------------------------------------------------   0
m150501_iid=2948495_0      ------------------------------------------------------------   0
m150504_iid=3238195_0      ------------------------------------------------------------   0
m150503_iid=3388511_0      ------------------------------------------------------------   0
m150505_iid=3777611_0      ------------------------------------------------------------   0
m150430_iid=2438833_0      ------------------------------------------------------------   0
m141108_iid=1698969_0      TTTTACATTTACTTATCAATCTATATTACAATATCATCAATCTTCATTTTTACATTAAAA   479
m150430_iid=3059190_0      ------------------------------------------------------------   0
m141107_iid=1383067_0      ------------------------------------------------------------   0
m141101_iid=597008_0       ------------------------------------------------------------   0
m150506_iid=2596496_0      TTTTACATTTACTTATCAATCTATATTACAATATCATCAATCTTCATTTTTACATTAAAA   820
m141102_iid=910051_0       TTTTACATTTACTTATCAATCTATATTACAATATCATCAATCTTCATTTTTACATTAAAA   306
m150505_iid=3784017_0      ------------------------------------------------------------   0
m141101_iid=682151_0       ------------------------------------------------------------   0
m150501_iid=3748029_0      ------------------------------------------------------------   0
m150504_iid=2876272_0      TTTTACATTTACTTATCAATCTATATTACAATATCATCAATCTTCATTTTTACATTAAAA   587
NC_042820.1                TTTTACATTTACTTATCAATCTATATTACAATATCATCAATCTTCATTTTTACATTAAAA   827
m141102_iid=1228825_0      TTTTACATTTACTTATCAATCTATATTACAATATCATCAATCTTCATTTTTACATTAAAA   663
m150504_iid=2876274_0      TTTTACATTTACTTATCAATCTATATTACAATATCATCAATCTTCATTTTTACATTAAAA   657
m141030_iid=80036_0        TTTTACATTTACTTATCAATCTATATTACAATATCATCAATCTTCATTTTTACATTAAAA   425
m150506_iid=2596494_0      TTTTACATTTACTTATCAATCTATATTACAATATCATCAATCTTCATTTTTACATTAAAA   823
m150503_iid=2526862_0      TTTTACATTTACTTATCAATCTATATTACAATATCATCAATCTTCATTTTTACATTAAAA   509
m150506_iid=2567000_0      ------------------------------------------------------------   0
m150507_iid=3073271_0      ------------------------------------------------------------   0
m150504_iid=3156087_0      ------------------------------------------------------------   0
m150505_iid=2498502_0      ------------------------------------------------------------   0
m141102_iid=754227_0       ------------------------------------------------------------   0
m150507_iid=3469514_0      TTTTACATTTACTTATCAATCTATATTACAATATCATCAATCTTCATTTTTACATTAAAA   351
Rdo_MitoGenome             TTTTACATTTACTTATCAATCTATATTACAATATCATCAATCTTCATTTTTACATTAAAA   827
m141101_iid=614933_0       ------------------------------------------------------------   0
```

nad2

```
m141031_iid=347546_0       ------------------------------------------------------------   0
m150506_iid=3127414_0      ------------------------------------------------------------   0
m141102_iid=1245517_0      ------------------------------------------------------------   0
m141107_iid=1405727_0      ------------------------------------------------------------   0
```

```
m141102_iid=987614_0       ------------------------------------------------------------   0
m141108_iid=1701429_0      ------------------------------------------------------------   0
m141102_iid=987286_0       ------------------------------------------------------------   0
m150430_iid=3272125_0      ------------------------------------------------------------   0
m141102_iid=754225_0       ------------------------------------------------------------   0
m141107_iid=1409934_0      AGAAATAACTCC-TATATATTTATCAAATAATAACAAGAAATCAAAGCCAAT-TATTAAA   336
m150503_iid=3416920_0      ------------------------------------------------------------   0
m141108_iid=1800121_0      ------------------------------------------------------------   0
m141031_iid=147540_0       ------------------------------------------------------------   0
m150507_iid=3674924_0      ------------------------------------------------------------   0
m150503_iid=2923751_0      ------------------------------------------------------------   0
m141031_iid=115118_0       ------------------------------------------------------------   0
m150430_iid=2722448_0      ------------------------------------------------------------   0
m150507_iid=3102647_0      ------------------------------------------------------------   0
m141108_iid=1811866_0      ------------------------------------------------------------   0
m150501_iid=2948495_0      ------------------------------------------------------------   0
m150504_iid=3238195_0      ------------------------------------------------------------   0
m150503_iid=3388511_0      ------------------------------------------------------------   0
m150505_iid=3777611_0      ------------------------------------------------------------   0
m150430_iid=2438833_0      ------------------------------------------------------------   0
m141108_iid=1698969_0      AGAAATAACT-CCTATATATTTATCAAATAATAACAAGAAATCAAAGCCCAATTATTAAA   538
m150430_iid=3059190_0      ------------------------------------------------------------   0
m141107_iid=1383067_0      ------------------------------------------------------------   0
m141101_iid=597008_0       ------------------------------------------------------------   0
m150506_iid=2596496_0      AGAAATAACTCCCTATATATTTATCAAATAATAACAAGAAATCAAAGCCCAATTATTAAA   880
m141102_iid=910051_0       AGAAATAACTCCCTATATATTTATCAAATAATAACAAGAAATCAAAGCCCAATTATTAAA   366
m150505_iid=3784017_0      ------------------------------------------------------------   0
m141101_iid=682151_0       ------------------------------------------------------------   0
m150501_iid=3748029_0      ------------------------------------------------------------   0
m150504_iid=2876272_0      AGAAATAACTCCCTATATATTTATCAAATAATAACAAGAAATCAAAGCCCAATTATTAAA   647
NC_042820.1                AGAAATAACTCCCTATATATTTATCAAATAATAACAAGAAATCAAAGCCCAATTATTAAA   887
m141102_iid=1228825_0      AGAAATAACTCCCTATATATTTATCAAATAATAACAAGAAATCAAAGCCCAATTATTAAA   723
m150504_iid=2876274_0      AGAAATAACTCCCTATATATTTATCAAATAATAACAAGAAATCAAAGCCCAATTATTAAA   717
m141030_iid=80036_0        AGAAATAACTCCCTATATATTTATCAAATAATAACAAGAAATCAAAGCCCAATTATTAAA   485
m150506_iid=2596494_0      AGAAATAACTCCCTATATATTTATCAAATAATAACAAGAAATCAAAGCCCAATTATTAAA   883
m150503_iid=2526862_0      AGAAATAACTCCCTATATATTTATCAAATAATAACAAGAAATCAAAGCCCAATTATTAAA   569
m150506_iid=2567000_0      ------------------------------------------------------------   0
m150507_iid=3073271_0      ------------------------------------------------------------   0
m150504_iid=3156087_0      ------------------------------------------------------------   0
m150505_iid=2498502_0      ------------------------------------------------------------   0
m141102_iid=754227_0       ------------------------------------------------------------   0
m150507_iid=3469514_0      AGAAATAACTCCCTATATATTTATCAAATAATAACAAGAAATCAAAGCCCAATTATTAAA   411
Rdo_MitoGenome             AGAAATAACTCCCTATATATTTATCAAATAATAACAAGAAATCAAAGCCCAATTATTAAA   887
m141101_iid=614933_0       ------------------------------------------------------------   0
```

nad2

```
m141031_iid=347546_0       ------------------------------------------------------------   0
m150506_iid=3127414_0      ------------------------------------------------------------   0
m141102_iid=1245517_0      ------------------------------------------------------------   0
m141107_iid=1405727_0      ------------------------------------------------------------   0
m141102_iid=987614_0       ------------------------------------------------------------   0
m141108_iid=1701429_0      ------------------------------------------------------------   0
m141102_iid=987286_0       ------------------------------------------------------------   0
m150430_iid=3272125_0      ------------------------------------------------------------   0
m141102_iid=754225_0       ------------------------------------------------------------   0
m141107_iid=1409934_0      AAAACATTTATTATCAATCTACTAAGAATAGGAGGATTGCCCCCTCTCCTAGGATTT-TT   395
m150503_iid=3416920_0      ------------------------------------------------------------   0
m141108_iid=1800121_0      ------------------------------------------------------------   0
m141031_iid=147540_0       ------------------------------------------------------------   0
m150507_iid=3674924_0      ------------------------------------------------------------   0
m150503_iid=2923751_0      ------------------------------------------------------------   0
m141031_iid=115118_0       ------------------------------------------------------------   0
m150430_iid=2722448_0      ------------------------------------------------------------   0
m150507_iid=3102647_0      ------------------------------------------------------------   0
m141108_iid=1811866_0      ------------------------------------------------------------   0
m150501_iid=2948495_0      ------------------------------------------------------------   0
m150504_iid=3238195_0      --------------------------------------------------------TTTT   4
m150503_iid=3388511_0      ------------------------------------------------------------   0
m150505_iid=3777611_0      ------------------------------------------------------------   0
m150430_iid=2438833_0      ------------------------------------------------------------   0
m141108_iid=1698969_0      AAAACATTTATTATCAATCTACTAAGAATAGGAGGATTG-CCCCTCTCCTAGGATTT-TT   596
```

```
m150430_iid=3059190_0      ------------------------------------------------------------   0
m141107_iid=1383067_0      ------------------------------------------------------------   0
m141101_iid=597008_0       ------------------------------------------------------------   0
m150506_iid=2596496_0      AAAACATTTATTATCAATCTACTAAGAATAGGAGGATTGCCCCCTCTCCTAGGATTT-TT   939
m141102_iid=910051_0       AAAACATTTATTATCAATCTACTAAGAATAGGAGGATTGCCCCCTCTCCTAGGATTT-TT   425
m150505_iid=3784017_0      ------------------------------------------------------------   0
m141101_iid=682151_0       ------------------------------------------------------------   0
m150501_iid=3748029_0      ------------------------------------------------------------   0
m150504_iid=2876272_0      AAAACATTTATTATCAATCTACTAAGAATAGGAGGATTGCCCCCTCTCCTAGGATTT-TT   706
NC_042820.1                AAAACATTTATTATCAATCTACTAAGAATAGGAGGATTGCCCCCTCTCCTAGGATTT-TT   946
m141102_iid=1228825_0      AAAACATTTATTATCAATCTACTAAGAATAGGAGGATTGCCCCCTCTCCTAGGATTT-TT   782
m150504_iid=2876274_0      AAAACATTTATTATCAATCTACTAAGAATAGGAGGATTGCCCCCTCTCCTAGGATTT-TT   776
m141030_iid=80036_0        AAAACATTTATTATCAATCTACTAAGAATAGGAGGATTGCCCCCTCTCCTAGGATTT-TT   544
m150506_iid=2596494_0      AAAACATTTATTATCAATCTACTAAGAATAGGAGGATTGCCCCCTCTCCTAGGATTT-TT   942
m150503_iid=2526862_0      AAAACATTTATTATCAATCTACTAAGAATAGGAGGATTG--CCCTCTCCTAGGATTT-TT   626
m150506_iid=2567000_0      ------------------------------------------------------------   0
m150507_iid=3073271_0      ------------------------------------------------------------   0
m150504_iid=3156087_0      ------------------------------------------------------------   0
m150505_iid=2498502_0      ------------------------------------------------------------   0
m141102_iid=754227_0       ------------------------------------------------------------   0
m150507_iid=3469514_0      AAAACATTTATTATCAATCTACTAAGAATAGGAGGATTGCCCCCTCTCCTAGGATTTTTT   471
Rdo_MitoGenome             AAAACATTTATTATCAATCTACTAAGAATAGGAGGATTGCCCCCTCTCCTAGGATTT-TT   946
m141101_iid=614933_0       ------------------------------------------------------------   0
```

nad2

```
m141031_iid=347546_0       ------------------------------------------------------------   0
m150506_iid=3127414_0      ------------------------------------------------------------   0
m141102_iid=1245517_0      ------------------------------------------------------------   0
m141107_iid=1405727_0      ------------------------------------------------------------   0
m141102_iid=987614_0       ------------------------------------------------------------   0
m141108_iid=1701429_0      ------------------------------------------------------------   0
m141102_iid=987286_0       ------------------------------------------------------------   0
m150430_iid=3272125_0      ------------------------------------------------------------   0
m141102_iid=754225_0       ------------------------------------------------------------   0
m141107_iid=1409934_0      TCCCAAATGAATAGTAATTAACAGAATAATCCAAAGAAACAATCTAATTTTAAGAACCTT   455
m150503_iid=3416920_0      ------------------------------------------------------------   0
m141108_iid=1800121_0      ------------------------------------------------------------   0
m141031_iid=147540_0       ------------------------------------------------------------   0
m150507_iid=3674924_0      ------------------------------------------------------------   0
m150503_iid=2923751_0      ------------------------------------------------------------   0
m141031_iid=115118_0       ------------------------------------------------------------   0
m150430_iid=2722448_0      ------------------------------------------------------------   0
m150507_iid=3102647_0      ------------------------------------------------------------   0
m141108_iid=1811866_0      ------------------------------------------------------------   0
m150501_iid=2948495_0      ------------------------------------------------------------   0
m150504_iid=3238195_0      TCCCAAATGAATAGTAATTAACAGAATAATCCAAAGAAACAATCTAATTTTAAGAACCTT   64
m150503_iid=3388511_0      ------------------------------------------------------------   0
m150505_iid=3777611_0      ------------------------------------------------------------   0
m150430_iid=2438833_0      ------------------------------------------------------------   0
m141108_iid=1698969_0      TCCCAAATGAATAGTAATTAACAGAATAATCCAAAGAAACAATCTAATTTTAAGAACCTT   656
m150430_iid=3059190_0      ------------------------------------------------------------   0
m141107_iid=1383067_0      ------------------------------------------------------------   0
m141101_iid=597008_0       ------------------------------------------------------------   0
m150506_iid=2596496_0      TCCCAAATGAATAGTAATTAACAGAATAATCCAAAGAAACAATCTAATTTTAAGAACCTT   999
m141102_iid=910051_0       TCCCAAATGAATAGTAATTAACAGAATAATCCAAAGAAACAATCTAATTTTAAGAACCTT   485
m150505_iid=3784017_0      ------------------------------------------------------------   0
m141101_iid=682151_0       ------------------------------------------------------------   0
m150501_iid=3748029_0      ------------------------------------------------------------   0
m150504_iid=2876272_0      TCCCAAATGAATAGTAATTAACAGAATAATCCAAAGAAACAATCTAATTTTAAGAACCTT   766
NC_042820.1                TCCCAAATGAATAGTAATTAACAGAATAATCCAAAGAAACGATCTGATTTTAAGAACCTT   1006
m141102_iid=1228825_0      TCCCAAATGAATAGTAATTAACAGAATAATCCAAAGAAACAATCTAATTTTAAGAACCTT   842
m150504_iid=2876274_0      TCCCAAATGAATAGTAATTAACAGAATAATCCAAAGAAACAATCTAATTTTAAGAACCTT   836
m141030_iid=80036_0        TCCCAAATGAATAGTAATTAACAGAATAATCCAAAGAAACAATCTAATTTTAAGAACCTT   604
m150506_iid=2596494_0      TCCCAAATGAATAGTAATTAACAGAATAATCCAAAGAAACAATCTAATTTTAAGAACCTT   1002
m150503_iid=2526862_0      TCCCAAATGAATAGTAATTAACAGAATAATCCAAAGAAACAATCTAATTTTAAGAACCTT   686
m150506_iid=2567000_0      ------------------------------------------------------------   0
m150507_iid=3073271_0      ------------------------------------------------------------   0
m150504_iid=3156087_0      ------------------------------------------------------------   0
m150505_iid=2498502_0      ------------------------------------------------------------   0
m141102_iid=754227_0       ------------------------------------------------------------   0
m150507_iid=3469514_0      TCCCAAATGAATAGTAATTAACAGAATAATCCAAAGAAACAATCTAATTTTAAGAACCTT   531
```

```
Rdo_MitoGenome            TCCCAAATGAATAGTAATTAACAGAATAATCCAAAGAAACAATCTAATTTTAAGAACCTT   1006
m141101_iid=614933_0      ------------------------------------------------------------   0

                                                nad2

m141031_iid=347546_0      ------------------------------------------------------------   0
m150506_iid=3127414_0     ------------------------------------------------------------   0
m141102_iid=1245517_0     ------------------------------------------------------------   0
m141107_iid=1405727_0     ------------------------------------------------------------   0
m141102_iid=987614_0      ------------------------------------------------------------   0
m141108_iid=1701429_0     ------------------------------------------------------------   0
m141102_iid=987286_0      ------------------------------------------------------------   0
m150430_iid=3272125_0     ------------------------------------------------------------   0
m141102_iid=754225_0      ------------------------------------------------------------   0
m141107_iid=1409934_0     CATAGTAATAACAACATTATTAACACTATACTTTTATATACAAATTTCAATACCAATCCT   515
m150503_iid=3416920_0     ------------------------------------------------------------   0
m141108_iid=1800121_0     ------------------------------------------------------------   0
m141031_iid=147540_0      ------------------------------------------------------------   0
m150507_iid=3674924_0     ------------------------------------------------------------   0
m150503_iid=2923751_0     ------------------------------------------------------------   0
m141031_iid=115118_0      ------------------------------------------------------------   0
m150430_iid=2722448_0     ------------------------------------------------------------   0
m150507_iid=3102647_0     ------------------------------------------------------------   0
m141108_iid=1811866_0     ------------------------------------------------------------   0
m150501_iid=2948495_0     ------------------------------------------------------------   0
m150504_iid=3238195_0     CATAGTAATAACAACATTATTAACACTATACTTTTATATACAAATTTCAATACCAATCCT   124
m150503_iid=3388511_0     ------------------------------------------------------------   0
m150505_iid=3777611_0     ------------------------------------------------------------   0
m150430_iid=2438833_0     ------------------------------------------------------------   0
m141108_iid=1698969_0     CATAGTAATAACAACATTATTAACACTATACTTTTATATACAAATTTCAATACCAATCCT   716
m150430_iid=3059190_0     ------------------------------------------------------------   0
m141107_iid=1383067_0     ------------------------------------------------------------   0
m141101_iid=597008_0      ------------------------------------------------------------   0
m150506_iid=2596496_0     CATAGTAATAACAACATTATTAACACTATACTTTTATATACAAATTTCAATACCAATCCT   1059
m141102_iid=910051_0      CATAGTAATAACAACATTATTAACACTATACTTTTATATACAAATTTCAATACCAATCCT   545
m150505_iid=3784017_0     ------------------------------------------------------------   0
m141101_iid=682151_0      ------------------------------------------------------------   0
m150501_iid=3748029_0     ------------------------------------------------------------   0
m150504_iid=2876272_0     CATAGTAATAACAACATTATTAACACTATACTTTTATATACAAATTTCAATACCAATCCT   826
NC_042820.1               CATAGTAATAACAACATTATTAACACTATACTTCTATATACAAATTTCAATACCAATCCT   1066
m141102_iid=1228825_0     CATAGTAATAACAACATTATTAACACTATACTTTTATATACAAATTTCAATACCAATCCT   902
m150504_iid=2876274_0     CATAGTAATAACAACATTATTAACACTATACTTTTATATACAAATTTCAATACCAATCCT   896
m141030_iid=80036_0       CATAGTAATAACAACATTATTAACACTATACTTTTATATACAAATTTCAATACCAATCCT   664
m150506_iid=2596494_0     CATAGTAATAACAACATTATTAACACTATACTTTTATATACAAATTTCAATACCAATCCT   1062
m150503_iid=2526862_0     CATAGTAATAACAACATTATTAACACTATACTTTTATATACAAATTTCAATACCAATCCT   746
m150506_iid=2567000_0     ------------------------------------------------------------   0
m150507_iid=3073271_0     ------------------------------------------------------------   0
m150504_iid=3156087_0     ------------------------------------------------------------   0
m150505_iid=2498502_0     ------------------------------------------------------------   0
m141102_iid=754227_0      -------------------TTAACACTA--ACTTTTAATACAAATTTCAATACCAATCCT   39
m150507_iid=3469514_0     CATAGTAATAACAACATTATTAACACTATACTTTTATATACAAATTTCAATACCAATCCT   591
Rdo_MitoGenome            CATAGTAATAACAACATTATTAACACTATACTTTTATATACAAATTTCAATACCAATCCT   1066
m141101_iid=614933_0      ------------------------------------------------------------   0

                                                nad2

m141031_iid=347546_0      ------------------------------------------------------------   0
m150506_iid=3127414_0     ------------------------------------------------------------   0
m141102_iid=1245517_0     ------------------------------------------------------------   0
m141107_iid=1405727_0     ------------------------------------------------------------   0
m141102_iid=987614_0      ------------------------------------------------------------   0
m141108_iid=1701429_0     ------------------------------------------------------------   0
m141102_iid=987286_0      ------------------------------------------------------------   0
m150430_iid=3272125_0     ------------------------------------------------------------   0
m141102_iid=754225_0      -----------------AAAATAAAAATAAAATGAAGGAAAAA-AACCCC---CAATTCT   39
m141107_iid=1409934_0     TACAATGTATAAAATAAAAA-------TAAAATGAAGGAAAA-AAAACCCCAACAATTCT   567
m150503_iid=3416920_0     ------------------------------------------------------------   0
m141108_iid=1800121_0     ------------------------------------------------------------   0
m141031_iid=147540_0      ------------------------------------------------------------   0
m150507_iid=3674924_0     ------------------------------------------------------------   0
m150503_iid=2923751_0     ------------------------------------------------------------   0
m141031_iid=115118_0      ------------------------------------------------------------   0
m150430_iid=2722448_0     ------------------------------------------------------------   0
```

```
m150507_iid=3102647_0      ------------------------------------------------------------   0
m141108_iid=1811866_0      ------------------------------------------------------------   0
m150501_iid=2948495_0      ------------------------------------------------------------   0
m150504_iid=3238195_0      TACAATGTATAAAAATAAA-------ATAAAATGAAGGAAAA-AAAACCCCAACAATTCT   176
m150503_iid=3388511_0      ------------------------------------------------------------   0
m150505_iid=3777611_0      ------------------------------------------------------------   0
m150430_iid=2438833_0      ------------------------------------------------------------   0
m141108_iid=1698969_0      TACAATGTATAAAATAAAAA-------TAAAATGAAGGAAAAAAA--CCCCAACAATTCT   767
m150430_iid=3059190_0      ------------------------------------------------------------   0
m141107_iid=1383067_0      ------------------------------------------------------------   0
m141101_iid=597008_0       ------------------------------------------------------------   0
m150506_iid=2596496_0      TACAATGTATAAAATAAA-------AATAAAATGAAGGAAAAAAAAACCCCCAACAATTCT   1112
m141102_iid=910051_0       TACAATGTATAAATAAAAATAAAAATAAAAAATGAAGGA---AAAAACCCCCACAATTCT   602
m150505_iid=3784017_0      ------------------------------------------------------------   0
m141101_iid=682151_0       ------------------------------------------------------------   0
m150501_iid=3748029_0      ------------------------------------------------------------   0
m150504_iid=2876272_0      TACAATGTATAAAATAAAAA-------AAAAT--GAAGGAAAAAAAACCCCCAACAATTCT   877
NC_042820.1                TACAATGTATAAAATAAAAA-------TAAAATGAAGGAAAAAAAAACCCCCAACAATTCT   1119
m141102_iid=1228825_0      TACAATGTATAAAATAAAAA-------TAAAATGAAGG-AAAAAAACCCCCAACAATTCT   954
m150504_iid=2876274_0      TACAATGTATAAAATAAAAA-------TAAAATGAAGGAAAAAAAAACCCCCAACAATTCT   949
m141030_iid=80036_0        TACAATGTATAAAATAAAAA-------TAAAATGAAGGA--AAAAAACCCCAACAATTCT   715
m150506_iid=2596494_0      TACAATGTATAAAATAAAAA-------TAAAATGAAGGAA-AAAAACCCCCAACAATTCT   1114
m150503_iid=2526862_0      TACAATGTATAAAA-AAAAA-------TAAAATGAAGGAAAAAAAAACCCCCAACAATTCT   798
m150506_iid=2567000_0      ------------------------------------------------------------   0
m150507_iid=3073271_0      TACAATGTATAAAATAAAA--------TAAAATGAAGGAAAAAAAAACC-CCAACAATTCT   51
m150504_iid=3156087_0      ------------------------------------------------------------   0
m150505_iid=2498502_0      ------------------------------------------------------------   0
m141102_iid=754227_0       TACAATGTATAAAATAAAAA-------TAAAATGAAGGAAAAAAAAACCCCCAACAATTCT   92
m150507_iid=3469514_0      TACAATGTATAAAATAAAAA-------TAAAATGAAGGAAAAAA-AACCCCAACAATTCT   643
Rdo_MitoGenome             TACAATGTATAAAATAAAAA-------TAAAATGAAGGAAAAAAAAACCCCCAACAATTCT   1119
m141101_iid=614933_0       ------------------------------------------------------------   0
```

nad2

```
m141031_iid=347546_0       ------------------------------------------------------------   0
m150506_iid=3127414_0      ------------------------------------------------------------   0
m141102_iid=1245517_0      ------------------------------------------------------------   0
m141107_iid=1405727_0      ------------------------------------------------------------   0
m141102_iid=987614_0       ------------------------------------------------------------   0
m141108_iid=1701429_0      ------------------------------------------------------------   0
m141102_iid=987286_0       ------------------------------------------------------------   0
m150430_iid=3272125_0      ------------------------------------------------------------   0
m141102_iid=754225_0       TCATAAATAAAATAAACATAATCAATCTGCTAAGAATTCCTGTGTGTACAATTTGATTT-   98
m141107_iid=1409934_0      TCATAAATAAAATAAACATAATCAATCTGCTAAGAATTCCTGTGTGTACAATTTGATTTA   627
m150503_iid=3416920_0      ------------------------------------------------------------   0
m141108_iid=1800121_0      ------------------------------------------------------------   0
m141031_iid=147540_0       ------------------------------------------------------------   0
m150507_iid=3674924_0      ------------------------------------------------------------   0
m150503_iid=2923751_0      ------------------------------------------------------------   0
m141031_iid=115118_0       ------------------------------------------------------------   0
m150430_iid=2722448_0      ------------------------------------------------------------   0
m150507_iid=3102647_0      ------------------------------------------------------------   0
m141108_iid=1811866_0      ------------------------------------------------------------   0
m150501_iid=2948495_0      ------------------------------------------------------------   0
m150504_iid=3238195_0      TCATAAATA-AATAAACATAATCAATCTGCTAAGAATTCCTGTGTGTACAATTTGATTTA   235
m150503_iid=3388511_0      ------------------------------------------------------------   0
m150505_iid=3777611_0      ------------------------------------------------------------   0
m150430_iid=2438833_0      ------------------------------------------------------------   0
m141108_iid=1698969_0      TCATAAATAAAATAAACATAATCAATCTGCTAAGAATTCCTGTGTGTACAATTTGATTTA   827
m150430_iid=3059190_0      ------------------------------------------------------------   0
m141107_iid=1383067_0      ------------------------------------------------------------   0
m141101_iid=597008_0       ------------------------------------------------------------   0
m150506_iid=2596496_0      TCATAAATAAAATAAACATAATCAATCTGCTAAGAATTCCTGTGTGTACAATTTGATTTA   1172
m141102_iid=910051_0       TCATAAATAAAATAAACATAATCAATCTGCTAAGAATTCCTGTGTGTACAATTTGATTTA   662
m150505_iid=3784017_0      ------------------------------------------------------------   0
m141101_iid=682151_0       ------------------------------------------------------------   0
m150501_iid=3748029_0      ------------------------------------------------------------   0
m150504_iid=2876272_0      TCATAAATAAAATAAACATAATCAATCTGCTAAGAATTCCTGTGTGTACAATTTGATTTA   937
NC_042820.1                TCATAAATAAAATAAACATAATCAATCTGCTAAGAATTCCTGTATGTACAATTTGATTTA   1179
m141102_iid=1228825_0      TCAT-----AAATAAACATAATCAATCTGCTAAGAATTCCTGTGTGTACAATTTGATTTA   1009
m150504_iid=2876274_0      TCATAAATAAAATAAACATAATCAATCTGCTAAGAATTCCTGTGTGTACAATTTGATTTA   1009
m141030_iid=80036_0        TCATAAATAAAATAAACATAATCAATCTGCTAAGAATTCCTGTGTGTACAATTTGATTTA   775
```

```
m150506_iid=2596494_0      TCATAAATAAAATAAACATAATCAATCTGCTAAGAATTCCTGTGTGTACAATTTGATTTA   1174
m150503_iid=2526862_0      TCATAAATAAAATAAACATAATCAATCTGCTAAGAATTCCTGTGTGTACAATTTGATTTA   858
m150506_iid=2567000_0      ------------------------------------------------------------   0
m150507_iid=3073271_0      TCATAAATAAAATAAACATAATCAATCTGCTAAGAATTCCTGTGTGTACAATTTGATTTA   111
m150504_iid=3156087_0      ------------------------------------------------------------   0
m150505_iid=2498502_0      ------------------------------------------------------------   0
m141102_iid=754227_0       TCATAAATAAAATAAACATAATCAATCTGCTAAGAATTCCTGTGTGTACAATTTGATTTA   152
m150507_iid=3469514_0      TCATAAATAAAATAAACATAATCAATCTGCTAAGAATTCCTGTGTGTACAATTTGATTTA   703
Rdo_MitoGenome             TCATAAATAAAATAAACATAATCAATCTGCTAAGAATTCCTGTGTGTACAATTTGATTTA   1179
m141101_iid=614933_0       ------------------------------------------------------------   0
```

nad2

```
m141031_iid=347546_0       ------------------------------------------------------------   0
m150506_iid=3127414_0      ------------------------------------------------------------   0
m141102_iid=1245517_0      ------------------------------------------------------------   0
m141107_iid=1405727_0      ------------------------------------------------------------   0
m141102_iid=987614_0       ------------------------------------------------------------   0
m141108_iid=1701429_0      ------------------------------------------------------------   0
m141102_iid=987286_0       ------------------------------------------------------------   0
m150430_iid=3272125_0      ------------------------------------------------------------   0
m141102_iid=754225_0       ACTTTTATTAAGGATTTAAGTTAAATCAAACTAACAGCCTTCAAGAGCTGTAAATAGCAA   158
m141107_iid=1409934_0      ACTTTTATTAAGGATTTAAGTTAAATCAAACTAACAGCCTTCAAAGCTGTAAATAAGCAA   687
m150503_iid=3416920_0      ------------------------------------------------------------   0
m141108_iid=1800121_0      ------------------------------------------------------------   0
m141031_iid=147540_0       ------------------------------------------------------------   0
m150507_iid=3674924_0      ------------------------------------------------------------   0
m150503_iid=2923751_0      ------------------------------------------------------------   0
m141031_iid=115118_0       ------------------------------------------------------------   0
m150430_iid=2722448_0      ------------------------------------------------------------   0
m150507_iid=3102647_0      ------------------------------------------------------------   0
m141108_iid=1811866_0      ------------------------------------------------------------   0
m150501_iid=2948495_0      ------------------------------------------------------------   0
m150504_iid=3238195_0      ACTTTTATTAAGGATTTAAGTTAAATCAAACTAACAGCCTTCAAAGCTGTAA-AAAGCAA   294
m150503_iid=3388511_0      ------------------------------------------------------------   0
m150505_iid=3777611_0      ------------------------------------------------------------   0
m150430_iid=2438833_0      ------------------------------------------------------------   0
m141108_iid=1698969_0      ACTTTTATTAAGGATTTAAGTTAAATCAAACTAACAGCCTTCAAAGCTGTAAATAAGCAA   887
m150430_iid=3059190_0      ------------------------------------------------------------   0
m141107_iid=1383067_0      ------------------------------------------------------------   0
m141101_iid=597008_0       ------------------------------------------------------------   0
m150506_iid=2596496_0      ACTTTTATTAAGGATTTAAGTTAAATCAAACTAACAGCCTTCAAAGCTGTAAATAAGCAA   1232
m141102_iid=910051_0       ACTTTTATTAAGGATTTAAGTTAAATCAAACTAACAGCCTTCAAAGCTGTAAATAAGCAA   722
m150505_iid=3784017_0      ------------------------------------------------------------   0
m141101_iid=682151_0       ------------------------------------------------------------   0
m150501_iid=3748029_0      ------------------------------------------------------------   0
m150504_iid=2876272_0      ACTTTTATTAAGGATTTAAGTTAAATCAAACTAACAGCCTTCAAAGCTGTAAATAAGCAA   997
NC_042820.1                ACTTTTATTAAGGATTTAAGTTAAATCAAACTAACAGCCTTCAAAGCTGTAAATAAGCAA   1239
m141102_iid=1228825_0      ACTTTTATTAAGGATTTAAGTTAAATCAAACTAACAGCCTTCAAAGCTGTAAATAAGCAA   1069
m150504_iid=2876274_0      ACTTTTATTAAGGATTTAAGTTAAATCAAACTAACAGCCTTCAAAGCTGTAAATAAGCAA   1069
m141030_iid=80036_0        ACTTTTATTAAGGATTTAAGTTAAATCAAACTAACAGCCTTCAAAGCTGTAAATAAGCAA   835
m150506_iid=2596494_0      ACTTTTATTAAGGATTTAAGTTAAATCAAACTAACAGCCTTCAAAGCTGTAAATAAGCAA   1234
m150503_iid=2526862_0      ACTTTTATTAAGGATTTAAGTTAAATCAAACTAACAGCCTTCAAAGCTGTAAATAAGCAA   918
m150506_iid=2567000_0      ------------------------------------------------------------   0
m150507_iid=3073271_0      ACTTTTATTAAGGATTTAAGTTAAATCAAACTAACAGCCTTCAAAGCTGTAAATAAGCAA   171
m150504_iid=3156087_0      ------------------------------------------------------------   0
m150505_iid=2498502_0      ------------------------------------------------------------   0
m141102_iid=754227_0       ACTTTTATTAAGGATTTAAGTTAAATCAAACTAACAGCCTTCAAAGCTGTAAATAAGCAA   212
m150507_iid=3469514_0      ACTTTTATTAAGGATTTAAGTTAAATCAAACTAACAGCCTTCAAAGCTGTAAATAAGCAA   763
Rdo_MitoGenome             ACTTTTATTAAGGATTTAAGTTAAATCAAACTAACAGCCTTCAAAGCTGTAAATAAGCAA   1239
m141101_iid=614933_0       ------------------------------------------------------------   0
```

nad2

```
m141031_iid=347546_0       ------------------------------------------------------------   0
m150506_iid=3127414_0      ------------------------------------------------------------   0
m141102_iid=1245517_0      ------------------------------------------------------------   0
m141107_iid=1405727_0      ------------------------------------------------------------   0
m141102_iid=987614_0       ------------------------------------------------------------   0
m141108_iid=1701429_0      ------------------------------------------------------------   0
m141102_iid=987286_0       ------------------------------------------------------------   0
m150430_iid=3272125_0      ------------------------------------------------------------   0
m141102_iid=754225_0       ATGTTTAAGCCTTAGAGCTTAACTCACCTTTAAATTTGCAATTTAAAATCATTTTGAATA   218
```

```
m141107_iid=1409934_0      ATGTTTAAGCCTTAGAGCTTAACTCACCTTTAAATTTGCAATTTAAAATCATTTTGAATA   747
m150503_iid=3416920_0      ------------------------------------------------------------   0
m141108_iid=1800121_0      ------------------------------------------------------------   0
m141031_iid=147540_0       ------------------------------------------------------------   0
m150507_iid=3674924_0      ------------------------------------------------------------   0
m150503_iid=2923751_0      ------------------------------------------------------------   0
m141031_iid=115118_0       ------------------------------------------------------------   0
m150430_iid=2722448_0      ------------------------------------------------------------   0
m150507_iid=3102647_0      ------------------------------------------------------------   0
m141108_iid=1811866_0      ------------------------------------------------------------   0
m150501_iid=2948495_0      ------------------------------------------------------------   0
m150504_iid=3238195_0      ATGTTTAAGCCTTAGAGCTTAACTCACCTTTAAATTTGCAATTTAAAATCATTTTGAATA   354
m150503_iid=3388511_0      ------------------------------------------------------------   0
m150505_iid=3777611_0      ------------------------------------------------------------   0
m150430_iid=2438833_0      ------------------------------------------------------------   0
m141108_iid=1698969_0      ATGTTTAAGCCTTAGAGCTTAACTCACCTTTAAATTTGCAATTTAAAATCATTTTGAATA   947
m150430_iid=3059190_0      ------------------------------------------------------------   0
m141107_iid=1383067_0      ------------------------------------------------------------   0
m141101_iid=597008_0       ------------------------------------------------------------   0
m150506_iid=2596496_0      ATGTTTAAGCCTTAGAGCTTAACTCACCTTTAAATTTGCAATTTAAAATCATTTTGAATA   1292
m141102_iid=910051_0       ATGTTTAAGCCTTAGAGCTTAACTCACCTTTAAATTTGCAATTTAAAATCATTTTGAATA   782
m150505_iid=3784017_0      ------------------------------------------------------------   0
m141101_iid=682151_0       ------------------------------------------------------------   0
m150501_iid=3748029_0      ------------------------------------------------------------   0
m150504_iid=2876272_0      ATGTTTAAGCCTTAGAGCTTAACTCACCTTTAAATTTGCAATTTAAAATCATTTTGAATA   1057
NC_042820.1                ATGTTTAAGCCTTAGAGCTTAACTCACCTTTAAATTTGCAATTTAAAATCATTTTGAATA   1299
m141102_iid=1228825_0      ATGTTTAAGCCTTAGAGCTTAACTCACCTTTAAATTTGCAATTTAAAATCATTTTGAATA   1129
m150504_iid=2876274_0      ATGTTTAAGCCTTAGAGCTTAACTCACCTTTAAATTTGCAATTTAAAATCATTTTGAATA   1129
m141030_iid=80036_0        ATGTTTAAGCCTTAGAGCTTAACTCACCTTTAAATTTGCAATTTAAAATCATTTTGAATA   895
m150506_iid=2596494_0      ATGTTTAAGCCTTAGAGCTTAACTCACCTTTAAATTTGCAATTTAAAATCATTTTGAATA   1294
m150503_iid=2526862_0      ATGTTTAAGCCTTAGAGCTTAACTCACCTTTAAATTTGCAATTTAAAATCATTTTGAATA   978
m150506_iid=2567000_0      ------------------------------------------------------------   0
m150507_iid=3073271_0      ATGTTTAAGCCTTAGAGCTTAACTCACCTTTAAATTTGCAATTTAAAATCATTTTGAATA   231
m150504_iid=3156087_0      ------AAGCCTTAGAGCTTAACTCACCTTTAAATTTGCAATTTAAAATCATTTTGAATA   54
m150505_iid=2498502_0      ------------------------------------------------------------   0
m141102_iid=754227_0       ATGTTTAAGCCTTAGAGCTTAACTCACCTTTAAATTTGCAATTTAAAATCATTTTGAATA   272
m150507_iid=3469514_0      ATGTTTAAGCCTTAGAGCTTAACTCACCTTTAAATTTGCAATTTAAAATCATTTTGAATA   823
Rdo_MitoGenome             ATGTTTAAGCCTTAGAGCTTAACTCACCTTTAAATTTGCAATTTAAAATCATTTTGAATA   1299
m141101_iid=614933_0       ------------------------------------------------------------   0


m141031_iid=347546_0       ------------------------------------------------------------   0
m150506_iid=3127414_0      ------------------------------------------------------------   0
m141102_iid=1245517_0      ------------------------------------------------------------   0
m141107_iid=1405727_0      ------------------------------------------------------------   0
m141102_iid=987614_0       ------------------------------------------------------------   0
m141108_iid=1701429_0      ------------------------------------------------------------   0
m141102_iid=987286_0       ------------------------------------------------------------   0
m150430_iid=3272125_0      ------------------------------------------------------------   0
m141102_iid=754225_0       TAAGACCGATGAGAAGGAAAA-CCCGTAAATAGATTTACAATCTAACGCTTAAACTCAGC   277
m141107_iid=1409934_0      TAAGACCGATGAGAAGGAAAA-CCCGTAAATAGATTTACAATCTAACGCTTAAACTCAGC   806
m150503_iid=3416920_0      ------------------------------------------------------------   0
m141108_iid=1800121_0      ------------------------------------------------------------   0
m141031_iid=147540_0       ------------------------------------------------------------   0
m150507_iid=3674924_0      ------------------------------------------------------------   0
m150503_iid=2923751_0      ------------------------------------------------------------   0
m141031_iid=115118_0       ------------------------------------------------------------   0
m150430_iid=2722448_0      ------------------------------------------------------------   0
m150507_iid=3102647_0      ------------------------------------------------------------   0
m141108_iid=1811866_0      ------------------------------------------------------------   0
m150501_iid=2948495_0      ------------------------------------------------------------   0
m150504_iid=3238195_0      TAAGACCGATGAGAAGGAAAACCCCGTAAATAGATTTACAATCTAACGCTTAAACTCAGC   414
m150503_iid=3388511_0      ------------------------------------------------------------   0
m150505_iid=3777611_0      ------------------------------------------------------------   0
m150430_iid=2438833_0      ------------------------------------------------------------   0
m141108_iid=1698969_0      TAAGACCGATGAGAAGGAAAACCCCGTAAATAGATTTACAATCTAACGCTTAAACTCAGC   1007
m150430_iid=3059190_0      ------------------------------------------------------------   0
m141107_iid=1383067_0      ------------------------------------------------------------   0
m141101_iid=597008_0       ------------------------------------------------------------   0
m150506_iid=2596496_0      TAAGACCGATGAGAAGGAAAACCCCGTAAATAGATTTACAATCTAACGCTTAAACTCAGC   1352
m141102_iid=910051_0       TAAGACCGATGAGAAGGAAAACCCCGTAAATAGATTTACAATCTAACGCTTAAACTCAGC   842
```

```
m150505_iid=3784017_0      ------------------------------------------------------------   0
m141101_iid=682151_0       ------------------------------------------------------------   0
m150501_iid=3748029_0      ------------------------------------------------------------   0
m150504_iid=2876272_0      TAAGACCGATGAGAAGGAAAACCCCGTAAATAGATTTACAATCTAACGCTTAAACTCAGC   1117
NC_042820.1                TAAGACCGATGAGAAGGAAAACCCCGTAAATAGATTTACAATCTAACGCTTAAACTCAGC   1359
m141102_iid=1228825_0      TAAGACCGATGAGAAGGAAAACCCCGTAAATAGATTTACAATCTAACGCTTAAACTCAGC   1189
m150504_iid=2876274_0      TAAGACCGATGAGAAGGAAAACCCCGTAAATAGATTTACAATCTAACGCTTAAACTCAGC   1189
m141030_iid=80036_0        TAAGACCGATGAGAAGGAAAACCCCGTAAATAGATTTACAATCTAACGCTTAAACTCAGC   955
m150506_iid=2596494_0      TAAGACCGATGAGAAGGAAAACCCCGTAAATAGATTTACAATCTAACGCTTAAACTCAGC   1354
m150503_iid=2526862_0      TAAGACCGATGAGAAGGAAAACCCCGTAAATAGATTTACAATCTAACGCTTAAACTCAGC   1038
m150506_iid=2567000_0      ------------------------------------------------------------   0
m150507_iid=3073271_0      TAAGACCGATGAGAAGGAAAACCCCGTAAATAGATTTACAATCTAACGCTTAAACTCAGC   291
m150504_iid=3156087_0      TAAGACCGATGAGAAGGAAAACCCCGTAAATAGATTTACAATCTAACGCTTAAACTCAGC   114
m150505_iid=2498502_0      ------------------------------------------------------------   0
m141102_iid=754227_0       TAAGACCGATGAGAAGGAAAA-CCCGTAAATAGATTTACAATCTAACGCTTAAACTCAGC   331
m150507_iid=3469514_0      TAAGACCGATGAGAAGGAAAACCCCGTAAATAGATTTACAATCTAACGCTTAAACTCAGC   883
Rdo_MitoGenome             TAAGACCGATGAGAAGGAAAACCCCGTAAATAGATTTACAATCTAACGCTTAAACTCAGC   1359
m141101_iid=614933_0       ------------------------------------------------------------   0
```

```
m141031_iid=347546_0       ------------------------------------------------------------   0
m150506_iid=3127414_0      ------------------------------------------------------------   0
m141102_iid=1245517_0      ------------------------------------------------------------   0
m141107_iid=1405727_0      ------------------------------------------------------------   0
m141102_iid=987614_0       ------------------------------------------------------------   0
m141108_iid=1701429_0      ------------------------------------------------------------   0
m141102_iid=987286_0       ------------------------------------------------------------   0
m150430_iid=3272125_0      ------------------------------------------------------------   0
m141102_iid=754225_0       CACCTCAACGAATAAATGATTATATTCAACAAACCACAAAGACATCGGAACACTATACTT   337
m141107_iid=1409934_0      CACCTCAACGAATAAATGATTATATTCAACAAACCACAAAGACATCGGAACACTATACTT   866
m150503_iid=3416920_0      ------------------------------------------------------------   0
m141108_iid=1800121_0      ------------------------------------------------------------   0
m141031_iid=147540_0       ------------------------------------------------------------   0
m150507_iid=3674924_0      ------------------------------------------------------------   0
m150503_iid=2923751_0      ------------------------------------------------------------   0
m141031_iid=115118_0       ------------------------------------------------------------   0
m150430_iid=2722448_0      ------------------------------------------------------------   0
m150507_iid=3102647_0      ------------------------------------------------------------   0
m141108_iid=1811866_0      ------------------------------------------------------------   0
m150501_iid=2948495_0      ------------------------------------------------------------   0
m150504_iid=3238195_0      CACCTCAACGAATAAATGATTATATTCAACAAACCACAAAGACATCGGAACACTATACTT   474
m150503_iid=3388511_0      ------------------------------------------------------------   0
m150505_iid=3777611_0      ------------------------------------------------------------   0
m150430_iid=2438833_0      ------------------------------------------------------------   0
m141108_iid=1698969_0      CACCTCAACGAATAAATGATTATATTCAACAAACCACAAAGACATCGGAACACTATACTT   1067
m150430_iid=3059190_0      ------------------------------------------------------------   0
m141107_iid=1383067_0      ------------------------------------------------------------   0
m141101_iid=597008_0       ------------------------------------------------------------   0
m150506_iid=2596496_0      CACCTCAACGAATAAATGATTATATTCAACAAACCACAAAGACATCGGAACACTATACTT   1412
m141102_iid=910051_0       CACCTCAACGAATAAATGATTATATTCAACAAACCACAAAGACATCGGAACACTATACTT   902
m150505_iid=3784017_0      ------------------------------------------------------------   0
m141101_iid=682151_0       ------------------------------------------------------------   0
m150501_iid=3748029_0      ------------------------------------------------------------   0
m150504_iid=2876272_0      CACCTCAACGAATAAATGATTATATTCAACAAACCACAAAGACATCGGAACACTATACTT   1177
NC_042820.1                CACCTCAACGAATAAATGATTATATTCAACAAACCACAAAGACATCGGAACACTATACTT   1419
m141102_iid=1228825_0      CACCTCAACGAATAAATGATTATATTCAACAAACCACAAAGACATCGGAACACTATACTT   1249
m150504_iid=2876274_0      CACCTCAACGAATAAATGATTATATTCAACAAACCACAAAGACATCGGAACACTATACTT   1249
m141030_iid=80036_0        CACCTCAACGAATAAATGATTATATTCAACAAACCACAAAGACATCGGAACACTATACTT   1015
m150506_iid=2596494_0      CACCTCAACGAATAAATGATTATATTCAACAAACCACAAAGACATCGGAACACTATACTT   1414
m150503_iid=2526862_0      CACCTCAACGAATAAATGATTATATTCAACAAACCACAAAGACATCGGAACACTATACTT   1098
m150506_iid=2567000_0      ------------------------------------------------------------   0
m150507_iid=3073271_0      CACCTCAACGAATAAATGATTATATTCAACAAACCACAAAGACATCGGAACACTATACTT   351
m150504_iid=3156087_0      CACCTCAACGAATAAATGATTATATTCAACAAACCACAAAGACATCGGAACACTATACTT   174
m150505_iid=2498502_0      ------------------------------------------------------------   0
m141102_iid=754227_0       CACCTCAACGAATAAATGATTATATTCAACAAACCACAAA----ACATAACACTATACTT   387
m150507_iid=3469514_0      CACCTCAACGAATAAATGATTATATTCAACAAACCACAAAGACATCGGAACACTATACTT   943
Rdo_MitoGenome             CACCTCAACGAATAAATGATTATATTCAACAAACCACAAAGACATCGGAACACTATACTT   1419
m141101_iid=614933_0       ------------------------------------------------------------   0
```

cox1

```
m141031_iid=347546_0       ------------------------------------------------------------   0
```

```
m150506_iid=3127414_0      ------------------------------------------------------------   0
m141102_iid=1245517_0      ------------------------------------------------------------   0
m141107_iid=1405727_0      ------------------------------------------------------------   0
m141102_iid=987614_0       ------------------------------------------------------------   0
m141108_iid=1701429_0      ------------------------------------------------------------   0
m141102_iid=987286_0       ------------------------------------------------------------   0
m150430_iid=3272125_0      ------------------------------------------------------------   0
m141102_iid=754225_0       CATCTTCGGAATCTGATCAGGAATAGTAGGAACAGCCCTAAGAATACTAATCCGATCCGA   397
m141107_iid=1409934_0      CATCTTCGGAATCTGATCAGGAATAGTAGGAACAGCCCTAAGAATACTAATCCGATCCGA   926
m150503_iid=3416920_0      ------------------------------------------------------------   0
m141108_iid=1800121_0      ------------------------------------------------------------   0
m141031_iid=147540_0       ------------------------------------------------------------   0
m150507_iid=3674924_0      ------------------------------------------------------------   0
m150503_iid=2923751_0      ------------------------------------------------------------   0
m141031_iid=115118_0       ------------------------------------------------------------   0
m150430_iid=2722448_0      ------------------------------------------------------------   0
m150507_iid=3102647_0      ------------------------------------------------------------   0
m141108_iid=1811866_0      ------------------------------------------------------------   0
m150501_iid=2948495_0      ------------------------------------------------------------   0
m150504_iid=3238195_0      CATCTTCGGAATCTGATCAGGAATAGTAGGAACAGCCCTAAGAATACTAATCCGATCCGA   534
m150503_iid=3388511_0      ------------------------------------------------------------   0
m150505_iid=3777611_0      ------------------------------------------------------------   0
m150430_iid=2438833_0      ------------------------------------------------------ATCCGA   6
m141108_iid=1698969_0      CATCTTCGGAATCTGATCAGGAATAGTAGGAACAGCCCTAAGAATACTAATCCGATCCGA   1127
m150430_iid=3059190_0      ------------------------------------------------------------   0
m141107_iid=1383067_0      ------------------------------------------------------------   0
m141101_iid=597008_0       ------------------------------------------------------------   0
m150506_iid=2596496_0      CATCTTCGGAATCTGATCAGGAATAGTAGGAACAGCCCTAAGAATACTAATCCGATCCGA   1472
m141102_iid=910051_0       CATCTTCGGAATCTGATCAGGAATAGTAGGAACAGCCCTAAGAATACTAATCCGATCCGA   962
m150505_iid=3784017_0      ------------------------------------------------------------   0
m141101_iid=682151_0       ------------------------------------------------------------   0
m150501_iid=3748029_0      ------------------------------------------------------------   0
m150504_iid=2876272_0      CATCTTCGGAATCTGATCAGGAATAGTAGGAACAGCCCTAAGAATACTAATCCGATCCGA   1237
NC_042820.1                CATCTTCGGAATCTGATCAGGAATAGTAGGAACAGCCCTAAGAATACTAATCCGATCCGA   1479
m141102_iid=1228825_0      CATCTTCGGAATCTGATCAGGAATAGTAGGAACAGCCCTAAGAATACTAATCCGATCCGA   1309
m150504_iid=2876274_0      CATCTTCGGAATCTGATCAGGAATAGTAGGAACAGCCCTAAGAATACTAATCCGATCCGA   1309
m141030_iid=80036_0        CATCTTCGGAATCTGATCAGGAATAGTAGGAACAGCCCTAAGAATACTAATCCGATCCGA   1075
m150506_iid=2596494_0      CATCTTCGGAATCTGATCAGGAATAGTAGGAACAGCCCTAAGAATACTAATCCGATCCGA   1474
m150503_iid=2526862_0      CATCTTCGGAATCTGATCAGGAATAGTAGGAACAGCCCTAAGAATACTAATCCGATCCGA   1158
m150506_iid=2567000_0      ------------------------------------------------------------   0
m150507_iid=3073271_0      CATCTTCGGAATCTGATCAGGAATAGTAGGAACAGCCCTAAGAATACTAATCCGATCCGA   411
m150504_iid=3156087_0      CATCTTCGGAATCTGATCAGGAATAGTAGGAACAGCCCTAAGAATACTAATCCGATCCGA   234
m150505_iid=2498502_0      ------------------------------------------------------------   0
m141102_iid=754227_0       CATCTTCGGAATCTGATCAGGAATAGTAGGAACAGCCCTAAGAATACTAATCCGATCCGA   447
m150507_iid=3469514_0      CATCTTCGGAATCTGATCAGGAATAGTAGGAACAGCCCTAAGAATACTAATCCGATCCGA   1003
Rdo_MitoGenome             CATCTTCGGAATCTGATCAGGAATAGTAGGAACAGCCCTAAGAATACTAATCCGATCCGA   1479
m141101_iid=614933_0       ----------------------------GGAACAGCCCTAAGAATACTAATCCGATCCGA   32
                                                 cox1
m141031_iid=347546_0       ------------------------------------------------------------   0
m150506_iid=3127414_0      ------------------------------------------------------------   0
m141102_iid=1245517_0      ------------------------------------------------------------   0
m141107_iid=1405727_0      ------------------------------------------------------------   0
m141102_iid=987614_0       ------------------------------------------------------------   0
m141108_iid=1701429_0      ------------------------------------------------------------   0
m141102_iid=987286_0       ------------------------------------------------------------   0
m150430_iid=3272125_0      ------------------------------------------------------------   0
m141102_iid=754225_0       ATTAGGAAATCCAGGAGCCCTAATTGGAGATGACCAAA-TTTATAACGTAATTGTAACAG   456
m141107_iid=1409934_0      ATTAGGAAATCCAGGAGCCCTAATTGGAGATGACCAAA-TTTATAACGTAATTGTAACAG   985
m150503_iid=3416920_0      ------------------------------------------------------------   0
m141108_iid=1800121_0      ------------------------------------------------------------   0
m141031_iid=147540_0       ------------------------------------------------------------   0
m150507_iid=3674924_0      ----------------------------------------------------TGTAACAG   8
m150503_iid=2923751_0      ------------------------------------------------------------   0
m141031_iid=115118_0       ------------------------------------------------------------   0
m150430_iid=2722448_0      ------------------------------------------------------------   0
m150507_iid=3102647_0      ------------------------------------------------------------   0
m141108_iid=1811866_0      ------------------------------------------------------------   0
m150501_iid=2948495_0      ------------------------------------------------------------   0
m150504_iid=3238195_0      T-TAGGAAATCCAGGAGCCCTAATTGGAGATGACCAAA-TTTATAACGTAATTGTAACAG   592
m150503_iid=3388511_0      ------------------------------------------------------------   0
```

```
m150505_iid=3777611_0       ------------------------------------------------------------   0
m150430_iid=2438833_0       ATTAGGAAATC-AGGAGCCCTAATTGGAGATGACCAAA-TTTATAACGTAATTGTAACAG   64
m141108_iid=1698969_0       ATTAGGAAATCCAGGAGCCCTAATTGGAGATGACCAAA-TTTATAACGTAATTGTAACAG   1186
m150430_iid=3059190_0       ------------------------------------------------------------   0
m141107_iid=1383067_0       ------------------------------------------------------------   0
m141101_iid=597008_0        ------------------------------------------------------------   0
m150506_iid=2596496_0       ATTAGGAAATCCAGGAGCCCTAATTGGAGATGACCAAA-TTTATAACGTAATTGTAACAG   1531
m141102_iid=910051_0        ATTAGGAAATCCAGGAGCCCTAATTGGAGATGACCAAA-TTTATAACGTAATTGTAACAG   1021
m150505_iid=3784017_0       ------------------------------------------------------------   0
m141101_iid=682151_0        ------------------------------------------------------------   0
m150501_iid=3748029_0       ------------------------------------------------------------   0
m150504_iid=2876272_0       ATTAGGAAATCCAGGAGCCCTAATTGGAGATGACCAAA-TTTATAACGTAATTGTAACAG   1296
NC_042820.1                 ATTAGGAAATCCAGGAGCCCTAATTGGAGATGACCAAA-TTTATAACGTAATTGTAACAG   1538
m141102_iid=1228825_0       ATTAGGAAATCCAGGAGCCCTAATTGGAGATGACCAAA-TTTATAACGTAATTGTAACAG   1368
m150504_iid=2876274_0       ATTAGGAAATCCAGGAGCCCTAATTGGAGATGACCAAA-TTTATAACGTAATTGTAACAG   1368
m141030_iid=80036_0         ATTAGGAAATCCAGGAGCCCTAATTGGAGATGACCAAA-TTTATAACGTAATTGTAACAG   1134
m150506_iid=2596494_0       ATTAGGAAATCCAGGAGCCCTAATTGGAGATGACCAAA-TTTATAACGTAATTGTAACAG   1533
m150503_iid=2526862_0       ATTAGGAAATCCAGGAGCCCTAATTGGAGATGACCAAA-TTTATAACGTAATTGTAACAG   1217
m150506_iid=2567000_0       ------------------------------------------------------------   0
m150507_iid=3073271_0       ATTAGGAAATCCAGGAGCCCTAATTGGAGATGACCAAA-TTTATAACGTAATTGTAACAG   470
m150504_iid=3156087_0       ATTAGGAAATCCAGGAGCCCTAATTGGAGATGACCAAA-TTTATAACGTAATTGTAACAG   293
m150505_iid=2498502_0       ------------------------------------------------------------   0
m141102_iid=754227_0        ATTAGGAAATCCAGGAGCCCTAATTGGAGATGACCAAA-TTTATAACGTAATTGTAACAG   506
m150507_iid=3469514_0       ATTAGGAAATCCAGGAGCCCTAATTGGAGATGACCAAAATTTATAACGTAATTGTAACAG   1063
Rdo_MitoGenome              ATTAGGAAATCCAGGAGCCCTAATTGGAGATGACCAAA-TTTATAACGTAATTGTAACAG   1538
m141101_iid=614933_0        ATTAGGAAATCCAGGAGCCCTAATTGGAGATGACCAAA-TTTATAACGTAATTGTAACAG   91
```

cox1

```
m141031_iid=347546_0        ------------------------------------------------------------   0
m150506_iid=3127414_0       ------------------------------------------------------------   0
m141102_iid=1245517_0       ------------------------------------------------------------   0
m141107_iid=1405727_0       ------------------------------------------------------------   0
m141102_iid=987614_0        ------------------------------------------------------------   0
m141108_iid=1701429_0       ------------------------------------------------------------   0
m141102_iid=987286_0        ------------------------------------------------------------   0
m150430_iid=3272125_0       ------------------------------------------------------------   0
m141102_iid=754225_0        CACATGCATTCATCATAAT-TTTCTTCATAGTT-ATACCAATAATAATTGGAGGATTCGG   514
m141107_iid=1409934_0       CACATGCATTCATCATAAT-TTTCTTCATAGTT-ATACCAATAATAATTGGAGGATTCGG   1043
m150503_iid=3416920_0       ------------------------------------------------------------   0
m141108_iid=1800121_0       ------------------------------------------------------------   0
m141031_iid=147540_0        ------------------------------------------------------------   0
m150507_iid=3674924_0       CACATGCATTCATCATAATTTTTCTTCAAGTTATATACCAAT--AATATGGAGGATTCGA   66
m150503_iid=2923751_0       ------------------------------------------------------------   0
m141031_iid=115118_0        ------------------------------------------------------------   0
m150430_iid=2722448_0       ------------------------------------------------------------   0
m150507_iid=3102647_0       ------------------------------------------------------------   0
m141108_iid=1811866_0       ------------------------------------------------------------   0
m150501_iid=2948495_0       ------------------------------------------------------------   0
m150504_iid=3238195_0       CACATGCATTCATCATAAT-TTTCTTCATAGTT-ATACCAATAATAATTGGAGGATTCGG   650
m150503_iid=3388511_0       ------------------------------------------------------------   0
m150505_iid=3777611_0       ------------------------------------------------------------   0
m150430_iid=2438833_0       CACATGCATTCATCATAAT-TTTCTTCATAGTT-ATACCAATAATAA-TGGAGGATTCGG   121
m141108_iid=1698969_0       CACATGCATTCATCATAAT-TTTCTTCATAGTT-ATACCAATAATAA-TGGAGGATTCGG   1243
m150430_iid=3059190_0       ------------------------------------------------------------   0
m141107_iid=1383067_0       ------------------------------------------------------------   0
m141101_iid=597008_0        ------------------------------------------------------------   0
m150506_iid=2596496_0       CACATGCATTCATCATAAT-TTTCTTCATAGTT-ATACCAATAATAATTGGAGGATTCGG   1589
m141102_iid=910051_0        CACATGCATTCATCATAAT-TTTCTTCATAGTT-ATACCAATAATAATTGGAGGATTCGG   1079
m150505_iid=3784017_0       ------------------------------------------------------------   0
m141101_iid=682151_0        ------------------------------------------------------------   0
m150501_iid=3748029_0       ------------------------------------------------------------   0
m150504_iid=2876272_0       CACATGCATTCATCATAAT-TTTCTTCATAGTT-ATACCAATAATAATTGGAGGATTCGG   1354
NC_042820.1                 CACATGCATTCATCATAAT-TTTCTTCATAGTT-ATACCAATAATAATTGGAGGATTCGG   1596
m141102_iid=1228825_0       CACATGCATTCATCATAAT-TTTCTTCATAGTT-ATACCAATAATAATTGGAGGATTCGG   1426
m150504_iid=2876274_0       CACATGCATTCATCATAAT-TTTCTTCATAGTT-ATACCAATAATAATTGGAGGATTCGG   1426
m141030_iid=80036_0         CACATGCATTCATCATAAT-TTTCTTCATAGTT-ATA-CAATAATAATTGGAGGATTCGG   1191
m150506_iid=2596494_0       CACATGCATTCATCATAAT-TTTCTTCATAGTT-ATACCAATAATAATTGGAGGATTCGG   1591
m150503_iid=2526862_0       CACATGCATTCATCATAAT-TTTCTTCATAGTT-ATACCAATAATAATTGGAGGATTCGG   1275
m150506_iid=2567000_0       ------------------------------------------------------------   0
m150507_iid=3073271_0       CACATGCATTCATCATAAT-TTTCTTCATAGTT-ATACCAATAATAATTGGAGGATTCGG   528
m150504_iid=3156087_0       CACATGCATTCATCATAAT-TTTCTTCATAGTT-ATACCAATAATAATTGGAGGATTCGG   351
```

```
m150505_iid=2498502_0     ------------------------------------------------------------   0
m141102_iid=754227_0      CACATGCATTCATCATAAT-TTTCTTCATAGTT-ATACCAATAATAATTGGAGGATTCGG   564
m150507_iid=3469514_0     CACATGCATTCATCATAAT-TTTCTTCATAGTT-ATACCAATAATAATTGGAGGATTCGG   1121
Rdo_MitoGenome            CACATGCATTCATCATAAT-TTTCTTCATAGTT-ATACCAATAATAATTGGAGGATTCGG   1596
m141101_iid=614933_0      CACATGCATTCATCATAAATTTTCTTCATAGTT-ATAC-AATAATAATTGGAGGATTCGG   149
```

cox1

```
m141031_iid=347546_0      ------------------------------------------------------------   0
m150506_iid=3127414_0     ------------------------------------------------------------   0
m141102_iid=1245517_0     ------------------------------------------------------------   0
m141107_iid=1405727_0     ------------------------------------------------------------   0
m141102_iid=987614_0      ------------------------------------------------------------   0
m141108_iid=1701429_0     ------------------------------------------------------------   0
m141102_iid=987286_0      ------------------------------------------------------------   0
m150430_iid=3272125_0     ------------------------------------------------------------   0
m141102_iid=754225_0      AAATTGACTAGTTCCACTAATAATTGGAGCACCAGATA-TAGCATTCCCTCGAATAAACA   573
m141107_iid=1409934_0     AAATTGACTAGTTCCACTAATAATTGGAGCACCAGATA-TAGCATTCCCTCGAATAAACA   1102
m150503_iid=3416920_0     ------------------------------------------------------------   0
m141108_iid=1800121_0     ------------------------------------------------------------   0
m141031_iid=147540_0      ------------------------------------------------------------   0
m150507_iid=3674924_0     AATTGACTAAGTTCCACTAATAATTGGAGCACCAGATATAAGCATTCCCTCGAATAAACA   126
m150503_iid=2923751_0     ------------------------------------------------------------   0
m141031_iid=115118_0      ------------------------------------------------------------   0
m150430_iid=2722448_0     ------------------------------------------------------------   0
m150507_iid=3102647_0     ------------------------------------------------------------   0
m141108_iid=1811866_0     ------------------------------------------------------------   0
m150501_iid=2948495_0     ------------------------------------------------------------   0
m150504_iid=3238195_0     AAATTGACTAGTTCCACTAATAATTGGAGCACCAGATA-TAGCATTCCCTCGAATAAACA   709
m150503_iid=3388511_0     ------------------------------------------------------------   0
m150505_iid=3777611_0     ------------------------------------------------------------   0
m150430_iid=2438833_0     AAATTGACTAGTTCCACTAATAATTGGAGCACCAGATA-TAGCATTCCCTCGAATAAACA   180
m141108_iid=1698969_0     AAATTGACTAGTTCCACTAATAATTGGAGCACCAGATA-TAGCATTCCCTCGAATAAACA   1302
m150430_iid=3059190_0     ------------------------------------------------------------   0
m141107_iid=1383067_0     ------------------------------------------------------------   0
m141101_iid=597008_0      ------------------------------------------------------------   0
m150506_iid=2596496_0     AAATTGACTAGTTCCACTAATAATTGGAGCACCAGATA-TAGCATTCCCTCGAATAAACA   1648
m141102_iid=910051_0      AAATTGACTAGTTCCACTAATAATTGGAGCACCAGATA-TAGCATTCCCTCGAATAAACA   1138
m150505_iid=3784017_0     ------------------------------------------------------------   0
m141101_iid=682151_0      ------------------------------------------------------------   0
m150501_iid=3748029_0     ------------------------------------------------------------   0
m150504_iid=2876272_0     AAATTGACTAGTTCCACTAATAATTGGAGCACCAGATA-TAGCATTCCCTCGAATAAACA   1413
NC_042820.1               AAATTGACTAGTTCCACTAATAATTGGAGCACCAGATA-TAGCATTCCCTCGAATAAACA   1655
m141102_iid=1228825_0     AAATTGACTAGTTCCACTAATAATTGGAGCACCAGATA-TAGCATTCCCTCGAATAAACA   1485
m150504_iid=2876274_0     AAATTGACTAGTTCCACTAATAATTGGAGCACCAGATA-TAGCATTCCCTCGAATAAACA   1485
m141030_iid=80036_0       AAATTGACTAGTTCCACTAATAATTGGAGCACCAGATA-TAGCATTCCCTCGAATAAACA   1250
m150506_iid=2596494_0     AAATTGACTAGTTCCACTAATAATTGGAGCACCAGATA-TAGCATTCCCTCGAATAAACA   1650
m150503_iid=2526862_0     AAATTGACTAGTTCCACTAATAATTGGAGCACCAGATA-TAGCATTCCCTCGAATAAACA   1334
m150506_iid=2567000_0     ------------------------------------------------------------   0
m150507_iid=3073271_0     AAATTGACTAGTTCCACTAATAATTGGAGCACCAGATA-TAGCATTCCCTCGAATAAACA   587
m150504_iid=3156087_0     AAATTGACTAGTTCCACTAATAATTGGAGCACCAGATA-TAGCATTCCCTCGAATAAACA   410
m150505_iid=2498502_0     ------------------------------------------------------------   0
m141102_iid=754227_0      AAATTGACTAGTTCCACTAATAATTGGAGCACCAGATA-TAGCATTCCCTCGAATAAACA   623
m150507_iid=3469514_0     AAATTGACTAGTTCCACTAATAATTGGAGCACCAGATA-TAGCATTCCCTCGAATAAACA   1180
Rdo_MitoGenome            AAATTGACTAGTTCCACTAATAATTGGAGCACCAGATA-TAGCATTCCCTCGAATAAACA   1655
m141101_iid=614933_0      AAATTGACTAGTTCCACTAATAATTGGAGCACCAGATA-TAGCATTCCCTCGAATAAACA   208
```

cox1

```
m141031_iid=347546_0      ------------------------------------------------------------   0
m150506_iid=3127414_0     ------------------------------------------------------------   0
m141102_iid=1245517_0     ------------------------------------------------------------   0
m141107_iid=1405727_0     ------------------------------------------------------------   0
m141102_iid=987614_0      ------------------------------------------------------------   0
m141108_iid=1701429_0     ------------------------------------------------------------   0
m141102_iid=987286_0      ------------------------------------------------------------   0
m150430_iid=3272125_0     ------------------------------------------------------------   0
m141102_iid=754225_0      ACATAAGATTTTGGCTTCTTCCACCTCCT-----TAACCCTTTTACTAACAAGAAGAATT   628
m141107_iid=1409934_0     ACATAAGATTTTGGCTTCTTCCACCCTCCTTA----ACCCTTTTACTAACAAGAAGAATT   1158
m150503_iid=3416920_0     ------------------------------------------------------------   0
m141108_iid=1800121_0     ------------------------------------------------------------   0
m141031_iid=147540_0      -----------TGGCTTCTTCCACCCTCCTT-----AACCCTTTACTAACAAGAAGAATT   44
m150507_iid=3674924_0     ACATAAGATTTTGGCTTCTTCCACCCTCCCTTAACCACCCTTTTACTAACAAGAAGAATG   186
```

```
m150503_iid=2923751_0      -----------TGGCTTCTTCCACCCTCCTTA----AC-CTTTTACTAACAAGAAGAATT   44
m141031_iid=115118_0       ------------------------------------------------------------   0
m150430_iid=2722448_0      ------------------------------------------------------------   0
m150507_iid=3102647_0      ------------------------------------------------------------   0
m141108_iid=1811866_0      ------------------------------------------------------------   0
m150501_iid=2948495_0      ------------------------------------------------------------   0
m150504_iid=3238195_0      ACATAAGATTTTGGCTTCTTCCACCCTCCTTA----ACCCTTTTACTAACAAGAAGAATT   765
m150503_iid=3388511_0      ------------------------------------------------------------   0
m150505_iid=3777611_0      ------------------------------------------------------------   0
m150430_iid=2438833_0      ACATAAGATTTTGGCTT-TTCCACCCTCCTTA----ACCCTTTTACTAACAAGAAGAATT   235
m141108_iid=1698969_0      ACATAAGATTTTGGCTTCTTCCACCCTCCTTA----ACCCTTTTACTAACAAGAAGAATT   1358
m150430_iid=3059190_0      ------------------------------------------------------------   0
m141107_iid=1383067_0      ------------------------------------------------------------   0
m141101_iid=597008_0       ------------------------------------------------------------   0
m150506_iid=2596496_0      ACATAAGATTTTGGCTTCTTCCACCCTCCTTA----ACCCTTTTACTAACAAGAAGAATT   1704
m141102_iid=910051_0       ACATAAGATTTTGGCTTCTTCCACCCTCCTTA----ACCCTTTTACTAACAAGAAGAATT   1194
m150505_iid=3784017_0      ------------------------------------------------------------   0
m141101_iid=682151_0       ------------------------------------------------------------   0
m150501_iid=3748029_0      ------------------------------------------------------------   0
m150504_iid=2876272_0      ACATAAGATTTTGGCTTCTTCCACCCTCCTTA----ACCCTTTTACTAACAAGAAGAATT   1469
NC_042820.1                ACATAAGATTTTGGCTTCTTCCACCCTCCTTA----ACCCTTTTACTAACAAGAAGAATT   1711
m141102_iid=1228825_0      ACATAAGATTTTGGCTTCTTCCACCCTCCTTA----ACCCTTTTACTAACAAGAAGAATT   1541
m150504_iid=2876274_0      ACATAAGATTTTGGCTTCTTCCACCCTCCTTA----ACCCTTTTACTAACAAGAAGAATT   1541
m141030_iid=80036_0        ACATAAGATTTTGGCTTCTTCCACCCTCCTTA----ACCCTTTTACTAACAAGAAGAATT   1306
m150506_iid=2596494_0      ACATAAGATTTTGGCTTCTTCCACCCTCCTTA----ACCCTTTTACTAACAAGAAGAATT   1706
m150503_iid=2526862_0      ACATAAGATTTTGGCTTCTTCCACCCTCCTTA----ACCCTTTTACTAACAAGAAGAATT   1390
m150506_iid=2567000_0      ------------------------------------------------------------   0
m150507_iid=3073271_0      ACATAAGATTTTGGCTTCTTCCACCCTCCTTA----ACCCTTTTACTAACAAGAAGAATT   643
m150504_iid=3156087_0      ACATAAGATTTTGGCTTCTTCCACCCTCCTTA----ACCCTTTTACTAACAAGAAGAATT   466
m150505_iid=2498502_0      ------------------------------------------------------------   0
m141102_iid=754227_0       ACATAAGATTTTGGCTTCTTCCAC-CTCCTTA----ACCCTTTTACTAACAAGAAGAATT   678
m150507_iid=3469514_0      ACATAAGATTTTGGCTTCTTCCACCCTCCTTA----ACCCTTTTACTAACAAGAAGAATT   1236
Rdo_MitoGenome             ACATAAGATTTTGGCTTCTTCCACCCTCCTTA----ACCCTTTTACTAACAAGAAGAATT   1711
m141101_iid=614933_0       ACATAAGATTTTGGCTTCTTCCACCCTCCTTA----ACCCTTTTACTAACAAGAAGAATT   264
```

cox1

```
m141031_iid=347546_0       ------------------------------------------------------------   0
m150506_iid=3127414_0      ------------------------------------------------------------   0
m141102_iid=1245517_0      ------------------------------------------------------------   0
m141107_iid=1405727_0      ------------------------------------------------------------   0
m141102_iid=987614_0       -------------------CAG--GATGAACAGTTTATTCACCTCTATCTA-ATAATACA   38
m141108_iid=1701429_0      ------------------------------------------------------------   0
m141102_iid=987286_0       ------------------------------------------------------------   0
m150430_iid=3272125_0      ------------------------------------------------------------   0
m141102_iid=754225_0       GTAGAGACAGGAGCAGGAACAG--GATGAACAGTTTATCCACCTCTATCTA-ATAATACA   685
m141107_iid=1409934_0      GTAGAGACAGGAGCAGGAACAG--GATGAACAGTTTATCCACCTCTATCTA-ATAATACA   1215
m150503_iid=3416920_0      ------------------------------------------------------------   0
m141108_iid=1800121_0      ------------------------------------------------------------   0
m141031_iid=147540_0       GTAGAGACAGGAGCA-GAACAG--GATGAACAGTTTATCCACCTCTATCTATATAATACA   101
m150507_iid=3674924_0      TTAGAGACAGGAGCAGGAACAGAGAATGAACAGTTTATCCACCTCTATCTA-ATAATACA   245
m150503_iid=2923751_0      GTAGAGACAGGAGCAGGAACAG--GATGAACAGTTTATCCACCTCTATCTA-ATAATACA   101
m141031_iid=115118_0       ------------------------------------------------------------   0
m150430_iid=2722448_0      ------------------------------------------------------------   0
m150507_iid=3102647_0      ------------------------------------------------------------   0
m141108_iid=1811866_0      ------------------------------------------------------------   0
m150501_iid=2948495_0      ------------------------------------------------------------   0
m150504_iid=3238195_0      GTAGAGACAGGAGCAGGAACAG--GATGAACAGTTTATCCACCTCTATCTA-ATAATACA   822
m150503_iid=3388511_0      ------------------------------------------------------------   0
m150505_iid=3777611_0      ------------------------------------------------------------   0
m150430_iid=2438833_0      GTAGAGACAGGAGCAGGAACAG--GATGAACAGTTTATCCACCTCTATCTA-ATAATACA   292
m141108_iid=1698969_0      GTAGAGACAGGAG------CAG--GATGAACAGTTTATCCACCTCTATCTA-ATAATACA   1409
m150430_iid=3059190_0      ------------------------------------------------------------   0
m141107_iid=1383067_0      ------------------------------------------------------------   0
m141101_iid=597008_0       ------------------------------------------------------------   0
m150506_iid=2596496_0      GTAGAGACAGGAGCAGGAACAG--GATGAACAGTTTATCCACCTCTATCTA-ATAATACA   1761
m141102_iid=910051_0       GTAGAGACAGGAGCAGGAACAG--GATGAACAGTTTATCCACCTCTATCTA-ATAATACA   1251
m150505_iid=3784017_0      -----GACAGGAGCAGGAACAG--GATGAACAGTTTATCCACCTCTATCTA-ATAATACA   52
m141101_iid=682151_0       ------------------------------------------------------------   0
m150501_iid=3748029_0      ------------------------------------------------------------   0
m150504_iid=2876272_0      GTAGAGACAGGAGCAGGAACAG--GATGAACAGTTTATCCACCTCTATCTA-ATAATACA   1526
NC_042820.1                GTAGAGACAGGAGCAGGAACAG--GATGAACAGTTTATCCACCTCTATCTA-ATAATACA   1768
```

```
m141102_iid=1228825_0      GTAGAGACAGGAGCAGGAACAG--GATGAACAGTTTATCCACCTCTATCTA-ATAATACA   1598
m150504_iid=2876274_0      GTAGAGACAGGAGCAGGAACAG--GATGAACAGTTTATCCACCTCTATCTA-ATAATACA   1598
m141030_iid=80036_0        GTAGAGACAGGAGCAGGAACAG--GATGAACAGTTTATCCACCTCTATCTA-ATAATACA   1363
m150506_iid=2596494_0      GTAGAGACAGGAGCAGGAACAG--GATGAACAGTTTATCCACCTCTATCTA-ATAATACA   1763
m150503_iid=2526862_0      GTAGAGACAGGAGCAGGAACAG--GATGAACAGTTTATCCACCTCTATCTA-ATAATACA   1447
m150506_iid=2567000_0      ------------------------------------------------------------   0
m150507_iid=3073271_0      GTAGAGACAGGAGCAGGAACAG--GATGAACAGTTTATCCACCTCTATCTA-ATAATACA   700
m150504_iid=3156087_0      GTAGAGACAGGAGCAGGAACAG--GATGAACAGTTTATCCACCTCTATCTA-ATAATACA   523
m150505_iid=2498502_0      ------------------------------------------------------------   0
m141102_iid=754227_0       GTAGAGACAGGAGCAGGAACAG--GATGAACAGTTTATCCACCTCTATCTA-ATAATACA   735
m150507_iid=3469514_0      GTAGAGACAGGAGCAGGAACAG--GATGAACAGTTTATCCACCTCTATCTA-ATAATACA   1293
Rdo_MitoGenome             GTAGAGACAGGAGCAGGAACAG--GATGAACAGTTTATCCACCTCTATCTA-ATAATACA   1768
m141101_iid=614933_0       GTAGAGACAGGAGCAGGAACAG--GATGAACAGTTTATCCACCTCTATCTA-ATAATACA   321
```

cox1

```
m141031_iid=347546_0       ------------------------------------------------------------   0
m150506_iid=3127414_0      ------------------------------------------------------------   0
m141102_iid=1245517_0      ------------------------------------------------------------   0
m141107_iid=1405727_0      ------------------------------------------------------------   0
m141102_iid=987614_0       GCCCATAGAGGAGCTTCTGTTGATTTAGCAATTTTTAGATTACATTTAGCAGGAATTTCA   98
m141108_iid=1701429_0      ------------------------------------------------------------   0
m141102_iid=987286_0       ------------------------------------------------------------   0
m150430_iid=3272125_0      ------------------------------------------------------------   0
m141102_iid=754225_0       GCCCATAGAGGAGCTTCTGTTGATTTAGCAATTTTTAGATTACATTTAGCAGGAATTTCA   745
m141107_iid=1409934_0      GCCCATAGAGGAGCTTCTGTTGATTTAGCAATTTTTAGATTACATTTAGCAGGAATTTCA   1275
m150503_iid=3416920_0      ----------------------------------------TACATTTAGCAGGAATTTCA   20
m141108_iid=1800121_0      ------------------------------------------------------------   0
m141031_iid=147540_0       GCCCATAGAGGAGCTTCTGTTGATTTAGCAATTTTTAGATTACATTTAGCAGGAATTTCA   161
m150507_iid=3674924_0      GCCCATAGAGGAGCTTCTGTTGATTTAGCAATTTTTAGATTACATTTAGCAGGAATTTCA   305
m150503_iid=2923751_0      GCCCATAGAGGAGCTTCTGTTGATTTAGCAATTTTTAGATTACATTTAGCAGGAATTTCA   161
m141031_iid=115118_0       ------------------------------------------------------------   0
m150430_iid=2722448_0      ------------------------------------------------------------   0
m150507_iid=3102647_0      ------------------------------------------------------------   0
m141108_iid=1811866_0      ------------------------------------------------------------   0
m150501_iid=2948495_0      ------------------------------------------------------------   0
m150504_iid=3238195_0      GCCCATAGAGGAGCTTCTGTTGATTTAGCAATTTTTAGATTACATTTAGCAGGAATTTCA   882
m150503_iid=3388511_0      ------------------------------------------------------------   0
m150505_iid=3777611_0      ------------------------------------------------------------   0
m150430_iid=2438833_0      G-CCATAGAGGAGCTTCTGTTGATTTAGCAATTTTTAGATTACATTTAGCAGGAATTTCA   351
m141108_iid=1698969_0      GCCCATAGAGGAGCTTCTGTTGATTTAGCAATTTTTAGATTACATTTAGCAGGAATTTCA   1469
m150430_iid=3059190_0      ------------------------------------------------------------   0
m141107_iid=1383067_0      ------------------------------------------------------------   0
m141101_iid=597008_0       ------------------------------------------------------------   0
m150506_iid=2596496_0      GCCCATAGAGGAGCTTCTGTTGATTTAGCAATTTTTAGATTACATTTAGCAGGAATTTCA   1821
m141102_iid=910051_0       GCCCATAGAGGAGCTTCTGTTGATTTAGCAATTTTTAGATTACATTTAGCAGGAATTTCA   1311
m150505_iid=3784017_0      GCCCATAGAGGAGCTTCTGTTGATTTAGC-ATTTTTAGATTACATTTAGCAGGAATTTCA   111
m141101_iid=682151_0       ------------------------------------------------------------   0
m150501_iid=3748029_0      ------------------------------------------------------------   0
m150504_iid=2876272_0      GCCCATAGAGGAGCTTCTGTTGATTTAGCAATTTTTAGATTACATTTAGCAGGAATTTCA   1586
NC_042820.1                GCCCATAGAGGAGCTTCTGTTGATTTAGCAATTTTTAGATTACATTTAGCAGGAATTTCA   1828
m141102_iid=1228825_0      GCCCATAGAGGAGCTTCTGTTGATTTAGCAATTTTTAGATTACATTTAGCAGGAATTTCA   1658
m150504_iid=2876274_0      GCCCATAGAGGAGCTTCTGTTGATTTAGCAATTTTTAGATTACATTTAGCAGGAATTTCA   1658
m141030_iid=80036_0        GCCCATAGAGGAGCTTCTGTTGATTTAGCAATTTTTAGATTACATTTAGCAGGAATTTCA   1423
m150506_iid=2596494_0      GCCCATAGAGGAGCTTCTGTTGATTTAGCAATTTTTAGATTACATTTAGCAGGAATTTCA   1823
m150503_iid=2526862_0      GCCCATAGAGGAGCTTCTGTTGATTTAGCAATTTTTAGATTACATTTAGCAGGAATTTCA   1507
m150506_iid=2567000_0      ------------------------------------------------------------   0
m150507_iid=3073271_0      GCCCATAGAGGAGCTTCTGTTGATTTAGCAATTTTTAGATTACATTTAGCAGGAATTTCA   760
m150504_iid=3156087_0      GCCCATAGAGGAGCTTCTGTTGATTTAGCAATTTTTAGATTACATTTAGCAGGAATTTCA   583
m150505_iid=2498502_0      ------------------------------------------------------------   0
m141102_iid=754227_0       GCCCATAGAGGAGCTTCTGTTGATTTAGCAATTTTTAGATTACATTTAGCAGGAATTTCA   795
m150507_iid=3469514_0      GCCCATAGAGGAGCTTCTGTTGATTTAGCAATTTTTAGATTACATTTAGCAGGAATTTCA   1353
Rdo_MitoGenome             GCCCATAGAGGAGCTTCTGTTGATTTAGCAATTTTTAGATTACATTTAGCAGGAATTTCA   1828
m141101_iid=614933_0       GCCCATAGAGG-ACTTCTGTTGATTTAGCAATTTTTAGATTACATTTAGCAGGAATTTCA   380
```

```
m141031_iid=347546_0       ------------------------------------------------------------   0
m150506_iid=3127414_0      ------------------------------------------------------------   0
m141102_iid=1245517_0      ----------------------------CTAC-AATCATTAATATACGACCAAAAGGAAT   31
m141107_iid=1405727_0      ------------------------------------------------------------   0
m141102_iid=987614_0       TCAATTCTTGGAGCAGTAAACTTTATTACTAC-AATCATTAATATACGACCAAAAGGAAT   157
m141108_iid=1701429_0      ------------------------------------------------------------   0
```

```
m141102_iid=987286_0        ------------------------------------------------------------   0
m150430_iid=3272125_0       ------------------------------------------------------------   0
m141102_iid=754225_0        TCAATTCTTGGAGCAGTAAACTTTATTACTACA-ATCATTAATATACGACCAAAAGGAAT   804
m141107_iid=1409934_0       TCAATTCTTGGAGCAGTAAACTTTATTACTACA-ATCATTAATATACGACCAAAAGGAAT   1334
m150503_iid=3416920_0       TCAATTCTTGGAGCAGTAA-CTTTATTACTAC-AATCATTAATATACGACCAAAAGGAAT   78
m141108_iid=1800121_0       ------------------------------------------------------------   0
m141031_iid=147540_0        TCAATTCTTGGAGCAGTAAACTTTATTACTAC-AATCATTAATATACGACCAAAAGGAAT   220
m150507_iid=3674924_0       TCAATTCTTGGAGCAGTAAACTTTATTACTACA-ATCATTAATATACGACCAAAAGGAAT   364
m150503_iid=2923751_0       TCAATTCTTGGAGCAGTAAACTTTATTACTACA-ATCATTAATATACGAC-AAAAGGAAT   219
m141031_iid=115118_0        ------------------------------------------------------------   0
m150430_iid=2722448_0       ------------------------------------------------------------   0
m150507_iid=3102647_0       ------------------------------------------------------------   0
m141108_iid=1811866_0       ------------------------------------------------------------   0
m150501_iid=2948495_0       ------------------------------------------------------------   0
m150504_iid=3238195_0       TCAATTCTTGGAGCAGTAAACTTTATTACTACA-ATCATTAATATACGACCAAAAGGAAT   941
m150503_iid=3388511_0       ------------------------------------------------------------   0
m150505_iid=3777611_0       -----TCTTGGAGCAGTAAACTTTATTACTACAAATCATTAATATACGACCAAAAGGAAT   55
m150430_iid=2438833_0       TCAATTCTTGGAGCAGTAAACTTTATTACTAC-AATCATTAATATACGACCAAAAGGAAT   410
m141108_iid=1698969_0       TCAATTCTTGGAGCAGTAAACTTTATTACTAC-AATCATTAATATACGACCAAAAGGAAT   1528
m150430_iid=3059190_0       ------------------------------------------------------------   0
m141107_iid=1383067_0       ------------------------------------------------------------   0
m141101_iid=597008_0        ------------------------------------------------------------   0
m150506_iid=2596496_0       TCAATTCTTGGAGCAGTAAACTTTATTACTAC-AATCATTAATATACGACCAAAAGGAAT   1880
m141102_iid=910051_0        TCAATTCTTGGAGCAGTAAACTTTATTACTAC-AATCATTAATATACGACCAAAAGGAAT   1370
m150505_iid=3784017_0       TCAATTCTTGGAGCAGTAAACTTTATTACTAC-AATCATTAATATACGACCAAAAGGAAT   170
m141101_iid=682151_0        ------------------------------------------------------------   0
m150501_iid=3748029_0       ------------------------------------------------------------   0
m150504_iid=2876272_0       TCAATTCTTGGAGCAGTAAACTTTATTACTAC-AATCATTAATATACGACCAAAAGGAAT   1645
NC_042820.1                 TCAATTCTTGGAGCAGTAAACTTTATTACTAC-AATCATTAATATACGACCAAAAGGAAT   1887
m141102_iid=1228825_0       TCAATTCTTGGAGCAGTAAACTTTATTACTAC-AATCATTAATATACGACCAAAAGGAAT   1717
m150504_iid=2876274_0       TCAATTCTTGGAGCAGTAAACTTTATTACTAC-AATCATTAATATACGACCAAAAGGAAT   1717
m141030_iid=80036_0         TCAATTCTTGGAGCAGTAAACTTTATTACTAC-AATCATTAATATACGACCAAAAGGAAT   1482
m150506_iid=2596494_0       TCAATTCTTGGAGCAGTAAACTTTATTACTAC-AATCATTAATATACGACCAAAAGGAAT   1882
m150503_iid=2526862_0       TCAATTCTTGGAGCAGTAAACTTTATTACTAC-AATCATTAATATACGACCAAAAGGAAT   1566
m150506_iid=2567000_0       ------------------------------------------------------------   0
m150507_iid=3073271_0       TCAATTCTTGGAGCAGTAAACTTTATTACTAC-AATCATTAATATACGACCAAAAGGAAT   819
m150504_iid=3156087_0       TCAATTCTTGGAGCAGTAAACTTTATTACTAC-AATCATTAATATACGACCAAAAGGAAT   642
m150505_iid=2498502_0       ------------------------------------------------------------   0
m141102_iid=754227_0        TCAATTCTTGGAGCAGTAAACTTTATTACTAC-AATCATTAATATACGACCAAAAGGAAT   854
m150507_iid=3469514_0       TCAATTCTTGGAGCAGTAAACTTTATTACTAC-AATCATTAATATACGACCAAAAGGAAT   1412
Rdo_MitoGenome              TCAATTCTTGGAGCAGTAAACTTTATTACTAC-AATCATTAATATACGACCAAAAGGAAT   1887
m141101_iid=614933_0        TCAATTCTTGGAGCAGTAAACTTTATTACTAC-AATCATTAATATACGACCAAAAGGAAT   439
```

cox1

```
m141031_iid=347546_0        ------------------------------------------------------------   0
m150506_iid=3127414_0       ------------------------------------------------------------   0
m141102_iid=1245517_0       AACA-CAGAACGAATCCCCCTATTTGTATGATCAGTAGGGATCACAGCTTTGCTTTTAT-   89
m141107_iid=1405727_0       ------------------------------------------------------------   0
m141102_iid=987614_0        AACACCAGAACGAAT-CCCCTATTTGTATGATCAGTAGGGATCACAGCTTTGCTTTTAT-   215
m141108_iid=1701429_0       ------------------------------------------------------------   0
m141102_iid=987286_0        ------------------------------------------------------------   0
m150430_iid=3272125_0       ------------------------------------------------------------   0
m141102_iid=754225_0        AACACCAGAACGAATCCCCCTATTTGTATGATCAGTAGGGATCACAGCTTTGCTTTTAT-   863
m141107_iid=1409934_0       AACACCAGAACGAATCCCCCTATTTGTATGATCAGTAGGGATCACAGCTTTGCTTTTAT-   1393
m150503_iid=3416920_0       AACACCAGAACGAATCC--CTATTTGTATGATCAGTAGG-ATCACAGCTTTGCTTTTAT-   134
m141108_iid=1800121_0       ------------------------------------------------------------   0
m141031_iid=147540_0        AACACCAGAACGAATCCCCCTATTTGTATGATCAGTAGGGATCACAGCTTTGCTTTTAT-   279
m150507_iid=3674924_0       AAACCAAGAACGAATCC-CCTATTTGTATGATCAGTAGGGATCACAGCTTTGCTTTTATT   423
m150503_iid=2923751_0       AACACCAGAACGAATCCCCCTATTTGTATGATCAGTAGGGATCACAGCTTTGCTTTA-T-   277
m141031_iid=115118_0        ------------------------------------------------------------   0
m150430_iid=2722448_0       ------------------------------------------------------------   0
m150507_iid=3102647_0       ------------------------------------------------------------   0
m141108_iid=1811866_0       ------------------------------------------------------------   0
m150501_iid=2948495_0       ------------------------------------------------------------   0
m150504_iid=3238195_0       AACACCAGAACGAATCCCCCTATTTGTATGATCAGTAGGGATCACAGCTTTGCTTTTAT-   1000
m150503_iid=3388511_0       ------------------------------------------------------------   0
m150505_iid=3777611_0       AACACCAGAACGAAT-CCCCTATTTGTATGATCAGTAGGGATCACAGCTTTGCTTTTAT-   113
m150430_iid=2438833_0       AACACCAGAACGAATCCCCCTATTTGTATGATCAGTAGGGATCACAGCTTTGC-TTTAT-   468
m141108_iid=1698969_0       AACACCAGAACGAATCCCCCTATTTGTATGATCAGTAGGGATCACAGCTTTGCTTTTAT-   1587
m150430_iid=3059190_0       ------------------------------------------------------------   0
m141107_iid=1383067_0       ------------------------------------------------------------   0
```

```
m141101_iid=597008_0        ------------------------------------------------------------   0
m150506_iid=2596496_0       AACACCAGAACGAATCCCCCTATTTGTATGATCAGTAGGGATCACAGCTTTGCTTTTAT-   1939
m141102_iid=910051_0        AACACCAGAACGAATCCCCCTATTTGTATGATCAGTAGGGATCACAGCTTTGCTTTTAT-   1429
m150505_iid=3784017_0       AACACCAGAACGAATCCCCCTATTTGTATGATCAGTAGGGATCACAGCTTTGCTTTTAT-   229
m141101_iid=682151_0        ------------------------------------------------------------   0
m150501_iid=3748029_0       ------------------------------------------------------------   0
m150504_iid=2876272_0       AACACCAGAACGAAT-CCCCTATTTGTATGATCAGTAGGGATCACAGCTTTGCTTTTAT-   1703
NC_042820.1                 AACACCAGAACGAATCCCCCTATTTGTATGATCAGTAGGAATCACAGCTTTGCTTTTAT-   1946
m141102_iid=1228825_0       AACACCAGAACGAATCCCCCTATTTGTATGATCAGTAGGGATCACAGCTT---TTTTAT-   1773
m150504_iid=2876274_0       AACACCAGAACGAATCCCCCTATTTGTATGATCAGTAGGGATCACAGCTTTGCTTTTAT-   1776
m141030_iid=80036_0         AACACCAGAACGAATCCCCCTATTTGTATGATCAGTAGGGATCACAGCTTTGCTTTTAT-   1541
m150506_iid=2596494_0       AACACCAGAACGAATCCCCCTATTTGTATGATCAGTAGGGATCACAGCTTTGCTTTTAT-   1941
m150503_iid=2526862_0       AACACCAGAACGAATCCCCCTATTTGTATGATCAGTAGGGATCACAGCTTTGCTTTTAT-   1625
m150506_iid=2567000_0       ------------------------------------------------------------   0
m150507_iid=3073271_0       AACACCAGAACGAATCCCCCTATTTGTATGATCAGTAGGGATCACAGCTTTGCTTTTAT-   878
m150504_iid=3156087_0       AACACCAGAACGAATCCCCCTATTTGTATGATCAGTAGGGATCACAGCTTTGCTTTTAT-   701
m150505_iid=2498502_0       ------------------------------------------------------------   0
m141102_iid=754227_0        AACACCAGAACGAATCCCCCTATTTGTATGATCAGTAGGGATCACAGCTTTGCTTTTAT-   913
m150507_iid=3469514_0       AACACCAGAACGAATCCCCCTATTTGTATGATCAGTAGGGATCACAGCTTTGCTTTTAT-   1471
Rdo_MitoGenome              AACACCAGAACGAATCCCCCTATTTGTATGATCAGTAGGGATCACAGCTTTGCTTTTAT-   1946
m141101_iid=614933_0        AACACCAGAACGAATCCCCCTATTTGTATGATCAGTAGGGATCACAGCTTTGCTTTTAT-   498
```

cox1

```
m141031_iid=347546_0        ------------------------------------------------------------   0
m150506_iid=3127414_0       ------------------------------------------------------------   0
m141102_iid=1245517_0       TATTATCTCTTCCGGTTCTAGCTGGAGCTATCACTATATTA-TTAACAGACCGAA-ACTT   147
m141107_iid=1405727_0       ------------------------------------------------------------   0
m141102_iid=987614_0        TATTATCTCTTCCGGTTCTAGCTGGAGCTATCACTATATTA-TTAACAGACCGAA-ACTT   273
m141108_iid=1701429_0       ------------------------------------------------------------   0
m141102_iid=987286_0        ------------------------------------------------------------   0
m150430_iid=3272125_0       ------------------------------------------------------------   0
m141102_iid=754225_0        TATTATCTCTTCCGGTTCTAGCTGGAGCTATCACTATATTA-TTAACAGACCGAA-ACTT   921
m141107_iid=1409934_0       TATTATCTCTTCCGGTTCTAGCTGGAGCTATCACTATATTA-TTAACAGACCGAA-ACTT   1451
m150503_iid=3416920_0       TATTATCTCTTCCGGTTCTAGCTGGAGCTATCACTATAT----TAACAGACCGAAAACTT   190
m141108_iid=1800121_0       ------------------------------------------------------A-ACTT   5
m141031_iid=147540_0        TATTATCTCTTCCGGTTCTAGCTGGAGCTATCACTATATTA-TTAACAGACCGAA-ACTT   337
m150507_iid=3674924_0       TATTATCTCTTCCGGTTCTAGCTGGAGCTATCACTATATTATTTAACAGACCGAA-ACTT   482
m150503_iid=2923751_0       TATTATCTCTTCCGGTTCTAGCTGGAGCTATCACTATATTA-TTAACAGACCGAA-ACTT   335
m141031_iid=115118_0        ------------------------------------------------------------   0
m150430_iid=2722448_0       ------------------------------------------------------------   0
m150507_iid=3102647_0       ------------------------------------------------------------   0
m141108_iid=1811866_0       ------------------------------------------------------------   0
m150501_iid=2948495_0       ------------------------------------------------------------   0
m150504_iid=3238195_0       TATTATCTCTTCCGGTTCTAGCTGGAGCTATCACTATATTA-TTAACAGACCGAA-ACTT   1058
m150503_iid=3388511_0       ------------------------------------------------------------   0
m150505_iid=3777611_0       TATTATCTCTTCCGGTTCTAGCTGGAGCTATCACTATATTA-TTAACAGACCGAA-ACTT   171
m150430_iid=2438833_0       TATTATCTCTTCCGGTTCTAGCTGGAGCTATCACTATATTA-TTAACAGACCGAA-ACTT   526
m141108_iid=1698969_0       TATTATCTCTTCCGGTTCTAGCTGGAGCTATCACTATATTA-TTAACAGACCGAA-ACTT   1645
m150430_iid=3059190_0       ------------------------------------------------------------   0
m141107_iid=1383067_0       ------------------------------------------------------------   0
m141101_iid=597008_0        ------------------------------------------------------------   0
m150506_iid=2596496_0       TATTATCTCTTCCGGTTCTAGCTGGAGCTATCACTATATTA-TTAACAGACCGAA-ACTT   1997
m141102_iid=910051_0        TATTATCTCTTCCGGTTCTAGCTGGAGCTATCACTATATTA-TTAACAGACCGAA-ACTT   1487
m150505_iid=3784017_0       TATTATCTCTTCCGGTT-TAGCTGGAGCTATCACTATATTA-TTAACAGACCGAA-ACTT   286
m141101_iid=682151_0        ------------------------------------------------------------   0
m150501_iid=3748029_0       ------------------------------------------------------------   0
m150504_iid=2876272_0       TATTATCTCTTCCGGTTCTAGCTGGAGCTATCACTATATTA-TTAACAGACCGAA-ACTT   1761
NC_042820.1                 TATTATCTCTTCCGGTTCTAGCTGGAGCTATCACTATATTA-TTAACAGACCGAA-ACTT   2004
m141102_iid=1228825_0       TATTATCTCTTCCGGTTCTAGCTGGAGCTATCACTATATTA-TTAACAGACCGAA-ACTT   1831
m150504_iid=2876274_0       TATTATCTCTTCCGGTTCTAGCTGGAGCTATCACTATATTA-TTAACAGACCGAA-ACTT   1834
m141030_iid=80036_0         TATTATCTCTTCCGGTTCTAGCTGGAGCTATCACTATATTA-TTAACAGACCGAA-ACTT   1599
m150506_iid=2596494_0       TATTATCTCTTCCGGTTCTAGCTGGAGCTATCACTATATTA-TTAACAGACCGAA-ACTT   1999
m150503_iid=2526862_0       TATTATCTCTTCCGGTTCTAGCTGGAGCTATCACTATATTA-TTAACAGACCGAA-ACTT   1683
m150506_iid=2567000_0       ------------------------------------------------------------   0
m150507_iid=3073271_0       TATTATCTCTTCCGGTTCTAGCTGGAGCTATCACTATATTA-TTAACAGACCGAA-ACTT   936
m150504_iid=3156087_0       TATTATCTCTTCCGGTTCTAGCTGGAGCTATCACTATATTA-TTAACAGACCGAA-ACTT   759
m150505_iid=2498502_0       ------------------------------------------------------------   0
m141102_iid=754227_0        TATTATCTCTTCCGGTTCTAGCTGGAGCTATCACTATATTA-TTAACAGACCGAA-ACTT   971
m150507_iid=3469514_0       TATTATCTCTTCCGGTTCTAGCTGGAGCTATCACTATATTA-TTAACAGACCGAA-ACTT   1529
Rdo_MitoGenome              TATTATCTCTTCCGGTTCTAGCTGGAGCTATCACTATATTA-TTAACAGACCGAA-ACTT   2004
m141101_iid=614933_0        TATTATCTCTTCCGGTTCTAGCTGGAGCTATCACTATATTA-TTAACAGACCGAA-ACTT   556
```

cox1

```
m141031_iid=347546_0      ------------------------------------------------------------  0
m150506_iid=3127414_0     ------------------------------------------------------------  0
m141102_iid=1245517_0     AAATACTTCATT-TTTTGATCCAGCAGGAGGAGGGGACCCTATTTTGTATCAACACCTAT  206
m141107_iid=1405727_0     ------------------------------------------------------------  0
m141102_iid=987614_0      AAATACTTCATTT-TTTGATCCAGCAGGAGGAGGGGACCCTATTTTGTATCAACACCTAT  332
m141108_iid=1701429_0     ------------------------------------------------------------  0
m141102_iid=987286_0      ------------------------------------------------------------  0
m150430_iid=3272125_0     ------------------------------------------------------------  0
m141102_iid=754225_0      AAATACTTCATT-TTTTGATCCAGCAGGAGGAGGGGACCCTATTTTGTATCAACACCTAT  980
m141107_iid=1409934_0     AAATACTTCATT-TTTTGATCCAGCAGGAGGAGGGGACCCTATTTTGTATCAACACCTAT  1510
m150503_iid=3416920_0     AAATACTTCAT--TTTTGATCCAGCAGGAGGAGG-GACCCTATTTTGTATCAACACCTAT  247
m141108_iid=1800121_0     AAATACTTCATT-TTTTGATCCAGCAGGAGGAGGGGACCCTATTTTGTATCAACACT-AT  63
m141031_iid=147540_0      AAATACTTCATTT-TTTGATCCAGCAGGAGGAGGGGACCCTATTTTGTATCAACACCTAT  396
m150507_iid=3674924_0     AAATACTTCATTTTTTTGATCCAGCAGGAGGAGGGGACCCTATTTTGTATCAACACT-AT  541
m150503_iid=2923751_0     AAATACTTCATTT-TTTGATCCAGCAGGAGGAGGGGACCCTATTTTGTATCAACACCTAT  394
m141031_iid=115118_0      ------------------------------------------------------------  0
m150430_iid=2722448_0     ------------------------------------------------------------  0
m150507_iid=3102647_0     ------------------------------------------------------------  0
m141108_iid=1811866_0     ------------------------------------------------------------  0
m150501_iid=2948495_0     ------------------------------------------------------------  0
m150504_iid=3238195_0     AAATACTTCATTT-TTTGATCCAGCAGGAGGAGG-GACCCTATTTTGTATCAACACCTAT  1116
m150503_iid=3388511_0     ------------------------------------------------------------  0
m150505_iid=3777611_0     AAATACTTCATTT-TTTGATCCAGCAGGAGGAGGGGACCCTATTTTGTATCAACACCTAT  230
m150430_iid=2438833_0     AAATACTTCATTT-TTTGATCCAGCAGGAGGAGGGGACCCTATTTTGTATCAACA-CTAT  584
m141108_iid=1698969_0     AAATACTTCATTT-TTTGATCCAGCAGGAGGAGGGGACCCTATTTTGTATCAACACCTAT  1704
m150430_iid=3059190_0     ------------------------------------------------------------  0
m141107_iid=1383067_0     ------------------------------------------------------------  0
m141101_iid=597008_0      ------------------------------------------------------------  0
m150506_iid=2596496_0     AAATACTTCATTT-TTTGATCCAGCAGGAGGAGGGGACCCTATTTTGTATCAACACCTAT  2056
m141102_iid=910051_0      AAATACTTCATTT-TTTGATCCAGCAGGAGGAGGGGACCCTATTTTGTATCAACACCTAT  1546
m150505_iid=3784017_0     AAATACTTCATTT-TTTGATCCAGCAGGAGGAGGGGACCCTATTTTGTATCAACACCTAT  345
m141101_iid=682151_0      ------------------------------------------------------------  0
m150501_iid=3748029_0     ------------------------------------------------------------  0
m150504_iid=2876272_0     AAATACTTCATTT-TTTGATCCAGCAGGAGGAGGGGACCCTATTTTGTATCAACACCTAT  1820
NC_042820.1               AAATACTTCATTT-TTTGATCCAGCAGGAGGAGGGGACCCTATTTTGTATCAACACCTAT  2063
m141102_iid=1228825_0     AAATACTTCATTT-TTTGATCCAGCAGGAGGAGGGGACCCTATTTTGTATCAACACCTAT  1890
m150504_iid=2876274_0     AAATACTTCATTT-TTTGATCCAGCAGGAGGAGGGGACCCTATTTTGTATCAACACCTAT  1893
m141030_iid=80036_0       AAATACTTCATTT-TTTGATCCAGCAGGAGGAGGGGACCCTATTTTGTATCAACACCTAT  1658
m150506_iid=2596494_0     AAATACTTCATTT-TTTGATCCAGCAGGAGGAGGGGACCCTATTTTGTATCAACACCTAT  2058
m150503_iid=2526862_0     AAATACTTCATTT-TTTGATCCAGCAGGAGGAGGGGACCCTATTTTGTATCAACACCTAT  1742
m150506_iid=2567000_0     ------------------------------------------------------------  0
m150507_iid=3073271_0     AAATACTTCATTT-TTTGATCCAGCAGGAGGAGGGGACCCTATTTTGTATCAACACCTAT  995
m150504_iid=3156087_0     AAATACTTCATTT-TTTGATCCAGCAGGAGGAGGGGACCCTATTTTGTATCAACACCTAT  818
m150505_iid=2498502_0     ------------------------------------------------------------  0
m141102_iid=754227_0      AAATACTTCATTT-TTTGATCCAGCAGGAGGAGGGGACCCTATTTTGTATCAACACCTAT  1030
m150507_iid=3469514_0     AAATACTTCATTT-TTTGATCCAGCAGGAGGAGGGGACCCTATTTTGTATCAACACCTAT  1588
Rdo_MitoGenome            AAATACTTCATTT-TTTGATCCAGCAGGAGGAGGGGACCCTATTTTGTATCAACACCTAT  2063
m141101_iid=614933_0      AAATACTTCATTT-TTTGATCCAGCAGGAGGAGGGGACCCTATTTTGTATCAACACCTAT  615
```

cox1

```
m141031_iid=347546_0      ------------------------------------------------------------  0
m150506_iid=3127414_0     ------------------------------------------------------------  0
m141102_iid=1245517_0     TCTGATTTTTTGG-ACATCCAGAAGTTTACATTTTAATCCTACA-GGATTTGGTAT-AAT  263
m141107_iid=1405727_0     ------------------------------------------------------------  0
m141102_iid=987614_0      TCTGATTTTTTGGA-CATCCAGAAGTTTACATTTTAATCCTACCAGGATTTGGTATAA-T  390
m141108_iid=1701429_0     ------------------------------------------------------------  0
m141102_iid=987286_0      ------------------------------------------------------------  0
m150430_iid=3272125_0     ------------------------------------------------------------  0
m141102_iid=754225_0      TCTGATTTTTTG-GACATCCAGAAGTTTACATTTTAATCCTACCAGGATTTGGTATAA-T  1038
m141107_iid=1409934_0     TCTGATTTTTTG-GACATCCAGAAGTTTACATTTTAATCCTACCAGGATTTGGTATAA-T  1568
m150503_iid=3416920_0     TCTGATTTTTTG-GACATCCAGAAGTTTACATTTTAATCCTACCAGGATTTGGTATAA-T  305
m141108_iid=1800121_0     TCTGATTTTTTG-GACATCCAGAAGTTTACATTTTAATCCTACCAGGATTTGGTATAA-T  121
m141031_iid=147540_0      TCTGATTTTTTGGA-CATCCAGAAGTTTACATTTTAATCCTACCAGGATTTGGTATAA-T  454
m150507_iid=3674924_0     TCTGATTTTTTG-GACATCCAGAAGTTTACATTTTAATCCTACCAGGATTTGGTATAATT  600
m150503_iid=2923751_0     TCTGATTTTTTGG-ACATCCAGAAGTTTACATTTTAATCCTACCAGGATTTGGTATAA-T  452
m141031_iid=115118_0      ------------------------------------------------------------  0
m150430_iid=2722448_0     ------------------------------------------------------------  0
m150507_iid=3102647_0     ------------------------------------------------------------  0
m141108_iid=1811866_0     ------------------------------------------------------------  0
```

```
m150501_iid=2948495_0      ------------------------------------------------------------   0
m150504_iid=3238195_0      TCTGATTTTTTGG-ACATCCAGAAGTTTACATTTTAATCCTACCAGGATTTGGTATAA-T   1174
m150503_iid=3388511_0      ------------------------------------------------------------   0
m150505_iid=3777611_0      TCTGATTTTTTGGACCATCCAGAAGTTTACATTTTAATCCTACCAGGATTTGGTATAA-T   289
m150430_iid=2438833_0      TCTGATTTTTTGGA-CATCCAGAAGTTTACATTTTAATCCTACCAGGATTTGGTATAA-T   642
m141108_iid=1698969_0      TCTGATTTTTTGGA-CATCCAGAAGTTTACATTTTAATCCTACCAGGATTTGGTATAA-T   1762
m150430_iid=3059190_0      ------------------------------------------------------------   0
m141107_iid=1383067_0      ------------------------------------------------------------   0
m141101_iid=597008_0       ------------------------------------------------------------   0
m150506_iid=2596496_0      TCTGATTTTTTGGA-CATCCAGAAGTTTACATTTTAATCCTACCAGGATTTGGTATAA-T   2114
m141102_iid=910051_0       TCTGATTTTTTGGA-CATCCAGAAGTTTACATTTTAATCCTACCAGGATTTGGTATAA-T   1604
m150505_iid=3784017_0      TCTGATTTTTTGGA-CATCCAGAAGTTTACATTTTAATCCTACCAGGATTTGGTATAA-T   403
m141101_iid=682151_0       ------------------------------------------------------------   0
m150501_iid=3748029_0      ------------------------------------------CCAGGATTTGGTATAA-T   17
m150504_iid=2876272_0      TCTGATTTTTTGGA-CATCCAGAAGTTTACATTTTAATCCTACCAGGATTTGGTATAA-T   1878
NC_042820.1                TCTGATTTTTTGGA-CATCCAGAAGTTTACATTTTAATCCTACCAGGATTTGGTATAA-T   2121
m141102_iid=1228825_0      TCTGATTTTTTGGA-CATCCAGAAGTTTACATTTTAATCCTACCAGGATTTGGTATAA-T   1948
m150504_iid=2876274_0      TCTGATTTTTTGGA-CATCCAGAAGTTTACATTTTAATCCTACCAGGATTTGGTATAA-T   1951
m141030_iid=80036_0        TCTGATTTTTTGGA-CATCCAGAAGTTTACATTTTAATCCTACCAGGATTTGGTATAA-T   1716
m150506_iid=2596494_0      TCTGATTTTTTGGA-CATCCAGAAGTTTACATTTTAATCCTACCAGGATTTGGTATAA-T   2116
m150503_iid=2526862_0      TCTGATTTTTTGGA-CATCCAGAAGTTTACATTTTAATCCTACCAGGATTTGGTATAA-T   1800
m150506_iid=2567000_0      ------------------------------------------------------------   0
m150507_iid=3073271_0      TCTGATTTTTTGGA-CATCCAGAAGTTTACATTTTAATCCTACCAGGATTTGGTATAA-T   1053
m150504_iid=3156087_0      TCTGATTTTTTGGA-CATCCAGAAGTTTACATTTTAATCCTACCAGGATTTGGTATAA-T   876
m150505_iid=2498502_0      ------------------------------------------------------------   0
m141102_iid=754227_0       TCTGATTTTTTGGA-CATCCAGAAGTTTACATTTTAATCCTACCAGGATTTGGTATAA-T   1088
m150507_iid=3469514_0      TCTGATTTTTTGGA-CATCCAGAAGTTTACATTTTAATCCTACCAGGATTTGGTATAA-T   1646
Rdo_MitoGenome             TCTGATTTTTTGGA-CATCCAGAAGTTTACATTTTAATCCTACCAGGATTTGGTATAA-T   2121
m141101_iid=614933_0       TCTGATTTTTTGGA-CATCCAGAAGTTTACATTTTAATCCTACCAGGATTTGGTATAA-T   673
```

cox1

```
m141031_iid=347546_0       ------------------------------------------------------------   0
m150506_iid=3127414_0      ------------------------------------------------------------   0
m141102_iid=1245517_0      TTCTCATATTATTAGACATGAAAGAGGAAAAAAGGAAACCTTTGGTTCTCTAGGGATAAT   323
m141107_iid=1405727_0      ------------------------------------------------------------   0
m141102_iid=987614_0       TTCTCATATTATTAGACATGAAAGAGGAAAAAAGGAAACCTTTGGTTCTCTAGGGATAAT   450
m141108_iid=1701429_0      ------------------------------------------------------------   0
m141102_iid=987286_0       ------------------------------------------------------------   0
m150430_iid=3272125_0      ------------------------------------------------------------   0
m141102_iid=754225_0       TTCTCATATTATTAGACATGAAAGAGGAAAAAAGGAAACCTTTGGTTCTCTAGGGATAAT   1098
m141107_iid=1409934_0      TTCTCATATTATTAGACATGAAAGAGGAAAAAAGGAAACCTTTGGTTCTCTAGGGATAAT   1628
m150503_iid=3416920_0      TTCTCATATTATTAGACATGAAAGAGGAAAAAAGGAAACCTTTGGTTCTCTAGGGATAAT   365
m141108_iid=1800121_0      TTCTCATATTATTAGACATGAAAGAGGAAAAAAGGAAACCTTTGGTTCTCTAGGGATAAT   181
m141031_iid=147540_0       TTCTCATATTATTAGACATGAAAGAGGAAAAAAGGAAACCTTTGGTTCTCTAGGGATAAT   514
m150507_iid=3674924_0      TTCTCATATTATTAGACATGAAAGAGGAAAAAAGGAAACCTTTGGTTCTCTAGGGATAAT   660
m150503_iid=2923751_0      TTCTCATATTATTAGACATGAAAGAGGAAAAAAGGAAACCTTTGGTTCTCTAGGGATAAT   512
m141031_iid=115118_0       ------------------------------------------------------------   0
m150430_iid=2722448_0      ------------------------------------------------------------   0
m150507_iid=3102647_0      ------------------------------------------------------------   0
m141108_iid=1811866_0      ------------------------------------------------------------   0
m150501_iid=2948495_0      ------------------------------------------------------------   0
m150504_iid=3238195_0      TTCTCATATTATTAGACATGAAAGAGGAAAAAAGGAAACCTTTGGTTCTCTAGGGATAAT   1234
m150503_iid=3388511_0      ------------------------------------------------------------   0
m150505_iid=3777611_0      TTCTCATATTATTAGACATGAAAGAGGAAAAAAGGAAACCTTTGGTTCTCTAGGGATAAT   349
m150430_iid=2438833_0      T--TCATATTATTAGACATGAAAGAGGAAAAAAGGAAACCTTTGGTTCTCTAGGGATAAT   700
m141108_iid=1698969_0      TTCTCATATTATTAGACATGAAAGAGGAAAAAAGGAAACCTTTGGTTCTCTAGGGATAAT   1822
m150430_iid=3059190_0      ------------------------------------------------------------   0
m141107_iid=1383067_0      ------------------------------------------------------------   0
m141101_iid=597008_0       ------------------------------------------------------------   0
m150506_iid=2596496_0      TTCTCATATTATTAGACATGAAAGAGGAAAAAAGGAAACCTTTGGTTCTCTAGGGATAAT   2174
m141102_iid=910051_0       TTCTCATATTATTAGACATGAAAGAGGAAAAAAGGAAACCTTTGGTTCTCTAGGGATAAT   1664
m150505_iid=3784017_0      TTCTCATATTATTAGACATGAAAGAGGAAAAAAGGAAACCTTTGGTTCTCTAGGGATAAT   463
m141101_iid=682151_0       ------------------------------------------------------------   0
m150501_iid=3748029_0      TTCTCATATTATTAGACATGAAAGAGGAAAAAAGGAAACCTTTGGTTCTCTAGGGATAAT   77
m150504_iid=2876272_0      TTCTCATATTATTAGACATGAAAGAGGAAAAAAGGAAACCTTTGGTTCTCTAGGGATAAT   1938
NC_042820.1                TTCTCATATTATTAGACATGAAAGAGGAAAAAAGGAAACCTTTGGTTCTCTAGGGATAAT   2181
m141102_iid=1228825_0      TTCTCATATTATTAGACATGAAAGAGGAAAAAAGGAAACCTTTGGTTCTCTAGGGATAAT   2008
m150504_iid=2876274_0      TTCTCATATTATTAGACATGAAAGAGGAAAAAAGGAAACCTTTGGTTCTCTAGGGATAAT   2011
m141030_iid=80036_0        TTCTCATATTATTAGACATGAAAGAGGAAAAAAGGAAACCTTTGGTTCTCTAGGGATAAT   1776
m150506_iid=2596494_0      TTCTCATATTATTAGACATGAAAGAGGAAAAAAGGAAACCTTTGGTTCTCTAGGGATAAT   2176
m150503_iid=2526862_0      TTCTCATATTATTAGACATGAAAGAGGAAAAAAGGAAACCTTTGGTTCTCTAGGGATAAT   1860
```

```
m150506_iid=2567000_0      ------------------------------------------------------------   0
m150507_iid=3073271_0      TTCTCATATTATTAGACATGAAAGAGGAAAAAAGGAAACCTTTGGTTCTCTAGGGATAAT   1113
m150504_iid=3156087_0      TTCTCATATTATTAGACATGAAAGAGGAAAAAAGGAAACCTTTGGTTCTCTAGGGATAAT   936
m150505_iid=2498502_0      ------------------------------------------------------------   0
m141102_iid=754227_0       TTCTCATATTATTAGACATGAAAGAGGAAAAAAGGAAACCTTTGGTTCTCTAGGGATAAT   1148
m150507_iid=3469514_0      TTCTCATATTATTAGACATGAAAGAGGAAAAAAGGAAACCTTTGGTTCTCTAGGGATAAT   1706
Rdo_MitoGenome             TTCTCATATTATTAGACATGAAAGAGGAAAAAAGGAAACCTTTGGTTCTCTAGGGATAAT   2181
m141101_iid=614933_0       TTCTCATATTATTAGACATGAAAGAGGAAAAAAGGAAACCTTTGGTTCTCTAGGGATAAT   733

                                                     cox1
m141031_iid=347546_0       ------------------------------------------------------------   0
m150506_iid=3127414_0      ------------------------------------------------------------   0
m141102_iid=1245517_0      -TTACGCAATAATAGCAATTGGACTATTAGGATTTATTGTATGAGCACATCACATATTTA   382
m141107_iid=1405727_0      ------------------------------------------------------------   0
m141102_iid=987614_0       -TTACGCAATAATAGCAATTGGACTATTAGGATTTATTGTATGAGCACATCACATATTTA   509
m141108_iid=1701429_0      ------------------------------------------------------------   0
m141102_iid=987286_0       ------------------------------------------------------------   0
m150430_iid=3272125_0      ------------------------------------------------------------   0
m141102_iid=754225_0       -TTACGCAATAATAGCAATTGGACTATTAGGATTTATTGTATGAGCACATCACATATTTA   1157
m141107_iid=1409934_0      -TTACGCAATAATAGCAATTGGACTATTAGGATTTATTGTATGAGCACATCACATATTTA   1687
m150503_iid=3416920_0      -TTACGCAATAATAGCAATTGGACTATTAGGATTTATTGTATGAGCACATCACATATTTA   424
m141108_iid=1800121_0      -TTACGCAATAATAGCAATTGGACTATTAGGATTTATTGTATGAGCACATCACATATTTA   240
m141031_iid=147540_0       -TTACGCAATAATAGCAATTGGACTATTAGGATTTATTGTATGAGCACATCACATATTTA   573
m150507_iid=3674924_0      TTAACGCAATAATAGCAATTGGACTATTAGGATTTATTGTATGAGCACATCACATA-TTA   719
m150503_iid=2923751_0      -TTACGCAATAATAGCAATTGGACTATTAGGATTTATTGTATGAGCACATCACATATTTA   571
m141031_iid=115118_0       ------------------------------------------------------------   0
m150430_iid=2722448_0      ------------------------------------------------------------   0
m150507_iid=3102647_0      ------------------------------------------------------------   0
m141108_iid=1811866_0      ------------------------------------------------------------   0
m150501_iid=2948495_0      ------------------------------------------------------------   0
m150504_iid=3238195_0      -TTACGCAATAATAGCAATTGGACTATTAGGATTTATTGTATGAGCACATCACATATTTA   1293
m150503_iid=3388511_0      ------------------------------------------------------------   0
m150505_iid=3777611_0      -TTACGCAATAATAGCAATTGGACTATTAGGATTTATTGTATGAGCACATCACATATTTA   408
m150430_iid=2438833_0      -TTACGCAATAATAGCAATTGGACTATTAGGATTTATTGTATGAGCACATCACATATTTA   759
m141108_iid=1698969_0      -TTACGCAATAATAGCAATTGGACTATTAGGATTTATTGTATGAGCACATCACATATTTA   1881
m150430_iid=3059190_0      ------------------------------------------------------------   0
m141107_iid=1383067_0      ------------------------------------------------------------   0
m141101_iid=597008_0       ------------------------------------------------------------   0
m150506_iid=2596496_0      -TTACGCAATAATAGCAATTGGACTATTAGGATTTATTGTATGAGCACATCACATATTTA   2233
m141102_iid=910051_0       -TTACGCAATAATAGCAATTGGACTATTAGGATTTATTGTATGAGCACATCACATATTTA   1723
m150505_iid=3784017_0      -TTACGCAATAATAGCAATTGGACTATTAGGATTTATTGTATGAGCACATCACATATTTA   522
m141101_iid=682151_0       ------------------------------------------------------------   0
m150501_iid=3748029_0      -TTACGCAATAATAGCAATTGGACTATTAGGATTTATTGTATGAGCACATCACATATTTA   136
m150504_iid=2876272_0      -TTACGCAATAATAGCAATTGGACTATTAGGATTTATTGTATGAGCACATCACATATTTA   1997
NC_042820.1                -TTACGCAATAATAGCAATTGGACTATTAGGATTTATTGTATGAGCACATCACATATTTA   2240
m141102_iid=1228825_0      -TTACGCAATAATAGCAATTGGACTATTAGGATTTATTGTATGAGCACATCACATATTTA   2067
m150504_iid=2876274_0      -TTACGCAATAATAGCAATTGGACTATTAGGATTTATTGTATGAGCACATCACATATTTA   2070
m141030_iid=80036_0        -TTACGCAATAATAGCAATTGGACTATTAGGATTTATTGTATGAGCACATCACATATTTA   1835
m150506_iid=2596494_0      -TTACGCAATAATAGCAATTGGACTATTAGGATTTATTGTATGAGCACATCACATATTTA   2235
m150503_iid=2526862_0      -TTACGCAATAATAGCAATTGGACTATTAGGATTTATTGTATGAGCACATCACATATTTA   1919
m150506_iid=2567000_0      ------------------------------------------------------------   0
m150507_iid=3073271_0      -TTACGCAATAATAGCAATTGGACTATTAGGATTTATTGTATGAGCACATCACATATTTA   1172
m150504_iid=3156087_0      -TTACGCAATAATAGCAATTGGACTATTAGGATTTATTGTATGAGCACATCACATATTTA   995
m150505_iid=2498502_0      ------------------------------------------------------------   0
m141102_iid=754227_0       -TTACGCAATAATAGCAATTGGACTATTAGGATTTATTGTATGAGCACATCACATATTTA   1207
m150507_iid=3469514_0      -TTACGCAATAATAGCAATTGGACTATTAGGATTTATTGTATGAGCACATCACATATTTA   1765
Rdo_MitoGenome             -TTACGCAATAATAGCAATTGGACTATTAGGATTTATTGTATGAGCACATCACATATTTA   2240
m141101_iid=614933_0       -TTACGCAATAATAGCAATTGGACTATTAGGATTTATTGTATGAGCACATCACATATTTA   792

                                                     cox1
m141031_iid=347546_0       ------------------------------------------------------------   0
m150506_iid=3127414_0      ------------------------------------------------------------   0
m141102_iid=1245517_0      CTGTAGGAATAGACGTAGATACCCGAGCATATTTTACTTCAGCAACAATAATTATTGCAG   442
m141107_iid=1405727_0      ------------------------------------------------------------   0
m141102_iid=987614_0       CTGTAGGAATAGACGTAGATACCCGAGCATATTTTACTTCAGCAACAATAATTATTGCAG   569
m141108_iid=1701429_0      ------------------------------------------------------------   0
m141102_iid=987286_0       ------------------------------------------------------------   0
m150430_iid=3272125_0      -------------CGTAGATACCCGAGCATAT-TTACTTCAGCAACAATAATTATTGCAG   46
m141102_iid=754225_0       CTGTAGGAATAGACGTAGATACCCGAGCATATTTTACTTCAGCAACAATAATTATTGCAG   1217
m141107_iid=1409934_0      CTGTAGGAATAGACGTAGATACCCGAGCATATTTTACTTCAGCAACAATAATTATTGCAG   1747
m150503_iid=3416920_0      CTGTAGGAATAGACGTAGATA-CCGAGCATATTTTACTTCAGCAACAATAATTATTGCAG   483
```

```
m141108_iid=1800121_0      CTGTAGGAATAGACGTAGATACCCGAGCATATTTTACTTCAGCAACAATAATTATTGCAG   300
m141031_iid=147540_0       CTGTAGGAATAGACGTAGATACCCGAGCATATTTTACTTCAGCAACAATAATTATTGCAG   633
m150507_iid=3674924_0      CTGTAGGAATAGACGTAGATACCCGAGCATATTTTACTTCAGCAACAATAATTATTGCAG   779
m150503_iid=2923751_0      CTGTAGGAATAGACGTAGATACCCGAGCATATTTTACTTCAGCAACAATAATTATTGCAG   631
m141031_iid=115118_0       ------------------------------------------------------------   0
m150430_iid=2722448_0      ------------------------------------------------------------   0
m150507_iid=3102647_0      ------------------------------------------------------------   0
m141108_iid=1811866_0      ------------------------------------------------------------   0
m150501_iid=2948495_0      ------------------------------------------------------------   0
m150504_iid=3238195_0      CTGTAGGAATAGACGTAGATACCCGAGCATATTTTACTTCAGCAACAATAATTATTGCAG   1353
m150503_iid=3388511_0      ------------------------------------------------------------   0
m150505_iid=3777611_0      CTGTAGGAATAGACGTAGATACCCGAGCATATTTTACTTCAGCAACAATAATTATTGCAG   468
m150430_iid=2438833_0      CTGTAGGAATAGACGTAGATA-CCGAGCATATTTTACTTCAGCAACAATAATTATTGCAG   818
m141108_iid=1698969_0      CTGTAGGAATAGACGTAGATACCCGAGCATATTTTACTTCAGCAACAATAATTATTGCAG   1941
m150430_iid=3059190_0      ------------------------------------------------------------   0
m141107_iid=1383067_0      ------------------------------------------------------------   0
m141101_iid=597008_0       ------------------------------------------------------------   0
m150506_iid=2596496_0      CTGTAGGAATAGACGTAGATACCCGAGCATATTTTACTTCAGCAACAATAATTATTGCAG   2293
m141102_iid=910051_0       CTGTAGGAATAGACGTAGATACCCGAGCATATTTTACTTCAGCAACAATAATTATTGCAG   1783
m150505_iid=3784017_0      CTGTAGGAATAGACGTAGATACCCGAGCATATTTTACTTCAGCAACAATAATTATTGCAG   582
m141101_iid=682151_0       ------------------------------------------------------------   0
m150501_iid=3748029_0      CTGTAGGAATAGACGTAGATACCCGAGCATATTTTACTTCAGCAACAATAATTATTGCAG   196
m150504_iid=2876272_0      CTGTAGGAATAGACGTAGATACCCGAGCATATTTTACTTCAGCAACAATAATTATTGCAG   2057
NC_042820.1                CTGTAGGAATAGACGTAGATACCCGAGCATATTTTACTTCAGCAACAATAATTATTGCAG   2300
m141102_iid=1228825_0      CTGTAGGAATAGACGTAGATACCCGAGCATATTTTACTTCAGCAACAATAATTATTGCAG   2127
m150504_iid=2876274_0      CTGTAGGAATAGACGTAGATACCCGAGCATATTTTACTTCAGCAACAATAATTATTGCAG   2130
m141030_iid=80036_0        CTGTAGGAATAGACGTAGATACCCGAGCATATTTTACTTCAGCAACAATAATTATTGCAG   1895
m150506_iid=2596494_0      CTGTAGGAATAGACGTAGATACCCGAGCATATTTTACTTCAGCAACAATAATTATTGCAG   2295
m150503_iid=2526862_0      CTGTAGGAATAGACGTAGATACCCGAGCATATTTTACTTCAGCAACAATAATTATTGCAG   1979
m150506_iid=2567000_0      ------------------------------------------------------------   0
m150507_iid=3073271_0      CTGTAGGAATAGACGTAGATACCCGAGCATATTTTACTTCAGCAACAATAATTATTGCAG   1232
m150504_iid=3156087_0      CTGTAGGAATAGACGTAGATACCCGAGCATATTTTACTTCAGCAACAATAATTATTGCAG   1055
m150505_iid=2498502_0      ------------------------------------------------------------   0
m141102_iid=754227_0       CTGTAGGAATAGACGTAGATACCCGAGCATATTTTACTTCAGCAACAATAATTATTGCAG   1267
m150507_iid=3469514_0      CTGTAGGAATAGACGTAGATACCCGAGCATATTTTACTTCAGCAACAATAATTATTGCAG   1825
Rdo_MitoGenome             CTGTAGGAATAGACGTAGATACCCGAGCATATTTTACTTCAGCAACAATAATTATTGCAG   2300
m141101_iid=614933_0       CTGTAGGAATAGACGTAGATACCCGAGCATATTTTACTTCAGCAACAATAATTATTGCAG   852
```

cox1

```
m141031_iid=347546_0       ------------------------------------------------------------   0
m150506_iid=3127414_0      ------------------------------------------------------------   0
m141102_iid=1245517_0      TTCCAACCGGAATTAAAGTATTTAG-ATGATTAGCTACCCTC-CACGGAACACAAATAAA   500
m141107_iid=1405727_0      ------------------------------------------------------------   0
m141102_iid=987614_0       TTCCAACCGGAATTAAAGTATTTAG-ATGATTAGCTACCCTC-CACGGAACACAAATAAA   627
m141108_iid=1701429_0      ------------------------------------------------------------   0
m141102_iid=987286_0       ------------------------------------------------------------   0
m150430_iid=3272125_0      TTCCAACCGGAATTAAAGTATTTAGAATGATTAGCTACCCTCCCACGGAACACAAATAAA   106
m141102_iid=754225_0       TTCCAACCGGAATTAAAGTATTTAG-ATGATTAGCTACCCTC-CACGGAACACAAATAAA   1275
m141107_iid=1409934_0      TTCCAACCGGAATTAAAGTATTTAG-ATGATTAGCTACCCTC-CACGGAACACAAATAAA   1805
m150503_iid=3416920_0      TTCCAACCGGAATTAAAGTATTTAG-ATGATTAGCTACCCTC-CACGGAACACAAATAAA   541
m141108_iid=1800121_0      TTCCAACCGGAATTAAAGTATTTAG-ATGATTAGCTAC-CTC-CACGGAACACAAATA-A   356
m141031_iid=147540_0       TTCCAACCGGAATTAAAGTATTTAG-ATGATTAGCTACCCTC-CACGGAACACAAATAAA   691
m150507_iid=3674924_0      TTCCAACCGGAATTAAAGTATTTAG-ATGATTAGCTACCTCT-CACGGAACACAAATAAA   837
m150503_iid=2923751_0      TTCCAACCGGAATTAAAGTATTTAG-ATGATTAGCTACCCTC-CACGGAACACAAATAAA   689
m141031_iid=115118_0       ------------------------------------------------------------   0
m150430_iid=2722448_0      ------------------------------------------------------------   0
m150507_iid=3102647_0      ------------------------------------------------------------   0
m141108_iid=1811866_0      ------------------------------------------------------------   0
m150501_iid=2948495_0      ------------------------------------------------------------   0
m150504_iid=3238195_0      TTCCAACCGGAATTAAAGTATTTAG-ATGATTAGCTACCCTC-CACGGAACACAAATAAA   1411
m150503_iid=3388511_0      ------------------------------------------------------------   0
m150505_iid=3777611_0      TTCCAACCGGAATTAAAGTATTTAG-ATGATTAGCTACCCTC-CACGGAACACAAATAAA   526
m150430_iid=2438833_0      TTCCAACCGGAATTAAAGTATTTAG-ATGATTAGCTACCCTC-CACGGAACACAAATAAA   876
m141108_iid=1698969_0      TTCCAACCGGAATTAAAGTATTTAG-ATGATTAGCTACCCTC-CACGGAACACAAATAAA   1999
m150430_iid=3059190_0      ------------------------------------------------------------   0
m141107_iid=1383067_0      ------------------------------------------------------------   0
m141101_iid=597008_0       ------------------------------------------------------------   0
m150506_iid=2596496_0      TTCCAACCGGAATTAAAGTATTTAG-ATGATTAGCTACCCTC-CACGGAACACAAATAAA   2351
m141102_iid=910051_0       TTCCAACCGGAATTAAAGTATTTAG-ATGATTAGCTACCCTC-CACGGAACACAAATAAA   1841
m150505_iid=3784017_0      TTCCAACCGGAATTAAAGTATTTAG-ATGATTAGCTACCCTC-CACGGAACACAAATAAA   640
m141101_iid=682151_0       ------------------------------------------------------------   0
```

```
m150501_iid=3748029_0      TTCCAACCGGAATTAAAGTATTTAG-ATGATTAGCTACCCTC-CACGGAACACAAATAAA   254
m150504_iid=2876272_0      TTCCAACCGGAATTAAAGTATTTAG-ATGATTAGCTACCCTC-CACGGAACACAAATAAA   2115
NC_042820.1                TTCCAACCGGAATTAAAGTATTTAG-ATGATTAGCTACCCTC-CACGGAACACAAATAAA   2358
m141102_iid=1228825_0      TTCCAACCGGAATTAAAGTATTTAG-ATGATTAGCTACCCTC-CACGGAACACAAATAAA   2185
m150504_iid=2876274_0      TTCCAACCGGAATTAAAGTATTTAG-ATGATTAGCTACCCTC-CACGGAACACAAATAAA   2188
m141030_iid=80036_0        TTCCAACCGGAATTAAAGTATTTAG-ATGATTAGCTACCCTC-CACGGAACACAAATAAA   1953
m150506_iid=2596494_0      TTCCAACCGGAATTAAAGTATTTAG-ATGATTAGCTACCCTC-CACGGAACACAAATAAA   2353
m150503_iid=2526862_0      TTCCAACCGGAATTAAAGTATTTAG-ATGATTAGCTACCCTC-CACGGAACACAAATAAA   2037
m150506_iid=2567000_0      ------------------------------------------------------------   0
m150507_iid=3073271_0      TTCCAACCGGAATTAAAGTATTTAG-ATGATTAGCTACCCTC-CACGGAACACAAATAAA   1290
m150504_iid=3156087_0      TTCCAACCGGAATTAAAGTATTTAG-ATGATTAGCTACCCTC-CACGGAACACAAATAAA   1113
m150505_iid=2498502_0      ------------------------------------------------------------   0
m141102_iid=754227_0       TTCCAACCGGAATTAAAGTATTTAG-ATGATTAGCTACCCTC-CACGGAACACAAATAAA   1325
m150507_iid=3469514_0      TTCCAACCGGAATTAAAGTATTTAG-ATGATTAGCTACCCTC-CACGGAACACAAATAAA   1883
Rdo_MitoGenome             TTCCAACCGGAATTAAAGTATTTAG-ATGATTAGCTACCCTC-CACGGAACACAAATAAA   2358
m141101_iid=614933_0       TTCCAACCGGAATTAAAGTATTTAG-ATGATTAGCTACCCTC-CACGGAACACAAATAAA   910
```

cox1

```
m141031_iid=347546_0       ------------------------------------------------------------   0
m150506_iid=3127414_0      ------------------------------------------------------------   0
m141102_iid=1245517_0      TTATTCACCTTCAATAA-TTGATCATTAGGATTTGTCTTTCTATTTACCGTAGGAG-ATT   558
m141107_iid=1405727_0      ------------------------------------------------------------   0
m141102_iid=987614_0       TTATTCACCTTCAATAATATGATCATTAGGATTTGTCTTTCTATTTACCGTAGGAGGATT   687
m141108_iid=1701429_0      ------------------------------------------------------------   0
m141102_iid=987286_0       ------------------------------------------------------------   0
m150430_iid=3272125_0      TTATTCACCTTCAATAATATGATCATTAGGATTTGTCTTTCTATTTACCGTAGGAGGATT   166
m141102_iid=754225_0       TTATTCACCTTCAATAATATGATCATTAGGATTTGTCTTTCTATTTACCGTAGGAGGATT   1335
m141107_iid=1409934_0      TTATTCACCTTCAATAATATGATCATTAGGATTTGTCTTTCTATTTACCGTAGGAGGATT   1865
m150503_iid=3416920_0      TTATTCACCTTCAATAATATGATCATTAGGATTTGTCTTTCTATTTACCGTAGGAGGATT   601
m141108_iid=1800121_0      TTATTCACCTTCAATAATATGATCATTAGGATTTGTCTTTCTATTTACCGTAGGAGGATT   416
m141031_iid=147540_0       TTATTCACCTTCAATAATATGATCATTAGGATTTGTCTTTCTATTTACCGTAGGAGGATT   751
m150507_iid=3674924_0      TTATTCACCTTCAATAATATGATCATTAGGATTTGTCTTTCTATTTACCGTAGGAGGATT   897
m150503_iid=2923751_0      TTATTCACCTTCAATAATATGATCATTAGGATTTGTCTTTCTATTTACCGTAGGAGGATT   749
m141031_iid=115118_0       ------------------------------------------------------------   0
m150430_iid=2722448_0      ------------------------------------------------------------   0
m150507_iid=3102647_0      ------------------------------------------------------------   0
m141108_iid=1811866_0      ------------------------------------------------------------   0
m150501_iid=2948495_0      ------------------------------------------------------------   0
m150504_iid=3238195_0      TTATTCACCTTCAATAATATGATCATTAGGATTTGTCTTTCTATTTACCGTAGGAGGATT   1471
m150503_iid=3388511_0      ------------------------------------------------------------   0
m150505_iid=3777611_0      TTATTCACCTTCAATAATATGATCATTAGGATTTGTCTTTCTATTTACCGTAGGAGGATT   586
m150430_iid=2438833_0      TTATTCACCTTCAATAATATGATCATTAGGATTTGTCTTTCTATTTACCGTAGGAGGATT   936
m141108_iid=1698969_0      TTATTCACCTTCAATAATATGATCATTAGGATTTGTCTTTCTATTTACCGTAGGAGGATT   2059
m150430_iid=3059190_0      ------------------------------------------------------------   0
m141107_iid=1383067_0      ------------------------------------------------------------   0
m141101_iid=597008_0       ------------------------------------------------------------   0
m150506_iid=2596496_0      TTATTCACCTTCAATAATATGATCATTAGGATTTGTCTTTCTATTTACCGTAGGAGGATT   2411
m141102_iid=910051_0       TTATTCACCTTCAATAATATGATCATTAGGATTTGTCTTTCTATTTACCGTAGGAGGATT   1901
m150505_iid=3784017_0      TTATTCACCTTCAATAATATGATCATTAGGATTTGTCTTTCTATTTACCGTAGGAGGATT   700
m141101_iid=682151_0       ------------------------------------------------------------   0
m150501_iid=3748029_0      TTATTCACCTTCAATAATATGATCATTAGGATTTGTCTTTCTATTTACCGTAGGAGGATT   314
m150504_iid=2876272_0      TTATTCACCTTCAATAATATGATCATTAGGATTTGTCTTTCTATTTACCGTAGGAGGATT   2175
NC_042820.1                TTATTCACCTTCAATAATATGATCATTAGGATTTGTCTTTCTATTTACCGTAGGAGGATT   2418
m141102_iid=1228825_0      TTATTCACCTTCAATAATATGATCATTAGGATTTGTCTTTCTATTTACCGTAGGAGGATT   2245
m150504_iid=2876274_0      TTATTCACCTTCAATAATATGATCATTAGGATTTGTCTTTCTATTTACCGTAGGAGGATT   2248
m141030_iid=80036_0        TTATTCACCTTCAATAATATGATCATTAGGATTTGTCTTTCTATTTACCGTAGGAGGATT   2013
m150506_iid=2596494_0      TTATTCACCTTCAATAATATGATCATTAGGATTTGTCTTTCTATTTACCGTAGGAGGATT   2413
m150503_iid=2526862_0      TTATTCACCTTCAATAATATGATCATTAGGATTTGTCTTTCTATTTACCGTAGGAGGATT   2097
m150506_iid=2567000_0      -----------CAATAATATGATCATTAGGATTTGTCTTTCTATTTACCGTAGGAGGATT   49
m150507_iid=3073271_0      TTATTCACCTTCAATAATATGATCATTAGGATTTGTCTTTCTATTTACCGTAGGAGGATT   1350
m150504_iid=3156087_0      TTATTCACCTTCAATAATATGATCATTAGGATTTGTCTTTCTATTTACCGTAGGAGGATT   1173
m150505_iid=2498502_0      ------------------------------------------------------------   0
m141102_iid=754227_0       TTATTCACCTTCAATAATATGATCATTAGGATTTGTCTTTCTATTTACCGTAGGAGGATT   1385
m150507_iid=3469514_0      TTATTCACCTTCAATAATATGATCATTAGGATTTGTCTTTCTATTTACCGTAGGAGGATT   1943
Rdo_MitoGenome             TTATTCACCTTCAATAATATGATCATTAGGATTTGTCTTTCTATTTACCGTAGGAGGATT   2418
m141101_iid=614933_0       TTATTCACCTTCAATAATATGATCATTAGGATTTGTCTTTCTATTTACCGTAGGAGGATT   970
```

cox1

```
m141031_iid=347546_0       ------------------------------------------------------------   0
m150506_iid=3127414_0      ------------------------------------------------------------   0
m141102_iid=1245517_0      AACAGGAGTAGTATTAGCAAATTCATCCATTGATATTATTCTACACGATACATATTATGT   618
```

```
m141107_iid=1405727_0      ------------------------------------------------------------   0
m141102_iid=987614_0       AACAGGAGTAGTATTAGCAAATTCATCCATTGATATTATTCTACACGATACATATTATGT   747
m141108_iid=1701429_0      ------------------------------------------------------------   0
m141102_iid=987286_0       ------------------------------------------------------------   0
m150430_iid=3272125_0      AACAGGAGTAGTATTAGCAAATTCATCCATTGATATTATTCTACACGATACATATTATGT   226
m141102_iid=754225_0       AACAGGAGTAGTATTAGCAAATTCATCCATTGATATTATTCTACACGATACATATTATGT   1395
m141107_iid=1409934_0      AACAGGAGTAGTATTAGCAAATTCATCCATTGATATTATTCTACACGATACATATTATGT   1925
m150503_iid=3416920_0      AACAGGAGTAGTATTAGCAAATTCATCCATTGATATTATTCTACACGATACATATTATGT   661
m141108_iid=1800121_0      AACAGGAGTAGTATTAGCAAT--TATCCATTGATATATTCTAACACGATACATATTATGT   474
m141031_iid=147540_0       AACAGGAGTAGTATTAGCAAATTCATCCATTGATATTATTCTACACGATACATATTATGT   811
m150507_iid=3674924_0      AACAGGAGTAGTATTAGCAAATTCATCCATTGATATTATTCTACACGATACATATTATGT   957
m150503_iid=2923751_0      AACAGGAGTAGTATTAGCAAATTCATCCATTGATATTATTCTACACGATACATATTATGT   809
m141031_iid=115118_0       ------------------------------------------------------------   0
m150430_iid=2722448_0      ------------------------------------------------------------   0
m150507_iid=3102647_0      ------------------------------------------------------------   0
m141108_iid=1811866_0      ------------------------------------------------------------   0
m150501_iid=2948495_0      ------------------------------------------------------------   0
m150504_iid=3238195_0      AACAGGAGTAGTATTAGCAAATTCATCCATTGATATTATTCTACACGATACATATTATGT   1531
m150503_iid=3388511_0      ------------------------------------------------------------   0
m150505_iid=3777611_0      AACAGGAGTAGTATTAGCAAATTCATCCATTGATATTATTCTACACGATACATATTATGT   646
m150430_iid=2438833_0      AACAGGAGTAGTATTAGCAAATTCATCCATTGATATTATTCTACACGATACATATTATGT   996
m141108_iid=1698969_0      AACAGGAGTAGTATTAGCAAATTCATCCATTGATATTATTCTACACGATACATATTATGT   2119
m150430_iid=3059190_0      ------------------------------------------------------------   0
m141107_iid=1383067_0      ------------------------------------------------------------   0
m141101_iid=597008_0       ------------------------------------------------------------   0
m150506_iid=2596496_0      AACAGGAGTAGTATTAGCAAATTCATCCATTGATATTATTCTACACGATACATATTATGT   2471
m141102_iid=910051_0       AACAGGAGTAGTATTAGCAAATTCATCCATTGATATTATTCTACACGATACATATTATGT   1961
m150505_iid=3784017_0      AACAGGAGTAGTATTAGCAAATTCATCCATTGATATTATTCTACACGATACATATTATGT   760
m141101_iid=682151_0       ------------------------------------------------------------   0
m150501_iid=3748029_0      AACAGGAGTAGTATTAGCAAATTCATCCATTGATATTATTCTACACGATACATATTATGT   374
m150504_iid=2876272_0      AACAGGAGTAGTATTAGCAAATTCATCCATTGATATTATTCTACACGATACATATTATGT   2235
NC_042820.1                AACAGGAGTAGTATTAGCAAATTCATCCATTGATATTATTCTACACGATACATATTATGT   2478
m141102_iid=1228825_0      AACAGGAGTAGTATTAGCAAATTCATCCATTGATATTATTCTACACGATACATATTATGT   2305
m150504_iid=2876274_0      AACAGGAGTAGTATTAGCAAATTCATCCATTGATATTATTCTACACGATACATATTATGT   2308
m141030_iid=80036_0        AACAGGAGTAGTATTAGCAAATTCATCCATTGATATTATTCTACACGATACATATTATGT   2073
m150506_iid=2596494_0      AACAGGAGTAGTATTAGCAAATTCATCCATTGATATTATTCTACACGATACATATTATGT   2473
m150503_iid=2526862_0      AACAGGAGTAGTATTAGCAAATTCATCCATTGATATTATTCTACACGATACATATTATGT   2157
m150506_iid=2567000_0      AACAGGAGTAGTATTAGCAAATTCATCCATTGATATTATTCTACACGATACATATTATGT   109
m150507_iid=3073271_0      AACAGGAGTAGTATTAGCAAATTCATCCATTGATATTATTCTACACGATACATATTATGT   1410
m150504_iid=3156087_0      AACAGGAGTAGTATTAGCAAATTCATCCATTGATATTATTCTACACGATACATATTATGT   1233
m150505_iid=2498502_0      ------------------------------------------------------------   0
m141102_iid=754227_0       AACAGGAGTAGTATTAGCAAATTCATCCATTGATATTATTCTACACGATACATATTATGT   1445
m150507_iid=3469514_0      AACAGGAGTAGTATTAGCAAATTCATCCATTGATATTATTCTACACGATACATATTATGT   2003
Rdo_MitoGenome             AACAGGAGTAGTATTAGCAAATTCATCCATTGATATTATTCTACACGATACATATTATGT   2478
m141101_iid=614933_0       AACAGGAGTAGTATTAGCAAATTCATCCATTGATATTATTCTACACGATACATATTATGT   1030
```

cox1

```
m141031_iid=347546_0       ------------------------------------------------------------   0
m150506_iid=3127414_0      ------------------------------------------------------------   0
m141102_iid=1245517_0      AGTAGCCCACTTCCACTACGTGCTATCCATA-GAGCAGTATTTGCAATTATAGCAGGAAT   677
m141107_iid=1405727_0      ------------------------------------------------------------   0
m141102_iid=987614_0       AGTAGCCCACTTCCACTACGTGCTATCCATAGGAGCAGTATTTGCAATTATAGCAGGAAT   807
m141108_iid=1701429_0      ------------------------------------------------------------   0
m141102_iid=987286_0       ------------------------------------------------------------   0
m150430_iid=3272125_0      AGTAGCCCACTTCCACTACGTGCTATCCATAGGAGCAT-ATTTGCAATTATAGCAGGAAT   285
m141102_iid=754225_0       AGTAGCCCACTTCCACTACGTGCTATCCATAGGAGCAGTATTTGCAATTATAGCAGGAAT   1455
m141107_iid=1409934_0      AGTAGCCCACTTCCACTACGTGCTATCCATAGGAGCAGTATTTGCAATTATAGCAGGAAT   1985
m150503_iid=3416920_0      AGTAGCCCACTTCCACTACGTGCTATCCATAGGAGCAGTATTTGCAATTATAGCAGGAAT   721
m141108_iid=1800121_0      AGTAGC-CACTTCCACTACGTGCTATCCATAGGAGCAGTATTTGCAATTATAGCAGGAAT   533
m141031_iid=147540_0       AGTAGCCCACTTCCACTACGTGCTATCCATAGGAGCAGTATTTGCAATTATAGCAGGAAT   871
m150507_iid=3674924_0      AGTAGCCCACTTCCACTACGTGCTATCCATAGGAGCAGTATTTGCAATTATAGCAGGAAT   1017
m150503_iid=2923751_0      AGTAGCCCACTTCCACTACGTGCTATCCATAGGAGCAGTATTTGCAATTATAGCAGGAAT   869
m141031_iid=115118_0       ------------------------------------------------------------   0
m150430_iid=2722448_0      ------------------------------------------------------------   0
m150507_iid=3102647_0      ------------------------------------------------------------   0
m141108_iid=1811866_0      ------------------------------------------------------------   0
m150501_iid=2948495_0      ------------------------------------------------------------   0
m150504_iid=3238195_0      AGTAGCCCACTTCCACTACGTGCTATCCATAGGAGCAGTATTTGCAATTATAGCAGGAAT   1591
m150503_iid=3388511_0      ------------------------------------------------------------   0
m150505_iid=3777611_0      AGTAGCCCACTTCCACTACGTGCTATCCATAGGAGCAGTATTTGCAATTATAGCAGGAAT   706
m150430_iid=2438833_0      AGTAGCCCACTTCCACTACGTGCTATCCATAGGAGCAGTATTTGCAATTATAGCAGGAAT   1056
```

```
m141108_iid=1698969_0      AGTAGCCCACTTCCACTACGTGCTATCCATAGGAGCAGTATTTGCAATTATAGCAGGAAT   2179
m150430_iid=3059190_0      ------------------------------------------------------------   0
m141107_iid=1383067_0      ------------------------------------------------------------   0
m141101_iid=597008_0       ------------------------------------------------------------   0
m150506_iid=2596496_0      AGTAGCCCACTTCCACTACGTGCTATCCATAGGAGCAGTATTTGCAATTATAGCAGGAAT   2531
m141102_iid=910051_0       AGTAGCCCACTTCCACTACGTGCTATCCATAGGAGCAGTATTTGCAATTATAGCAGGAAT   2021
m150505_iid=3784017_0      AGTAGCCCACTTCCACTACGTGCTATCCATAGGAGCAGTATTTGCAATTATAGCAGGAAT   820
m141101_iid=682151_0       ------------------------------------------------------------   0
m150501_iid=3748029_0      AGTAGCCCACTTCCACTACGTGCTATCCATAGGAGCAGTATTTGCAATTATAGCAGGAAT   434
m150504_iid=2876272_0      AGTAGCCCACTTCCACTACGTGCTATCCATAGGAGCAGTATTTGCAATTATAGCAGGAAT   2295
NC_042820.1                AGTAGCCCACTTCCACTACGTGCTATCCATAGGAGCAGTATTTGCAATTATAGCAGGAAT   2538
m141102_iid=1228825_0      AGTAGCCCACTTCCACTACGTGCTATCCATAGGAGCAGTATTTGCAATTATAGCAGGAAT   2365
m150504_iid=2876274_0      AGTAGCCCACTTCCACTACGTGCTATCCATAGGAGCAGTATTTGCAATTATAGCAGGAAT   2368
m141030_iid=80036_0        AGTAGCCCACTTCCACTACGTGCTATCCATAGGAGCAGTATTTGCAATTATAGCAGGAAT   2133
m150506_iid=2596494_0      AGTAGCCCACTTCCACTACGTGCTATCCATAGGAGCAGTATTTGCAATTATAGCAGGAAT   2533
m150503_iid=2526862_0      AGTAGCCCACTTCCACTACGTGCTATCCATAGGAGCAGTATTTGCAATTATAGCAGGAAT   2217
m150506_iid=2567000_0      AGTAGCCCACTTCCACTACGTGCTATCCATAGGAGCAGTATTTGCAATTATAGCAGGAAT   169
m150507_iid=3073271_0      AGTAGCCCACTTCCACTACGTGCTATCCATAGGAGCAGTATTTGCAATTATAGCAGGAAT   1470
m150504_iid=3156087_0      AGTAGCCCACTTCCACTACGTGCTATCCATAGGAGCAGTATTTGCAATTATAGCAGGAAT   1293
m150505_iid=2498502_0      ------------------------------------------------------------   0
m141102_iid=754227_0       AGTAGCCCACTTCCACTACGTGCTATCCATAGGAGCAGTATTTGCAATTATAGCAGGAAT   1505
m150507_iid=3469514_0      AGTAGCCCACTTCCACTACGTGCTATCCATAGGAGCAGTATTTGCAATTATAGCAGGAAT   2063
Rdo_MitoGenome             AGTAGCCCACTTCCACTACGTGCTATCCATAGGAGCAGTATTTGCAATTATAGCAGGAAT   2538
m141101_iid=614933_0       AGTAGCCCACTTCCACTACGTGCTATCCATAGGAGCAGTATTTGCAATTATAGCAGGAAT   1090
```

cox1

```
m141031_iid=347546_0       ------------------------------------------------------------   0
m150506_iid=3127414_0      ------------------------------------------------------------   0
m141102_iid=1245517_0      TATTCAATGATTCCCACTATTTACAGGATTAACACTTAATGAAA-AATACTTAAAATCCA   736
m141107_iid=1405727_0      ------------------------------------------------------------   0
m141102_iid=987614_0       TATTCAATGATTCCCACTATTTACAGGATTAACACTTAATGAAAAAATACTTAAAATCCA   867
m141108_iid=1701429_0      ------------------------------------------------------------   0
m141102_iid=987286_0       ------------------------------------------------------------   0
m150430_iid=3272125_0      TATTCAATGATTCCCACTATTTACAGGATTAACACTTAATGAAAAAATACTTAAAATCCA   345
m141102_iid=754225_0       TATTCAATGATTCCCACTATTTACAGGATTAACACTTAATGAAAAAATACTTAAAATCCA   1515
m141107_iid=1409934_0      TATTCAATGATTCCCACTATTTACAGGATTAACACTTAATGAAAAAATACTTAAAATCCA   2045
m150503_iid=3416920_0      TATTCAATGATTCCCACTATTTACAGGATTAACACTTAATGAAAAAATACTTAAAATCCA   781
m141108_iid=1800121_0      TATTCAATGATTC-CACTATTTACAGGATTAACACTTAATGAAAAAATACTTA-AATCCA   591
m141031_iid=147540_0       TATTCAATGATTCCCACTATTTACAGGATTAACACTTAATGAAAAAATACTTAAAATCCA   931
m150507_iid=3674924_0      TATTCAATGATTTCCACTATTTACAGGATTAACACTTAATGAAAAAATACTTAAAATCCA   1077
m150503_iid=2923751_0      TATTCAATGATTCCCACTATTTACAGGATTAACACTTAATGAAAAAATACTTAAAATCCA   929
m141031_iid=115118_0       ------------------------------------------------------------   0
m150430_iid=2722448_0      ------------------------------------------------------------   0
m150507_iid=3102647_0      ------------------------------------------------------------   0
m141108_iid=1811866_0      ------------------------------------------------------------   0
m150501_iid=2948495_0      ------------------------------------------------------------   0
m150504_iid=3238195_0      TATTCAATGATTCCCACTATTTACAGGATTAACACTTAATGAAAAAATACTTAAAATCCA   1651
m150503_iid=3388511_0      ------------------------------------------------------------   0
m150505_iid=3777611_0      TATTCAATGATTCCCACTATTTACAGGATTAACACTTAATGAAAAAATACTTAAAATCCA   766
m150430_iid=2438833_0      TATTCAATGATTCCCACTATTTACAGGATTAACACTTAATGAAAAAATACTTAAAATCCA   1116
m141108_iid=1698969_0      TATTCAATGATTCCCACTATTTACAGGATTAACACTTAATGAAAAAATACTTAAAATCCA   2239
m150430_iid=3059190_0      ------------------------------------------------------------   0
m141107_iid=1383067_0      ------------------------------------------------------------   0
m141101_iid=597008_0       ------------------------------------------------------------   0
m150506_iid=2596496_0      TATTCAATGATTCCCACTATTTACAGGATTAACACTTAATGAAAAAATACTTAAAATCCA   2591
m141102_iid=910051_0       TATTCAATGATTCCCACTATTTACAGGATTAACACTTAATGAAAAAATACTTAAAATCCA   2081
m150505_iid=3784017_0      TATTCAATGATTCCCACTATTTACAGGATTAACACTTAATGAAAAAATACTTAAAATCCA   880
m141101_iid=682151_0       ------------------------------------------------------------   0
m150501_iid=3748029_0      TATTCAATGATTCCCACTATTTACAGGATTAACACTTAATGAAAAAATACTTAAAATCCA   494
m150504_iid=2876272_0      TATTCAATGATTCCCACTATTTACAGGATTAACACTTAATGAAAAAATACTTAAAATCCA   2355
NC_042820.1                TATTCAATGATTCCCACTATTTACAGGATTAACACTTAATGAAAAAATACTTAAAATCCA   2598
m141102_iid=1228825_0      TATTCAATGATTCCCACTATTTACAGGATTAACACTTAATGAAAAAATACTTAAAATCCA   2425
m150504_iid=2876274_0      TATTCAATGATTCCCACTATTTACAGGATTAACACTTAATGAAAAAATACTTAAAATCCA   2428
m141030_iid=80036_0        TATTCAATGATTCCCACTATTTACAGGATTAACACTTAATGAAAAAATACTTAAAATCCA   2193
m150506_iid=2596494_0      TATTCAATGATTCCCACTATTTACAGGATTAACACTTAATGAAAAAATACTTAAAATCCA   2593
m150503_iid=2526862_0      TATTCAATGATTCCCACTATTTACAGGATTAACACTTAATGAAAAAATACTTAAAATCCA   2277
m150506_iid=2567000_0      TATTCAATGATTCCCACTATTTACAGGATTAACACTTAATGAAAAAATACTTAAAATCCA   229
m150507_iid=3073271_0      TATTCAATGATTCCCACTATTTACAGGATTAACACTTAATGAAAAAATACTTAAAATCCA   1530
m150504_iid=3156087_0      TATTCAATGATTCCCACTATTTACAGGATTAACACTTAATGAAAAAATACTTAAAATCCA   1353
m150505_iid=2498502_0      ------------------------------------------------------------   0
m141102_iid=754227_0       TATTCAATGATTCCCACTATTTACAGGATTAACACTTAATGAAAAAATACTTAAAATCCA   1565
```

```
m150507_iid=3469514_0      TATTCAATGATTCCCACTATTTACAGGATTAACACTTAATGAAAAAATACTTAAAATCCA   2123
Rdo_MitoGenome             TATTCAATGATTCCCACTATTTACAGGATTAACACTTAATGAAAAAATACTTAAAATCCA   2598
m141101_iid=614933_0       TATTCAATGATTCCCACTATTTACAGGATTAACACTTAATGAAAAAATACTTAAAATCCA   1150
```

cox1

```
m141031_iid=347546_0       ------------------------------------------------------------   0
m150506_iid=3127414_0      ------------------------------------------------------------   0
m141102_iid=1245517_0      ATTCCTTATTATATTTATAGGAGTAAATATTACATTCTTCCCTCAACATTTTTTAGGATT   796
m141107_iid=1405727_0      ------------------------------------------------------------   0
m141102_iid=987614_0       ATTCCTTATTATATTTATAGGAGTAAATATTACATTCTTCCCTCAACATTTTTTAGGATT   927
m141108_iid=1701429_0      ------------------------------------------------------------   0
m141102_iid=987286_0       ------------------------------------------------------------   0
m150430_iid=3272125_0      ATTCCTTATTATATTTATAGGAGTAAATATTACATTCTTCCCTCAACATTTTTTAGGATT   405
m141102_iid=754225_0       ATTCCTTATTATATTTATAGGAGTAAATATTACATTCTTCCCTCAACATTTTTTAGGATT   1575
m141107_iid=1409934_0      ATTCCTTATTATATTTATAGGAGTAAATATTACATTCTTCCCTCAACATTTTTTAGGATT   2105
m150503_iid=3416920_0      ATTCCTTATTATATTTATAGGAGTAAATATTACATTCTTCCCTCAACATTTTTTAGGATT   841
m141108_iid=1800121_0      ATTCCTTATTATATTTATAGGAGTAAATATTACATTCTTCCCTCAACATTTTTTAGGATT   651
m141031_iid=147540_0       ATTCCTTATTATATTTATAGGAGTAAATATTACATTCTTCCCTCAACATTTTTTAGGATT   991
m150507_iid=3674924_0      ATTCCTTATTATATTTATAGGAGTAAATATTACATTCTTCCCTCAACATTTTTTAGGATT   1137
m150503_iid=2923751_0      ATTCCTTATTATATTTATAGGAGTAAATATTACATTCTTCCCTCAACATTTTTTAGGATT   989
m141031_iid=115118_0       ------------------------------------------------------------   0
m150430_iid=2722448_0      ------------------------------------------------------------   0
m150507_iid=3102647_0      ------------------------------------------------------------   0
m141108_iid=1811866_0      ------------------------------------------------------------   0
m150501_iid=2948495_0      ------------------------------------------------------------   0
m150504_iid=3238195_0      ATTCCTTATTATATTTATAGGAGTAAATATTACATTCTTCCCTCAACATTTTTTAGGATT   1711
m150503_iid=3388511_0      ------------------------------------------------------------   0
m150505_iid=3777611_0      ATTCCTTATTATATTTATAGGAGTAAATATTACATTCTTCCCTCAACATTTTTTAGGATT   826
m150430_iid=2438833_0      ATTCCTTATTATATTTATAGGAGTAAATATTACATTCTTCCCTCAACATTTTTTAGGATT   1176
m141108_iid=1698969_0      ATTCCTTATTATATTTATAGGAGTAAATATTACATTCTTCCCTCAACATTTTTTAGGATT   2299
m150430_iid=3059190_0      ------------------------------------------------------------   0
m141107_iid=1383067_0      ------------------------------------------------------------   0
m141101_iid=597008_0       ------------------------------------------------------------   0
m150506_iid=2596496_0      ATTCCTTATTATATTTATAGGAGTAAATATTACATTCTTCCCTCAACATTTTTTAGGATT   2651
m141102_iid=910051_0       ATTCCTTATTATATTTATAGGAGTAAATATTACATTCTTCCCTCAACATTTTTTAGGATT   2141
m150505_iid=3784017_0      ATTCCTTATTATATTTATAGGAGTAAATATTACATTCTTCCCTCAACATTTTTTAGGATT   940
m141101_iid=682151_0       -------------------------------------------------TTTTTAGGATT   11
m150501_iid=3748029_0      ATTCCTTATTATATTTATAGGAGTAAATATTACATTCTTCCCTCAACATTTTTTAGGATT   554
m150504_iid=2876272_0      ATTCCTTATTATATTTATAGGAGTAAATATTACATTCTTCCCTCAACATTTTTTAGGATT   2415
NC_042820.1                ATTCCTTATTATATTTATAGGAGTAAATATTACATTCTTCCCTCAACATTTTTTAGGATT   2658
m141102_iid=1228825_0      ATTCCTTATTATATTTATAGGAGTAAATATTACATTCTTCCCTCAACATTTTTTAGGATT   2485
m150504_iid=2876274_0      ATTCCTTATTATATTTATAGGAGTAAATATTACATTCTTCCCTCAACATTTTTTAGGATT   2488
m141030_iid=80036_0        ATTCCTTATTATATTTATAGGAGTAAATATTACATTCTT-CCTCAACATTTTTTAGGATT   2252
m150506_iid=2596494_0      ATTCCTTATTATATTTATAGGAGTAAATATTACATTCTTCCCTCAACATTTTTTAGGATT   2653
m150503_iid=2526862_0      ATTCCTTATTATATTTATAGGAGTAAATATTACATTCTTCCCTCAACATTTTTTAGGATT   2337
m150506_iid=2567000_0      ATTCCTTATATATTTT--AGGAGTAAATATTACATTCTTCCCTCAACATTTTTTAGGATT   287
m150507_iid=3073271_0      ATTCCTTATTATATTTATAGGAGTAAATATTACATTCTTCCCTCAACATTTTTTAGGATT   1590
m150504_iid=3156087_0      ATTCCTTATTATATTTATAGGAGTAAATATTACATTCTTCCCTCAACATTTTTTAGGATT   1413
m150505_iid=2498502_0      ------------------------------------------------------------   0
m141102_iid=754227_0       ATTCCTTATTATATTTATAGGAGTAAATATTACATTCTTCCCTCAACATTTTTTAGGATT   1625
m150507_iid=3469514_0      ATTCCTTATTATATTTATAGGAGTAAATATTACATTCTTCCCTCAACATTTTTTAGGATT   2183
Rdo_MitoGenome             ATTCCTTATTATATTTATAGGAGTAAATATTACATTCTTCCCTCAACATTTTTTAGGATT   2658
m141101_iid=614933_0       ATTCCTTATTATATTTATAGGAGTAAATATTACATTCTTCCCTCAACATTTTTTAGGATT   1210
```

cox1

```
m141031_iid=347546_0       ------------------------------------------------------------   0
m150506_iid=3127414_0      ------------------------------------------------------------   0
m141102_iid=1245517_0      AAGAGGTATA---CCGACGATACTCAGATTATCCAGATGCATATCTCACATGAAATATTA   853
m141107_iid=1405727_0      ------------------------------------------------------------   0
m141102_iid=987614_0       AAGAGGTAT-ACCACGACGATACTCAGATTATCCAGATGCATATCTCACATGAAATATTA   986
m141108_iid=1701429_0      ------------------------------------------------------------   0
m141102_iid=987286_0       ------------------------------------------------------------   0
m150430_iid=3272125_0      AAGAGGTATAACCACGACGATACTCAGATTATCCAGATGCATATCTCACATGAAATATTA   465
m141102_iid=754225_0       AAGAGGTAT-ACCACGACGATACTCAGATTATCCAGATGCATATCTCACATGAAATATTA   1634
m141107_iid=1409934_0      AAGAGGTAT-ACCACGACGATACTCAGATTATCCAGATGCATATCTCACATGAAATATTA   2164
m150503_iid=3416920_0      AAGAGGTAT-ACCACGACGATACTCAGATTATCCAGATGCATATCTCACATGAAATATTA   900
m141108_iid=1800121_0      AAGAGGTATAACCACGACGATAACTAGAT-TATCAGATGCATATCTCACATGAAT-ATTA   709
m141031_iid=147540_0       AAGAGGTAT-ACCACGACGATACTCAGATTATCCAGATGCATATCTCACATGAAATATTA   1050
m150507_iid=3674924_0      AAGAGGTAT-ACCACGACGATACTCAGATTATCCAGATGCATATCTCACATGAAATATTA   1196
m150503_iid=2923751_0      AAGAGGTAT-ACCACGACGATACTCAGATTATCCAGATGCATATCTCACATGAAATATTA   1048
m141031_iid=115118_0       ------------------------------------------------------------   0
```

```
m150430_iid=2722448_0      ------------------------------------------------------------   0
m150507_iid=3102647_0      ------------------------------------------------------------   0
m141108_iid=1811866_0      --------------------------------CCAGATGCATATCTCACATGAAATATTA   28
m150501_iid=2948495_0      ------------------------------------------------------------   0
m150504_iid=3238195_0      AAGAGGTAT-ACCACGACGATACTCAGATTATCCAGATGCATATCTCACATGAAATATTA   1770
m150503_iid=3388511_0      ------------------------------------------------------------   0
m150505_iid=3777611_0      AAGAGGTAT-ACCACGACGATACTCAGATTATCCAGATGCATATCTCACATGAAATATTA   885
m150430_iid=2438833_0      AAGAGGTAT-ACCACGACGATACTCAGATTATCCAGATGCATATCTCACATGAAATATTA   1235
m141108_iid=1698969_0      AAGAGGTAT-ACCACGACGATACTCAGATTATCCAGATGCATATCTCACATGAAATATTA   2358
m150430_iid=3059190_0      ------------------------------------------------------------   0
m141107_iid=1383067_0      ------------------------------------------------------------   0
m141101_iid=597008_0       ------------------------------------------------------------   0
m150506_iid=2596496_0      AAGAGGTAT-ACCACGACGATACTCAGATTATCCAGATGCATATCTCACATGAAATATTA   2710
m141102_iid=910051_0       AAGAGGTAT-ACCACGACGATACTCAGATTATCCAGATGCATATCTCACATGAAATATTA   2200
m150505_iid=3784017_0      AAGAGGTAT-ACCACGACGATACTCAGATTATCCAGATGCATATCTCACATGAAATATTA   999
m141101_iid=682151_0       AAGAGGTAT-ACCACGACGATACTCAGATTATCCAGATGCATATCTCACATGAAATATTA   70
m150501_iid=3748029_0      AAGAGGTAT-ACCACGACGATACTCAGATTATCCAGATGCATATCTCACATGAAATATTA   613
m150504_iid=2876272_0      AAGAGGTAT-ACCACGACGATACTCAGATTATCCAGATGCATATCTCACATGAAATATTA   2474
NC_042820.1                AAGAGGTAT-ACCACGACGATACTCAGATTATCCAGATGCATATCTCACATGAAATATTA   2717
m141102_iid=1228825_0      AAGAGGTAT-ACCACGACGATACTCAGATTATCCAGATGCATATCTCACATGAAATATTA   2544
m150504_iid=2876274_0      AAGAGGTAT-ACCACGACGATACTCAGATTATCCAGATGCATATCTCACATGAAATATTA   2547
m141030_iid=80036_0        AAGAGGTAT-ACCACGACGATACTCAGATTATCCAGATGCATATCTCACATGAAATATTA   2311
m150506_iid=2596494_0      AAGAGGTAT-ACCACGACGATACTCAGATTATCCAGATGCATATCTCACATGAAATATTA   2712
m150503_iid=2526862_0      AAGAGGTAT-ACCACGACGATACTCAGATTATCCAGATGCATATCTCACATGAAATATTA   2396
m150506_iid=2567000_0      AAGAGGTAT-ACCACGACGATACTCAGATTATCCAGATGCATATCTCACATGAAATATTA   346
m150507_iid=3073271_0      AAGAGGTAT-ACCACGACGATACTCAGATTATCCAGATGCATATCTCACATGAAATATTA   1649
m150504_iid=3156087_0      AAGAGGTAT-ACCACGACGATACTCAGATTATCCAGATGCATATCTCACATGAAATATTA   1472
m150505_iid=2498502_0      ------------------------------------------------------------   0
m141102_iid=754227_0       AAGAGGTAT-ACCACGACGATACTCAGATTATCCAGATGCATATCTCACATGAAATATTA   1684
m150507_iid=3469514_0      AAGAGGTAT-ACCACGACGATACTCAGATTATCCAGATGCATATCTCACATGAAATATTA   2242
Rdo_MitoGenome             AAGAGGTAT-ACCACGACGATACTCAGATTATCCAGATGCATATCTCACATGAAATATTA   2717
m141101_iid=614933_0       AAGAGGTAT-ACCACGACGATACTCAGATTATCCAGATGCATATCTCACATGAAATATTA   1269
```

cox1

```
m141031_iid=347546_0       ------------------------------------------------------------   0
m150506_iid=3127414_0      ------------------------------------------------------------   0
m141102_iid=1245517_0      TTTCATCAATCGGATCAATAA--TGAACAGTAAGAATTATTTTTATGTCATTCATCATAT   911
m141107_iid=1405727_0      ------------------------------------------------------------   0
m141102_iid=987614_0       TTTCATCAATCGGATCAATAATAAGAACAGTAAGAATTATTTTTATGTCATTCATCATAT   1046
m141108_iid=1701429_0      ------------------------------------------------------------   0
m141102_iid=987286_0       ------------------------------------------------------------   0
m150430_iid=3272125_0      TTTCATCAATCGGATCAATAATAAGAACAGTAAGAATTATTTTTATGTCATTCATCATAT   525
m141102_iid=754225_0       TTTCATCAATCGGATCAATAATAAGAACAGTAAGAATTATTTTTATGTCATTCATCATAT   1694
m141107_iid=1409934_0      TTTCATCAATCGGATCAATAATAAGAACAGTAAGAATTATTTTTATGTCATTCATCATAT   2224
m150503_iid=3416920_0      TTTCATCAATCGGATCAATAATAAGAACAGTAAGAATTATTTTTTTGTCATTCATCATAT   960
m141108_iid=1800121_0      TTTCATCAATCGGATCAAATATAAGAACAGTA-GAATTATTTTTATGTCATTCATCATAT   768
m141031_iid=147540_0       TTTCATCAATCGGATCAATAATAAGAACAGTAAGAATTATTTTTATGTCATTCATCATAT   1110
m150507_iid=3674924_0      TTCATCATATCGGATCAATAATAAGAACAGTAAGAATTATTTTTATGTCATTCATCATAT   1256
m150503_iid=2923751_0      TTTCATCAATCGGATCAATAATAAGAACAGTAAGAATTATTTTTATGTCATTCATCATAT   1108
m141031_iid=115118_0       ------------------------------------------------------------   0
m150430_iid=2722448_0      ------------------------------------------------------------   0
m150507_iid=3102647_0      ------------------------------------------------------------   0
m141108_iid=1811866_0      TTTCATCAATCGGATCAATAATAAGAACAGTAAGAATTATTTTTATGTCATTCATCATAT   88
m150501_iid=2948495_0      ------------------------------------------------------------   0
m150504_iid=3238195_0      TTTCATCAATCGGATCAATAATAAGAACAGTAAGAATTATTTTTATGTCATTCATCATAT   1830
m150503_iid=3388511_0      ------------------------------------------------------------   0
m150505_iid=3777611_0      TTTCATCAATCGGATCAATAATAAGAACAGTAAGAATTATTTTTATGTCATTCATCATAT   945
m150430_iid=2438833_0      TTTCATCAATCGGATCAATAATAAGAACAGTAAGAATTATTTTTATGTCATTCATCATAT   1295
m141108_iid=1698969_0      TTTCATCAATCGGATCAATAATAAGAACAGTAAGAATTATTTTTATGTCATTCATCATAT   2418
m150430_iid=3059190_0      ------------------------------------------------------------   0
m141107_iid=1383067_0      ------------------------------------------------------------   0
m141101_iid=597008_0       ------------------------------------------------------------   0
m150506_iid=2596496_0      TTTCATCAATCGGATCAATAATAAGAACAGTAAGAATTATTTTTATGTCATTCATCATAT   2770
m141102_iid=910051_0       TTTCATCAATCGGATCAATAATAAGAACAGTAAGAATTATTTTTATGTCATTCATCATAT   2260
m150505_iid=3784017_0      TTTCATCAATCGGATCAATAATAAGAACAGTAAGAATTATTTTTATGTCATTCATCATAT   1059
m141101_iid=682151_0       TTTCATCAATCGGATCAATAATAAGAACAGTAAGAATTATTTTTATGTCATTCATCATAT   130
m150501_iid=3748029_0      TTTCATCAATCGGATCAATAATAAGAACAGTAAGAATTATTTTTATGTCATTCATCATAT   673
m150504_iid=2876272_0      TTTCATCAATCGGATCAATAATAAGAACAGTAAGAATTATTTTTATGTCATTCATCATAT   2534
NC_042820.1                TTTCATCAATCGGATCAATAATAAGAACAGTAAGAATTATTTTTATGTCATTCATCATAT   2777
m141102_iid=1228825_0      TTTCATCAATCGGATCAATAATAAGAACAGTAAGAATTATTTTTATGTCATTCATCATAT   2604
m150504_iid=2876274_0      TTTCATCAATCGGATCAATAATAAGAACAGTAAGAATTATTTTTATGTCATTCATCATAT   2607
```

```
m141030_iid=80036_0        TTTCATCAATCGGATCAATAATAAGAACAGTAAGAATTATTTTTATGTCATTCATCATAT   2371
m150506_iid=2596494_0      TTTCATCAATCGGATCAATAATAAGAACAGTAAGAATTATTTTTATGTCATTCATCATAT   2772
m150503_iid=2526862_0      TTTCATCAATCGGATCAATAATAAGAACAGTAAGAATTATTTTTATGTCATTCATCATAT   2456
m150506_iid=2567000_0      TTTCATCAATCGGATCAATAATAAGAACAGTAAGAATTATTTTTATGTCATTCATCATAT   406
m150507_iid=3073271_0      TTTCATCAATCGGATCAATAATAAGAACAGTAAGAATTATTTTTATGTCATTCATCATAT   1709
m150504_iid=3156087_0      TTTCATCAATCGGATCAATAATAAGAACAGTAAGAATTATTTTTATGTCATTCATCATAT   1532
m150505_iid=2498502_0      ------------------------------------------------------------   0
m141102_iid=754227_0       TTTCATCAATCGGATCAATAATAAGAACAGTAAGAATTATTTTTATGTCATTCATCATAT   1744
m150507_iid=3469514_0      TTTCATCAATCGGATCAATAATAAGAACAGTAAGAATTATTTTTATGTCATTCATCATAT   2302
Rdo_MitoGenome             TTTCATCAATCGGATCAATAATAAGAACAGTAAGAATTATTTTTATGTCATTCATCATAT   2777
m141101_iid=614933_0       TTTCATCAATCGGATCAATAATAAGAACAGTAAGAATTATTTTTATGTCATTCATCATAT   1329
```

cox1

```
m141031_iid=347546_0       ------------------------------------------------------------   0
m150506_iid=3127414_0      ------------------------------------------------------------   0
m141102_iid=1245517_0      GAGAATCATT-ATCATCTAAACGAAAGAATATCAGAAATAACCAATTATCATCAGCAATT   970
m141107_iid=1405727_0      ------------------------------------------------------------   0
m141102_iid=987614_0       GAGAATCATT-ATCATCTAAACGAAAGAATATCAGAAATAACCAATTATCATCAGCAATT   1105
m141108_iid=1701429_0      ------------------------------------------------------------   0
m141102_iid=987286_0       ------------------------------------------------------------   0
m150430_iid=3272125_0      GAGAATCATT-ATCATCTAAACGAAAGA-TATCAGAAATAACCAATTATCATCAGCAATT   583
m141102_iid=754225_0       GAGAATCATT-ATCATCTAAACGAAAGAATATCAGAAATAACCAATTATCATCAGCAATT   1753
m141107_iid=1409934_0      GAGAATCATT-ATCATCTAAACGAAAGAATATCAGAAATAACCAATTATCATCAGCAATT   2283
m150503_iid=3416920_0      GAGAATCATT-ATCATCTAAACGAAAGAATATCAGAAATAACCAATTATCATCAGCAATT   1019
m141108_iid=1800121_0      GAGAATCATT-ATCATCTAAACGAAAGAATATCAGAAATAACCAATTATCATCAGCAATT   827
m141031_iid=147540_0       GAGAATCATT-ATCATCTAAACGAAAGAATATCAGAAATAACCAATTATCATCAGCAATT   1169
m150507_iid=3674924_0      GAGAATCATTAATCATCTAAACGAAAGAATATCAGAAATAACCAATTATCATCAGCAATT   1316
m150503_iid=2923751_0      GAGAATCATT-ATCATCTAAACGAAAGAATATCAGAAATAACCAATTATCATCAGCAATT   1167
m141031_iid=115118_0       --------------------------GAATATCAGAAATAACCAATTATCATCAACAATT   34
m150430_iid=2722448_0      ------------------------------------------------------------   0
m150507_iid=3102647_0      ------------------------------------------------------------   0
m141108_iid=1811866_0      GAGAATCATT-ATCATCTAAACGAAAGAATATCAGAA-TAACCAATTATCATCAGCAATT   146
m150501_iid=2948495_0      ------------------------------------------------------------   0
m150504_iid=3238195_0      GAGAATCATT-ATCATCTAAACGAAAGAATATCAGAAATAACCAATTATCATCAGCAATT   1889
m150503_iid=3388511_0      ------------------------------------------------------------   0
m150505_iid=3777611_0      GAGAATCATT-ATCATCTAAACGAAAGAATATCAGAAATAACCAATTATCATCAGCAATT   1004
m150430_iid=2438833_0      GAGAATCATT-ATCATCTAAACGAAAGAATATCAGAAATAACCAATTATCATCAGCAATT   1354
m141108_iid=1698969_0      GAGAATCATT-ATCATCTAAACGAAAGAATATCAGAAATAACCAATTATCATCAGCAATT   2477
m150430_iid=3059190_0      ------------------------------------------------------------   0
m141107_iid=1383067_0      ------------------------------------------------------------   0
m141101_iid=597008_0       ------------------------------------------------------------   0
m150506_iid=2596496_0      GAGAATCATT-ATCATCTAAACGAAAGAATATCAGAAATAACCAATTATCATCAGCAATT   2829
m141102_iid=910051_0       GAGAATCATT-ATCATCTAAACGAAAGAATATCAGAAATAACCAATTATCATCAGCAATT   2319
m150505_iid=3784017_0      GAGAATCATT-ATCATCTAAACGAAAGAATATCAGAAATAACCAATTATCATCAGCAATT   1118
m141101_iid=682151_0       GAGAATCATT-ATCATCTAAACGAAAGAATATCAGAAATAACCAATTATCATCAGCAATT   189
m150501_iid=3748029_0      GAGAATCATT-ATCATCTAAACGAAAGAATATCAGAAATAACCAATTATCATCAGCAATT   732
m150504_iid=2876272_0      GAGAATCATT-ATCATCTAAACGAAAGAATATCAGAAATAACCAATTATCATCAGCAATT   2593
NC_042820.1                GAGAATCATT-ATCATCTAAACGAAAGAATATCAGAAATAACCAATTATCATCAGCAATT   2836
m141102_iid=1228825_0      GAGAATCATT-ATCATCTAAACGAAAGAATATCAGAAATAACCAATTATCATCAGCAATT   2663
m150504_iid=2876274_0      GAGAATCATT-ATCATCTAAACGAAAGAATATCAGAAATAACCAATTATCATCAGCAATT   2666
m141030_iid=80036_0        GAGAATCATT-ATCATCTAAACGAAAGAATATCAGAAATAACCAATTATCATCAGCAATT   2430
m150506_iid=2596494_0      GAGAATCATT-ATCATCTAAACGAAAGAATATCAGAAATAACCAATTATCATCAGCAATT   2831
m150503_iid=2526862_0      GAGAATCATT-ATCATCTAAACGAAAGAATATCAGAAATAACCAATTATCATCAGCAATT   2515
m150506_iid=2567000_0      GAGAATCATT-ATCATCTAAACGAAAGAATATCAGAAATAACCAATTATCATCAGCAATT   465
m150507_iid=3073271_0      GAGAATCATT-ATCATCTAAACGAAAGAATATCAGAAATAACCAATTATCATCAGCAATT   1768
m150504_iid=3156087_0      GAGAATCATT-ATCATCTAAACGAAAGAATATCAGAAATAACCAATTATCATCAGCAATT   1591
m150505_iid=2498502_0      ------------------------------------------------------------   0
m141102_iid=754227_0       GAGAATCATT-ATCATCTAAACGAAAGAATATCAGAAATAACCAATTATCATCAGCAATT   1803
m150507_iid=3469514_0      GAGAATCATT-ATCATCTAAACGAAAGAATATCAGAAATAACCAATTATCATCAGCAATT   2361
Rdo_MitoGenome             GAGAATCATT-ATCATCTAAACGAAAGAATATCAGAAATAACCAATTATCATCAGCAATT   2836
m141101_iid=614933_0       GAGAATCATT-ATCATCTAAACGAAAGAATATCAGAAATAACCAATTATCATCAGCAATT   1388
```

cox1

```
m141031_iid=347546_0       ------------------------------------------------------------   0
m150506_iid=3127414_0      ------------------------------------------------------------   0
m141102_iid=1245517_0      GAATGACTTCAAAATTCACC-CCAGAAGAACACAGATACTCAGAACTTCCTATCCTAGCT   1029
m141107_iid=1405727_0      ------------------------------------------------------------   0
m141102_iid=987614_0       GAATGACTTCAAAATTCACCCCCAGAAGAACACAGATACTCAGAACTTCCTATCCTAGCT   1165
m141108_iid=1701429_0      ------------------------------------------------------------   0
m141102_iid=987286_0       ------------------------------------------------------------   0
m150430_iid=3272125_0      GAATGACTTCAAAATTCACC-CCAGAAGAACACAGATACTCAGAACTTCCTATCCTAGCT   642
```

```
m141102_iid=754225_0       GAATGACTTCAAAATTCACCCCCAGAAGAACACAGATACTCAGAACTTCCTATCCTAGCT   1813
m141107_iid=1409934_0      GAATGACTTCAAAATTCACCCCCAGAAGAACACAGATACTCAGAACTTCCTATCCTAGCT   2343
m150503_iid=3416920_0      GAATGACTTCAAAATTCACCCCCAGAAGAACACAGATACTCAGAACTTCCTATCCTAGCT   1079
m141108_iid=1800121_0      GAATGACTTCAAATTCACCCCCAGAAGAACACAGATACTCAGAACTTCCTATCCTAGCT   887
m141031_iid=147540_0       GAATGACTTCAAAATTCACCCCCAGAAGAACACAGATACTCAGAACTTCCTATCCTAGCT   1229
m150507_iid=3674924_0      GAATGACTTCAAAATTCACCCCCAGAAGAACACAGATACTCAGAACTTCCTATCCTAGCT   1376
m150503_iid=2923751_0      GAATGACTTCAAAATTCACCCCCAGAAGAACACAGATACTCAGAACTTCCTATCCTAGCT   1227
m141031_iid=115118_0       GAATGACTTCAAAATTCACCCCCAGAAGAACACAGATACTCAGAACTTCCTATCCTAGCT   94
m150430_iid=2722448_0      ------------------------------------------GAACTTCCTATCCTAGCT   18
m150507_iid=3102647_0      ------------------------------------------------------------   0
m141108_iid=1811866_0      GAATGACTTCAAAATTCACCCCCAGAAGAACACAGATACTCAGAACTTCCTATCCTAGCT   206
m150501_iid=2948495_0      ------------------------------------------------------------   0
m150504_iid=3238195_0      GAATGACTTCAAAATTCACCCCCAGAAGAACACAGATACTCAGAACTTCCTATCCTAGCT   1949
m150503_iid=3388511_0      ------------------------------------------------------------   0
m150505_iid=3777611_0      GAATGACTTCAAAATTCACCCCCAGAAGAACACAGATACTCAGAACTTCCTATCCTAGCT   1064
m150430_iid=2438833_0      GAATGACTTCAAAATTCACCCCCAGAAGAACACAGATACTCAGAACTTCCTATCCTAGCT   1414
m141108_iid=1698969_0      GAATGACTTCAAAATTCACCCCCAGAAGAACACAGATACTCAGAACTTCCTATCCTAGCT   2537
m150430_iid=3059190_0      ------------------------------------------------------------   0
m141107_iid=1383067_0      ------------------------------------------------------------   0
m141101_iid=597008_0       ------------------------------------------------------------   0
m150506_iid=2596496_0      GAATGACTTCAAAATTCACCCCCAGAAGAACACAGATACTCAGAACTTCCTATCCTAGCT   2889
m141102_iid=910051_0       GAATGACTTCAAAATTCACCCCCAGAAGAACACAGATACTCAGAACTTCCTATCCTAGCT   2379
m150505_iid=3784017_0      GAATGACTTCAAAATTCACCCCCAGAAGAACACAGATACTCAGAACTTCCTATCCTAGCT   1178
m141101_iid=682151_0       GAATGACTTCAAAATTCACCCCCAGAAGAACACAGATACTCAGAACTT-CTATCCTAGCT   248
m150501_iid=3748029_0      GAATGACTTCAAAATTCA-CCCCAGAAGAACACAGATACTCAGAACTTCCTATCCTAGCT   791
m150504_iid=2876272_0      GAATGACTTCAAAATTCACCCCCAGAAGAACACAGATACTCAGAACTTCCTATCCTAGCT   2653
NC_042820.1                GAATGACTTCAAAATTCACCCCCAGCAGAACACAGATACTCAGAACTTCCTATCCTAGCT   2896
m141102_iid=1228825_0      GAATGACTTCAAAATTCACCCCCAGAAGAACACAGATACTCAGAACTTCCTATCCTAGCT   2723
m150504_iid=2876274_0      GAATGACTTCAAAATTCACCCCCAGAAGAACACAGATACTCAGAACTTCCTATCCTAGCT   2726
m141030_iid=80036_0        GAATGACTTCAAAATTCACCCCCAGAAGAACACAGATACTCAGAACTTCCTATCCTAGCT   2490
m150506_iid=2596494_0      GAATGACTTCAAAATTCACCCCCAGAAGAACACAGATACTCAGAACTTCCTATCCTAGCT   2891
m150503_iid=2526862_0      GAATGACTTCAAAATTCACCCCCAGAAGAACACAGATACTCAGAACTTCCTATCCTAGCT   2575
m150506_iid=2567000_0      GAATGACTTCAAAATTCA-CCCCAGAAGAACACAGATACTCAGAACTTCCTATCCTAGCT   524
m150507_iid=3073271_0      GAATGACTTCAAAATTCACCCCCAGAAGAACACAGATACTCAGAACTTCCTATCCTAGCT   1828
m150504_iid=3156087_0      GAATGACTTCAAAATTCACCCCCAGAAGAACACAGATACTCAGAACTTCCTATCCTAGCT   1651
m150505_iid=2498502_0      ------------------------------------------------------------   0
m141102_iid=754227_0       GAATGACTTCAAAATTCACCCCCAGAAGAACACAGATACTCAGAACTTCCTATCCTAGCT   1863
m150507_iid=3469514_0      GAATGACTTCAAAATTCACCCCCAGAAGAACACAGATACTCAGAACTTCCTATCCTAGCT   2421
Rdo_MitoGenome             GAATGACTTCAAAATTCACCCCCAGAAGAACACAGATACTCAGAACTTCCTATCCTAGCT   2896
m141101_iid=614933_0       GAATGACTTCAAAATTCACCCCCAGAAGAACACAGATACTCAGAACTTCCTATCCTAGCT   1448
```

cox1

```
m141031_iid=347546_0       ------------------------------------------------------------   0
m150506_iid=3127414_0      ------------------------------------------------------------   0
m141102_iid=1245517_0      AAA-TTCTAATATGGCAGAATAGTGCAATGAATTTAAGATTCATATATA---AGTTAAAC   1085
m141107_iid=1405727_0      ------------------------------------------------------------   0
m141102_iid=987614_0       AAA-TTCTAATATGGCAGAATAGTGCAATGAATTTAAGATTCATATA--TAAGTT-AAAC   1221
m141108_iid=1701429_0      ------------------------------------------------------------   0
m141102_iid=987286_0       ------------------------------------------------------------   0
m150430_iid=3272125_0      AAA-TTCTAATATGGCAGAATAGTGCAATGAATTTAAGATTCATATAAA---GTTAAACT   698
m141102_iid=754225_0       AAA-TTCTAATATGGCAGAATAGTGCAATGAATTTAAGATTCATATATA---AGTTAAAC   1869
m141107_iid=1409934_0      AAA-TTCTAATATGGCAGAATAGTGCAATGAATTTAAGATTCATATATAA---GTTAAAC   2399
m150503_iid=3416920_0      AAA-TTCTAATATGGCAGAATAGTGCAATGAATTTAAGATTCATATATA--AGT-TAAAC   1135
m141108_iid=1800121_0      AAA-TTCTAATATGGCAGAATAGTGCAATGATTT-AAGATTCATATA--TAAGTTAAACT   943
m141031_iid=147540_0       AAA-TTCTAATATGGCAGAATAGTGCAATGAATTTAAGATTCATATATAA---GTTAAAC   1285
m150507_iid=3674924_0      AAA-TTCTAATATGGCAGAATAGTGCAATGAATTTAAGATTCATATATAA---GTTAAAC   1432
m150503_iid=2923751_0      AAA-TTCTAATATGGCAGAATAGTGCAATGAATTTAAGATTCATATATA--AGT-TAAAC   1283
m141031_iid=115118_0       AAA-TTCTAATATGGCAGAATAGTGCAATGAATTTAAGATTCATAATTATAAGTTAAACT   153
m150430_iid=2722448_0      AAATTTCTAATATGGCAGAATAGTGCAATGAATTTAAGATTCATATA--TAAGTTAAACT   76
m150507_iid=3102647_0      ------------------------------------------------------------   0
m141108_iid=1811866_0      AAA-TT-TAATATGGCAGAATAGTGCAATGAATTTAAGATTCATATA--TAAGTTTAAAC   262
m150501_iid=2948495_0      ------------------------------------------------------------   0
m150504_iid=3238195_0      AAA-TTCTAATATGGCAGAATAGTGCAATGAATTTAAGATTCATATA--TAAGTT-AAAC   2005
m150503_iid=3388511_0      ------------------------------------------------------------   0
m150505_iid=3777611_0      AAA-TTCTAATATGGCAGAATAGTGCAATGAATTTAAGATTCATATA--TAAGTT-AAAC   1120
m150430_iid=2438833_0      AAA-TTCTAATATGGCAGAATAGTGCAATGAATTTAAGATTCATATA--TAAGTT-AAAC   1470
m141108_iid=1698969_0      AAA-TTCTA----GGCAGAATAGTGCAATGAATTTAAGATTCATATA--TAAGTT-AAAC   2589
m150430_iid=3059190_0      ------------------------------------------------------------   0
m141107_iid=1383067_0      ------------------------------------------------------------   0
m141101_iid=597008_0       ------------------------------------------------------------   0
m150506_iid=2596496_0      AAA-TTCTAATATGGCAGAATAGTGCAATGAATTTAAGATTCATATA--TAAGTT-AAAC   2945
```

```
m141102_iid=910051_0       AAA-TTCTAATATGGCAGAATAGTGCAATGAATTTAAGATTCATATA--TAAGTT-AAAC   2435
m150505_iid=3784017_0      AAA-TTCTAATATGGCAGAATAGTGCAATGAATTTAAGATTCATATA--TAAGTT-AAAC   1234
m141101_iid=682151_0       AAA-TTCTAATATGGCAGAATAGTGCAATGAATTTAAGATTCATATA--TAAGTT-AAAC   304
m150501_iid=3748029_0      AAA-TTCTAATATGGCAGAATAGTGCAATGAATTTAAGATTCATATA--TAAGTT-AAAC   847
m150504_iid=2876272_0      AAA-TTCTAATATGGCAGAATAGTGCAATGAATTTAAGATTCATATA--TAAGTT-AAAC   2709
NC_042820.1                AAA-TTCTAATATGGCAGAATAGTGCAATGAATTTAAGATTCATATA--TAAGTT-AAAC   2952
m141102_iid=1228825_0      AAA-TTCTAATATGGCAGAATAGTGCAATGAATTTAAGATTCATATA--TAAGTT-AAAC   2779
m150504_iid=2876274_0      AAA-TTCTAATATGGCAGAATAGTGCAATGAATTTAAGATTCATATA--TAAGTT-AAAC   2782
m141030_iid=80036_0        AAA-TTCTAATATGGCAGAATAGTGCAATGAATTTAAGATTCATATA--TAAGTT-AAAC   2546
m150506_iid=2596494_0      AAA-TTCTAATATGGCAGAATAGTGCAATGAATTTAAGATTCATATA--TAAGTT-AAAC   2947
m150503_iid=2526862_0      AAA-TTCTAATATGGCAGAATAGTGCAATGAATTTAAGATTCATATA--TAAGTT-AAAC   2631
m150506_iid=2567000_0      AAA-TTCTAATATGGCAGAATAGTGCAATGAATTTAAGATTCATATA--TAAGTT-AAAC   580
m150507_iid=3073271_0      AAA-TTCTAATATGGCAGAATAGTGCAATGAATTTAAGATTCATATA--TAAGTT-AAAC   1884
m150504_iid=3156087_0      AAA-TTCTAATATGGCAGAATAGTGCAATGAATTTAAGATTCATATA--TAAGTT-AAAC   1707
m150505_iid=2498502_0      ------------------------------------------------------------   0
m141102_iid=754227_0       AAA-TTCTAATATGGCAGAATAGTGCAATGAATTTAAGATTCATATA--TAAGTT-AAAC   1919
m150507_iid=3469514_0      AAA-TTCTAATATGGCAGAATAGTGCAATGAATTTAAGATTCATATA--TAAGTT-AAAC   2477
Rdo_MitoGenome             AAA-TTCTAATATGGCAGAATAGTGCAATGAATTTAAGATTCATATA--TAAGTT-AAAC   2952
m141101_iid=614933_0       AAA-TTCTAATATGGCAGAATAGTGCAATGAATTTAAGATTCATATA--TAAGTT-AAAC   1504
```

cox1 →

```
m141031_iid=347546_0       ------------------------------------------------------------   0
m150506_iid=3127414_0      ------------------------------------------------------------   0
m141102_iid=1245517_0      TTTTTTAGAAATAGCAACATGAAAAATAACATCAA-TGAGAAATAGAAATTCCCCGGCAA   1144
m141107_iid=1405727_0      ------------------------------------------------------------   0
m141102_iid=987614_0       TTTTTTAGAAATAGCAACATGAAAAATAACATCAA-TGAGAAATAGAAATTCCCCGGCAA   1280
m141108_iid=1701429_0      ------------------------------------------------------------   0
m141102_iid=987286_0       ------------------------------------------------------------   0
m150430_iid=3272125_0      TTTTTTAGAAATAGCAACATGAAAAATAACATTCAATGAGAAATAGAAATTCCCCGGCAA   758
m141102_iid=754225_0       TTTTTTAGAAATAGCAACATGAAAAATAACATCAA-TGAGAAATAGAAATTCCCCGGCAA   1928
m141107_iid=1409934_0      TTTTTTAGAAATAGCAACATGAAAAATAACATCAA-TGAGAAATAGAAA-TTCCCGGCAA   2457
m150503_iid=3416920_0      TTTTTTAGAAATAGCAACATGAAAAATAACATCAA-TGAGAAATAGAAATTCCCCGGCAA   1194
m141108_iid=1800121_0      TTTTTTAGAAATAGCAACATGAAAAATAACATCAATG-A-GAATAGAAATTCCCCGGCAA   1001
m141031_iid=147540_0       TTTTTTAGAAATAGCAACATGAAAAATAACATCAA-TGAGAAATAGAAATTCCCCGGCAA   1344
m150507_iid=3674924_0      TTTTTTAGAAATAGCAACATGAAAAATAACATCAA-TGAGAAATAGAAATTCCCCGGCAA   1491
m150503_iid=2923751_0      TTTTTTAGAAATAGCAACATGAAAAATAACATCAA-TGAGAAATAGAAATTCCCCGGCAA   1342
m141031_iid=115118_0       TTTTTCAGAAATAGCAACATGAACAAATA--TCAATAGAGAATAGAAAATTCCCC-----   206
m150430_iid=2722448_0      TTTTTAGGAAATAGCAACATGAAAAATAACATCAA-TGAGAAATAGAAATTCCCCGGCAA   135
m150507_iid=3102647_0      ------------------------------------------------------------   0
m141108_iid=1811866_0      TTTTTTAGAAATAGCAACATGAAA-ATAACATCAA-TGAGAAATAGAAATTCCCCGGCAA   320
m150501_iid=2948495_0      ------------------------------------------------------------   0
m150504_iid=3238195_0      TTTT-TTGAAATAGCAACATGAAAAATAACATCAA-TGAGAAATAGAAATTCCCCGGCAA   2063
m150503_iid=3388511_0      ------------------------------------------------------------   0
m150505_iid=3777611_0      TTTTTTAGAAATAGCAACATGAAAAATAACATCAA-TGAGAAATAGAAATTCCCCGGCAA   1179
m150430_iid=2438833_0      TTTTTTAGAAATAGCAACATGAAAAATAACATCAA-TGAGAAATAGAAATTCCCCGGCAA   1529
m141108_iid=1698969_0      TTTTTTAGAAATAGCAACATGAAAAATAACATCAA-TGAGAAATAGAAATTCCCCGGCAA   2648
m150430_iid=3059190_0      ------------------------------------------------------------   0
m141107_iid=1383067_0      ------------------------------------------------------------   0
m141101_iid=597008_0       ------------------------------------------------------------   0
m150506_iid=2596496_0      TTTTTTAGAAATAGCAACATGAAAAATAACATCAA-TGAGAAATAGAAATTCCCCGGCAA   3004
m141102_iid=910051_0       TTTTTTAGAAATAGCAACATGAAAAATAACATCAA-TGAGAAATAGAAATTCCCCGGCAA   2494
m150505_iid=3784017_0      TTTTTTAGAAATAGCAACATGAAAAATAACATCAA-TGAGAAATAGAAATTCCCCGGCAA   1293
m141101_iid=682151_0       TTTTTTAGAAATAGCAACATGAAAAATAACATCAA-TGAGAAATAGAAATTCCCCGGCAA   363
m150501_iid=3748029_0      TTTTTTAGAAATAGCAACATGAAAAATAACATCAA-TGAGAAATAGAAATTCCCCGGCAA   906
m150504_iid=2876272_0      TTTTTTAGAAATAGCAACATGAAAAATAACATCAA-TGAGAAATAGAAATTCCCCGGCAA   2768
NC_042820.1                TTTTTTAGAAATAGCAACATGAAAAATAACATCAA-TGAGAAATAGAAATTCCCCGGCAA   3011
m141102_iid=1228825_0      TTTTTTAGAAATAGCAACATGAAAAATAACATCAA-TGAGAAATAGAAATTCCCCGGCAA   2838
m150504_iid=2876274_0      TTTTTTAGAAATAGCAACATGAAAAATAACATCAA-TGAGAAATAGAAATTCCCCGGCAA   2841
m141030_iid=80036_0        TTTTTTAGAAATAGCAACATGAAAAATAACATCAA-TGAGAAATAGAAATTCCCCGGCAA   2605
m150506_iid=2596494_0      TTTTTTAGAAATAGCAACATGAAAAATAACATCAA-TGAGAAATAGAAATTCCCCGGCAA   3006
m150503_iid=2526862_0      TTTTTTAGAAATAGCAACATGAAAAATAACATCAA-TGAGAAATAGAAATTCCCCGGCAA   2690
m150506_iid=2567000_0      TTTTTTAGAAATAGCAACATGAAAAATAACATCAA-TGAGAAATAGAAATTCCCCGGCAA   639
m150507_iid=3073271_0      TTTTTTAGAAATAGCAACATGAAAAATAACATCAA-TGAGAAATAGAAATTCCCCGGCAA   1943
m150504_iid=3156087_0      TTTTTTAGAAATAGCAACATGAAAAATAACATCAA-TGAGAAATAGAAATTCCCCGGCAA   1766
m150505_iid=2498502_0      ------------------------------------------------------------   0
m141102_iid=754227_0       TTTTTTAGAAATAGCAACATGAAAAATAACATCAA-TGAGAAATAGAAATTCCCCGGCAA   1978
m150507_iid=3469514_0      TTTTTTAGAAATAGCAACATGAAAAATAACATCAA-TGAGAAATAGAAATTCCCCGGCAA   2536
Rdo_MitoGenome             TTTTTTAGAAATAGCAACATGAAAAATAACATCAA-TGAGAAATAGAAATTCCCCGGCAA   3011
m141101_iid=614933_0       TTTTTTAGAAATAGCAACATGAAAAATAACATCAA-TGAGAAATAGAAATTCCCCGGCAA   1563
```

cox2 →

```
m141031_iid=347546_0     ------------------------------------------------------------  0
m150506_iid=3127414_0    ------------------------------------------------------------  0
m141102_iid=1245517_0    TAGAACAATTAACCTTCTTTCATGACCACACAATAACAGTTTTAATTATAATTACAATCC  1204
m141107_iid=1405727_0    ------------------------------------------------------------  0
m141102_iid=987614_0     TAGAACAATTAACCTTCTTTCATGACCACACAATAACAGTTTTAATTATAATTACAATCC  1340
m141108_iid=1701429_0    ------------------------------------------------------------  0
m141102_iid=987286_0     ------------------------------------------------------------  0
m150430_iid=3272125_0    TAGAACAATTAACCTTCTTTCATGACCACACAATAACAGTTTTAATTATAATTACAATCC  818
m141102_iid=754225_0     TAGAACAATTAACCTTCTTTCATGACCACACAATAACAGTTTTAATTATAATTACAATCC  1988
m141107_iid=1409934_0    TAGAACAATTAACCTTCTTTCATGACCACACAATAACAGTTTTAATTATAATTACAATCC  2517
m150503_iid=3416920_0    TAGAACAATTAACCTTCTTTCATGACCACACAATAACAGTTTTAATTATAATTACAATCC  1254
m141108_iid=1800121_0    TAGAACAATTAACCTTCTTTCATGACCACACAATAACAGTTTTAATTATAATTACAATCC  1061
m141031_iid=147540_0     TAGAACAATTAACCTTCTTTCATGACCACACAATAACAGTTTTAATTATAATTACAATCC  1404
m150507_iid=3674924_0    TAGAACAATTAACCTTCTTTCATGACCACACAATAACAGTTTTAATTATAATTACAATCC  1551
m150503_iid=2923751_0    TAGAACAATTAACCTTCTTTCATGACCACACAATAACAGTTTTAATTATAATTACAATCC  1402
m141031_iid=115118_0     CAGAACAATTAACCTTCTTTCATGACCACACAATAAGTTT-----TAATATTCACAATCC  261
m150430_iid=2722448_0    TAGAACAATTAACCTTCTTTCATGACCACACAATAACAGTTTTAATTATAATTACAATCC  195
m150507_iid=3102647_0    ------------------------------------------------------------  0
m141108_iid=1811866_0    TAGAACAATTAACCTTCTTTCATGACCACACAATAACAGTTTTAATTATAATTACAATCC  380
m150501_iid=2948495_0    ------------------------------------------------------------  0
m150504_iid=3238195_0    TAGAACAATTAACCTTCTTTCATGACCACACAATAACAGTTTTAATTATAATTACAATCC  2123
m150503_iid=3388511_0    ------------------------------------------------------------  0
m150505_iid=3777611_0    TAGAACAATTAACCTTCTTTCATGACCACACAATAACAGTTTTAATTATAATTACAATCC  1239
m150430_iid=2438833_0    TAGAACAATTAACCTTCTTTCATGACCACACAATAACAGTTTTAATTATAATTACAATCC  1589
m141108_iid=1698969_0    TAGAACAATTAACCTTCTTTCATGACCACACAATAACAGTTTTAATTATAATTACAATCC  2708
m150430_iid=3059190_0    ------------------------------------------------------------  0
m141107_iid=1383067_0    ------------------------------------------------------------  0
m141101_iid=597008_0     ------------------------------------------------------------  0
m150506_iid=2596496_0    TAGAACAATTAACCTTCTTTCATGACCACACAATAACAGTTTTAATTATAATTACAATCC  3064
m141102_iid=910051_0     TAGAACAATTAACCTTCTTTCATGACCACACAATAACAGTTTTAATTATAATTACAATCC  2554
m150505_iid=3784017_0    TAGAACAATTAACCTTCTTTCATGACCACACAATAACAGTTTTAATTATAATTACAATCC  1353
m141101_iid=682151_0     TAGAACAATTAACCTTCTTTCATGACCACACAATAACAGTTTTAATTATAATTACAATCC  423
m150501_iid=3748029_0    TAGAACAATTAACCTTCTTTCATGACCACACAATAACAGTTTTAATTATAATTACAATCC  966
m150504_iid=2876272_0    TAGAACAATTAACCTTCTTTCATGACCACACAATAACAGTTTTAATTATAATTACAATCC  2828
NC_042820.1              TAGAACAATTAACCTTCTTTCATGACCACACAATAACAGTTTTAATTATAATTACAATCC  3071
m141102_iid=1228825_0    TAGAACAATTAACCTTCTTTCATGACCACACAATAACAGTTTTAATTATAATTACAATCC  2898
m150504_iid=2876274_0    TAGAACAATTAACCTTCTTTCATGACCACACAATAACAGTTTTAATTATAATTACAATCC  2901
m141030_iid=80036_0      TAGAACAATTAACCTTCTTTCATGACCACACAATAACAGTTTTAATTATAATTACAATCC  2665
m150506_iid=2596494_0    TAGAACAATTAACCTTCTTTCATGACCACACAATAACAGTTTTAATTATAATTACAATCC  3066
m150503_iid=2526862_0    TAGAACAATTAACCTTCTTTCATGACCACACAATAACAGTTTTAATTATAATTACAATCC  2750
m150506_iid=2567000_0    TAGAACAATTAACCTTCTTTCATGACCACACAATAACAGTTTTAATTATAATTACAATCC  699
m150507_iid=3073271_0    TAGAACAATTAACCTTCTTTCATGACCACACAATAACAGTTTTAATTATAATTACAATCC  2003
m150504_iid=3156087_0    TAGAACAATTAACCTTCTTTCATGACCACACAATAACAGTTTTAATTATAATTACAATCC  1826
m150505_iid=2498502_0    ------------------------------------------------------------  0
m141102_iid=754227_0     TAGAACAATTAACCTTCTTTCATGACCACACAATAACAGTTTTAATTATAATTACAATCC  2038
m150507_iid=3469514_0    TAGAACAATTAACCTTCTTTCATGACCACACAATAACAGTTTTAATTATAATTACAATCC  2596
Rdo_MitoGenome           TAGAACAATTAACCTTCTTTCATGACCACACAATAACAGTTTTAATTATAATTACAATCC  3071
m141101_iid=614933_0     TAGAACAATTAACCTTCTTTCATGACCACACAATAACAGTTTTAATTATAATTACAATCC  1623
```

cox2

```
m141031_iid=347546_0     ------------------------------------------------------------  0
m150506_iid=3127414_0    ------------------------------------------------------------  0
m141102_iid=1245517_0    TAGTAGGATACCTA---TCAACACTATTTACAAATAAGTTAACATATCGGTTCCTATTAG  1261
m141107_iid=1405727_0    ------------------------------------------------------------  0
m141102_iid=987614_0     TAGTAGGATACCTAATATCAACACTATTTACAAATAAGTTAACATATCGGTTCCTATTAG  1400
m141108_iid=1701429_0    ------------------------------------------------------------  0
m141102_iid=987286_0     ------------------------------------------------------------  0
m150430_iid=3272125_0    TAGTAGGATACCTAATATCAACACTATTTACAAATAAGTTAACATATCGGTTCCTATTAG  878
m141102_iid=754225_0     TAGTAGGATACCTAATATCAACACTATTTACAAATAAGTTAACATATCGGTTCCTATTAG  2048
m141107_iid=1409934_0    TAGTAGGATACCTAATATCAACACTATTTACAAATAAGTTAACATATCGGTTCCTATTAG  2577
m150503_iid=3416920_0    TAGTAGGATACCTAATATCAACACTATTTACAAATAAGTTAACATATCGGTTCCTATTAG  1314
m141108_iid=1800121_0    TAGTAGGATACCTAATATCAACACTATTTACAAATAAGTTAACATATCGGTTCCTATTAG  1121
m141031_iid=147540_0     TAGTAGGATACCTAATATCAACACTATTTACAAATAAGTTAACATATCGGTTCCTATTAG  1464
m150507_iid=3674924_0    TAGTAGGATACCTAATATCAACACTATTTACAAATAAGTTAACATATCGGTTCCTATTAG  1611
m150503_iid=2923751_0    TAGTAGGATACCTAATATCAACACTATTTACAAATAAGTTAACATATCGGTTCCTATTAG  1462
m141031_iid=115118_0     TAGTAGTCAATCA----------ACTATTACAAATAAGTTAACATATCGGTTCCTATTAG  311
m150430_iid=2722448_0    TAGTAGGATACCTAATATCAACACTATTTACAAATAAGTTAACATATCGGTTCCTATTAG  255
m150507_iid=3102647_0    ------------------------------------------------------------  0
m141108_iid=1811866_0    TAGTAGGATACCTAATATCAACACTATTTACAAATAAGTTAACATATCGGTTCCTATTAG  440
m150501_iid=2948495_0    ------------------------------------------------------------  0
m150504_iid=3238195_0    TAGTAGGATACCTAATATCAACACTATTTACAAATAAGTTAACATATCGGTTCCTATTAG  2183
```

m150503_iid=3388511_0	--	0
m150505_iid=3777611_0	TAGTAGGATACCTAATATCAACACTATTTACAAATAAGTTAACATATCGGTTCCTATTAG	1299
m150430_iid=2438833_0	TAGTAGGATACCTAATATCAACACTATTTACAAATAAGTTAACATATCGGTTCCTATTAG	1649
m141108_iid=1698969_0	TAGTAGGATACCTAATATCAACACTATTTACAAATAAGTTAACATATCGGTTCCTATTAG	2768
m150430_iid=3059190_0	--	0
m141107_iid=1383067_0	--	0
m141101_iid=597008_0	--	0
m150506_iid=2596496_0	TAGTAGGATACCTAATATCAACACTATTTACAAATAAGTTAACATATCGGTTCCTATTAG	3124
m141102_iid=910051_0	TAGTAGGATACCTAATATCAACACTATTTACAAATAAGTTAACATATCGGTTCCTATTAG	2614
m150505_iid=3784017_0	TAGTAGGATACCTAATATCAACACTATTTACAAATAAGTTAACATATCGGTTCCTATTAG	1413
m141101_iid=682151_0	TAGTAGGATACCTAATATCAACACTATTTACAAATAAGTTAACATATCGGTTCCTATTAG	483
m150501_iid=3748029_0	TAGTAGGATACCTAATATCAACACTATTTACAAATAAGTTAACATATCGGTTCCTATTAG	1026
m150504_iid=2876272_0	TAGTAGGATACCTAATATCAACACTATTTACAAATAAGTTAACATATCGGTTCCTATTAG	2888
NC_042820.1	TAGTAGGATACCTAATATCAACACTATTTACAAATAAGTTAACATATCGGTTCCTATTAG	3131
m141102_iid=1228825_0	TAGTAGGATACCTAATATCAACACTATTTACAAATAAGTTAACATATCGGTTCCTATTAG	2958
m150504_iid=2876274_0	TAGTAGGATACCTAATATCAACACTATTTACAAATAAGTTAACATATCGGTTCCTATTAG	2961
m141030_iid=80036_0	TAGTAGGATACCTAATATCAACACTATTTACAAATAAGTTAACATATCGGTTCCTATTAG	2725
m150506_iid=2596494_0	TAGTAGGATACCTAATATCAACACTATTTACAAATAAGTTAACATATCGGTTCCTATTAG	3126
m150503_iid=2526862_0	TAGTAGGATACCTAATATCAACACTATTTACAAATAAGTTAACATATCGGTTCCTATTAG	2810
m150506_iid=2567000_0	TAGTAGGATACCTAATATCAACACTATTTACAAATAAGTTAACATATCGGTTCCTATTAG	759
m150507_iid=3073271_0	TAGTAGGATACCTAATATCAACACTATTTACAAATAAGTTAACATATCGGTTCCTATTAG	2063
m150504_iid=3156087_0	TAGTAGGATACCTAATATCAACACTATTTACAAATAAGTTAACATATCGGTTCCTATTAG	1886
m150505_iid=2498502_0	--	0
m141102_iid=754227_0	TAGTAGGATACCTAATATCAACACTATTTACAAATAAGTTAACATATCGGTTCCTATTAG	2098
m150507_iid=3469514_0	TAGTAGGATACCTAATATCAACACTATTTACAAATAAGTTAACATATCGGTTCCTATTAG	2656
Rdo_MitoGenome	TAGTAGGATACCTAATATCAACACTATTTACAAATAAGTTAACATATCGGTTCCTATTAG	3131
m141101_iid=614933_0	TAGTAGGATACCTAATATCAACACTATTTACAAATAAGTTAACATATCGGTTCCTATTAG	1683

cox2

m141031_iid=347546_0	--	0
m150506_iid=3127414_0	--	0
m141102_iid=1245517_0	AAGGACAAACAATTGAAATAATTTGAACTATTCTACCAGCAATTACATTAATTTTTATTG	1321
m141107_iid=1405727_0	--	0
m141102_iid=987614_0	AAGGACAAACAATTGAAATAATTTGAACTATTCTACCAGCAATTACATTAATTTTTATTG	1460
m141108_iid=1701429_0	--	0
m141102_iid=987286_0	--	0
m150430_iid=3272125_0	AAGGACAAACAT-TGAAATAATTTGAACTATTCTACCAGCAATTACATTAATTTTTATTG	937
m141102_iid=754225_0	AAGGACAAACAATTGAAATAATTTGAACTATTCTACCAGCAATTACATTAATTTTTATTG	2108
m141107_iid=1409934_0	AAGGACAAACAATTGAAATAATTTGAACTATTCTACCAGCAATTACATTAATTTTTATTG	2637
m150503_iid=3416920_0	AAGGACAAACAATTGAAATAATTTGAACTATTCTACCAGCAATTACATTAATTTTTATTG	1374
m141108_iid=1800121_0	AAGGACAAACAATTGAAATAATTTGAACTATTCTACCAGCAATTACATTAATTTTTATTG	1181
m141031_iid=147540_0	AAGGACAAACAATTGAAATAATTTGAACTATTCTACCAGCAATTACATTAATTTTTATTG	1524
m150507_iid=3674924_0	AAGGACAAACAATTGAAATAATTTGAACTATTCTACCAGCAATTACATTAATTTTTATTG	1671
m150503_iid=2923751_0	AAGGACAAACAATTGAAATAATTTGAACTATTCTACCAGCAT-TACATTAATTTTTATTG	1521
m141031_iid=115118_0	AAGGACC---AATTGAAATAATTTGAACTATTCTACCAGCAATTACATTAATTTTTATTG	368
m150430_iid=2722448_0	AAGGACAAACAATTGAAATAATTTGAACTATTCTACCAGCAATTACATTAATTTTTATTG	315
m150507_iid=3102647_0	--	0
m141108_iid=1811866_0	AAGGACAAACAATTGAAATAATTTGAACTATTCTACCAGCAATTACATTAATTTTTATTG	500
m150501_iid=2948495_0	--	0
m150504_iid=3238195_0	AAGGACAAACAATTGAAATAATTTGAACTATTCTACCAGCAATTACATTAATTTTTATTG	2243
m150503_iid=3388511_0	--	0
m150505_iid=3777611_0	AAGGACAAACAATTGAAATAATTTGAACTATTCTACCAGCAATTACATTAATTTTTATTG	1359
m150430_iid=2438833_0	AAGGACAAACAATTGAAATAATTTGAACTATTCTACCAGCAATTACATTAATTTTTATTG	1709
m141108_iid=1698969_0	AAGGACAAACAATTGAAATAATTTGAACTATTCTACCAGCAATTACATTAATTTTTATTG	2828
m150430_iid=3059190_0	--	0
m141107_iid=1383067_0	--	0
m141101_iid=597008_0	--	0
m150506_iid=2596496_0	AAGGACAAACAATTGAAATAATTTGAACTATTCTACCAGCAATTACATTAATTTTTATTG	3184
m141102_iid=910051_0	AAGGACAAACAATTGAAATAATTTGAACTATTCTACCAGCAATTACATTAATTTTTATTG	2674
m150505_iid=3784017_0	AAGGACAAACAATTGAAATAATTTGAACTATTCTACCAGCAATTACATTAATTTTTATTG	1473
m141101_iid=682151_0	AAGGACAAACAATTGAAATAATTTGAACTATT-TACCAGCAATTACATTAATTTTTATTG	542
m150501_iid=3748029_0	AAGGACAAACAATTGAAATAATTTGAACTATTCTACCAGCAATTACATTAATTTTTATTG	1086
m150504_iid=2876272_0	AAGGACAAACAATTGAAATAATTTGAACTATTCTACCAGCAATTACATTAATTTTTATTG	2948
NC_042820.1	AAGGACAAACAATTGAAATAATTTGAACTATTCTACCAGCAATTACATTAATTTTTATTG	3191
m141102_iid=1228825_0	AAGGACAAACAATTGAAATAATTTGAACTATTCTACCAGCAATTACATTAATTTTTATTG	3018
m150504_iid=2876274_0	AAGGACAAACAATTGAAATAATTTGAACTATTCTACCAGCAATTACATTAATTTTTATTG	3021
m141030_iid=80036_0	AAGGACAAACAATTGAAATAATTTGAACTATTCTACCAGCAATTACATTAATTTTTATTG	2785
m150506_iid=2596494_0	AAGGACAAACAATTGAAATAATTTGAACTATTCTACCAGCAATTACATTAATTTTTATTG	3186
m150503_iid=2526862_0	AAGGACAAACAATTGAAATAATTTGAACTATTCTACCAGCAATTACATTAATTTTTATTG	2870
m150506_iid=2567000_0	AAGGACAAACAATTGAAATAATTTGAACTATTCTACCAGCAATTACATTAATTTTTATTG	819
m150507_iid=3073271_0	AAGGACAAACAATTGAAATAATTTGAACTATTCTACCAGCAATTACATTAATTTTTATTG	2123

```
m150504_iid=3156087_0      AAGGACAAACAATTGAAATAATTTGAACTATTCTACCAGCAATTACATTAATTTTTATTG   1946
m150505_iid=2498502_0      ------------------------------------------------------------   0
m141102_iid=754227_0       AAGGACAAACAATTGAAATAATTTGAACTATTCTACCAGCAATTACATTAATTTTTATTG   2158
m150507_iid=3469514_0      AAGGACAAACAATTGAAATAATTTGAACTATTCTACCAGCAATTACATTAATTTTTATTG   2716
Rdo_MitoGenome             AAGGACAAACAATTGAAATAATTTGAACTATTCTACCAGCAATTACATTAATTTTTATTG   3191
m141101_iid=614933_0       AAGGACAAACAATTGAAATAATTTGAACTATTCTACCAGCAATTACATTAATTTTTATTG   1743
```

cox2

```
m141031_iid=347546_0       ------------------------------------------------------------   0
m150506_iid=3127414_0      ------------------------------------------------------------   0
m141102_iid=1245517_0      CCTTACCCTCACTTCAA-ATTCTTTACACACTAGATGAAA-TTATTAACCCATCCATATC   1379
m141107_iid=1405727_0      ------------------------------------------------------------   0
m141102_iid=987614_0       CCTTACCCTCACTTCAA-ATTCTTTACACACTAGATGAAA-TTATTAACCCATCCATATC   1518
m141108_iid=1701429_0      ------------------------------------------------------------   0
m141102_iid=987286_0       ------------------------------------------------------------   0
m150430_iid=3272125_0      CCTTACCCTCACTTCAA-ATTCTTTACACACTAGATGAAATT--ATTAACCATCCATATC   994
m141102_iid=754225_0       CCTTACCCTCACTTCAA-ATTCTTTACACACTAGATGAAA-TTATTAACCCATCCATATC   2166
m141107_iid=1409934_0      CCTTACCCTCACTTCAA-ATTCTTTACACACTAGATGAAA-TTATTAACCCATCCATATC   2695
m150503_iid=3416920_0      CCTTACCCTCACTTCAA-ATTCTTTACACACTAGATGAAA-TTATTAACCCATCCATATC   1432
m141108_iid=1800121_0      CCTTACCCTCACTTCAAAATTCTTTACACACTAGATGAAA-TTATTAACCCATCCATATC   1240
m141031_iid=147540_0       CCTTACCCTCACTTCAA-ATTCTTTACACACTAGATGAAA-TTATTAACCCATCCATATC   1582
m150507_iid=3674924_0      CCTTACCCTCACTTCAA-ATTCTTTACACACTAGATGAAA-TTATTAACCCATCCATATC   1729
m150503_iid=2923751_0      CCTTACCCTCACTTCAA-ATTCTTTACACACTAGATGAAA-TTATTAACCCATCCATATC   1579
m141031_iid=115118_0       CCTTACCCTCACTTCAA-ATTCTTTACACACTAGATGAAA-TTATTAACCCATCCATATC   426
m150430_iid=2722448_0      CCTTACCCTCACTTCAA-ATTCTTTACACACTAGATGAAA-TTATTAACCCATCCATATC   373
m150507_iid=3102647_0      ------------------------------------------------------------   0
m141108_iid=1811866_0      CCTTACCCTCACTTCAA-ATTCTTTACACACTAGATGAAA-TTATTAACCCATCCATATC   558
m150501_iid=2948495_0      ------------------------------------------------------------   0
m150504_iid=3238195_0      CCTTACCCTCACTTCAA-ATTCTTTACACACTAGATGAAA-TTATTAACCCATCCATATC   2301
m150503_iid=3388511_0      ---------------------------------------------------------ATC   3
m150505_iid=3777611_0      CCTTACCCTCACTTCAA-ATTCTTTACACACTAGATGAAA-TTATTAACCCATCCATATC   1417
m150430_iid=2438833_0      CCTTACCCTCACTTCAA-ATTCTTTACACACTAGATGAAA-TTATTAACCCATCCATATC   1767
m141108_iid=1698969_0      CCTTACCCTCACTTCAA-ATTCTTTACACACTAGATGAAA-TTATTAACCCATCCATATC   2886
m150430_iid=3059190_0      ------------------------------------------------------------   0
m141107_iid=1383067_0      ------------------------------------------------------------   0
m141101_iid=597008_0       ------------------------------------------------------------   0
m150506_iid=2596496_0      CCTTACCCTCACTTCAA-ATTCTTTACACACTAGATGAAA-TTATTAACCCATCCATATC   3242
m141102_iid=910051_0       CCTTACCCTCACTTCAA-ATTCTTTACACACTAGATGAAA-TTATTAACCCATCCATATC   2732
m150505_iid=3784017_0      CCTTACCCTCACTTCAA-ATTCTTTACACACTAGATGAAA-TTATTAACCCATCCATATC   1531
m141101_iid=682151_0       CCTTACCCTCACTTCAA-ATTCTTTACACACTAGATGAAATTTATTAACCCATCCATATC   601
m150501_iid=3748029_0      CCTTACCCTCACTTCAA-ATTCTTTACACACTAGATGAAA-TTATTAACCCATCCATATC   1144
m150504_iid=2876272_0      CCTTACCCTCACTTCAA-ATTCTTTACACACTAGATGAAA-TTATTAACCCATCCATATC   3006
NC_042820.1                CCTTACCCTCACTTCAG-ATTCTTTACACACTAGATGAAA-TTATTAACCCATCCATATC   3249
m141102_iid=1228825_0      CCTTACCCTCACTTCAA-ATTCTTTACACACTAGATGAAA-TTATTAACCCATCCATATC   3076
m150504_iid=2876274_0      CCTTACCCTCACTTCAA-ATTCTTTACACACTAGATGAAA-TTATTAACCCATCCATATC   3079
m141030_iid=80036_0        CCTTACCCTCACTTCAA-ATTCTTTACACACTAGATGAAA-TTATTAACCCATCCATATC   2843
m150506_iid=2596494_0      CCTTACCCTCACTTCAA-ATTCTTTACACACTAGATGAAA-TTATTAACCCATCCATATC   3244
m150503_iid=2526862_0      CCTTACCCTCACTTCAA-ATTCTTTACACACTAGATGAAA-TTATTAACCCATCCATATC   2928
m150506_iid=2567000_0      CCTTACCCTCACTTCAA-ATTCTTTACACACTAGATGAAA-TTATTAACCCATCCATATC   877
m150507_iid=3073271_0      CCTTACCCTCACTTCAA-ATTCTTTACACACTAGATGAAA-TTATTAACCCATCCATATC   2181
m150504_iid=3156087_0      CCTTACCCTCACTTCAA-ATTCTTTACACACTAGATGAAA-TTATTAACCCATCCATATC   2004
m150505_iid=2498502_0      ------------------------------------------------------------   0
m141102_iid=754227_0       CCTTACCCTCACTTCAA-ATTCTTTACACACTAGATGAAA-TTATTAACCCATCCATATC   2216
m150507_iid=3469514_0      CCTTACCCTCACTTCAA-ATTCTTTACACACTAGATGAAA-TTATTAACCCATCCATATC   2774
Rdo_MitoGenome             CCTTACCCTCACTTCAA-ATTCTTTACACACTAGATGAAA-TTATTAACCCATCCATATC   3249
m141101_iid=614933_0       CCTTACCCTCACTTCAA-ATTCTTTACACACTAGATGAAA-TTATTAACCCATCCATATC   1801
```

cox2

```
m141031_iid=347546_0       ------------------------------------------------------------   0
m150506_iid=3127414_0      ------------------------------------------------------------   0
m141102_iid=1245517_0      AGTTAAATCTATTGGACATCAATGATACTGAAGATATGAAT--ATTCAGACTTCAAAAAA   1437
m141107_iid=1405727_0      ------------------------------------------------------------   0
m141102_iid=987614_0       AGTTAAATCTATTGGACATCAATGATACTGAAGATATGAAT--ATTCAGACTTCAAAAAA   1576
m141108_iid=1701429_0      ------------------------------------------------------------   0
m141102_iid=987286_0       ------------------------------------------------------------   0
m150430_iid=3272125_0      AGTTAAATCTATTGGACATCAATGATACTGAAGATATGAAA-TATTCAGACTTCAAAAAA   1053
m141102_iid=754225_0       AGTTAAATCTATTGGACATCAATGATACTGAAGATATGAAT--ATTCAGACTTCAAAAAA   2224
m141107_iid=1409934_0      AGTTAAATCTATTGGACATCAATGATACTGAAGATATGAAT--ATTCAGACTTCAAAAAA   2753
m150503_iid=3416920_0      AGTTAAATCTATTGGACATCAATGATACTGAAGATATGAAT--ATTCAGACTTCAAAAAA   1490
m141108_iid=1800121_0      AGTTAAATCTATTGGACATCAATGATACTGAAGATATGAATAATTTCAGACTTCAAAAAA   1300
m141031_iid=147540_0       AGTTAAATCTATTGGACATCAATGATACTGAAGATATGAAT--ATTCAGACTTCAAAAAA   1640
m150507_iid=3674924_0      AGTTAAATCTATTGGACATCAATGATACTGAAGATATGAAT--ATTCAGACTTCAAAAAA   1787
```

```
m150503_iid=2923751_0     AGTTAAATCTATTGGACATCAATGATACTGAAGATATGAAT--ATTCAGACTTCAAAAAA   1637
m141031_iid=115118_0      AGTTAAATCTATTGGACATCAATGATACTGAAGATATGAAT--ATTCAGACTTCAAAAAA   484
m150430_iid=2722448_0     AGTTAAATCTATTGGACATCAATGATACTGAAGATATGAAT--ATTCAGACTTCAAAAAA   431
m150507_iid=3102647_0     ------------------------------------------------------------   0
m141108_iid=1811866_0     AGTTAAATCTATTGGACATCAATGATACTGAAGATATGAAT--ATTCAGACTTCAAAAAA   616
m150501_iid=2948495_0     ----------------------------TGAAGATATGAAT--ATTCAGACTTCAAA-AA   29
m150504_iid=3238195_0     AGTTAAATCTATTGGACATCAATGATACTGAAGATATGAAT--ATTCAGACTTCAAA-AA   2358
m150503_iid=3388511_0     AGTTAAATCTATTGGACATCAATGATACTGAAGATATGAAT--ATTCAGACTTCAAAAAA   61
m150505_iid=3777611_0     AGTTAAATCTATTGGACATCAATGATACTGAAGATATGAAT--ATTCAGACTTCAAAAAA   1475
m150430_iid=2438833_0     AGTTAAATCTATTGGACATCAATGATACTGAAGATATGAAT--ATTCAGACTTCAAAAAA   1825
m141108_iid=1698969_0     AGTTAAATCTATTGGACATCAATGATACTGAAGATATGAAT--ATTCAGACTTCAAAAAA   2944
m150430_iid=3059190_0     ------------------------------------------------------------   0
m141107_iid=1383067_0     ------------------------------------------------------------   0
m141101_iid=597008_0      ------------------------------------------------------------   0
m150506_iid=2596496_0     AGTTAAATCTATTGGACATCAATGATACTGAAGATATGAAT--ATTCAGACTTCAAAAAA   3300
m141102_iid=910051_0      AGTTAAATCTATTGGACATCAATGATACTGAAGATATGAAT--ATTCAGACTTCAAAAAA   2790
m150505_iid=3784017_0     AGTTAAATCTATTGGACATCAATGATACTGAAGATATGAAT--ATTCAGACTTCAAAAAA   1589
m141101_iid=682151_0      AGTTAAATCTATTGGACATCAATGATACTGAAGATATGAAT--ATTCAGACTTCAAAAAA   659
m150501_iid=3748029_0     AGTTAAATCTATTGGACATCAATGATACTGAAGATATGAAT--ATTCAGACTTCAAAAAA   1202
m150504_iid=2876272_0     AGTTAAATCTATTGGACATCAATGATACTGAAGATATGAAT--ATTCAGACTTCAAAAAA   3064
NC_042820.1               AGTTAAATCTATTGGACATCAATGATACTGAAGATATGAAT--ATTCAGACTTCAAAAAA   3307
m141102_iid=1228825_0     AGTTAAATCTATTGGACATCAATGATACTGAAGATATGAAT--ATTCAGACTTCAAAAAA   3134
m150504_iid=2876274_0     AGTTAAATCTATTGGACATCAATGATACTGAAGATATGAAT--ATTCAGACTTCAAAAAA   3137
m141030_iid=80036_0       AGTTAAATCTATTGGACATCAATGATACTGAAGATATGAAT--ATTCAGACTTCAAAAAA   2901
m150506_iid=2596494_0     AGTTAAATCTATTGGACATCAATGATACTGAAGATATGAAT--ATTCAGACTTCAAAAAA   3302
m150503_iid=2526862_0     AGTTAAATCTATTGGACATCAATGATACTGAAGATATGAAT--ATTCAGACTTCAAAAAA   2986
m150506_iid=2567000_0     AGTTAAATCTATTGGACATCAATGATACTGAAGATATGAAT--ATTCAGACTTCAAAAAA   935
m150507_iid=3073271_0     AGTTAAATCTATTGGACATCAATGATACTGAAGATATGAAT--ATTCAGACTTCAAAAAA   2239
m150504_iid=3156087_0     AGTTAAATCTATTGGACATCAATGATACTGAAGATATGAAT--ATTCAGACTTCAAAAAA   2062
m150505_iid=2498502_0     ------------------------------------------------------------   0
m141102_iid=754227_0      AGTTAAATCTATTGGACATCAATGATACTGAAGATATGAAT--ATTCAGACTTCAAAAAA   2274
m150507_iid=3469514_0     AGTTAAATCTATTGGACATCAATGATACTGAAGATATGAAT--ATTCAGACTTCAAAAAA   2832
Rdo_MitoGenome            AGTTAAATCTATTGGACATCAATGATACTGAAGATATGAAT--ATTCAGACTTCAAAAAA   3307
m141101_iid=614933_0      AGTTAAATCTATTGGACATCAATGATACTGAAGATATGAAT--ATTCAGACTTCAAAAAA   1859
```

cox2

```
m141031_iid=347546_0      ------------------------------------------------------------   0
m150506_iid=3127414_0     ------------------------------------------------------------   0
m141102_iid=1245517_0     ACCGAATTCGATTCATATATAAAATCACCAAACGAAATCAAAAAATCAAGAATTCCGATT   1497
m141107_iid=1405727_0     ------------------------------------------------------------   0
m141102_iid=987614_0      ACCGAATTCGATTCATATATAAAATCACCAAACGAAATCAAA-AATCAAGAATTCCGATT   1635
m141108_iid=1701429_0     ------------------------------------------------------------   0
m141102_iid=987286_0      ------------------------------------------------------------   0
m150430_iid=3272125_0     ACCGAATTCGATTCATATATAAAATCACCAAACGAAATCAAA-AATCAAGAATTCCGATT   1112
m141102_iid=754225_0      ACCGAATTCGATTCATATATAAAATCACCAAACGAAATCAAA-AATCAAGAATTCCGATT   2283
m141107_iid=1409934_0     ACCGAATTCGATTCATATATAAAATCACCAAACGAAATCAAA-AATCAAGAATTCCGATT   2812
m150503_iid=3416920_0     ACCGAATTCGATTCATATATAAAATCACCAAACGAAATCAAA-AATCAAGAATTCCGATT   1549
m141108_iid=1800121_0     ACCGAATTCGATTCATATATAAAATCACCAAACGAAATCAAA-AATCAAGAATTCCGATT   1359
m141031_iid=147540_0      ACCGAATTCGATTCATATATAAAATCACCAAACGAAATCAAA-AATCAAGAATTCCGATT   1699
m150507_iid=3674924_0     ACCGAATTCGATTCATATATAAAATCACCAAACGAAATCAAA-AATCAAGAATTCCGATT   1846
m150503_iid=2923751_0     ACCGAATTCGATTCATATATAAAATCACCAAACGAAATCAAA-AATCAAGAATTCCGATT   1696
m141031_iid=115118_0      ACCGAATTCGATTCATATATAAAATCACCAAACGAAATCAAA-AATCAAGAATTCCGATT   543
m150430_iid=2722448_0     ACCGAATTCGATTCATATATAAAATCACCAAACGAAATCAAA-AATCAAGAATTCCGATT   490
m150507_iid=3102647_0     ------------------------------------------------------------   0
m141108_iid=1811866_0     ACCGAATTCGATTCATATATAAAATCACCAAACGAAATCAAA-AATCAAGAATTCCGATT   675
m150501_iid=2948495_0     ACCGAATTCGATTCATATATAAAATCACCAAACGAAATCAAA-AATCAAGAATTCCGATT   88
m150504_iid=3238195_0     ACCGAATTCGATTCATATATAAAATCACCAAACGAAATCAAA-AATCAAGAATTCCGATT   2417
m150503_iid=3388511_0     ACCGAATTCGATTCATATATAAAATC-CCAAACGAATC-AA-AATCAAGAATTCCGATT   118
m150505_iid=3777611_0     ACCGAATTCGATTCATATATAAAATCACCAAACGAAATCAAA-AATCAAGAATTCCGATT   1534
m150430_iid=2438833_0     ACCGAATTCGATTCATATATAAAATCACCAAACGAAATCAAA-AATCAAGAATTCCGATT   1884
m141108_iid=1698969_0     ACCGAATTCGATTCATATATAAAATCACCAAACGAAATCAAA-AATCAAGAATTCCGATT   3003
m150430_iid=3059190_0     ------------------------------------------------------------   0
m141107_iid=1383067_0     ------------------------------------------------------------   0
m141101_iid=597008_0      ------------------------------------------------------------   0
m150506_iid=2596496_0     ACCGAATTCGATTCATATATAAAATCACCAAACGAAATCAAA-AATCAAGAATTCCGATT   3359
m141102_iid=910051_0      ACCGAATTCGATTCATATATAAAATCACCAAACGAAATCAAA-AATCAAGAATTCCGATT   2849
m150505_iid=3784017_0     ACCGAATTCGATTCATATATAAAATCACCAAACGAAATCAAA-AATCAAGAATTCCGATT   1648
m141101_iid=682151_0      ACCGAATTCGATTCATATATAAAATCACCAAACGAAATCAAA-AATCAAGAATTCCGATT   718
m150501_iid=3748029_0     ACCGAATTCGATTCATATATAAAATCACCAAACGAAATCAAA-AATCAAGAATTCCGATT   1261
m150504_iid=2876272_0     ACCGAATTCGATTCATATATAAAATCACCAAACGAAATCAAA-AATCAAGAATTCCGATT   3123
NC_042820.1               ACCGAATTCGATTCATATATAAAATCACCAAACGAAATCAAA-AATCAAGAATTCCGATT   3366
```

```
m141102_iid=1228825_0      ACCGAATTCGATTCATATATAAAATCACCAAACGAAATCAAA-AATCAAGAATTCCGATT   3193
m150504_iid=2876274_0      ACCGAATTCGATTCATATATAAAATCACCAAACGAAATCAAA-AATCAAGAATTCCGATT   3196
m141030_iid=80036_0        ACCGAATTCGATTCATATATAAAATCACCAAACGAAATCAAA-AATCAAGAATTCCGATT   2960
m150506_iid=2596494_0      ACCGAATTCGATTCATATATAAAATCACCAAACGAAATCAAA-AATCAAGAATTCCGATT   3361
m150503_iid=2526862_0      ACCGAATTCGATTCATATATAAAATCACCAAACGAAATCAAA-AATCAAGAATTCCGATT   3045
m150506_iid=2567000_0      ACCGAATTCGATTCATATATAAAATCACCAAACGAAATCAAA-AATCAAGAATTCCGATT   994
m150507_iid=3073271_0      ACCGAATTCGATTCATATATAAAATCACCAAACGAAATCAAA-AATCAAGAATTCCGATT   2298
m150504_iid=3156087_0      ACCGAATTCGATTCATATATAAAATCACCAAACGAAATCAAA-AATCAAGAATTCCGATT   2121
m150505_iid=2498502_0      ------------------------------------------------------------   0
m141102_iid=754227_0       ACCGAATTCGATTCATATATAAAATCACCAAACGAAATCAAA-AATCAAGAATTCCGATT   2333
m150507_iid=3469514_0      ACCGAATTCGATTCATATATAAAATCACCAAACGAAATCAAA-AATCAAGAATTCCGATT   2891
Rdo_MitoGenome             ACCGAATTCGATTCATATATAAAATCACCAAACGAAATCAAA-AATCAAGAATTCCGATT   3366
m141101_iid=614933_0       ACCGAATTCGATTCATATATAAAATCACCAAACGAAATCAAA-AATCAAGAATTCCGATT   1918

                                                    cox2

m141031_iid=347546_0       ------------------------------------------------------------   0
m150506_iid=3127414_0      ------------------------------------------------------------   0
m141102_iid=1245517_0      ACTAGATGTAGACAATCGAATAACCTTACAAT--AAAGACAAAAATTCGATTATTAG-TT   1554
m141107_iid=1405727_0      ------------------------------------------------------------   0
m141102_iid=987614_0       ACTAGATGTAGACAATCGAATAACCTTACCAATA-AAGACAAAAATTCGATTATTAG-TT   1693
m141108_iid=1701429_0      ------------------------------------------------------------   0
m141102_iid=987286_0       ------------------------------------------------------------   0
m150430_iid=3272125_0      ACTAGATGTAGACAATCGAATAACCTTACCAATAAAAGACAAAAATTCGATTATTAGTTT   1172
m141102_iid=754225_0       ACTAGATGTAGACAATCGAATAACCTTACCAATA-AAGACAAAAATTCGATTATTAG-TT   2341
m141107_iid=1409934_0      ACTAGATGTAGACAATCGAATAACCTTACCAATA-AAGACAAAAATTCGATTATTAG-TT   2870
m150503_iid=3416920_0      ACTAGATGTAGACAATCGAATAACCTTACCAATA-AAGACAAAAATTCGATTATTAG-TT   1607
m141108_iid=1800121_0      ACTAGATGTAGACAATCGAATAACCTTACCAATA-AAGACAAAAATTCGATTATTAG-TT   1417
m141031_iid=147540_0       ACTAGATGTAGACAATCGAATAACCTTACCAATA-AAGACAAAAATTCGATTATTAG-TT   1757
m150507_iid=3674924_0      ACTAGATGTAGACAATCGAATAACCTTACCAATA-AAGACAAAAATTCGATTATTAG-TT   1904
m150503_iid=2923751_0      ACTAGATGTAGACAATCGAATAACCTTACCAATA-AAGACAAAAATTCGATTATTAG-TT   1754
m141031_iid=115118_0       ACTAGATGTAGACAATCGAATAACCTTACCAATA-AAGACAAAAATTCGATTATTAG-TT   601
m150430_iid=2722448_0      ACTAGATGTAGACAATCGAATAACCTTACCAATAAAAGACAAAAATTCGATTATTAGTTT   550
m150507_iid=3102647_0      ------------------------------------------------------------   0
m141108_iid=1811866_0      ACTAGATGTAGACA-TCGAATAACCTTACCAATA-AAGACAAA-ATTCGATTATTAG-TT   731
m150501_iid=2948495_0      ACTAGATGTAGACAATCGAATAACCTTACCAATA-AAGACAAA-ATTCGATTATTAG-TT   145
m150504_iid=3238195_0      ACTAGATGTAGACAATCGAATAACCTTACCAATA-AAGACAAAAATTCGATTATTAG-TT   2475
m150503_iid=3388511_0      ACTAGATGTAGACAATCGAATAACCTTA-CAATA-AAGACAAAAATTCGATTATTAG-TT   175
m150505_iid=3777611_0      ACTAGATGTAGACAATCGAATAACCTTACCAATA-AAGACAAAAATTCGATTATTAG-TT   1592
m150430_iid=2438833_0      ACTAGATGTAGACAATCGAATAACCTTACCAATA-AAGACAAAAATTCGATTATTAG-TT   1942
m141108_iid=1698969_0      ACTAGATGTAGACAATCGAATAACCTTACCAATA-AAGACAAAAATTCGATTATTAG-TT   3061
m150430_iid=3059190_0      ------------------------------------------------------------   0
m141107_iid=1383067_0      ------------------------------------------------------------   0
m141101_iid=597008_0       ------------------------------------------------------------   0
m150506_iid=2596496_0      ACTAGATGTAGACAATCGAATAACCTTACCAATA-AAGACAAAAATTCGATTATTAG-TT   3417
m141102_iid=910051_0       ACTAGATGTAGACAATCGAATAACCTTACCAATA-AAGACAAAAATTCGATTATTAG-TT   2907
m150505_iid=3784017_0      ACTAGATGTAGACAATCGAATAACCTTACCAATA-AAGACAAAAATTCGATTATTAG-TT   1706
m141101_iid=682151_0       ACTAGATGTAGACAATCGAATAACCTTACCAATA-AAGACAAAAATTCGATTATTAG-TT   776
m150501_iid=3748029_0      ACTAGATGTAGACAATCGAATAACCTTACCAATA-AAGACAAAAATTCGATTATTAG-TT   1319
m150504_iid=2876272_0      ACTAGATGTAGACAATCGAATAACCTTACCAATA-AAGACAAAAATTCGATTATTAG-TT   3181
NC_042820.1                ACTAGATGTAGACAATCGAATAACCTTACCAATA-AAGACAAAAATTCGATTATTAG-TT   3424
m141102_iid=1228825_0      ACTAGATGTAGACAATCGAATAACCTTACCAATA-AAGACAAAAATTCGATTATTAG-TT   3251
m150504_iid=2876274_0      ACTAGATGTAGACAATCGAATAACCTTACCAATA-AAGACAAAAATTCGATTATTAG-TT   3254
m141030_iid=80036_0        ACTAGATGTAGACAATCGAATAACCTTACCAATA-AAGACAAAAATTCGATTATTAG-TT   3018
m150506_iid=2596494_0      ACTAGATGTAGACAATCGAATAACCTTACCAATA-AAGACAAAAATTCGATTATTAG-TT   3419
m150503_iid=2526862_0      ACTAGATGTAGACAATCGAATAACCTTACCAATA-AAGACAAAAATTCGATTATTAG-TT   3103
m150506_iid=2567000_0      ACTAGATGTAGACAATCGAATAACCTTACCAATA-AAGACAAAAATTCGATTATTAG-TT   1052
m150507_iid=3073271_0      ACTAGATGTAGACAATCGAATAACCTTACCAATA-AAGACAAAAATTCGATTATTAG-TT   2356
m150504_iid=3156087_0      ACTAGATGTAGACAATCGAATAACCTTACCAATA-AAGACAAAAATTCGATTATTAG-TT   2179
m150505_iid=2498502_0      ------------------------------------------------------------   0
m141102_iid=754227_0       ACTAGATGTAGACAATCGAATAACCTTACCAATA-AAGACAAAAATTCGATTATTAG-TT   2391
m150507_iid=3469514_0      ACTAGATGTAGACAATCGAATAACCTTACCAATA-AAGACAAAAATTCGATTATTAG-TT   2949
Rdo_MitoGenome             ACTAGATGTAGACAATCGAATAACCTTACCAATA-AAGACAAAAATTCGATTATTAG-TT   3424
m141101_iid=614933_0       ACTAGATGTAGACAATCGAATAACCTTACCAATA-AAGACAAAAATTCGATTATTAG-TT   1976

                                                    cox2

m141031_iid=347546_0       ------------------------------------------------------------   0
m150506_iid=3127414_0      ------------------------------------------------------------   0
m141102_iid=1245517_0      TCGTCAACAGACGTAATTCACTCATGAACAATTCCATCCCTTGGAGTAAAGATTGATGCA   1614
m141107_iid=1405727_0      ------------------------------------------------------------   0
m141102_iid=987614_0       TCGTCAACAGACGTAATTCACTCATGAACAATTCCATCCCTTGGAGTAAAGATTGATGCA   1753
m141108_iid=1701429_0      ------------------------------------------------------------   0
```

```
m141102_iid=987286_0      ------------------------------------------------------------  0
m150430_iid=3272125_0     TCGTCAACAGACGTAATTCACTCATGAACAATTCCATCCCTTGGAGGTA-AATTGATGCA  1231
m141102_iid=754225_0      TCGTCAACAGACGTAATTCACTCATGAACAATTCCATCCCTTGGAGTAAAGATTGATGCA  2401
m141107_iid=1409934_0     TCGTCAACAGACGTAATTCACTCATGAACAATTCCATCCCTTGGAGTAAAGATTGATGCA  2930
m150503_iid=3416920_0     TCGTCAACAGACGTAATTCACTCATGAACAATTCCATCCCTTGGAGTAAAGATTGATGCA  1667
m141108_iid=1800121_0     TCGTCAACAGACGTAATTCACTCATGAACAATTCCATCCTT-GGAG-TAAGATTGATGCA  1475
m141031_iid=147540_0      TCGTCAACAGACGTAATTCACTCATGAACAATTCCATCCCTTGGAGTAAAGATTGATGCA  1817
m150507_iid=3674924_0     TCGTCAACAGACGTAATTCACTCATGAACAATTCCATCCCTTGGAGTAAAGATTGATGCA  1964
m150503_iid=2923751_0     TCGTCAACAGACGTAATTCACTCATGAACAATTCCATCCCTTGGAGTAAAGATTGATGCA  1814
m141031_iid=115118_0      TCGTCAACAGACGTAATTCACTCATGAACAATTCCATCCCTTGGAGTAAAGATTGATGCA  661
m150430_iid=2722448_0     TCGTCAACAGACGTAATTCACTCATGAACAATTCCATCCCTTGGAGTAAAGATTGATGCA  610
m150507_iid=3102647_0     ------------------------------------------------------------  0
m141108_iid=1811866_0     TCGTCAACAGACGTAATTCACTCATGAACAATTCCATCCCTTGGAGTAAAGATTGATGCA  791
m150501_iid=2948495_0     TCGTCAACAGACGTAATTCACTCATGAACAATTCCATCCCTTGGAGTAAAGATTGATGCA  205
m150504_iid=3238195_0     TCGTCAACAGACGTAATTCACTCATGAACAATTCCATCCCTTGGAGTAAAGATTGATGCA  2535
m150503_iid=3388511_0     TCGTCAACAGACGTAATTCACTCATGAACAATTCCATCCCTTGGAGTAAAGATTGATGCA  235
m150505_iid=3777611_0     TCGTCAACAGACGTAATTCACTCATGAACAATTCCATCCCTTGGAGTAAAGATTGATGCA  1652
m150430_iid=2438833_0     TCGTCAACAGACGTAATTCACTCATGAACAATTCCATCCCTTGGAGTAAAGATTGATGCA  2002
m141108_iid=1698969_0     TCGTCAACAGACGTAATTCACTCATGAACAATTCCATCCCTTGGAGTAAAGATTGATGCA  3121
m150430_iid=3059190_0     ------------------------------------------------------------  0
m141107_iid=1383067_0     ------------------------------------------------------------  0
m141101_iid=597008_0      ------------------------------------------------------------  0
m150506_iid=2596496_0     TCGTCAACAGACGTAATTCACTCATGAACAATTCCATCCCTTGGAGTAAAGATTGATGCA  3477
m141102_iid=910051_0      TCGTCAACAGACGTAATTCACTCATGAACAATTCCATCCCTTGGAGTAAAGATTGATGCA  2967
m150505_iid=3784017_0     TCGTCAACAGACGTAATTCACTCATGAACAATTCCATCCCTTGGAGTAAAGATTGATGCA  1766
m141101_iid=682151_0      TCGTCAACAGACGTAATTCACTCATGAACAATTCCATCCCTTGGAGTAAAGATTGATGCA  836
m150501_iid=3748029_0     TCGTCAACAGACGTAATTCACTCATGAACAATTCCATCCCTTGGAGTAAAGATTGATGCA  1379
m150504_iid=2876272_0     TCGTCAACAGACGTAATTCACTCATGAACAATTCCATCCCTTGGAGTAAAGATTGATGCA  3241
NC_042820.1               TCGTCAACAGACGTAATTCACTCATGAACAATTCCATCCCTTGGAGTAAAGATTGATGCA  3484
m141102_iid=1228825_0     TCGTCAACAGACGTAATTCACTCATGAACAATTCCATCCCTTGGAGTAAAGATTGATGCA  3311
m150504_iid=2876274_0     TCGTCAACAGACGTAATTCACTCATGAACAATTCCATCCCTTGGAGTAAAGATTGATGCA  3314
m141030_iid=80036_0       TCGTCAACAGACGTAATTCACTCATGAACAATTCCATCCCTTGGAGTAAAGATTGATGCA  3078
m150506_iid=2596494_0     TCGTCAACAGACGTAATTCACTCATGAACAATTCCATCCCTTGGAGTAAAGATTGATGCA  3479
m150503_iid=2526862_0     TCGTCAACAGACGTAATTCACTCATGAACAATTCCATCCCTTGGAGTAAAGATTGATGCA  3163
m150506_iid=2567000_0     TCGTCAACAGACGTAATTCACTCATGAACAATTCCATCCCTTGGAGTAAAGATTGATGCA  1112
m150507_iid=3073271_0     TCGTCAACAGACGTAATTCACTCATGAACAATTCCATCCCTTGGAGTAAAGATTGATGCA  2416
m150504_iid=3156087_0     TCGTCAACAGACGTAATTCACTCATGAACAATTCCATCCCTTGGAGTAAAGATTGATGCA  2239
m150505_iid=2498502_0     ------------------------------------------------------------  0
m141102_iid=754227_0      TCGTCAACAGACGTAATTCACTCATGAACAATTCCATCCCTTGGAGTAAAGATTGATGCA  2451
m150507_iid=3469514_0     TCGTCAACAGACGTAATTCACTCATGAACAATTCCATCCCTTGGAGTAAAGATTGATGCA  3009
Rdo_MitoGenome            TCGTCAACAGACGTAATTCACTCATGAACAATTCCATCCCTTGGAGTAAAGATTGATGCA  3484
m141101_iid=614933_0      TCGTCAACAGACGTAATTCACTCATGAACAATTCCATCCCTTGGAGTAAAGATTGATGCA  2036
```

cox2

```
m141031_iid=347546_0      ------------------------------------------------------------  0
m150506_iid=3127414_0     -----------------------AAGAATATTCATCATTAATCCAGGACTAATATACGGA  38
m141102_iid=1245517_0     ACACCAGGGCGTCTAAACCAAGCAAGAATATTCATCATTAATCCAGGACTAATATACGGA  1674
m141107_iid=1405727_0     ------------------------------------------------------------  0
m141102_iid=987614_0      ACACCAGGGCGTCTAAACCAAGCAAGAATATTCATCATTAATCCAGGACTAATATACGGA  1813
m141108_iid=1701429_0     ------------------------------------------------------------  0
m141102_iid=987286_0      ------------------------------------------------------------  0
m150430_iid=3272125_0     ACACCAGGGCGTCTAAACCAAGCAAGAATATTCATCATTAATCCAGGACTAATATACGGA  1291
m141102_iid=754225_0      ACACCAGGGCGTCTAAACCAAGCAAGAATATTCATCATTAATCCAGGACTAATATACGGA  2461
m141107_iid=1409934_0     ACACCAGGGCGTCTAAACCAAGCAAGAATATTCATCATTAATCCAGGACTAATATACGGA  2990
m150503_iid=3416920_0     ACACCAGGGCGTCTAAACCAAGCAAGAATATTCATCATTAATCCAGGACTAATATACGGA  1727
m141108_iid=1800121_0     ACACCAGGGCGTCTAAACCAAGCAAGAATATTCATCATTAATCCAGGACTAATATACGGA  1535
m141031_iid=147540_0      ACACCAGGGCGTCTAAACCAAGCAAGAATATTCATCATTAATCCAGGACTAATATACGGA  1877
m150507_iid=3674924_0     ACACCAGGGCGTCTAAACCAAGCAAGAATATTCATCATTAATCCAGGACTAATATACGGA  2024
m150503_iid=2923751_0     ACACCAGGGCGTCTAAACCAAGCAAGAATATTCATCATTAATCCAGGACTAATATACGGA  1874
m141031_iid=115118_0      ACACCAGGGCGTCTAAACCAAGCAAGAATATTCATCATTAATCCAGGACTAATATACGGA  721
m150430_iid=2722448_0     ACACCAGGGCGTCTAAACCAAGCAAGAATATTCATCATTAATCCAGGACTAATATACGGA  670
m150507_iid=3102647_0     ------------------------------------------------------------  0
m141108_iid=1811866_0     ACACCAGGGCGTCTAAACCAAGCAAGAATATTCATCATTAATCCAGGACTAATATACGGA  851
m150501_iid=2948495_0     ACACCAGGGCGTCTAAACCAAGCAAGAATATTCATCATTAATCCAGGACTAATATACGGA  265
m150504_iid=3238195_0     ACACCAGGGCGTCTAAACCAAGCAAGAATATTCATCATTAATCCAGGACTAATATACGGA  2595
m150503_iid=3388511_0     ACACCAGGGCGTCTAAACCAAGCAAGAATATTCATCATTAATCCAGGACTAATATACGGA  295
m150505_iid=3777611_0     ACACCAGGGCGTCTAAACCAAGCAAGAATATTCATCATTAATCCAGGACTAATATACGGA  1712
m150430_iid=2438833_0     ACACCAGGGCGTCTAAACCAAGCAAGAATATTCATCATTAATCCAGGACTAATATACGGA  2062
m141108_iid=1698969_0     ACACCAGGGCGTCTAAACCAAGCAAGAATATTCATCATTAATCCAGGACTAATATACGGA  3181
m150430_iid=3059190_0     ------------------------------------------------------------  0
m141107_iid=1383067_0     ------------------------------------------------------------  0
```

```
m141101_iid=597008_0       ------------------------------------------------------------   0
m150506_iid=2596496_0      ACACCAGGGCGTCTAAACCAAGCAAGAATATTCATCATTAATCCAGGACTAATATACGGA   3537
m141102_iid=910051_0       ACACCAGGGCGTCTAAACCAAGCAAGAATATTCATCATTAATCCAGGACTAATATACGGA   3027
m150505_iid=3784017_0      ACACCAGGGCGTCTAAACCAAGCAAGAATATTCATCATTAATCCAGGACTAATATACGGA   1826
m141101_iid=682151_0       ACACCAGGGCGTCTAAACCAAGCAAGAATATTCATCATTAATCCAGGACTAATATACGGA   896
m150501_iid=3748029_0      ACACCAGGGCGTCTAAACCAAGCAAGAATATTCATCATTAATCCAGGACTAATATACGGA   1439
m150504_iid=2876272_0      ACACCAGGGCGTCTAAACCAAGCAAGAATATTCATCATTAATCCAGGACTAATATACGGA   3301
NC_042820.1                ACACCAGGGCGTCTAAACCAAGCAAGAATATTCATCATTAATCCAGGACTAATATACGGA   3544
m141102_iid=1228825_0      ACACCAGGGCGTCTAAACCAAGCAAGAATATTCATCATTAATCCAGGACTAATATACGGA   3371
m150504_iid=2876274_0      ACACCAGGGCGTCTAAACCAAGCAAGAATATTCATCATTAATCCAGGACTAATATACGGA   3374
m141030_iid=80036_0        ACACCAGGGCGTCTAAACCAAGCAAGAATATTCATCATTAATCCAGGACTAATATACGGA   3138
m150506_iid=2596494_0      ACACCAGGGCGTCTAAACCAAGCAAGAATATTCATCATTAATCCAGGACTAATATACGGA   3539
m150503_iid=2526862_0      ACACCAGGGCGTCTAAACCAAGCAAGAATATTCATCATTAATCCAGGACTAATATACGGA   3223
m150506_iid=2567000_0      ACACCAGGGCGTCTAAACCAAGCAAGAATATTCATCATTAATCCAGGACTAATATACGGA   1172
m150507_iid=3073271_0      ACACCAGGGCGTCTAAACCAAGCAAGAATATTCATCATTAATCCAGGACTAATATACGGA   2476
m150504_iid=3156087_0      ACACCAGGGCGTCTAAACCAAGCAAGAATATTCATCATTAATCCAGGACTAATATACGGA   2299
m150505_iid=2498502_0      ------------------------------------------------------------   0
m141102_iid=754227_0       ACACCAGGGCGTCTAAACCAAGCAAGAATATTCATCATTAATCCAGGACTAATATACGGA   2511
m150507_iid=3469514_0      ACACCAGGGCGTCTAAACCAAGCAAGAATATTCATCATTAATCCAGGACTAATATACGGA   3069
Rdo_MitoGenome             ACACCAGGGCGTCTAAACCAAGCAAGAATATTCATCATTAATCCAGGACTAATATACGGA   3544
m141101_iid=614933_0       ACACCAGGGCGTCTAAACCAAGCAAGAATATTCATCATTAATCCAGGACTAATATACGGA   2096
```

cox2

```
m141031_iid=347546_0       ------------------------------------------------------------   0
m150506_iid=3127414_0      CAATGTTCAGAAATCTGTGGAGCAAATCATAGATTT-TACCAATTGTTGTAGAAAGAATT   97
m141102_iid=1245517_0      CAATGTTCAGAAATCTGTGGAGCAAATCATAGATTTATACCAATTGTTGTAGAAAGAATT   1734
m141107_iid=1405727_0      ------------------------------------------------------------   0
m141102_iid=987614_0       CAATGTTCAGAAATCTGTGGAGCAAATCATAGATTTATACCAATTGTTGTAGAAAGAATT   1873
m141108_iid=1701429_0      ------------------------------------------------------------   0
m141102_iid=987286_0       ------------------------------------------------------------   0
m150430_iid=3272125_0      CAATGTTCAGAAATCTGTGAG--AAATCATAGATTTATACCAATTGTTGTAGAAAGAATT   1349
m141102_iid=754225_0       CAATGTTCAGAAATCTGTGGAGCAAATCATAGATTTATACCAATTGTTGTAGAAAGAATT   2521
m141107_iid=1409934_0      CAATGTTCAGAAATCTGTGGAGCAAATCATAGATTTATACCAATTGTTGTAGAAAGAATT   3050
m150503_iid=3416920_0      CAATGTTCAGAAATCTGTGGAGCAAATCATAGATTTATACCAATTGTTGTAGAAAGAATT   1787
m141108_iid=1800121_0      CAATGTTCAGAAATCTGTGGAGCAAATCATAGATTTATACCAATTGTTGTAGAAAGAATT   1595
m141031_iid=147540_0       CAATGTTCAGAAATCTGTGGAGCAAATCATAGATTTATACCAATTGTTGTAGAAAGAATT   1937
m150507_iid=3674924_0      CAATGTTCAGAAATCTGTGGAGCAAATCATAGATTTATACCAATTGTTGTAGAAAGAATT   2084
m150503_iid=2923751_0      CAATGTTCAGAAATCTGTGGAGCAAATCATAGATTTATACCAATTGTTGTAGAAAGAATT   1934
m141031_iid=115118_0       CAATGTTCAGAAATCTGTGGAGCAAATCATAGATTTATACCAATTGTTGTAGAAAGAATT   781
m150430_iid=2722448_0      CAATGTTCAGA-ATCTGTGGAGCAAATCATAGATTTATACCAATTGTTGTAGAAAGAATT   729
m150507_iid=3102647_0      ------------------------------------------------------------   0
m141108_iid=1811866_0      CAATGTTCAGAAATCTGTGGAGCAAATCATAGATTTATACCAATTGTTGTAGAAAGAATT   911
m150501_iid=2948495_0      CAATGTTCAGAAATCTGTGGAGCAAATCATAGATTTATACCAATTGTTGTAGAAAGAATT   325
m150504_iid=3238195_0      CAATGTTCAGAAATCTGTGGAGCAAATCATAGATTTATACCAATTGTTGTAGAAAGAATT   2655
m150503_iid=3388511_0      CAATGTTCAGAAATCTGTGGAGCAAATCATAGATTTATACCAATTGTTGTAGAAAGAATT   355
m150505_iid=3777611_0      CAATGTTCAGAAATCTGTGGAGCAAATCATAGATTTATACCAATTGTTGTAGAAAGAATT   1772
m150430_iid=2438833_0      CAATGTTCAGAAATCTGTGGAGCAAATCATAGATTTATACCAATTGTTGTAGAAAGAATT   2122
m141108_iid=1698969_0      CAATGTTCAGAAATCTGTGGAGCAAATCATAGATTTATACCAATTGTTGTAGAAAGAATT   3241
m150430_iid=3059190_0      ------------------------------------------------------------   0
m141107_iid=1383067_0      ------------------------------------------------------------   0
m141101_iid=597008_0       ------------------------------------------------------------   0
m150506_iid=2596496_0      CAATGTTCAGAAATCTGTGGAGCAAATCATAGATTTATACCAATTGTTGTAGAAAGAATT   3597
m141102_iid=910051_0       CAATGTTCAGAAATCTGTGGAGCAAATCATAGATTTATACCAATTGTTGTAGAAAGAATT   3087
m150505_iid=3784017_0      CAATGTTCAGAAATCTGTGGAGCAAATCATAGATTTATACCAATTGTTGTAGAAAGAATT   1886
m141101_iid=682151_0       CAATGTTCAGAAATCTGTGGAGCAAATCATAGATTTATACCAATTGTTGTAGAAAGAATT   956
m150501_iid=3748029_0      CAATGTTCAGAAATCTGTGGAGCAAATCATAGATTTATACCAATTGTTGTAGAAAGAATT   1499
m150504_iid=2876272_0      CAATGTTCAGAAATCTGTGGAGCAAATCATAGATTTATACCAATTGTTGTAGAAAGAATT   3361
NC_042820.1                CAATGTTCAGAAATCTGTGGAGCAAATCATAGATTTATACCAATTGTTGTAGAAAGAATT   3604
m141102_iid=1228825_0      CAATGTTCAGAAATCTGTGGAGCAAATCATAGATTTATACCAATTGTTGTAGAAAGAATT   3431
m150504_iid=2876274_0      CAATGTTCAGAAATCTGTGGAGCAAATCATAGATTTATACCAATTGTTGTAGAAAGAATT   3434
m141030_iid=80036_0        CAATGTTCAGAAATCTGTGGAGCAAATCATAGATTTATACCAATTGTTGTAGAAAGAATT   3198
m150506_iid=2596494_0      CAATGTTCAGAAATCTGTGGAGCAAATCATAGATTTATACCAATTGTTGTAGAAAGAATT   3599
m150503_iid=2526862_0      CAATGTTCAGAAATCTGTGGAGCAAATCATAGATTTATACCAATTGTTGTAGAAAGAATT   3283
m150506_iid=2567000_0      CAATGTTCAGAAATCTGTGGAGCAAATCATAGATTTATACCAATTGTTGTAGAAAGAATT   1232
m150507_iid=3073271_0      CAATGTTCAGAAATCTGTGGAGCAAATCATAGATTTATACCAATTGTTGTAGAAAGAATT   2536
m150504_iid=3156087_0      CAATGTTCAGAAATCTGTGGAGCAAATCATAGATTTATACCAATTGTTGTAGAAAGAATT   2359
m150505_iid=2498502_0      -------------------------------------------------TAGAAAGAATT   11
m141102_iid=754227_0       CAATGTTCAGAAATCTGTGGAGCAAATCATAGATTTATACCAATTGTTGTAGAAAGAATT   2571
m150507_iid=3469514_0      CAATGTTCAGAAATCTGTGGAGCAAATCATAGATTTATACCAATTGTTGTAGAAAGAATT   3129
Rdo_MitoGenome             CAATGTTCAGAAATCTGTGGAGCAAATCATAGATTTATACCAATTGTTGTAGAAAGAATT   3604
m141101_iid=614933_0       CAATGTTCAGAAATCTGTGGAGCAAATCATAGATTTATACCAATTGTTGTAGAAAGAATT   2156
```

cox2

```
m141031_iid=347546_0      ------------------------------------------------------------   0
m150506_iid=3127414_0     ACT-CTAATAAATTTATTGAGTGAATTAAAAACTCATTAGATAACTAAAAGTTAGTAATG   156
m141102_iid=1245517_0     ACTCCTAATAAATTTATTGAGTGAATTAAAAACTCATTAGATAACTAAAAGTTAGTAATG   1794
m141107_iid=1405727_0     ------------------------------------------------------------   0
m141102_iid=987614_0      ACTCCTAATAAATTTATTGAGTGAATTAAAAACTCATTAGATAACTAAAAGTTAGTAATG   1933
m141108_iid=1701429_0     ------------------------------------------------------------   0
m141102_iid=987286_0      ------------------------------------------------------------   0
m150430_iid=3272125_0     ACTCCTAATAAATTTATTGAGTGAATTAAAAACTCATTAGATAACTAAAAGTTAGTAATG   1409
m141102_iid=754225_0      ACTCCTAATAAATTTATTGAGTGAATTAAAAACTCATTAGATAACTAAAAGTTAGTAATG   2581
m141107_iid=1409934_0     ACTCCTAATAAATTTATTGAGTGAATTAAAAACTCATTAGATAACTAAAAGTTAGTAATG   3110
m150503_iid=3416920_0     ACTCCTAATAAATTTATTGAGTGAATTAAAAACTCATTAGATAACTAAAAGTTAGTAATG   1847
m141108_iid=1800121_0     ACTCCTAATAAATTTATTGAGTGAATTAAAAACTCATTAGATAACTAAAAGTTAGTAATG   1655
m141031_iid=147540_0      ACTCCTAATAAATTTATTGAGTGAATTAAAAACTCATTAGATAACTAAAAGTTAGTAATG   1997
m150507_iid=3674924_0     ACTCCTAATAAATTTATTGAGTGAATTAAAAACTCATTAGATAACTAAAAGTTAGTAATG   2144
m150503_iid=2923751_0     ACTCCTAATAAATTTATTGAGTGAATTAAAAACTCATTAGATAACTAAAAGTTAGTAATG   1994
m141031_iid=115118_0      ACTCCTAATAAATTTATTGAGTGAATTAAAAACTCATTAGATAACTAAAAGTTAGTAATG   841
m150430_iid=2722448_0     ACTCCTAATAAATTTATTGAGTGAATTAAAAACTCATTAGATAACTAAAAGTTAGTAATG   789
m150507_iid=3102647_0     ------------------------------------------------------------   0
m141108_iid=1811866_0     ACTCCTAATAAATTTATTGAGTGAATTAAAAACTCATTAGATAACTAAAAGTTAGTAATG   971
m150501_iid=2948495_0     ACTCCTAATAAATTTATTGAGTGAATTAAAAACTCATTAGATAACTAAAAGTTAGTAATG   385
m150504_iid=3238195_0     ACTCCTAATAAATTTATTGAGTGAATTAAAAACTCATTAGATAACTAAAAGTTAGTAATG   2715
m150503_iid=3388511_0     ACTCCTAATAAATTTATTGAGTGAATTAAAAACTCATTAGATAACTAAAAGTTAGTAATG   415
m150505_iid=3777611_0     ACTCCTAATAAATTTATTGAGTGAATTAAAAACTCATTAGATAACTAAAAGTTAGTAATG   1832
m150430_iid=2438833_0     ACTCCTAATAAATTTATTGAGTGAATTAAAAACTCATTAGATAACTAAAAGTTAGTAATG   2182
m141108_iid=1698969_0     ACTCCTAATAAATTTATTGAGTGAATTAAAAACTCATTAGATAACTAAAAGTTAGTAATG   3301
m150430_iid=3059190_0     ------------------------------------------------------------   0
m141107_iid=1383067_0     ------------------------------------------------------------   0
m141101_iid=597008_0      ------------------------------------------------------------   0
m150506_iid=2596496_0     ACTCCTAATAAATTTATTGAGTGAATTAAAAACTCATTAGATAACTAAAAGTTAGTAATG   3657
m141102_iid=910051_0      ACTCCTAATAAATTTATTGAGTGAATTAAAAACTCATTAGATAACTAAAAGTTAGTAATG   3147
m150505_iid=3784017_0     ACTCCTAATAAATTTATTGAGTGAATTAAAAACTCATTAGATAACTAAAAGTTAGTAATG   1946
m141101_iid=682151_0      ACTCCTAATAAATTTATTGAGTGAATTAAAAACTCATTAGATAACTAAAAGTTAGTAATG   1016
m150501_iid=3748029_0     ACTCCTAATAAATTTATTGAGTGAATTAAAAACTCATTAGATAACTAAAAGTTAGTAATG   1559
m150504_iid=2876272_0     ACTCCTAATAAATTTATTGAGTGAATTAAAAACTCATTAGATAACTAAAAGTTAGTAATG   3421
NC_042820.1               ACTCCTAATAAATTTATTGAGTGAATTAAAAACTCATTAGATAACTAAAAGTTAGTAATG   3664
m141102_iid=1228825_0     ACTCCTAATAAATTTATTGAGTGAATTAAAAACTCATTAGATAACTAAAAGTTAGTAATG   3491
m150504_iid=2876274_0     ACTCCTAATAAATTTATTGAGTGAATTAAAAACTCATTAGATAACTAAAAGTTAGTAATG   3494
m141030_iid=80036_0       ACTCCTAATAAATTTATTGAGTGAATTAAAAACTCATTAGATAACTAAAAGTTAGTAATG   3258
m150506_iid=2596494_0     ACTCCTAATAAATTTATTGAGTGAATTAAAAACTCATTAGATAACTAAAAGTTAGTAATG   3659
m150503_iid=2526862_0     ACTCCTAATAAATTTATTGAGTGAATTAAAAACTCATTAGATAACTAAAAGTTAGTAATG   3343
m150506_iid=2567000_0     ACTCCTAATAAATTTATTGAGTGAATTAAAAACTCATTAGATAACTAAAAGTTAGTAATG   1292
m150507_iid=3073271_0     ACTCCTAATAAATTTATTGAGTGAATTAAAAACTCATTAGATAACTAAAAGTTAGTAATG   2596
m150504_iid=3156087_0     ACTCCTAATAAATTTATTGAGTGAATTAAAAACTCATTAGATAACTAAAAGTTAGTAATG   2419
m150505_iid=2498502_0     ACTCCTAATAAATTTATTGAGTGAATTAAAAACTCATTAGATAACTAAAAGTTAGTAATG   71
m141102_iid=754227_0      ACTCCTAATAAATTTATTGAGTGAATTAAAAACTCATTAGATAACTAAAAGTTAGTAATG   2631
m150507_iid=3469514_0     ACTCCTAATAAATTTATTGAGTGAATTAAAAACTCATTAGATAACTAAAAGTTAGTAATG   3189
Rdo_MitoGenome            ACTCCTAATAAATTTATTGAGTGAATTAAAAACTCATTAGATAACTAAAAGTTAGTAATG   3664
m141101_iid=614933_0      ACTCCTAATAAATTTATTGAGTGAATTAAAAACTCATTAGATAACTAAAAGTTAGTAATG   2216
```

cox2

```
m141031_iid=347546_0      ------------------------------------------------------------   0
m150506_iid=3127414_0     GT-TCTTAAACAAAATAGTAGAGTAACGATTACTTACTTCTAATGAAAAATTTAGTTAA-   214
m141102_iid=1245517_0     GTCTCTTAAACCAAAAAATAGTAGAGTAACGAC-TACTTCTAATGAAAAATTTAGTTAAA   1853
m141107_iid=1405727_0     ------------------------------------------------------------   0
m141102_iid=987614_0      GTCTCTTAAACCAAAAAATAGTAGAGTAACGAC-TACTTCTAATGAAAAATTTAGTTAA-   1991
m141108_iid=1701429_0     ------------------------------------------------------------   0
m141102_iid=987286_0      ------------------------------------------------------------   0
m150430_iid=3272125_0     GTCTCTTAAACCAAAAAATAGTAGAGTAACGAC-TACTTCTAATGAAAAATTTAGTTAAA   1468
m141102_iid=754225_0      GTCTCTTAAACCAAAAAATAGTAGAGTAACGAC-TACTTCTAATGAAAAATTTAGTTAA-   2639
m141107_iid=1409934_0     GTCTCTTAAACCAAAAAATAGTAGAGTAACGAC-TACTTCTAATGAAAAATTTAGTTAA-   3168
m150503_iid=3416920_0     GTCTCTTAAACCAAAAAATAGTAGAGTAACGAC-TACTTCTAATGAAAAATTTAGTTAA-   1905
m141108_iid=1800121_0     GTCTCTTAAACA-AAAAATAGTAGAGTAACGAC-TACTTCTAATGAAAAATTTAGTTAA-   1712
m141031_iid=147540_0      GTCTCTTAAACCAAAAAATAGTAGAGTAACGAC-TACTTCTAATGAAAAATTTAGTTAA-   2055
m150507_iid=3674924_0     GTCTCTTAAACCAAAAAATAGTAGAGTAACGAC-TACTTCTAATGAAAAATTTAGTTAA-   2202
m150503_iid=2923751_0     GTCTCTTAAACCAAAAAATAGTAGAGTAACGAC-TACTTCTAATGAAAAATTTAGTTAA-   2052
m141031_iid=115118_0      GTCTCTTAAACCAAAAAATAGTAGAGTAACGAC-TACTTCTAATGAAAAATTTAGTTAA-   899
m150430_iid=2722448_0     GTCTCTTAAACCAAAAAATAGTAGAGTAACGAC-TACTTCTAATGAAAAATTTAGTTAA-   847
m150507_iid=3102647_0     ------------------------------------------------------------   0
m141108_iid=1811866_0     GTCTCTTAAACCAAAAAATAGTAGAGTAACGAC-TACTTCTAATGAAAAATTTAGTTAA-   1029
```

```
m150501_iid=2948495_0      GTCTCTTAAACCAAAAAATAGTAGAGTAACGAC-TACTTCTAATGAAAAATTTAGTTAA-   443
m150504_iid=3238195_0      GTCTCTTAAACCAAAAAATAGTAGAGTAACGAC-TACTTCTAATGAAAAATTTAGTTAA-   2773
m150503_iid=3388511_0      GTCTCTTAAACCAAAAAATAGTAGAGTAACGAC-TACTTCTAATGAAAAATTTAGTTAA-   473
m150505_iid=3777611_0      GTCTCTTAAACCAAAAAATAGTAGAGTAACGAC-TACTTCTAATGAAAAATTTAGTTAA-   1890
m150430_iid=2438833_0      GTCTCTTAAACCAAAAAATAGTAGAGTAACGAC-TACTTCTAATGAAAAATTTAGTTAA-   2240
m141108_iid=1698969_0      GTCTCTTAAACCAAAAAATAGTAGAGTAACGAC-TACTTCTAATGAAAAATTTAGTTAA-   3359
m150430_iid=3059190_0      ------------------------------------------------------------   0
m141107_iid=1383067_0      ------------------------------------------------------------   0
m141101_iid=597008_0       ------------------------------------------------------------   0
m150506_iid=2596496_0      GTCTCTTAAACCAAAAAATAGTAGAGTAACGAC-TACTTCTAATGAAAAATTTAGTTAA-   3715
m141102_iid=910051_0       GTCTCTTAAACCAAAAAATAGTAGAGTAACGAC-TACTTCTAATGAAAAATTTAGTTAA-   3205
m150505_iid=3784017_0      GTCTCTTAAACCAAAAAATAGTAGAGTAACGAC-TACTTCTAATGAAAAATTTAGTTAA-   2004
m141101_iid=682151_0       GTCTCTTAAACCAAAAAATAGTAGAGTAACGAC-TACTTCTAATGAAAAATTTAGTTAA-   1074
m150501_iid=3748029_0      GTCTCTTAAACCAAAAAATAGTAGAGTAACGAC-TACTTCTAATGAAAAATTTAGTTAA-   1617
m150504_iid=2876272_0      GTCTCTTAAACCAAAAAATAGTAGAGTAACGAC-TACTTCTAATGAAAAATTTAGTTAA-   3479
NC_042820.1                GTCTCTTAAACCAAAAAATAGTAGAGTAACGAC-TACTTCTAATGAAAAATTTAGTTAA-   3722
m141102_iid=1228825_0      GTCTCTTAAACCAAAAAATAGTAGAGTAACGAC-TACTTCTAATGAAAAATTTAGTTAA-   3549
m150504_iid=2876274_0      GTCTCTTAAACCAAAAAATAGTAGAGTAACGAC-TACTTCTAATGAAAAATTTAGTTAA-   3552
m141030_iid=80036_0        GTCTCTTAAACCAAAAAATAGTAGAGTAACGAC-TACTTCTAATGAAAAATTTAGTTAA-   3316
m150506_iid=2596494_0      GTCTCTTAAACCAAAAAATAGTAGAGTAACGAC-TACTTCTAATGAAAAATTTAGTTAA-   3717
m150503_iid=2526862_0      GTCTCTTAAACCAAAAAATAGTAGAGTAACGAC-TACTTCTAATGAAAAATTTAGTTAA-   3401
m150506_iid=2567000_0      GTCTCTTAAACCAAAAAATAGTAGAGTAACGAC-TACTTCTAATGAAAAATTTAGTTAA-   1350
m150507_iid=3073271_0      GTCTCTTAAACCAAAAAATAGTAGAGTAACGAC-TACTTCTAATGAAAAATTTAGTTAA-   2654
m150504_iid=3156087_0      GTCTCTTAAACCAAAAAATAGTAGAGTAACGAC-TACTTCTAATGAAAAATTTAGTTAA-   2477
m150505_iid=2498502_0      GTCTCTTAAACCAAAAAATAGTAGAGTAACGAC-TACTTCTAATGAAAAATTTAGTTAA-   129
m141102_iid=754227_0       GTCTCTTAAACCAAAAAATAGTAGAGTAACGAC-TACTTCTAATGAAAAATTTAGTTAA-   2689
m150507_iid=3469514_0      GTCTCTTAAACCAAAAAATAGTAGAGTAACGAC-TACTTCTAATGAAAAATTTAGTTAA-   3247
Rdo_MitoGenome             GTCTCTTAAACCAAAAAATAGTAGAGTAACGAC-TACTTCTAATGAAAAATTTAGTTAA-   3722
m141101_iid=614933_0       GTCTCTTAAACCAAAAAATAGTAGAGTAACGAC-TACTTCTAATGAAAAATTTAGTTAA-   2274


m141031_iid=347546_0       ------------------------------------------------------------   0
m150506_iid=3127414_0      AAAATAACATCAACTTGTC-AGTTGAAATTACGATTTCGTAATTTTTGATTCCCACAAAT   273
m141102_iid=1245517_0      --AATAACATCAAC-TGTCAAGTTGAAATTACGATTTCGTAATTTTTGATT-CCACAAAT   1909
m141107_iid=1405727_0      ------------------------------------------------------------   0
m141102_iid=987614_0       AAAATAACATCAACTTGTCAAGTTGAAATTACGATTTCGTAATTTTTGATT-CCACAAAT   2050
m141108_iid=1701429_0      ------------------------------------------------------------   0
m141102_iid=987286_0       -------------------------------------------TTTTGATT-CCACAAAT   16
m150430_iid=3272125_0      AAAATAACATCAACTTGTCAAGTTGAAATTACGATTTCGTAATTTTTGATT-CCACAAAT   1527
m141102_iid=754225_0       AAAATAACATCAACTTGTCAAGTTGAAATTACGATTTCGTAATTTTTGATT-CCACAAAT   2698
m141107_iid=1409934_0      AAAATAACATCAACTTGTCAAGTTGAAATTACGATTTCGTAATTTTTGATT-CCACAAAT   3227
m150503_iid=3416920_0      AAAATAACATCAACTTGTCAAGTTGAAATTACGATTTCGTAATTTTTGATT-CCACAAAT   1964
m141108_iid=1800121_0      AAAATAACATCAACTTGTCAAGTTGAAATTACGATTTCGTAATTTTTGATT-CCACAAAT   1771
m141031_iid=147540_0       AAAATAACATCAACTTGTCAAGTTGAAATTACGATTTCGTAATTTTTGATT-CCACAAAT   2114
m150507_iid=3674924_0      AAAATAACATCAACTTGTCAAGTTGAAATTACGATTTCGTAATTTTTGATT-CCACAAAT   2261
m150503_iid=2923751_0      AAAATAACATCAACTTGTCAAGTTGAAATTACGATTTCGTAATTTTTGATT-CCACAAAT   2111
m141031_iid=115118_0       AAAATAACATCAACTTGTCAAGTTGAAATTACGATTTCGTAATTTTTGATT-CCACAAAT   958
m150430_iid=2722448_0      AAAATAACATCAACTTGTCAAGTTGAAATTACGATTTCGTAATTTTTGATT-CCACAAAT   906
m150507_iid=3102647_0      ------------------------------------------------------------   0
m141108_iid=1811866_0      AAAATAACATCAACTTGTCAAGTTGAAATTACGATTTCGTAATTTTTGATT-CCACAAAT   1088
m150501_iid=2948495_0      AAAATAACATCAACTTGTCAAGTTGAAATTACGATTTCGTAATTTTTGATT-CCACAAAT   502
m150504_iid=3238195_0      AAAATAACATCAACTTGTCAAGTTGAAATTACGATTTCGTAATTTTTGATT-CCACAAAT   2832
m150503_iid=3388511_0      AAAATAACATCAACTTGTCAAGTTGAAATTACGATTTCGTAATTTTTGATT-CCACAAAT   532
m150505_iid=3777611_0      AAAATAACATCAACTTGTCAAGTTGAAATTACGATTTCGTAATTTTTGATT-CCACAAAT   1949
m150430_iid=2438833_0      AA---AACATCAACTTGTCAAGTTGAAATTACGATTTCGTAATTTTTGATT-CCACAAAT   2296
m141108_iid=1698969_0      AAAATAACATCAACTTGTCAAGTTGAAATTACGATTTCGTAATTTTTGATT-CCACAAAT   3418
m150430_iid=3059190_0      ------------------------------------------------------------   0
m141107_iid=1383067_0      ------------------------------------------------------------   0
m141101_iid=597008_0       ------------------------------------------------------------   0
m150506_iid=2596496_0      AAAATAACATCAACTTGTCAAGTTGAAATTACGATTTCGTAATTTTTGATT-CCACAAAT   3774
m141102_iid=910051_0       AAAATAACATCAACTTGTCAAGTTGAAATTACGATTTCGTAATTTTTGATT-CCACAAAT   3264
m150505_iid=3784017_0      AAAATAACATCAACTTGTCAAGTTGAAATTACGATTTCGTAATTTTTGATT-CCACAAAT   2063
m141101_iid=682151_0       AAAATAACATCAACTTGTCAAGTTGAAATTACGATTTCGTAATTTTTGATT-CCACAAAT   1133
m150501_iid=3748029_0      AAAA-AACATCAACTTGTCAAGTTGAAATTACGATTTCGTAATTTTTGATT-CCACAAAT   1675
m150504_iid=2876272_0      AAAATAACATCAACTTGTCAAGTTGAAATTACGATTTCGTAATTTTTGATT-CCACAAAT   3538
NC_042820.1                AAAATAACATCAACTTGTCAAGTTGAAATTACGATTTCGTAATTTTTGATT-CCACAAAT   3781
m141102_iid=1228825_0      AAAATAACATCAACTTGTCAAGTTGAAATTACGATTTCGTAATTTTTGATT-CCACAAAT   3608
m150504_iid=2876274_0      AAAATAACATCAACTTGTCAAGTTGAAATTACGATTTCGTAATTTTTGATT-CCACAAAT   3611
m141030_iid=80036_0        AAAATAACATCAACTTGTCAAGTTGAAATTACGATTTCGTAATTTTTGATT-CCACAAAT   3375
m150506_iid=2596494_0      AAAATAACATCAACTTGTCAAGTTGAAATTACGATTTCGTAATTTTTGATT-CCACAAAT   3776
m150503_iid=2526862_0      AAAATAACATCAACTTGTCAAGTTGAAATTACGATTTCGTAATTTTTGATT-CCACAAAT   3460
```

```
m150506_iid=2567000_0      AAAATAACATCAACTTGTCAAGTTGAAATTACGATTTCGTAATTTTTGATT-CCACAAAT   1409
m150507_iid=3073271_0      AAAATAACATCAACTTGTCAAGTTGAAATTACGATTTCGTAATTTTTGATT-CCACAAAT   2713
m150504_iid=3156087_0      AAAATAACATCAACTTGTCAAGTTGAAATTACGATTTCGTAATTTTTGATT-CCACAAAT   2536
m150505_iid=2498502_0      AAAATAACATCAACTTGTCAAGTTGAAATTACGATTTCGTAATTTTTGATT-CCACAAAT   188
m141102_iid=754227_0       AAAATAACATCAACTTGTCAAGTTGAAATTACGATTTCGTAATTTTTGATT-CCACAAAT   2748
m150507_iid=3469514_0      AAAATAACATCAACTTGTCAAGTTGAAATTACGATTTCGTAATTTTTGATT-CCACAAAT   3306
Rdo_MitoGenome             AAAATAACATCAACTTGTCAAGTTGAAATTACGATTTCGTAATTTTTGATT-CCACAAAT   3781
m141101_iid=614933_0       AAAATAACATCAACTTGTCAAGTTGAAATTACGATTTCGTAATTTTTGATT-CCACAAAT   2333

                                                                              atp8
m141031_iid=347546_0       ------------------------------------------------------------   0
m150506_iid=3127414_0      ATCTCCATTAAATTGAATAACCTTA-TTATTTTCTTTACAATTACTTTAGTAATCCTATC   332
m141102_iid=1245517_0      ATCTCCATTAAATTGAATAACCTTATTTATTTTCTTTACAATTACTTTAGTAATCATATC   1969
m141107_iid=1405727_0      ------------------------------------------------------------   0
m141102_iid=987614_0       ATCTCCATTAAATTGAATAACCTTATTTATTTTCTTTACAATTACTTTAGTAATCATATC   2110
m141108_iid=1701429_0      ------------------------------------------------------------   0
m141102_iid=987286_0       ATCTCCATTAAATTGAATAACCTTATT---TATCTTTACAATTACTTTAGTAATCAA-TC   72
m150430_iid=3272125_0      ATCTCCATTAAATTGAATAACCTTATTTTATTTCTTTACAATTACTTTAGTAATCATATC   1587
m141102_iid=754225_0       ATCTCCATTAAATTGAATAACCTTATTTATTTTCTTTACAATTACTTTAGTAATCATATC   2758
m141107_iid=1409934_0      ATCTCCATTAAATTGAATAACCTTATTTATTTTCTTTACAATTACTTTAGTAATCATATC   3287
m150503_iid=3416920_0      ATCTCCATTAAATTGAATAACCTTATTTATTTTCTTTACAATTACTTTAGTAATCATATC   2024
m141108_iid=1800121_0      ATCTCCATTAAATTGAATAACCTTATTTATTTTCTTTACAATTACTTTAGTAATCATATC   1831
m141031_iid=147540_0       ATCTCCATTAAATTGAATAACCTTATTTATTTTCTTTACAATTACTTTAGTAATCATATC   2174
m150507_iid=3674924_0      ATCTCCATTAAATTGAATAACCTTATTTATTTTCTTTACAATTACTTTAGTAATCATATC   2321
m150503_iid=2923751_0      ATCTCCATTAAATTGAATAACCTTATTTATTTTCTTTACAATTACTTTAGTAATCATATC   2171
m141031_iid=115118_0       ATCTCCATTAAATTGAATAACCTTATTTATTTTCTTTACAATTACTTTAGTAATCATATC   1018
m150430_iid=2722448_0      ATCTCCATTAAATTGAATAACCTTATTTATTTTCTTTACAATTACTTTAGTAATCATATC   966
m150507_iid=3102647_0      ------------------------------------------------------------   0
m141108_iid=1811866_0      ATCTCCATTAAATTGAATATTATT--------TC---TTTATTACTTTAGTAATCATATC   1137
m150501_iid=2948495_0      ATCTCCATTAAATTGAATAACCTTATTTATTTTCTTTACAATTACTTTAGTAATCATATC   562
m150504_iid=3238195_0      ATCTCCATTAAATTGAATAACCTTATTTATTTTCTTTACAATTACTTTAGTAATCATATC   2892
m150503_iid=3388511_0      ATCTCCATTAAATTGAATAACCTTATTTATTTTCTTTACAATTACTTTAGTAATCATATC   592
m150505_iid=3777611_0      ATCTCCATTAAATTGAATAACCTTATTTATTTTCTTTACAATTACTTTAGTAATCATATC   2009
m150430_iid=2438833_0      ATCTCCATTAAATTGAATAACCTTATTTATTTTCTTTACAATTACTTTAGTAATCATATC   2356
m141108_iid=1698969_0      ATCTCCATTAAATTGAATAACCTTATTTATTTTCTTTACAATTACTTTAGTAATCATATC   3478
m150430_iid=3059190_0      ------------------------------------------------------------   0
m141107_iid=1383067_0      ------------------------------------------------------------   0
m141101_iid=597008_0       ------------------------------------------------------------   0
m150506_iid=2596496_0      ATCTCCATTAAATTGAATAACCTTATTTATTTTCTTTACAATTACTTTAGTAATCATATC   3834
m141102_iid=910051_0       ATCTCCATTAAATTGAATAACCTTATTTATTTTCTTTACAATTACTTTAGTAATCATATC   3324
m150505_iid=3784017_0      ATCTCCATTAAATTGAATAACCTTATTTATTTTCTTTACAATTACTTTAGTAATCATATC   2123
m141101_iid=682151_0       ATCTCCATTAAATTGAATAACCTTATTTATTTTCTTTACAATTACTTTAGTAATCATATC   1193
m150501_iid=3748029_0      ATCTCCATTAAATTGAATAACCTTATTTATTTTCTTTACAATTACTTTAGTAATCATATC   1735
m150504_iid=2876272_0      ATCTCCATTAAATTGAATAACCTTATTTATTTTCTTTACAATTACTTTAGTAATCATATC   3598
NC_042820.1                ATCTCCATTAAATTGAACAACCTTATTTATTTTCTTTACAATTACTTTAGTAATCATATC   3841
m141102_iid=1228825_0      ATCTCCATTAAATTGAATAACCTTATTTATTTTCTTTACAATTACTTTAGTAATCATATC   3668
m150504_iid=2876274_0      ATCTCCATTAAATTGAATAACCTTATTTATTTTCTTTACAATTACTTTAGTAATCATATC   3671
m141030_iid=80036_0        ATCTCCATTAAATTGAATAACCTTATTTATTTTCTTTACAATTACTTTAGTAATCATATC   3435
m150506_iid=2596494_0      ATCTCCATTAAATTGAATAACCTTATTTATTTTCTTTACAATTACTTTAGTAATCATATC   3836
m150503_iid=2526862_0      ATCTCCATTAAATTGAATAACCTTATTTATTTTCTTTACAATTACTTTAGTAATCATATC   3520
m150506_iid=2567000_0      ATCTCCATTAAATTGAATAACCTTATTTATTTTCTTTACAATTACTTTAGTAATCATATC   1469
m150507_iid=3073271_0      ATCTCCATTAAATTGAATAACCTTATTTATTTTCTTTACAATTACTTTAGTAATCATATC   2773
m150504_iid=3156087_0      ATCTCCATTAAATTGAATAACCTTATTTATTTTCTTTACAATTACTTTAGTAATCATATC   2596
m150505_iid=2498502_0      ATCTCCATTAAATTGAATAACCTTATTTATTTTCTTTACAATTACTTTAGTAATCATATC   248
m141102_iid=754227_0       ATCTCCATTAAATTGAATAACCTTATTTATTTTCTTTACAATTACTTTAGTAATCATATC   2808
m150507_iid=3469514_0      ATCTCCATTAAATTGAATAACCTTATTTATTTTCTTTACAATTACTTTAGTAATCATATC   3366
Rdo_MitoGenome             ATCTCCATTAAATTGAATAACCTTATTTATTTTCTTTACAATTACTTTAGTAATCATATC   3841
m141101_iid=614933_0       ATCTCCATTAAATTGAATAACCTTATTTATTTTCTTTACAATTACTTTAGTAATCATATC   2393

                                                      atp8
m141031_iid=347546_0       ------------------------------------------------------------   0
m150506_iid=3127414_0      AACAATAAATTTTTATAATTACAAAACTGAACCCCTCAAAGGAGAAAAAACAATTTCTAT   392
m141102_iid=1245517_0      AACAATAAATTTTTATAATTACAAACCTGAACCCCTCAAAGGAGAAAAAACAATTTCTAT   2029
m141107_iid=1405727_0      ------------------------------------------------------------   0
m141102_iid=987614_0       AACAATAAATTTTTATAATTACAAACCTGAACCCCTCAAAGGAGAAAAAACAATTTCTAT   2170
m141108_iid=1701429_0      ------------------------------------------------------------   0
m141102_iid=987286_0       AACAATA-ATTTTTATAATTACAAACCTGAACCCCTA-AAGGAGAAAAAACAATTTCTAT   130
m150430_iid=3272125_0      AACAATAAATTTTTATAATTACAAACCTGAACCCCTCAAAGGAGAAAAAACAATTTCTAT   1647
m141102_iid=754225_0       AACAATAAATTTTTATAATTACAAACCTGAACCCCTCAAAGGAGAAAAAACAATTTCTAT   2818
m141107_iid=1409934_0      AACAATAAATTTTTATAATTACAAACCTGAACCCCTCAAAGGAGAAAAAACAATTTCTAT   3347
m150503_iid=3416920_0      AACAATAAATTTTTATAATTACAAACCTGAACCCCTCAAAGGAGAAAAA--AATTTCTAT   2082
```

```
m141108_iid=1800121_0       AACAATAAATTTTTATAATTACAAACCTGAACCCCTCAAAGGAGAAAAAACAATTTCTAT   1891
m141031_iid=147540_0        AACAATAAATTTTTATAATTACAAACCTGAACCCCTCAAAGGAGAAAAAACAATTTCTAT   2234
m150507_iid=3674924_0       AACAATAAATTTTTATAATTACAAACCTGAACCCCTCAAAGGAGAAAAAACAATTTCTAT   2381
m150503_iid=2923751_0       AACAATAAATTTTTATAATTACAAACCTGAACCCCTCAAAGGAGAAAAAACAATTTCTAT   2231
m141031_iid=115118_0        AACAATAAATTTTTATAATTACAAACCTGAACCCCTCAAAGGAGAAAAAACAATTTCTAT   1078
m150430_iid=2722448_0       AACAATAAATTTTTATAATTACAAACCTGAACCCCTCAAAGGAGAAAAAACAATTTCTAT   1026
m150507_iid=3102647_0       ------------------------------------------------------------   0
m141108_iid=1811866_0       AACAATAAATTTTTATAATTACAAACCTGAACCCCTCAAAGGAGAAAAAACAATTTCTAT   1197
m150501_iid=2948495_0       AACAATAAATTTTTATAATTACAAACCTGAACCCCTCAAAGGAGAAAAAACAATTTCTAT   622
m150504_iid=3238195_0       AACAATAAATTTTTATAATTACAAACCTGAACCCCTCAAAGGAGAAAAAACAATTTCTAT   2952
m150503_iid=3388511_0       AACAATAAATTTTTATAATTACAAACCTGAACCCCTCAAAGGAGAAAAAACAATTTCTAT   652
m150505_iid=3777611_0       AACAATAAATTTTTATAATTACAAACCTGAA-CCCTCAAAGGAGAAAAAACAATTTCTAT   2068
m150430_iid=2438833_0       AACAATAAATTTTTATAATTACAAACCTGAACCCCTCAAAGGAGAAAAAACAATTTCTAT   2416
m141108_iid=1698969_0       AACAATAAATTTTTATAATTACAAACCTGAACCCCTCAAAGGAGAAAAAACAATTTCTAT   3538
m150430_iid=3059190_0       ------------------------------------------------------------   0
m141107_iid=1383067_0       ------------------------------------------------------------   0
m141101_iid=597008_0        ------------------------------------------------------------   0
m150506_iid=2596496_0       AACAATAAATTTTTATAATTACAAACCTGAACCCCTCAAAGGAGAAAAAACAATTTCTAT   3894
m141102_iid=910051_0        AACAATAAATTTTTATAATTACAAACCTGAACCCCTCAAAGGAGAAAAAACAATTTCTAT   3384
m150505_iid=3784017_0       AACAATAAATTTTTATAATTACAAACCTGAACCCCTCAAAGGAGAAAAAAAATTTCTATA   2183
m141101_iid=682151_0        AACAATAAATTTTTATAATTACAAACCTGAACCCCTCAAAGGAGAAAAAACAATTTCTAT   1253
m150501_iid=3748029_0       AACAATAAATTTTTATAATTACAAACCTGAACCCCTCAAAGGAGAAAAAACAATTTCTAT   1795
m150504_iid=2876272_0       AACAATAAATTTTTATAATTACAAACCTGAACCCCTCAAAGGAGAAAAAACAATTTCTAT   3658
NC_042820.1                 AACAATAAATTTTTATAATTACAAACCTGAACCCCTCAAAGGAGAAAAAACAATTTCTAT   3901
m141102_iid=1228825_0       AACAATAAATTTTTATAATTACAAACCTGAACCCCTCAAAGGAGAAAAAACAATTTCTAT   3728
m150504_iid=2876274_0       AACAATAAATTTTTATAATTACAAACCTGAACCCCTCAAAGGAGAAAAAACAATTTCTAT   3731
m141030_iid=80036_0         AACAATAAATTTTTATAATTACAAACCTGAACCCCTCAAAGGAGAAAAAACAATTTCTAT   3495
m150506_iid=2596494_0       AACAATAAATTTTTATAATTACAAACCTGAACCCCTCAAAGGAGAAAAAACAATTTCTAT   3896
m150503_iid=2526862_0       AACAATAAATTTTTATAATTACAAACCTGAACCCCTCAAAGGAGAAAAAACAATTTCTAT   3580
m150506_iid=2567000_0       AACAATAAATTTTTATAATTACAAACCTGAACCCCTCAAAGGAGAAAAAACAATTTCTAT   1529
m150507_iid=3073271_0       AACAATAAATTTTTATAATTACAAACCTGAACCCCTCAAAGGAGAAAAAACAATTTCTAT   2833
m150504_iid=3156087_0       AACAATAAATTTTTATAATTACAAACCTGAACCCCTCAAAGGAGAAAAAACAATTTCTAT   2656
m150505_iid=2498502_0       AACAATAAATTTTTATAATTACAAACCTGAACCCCTCAAAGGAGAAAAAACAATTTCTAT   308
m141102_iid=754227_0        AACAATAAATTTTTATAATTACAAACCTGAACCCCTCAAAGGAGAAAAAACAATTTCTAT   2868
m150507_iid=3469514_0       AACAATAAATTTTTATAATTACAAACCTGAACCCCTCAAAGGAGAAAAAACAATTTCTAT   3426
Rdo_MitoGenome              AACAATAAATTTTTATAATTACAAACCTGAACCCCTCAAAGGAGAAAAAACAATTTCTAT   3901
m141101_iid=614933_0        AACAATAAATTTTTATAATTACAAACCTGAACCCCTCAAAGGAGAAAAAACAATTTCTAT   2453
```

atp8

```
m141031_iid=347546_0        ------------------------------------------------------------   0
m150506_iid=3127414_0       AAGAAAAAAAAACTG-AAATGATAAGAAATCTATTTTCATCATTTGATCCATCTACTCAA   451
m141102_iid=1245517_0       AAGAAAAA-AAAATGAAAATGATAAGAAATCTATTTTCATCATTTGATCCATCTACTCAA   2088
m141107_iid=1405727_0       ------------------------------------------------------------   0
m141102_iid=987614_0        AAGAAAAAAAAACTGAAAATGATAAGAAATCTATTTTCATCATTTGATCCATCTACTCAA   2230
m141108_iid=1701429_0       ------------------------------------------------------------   0
m141102_iid=987286_0        AAGAAA-AAAAACTGAAAATGATAAGAAATCTATTTTCATCATTTGATCCATCTACTCAA   189
m150430_iid=3272125_0       AAGAAAAAAAAACTGAAAATGATAAGAAATCTATTTTCATCATTTGATCCATCTACTCAA   1707
m141102_iid=754225_0        AAGAAA-AAAAACTGAAAATGATAAGAAATCTATTTTCATCATTTGATCCATCTACTCAA   2877
m141107_iid=1409934_0       AAGAAAAAAAAACTGAAAATGATAAGAAATCTATTTTCATCATTTGATCCATCTACTCAA   3407
m150503_iid=3416920_0       AAGAAAAAAAAACTGAAAATGATAAGAAATCTATTTTCATCATTTGATCCATCTACTCAA   2142
m141108_iid=1800121_0       AAGAAAAAAAAACTGAAAATGATAAGAAATCTATTTTCATCATTTGATCCATCTACTCAA   1951
m141031_iid=147540_0        AAG-AAAAAAAACTGAAAATGATAAGAAATCTATTTTCATCATTTGATCCATCTACTCAA   2293
m150507_iid=3674924_0       AAGAAAAAAAAACTGAAAATGATAAGAAATCTATTTTCATCATTTGATCCATCTACTCAA   2441
m150503_iid=2923751_0       AAGAAAAAAAAACTGAAAATGATAAGAAATCTATTTTCATCATTTGATCCATCTACTCAA   2291
m141031_iid=115118_0        AAGAAAAAAAAACTGAAAATGATAAGAAATCTATTTTCATCATTTGATCCATCTACTCAA   1138
m150430_iid=2722448_0       AAGAAAAAAAAACTGAAAATGATAAGAAATCTATTTTCATCATTTGATCCATCTACTCAA   1086
m150507_iid=3102647_0       ------------------------------------------------------------   0
m141108_iid=1811866_0       AAGAAAAAAAAACTGAAAATGATAAGAAATCTATTTTCATCATTTGATCCATCTACTCAA   1257
m150501_iid=2948495_0       AAGAAAAAAAAACTGAAAATGATAAGAAATCTATTTTCATCATTTGATCCATCTACTCAA   682
m150504_iid=3238195_0       AAGAAAAAAAAACTGAAAATGATAAGAAATCTATTTTCATCATTTGATCCATCTACTCAA   3012
m150503_iid=3388511_0       AAGAAAAAAAAACTGAAAATGATAAGAAATCTATTTTCATCATTTGATCCATCTACTCAA   712
m150505_iid=3777611_0       AAG-AAAAAAAACTGAAAATGATAAGAAATCTATTTTCATCATTTGATCCATCTACTCAA   2127
m150430_iid=2438833_0       AAGAAAAAAAAACTGAAAATGATAAGAAATCTATTTTCATCATTTGATCCATCTACTCAA   2476
m141108_iid=1698969_0       AAGAAAAAAAAACTGAAAATGATAAGAAATCTATTTTCATCATTTGATCCATCTACTCAA   3598
m150430_iid=3059190_0       ------------------------------------------------------------   0
m141107_iid=1383067_0       ------------------------------------------------------------   0
m141101_iid=597008_0        ------------------------------------------------------------   0
m150506_iid=2596496_0       AAGAAAAAAAAACTGAAAATGATAAGAAATCTATTTTCATCATTTGATCCATCTACTCAA   3954
m141102_iid=910051_0        AAGAAAAAAAAACTGAAAATGATAAGAAATCTATTTTCATCATTTGATCCATCTACTCAA   3444
m150505_iid=3784017_0       AGAAAAAAAAAACTGAAAATGATAAGAAATCTATTTTCATCATTTGATCCATCTACTCAA   2243
m141101_iid=682151_0        AAGAAAAAAAAACTGAAAATGATAAGAAATCTATTTTCATCATTTGATCCATCTACTCAA   1313
```

```
m150501_iid=3748029_0      AAG-AAAAAAAACTGAAAATGATAAGAAATCTATTTTCATCATTTGATCCATCTACTCAA   1854
m150504_iid=2876272_0      AAGAAAAAAAAACTGAAAATGATAAGAAATCTATTTTCATCATTTGATCCATCTACTCAA   3718
NC_042820.1                AAGAAAAAAAAACTGAAAATGATAAGAAATCTATTTTCATCATTTGATCCATCTACTCAA   3961
m141102_iid=1228825_0      AAGAAAAAAAAACTGAAAATGATAAGAAATCTATTTTCATCATTTGATCCATCTACTCAA   3788
m150504_iid=2876274_0      AAGAAAAAAAAACTGAAAATGATAAGAAATCTATTTTCATCATTTGATCCATCTACTCAA   3791
m141030_iid=80036_0        AAGAAAAAAAAACTGAAAATGATAAGAAATCTATTTTCATCATTTGATCCATCTACTCAA   3555
m150506_iid=2596494_0      AAGAAAAAAAAACTGAAAATGATAAGAAATCTATTTTCATCATTTGATCCATCTACTCAA   3956
m150503_iid=2526862_0      AAGAAAAAAAAACTGAAAATGATAAGAAATCTATTTTCATCATTTGATCCATCTACTCAA   3640
m150506_iid=2567000_0      AAGAAAAAAAAACTGAAAATGATAAGAAATCTATTTTCATCATTTGATCCATCTACTCAA   1589
m150507_iid=3073271_0      AAGAAAAAAAAACTGAAAATGATAAGAAATCTATTTTCATCATTTGATCCATCTACTCAA   2893
m150504_iid=3156087_0      AAGAAAAAAAAACTGAAAATGATAAGAAATCTATTTTCATCATTTGATCCATCTACTCAA   2716
m150505_iid=2498502_0      AAGAAA-AAAAACTGAAAATGATAAGAAATCTATTTTCATCATTTGATCCATCTACTCAA   367
m141102_iid=754227_0       AAGAAAAAAAAACTGAAAATGATAAGAAATCTATTTTCATCATTTGATCCATCTACTCAA   2928
m150507_iid=3469514_0      AAGAAAAAAAAACTGAAAATGATAAGAAATCTATTTTCATCATTTGATCCATCTACTCAA   3486
Rdo_MitoGenome             AAGAAAAAAAAACTGAAAATGATAAGAAATCTATTTTCATCATTTGATCCATCTACTCAA   3961
m141101_iid=614933_0       AAGAAAAAAAAACTGAAAATGATAAGAAATCTATTTTCATCATTTGATCCATCTACTCAA   2513
```

atp8 *atp6*

```
m141031_iid=347546_0       ------------------------------------------------------------   0
m150506_iid=3127414_0      ATTCTCT-CTTAAATTGATTAAGATCATTAATTATCCTTATAATTATACCAATAAATTTT   510
m141102_iid=1245517_0      ATTCTCTCCTTAAATTGATTAAGATCATTAATTATCCTTATAATTATACCAATAAATTTT   2148
m141107_iid=1405727_0      ------------------------------------------------------------   0
m141102_iid=987614_0       ATTCTCTCCTTAAATTGATTAAGATCATTAATTATCCTTATAATTATACCAATAAATTTT   2290
m141108_iid=1701429_0      ------------------------------------------------------------   0
m141102_iid=987286_0       ATTCTCTCCTTAA-ATGATTAAGATCATTAATTATCCTTATAATTATACCAATAAATTTT   248
m150430_iid=3272125_0      ATTCTCTCCTTAAATTGATTAAGATCATTAATTATCCTTATAATTATACCAATAAATTTT   1767
m141102_iid=754225_0       ATTCTCTCCTTAAATTGATTAAGATCATTAATTATCCTTATAATTATACCAATAAATTTT   2937
m141107_iid=1409934_0      ATTCTCTCCTTAAATTGATTAAGATCATTAATTATCCTTATAATTATACCAATAAATTTT   3467
m150503_iid=3416920_0      ATTCTCTCCTTAAATTGATTAAGATCATTAATTATCCTTATAATTATACCAATAAATTTT   2202
m141108_iid=1800121_0      ATTCTCTCCTTAAATTGATTAAGATCATTAATTATCCTTATAATTATACCAATAAATTTT   2011
m141031_iid=147540_0       ATTCTCTCCTTAAATTGATTAAGATCATTAATTATCCTTATAATTATACCAATAAATTTT   2353
m150507_iid=3674924_0      ATTCTCTCCTTAAATTGATTAAGATCATTAATTATCCTTATAATTATACCAATAAATTTT   2501
m150503_iid=2923751_0      ATTCTCTCCTTAAATTGATTAAGATCATTAATTATCCTTATAATTATACCAATAAATTTT   2351
m141031_iid=115118_0       ATTCTCTCCTTAAATTGATTAAGATCATTAATTATCCTTATAATTATACCAATAAATTTT   1198
m150430_iid=2722448_0      ATTCTCTCCTTAAATTGATTAAGATCATTAATTATCCTTATAATTATACCAATAAATTTT   1146
m150507_iid=3102647_0      ------------------------------------------------------------   0
m141108_iid=1811866_0      ATTCTCTCCTTAAATTGATTAAGATCATTAATTATCCTTATAATTATACCAATAAATTTT   1317
m150501_iid=2948495_0      ATTCTCTCCTTAAATTGATTAAGATCATTAATTATCCTTATAATTATACCAATAAATTTT   742
m150504_iid=3238195_0      ATTCTCTCCTTAAATTGATTAAGATCATTAATTATCCTTATAATTATACCAATAAATTTT   3072
m150503_iid=3388511_0      ATTCTCTCCTTAAATTGATTAAGATCATTAATTATCCTTATAATTATACCAATAAATTTT   772
m150505_iid=3777611_0      ATTCTCTCCTTAAATTGATTAAGATCATTAATTATCCTTATAATTATACCAATAAATTTT   2187
m150430_iid=2438833_0      ATTCTCTCCTTAAATTGATTAAGATCATTAATTATCCTTATAATTATACCAATAAATTTT   2536
m141108_iid=1698969_0      ATTCTCTCCTTAAATTGATTAAGATCATTAATTATCCTTATAATTATACCAATAAATTTT   3658
m150430_iid=3059190_0      ------------------------------------------------------------   0
m141107_iid=1383067_0      ------------------------------------------------------------   0
m141101_iid=597008_0       ------------------------------------------------------------   0
m150506_iid=2596496_0      ATTCTCTCCTTAAATTGATTAAGATCATTAATTATCCTTATAATTATACCAATAAATTTT   4014
m141102_iid=910051_0       ATTCTCTCCTTAAATTGATTAAGATCATTAATTATCCTTATAATTATACCAATAAATTTT   3504
m150505_iid=3784017_0      ATTCTCTCCTTAAATTGATTAAGATCATTAATTATCCTTATAATTATACCAATAAATTTT   2303
m141101_iid=682151_0       ATTCTCTCCTTAAATTGATTAAGATCATTAATTATCCTTATAATTATACCAATAAATTTT   1373
m150501_iid=3748029_0      ATTCTCTCCTTAAATTGATTAAGATCATTAATTATCCTTATAATTATACCAATAAATTTT   1914
m150504_iid=2876272_0      ATTCTCTCCTTAAATTGATTAAGATCATTAATTATCCTTATAATTATACCAATAAATTTT   3778
NC_042820.1                ATTCTCTCCTTAAATTGATTAAGATCATTAATTATCCTTATAATTATACCAATAAATTTT   4021
m141102_iid=1228825_0      ATTCTCTCCTTAAATTGATTAAGATCATTAATTATCCTTATAATTATACCAATAAATTTT   3848
m150504_iid=2876274_0      ATTCTCTCCTTAAATTGATTAAGATCATTAATTATCCTTATAATTATACCAATAAATTTT   3851
m141030_iid=80036_0        ATTCTCTCCTTAAATTGATTAAGATCATTAATTATCCTTATAATTATACCAATAAATTTT   3615
m150506_iid=2596494_0      ATTCTCTCCTTAAATTGATTAAGATCATTAATTATCCTTATAATTATACCAATAAATTTT   4016
m150503_iid=2526862_0      ATTCTCTCCTTAAATTGATTAAGATCATTAATTATCCTTATAATTATACCAATAAATTTT   3700
m150506_iid=2567000_0      ATTCTCTCCTTAAATTGATTAAGATCATTAATTATCCTTATAATTATACCAATAAATTTT   1649
m150507_iid=3073271_0      ATTCTCTCCTTAAATTGATTAAGATCATTAATTATCCTTATAATTATACCAATAAATTTT   2953
m150504_iid=3156087_0      ATTCTCTCCTTAAATTGATTAAGATCATTAATTATCCTTATAATTATACCAATAAATTTT   2776
m150505_iid=2498502_0      ATTCTCTCCTTAAATTGATTAAGATCATTAATTATCCTTATAATTATACCAATAAATTTT   427
m141102_iid=754227_0       ATTCTCTCCTTAAATTGATTAAGATCATTAATTATCCTTATAATTATACCAATAAATTTT   2988
m150507_iid=3469514_0      ATTCTCTCCTTAAATTGATTAAGATCATTAATTATCCTTATAATTATACCAATAAATTTT   3546
Rdo_MitoGenome             ATTCTCTCCTTAAATTGATTAAGATCATTAATTATCCTTATAATTATACCAATAAATTTT   4021
m141101_iid=614933_0       ATTCTCTCCTTAAATTGATTAAGATCATTAATTATCCTTATAATTATACCAATAAATTTT   2573
```

atp6

```
m141031_iid=347546_0       ------------------------------------------------------------   0
m150506_iid=3127414_0      TGATTAAT-CCGTCACGTATATCTTTATTATGAATAAAAATTTCA--AAAAACTTCATAA   567
m141102_iid=1245517_0      TGATTAATCCCGTCACGTATATCTTTATTATGAATAAAAATTTCAA-AAAAACTTCATAA   2207
```

```
m141107_iid=1405727_0     ------------------------------------------------------------   0
m141102_iid=987614_0      TGATTAATCCCGTCACGTATATCTTTATTATGAATAAAAATTTCAA-AAAAACTTCATAA   2349
m141108_iid=1701429_0     ------------------------------------------------------------   0
m141102_iid=987286_0      TGATTAA-TCCGTCACGTATATCTTTATTATGAATAAAAATTTCAA-AAAAACTTCATAA   306
m150430_iid=3272125_0     TGATTAA-TCCGTCACGTATATCTTTATTATGAATAAAAATTTCAA--AAAACTTCATAA   1824
m141102_iid=754225_0      TGATTAATCCCGTCACGTATATCTTTATTATGAATAAAAATTTCAA-AAAAACTTCATAA   2996
m141107_iid=1409934_0     TGATTAATCCCGTCACGTATATCTTTATTATGAATAAAAATTTCAA-AAAAACTTCATAA   3526
m150503_iid=3416920_0     TGATTAATCCCGTCACGTATATCTTTATTATGAATAAAAATTTCAA--AAAACTTCATAA   2260
m141108_iid=1800121_0     TGATTAA--TCGTCACGTATATCTTTATTATGAATAAAAATTTCAAAAAAAACTTCATAA   2069
m141031_iid=147540_0      TGATTAATCCCGTCACGTATATCTTTATTATGAATAAAAATTTCAA-AAAAACTTCATAA   2412
m150507_iid=3674924_0     TGATTAATCCCGTCACGTATATCTTTATTATGAATAAAAATTTCAA-AAAAACTTCATAA   2560
m150503_iid=2923751_0     TGATTAATCCCGTCACGTATATCTTTATTATGAATAAAAATTTCAA-AAAAACTTCATAA   2410
m141031_iid=115118_0      TGATTAATCCCGTCACGTATATCTTTATTATGAATAAAAATTTCAA-AAAAACTTCATAA   1257
m150430_iid=2722448_0     TGATTAATCCCGTCACGTATATCTTTATTATGAATAAAAATTTCAA--AAAACTTCATAA   1204
m150507_iid=3102647_0     ------------------------------------------------------------   0
m141108_iid=1811866_0     TGATTAATCCCGTCACGTATATCTTTATTATGAATAAAAATTTCAA-AAAAACTTCATAA   1376
m150501_iid=2948495_0     TGATTAATCCCGTCACGTATATCTTTATTATGAATAAAAATTTCAA-AAAAACTTCATAA   801
m150504_iid=3238195_0     TGATTAATCCCGTCACGTATATCTTTATTATGAATAAAAATTTCAA-AAAAACTTCATAA   3131
m150503_iid=3388511_0     TGATTAATCCCGTCACGTATATCTTTATTATGAATAAAAATTTCAA-AAAAACTTCATAA   831
m150505_iid=3777611_0     TGATTAATCCCGTCACGTATATCTTTATTATGAATAAAAATTTCAA-AAAAACTTCATAA   2246
m150430_iid=2438833_0     TGATTAATCCCGTCACGTATATCTTTATTATGAATAAAAATTTCAA-AAAAACTTCATAA   2595
m141108_iid=1698969_0     TGATTAATCCCGTCACGTATATCTTTATTATGAATAAAAATTTCAA-AAAAACTTCATAA   3717
m150430_iid=3059190_0     ------------------------------------------------------------   0
m141107_iid=1383067_0     ------------------------------------------------------------   0
m141101_iid=597008_0      ------------------------------------------------------------   0
m150506_iid=2596496_0     TGATTAATCCCGTCACGTATATCTTTATTATGAATAAAAATTTCAA-AAAAACTTCATAA   4073
m141102_iid=910051_0      TGATTAATCCCGTCACGTATATCTTTATTATGAATAAAAATTTCAA-AAAAACTTCATAA   3563
m150505_iid=3784017_0     TGATTAATCCCGTCACGTATATCTTTATTATGAATAAAAATTTCAA-AAAAACTTCATAA   2362
m141101_iid=682151_0      TGATTAATCCCGTCACGTATATCTTTATTATGAATAAAAATTTCAA-AAAAACTTCATAA   1432
m150501_iid=3748029_0     TGATTAATCCCGTCACGTATATCTTTATTATGAATAAAAATTTCAA-AAAAACTTCATAA   1973
m150504_iid=2876272_0     TGATTAATCCCGTCACGTATATCTTTATTATGAATAAAAATTTCAA-AAAAACTTCATAA   3837
NC_042820.1               TGATTAATCCCGTCACGTATATCTTTATTATGAATAAAAATTTCAA-AAAAACTTCATAA   4080
m141102_iid=1228825_0     TGATTAATCCCGTCACGTATATCTTTATTATGAATAAAAATTTCAA-AAAAACTTCATAA   3907
m150504_iid=2876274_0     TGATTAATCCCGTCACGTATATCTTTATTATGAATAAAAATTTCAA-AAAAACTTCATAA   3910
m141030_iid=80036_0       TGATTAATCCCGTCACGTATATCTTTATTATGAATAAAAATTTCAA-AAAAACTTCATAA   3674
m150506_iid=2596494_0     TGATTAATCCCGTCACGTATATCTTTATTATGAATAAAAATTTCAA-AAAAACTTCATAA   4075
m150503_iid=2526862_0     TGATTAATCCCGTCACGTATATCTTTATTATGAATAAAAATTTCAA-AAAAACTTCATAA   3759
m150506_iid=2567000_0     TGATTAATCCCGTCACGTATATCTTTATTATGAATAAAAATTTCAA-AAAAACTTCATAA   1708
m150507_iid=3073271_0     TGATTAATCCCGTCACGTATATCTTTATTATGAATAAAAATTTCAA-AAAAACTTCATAA   3012
m150504_iid=3156087_0     TGATTAATCCCGTCACGTATATCTTTATTATGAATAAAAATTTCAA-AAAAACTTCATAA   2835
m150505_iid=2498502_0     TGATTAATCCCGTCACGTATATCTTTATTATGAATAAAAATTTCAA-AAAAACTTCATAA   486
m141102_iid=754227_0      TGATTAATCCCGTCACGTATATCTTTATTATGAATAAAAATTTCAA-AAAAACTTCATAA   3047
m150507_iid=3469514_0     TGATTAATCCCGTCACGTATATCTTTATTATGAATAAAAATTTCAA-AAAAACTTCATAA   3605
Rdo_MitoGenome            TGATTAATCCCGTCACGTATATCTTTATTATGAATAAAAATTTCAA-AAAAACTTCATAA   4080
m141101_iid=614933_0      TGATTAATCCCGTCACGTATATCTTTATTATGAATAAAAATTTCAA-AAAAACTTCATAA   2632
```

atp6

```
m141031_iid=347546_0      ------------------------------------------------------------   0
m150506_iid=3127414_0     CGAATTTAA-ATCTTAATTGG--AAAAAAAACGGATCATCATTAATGTTCAC--TCCTAC   622
m141102_iid=1245517_0     CGAATTTAA-ATCTTAATTGGA-AAAAACAACGGATCATCATTAATGTTCACCTCCCTAC   2265
m141107_iid=1405727_0     ------------------------------------------------------------   0
m141102_iid=987614_0      CGAATTTAAAATCTTAATTGGAA-AAAACAACGGATCATCATTAATGTTCACCTCCCTAC   2408
m141108_iid=1701429_0     ------------------------------------------------------------   0
m141102_iid=987286_0      CGAATTTAAAATCTTAATTGGAA-AAAACAACGGATCATCATTAATGTTCACCTCCCTAC   365
m150430_iid=3272125_0     CGAATTTAAAATCTTAATTGGAAAAAAACAACGGATCATCATTAATGTTCACCTCCCTAC   1884
m141102_iid=754225_0      CGAATTTAAAATCTTAATTGGAA-AAAACAACGGATCATCATTAATGTTCACCTCCCTAC   3055
m141107_iid=1409934_0     CGAATTTAAAATCTTAATTGGAA-A-AAAAACGGATCATCATTAATGTTCACCTCCCTAC   3584
m150503_iid=3416920_0     CGAATTTAAAATCTTAATTGGAA-AAAACAACGGATCATCATTAATGTTCACCTCCCTAC   2319
m141108_iid=1800121_0     CGAATTTAAAATCTTAATTGGA--AAAACAACGGATCATCATTAATGTTCACCTCCCTAC   2127
m141031_iid=147540_0      CGAATTTAAAATCTTAATTGGAA-AAAACAACGGATCATCATTAATGTTCACCTCCCTAC   2471
m150507_iid=3674924_0     CGAATTTAAAATCTTAATTGGAA-AAAACAACGGATCATCATTAATGTTCACCTCCCTAC   2619
m150503_iid=2923751_0     CGAATTTAAAATCTTAATTGGAA-AAAACAACGGATCATCATTAATGTTCACCTCCCTAC   2469
m141031_iid=115118_0      CGAATTTAAAATCTTAATTGGAA-AAAACAACGGATCATCATTAATGTTCACCTCCCTAC   1316
m150430_iid=2722448_0     CGAATTTAAAATCTTAATTGGAA-AAAACAACGGATCATCATTAATGTTCACCTCCCTAC   1263
m150507_iid=3102647_0     ------------------------------------------------------------   0
m141108_iid=1811866_0     CGAATTTAAAATCTTAATTGGAA-AAAACAACGGATCATCATTAATGTTCACCTCCCTAC   1435
m150501_iid=2948495_0     CGAATTTAAAATCTTAATTGGAA-AAAACAACGGATCATCATTAATGTTCACCTCCCTAC   860
m150504_iid=3238195_0     CGAATTTAAAATCTTAATTGGAA-AAAACAACGGATCATCATTAATGTTCACCTCCCTAC   3190
m150503_iid=3388511_0     CGAATTTAAAATCTTAATTGG-A-AAAACAACGGATCATCATTAATGTTCACCT-CCTAC   888
m150505_iid=3777611_0     CGAATTTAAAATCTTAATTGGAA-AAAACAACGGATCATCATTAATGTTCACCTCCCTAC   2305
m150430_iid=2438833_0     CGAATTTAAAATCTTAATTGGAA-AAAACAACGGATCATCATTAATGTTCACCTCCCTAC   2654
```

```
m141108_iid=1698969_0      CGAATTTAAAATCTTAATTGGAA-AAAACAACGGATCATCATTAATGTTCACCTCCCTAC   3776
m150430_iid=3059190_0      ------------------------------------------------------------   0
m141107_iid=1383067_0      ------------------------------------------------------------   0
m141101_iid=597008_0       ------------------------------------------------------------   0
m150506_iid=2596496_0      CGAATTTAAAATCTTAATTGGAA-AAAACAACGGATCATCATTAATGTTCACCTCCCTAC   4132
m141102_iid=910051_0       CGAATTTAAAATCTTAATTGGAA-AAAACAACGGATCATCATTAATGTTCACCTCCCTAC   3622
m150505_iid=3784017_0      CGAATTTAAAATCTTAATTGG-A-AAAACAACGGATCATCATTAATGTTCACCTCCCTAC   2420
m141101_iid=682151_0       CGAATTTAAAATCTTAATTGGAA-AAAACAACGGATCATCATTAATGTTCACCTCCCTAC   1491
m150501_iid=3748029_0      CGAATTTAAAATCTTAATTGGAA-AAAACAACGGATCATCATTAATGTTCACCTCCCTAC   2032
m150504_iid=2876272_0      CGAATTTAAAATCTTAATTGGAA-AAAACAACGGATCATCATTAATGTTCACCTCCCTAC   3896
NC_042820.1                CGAATTTAAAATCTTAATTGGAA-AAAACAACGGATCATCATTAATGTTCACCTCCCTAC   4139
m141102_iid=1228825_0      CGAATTTAAAATCTTAATTGGAA-AAAACAACGGATCATCATTAATGTTCACCTCCCTAC   3966
m150504_iid=2876274_0      CGAATTTAAAATCTTAATTGGAA-AAAACAACGGATCATCATTAATGTTCACCTCCCTAC   3969
m141030_iid=80036_0        CGAATTTAAAATCTTAATTGGAA-AAAACAACGGATCATCATTAATGTTCACCTCCCTAC   3733
m150506_iid=2596494_0      CGAATTTAAAATCTTAATTGGAA-AAAACAACGGATCATCATTAATGTTCACCTCCCTAC   4134
m150503_iid=2526862_0      CGAATTTAAAATCTTAATTGGAA-AAAACAACGGATCATCATTAATGTTCACCTCCCTAC   3818
m150506_iid=2567000_0      CGAATTTAAAATCTTAATTGGAA-AAAACAACGGATCATCATTAATGTTCACCTCCCTAC   1767
m150507_iid=3073271_0      CGAATTTAAAATCTTAATTGGAA-AAAACAACGGATCATCATTAATGTTCACCTCCCTAC   3071
m150504_iid=3156087_0      CGAATTTAAAATCTTAATTGGAA-AAAACAACGGATCATCATTAATGTTCACCTCCCTAC   2894
m150505_iid=2498502_0      CGAATTTAAAATCTTAATTGGAA-AAAACAACGGATCATCATTAATGTTCACCTCCCTAC   545
m141102_iid=754227_0       CGAATTTAAAATCTTAATTGGAA-AAAACAACGGATCATCATTAATGTTCACCTCCCTAC   3106
m150507_iid=3469514_0      CGAATTTAAAATCTTAATTGGAA-AAAACAACGGATCATCATTAATGTTCACCTCCCTAC   3664
Rdo_MitoGenome             CGAATTTAAAATCTTAATTGGAA-AAAACAACGGATCATCATTAATGTTCACCTCCCTAC   4139
m141101_iid=614933_0       CGAATTTAAAATCTTAATTGGAA-AAAACAACGGATCATCATTAATGTTCACCTCCCTAC   2691
```

atp6 →

```
m141031_iid=347546_0       ------------------------------------------------------------   0
m150506_iid=3127414_0      TACTTCTAATTATAATTAATAATTTTA-----TAGGACTTTT-CCGTACAT-TTTACAAG   675
m141102_iid=1245517_0      TACTTCTAATTATAATTAATAATTT-TA----TAGGACTTTTCCCGTACATCTTTACAAG   2320
m141107_iid=1405727_0      ------------------------------------------------------------   0
m141102_iid=987614_0       TACTTCTAATTATAATTAATA-ATTTTATAGG----ACTTTTCCCGTACATCTTTACAAG   2463
m141108_iid=1701429_0      ------------------------------------------------------------   0
m141102_iid=987286_0       TACTTCTAATTATAATTAATAA--TTT---TATAGGACTTTTCCCGTACATCTTTACAAG   420
m150430_iid=3272125_0      TACTTCTAATTATAATTAATAATTTTT---TATAGGACTTTTCCCGTACATCTTTACAAG   1941
m141102_iid=754225_0       TACTTCTAATTATAATTAATAAT-TTTATAGG----ACTTTTCCCGTACATCTTTACAAG   3110
m141107_iid=1409934_0      TACTTCTAATTATAATTAATAAT-TTTATAGGACTAGCTTTTCCCGTACATCTTTACAAG   3643
m150503_iid=3416920_0      TACTTCTAATTATAATTAATA-ATTTTATAGG----ACTTTTCCCGTACATCTTTACAAG   2374
m141108_iid=1800121_0      TACTTCTAATTATAATTAATAATTTTTATAGG----ACTTTTCCCGTACATCTTTACAAA   2183
m141031_iid=147540_0       TACTTCTAATTATAATTAATA-ATTTTATAGG----ACTTTTCCCGTACATCTTTACAAG   2526
m150507_iid=3674924_0      TACTTCTAATTATAATTAATA-ATTTTATAGG----ACTTTTCCCGTACATCTTTACAAG   2674
m150503_iid=2923751_0      TACTTCTAATTATAATTAATA-ATTTTATAGG----ACTTTTCCCGTACATCTTTACAAG   2524
m141031_iid=115118_0       TACTTCTAATTATAATTAATA-ATTTTATAGG----ACTTTTCCCGTACATCTTTACAAG   1371
m150430_iid=2722448_0      TACTTCTAATTATAATTAATA-ATTTTATAGG----ACTTTTCCCGTACATCTTTACAAG   1318
m150507_iid=3102647_0      ------------------------------------------------------------   0
m141108_iid=1811866_0      TACTTCTAATTATAATTAATA-ATTTTATAGG----ACTTTTCCCGTACATCTTTACAAG   1490
m150501_iid=2948495_0      TACTTCTAATTATAATTAATA-ATTTTATAGG----ACTTTTCCCGTACATCTTTACAAG   915
m150504_iid=3238195_0      TACTTCTAATTATAATTAATA-ATTTTATAGG----ACTTTTCCCGTACATCTTTACAAG   3245
m150503_iid=3388511_0      TACTTCTAATTATAATTAATA-ATTTTATAGG----ACTTTTCCCGTACATCTTTACAAG   943
m150505_iid=3777611_0      TACTTCTAATTATAATTAATA-ATTTTATAGG----ACTTTTCCCGTACATCTTTACAAG   2360
m150430_iid=2438833_0      TACTTCTAATTATAATTAATA-ATTTTATAGG----ACTTTTCCCGTACATCTTTACAAG   2709
m141108_iid=1698969_0      TACTTCTAATTATAATTAATA-ATTTTATAGG----ACTTTTCCCGTACATCTTTACAAG   3831
m150430_iid=3059190_0      ------------------------------------------------------------   0
m141107_iid=1383067_0      ------------------------------------------------------------   0
m141101_iid=597008_0       ------------------------------------------------------------   0
m150506_iid=2596496_0      TACTTCTAATTATAATTAATA-ATTTTATAGG----ACTTTTCCCGTACATCTTTACAAG   4187
m141102_iid=910051_0       TACTTCTAATTATAATTAATA-ATTTTATAGG----ACTTTTCCCGTACATCTTTACAAG   3677
m150505_iid=3784017_0      TACTTCTAATTATAATTAATA-ATTTTATAGG----ACTTTTCCCGTACATCTTTACAAG   2475
m141101_iid=682151_0       TACTTCTAATTATAATTAATA-ATTTTATAGG----ACTTTTCCCGTACATCTTTACAAG   1546
m150501_iid=3748029_0      TACTTCTAATTATAATTAATA-ATTTTATAGG----ACTTTTCCCGTACATCTTTACAAG   2087
m150504_iid=2876272_0      TACTTCTAATTATAATTAATA-ATTTTATAGG----ACTTTTCCCGTACATCTTTACAAG   3951
NC_042820.1                TACTTCTAATTATAATTAATA-ATTTTATAGG----ACTTTTCCCGTACATCTTTACAAG   4194
m141102_iid=1228825_0      TACTTCTAATTATAATTAATA-ATTTTATAGG----ACTTTTCCCGTACATCTTTACAAG   4021
m150504_iid=2876274_0      TACTTCTAATTATAATTAATA-ATTTTATAGG----ACTTTTCCCGTACATCTTTACAAG   4024
m141030_iid=80036_0        TACTTCTAATTATAATTA--A-ATTTTATAGG----ACTTTTCCCGTACATCTTTACAAG   3786
m150506_iid=2596494_0      TACTTCTAATTATAATTAATA-ATTTTATAGG----ACTTTTCCCGTACATCTTTACAAG   4189
m150503_iid=2526862_0      TACTTCTAATTATAATTAATA-ATTTTATAGG----ACTTTTCCCGTACATCTTTACAAG   3873
m150506_iid=2567000_0      TACTTCTAATTATAATTAATA-ATTTTATAGG----ACTTTTCCCGTACATCTTTACAAG   1822
m150507_iid=3073271_0      TACTTCTAATTATAATTAATA-ATTTTATAGG----ACTTTTCCCGTACATCTTTACAAG   3126
m150504_iid=3156087_0      TACTTCTAATTATAATTAATA-ATTTTATAGG----ACTTTTCCCGTACATCTTTACAAG   2949
m150505_iid=2498502_0      TACTTCTAATTATAATTAATA-ATTTTATAGG----ACTTTTCCCGTACATCTTTACAAG   600
m141102_iid=754227_0       TACTTCTAATTATAATTAATA-ATTTTATAGG----ACTTTTCCCGTACATCTTTACAAG   3161
```

```
m150507_iid=3469514_0     TACTTCTAATTATAATTAATA-ATTTTATAGG----ACTTTTCCCGTACATCTTTACAAG   3719
Rdo_MitoGenome            TACTTCTAATTATAATTAATA-ATTTTATAGG----ACTTTTCCCGTACATCTTTACAAG   4194
m141101_iid=614933_0      TACTTCTAATTATAATTAATA-ATTTTATAGG----ACTTTTCCCGTACATCTTTACAAG   2746

                                                  atp6
m141031_iid=347546_0      ------------------------------------------------------------   0
m150506_iid=3127414_0     AACA-AGACATTTAACAATAACTTTAACCTTAGCTTTA-CATTATGAACAAGATTTATAA   733
m141102_iid=1245517_0     AACA-AGACATTTAACAATAACTTTAACCTTAGCTTTACCATTATGAACAAGATTTATAA   2379
m141107_iid=1405727_0     ------------------------------------------------------------   0
m141102_iid=987614_0      AACA-AGACATTTAACAATAACTTTAACCTTAGCTTTACCATTATGAACAAGATTTATAA   2522
m141108_iid=1701429_0     ------------------------------------------------------------   0
m141102_iid=987286_0      AACA-AGACATTTAACAATAACTTTAACCTTAGCTTTACCATTATGAACAAGATTTATAA   479
m150430_iid=3272125_0     AACAAAGACATTTAACAATAACTTTAACCTTAGCTTTACCATTATGAACAAGATTTATAA   2001
m141102_iid=754225_0      AACA-AGACATTTAACAATAACTTTAACCTTAGCTTTACCATTATGAACAAGATTTATAA   3169
m141107_iid=1409934_0     AACA-AGACATTTAACAATAACTTTAACCTTAGCTTTACCATTATGAACAAGATTTATAA   3702
m150503_iid=3416920_0     AACA-AGACATTTAACAATAACTTTAACCTTAGCTTTACCATTATGAACAAGATTTATAA   2433
m141108_iid=1800121_0     ACAA-GACAATTTAACAATAACTTTAACCTTAGCTTTACCATTATGAACAAGATTTATAA   2242
m141031_iid=147540_0      AACA-AGACATTTAACAATAACTTTAACCTTAGCTTTACCATTATGAACAAGATTTATAA   2585
m150507_iid=3674924_0     AACA-AGACATTTAACAATAACTTTAACCTTAGCTTTACCATTATGAACAAGATTTATAA   2733
m150503_iid=2923751_0     AACA-AGACATTTAACAATAACTTTAACCTTAGCTTTACCATTATGAACAAGATTTATAA   2583
m141031_iid=115118_0      AACA-AGACATTTAACAATAACTTTAACCTTAGCTTTACCATTATGAACAAGATTTATAA   1430
m150430_iid=2722448_0     AACA-AGACATTTAACAATAACTTTAACCTTAGCTTTACCATTATGAACAAGATTTATAA   1377
m150507_iid=3102647_0     ------------------------------------------------------------   0
m141108_iid=1811866_0     AACA-AGACATTTAACAATAACTTTAACCTTAGCTTTACCATTATGAACAAGATTTATAA   1549
m150501_iid=2948495_0     AACA-AGACATTTAACAATAACTTTAACCTTAGCTTTACCATTATGAACAAGATTTATAA   974
m150504_iid=3238195_0     AACA-AGACATTTAACAATAACTTTAACCTTAGCTTTACCATTATGAACAAGATTTATAA   3304
m150503_iid=3388511_0     AACA-AGACATTTAACAATAACTTTAACCTTAGCTTTACCATTATGAACAAGATTTATAA   1002
m150505_iid=3777611_0     AACA-AGACATTTAACAATAACTTTAACCTTAGCTTTACCATTATGAACAAGATTTATAA   2419
m150430_iid=2438833_0     AA-A-AGACATTTAACAATAACTTTAACCTTAGCTTTACCATTATGAACAAGATTTATAA   2767
m141108_iid=1698969_0     AACA-AGACATTTAACAATAACTTTAACCTTAGCTTTACCATTATGAACAAGATTTATAA   3890
m150430_iid=3059190_0     ------------------------------------------------------------   0
m141107_iid=1383067_0     ------------------------------------------------------------   0
m141101_iid=597008_0      ------------------------------------------------------------   0
m150506_iid=2596496_0     AACA-AGACATTTAACAATAACTTTAACCTTAGCTTTACCATTATGAACAAGATTTATAA   4246
m141102_iid=910051_0      AACA-AGACATTTAACAATAACTTTAACCTTAGCTTTACCATTATGAACAAGATTTATAA   3736
m150505_iid=3784017_0     AACA-AGACATTTAACAATAACTTTAACCTTAGCTTTACCATTATGAACAAGATTTATAA   2534
m141101_iid=682151_0      AACA-AGACATTTAACAATAACTTTAACCTTAGCTTTACCATTATGAACAAGATTTATAA   1605
m150501_iid=3748029_0     AACA-AGACATTTAACAATAACTTTAACCTTAGCTTTACCATTATGAACAAGATTTATAA   2146
m150504_iid=2876272_0     AACA-AGACATTTAACAATAACTTTAACCTTAGCTTTACCATTATGAACAAGATTTATAA   4010
NC_042820.1               AACA-AGACATTTAACAATAACTTTAACCTTAGCTTTACCATTATGAACAAGATTTATAA   4253
m141102_iid=1228825_0     AACA-AGACATTTAACAATAACTTTAACCTTAGCTTTACCATTATGAACAAGATTTATAA   4080
m150504_iid=2876274_0     AACA-AGACATTTAACAATAACTTTAACCTTAGCTTTACCATTATGAACAAGATTTATAA   4083
m141030_iid=80036_0       AACA-AGACATTTAACAATAACTTTAACCTTAGCTTTACCATTATGAACAAGATTTATAA   3845
m150506_iid=2596494_0     AACA-AGACATTTAACAATAACTTTAACCTTAGCTTTACCATTATGAACAAGATTTATAA   4248
m150503_iid=2526862_0     AACA-AGACATTTAACAATAACTTTAACCTTAGCTTTACCATTATGAACAAGATTTATAA   3932
m150506_iid=2567000_0     AACA-AGACATTTAACAATAACTTTAACCTTAGCTTTACCATTATGAACAAGATTTATAA   1881
m150507_iid=3073271_0     AACA-AGACATTTAACAATAACTTTAACCTTAGCTTTACCATTATGAACAAGATTTATAA   3185
m150504_iid=3156087_0     AACA-AGACATTTAACAATAACTTTAACCTTAGCTTTACCATTATGAACAAGATTTATAA   3008
m150505_iid=2498502_0     AACA-AGACATTTAACAATAACTTTAACCTTAGCTTTACCATTATGAACAAGATTTATAA   659
m141102_iid=754227_0      AACA-AGACATTTAACAATAACTTTAACCTTAGCTTTACCATTATGAACAAGATTTATAA   3220
m150507_iid=3469514_0     AACA-AGACATTTAACAATAACTTTAACCTTAGCTTTACCATTATGAACAAGATTTATAA   3778
Rdo_MitoGenome            AACA-AGACATTTAACAATAACTTTAACCTTAGCTTTACCATTATGAACAAGATTTATAA   4253
m141101_iid=614933_0      AACA-AGACATTTAACAATAACTTTAACCTTAGCTTTACCATTATGAACAAGATTTATAA   2805

                                                  atp6
m141031_iid=347546_0      ------------------------------------------------------------   0
m150506_iid=3127414_0     TTTACGGATGATTTAATAATACAATCTCTATACTCGCACACTTAGT-CCTCAAGGAACAC   792
m141102_iid=1245517_0     TTTACGGATGATTTAATAATACAATCTCTATACTCGCACACTTAGTCCCTCAAGGAACAC   2439
m141107_iid=1405727_0     ------------------------------------------------------------   0
m141102_iid=987614_0      TTTACGGATGATTTAATAATACAATCTCTATACTCGCACACTTAGTCCCTCAAGGAACAC   2582
m141108_iid=1701429_0     ------------------------------------------------------------   0
m141102_iid=987286_0      TTTACGGATGATTTAATAATACAATCTCTATACTCGCACACTTAGTCCCTCAAGGAACAC   539
m150430_iid=3272125_0     TTTACGGATGATTTAATAATACAATCTCTATACTCGCACACTTAGTCCCTCAAGGAACAC   2061
m141102_iid=754225_0      TTTACGGATGATTTAATAATACAATCTCTATACTCGCACACTTAGTCCCTCAAGGAACAC   3229
m141107_iid=1409934_0     TTTACGGATGATTTAATAATACAATCTCTATACTCGCACACTTAGTCCCTCAAGGAACAC   3762
m150503_iid=3416920_0     TTTACGGATGATTTAATAATACAATCTCTATACTCGCACACTTAGTCCCTCAAGGAACAC   2493
m141108_iid=1800121_0     TTTACGGATGATTTAATAATACAATCTCTATACTCGCACACTTAGTCCCTCAAGGAACAC   2302
m141031_iid=147540_0      TTTACGGATGATTTAATAATACAATCTCTATACTCGCACACTTAGTCCCTCAAGGAACAC   2645
m150507_iid=3674924_0     TTTACGGATGATTTAATAATACAATCTCTATACTCGCACACTTAGTCCCTCAAGGAACAC   2793
m150503_iid=2923751_0     TTTACGGATGATTTAATAATACAATCTCTATACTCGCACACTTAGTCCCTCAAGGAACAC   2643
m141031_iid=115118_0      TTTACGGATGATTTAATAATACAATCTCTATACTCGCACACTTAGTCCCTCAAGGAACAC   1490
```

```
m150430_iid=2722448_0      TTTACGGATGATTTAATAATACAATCTCTATACTCGCACACTTAGTCCCTCAAGGAACAC   1437
m150507_iid=3102647_0      ------------------------------------------------------------   0
m141108_iid=1811866_0      TTTACGGATGATTTAATAATACAATCTCTATACTCGCACACTTAGTCCCTCAAGGAACAC   1609
m150501_iid=2948495_0      TTTACGGATGATTTAATAATACAATCTCTATACTCGCACACTTAGTCCCTCAAGGAACAC   1034
m150504_iid=3238195_0      TTTACGGATGATTTAATAATACAATCTCTATACTCGCACACTTAGTCCCTCAAGGAACAC   3364
m150503_iid=3388511_0      TTTACGGATGATTTAATAATACAATCTCTATACTCGCACACTTAGT-CCTCAAGGAACAC   1061
m150505_iid=3777611_0      TTTACGGATGATTTAATAATACAATCTCTATACTCGCACACTTAGTCCCTCAAGGAACAC   2479
m150430_iid=2438833_0      TTTACGGATGATTTAATAATACAATCTCTATACTCGCACACTTAG----TCAAGGAACAC   2823
m141108_iid=1698969_0      TTTACGGATGATTTAATAATACAATCTCTATACTCGCACACTTAGTCCCTCAAGGAACAC   3950
m150430_iid=3059190_0      ------------------------------------------------------------   0
m141107_iid=1383067_0      ------------------------------------------------------------   0
m141101_iid=597008_0       ------------------------------------------------------------   0
m150506_iid=2596496_0      TTTACGGATGATTTAATAATACAATCTCTATACTCGCACACTTAGTCCCTCAAGGAACAC   4306
m141102_iid=910051_0       TTTACGGATGATTTAATAATACAATCTCTATACTCGCACACTTAGTCCCTCAAGGAACAC   3796
m150505_iid=3784017_0      TTTACGGATGATTTAATAATACAATCTCTATACTCGCACACTTAGTCCCTCAAGGAACAC   2594
m141101_iid=682151_0       TTTACGGATGATTTAATAATACAATCTCTATACTCGCACACTTAGTCCCTCAAGGAACAC   1665
m150501_iid=3748029_0      TTTACGGATGATTTAATAATACAATCTCTATACTCGCACACTTAGTCCCTCAAGGAACAC   2206
m150504_iid=2876272_0      TTTACGGATGATTTAATAATACAATCTCTATACTCGCACACTTAGTCCCTCAAGGAACAC   4070
NC_042820.1                TTTACGGATGATTTAATAATACAATCTCTATACTCGCACACTTAGTCCCTCAAGGAACAC   4313
m141102_iid=1228825_0      TTTACGGATGATTTAATAATACAATCTCTATACTCGCACACTTAGTCCCTCAAGGAACAC   4140
m150504_iid=2876274_0      TTTACGGATGATTTAATAATACAATCTCTATACTCGCACACTTAGTCCCTCAAGGAACAC   4143
m141030_iid=80036_0        TTTACGGATGATTTAATAATACAATCTCTATACTCGCACACTTAGTCCCTCAAGGAACAC   3905
m150506_iid=2596494_0      TTTACGGATGATTTAATAATACAATCTCTATACTCGCACACTTAGTCCCTCAAGGAACAC   4308
m150503_iid=2526862_0      TTTACGGATGATTTAATAATACAATCTCTATACTCGCACACTTAGTCCCTCAAGGAACAC   3992
m150506_iid=2567000_0      TTTACGGATGATTTAATAATACAATCTCTATACTCGCACACTTAGTCCCTCAAGGAACAC   1941
m150507_iid=3073271_0      TTTACGGATGATTTAATAATACAATCTCTATACTCGCACACTTAGTCCCTCAAGGAACAC   3245
m150504_iid=3156087_0      TTTACGGATGATTTAATAATACAATCTCTATACTCGCACACTTAGTCCCTCAAGGAACAC   3068
m150505_iid=2498502_0      TTTACGGATGATTTAATAATACAATCTCTATACTCGCACACTTAGTCCCTCAAGGAACAC   719
m141102_iid=754227_0       TTTACGGATGATTTAATAATACAATCTCTATACTCGCACACTTAGTCCCTCAAGGAACAC   3280
m150507_iid=3469514_0      TTTACGGATGATTTAATAATACAATCTCTATACTCGCACACTTAGTCCCTCAAGGAACAC   3838
Rdo_MitoGenome             TTTACGGATGATTTAATAATACAATCTCTATACTCGCACACTTAGTCCCTCAAGGAACAC   4313
m141101_iid=614933_0       TTTACGGATGATTTAATAATACAATCTCTATACTCGCACACTTAGTCCCTCAAGGAACAC   2865
```

atp6

```
m141031_iid=347546_0       ------------------------------------------------------------   0
m150506_iid=3127414_0      CT-CCATTTTAATACTTTTAATAAGTTGTATTGAAACAATTAGAAATATTATTCGACCAG   851
m141102_iid=1245517_0      CTCCCATTTTAATACCTTTTATAGTATGTATTGAAACAATTAGAAATATTATTCGACCAG   2499
m141107_iid=1405727_0      ------------------------------------------------------------   0
m141102_iid=987614_0       CTCCCATTTTAATACCTTTTATAGTATGTATTGAAACAATTAGAAATATTATTCGACCAG   2642
m141108_iid=1701429_0      ------------------------------------------------------------   0
m141102_iid=987286_0       CTCCCATTTTAATACCTTTTATAGTATGTATTGAAACAATTAGAAATATTATTCGACCAG   599
m150430_iid=3272125_0      CTCCCATTTTAATACCTTTTATAGTATGTATTGAAACAATTAGAAATATTATTCGACCAG   2121
m141102_iid=754225_0       CTCCCATTTTAATACCTTTTATAGTATGTATTGAAACAATTAGAAATATTATTCGACCAG   3289
m141107_iid=1409934_0      CTCCCATTTTAATACCTTTTATAGTATGTATTGAAACAATTAGAAATATTATTCGACCAG   3822
m150503_iid=3416920_0      CTCCCATTTTAATACCTTTTATAGTATGTATTGAAACAATTAGAAATATTATTCGACCAG   2553
m141108_iid=1800121_0      CTCCCATTTTAATACCTTTTATAGTATGTATTGAAACAATTAGAAATATTATTCGACCAG   2362
m141031_iid=147540_0       CTCCCATTTTAATACCTTTTATAGTATGTATTGAAACAATTAGAAATATTATTCGACCAG   2705
m150507_iid=3674924_0      CTCCCATTTTAATACCTTTTATAGTATGTATTGAAACAATTAGAAATATTATTCGACCAG   2853
m150503_iid=2923751_0      CTCCCATTTTAATACCTTTTATAGTATGTATTGAAACAATTAGAAATATTATTCGACCAG   2703
m141031_iid=115118_0       CTCCCATTTTAATACCTTTTATAGTATGTATTGAAACAATTAGAAATATTATTCGACCAG   1550
m150430_iid=2722448_0      CTCCCATTTTAATACCTTTTATAGTATGTATTGAAACAATTAGAAATATTATTCGACCAG   1497
m150507_iid=3102647_0      ------------------------------------------------------------   0
m141108_iid=1811866_0      CTCCCATTTTAATACCTTTTATAGTATGTATTGAAACAATTAGAAATATTATTCGACCAG   1669
m150501_iid=2948495_0      CTCCCATTTTAATACCTTTTATAGTATGTATTGAAACAATTAGAAATATTATTCGACCAG   1094
m150504_iid=3238195_0      CTCCCATTTTAATACCTTTTATAGTATGTATTGAAACAATTAGAAATATTATTCGACCAG   3424
m150503_iid=3388511_0      CTCCCATTTTAATACCTTTTATAGTATGTATTGAAACAATTAGAAATATTATTCGACCAG   1121
m150505_iid=3777611_0      CTCCCATTTTAATACCTTTTATAGTATGTATTGAAACAATTAGAAATATTATTCGACCAG   2539
m150430_iid=2438833_0      CTCCCATTTTAATACC-TTTATAGTATGTATTGAAACAATTAGAAATATTATTCGACCAG   2882
m141108_iid=1698969_0      CTCCCATTTTAATACCTTTTATAGTATGTATTGAAACAATTAGAAATATTATTCGACCAG   4010
m150430_iid=3059190_0      ------------------------------------------------------------   0
m141107_iid=1383067_0      ------------------------------------------------------------   0
m141101_iid=597008_0       ------------------------------------------------------------   0
m150506_iid=2596496_0      CTCCCATTTTAATACCTTTTATAGTATGTATTGAAACAATTAGAAATATTATTCGACCAG   4366
m141102_iid=910051_0       CTCCCATTTTAATACCTTTTATAGTATGTATTGAAACAATTAGAAATATTATTCGACCAG   3856
m150505_iid=3784017_0      CTCCCATTTTAATACCTTTTATAGTATGTATTGAAACAATTAGAAATATTATTCGACCAG   2654
m141101_iid=682151_0       CTCCCATTTTAATACCTTTTATAGTATGTATTGAAACAATTAGAAATATTATTCGACCAG   1725
m150501_iid=3748029_0      CTCCCATTTTAATACCTTTTATAGTATGTATTGAAACAATTAGAAATATTATTCGACCAG   2266
m150504_iid=2876272_0      CTCCCATTTTAATACCTTTTATAGTATGTATTGAAACAATTAGAAATATTATTCGACCAG   4130
NC_042820.1                CTCCCATTTTAATACCTTTTATAGTATGTATTGAAACAATTAGAAATATTATTCGACCAG   4373
m141102_iid=1228825_0      CTCCCATTTTAATACCTTTTATAGTATGTATTGAAACAATTAGAAATATTATTCGACCAG   4200
m150504_iid=2876274_0      CTCCCATTTTAATACCTTTTATAGTATGTATTGAAACAATTAGAAATATTATTCGACCAG   4203
```

```
m141030_iid=80036_0        CTCCCATTTTAATACCTTTTATAGTATGTATTGAAACAATTAGAAATATTATTCGACCAG   3965
m150506_iid=2596494_0      CTCCCATTTTAATACCTTTTATAGTATGTATTGAAACAATTAGAAATATTATTCGACCAG   4368
m150503_iid=2526862_0      CTCCCATTTTAATACCTTTTATAGTATGTATTGAAACAATTAGAAATATTATTCGACCAG   4052
m150506_iid=2567000_0      CTCCCATTTTAATACCTTTTATAGTATGTATTGAAACAATTAGAAATATTATTCGACCAG   2001
m150507_iid=3073271_0      CTCCCATTTTAATACCTTTTATAGTATGTATTGAAACAATTAGAAATATTATTCGACCAG   3305
m150504_iid=3156087_0      CTCCCATTTTAATACCTTTTATAGTATGTATTGAAACAATTAGAAATATTATTCGACCAG   3128
m150505_iid=2498502_0      CTCCCATTTTAATACCTTTTATAGTATGTATTGAAACAATTAGAAATATTATTCGACCAG   779
m141102_iid=754227_0       CTCCCATTTTAATACCTTTTATAGTATGTATTGAAACAATTAGAAATATTATTCGACCAG   3340
m150507_iid=3469514_0      CTCCCATTTTAATACCTTTTATAGTATGTATTGAAACAATTAGAAATATTATTCGACCAG   3898
Rdo_MitoGenome             CTCCCATTTTAATACCTTTTATAGTATGTATTGAAACAATTAGAAATATTATTCGACCAG   4373
m141101_iid=614933_0       CTCCCATTTTAATACCTTTTATAGTATGTATTGAAACAATTAGAAATATTATTCGACCAG   2925
```

atp6 →

```
m141031_iid=347546_0       ------------------------------------------------------------   0
m150506_iid=3127414_0      GCACATTAGCAATTCGTTTAACAGCAAT-AATAATTGCAGGATACACTTACTT-------   903
m141102_iid=1245517_0      GCACATTAGCAATTCGTTTAACAGCAAA-TATAATTGCAGG--ACACTTACTT-------   2549
m141107_iid=1405727_0      ------------------------------------------------------------   0
m141102_iid=987614_0       GCACATTAGCAATTCGTTTAACAGCAAA-TATAATTGCAGG--ACACTTACTTTACTTAC   2699
m141108_iid=1701429_0      ------------------------------------------------------------   0
m141102_iid=987286_0       GCACATTAGCAATTCGTTTAACAGCAAA-TATAATTGCAGG--ACACTTACTT-------   649
m150430_iid=3272125_0      GCACATTAGCAATTCGTTTAACAGCAAA-TAT-ATTGCAGG--ACACTTACTT-------   2170
m141102_iid=754225_0       GCACATTAGCAATTCGTTTAACAGCAAA-TATAATTGCAGG--ACACTTACTT-------   3339
m141107_iid=1409934_0      GCACATTAGCAATTCGTTTAACAGCAAA-TATAATTGCAGG--ACACTTACTT-------   3872
m150503_iid=3416920_0      GCACATTAGCAATTCGTTTAACAGCAAA-TATAATTGCAGG--ACACTTACTT-------   2603
m141108_iid=1800121_0      GCACATTAGCAATTCGTTTAACAGCAAAATATAATTGCAGG--ACACTTACTT-------   2413
m141031_iid=147540_0       GCACATTAGCAATTCGTTTAACAGCAAA-TATAATTGCAGG--ACACTTACTT-------   2755
m150507_iid=3674924_0      GCACATTAGCAATTCGTTTAACAGCAAA-TATAATTGCAGG--ACACTTACTT-------   2903
m150503_iid=2923751_0      GCACATTAGCAATTCGTTTAACAGCAAA-T---ATTGCAGG--ACACTTACTT-------   2750
m141031_iid=115118_0       GCACATTAGCAATTCGTTTAACAGCAAA-TATAATTGCAGG--ACACTTACTT-------   1600
m150430_iid=2722448_0      GCACATTAGCAATTCGTTTAACAGCAAA-TATAATTGCAGG--ACACTTACTT-------   1547
m150507_iid=3102647_0      ------------------------------------------------------------   0
m141108_iid=1811866_0      GCACATTAGCAATTCGTTTAACAGCAAA-TATAATTGCAGG--ACACTTACTT-------   1719
m150501_iid=2948495_0      GCACATTAGCAATTCGTTTAACAGCAAA-TATAATTGCAGG--ACACTTACTT-------   1144
m150504_iid=3238195_0      GCACATTAGCAATTCGTTTAACAGCAAA-TATAATTGCAGG--ACACTTACTT-------   3474
m150503_iid=3388511_0      GCACATTAGCAATTCGTTTAACAGCAAA-TATAATTGCAGG--ACACTTACTT-------   1171
m150505_iid=3777611_0      GCACATTAGCAATTCGTTTAACAGCAAA-TATAATTGCAGG--ACACTTACTT-------   2589
m150430_iid=2438833_0      GCACATTAGCAATTCGTTTAACAGCAAA-TATAATTGCAGG--ACACTTACTT-------   2932
m141108_iid=1698969_0      GCACATTAGCAATTCGTTTAACAGCAAA-TATAATTGCA----ACACTTACTT-------   4058
m150430_iid=3059190_0      ------------------------------------------------------------   0
m141107_iid=1383067_0      ------------------------------------------------------------   0
m141101_iid=597008_0       ------------------------------------------------------------   0
m150506_iid=2596496_0      GCACATTAGCAATTCGTTTAACAGCAAA-TATAATTGCAGG--ACACTTACTT-------   4416
m141102_iid=910051_0       GCACATTAGCAATTCGTTTAACAGCAAA-TATAATTGCAGG--ACACTTACTT-------   3906
m150505_iid=3784017_0      GCACATTAGCAATTCGTTTAACAGCAAA-TATAATTGCAGG--ACACTTACTT-------   2704
m141101_iid=682151_0       GCACATTAGCAATTCGTTTAACAGCAAA-TATAATTGCAGG--ACACTTACTT-------   1775
m150501_iid=3748029_0      GCACATTAGCAATTCGTTTAACAGCAAA-TATAATTGCAGG--ACACTTACTT-------   2316
m150504_iid=2876272_0      GCACATTAGCAATTCGTTTAACAGCAAA-TATAATTGCAGG--ACACTTACTT-------   4180
NC_042820.1                GCGCATTAGCAATTCGTTTAACAGCAAA-TATAATTGCAGG--ACACTTACTT-------   4423
m141102_iid=1228825_0      GCACATTAGCAATTCGTTTAACAGCAAA-TATAATTGCAGG--ACACTTACTT-------   4250
m150504_iid=2876274_0      GCACATTAGCAATTCGTTTAACAGCAAA-TATAATTGCAGG--ACACTTACTT-------   4253
m141030_iid=80036_0        GCACATTAGCAATTCGTTTAACAGCAAA-TATAATTGCAGG--ACACTTACTT-------   4015
m150506_iid=2596494_0      GCACATTAGCAATTCGTTTAACAGCAAA-TATAATTGCAGG--ACACTTACTT-------   4418
m150503_iid=2526862_0      GCACATTAGCAATTCGTTTAACAGCAAA-TATAATTGCAGG--ACACTTACTT-------   4102
m150506_iid=2567000_0      GCACATTAGCAATTCGTTTAACAGCAAA-TATAATTGCAGG--ACACTTACTT-------   2051
m150507_iid=3073271_0      GCACATTAGCAATTCGTTTAACAGCAAA-TATAATTGCAGG--ACACTTACTT-------   3355
m150504_iid=3156087_0      GCACATTAGCAATTCGTTTAACAGCAAA-TATAATTGCAGG--ACACTTACTT-------   3178
m150505_iid=2498502_0      GCACATTAGCAATTCGTTTAACAGCAAA-TATAATTGCAGG--ACACTTACTT-------   829
m141102_iid=754227_0       GCACATTAGCAATTCGTTTAACAGCAAA-TATAATTGCAGG--ACACTTACTT-------   3390
m150507_iid=3469514_0      GCACATTAGCAATTCGTTTAACAGCAAA-TATAATTGCAGG--ACACTTACTT-------   3948
Rdo_MitoGenome             GCACATTAGCAATTCGTTTAACAGCAAA-TATAATTGCAGG--ACACTTACTT-------   4423
m141101_iid=614933_0       GCACATTAGCAATTCGTTTAACAGCAAA-TATAATTGCAGG--ACACTTACTT-------   2975
```

atp6 →

```
m141031_iid=347546_0       ------------------------------------------------------------   0
m150506_iid=3127414_0      TTAACCCTTCTAGGTAAT-CAGGAGCAGGAATTTCATCAATTCTTGTAGAAGTCTTAATT   962
m141102_iid=1245517_0      TTAACCCTTCTAGGTAATACAGGAGCAGGAATTTCATCAATTCTTGTAAGAATCTTAATT   2609
m141107_iid=1405727_0      ------------------------------------------------------------   0
m141102_iid=987614_0       TTAACCCTTCTAGGTAATACAGGAGCAGGAATTTCATCAATTCTTGTAAGAATCTTAATT   2759
m141108_iid=1701429_0      ------------------------------------------------------------   0
m141102_iid=987286_0       TTAACCCTTCTAGGTAATACAGGAGCAGGAATTTCATCAATTCTTGTAAGAATCTTAA-T   708
m150430_iid=3272125_0      TTAACCCTTCTAGGTAATACAGGAGCAGGAATTTCATCAATTCTTGTAAGAATCTTTAAT   2230
```

```
m141102_iid=754225_0       TTAACCCTTCTAGGTAATACAGGAGCAGGAATTTCATCAATTCTTGTAAGAATCTTAATT   3399
m141107_iid=1409934_0      TTAACCCTTCTAGGTAATACAGGAGCAGGAATTTCATCAATTCTTGTAAGAATCTTAATT   3932
m150503_iid=3416920_0      TTAACCCTTCTAGGTAATACAGGAGCAGGAATTTCATCAATTCTTGTAAGAATCTTAATT   2663
m141108_iid=1800121_0      TTAACCCTTCTAGGTAATACAGGAGCAGGAATTTCATCAATTCTTGTAAGAATCTTAATT   2473
m141031_iid=147540_0       TTAACCCTTCTAGGTAATACAGGAGCAGGAATTTCATCAATTCTTGTAAGAATCTTAATT   2815
m150507_iid=3674924_0      TTAACCCTTCTAGGTAATACAGGAGCAGGAATTTCATCAATTCTTGTAAGAATCTTAATT   2963
m150503_iid=2923751_0      TTAACCCTTCTAGGTAATACAGGAGCAGGAATTTCATCAATTCTTGTAAGAATCTTAATT   2810
m141031_iid=115118_0       TTAACCCTTCTAGGTAATACAGGAGCAGGAATTTCATCAATTCTTGTAAGAATCTTAATT   1660
m150430_iid=2722448_0      TTAACCCTTCTAGGTAATACAGGAGCAGGAATTTCATCAATTCTTGTAA--ATCTTAATT   1605
m150507_iid=3102647_0      ------------------------------------------------------------   0
m141108_iid=1811866_0      TTAACCCTTCTAGGTAATACAGGAGCAGGAATTTCATCAATTCTTGTAAGAATCTTAATT   1779
m150501_iid=2948495_0      TTAACCCTTCTAGGTAATACAGGAGCAGGAATTTCATCAATTCTTGTAAGAATCTTAATT   1204
m150504_iid=3238195_0      TTAACCCTTCTAGGTAATACAGGAGCAGGAATTTCATCAATTCTTGTAAGAATCTTAATT   3534
m150503_iid=3388511_0      TTAACCCTTCTAGGTAATACAGGAGCAGGAATTTCATCAATTCTTGTAAGAATCTTAATT   1231
m150505_iid=3777611_0      TTAACCCTTCTAGGTAATACAGGAGCAGGAATTTCATCAATTCTTGTAAGAATCTTAATT   2649
m150430_iid=2438833_0      TTAACCCTTCTAGGTAATACAGGAGCAGGAATTTCATCAATTCTTGTAAGAATCTTAATT   2992
m141108_iid=1698969_0      TT--CCCTTCTAGGTAATACAGGAGCAGGAATTTCATCAATTCTTGTAAGAATCTTAATT   4116
m150430_iid=3059190_0      ------------------------------------------------------------   0
m141107_iid=1383067_0      ------------------------------------------------------------   0
m141101_iid=597008_0       ------------------------------------------------------------   0
m150506_iid=2596496_0      TTAACCCTTCTAGGTAATACAGGAGCAGGAATTTCATCAATTCTTGTAAGAATCTTAATT   4476
m141102_iid=910051_0       TTAACCCTTCTAGGTAATACAGGAGCAGGAATTTCATCAATTCTTGTAAGAATCTTAATT   3966
m150505_iid=3784017_0      TTAACCCTTCTAGGTAATACAGGAGCAGGAATTTCATCAATTCTTGTAAGAATCTTAATT   2764
m141101_iid=682151_0       TTAACCCTTCTAGGTAATACAGGAGCAGGAATTTCATCAATTCTTGTAAGAATCTTAATT   1835
m150501_iid=3748029_0      TTAACCCTTCTAGGTAATACAGGAGCAGGAATTTCATCAATTCTTGTAAGAATCTTAATT   2376
m150504_iid=2876272_0      TTAACCCTTCTAGGTAATACAGGAGCAGGAATTTCATCAATTCTTGTAAGAATCTTAATT   4240
NC_042820.1                TTAACCCTTCTAGGTAATACAGGAGCAGGAATTTCATCAATTCTTGTAAGAATCTTAATT   4483
m141102_iid=1228825_0      TTAACCCTTCTAGGTAATACAGGAGCAGGAATTTCATCAATTCTTGTAAGAATCTTAATT   4310
m150504_iid=2876274_0      TTAACCCTTCTAGGTAATACAGGAGCAGGAATTTCATCAATTCTTGTAAGAATCTTAATT   4313
m141030_iid=80036_0        TTAACCCTTCTAGGTAATACAGGAGCAGGAATTTCATCAATTCTTGTAAGAATCTTAATT   4075
m150506_iid=2596494_0      TTAACCCTTCTAGGTAATACAGGAGCAGGAATTTCATCAATTCTTGTAAGAATCTTAATT   4478
m150503_iid=2526862_0      TTAACCCTTCTAGGTAATACAGGAGCAGGAATTTCATCAATTCTTGTAAGAATCTTAATT   4162
m150506_iid=2567000_0      TTAACCCTTCTAGGTAATACAGGAGCAGGAATTTCATCAATTCTTGTAAGAATCTTAATT   2111
m150507_iid=3073271_0      TTAACCCTTCTAGGTAATACAGGAGCAGGAATTTCATCAATTCTTGTAAGAATCTTAATT   3415
m150504_iid=3156087_0      TTAACCCTTCTAGGTAATACAGGAGCAGGAATTTCATCAATTCTTGTAAGAATCTTAATT   3238
m150505_iid=2498502_0      TTAACCCTTCTAGGTAATACAGGAGCAGGAATTTCATCAATTCTTGTAAGAATCTTAATT   889
m141102_iid=754227_0       TTAACCCTTCTAGGTAATACAGGAGCAGGAATTTCATCAATTCTTGTAAGAATCTTAATT   3450
m150507_iid=3469514_0      TTAACCCTTCTAGGTAATACAGGAGCAGGAATTTCATCAATTCTTGTAAGAATCTTAATT   4008
Rdo_MitoGenome             TTAACCCTTCTAGGTAATACAGGAGCAGGAATTTCATCAATTCTTGTAAGAATCTTAATT   4483
m141101_iid=614933_0       TTAACCCTTCTAGGTAATACAGGAGCAGGAATTTCATCAATTCTTGTAAGAATCTTAATT   3035
```

atp6

```
m141031_iid=347546_0       ------------------------------------------------------------   0
m150506_iid=3127414_0      TT-AACTCAAT--TTTACTTTTAGTTCTAGAAT-AGCAGTAGCAATTATTCAATCAATAC   1018
m141102_iid=1245517_0      TT-AACTCAAAT-TTTACTTTAGTTTCTAGAATCAGCAGTAGCAATTATTCAATC-ATAC   2666
m141107_iid=1405727_0      ------------------------------------------------------------   0
m141102_iid=987614_0       TT-AACTCAAAT-TTTACTTTTAGTTCTAGAATCAGCAGTAGCAATTATTCAATC-ATAC   2816
m141108_iid=1701429_0      ------------------------------------------------------------   0
m141102_iid=987286_0       TTTAACTCA-AATTTTACTTTTAGTTCTAGAATCAGCAGTAGCAATTATTCAATC-ATAC   766
m150430_iid=3272125_0      TTTAACTCA-AATTTTACTTTTAGTTCTAGAATCAGCAGTAGCAATTATTCAATC-ATAC   2288
m141102_iid=754225_0       TT-AACTCAAAT-TTTACTTTTAGTTCTAGAATCAGCA---GCAATTATTCAATC-ATAC   3453
m141107_iid=1409934_0      TT-AACTCAAAT-TTTACTTTTAGTTCTAGAATCAGCAGTAGCAATTATTCAATC-ATAC   3989
m150503_iid=3416920_0      TT-AACTCAAAT-TTTACTTTTAGTTCTAGAATCAGCAGTAGCAATTATTCAATC-ATAC   2720
m141108_iid=1800121_0      TT-AACTCAAATTTTTACTTTTAGTTCTAGAATCAGCAGTAGCAATTATTCAATC-ATAC   2531
m141031_iid=147540_0       TT-AACTCAAAT-TTTACTTTTAGTTCTAGAATCAGCAGTAGCAATTATTCAATC-ATAC   2872
m150507_iid=3674924_0      TT-AACTCAAAT-TTTACTTTTAGTTCTAGAATCAGCAGTAGCAATTATTCAATC-ATAC   3020
m150503_iid=2923751_0      TT-AACTCAAAT-TTTACTTTTAGTTCTAGAATCAGCAGTAGCAATTATTCAATC-ATAC   2867
m141031_iid=115118_0       TT-AACTCAAAT-TTTACTTTTAGTTCTAGAATCAGCAGTAGCAATTATTCAATC-ATAC   1717
m150430_iid=2722448_0      TT-AACTCAAAT-TTTACTTTTAGTTCTAGAATCAGCAGTAGCAATTATTCAATC-ATAC   1662
m150507_iid=3102647_0      ------------------------------------------------------------   0
m141108_iid=1811866_0      TT-AACTCAAAT-TTTACTTTTAGTTCTAGAATCAGCAGTAGCAATTATTCAATC-ATAC   1836
m150501_iid=2948495_0      TT-AACTCAAAT-TTTACTTTTAGTTCTAGAATCAGCAGTAGCAATTATTCAATC-ATAC   1261
m150504_iid=3238195_0      TT-AACTCAAAT-TTTACTTTTAGTTCTAGAATCAGCAGTAGCAATTATTCAATC-ATAC   3591
m150503_iid=3388511_0      TT-AACTCAAAT-TTTACTTTTAGTTCTAGAATCAGCAGTAGCAATTATTCAATC-ATAC   1288
m150505_iid=3777611_0      TT-AACTCAAAT-TTTACTTTTAGTTCTAGAATCAGCAGTAGCAATTATTCAATC-ATAC   2706
m150430_iid=2438833_0      TT-AACTCAAAT-TTTACTTTTAGTTCTAGAATCAGCAGTAGCAATTATTCAATC-ATAC   3049
m141108_iid=1698969_0      TT-AACTCAAAT-TTTACTTTTAGTTCTAGAATCAGCAGTAGCAATTATTCAATC-ATAC   4173
m150430_iid=3059190_0      ------------------------------------------------------------   0
m141107_iid=1383067_0      ------------------------------------------------------------   0
m141101_iid=597008_0       ------------------------------------------------------------   0
m150506_iid=2596496_0      TT-AACTCAAAT-TTTACTTTTAGTTCTAGAATCAGCAGTAGCAATTATTCAATC-ATAC   4533
```

m141102_iid=910051_0	TT-AACTCAAAT-TTTACTTTTAGTTCTAGAATCAGCAGTAGCAATTATTCAATC-ATAC	4023
m150505_iid=3784017_0	TT-AACTCAAAT-TTTACTTTTAGTTCTAGAATCAGCAGTAGCAATTATTCAATC-ATAC	2821
m141101_iid=682151_0	TT-AACTCAAAT-TTTACTTTTAGTTCTAGAATCAGCAGTAGCAATTATTCAATC-ATAC	1892
m150501_iid=3748029_0	TT-AACTCAAAT-TTTACTTTTAGTTCTAGAATCAGCAGTAGCAATTATTCAATC-ATAC	2433
m150504_iid=2876272_0	TT-AACTCAAAT-TTTACTTTTAGTTCTAGAATCAGCAGTAGCAATTATTCAATC-ATAC	4297
NC_042820.1	TT-AACTCAAAT-TTTACTTTTAGTTCTAGAATCAGCAGTAGCAATTATTCAATC-ATAC	4540
m141102_iid=1228825_0	TT-AACTCAAAT-TTTACTTTTAGTTCTAGAATCAGCAGTAGCAATTATTCAATC-ATAC	4367
m150504_iid=2876274_0	TT-AACTCAAAT-TTTACTTTTAGTTCTAGAATCAGCAGTAGCAATTATTCAATC-ATAC	4370
m141030_iid=80036_0	TT-AACTCAAAT-TTTACTTTTAGTTCTAGAATCAGCAGTAGCAATTATTCAATC-ATAC	4132
m150506_iid=2596494_0	TT-AACTCAAAT-TTTACTTTTAGTTCTAGAATCAGCAGTAGCAATTATTCAATC-ATAC	4535
m150503_iid=2526862_0	TT-AACTCAAAT-TTTACTTTTAGTTCTAGAATCAGCAGTAGCAATTATTCAATC-ATAC	4219
m150506_iid=2567000_0	TT-AACTCAAAT-TTTACTTTTAGTTCTAGAATCAGCAGTAGCAATTATTCAATC-ATAC	2168
m150507_iid=3073271_0	TT-AACTCAAAT-TTTACTTTTAGTTCTAGAATCAGCAGTAGCAATTATTCAATC-ATAC	3472
m150504_iid=3156087_0	TT-AACTCAAAT-TTTACTTTTAGTTCTAGAATCAGCAGTAGCAATTATTCAATC-ATAC	3295
m150505_iid=2498502_0	TT-AACTCAAAT-TTTACTTTTAGTTCTAGAATCAGCAGTAGCAATTATTCAATC-ATAC	946
m141102_iid=754227_0	TT-AACTCAAAT-TTTACTTTTAGTTCTAGAATCAGCAGTAGCAATTATTCAATC-ATAC	3507
m150507_iid=3469514_0	TT-AACTCAAAT-TTTACTTTTAGTTCTAGAATCAGCAGTAGCAATTATTCAATC-ATAC	4065
Rdo_MitoGenome	TT-AACTCAAAT-TTTACTTTTAGTTCTAGAATCAGCAGTAGCAATTATTCAATC-ATAC	4540
m141101_iid=614933_0	TT-AACTCAAAT-TTTACTTTTAGTTCTAGAATCAGCAGTAGCAATTATTCAATC-ATAC	3092

atp6

m141031_iid=347546_0	--	0
m150506_iid=3127414_0	GTATTTGCAATTTTAAGA-ACCCTTTATTCAAGAGAAGTAA--TAATGACAAAAAAAAAT	1075
m141102_iid=1245517_0	GTATTTGCAATTTTAAGA-ACCCTTTATTCAAGAGAAGTAAACTAATGACAAAA-AAAT	2724
m141107_iid=1405727_0	--	0
m141102_iid=987614_0	GTATTTGCAATTTTAAGA-ACCCTTTATTCAAGAGAAGTAAACTAATGACAAAAAAAAAT	2875
m141108_iid=1701429_0	--	0
m141102_iid=987286_0	GTATTTGCAATTTTAAGA-ACCCTTTATTCAAGAGAAGTAAACTAATGACAAAA-AAAT	824
m150430_iid=3272125_0	GTATTTGCAATTTTAAGAAACCCTTTATTCAAGAGAAGTAAACTAATGACAAAAAAAAAT	2348
m141102_iid=754225_0	GTATTTGCAATTTTAAGA-ACCCTTTATTCAAGAGAAGTAAACTAATGACAAAAAAAAAT	3512
m141107_iid=1409934_0	GTATTTGCAATTTTAAGA-ACCCTTTATTCAAGAGAAGTAAACTAATGACAAAAAAAAAT	4048
m150503_iid=3416920_0	GTATTTGCAATTTTAAGA-ACCCTTTATTCAAGAGAAGTAAACTAATGACAAAAAAAAAT	2779
m141108_iid=1800121_0	GTATTTGCAATTTTAAGA-ACCCTTTATTCAAGAGAAGTAAACTAATGACAAAAAAAAAT	2590
m141031_iid=147540_0	GTATTTGCAATTTTAAGA-ACCCTTTATTCAAGAGAAGTAAACTAATGACAAAAAAAAAT	2931
m150507_iid=3674924_0	GTATTTGCAATTTTAAGA-ACCCTTTATTCAAGAGAAGTAAACTAATGACAAAAAAAAAT	3079
m150503_iid=2923751_0	GTATTTGCAATTTTAAGA-ACCCTTTATTCAAGAGAAGTAAACTAATGACAAAAAAAAAT	2926
m141031_iid=115118_0	GTATTTGCAATTTTAAGA-ACCCTTTATTCAAGAGAAGTAAACTAATGACAAAAAAAAAT	1776
m150430_iid=2722448_0	GTATTTGCAATTTTAAGA-ACCCTTTATTCAAGAGAAGTAAACTAATGACAAAAAAAAAT	1721
m150507_iid=3102647_0	---------------------------------------AAACTAATGACAAAAAAAAAT	21
m141108_iid=1811866_0	GTATTTGCAATTTTAAGA-ACCCTTTATTCAAGAGAAGTAAACTAATGACAAAAAAAAAT	1895
m150501_iid=2948495_0	GTATTTGCAATTTTAAGA-ACCCTTTATTCAAGAGAAGTAAACTAATGACAAAA-AAAT	1319
m150504_iid=3238195_0	GTATTTGCAATTTTAAGA-ACCCTTTATTCAAGAGAAGTAAACTAATGACAAAAAAAAAT	3650
m150503_iid=3388511_0	GTATTTGCAATTTTAAGA-ACCCTTTATTCAAGAGAAGTAAACTAATGACAAAAAAAAAT	1347
m150505_iid=3777611_0	GTATTTGCAATTTTAAGA-ACCCTTTATTCAAGAGAAGTAAACTAATGACAAAAAAAAAT	2765
m150430_iid=2438833_0	GTATTTGCAATTTTAAGA-A-CCTTTATTCAAGAGAAGTAAACTAATGAC-AAAAAAAAT	3106
m141108_iid=1698969_0	GTATTTGCAATTTTAAGA-ACCCTTTATTCAAGAGAAGTAAACTAATGACAAAAAAAAAT	4232
m150430_iid=3059190_0	--	0
m141107_iid=1383067_0	--	0
m141101_iid=597008_0	--	0
m150506_iid=2596496_0	GTATTTGCAATTTTAAGA-ACCCTTTATTCAAGAGAAGTAAACTAATGACAAAAAAAAAT	4592
m141102_iid=910051_0	GTATTTGCAATTTTAAGA-ACCCTTTATTCAAGAGAAGTAAACTAATGACAAAA-AAAT	4081
m150505_iid=3784017_0	GTATTTGCAATTTTAAGA-ACCCTTTATTCAAGAGAAGTAAACTAATGACAAAAAAAAAT	2880
m141101_iid=682151_0	GTATTTGCAATTTTAAGA-ACCCTTTATTCAAGAGAAGTAAACTAATGACAAAAAAAAAT	1951
m150501_iid=3748029_0	GTATTTGCAATTTTAAGA-ACCCTTTATTCAAGAGAAGTAAACTAATGACAAAAAAAAAT	2492
m150504_iid=2876272_0	GTATTTGCAATTTTAAGA-ACCCTTTATTCAAGAGAAGTAAACTAATGACAAAAAAAAAT	4356
NC_042820.1	GTATTTGCAATTTTAAGA-ACCCTTTATTCAAGAGAAGTAAACTAATGACAAAAAAAAAT	4599
m141102_iid=1228825_0	GTATTTGCAATTTTAAGA-ACCCTTTATTCAAGAGAAGTAAACTAATGACAAAAAAAAAT	4426
m150504_iid=2876274_0	GTATTTGCAATTTTAAGA-ACCCTTTATTCAAGAGAAGTAAACTAATGACAAAAAAAAAT	4429
m141030_iid=80036_0	GTATTTGCAATTTTAAGA-ACCCTTTATTCAAGAGAAGTAAACTAATGACAAAAAAAAAT	4191
m150506_iid=2596494_0	GTATTTGCAATTTTAAGA-ACCCTTTATTCAAGAGAAGTAAACTAATGACAAAAAAAAAT	4594
m150503_iid=2526862_0	GTATTTGCAATTTTAAGA-ACCCTTTATTCAAGAGAAGTAAACTAATGACAAAAAAAAAT	4278
m150506_iid=2567000_0	GTATTTGCAATTTTAAGA-ACCCTTTATTCAAGAGAAGTAAACTAATGACAAAAAAAAAT	2227
m150507_iid=3073271_0	GTATTTGCAATTTTAAGA-ACCCTTTATTCAAGAGAAGTAAACTAATGACAAAAAAAAAT	3531
m150504_iid=3156087_0	GTATTTGCAATTTTAAGA-ACCCTTTATTCAAGAGAAGTAAACTAATGACAAAAAAAAAT	3354
m150505_iid=2498502_0	GTATTTGCAATTTTAAGA-ACCCTTTATTCAAGAGAAGTAAACTAATGACAAAA-AAAT	1004
m141102_iid=754227_0	GTATTTGCAATTTTAAGA-ACCCTTTATTCAAGAGAAGTAAACTAATGACAAAAAAAAAT	3566
m150507_iid=3469514_0	GTATTTGCAATTTTAAGA-ACCCTTTATTCAAGAGAAGTAAACTAATGACAAAAAAAAAT	4124
Rdo_MitoGenome	GTATTTGCAATTTTAAGA-ACCCTTTATTCAAGAGAAGTAAACTAATGACAAAAAAAAAT	4599
m141101_iid=614933_0	GTATTTGCAATTTTAAGA-ACCCTTTATTCAAGAGAAGTAAACTAATGACAAAAAAAAAT	3151

atp6 *cox3*

```
m141031_iid=347546_0      ------------------------------------------------------------   0
m150506_iid=3127414_0     CACCCTTTTC-CTTAGTAGATGT------AAGACCATGACCAATTCTTGGAGCATTAAGA   1128
m141102_iid=1245517_0     CACCCTTTTCACTTAGTAGATGT------AAGACCATGACCAATTCTTGGAGCATTAAGA   2778
m141107_iid=1405727_0     ------------------------------------------------------------   0
m141102_iid=987614_0      CACCCTTTTCACTTAGTAGATGT------AAGACCATGACCAATTCTTGGAGCATTAAGA   2929
m141108_iid=1701429_0     ------------------------------------------------------------   0
m141102_iid=987286_0      CACCCTTTTCACTTAGTAGATGT------AAGACCATGACCAATTCTTGGAGCATTAAGA   878
m150430_iid=3272125_0     CACCCTTTTCACTTAGTAGATGT------AAGACCATGACCAATTCTTGGAGCATTAAGA   2402
m141102_iid=754225_0      CACCCTTTTCACTTAGTAGATGT------AAGACCATGACCAATTCTTGGAGCATTAAGA   3566
m141107_iid=1409934_0     CACCCTTTTCACTTAGTAGATTTGTGATTGAGACCATGACCAATTCTTGGAGCATTAAGA   4108
m150503_iid=3416920_0     CACCCTTTTCACTTAGTAGATGT------AAGACCATGACCAATTCTTGGAGCATTAAGA   2833
m141108_iid=1800121_0     CACCCTTTTCACTTAGTAGATGT------AAGACCATGACCAATTCTTGGAGCATTAAGA   2644
m141031_iid=147540_0      CACCCTTTTCACTTAGTAGATGT------AAGACCATGACCAATTCTTGGAGCATTAAGA   2985
m150507_iid=3674924_0     CACCCTTTTCACTTAGTAGATGT------AAGACCATGACCAATTCTTGGAGCATTAAGA   3133
m150503_iid=2923751_0     CACCCTTTTCACTTAGTAGATGT------AAGACCATGACCAATTCTTGGAGCATTAAGA   2980
m141031_iid=115118_0      CACCCTTTTCACTTAGTAGATGT------AAGACCATGACCAATTCTTGGAGCATTAAGA   1830
m150430_iid=2722448_0     CACCCTTTTCACTTAGTAGATGT------AAGACCATGACCAATTCTTGGAGCATTAAGA   1775
m150507_iid=3102647_0     CACCCTTTTCACTTAGTAGATGT------AAGACCATGACCAATTCTTGGAGCATTAAGA   75
m141108_iid=1811866_0     CACCCTTTTCACTTAGTAGATGT------AAGACCATGACCAATTCTTGGAGCATTAAGA   1949
m150501_iid=2948495_0     CACCCTTTTCACTTAGTAGATGT------AAGACCATGACCAATTCTTGGAGCATTAAGA   1373
m150504_iid=3238195_0     CACCCTTTTCACTTAGTAGATGT------AAGACCATGACCAATTCTTGGAGCATTAAGA   3704
m150503_iid=3388511_0     CACCCTTTTCACTTAGTAGATGT------AAGACCATGACCAATTCTTGGAGCATTAAGA   1401
m150505_iid=3777611_0     CACCCTTTTCACTTAGTAGATGT------AAGACCATGACCAATTCTTGGAGCATTAAGA   2819
m150430_iid=2438833_0     CACCCTTTTCACTTAGTAGATGT------AAGACCATGACCAATTCTTGGAGCATTAAGA   3160
m141108_iid=1698969_0     CACCCTTTTCACTTAGTAGATGT------AAGACCATGACCAATTCTTGGAGCATTAAGA   4286
m150430_iid=3059190_0     ---------------------------------------------------GCATTAAGA   9
m141107_iid=1383067_0     ----------------------------------------CAATTCTTGGAGCATTAAGA   20
m141101_iid=597008_0      ------------------------------------------------------------   0
m150506_iid=2596496_0     CACCCTTTTCACTTAGTAGATGT------AAGACCATGACCAATTCTTGGAGCATTAAGA   4646
m141102_iid=910051_0      CACCCTTTTCACTTAGTAGATGT------AAGACCATGACCAATTCTTGGAGCATTAAGA   4135
m150505_iid=3784017_0     CACCCTTTTCACTTAGTAGATGT------AAGACCATGACCAATTCTTGGAGCATTAAGA   2934
m141101_iid=682151_0      CACCCTTTTCACTTAGTAGATGT------AAGACCATGACCAATTCTTGGAGCATTAAGA   2005
m150501_iid=3748029_0     CACCCTTTTCACTTAGTAGATGT------AAGACCATGACCAATTCTTGGAGCATTAAGA   2546
m150504_iid=2876272_0     CACCCTTTTCACTTAGTAGATGT------AAGACCATGACCAATTCTTGGAGCATTAAGA   4410
NC_042820.1               CACCCTTTTCACTTAGTAGATGT------AAGACCATGACCAATTCTTGGAGCATTAAGA   4653
m141102_iid=1228825_0     CACCCTTTTCACTTAGTAGATGT------AAGACCATGACCAATTCTTGGAGCATTAAGA   4480
m150504_iid=2876274_0     CACCCTTTTCACTTAGTAGATGT------AAGACCATGACCAATTCTTGGAGCATTAAGA   4483
m141030_iid=80036_0       CACCCTTTTCACTTAGTAGATGT------AAGACCATGACCAATTCTTGGAGCATTAAGA   4245
m150506_iid=2596494_0     CACCCTTTTCACTTAGTAGATGT------AAGACCATGACCAATTCTTGGAGCATTAAGA   4648
m150503_iid=2526862_0     CACCCTTTTCACTTAGTAGATGT------AAGACCATGACCAATTCTTGGAGCATTAAGA   4332
m150506_iid=2567000_0     CACCCTTTTCACTTAGTAGATGT------AAGACCATGACCAATTCTTGGAGCATTAAGA   2281
m150507_iid=3073271_0     CACCCTTTTCACTTAGTAGATGT------AAGACCATGACCAATTCTTGGAGCATTAAGA   3585
m150504_iid=3156087_0     CACCCTTTTCACTTAGTAGATGT------AAGACCATGACCAATTCTTGGAGCATTAAGA   3408
m150505_iid=2498502_0     CACCCTTTTCACTTAGTAGATGT------AAGACCATGACCAATTCTTGGAGCATTAAGA   1058
m141102_iid=754227_0      CACCCTTTTCACTTAGTAGATGT------AAGACCATGACCAATTCTTGGAGCATTAAGA   3620
m150507_iid=3469514_0     CACCCTTTTCACTTAGTAGATGT------AAGACCATGACCAATTCTTGGAGCATTAAGA   4178
Rdo_MitoGenome            CACCCTTTTCACTTAGTAGATGT------AAGACCATGACCAATTCTTGGAGCATTAAGA   4653
m141101_iid=614933_0      CACCCTTTTCACTTAGTAGATGT------AAGACCATGACCAATTCTTGGAGCATTAAGA   3205
```

cox3 →

```
m141031_iid=347546_0      ------------------------------------------------------------   0
m150506_iid=3127414_0     GCCATAT-CACAATAGTAGGATTAATTAAATGATTTCATATATATCAAGTAAACCTTTTT   1187
m141102_iid=1245517_0     GCCATATCCACAATAGTAGGATTAATTAAATGATTTCATATATATCAAGTAAACCTTTTT   2838
m141107_iid=1405727_0     -------------------------------------ATATATATCAAGTAAACCTTTTT   23
m141102_iid=987614_0      GCCATATCCACAATAGTAGGATTAATTAAATGATTTCATATATATCAAGTAAACCTTTTT   2989
m141108_iid=1701429_0     ------------------------------------------------------------   0
m141102_iid=987286_0      GCCATATCCACAATAGTAGGATTAATTAAATGATTTCATATATATCAAGTAAACCTTTTT   938
m150430_iid=3272125_0     GCCATATCCACAATAGTAGGATTAATTAAATGATTTCATATATATCAAGTAAACCTTTTT   2462
m141102_iid=754225_0      GCCATATCCACAATAGTAGGATTAATTAAATGATTTCATATATATCAAGTAAACCTTTTT   3626
m141107_iid=1409934_0     GCCATATCCACAATAGTAGGATTAATTAAATGATTTCATATATATCAAGTAAACCTTTTT   4168
m150503_iid=3416920_0     GCCATATCCACAATAGTAGGATTAATTAAATGATTTCATATATATCAAGTAAACCTTTTT   2893
m141108_iid=1800121_0     GCCATATCCACAATAGTAGGATTAATTAAATGATTTCATATATATCAAGTAAACCTTTTT   2704
m141031_iid=147540_0      GCCATATCCACAATAGTAGGATTAATTAAATGATTTCATATATATCAAGTAAACCTTTTT   3045
m150507_iid=3674924_0     GCCATATCCACAATAGTAGGATTAATTAAATGATTTCATATATATCAAGTAAACCTTTTT   3193
m150503_iid=2923751_0     GCCATATCCACAATAGTAGGATTAATTAAATGATTTCATATATATCAAGTAAACCTTT-T   3039
m141031_iid=115118_0      GCCATATCCACAATAGTAGGATTAATTAAATGATTTCATATATATCAAGTAAACCTTTTT   1890
m150430_iid=2722448_0     GCCATATCCACAATAGTAGGATTAATTAAATGATTTCATATATATCAAGTAAACCTTT-T   1834
m150507_iid=3102647_0     GCCATATCCACAATAGTAGGATTAATTAAATGATTTCATATATATCAAGTAAACCTTTTT   135
m141108_iid=1811866_0     GCCATATCCACAATAGTAGGATTAATTAAATGATTTCATATATATCAAGTAAACCTTTTT   2009
m150501_iid=2948495_0     GCCATATCCACAATAGTAGGATTAATTAAATGATTTCATATATATCAAGTAAACCTTTTT   1433
m150504_iid=3238195_0     GCCATATCCACAATAGTAGGATTAATTAAATGATTTCATATATATCAAGTAAACCTTTTT   3764
```

```
m150503_iid=3388511_0      GCCATATCCACAATAGTAGGATTAATTAAATGATTTCATATATATCAAGTAAACCTTTTT   1461
m150505_iid=3777611_0      GCCATATCCACAATAGTAGGATTAATTAAATGATTTCATATATATCAAGTAAACCTTTTT   2879
m150430_iid=2438833_0      GCCATATCCACAATAGTAGGATTAATTAAATGATTTCATATATATCAAGTAAACCTTTTT   3220
m141108_iid=1698969_0      GCCATATCCACAATAGTAGGATTAATTAAATGATTTCATATATATCAAGTAAACCTTTTT   4346
m150430_iid=3059190_0      GCCATATCCACAATAGTAGGATTAATTAAATGATTTCATATATATCAAGTAAACCTTTTT   69
m141107_iid=1383067_0      GCCATATCCACAATAGTAGGATTAATTAAATGATTTCATATATATCAAGTAAACTT--TT   78
m141101_iid=597008_0       ------------------------------------------------------------   0
m150506_iid=2596496_0      GCCATATCCACAATAGTAGGATTAATTAAATGATTTCATATATATCAAGTAAACCTTTTT   4706
m141102_iid=910051_0       GCCATATCCACAATAGTAGGATTAATTAAATGATTTCATATATATCAAGTAAACCTTTTT   4195
m150505_iid=3784017_0      GCCATATCCACAATAGTAGGATTAATTAAATGATTTCATATATATCAAGTAAACCTTTTT   2994
m141101_iid=682151_0       GCCATATCCACAATAGTAGGATTAATTAAATGATTTCATATATATCAAGTAAACCTTTTT   2065
m150501_iid=3748029_0      GCCATATCCACAATAGTAGGATTAATTAAATGATTTCATATATATCAAGTAAACCTTTTT   2606
m150504_iid=2876272_0      GCCATATCCACAATAGTAGGATTAATTAAATGATTTCATATATATCAAGTAAACCTTTTT   4470
NC_042820.1                GCCATATCCACAATAGTAGGATTAATTAAATGATTTCATATATATCAAGTAAACCTTTTT   4713
m141102_iid=1228825_0      GCCATATCCACAATAGTAGGATTAATTAAATGATTTCATATATATCAAGTAAACCTTTTT   4540
m150504_iid=2876274_0      GCCATATCCACAATAGTAGGATTAATTAAATGATTTCATATATATCAAGTAAACCTTTTT   4543
m141030_iid=80036_0        GCCATATCCACAATAGTAGGATTAATTAAATGATTTCATATATATCAAGTAAACCTTTTT   4305
m150506_iid=2596494_0      GCCATATCCACAATAGTAGGATTAATTAAATGATTTCATATATATCAAGTAAACCTTTTT   4708
m150503_iid=2526862_0      GCCATATCCACAATAGTAGGATTAATTAAATGATTTCATATATATCAAGTAAACCTTTTT   4392
m150506_iid=2567000_0      GCCATATCCACAATAGTAGGATTAATTAAATGATTTCATATATATCAAGTAAACCTTTTT   2341
m150507_iid=3073271_0      GCCATATCCACAATAGTAGGATTAATTAAATGATTTCATATATATCAAGTAAACCTTTTT   3645
m150504_iid=3156087_0      GCCATATCCACAATAGTAGGATTAATTAAATGATTTCATATATATCAAGTAAACCTTTTT   3468
m150505_iid=2498502_0      GCCATATCCACAATAGTAGGATTAATTAAATGATTTCATATATATCAAGTAAACCTTTTT   1118
m141102_iid=754227_0       GCCATATCCACAATAGTAGGATTAATTAAATGATTTCATATATATCAAGTAAACCTTTTT   3680
m150507_iid=3469514_0      GCCATATCCACAATAGTAGGATTAATTAAATGATTTCATATATATCAAGTAAACCTTTTT   4238
Rdo_MitoGenome             GCCATATCCACAATAGTAGGATTAATTAAATGATTTCATATATATCAAGTAAACCTTTTT   4713
m141101_iid=614933_0       GCCATATCCACAATAGTAGGATTAATTAAATGATTTCATATATATCAAGTAAACCTTTTT   3265
```

cox3

```
m141031_iid=347546_0       ------------------------------------------------------------   0
m150506_iid=3127414_0      TTGGTAGGT-TTACTTTCTACATCTCTTATTATATATCAATGAT--GACGAGATATCACA   1244
m141102_iid=1245517_0      TTTGGTAGGTTTACTTTCTACATCTCTTATTATATATCAATGAT--GACGAGATATCACA   2896
m141107_iid=1405727_0      TTGGTAGGTTTTACTTTCTACATCTCTTATTATATATCAATGAT--GACGAGATATCACA   81
m141102_iid=987614_0       TTGGTAGGT-TTACTTTCTACATCTCTTATTATATATCAATGAT--GACGAGATATCACA   3046
m141108_iid=1701429_0      ------------------------------------------------------------   0
m141102_iid=987286_0       TTGGTAGGT-TTACTTTCTACATCTCTTATTATATATCAA-T-GATGACGAGATATCACA   995
m150430_iid=3272125_0      TTGGTAGGT-TTACTTTCTACATCTCTTATTATATATCAATT-GATGACG--ATATCACA   2518
m141102_iid=754225_0       TTGGTAGGT-TTACTTTCTACATCTCTTATTATATATCAATGAT--GACGAGATATCACA   3683
m141107_iid=1409934_0      TTGGTAGGT-TTACTTTCTACATCTCTTATTATATATCAATGAT--GACGAGATATCACA   4225
m150503_iid=3416920_0      TTGGTAGGT-TTACTTTCTACATCTCTTATTATATATCAATGAT--GACGAGATATCACA   2950
m141108_iid=1800121_0      TTGGTAGGT-TTACTTTCTACATCTCTTATTATATATCAATGAT--GACGAGATATCACA   2761
m141031_iid=147540_0       TTGGTAGGT-TTACTTTCTACATCTCTTATTATATATCAATGAT--GACGAGATATCACA   3102
m150507_iid=3674924_0      TTGGTAGGT-TTACTTTCTACATCTCTTATTATATATCAATGATGAGACGAGATATCACA   3252
m150503_iid=2923751_0      TTGGTAGGT-TTACTTTCTACATCTCTTATTATATATCAATGAT--GACGAGATATCACA   3096
m141031_iid=115118_0       TTGGTAGGT-TTACTTTCTACATCTCTTATTATATATCAATGAT--GACGAGATATCACA   1947
m150430_iid=2722448_0      TTGGTAGGT-TTACTTTCTACATCTCTTATTATATATCAATGAT--GACGAGATATCACA   1891
m150507_iid=3102647_0      TTGGTAGGT-TTACTTTCTACATCTCTTATTATATATCAATGAT--GACGAGATATCACA   192
m141108_iid=1811866_0      TTGGTAGGT-TTACTTTCTACATCTCTTATTATATATCAATGAT--GACGAGATATCACA   2066
m150501_iid=2948495_0      TTGGTAGGT-TTACTTTCTACATCTCTTATTATATATCAATGAT--GACGAGATATCACA   1490
m150504_iid=3238195_0      TTGGTAGGT-TTACTTTCTACATCTCTTATTATATATCAATGAT--GACGAGATATCACA   3821
m150503_iid=3388511_0      TTGGTAGGT-TTACTTTCTACATCTCTTATTATATATCAATGAT--GACGAGATATCACA   1518
m150505_iid=3777611_0      TTGGTAGGT-TTACTTTCTACATCTCTTATTATATATCAATGAT--GACGAGATATCACA   2936
m150430_iid=2438833_0      TTGGTAGGT-TTACTTTCTACATCTCTTATTATATATCAATGAT--GACGAGATATCACA   3277
m141108_iid=1698969_0      TTGGTAGGT-TTACTTTCTACATCTCTTATTATATATCAATGAT--GACGAGATATCACA   4403
m150430_iid=3059190_0      TTGGTA-GT-TTACTTTCTACATCTCTTATTATATATCAATGAT--GACGAGATATCACA   125
m141107_iid=1383067_0      TTGGTAGGT-TTACTTTCTACATCTCTTATTATATATCAATGAT--GACGAGATATCACA   135
m141101_iid=597008_0       ------------------------------------------------------------   0
m150506_iid=2596496_0      TTGGTAGGT-TTACTTTCTACATCTCTTATTATATATCAATGAT--GACGAGATATCACA   4763
m141102_iid=910051_0       TTGGTAGGT-TTACTTTCTACATCTCTTATTATATATCAATGAT--GACGAGATATCACA   4252
m150505_iid=3784017_0      TTGGTAGGT-TTACTTTCTACATCTCTTATTATATATCAATGAT--GACGAGATATCACA   3051
m141101_iid=682151_0       TTGGTAGGT-TTACTTTCTACATCTCTTATTATATATCAATGAT--GACGAGATATCACA   2122
m150501_iid=3748029_0      TTGGTAGGT-TTACTTTCTACATCTCTTATTATATATCAATGAT--GACGAGATATCACA   2663
m150504_iid=2876272_0      TTGGTAGGT-TTACTTTCTACATCTCTTATTATATATCAATGAT--GACGAGATATCACA   4527
NC_042820.1                TTGGTAGGT-TTACTTTCTACATCTCTTATTATATATCAATGAT--GACGAGATATCACA   4770
m141102_iid=1228825_0      TTGGTAGGT-TTACTTTCTACATCTCTTATTATATATCAATGAT--GACGAGATATCACA   4597
m150504_iid=2876274_0      TTGGTAGGT-TTACTTTCTACATCTCTTATTATATATCAATGAT--GACGAGATATCACA   4600
m141030_iid=80036_0        TTGGTAGGT-TTACTTTCTACATCTCTTATTATATATCAATGAT--GACGAGATATCACA   4362
m150506_iid=2596494_0      TTGGTAGGT-TTACTTTCTACATCTCTTATTATATATCAATGAT--GACGAGATATCACA   4765
m150503_iid=2526862_0      TTGGTAGGT-TTACTTTCTACATCTCTTATTATATATCAATGAT--GACGAGATATCACA   4449
m150506_iid=2567000_0      TTGGTAGGT-TTACTTTCTACATCTCTTATTATATATCAATGAT--GACGAGATATCACA   2398
m150507_iid=3073271_0      TTGGTAGGT-TTACTTTCTACATCTCTTATTATATATCAATGAT--GACGAGATATCACA   3702
```

```
m150504_iid=3156087_0      TTGGTAGGT-TTACTTTCTACATCTCTTATTATATATCAATGAT--GACGAGATATCACA   3525
m150505_iid=2498502_0      TTGGTAGGT-TTACTTTCTACATCTCTTATTATATATCAATGAT--GACGAGATATCACA   1175
m141102_iid=754227_0       TTGGTAGGT-TTACTTTCTACATCTCTTATTATATATCAATGAT--GACGAGATATCACA   3737
m150507_iid=3469514_0      TTGGTAGGT-TTACTTTCTACATCTCTTATTATATATCAATGAT--GACGAGATATCACA   4295
Rdo_MitoGenome             TTGGTAGGT-TTACTTTCTACATCTCTTATTATATATCAATGAT--GACGAGATATCACA   4770
m141101_iid=614933_0       TTGGTAGGT-TTACTTTCTACATC[illegible]TATCAATGAT--GACGAGATATCACA   3322
                                                   cox3
m141031_iid=347546_0       -------------------------[illegible]---------------------------   0
m150506_iid=3127414_0      CGTGAAGGTT--TTTCAAGGACACCACACATTTATCGTTACTATAGGATTACGATGAGGA   1302
m141102_iid=1245517_0      CGTGAAGGTTCATTTCAAGGACACCACACATTTATCGTTACTATAGGATTACGATGAGGA   2956
m141107_iid=1405727_0      CGTGAAGGTTCATTTCAAGGACACC-CACATTTATCGTTACTATAGGATTACGATGAGGA   140
m141102_iid=987614_0       CGTGAAGGTTCATTTCAAGGACACCACACATTTATCGTTACTATAGGATTACGATGAGGA   3106
m141108_iid=1701429_0      ------------------------------------------------------------   0
m141102_iid=987286_0       CGTGAAGGTTCATTTCAAGGACACCACACATTTATCGTTACTATAGGATTACGATGAGGA   1055
m150430_iid=3272125_0      CGTGAAGGTTCATTTCAAGGACACCACACATTTATCGTTACTATAGGATTACGATGAGGA   2578
m141102_iid=754225_0       CGTGAAGGTTCATTTCAAGGACACCACACATTTATCGTTACTATAGGATTACGATGAGGA   3743
m141107_iid=1409934_0      CGTGAAGGTTCATTTCAAGGACACCACACATTTATCGTTACTATAGGATTACGATGAGGA   4285
m150503_iid=3416920_0      CGTGAAGGTTCATTTCAAGGACACCACACATTTATCGTTACTATAGGATTACGATGAGGA   3010
m141108_iid=1800121_0      CGTGAAGGTTCATTTCAAGGACACCACACATTTATCGTTACTATAGGATTACGATGAGGA   2821
m141031_iid=147540_0       CGTGAAGGTTCATTTCAAGGACACCACACATTTATCGTTACTATAGGATTACGATGAGGA   3162
m150507_iid=3674924_0      CGTGAAGGTTCATTTCAAGGACACCACACATTTATCGTTACTATAGGATTACGATGAGGA   3312
m150503_iid=2923751_0      CGTGAAGGTTCATTTCAAGGACACCACACATTTATCGTTACTATAGGATTACGATGAGGA   3156
m141031_iid=115118_0       CGTGAAGGTTCATTTCAAGGACACCACACATTTATCGTTACTATAGGATTACGATGAGGA   2007
m150430_iid=2722448_0      CGTGAAGGTTCATTTCAAGGACACCACACATTTATCGTTACTATAGGATTACGATGAGGA   1951
m150507_iid=3102647_0      CGTGAAGGTTCATTTCAAGGACACCACACATTTATCGTTACTATAGGATTACGATGAGGA   252
m141108_iid=1811866_0      CGTGAAGGTTCATTTCAAGGACACCACACATTTATCGTTACTATAGGATTACGATGAGGA   2126
m150501_iid=2948495_0      CGTGAAGGTTCATTTCAAGGACACCACACATTTATCGTTACTATAGGATTACGATGAGGA   1550
m150504_iid=3238195_0      CGTGAAGGTTCATTTCAAGGACACCACACATTTATCGTTACTATAGGATTACGATGAGGA   3881
m150503_iid=3388511_0      CGTGAAGGTTCATTTCAAGGACACCACACATTTATCGTTACTATAGGATTACGATGAGGA   1578
m150505_iid=3777611_0      CGTGAAGGTTCATTTCAAGGACACCACACATTTATCGTTACTATAGGATTACGATGAGGA   2996
m150430_iid=2438833_0      CGTGAAGGTTCATTTCAAGGACACCACACATTTATCGTTACTATAGGATTACGATGAGGA   3337
m141108_iid=1698969_0      CGTGAAGGTTCATTTCAAGGACACCACACATTTATCGTTACTATAGGATTACGATGAGGA   4463
m150430_iid=3059190_0      CGTGAAGGTTCATTTCAAGGACACCACACATTTATCGTTACTATAGGATTACGATGAGGA   185
m141107_iid=1383067_0      CGTGAAGGTTCATTTCAAGGACACCACACATTTATCGTTACTATAGGATTACGATGAGGA   195
m141101_iid=597008_0       ------------------------------------------------------------   0
m150506_iid=2596496_0      CGTGAAGGTTCATTTCAAGGACACCACACATTTATCGTTACTATAGGATTACGATGAGGA   4823
m141102_iid=910051_0       CGTGAAGGTTCATTTCAAGGACACCACACATTTATCGTTACTATAGGATTACGATGAGGA   4312
m150505_iid=3784017_0      CGTGAAGGTTCATTTCAAGGACACCACACATTTATCGTTACTATAGGATTACGATGAGGA   3111
m141101_iid=682151_0       CGTGAAGGTTCATTTCAAGGACACCACACATTTATCGTTACTATAGGATTACGATGAGGA   2182
m150501_iid=3748029_0      CGTGAAGGTTCATTTCAAGGACACCACACATTTATCGTTACTATAGGATTACGATGAGGA   2723
m150504_iid=2876272_0      CGTGAAGGTTCATTTCAAGGACACCACACATTTATCGTTACTATAGGATTACGATGAGGA   4587
NC_042820.1                CGTGAAGGTTCATTTCAAGGACACCACACATTTATCGTTACTATAGGATTACGATGAGGA   4830
m141102_iid=1228825_0      CGTGAAGGTTCATTTCAAGGACACCACACATTTATCGTTACTATAGGATTACGATGAGGA   4657
m150504_iid=2876274_0      CGTGAAGGTTCATTTCAAGGACACCACACATTTATCGTTACTATAGGATTACGATGAGGA   4660
m141030_iid=80036_0        CGTGAAGGTTCATTTCAAGGACACCACACATTTATCGTTACTATAGGATTACGATGAGGA   4422
m150506_iid=2596494_0      CGTGAAGGTTCATTTCAAGGACACCACACATTTATCGTTACTATAGGATTACGATGAGGA   4825
m150503_iid=2526862_0      CGTGAAGGTTCATTTCAAGGACACCACACATTTATCGTTACTATAGGATTACGATGAGGA   4509
m150506_iid=2567000_0      CGTGAAGGTTCATTTCAAGGACACCACACATTTATCGTTACTATAGGATTACGATGAGGA   2458
m150507_iid=3073271_0      CGTGAAGGTTCATTTCAAGGACACCACACATTTATCGTTACTATAGGATTACGATGAGGA   3762
m150504_iid=3156087_0      CGTGAAGGTTCATTTCAAGGACACCACACATTTATCGTTACTATAGGATTACGATGAGGA   3585
m150505_iid=2498502_0      CGTGAAGGTTCATTTCAAGGACACCACACATTTATCGTTACTATAGGATTACGATGAGGA   1235
m141102_iid=754227_0       CGTGAAGGTTCATTTCAAGGACACCACACATTTATCGTTACTATAGGATTACGATGAGGA   3797
m150507_iid=3469514_0      CGTGAAGGTTCATTTCAAGGACACCACACATTTATCGTTACTATAGGATTACGATGAGGA   4355
Rdo_MitoGenome             CGTGAAGGTTCATTTCAAGGACACCACACATTTATCGTTACTATAGGATTACGATGAGGA   4830
m141101_iid=614933_0       CGTGAAGGTTCATTTCAAGGACACCACACATTTATCGTTACTATAGGATTACGATGAGGA   3382

                                                   cox3
m141031_iid=347546_0       ------------------------------------------------------------   0
m150506_iid=3127414_0      ATAA-TTTTATTTATTACATCAGAAGTATTTTTCTTCATTTCATTTTTT--TGAGGATTT   1359
m141102_iid=1245517_0      ATAA-TTTTATTTATTACATCAGAAGTATTTTTCTTCATTTCATTTTTT--TGAGGATTT   3013
m141107_iid=1405727_0      TATAATTTTATTTATTACATCAGAAGTATTTTTCTTCATTTCATTTTTT-TGA-GGATTT   198
m141102_iid=987614_0       ATAA-TTTTATTTATTACATCAGAAGTATTTTTCTTCATTTCATTTTT--TGAGG-ATTT   3162
m141108_iid=1701429_0      ------------------------------------------------------------   0
m141102_iid=987286_0       ATAA-TTTTATTTATTACATCAGAAGTATTTTTCTTCATTTCATTTTT--TTGAGGATTT   1112
m150430_iid=3272125_0      ATAA-TTTTATTTATTACATCAGAAGTATTTTTCTTCATTTCATTTTT--TTGAGGATTT   2635
m141102_iid=754225_0       ATAA-TTTTATTTATTACATCAGAAGTATTTTTCTTCATTTCATTTTT--TTGAGGATTT   3800
m141107_iid=1409934_0      ATAA-TTTTATTTATTACATCAGAAGTATTTTTCTTCATTTCATTTTT--TTGAGGATTT   4342
m150503_iid=3416920_0      ATAA-TTTTATTTATTACATCAGAAGTATTTTTCTTCATTTCATTTTT--TTGAGGATTT   3067
m141108_iid=1800121_0      ATAA-TTTTATTTATTACATCAGAAGTATTTTTCTTCATTTCATTTTT--TTGAGGATTT   2878
m141031_iid=147540_0       ATAA-TTTTATTTATTACATCAGAAGTATTTTTCTTCATTTCATTTTTTTT--GAGGATTT   3219
m150507_iid=3674924_0      ATAA-TTTTATTTATTACATCAGAAGTATTTTTCTTCATTTCATTTTTTTTT-GAGGATTT   3370
m150503_iid=2923751_0      ATAA-TTTTATTTATTACATCAGAAGTATTTTTCTTCATTTCATTTTT--TTGAGGATTT   3213
```

```
m141031_iid=115118_0        ATAA-TTTTATTTATTACATCAGAAGTATTTTTCTTCATTTCATTTTTT--TTGAGGATTT   2064
m150430_iid=2722448_0       ATAA-TTTTATTTATTACATCAGAAGTATTTTTCTTCATTTCATTTTTT---TGAGGATTT   2007
m150507_iid=3102647_0       ATAA-TTTTATTTATTACAT-AGAAGTATTTTTCTTCATTTCATTTTTTTTT-GAGGATTT   309
m141108_iid=1811866_0       ATAA-TTTTATTTATTACATCAGAAGTATTTTTCTTCATTTCATTTTTT--TTGAGGATTT   2183
m150501_iid=2948495_0       ATAA-TTTTATTTATTACATCAGAAGTATTTTTCTTCATTTCATTTTTTT--TGAGGATTT   1607
m150504_iid=3238195_0       ATAA-TTTTATTTATTACATCAGAAGTATTTTTCTTCATTTCATTTTTTT-TGA-GGATTT   3938
m150503_iid=3388511_0       ATAA-TTTTATTTATTACATCAGAAGTATTTTTCTTCATTTCATTTTTTT-TGAGG-ATTT   1635
m150505_iid=3777611_0       ATAA-TTTTATTTATTACATCAGAAGTATTTTTCTTCATTTCATTTTTTT-TGAGG-ATTT   3053
m150430_iid=2438833_0       ATAA-TTTTATTTATTACATCAGAAGTATTTTTCTTCATTTCATTTTTTT-TGAGG-ATTT   3394
m141108_iid=1698969_0       ATAA-TTTTATTTATTACATCAGAAGTATTTTTCTTCATTTCATTTTTTT-TGAGG-ATTT   4520
m150430_iid=3059190_0       ATAA-TTTTATTTATTACATCAGAAGTATTTTTCTTCATTTCATTTTTTT-TGAGG-ATTT   242
m141107_iid=1383067_0       ATAA-TTTTATTTATTACATCAGAAGTATTTTTCTTCATTTCATTTTTTTTTGAGGATTTT   254
m141101_iid=597008_0        ------------------------------------------------------------   0
m150506_iid=2596496_0       ATAA-TTTTATTTATTACATCAGAAGTATTTTTCTTCATTTCATTTTTTT-TGAGG-ATTT   4880
m141102_iid=910051_0        ATAA-TTTTATTTATTACATCAGAAGTATTTTTCTTCATTTCATTTTTTT-TGAGG-ATTT   4369
m150505_iid=3784017_0       ATAA-TTTTATTTATTACATCAGAAGTATTTTTCTTCATTTCATTTTTTT-TGAGG-ATTT   3168
m141101_iid=682151_0        ATAA-TTTTATTTATTACATCAGAAGTATTTTTCTTCATTTCATTTTTTT-TGAGG-ATTT   2239
m150501_iid=3748029_0       ATAA-TTTTATTTATTACATCAGAAGTATTTTTCTTCATTTCATTTTTTT-TGAGG-ATTT   2780
m150504_iid=2876272_0       ATAA-TTTTATTTATTACATCAGAAGTATTTTTCTTCATTTCATTTTTTT-TGAGG-ATTT   4644
NC_042820.1                 ATAA-TTTTATTTATTACATCAGAAGTATTTTTCTTCATTTCATTTTTTT-TGAGG-ATTT   4887
m141102_iid=1228825_0       ATAA-TTTTATTTATTACATCAGAAGTATTTTTCTTCATTTCATTTTTTT-TGAGG-ATTT   4714
m150504_iid=2876274_0       ATAA-TTTTATTTATTACATCAGAAGTATTTTTCTTCATTTCATTTTTTT-TGAGG-ATTT   4717
m141030_iid=80036_0         ATAA-TTTTATTTATTACATCAGAAGTATTTTTCTTCATTTCATTTTTTT-TGAGG-ATTT   4479
m150506_iid=2596494_0       ATAA-TTTTATTTATTACATCAGAAGTATTTTTCTTCATTTCATTTTTTT-TGAGG-ATTT   4882
m150503_iid=2526862_0       ATAA-TTTTATTTATTACATCAGAAGTATTTTTCTTCATTTCATTTTTTT-TGAGG-ATTT   4566
m150506_iid=2567000_0       ATAA-TTTTATTTATTACATCAGAAGTATTTTTCTTCATTTCATTTTTTT-TGAGG-ATTT   2515
m150507_iid=3073271_0       ATAA-TTTTATTTATTACATCAGAAGTATTTTTCTTCATTTCATTTTTTT-TGAGG-ATTT   3819
m150504_iid=3156087_0       ATAA-TTTTATTTATTACATCAGAAGTATTTTTCTTCATTTCATTTTTTT-TGAGG-ATTT   3642
m150505_iid=2498502_0       ATAA-TTTTATTTATTACATCAGAAGTATTTTTCTTCATTTCATTTTTTT-TGAGG-ATTT   1292
m141102_iid=754227_0        ATAA-TTTTATTTATTACATCAGAAGTATTTTTCTTCATTTCATTTTTTT-TGAGG-ATTT   3854
m150507_iid=3469514_0       ATAA-TTTTATTTATTACATCAGAAGTATTTTTCTTCATTTCATTTTTTT-TGAGG-ATTT   4412
Rdo_MitoGenome              ATAA-TTTTATTTATTACATCAGAAGTATTTTTCTTCATTTCATTTTTTT-TGAGG-ATTT   4887
m141101_iid=614933_0        ATAA-TTTTATTTATTACATCAGAAGTATTTTTCTTCATTTCATTTTTTT-TGAGG-ATTT   3439
```

cox3

```
m141031_iid=347546_0        ------------------------------------------------------------   0
m150506_iid=3127414_0       TTCCACAGAAGATTATCTCCATCAATTGAAATTGGGATAAACTGACCACCTTTGGGAATT   1419
m141102_iid=1245517_0       TTCCACAGAAGATTATCTCCATCAATTGAAATTGGGATAAACTGACCACCTTTGGGAATT   3073
m141107_iid=1405727_0       TTCCACAGAAGATTATCTCCATCAATTGAAATTGGGATAAACTGACCACCTTTGGGAATT   258
m141102_iid=987614_0        TTCCACAGAAGATTATCTCCATCAATTGAAATTGGGATAAACTGACCACCTTTGGGAATT   3222
m141108_iid=1701429_0       ------------------------------------------------------------   0
m141102_iid=987286_0        TTCCACAGAAGATTATCTCCATCAATTGAAATTGGGATAAACTGACCACCTTTGGGAATT   1172
m150430_iid=3272125_0       TTCCACAGAAGATTATCTCCATCAATTGAAATTGGGATAAACTGACCACCTTTGGGAATT   2695
m141102_iid=754225_0        TTCCACAGAAGATTATCTCCATCAATTGAAATTGGGATAAACTGACCACCTTTGGGAATT   3860
m141107_iid=1409934_0       TTCCACAGAAGATTATCTCCATCAATTGAAATTGGGATAAACTGACCACCTTTGGGAATT   4402
m150503_iid=3416920_0       TTCCACAGAAGATTATCTCCATCAATTGAAATTGGGATAAACTGACCACCTTTGGGAATT   3127
m141108_iid=1800121_0       TTCCACAGAAGATTATCTCCATCAATTGAAATTGGGATAAACTGACCACCTTTGGGAATT   2938
m141031_iid=147540_0        TTCCACAGAAGATTATCTCCATCAATTGAAATTGGGATAAACTGACCACCTTTGGGAATT   3279
m150507_iid=3674924_0       TTCCACAGAAGATTATCTCCATCAATTGAAATTGGGATAAACTGACCACCTTTGGGAATT   3430
m150503_iid=2923751_0       TTCCACAGAAGATTATCTCCATCAATTGAAATTGGGATAAACTGACCACCTTTGGGAATT   3273
m141031_iid=115118_0        TTCCACAGAAGATTATCTCCATCAATTGAAATTGGGATAAACTGACCACCTTTGGGAATT   2124
m150430_iid=2722448_0       TTCCACAGAAGATTATCTCCATCAATTGAAATTGGGATAAACTGACCACCTTTGGGAATT   2067
m150507_iid=3102647_0       TTCCACAGAAGATTATCTCCATCAATTGAAATTGGGATAAACTGACCACCTTTGGGAATT   369
m141108_iid=1811866_0       TTCCACAGAAGATTATCTCCATCAATTGAAATTGGGATAAACTGACCACCTTTGGGAATT   2243
m150501_iid=2948495_0       TTCCACAGAAGATTATCTCCATCAATTGAAATTGGGATAAACTGACCACCTTTGGGAATT   1667
m150504_iid=3238195_0       TTCCACAGAAGATTATCTCCATCAATTGAAATTGGGATAAACTGACCACCTTTGGGAATT   3998
m150503_iid=3388511_0       TTCCACAGAAGATTATCTCCATCAATTGAAATTGGGATAAACTGACCACCTTTGGGAATT   1695
m150505_iid=3777611_0       TTCCACAGAAGATTATCTCCATCAATTGAAATTGGGATAAACTGACCACCTTTGGGAATT   3113
m150430_iid=2438833_0       TTCCACAGAAGATTATCTCCATCAATTGAAATTGGGATAAACTGACCACCTTTGGGAATT   3454
m141108_iid=1698969_0       TTCCACAGAAGATTATCTCCATCAATTGAAATTGGGATAAACTGACCACCTTTGGGAATT   4580
m150430_iid=3059190_0       TTCCACAGAAGATTATCTCCATCAATTGAAATTGGGATAAACTGACCACCTTTGGGAATT   302
m141107_iid=1383067_0       TTCCACAGAAGATTATCTCCATCAATTGAAATTGGGATAAACTGACCACCTTTGGGAATT   314
m141101_iid=597008_0        ------------------------------------------------------------   0
m150506_iid=2596496_0       TTCCACAGAAGATTATCTCCATCAATTGAAATTGGGATAAACTGACCACCTTTGGGAATT   4940
m141102_iid=910051_0        TTCCACAGAAGATTATCTCCATCAATTGAAATTGGGATAAACTGACCACCTTTGGGAATT   4429
m150505_iid=3784017_0       TTCCACAGAAGATTATCTCCATCAATTGAAATTGGGATAAACTGACCACCTTTGGGAATT   3228
m141101_iid=682151_0        TTCCACAGAAGATTATCTCCATCAATTGAAATTGGGATAAACTGACCACCTTTGGGAATT   2299
m150501_iid=3748029_0       TTCCACAGAAGATTATCTCCATCAATTGAAATTGGGATAAACTGACCACCTTTGGGAATT   2840
m150504_iid=2876272_0       TTCCACAGAAGATTATCTCCATCAATTGAAATTGGGATAAACTGACCACCTTTGGGAATT   4704
NC_042820.1                 TTCCACAGAAGATTATCTCCATCAATTGAAATTGGGATAAACTGACCACCTTTGGGAATT   4947
m141102_iid=1228825_0       TTCCACAGAAGATTATCTCCATCAATTGAAATTGGGATAAACTGACCACCTTTGGGAATT   4774
```

```
m150504_iid=2876274_0      TTCCACAGAAGATTATCTCCATCAATTGAAATTGGGATAAACTGACCACCTTTGGGAATT   4777
m141030_iid=80036_0        TTCCACAGAAGATTATCTCCATCAATTGAAATTGGGATAAACTGACCACCTTTGGGAATT   4539
m150506_iid=2596494_0      TTCCACAGAAGATTATCTCCATCAATTGAAATTGGGATAAACTGACCACCTTTGGGAATT   4942
m150503_iid=2526862_0      TTCCACAGAAGATTATCTCCATCAATTGAAATTGGGATAAACTGACCACCTTTGGGAATT   4626
m150506_iid=2567000_0      TTCCACAGAAGATTATCTCCATCAATTGAAATTGGGATAAACTGACCACCTTTGGGAATT   2575
m150507_iid=3073271_0      TTCCACAGAAGATTATCTCCATCAATTGAAATTGGGATAAACTGACCACCTTTGGGAATT   3879
m150504_iid=3156087_0      TTCCACAGAAGATTATCTCCATCAATTGAAATTGGGATAAACTGACCACCTTTGGGAATT   3702
m150505_iid=2498502_0      TTCCACAGAAGATTATCTCCATCAATTGAAATTGGGATAAACTGACCACCTTTGGGAATT   1352
m141102_iid=754227_0       TTCCACAGAAGATTATCTCCATCAATTGAAATTGGGATAAACTGACCACCTTTGGGAATT   3914
m150507_iid=3469514_0      TTCCACAGAAGATTATCTCCATCAATTGAAATTGGGATAAACTGACCACCTTTGGGAATT   4472
Rdo_MitoGenome             TTCCACAGAAGATTATCTCCATCAATTGAAATTGGGATAAACTGACCACCTTTGGGAATT   4947
m141101_iid=614933_0       TTCCACAGAAGATTATCTCCATCAATTGAAATTGGGATAAACTGACCACCTTTGGGAATT   3499
```

cox3

```
m141031_iid=347546_0       ------------------------------------------------------------   0
m150506_iid=3127414_0      TT-ACCTTCAATCCTTTAAGAATTCCTCTTCTTAATACACTAATTCTTCTTTACTAGAGG   1478
m141102_iid=1245517_0      TTAACCTTCAATCCTTTAAGAATTCCTCTTCTTAATACACTAATTCTTC-TTACTAGAGG   3132
m141107_iid=1405727_0      TTAACCTTCAATCCTTTAAGAATTCCTCTTCTTAATACACTAATTCTTC-TTACTAGA-G   316
m141102_iid=987614_0       TTAACCTTCAATCCTTTAAGAATTCCTCTTCTTAATACACTAATTCTTC-TTACTAGAGG   3281
m141108_iid=1701429_0      --------------------------------------------TCTTC-TTACTAGAGG   15
m141102_iid=987286_0       TTAACCTTCAATCCTTTAAGAATTCCTCTTCTTAATACACTAATTCTTC-TTACTAGAGG   1231
m150430_iid=3272125_0      TTAACCTTCAATCCTTTAAGAATTCCTCTTCTTAATACACTAATTCTTC-TTACTAGAGG   2754
m141102_iid=754225_0       TTAACCTTCAATCCTTTAAGAATTCCTCTTCTTAATACACTAATTCTTC-TTACTAGAGG   3919
m141107_iid=1409934_0      TTAACCTTCAATCCTTTAAGAATTCCTCTTCTTAATACACTAATTCTTC-TTACTAGAGG   4461
m150503_iid=3416920_0      TTAACCTTCAATCCTTTAAGAATTCCTCTTCTTAATACACTAATTCTTC-TTACTAGAGG   3186
m141108_iid=1800121_0      TTAACCTTCAATCCTTTAAGAATTCCTCTTCTTAATACACTAATTCTTC-TTACTAGAGG   2997
m141031_iid=147540_0       TTAACCTTCAATCCTTTAAGAATTCCTCTTCTTAATACACTAATTCTTC-TTACTAGAGG   3338
m150507_iid=3674924_0      TTAACCTTCAATCCTTTAAGAATTCCTCTTCTTAATACACTAATTCTTC-TTACTAGAGG   3489
m150503_iid=2923751_0      TTAACCTTCAATCCTTTAAGAATTCCTCTTCTTAATACACTAATTCTTC-TTACTAGAGG   3332
m141031_iid=115118_0       TTAACCTTCAATCCTTTAAGAATTCCTCTTCTTAATACACTAATTCTTC-TTACTAGAGG   2183
m150430_iid=2722448_0      TTAACCTTCAATCCTTTAAGAATTCCTCTTCTTAATACACTAATTCTTC-TTACTAGAGG   2126
m150507_iid=3102647_0      TTAACCTTCAATCCTTTAAGAATTCCTCTTCTTAATACACTAATTCTTC-TTACTAGAGG   428
m141108_iid=1811866_0      TTAACCTTCAATCCTTTAAGAATTCCTCTTCTTAATACACTAATTCTTC-TTACTAGAGG   2302
m150501_iid=2948495_0      TTAACCTTCAATCCTTTAAGAATTCCTCTTCTTAATACACTAATTCTTC-TTACTAGAGG   1726
m150504_iid=3238195_0      TTAACCTTCAATCCTTTAAGAATTCCTCTTCTTAATACACTAATTCTTC-TTACTAGAGG   4057
m150503_iid=3388511_0      TTAACCTTCAATCCTTTAAGAATTCCTCTTCTTAATACACTAATTCTTC-TTACTAGAGG   1754
m150505_iid=3777611_0      TTAACCTTCAATCCTTTAAGAATTCCTCTTCTTAATACACTAATTCTTC-TTACTAGAGG   3172
m150430_iid=2438833_0      TTAACCTTCAATCCTTTAAGAATTCCTCTTCTTAATACACTAATTCTTC-TTACTAGAGG   3513
m141108_iid=1698969_0      TTAACCTTCAATCCTTTAAGAATTCCTCTTCTTAATACACTAATTCTTC-TTACTAGAGG   4639
m150430_iid=3059190_0      TTAACCTTCAATCCTTTAAGAATTCCTCTTCTTAATACACTAATTCTTC-TTACTAGAGG   361
m141107_iid=1383067_0      TTAACCTTCAATCCTTTAAGAATTCCTCTTCTTAATACACTAATTCTTC-TTACTAGAGG   373
m141101_iid=597008_0       ------------------------------------------------------------   0
m150506_iid=2596496_0      TTAACCTTCAATCCTTTAAGAATTCCTCTTCTTAATACACTAATTCTTC-TTACTAGAGG   4999
m141102_iid=910051_0       TTAACCTTCAATCCTTTAAGAATTCCTCTTCTTAATACACTAATTCTTC-TTACTAGAGG   4488
m150505_iid=3784017_0      TTAACCTTCAATCCTTTAAGAATTCCTCTTCTTAATACACTAATTCTTC-TTACTAGAGG   3287
m141101_iid=682151_0       TTAACCTTCAATCCTTTAAGAATTCCTCTTCTTAATACACTAATTCTTC-TTACTAGAGG   2358
m150501_iid=3748029_0      TTAACCTTCAATCCTTTAAGAATTCCTCTTCTTAATACACTAATTCTTC-TTACTAGAGG   2899
m150504_iid=2876272_0      TTAACCTTCAATCCTTTAAGAATTCCTCTTCTTAATACACTAATTCTTC-TTACTAGAGG   4763
NC_042820.1                TTAACCTTCAATCCTTTAAGAATTCCTCTTCTTAATACACTAATTCTTC-TTACTAGAGG   5006
m141102_iid=1228825_0      TTAACCTTCAATCCTTTAAGAATTCCTCTTCTTAATACACTAATTCTTC-TTACTAGAGG   4833
m150504_iid=2876274_0      TTAACCTTCAATCCTTTAAGAATTCCTCTTCTTAATACACTAATTCTTC-TTACTAGAGG   4836
m141030_iid=80036_0        TTAACCTTCAATCCTTTAAGAATTCCTCTTCTTAATACACTAATTCTTC-TTACTAGAGG   4598
m150506_iid=2596494_0      TTAACCTTCAATCCTTTAAGAATTCCTCTTCTTAATACACTAATTCTTC-TTACTAGAGG   5001
m150503_iid=2526862_0      TTAACCTTCAATCCTTTAAGAATTCCTCTTCTTAATACACTAATTCTTC-TTACTAGAGG   4685
m150506_iid=2567000_0      TTAACCTTCAATCCTTTAAGAATTCCTCTTCTTAATACACTAATTCTTC-TTACTAGAGG   2634
m150507_iid=3073271_0      TTAACCTTCAATCCTTTAAGAATTCCTCTTCTTAATACACTAATTCTTC-TTACTAGAGG   3938
m150504_iid=3156087_0      TTAACCTTCAATCCTTTAAGAATTCCTCTTCTTAATACACTAATTCTTC-TTACTAGAGG   3761
m150505_iid=2498502_0      TTAACCTTCAATCCTTTAAGAATTCCTCTTCTTAATACACTAATTCTTC-TTACTAGAGG   1411
m141102_iid=754227_0       TTAACCTTCAATCCTTTAAGAATTCCTCTTCTTAATACACTAATTCTTC-TTACTAGAGG   3973
m150507_iid=3469514_0      TTAACCTTCAATCCTTTAAGAATTCCTCTTCTTAATACACTAATTCTTC-TTACTAGAGG   4531
Rdo_MitoGenome             TTAACCTTCAATCCTTTAAGAATTCCTCTTCTTAATACACTAATTCTTC-TTACTAGAGG   5006
m141101_iid=614933_0       TTAACCTTCAATCCTTTAAGAATTCCTCTTCTTAATACACTAATTCTTC-TTACTAGAGG   3558
```

cox3

```
m141031_iid=347546_0       ------------------------------------------------------------   0
m150506_iid=3127414_0      ACTTACAGTAACATGAGCACATCATAGATTAATAAGAAAATAACTGAAAACAAGC-AATC   1537
m141102_iid=1245517_0      ACTTACAGTAACATGAGCACATCATAGATTAAT-AGAAAATAACTGAAAACAAGCAAATC   3191
m141107_iid=1405727_0      ACTTACAGTAACATGAGCACATCATAGATTAAT-AGAAAATAACTGAAAACAAGCAAATC   375
m141102_iid=987614_0       ACTTACAGTAACATGAGCACATCATAGATTAAT-AGAAAATAACTGAAAACAAGCAAATC   3340
m141108_iid=1701429_0      ACTTACAGTAACATGAGCACATCATAGATTAAT-AGAAAATAACTGAAAACAAGCAAATC   74
m141102_iid=987286_0       ACTTACAGTAACATGAGCACATCATAGATTAAT-AGAAAATAACTGAAAACAAGCAAATC   1290
```

m150430_iid=3272125_0	ACTTACAGTAACATGAGCACATCATAGATTAAT-AGAAAATAACTGAAAACAAGCAAATC	2813
m141102_iid=754225_0	ACTTACAGTAACATGAGCACATCATAGATTAAT-AGAAAATAACTGAAAACAAGCAAATC	3978
m141107_iid=1409934_0	ACTTACAGTAACATGAGCACATCATAGATTAAT-AGAAAATAACTGAAAACAAGCAAATC	4520
m150503_iid=3416920_0	ACTTACAGTAACATGAGCACATCATAGATTAAT-AGAAAATAACTGAAAACAAGCAAATC	3245
m141108_iid=1800121_0	ACTTACAGTAACATGAGCACATCATAGATTAAT-AGAAAATAACTGAAAACAAGCAAATC	3056
m141031_iid=147540_0	ACTTACAGTAACATGAGCACATCATAGATTAAT-AGAAAATAACTGAAAACAAGCAAATC	3397
m150507_iid=3674924_0	ACTTACAGTAACATGAGCACATCATAGATTAAT-AGAAAATAACTGAAAACAAGCAAATC	3548
m150503_iid=2923751_0	ACTTACAGTAACATGAGCACATCATAGATTAAT-AGAAAATAACTGAAAACAAGCAAATC	3391
m141031_iid=115118_0	ACTTACAGTAACATGAGCACATCATAGATTAAT-AGAAAATAACTGAAAACAAGCAAATC	2242
m150430_iid=2722448_0	ACTTACAGTAACATGAGCACATCATAGATTAAT-AGAAAATAACTGAAAACAAGCAAATC	2185
m150507_iid=3102647_0	ACTTACAGTAACATGAGCACATCATAGATTAAT-AGAAAATAACTGAAAACAAGCAAATC	487
m141108_iid=1811866_0	ACTTACAGTAACATGAGCACATCATAGATTAAT-AGAAAATAACTGAAAACAAGCAAATC	2361
m150501_iid=2948495_0	ACTTACAGTAACATGAGCACATCATAGATTAAT-AGAAAATAACTGAAAACAAGCAAATC	1785
m150504_iid=3238195_0	ACTTACAGTAACATGAGCACATCATAGATTAAT-AGAAAATAACTGAAAACAAGCAAATC	4116
m150503_iid=3388511_0	ACTTACAGTAACATGAGCACATCATAGATTAAT-AGAAAATAACTGAAAACAAGCAAATC	1813
m150505_iid=3777611_0	ACTTACAGTAACATGAGCACATCATAGATTAAT-AGAAAATAACTGAAAACAAGCAAATC	3231
m150430_iid=2438833_0	ACTTACAGTAACATGAGCACATCATAGATTAAT-AGAAAATAACTGAAAACAAGCAAATC	3572
m141108_iid=1698969_0	ACTTACAGTAACATGAGCACATCATAGATTAAT-AGAAAATAACTGAAAACAAGCAAATC	4698
m150430_iid=3059190_0	ACTTACAGTAACATGAGCACATCATAGATTAAT-AGAAAATAACTGAAAACAAGCAAATC	420
m141107_iid=1383067_0	ACTTACAGTAACATGAGCACATCATAGATTAAT-AGAAAATAACTGAAAACAAGCAAATC	432
m141101_iid=597008_0	--	0
m150506_iid=2596496_0	ACTTACAGTAACATGAGCACATCATAGATTAAT-AGAAAATAACTGAAAACAAGCAAATC	5058
m141102_iid=910051_0	ACTTACAGTAACATGAGCACATCATAGATTAAT-AGAAAATAACTGAAAACAAGCAAATC	4547
m150505_iid=3784017_0	ACTTACAGTAACATGAGCACATCATAGATTAAT-AGAAAATAACTGAAAACAAGCAAATC	3346
m141101_iid=682151_0	ACTTACAGTAACATGAGCACATCATAGATTAAT-AGAAAATAACTGAAAACAAGCAAATC	2417
m150501_iid=3748029_0	ACTTACAGTAACATGAGCACATCATAGATTAAT-AGAAAATAACTGAAAACAAGCAAATC	2958
m150504_iid=2876272_0	ACTTACAGTAACATGAGCACATCATAGATTAAT-AGAAAATAACTGAAAACAAGCAAATC	4822
NC_042820.1	ACTTACAGTAACATGAGCACATCATAGATTAAT-AGAAAATAACTGAAAACAAGCAAATC	5065
m141102_iid=1228825_0	ACTTACAGTAACATGAGCACATCATAGATTAAT-AGAAAATAACTGAAAACAAGCAAATC	4892
m150504_iid=2876274_0	ACTTACAGTAACATGAGCACATCATAGATTAAT-AGAAAATAACTGAAAACAAGCAAATC	4895
m141030_iid=80036_0	ACTTACAGTAACATGAGCACATCATAGATTAAT-AGAAAATAACTGAAAACAAGCAAATC	4657
m150506_iid=2596494_0	ACTTACAGTAACATGAGCACATCATAGATTAAT-AGAAAATAACTGAAAACAAGCAAATC	5060
m150503_iid=2526862_0	ACTTACAGTAACATGAGCACATCATAGATTAAT-AGAAAATAACTGAAAACAAGCAAATC	4744
m150506_iid=2567000_0	ACTTACAGTAACATGAGCACATCATAGATTAAT-AGAAAATAACTGAAAACAAGCAAATC	2693
m150507_iid=3073271_0	ACTTACAGTAACATGAGCACATCATAGATTAAT-AGAAAATAACTGAAAACAAGCAAATC	3997
m150504_iid=3156087_0	ACTTACAGTAACATGAGCACATCATAGATTAAT-AGAAAATAACTGAAAACAAGCAAATC	3820
m150505_iid=2498502_0	ACTTACAGTAACATGAGCACATCATAGATTAAT-AGAAAATAACTGAAAACAAGCAAATC	1470
m141102_iid=754227_0	ACTTACAGTAACATGAGCACATCATAGATTAAT-AGAAAATAACTGAAAACAAGCAAATC	4032
m150507_iid=3469514_0	ACTTACAGTAACATGAGCACATCATAGATTAAT-AGAAAATAACTGAAAACAAGCAAATC	4590
Rdo_MitoGenome	ACTTACAGTAACATGAGCACATCATAGATTAAT-AGAAAATAACTGAAAACAAGCAAATC	5065
m141101_iid=614933_0	ACTTACAGTAACATGAGCACATCATAGATTAAT-AGAAAATAACTGAAAACAAGCAAATC	3617

cox3

m141031_iid=347546_0	--	0
m150506_iid=3127414_0	AAGGACTAACACTAACAATCATTCTTGGTTTATATTTTACTATTCTTCAAGCTTACGAAT	1597
m141102_iid=1245517_0	AAGGACTAACACTAACAATCATTCTTGGTTTATATTTTACTATTCTTCAAGCTTACGAAT	3251
m141107_iid=1405727_0	AAGGACTAACACTAACAATCATTCTTGGTTTATATTTTACTATTCTTCAAGCTTACGAAT	435
m141102_iid=987614_0	AAGGACTAACACTAACAATCATTCTTGGTTTATATTTTACTATTCTTCAAGCTTACGAAT	3400
m141108_iid=1701429_0	AAGGACTAACACTAACAATCATTCTTGGTTTATATTTTACTATTCTTCAAGCTTACGAAT	134
m141102_iid=987286_0	AAGGACTAACACTAACAATCATTCTTGGTTTATATTTTACTATTCTTCAAGCTTACGAAT	1350
m150430_iid=3272125_0	AAGGACTAACACTAACAATCATTCTTGGTTTATATTTTACTATTCTTCAAGCTTACGAAT	2873
m141102_iid=754225_0	AAGGACTAACACTAACAATCATTCTTGGTTTATATTTTACTATTCTTCAAGCTTACGAAT	4038
m141107_iid=1409934_0	AAGGACTAACACTAACAATCATTCTTGGTTTATATTTTACTATTCTTCAAGCTTACGAAT	4580
m150503_iid=3416920_0	AAGGACTAACACTAACAATCATTCTTGGTTTATATTTTACTATTCTTCAAGCTTACGAAT	3305
m141108_iid=1800121_0	AAGGACTAACACTAACAATCATTCTTGGTTTATATTTTACTATTCTTCAAGCTTACGAAT	3116
m141031_iid=147540_0	AAGGACTAACACTAACAATCATTCTTGGTTTATATTTTACTATTCTTCAAGCTTACGAAT	3457
m150507_iid=3674924_0	AAGGACTAACACTAACAATCATTCTTGGTTTATATTTTACTATTCTTCAAGCTTACGAAT	3608
m150503_iid=2923751_0	AAGGACTAACACTAACAATCATTCTTGGTTTATATTTTACTATTCTTCAAGCTTACGAAT	3451
m141031_iid=115118_0	AAGGACTAACACTAACAATCATTCTTGGTTTATATTTTACTATTCTTCAAGCTTACGAAT	2302
m150430_iid=2722448_0	AAGGACTAACACTAACAATCATTCTTGGTTTATATTTTACTATTCTTCAAGCTTACGAAT	2245
m150507_iid=3102647_0	AAGGACTAACACTAACAATCATTCTTGGTTTATATTTTACTATTCTTCAAGCTTACGAAT	547
m141108_iid=1811866_0	AAGGACTAACACTAACAATCATTCTTGGTTTATATTTTACTATTCTTCAAGCTTACGAAT	2421
m150501_iid=2948495_0	AAGGACTAACACTAACAATCATTCTTGGTTTATATTTTACTATTCTTCAAGCTTACGAAT	1845
m150504_iid=3238195_0	AAGGACTAACACTAACAATCATTCTTGGTTTATATTTTACTATTCTTCAAGCTTACGAAT	4176
m150503_iid=3388511_0	AAGGACTAACACTAACAATCATTCTTGGTTTATATTTTACTATTCTTCAAGCTTACGAAT	1873
m150505_iid=3777611_0	AAGGACTAACACTAACAATCATTCTTGGTTTATATTTTACTATTCTTCAAGCTTACGAAT	3291
m150430_iid=2438833_0	AAGGACTAACACTAACAATCATTCTTGGTTTATATTTTACTATTCTTCAAGCTTACGAAT	3632
m141108_iid=1698969_0	AAGGACTAACACTAACAATCATTCTTGGTTTATATTTTACTATTCTTCAAGCTTACGAAT	4758
m150430_iid=3059190_0	AAGGACTAACACTAACAATCATTCTTGGTTTATATTTTACTATTCTTCAAGCTTACGAAT	480
m141107_iid=1383067_0	AAGGACTAACACTAACAATCATTCTTGGTTTATATTTTACTATTCTTCAAGCTTACGAAT	492
m141101_iid=597008_0	--	0

```
m150506_iid=2596496_0      AAGGACTAACACTAACAATCATTCTTGGTTTATATTTTACTATTCTTCAAGCTTACGAAT   5118
m141102_iid=910051_0       AAGGACTAACACTAACAATCATTCTTGGTTTATATTTTACTATTCTTCAAGCTTACGAAT   4607
m150505_iid=3784017_0      AAGGACTAACACTAACAATCATTCTTGGTTTATATTTTACTATTCTTCAAGCTTACGAAT   3406
m141101_iid=682151_0       AAGGACTAACACTAACAATCATTCTTGGTTTATATTTTACTATTCTTCAAGCTTACGAAT   2477
m150501_iid=3748029_0      AAGGACTAACACTAACAATCATTCTTGGTTTATATTTTACTATTCTTCAAGCTTACGAAT   3018
m150504_iid=2876272_0      AAGGACTAACACTAACAATCATTCTTGGTTTATATTTTACTATTCTTCAAGCTTACGAAT   4882
NC_042820.1                AAGGACTAACACTAACAATCATTCTTGGTTTATATTTTACTATTCTTCAAGCTTACGAAT   5125
m141102_iid=1228825_0      AAGGACTAACACTAACAATCATTCTTGGTTTATATTTTACTATTCTTCAAGCTTACGAAT   4952
m150504_iid=2876274_0      AAGGACTAACACTAACAATCATTCTTGGTTTATATTTTACTATTCTTCAAGCTTACGAAT   4955
m141030_iid=80036_0        AAGGACTAACACTAACAATCATTCTTGGTTTATATTTTACTATTCTTCAAGCTTACGAAT   4717
m150506_iid=2596494_0      AAGGACTAACACTAACAATCATTCTTGGTTTATATTTTACTATTCTTCAAGCTTACGAAT   5120
m150503_iid=2526862_0      AAGGACTAACACTAACAATCATTCTTGGTTTATATTTTACTATTCTTCAAGCTTACGAAT   4804
m150506_iid=2567000_0      AAGGACTAACACTAACAATCATTCTTGGTTTATATTTTACTATTCTTCAAGCTTACGAAT   2753
m150507_iid=3073271_0      AAGGACTAACACTAACAATCATTCTTGGTTTATATTTTACTATTCTTCAAGCTTACGAAT   4057
m150504_iid=3156087_0      AAGGACTAACACTAACAATCATTCTTGGTTTATATTTTACTATTCTTCAAGCTTACGAAT   3880
m150505_iid=2498502_0      AAGGACTAACACTAACAATCATTCTTGGTTTATATTTTACTATTCTTCAAGCTTACGAAT   1530
m141102_iid=754227_0       AAGGACTAACACTAACAATCATTCTTGGTTTATATTTTACTATTCTTCAAGCTTACGAAT   4092
m150507_iid=3469514_0      AAGGACTAACACTAACAATCATTCTTGGTTTATATTTTACTATTCTTCAAGCTTACGAAT   4650
Rdo_MitoGenome             AAGGACTAACACTAACAATCATTCTTGGTTTATATTTTACTATTCTTCAAGCTTACGAAT   5125
m141101_iid=614933_0       AAGGACTAACACTAACAATCATTCTTGGTTTATATTTTACTATTCTTCAAGCTTACGAAT   3677
```

cox3

```
m141031_iid=347546_0       ------------------------------------------------------------   0
m150506_iid=3127414_0      ATATTGAAGCACCATTCACTATCT-CCGATTCCGTTTACGGATCAAGTTT-TTTATAGCA   1655
m141102_iid=1245517_0      ATATTGAAGCACCATTCACTATCT-CCGATTCCGTTTACGGATCAAGTTTCTTTATAGCA   3310
m141107_iid=1405727_0      ATATTGAAGCACCATTCACTATCT-CCGATTCCGTTTACGGATCAAGTTTCTTTATAGCA   494
m141102_iid=987614_0       ATATTGAAGCACCATTCACTATCT-CCGATTCCGTTTACGGATCAAGTTTCTTTATAGCA   3459
m141108_iid=1701429_0      ATATTGAAGCACCATTCACTATCT-CCGATTCCGTTTACGGATCAAGTTTCTTTATAGCA   193
m141102_iid=987286_0       ATATTGAAGCACCATTCACTATCT-CCGATTCCGTTTACGGATCAAGTTTCTTTATAGCA   1409
m150430_iid=3272125_0      ATATTGAAGCACCATTCACTATCT-CCGATTCCGTTTACGGATCAAGTTTCTTTATAGCA   2932
m141102_iid=754225_0       ATATTGAAGCACCATTCACTATCT-CCGATTCCGTTTACGGATCAAGTTTCTTTATAGCA   4097
m141107_iid=1409934_0      ATATTGAAGCACCATTCACTATCT-CCGATTCCGTTTACGGATCAAGTTTCTTTATAGCA   4639
m150503_iid=3416920_0      ATATTGAAGCACCATTCACTATCT-CCGATTCCGTTTACGGATCAAGTTTCTTTATAGCA   3364
m141108_iid=1800121_0      ATATTGAAGCACCATTCACTATCT-CCGATTCCGTTTACGGATCAAGTTTCTTTATAGCA   3175
m141031_iid=147540_0       ATATTGAAGCACCATTCACTATCT-CCGATTCCGTTTACGGATCAAGTTTCTTTATAGCA   3516
m150507_iid=3674924_0      ATATTGAAGCACCATTCACTATCT-CCGATTCCGTTTACGGATCAAGTTTCTTTATAGCA   3667
m150503_iid=2923751_0      ATATTGAAGCACCATTCACTATCT-CCGATTCCGTTTACGGATCAAGTTTCTTTATAGCA   3510
m141031_iid=115118_0       ATATTGAAGCACCATTCACTATCT-CCGATTCCGTTTACGGATCAAGTTTCTTTATAGCA   2361
m150430_iid=2722448_0      ATATTGAAGCACCATTCACTATCT-CCGATTCCGTTTACGGATCAAGTTTCTTTATAGCA   2304
m150507_iid=3102647_0      ATATTGAAGCACCATTCACTATCT-CCGATTCCGTTTACGGATCAAGTTTCTTTATAGCA   606
m141108_iid=1811866_0      ATATTGAAGCACCATTCACTATCT-CCGATTCCGTTTACGGATCAAGTTTCTTTATAGCA   2480
m150501_iid=2948495_0      ATATTGAAGCACCATTCACTATCT-CCGATTCCGTTTACGGATCAAGTTTCTTTATAGCA   1904
m150504_iid=3238195_0      ATATTGAAGCACCATTCACTATCT-CCGATTCCGTTTACGGATCAAGTTTCTTTATAGCA   4235
m150503_iid=3388511_0      ATATTGAAGCACCATTCACTATCT-CCGATTCCGTTTACGGATCAAGTTTCTTTATAGCA   1932
m150505_iid=3777611_0      ATATTGAAGCACCATTCACTATCT-CCGATTCCGTTTACGGATCAAGTTTCTTTATAGCA   3350
m150430_iid=2438833_0      ATATTGAAGCACCATTCACTATCT-CCGATTCCGTTTACGGATCAAGTTTCTTTATAGCA   3691
m141108_iid=1698969_0      ATATTGAAGCACCATTCACTATCT-CCGATTCCGTTTACGGATCAAGTTTCTTTATAGCA   4817
m150430_iid=3059190_0      ATATTGAAGCACCATTCACTATCT-CCGATTCCGTTTACGGATCAAGTTTCTTTATAGCA   539
m141107_iid=1383067_0      ATATTGAAGCACCATTCACTATCT-CCGATTCCGTTTACGGATCAAGTTTCTTTATAGCA   551
m141101_iid=597008_0       ------------------------------------------------------------   0
m150506_iid=2596496_0      ATATTGAAGCACCATTCACTATCTATCGATTCCGTTTACGGATCAAGTTTCTTTATAGCA   5178
m141102_iid=910051_0       ATATTGAAGCACCATTCACTATCT-CCGATTCCGTTTACGGATCAAGTTTCTTTATAGCA   4666
m150505_iid=3784017_0      ATATTGAAGCACCATTCACTATCT-CCGATTCCGTTTACGGATCAAGTTTCTTTATAGCA   3465
m141101_iid=682151_0       ATATTGAAGCACCATTCACTATCT-CCGATTCCGTTTACGGATCAAGTTTCTTTATAGCA   2536
m150501_iid=3748029_0      ATATTGAAGCACCATTCACTATCT-CCGATTCCGTTTACGGATCAAGTTTCTTTATAGCA   3077
m150504_iid=2876272_0      ATATTGAAGCACCATTCACTATCT-CCGATTCCGTTTACGGATCAAGTTTCTTTATAGCA   4941
NC_042820.1                ATATTGAAGCACCATTCACTATCT-CCGATTCCGTTTACGGATCAAGTTTCTTTATAGCA   5184
m141102_iid=1228825_0      ATATTGAAGCACCATTCACTATCT-CCGATTCCGTTTACGGATCAAGTTTCTTTATAGCA   5011
m150504_iid=2876274_0      ATATTGAAGCACCATTCACTATCT-CCGATTCCGTTTACGGATCAAGTTTCTTTATAGCA   5014
m141030_iid=80036_0        ATATTGAAGCACCATTCACTATCT-CCGATTCCGTTTACGGATCAAGTTTCTTTATAGCA   4776
m150506_iid=2596494_0      ATATTGAAGCACCATTCACTATCT-CCGATTCCGTTTACGGATCAAGTTTCTTTATAGCA   5179
m150503_iid=2526862_0      ATATTGAAGCACCATTCACTATCT-CCGATTCCGTTTACGGATCAAGTTTCTTTATAGCA   4863
m150506_iid=2567000_0      ATATTGAAGCACCATTCACTATCT-CCGATTCCGTTTACGGATCAAGTTTCTTTATAGCA   2812
m150507_iid=3073271_0      ATATTGAAGCACCATTCACTATCT-CCGATTCCGTTTACGGATCAAGTTTCTTTATAGCA   4116
m150504_iid=3156087_0      ATATTGAAGCACCATTCACTATCT-CCGATTCCGTTTACGGATCAAGTTTCTTTATAGCA   3939
m150505_iid=2498502_0      ATATTGAAGCACCATTCACTATCT-CCGATTCCGTTTACGGATCAAGTTTCTTTATAGCA   1589
m141102_iid=754227_0       ATATTGAAGCACCATTCACTATCT-CCGATTCCGTTTACGGATCAAGTTTCTTTATAGCA   4151
m150507_iid=3469514_0      ATATTGAAGCACCATTCACTATCT-CCGATTCCGTTTACGGATCAAGTTTCTTTATAGCA   4709
Rdo_MitoGenome             ATATTGAAGCACCATTCACTATCT-CCGATTCCGTTTACGGATCAAGTTTCTTTATAGCA   5184
m141101_iid=614933_0       ATATTGAAGCACCATTCACTATCT-CCGATTCCGTTTACGGATCAAGTTTCTTTATAGCA   3736
```

cox3

```
m141031_iid=347546_0        ------------------------------------------------------------   0
m150506_iid=3127414_0       ACAGGATTCCATGGACTACA-TGTGATTATTGGAACAACATTTCTTAGAGTTTGCCTTTT   1714
m141102_iid=1245517_0       ACAGGATTCCATGGACTACA-TGTGATTATTGGAACAACATTTCTTAGAGTTTGCCTTTT   3369
m141107_iid=1405727_0       ACAGGATTCCATGGACTACA-TGTGATTATTGGAACAACATTTCTTAGAGTTTGCCTTTT   553
m141102_iid=987614_0        ACAGGATTCCATGGACTACA-TGTGATTATTGGAACAACATTTCTTAGAGTTTGCCTTTT   3518
m141108_iid=1701429_0       ACAGGATTCCATGGACTACA-TGTGATTATTGGAACAACATTTCTTAGAGTTTGCCTTTT   252
m141102_iid=987286_0        ACAGGATTCCATGGACTACA-TGTGATTATTGGAACAACATTTCTTAGAGTTTGCCTTTT   1468
m150430_iid=3272125_0       ACAGGATTCCATGGACTACA-TGTGATTATTGGAACAACATTTCTTAGAGTTTGCCTTTT   2991
m141102_iid=754225_0        ACAGGATTCCATGGACTACA-TGTGATTATTGGAACAACATTTCTTAGAGTTTGCCTTTT   4156
m141107_iid=1409934_0       ACAGGATTCCATGGACTACATTGTGATTATTGGAACAACATTTCTTAGAGTTTGCCTTTT   4699
m150503_iid=3416920_0       ACAGGATTCCATGGACTACA-TGTGATTATTGGAACAACATTTCTTAGAGTTTGCCTTTT   3423
m141108_iid=1800121_0       ACAGGATTCCATGGACTACA-TGTGATTATTGGAACAACATTTCTTAGAGTTTGCCTTTT   3234
m141031_iid=147540_0        ACAGGATTCCATGGACTACA-TGTGATTATTGGAACAACATTTCTTAGAGTTTGCCTTTT   3575
m150507_iid=3674924_0       ACAGGATTCCATGGACTACA-TGTGATTATTGGAACAACATTTCTTAGAGTTTGCCTTTT   3726
m150503_iid=2923751_0       ACAGGATTCCATGGACTACA-TGTGATTATTGGAACAACATTTCTTAGAGTTTGCCTTTT   3569
m141031_iid=115118_0        ACAGGATTCCATGGACTACA-TGTGATTATTGGAACAACATTTCTTAGAGTTTGCCTTTT   2420
m150430_iid=2722448_0       ACAGGATTCCATGGACTACA-TGTGATTATTGGAACAACATTTCTTAGAGTTTGCT-TTT   2362
m150507_iid=3102647_0       ACAGGATTCCATGGACTACA-TGTGATTATTGGAACAACATTTCTTAGAGTTTGCCTTTT   665
m141108_iid=1811866_0       ACAGGATTCCATGGACTACA-TGTGATTATTGGAACAACATTTCTTAGAGTTTGCCTTTT   2539
m150501_iid=2948495_0       ACAGGATTCCATGGACTACA-TGTGATTATTGGAACAACATTTCTTAGAGTTTGCCTTTT   1963
m150504_iid=3238195_0       ACAGGATTCCATGGACTACA-TGTGATTATTGGAACAACATTTCTTAGAGTTTGCCTTTT   4294
m150503_iid=3388511_0       ACAGGATTCCATGGACTACA-TGTGATTATTGGAACAACATTTCTTAGAGTTTGCCTTTT   1991
m150505_iid=3777611_0       ACAGGATTCCATGGACTACA-TGTGATTATTGGAACAACATTTCTTAGAGTTTGCCTTTT   3409
m150430_iid=2438833_0       ACAGGATTCCATGGACTACA-TGTGATTATTGGAACAACATTTCTTAGAGTTTGCCTTTT   3750
m141108_iid=1698969_0       ACAGGATTCCATGGACTACA-TGTGATTATTGGAACAACATTTCTTAGAGTTTGCCTTTT   4876
m150430_iid=3059190_0       ACAGGATTCCATGGACTACA-TGTGATTATTGGAACAACATTTCTTAGAGTTTGCCTTTT   598
m141107_iid=1383067_0       ACAGGATTCCATGGACTACA-TGTGATTATTGGAACAACATTTCTTAGAGTTTGCCTTTT   610
m141101_iid=597008_0        ------------------------------------------------------------   0
m150506_iid=2596496_0       ACAGGATTCCATGGACTACA-TGTGATTATTGGAACAACATTTCTTAGAGTTTGCCTTTT   5237
m141102_iid=910051_0        ACAGGATTCCATGGACTACA-TGTGATTATTGGAACAACATTTCTTAGAGTTTGCCTTTT   4725
m150505_iid=3784017_0       ACAGGATTCCATGGACTACA-TGTGATTATTGGAACAACATTTCTTAGAGTTTGCCTTTT   3524
m141101_iid=682151_0        ACAGGATTCCATGGACTACA-TGTGATTATTGGAACAACATTTCTTAGAGTTTGCCTTTT   2595
m150501_iid=3748029_0       ACAGGATTCCATGGACTACA-TGTGATTATTGGAACAACATTTCTTAGAGTTTGCCTTTT   3136
m150504_iid=2876272_0       ACAGGATTCCATGGACTACA-TGTGATTATTGGAACAACATTTCTTAGAGTTTGCCTTTT   5000
NC_042820.1                 ACAGGATTCCATGGACTACA-TGTGATTATTGGAACAACATTTCTTAGAGTTTGCCTTTT   5243
m141102_iid=1228825_0       ACAGGATTCCATGGACTACA-TGTGATTATTGGAACAACATTTCTTAGAGTTTGCCTTTT   5070
m150504_iid=2876274_0       ACAGGATTCCATGGACTACA-TGTGATTATTGGAACAACATTTCTTAGAGTTTGCCTTTT   5073
m141030_iid=80036_0         ACAGGATTCCATGGACTACA-TGTGATTATTGGAACAACATTTCTTAGAGTTTGCCTTTT   4835
m150506_iid=2596494_0       ACAGGATTCCATGGACTACA-TGTGATTATTGGAACAACATTTCTTAGAGTTTGCCTTTT   5238
m150503_iid=2526862_0       ACAGGATTCCATGGACTACA-TGTGATTATTGGAACAACATTTCTTAGAGTTTGCCTTTT   4922
m150506_iid=2567000_0       ACAGGATTCCATGGACTACA-TGTGATTATTGGAACAACATTTCTTAGAGTTTGCCTTTT   2871
m150507_iid=3073271_0       ACAGGATTCCATGGACTACA-TGTGATTATTGGAACAACATTTCTTAGAGTTTGCCTTTT   4175
m150504_iid=3156087_0       ACAGGATTCCATGGACTACA-TGTGATTATTGGAACAACATTTCTTAGAGTTTGCCTTTT   3998
m150505_iid=2498502_0       ACAGGATTCCATGGACTACA-TGTGATTATTGGAACAACATTTCTTAGAGTTTGCCTTTT   1648
m141102_iid=754227_0        ACAGGATTCCATGGACTACA-TGTGATTATTGGAACAACATTTCTTAGAGTTTGCCTTTT   4210
m150507_iid=3469514_0       ACAGGATTCCATGGACTACA-TGTGATTATTGGAACAACATTTCTTAGAGTTTGCCTTTT   4768
Rdo_MitoGenome              ACAGGATTCCATGGACTACA-TGTGATTATTGGAACAACATTTCTTAGAGTTTGCCTTTT   5243
m141101_iid=614933_0        ACAGGATTCCATGGACTACA-TGTGATTATTGGAACAACATTTCTTAGAGTTTGCCTTTT   3795
```

cox3

```
m141031_iid=347546_0        ------------------------------------CACTTCGGATTTGAAGCAGCAGCC   24
m150506_iid=3127414_0       A--CGTCATCTAATAAATCATTTCTCAATAATTCACCACTTCGGATTTGAAGCAGCAGCC   1772
m141102_iid=1245517_0       --ACGTCATCTAATAAATCATTTCTCAATAATTCACCACTTCGGATTTGAAGCAGCAGCC   3427
m141107_iid=1405727_0       ---CGTCATCTAATAAATCATTTCTCAATAATTCACCACTTCGGATTTGAAGCAGCAGCC   610
m141102_iid=987614_0        A--CGTCATCTAATAAATCATTTCTCAATAATTCACCACTTCGGATTTGAAGCAGCAGCC   3576
m141108_iid=1701429_0       --ACGTCATCTAATAAATCATTTCTCAATAATTCACCACTTCGGATTTGAAGCAGCAGCC   310
m141102_iid=987286_0        --ACGTCATCTAATAAATCATTTCTCAATAATTCACCACTTCGGATTTGAAGCAGCAGCC   1526
m150430_iid=3272125_0       --ACGTCATCTAATAAATCATTTCTCAATAATTCACCACTTCGGATTTGAAGCAGCAGCC   3049
m141102_iid=754225_0        --ACGTCATCTAATAAATCATTTCTCAATAATTCACCACTTCGGATTTGAAGCAGCAGCC   4214
m141107_iid=1409934_0       --ACGTCATCTAATAAATCATTTCTCAATAATTCACCACTTCGGATTTGAAGCAGCAGCC   4757
m150503_iid=3416920_0       --ACGTCATCTAATAAATCATTTCTCAATAATTCACCACTTCGGATTTGAAGCAGCAGCC   3481
m141108_iid=1800121_0       --ACGTCATCTAATAAATCATTTCTCAATAATTCACCACTTCGGATTTGAAGCAGCAGCC   3292
m141031_iid=147540_0        A--CGTCATCTAATAAATCATTTCTCAATAATTCACCACTTCGGATTTGAAGCAGCAGCC   3633
m150507_iid=3674924_0       ACTCGTCATCTAATAAATCATTTCTCAATAATTCACCACTTCGGATTTGAAGCAGCAGCC   3786
m150503_iid=2923751_0       A--CGTCATCTAATAAATCATTTCTCAATAATTCACCACTTCGGATTTGAAGCAGCAGCC   3627
m141031_iid=115118_0        A--CGTCATCTAATAAATCATTTCTCAATAATTCACCACTTCGGATTTGAAGCAGCAGCC   2478
m150430_iid=2722448_0       A--CGTCATCTAATAAATCATTTCTCAATAATTCACCACTTCGGATTTGAAGCAGCAGCC   2420
m150507_iid=3102647_0       A--CGTCATCTAATAAATCATTTCTCAATAATTCACCACTTCGGATTTGAAGCAGCAGCC   723
m141108_iid=1811866_0       A--CGTCATCTAATAAATCATTTCTCAATAATTCACCACTTCGGATTTGAAGCAGCAGCC   2597
m150501_iid=2948495_0       A--CGTCATCTAATAAATCATTTCTCAATAATTCACCACTTCGGATTTGAAGCAGCAGCC   2021
```

```
m150504_iid=3238195_0      A--CGTCATCTAATAAATCATTTCTCAATAATTCACCACTTCGGATTTGAAGCAGCAGCC   4352
m150503_iid=3388511_0      A--CGTCATCTAATAAATCATTTCTCAATAATTCACCACTTCGGATTTGAAGCAGCAGCC   2049
m150505_iid=3777611_0      A--CGTCATCTAATAAATCATTTCTCAATAATTCACCACTTCGGATTTGAAGCAGCAGCC   3467
m150430_iid=2438833_0      A--CGTCATCTAATAAATCATTTCTCAATAATTCACCACTTCGGATTTGAAGCAGCAGCC   3808
m141108_iid=1698969_0      A--CGTCATCTAATAAATCATTTCTCAATAATTCACCACTTCGGATTTGAAGCAGCAGCC   4934
m150430_iid=3059190_0      A--CGTCATCTAATAAATCATTTCTCAATAATTCACCACTTCGGATTTGAAGCAGCAGCC   656
m141107_iid=1383067_0      A--CGTCATCTAATAAATCATTTCTCAATAATTCACCACTTCGGATTTGAAGCAGCAGCC   668
m141101_iid=597008_0       ------------------------------------------------------------   0
m150506_iid=2596496_0      A--CGTCATCTAATAAATCATTTCTCAATAATTCACCACTTCGGATTTGAAGCAGCAGCC   5295
m141102_iid=910051_0       A--CGTCATCTAATAAATCATTTCTCAATAATTCACCACTTCGGATTTGAAGCAGCAGCC   4783
m150505_iid=3784017_0      A--CGTCATCTAATAAATCATTTCTCAATAATTCACCACTTCGGATTTGAAGCAGCAGCC   3582
m141101_iid=682151_0       A--CGTCATCTAATAAATCATTTCTCAATAATTCACCACTTCGGATTTGAAGCAGCAGCC   2653
m150501_iid=3748029_0      A--CGTCATCTAATAAATCATTTCTCAATAATTCACCACTTCGGATTTGAAGCAGCAGCC   3194
m150504_iid=2876272_0      A--CGTCATCTAATAAATCATTTCTCAATAATTCACCACTTCGGATTTGAAGCAGCAGCC   5058
NC_042820.1                A--CGTCATCTAATAAATCATTTCTCAATAATTCACCACTTCGGATTTGAAGCAGCAGCC   5301
m141102_iid=1228825_0      A--CGTCATCTAATAAATCATTTCTCAATAATTCACCACTTCGGATTTGAAGCAGCAGCC   5128
m150504_iid=2876274_0      A--CGTCATCTAATAAATCATTTCTCAATAATTCACCACTTCGGATTTGAAGCAGCAGCC   5131
m141030_iid=80036_0        A--CGTCATCTAATAAATCATTTCTCAATAATTCACCACTTCGGATTTGAAGCAGCAGCC   4893
m150506_iid=2596494_0      A--CGTCATCTAATAAATCATTTCTCAATAATTCACCACTTCGGATTTGAAGCAGCAGCC   5296
m150503_iid=2526862_0      A--CGTCATCTAATAAATCATTTCTCAATAATTCACCACTTCGGATTTGAAGCAGCAGCC   4980
m150506_iid=2567000_0      A--CGTCATCTAATAAATCATTTCTCAATAATTCACCACTTCGGATTTGAAGCAGCAGCC   2929
m150507_iid=3073271_0      A--CGTCATCTAATAAATCATTTCTCAATAATTCACCACTTCGGATTTGAAGCAGCAGCC   4233
m150504_iid=3156087_0      A--CGTCATCTAATAAATCATTTCTCAATAATTCACCACTTCGGATTTGAAGCAGCAGCC   4056
m150505_iid=2498502_0      A--CGTCATCTAATAAATCATTTCTCAATAATTCACCACTTCGGATTTGAAGCAGCAGCC   1706
m141102_iid=754227_0       A--CGTCATCTAATAAATCATTTCTCAATAATTCACCACTTCGGATTTGAAGCAGCAGCC   4268
m150507_iid=3469514_0      A--CGTCATCTAATAAATCATTTCTCAATAATTCACCACTTCGGATTTGAAGCAGCAGCC   4826
Rdo_MitoGenome             A--CGTCATCTAATAAATCATTTCTCAATAATTCACCACTTCGGATTTGAAGCAGCAGCC   5301
m141101_iid=614933_0       A--CGTCATCTAATAAATCATTTCTCAATAATTCACCACTTCGGATTTGAAGCAGCAGCC   3853

                                                     cox3

m141031_iid=347546_0       TGATACTGACACTTCGTAGACGTAGTTTGGCTATTCTTA-TATTTATCAATTTACTGATG   83
m150506_iid=3127414_0      TGATACTGACACTTCGTAGACGTAGTTTGGCTATTCTTA-TATTTATCAATTTACTGATG   1831
m141102_iid=1245517_0      TGATACTGACACTTCGTAGACGTAGTTTGGCTATTCTTA-TATTTATCAATTTACTGATG   3486
m141107_iid=1405727_0      TGATACTGACACTTCGTAGACGTAGTTTGGCTATTCTTA-TATTTATCAATTTACTGATG   669
m141102_iid=987614_0       TGATACTGACACTTCGTAGACGTAGTTTGGCTATTCTTA-TATTTATCAATTTACTGATG   3635
m141108_iid=1701429_0      TGATACTGACACTTCGTAGACGTAGTTTGGCTATTCTTAT-ATTTATCAATTTACTGATG   369
m141102_iid=987286_0       TGATACTGACACTTCGTAGACGTAGTTTGGCTATTCTTAT-ATTTATCAATTTACTGATG   1585
m150430_iid=3272125_0      TGATACTGACACTTCGTAGACGTAGTTTGGCTATTCTTAT-ATTTATCAATTTACTGATG   3108
m141102_iid=754225_0       TGATACTGACACTTCGTAGACGTAGTTTGGCTATTCTTA-TATTTATCAATTTACTGATG   4273
m141107_iid=1409934_0      TGATACTGACACTTCGTAGACGTAGTTTGGCTATTCTTTATATTTATCAATTTACTGATG   4817
m150503_iid=3416920_0      TGATACTGACACTTCGTAGACGTAGTTTGGCTATTCTTA-TATTTATCAATTTACTGATG   3540
m141108_iid=1800121_0      TGATACTGACACTTCGTAGACGTAGTTTGGCTATTCTTA-TATTTATCAATTTACTGATG   3351
m141031_iid=147540_0       TGATACTGACACTTCGTAGACGTAGTTTGGCTATTCTTA-TATTTATCAATTTACTGATG   3692
m150507_iid=3674924_0      TGATACTGACACTTCGTAGACGTAGTTTGGCTATTCTTA-TATTTATCAATTTACTGATG   3845
m150503_iid=2923751_0      TGATACTGACACTTCGTAGACGTAGTTTGGCTATTCTTA-TATTTATCAATTTACTGATG   3686
m141031_iid=115118_0       TGATACTGACACTTCGTAGACGTAGTTTGGCTATTCTTA-TATTTATCAATTTACTGATG   2537
m150430_iid=2722448_0      TGATACTGACACTTCGTAGACGTAGTTTGGCTATTCTTA-TATTTATCAATTTACTGATG   2479
m150507_iid=3102647_0      TGATACTGACACTTCGTAGACGTAGTTTGGCTATTCTTA-TATTTATCAATTTACTGATG   782
m141108_iid=1811866_0      TGATACTGACACTTCGTAGACGTAGTTTGGCTATTCTTA-TATTTATCAATTTACTGATG   2656
m150501_iid=2948495_0      TGATACTGACACTTCGTAGACGTAGTTTGGCTATTCTTA-TATTTATCAATTTACTGATG   2080
m150504_iid=3238195_0      TGATACTGACACTTCGTAGACGTAGTTTGGCTATTCTTA-TATTTATCAATTTACTGATG   4411
m150503_iid=3388511_0      TGATACTGACACTTCGTAGACGTAGTTTGGCTATTCTTA-TATTTATCAATTTACTGATG   2108
m150505_iid=3777611_0      TGATACTGACACTTCGTAGACGTAGTTTGGCTATTCTTA-TATTTATCAATTTACTGATG   3526
m150430_iid=2438833_0      TGATACTGACACTTCGTAGACGTAGTTTGGCTATTCTTA-TATTTATCAATTTACTGATG   3867
m141108_iid=1698969_0      TGATACTGACACTTCGTAGACGTAGTTTGGCTATTCTTA-TATTTATCAATTTACTGATG   4993
m150430_iid=3059190_0      TGATACTGACACTTCGTAGACGTAGTTTGGCTATTCTTA-TATTTATCAATTTACTGATG   715
m141107_iid=1383067_0      TGATACTGACACTTCGTAGACGTAGTTTGGCTATTCTTA-TATTTATCAATTTACTGATG   727
m141101_iid=597008_0       ------------------------------------------------------------   0
m150506_iid=2596496_0      TGATACTGACACTTCGTAGACGTAGTTTGGCTATTCTTA-TATTTATCAATTTACTGATG   5354
m141102_iid=910051_0       TGATACTGACACTTCGTAGACGTAGTTTGGCTATTCTTA-TATTTATCAATTTACTGATG   4842
m150505_iid=3784017_0      TGATACTGACACTTCGTAGACGTAGTTTGGCTATTCTTA-TATTTATCAATTTACTGATG   3641
m141101_iid=682151_0       TGATACTGACACTTCGTAGACGTAGTTTGGCTATTCTTA-TATTTATCAATTTACTGATG   2712
m150501_iid=3748029_0      TGATACTGACACTTCGTAGACGTAGTTTGGCTATTCTTA-TATTTATCAATTTACTGATG   3253
m150504_iid=2876272_0      TGATACTGACACTTCGTAGACGTAGTTTGGCTATTCTTA-TATTTATCAATTTACTGATG   5117
NC_042820.1                TGATACTGACACTTCGTAGACGTAGTTTGGCTATTCTTA-TATTTATCAATTTACTGATG   5360
m141102_iid=1228825_0      TGATACTGACACTTCGTAGACGTAGTTTGGCTATTCTTA-TATTTATCAATTTACTGATG   5187
m150504_iid=2876274_0      TGATACTGACACTTCGTAGACGTAGTTTGGCTATTCTTA-TATTTATCAATTTACTGATG   5190
m141030_iid=80036_0        TGATACTGACACTTCGTAGACGTAGTTTGGCTATTCTTA-TATTTATCAATTTACTGATG   4952
m150506_iid=2596494_0      TGATACTGACACTTCGTAGACGTAGTTTGGCTATTCTTA-TATTTATCAATTTACTGATG   5355
m150503_iid=2526862_0      TGATACTGACACTTCGTAGACGTAGTTTGGCTATTCTTA-TATTTATCAATTTACTGATG   5039
m150506_iid=2567000_0      TGATACTGACACTTCGTAGACGTAGTTTGGCTATTCTTA-TATTTATCAATTTACTGATG   2988
```

```
m150507_iid=3073271_0     TGATACTGACACTTCGTAGACGTAGTTTGGCTATTCTTA-TATTTATCAATTTACTGATG   4292
m150504_iid=3156087_0     TGATACTGACACTTCGTAGACGTAGTTTGGCTATTCTTA-TATTTATCAATTTACTGATG   4115
m150505_iid=2498502_0     TGATACTGACACTTCGTAGACGTAGTTTGGCTATTCTTA-TATTTATCAATTTACTGATG   1765
m141102_iid=754227_0      TGATACTGACACTTCGTAGACGTAGTTTGGCTATTCTTA-TATTTATCAATTTACTGATG   4327
m150507_iid=3469514_0     TGATACTGACACTTCGTAGACGTAGTTTGGCTATTCTTA-TATTTATCAATTTACTGATG   4885
Rdo_MitoGenome            TGATACTGACACTTCGTAGACGTAGTTTGGCTATTCTTA-TATTTATCAATTTACTGATG   5360
m141101_iid=614933_0      TGATACTGACACTTCGTAGACGTAGTTTGGCTATTCTTA-TATTTATCAATTTACTGATG   3912

                                                    cox3

m141031_iid=347546_0      AGGTAAATATTTATATAGTATA-AAAATTATTTTTAACTTCCAAATTAAAAGATCTAGAA   142
m150506_iid=3127414_0     AGGTAAATATTTATATAGTAAT-AAAATTATTTTTAACTTCCAAT-TAAAAGATCTAGAA   1889
m141102_iid=1245517_0     AGGTAAATATTTATATAGTATAAA-AATTATTTTTAACTTCCAAT-TAAAAGATCTAGAA   3544
m141107_iid=1405727_0     AGGTAAATATTTATATAGTATAAA-AATTATTTTTAACTTCCAAT-TAAAAGATCTAGAA   727
m141102_iid=987614_0      AGGTAAATATTTATATAGTATA-AAAATTATTTTTAACTTCCAAT-TAAAAGATCTAGAA   3693
m141108_iid=1701429_0     AGGTAAATATTTATATAGTATAAA-AATTATTTTTAACTTCCAAT-TAAAAGATCTAGAA   427
m141102_iid=987286_0      AGGTAAATATTTATATAGTATAAA-AATTATTTTTAACTTCCAAT-TAAAAGATCTAGAA   1643
m150430_iid=3272125_0     AGGTAAATATTTATATAGTATAAA-AATTATTTTTAACTTCCAAT-TAAAAGATCTAGAA   3166
m141102_iid=754225_0      AGGTAAATATTTATATAGTATAA-AAATTATTTTTAACTTCCAAT-TAAAAGATCTAGAA   4331
m141107_iid=1409934_0     AGGTAAATATTTATATATAGTATAAAATTATTTTTAACTTCCAAT-TAAAAGATCTAGAA   4876
m150503_iid=3416920_0     AGGTAAATATTTATATAGTATA-AAAATT-ATTTTAACTTCCAAT-TAAAAGATCTAGAA   3597
m141108_iid=1800121_0     AGGTAAATATTTATATAGTATA-AA-ATTATTTTTAACTTCCAAT-TAAAAGATCTAGAA   3408
m141031_iid=147540_0      AGGTAAATATTTATATAGTATA-AAAATTATTTTTAACTTCCAAT-TAAAAGATCTAGAA   3750
m150507_iid=3674924_0     AGGTAAATATTTATATAGTATA-AAAATTATTTTTAACTTCCAAT-TAAAAGATCTAGAA   3903
m150503_iid=2923751_0     AGGTAAATATTTATATAGTATA-AAAATTATTTTTAACTTCCAAT-TAAAAGATCTAGAA   3744
m141031_iid=115118_0      AGGTAAATATTTATATAGTATA-AAAATT-ATTTTAACTTCCAAT-TAAAAGATCTAGAA   2594
m150430_iid=2722448_0     AGGTAAATATTTATATAGTATA-AAAATTATTTTTAACTTCCAAT-TAAAAGATCTAGAA   2537
m150507_iid=3102647_0     AGGTAAATATTTATATAGTATA-AAAATTATTTTTAACTTCCAAT-TAAAAGATCTAGAA   840
m141108_iid=1811866_0     AGGTAAATATTTATATAGTATA-AAAATTATTTTTAACTTCCAAT-TAAAAGATCTAGAA   2714
m150501_iid=2948495_0     AGGTAAATATTTATATAGTATA-AAAATTATTTTTAACTTCCAAT-TAAAAGATCTAGAA   2138
m150504_iid=3238195_0     AGGTAAATATTTATATAGTATA-AAAATTATTTTTAACTTCCAAT-TAAAAGATCTAGAA   4469
m150503_iid=3388511_0     AGGTAAATATTTATATAGTATA-AAAATTATTTTTAACTTCCAAT-TAAAAGATCTAGAA   2166
m150505_iid=3777611_0     AGGTAAATATTTATATAGTATA-AAAATTATTTTTAACTTCCAAT-TAAAAGATCTAGAA   3584
m150430_iid=2438833_0     AGGTAAATATTTATATAGTATA-AAAATTATTTTTAACTTCCAAT-TAAAAGATCTAGAA   3925
m141108_iid=1698969_0     AGGTAAATATTTATATAGTATA-AAAATTATTTTTAACTTCCAAT-TAAAAGATCTAGAA   5051
m150430_iid=3059190_0     AGGTAAATATTTATATAGTATA-AAAATTATTTTTAACTTCCAAT-TAAAAGATCTAGAA   773
m141107_iid=1383067_0     AGGTAAATATTTATATAGTATA-AAAATTATTTTTAACTTCCAAT-TAAAAGATCTAGAA   785
m141101_iid=597008_0      ------------------------------------------------------------   0
m150506_iid=2596496_0     AGGTAAATATTTATATAGTATA-AAAATTATTTTTAACTTCCAAT-TAAAAGATCTAGAA   5412
m141102_iid=910051_0      AGGTAAATATTTATATAGTATA-AAAATTATTTTTAACTTCCAAT-TAAAAGATCTAGAA   4900
m150505_iid=3784017_0     AGGTAAATATTTATATAGTAT--AAAATTATTTTTAACTTCCAAT-TAAAAGATCTAGAA   3698
m141101_iid=682151_0      AGGTAAATATTTATATAGTATA-AAAATTATTTTTAACTTCCAAT-TAAAAGATCTAGAA   2770
m150501_iid=3748029_0     AGGTAAATATTTATATAGTATA-AAAATTATTTTTAACTTCCAAT-TAAAAGATCTAGAA   3311
m150504_iid=2876272_0     AGGTAAATATTTATATAGTATA-AAAATTATTTTTAACTTCCAAT-TAAAAGATCTAGAA   5175
NC_042820.1               AGGTAGATATTTATATAGTATA-AAAATTATTTTTAACTTCCAAT-TAAAAGATCTAGAA   5418
m141102_iid=1228825_0     AGGTAAATATTTATATAGTATA-AAAATTATTTTTAACTTCCAAT-TAAAAGATCTAGAA   5245
m150504_iid=2876274_0     AGGTAAATATTTATATAGTATA-AAAATTATTTTTAACTTCCAAT-TAAAAGATCTAGAA   5248
m141030_iid=80036_0       AGGTAAATATTTATATAGTATA-AAAATTATTTTTAACTTCCAAT-TAAAAGATCTAGAA   5010
m150506_iid=2596494_0     AGGTAAATATTTATATAGTATA-AAAATTATTTTTAACTTCCAAT-TAAAAGATCTAGAA   5413
m150503_iid=2526862_0     AGGTAAATATTTATATAGTATA-AAAATTATTTTTAACTTCCAAT-TAAAAGATCTAGAA   5097
m150506_iid=2567000_0     AGGTAAATATTTATATAGTATA-AAAATTATTTTTAACTTCCAAT-TAAAAGATCTAGAA   3046
m150507_iid=3073271_0     AGGTAAATATTTATATAGTATA-AAAATTATTTTTAACTTCCAAT-TAAAAGATCTAGAA   4350
m150504_iid=3156087_0     AGGTAAATATTTATATAGTATA-AAAATTATTTTTAACTTCCAAT-TAAAAGATCTAGAA   4173
m150505_iid=2498502_0     AGGTAAATATTTATATAGTATA-AAAATTATTTTTAACTTCCAAT-TAAAAGATCTAGAA   1823
m141102_iid=754227_0      AGGTAAATATTTATATAGTATA-AAAATTATTTTTAACTTCCAAT-TAAAAGATCTAGAA   4385
m150507_iid=3469514_0     AGGTAAATATTTATATAGTATA-AAAATTATTTTTAACTTCCAAT-TAAAAGATCTAGAA   4943
Rdo_MitoGenome            AGGTAAATATTTATATAGTATA-AAAATTATTTTTAACTTCCAAT-TAAAAGATCTAGAA   5418
m141101_iid=614933_0      AGGTAAATATTTATATAGTATA-AAAATTATTTTTAACTTCCAAT-TAAAAGATCTAGAA   3970

                                                    cox3

m141031_iid=347546_0      ATAGTATAAATAATAAAAAATTATTTTAATTTCAAAGAATAATATCTTTTCAATCAGATT   202
m150506_iid=3127414_0     ATAGTATAAATAATAAAA-ATTATTTTAATTTCAAGAATAATTATCTTTTCAATCAGATT   1948
m141102_iid=1245517_0     ATAGTATAAATAATAAA-AATTATTTTAATTTCAAGAATAATTATCTTTTCAATCAGATT   3603
m141107_iid=1405727_0     ATAGTATAAATAATAAA---ATTATTTTATTTCAAGAATAATTATCTTTTCAATCAGATT   784
m141102_iid=987614_0      ATAGTATAAATAATAAA-AATTATTTTAATTTCAAGAATAATTATCTTTTCAATCAGATT   3752
m141108_iid=1701429_0     ATAGTATAAATAATAAA-AATTATTTTAATTTCAAGAATAATTATCTTTTCAATCAGATT   486
m141102_iid=987286_0      ATAGTATAAATAAT-AAAAATTATTTTAATTTCAAGAATAATTATCTTTTCAATCAGATT   1702
m150430_iid=3272125_0     ATAGTAAATAAATAATAAAATTATTTTAATTTCAAGAATAATTATCTTTTCAATCAGATT   3226
m141102_iid=754225_0      ATAGTATAAATAATAAA-AATTATTTTAATTTCAAGAATAATTATCTTTTCAATCAGATT   4390
m141107_iid=1409934_0     ATAGTATAAATAATAAAAAATTATTTTAATTTCAAGAATAATTATCTTTTCAATCAGATT   4936
m150503_iid=3416920_0     ATAGTATAAATAATAAA-AATTATTTTAATTTCAAGAATAATTATCTTTTCAATCAGATT   3656
m141108_iid=1800121_0     ATAGTATAAATAATAA--AATTATTTTAATTTCAAGAATAATTATCTTTTCAATCAGATT   3466
```

```
m141031_iid=147540_0        ATAGTATAAATAATAAA-AATTATTTTAATTTCAAGAATAATTATCTTTTCAATCAGATT   3809
m150507_iid=3674924_0       ATAGTATAAATAATAAA-AATTATTTTAATTTCAAGAATAATTATCTTTTCAATCAGATT   3962
m150503_iid=2923751_0       ATAGTATAAATAATAAA-AATTATTTTAATTTCAAGAATAATTATCTTTTCAATCAGATT   3803
m141031_iid=115118_0        ATAGTATAAATAATAAA-AATTATTTTAATTTCAAGAATAATTATCTTTTCAATCAGATT   2653
m150430_iid=2722448_0       ATAGTATAAATAATAAA-AATTATTTTAATTTCAAGAATAATTATCTTTTCAATCAGATT   2596
m150507_iid=3102647_0       ATAGTATAAATAATAAA-AATTATTTTAATTTCAAGAATAATTATCTTTTCAATCAGATT   899
m141108_iid=1811866_0       ATAGTATAAATAATAAA-AATTATTTTAATTTCAAGAATAATTATCTTTTCAATCAGATT   2773
m150501_iid=2948495_0       ATAGTATAAATAATAAA-AATTATTTTAATTTCAAGAATAATTATCTTTTCAATCAGATT   2197
m150504_iid=3238195_0       ATAGTATAAATAATAAA-AATTATTTTAATTTCAAGAATAATTATCTTTTCAATCAGATT   4528
m150503_iid=3388511_0       ATAGTATAAATAATAAA-AATTATTTTAATTTCAAGAATAATTATCTTTTCAATCAGATT   2225
m150505_iid=3777611_0       ATAGTATAAATAATAAA-AATTATTTTAATTTCAAGAATAATTATCTTTTCAATCAGATT   3643
m150430_iid=2438833_0       ATAGTATAAATAATAAA-AATTATTTTAATTTCAAGAATAATTATCTTTTCAATCAGATT   3984
m141108_iid=1698969_0       ATAGTATAAATAATAAA-AATTATTTTAATTTCAAGAATAATTATCTTTTCAATCAGATT   5110
m150430_iid=3059190_0       ATAGTATAAATAATAAA-AATTATTTTAATTTCAAGAATAATTATCTTTTCAATCAGATT   832
m141107_iid=1383067_0       ATAGTATAAATAATAAA-A--AATTTTAATTTCAAGAATAATTATCTTTTCAATCAGATT   842
m141101_iid=597008_0        ------------------------------------------------------------   0
m150506_iid=2596496_0       ATAGTATAAATAATAAA-AATTATTTTAATTTCAAGAATAATTATCTTTTCAATCAGATT   5471
m141102_iid=910051_0        ATAGTATAAATAATAAA-AATTATTTTAATTTCAAGAATAATTATCTTTTCAATCAGATT   4959
m150505_iid=3784017_0       ATAGTATAAATAATAAA-AATTATTTTAATTTCAAGAATAATTATCTTTTCAATCAGATT   3757
m141101_iid=682151_0        ATAGTATAAATAATAAA-AATTATTTTAATTTCAAGAATAATTATCTTTTCAATCAGATT   2829
m150501_iid=3748029_0       ATAGTATAAATAATAAA-AATTATTTTAATTTCAAGAATAATTATCTTTTCAATCAGATT   3370
m150504_iid=2876272_0       ATAGTATAAATAATAAA-AATTATTTTAATTTCAAGAATAATTATCTTTTCAATCAGATT   5234
NC_042820.1                 ATAGTATAAATAATAAA-AATTATTTTAATTTCAAGAATAATTATCTTTTCAATCAGATT   5477
m141102_iid=1228825_0       ATAGTATAAATAATAAA-AATTATTTTAATTTCAAGAATAATTATCTTTTCAATCAGATT   5304
m150504_iid=2876274_0       ATAGTATAAATAATA-A-AATTATTTTAATTTCAAGAATAATTATCTTTTCAATCAGATT   5306
m141030_iid=80036_0         ATAGTATAAATAAT--A-AATTATTTTAATTTCAAGAATAATTATCTTTTCAATCAGATT   5067
m150506_iid=2596494_0       ATAGTATAAATAATAAA-AATTATTTTAATTTCAAGAATAATTATCTTTTCAATCAGATT   5472
m150503_iid=2526862_0       ATAGTATAAATAATAAA-AATTATTTTAATTTCAAGAATAATTATCTTTTCAATCAGATT   5156
m150506_iid=2567000_0       ATAGTATAAATAATAAA-AATTATTTTAATTTCAAGAATAATTATCTTTTCAATCAGATT   3105
m150507_iid=3073271_0       ATAGTATAAATAATAAA-AATTATTTTAATTTCAAGAATAATTATCTTTTCAATCAGATT   4409
m150504_iid=3156087_0       ATAGTATAAATAATAAA-AATTATTTTAATTTCAAGAATAATTATCTTTTCAATCAGATT   4232
m150505_iid=2498502_0       ATAGTATAAATAATAAA-AATTATTTTAATTTCAAGAATAATTATCTTTTCAATCAGATT   1882
m141102_iid=754227_0        ATAGTATAAATAATAAA-AATTATTTTAATTTCAAGAATAATTATCTTTTCAATCAGATT   4444
m150507_iid=3469514_0       ATAGTATAAATAATAAA-AATTATTTTAATTTCAAGAATAATTATCTTTTCAATCAGATT   5002
Rdo_MitoGenome              ATAGTATAAATAATAAA-AATTATTTTAATTTCAAGAATAATTATCTTTTCAATCAGATT   5477
m141101_iid=614933_0        ATAGTATAAATAATAAA-AATTATTTTAATTTCAAGAATAATTATCTTTTCAATCAGATT   4029

                              cox3        nad3

m141031_iid=347546_0        AATCTTAATGATAATAAATCAAGTAATCTCAAAAAAAACATTTAAAGACCGAGAAAA-AA   261
m150506_iid=3127414_0       AATCTTAATGATAATAAATCAAGTAATCTCAAAAAAAACATTTAAAGACG-AGA-AA-AA   2005
m141102_iid=1245517_0       AATCTTAATGATAATAAATCAAGTAATCTCAAAA-AAACATTTAAAGACCGAGAAAAA--   3660
m141107_iid=1405727_0       AATCTTAATGATAATAAATCAAGTAATCTCAAAAAAAACATTTAAAGACCGAGAAA--AA   842
m141102_iid=987614_0        AATCTTAATGATAATAAATCAAGTAATCTCAAAAAAAACATTTAAAGACCGAGAAA--AA   3810
m141108_iid=1701429_0       AATCTTAATGATAATAAATCAAGTAATCTCAAAAAAAACATTTAAAGACCGAGAAAA-AA   545
m141102_iid=987286_0        AATCTTAATGATAATAAATCAAGTAATCTCAAAAAAAACATTTAAAGACCGAGAAAAAAA   1762
m150430_iid=3272125_0       AATCTTAATGATAATAAATCAAGTAATCTCAAAA-AAACATTTAAAGACCGAGAAA--AA   3283
m141102_iid=754225_0        AATCTTAATGATAATAAATCAAGTAATCTCAAAAAAAACATTTAAAGACCGAGAAAA-AA   4449
m141107_iid=1409934_0       AATCTTAATGATAATAAATCAAGTAATCTCAAAAAAAACATTTAAAGACCGAGAAAA-AA   4995
m150503_iid=3416920_0       AATCTTAATGATAATAAATCAAGTAATCTCAAAA-AAACATTTAAAGACCGAGAAAA-AA   3714
m141108_iid=1800121_0       AATCTTAATGATAATAAATCAAGTAATCTCAAAAAAAACATTTAAAGACCGAGAAAA-AA   3525
m141031_iid=147540_0        AATCTTAATGATAATAAATCAAGTAATCTCAAAAAAAACATTTAAAGACCGAGAAAA-AA   3868
m150507_iid=3674924_0       AATCTTAATGATAATAAATCAAGTAATCTCAAAAAAAACATTTAAAGACCGAGAAAA-AA   4021
m150503_iid=2923751_0       AATCTTAATGATAATAAATCAAGTAATCTCAAAAAAAACATTTAAAGACCGAGAAAA-AA   3862
m141031_iid=115118_0        AATCTTAATGATAATAAATCAAGTAATCTCAAAAAAAACATTTAAAGACCGAGAAAA-AA   2712
m150430_iid=2722448_0       AATCTTAATGATAATAAATCAAGTAATCTCAAAA-AAACATTTAAAGACCGAGAAAA--A   2653
m150507_iid=3102647_0       AATCTTAATGATAATAAATCAAGTAATCTCAAAAAAAACATTTAAAGACCGAGAAAA-AA   958
m141108_iid=1811866_0       AATCTTAATGATAATAAATCAAGTAATCTCAAAA-AAACATTTAAAGACCGAGAAAA-AA   2831
m150501_iid=2948495_0       AATCTTAATGATAATAAATCAAGTAATCTCAAAAAAAACATTTAAAGACCGAGAAAA-AA   2256
m150504_iid=3238195_0       AATCTTAATGATAATAAATCAAGTAATCTCAAAAAAAACATTTAAAGACCGAGAAAA-AA   4587
m150503_iid=3388511_0       AATCTTAATGATAATAAATCAAGTAATCTC-AAAAAAACATTTAAAGACCGAGAAAA-AA   2283
m150505_iid=3777611_0       AATCTTAATGATAATAAATCAAGTAATCTCAAAAAAAACATTTAAAGACCGAGAAAA-AA   3702
m150430_iid=2438833_0       AATCTTAATGATAATAAATCAAGTAATCTCAAAAAAAACATTTAAAGACCGAGAAAA-AA   4043
m141108_iid=1698969_0       AATCTTAATGATAATAAATCAAGTAATCTCAAAAAAAACATTTAAAGACCGAGAAAA-AA   5169
m150430_iid=3059190_0       AATCTTAATGATAATAAATCAAGTAATCTCAAAAAAAACATTTAAAGACCGAGAAAA-AA   891
m141107_iid=1383067_0       AATCTTAATGATAATAAATCAAGTAATCTCAAAAAAAACATTTAAAGACCGAGAAAA-AA   901
m141101_iid=597008_0        ------------------------------------------------------------   0
m150506_iid=2596496_0       AATCTTAATGATAATAAATCAAGTAATCTCAAAAAAAACATTTAAAGACCGAGAAAA-AA   5530
m141102_iid=910051_0        AATCTTAATGATAATAAATCAAGTAATCTCAAAA-AAACATTTAAAGACCGAGAAAA-AA   5017
m150505_iid=3784017_0       AATCTTAATGATAATAAATCAAGTAATCTCAAAAAAAACATTTAAAGACCGAGAAAA-AA   3816
m141101_iid=682151_0        AATCTTAATGATAATAAATCAAGTAATCTCAAAAAAAACATTTAAAGACCGAGAAAA-AA   2888
m150501_iid=3748029_0       AATCTTAATGATAATAAATCAAGTAATCTCAAAAAAAACATTTAAAGACCGAGAAAA-AA   3429
```

```
m150504_iid=2876272_0      AATCTTAATGATAATAAATCAAGTAATCTCAAAAAAAACATTTAAAGACCGAGAAAA-AA   5293
NC_042820.1                AATCTTAATGATAATAAATCAAGTAATCTCAAAAAAAACATTTAAAGACCGAGAAAA-AA   5536
m141102_iid=1228825_0      AATCTTAATGATAATAAATCAAGTAATCTCAAAAAAAACATTTAAAGACCGAGAAAA-AA   5363
m150504_iid=2876274_0      AATCTTAATGATAATAAATCAAGTAATCTCAAAAAAAACATTTAAAGACCGAGAAAA-AA   5365
m141030_iid=80036_0        AATCTTAATGATAATAAATCAAGTAATCTCAAAAAAAACATTTAAAGACCGAGAAAA-AA   5126
m150506_iid=2596494_0      AATCTTAATGATAATAAATCAAGTAATCTCAAAAAAAACATTTAAAGACCGAGAAAA-AA   5531
m150503_iid=2526862_0      AATCTTAATGATAATAAATCAAGTAATCTCAAAAAAAACATTTAAAGACCGAGAAAA-AA   5215
m150506_iid=2567000_0      AATCTTAATGATAATAAATCAAGTAATCTCAAAAAAAACATTTAAAGACCGAGAAAA-AA   3164
m150507_iid=3073271_0      AATCTTAATGATAATAAATCAAGTAATCTCAAAAAAAACATTTAAAGACCGAGAAAA-AA   4468
m150504_iid=3156087_0      AATCTTAATGATAATAAATCAAGTAATCTCAAAAAAAACATTTAAAGACCGAGAAAA-AA   4291
m150505_iid=2498502_0      AATCTTAATGATAATAAATCAAGTAATCTCAAAAAAAACATTTAAAGACCGAGAAAA-AA   1941
m141102_iid=754227_0       AATCTTAATGATAATAAATCAAGTAATCTCAAAAAAAACATTTAAAGACCGAGAAAA-AA   4503
m150507_iid=3469514_0      AATCTTAATGATAATAAATCAAGTAATCTCAAAAAAAACATTTAAAGACCGAGAAAA-AA   5061
Rdo_MitoGenome             AATCTTAATGATAATAAATCAAGTAATCTCAAAAAAAACATTTAAAGACCGAGAAAA-AA   5536
m141101_iid=614933_0       AATCTTAATGATAATAAATCAAGTAATCTCAAAAAAAACATTTAAAGACCGAGAAAA-AA   4088

                                                  nad3

m141031_iid=347546_0       TATCCCCATATGAATGTGGGT-TGACCCAAAATCACACGCCCG--TATTCCATTATC-AA   317
m150506_iid=3127414_0      TATCCCCATATGAATGTGGGTTTGACCCAAAATCACACGCCCG--TATTCCATTATCCGA   2063
m141102_iid=1245517_0      ATATCCCATATGAATGTGGGTTTGACCCAAAATCACACGCCCGT--ATTCCATTATC-AA   3717
m141107_iid=1405727_0      TATCCCCATATGAATGTGGGTTTGACCCAA-ATCACACGCCCGT--ATTCCATTATC-AA   898
m141102_iid=987614_0       ATATCCCATATGAATGTGGGTTTGACCCAAAATCACACGCCCG--TATTCCATTATC-AA   3867
m141108_iid=1701429_0      TATCCCCATATGAATGTGGGTTTGACCCAAAATCACACGCCCGT--ATTCCATTATC-AA   602
m141102_iid=987286_0       TATCCCCATATGAATGTGGGTTTGACCCAAAATCACACGCCCGT--ATTCCATTATC-AA   1819
m150430_iid=3272125_0      TATCCCCATATGAATGTGGGTTTGACCCAAAATCACACGCCCGT--ATTCCATTATC-AA   3340
m141102_iid=754225_0       TATCCCCATATGAATGTGGGTTTGACCCAAAATCACACGCCCGT--ATTCCATTATC-AA   4506
m141107_iid=1409934_0      TATCCCCATATGAATGTGGGTTTGACCCAAAATCACACGCCCGT--ATTCCATTATC-AA   5052
m150503_iid=3416920_0      TATCCCCATATGAATGTGGGTTTGACCCAAAATCACACGCCCGT--ATTCCATTATC-AA   3771
m141108_iid=1800121_0      TATCCCCATATGAATGTGGGTTTGACCCAAAATCACACGCCCGT--ATTCCATTATC-AA   3582
m141031_iid=147540_0       TATCCCCATATGAATGTGGGTTTGACCCAAAATCACACGCCCG--TATTCCATTATC-AA   3925
m150507_iid=3674924_0      TATCCCCATATGAATGTGGGTTTGACCCAAAATCACACGCCCGT--ATTCCATTATC-AA   4078
m150503_iid=2923751_0      TATCCCCATATGAATGTGGGTTTGACCCAAAATCACACGCCCGT--ATTCCATTATC-AA   3919
m141031_iid=115118_0       TATCCCCATATGAATGTGGGTTTGACCCAAAATCACACGCCCGT--ATTCCATTATC-AA   2769
m150430_iid=2722448_0      AATCCCCATATGAATGTGGGTTTGACCCAAAATCACACGCCCGT--ATTCCATTATC-AA   2710
m150507_iid=3102647_0      TATCCCCATATGAATGTGGGTTTGACCCAAAATCACACGCCCGT--ATTCCATTATC-AA   1015
m141108_iid=1811866_0      TATCCCCATATGAATGTGG-TTTGACCCAAAATCACACGCCCGT--ATTCCATTATC-AA   2887
m150501_iid=2948495_0      TATCCCCATATGAATGTGGGTTTGACCCAAAATCACACGCCCGT--ATTCCATTATC-AA   2313
m150504_iid=3238195_0      TATCCCCATATGAATGTGGGTTTGACCCAAAATCACACGCCCGT--ATTCCATTATC-AA   4644
m150503_iid=3388511_0      TATCCCCATATGAATGTGGGTTTGACCCAAAATCACACGCCCG--TATTCCATTATC-AA   2340
m150505_iid=3777611_0      TATCCCCATATGAATGTGGGTTTGACCCAAAATCACACGCCCGTCTATTCCATTATC-AA   3761
m150430_iid=2438833_0      TATCCCCATATGAATGTGGGTTTGACCCAAAATCACACGCCCG--TATTCCATTATC-AA   4100
m141108_iid=1698969_0      TATCCCCATATGAATGTGGGTTTGACCCAAAATCACACGCCCG--TATTCCATTATC-AA   5226
m150430_iid=3059190_0      TATCCCCATATGAATGTGGGTTTGACCCAAAATCACACGCCCG--TATTCCATTATC-AA   948
m141107_iid=1383067_0      TATCCCCATATGAATGTGGGTTTGACCCAAAATCACACGCCCG--TATTCCATTATC-AA   958
m141101_iid=597008_0       ------------------------------------------------------------   0
m150506_iid=2596496_0      TATCCCCATATGAATGTGGGTTTGACCCAAAATCACACGCCCG--TATTCCATTATC-AA   5587
m141102_iid=910051_0       TATCCCCATATGAATGTGGGTTTGACCCAAAATCACACGCCCG--TATTCCATTATC-AA   5074
m150505_iid=3784017_0      TATCCCCATATGAATGTGGGTTTGACCCAAAATCACACGCCCG--TATTCCATTATC-AA   3873
m141101_iid=682151_0       TAT-CCCATATGAATGTGGGTTTGACCCAAAATCACACGCCCG--TATTCCATTATC-AA   2944
m150501_iid=3748029_0      TATCCCCATATGAATGTGGGTTTGACCCAAAATCACACGCCCG--TATTCCATTATC-AA   3486
m150504_iid=2876272_0      TATCCCCATATGAATGTGGGTTTGACCCAAAATCACACGCCCG--TATTCCATTATC-AA   5350
NC_042820.1                TATCCCCATATGAATGTGGGTTTGACCCAAAATCACACGCCCG--TATTCCATTATC-AA   5593
m141102_iid=1228825_0      TATCCCCATATGAATGTGGGTTTGACCCAAAATCACACGCCCG--TATTCCATTATC-AA   5420
m150504_iid=2876274_0      TATCCCCATATGAATGTGGGTTTGACCCAAAATCACACGCCCG--TATTCCATTATC-AA   5422
m141030_iid=80036_0        TATCCCCATATGAATGTGGGTTTGACCCAAAATCACACGCCCG--TATTCCATTATC-AA   5183
m150506_iid=2596494_0      TATCCCCATATGAATGTGGGTTTGACCCAAAATCACACGCCCG--TATTCCATTATC-AA   5588
m150503_iid=2526862_0      TATCCCCATATGAATGTGGGTTTGACCCAAAATCACACGCCCG--TATTCCATTATC-AA   5272
m150506_iid=2567000_0      TATCCCCATATGAATGTGGGTTTGACCCAAAATCACACGCCCG--TATTCCATTATC-AA   3221
m150507_iid=3073271_0      TATCCCCATATGAATGTGGGTTTGACCCAAAATCACACGCCCG--TATTCCATTATC-AA   4525
m150504_iid=3156087_0      TATCCCCATATGAATGTGGGTTTGACCCAAAATCACACGCCCG--TATTCCATTATC-AA   4348
m150505_iid=2498502_0      TATCCCCATATGAATGTGGGTTTGACCCAAAATCACACGCCCG--TATTCCATTATC-AA   1998
m141102_iid=754227_0       TATCCCCATATGAATGTGGGTTTGACCCAAAATCACACGCCCG--TATTCCATTATC-AA   4560
m150507_iid=3469514_0      TATCCCCATATGAATGTGGGTTTGACCCAAAATCACACGCCCG--TATTCCATTATC-AA   5118
Rdo_MitoGenome             TATCCCCATATGAATGTGGGTTTGACCCAAAATCACACGCCCG--TATTCCATTATC-AA   5593
m141101_iid=614933_0       TATCCCCATATGAATGTGGGTTTGACCCAAAATCACACGCCCG--TATTCCATTATC-AA   4145

                                                  nad3

m141031_iid=347546_0       TCCGATTT-TTTCTTATCACAATAATCTTTCTAAT-TTTGATGTAGAAATTACTCTTCT-   374
m150506_iid=3127414_0      ACCGATTT-TTTCTTATCACAATAATCTTTCTAATTTTTGATGTAGAAATTACTCTTCT-   2121
m141102_iid=1245517_0      TCCGATTT-TTTCTTATCACAATAATCTTTCTAATTTTTGATGTAGAAATTACTCTTCT-   3775
m141107_iid=1405727_0      TCCGATTT-TTTCTTATCACAATAATCTTTCTAATTTTTGATGTAGAAATTACTCTT--T   955
```

```
m141102_iid=987614_0       TCCGATTT-TTTCTTATCACAATAATCTTTCTAATTTTTGATGTAGAAATTACTCTTCT-   3925
m141108_iid=1701429_0      TCCGATTT-TTTCTTATCACAATAATCTTTCTAATTTTTGATGTAGAAATTACTCTTCT-   660
m141102_iid=987286_0       TCCGATTT-TTTCTTATCACAATAATCTTTCTAATTTTTGATGTAGAAATTACTCTTCT-   1877
m150430_iid=3272125_0      TCCGATTTTTTTCTTATCACAATAATCTTTCTAATTTTTGATGTAGAAATTACTCTTCT-   3399
m141102_iid=754225_0       TCCGATTT-TTTCTTATCACAATAATCTTTCTAATTTTTGATGTAGAAATTACTCTTCT-   4564
m141107_iid=1409934_0      TCCGATTT-TTTCTTATCACAATAATCTTTCTAATTTTTGATGTAGAAATTACTCTTCT-   5110
m150503_iid=3416920_0      TCCGATTT-TTTCTTATCACAATAATCTTTCTAATTTTTGATGTAGAAATTACTCTTCT-   3829
m141108_iid=1800121_0      TCCGATTT-TTTCTTATCACAATAATCTTTCTAATTTTTGATGTAGAAATTACTCTTCT-   3640
m141031_iid=147540_0       TCCGATTT-TTTCTTATCACAATAATCTTTCTAATTTTTGATGTAGAAATTACTCTTCT-   3983
m150507_iid=3674924_0      TCCGATTT-TTTCTTATCACAATAATCTTTCTAATTTTTGATGTAGAAATTACTCTTCT-   4136
m150503_iid=2923751_0      TCCGATTT-TTTCTTATCACAATAATCTTTCTAATTTTTGATGTAGAAATTACTCTTCT-   3977
m141031_iid=115118_0       TCCGATTT-TTTCTTATCACAATAATCTTTCTAATTTTTGATGTAGAAATTACTCTTCT-   2827
m150430_iid=2722448_0      TCCGATTT-TTTCT-ATCACAATAATCTTTCTAATTTTTGATGTAGAAATTACTCTTCT-   2767
m150507_iid=3102647_0      TCCGATTT-TTTCTTATCACAATAATCTTTCTAATTTTTGATGTAGAAATTACTCTTCT-   1073
m141108_iid=1811866_0      TCCGATTT-TTTCTTATCACAATAATCTTTCTAATTTTTGATGTAGAAATTACTCTTCT-   2945
m150501_iid=2948495_0      TCCGATTT-TTTCTTATCACAATAATCTTTCTAATTTTTGATGTAGAAATTACTCTTCT-   2371
m150504_iid=3238195_0      TCCGATTT-TTTCTTATCACAATAATCTTTCTAATTTTTGATGTAGAAATTACTCTTCT-   4702
m150503_iid=3388511_0      TCCGATTT-TTTCTTATCACAATAATCTTTCTAATTTTTGATGTAGAAATTACTCTTCT-   2398
m150505_iid=3777611_0      TCCGATTT-TTTCTTATCACAATAATCTTTCTAATTTTTGATGTAGAAATTACTCTTCT-   3819
m150430_iid=2438833_0      TCCGATTT-TTTCTTATCACAATAATCTTTCTAATTTTTGATGTAGAAATTACTCTTCT-   4158
m141108_iid=1698969_0      TCCGATTT-TTTCTTATCACAATAATCTTTCTAATTTTTGATGTAGAAATTACTCTTCT-   5284
m150430_iid=3059190_0      TCCGATTT-TTTCTTATCACAATAATCTTTCTAATTTTTGATGTAGAAATTACTCTTCTT   1007
m141107_iid=1383067_0      TCCGATTT-TTTCTTATCACAATAATCTTTCTAATTTTTGATGTAGAAATTACTCTTCT-   1016
m141101_iid=597008_0       ------------------------------------------------------------   0
m150506_iid=2596496_0      TCCGATTT-TTTCTTATCACAATAATCTTTCTAATTTTTGATGTAGAAATTACTCTTCT-   5645
m141102_iid=910051_0       TCCGATTT-TTTCTTATCACAATAATCTTTCTAATTTTTGATGTAGAAATTACTCTTCT-   5132
m150505_iid=3784017_0      TCCGATTT-TTTCTTATCACAATAATCTTTCTAATTTTTGATGTAGAAATTACTCTTCT-   3931
m141101_iid=682151_0       TCCGATTT-TTTCTTATCACAATAATCTTTCTAATTTTTGATGTAGAAATTACTCTTCT-   3002
m150501_iid=3748029_0      TCCGATTT-TTTCTTATCACAATAATCTTTCTAATTTTTGATGTAGAAATTACTCTTCT-   3544
m150504_iid=2876272_0      TCCGATTT-TTTCTTATCACAATAATCTTTCTAATTTTTGATGTAGAAATTACTCTTCT-   5408
NC_042820.1                TCCGATTT-TTTCTTATTACAGTAATCTTTCTAATTTTTGATGTAGAAATTACTCTTCT-   5651
m141102_iid=1228825_0      TCCGATTT-TTT-TTATCACAATAATCTTTCTAATTTTTGATGTAGAAATTACTCTTCT-   5477
m150504_iid=2876274_0      TCCGATTT-TTTCTTATCACAATAATCTTTCTAATTTTTGATGTAGAAATTACTCTTCT-   5480
m141030_iid=80036_0        TCCGATTT-TTTCTTATCACAATAATCTTTCTAATTTTTGATGTAGAAATTACTCTTCT-   5241
m150506_iid=2596494_0      TCCGATTT-TTTCTTATCACAATAATCTTTCTAATTTTTGATGTAGAAATTACTCTTCT-   5646
m150503_iid=2526862_0      TCCGATTT-TTTCTTATCACAATAATCTTTCTAATTTTTGATGTAGAAATTACTCTTCT-   5330
m150506_iid=2567000_0      TCCGATTT-TTTCTTATCACAATAATCTTTCTAATTTTTGATGTAGAAATTACTCTTCT-   3279
m150507_iid=3073271_0      TCCGATTT-TTTCTTATCACAATAATCTTTCTAATTTTTGATGTAGAAATTACTCTTCT-   4583
m150504_iid=3156087_0      TCCGATTT-TTTCTTATCACAATAATCTTTCTAATTTTTGATGTAGAAATTACTCTTCT-   4406
m150505_iid=2498502_0      TCCGATTT-TTTCTTATCACAATAATCTTTCTAATTTTTGATGTAGAAATTACTCTTCT-   2056
m141102_iid=754227_0       TCCGATTT-TTTCTTATCACAATAATCTTTCTAATTTTTGATGTAGAAATTACTCTTCT-   4618
m150507_iid=3469514_0      TCCGATTT-TTTCTTATCACAATAATCTTTCTAATTTTTGATGTAGAAATTACTCTTCT-   5176
Rdo_MitoGenome             TCCGATTT-TTTCTTATCACAATAATCTTTCTAATTTTTGATGTAGAAATTACTCTTCT-   5651
m141101_iid=614933_0       TCCGATTT-TTTCTTATCACAATAATCTTTCTAATTTTTGATGTAGAAATTACTCTTCT-   4203
```

nad3

```
m141031_iid=347546_0       TCTTCCAGCAATTAATAACCTTAAAACTACAAATCCACTGAATTTCCTAATTACATTTAT   434
m150506_iid=3127414_0      TCTTCCAGCAATTAATAACCTTAAAACTACA-ATCCACTGGAATTCCTAATTACATTTAT   2180
m141102_iid=1245517_0      TCTTCCAGCAATTAATAACCTTAAAACTACA-ATCCACTGGAATTCCTAATTACATTTAT   3834
m141107_iid=1405727_0      TCTTCCAGCAATTAATAACCTTAAAACTACAAATCCACTGGAATTCCTAATTACATTTAT   1015
m141102_iid=987614_0       TCTTCCAGCAATTAATAACCTTAAAACTACAAATCCACTGGAATTCCTAATTACATTTAT   3985
m141108_iid=1701429_0      TCTTCCAGCAATTAATAACCTTAAAACTACAAATCCACTGGAATTCCTAATTACATTTAT   720
m141102_iid=987286_0       TCTTCCAGCAATTAATAACCTTAAAACTACAAATCCACTGGAATTCCTAATTACATTTAT   1937
m150430_iid=3272125_0      TCTTCCAGCAATTAATAACCTTAAAACTACAAATCCACTGGAATTCCTAATTACATTTAT   3459
m141102_iid=754225_0       TCTTCCAGCAATTAATAACCTTAAAACTACAAATCCACTGGAATTCCTAATTACATTTAT   4624
m141107_iid=1409934_0      TCTTCCAGCAATTAATAACCTTAAAACTACAAATCCACTGGAATTCCTAATTACATTTAT   5170
m150503_iid=3416920_0      TCTTCCAGCAATTAATAACCTTAAAACTACAAATCCACTGGAATTCCTAATTACATTTAT   3889
m141108_iid=1800121_0      TCTTCCAGCAATTAATAACCTTAAAACTACAAATCCACTGGAATTCCTAATTACATTTAT   3700
m141031_iid=147540_0       TCTTCCAGCAATTAATAACCTTAAAACTACAAATCCACTGGAATTCCTAATTACATTTAT   4043
m150507_iid=3674924_0      TCTTCCAGCAATTAATAACCTTAAAACTACAAATCCACTGGAATTCCTAATTACATTTAT   4196
m150503_iid=2923751_0      TCTTCCAGCAATTAATAACCTTAAAACTACAAATCCACTGGAATTCCTAATTACATTTAT   4037
m141031_iid=115118_0       TCTTCCAGCAATTAATAACCTTAAAACTACAAATCCACTGGAATTCCTAATTACATTTAT   2887
m150430_iid=2722448_0      TCTTCCAGCAATTAATAACCTTAAAACTACAAATCCACTGGAATTCCTAATTACATTTAT   2827
m150507_iid=3102647_0      TCTTCCAGCAATTAATAACCTTAAAACTACAAATCCACTGGAATTCCTAATTACATTTAT   1133
m141108_iid=1811866_0      TCTTCCAGCAATTAATAACCTTAAAACTACAAATCCACTGGAATTCCTAATTACATTTAT   3005
m150501_iid=2948495_0      TCTTCCAGCAATTAATAACCTTAAAACTACAAATCCACTGGAATTCCTAATTACATTTAT   2431
m150504_iid=3238195_0      TCTTCCAGCAATTAATAACCTTAAAACTACAAATCCACTGGAATTCCTAATTACATTTAT   4762
m150503_iid=3388511_0      TCTTCCAGCAATTAATAACCTTAAAACTACAAATCCACTGGAATTCCTAATTACATTTAT   2458
m150505_iid=3777611_0      TCTTCCAGCAATTAATAACCTTAAAACTACAAATCCACTGGAATTCCTAATTACATTTAT   3879
m150430_iid=2438833_0      TCTTCCAGCAATTAATAACCTTAAAACTACAAATCCACTGGAATTCCTAATTACATTTAT   4218
m141108_iid=1698969_0      TCTTCCAGCAATTAATAACCTTAAAACTACAAATCCACTGGAATTCCTAATTACATTTAT   5344
```

```
m150430_iid=3059190_0      TCTTCCAGCAATTAATAACCTTAAAACTACAAATCCACTGGAATTCCTAATTACATTTAT   1067
m141107_iid=1383067_0      TCTTCCAGCAATTAATAACCTTAAAACTACAAATCCACTGGAATTCCTAATTACATTTAT   1076
m141101_iid=597008_0       ------------------------------------------------------------   0
m150506_iid=2596496_0      TCTTCCAGCAATTAATAACCTTAAAACTACAAATCCACTGGAATTCCTAATTACATTTAT   5705
m141102_iid=910051_0       TCTTCCAGCAATTAATAACCTTAAAACTACAAATCCACTGGAATTCCTAATTACATTTAT   5192
m150505_iid=3784017_0      TCTTCCAGCAATTAATAACCTTAAAACTACAAATCCACTGGAATTCCTAATTACATTTAT   3991
m141101_iid=682151_0       TCTTCCAGCAATTAATAACCTTAAAACTACAAATCCACTGGAATTCCTAATTACATTTAT   3062
m150501_iid=3748029_0      TCTTCCAGCAATTAATAACCTTAAAACTACAAATCCACTGGAATTCCTAATTACATTTAT   3604
m150504_iid=2876272_0      TCTTCCAGCAATTAATAACCTTAAAACTACAAATCCACTGGAATTCCTAATTACATTTAT   5468
NC_042820.1                TCTTCCGGCAATTAATAACCTTAAAACTACAAATCCACTGGAATTCCTAATTACATTTAT   5711
m141102_iid=1228825_0      TCTTCCAGCAATTAATAACCTTAAAACTACAAATCCACTGGAATTCCTAATTACATTTAT   5537
m150504_iid=2876274_0      TCTTCCAGCAATTAATAACCTTAAAACTACAAATCCACTGGAATTCCTAATTACATTTAT   5540
m141030_iid=80036_0        TCTTCCAGCAATTAATAACCTTAAAACTACAAATCCACTGGAATTCCTAATTACATTTAT   5301
m150506_iid=2596494_0      TCTTCCAGCAATTAATAACCTTAAAACTACAAATCCACTGGAATTCCTAATTACATTTAT   5706
m150503_iid=2526862_0      TCTTCCAGCAATTAATAACCTTAAAACTACAAATCCACTGGAATTCCTAATTACATTTAT   5390
m150506_iid=2567000_0      TCTTCCAGCAATTAATAACCTTAAAACTACAAATCCACTGGAATTCCTAATTACATTTAT   3339
m150507_iid=3073271_0      TCTTCCAGCAATTAATAACCTTAAAACTACAAATCCACTGGAATTCCTAATTACATTTAT   4643
m150504_iid=3156087_0      TCTTCCAGCAATTAATAACCTTAAAACTACAAATCCACTGGAATTCCTAATTACATTTAT   4466
m150505_iid=2498502_0      TCTTCCAGCAATTAATAACCTTAAAACTACAAATCCACTGGAATTCCTAATTACATTTAT   2116
m141102_iid=754227_0       TCTTCCAGCAATTAATAACCTTAAAACTACAAATCCACTGGAATTCCTAATTACATTTAT   4678
m150507_iid=3469514_0      TCTTCCAGCAATTAATAACCTTAAAACTACAAATCCACTGGAATTCCTAATTACATTTAT   5236
Rdo_MitoGenome             TCTTCCAGCAATTAATAACCTTAAAACTACAAATCCACTGGAATTCCTAATTACATTTAT   5711
m141101_iid=614933_0       TCTTCCAGCAATTAATAACCTTAAAACTACAAATCCACTGGAATTCCTAATTACATTTAT   4263
```

nad3

```
m141031_iid=347546_0       TTTCTTCATCTCAATTTTAAACGTTAGGGACAATTCATGAATGAAAACAAGGAGCATTGA   494
m150506_iid=3127414_0      TTTCTTCATCTCAATTT-TAACGTTAGGGACAATTCATGAATGAAAACAAGGAGCATTGA   2239
m141102_iid=1245517_0      TTTCTTCATCTCAAT-TTTAACGTTAGGGACAATTCATGAATGAAAACAAGGAGCATTGA   3893
m141107_iid=1405727_0      TTTCTTCATCTCAAT-TTTAACGTTAGGGACAATTCATGAATGAAAACAAGGAGCATTGA   1074
m141102_iid=987614_0       TTTCTTCATCTCAAT-TTTAACGTTAGGGACAATTCATGAATGAAAACAAGGAGCATTGA   4044
m141108_iid=1701429_0      TTTCTTCATCTCAAT-TTTAACGTTAGGGACAATTCATGAATGAAAACAAGGAGCATTGA   779
m141102_iid=987286_0       TTTCTTCATCTCAAT-TTTAACGTTAGGGACAATTCATGAATGAAAACAAGGAGCATTGA   1996
m150430_iid=3272125_0      TTTCTTCATCTCAAATTTTAACGTTAGGGACAATTCATGAATGAAAACAAGGAGCATTGA   3519
m141102_iid=754225_0       TTTCTTCATCTCAAT-TTTAACGTTAGGGACAATTCATGAATGAAAACAAGGAGCATTGA   4683
m141107_iid=1409934_0      TTTCTTCATCTCAAT-TTTAACGTTAGGGACAATTCATGAATGAAAACAAGGAGCATTGA   5229
m150503_iid=3416920_0      TTTCTTCATCTCAAT-TTTAACGTTAGGGACAATTCATGAATGAAAACAAGGAGCATTGA   3948
m141108_iid=1800121_0      TTTCTTCATCTCAAT-TTTAACGTTAGGGACAATTCATGAATGAAAACAAGGAGCATTGA   3759
m141031_iid=147540_0       TTTCTTCATCTCAAT-TTTAACGTTAGGGACAATTCATGAATGAAAACAAGGAGCATTGA   4102
m150507_iid=3674924_0      TTTCTTCATCTCAAT-TTTAACGTTAGGGACAATTCATGAATGAAAACAAGGAGCATTGA   4255
m150503_iid=2923751_0      TTTCTTCATCTCAAT-TTTAACGTTAGGGACAATTCATGAATGAAAACAAGGAGCATTGA   4096
m141031_iid=115118_0       TTTCTTCATCTCAAT-TTTAACGTTAGGGACAATTCATGAATGAAAACAAGGAGCATTGA   2946
m150430_iid=2722448_0      TTTCTTCATCTCAAT-TTTAACGTTAGGGACAATTCATGAA---AAACAAGGAGCATTGA   2883
m150507_iid=3102647_0      TTTCTTCATCTCAAT-TTTAACGTTAGGGACAATTCATGAATGAAAACAAGGAGCATTGA   1192
m141108_iid=1811866_0      TTTCTTCATCTCAAT-TTTAACGTTAGGGACAATTCATGAATGAAAACAAGGAGCATTGA   3064
m150501_iid=2948495_0      TTTCTTCATCTCAAT-TTTAACGTTAGGGACAATTCATGAATGAAAACAAGGAGCATTGA   2490
m150504_iid=3238195_0      TTTCTTCATCTCAAT-TTTAACGTTAGGGACAATTCATGAATGAAAACAAGGAGCATTGA   4821
m150503_iid=3388511_0      TTTCTTCATCTCAAT-TTTAACGTTAGGGACAATTCATGAATGAAAACAAGGAGCATTGA   2517
m150505_iid=3777611_0      TTTCTTCATCTCAAT-TTTAACGTTAGGGACAATTCATGAATGAAAACAAGGAGCATTGA   3938
m150430_iid=2438833_0      TTTCTTCATCTCAAT-TTTAACGTTAGGGACAATTCATGAATGAAAACAAGGAGCATTGA   4277
m141108_iid=1698969_0      TTTCTTCATCTCAAT-TTTAACGTTAGGGACAATTCATGAATGAAAACAAGGAGCATTGA   5403
m150430_iid=3059190_0      TTTCTTCATCTCAAT-TTTAACGTTAGGGACAATTCATGAATGAAAACAAGGAGCATTGA   1126
m141107_iid=1383067_0      TTTCTTCATCTCAAT-TTTAACGTTAGGGACAATTCATGAATGAAAACAAGGAGCATTGA   1135
m141101_iid=597008_0       ------------------------------------------------------------   0
m150506_iid=2596496_0      TTTCTTCATCTCAAT-TTTAACGTTAGGGACAATTCATGAATGAAAACAAGGAGCATTGA   5764
m141102_iid=910051_0       TTTCTTCATCTCAAT-TTTAACGTTAGGGACAATTCATGAATGAAAACAAGGAGCATTGA   5251
m150505_iid=3784017_0      TTTCTTCATCTCAAT-TTTAACGTTAGGGACAATTCATGAATGAAAACAAGGAGCATTGA   4050
m141101_iid=682151_0       TTTCTTCATCTCAAT-TTTAACGTTAGGGACAATTCATGAATGAAAACAAGGAGCATTGA   3121
m150501_iid=3748029_0      TTTCTTCATCTCAAT-TTTAACGTTAGGGACAATTCATGAATGAAAACAAGGAGCATTGA   3663
m150504_iid=2876272_0      TTTCTTCATCTCAAT-TTTAACGTTAGGGACAATTCATGAATGAAAACAAGGAGCATTGA   5527
NC_042820.1                TTTCTTCATCTCAAT-TTTAACGTTAGGGACAATTCATGAATGAAAACAAGGAGCATTGA   5770
m141102_iid=1228825_0      TTTCTTCATCTCAAT-TTTAACGTTAGGGACAATTCATGAATGAAAACAAGGAGCATTGA   5596
m150504_iid=2876274_0      TTTCTTCATCTCAAT-TTTAACGTTAGGGACAATTCATGAATGAAAACAAGGAGCATTGA   5599
m141030_iid=80036_0        TTTCTTCATCTCAAT-TTTAACGTTAGGGACAATTCATGAATGAAAACAAGGAGCATTGA   5360
m150506_iid=2596494_0      TTTCTTCATCTCAAT-TTTAACGTTAGGGACAATTCATGAATGAAAACAAGGAGCATTGA   5765
m150503_iid=2526862_0      TTTCTTCATCTCAAT-TTTAACGTTAGGGACAATTCATGAATGAAAACAAGGAGCATTGA   5449
m150506_iid=2567000_0      TTTCTTCATCTCAAT-TTTAACGTTAGGGACAATTCATGAATGAAAACAAGGAGCATTGA   3398
m150507_iid=3073271_0      TTTCTTCATCTCAAT-TTTAACGTTAGGGACAATTCATGAATGAAAACAAGGAGCATTGA   4702
m150504_iid=3156087_0      TTTCTTCATCTCAAT-TTTAACGTTAGGGACAATTCATGAATGAAAACAAGGAGCATTGA   4525
m150505_iid=2498502_0      TTTCTTCATCTCAAT-TTTAACGTTAGGGACAATTCATGAATGAAAACAAGGAGCATTGA   2175
m141102_iid=754227_0       TTTCTTCATCTCAAT-TTTAACGTTAGGGACAATTCATGAATGAAAACAAGGAGCATTGA   4737
m150507_iid=3469514_0      TTTCTTCATCTCAAT-TTTAACGTTAGGGACAATTCATGAATGAAAACAAGGAGCATTGA   5295
```

```
Rdo_MitoGenome            TTTCTTCATCTCAAT-TTTAACGTTAGGGACAATTCATGAATGAAAACAAGGAGCATTGA   5770
m141101_iid=614933_0      TTTCTTCATCTCAAT-TTTAACGTTAGGGACAATTCATGAATGAAAACAAGGAGCATTGA   4322
```

nad3

```
m141031_iid=347546_0      ATTGAAA-AAACTAGGATAATAGTTTAAAAAACATTTAATTTGCATTTAAAAGAAGTTAA   553
m150506_iid=3127414_0     ATTGAA--AAACTAGGATAATAGTTTAAAAAACATTTAATTTGCATTTAAAAGAAGTTAA   2297
m141102_iid=1245517_0     ATTGAAA-AAACTAGGATAATAGTTTAAAAAACATTTAATTTGCATTTAAAAGAAGTTAA   3952
m141107_iid=1405727_0     ATTGAAA-AAACTAGGATAATAGTTTAAAAAACATTTAATTTGCATTTAAAAGAAGTTAA   1133
m141102_iid=987614_0      ATTGAAA-AAACTAGGATAATAGTTTAAAAAACATTTAATTTGCATTTAAAAGAAGTTAA   4103
m141108_iid=1701429_0     ATTGAAAAAAACTAGGATAATAGTTTAAAAAACATTTAATTTGCATTTAAAAGAAGTTAA   839
m141102_iid=987286_0      ATTGAAA-AAACTAGGATAATAGTTTAAAAAACATTTAATTTGCATTTAAAAGAAGTTAA   2055
m150430_iid=3272125_0     ATTGAAA-AAACTAGGATAATAGTTTAAAAAACATTTAATTTGCATTTAAAAGAAGTTAA   3578
m141102_iid=754225_0      ATTGAAA-AAACTAGGATAATAGTTTAAAAAACATTTAATTTGCATTTAAAAGAAGTTAA   4742
m141107_iid=1409934_0     ATTGAAA-AAACTAGGATAATAGTTTAAAAAACATTTAATTTGCATTTAAAAGAAGTTAA   5288
m150503_iid=3416920_0     ATTGAAA-AAACTAGGATAATAGTTTAAAAAACATTTAATTTGCATTTAAAAGAAGTTAA   4007
m141108_iid=1800121_0     ATTGAAA-AAACTAGGATAATAGTTTAAAAAACATTTAATTTGCATTTAAAAGAAGTTAA   3818
m141031_iid=147540_0      ATTGAAA-AAACTAGGATAATAGTTTAAAAAACATTTAATTTGCATTTAAAAGAAGTTAA   4161
m150507_iid=3674924_0     ATTGAAA-AAACTAGGATAATAGTTTAAAA-AAATTTAATTTGCATTTAAAAGAAGTTAA   4313
m150503_iid=2923751_0     ATTGAAA-AAACTAGGATAATAGTTTAAAAAACATTTAATTTGCATTTAAAAGAAGTTAA   4155
m141031_iid=115118_0      ATTGAAA-AAACTAGGATAATAGTTTAAAAAACATTTAATTTGCATTTAAAAGAAGTTAA   3005
m150430_iid=2722448_0     ATTGAAA-AAACTAGGATAATAGTTTAAAAAACATTTAATTTGCATTTAAAAGAAGTTAA   2942
m150507_iid=3102647_0     ATTGAAA-AAACTAGGATAATAGTTTAAAAAACATTTAATTTGCATTTAAAAGAAGTTAA   1251
m141108_iid=1811866_0     ATTGAAA-AAACTAGGATAATAGTTTAAAAAACATTTAATTTGCATTTAAAAGAAGTTAA   3123
m150501_iid=2948495_0     ATTGAAA-AAACTAGGATAATAGTTTAAAAAACATTTAATTTGCATTTAAAAGAAGTTAA   2549
m150504_iid=3238195_0     ATTGAAAAAAACTAGGATAATAGTTTAAAAAACATTTAATTTGCATTTAAAAGAAGTTAA   4881
m150503_iid=3388511_0     ATTGAAA-AAACTAGGATAATAGTTTAAAAAACATTTAATTTGCATTTAAAAGAAGTTAA   2576
m150505_iid=3777611_0     ATTGAAA-AAACTAGGATAATAGTTTAAAAAACATTTAATTTGCATTTAAAAGAAGTTAA   3997
m150430_iid=2438833_0     ATTGAAA-AAACTAGGATAATAGTTTAAAAAACATTTAATTTGCATTTAAAAGAAGTTAA   4336
m141108_iid=1698969_0     ATTGAAA-AAACTAGGATAATAGTTTAAAAAACATTTAATTTGCATTTAAAAGAAGTTAA   5462
m150430_iid=3059190_0     ATTGAAA-AAACTAGGATAATAGTTTAAAAAACATTTAATTTGCATTTAAAAGAAGTTAA   1185
m141107_iid=1383067_0     ATTGAAA-AAACTAGGATAATAGTTTAAAAAACATTTAATTTGCATTTAAAAGAAGTTAA   1194
m141101_iid=597008_0      ------------------------------------------------------------   0
m150506_iid=2596496_0     ATTGAAA-AAACTAGGATAATAGTTTAAAAAACATTTAATTTGCATTTAAAAGAAGTTAA   5823
m141102_iid=910051_0      ATTGAAA-AAACTAGGATAATAGTTTAAAAAACATTTAATTTGCATTTAAAAGAAGTTAA   5310
m150505_iid=3784017_0     ATTGAAA-AAACTAGGATAATAGTTTAAAAAACATTTAATTTGCATTTAAAAGAAGTTAA   4109
m141101_iid=682151_0      ATTGAAA-AAACTAGGATAATAGTTTAAAAAACATTTAATTTGCATTTAAAAGAAGTTAA   3180
m150501_iid=3748029_0     ATTGAAA-AAACTAGGATAATAGTTTAAAAAACATTTAATTTGCATTTAAAAGAAGTTAA   3722
m150504_iid=2876272_0     ATTGAAA-AAACTAGGATAATAGTTTAAAAAACATTTAATTTGCATTTAAAAGAAGTTAA   5586
NC_042820.1               ATTGAAA-AAACTAGGATAATAGTTTAAAAAACATTTAATTTGCATTTAAAAGAAGTTAA   5829
m141102_iid=1228825_0     ATTGAAA-AAACTAGGATAATAGTTTAAAAAACATTTAATTTGCATTTAAAAGAAGTTAA   5655
m150504_iid=2876274_0     ATTGAAA-AAACTAGGATAATAGTTTAAAAAACATTTAATTTGCATTTAAAAGAAGTTAA   5658
m141030_iid=80036_0       ATTGAAA-AAACTAGGATAATAGTTTAAAAAACATTTAATTTGCATTTAAAAGAAGTTAA   5419
m150506_iid=2596494_0     ATTGAAA-AAACTAGGATAATAGTTTAAAAAACATTTAATTTGCATTTAAAAGAAGTTAA   5824
m150503_iid=2526862_0     ATTGAAA-AAACTAGGATAATAGTTTAAAAAACATTTAATTTGCATTTAAAAGAAGTTAA   5508
m150506_iid=2567000_0     ATTGAAA-AAACTAGGATAATAGTTTAAAAAACATTTAATTTGCATTTAAAAGAAGTTAA   3457
m150507_iid=3073271_0     ATTGAAA-AAACTAGGATAATAGTTTAAAAAACATTTAATTTGCATTTAAAAGAAGTTAA   4761
m150504_iid=3156087_0     ATTGAAA-AAACTAGGATAATAGTTTAAAAAACATTTAATTTGCATTTAAAAGAAGTTAA   4584
m150505_iid=2498502_0     ATTGAAA-AAACTAGGATAATAGTTTAAAAAACATTTAATTTGCATTTAAAAGAAGTTAA   2234
m141102_iid=754227_0      ATTGAAA-AAACTAGGATAATAGTTTAAAAAACATTTAATTTGCATTTAAAAGAAGTTAA   4796
m150507_iid=3469514_0     ATTGAAA-AAACTAGGATAATAGTTTAAAAAACATTTAATTTGCATTTAAAAGAAGTTAA   5354
Rdo_MitoGenome            ATTGAAA-AAACTAGGATAATAGTTTAAAAAACATTTAATTTGCATTTAAAAGAAGTTAA   5829
m141101_iid=614933_0      ATTGAAA-AAACTAGGATAATAGTTTAAAAAACATTTAATTTGCATTTAAAAGAAGTTAA   4381
```

nad3

```
m141031_iid=347546_0      AATTAACTTATCTTAAATAAGAAACAATAAAATTTATTTAGTTTCGACCTAAAATTTTGA   613
m150506_iid=3127414_0     AATTAACTT--CTTAAATAAGAAACAATAA-TTGTATTTAGTTTCGACCTAAA-TTTTGA   2353
m141102_iid=1245517_0     AATTAACTTATCTTAAATAAGAAACAATAAATTGTATTTAGTTTCGACCTAAAATTTTGA   4012
m141107_iid=1405727_0     -ATTAACTTATCTTAAATAAGAAACAT-AAATTGTATTTAGTTTCGACCTAAAATTTTGA   1191
m141102_iid=987614_0      AATTAACTTATCTTAAATAAGAAACAATAAATTGTATTTAGTTTCGACCTAAAATTTTGA   4163
m141108_iid=1701429_0     AATTAACTTATCTTAAATAAGAAACAATAAATTGTATTTAGTTTCGACCTAAAATTTTGA   899
m141102_iid=987286_0      AATTAACTTATCTTAAATAAGAAACAATAAATTGTATTTAGTTTCGACCTAAAATTTTGA   2115
m150430_iid=3272125_0     AATTAACTTATCTTAAATAAGAAACAATAAATTGTATTTAGTTTCGACCTAAAATTTTGA   3638
m141102_iid=754225_0      AATTAACTTATCTTAAATAAGAAACAATAAATTGTATTTAGTTTCGACCTAAAATTTTGA   4802
m141107_iid=1409934_0     AATTAACTTATCTTAAATAAGAAA-AATAAATTGTATTTAGTTTCGACCTAAAATTTTGA   5347
m150503_iid=3416920_0     AATTAACTTATCTTAAATAAGAAACAATAAATTGTATTTAGTTTCGACCTAAAATTTTGA   4067
m141108_iid=1800121_0     AATTAACTTATCTTAAATAAGAAACAATAAATTGTATTTAGTTTCGACCTAAAATTTTGA   3878
m141031_iid=147540_0      AATTAACTTATCTTAAATAAGAAACAATAAATTGTATTTAGTTTCGACCTAAAATTTTGA   4221
m150507_iid=3674924_0     AATTAACTTATCTTAAATAAGAAACAATAAATTGTATTTAGTTTCGACCTAAAATTTTGA   4373
m150503_iid=2923751_0     AATTAACTTATCTTAAATAAGAAACAATAAATTGTATTTAGTTTCGACCTAAAATTTTGA   4215
m141031_iid=115118_0      AATTAACTTATCTTAAATAAGAAACAATAAATTGTATTTAGTTTCGACCTAAAATTTTGA   3065
m150430_iid=2722448_0     AATTAACTTATCTTAAATAAGAAACAATAAATTGTATTTAGTTTCGACCTAAAATTTTGA   3002
```

```
m150507_iid=3102647_0      AATTAACTTATCTTAAATAAGAAACAATAAATTGTATTTAGTTTCGACCTAAAATTTTGA   1311
m141108_iid=1811866_0      AATTAACTTATCTTAAATAAGAAACAATAAATTGTATTTAGTTTCGACCTAAAATTTTGA   3183
m150501_iid=2948495_0      AATTAACTTATCTTAAATAAGAAACAATAAATTGTATTTAGTTTCGACCTAAAATTTTGA   2609
m150504_iid=3238195_0      AATTAACTTATCTTAAATAAGAAACAATAAATTGTATTTAGTTTCGACCTAAAATTTTGA   4941
m150503_iid=3388511_0      AATTAACTTATCTTAAATAAGAAACAATAAATTGTATTTAGTTTCGACCTAAAATTTTGA   2636
m150505_iid=3777611_0      AATTAACTTATCTTAAATAAGAAACAATAAATTGTATTTAGTTTCGACCTAAAATTTTGA   4057
m150430_iid=2438833_0      AATTAACTTATCTTAAATAAGAAACAATAAATTGTATTTAGTTTCGACCTAAAATTTTGA   4396
m141108_iid=1698969_0      AATTAACTTATCTTAAATAAGAAACAATAAATTGTATTTAGTTTCGACCTAAAATTTTGA   5522
m150430_iid=3059190_0      AATTAACTTATCTTAAATAAGAAACAATAAATTGTATTTAGTTTCGACCTAAAATTTTGA   1245
m141107_iid=1383067_0      AATTAACTTATCTTAAATAAGAAACAATAAATTGTATTTAGTTTCGACCTAAAATTTTGA   1254
m141101_iid=597008_0       ------------------------------------------------------------   0
m150506_iid=2596496_0      AATTAACTTATCTTAAATAAGAAACAATAAATTGTATTTAGTTTCGACCTAAAATTTTGA   5883
m141102_iid=910051_0       AATTAACTTATCTTAAATAAGAAACAATAAATTGTATTTAGTTTCGACCTAAAATTTTGA   5370
m150505_iid=3784017_0      AATTAACTTATCTTAAATAAGAAACAATAAATTGTATTTAGTTTCGACCTAAAATTTTGA   4169
m141101_iid=682151_0       AATTAACTTATCTTAAATAAGAAACAATAAATTGTATTTAGTTTCGACCTAAAATTTTGA   3240
m150501_iid=3748029_0      AATTAACTTATCTTAAATAAGAAACAATAAATTGTATTTAGTTTCGACCTAAAATTTTGA   3782
m150504_iid=2876272_0      AATTAACTTATCTTAAATAAGAAACAATAAATTGTATTTAGTTTCGACCTAAAATTTTGA   5646
NC_042820.1                AATTAACTTATCTTAAATAAGAAACAATAAATTGTATTTAGTTTCGACCTAAAATTTTGA   5889
m141102_iid=1228825_0      AATTAACTTATCTTAAATAAGAAACAATAAATTGTATTTAGTTTCGACCTAAAATTTTGA   5715
m150504_iid=2876274_0      AATTAACTTATCTTAAATAAGAAACAATAAATTGTATTTAGTTTCGACCTAAAATTTTGA   5718
m141030_iid=80036_0        AATTAACTTATCTTAAATAAGAAACAATAAATTGTATTTAGTTTCGACCTAAAATTTTGA   5479
m150506_iid=2596494_0      AATTAACTTATCTTAAATAAGAAACAATAAATTGTATTTAGTTTCGACCTAAAATTTTGA   5884
m150503_iid=2526862_0      AATTAACTTATCTTAAATAAGAAACAATAAATTGTATTTAGTTTCGACCTAAAATTTTGA   5568
m150506_iid=2567000_0      AATTAACTTATCTTAAATAAGAAACAATAAATTGTATTTAGTTTCGACCTAAAATTTTGA   3517
m150507_iid=3073271_0      AATTAACTTATCTTAAATAAGAAACAATAAATTGTATTTAGTTTCGACCTAAAATTTTGA   4821
m150504_iid=3156087_0      AATTAACTTATCTTAAATAAGAAACAATAAATTGTATTTAGTTTCGACCTAAAATTTTGA   4644
m150505_iid=2498502_0      AATTAACTTATCTTAAATAAGAAACAATAAATTGTATTTAGTTTCGACCTAAAATTTTGA   2294
m141102_iid=754227_0       AATTAACTTATCTTAAATAAGAAACAATAAATTGTATTTAGTTTCGACCTAAAATTTTGA   4856
m150507_iid=3469514_0      AATTAACTTATCTTAAATAAGAAACAATAAATTGTATTTAGTTTCGACCTAAAATTTTGA   5414
Rdo_MitoGenome             AATTAACTTATCTTAAATAAGAAACAATAAATTGTATTTAGTTTCGACCTAAAATTTTGA   5889
m141101_iid=614933_0       AATTAACTTATCTTAAATAAGAAACAATAAATTGTATTTAGTTTCGACCTAAAATTTTGA   4441


m141031_iid=347546_0       TGATAAACATCCTTATTTTTAATTGAAACCAA-AATAGAGGTGTACACTCGTTAATGAAT   672
m150506_iid=3127414_0      TGATAAACATCCTTATTTTTAATTGAAACCAA-AATAGAGGTGTACCACTGTTAATGGT-   2411
m141102_iid=1245517_0      TGATAAACATCCTTATTTTTAATTGAAACCAA-AATAGAGGTGTACCACTGTTAATGGT-   4070
m141107_iid=1405727_0      TGATAAACATCCTTATTTTTAATTGAAACCAAAAATAGAGGTGTACCACTGTTAATGGT-   1250
m141102_iid=987614_0       TGATAAACATCCTTATTTTTAATTGAAACCAA-AATAGAGGTGTACCACTGTTAATGGT-   4221
m141108_iid=1701429_0      TGATAAACATCCTTATTTTTAATTGAAACCAA-AATAGAGGTGTACCACTGTTAATGGT-   957
m141102_iid=987286_0       TGATAAACATCCTTATTTTTAATTGAAACAAAACAAAGAGGTGTACCACTGTTAATGGT-   2174
m150430_iid=3272125_0      TGATAAACATCCTTATTTTTAATTGAAACCAA-AATAGAGGTGTACCACTGTTAATGGT-   3696
m141102_iid=754225_0       TGATAAACATCCTTATTTTTAATTGAAACCAA-AATAGAGGTGTACCACTGTTAATGGT-   4860
m141107_iid=1409934_0      TGATAAACATCCTTATTTTTAATTGAAACCAA-AATAGAGGTGTACCACTGTTAATGGT-   5405
m150503_iid=3416920_0      TGATAAACATCCTTATTTTTAATTGAAACCAA-AATAGAGGTGTACCACTGTTAATGGT-   4125
m141108_iid=1800121_0      TGATAAACATCCTTATTTTTAATTGAAACCAA-AATAGAGGTGTACCACTGTTAATGGT-   3936
m141031_iid=147540_0       TGATAAACATCCTTATTTTTAATTGAAACCAA-AATAGAGGTGTACCACTGTTAATGGT-   4279
m150507_iid=3674924_0      TGATAAACATCCTTATTTTTAATTGAAACCAA-AATAGAGGTGTACCACTGTTAATGGT-   4431
m150503_iid=2923751_0      TGATAAACATCCTTATTTTTAATTGAAACCAA-AATAGAGGTGTACCACTGTTAATGGT-   4273
m141031_iid=115118_0       TGATAAACATCCTTATTTTTAATTGAAACCAA-AATAGAGGTGTACCACTGTTAATGGT-   3123
m150430_iid=2722448_0      TGATAAACATCCTTATTTTTAATTGAAACCAA-AATAGAGGTGTACCACTGTTAATGGT-   3060
m150507_iid=3102647_0      TGATAAACATCCTTATTTTTAATTGAAACCAA-AATAGAGGTGTACCACTGTTAATGGT-   1369
m141108_iid=1811866_0      TGATAAACATCCTTATTTTTAATTGAAACCAA-AATAGAGGTGTACCACTGTTAATGGT-   3241
m150501_iid=2948495_0      TGATAAACATCCTTATTTTTAATTGAAACCAA-AATAGAGGTGTACCACTGTTAATGGT-   2667
m150504_iid=3238195_0      TGATAAACATCCTTATTTTTAATTGAAACCAA-AATAGAGGTGTACCACTGTTAATGGT-   4999
m150503_iid=3388511_0      TGATAAACATCCTTATTTTTAATTGAAACCAA-AATAGAGGTGTACCACTGTTAATGGT-   2694
m150505_iid=3777611_0      TGATAAACATCCTTATTTTTAATTGAAACCAA-AATAGAGGTGTACCACTGTTAATGGT-   4115
m150430_iid=2438833_0      TGATAAACATCCTTATTTTTAATTGAAACCAA-AATAGAGGTGTACCACTGTTAATGGT-   4454
m141108_iid=1698969_0      TGATAAACATCCTTATTTTTAATTGAAACCAA-AATAGAGGTGTACCACTGTTAATGGT-   5580
m150430_iid=3059190_0      TGATAAACATCCTTATTTTTAATTGAAACCAA-AATAGAGGTGTACCACTGTTAATGGT-   1303
m141107_iid=1383067_0      TGATAAACATCCTTATTTTTAATTGAAACCAA-AATAGAGGTGTACCACTGTTAATGGT-   1312
m141101_iid=597008_0       ------------------------------------------------------------   0
m150506_iid=2596496_0      TGATAAACATCCTTATTTTTAATTGAAACCAA-AATAGAGGTGTACCACTGTTAATGGT-   5941
m141102_iid=910051_0       TGATAAACATCCTTATTTTTAATTGAAACCAA-AATAGAGGTGTACCACTGTTAATGGT-   5428
m150505_iid=3784017_0      TGATAAACATCCTTATTTTTAATTGAAACCAA-AATAGAGGTGTACCACTGTTAATGGT-   4227
m141101_iid=682151_0       TGATAAACATCCTTATTTTTAATTGAAACCAA-AATAGAGGTGTACCACTGTTAATGGT-   3298
m150501_iid=3748029_0      TGATAAACATCCTTATTTTTAATTGAAACCAA-AATAGAGGTGTACCACTGTTAATGGT-   3840
m150504_iid=2876272_0      TGATAAACATCCTTATTTTTAATTGAAACCAA-AATAGAGGTGTACCACTGTTAATGGT-   5704
NC_042820.1                TGATAAACATCCTTATTTTTAATTGAAACCAA-AATAGAGGTGTACCACTGTTAATGGT-   5947
m141102_iid=1228825_0      TGATAAACATCCTTATTTTTAATTGAAACCAA-AATAGAGGTGTACCACTGTTAATGGT-   5773
m150504_iid=2876274_0      TGATAAACATCCTTATTTTTAATTGAAACCAA-AATAGAGGTGTACCACTGTTAATGGT-   5776
m141030_iid=80036_0        TGATAAACATCCTTATTTTTAATTGAAACCAA-AATAGAGGTGTACCACTGTTAATGGT-   5537
```

```
m150506_iid=2596494_0      TGATAAACATCCTTATTTTTAATTGAAACCAA-AATAGAGGTGTACCACTGTTAATGGT-   5942
m150503_iid=2526862_0      TGATAAACATCCTTATTTTTAATTGAAACCAA-AATAGAGGTGTACCACTGTTAATGGT-   5626
m150506_iid=2567000_0      TGATAAACATCCTTATTTTTAATTGAAACCAA-AATAGAGGTGTACCACTGTTAATGGT-   3575
m150507_iid=3073271_0      TGATAAACATCCTTATTTTTAATTGAAACCAA-AATAGAGGTGTACCACTGTTAATGGT-   4879
m150504_iid=3156087_0      TGATAAACATCCTTATTTTTAATTGAAACCAA-AATAGAGGTGTACCACTGTTAATGGT-   4702
m150505_iid=2498502_0      TGATAAACATCCTTATTTTTAATTGAAACCAA-AATAGAGGTGTACCACTGTTAATGGT-   2352
m141102_iid=754227_0       TGATAAACATCCTTATTTTTAATTGAAACCAA-AATAGAGGTGTACCACTGTTAATGGT-   4914
m150507_iid=3469514_0      TGATAAACATCCTTATTTTTAATTGAAACCAA-AATAGAGGTGTACCACTGTTAATGGT-   5472
Rdo_MitoGenome             TGATAAACATCCTTATTTTTAATTGAAACCAA-AATAGAGGTGTACCACTGTTAATGGT-   5947
m141101_iid=614933_0       TGATAAACATCCTTATTTTTAATTGAAACCAA-AATAGAGGTGTACCACTGTTAATGGT-   4499


m141031_iid=347546_0       AGGAGTAAAATTTGTTTAAATCCAATTAAGA-ATATGATAATCAAG--AAAGCTT----C   725
m150506_iid=3127414_0      ------AAAATTGATTTAAATCCAATTAAGAAATATGATAATCAAGAAGAAGCTT----C   2461
m141102_iid=1245517_0      ------AAAATTGATTTAAATCCAATTAAGAAATATGATAATCAAGAAGAAGCTT----C   4120
m141107_iid=1405727_0      ------AAAATTGATTTAAATCCAATTAAGAAATATGATAATCAAGAAGAAGCTT----C   1300
m141102_iid=987614_0       ------AAAATTGATTTAAATCCAATTAAGAAATATGATAATCAAGAAGAAGCTT----C   4271
m141108_iid=1701429_0      ------AAAATTGATTTAAATCCAATTAAGAAATATGATAATCAAGAAGAAGCTT----C   1007
m141102_iid=987286_0       ------AA-ATTGATTTAAATCCAATTAAGAAATATGATAATCAAGAAGAAGCTT----C   2223
m150430_iid=3272125_0      ------AAAATTGATTTAAATCCAATTAAGAAATATGATAATCAAGAAGAAGCTT----C   3746
m141102_iid=754225_0       ------AAAATTGATTTAAATCCAATTAAGAAATATGATAATCAAGAAGAAGCTT----C   4910
m141107_iid=1409934_0      ------AAAATTGATTTAAATCCAATTAAGAAATATGATAATCAAGAAGAAGCTT----C   5455
m150503_iid=3416920_0      ------AAAATTGATTTAAATCCAATTAAGAAATATGATAATCAAGAAGAAGCTT----C   4175
m141108_iid=1800121_0      ------AAAATTGATTTAAATCCAATTAAGAAATATGATAATCAAGAAGAAGCTT----C   3986
m141031_iid=147540_0       ------AAAATTGATTTAAATCCAATTAAGAAATATGATAATCAAGAAGAAGCTT----C   4329
m150507_iid=3674924_0      ------AAAATTGATTTAAATCCAATTAAGAAATATGATAATCAAGAAGAAGCTTCTA--   4483
m150503_iid=2923751_0      ------AAAATTGATTTAAATCCAATTAAGAAATATGATAATCAAGAAGAAGCTTCTA--   4325
m141031_iid=115118_0       ------AAAATTGATTTAAATCCAATTAAGAAATATGATAATCAAGAAGAAGCTTCTA--   3175
m150430_iid=2722448_0      ------AAAATTGATTTAAATCCAATTAAGAAATATGATAATCAAGAAGAAGCTTCTA--   3112
m150507_iid=3102647_0      ------AAAATTGATTTAAATCCAATTAAGAAATATGATAATCAAGAAGAAGCTTCTA--   1421
m141108_iid=1811866_0      ------AAAATTGATTTAAATCCAATTAAGAAATATGATAATCAAGAAGAAGCTTCTA--   3293
m150501_iid=2948495_0      ------AAAATTGATTTAAATCCAATTAAGAAATATGATAATCAAGAAGAAGCTTCTA--   2719
m150504_iid=3238195_0      ------AAAATTGATTTAAATCCAATTAAGAAATATGATAATCAAGAAGAAGCTTCTA--   5051
m150503_iid=3388511_0      ------AAAATTGATTTAAATCCAATTAAGAAATATGATAATCAAGAAGAAGCTTCTAAC   2748
m150505_iid=3777611_0      ------AAAATTGATTTAAATCCAATTAAGAAATATGATAATCAAGAAGAAGCTT----C   4165
m150430_iid=2438833_0      ------AAAATTGATTTAAATCCAATTAAGAAATATGATAATCAAGAAGAAGCTT----C   4504
m141108_iid=1698969_0      ------AAAATTGATTTAAATCCAATTAAGAAATATGATAATCAAGAAGAAGCTT----C   5630
m150430_iid=3059190_0      ------AAAATTGATTTAAATCCAATTAAGAAATATGATAATCAAGAAGAAGCTT----C   1353
m141107_iid=1383067_0      ------AAAATTGATTTAAATCCAATTAAGAAATATGATAATCAAGAAGAAGCTT----C   1362
m141101_iid=597008_0       ------------------------------------------------------------   0
m150506_iid=2596496_0      ------AAAATTGATTTAAATCCAATTAAGAAATATGATAATCAAGAAGAAGCTT----C   5991
m141102_iid=910051_0       ------AAAATTGATTTAAATCCAATTAAGAAATATGATAATCAAGAAGAAGCTT----C   5478
m150505_iid=3784017_0      ------AAAATTGATTTAAATCCAATTAAGAAATATGATAATCAAGAAGAAGCTT----C   4277
m141101_iid=682151_0       ------AAAATTGATTTAAATCCAATTAAGAAATATGATAATCAAGAAGAAGCTT----C   3348
m150501_iid=3748029_0      ------AAAATTGATTTAAATCCAATTAAGAAATATGATAATCAAGAAGAAGCTT----C   3890
m150504_iid=2876272_0      ------AAAATTGATTTAAATCCAATTAAGAAATATGATAATCAAGAAGAAGCTT----C   5754
NC_042820.1                ------AAAATTGATTTAAATCCAATTAAGAAATATGATAATCAAGAAGAAGCTT----C   5997
m141102_iid=1228825_0      ------AAAATTGATTTAAATCCAATTAAGAAATATGATAATCAAGAAGAAGCTT----C   5823
m150504_iid=2876274_0      ------AAAATTGATTTAAATCCAATTAAGAAATATGATAATCAAGAAGAAGCTT----C   5826
m141030_iid=80036_0        ------AAAATTGATTTAAATCCAATTAAGAAATATGATAATCAAGAAGAAGCTT----C   5587
m150506_iid=2596494_0      ------AAAATTGATTTAAATCCAATTAAGAAATATGATAATCAAGAAGAAGCTT----C   5992
m150503_iid=2526862_0      ------AAAATTGATTTAAATCCAATTAAGAAATATGATAATCAAGAAGAAGCTT----C   5676
m150506_iid=2567000_0      ------AAAATTGATTTAAATCCAATTAAGAAATATGATAATCAAGAAGAAGCTT----C   3625
m150507_iid=3073271_0      ------AAAATTGATTTAAATCCAATTAAGAAATATGATAATCAAGAAGAAGCTT----C   4929
m150504_iid=3156087_0      ------AAAATTGATTTAAATCCAATTAAGAAATATGATAATCAAGAAGAAGCTT----C   4752
m150505_iid=2498502_0      ------AAAATTGATTTAAATCCAATTAAGAAATATGATAATCAAGAAGAAGCTT----C   2402
m141102_iid=754227_0       ------AAAATTGATTTAAATCCAATTAAGAAATATGATAATCAAGAAGAAGCTT----C   4964
m150507_iid=3469514_0      ------AAAATTGATTTAAATCCAATTAAGAAATATGATAATCAAGAAGAAGCTT----C   5522
Rdo_MitoGenome             ------AAAATTGATTTAAATCCAATTAAGAAATATGATAATCAAGAAGAAGCTT----C   5997
m141101_iid=614933_0       ------AAAATTGATTTAAATCCAATTAAGAAATATGATAATCAAGAAGAAGCTT----C   4549


m141031_iid=347546_0       TAACTTCAATCTTTAGCGGTGTTGAAATCCGTTAATATTTATTTATATATAGTTTAAGAA   785
m150506_iid=3127414_0      TAACTTCAATCTTTAGCGGTGAA--AATCCGTTAATATTTCTATTTATATAGTTTAAGAA   2519
m141102_iid=1245517_0      TAACTTCAATCTTTAGCGGTGAA--AATCCGTTAATATTTCTATTTATATAGTTTAA-GA   4177
m141107_iid=1405727_0      TAACTTCAATCTTTAGCGGTGAA--AATCCGTTAATATTTCTATTTAATATAGTTTAAGA   1358
m141102_iid=987614_0       TAACTTCAATCTTTAGCGGTGAA--AATCCGTTAATATTTCTATTTATATAGTTTAAGAA   4329
m141108_iid=1701429_0      TAACTTCAATCTTTAGCGGTGAA--AATCCGTTAATATTTCTATTTATATAGTTTAAGAA   1065
m141102_iid=987286_0       TAACTTCAATCTTTAGCGGTGAA--AATCCGTTAATATTTCTATTTATATAGTTTAAGAA   2281
m150430_iid=3272125_0      TAACTTCAATCTTTAGCGGTGAA--AATCCGTTAATATTTCTATTTATATAGTTTAAGAA   3804
m141102_iid=754225_0       TAACTTCAATCTTTAGCGGTGAA--AATCCGTTAATATTTCTATTTATATAGTTTAAGAA   4968
```

```
m141107_iid=1409934_0      TAACTTCAATCTTTAGCGGTGAA--AATCCGTTAATATTTCTATTTATATAGTTTAAGAA   5513
m150503_iid=3416920_0      TAACTTCAATCTTTAGCGGTGAA--AATCCGTTAATATTTCTATTTATATAGTTTAAGAA   4233
m141108_iid=1800121_0      TAACTTCAATCTTTAGCGGTGAA--AATCCGTTAATATTTCTATTTATATAGTTTAAGAA   4044
m141031_iid=147540_0       TAACTTCAATCTTTAGCGGTGAA--AATCCGTTAATATTTCTATTTATATAGTTTAAGAA   4387
m150507_iid=3674924_0      --ACTTCAATCTTTAGCGGTGAA--AATCCGTTAATATTTCTATTTATATAGTTTAAGAA   4539
m150503_iid=2923751_0      --ACTTCAATCTTTAGCGGTGAA--AATCCGTTAATATTTCTATTTATATAGTTTAAGAA   4381
m141031_iid=115118_0       --ACTTCAATCTTTAGCGGTGAA--AATCCGTTAATATTTCTATTTATATAGTTTAAGAA   3231
m150430_iid=2722448_0      --ACTTCAATCTTTAGCGGTGAA--AATCCGTTAATATTTCTATTTATATAGTTTAAGAA   3168
m150507_iid=3102647_0      --ACTTCAATCTTTAGCGGTGAA--AATCCGTTAATATTTCTATTTATATAGTTTAAGAA   1477
m141108_iid=1811866_0      --ACTTCAATCTTTAGCGGTGAA--AATCCGTTAATATTTCTATTTATATAGTTTAAGAA   3349
m150501_iid=2948495_0      --ACTTCAATCTTTAGCGGTGAA--AATCCGTTAATATTTCTATTTATATAGTTTAAGAA   2775
m150504_iid=3238195_0      --ACTTCAATCTTTAGCGGTGAA--AATCCGTTAATATTTCTATTTATATAGTTTAAGAA   5107
m150503_iid=3388511_0      TAACTTCAATCTTTAGCGGTGAA--AATCCGTTAATATTTCTATTTATATAGTTTAAGAA   2806
m150505_iid=3777611_0      TAACTTCAATCTTTAGCGGTGAA--AATCCGTTAATATTTCTATTTATATAGTTTAAGAA   4223
m150430_iid=2438833_0      TAACTTCAATCTTTAGCGGTGAA--AATCCGTTAATATTTCTATTTATATAGTTTAAGAA   4562
m141108_iid=1698969_0      TAACTTCAATCTTTAGCGGTGAA--AATCCGTTAATATTTCTATTTATATAGTTTAAGAA   5688
m150430_iid=3059190_0      TAACTTCAATCTTTAGCGGTGAA--AATCCGTTAATATTTCTATTTATATAGTTTAAGAA   1411
m141107_iid=1383067_0      TAACTTCAATCTTTAGCGGTGAA--AATCCGTTAATATTTCTATTTATATAGTTTAAGAA   1420
m141101_iid=597008_0       ------------------------------------------------------------   0
m150506_iid=2596496_0      TAACTTCAATCTTTAGCGGTGAA--AATCCGTTAATATTTCTATTTATATAGTTTAAGAA   6049
m141102_iid=910051_0       TAACTTCAATCTTTAGCGGTGAA--AATCCGTTAATATTTCTATTTATATAGTTTAAGAA   5536
m150505_iid=3784017_0      TAACTTCAATCTTTAGCGGTGAA--AATCCGTTAATATTTCTATTTATATAGTTTAAGAA   4335
m141101_iid=682151_0       TAACTTCAATCTTTAGCGGTGAA--AATCCGTTAATATTTCTATT--TATAGTTTAAGAA   3404
m150501_iid=3748029_0      TAACTTCAATCTTTAGCGGTGAA--AATCCGTTAATATTTCTATTTATATAGTTTAAGAA   3948
m150504_iid=2876272_0      TAACTTCAATCTTTAGCGGTGAA--AATCCGTTAATATTTCTATTTATATAGTTTAAGAA   5812
NC_042820.1                TAACTTCAATCTTTAGCGGTGAA--AATCCGTTAATATTTCTATTTATATAGTTTAAGAA   6055
m141102_iid=1228825_0      TAACTTCAATCTTTAGCGGTGAA--AATCCGTTAATATTTCTATTTATATAGTTTAAGAA   5881
m150504_iid=2876274_0      TAACTTCAATCTTTAGCGGTGAA--AATCCGTTAATATTTCTATTTATATAGTTTAAGAA   5884
m141030_iid=80036_0        TAACTTCAATCTTTAGCGGTGAA--AATCCGTTAATATTTCTATTTATATAGTTTAAGAA   5645
m150506_iid=2596494_0      TAACTTCAATCTTTAGCGGTGAA--AATCCGTTAATATTTCTATTTATATAGTTTAAGAA   6050
m150503_iid=2526862_0      TAACTTCAATCTTTAGCGGTGAA--AATCCGTTAATATTTCTATTTATATAGTTTAAGAA   5734
m150506_iid=2567000_0      TAACTTCAATCTTTAGCGGTGAA--AATCCGTTAATATTTCTATTTATATAGTTTAAGAA   3683
m150507_iid=3073271_0      TAACTTCAATCTTTAGCGGTGAA--AATCCGTTAATATTTCTATTTATATAGTTTAAGAA   4987
m150504_iid=3156087_0      TAACTTCAATCTTTAGCGGTGAA--AATCCGTTAATATTTCTATTTATATAGTTTAAGAA   4810
m150505_iid=2498502_0      TAACTTCAATCTTTAGCGGTGAA--AATCCGTTAATATTTCTATTTATATAGTTTAAGAA   2460
m141102_iid=754227_0       TAACTTCAATCTTTAGCGGTGAA--AATCCGTTAATATTTCTATTTATATAGTTTAAGAA   5022
m150507_iid=3469514_0      TAACTTCAATCTTTAGCGGTGAA--AATCCGTTAATATTTCTATTTATATAGTTTAAGAA   5580
Rdo_MitoGenome             TAACTTCAATCTTTAGCGGTGAA--AATCCGTTAATATTTCTATTTATATAGTTTAAGAA   6055
m141101_iid=614933_0       TAACTTCAATCTTTAGCGGTGAA--AATCCGTTAATATTTCTATTTATATAGTTTAAGAA   4607


m141031_iid=347546_0       -A-AAATTACATTTTCAATGTATAAATATTAATATAATTTATTTAAATACTTAAAAACAC   843
m150506_iid=3127414_0      -AAACATTACATTTTCAATGTAAAATTAATATTATA-TATTTATAAATACTTAAAAACAC   2577
m141102_iid=1245517_0      AAAACATTACATTTTCAATGTAAAATTAATATTAA--TATTTATAAATACTTAAAAACAC   4235
m141107_iid=1405727_0      AAAACATTACATTTTCAATGTAAAATTAATATTAA--TATTTATAAATACTTAAAAACAC   1416
m141102_iid=987614_0       -AAACATTACATTTTCAATGTAAAATTAATATTAA--TATTTATAAATACTTAAAAACAC   4386
m141108_iid=1701429_0      AAAACATTACATTTTCAATGTAAAATTAATATTAA--TATTTATAAATACTTAAAAACAC   1123
m141102_iid=987286_0       -AAACATTACATTTTCAATGTAAAATTAATATTAA--TATTTATAAATACTTAAAAACAC   2338
m150430_iid=3272125_0      -AAACATTACATTTTCAATGTAAAATTAATATTAA--TATTTATAAATACTTAAAAACAC   3861
m141102_iid=754225_0       -AAACATTACATTTTCAATGTAAAATTAATATTAA--TATTTATAAATACTTAAAAACAC   5025
m141107_iid=1409934_0      -AAACATTACATTTTCAATGTAAAATTAATATTAA--TATTTATAAATACTTAAAAACAC   5570
m150503_iid=3416920_0      -A--AATTACATTTTCAATGTAAAATTAATATTAA--TATTTATAAATACTTAAAAACAC   4288
m141108_iid=1800121_0      -AAACATTACATTTTCAATGTAAAATTAATATTAA--TATTTATAAATACTTAAAAACAC   4101
m141031_iid=147540_0       -AAACATTACATTTTCAATGTAAAATTAATATTAA--TATTTATAAATACTTAAAAACAC   4444
m150507_iid=3674924_0      -AAACATTACATTTTCAATGTAAAATTAATATTAA--TATTTATAAATACTTAAAAACAC   4596
m150503_iid=2923751_0      -AAACATTACATTTTCAATGTAAAATTAATATTAA--TATTTATAAATACTTAAAAACAC   4438
m141031_iid=115118_0       -AAACATTACATTTTCAATGTAAAATTAATATTAA--TATTTATAAATACTTAAAAACAC   3288
m150430_iid=2722448_0      -AAACATTACATTTTCAATGTAAAATTAATATTAA--TATTTATAAATACTT-AAAACAC   3224
m150507_iid=3102647_0      -AAACATTACATTTTCAATGTAAAATTAATATTAA--TATTTATAAATACTTAAAAACAC   1534
m141108_iid=1811866_0      -AAACATTACATTTTCAATGTAAAATTAATATTAA--TATTTATAAATACTTAAAAACAC   3406
m150501_iid=2948495_0      -AAACATTACATTTTCAATGTAAAATTAATATTAA--TATTTATAAATACTTAAAAACAC   2832
m150504_iid=3238195_0      -AAACATTACATTTTCAATGTAAAATTAATATTAA--TATTTATAAATACTTAAAAACAC   5164
m150503_iid=3388511_0      -AAACATTACATTTTCAATGTAAAATTAATATTAA--TATTTATAAATACTTAAAAACAC   2863
m150505_iid=3777611_0      -AAACATTACATTTTCAATGTAAAATTAATATTAA--TATTTATAAATACTTAAAAACAC   4280
m150430_iid=2438833_0      -AAACATTACATTTTCAATGTAAAATTAATATTAA--TATTTATAAATACTTAAAAACAC   4619
m141108_iid=1698969_0      -AAACATTACATTTTCAATGTAAAATTAATATTAA--TATTTATAAATACTTAAAAACAC   5745
m150430_iid=3059190_0      -AAACATTACATTTTCAATGTAAAATTAATATTAA--TATTTATAAATACTTAAAAACAC   1468
m141107_iid=1383067_0      -A--AATTACATTTTCAATGTAAAATTAATATTAA--TATTTATAAATACTTAAAAACAC   1475
m141101_iid=597008_0       ------------------------------------------------------------   0
m150506_iid=2596496_0      -AAACATTACATTTTCAATGTAAAATTAATATTAA--TATT-TAAAATACTTAAAAACAC   6105
m141102_iid=910051_0       -AAACATTACATTTTCAATGTAAAATTAATATTAA--TATTTA-TAAAACTTAAAAACAC   5592
```

```
m150505_iid=3784017_0      -AAACATTACATTTTCAATGTAAAATTAATATTAA--TATTTATAAATACTTAAAAACAC   4392
m141101_iid=682151_0       -AAACATTACATTTTCAATGTAAAATTAATATTAA--TATTTATAAATACTTAAAAACAC   3461
m150501_iid=3748029_0      -AAACATTACATTTTCAATGTAAAATTAATATTAA--TATTTATAAATACTTAAAAACAC   4005
m150504_iid=2876272_0      -AAACATTACATTTTCAATGTAAAATTAATATTAA--TATTTATAAATACTTAAAAACAC   5869
NC_042820.1                -AAACATTACATTTTCAATGTAAAATTAATATTAA--TATTTATAAATACTTAAAAACAC   6112
m141102_iid=1228825_0      -AAACATTACATTTTCAATGTAAAATTAATATTAA--TATTTATAAATACTTAAAAACAC   5938
m150504_iid=2876274_0      -AAACATTACATTTTCAATGTAAAATTAATATTAA--TATTTATAAATACTTAAAAACAC   5941
m141030_iid=80036_0        -AAACATTACATTTTCAATGTAAAATTAATATTAA--TATTTATAAATACTTAAAAACAC   5702
m150506_iid=2596494_0      -AAACATTACATTTTCAATGTAAAATTAATATTAA--TATTTATAAATACTTAAAAACAC   6107
m150503_iid=2526862_0      -AAACATTACATTTTCAATGTAAAATTAATATTAA--TATTTATAAATACTTAAAAACAC   5791
m150506_iid=2567000_0      -AAACATTACATTTTCAATGTAAAATTAATATTAA--TATTTATAAATACTTAAAAACAC   3740
m150507_iid=3073271_0      -AAACATTACATTTTCAATGTAAAATTAATATTAA--TATTTATAAATACTTAAAAACAC   5044
m150504_iid=3156087_0      -AAACATTACATTTTCAATGTAAAATTAATATTAA--TATTTATAAATACTTAAAAACAC   4867
m150505_iid=2498502_0      -AAACATTACATTTTCAATGTAAAATTAATATTAA--TATTTATAAATACTTAAAAACAC   2517
m141102_iid=754227_0       -AAACATTACATTTTCAATGTAAAATTAATATTAA--TATTTATAAATACTTAAAAACAC   5079
m150507_iid=3469514_0      -AAACATTACATTTTCAATGTAAAATTAATATTAA--TATTTATAAATACTTAAAAACAC   5637
Rdo_MitoGenome             -AAACATTACATTTTCAATGTAAAATTAATATTAA--TATTTATAAATACTTAAAAACAC   6112
m141101_iid=614933_0       -AAACATTACATTTTCAATGTAAAATTAATATTAA--TATTTATAAATACTTAAAAACAC   4664


m141031_iid=347546_0       AAG--TTACCTTAATTATCTTCAATATTAAACTCTT--TTAAGCTATTTAAGTATAAACA   899
m150506_iid=3127414_0      AAGTGTTACCTT-AATATCTTCAATATTAAACTCTTTTTTAAGCTATTTAAGTA-AAACA   2635
m141102_iid=1245517_0      AAGTGTTACCTT-AATATCTTCAATATTAAACTCTT-TTTAAGCTATTTAAGTA-AAACA   4292
m141107_iid=1405727_0      AAGTGTTAC-TT-AATATCTTCAATATTAAACTCTT-TTTAAGCTATTTAAGTA-AAACA   1472
m141102_iid=987614_0       AAGTGTTACCTT-AATATCTTCAATATTAAACTC-TTTTTAAGCTATTTAAGTA-AAACA   4443
m141108_iid=1701429_0      AAGTGTTACCTT-AATATCTTCAATATTAAACTCTTT-TTAAGCTATTTAAGTA-AAACA   1180
m141102_iid=987286_0       AAGTGTTACCTT-AATATCTTCAATATTAAACTCTTT-TTAAGCTATTTAAGTA-AAACA   2395
m150430_iid=3272125_0      AAGTGTTACCTT-AATATCTTCAATATTAAACTCTTT-TTAAGCTATTTAAGTA-AAAA-   3917
m141102_iid=754225_0       AAGTGTTACCTT-AATATCTTCAATATTAAACTCTTT-TTAAGCTATTTAAGTA-AAACA   5082
m141107_iid=1409934_0      AAGTGTTACCTT-AATATCTTCAATATTAAACTCTTT-TTAAGCTATTTAAGTA-AAACA   5627
m150503_iid=3416920_0      AAGTGTTACCTT-AATATCTTCAATATTAAACTCTTT-TTAAGCTATTTAAGTA-AAACA   4345
m141108_iid=1800121_0      AAGTGTTACCTT-AATATCTTCAATATTAAACTCTTT-TTAAGCTATTTAAGTA-AAACA   4158
m141031_iid=147540_0       AAGTGTTACCTT-AATATCTTCAATATTAAACTC-TTTTTAAGCTATTTAAGTA-AAACA   4501
m150507_iid=3674924_0      AAGTGTTACCTT-AATATCTTCAATATTAAACTCTTT-TTAAGCTATTTAAGTA-AAACA   4653
m150503_iid=2923751_0      AAGTGTTACCTT-AATATCTTCAATATTAAACTCTTT-TTAAGCTATTTAAGTA-AAACA   4495
m141031_iid=115118_0       AAGTGTTACCTT-AATATCTTCAATATTAAACTCTTT-TTAAGCTATTTAAGTA-AAACA   3345
m150430_iid=2722448_0      AAGTGTTACCTT-AATATCTTCAATATTAAACTCTTT-TTAAGCTATTTAAGTA-AAACA   3281
m150507_iid=3102647_0      AAGTGTTACCTT-AATATCTTCAATATTAAACTCTTT-TTAAGCTATTTAAGTA-AAACA   1591
m141108_iid=1811866_0      AAGTGTTACCTT-AATATCTTCAATATTAAACTCTTT-TTAAGCTATTTAAGTA-AAACA   3463
m150501_iid=2948495_0      AAGTGTTACCTT-AATATCTTCAATATTAAACTCTTT-TTAAGCTATTTAAGTA-AAACA   2889
m150504_iid=3238195_0      AAGTGTTACCTT-AATATCTTCAATATTAAACTCTTT-TTAAGCTATTTAAGTA-AAACA   5221
m150503_iid=3388511_0      AAGTGTTACCTT-AATATCTTCAATATTAAACTC-TTTTTAAGCTATTTAAGTA-AAACA   2920
m150505_iid=3777611_0      AAGTGTTACCTT-AATATCTTCAATATTAAACTC-TTTTTAAGCTATTTAAGTA-AAACA   4337
m150430_iid=2438833_0      AAGTGTTACCTT-AATATCTTCAATATTAAACTC-TTTTTAAGCTATTTAAGTA-AAACA   4676
m141108_iid=1698969_0      AAGTGTTACCTT-AATATCTTCAATATTAAACTC-TTTTTAAGCTATTTAAGTA-AAACA   5802
m150430_iid=3059190_0      AAGTGTTACCTT-AATATCTTCAATATTAAACTCTTT-TTAAGCTATTTAAGTA-AAACA   1525
m141107_iid=1383067_0      AAGTGTTACCTT-AATATCTTCAATATTAAACTCTT--TTAAGCTATTTAAGTA-AAACA   1531
m141101_iid=597008_0       ------------------------------------------------------------   0
m150506_iid=2596496_0      AAGTGTTACCTT-AATATCTTCAATATTAAACTCTTT-TTAAGCTATTTAAGTA-AAACA   6162
m141102_iid=910051_0       AAGTGTTACCTT-AATATCTTCAATATTAAACTCTTT-TTAAGCTATTTAAGTA-AAACA   5649
m150505_iid=3784017_0      AAGTGTTACCTT-AATATCTTCAATATTAAACTC-TTTTTAAGCTATTTAAGTA-AAACA   4449
m141101_iid=682151_0       AAGTGTTACCTT-AATATCTTCAATATTAAACTCTTTTTTAAGCTATTTAAGTA-AAACA   3519
m150501_iid=3748029_0      AAGTGTTACCTT-AATATCTTCAATATTAAACTC-TTTTTAAGCTATTTAAGTA-AAACA   4062
m150504_iid=2876272_0      AAGTGTTACCTT-AATATCTTCAATATTAAACTC-TTTTTAAGCTATTTAAGTA-AAACA   5926
NC_042820.1                AAGTGTTACCTT-AATATCTTCAATATTAAACTC-TTTTTAAGCTATTTAAGTA-AAACA   6169
m141102_iid=1228825_0      AAGTGTTACCTT-AATATCTTCAATATTAAACTC-TTTTTAAGCTATTTAAGTA-AAACA   5995
m150504_iid=2876274_0      AAGTGTTACCTT-AATATCTTCAATATTAAACTC-TTTTTAAGCTATTTAAGTA-AAACA   5998
m141030_iid=80036_0        AAGTGTTACCTT-AATATCTTCAATATTAAACTC-TTTTTAAGCTATTTAAGTA-AAACA   5759
m150506_iid=2596494_0      AAGTGTTACCTT-AATATCTTCAATATTAAACTC-TTTTTAAGCTATTTAAGTA-AAACA   6164
m150503_iid=2526862_0      AAGTGTTACCTT-AATATCTTCAATATTAAACTC-TTTTTAAGCTATTTAAGTA-AAACA   5848
m150506_iid=2567000_0      AAGTGTTACCTT-AATATCTTCAATATTAAACTC-TTTTTAAGCTATTTAAGTA-AAACA   3797
m150507_iid=3073271_0      AAGTGTTACCTT-AATATCTTCAATATTAAACTC-TTTTTAAGCTATTTAAGTA-AAACA   5101
m150504_iid=3156087_0      AAGTGTTACCTT-AATATCTTCAATATTAAACTC-TTTTTAAGCTATTTAAGTA-AAACA   4924
m150505_iid=2498502_0      AAGTGTTACCTT-AATATCTTCAATATTAAACTC-TTTTTAAGCTATTTAAGTA-AAACA   2574
m141102_iid=754227_0       AAGTGTTACCTT-AATATCTTCAATATTAAACTC-TTTTTAAGCTATTTAAGTA-AAACA   5136
m150507_iid=3469514_0      AAGTGTTACCTT-AATATCTTCAATATTAAACTC-TTTTTAAGCTATTTAAGTA-AAACA   5694
Rdo_MitoGenome             AAGTGTTACCTT-AATATCTTCAATATTAAACTC-TTTTTAAGCTATTTAAGTA-AAACA   6169
m141101_iid=614933_0       AAGTGTTACCTT-AATATCTTCAATATTAAACTC-TTTTTAAGCTATTTAAGTA-AAACA   4721

                                                             nad5

m141031_iid=347546_0       AAATAAGAAAAGAAACCCAAAAAATTAACATAATTAAAAATAACTTTAATTGATTAGAAA   959
```

```
m150506_iid=3127414_0      AAATAAGAAAAGAAACCCAAAAAATTAACATAATTAA-AATAACTTTAATTGATTAGAAA   2694
m141102_iid=1245517_0      AAATAAGAAAAGAAACCCAAAAAATTAACATAATTAAAAATAACTTTAATTGATTAGAAA   4352
m141107_iid=1405727_0      AAATAAGAAAAGAAACCCAAAA-ATTAACATAATTAAAAATAACTTTAATTGATTAGAAA   1531
m141102_iid=987614_0       AAATAAGAAAAGAAACCCAAAAAATTAACATAATTAAAAATAACTTTAATTGATTAGAAA   4503
m141108_iid=1701429_0      AAATAAGAAAAGAAACCCAAAAAATTAACATAATTAAAAATAACTTTAATTGATTAGAAA   1240
m141102_iid=987286_0       AAATAAGAAAAGAAACCCAAAAAATTAACATAATTAAAAATAACTTTAATTGATTAGAAA   2455
m150430_iid=3272125_0      AAATAAGAAAAGAAACCCAAAAAATTAACATAATTAAAAATAACTTTAATTGATTAGAAA   3977
m141102_iid=754225_0       AAATAAGAAAAGAAACCCAAAAAATTAACATAATTAAAAATAACTTTAATTGATTAGAAA   5142
m141107_iid=1409934_0      AAATAAGAAAAGAAACCCAAAAAATTAACATAATTAAAAATAACTTTAATTGATTAGAAA   5687
m150503_iid=3416920_0      AAATAAGAAAAGAAACCCAAAAAATTAACATAATTAAAAATAACTTTAATTGATTAGAAA   4405
m141108_iid=1800121_0      AAATAAGAAAAGAAACCCAAAAAATTAACATAATTAAAAATAACTTTAATTGATTAGAAA   4218
m141031_iid=147540_0       AAATAAGAAAAGAAACCCAAAAAATTAACATAATTAAAAATAACTTTAATTGATTAGAAA   4561
m150507_iid=3674924_0      AAATAAGAAAAGAAACCCAAAAAATTAACATAATTAAAAATAACTTTAATTGATTAGAAA   4713
m150503_iid=2923751_0      AAATAAGAAAAGAAACCCAAAAAATTAACATAATTAAAAATAACTTTAATTGATTAGAAA   4555
m141031_iid=115118_0       AAATAAGAAAAGAAACCCAAAAAATTAACATAATTAAAAATAACTTTAATTGATTAGAAA   3405
m150430_iid=2722448_0      AAATAAGAAAAGAAACCCAAAAAATTAACATAATTAAAAATAACTTTAATTGATTAGAAA   3341
m150507_iid=3102647_0      AAATAAGAAAAGAAACCCAAAAAATTAACATAATTA---AAAACTTTAATTGATTAGAAA   1648
m141108_iid=1811866_0      AAATAAGAAAAGAAACCCAAAAAATTAACATAATTAAAAATAACTTTAATTGATTAGAAA   3523
m150501_iid=2948495_0      AAATAAGAAAAGAAACCCAAAAAATTAACATAATTAAAAATAACTTTAATTGATTAGAAA   2949
m150504_iid=3238195_0      AAATAAGAAAAGAAACCCAAAAAATTAACATAATTAAAAATAACTTTAATTGATTAGAAA   5281
m150503_iid=3388511_0      AAATAAGAAAAGAAACCCAAAAAATTAACATAATTAAAAATAACTTTAATTGATTAGAAA   2980
m150505_iid=3777611_0      AAATAAGAAAAGAAACCCAAAAAATTAACATAATTAAAAATAACTTTAATTGATTAGAAA   4397
m150430_iid=2438833_0      AAATAAGAAAAGAAACCCAAAAAATTAACATAATTAAAAATAACTTTAATTGATTAGAAA   4736
m141108_iid=1698969_0      AAATAAGAAAAGAAACCCAAAAAATTAACATAATTAAAAATAACTTTAATTGATTAGAAA   5862
m150430_iid=3059190_0      AAATAAGAAAAGAAACCCAAAAAATTAACATAATTAAAAATAACTTTAATTGATTAGAAA   1585
m141107_iid=1383067_0      AAATAAGAAAAGAAACCCAAAAAATTAACATAATTAAAAATAACTTTAATTGATTAGAAA   1591
m141101_iid=597008_0       ------------------------------------------------------------   0
m150506_iid=2596496_0      AAATAAGAAAAGAAACCCAAAAAATTAACATAATTAAAAATAACTTTAATTGATTAGAAA   6222
m141102_iid=910051_0       AAATAAGAAAAGAAACCCAAAAAATTAACATAATTAAAAATAACTTTAATTGATTAGAAA   5709
m150505_iid=3784017_0      AAATAAGAAAAGAAACCCAAAAAATTAACATAATTAAAAATAACTTTAATTGATTAGAAA   4509
m141101_iid=682151_0       AAATAAGAAAAGAAACCCAAAAAATTAACATAATTAAAAATAACTTTAATTGATTAGAAA   3579
m150501_iid=3748029_0      AAATAAGAAAAGAAACCCAAAAAATTAACATAATTAAAAATAACTTTAATTGATTAGAAA   4122
m150504_iid=2876272_0      AAATAAGAAAAGAAACCCAAAAAATTAACATAATTAAAAATAACTTTAATTGATTAGAAA   5986
NC_042820.1                AAATAAGAAAAGAAACCCAAAAAATTAACATAATTAAAAATAACTTTAATTGATTAGAAA   6229
m141102_iid=1228825_0      AAATAAGAAAAGAAACCCAAAAAATTAACATAATTAAAAATAACTTTAATTGATTAGAAA   6055
m150504_iid=2876274_0      AAATAAGAAAAGAAACCCAAAAAATTAACATAATTAAAAATAACTTTAATTGATTAGAAA   6058
m141030_iid=80036_0        AAATAAGAAAAGAAACCCAAAAAATTAACATAATTAAAAATAACTTTAATTGATTAGAAA   5819
m150506_iid=2596494_0      AAATAAGAAAAGAAACCCAAAAAATTAACATAATTAAAAATAACTTTAATTGATTAGAAA   6224
m150503_iid=2526862_0      AAATAAGAAAAGAAACCCAAAAAATTAACATAATTAAAAATAACTTTAATTGATTAGAAA   5908
m150506_iid=2567000_0      AAATAAGAAAAGAAACCCAAAAAATTAACATAATTAAAAATAACTTTAATTGATTAGAAA   3857
m150507_iid=3073271_0      AAATAAGAAAAGAAACCCAAAAAATTAACATAATTAAAAATAACTTTAATTGATTAGAAA   5161
m150504_iid=3156087_0      AAATAAGAAAAGAAACCCAAAAAATTAACATAATTAAAAATAACTTTAATTGATTAGAAA   4984
m150505_iid=2498502_0      AAATAAGAAAAGAAACCCAAAAAATTAACATAATTAAAAATAACTTTAATTGATTAGAAA   2634
m141102_iid=754227_0       AAATAAGAAAAGAAACCCAAAAAATTAACATAATTAAAAATAACTTTAATTGATTAGAAA   5196
m150507_iid=3469514_0      AAATAAGAAAAGAAACCCAAAAAATTAACATAATTAAAAATAACTTTAATTGATTAGAAA   5754
Rdo_MitoGenome             AAATAAGAAAAGAAACCCAAAAAATTAACATAATTAAAAATAACTTTAATTGATTAGAAA   6229
m141101_iid=614933_0       AAATAAGAAAAGAAACCCAAAAAATTAACATAATTAAAAATAACTTTAATTGATTAGAAA   4781
```

nad5

```
m141031_iid=347546_0       AATTAAAATGTAAACTTCGAGTTAGCTTTAAAATAT-TATTATAAAACCCTGGGCCCTAA   1018
m150506_iid=3127414_0      AAATTAAATG-TAACTTCGAGTTAGCTTTAAAATATTTATTATAAAACCCTGGGCCCTAA   2753
m141102_iid=1245517_0      AAATAAATTGTAA-----GTTAGAGCTTTAAAATATTTATTATAAAACCCTGGGCCCCTA   4407
m141107_iid=1405727_0      AAATTAAATGTAAACTTCGAGTTAGCTTTAAAATATTTATTATAAAACCCTGGGCCCCTA   1591
m141102_iid=987614_0       AAATTAAATGTAAACTTCGAGTTAGCTTTAAAATATTTATTATAAAACCCTGGGCCCCTA   4563
m141108_iid=1701429_0      AAATTAAATGTAAACTTCGAGTTAGCTTTAAAATATTTATTATAAAACCCTGGGCCCCTA   1300
m141102_iid=987286_0       AAATTAAATGTAAACTTCGAGTTAGCTTTAAAATATTTATTATAAAACCCTGGGCCCCTA   2515
m150430_iid=3272125_0      AAATTAAATGTAAACTTCGAGTTAGCTTTAAAATATT-TTTATAAAACCCTGGGCCCCTA   4036
m141102_iid=754225_0       AAATTAAATGTAAACTTCGAGTTAGCTTTAAAATATTTATTATAAAACCCTGGGCCCCTA   5202
m141107_iid=1409934_0      AAATTAAATGTAAACTTCGAGTTAGCTTTAAAATATTTATTATAAAACCCTGGGCCCCTA   5747
m150503_iid=3416920_0      AAATTAAATGTAAACTTCGAGTTAGCTTTAAAATATTTATTATAAAACCCTGGGCCCCTA   4465
m141108_iid=1800121_0      A-ATTAAATGTAAACTTCGAGTTAGCTTTAAAATATTTATTATAAAACCCTGGGCCCCTA   4277
m141031_iid=147540_0       AAATTAAATGTAAACTTCGAGTTAGCTTTAAAATATTTATTATAAAACCCTGGGCCCCTA   4621
m150507_iid=3674924_0      AAATTAAATGTAAACTTCGAGTTAGCTTTAAAATATTTATT-ATAAACCCTGGGCCCCTA   4772
m150503_iid=2923751_0      AAATTAAATGTAAACTTCGAGTTAGCTTTAAAATATTTATTATAAAACCCTGGGCCCCTA   4615
m141031_iid=115118_0       AAATTAAATGTAAACTTCGAGTTAGCTTTAAAATATTTATTATAAAACCCTGGGCCCCTA   3465
m150430_iid=2722448_0      AAATTAAATGTAAACTTCGAGTTAGCTTTAAAATATTTATTATAAAACCCTGGGCCCCTA   3401
m150507_iid=3102647_0      AAATTAAATGTAAACTTCGAGTTAGCTTTAAAATATTTATTATAAAACCCTGGGCCCCTA   1708
m141108_iid=1811866_0      AAATTAAATGTAAACTTCGAGTTAGCTTTAAAATATTTATTATAAAACCCTGGGCCCCTA   3583
m150501_iid=2948495_0      AAATTAAATGTAAACTTCGAGTTAGCTTTAAAATATTTATTATAAAACCCTGGGCCCCTA   3009
m150504_iid=3238195_0      AAATTAAATGTAAACTTCGAGTTAGCTTTAAA-TATTTATTATAAAACCCTGGGCCCCTA   5340
m150503_iid=3388511_0      AAATTAAATGTAAACTTCGAGTTAGCTTTAAAATATTTATTATAAAACCCTGGGCCCCTA   3040
```

```
m150505_iid=3777611_0      AAATTAAATGTAAACTTCGAGTTAGCTTTAAAATATTTATTATAAAACCCTGGCCCCTA   4457
m150430_iid=2438833_0      AAATTAAATGTAAACTTCGAGTTAGCTTTAAAATATTTATTATAAAACCCTGGCCCCTA   4796
m141108_iid=1698969_0      AAATTAAATGTAAACTTCGAGTTAGCTTTAAAATATTTATTATAAAACCCTGGCCCCTA   5922
m150430_iid=3059190_0      AAATTAAATGTAAACTTCGAGTTAGCTTTAAAATATTTATTATAAAACCCTGGCCCCTA   1645
m141107_iid=1383067_0      AAATTAAATGTAAACTTCGAGTTAGCTTTAAAATATTTATTATAAAACCCTGGCCCCTA   1651
m141101_iid=597008_0       -----------------------------------------------------------   0
m150506_iid=2596496_0      AAATTAAATGTAAACTTCGAGTTAGCTTTAAAATATTTATTATAAAACCCTGGCCCCTA   6282
m141102_iid=910051_0       AAATTAAATGTAAACTTCGAGTTAGCTTTAAAATATTTATTATAAAACCCTGGCCCCTA   5769
m150505_iid=3784017_0      AAATTAAATGTAAACTTCGAGTTAGCTTTAAAATATTTATTATAAAACCCTGGCCCCTA   4569
m141101_iid=682151_0       AAATTAAATGTAAACTTCGAGTTAGCTTTAAAATATTTATTATAAAACCCTGGCCCCTA   3639
m150501_iid=3748029_0      AAATTAAATGTAAACTTCGAGTTAGCTTTAAAATATTTATTATAAAACCCTGGCCCCTA   4182
m150504_iid=2876272_0      AAATTAAATGTAAACTTCGAGTTAGCTTTAAAATATTTATTATAAAACCCTGGCCCCTA   6046
NC_042820.1                AAATTAAATGTAAACTTCGAGTTAGCTTTAAAATATTTATTATAAAACCCTGGCCCCTA   6289
m141102_iid=1228825_0      AAATTAAATGTAAACTTCGAGTTAGCTTTAAAATATTTATTATAAAACCCTGGCCCCTA   6115
m150504_iid=2876274_0      AAATTAAATGTAAACTTCGAGTTAGCTTTAAAATATTTATTATAAAACCCTGGCCCCTA   6118
m141030_iid=80036_0        AAATTAAATGTAAACTTCGAGTTAGCTTTAAAATATTTATTATAAAACCCTGGCCCCTA   5879
m150506_iid=2596494_0      AAATTAAATGTAAACTTCGAGTTAGCTTTAAAATATTTATTATAAAACCCTGGCCCCTA   6284
m150503_iid=2526862_0      AAATTAAATGTAAACTTCGAGTTAGCTTTAAAATATTTATTATAAAACCCTGGCCCCTA   5968
m150506_iid=2567000_0      AAATTAAATGTAAACTTCGAGTTAGCTTTAAAATATTTATTATAAAACCCTGGCCCCTA   3917
m150507_iid=3073271_0      AAATTAAATGTAAACTTCGAGTTAGCTTTAAAATATTTATTATAAAACCCTGGCCCCTA   5221
m150504_iid=3156087_0      AAATTAAATGTAAACTTCGAGTTAGCTTTAAAATATTTATTATAAAACCCTGGCCCCTA   5044
m150505_iid=2498502_0      AAATTAAATGTAAACTTCGAGTTAGCTTTAAAATATTTATTATAAAACCCTGGCCCCTA   2694
m141102_iid=754227_0       AAATTAAATGTAAACTTCGAGTTAGCTTTAAAATATTTATTATAAAACCCTGGCCCCTA   5256
m150507_iid=3469514_0      AAATTAAATGTAAACTTCGAGTTAGCTTTAAAATATTTATTATAAAACCCTGGCCCCTA   5814
Rdo_MitoGenome             AAATTAAATGTAAACTTCGAGTTAGCTTTAAAATATTTATTATAAAACCCTGGCCCCTA   6289
m141101_iid=614933_0       AAATTAAATGTAAACTTCGAGTTAGCTTTAAAATATTTATTATAAAACCCTGGCCCCTA   4841

                                                  nad5

m141031_iid=347546_0       ATGTTTCAAGTCAACCTTGATC-AAAAGAAGAAATAAATAATCGGC-AAGTGTTAAAGGA   1076
m150506_iid=3127414_0      ATGT-TCAAGTCAACCTTGATC-AAAAGAAGAAATAAATAATCGGCCAAGTGTTAAAGGA   2811
m141102_iid=1245517_0      AATGTTCAAGTCAACCTTGATCAAAA-GAAGAAATAAATAATCGGCCAAGTGTTAAAGGA   4466
m141107_iid=1405727_0      AATGTTCAAGTCAACCTTGATCAAAAGAA--AAATAAATAATCGGCCAAGTGTTAAAGGA   1649
m141102_iid=987614_0       AATGTTCAAGTCAACCTTGATC-AAAAGAAGAAATAAATAATCGGCCAAGTGTTAAAGGA   4622
m141108_iid=1701429_0      AATGTTCAAGTCAACCTTGATCAA-AAGAAGAAATAAATAATCGGCCAAGTGTTAAAGGA   1359
m141102_iid=987286_0       AATGTTCAAGTCAACCTTGATCAA-AAGAAGAAATAAATAATCGGCCAAGTGTTAAAGGA   2574
m150430_iid=3272125_0      AATGTTCAAGTCAACCTTGATCAA-AAGAAGAAATAAATAATCGGCCAAGTGTTAAAGGA   4095
m141102_iid=754225_0       AATGTTCAAGTCAACCTTGATCAA-AAGAAGAAATAAATAATCGGCCAAGTGTTAAAGGA   5261
m141107_iid=1409934_0      AATGTTCAAGTCAACCTTGATCAA-AAGAAGAAATAAATAATCGGCCAAGTGTTAAAGGA   5806
m150503_iid=3416920_0      AATGTTCAAGTCAACCTTGATCAA-AAGAAGAAATAAATAATCGGCCAAGTGTTAAAGGA   4524
m141108_iid=1800121_0      AATGTTCAAGTCAACCTTGATCAA-AAGAAGAAATAAATAATCGGCCAAGTGTTAAAGGA   4336
m141031_iid=147540_0       AATGTTCAAGTCAACCTTGATC-AAAAGAAGAAATAAATAATCGGCCAAGTGTTAAAGGA   4680
m150507_iid=3674924_0      AATGTTCAAGTCAACCTTGATCAAA-AGAAGAAATAAATAATCGGCCAAGTGTTAAAGGA   4831
m150503_iid=2923751_0      AATGTTCAAGTCAACCTTGATCAAA-AGAAGAAATAAATAATCGGCCAAGTGTTAAAGGA   4674
m141031_iid=115118_0       AATGTTCAAGTCAACCTTGATCAAA-AGAAGAAATAAATAATCGGCCAAGTGTTAAAGGA   3524
m150430_iid=2722448_0      AATGTTCAAGTCAACCTTGATCAAA-AGAAGAAATAAATAATCGGCCAAGTGTTAAAGGA   3460
m150507_iid=3102647_0      AATGTTCAAGTCAACCTTGATCAA--AGAAGAAATAAATAATCGGCCAAGTGTTAAAGGA   1766
m141108_iid=1811866_0      AATGTTCAAGTCAACCTTGATCAA-AAGAAGAAATAAATAATCGGCCAAGTGTTAAAGGA   3642
m150501_iid=2948495_0      AATGTTCAAGTCAACCTTGATCAA-AAGAAGAAATAAATAATCGGCCAAGTGTTAAAGGA   3068
m150504_iid=3238195_0      AATGTTCAAGTCAACCTTGATCAA-AAGAAGAAATAAATAATCGGCCAAGTGTTAAAGGA   5399
m150503_iid=3388511_0      AATGTTCAAGTCAACCTTGATC-AAAAGAAGAAATAAATAATCGGCCAAGTGTTAAAGGA   3099
m150505_iid=3777611_0      AATGTTCAAGTCAACCTTGATC-AAAAGAAGAAATAAATAATCGGCCAAGTGTTAAAGGA   4516
m150430_iid=2438833_0      AATGTTCAAGTCAACCTTGATC-AAAAGAAGAAATAAATAATCGGCCAAGTGTTAAAGGA   4855
m141108_iid=1698969_0      AATGTTCAAGTCAACCTTGATCAAAAAGAAGAAATAAATAATCGGCCAAGTGTTAAAGGA   5982
m150430_iid=3059190_0      AATGTTCAAGTCAACCTTGATCAA-AAGAAGAAATAAATAATCGGCCAAGTGTTAAAGGA   1704
m141107_iid=1383067_0      AATGTTCAAGTCAACCTTGATCAA-AAGAAGAAATAAATAATCGGCCAAGTGTTAAAGGA   1710
m141101_iid=597008_0       ------------------------------------------------------------   0
m150506_iid=2596496_0      AATGTTCAAGTCAACCTTGATCAAA-AGAAGAAATAAATAATCGGCCAAGTGTTAAAGGA   6341
m141102_iid=910051_0       AATGTTCAAGTCAACCTTGATCAAA-AGAAGAAATAAATAATCGGCCAAGTGTTAAAGGA   5828
m150505_iid=3784017_0      AATGTTCAAGTCAACCTTGATCAAAAAGAAGAAATAAATAATCGGCCAAGTGTTAAAGGA   4629
m141101_iid=682151_0       AATGTTCAAGTCAACCTTGATC-AAAAGAAGAAATAAATAATCGGCCAAGTGTTAAAGGA   3698
m150501_iid=3748029_0      AATGTTCAAGTCAACCTTGATC-AAAAGAAGAAATAAATAATCGGCCAAGTGTTAAAGGA   4241
m150504_iid=2876272_0      AATGTTCAAGTCAACCTTGATC-AAAAGAAGAAATAAATAATCGGCCAAGTGTTAAAGGA   6105
NC_042820.1                AATGTTCAAGTCAACCTTGATC-AAAAGAAGAAATAAATAATCGGCCAAGTGTTAAAGGA   6348
m141102_iid=1228825_0      AATGTTCAAGTCAACCTTGATC-AAAAGAAGAAATAAATAATCGGCCAAGTGTTAAAGGA   6174
m150504_iid=2876274_0      AATGTTCAAGTCAACCTTGATC-AAAAGAAGAAATAAATAATCGGCCAAGTGTTAAAGGA   6177
m141030_iid=80036_0        AATGTTCAAGTCAACCTTGATC-AAAAGAAGAAATAAATAATCGGCCAAGTGTTAAAGGA   5938
m150506_iid=2596494_0      AATGTTCAAGTCAACCTTGATC-AAAAGAAGAAATAAATAATCGGCCAAGTGTTAAAGGA   6343
m150503_iid=2526862_0      AATGTTCAAGTCAACCTTGATC-AAAAGAAGAAATAAATAATCGGCCAAGTGTTAAAGGA   6027
m150506_iid=2567000_0      AATGTTCAAGTCAACCTTGATC-AAAAGAAGAAATAAATAATCGGCCAAGTGTTAAAGGA   3976
m150507_iid=3073271_0      AATGTTCAAGTCAACCTTGATC-AAAAGAAGAAATAAATAATCGGCCAAGTGTTAAAGGA   5280
m150504_iid=3156087_0      AATGTTCAAGTCAACCTTGATC-AAAAGAAGAAATAAATAATCGGCCAAGTGTTAAAGGA   5103
```

```
m150505_iid=2498502_0      AATGTTCAAGTCAACCTTGATC-AAAAGAAGAAATAAATAATCGGCCAAGTGTTAAAGGA   2753
m141102_iid=754227_0       AATGTTCAAGTCAACCTTGATC-AAAAGAAGAAATAAATAATCGGCCAAGTGTTAAAGGA   5315
m150507_iid=3469514_0      AATGTTCAAGTCAACCTTGATC-AAAAGAAGAAATAAATAATCGGCCAAGTGTTAAAGGA   5873
Rdo_MitoGenome             AATGTTCAAGTCAACCTTGATC-AAAAGAAGAAATAAATAATCGGCCAAGTGTTAAAGGA   6348
m141101_iid=614933_0       AATGTTCAAGTCAACCTTGATC-AAAAGAAGAAATAAATAATCGGCCAAGTGTTAAAGGA   4900


m141031_iid=347546_0       AAATTCTTTATCCCTAAAGAAAGAAATTAAAGGCAAAGCCTCATATTAACGAAGAAAACC   1136
m150506_iid=3127414_0      AAAATTCTTATCCCTAAAGAA-GAAATTAAAGGC-AAGCCTCATATTAACGAAGAAAACC   2869
m141102_iid=1245517_0      AAAATTCTTATCCCTAAAGAA-GAAATTAAAGGC-AAGCCTCATATTAACGAAGAAAACC   4524
m141107_iid=1405727_0      AAAATTCTTATCCCTAAAGAA-GAAATTAAAGGC-AAGCCTCATATTAACGAAGAA-ACC   1706
m141102_iid=987614_0       AAAATTCTTATCCCTAAAGAA-GAAATTAAAGGC-AAGCCTCATATTAACGAAGAAAACC   4680
m141108_iid=1701429_0      AAAATTCTTATCCCTAAAGAA-GAAATTAAAGGC-AAGCCTCATATTAACGAAGAAAACC   1417
m141102_iid=987286_0       AAAATTCTTATCCCTAAAGAA-GAAATTAAAGGC-AAGCCTCATATTAACGAAGAAAACC   2632
m150430_iid=3272125_0      AAAATTCTTATCCCTAAAGAA-GAAATTAAAGGC-AAGCCTCATATTAACGAAGAAAACC   4153
m141102_iid=754225_0       AAAATTCTTATCCCTAAAGAA-GAAATTAAAGGC-AAGCCTCATATTAACGAAGAAAACC   5319
m141107_iid=1409934_0      AAAATTCTTATCCCTAAAGAA-GAAATTAAAGGC-AAGCCTCATATTAACGAAGAAAACC   5864
m150503_iid=3416920_0      AAAATTCTTATCCCTAAAGAA-GAAATTAAAGGC-AAGCCTCATATTAACGAAGAAAACC   4582
m141108_iid=1800121_0      AAAATTCTTATCCCTAAAGAA-GAAATTAAAGGC-AAGCCTCATATTAACGAAGAAAACC   4394
m141031_iid=147540_0       AAAATTCTTATCCCTAAAGAA-GAAATTAAAGGC-AAGCCTCATATTAACGAAGAAAACC   4738
m150507_iid=3674924_0      AAAATTCTTATCCCTAAAGAA-GAAATTAAAGGC-AAGCCTCATATTAACGAAGAAAACC   4889
m150503_iid=2923751_0      AAAATTCTTATCCCTAAAGAA-GAAATTAAAGGC-AAGCCTCATATTAACGAAGAAAACC   4732
m141031_iid=115118_0       AAAATTCTTATCCCTAAAGAA-GAAATTAAAGGC-AAGCCTCATATTAACGAAGAAAACC   3582
m150430_iid=2722448_0      AAAATTCTTATCCCTAAAGAA-GAAATTAAAGGC-AAGCCTCATATTAACGAAGAAAACC   3518
m150507_iid=3102647_0      AAAATTCTTATCCCTAAAGAA-GAAATTAAAGGC-AAGCCTCATATTAACGAAGAAAACC   1824
m141108_iid=1811866_0      AAAATTCTTATCCCTAAAGAA-GAAATTAAAGGC-AAGCCTCATATTAACGAAGAAAACC   3700
m150501_iid=2948495_0      AAAATTCTTATCCCTAAAGAA-GAAATTAAAGGC-AAGCCTCATATTAACGAAGAAAACC   3126
m150504_iid=3238195_0      AAAATTCTTATCCCTAAAGAA-GAAATTAAAGGC-AAGCCTCATATTAACGAAGAAAACC   5457
m150503_iid=3388511_0      AAAATTCTTATCCCTAAAGAA-GAAATTAAAGGC-AAGCCTCATATTAACGAAGAAAACC   3157
m150505_iid=3777611_0      AAAATTCTTATCCCTAAAGAA-GAAATTAAAGGC-AAGCCTCATATTAACGAAGAAAACC   4574
m150430_iid=2438833_0      AAAATTCTTATCCCTAAAGAA-GAAATTAAAGGC-AAGCCTCATATTAACGAAGAAAACC   4913
m141108_iid=1698969_0      AAAATTCTTATCCCTAAAGAA-GAAATTAAAGGC-AAGCCTCATATTAACGAAGAAAACC   6040
m150430_iid=3059190_0      AAAATTCTTATCCCTAAAGAA-GAAATTAAAGGC-AAGCCTCATATTAACGAAGAAAACC   1762
m141107_iid=1383067_0      AAAATTCTTATCCCTAAAGAA-GAAATTAAAGGC-AAGCCTCATATTAACGAAGAAAACC   1768
m141101_iid=597008_0       ------------------------------------------------------------   0
m150506_iid=2596496_0      AAAATTCTTATCCCTAAAGAA-GAAATTAAAGGC-AAGCCTCATATTAACGAAGAAAACC   6399
m141102_iid=910051_0       AAAATTCTTATCCCTAAAGAA-GAAATTAAAGGC-AAGCCTCATATTAACGAAGAAAACC   5886
m150505_iid=3784017_0      AAAATTCTTATCCCTAAAGAA-GAAATTAAAGGC-AAGCCTCATATTAACGAAGAAAACC   4687
m141101_iid=682151_0       AAAATTCTTATCCCTAAAGAA-GAAATTAAAGGC-AAGCCTCATATTAACGAAGAAAACC   3756
m150501_iid=3748029_0      AAAATTCTTATCCCTAAAGAA-GAAATTAAAGGC-AAGCCTCATATTAACGAAGAAAACC   4299
m150504_iid=2876272_0      AAAATTCTTATCCCTAAAGAA-GAAATTAAAGGC-AAGCCTCATATTAACGAAGAAAACC   6163
NC_042820.1                AAAATTCTTATCCCTAAAGAA-GAAATTAAAGGC-AAGCCTCATATTAACGAAGAAAACC   6406
m141102_iid=1228825_0      AAAATTCTTATCCCTAAAGAA-GAAATTAAAGGC-AAGCCTCATATTAACGAAGAAAACC   6232
m150504_iid=2876274_0      AAAATTCTTATCCCTAAAGAA-GAAATTAAAGGC-AAGCCTCATATTAACGAAGAAAACC   6235
m141030_iid=80036_0        AAAATTCTTATCCCTAAAGAA-GAAATTAAAGGC-AAGCCTCATATTAACGAAGAAAACC   5996
m150506_iid=2596494_0      AAAATTCTTATCCCTAAAGAA-GAAATTAAAGGC-AAGCCTCATATTAACGAAGAAAACC   6401
m150503_iid=2526862_0      AAAATTCTTATCCCTAAAGAA-GAAATTAAAGGC-AAGCCTCATATTAACGAAGAAAACC   6085
m150506_iid=2567000_0      AAAATTCTTATCCCTAAAGAA-GAAATTAAAGGC-AAGCCTCATATTAACGAAGAAAACC   4034
m150507_iid=3073271_0      AAAATTCTTATCCCTAAAGAA-GAAATTAAAGGC-AAGCCTCATATTAACGAAGAAAACC   5338
m150504_iid=3156087_0      AAAATTCTTATCCCTAAAGAA-GAAATTAAAGGC-AAGCCTCATATTAACGAAGAAAACC   5161
m150505_iid=2498502_0      AAAATTCTTATCCCTAAAGAA-GAAATTAAAGGC-AAGCCTCATATTAACGAAGAAAACC   2811
m141102_iid=754227_0       AAAATTCTTATCCCTAAAGAA-GAAATTAAAGGC-AAGCCTCATATTAACGAAGAAAACC   5373
m150507_iid=3469514_0      AAAATTCTTATCCCTAAAGAA-GAAATTAAAGGC-AAGCCTCATATTAACGAAGAAAACC   5931
Rdo_MitoGenome             AAAATTCTTATCCCTAAAGAA-GAAATTAAAGGC-AAGCCTCATATTAACGAAGAAAACC   6406
m141101_iid=614933_0       AAAATTCTTATCCCTAAAGAA-GAAATTAAAGGC-AAGCCTCATATTAACGAAGAAAACC   4958


m141031_iid=347546_0       AAGAAAAATTTATAAAATTTAAAGATTTAGAATGATAATTTGAAAAGGAACGTGAAACTT   1196
m150506_iid=3127414_0      AAGAAAAATTTATAAAATTTAAAGATTTAGAATGATAATTTAAAGAAAAACGTGAAACTT   2929
m141102_iid=1245517_0      AAGAAAAATTTATAAAATTTAAAGATTTAGAATGATAATTTAAAGAAAAACGTGAAACTT   4584
m141107_iid=1405727_0      AAGAAAAATTTATAAAATTTAAAGATTTAGAATGATAATTTAAAGAAAAACGTGAAACTT   1766
m141102_iid=987614_0       AAGAAAAATTTATAAAATTTAAAGATTTAGAATGATAATTTAAAGAAAAACGTGAAACTT   4740
m141108_iid=1701429_0      AAGAAAAATTTATAAAATTTAAAGATTTAGAATGATAATTTAAAGAAAAACGTGAAACTT   1477
m141102_iid=987286_0       AAGAAAAATTTATAAAATTTAAAGATTTAGAATGATAATTTAAAGAAAAACGTGAAACTT   2692
m150430_iid=3272125_0      AAGAAAAATTTATAAAATTTAAAGATTTAGAATGATAATTTAAAGAAAAACGTGAAACTT   4213
m141102_iid=754225_0       AAGAAAAATTTATAAAATTTAAAGATTTAGAATGATAATTTAAAGAAAAACGTGAAACTT   5379
m141107_iid=1409934_0      AAGAAAAATTTATAAAATTTAAAGATTTAGAATGATAATTTAAAGAAAAACGTGAAACTT   5924
m150503_iid=3416920_0      AAGAAAAATTTATAAAATTTAAAGATTTAGAATGATAATTTAAAGAAAAACGTGAAACTT   4642
m141108_iid=1800121_0      AAGAAAATT-TATAAAATTTAAAGATTTAGAATGATAATTTAAAGAAAAACGTGAAACTT   4453
m141031_iid=147540_0       AAGAAAAATTTATAAAATTTAAAGATTTAGAATGATAATTTAAAGAAAAACGTGAAACTT   4798
m150507_iid=3674924_0      AAGAAAAATTTATAAAATTTAAAGATTTAGAATGATAATTTAAAGAAAAACGTGAAACTT   4949
```

```
m150503_iid=2923751_0      AAGAAAAATTTATAAAATTTAAAGATTTAGAATGATAATTTAAAGAAAAACGTGAAACTT   4792
m141031_iid=115118_0       AAGAAAAATTTATAAAATTTAAAGATTTAGAATGATAATTTAAAGAAAAACGTGAAACTT   3642
m150430_iid=2722448_0      AAGAAAAATTTATAAAATTTAAAGATTTAGAATGATAATTTAAAGAAAAACGTGAAACTT   3578
m150507_iid=3102647_0      AAGAAAATT-TATAAAATTTAAAGATTTAGAATGATAATTTAAAGAAAAACGTGAAACTT   1883
m141108_iid=1811866_0      AAGAAAAATTTATAAAATTTAAAGATTTAGAATGATAATTTAAAGAAAAACGTGAAACTT   3760
m150501_iid=2948495_0      AAGAAAATT-TATAAAATTTAAAGA[illegible]TAATTTAAAGAAAAACGTGAAACTT   3185
                                                   nad5
m150504_iid=3238195_0      AAGAAAAATTTATAAAATTTAAAGA[illegible]TAATTTAAAGAAAAACGTGAAACTT   5517
m150503_iid=3388511_0      AAGAAAAATTTATAAAATTTAAAGATTTAGAATGATAATTTAAAGAAAAACGTGAAACTT   3217
m150505_iid=3777611_0      AAGAAAAATTTATAAAATTTAAAGATTTAGAATGATAATTTAAAGAAAAACGTGAAACTT   4634
m150430_iid=2438833_0      AAGAAAAATTTATAAAATTTAAAGATTTAGAATGATAATTTAAAGAAAAACGTGAAACTT   4973
m141108_iid=1698969_0      AAGAAAAATTTATAAAATTTAAAGATTTAGAATGATAATTTAAAGAAAAACGTGAAACTT   6100
m150430_iid=3059190_0      AAGAAAATT-TATAAAATTTAAAGATTTAGAATGATAATTTAAAGAAAAACGTGAAACTT   1821
m141107_iid=1383067_0      AAGAAAAATTTATAAAATTTAAAGATTTAGAATGATAATTTAAAGAAAAACGTGAAACTT   1828
m141101_iid=597008_0       ------------------------------------------------------------   0
m150506_iid=2596496_0      AAGAAAAATTTATAAAATTTAAAGATTTAGAATGATAATTTAAAGAAAAACGTGAAACTT   6459
m141102_iid=910051_0       AAGAAAAATTTATAAAATTTAAAGATTTAGAATGATAATTTAAAGAAAAACGTGAAACTT   5946
m150505_iid=3784017_0      AAGAAAAATTTATAAAATTTAAAGATTTAGAATGATAATTTAAAGAAAAACGTGAAACTT   4747
m141101_iid=682151_0       AAGAAAAATTTATAAAATTTAAAGATTTAGAATGATAATTTAAAGAAAAACGTGAAACTT   3816
m150501_iid=3748029_0      AAGAAAAATTTATAAAATTTAAAGATTTAGAATGATAATTTAAAGAAAAACGTGAAACTT   4359
m150504_iid=2876272_0      AAGAAAAATTTATAAAATTTAAAGATTTAGAATGATAATTTAAAGAAAAACGTGAAACTT   6223
NC_042820.1                AAGAAAAATTTATAAAATTTAAAGATTTAGAATGATAATTTAAAGAAAAACGTGAAACTT   6466
m141102_iid=1228825_0      AAGAAAAATTTATAAAATTTAAAGATTTAGAATGATAATTTAAAGAAAAACGTGAAACTT   6292
m150504_iid=2876274_0      AAGAAAAATTTATAAAATTTAAAGATTTAGAATGATAATTTAAAGAAAAACGTGAAACTT   6295
m141030_iid=80036_0        AAGAAAAATTTATAAAATTTAAAGATTTAGAATGATAATTTAAAGAAAAACGTGAAACTT   6056
m150506_iid=2596494_0      AAGAAAAATTTATAAAATTTAAAGATTTAGAATGATAATTTAAAGAAAAACGTGAAACTT   6461
m150503_iid=2526862_0      AAGAAAAATTTATAAAATTTAAAGATTTAGAATGATAATTTAAAGAAAAACGTGAAACTT   6145
m150506_iid=2567000_0      AAGAAAAATTTATAAAATTTAAAGATTTAGAATGATAATTTAAAGAAAAACGTGAAACTT   4094
m150507_iid=3073271_0      AAGAAAAATTTATAAAATTTAAAGATTTAGAATGATAATTTAAAGAAAAACGTGAAACTT   5398
m150504_iid=3156087_0      AAGAAAAATTTATAAAATTTAAAGATTTAGAATGATAATTTAAAGAAAAACGTGAAACTT   5221
m150505_iid=2498502_0      AAGAAAAATTTATAAAATTTAAAGATTTAGAATGATAATTTAAAGAAAAACGTGAAACTT   2871
m141102_iid=754227_0       AAGAAAAATTTATAAAATTTAAAGATTTAGAATGATAATTTAAAGAAAAACGTGAAACTT   5433
m150507_iid=3469514_0      AAGAAAAATTTATAAAATTTAAAGATTTAGAATGATAATTTAAAGAAAAACGTGAAACTT   5991
Rdo_MitoGenome             AAGAAAAATTTATAAAATTTAAAGATTTAGAATGATAATTTAAAGAAAAACGTGAAACTT   6466
m141101_iid=614933_0       AAGAAAAATTTATAAAATTTAAAGATTTAGAATGATAATTTAAAGAAAAACGTGAAACTT   5018

                                                  nad5
m141031_iid=347546_0       CTACACCAATTAAAAGGCCAGAAAAAATTACAAATAAAGTTATTAAATTTTATATACAAA   1256
m150506_iid=3127414_0      CTACACCAATTAAAAGGCCAGAAAAAT-TACAAATAAAGTTATAA-ATTTTATATACAAG   2987
m141102_iid=1245517_0      CTACACCAATTAAAAGGCCAGAAAA-ATTACAAATAAAGTTATAAATTTTATATACAAAG   4643
m141107_iid=1405727_0      CTACACCAATTAAAAGGCCAGAAAAAAATTACAAATAAAGTTATAAATTTTATATACAAAG  1826
m141102_iid=987614_0       CTACACCAATTAAAAGGCCAGAAAAAAATTACAAATAAAGTTATAAATTTTATATACAAAG  4800
m141108_iid=1701429_0      CTACACCAATTAAAAGGCCAGAAAAAAATTACAAATAAAGTTATAAATTTTATATACAAAG  1537
m141102_iid=987286_0       CTACACCAATTAAAAGGCCAGAAAAAAATTACAAATAAAGTTATAAATTTTATATACAAAG  2752
m150430_iid=3272125_0      CTACACCAATTAAAAGGCCAGAAAAAAATTACAAATAAAGTTATAAATTTTATATACAAAG  4273
m141102_iid=754225_0       CTACACCAATTAAAAGGCCAGAAAAAAATTACAAATAAAGTTATAAATTTTATATACAAAG  5439
m141107_iid=1409934_0      CTACACCAATTAAAAGGCCAGAAAAAAATTACAAATAAAGTTATAAATTTTATATACAAAG  5984
m150503_iid=3416920_0      CTACACCAATTAAAAGGCCAGAAAAAAATTACAAATAAAGTTATAAATTTTATATACAAAG  4702
m141108_iid=1800121_0      CTACACCAATTAAAAGGCCAGAAAAAAATTACAAATAAAGTTATAAATTTTATATACAAAG  4513
m141031_iid=147540_0       CTACACCAATTAAAAGGCCAGAAAAAAATTACAAATAAAGTTATAAATTTTATATACAAAG  4858
m150507_iid=3674924_0      CTACACCAATTAAAAGGCCAGAAAAAAATTACAAATAAAGTTATAAATTTTATATACAAAG  5009
m150503_iid=2923751_0      CTACACCAATTAAAAGGCCAGAAAAAAATTACAAATAAAGTTATAAATTTTATATACAAAG  4852
m141031_iid=115118_0       CTACACCAATTAAAAGGCCAGAAAAAAATTACAAATAAAGTTATAAATTTTATATACAAAG  3702
m150430_iid=2722448_0      CTACACCAATTAAAAGGCCAGAAAAAAATTACAAATAAAGTTATAAATTTTATATACAAAG  3638
m150507_iid=3102647_0      CTACACCAATTAAAAGGCCAGAAAAAAATTACAAATAAAGTTATAAATTTTATATACAAAG  1943
m141108_iid=1811866_0      CTACACCAATTAAAAGGCCAGAAAAAAATTACAAATAAAGTTATAAATTTTATATACAAAG  3820
m150501_iid=2948495_0      CTACACCAATTAAAAGGCCAGAAAAAAATTACAAATAAAGTTATAAATTTTATATACAAAG  3245
m150504_iid=3238195_0      CTACACCAATTAAAAGGCCAGAAAAAAATTACAAATAAAGTTATAAATTTTATATACAAAG  5577
m150503_iid=3388511_0      CTACACCAATTAAAAGGCCAGAAAAAAATTACAAATAAAGTTATAAATTTTATATACAAAG  3277
m150505_iid=3777611_0      CTACACCAATTAAAAGGCCAGAAAAAAATTACAAATAAAGTTATAAATTTTATATACAAAG  4694
m150430_iid=2438833_0      CTACACCAATTAAAAGGCCAGAAAAAAATTACAAATAAAGTTATAAATTTTATATACAAAG  5033
m141108_iid=1698969_0      CTACACCAATTAAAAGGCCAGAAAAAAATTACAAATAAAGTTATAAATTTTATATACAAAG  6160
m150430_iid=3059190_0      CTACACCAATTAAAAGGCCAGAAAAAAATTACAAATAAAGTTATAAATTTTATATACAAAG  1881
m141107_iid=1383067_0      CTACACCAATTAAAAGGCCAGAAAAAAATTACAAATAAAGTTATAAATTTTATATACAAAG  1888
m141101_iid=597008_0       ------------------------------------------------------------   0
m150506_iid=2596496_0      CTACACCAATTAAAAGGCCAGAAAAAAATTACAAATAAAGTTATAAATTTTATATACAAAG  6519
m141102_iid=910051_0       CTACACCAATTAAAAGGCCAGAAAAAAATTACAAATAAAGTTATAAATTTTATATACAAAG  6006
m150505_iid=3784017_0      CTACACCAATTAAAAGGCCAGAAAAAAATTACAAATAAAGTTATAAATTTTATATACAAAG  4807
m141101_iid=682151_0       CTACACCAATTAAAAGGCCAGAAAAAAATTACAAATAAAGTTATAAATTTTATATACAAAG  3876
m150501_iid=3748029_0      CTACACCAATTAAAAGGCCAGAAAAAAATTACAAATAAAGTTATAAATTTTATATACAAAG  4419
m150504_iid=2876272_0      CTACACCAATTAAAAGGCCAGAAAAAAATTACAAATAAAGTTATAAATTTTATATACAAAG  6283
NC_042820.1                CTACACCAATTAAAAGGCCAGAAAAAAATTACAAATAAAGTTATAAATTTTATATACAAAG  6526
```

```
m141102_iid=1228825_0      CTACACCAATTAAAAGGCCAGAAAAAATTACAAATAAAGTTATAAATTTTATATACAAAG   6352
m150504_iid=2876274_0      CTACACCAATTAAAAGGCCAGAAAAAATTACAAATAAAGTTATAAATTTTATATACAAAG   6355
m141030_iid=80036_0        CTACACCAATTAAAAGGCCAGAAAAAATTACAAATAAAGTTATAAATTTTATATACAAAG   6116
m150506_iid=2596494_0      CTACACCAATTAAAAGGCCAGAAAAAATTACAAATAAAGTTATAAATTTTATATACAAAG   6521
m150503_iid=2526862_0      CTACACCAATTAAAAGGCCAGAAAAAATTACAAATAAAGTTATAAATTTTATATACAAAG   6205
m150506_iid=2567000_0      CTACACCAATTAAAAGGCCAGAAAAAATTACAAATAAAGTTATAAATTTTATATACAAAG   4154
m150507_iid=3073271_0      CTACACCAATTAAAAGGCCAGAAAAAATTACAAATAAAGTTATAAATTTTATATACAAAG   5458
m150504_iid=3156087_0      CTACACCAATTAAAAGGCCAGAAAAAATTACAAATAAAGTTATAAATTTTATATACAAAG   5281
m150505_iid=2498502_0      CTACACCAATTAAAAGGCCAGAAAAAATTACAAATAAAGTTATAAATTTTATATACAAAG   2931
m141102_iid=754227_0       CTACACCAATTAAAAGGCCAGAAAAAATTACAAATAAAGTTATAAATTTTATATACAAAG   5493
m150507_iid=3469514_0      CTACACCAATTAAAAGGCCAGAAAAAATTACAAATAAAGTTATAAATTTTATATACAAAG   6051
Rdo_MitoGenome             CTACACCAATTAAAAGGCCAGAAAAAATTACAAATAAAGTTATAAATTTTATATACAAAG   6526
m141101_iid=614933_0       CTACACCAATTAAAAGGCCAGAAAAAATTACAAATAAAGTTATAAATTTTATATACAAAG   5078
```

nad5

```
m141031_iid=347546_0       GGTAAACAA-TATAATAAGGAGTAGAA-ATATTAACCAAGAATAAAATGAACCAGCAACA   1314
m150506_iid=3127414_0      GTAAACAAA-TATAATAAGGAGTAGAAAATATTAACCAAGATAAAACTGAACCAGCAACA   3046
m141102_iid=1245517_0      GTAAACAAA-TATAATAAGGAGTAGAAAATATTAACCAAGATAAAACTGAACCAGCAACA   4702
m141107_iid=1405727_0      GTAAACAAA-TATAATAAGGAGTAGAAAATATTAACCAAGAT-AAACTGAACCAGCAACA   1884
m141102_iid=987614_0       GTAAACAAA-TATAATAAGGAGTAGAAAATATTAACCAAGATAAAACTGAACCAGCAACA   4859
m141108_iid=1701429_0      GTAAACAAA-TATAATAAGGAGTAGAAAATATTAACCAAGATAAAACTGAACCAGCAACA   1596
m141102_iid=987286_0       GTAAACAAA-TATAATAAGGAGTAGAAAATATTAACCAAGATAAAACTGAACCAGCAACA   2811
m150430_iid=3272125_0      GTAAACAAA-TATAATAAGGAGTAGAAAATATTAACCAAGATAAAACTGAACCAGCAACA   4332
m141102_iid=754225_0       GTAAACAAA-TATAATAAGGAGTAGAAAATATTAACCAAGATAAAACTGAACCAGCAACA   5498
m141107_iid=1409934_0      GTAAACAAA-TATAATAAGGAGTAGAAAATATTAACCAAGATAAAACTGAACCAGCAACA   6043
m150503_iid=3416920_0      GTAAACAAA-TATAATAAGGAGTAGAAAATATTAACCAAGATAAAACTGAACCAGCAACA   4761
m141108_iid=1800121_0      GTAAACAAA-TATAATAAGGAGTAGAAAATATTAACCAAGATAAAACTGAACCAGCAACA   4572
m141031_iid=147540_0       GTAAACAAA-TATAATAAGGAGTAGAAAATATTAACCAAGATAAAACTGAACCAGCAACA   4917
m150507_iid=3674924_0      GTAAACAAA-TATAATAAGGAGTAGAAAATATTAACCAAGATAAAACTGAACCAGCAACA   5068
m150503_iid=2923751_0      GTAAACAAA-TATAATAAGGAGTAGAAAATATTAACCAAGATAAAACTGAACCAGCAACA   4911
m141031_iid=115118_0       GTAAACAAA-TATAATAAGGAGTAGAAAATATTAACCAAGATAAAACTGAACCAGCAACA   3761
m150430_iid=2722448_0      GTAAACAAA-TATAATAAGGAGTAGAAAATATTAACCAAGATAAAACTGAACCAGCAACA   3697
m150507_iid=3102647_0      GTAAACAAA-TATAATAAGGAGTAGAAAATATTAACCAAGATAAAACTGAACCAGCAACA   2002
m141108_iid=1811866_0      GTAAACAAAATATAATAAGGAGTAGAAAATATTAACCAAGATAAAACTGAACCAGCAACA   3880
m150501_iid=2948495_0      GTAAACAAA-TATAATAAGGAGTAGAAAATATTAACCAAGATAAAACTGAACCAGCAACA   3304
m150504_iid=3238195_0      GTAAACAAA-TATAATAAGGAGTAGAAAATATTAACCAAGATAAAACTGAACCAGCAACA   5636
m150503_iid=3388511_0      GTAAACAAA-TATAATAAGGAGTAGAAAATATTAACCAAGATAAAACTGAACCAGCAACA   3336
m150505_iid=3777611_0      GTAAACAAA-TATAATAAGGAGTAGAAAATATTAACCAAGATAAAACTGAACCAGCAACA   4753
m150430_iid=2438833_0      GTAAACAAA-TATAATAAGGAGTAGAAAATATTAACCAAGATAAAACTGAACCAGCAACA   5092
m141108_iid=1698969_0      GTAAACAAA-TATAATAAGGAGTAGAAAATATTAACCAAGATAAAACTGAACCAGCAACA   6219
m150430_iid=3059190_0      GTAAACAAA-TATAATAAGGAGTAGAAAATATTAACCAAGATAAAACTGAACCAGCAACA   1940
m141107_iid=1383067_0      GTAAACAAA-TATAATAAGGAGTAGAAAATATTAACCAAGATAAAACTGAACCAGCAACA   1947
m141101_iid=597008_0       ------------------------------------------------------------   0
m150506_iid=2596496_0      GTAAACAAA-TATAATAAGGAGTAGAAAATATTAACCAAGATAAAACTGAACCAGCAACA   6578
m141102_iid=910051_0       GTAAACAAA-TATAATAAGGAGTAGAAAATATTAACCAAGATAAAACTGAACCAGCAACA   6065
m150505_iid=3784017_0      GTAAACAAA-TATAATAAGGAGTAGAAAATATTAACCAAGATAAAACTGAACCAGCAACA   4866
m141101_iid=682151_0       GTAAACAAA-TATAATAAGGAGTAGAAAATATTAACCAAGATAAAACTGAACCAGCAACA   3935
m150501_iid=3748029_0      GTAAACAAA-TATAATAAGGAGTAGAAAATATTAACCAAGATAAAACTGAACCAGCAACA   4478
m150504_iid=2876272_0      GTAAACAAA-TATAATAAGGAGTAGAAAATATTAACCAAGATAAAACTGAACCAGCAACA   6342
NC_042820.1                GTAAACAAA-TATAATAAGGAGTAGAAAATATTAACCAAGATAAAACTGAACCAGCAACA   6585
m141102_iid=1228825_0      GTAAACAAA-TATAATAAGGAGTAGAAAATATTAACCAAGATAAAACTGAACCAGCAACA   6411
m150504_iid=2876274_0      GTAAACAAA-TATAATAAGGAGTAGAAAATATTAACCAAGATAAAACTGAACCAGCAACA   6414
m141030_iid=80036_0        GTAAACAAA-TATAATAAGGAGTAGAAAATATTAACCAAGATAAAACTGAACCAGCAACA   6175
m150506_iid=2596494_0      GTAAACAAA-TATAATAAGGAGTAGAAAATATTAACCAAGATAAAACTGAACCAGCAACA   6580
m150503_iid=2526862_0      GTAAACAAA-TATAATAAGGAGTAGAAAATATTAACCAAGATAAAACTGAACCAGCAACA   6264
m150506_iid=2567000_0      GTAAACAAA-TATAATAAGGAGTAGAAAATATTAACCAAGATAAAACTGAACCAGCAACA   4213
m150507_iid=3073271_0      GTAAACAAA-TATAATAAGGAGTAGAAAATATTAACCAAGATAAAACTGAACCAGCAACA   5517
m150504_iid=3156087_0      GTAAACAAA-TATAATAAGGAGTAGAAAATATTAACCAAGATAAAACTGAACCAGCAACA   5340
m150505_iid=2498502_0      GTAAACAAA-TATAATAAGGAGTAGAAAATATTAACCAAGATAAAACTGAACCAGCAACA   2990
m141102_iid=754227_0       GTAAACAAA-TATAATAAGGAGTAGAAAATATTAACCAAGATAAAACTGAACCAGCAACA   5552
m150507_iid=3469514_0      GTAAACAAA-TATAATAAGGAGTAGAAAATATTAACCAAGATAAAACTGAACCAGCAACA   6110
Rdo_MitoGenome             GTAAACAAA-TATAATAAGGAGTAGAAAATATTAACCAAGATAAAACTGAACCAGCAACA   6585
m141101_iid=614933_0       GTAAACAAA-TATAATAAGGAGTAGAAAATATTAACCAAGATAAAACTGAACCAGCAACA   5137
```

nad5

```
m141031_iid=347546_0       ATTACAGGAAAAATCAAGCCTAATATACCAAAAAGTATAAAATTTCCACCCTCAGAATT-   1373
m150506_iid=3127414_0      ATTACAGGAAAAATCAAGCCTAA-ATACCAAAAAGTATAAAATTTCCACCCTCAGAAATT   3105
m141102_iid=1245517_0      ATTACAGGAAAAATCAAGCCTAATATACCAAAAAGTATAAAATTTCCACCCTCAGAAATT   4762
m141107_iid=1405727_0      ATTACAGGAAAAATCAAGCCTAATATACCAAAAAGTATAAAATTTCCACCCTCAGAAATT   1944
m141102_iid=987614_0       ATTACAGGAAAAATCAAGCCTAATATACCAAAAAGTATAAAATTTCCACCCTCAGAAATT   4919
m141108_iid=1701429_0      ATTACAGGAAAAATCAAGCCTAATATACCAAAAAGTATAAAATTTCCACCCTCAGAAATT   1656
```

```
m141102_iid=987286_0      ATTACAGGAAAAATCAAGCCTAATATACCAAAAAGTATAAAATTTCCACCCTCAGAAATT   2871
m150430_iid=3272125_0     ATTACAGGAAAAATCAAGCCTAATATACCAAAAAGTATAAAATTTCCACCCTCAGAAATT   4392
m141102_iid=754225_0      ATTACAGGAAAAATCAAGCCTAATATACCAAAAAGTATAAAATTTCCACCCTCAGAAATT   5558
m141107_iid=1409934_0     ATTACAGGAAAAATCAAGCCTAATATACCAAAAAGTATAAAATTTCCACCCTCAGAAATT   6103
m150503_iid=3416920_0     ATTACAGGAAAAATCAAGCCTAATATACCAAAAAGTATAAAATTTCCACCCTCAGAAATT   4821
m141108_iid=1800121_0     ATTACAGGAAAAATCAAGCCTAATATACCAAAAAGTATAAAATTTCCACCCTCAGAAATT   4632
m141031_iid=147540_0      ATTACAGGAAAAATCAAGCCTAATATACCAAAAAGTATAAAATTTCCACCCTCAGAAATT   4977
m150507_iid=3674924_0     ATTACAGGAAAAATCAAGCCTAATATACCAAAAAGTATAAAATTTCCACCCTCAGAAATT   5128
m150503_iid=2923751_0     ATTACAGGAAAAATCAAGCCTAATATACCAAAAAGTATAAAATTTCCACCCTCAGAAATT   4971
m141031_iid=115118_0      ATTACAGGAAAAATCAAGCCTAATATACCAAAAAGTATAAAATTTCCACCCTCAGAAATT   3821
m150430_iid=2722448_0     ATTACAGGAAAAATCAAGCCTAATATACCAAAAAGTATAAAATTTCCACCCTCAGAAATT   3757
m150507_iid=3102647_0     ATTACAGGAAAAATCAAGCCTAATATACCAAAAAGTATAAAATTTCCACCCTCAGAAATT   2062
m141108_iid=1811866_0     ATTACAGGAAAAATCAAGCCTAATATACCAAAAAGTATAAAATTTCCACCCTCAGAAATT   3940
m150501_iid=2948495_0     ATTACAGGAAAAATCAAGCCTAATATACCAAAAAGTATAAAATTTCCACCCTCAGAAATT   3364
m150504_iid=3238195_0     ATTACAGGAAAAATCAAGCCTAATATACCAAAAAGTATAAAATTTCCACCCTCAGAAATT   5696
m150503_iid=3388511_0     ATTACAGGAAAAATCAAGCCTAATATACCAAAAAGTATAAAATTTCCACCCTCAGAAATT   3396
m150505_iid=3777611_0     ATTACAGGAAAAATCAAGCCTAATATACCAAAAAGTATAAAATTTCCACCCTCAGAAATT   4813
m150430_iid=2438833_0     ATTACAGGAAAAATCAAGCCTAATATACCAAAAAGTATAAAATTTCCACCCTCAGAAATT   5152
m141108_iid=1698969_0     ATTACAGGAAAAATCAAGCCTAATATACCAAAAAGTATAAAATTTCCACCCTCAGAAATT   6279
m150430_iid=3059190_0     ATTACAGGAAAAATCAAGCCTAATATACCAAAAAGTATAAAATTTCCACCCTCAGAAATT   2000
m141107_iid=1383067_0     ATTACAGGAAAAATCAAGCCTAATATACCAAAAAGTATAAAATTTCCACCCTCAGAAATT   2007
m141101_iid=597008_0      ------------------------------------------------------------   0
m150506_iid=2596496_0     ATTACAGGAAAAATCAAGCCTAATATACCAAAAAGTATAAAATTTCCACCCTCAGAAATT   6638
m141102_iid=910051_0      ATTACAGGAAAAATCAAGCCTAATATACCAAAAAGTATAAAATTTCCACCCTCAGAAATT   6125
m150505_iid=3784017_0     ATTACAGGAAAAATCAAGCCTAATATACCAAAAAGTATAAAATTTCCACCCTCAGAAATT   4926
m141101_iid=682151_0      ATTACAGGAAAAATCAAGCCTAATATACCAAAAAGTATAAAATTTCCACCCTCAGAAATT   3995
m150501_iid=3748029_0     ATTACAGGAAAAATCAAGCCTAATATACCAAAAAGTATAAAATTTCCACCCTCAGAAATT   4538
m150504_iid=2876272_0     ATTACAGGAAAAATCAAGCCTAATATACCAAAAAGTATAAAATTTCCACCCTCAGAAATT   6402
NC_042820.1               ATTACAGGAAAAATCAAGCCTAATATACCAAAAAGTATAAAATTTCCACCCTCAGAAATT   6645
m141102_iid=1228825_0     ATTACAGGAAAAATCAAGCCTAATATACCAAAAAGTATAAAATTTCCACCCTCAGAAATT   6471
m150504_iid=2876274_0     ATTACAGGAAAAATCAAGCCTAATATACCAAAAAGTATAAAATTTCCACCCTCAGAAATT   6474
m141030_iid=80036_0       ATTACAGGAAAAATCAAGCCTAATATACCAAAAAGTATAAAATTTCCACCCTCAGAAATT   6235
m150506_iid=2596494_0     ATTACAGGAAAAATCAAGCCTAATATACCAAAAAGTATAAAATTTCCACCCTCAGAAATT   6640
m150503_iid=2526862_0     ATTACAGGAAAAATCAAGCCTAATATACCAAAAAGTATAAAATTTCCACCCTCAGAAATT   6324
m150506_iid=2567000_0     ATTACAGGAAAAATCAAGCCTAATATACCAAAAAGTATAAAATTTCCACCCTCAGAAATT   4273
m150507_iid=3073271_0     ATTACAGGAAAAATCAAGCCTAATATACCAAAAAGTATAAAATTTCCACCCTCAGAAATT   5577
m150504_iid=3156087_0     ATTACAGGAAAAATCAAGCCTAATATACCAAAAAGTATAAAATTTCCACCCTCAGAAATT   5400
m150505_iid=2498502_0     ATTACAGGAAAAATCAAGCCTAATATACCAAAAAGTATAAAATTTCCACCCTCAGAAATT   3050
m141102_iid=754227_0      ATTACAGGAAAAATCAAGCCTAATATACCAAAAAGTATAAAATTTCCACCCTCAGAAATT   5612
m150507_iid=3469514_0     ATTACAGGAAAAATCAAGCCTAATATACCAAAAAGTATAAAATTTCCACCCTCAGAAATT   6170
Rdo_MitoGenome            ATTACAGGAAAAATCAAGCCTAATATACCAAAAAGTATAAAATTTCCACCCTCAGAAATT   6645
m141101_iid=614933_0      ATTACAGGAAAAATCAAGCCTAATATACCAAAAAGTATAAAATTTCCACCCTCAGAAATT   5197
```

nad5

```
m141031_iid=347546_0      AAGAATAATGGAGAAAATTT-AAATAACCAA-AAATAAATAATATATTAAACGTACAGTA   1431
m150506_iid=3127414_0     AAGAATAATGGAGAAAAATTTAAATAACCAAAAAATAAATAATATATTAAACGTACAGTA   3165
m141102_iid=1245517_0     AAGAATAATGGAGAAAAATTTAAATAACCAAAAA-TAAATAATATATTAAACGTACAGTA   4821
m141107_iid=1405727_0     AAGAATAATGGAGAAAAATTTAAATAACCAAAAA-TAAATAATATATTAAACGTACAGTA   2003
m141102_iid=987614_0      AAGAATAATGGAGAAAAATTTAAATAACCAAAAAATAAATAATATATTAAACGTACAGTA   4979
m141108_iid=1701429_0     AAGAATAATGGAGAAAAATTTAAATAACCAAAAAATAAATAATATATTAAACGTACAGTA   1716
m141102_iid=987286_0      AAGAATAATGGAGAAAAATTTAAATAACCAAAAAATAAATAATATATTAAACGTACAGTA   2931
m150430_iid=3272125_0     AAGAATAATGGAGAAAAATTTAAATAACCAAAAAATAAATAATATATTAAACGTACAGTA   4452
m141102_iid=754225_0      AAGAATAATGGAGAAAAATTTAAATAACCAAAAAA-AAATAATATATTAAACGTACAGTA   5617
m141107_iid=1409934_0     AAGAATAATGGAGAAAAATTTAAATAACCAAAAAATAAATAATATATTAAACGTACAGTA   6163
m150503_iid=3416920_0     AAGAATAATGGAGAAAAATTTAAATAACCAAAAAATAAATAATATATTAAACGTACAGTA   4881
m141108_iid=1800121_0     AAGAATAATGGAGAAAAATTTAAATAACCAAAAAATAAATAATATATTAAACGTACAGTA   4692
m141031_iid=147540_0      AAGAATAATGGAGAAAAATTTAAATAACCAAAAAATAAATAATATATTAAACGTACAGTA   5037
m150507_iid=3674924_0     AAGAATAATGGAGAAAAATTTAAATAACCAAAAAATAAATAATATATTAAACGTACAGTA   5188
m150503_iid=2923751_0     AAGAATAATGGAGAAAAATTTAAATAACCAAAAAATAAATAATATATTAAACGTACAGTA   5031
m141031_iid=115118_0      AAGAATAATGGAGAAAAATTTAAATAACCAAAAAATAAATAATATATTAAACGTACAGTA   3881
m150430_iid=2722448_0     AAGAATAATGGAGAAAAATTTAAATAACCAAAAAATAAATAATATATTAAACGTACAGTA   3817
m150507_iid=3102647_0     AAGAATAATGGAGAAAAATTTAAATAACCAAAAAATAAATAATATATTAAACGTACAGTA   2122
m141108_iid=1811866_0     AAGAATAATGGAGAAAAATTTAAATAACCAAAAAATAAATAATATATTAAACGTACAGTA   4000
m150501_iid=2948495_0     AAGAATAATGGAGAAAAATTTAAATAACCAAAAA-TAAATAATATATTAAACGTACAGTA   3423
m150504_iid=3238195_0     AAGAATAATGGAGAAAAATTTAAATAACCAAAAAATAAATAATATATTAAACGTACAGTA   5756
m150503_iid=3388511_0     AAGAATAATGGAGAAAAATTTAAATAACCAAAAAATAAATAATATATTAAACGTACAGTA   3456
m150505_iid=3777611_0     AAGAATAATGGAGAAAAATTTAAATAACCAAAAAATAAATAATATATTAAACGTACAGTA   4873
m150430_iid=2438833_0     AAGAATAATGGAGAAAAATTTAAATAACCAAAAAATAAATAATATATTAAACGTACAGTA   5212
m141108_iid=1698969_0     AAGAATAATGGAGAAAAATTTAAATAACCAAAAAATAAATAATATATTAAACGTACAGTA   6339
m150430_iid=3059190_0     AAGAATAATGGAGAAAAATTTAAATAACCAAAAAATAAATAATATATTAAACGTACAGTA   2060
m141107_iid=1383067_0     AAGAATAATGGAGAAAAATTTAAATAACCAAAAAATAAATAATATATTAAACGTACAGTA   2067
```

```
m141101_iid=597008_0        ------------------------------------------------------------   0
m150506_iid=2596496_0       AAGAATAATGGAGAAAAATTTAAATAACCAAAAAATAAATAATATATTAAACGTACAGTA   6698
m141102_iid=910051_0        AAGAATAATGGAGAAAAATTTAAATAACCAAAAAATAAATAATATATTAAACGTACAGTA   6185
m150505_iid=3784017_0       AAGAATAATGGAGAAAAATTTAAATAACCAAAAAATAAATAATATATTAAACGTACAGTA   4986
m141101_iid=682151_0        AAGAATAATGGAGAAAAATTTAAATAACCAAAAAATAAATAATATATTAAACGTACAGTA   4055
m150501_iid=3748029_0       AAGAATAATGGAGAAAAATTTAAATAACCAAAAAATAAATAATATATTAAACGTACAGTA   4598
m150504_iid=2876272_0       AAGAATAATGGAGAAAAATTTAAATAACCAAAAAATAAATAATATATTAAACGTACAGTA   6462
NC_042820.1                 AAGAATAATGGAGAAAAATTTAAATAACCAAAAAATAAATAATATATTAAACGTACAGTA   6705
m141102_iid=1228825_0       AAGAATAATGGAGAAAAATTTAAATAACCAAAAAATAAATAATATATTAAACGTACAGTA   6531
m150504_iid=2876274_0       AAGAATAATGGAGAAAAATTTAAATAACCAAAAAATAAATAATATATTAAACGTACAGTA   6534
m141030_iid=80036_0         AAGAATAATGGAGAAAAATTTAAATAACCAAAAAATAAATAATATATTAAACGTACAGTA   6295
m150506_iid=2596494_0       AAGAATAATGGAGAAAAATTTAAATAACCAAAAAATAAATAATATATTAAACGTACAGTA   6700
m150503_iid=2526862_0       AAGAATAATGGAGAAAAATTTAAATAACCAAAAAATAAATAATATATTAAACGTACAGTA   6384
m150506_iid=2567000_0       AAGAATAATGGAGAAAAATTTAAATAACCAAAAAATAAATAATATATTAAACGTACAGTA   4333
m150507_iid=3073271_0       AAGAATAATGGAGAAAAATTTAAATAACCAAAAAATAAATAATATATTAAACGTACAGTA   5637
m150504_iid=3156087_0       AAGAATAATGGAGAAAAATTTAAATAACCAAAAAATAAATAATATATTAAACGTACAGTA   5460
m150505_iid=2498502_0       AAGAATAATGGAGAAAAATTTAAATAACCAAAAAATAAATAATATATTAAACGTACAGTA   3110
m141102_iid=754227_0        AAGAATAATGGAGAAAAATTTAAATAACCAAAAAATAAATAATATATTAAACGTACAGTA   5672
m150507_iid=3469514_0       AAGAATAATGGAGAAAAATTTAAATAACCAAAAAATAAATAATATATTAAACGTACAGTA   6230
Rdo_MitoGenome              AAGAATAATGGAGAAAAATTTAAATAACCAAAAAATAAATAATATATTAAACGTACAGTA   6705
m141101_iid=614933_0        AAGAATAATGGAGAAAAATTTAAATAACCAAAAAAT-AATAATATATTAAACGTACAGTA   5256
```

nad5

```
m141031_iid=347546_0        TAAGAAACTGTTAAACCTGTAGAGACAAAAAATAAAATATAAGAAAACATATTAACAAAT   1491
m150506_iid=3127414_0       TAAGAAACTGTTAAACCTGTAGAGACAAAAAATAAAATATAAGAAAACATATTAACAAAT   3225
m141102_iid=1245517_0       TAAGAAACTGTTAAACCTGTAGAGACAAAAAATAAAATATAAGAAAACATATTAACAAAT   4881
m141107_iid=1405727_0       TAAGAAACTGTTAA-ACTGTAGAGACAAAAAATAAAATATAAGAAAACATATTAACAAAT   2062
m141102_iid=987614_0        TAAGAAACTGTTAAACCTGTAGAGACAAAAAATAAAATATAAGAAAACATATTAACAAAT   5039
m141108_iid=1701429_0       TAAGAAACTGTTAAACCTGTAGAGACAAAAAATAAAATATAAGAAAACATATTAACAAAT   1776
m141102_iid=987286_0        TAAGAAACTGTTAAACCTGTAGAGACAAAAAATAAAATATAAGAAAACATATTAACAAAT   2991
m150430_iid=3272125_0       TAAGAAACTGTTAAACCTGTAGAGACAAAAAATAAAATATAAGAAAACATATTAACAAAT   4512
m141102_iid=754225_0        TAAGAAACTGTTAAACCTGTAGAGACAAAAAATAAAATATAAGAAAACATATTAACAAAT   5677
m141107_iid=1409934_0       TAAGAAACTGTTAAACCTGTAGAGACAAAAAATAAAATATAAGAAAACATATTAACAAAT   6223
m150503_iid=3416920_0       TAAGAAACTGTTAAACCTGTAGAGACAAAAAATAAAATATAAGAAAACATATTAACAAAT   4941
m141108_iid=1800121_0       TAAGAAACTGTTAAACCTGTAGAGACAAAAAATAAAATATAAGAAAACATATTAACAAAT   4752
m141031_iid=147540_0        TAAGAAACTGTTAAACCTGTAGAGACAAAAAATAAAATATAAGAAAACATATTAACAAAT   5097
m150507_iid=3674924_0       TAAGAAACTGTTAAACCTGTAGAGACAAAAAATAAAATATAAGAAAACATATTAACAAAT   5248
m150503_iid=2923751_0       TAAGAAACTGTTAAACCTGTAGAGACAAAAAT-AAAATATAAGAAAACATATTAACAAAT   5090
m141031_iid=115118_0        TAAGAAACTGTTAAACCTGTAGAGACAAAAAATAAAATATAAGAAAACATATTAACAAAT   3941
m150430_iid=2722448_0       TAAGAAACTGTTAAACCTGTAGAGACAAAAAATAAAATATAAGAAAACATATTAACAAAT   3877
m150507_iid=3102647_0       TAAGAAACTGTTAAACCTGTAGAGACAAAAAATAAAATATAAGAAAACATATTAACAAAT   2182
m141108_iid=1811866_0       TAAGAAACTGTTAAACCTGTAGAGACAAAAAATAAAATATAAGAAAACATATTAACAAAT   4060
m150501_iid=2948495_0       TAAGAAACTGTTAAACCTGTAGAGACAAAAAATAAAATATAAGAAAACATATTAACAAAT   3483
m150504_iid=3238195_0       TAAGAAACTGTTAAACCTGTAGAGACAAAAAATAAAATATAAGAAAACATATTAACAAAT   5816
m150503_iid=3388511_0       TAAGAAACTGTTAAACCTGTAGAGAC-AAAAATAAAATATAAGAAAACATATTAACAAAT   3515
m150505_iid=3777611_0       TAAGAAACTGTTAAACCTGTAGAGACAAAAAATAAAATATAAGAAAACATATTAACAAAT   4933
m150430_iid=2438833_0       TAAGAAACTGTTAAACCTGTAGAGACAAAAAATAAAATATAAGAAAACATATTAACAAAT   5272
m141108_iid=1698969_0       TAAGAAACTGTTAAACCTGTAGAGACAAAAAATAAAATATAAGAAAACATATTAACAAAT   6399
m150430_iid=3059190_0       TAAGAAACTGTTAAACCTGTAGAGACAAAAAATAAAATATAAGAAAACATATTAACAAAT   2120
m141107_iid=1383067_0       TAAGAAACTGTTAAACCTGTAGAGACAAAAAATAAAATATAAGAAAACATATTAACAAAT   2127
m141101_iid=597008_0        ------------------------------------------------------------   0
m150506_iid=2596496_0       TAAGAAACTGTTAAACCTGTAGAGACAAAAAATAAAATATAAGAAAACATATTAACAAAT   6758
m141102_iid=910051_0        TAAGAAACTGTTAAACCTGTAGAGACAAAAAATAAAATATAAGAAAACATATTAACAAAT   6245
m150505_iid=3784017_0       TAAGAAACTGTTAAACCTGTAGAGACAAAAAATAAAATATAAGAAAACATATTAACAAAT   5046
m141101_iid=682151_0        TAAGAAACTGTTAAACCTGTAGAGACAAAAAATAAAATATAAGAAAACATATTAACAAAT   4115
m150501_iid=3748029_0       TAAGAAACTGTTAAACCTGTAGAGACAAAAAATAAAATATAAGAAAACATATTAACAAAT   4658
m150504_iid=2876272_0       TAAGAAACTGTTAAACCTGTAGAGACAAAAAATAAAATATAAGAAAACATATTAACAAAT   6522
NC_042820.1                 TAAGAAACTGTTAAACCTGTAGAGACAAAAAATAAAATATAAGAAAACATATTAACAAAT   6765
m141102_iid=1228825_0       TAAGAAACTGTTAAACCTGTAGAGACAAAAAATAAAATATAAGAAAACATATTAACAAAT   6591
m150504_iid=2876274_0       TAAGAAACTGTTAAACCTGTAGAGACAAAAAATAAAATATAAGAAAACATATTAACAAAT   6594
m141030_iid=80036_0         TAAGAAACTGTTAAACCTGTAGAGACAAAAAATAAAATATAAGAAAACATATTAACAAAT   6355
m150506_iid=2596494_0       TAAGAAACTGTTAAACCTGTAGAGACAAAAAATAAAATATAAGAAAACATATTAACAAAT   6760
m150503_iid=2526862_0       TAAGAAACTGTTAAACCTGTAGAGACAAAAAATAAAATATAAGAAAACATATTAACAAAT   6444
m150506_iid=2567000_0       TAAGAAACTGTTAAACCTGTAGAGACAAAAAATAAAATATAAGAAAACATATTAACAAAT   4393
m150507_iid=3073271_0       TAAGAAACTGTTAAACCTGTAGAGACAAAAAATAAAATATAAGAAAACATATTAACAAAT   5697
m150504_iid=3156087_0       TAAGAAACTGTTAAACCTGTAGAGACAAAAAATAAAATATAAGAAAACATATTAACAAAT   5520
m150505_iid=2498502_0       TAAGAAACTGTTAAACCTGTAGAGACAAAAAATAAAATATAAGAAAACATATTAACAAAT   3170
m141102_iid=754227_0        TAAGAAACTGTTAAACCTGTAGAGACAAAAAATAAAATATAAGAAAACATATTAACAAAT   5732
m150507_iid=3469514_0       TAAGAAACTGTTAAACCTGTAGAGACAAAAAATAAAATATAAGAAAACATATTAACAAAT   6290
Rdo_MitoGenome              TAAGAAACTGTTAAACCTGTAGAGACAAAAAATAAAATATAAGAAAACATATTAACAAAT   6765
m141101_iid=614933_0        TAAGAAACTGTTAAACCTGTAGAGACAAAAAATAAAATATAAGAAAACATATTAACAAAT   5316
```

nad5

```
m141031_iid=347546_0       TTTATTGAATAAAACTCCAAAATAAA-TCCTTTGAATAAAAACCTGAAAGAAAAGGT-AA   1549
m150506_iid=3127414_0      TTTATTGAATAAAACTCCAAAATTAAATCCTTTGA-ATAAAACCTGAAAAAAAGGTA-AA   3283
m141102_iid=1245517_0      TTTATTGAATAAAACTCCAAAATTAAATCCTTTGAATAAAAACCTGAAAGAAAAGGTAAA   4941
m141107_iid=1405727_0      TTTATTGAATAAAACTCCAAAATTAAATCCTTTGAATAAAAACCTGAAAGAA-AGGTAAA   2121
m141102_iid=987614_0       TTTATTGAATAAAACTCCAAAATTAAATCCTTTGAATAAAAACCTGAAAGAAAAGGTAAA   5099
m141108_iid=1701429_0      TTTATTGAATAAAACTCCAAAATTAAATCCTTTGAATAAAAACCTGAAAGAAAAGGTAAA   1836
m141102_iid=987286_0       TTTATTGAATAAAACTCCAAAATTAAATCCTTTGAATAAAAACCTGAAAGAAAAGGTAAA   3051
m150430_iid=3272125_0      TTTATTGAATAAAACTCCAAAATTAAATCCTTTGAATAAAAACCTGAAAGAAAAGGTAAA   4572
m141102_iid=754225_0       TTTATTGAATAAAACTCCAAAATTAAATCCTTTGAATAAAAACCTGAAAGAAAAGGTAAA   5737
m141107_iid=1409934_0      TTTATTGAATAAAACTCCAAAATTAAATCCTTTGAATAAAAACCTGAAAGAAAAGGTAAA   6283
m150503_iid=3416920_0      TTTATTGAATAAAACTCCAAAATTAAATCCTTTGAATAAAAACCTGAAAGAAAAGGTAAA   5001
m141108_iid=1800121_0      TTTATTGAATAAAACTCCAAAATTAAATCCTTTGAATAAAAACCTGAAAGAAAAGGTAAA   4812
m141031_iid=147540_0       TTTATTGAATAAAACTCCAAAATTAAATCCTTTGAATAAAAACCTGAAAGAAAAGGTAAA   5157
m150507_iid=3674924_0      TTTATTGAATAAAACTCCAAAATTAAATCCTTTGAATAAAAACCTGAAAGAAAAGGTAAA   5308
m150503_iid=2923751_0      TTTATTGAATAAAACTCCAAAATTAAATCCTTTGAATAAAAACCTGAAAGAAAAGGTAAA   5150
m141031_iid=115118_0       TTTATTGAATAAAACTCCAAAATTAAATCCTTTGAATAAAAACCTGAAAGAAAAGGTAAA   4001
m150430_iid=2722448_0      TTTATTGAATAAAACTCCAAAATTAAATCCTTTGAATAAAAACCTGAAAGAAAAGGTAAA   3937
m150507_iid=3102647_0      TTTATTGAATAAAACTCCAAAATTAAATCCTTTGAATAAAAACCTGAAAGAAAAGGTAAA   2242
m141108_iid=1811866_0      TTTATTGAATAAAACTCCAAAATTAAATCCTTTGAATAAAAACCTGAAAGAAAAGGTAAA   4120
m150501_iid=2948495_0      TTTATTGAATAAAACTCCAAAATTAAATCCTTTGAATA-AAACCTGAAAGAAAAGGTAAA   3542
m150504_iid=3238195_0      TTTATTGAATAAAACTCCAAAATTAAATCCTTTGAATAAAAACCTGAAAGAAAAGGTAAA   5876
m150503_iid=3388511_0      TTTATTGAATAAAACTCCAAAATTAAATCCTTTGAATAAAAACCTGAAAGAAAAGGTAAA   3575
m150505_iid=3777611_0      TTTATTGAATAAAACTCCAAAATTAAATCCTTTGAATAAAAACCTGAAAGAAAAGGTAAA   4993
m150430_iid=2438833_0      TTTATTGAATAAAACTCCAAAATTAAATCCTTTGAATAAAAACCTGAAAGAAAAGGTAAA   5332
m141108_iid=1698969_0      TTTATTGAATAAAACTCCAAAATTAAATCCTTTGAATAAAAACCTGAAAGAAAAGGTAAA   6459
m150430_iid=3059190_0      TTTATTGAATAAAACTCCAAAATTAAATCCTTTGAATAAAAACCTGAAAGAAAAGGTAAA   2180
m141107_iid=1383067_0      TTTATTGAATAAAACTCCAAAATTAAATCCTTTGAATAAAAACCTGAAAGAAAAGGTAAA   2187
m141101_iid=597008_0       ------------------------------------------------------------   0
m150506_iid=2596496_0      TTTATTGAATAAAACTCCAAAATTAAATCCTTTGAATAAAAACCTGAAAGAAAAGGTAAA   6818
m141102_iid=910051_0       TTTATTGAATAAAACTCCAAAATTAAATCCTTTGAATAAAAACCTGAAAGAAAAGGTAAA   6305
m150505_iid=3784017_0      TTTATTGAATAAAACTCCAAAATTAAATCCTTTGAATAAAAACCTGAAAGAAAAGGTAAA   5106
m141101_iid=682151_0       TTTATTGAATAAAACTCCAAAATTAAATCCTTTGAATAAAAACCTGAAAGAAAAGGTAAA   4175
m150501_iid=3748029_0      TTTATTGAATAAAACTCCAAAATTAAATCCTTTGAATAAAAACCTGAAAGAAAAGGTAAA   4718
m150504_iid=2876272_0      TTTATTGAATAAAACTCCAAAATTAAATCCTTTGAATAAAAACCTGAAAGAAAAGGTAAA   6582
NC_042820.1                TTTATTGAATAAAACTCCGAAATTAAATCCTTTGAATAAAAACCTGAAAGAAAAGGTAAA   6825
m141102_iid=1228825_0      TTTATTGAATAAAACTCCAAAATTAAATCCTTTGAATAAAAACCTGAAAGAAAAGGTAAA   6651
m150504_iid=2876274_0      TTTATTGAATAAAACTCCAAAATTAAATCCTTTGAATAAAAACCTGAAAGAAAAGGTAAA   6654
m141030_iid=80036_0        TTTATTGAATAAAACTCCAAAATTAAATCCTTTGAATAAAAACCTGAAAGAAAAGGTAAA   6415
m150506_iid=2596494_0      TTTATTGAATAAAACTCCAAAATTAAATCCTTTGAATAAAAACCTGAAAGAAAAGGTAAA   6820
m150503_iid=2526862_0      TTTATTGAATAAAACTCCAAAATTAAATCCTTTGAATAAAAACCTGAAAGAAAAGGTAAA   6504
m150506_iid=2567000_0      TTTATTGAATAAAACTCCAAAATTAAATCCTTTGAATAAAAACCTGAAAGAAAAGGTAAA   4453
m150507_iid=3073271_0      TTTATTGAATAAAACTCCAAAATTAAATCCTTTGAATAAAAACCTGAAAGAAAAGGTAAA   5757
m150504_iid=3156087_0      TTTATTGAATAAAACTCCAAAATTAAATCCTTTGAATAAAAACCTGAAAGAAAAGGTAAA   5580
m150505_iid=2498502_0      TTTATTGAATAAAACTCCAAAATTAAATCCTTTGAATAAAAACCTGAAAGAAAAGGTAAA   3230
m141102_iid=754227_0       TTTATTGAATAAAACTCCAAAATTAAATCCTTTGAATAAAAACCTGAAAGAAAAGGTAAA   5792
m150507_iid=3469514_0      TTTATTGAATAAAACTCCAAAATTAAATCCTTTGAATAAAAACCTGAAAGAAAAGGTAAA   6350
Rdo_MitoGenome             TTTATTGAATAAAACTCCAAAATTAAATCCTTTGAATAAAAACCTGAAAGAAAAGGTAAA   6825
m141101_iid=614933_0       TTTATTGAATAAAACTCCAAAATTAAATCCTTTGAATAAAAACCTGAAAGAAAAGGTAAA   5376
```

nad5

```
m141031_iid=347546_0       ACACCAAGAGAAAAATTACAAATAATAAAAAAACAAGAAGTTAAAGGTATATAAGTTTAA   1609
m150506_iid=3127414_0      CCACAAAGAGAAAAATTACAAATAATAAAAAAACAAGAAGTTAAAGGTATA-AAGTTTAA   3342
m141102_iid=1245517_0      CCACAAAGAGAAAAT-TACAAATAATAAAAAAACAAGAAGTTAAAGGTATA-AAGTTTAA   4999
m141107_iid=1405727_0      CCACAAAGAGAAAATT-ACAAATAAT--AAAAAAAAGAAGTTAAAGGTATA-AAGTTTAA   2177
m141102_iid=987614_0       CCACAAAGAGAAAAATTACAAATAATAAAAAAACAAGAAGTTAAAGGTATA-AAGTTTAA   5158
m141108_iid=1701429_0      CCACAAAGAGAAAAATTACAAATAATAAAAAAACAAGAAGTTAAAGGTATA-AAGTTTAA   1895
m141102_iid=987286_0       CCACAAAGAGAAAAATTACAAATAATAAAAAAACAAGAAGTTAAAGGTATA-AAGTTTAA   3110
m150430_iid=3272125_0      CCACAAAGAGAAAAATTACAAATAATAAAAAAACAAGAAGTTAAAGGTATA-AAGTTTAA   4631
m141102_iid=754225_0       CCACAAAGAGAAAAATTACAAATAATAAAAAAACAAGAAGTTAAAGGTATA-AAGTTTAA   5796
m141107_iid=1409934_0      CCACAAAGAGAAAAATTACAAATAATAAA-AAAAAAGAAGTTAAAGGTATA-AAGTTTAA   6341
m150503_iid=3416920_0      CCACAAAGAGAAAAATTACAAATAATAAAAAAACAAGAAGTTAAAGGTATA-AAGTTTAA   5060
m141108_iid=1800121_0      CCACAAAGAGAAAAATTACAAATAATAAAAAAACAAGAAGTTAAAGGTATA-AAGTTTAA   4871
m141031_iid=147540_0       CCACAAAGAGAAAAATTACAAATAATAAAAAAACAAGAAGTTAAAGGTATA-AAGTTTAA   5216
m150507_iid=3674924_0      CCACAAAGAGAAAAATTACAAATAAT-AAAAAACAAGAAGTTAAAGGTATA-AAGTTTAA   5366
m150503_iid=2923751_0      -CACAAAGAGAAAAATTACAAATAAT-AAAAAACAAGAAGTTAAAGGTATA-AAGTTTAA   5207
m141031_iid=115118_0       CCACAAAGAGAAAAATTACAAATAATAAAAAAACAAGAAGTTAAAGGTATA-AAGTTTAA   4060
m150430_iid=2722448_0      CCACAAAGAGAAAAATTACAAATAATAAAAAAACAAGAAGTTAAAGGTATA-AAGTTTAA   3996
m150507_iid=3102647_0      CCACAAAGAGAAAAATTACAAATAATAAAAAAACAAGAAGTTAAAGGTATA-AAGTTTAA   2301
m141108_iid=1811866_0      CCACAAAGAGAAAAATTACAAATAATA-AAAAACAAGAAGTTAAAGGTATA-AAGTTTAA   4178
```

```
m150501_iid=2948495_0      CCACAAAGAGAAAAATTACAAATAATAAAAAAACAAGAAGTTAAAGGTATA-AAGTTTAA   3601
m150504_iid=3238195_0      CCACAAAGAGAAAATT-ACAAATA-ATAAAAAACAAGAAGTTAAAGGTATA-AAGTTTAA   5933
m150503_iid=3388511_0      CCACAAAGAGAAAAATTACAAATAATAAAAAAACAAGAAGTTAAAGGTATA-AAGTTTAA   3634
m150505_iid=3777611_0      CCACAAAGAGAAAAATTACAAATAATAAAAAAACAAGAAGTTAAAGGTATA-AAGTTTAA   5052
m150430_iid=2438833_0      CCACAAAGAGAAAAATTACAAATAATAAAAAAACAAGAAGTTAAAGGTATA-AAGTTTAA   5391
m141108_iid=1698969_0      CCACAAAGAGAAAAATTACAAATAATAAAAAAACAAGAAGTTAAAGGTATA-AAGTTTAA   6518
m150430_iid=3059190_0      CCACAAAGAGAAAAATTACAAATAATAAAAAAACAAGAAGTTAAAGGTATA-AAGTTTAA   2239
m141107_iid=1383067_0      CCACAAAGAGAAAAATTACAAATAATA-AAAAACAAGAAGTTAAAGGTATA-AAGTTTAA   2245
m141101_iid=597008_0       ------------------------------------------------------------   0
m150506_iid=2596496_0      CCACAAAGAGAAAAATTACAAATAATAAA-AAAAAAGAAGTTAAAGGTATA-AAGTTTAA   6876
m141102_iid=910051_0       CCACAAAGAGAAAAATTACAAATAATAAAAAAACAAGAAGTTAAAGGTATA-AAGTTTAA   6364
m150505_iid=3784017_0      CCACAAAGAGAAAAATTACAAATAATAAAAAAACAAGAAGTTAAAGGTATA-AAGTTTAA   5165
m141101_iid=682151_0       CCACAAAGAGAAAAATTACAAATAATAAAAAAACAAGAAGTTAAAGGTATA-AAGTTTAA   4234
m150501_iid=3748029_0      CCACAAAGAGAAAAATTACAAATAATAAAAAAACAAGAAGTTAAAGGTATA-AAGTTTAA   4777
m150504_iid=2876272_0      CCACAAAGAGAAAAATTACAAATAATAAAAAAACAAGAAGTTAAAGGTATA-AAGTTTAA   6641
NC_042820.1                CCACAAAGAGAAAAATTACAAATAATAAAAAAACAAGAAGTTAAAGGTATA-AAGTTTAA   6884
m141102_iid=1228825_0      CCACAAAGAGAAAAATTACAAATAATAAAAAAACAAGAAGTTAAAGGTATA-AAGTTTAA   6710
m150504_iid=2876274_0      CCACAAAGAGAAAAATTACAAATAATAAAAAAACAAGAAGTTAAAGGTATA-AAGTTTAA   6713
m141030_iid=80036_0        CCACAAAGAGAAAAATTACAAATAATAAAAAAACAAGAAGTTAAAGGTATA-AAGTTTAA   6474
m150506_iid=2596494_0      CCACAAAGAGAAAAATTACAAATAATAAAAAAACAAGAAGTTAAAGGTATA-AAGTTTAA   6879
m150503_iid=2526862_0      CCACAAAGAGAAAAATTACAAATAATAAAAAAA-AAGAAGTTAAAGGTATA-AAGTTTAA   6562
m150506_iid=2567000_0      CCACAAAGAGAAAAATTACAAATAATAAAAAAACAAGAAGTTAAAGGTATA-AAGTTTAA   4512
m150507_iid=3073271_0      CCACAAAGAGAAAAATTACAAATAATAAAAAAACAAGAAGTTAAAGGTATA-AAGTTTAA   5816
m150504_iid=3156087_0      CCACAAAGAGAAAAATTACAAATAATAAAAAAACAAGAAGTTAAAGGTATA-AAGTTTAA   5639
m150505_iid=2498502_0      CCACAAAGAGAAAAATTACAAATAATAAAAAAACAAGAAGTTAAAGGTATA-AAGTTTAA   3289
m141102_iid=754227_0       CCACAAAGAGAAAAATTACAAATAATAAAAAAACAAGAAGTTAAAGGTATA-AAGTTTAA   5851
m150507_iid=3469514_0      CCACAAAGAGAAAAATTACAAATAATAAAAAAACAAGAAGTTAAAGGTATA-AAGTTTAA   6409
Rdo_MitoGenome             CCACAAAGAGAAAAATTACAAATAATAAAAAAACAAGAAGTTAAAGGTATA-AAGTTTAA   6884
m141101_iid=614933_0       CCACAAAGAGAAAAATTACAAATAATAAAAAAACAAGAAGTTAAAGGTATA-AAGTTTAA   5435

                                                 nad5

m141031_iid=347546_0       AGCACTTCCTATAAAACGAATATCCTGAAAATTTATAAAATCTATGAATAAATGAACCAG   1669
m150506_iid=3127414_0      AGCACTTCCTATAAAACGAATATCCTGAAAATTTAT-AAATCTATGAATAAATGAACCAG   3401
m141102_iid=1245517_0      AGCACTTCCTATAAAACGAATATCCTGAAAATTTAT-AAATCTATGAATAAATGAA-CAG   5057
m141107_iid=1405727_0      AGCACTTCCTATAAAACGAATATCCTGAAAATTTAT-AAATCTATGAAT-AATGAACCAG   2235
m141102_iid=987614_0       AGCACTTCCTATAAAACGAATATCCTGAAAATTTAT-AAATCTATGAATAAATGAACCAG   5217
m141108_iid=1701429_0      AGCACTTCCTATAAAACGAATATCCTGAAAATTTAT-AAATCTATGAATAAATGAACCAG   1954
m141102_iid=987286_0       AGCACTTCCTATAAAACGAATATCCTGAAAATTTAT-AAATCTATGAATAAATGAACCAG   3169
m150430_iid=3272125_0      AGCACTTCCTATAAAACGAATATCCTGAAAATTTAT-AAATCTATGAATAAATGAACCAG   4690
m141102_iid=754225_0       AGCACTTCCTATAAAACGAATATCCTGAAAATTTAT-AAATCTATGAATAAATGAACCAG   5855
m141107_iid=1409934_0      AGCACTTCCTATAAAACGAATATCCTGAAAATTTAT-AAATCTATGAATAAATGAACCAG   6400
m150503_iid=3416920_0      AGCACTTCCTATAAAACGAATATCCTGAAAATTTAT-AAATCTATGAATAAATGAACCAG   5119
m141108_iid=1800121_0      AGCACTTCCTATAAAACGAATATCCTGAAAATTTAT-AAATCTATGAATAAATGAACCAG   4930
m141031_iid=147540_0       AGCACTTCCTATAAAACGAATATCCTGAAAATTTAT-AAATCTATGAATAAATGAACCAG   5275
m150507_iid=3674924_0      AGCACTTCCTATAAAACGAATATCCTGAAAATTTAT-AAATCTATGAATAAATGAACCAG   5425
m150503_iid=2923751_0      AGCACTTCCTATAAAACGAATATCCTGAAAATTTAT-AAATCTATGAATAAATGAACCAG   5266
m141031_iid=115118_0       AGCACTTCCTATAAAACGAATATCCTGAAAATTTAT-AAATCTATGAATAAATGAACCAG   4119
m150430_iid=2722448_0      AGCACTTCCTATAAAACGAATATCCTGAAAATTTAT-AAATCTATGAATAAATGAACCAG   4055
m150507_iid=3102647_0      AGCACTTCCTATAAAACGAATATCCTGAAAATTTAT-AAATCTATGAATAAATGAACCAG   2360
m141108_iid=1811866_0      AGCACTTCCTATAAAACGAATATCCTGAAAATTTAT-AAATCTATGAATAAATGAACCAG   4237
m150501_iid=2948495_0      AGCACTTCCTATAAAACGAATATCCTGAAAATTTAT-AAATCTATGAATAAATGAACCAG   3660
m150504_iid=3238195_0      AGCACTTCCTATAAAACGAATATCCTGAAAATTTAT-AAATCTATGAATAAATGAACCAG   5992
m150503_iid=3388511_0      AGCACTTCCTATAAAACGAATATCCTGAAAATTTAT-AAATCTATGAATAAATGAACCAG   3693
m150505_iid=3777611_0      AGCACTTCCTATAAAACGAATATCCTGAAAATTTAT-AAATCTATGAATAAATGAACCAG   5111
m150430_iid=2438833_0      AGCACTTCCTATAAAACGAATATCCTGAAAATTTAT-AAATCTATGAATAAATGAACCAG   5450
m141108_iid=1698969_0      AGCACTTCCTATAAAACGAATATCCTGAAAATTTAT-AAATCTATGAATAAATGAACCAG   6577
m150430_iid=3059190_0      AGCACTTCCTATAAAACGAATATCCTGAAAATTTAT-AAATCTATGAATAAATGAACCAG   2298
m141107_iid=1383067_0      AGCACTTCCTATAAAACGAATATCCTGAAAATTTAT-AAATCTATGAATAAATGAACCAG   2304
m141101_iid=597008_0       ------------------------------------------------------------   0
m150506_iid=2596496_0      AGCACTTCCTATAAAACGAATATCCTGAAAATTTAT-AAATCTATGAATAAATGAACCAG   6935
m141102_iid=910051_0       AGCACTTCCTATAAAACGAATATCCTGAAAATTTAT-AAATCTATGAATAAATGAACCAG   6423
m150505_iid=3784017_0      AGCACTTCCTATAAAACGAATATCCTGAAAATTTAT-AAATCTATGAATAAATGAACCAG   5224
m141101_iid=682151_0       AGCACTTCCTATAAAACGAATATCCTGAAAATTTAT-AAATCTATGAATAAATGAACCAG   4293
m150501_iid=3748029_0      AGCACTTCCTATAAAACGAATATCCTGAAAATTTAT-AAATCTATGAATAAATGAACCAG   4836
m150504_iid=2876272_0      AGCACTTCCTATAAAACGAATATCCTGAAAATTTAT-AAATCTATGAATAAATGAACCAG   6700
NC_042820.1                AGCACTTCCTATAAAACGAATATCCTGAAAATTTAT-AAATCTATGAATAAATGAACCAG   6943
m141102_iid=1228825_0      AGCACTTCCTATAAAACGAATATCCTGAAAATTTAT-AAATCTATGAATAAATGAACCAG   6769
m150504_iid=2876274_0      AGCACTTCCTATAAAACGAATATCCTGAAAATTTAT-AAATCTATGAATAAATGAACCAG   6772
m141030_iid=80036_0        AGCACTTCCTATAAAACGAATATCCTGAAAATTTAT-AAATCTATGAATAAATGAACCAG   6533
m150506_iid=2596494_0      AGCACTTCCTATAAAACGAATATCCTGAAAATTTAT-AAATCTATGAATAAATGAACCAG   6938
m150503_iid=2526862_0      AGCACTTCCTATAAAACGAATATCCTGAAAATTTAT-AAATCTATGAATAAATGAACCAG   6621
```

```
m150506_iid=2567000_0      AGCACTTCCTATAAAACGAATATCCTGAAAATTTAT-AAATCTATGAATAAATGAACCAG   4571
m150507_iid=3073271_0      AGCACTTCCTATAAAACGAATATCCTGAAAATTTAT-AAATCTATGAATAAATGAACCAG   5875
m150504_iid=3156087_0      AGCACTTCCTATAAAACGAATATCCTGAAAATTTAT-AAATCTATGAATAAATGAACCAG   5698
m150505_iid=2498502_0      AGCACTTCCTATAAAACGAATATCCTGAAAATTTAT-AAATCTATGAATAAATGAACCAG   3348
m141102_iid=754227_0       AGCACTTCCTATAAAACGAATATCCTGAAAATTTAT-AAATCTATGAATAAATGAACCAG   5910
m150507_iid=3469514_0      AGCACTTCCTATAAAACGAATATCCTGAAAATTTAT-AAATCTATGAATAAATGAACCAG   6468
Rdo_MitoGenome             AGCACTTCCTATAAAACGAATATCCTGAAAATTTAT-AAATCTATGAATAAATGAACCAG   6943
m141101_iid=614933_0       AGCACTTCCTATAAAACGAATATCCTGAAAATTTAT-AAATCTATGAATAAATGAACCAG   5494

                                                 nad5

m141031_iid=347546_0       CACATATAAACAATAAAGCCTTGAAAAGAGCATGAGATAAAAGATGAAAAAAAGCTAAAT   1729
m150506_iid=3127414_0      CACATATAAACAATAAAGCCTTGAAAAGAGCATGAGATAAAAGATGAAAAAAAGCTAAAT   3461
m141102_iid=1245517_0      CACATATAAACA-TAAAGCCTTGAAAAGAGCATGAGATAAAAGATGAAA-AAAGCTAAAT   5115
m141107_iid=1405727_0      CACATATAAACAAT-AAGCCTTGAAAAGAGCATGAGATAAAAGATGAA--AAAGCTAAAT   2292
m141102_iid=987614_0       CACATATAAACAATAAAGCCTTGAAAAGAGCATGAGATAAAAGATGAAAAAAAGCTAAAT   5277
m141108_iid=1701429_0      CACATATAAACAATAAAGCCTTGAAAAGAGCATGAGATAAAAGATGAAAAAAAGCTAAAT   2014
m141102_iid=987286_0       CACATATAAACAATAAAGCCTTGAAAAGAGCATGAGATAAAAGATGAAAAAAAGCTAAAT   3229
m150430_iid=3272125_0      CACATATAAACAATAAAGCCTTGAAAAGAGCATGAGATAAAAGATGAAAAAAAGCTAAAT   4750
m141102_iid=754225_0       CACATATAAACAATAAAGCCTTGAAAAGAGCATGAGATAAAAGATGAAAAAAAGCTAAAT   5915
m141107_iid=1409934_0      CACATATAAACAATAAAGCCTTGAAAAGAGCATGAGATAAAAGATGAAAAAAAGCTAAAT   6460
m150503_iid=3416920_0      CACATATAAACAATAAAGCCTTGAAAAGAGCATGAGATAAAAGATGAAAAAAAGCTAAAT   5179
m141108_iid=1800121_0      CACATATAAACAATAAAGCCTTGAAAAGAGCATGAGATAAAAGATGAAAAAAAGCTAAAT   4990
m141031_iid=147540_0       CACATATAAACAATAAAGCCTTGAAAAGAGCATGAGATAAAAGATGAAAAAAAGCTAAAT   5335
m150507_iid=3674924_0      CACATATAAACAATAAAGCCTTGAAAAGAGCATGAGATAAAAGATGAAAAAAAGCTAAAT   5485
m150503_iid=2923751_0      CACATAT-AACAATAAAGCCTTGAAAAGAGCATGAGATAAAAGATGAAAAAAAGCTAAAT   5325
m141031_iid=115118_0       CACATATAAACAATAAAGCCTTGAAAAGAGCATGAGATAAAAGATGAAAAAAAGCTAAAT   4179
m150430_iid=2722448_0      CACATATAAACAATAAAGCCTTGAAAAGAGCATGAGATAAAAGATGAAAAAAAGCTAAAT   4115
m150507_iid=3102647_0      CACATATAAACAATAAAGCCTTGAAAAGAGCATGAGATAAAAGATGAAAAAAAGCTAAAT   2420
m141108_iid=1811866_0      CACATATAAACAATAAAGCCTTGAAAAGAGCATGAGATAAAAGATGAAAAAAAGCTAAAT   4297
m150501_iid=2948495_0      CACATATAAACAATAAAGCCTTGAAAAGAGCATGAGATAAAAGATGAAAAAAAGCTAAAT   3720
m150504_iid=3238195_0      CACATATAAACAATAAAGCCTTGAAAAGAGCATGAGATAAAAGATGAAAAAAAGCTAAAT   6052
m150503_iid=3388511_0      CACATATAAACAATAAAGCCTTGAAAAGAGCATGAGATAAAAGATGAAAAAAAGCTAAAT   3753
m150505_iid=3777611_0      CACATATAAACAATAAAGCCTTGAAAAGAGCATGAGATAAAAGATGAAAAAAAGCTAAAT   5171
m150430_iid=2438833_0      CACATATAAACAATAAAGCCTTGAAAAGAGCATGAGATAAAAGATGAAAAAAAGCTAAAT   5510
m141108_iid=1698969_0      CACATATAAACAATAAAGCCTTGAAAAGAGCATGAGATAAAAGATGAAAAAAAGCTAAAT   6637
m150430_iid=3059190_0      CACATATAAACAATAAAGCCTTGAAAAGAGCATGAGATAAAAGATGAAAAAAAGCTAAAT   2358
m141107_iid=1383067_0      CACATATAAACAATAAAGCCTTGAAAAGAGCATGAGATAAAAGATGAAAAAAAGCTAAAT   2364
m141101_iid=597008_0       ------------------------------------------------------------   0
m150506_iid=2596496_0      CACATATAAACAATAAAGCCTTGAAAAGAGCATGAGATAAAAGATGAAAAAAAGCTAAAT   6995
m141102_iid=910051_0       CACATATAAACAATAAAGCCTTGAAAAGAGCATGAGATAAAAGATGAAAAAAAGCTAAAT   6483
m150505_iid=3784017_0      CACATATAAACAATAAAGCCTTGAAAAGAGCATGAGATAAAAGATGAAAAAAAGCTAAAT   5284
m141101_iid=682151_0       CACATATAAACAATAAAGCCTTGAAAAGAGCATGAGATAAAAGATGAAAAAAAGCTAAAT   4353
m150501_iid=3748029_0      CACATATAAACAATAAAGCCTTGAAAAGAGCATGAGATAAAAGATGAAAAAAAGCTAAAT   4896
m150504_iid=2876272_0      CACATATAAACAATAAAGCCTTGAAAAGAGCATGAGATAAAAGATGAAAAAAAGCTAAAT   6760
NC_042820.1                CACATATAAACAATAAAGCCTTGAAAAGAGCATGAGATAAAAGATGAAAAAAAGCTAAAT   7003
m141102_iid=1228825_0      CACATATAAACAATAAAGCCTTGAAAAGAGCATGAGATAAAAGATGAAAAAAAGCTAAAT   6829
m150504_iid=2876274_0      CACATATAAACAATAAAGCCTTGAAAAGAGCATGAGATAAAAGATGAAAAAAAGCTAAAT   6832
m141030_iid=80036_0        CACATATAAACAATAAAGCCTTGAAAAGAGCATGAGATAAAAGATGAAAAAAAGCTAAAT   6593
m150506_iid=2596494_0      CACATATAAACAATAAAGCCTTGAAAAGAGCATGAGATAAAAGATGAAAAAAAGCTAAAT   6998
m150503_iid=2526862_0      CACATATAAACAATAAAGCCTTGAAAAGAGCATGAGATAAAAGATGAAAAAAAGCTAAAT   6681
m150506_iid=2567000_0      CACATATAAACAATAAAGCCTTGAAAAGAGCATGAGATAAAAGATGAAAAAAAGCTAAAT   4631
m150507_iid=3073271_0      CACATATAAACAATAAAGCCTTGAAAAGAGCATGAGATAAAAGATGAAAAAAAGCTAAAT   5935
m150504_iid=3156087_0      CACATATAAACAATAAAGCCTTGAAAAGAGCATGAGATAAAAGATGAAAAAAAGCTAAAT   5758
m150505_iid=2498502_0      CACATATAAACAATAAAGCCTTGAAAAGAGCATGAGATAAAAGATGAAAAAAAGCTAAAT   3408
m141102_iid=754227_0       CACATATAAACAATAAAGCCTTGAAAAGAGCATGAGATAAAAGATGAAAAAAAGCTAAAT   5970
m150507_iid=3469514_0      CACATATAAACAATAAAGCCTTGAAAAGAGCATGAGATAAAAGATGAAAAAAAGCTAAAT   6528
Rdo_MitoGenome             CACATATAAACAATAAAGCCTTGAAAAGAGCATGAGATAAAAGATGAAAAAAAGCTAAAT   7003
m141101_iid=614933_0       CACATATAAACAATAAAGCCTTGAAAAGAGCATGAGATAAAAGATGAAAAAAAGCTAAAT   5554

                                                 nad5

m141031_iid=347546_0       ATTCACCACCTCTAAATAAAATTCTTATTATCAATCCTAATTGACTAAG-GTAGAAAGAG   1788
m150506_iid=3127414_0      ATTCACCACCTCTAAATAAAATTCTTATTATCAATCCTAATTGACTAAGAGTAGAAAGAG   3521
m141102_iid=1245517_0      ATTCACCACCTCTAAATAAAATTCTTATTATCAATCCTAATTGACTAAGAGTAGAAAGAG   5175
m141107_iid=1405727_0      ATTCACCACCTCTAAATAAAATTCTTA-TTTCAATCCTAATTGACTAAGAGTAGAAAGAG   2351
m141102_iid=987614_0       ATTCACCACCTCTAAATAAAATTCTTATTATCAATCCTAATTGACTAAGAGTAGAAAGAG   5337
m141108_iid=1701429_0      ATTCACCACCTCTAAATAAAATTCTTATTATCAATCCTAATTGACTAAGAGTAGAAAGAG   2074
m141102_iid=987286_0       ATTCACCACCTCTAAATAAAATTCTTATTATCAATCCTAATTGACTAAGAGTAGAAAGAG   3289
m150430_iid=3272125_0      ATTCACCACCTCTAAATAAAATTCTTATTATCAATCCTAATTGACTAAGAGTAGAAAGAG   4810
m141102_iid=754225_0       ATTCACCACCTCTAAATAAAATTCTTATTATCAATCCTAATTGACTAAGAGTAGAAAGAG   5975
m141107_iid=1409934_0      ATTCACCACCTCTAAATAAAATTCTTATTATCAATCCTAATTGACTAAGAGTAGAAAGAG   6520
m150503_iid=3416920_0      ATTCACCACCTCTAAATAAAATTCTTATTATCAATCCTAATTGACTAAGAGTAGAAAGAG   5239
```

```
m141108_iid=1800121_0      ATTCACCACCTCTAAATAAAATTCTTATTATCAATCCTAATTGACTAAGAGTAGAAAGAG   5050
m141031_iid=147540_0       ATTCACCACCTCTAAATAAAATTCTTATTATCAATCCTAATTGACTAAGAGTAGAAAGAG   5395
m150507_iid=3674924_0      ATTCACCACCTCTAAATAAAATTCTTATTATCAATCCTAATTGACTAAGAGTAGAAAGAG   5545
m150503_iid=2923751_0      ATTCACCACCTCTAAATAAAATTCTTATTATCAATCCTAATTGACTAAGAGTAGAAAGAG   5385
m141031_iid=115118_0       ATTCACCACCTCTAAATAAAATTCTTATTATCAATCCTAATTGACTAAGAGTAGAAAGAG   4239
m150430_iid=2722448_0      ATTCACCACCTCTAAATAAAATTCTTATTATCAATCCTAATTGACTAAGAGTAGAAAGAG   4175
m150507_iid=3102647_0      ATTCACCACCTCTAAATAAAATTCTTATTATCAATCCTAATTGACTAAGAGTAGAAAGAG   2480
m141108_iid=1811866_0      ATTCACCACCTCTAAATAAAATTCTTATTATCAATCCTAATTGACTAAGAGTAGAAAGAG   4357
m150501_iid=2948495_0      ATTCACCACCTCTAAATAAAATTCTTATTATCAATCCTAATTGACTAAGAGTAGAAAGAG   3780
m150504_iid=3238195_0      ATTCACCACCTCTAAATAAAATTCTTATTATCAATCCTAATTGACTAAGAGTAGAAAGAG   6112
m150503_iid=3388511_0      ATTCACCACCTCTAAATAAAATTCTTATTATCAATCCTAATTGACTAAGAGTAGAAAGAG   3813
m150505_iid=3777611_0      ATTCACCACCTCTAAATAAAATTCTTATTATCAATCCTAATTGACTAAGAGTAGAAAGAG   5231
m150430_iid=2438833_0      ATTCACCACCTCTAAATAAAATTCTTATTATCAATCCTAATTGACTAAGAGTAGAAAGAG   5570
m141108_iid=1698969_0      ATTCACCACCTCTAAATAAAATTCTTATTATCAATCCTAATTGACTAAGAGTAGAAAGAG   6697
m150430_iid=3059190_0      ATTCACCACCTCTAAATAAAATTCTTATTATCAATCCTAATTGACTAAGAGTAGAAAGAG   2418
m141107_iid=1383067_0      ATTCACCACCTCTAAATAAAATTCTTATTATCAATCCTAATTGACTAAGAGTAGAAAGAG   2424
m141101_iid=597008_0       ------------------------------------------------------------   0
m150506_iid=2596496_0      ATTCACCACCTCTAAATAAAATTCTTATTATCAATCCTAATTGACTAAGAGTAGAAAGAG   7055
m141102_iid=910051_0       ATTCACCACCTCTAAATAAAATTCTTATTATCAATCCTAATTGACTAAGAGTAGAAAGAG   6543
m150505_iid=3784017_0      ATTCACCACCTCTAAATAAAATTCTTATTATCAATCCTAATTGACTAAGAGTAGAAAGAG   5344
m141101_iid=682151_0       ATTCACCACCTCTAAATAAAATTCTTATTATCAATCCTAATTGACTAAGAGTAGAAAGAG   4413
m150501_iid=3748029_0      ATTCACCACCTCTAAATAAAATTCTTATTATCAATCCTAATTGACTAAGAGTAGAAAGAG   4956
m150504_iid=2876272_0      ATTCACCACCTCTAAATAAAATTCTTATTATCAATCCTAATTGACTAAGAGTAGAAAGAG   6820
NC_042820.1                ATTCACCACCTCTAAATAAAATTCTTATTATCAATCCTAATTGACTAAGAGTAGAAAGAG   7063
m141102_iid=1228825_0      ATTCACCACCTCTAAATAAAATTCTTATTATCAATCCTAATTGACTAAGAGTAGAAAGAG   6889
m150504_iid=2876274_0      ATTCACCACCTCTAAATAAAATTCTTATTATCAATCCTAATTGACTAAGAGTAGAAAGAG   6892
m141030_iid=80036_0        ATTCACCACCTCTAAATAAAATTCTTATTATCAATCCTAATTGACTAAGAGTAGAAAGAG   6653
m150506_iid=2596494_0      ATTCACCACCTCTAAATAAAATTCTTATTATCAATCCTAATTGACTAAGAGTAGAAAGAG   7058
m150503_iid=2526862_0      ATTCACCACCTCTAAATAAAATTCTTATTATCAATCCTAATTGACTAAGAGTAGAAAGAG   6741
m150506_iid=2567000_0      ATTCACCACCTCTAAATAAAATTCTTATTATCAATCCTAATTGACTAAGAGTAGAAAGAG   4691
m150507_iid=3073271_0      ATTCACCACCTCTAAATAAAATTCTTATTATCAATCCTAATTGACTAAGAGTAGAAAGAG   5995
m150504_iid=3156087_0      ATTCACCACCTCTAAATAAAATTCTTATTATCAATCCTAATTGACTAAGAGTAGAAAGAG   5818
m150505_iid=2498502_0      ATTCACCACCTCTAAATAAAATTCTTATTATCAATCCTAATTGACTAAGAGTAGAAAGAG   3468
m141102_iid=754227_0       ATTCACCACCTCTAAATAAAATTCTTATTATCAATCCTAATTGACTAAGAGTAGAAAGAG   6030
m150507_iid=3469514_0      ATTCACCACCTCTAAATAAAATTCTTATTATCAATCCTAATTGACTAAGAGTAGAAAGAG   6588
Rdo_MitoGenome             ATTCACCACCTCTAAATAAAATTCTTATTATCAATCCTAATTGACTAAGAGTAGAAAGAG   7063
m141101_iid=614933_0       ATTCACCACCTCTAAATAAAATTCTTATTATCAATCCTAATTGACTAAGAGTAGAAAGAG   5614
```

nad5

```
m141031_iid=347546_0       CAATGATTTTCTTTAAATCAAATTCAAAATTAGCTCCAAGCCCAGCTATAAATATAGTTA   1848
m150506_iid=3127414_0      CAATGATTTTCTTTAAATCAAATTCAAAATTAGCTCCAAGCCCAGCTATAAATATAGTTA   3581
m141102_iid=1245517_0      CAATGATTTTCTTTAAATCAAATTCAAAATTAGCTCCAAGC-CAGCTATAAATATAGTTA   5234
m141107_iid=1405727_0      CAATGATTTTCTTTAAATCAAATTCAAAATTAGCTCCAAGCCCAGCTATAAATATAGTTA   2411
m141102_iid=987614_0       CAATGATTTTCTTTAAATCAAATTCAAAATTAGCTCCAAGCCCAGCTATAAATATAGTTA   5397
m141108_iid=1701429_0      CAATGATTTTCTTTAAATCAAATTCAAAATTAGCTCCAAGCCCAGCTATAAATATAGTTA   2134
m141102_iid=987286_0       CAATGATTTTCTTTAAATCAAATTCAAAATTAGCTCCAAGCCCAGCTATAAATATAGTTA   3349
m150430_iid=3272125_0      CAATGATTTTCTTTAAATCAAATTCAAAATTAGCTCCAAGCCCAGCTATAAATATAGTTA   4870
m141102_iid=754225_0       CAATGATTTTCTTTAAATCAAATTCAAAATTAGCTCCAAGCCCAGCTATAAATATAGTTA   6035
m141107_iid=1409934_0      CAATGATTTTCTTTAAATCAAATTCAAAATTAGCTCCAAGCCCAGCTATAAATATAGTTA   6580
m150503_iid=3416920_0      CAATGATTTTCTTTAAATCAAATTCAAAATTAGCTCCAAGCCCAGCTATAAATATAGTTA   5299
m141108_iid=1800121_0      CAATGATTTTCTTTAAATCAAATTCAAAATTAGCTCCAAGCCCAGCTATAAATATAGTTA   5110
m141031_iid=147540_0       CAATGATTTTCTTTAAATCAAATTCAAAATTAGCTCCAAGCCCAGCTATAAATATAGTTA   5455
m150507_iid=3674924_0      CAATGATTTTCTTTAAATCAAATTCAAAATTAGCTCCAAGCCCAGCTATAAATATAGTTA   5605
m150503_iid=2923751_0      CAATGATTTTCTTTAAATCAAATTCAAAATTAGCTCCAAGCCCAGCTATAAATATAGTTA   5445
m141031_iid=115118_0       CAATGATTTTCTTTAAATCAAATTCAAAATTAGCTCCAAGCCCAGCTATAAATATAGTTA   4299
m150430_iid=2722448_0      CAATGATTTTCTTTAAATCAAATTCAAAATTAGCTCCAAGCCCAGCTATAAATATAGTTA   4235
m150507_iid=3102647_0      CAATGATTTTCTTTAAATCAAATTCAAAATTAGCTCCAAGCCCAGCTATAAATATAGTTA   2540
m141108_iid=1811866_0      CAATGATTTTCTTTAAATCAAATTCAAAATTAGCTCCAAGCCCAGCTATAAATATAGTTA   4417
m150501_iid=2948495_0      CAATGATTTTCTTTAAATCAAATTCAAAATTAGCTCCAAGCCCAGCTATAAATATAGTTA   3840
m150504_iid=3238195_0      CAATGATTTTCTTTAAATCAAATTCAAAATTAGCTCCAAGCCCAGCTATAAATATAGTTA   6172
m150503_iid=3388511_0      CAATGATTTTCTTTAAATCAAATTCAAAATTAGCTCCAAGCCCAGCTATAAATATAGTTA   3873
m150505_iid=3777611_0      CAATGATTTTCTTTAAATCAAATTCAAAATTAGCTCCAAGCCCAGCTATAAATATAGTTA   5291
m150430_iid=2438833_0      CAATGATTTTCTTTAAATCAAATTCAAAATTAGCTCCAAGCCCAGCTATAAATATAGTTA   5630
m141108_iid=1698969_0      CAATGATTTTCTTTAAATCAAATTCAAAATTAGCTCCAAGCCCAGCTATAAATATAGTTA   6757
m150430_iid=3059190_0      CAATGATTTTCTTTAAATCAAATTCAAAATTAGCTCCAAGCCCAGCTATAAATATAGTTA   2478
m141107_iid=1383067_0      CAATGATTTTCTTTAAATCAAATTCAAAATTAGCTCCAAGCCCAGCTATAAATATAGTTA   2484
m141101_iid=597008_0       ------------------------------------------------------------   0
m150506_iid=2596496_0      CAATGATTTTCTTTAAATCAAATTCAAAATTAGCTCCAAGCCCAGCTATAAATATAGTTA   7115
m141102_iid=910051_0       CAATGATTTTCTTTAAATCAAATTCAAAATTAGCTCCAAGCCCAGCTATAAATATAGTTA   6603
m150505_iid=3784017_0      CAATGATTTTCTTTAAATCAAATTCAAAATTAGCTCCAAGCCCAGCTATAAATATAGTTA   5404
m141101_iid=682151_0       CAATGATTTTCTTTAAATCAAATTCAAAATTAGCTCCAAGCCCAGCTATAAATATAGTTA   4473
```

```
m150501_iid=3748029_0      CAATGATTTTCTTTAAATCAAATTCAAAATTAGCTCCAAGCCCAGCTATAAATATAGTTA   5016
m150504_iid=2876272_0      CAATGATTTTCTTTAAATCAAATTCAAAATTAGCTCCAAGCCCAGCTATAAATATAGTTA   6880
NC_042820.1                CAATGATTTTCTTTAAATCAAATTCAAAATTAGCTCCAAGCCCAGCTATAAATATAGTTA   7123
m141102_iid=1228825_0      CAATGATTTTCTTTAAATCAAATTCAAAATTAGCTCCAAGCCCAGCTATAAATATAGTTA   6949
m150504_iid=2876274_0      CAATGATTTTCTTTAAATCAAATTCAAAATTAGCTCCAAGCCCAGCTATAAATATAGTTA   6952
m141030_iid=80036_0        CAATGATTTTCTTTAAATCAAATTCAAAATTAGCTCCAAGCCCAGCTATAAATATAGTTA   6713
m150506_iid=2596494_0      CAATGATTTTCTTTAAATCAAATTCAAAATTAGCTCCAAGCCCAGCTATAAATATAGTTA   7118
m150503_iid=2526862_0      CAATGATTTTCTTTAAATCAAATTCAAAATTAGCTCCAAGCCCAGCTATAAATATAGTTA   6801
m150506_iid=2567000_0      CAATGATTTTCTTTAAATCAAATTCAAAATTAGCTCCAAGCCCAGCTATAAATATAGTTA   4751
m150507_iid=3073271_0      CAATGATTTTCTTTAAATCAAATTCAAAATTAGCTCCAAGCCCAGCTATAAATATAGTTA   6055
m150504_iid=3156087_0      CAATGATTTTCTTTAAATCAAATTCAAAATTAGCTCCAAGCCCAGCTATAAATATAGTTA   5878
m150505_iid=2498502_0      CAATGATTTTCTTTAAATCAAATTCAAAATTAGCTCCAAGCCCAGCTATAAATATAGTTA   3528
m141102_iid=754227_0       CAATGATTTTCTTTAAATCAAATTCAAAATTAGCTCCAAGCCCAGCTATAAATATAGTTA   6090
m150507_iid=3469514_0      CAATGATTTTCTTTAAATCAAATTCAAAATTAGCTCCAAGCCCAGCTATAAATATAGTTA   6648
Rdo_MitoGenome             CAATGATTTTCTTTAAATCAAATTCAAAATTAGCTCCAAGCCCAGCTATAAATATAGTTA   7123
m141101_iid=614933_0       CAATGATTTTCTTTAAATCAAATTCAAAATTAGCTCCAAGCCCAGCTATAAATATAGTTA   5674
```

nad5

```
m141031_iid=347546_0       AACAAGAAAGCAATAATCCTAAAATTAAAACTTTATCACAAAAACA-TAACTAAAACGAA   1907
m150506_iid=3127414_0      AACAAGAAAGCAATAATCCTAAAATTAAAACTTTATCACAAAAACAATAACTAAAACGAA   3641
m141102_iid=1245517_0      AACAAGAAAGCAATAATCCTAAAATTAAAACTTTATCACAAAAACAATAACTAAAACGAA   5294
m141107_iid=1405727_0      AACAAGAAAGCAATAATCCTAAA-TTAAAACTTTATCACAAAAACAATAACTAAAACGAA   2470
m141102_iid=987614_0       AACAAGAAAGCAATAATCCTAAAATTAAAACTTTATCACAAAAACAATAACTAAAACGAA   5457
m141108_iid=1701429_0      AACAAGAAAGCAATAATCCTAAAATTAAAACTTTATCACAAAAACAATAACTAAAACGAA   2194
m141102_iid=987286_0       AACAAGAAAGCAATAATCCTAAAATTAAAACTTTATCACAAAAACAATAACTAAAACGAA   3409
m150430_iid=3272125_0      AACAAGAAAGCAATAATCCTAAAATTAAAACTTTATCACAAAAACAATAACTAAAACGAA   4930
m141102_iid=754225_0       AACAAGAAAGCAATAATCCTAAAATTAAAACTTTATCACAAAAACAATAACTAAAACGAA   6095
m141107_iid=1409934_0      AACAAGAAAGCAATAATCCTAAAATTAAAACTTTATCACAAAAACAATAACTAAAACGAA   6640
m150503_iid=3416920_0      AACAAGAAAGCAATAATCCTAAAATTAAAACTTTATCACAAAAACAATAACTAAAACGAA   5359
m141108_iid=1800121_0      AACAAGAAAGCAATAATCCTAAAATTAAAACTTTATCACAAAAACAATAACTAAAACGAA   5170
m141031_iid=147540_0       AACAAGAAAGCAATAATCCTAAAATTAAAACTTTATCACAAAAACAATAACTAAAACGAA   5515
m150507_iid=3674924_0      AACAAGAAAGCAATAATCCTAAAATTAAAACTTTATCACAAAAACAATAACTAAAACGAA   5665
m150503_iid=2923751_0      AACAAGAAAGCAATAATCCTAAAATTAAAACTTTATCACAAAAACAATAACTAAAA--AA   5503
m141031_iid=115118_0       AACAAGAAAGCAATAATCCTAAAATTAAAACTTTATCACAAAAACAATAACTAAAACGAA   4359
m150430_iid=2722448_0      AACAAGAAAGCAATAATCCTAAAATTAAAACTTTATCACAAAAACAATAACTAAAACGAA   4295
m150507_iid=3102647_0      AACAAGAAAGCAATAATCCTAAAATTAAAACTTTATCACAAAAACAATAACTAAAACGAA   2600
m141108_iid=1811866_0      AACAAGAAAGCAATAATCCTAAAATTAAAACTTTATCACAAAAACAATAACTAAAACGAA   4477
m150501_iid=2948495_0      AACAAGAAAGCAATAATCCTAAAATTAAAACTTTATCACAAAAACAATAACTAAAACGAA   3900
m150504_iid=3238195_0      AACAAGAAAGCAATAATCCTAAAATTAAAACTTTATCACAAAAACAATAACTAAAACGAA   6232
m150503_iid=3388511_0      AACAAGAAAGCAATAATCCTAAAATTAAAACTTTATCACAAAAACAATAACTAAAACGAA   3933
m150505_iid=3777611_0      AACAAGAAAGCAATAATCCTAAAATTAAAACTTTATCACAAAAACAATAACTAAAACGAA   5351
m150430_iid=2438833_0      AACAAGAAAGCAATAATCCTAAAATTAAAACTTTATCACAAAAA-AATAACTAAAACGAA   5689
m141108_iid=1698969_0      AACAAGAAAGCAATAATCCTAAAATTAAAACTTTATCACAAAAACAATAACTAAAACGAA   6817
m150430_iid=3059190_0      AACAAGAAAGCAATAATCCTAAAATTAAAACTTTATCACAAAAACAATAACTAAAACGAA   2538
m141107_iid=1383067_0      AACAAGAAAGCAATAATCCTAAAATTAAAACTTTATCACAAAAACAATAACTAAAACGAA   2544
m141101_iid=597008_0       ------------------------------------------------------------   0
m150506_iid=2596496_0      AACAAGAAAGCAATAATCCTAAAATTAAAACTTTATCACAAAAACAATAACTAAAACGAA   7175
m141102_iid=910051_0       AACAAGAAAGCAATAATCCTAAAATTAAAACTTTATCACAAAAACAATAACTAAAACGAA   6663
m150505_iid=3784017_0      AACAAGAAAGCAATAATCCTAAAATTAAAACTTTATCACAAAAACAATAACTAAAACGAA   5464
m141101_iid=682151_0       AACAAGAAAGCAATAATCCTAAAATTAAAACTTTATCACAAAAACAATAACTAAAACGAA   4533
m150501_iid=3748029_0      AACAAGAAAGCAATAATCCTAAAATTAAAACTTTATCACAAAAACAATAACTAAAACGAA   5076
m150504_iid=2876272_0      AACAAGAAAGCAATAATCCTAAAATTAAAACTTTATCACAAAAACAATAACTAAAACGAA   6940
NC_042820.1                AACAAGAAAGCAATAATCCTAAAATTAAAACTTTATCACAAAAACAATAACTAAAACGAA   7183
m141102_iid=1228825_0      AACAAGAAAGCAATAATCCTAAAATTAAAACTTTATCACAAAAACAATAACTAAAACGAA   7009
m150504_iid=2876274_0      AACAAGAAAGCAATAATCCTAAAATTAAAACTTTATCACAAAAACAATAACTAAAACGAA   7012
m141030_iid=80036_0        AACAAGAAAGCAATAATCCTAAAATTAAAACTTTATCACAAAAACAATAACTAAAACGAA   6773
m150506_iid=2596494_0      AACAAGAAAGCAATAATCCTAAAATTAAAACTTTATCACAAAAACAATAACTAAAACGAA   7178
m150503_iid=2526862_0      AACAAGAAAGCAATAATCCTAAAATTAAAACTTTATCACAAAAACAATAACTAAAACGAA   6861
m150506_iid=2567000_0      AACAAGAAAGCAATAATCCTAAAATTAAAACTTTATCACAAAAACAATAACTAAAACGAA   4811
m150507_iid=3073271_0      AACAAGAAAGCAATAATCCTAAAATTAAAACTTTATCACAAAAACAATAACTAAAACGAA   6115
m150504_iid=3156087_0      AACAAGAAAGCAATAATCCTAAAATTAAAACTTTATCACAAAAACAATAACTAAAACGAA   5938
m150505_iid=2498502_0      AACAAGAAAGCAATAATCCTAAAATTAAAACTTTATCACAAAAACAATAACTAAAACGAA   3588
m141102_iid=754227_0       AACAAGAAAGCAATAATCCTAAAATTAAAACTTTATCACAAAAACAATAACTAAAACGAA   6150
m150507_iid=3469514_0      AACAAGAAAGCAATAATCCTAAAATTAAAACTTTATCACAAAAACAATAACTAAAACGAA   6708
Rdo_MitoGenome             AACAAGAAAGCAATAATCCTAAAATTAAAACTTTATCACAAAAACAATAACTAAAACGAA   7183
m141101_iid=614933_0       AACAAGAAAGCAATAATCCTAAAATTAAAACTTTATCACAAAAACAATAACTAAAACGAA   5734
```

nad5

```
m141031_iid=347546_0       TTATTAAGTAAA-CCCAGCTGTTACTAAAGTAG--AAGAATGAA-CCAATGAAGATACAG   1963
m150506_iid=3127414_0      TTATTAAGTAAACCCCAGCTGTTACTA-AGTAG--AAGAATGAA-CCAATGAAGATACAG   3697
m141102_iid=1245517_0      TTATTAAGTAAACCCCAGCTGTTACTAAAGTAGAAG---AATGAACCAATGAAGATACAG   5351
```

```
m141107_iid=1405727_0      TTATTAAGTAAACCCCAGCTGTTAAAGTAAAAGTATAAGAAGAATGAAAATGAAGAGCAG   2530
m141102_iid=987614_0       TTATTAAGTAAACCCCAGCTGTTACTAAAGTAG--AAGAATGAA-CCAATGAAGATACAG   5514
m141108_iid=1701429_0      TTATTAAGTAAACCCCAGCTGTTACTAAAGTAG--AAGAATGAA-CCAATGAAGATACAG   2251
m141102_iid=987286_0       TTATTAAGTAAACCCCAGCTGTTACTAAAGTAG--AAGAATGAA-CCAATGAAGATACAG   3466
m150430_iid=3272125_0      TTATTAAGTAAACCCCAGCTGTTACTAAAGTAG--AAGAATGAA-CCAATGAAGATACAG   4987
m141102_iid=754225_0       TTATTAAGTAAACCCCAGCTGTTACTAAAGTAG--AAGAATGAA-CCAATGAAGATACAG   6152
m141107_iid=1409934_0      TTATTAAGTAAACCCCAGCTGTTACTAAAGTAG--AAGAATGAA-CCAATGAAGATACAG   6697
m150503_iid=3416920_0      TTATTAAGTAAACCCCAGCTGTTACTAAAGTAG--AAGAATGAA-CCAATGAAGATACAG   5416
m141108_iid=1800121_0      TTATTAAGTAAACCCCAGCTGTTACTAAAGTAG--AAGAATGAA-CCAATGAAGATACAG   5227
m141031_iid=147540_0       TTATTAAGTAAACCCCAGCTGTTACTAAAGTAG--AAGAATGAA-CCAATGAAGATACAG   5572
m150507_iid=3674924_0      TTATTAAGTAAACCCCAGCTGTTACTAAAGTAG--AAGAATGAA-CCAATGAAGATACAG   5722
m150503_iid=2923751_0      TTATTAAGTAAACCCCAGCTGTTACTAAAGTAG--AAGAATGAA-CCAATGAAGATACAG   5560
m141031_iid=115118_0       TTATTAAGTAAACCCCAGCTGTTACTAAAGTAG--AAGAATGAA-CCAATGAAGATACAG   4416
m150430_iid=2722448_0      TTATTAAGTAAACCCCAGCTGTTACTAAAGTAG--AAGAATGAA-CCAATGAAGATACAG   4352
m150507_iid=3102647_0      TTATTAAGTAAACCCCAGCTGTTACTAAAGTAG--AAGAATGAA-CCAATGAAGATACAG   2657
m141108_iid=1811866_0      TTATTAAGTAAACCCCAGCTGTTACTAAAGTAG--AAGAATGAA-CCAATGAAGATACAG   4534
m150501_iid=2948495_0      TTATTAAGTAAACCCCAGCTGTTACTAAAGTAG--AAGAATGAA-CCAATGAAGATACAG   3957
m150504_iid=3238195_0      TTATTAAGTAAACCCCAGCTGTTACTAAAGTAG--AAGAATGAA-CCAATGAAGATACAG   6289
m150503_iid=3388511_0      TTATTAAGTAAACCCCAGCTGTTACTAAAGTAG--AAGAATGAA-CCAATGAAGATACAG   3990
m150505_iid=3777611_0      TTATTAAGTAAACCCCAGCTGTTACTAAAGTAG--AAGAATGAA-CCAATGAAGATACAG   5408
m150430_iid=2438833_0      TTATTAAGTAAACCCCAGCTGTTACTAAAGTAG--AAGAATGAA-CCAATGAAGATACAG   5746
m141108_iid=1698969_0      TTATTAAGTAAACCCCAGCTGTTACTAAAGTAG--AAGAATGAA-CCAATGAAGATACAG   6874
m150430_iid=3059190_0      TTATTAAGTAAACCCCAGCTGTTACTAAAGTAG--AAGAATGAA-CCAATGAAGATACAG   2595
m141107_iid=1383067_0      TTATTAAGTAAACCCCAGCTGTTACTAAAGTAG--AAGAATGAA-CCAATGAAGATACAG   2601
m141101_iid=597008_0       ------------------------------------------------------------   0
m150506_iid=2596496_0      TTATTAAGTAAACCCCAGCTGTTACTAAAGTAG--AAGAATGAA-CCAATGAAGATACAG   7232
m141102_iid=910051_0       TTATTAAGTAAACCCCAGCTGTTACTAAAGTAGAGAAGAATGAA-CCAATGAAGATACAG   6722
m150505_iid=3784017_0      TTATTAAGTAAACCCCAGCTGTTACTAAAGTAG--AAGAATGAA-CCAATGAAGATACAG   5521
m141101_iid=682151_0       TTATTAAGTAAACCCCAGCTGTTACTAAAGTAG--AAGAATGAA-CCAATGAAGATACAG   4590
m150501_iid=3748029_0      TTATTAAGTAAACCCCAGCTGTTACTAAAGTAG--AAGAATGAA-CCAATGAAGATACAG   5133
m150504_iid=2876272_0      TTATTAAGTAAACCCCAGCTGTTACTAAAGTAG--AAGAATGAA-CCAATGAAGATACAG   6997
NC_042820.1                TTATTAAGTAAACCCCAGCTGTTACTAAAGTAG--AAGAATGAA-CCAATGAAGATACAG   7240
m141102_iid=1228825_0      TTATTAAGTAAACCCCAGCTGTTACTAAAGTAG--AAGAATGAA-CCAATGAAGATACAG   7066
m150504_iid=2876274_0      TTATTAAGTAAACCCCAGCTGTTACTAAAGTAG--AAGAATGAA-CCAATGAAGATACAG   7069
m141030_iid=80036_0        TTATTAAGTAAACCCCAGCTGTTACTAAAGTAG--AAGAATGAA-CCAATGAAGATACAG   6830
m150506_iid=2596494_0      TTATTAAGTAAACCCCAGCTGTTACTAAAGTAG--AAGAATGAA-CCAATGAAGATACAG   7235
m150503_iid=2526862_0      TTATTAAGTAAACCCCAGCTGTTACTAAAGTAG--AAGAATGAA-CCAATGAAGATACAG   6918
m150506_iid=2567000_0      TTATTAAGTAAACCCCAGCTGTTACTAAAGTAG--AAGAATGAA-CCAATGAAGATACAG   4868
m150507_iid=3073271_0      TTATTAAGTAAACCCCAGCTGTTACTAAAGTAG--AAGAATGAA-CCAATGAAGATACAG   6172
m150504_iid=3156087_0      TTATTAAGTAAACCCCAGCTGTTACTAAAGTAG--AAGAATGAA-CCAATGAAGATACAG   5995
m150505_iid=2498502_0      TTATTAAGTAAACCCCAGCTGTTACTAAAGTAG--AAGAATGAA-CCAATGAAGATACAG   3645
m141102_iid=754227_0       TTATTAAGTAAACCCCAGCTGTTACTAAAGTAG--AAGAATGAA-CCAATGAAGATACAG   6207
m150507_iid=3469514_0      TTATTAAGTAAACCCCAGCTGTTACTAAAGTAG--AAGAATGAA-CCAATGAAGATACAG   6765
Rdo_MitoGenome             TTATTAAGTAAACCCCAGCTGTTACTAAAGTAG--AAGAATGAA-CCAATGAAGATACAG   7240
m141101_iid=614933_0       TTATTAAGTAAACCCCAGCTGTTACTAAAGTAG--AAGAATGAA-CCAATGAAGATACAG   5791
```

nad5

```
m141031_iid=347546_0       GTGTAGGAGCTGCTATAGCAGCAGGTAACCAAGCAGAAAAAGGAA-TTTGAGCTCTTTTA   2022
m150506_iid=3127414_0      GTGTAGGAGCTGCTATAGCAGCAGGTAACCAAGCAGAAAAAGGAA-TTTGAGCTCTTTTA   3756
m141102_iid=1245517_0      GTGTAGGAGCTGCTATAGCAGCAGGTAACCAAGCAGAAAAAGGAA-TTTGAGCTCTTTTA   5410
m141107_iid=1405727_0      GTGTAGGAGCTGCTATAGCAGCAGGTAACCAAGCAGAAAAAGGATATTTGAGCTCTTTT-   2589
m141102_iid=987614_0       GTGTAGGAGCTGCTATAGCAGCAGGTAACCAAGCAGAAAAAGGAA-TTTGAGCTCTTTTA   5573
m141108_iid=1701429_0      GTGTAGGAGCTGCTATAGCAGCAGGTAACCAAGCAGAAAAAGGAA-TTTGAGCTCTTTTA   2310
m141102_iid=987286_0       GTGTAGGAGCTGCTATAGCAGCAGGTAACCAAGCAGAAAAAGGAA-TTTGAGCTCTTTTA   3525
m150430_iid=3272125_0      GTGTAGGAGCTGCTATAGCAGCAGGTAACCAAGCAGAAAAAGGAA-TTTGAGCTCTTTTA   5046
m141102_iid=754225_0       GTGTAGGAGCTGCTATAGCAGCAGGTAACCAAGCAGAAAAAGGAA-TTTGAGCTCTTTTA   6211
m141107_iid=1409934_0      GTGTAGGAGCTGCTATAGCAGCAGGTAACCAAGCAGAAAAAGGAA-TTTGAGCTCTTTTA   6756
m150503_iid=3416920_0      GTGTAGGAGCTGCTATAGCAGCAGGTAACCAAGCAGAAAAAGGAA-TTTGAGCTCTTTTA   5475
m141108_iid=1800121_0      GTGTAGGAGCTGCTATAGCAGCAGGTAACCAAGCAGAAAAAGGAA-TTTGAGCTCTTTTA   5286
m141031_iid=147540_0       GTGTAGGAGCTGCTATAGCAGCAGGTAACCAAGCAGAAAAAGGAA-TTTGAGCTCTTTTA   5631
m150507_iid=3674924_0      GTGTAGGAGCTGCTATAGCAGCAGGTAACCAAGCAGAAAAAGGAA-TTTGAGCTCTTTTA   5781
m150503_iid=2923751_0      GTGTAGGAGCTGCTATAGCAGCAGGTAACCAAGCAGAAAAAGGAA-TTTGAGCTCTTTTA   5619
m141031_iid=115118_0       GTGTAGGAGCTGCTATAGCAGCAGGTAACCAAGCAGAAAAAGGAA-TTTGAGCTCTTTTA   4475
m150430_iid=2722448_0      GTGTAGGAGCTGCTATAGCAGCAGGTAACCAAGCAGAAAAAGGAA-TTTGAGCTCTTTTA   4411
m150507_iid=3102647_0      GTGTAGGAGCTGCTATAGCAGCAGGTAACCAAGCAGAAAAAGGAA-TTTGAGCTCTTTTA   2716
m141108_iid=1811866_0      GTGTAGGAGCTGCTATAGCAGCAGGTAACCAAGCAGAAAAAGGAA-TTTGAGCTCTTTTA   4593
m150501_iid=2948495_0      GTGTAGGAGCTGCTATAGCAGCAGGTAACCAAGCAGAAAAAGGAA-TTTGAGCTCTTTTA   4016
m150504_iid=3238195_0      GTGTAGGAGCTGCTATAGCAGCAGGTAACCAAGCAGAAAAAGGAA-TTTGAGCTCTTTTA   6348
m150503_iid=3388511_0      GTGTAGGAGCTGCTATAGCAGCAGGTAACCAAGCAGAAAAAGGAA-TTTGAGCTCTTTTA   4049
m150505_iid=3777611_0      GTGTAGGAGCTGCTATAGCAGCAGGTAACCAAGCAGAAAAAGGAA-TTTGAGCTCTTTTA   5467
m150430_iid=2438833_0      GTGTAGGAGCTGCTATAGCAGCAGGTAACCAAGCAGAAAAAGGAA-TTTGAGCTCTTTTA   5805
```

m141108_iid=1698969_0	GTGTAGGAGCTGCTATAGCAGCAGGTAACCAAGCAGAAAAAGGAA-TTTGAGCTCTTTTA	6933
m150430_iid=3059190_0	GTGTAGGAGCTGCTATAGCAGCAGGTAACCAAGCAGAAAAAGGAA-TTTGAGCTCTTTTA	2654
m141107_iid=1383067_0	GTGTAGGAGCTGCTATAGCAGCAGGTAACCAAGCAGAAAAAGGAA-TTTGAGCTCTTTTA	2660
m141101_iid=597008_0	--	0
m150506_iid=2596496_0	GTGTAGGAGCTGCTATAGCAGCAGGTAACCAAGCAGAAAAAGGAA-TTTGAGCTCTTTTA	7291
m141102_iid=910051_0	GTGTAGGAGCTGCTATAGCAGCAGGTAACCAAGCAGAAAAAGGAA-TTTGAGCTCTTTTA	6781
m150505_iid=3784017_0	GTGTAGGAGCTGCTATAGCAGCAGGTAACCAAGCAGAAAAAGGAA-TTTGAGCTCTTTTA	5580
m141101_iid=682151_0	GTGTAGGAGCTGCTATAGCAGCAGGTAACCAAGCAGAAAAAGGAA-TTTGAGCTCTTTTA	4649
m150501_iid=3748029_0	GTGTAGGAGCTGCTATAGCAGCAGGTAACCAAGCAGAAAAAGGAA-TTTGAGCTCTTTTA	5192
m150504_iid=2876272_0	GTGTAGGAGCTGCTATAGCAGCAGGTAACCAAGCAGAAAAAGGAA-TTTGAGCTCTTTTA	7056
NC_042820.1	GTGTAGGAGCTGCTATAGCAGCAGGTAACCAAGCAGAAAAAGGAA-TTTGAGCTCTTTTA	7299
m141102_iid=1228825_0	GTGTAGGAGCTGCTATAGCAGCAGGTAACCAAGCAGAAAAAGGAA-TTTGAGCTCTTTTA	7125
m150504_iid=2876274_0	GTGTAGGAGCTGCTATAGCAGCAGGTAACCAAGCAGAAAAAGGAA-TTTGAGCTCTTTTA	7128
m141030_iid=80036_0	GTGTAGGAGCTGCTATAGCAGCAGGTAACCAAGCAGAAAAAGGAA-TTTGAGCTCTTTTA	6889
m150506_iid=2596494_0	GTGTAGGAGCTGCTATAGCAGCAGGTAACCAAGCAGAAAAAGGAA-TTTGAGCTCTTTTA	7294
m150503_iid=2526862_0	GTGTAGGAGCTGCTATAGCAGCAGGTAACCAAGCAGAAAAAGGAA-TTTGAGCTCTTTTA	6977
m150506_iid=2567000_0	GTGTAGGAGCTGCTATAGCAGCAGGTAACCAAGCAGAAAAAGGAA-TTTGAGCTCTTTTA	4927
m150507_iid=3073271_0	GTGTAGGAGCTGCTATAGCAGCAGGTAACCAAGCAGAAAAAGGAA-TTTGAGCTCTTTTA	6231
m150504_iid=3156087_0	GTGTAGGAGCTGCTATAGCAGCAGGTAACCAAGCAGAAAAAGGAA-TTTGAGCTCTTTTA	6054
m150505_iid=2498502_0	GTGTAGGAGCTGCTATAGCAGCAGGTAACCAAGCAGAAAAAGGAA-TTTGAGCTCTTTTA	3704
m141102_iid=754227_0	GTGTAGGAGCTGCTATAGCAGCAGGTAACCAAGCAGAAAAAGGAA-TTTGAGCTCTTTTA	6266
m150507_iid=3469514_0	GTGTAGGAGCTGCTATAGCAGCAGGTAACCAAGCAGAAAAAGGAA-TTTGAGCTCTTTTA	6824
Rdo_MitoGenome	GTGTAGGAGCTGCTATAGCAGCAGGTAACCAAGCAGAAAAAGGAA-TTTGAGCTCTTTTA	7299
m141101_iid=614933_0	GTGTAGGAGCTGCTATAGCAGCAGGTAACCAAGCAGAAAAAGGAA-TTTGAGCTCTTTTA	5850

nad5

m141031_iid=347546_0	GTAAAAGCCGCTAATATAATAAATAAACTA-ATATAAATAAATAATTATCATCAATAAAA	2081
m150506_iid=3127414_0	GTAAAAGCCGCTAATATAATAATAAACTAA-TAATAAATAAATAATTATCATCAATAAAA	3815
m141102_iid=1245517_0	GTAAAAGCCGCTAATATAATAAATTAA----TAATAAATAAATAATTATCATCAATAA-A	5465
m141107_iid=1405727_0	TTAAAAGCCGCTAATATAATAAATAAACTAATAATAAATAAATAATTATCATCAATA---	2646
m141102_iid=987614_0	GTAAAAGCCGCTAATATAATAAATAAACTAATAATAAATAAATAATTATCATCAATAAAA	5633
m141108_iid=1701429_0	GTAAAAGCCGCTAATATAATAAATAAACTAATAATAAATAAATAATTATCATCAATAAAA	2370
m141102_iid=987286_0	GTAAAAGCCGCTAATATAATAAATAAACTAATAATAAATAAATAATTATCATCAATAAAA	3585
m150430_iid=3272125_0	GTAAAAGCCGCTAATATAATAAATAAACTAATAATAAATAAATAATTATCATCAATAAAA	5106
m141102_iid=754225_0	GTAAAAGCCGCTAATATAATAAATAAACTAATAATAAATAAATAATTATCATCAATAAAA	6271
m141107_iid=1409934_0	GTAAAAGCCGCTAATATAATAAATAAACTAATAATAAATAAATAATTATCATCAATAAAA	6816
m150503_iid=3416920_0	GTAAAAGCCGCTAATATAATAAATAAACTAATAATAAATAAATAATTATCATCAATAAA-	5534
m141108_iid=1800121_0	GTAAAAGCCGCTAATATAATAAATAAACTAATAATAAATAAATAATTATCATCAATAAAA	5346
m141031_iid=147540_0	GTAAAAGCCGCTAATATAATAAATAAACTAATAATAAATAAATAATTATCATCAATAAAA	5691
m150507_iid=3674924_0	GTAAATAAAAATAATATAATAATA-AATAAATAATAATAAATAATATATCATCAATAAAA	5840
m150503_iid=2923751_0	GTAAAAGCCGCTAATATAATAAATAAACTAATAATAAATAAATAATTATCATCAATAAA-	5678
m141031_iid=115118_0	GTAAAAGCCGCTAATATAATAAATAAACTAATAATAAATAAATAATTATCATCAATAAAA	4535
m150430_iid=2722448_0	GTAAAAGCCGCTAATATAATAAATAAACTAATAATAAATAAATAATTATCATCAATAAAA	4471
m150507_iid=3102647_0	GTAAAAGCCGCTAATATAATAAATAAACTAATAATAAATAAATAATTATCATCAATAAA-	2775
m141108_iid=1811866_0	GTAAAAGCCGCTAATATAATAAATAAACTAATAATAAATAAATAATTATCATCAATAAAA	4653
m150501_iid=2948495_0	GTAAAAGCCGCTAATATAATAAATAAACTAATAATAAATAA-ATATTATCATCAATAAAA	4075
m150504_iid=3238195_0	GTAAAAGCCGCTAATATAATAAATAAACTAATAATAAATAAATAATTATCATCAATAAAA	6408
m150503_iid=3388511_0	GTAAAAGCCGCTAATATAATAAATAAACTAATAATA---TAAAAATTATCATCAATAAAA	4106
m150505_iid=3777611_0	GTAAAAGCCGCTAATATAATAAATAAACTAATAATAAATAAATAATTATCATCAATAAAA	5527
m150430_iid=2438833_0	GTAAAAGCCGCTAATATAATAAATAAACTAATAATAAATAAATAATTATCATCAATAAAA	5865
m141108_iid=1698969_0	GTAAAAGCCGCTAATATAATAAATAAACTAATAATAAATAAATAATTATCATCAATAAAA	6993
m150430_iid=3059190_0	GTAAAAGCCGCTAATATAATAAATAAACTAATAATAAATAAATAATTATCATCAATAAAA	2714
m141107_iid=1383067_0	GTAAAAGCCGCTAATATAATAAATAAACTAATAATAAATAAATAATTATCATCAATAAAA	2720
m141101_iid=597008_0	--	0
m150506_iid=2596496_0	GTAAAAGCCGCTAATATAATAAATAAACTAATAATAAATAAATAATTATCATCAATAAAA	7351
m141102_iid=910051_0	GTAAAAGCCGCTAATATAATAAATAAACTAATAATAAATAAATAATTATCATCAATAAAA	6841
m150505_iid=3784017_0	GTAAAAGCCGCTAATATAATAAATAAACTAATAATAAATAAATAATTATCATCAATAAAA	5640
m141101_iid=682151_0	GTAAAAGCCGCTAATATAATAAATAAACTAATAATAAATAAATAATTATCATCAATAAAA	4709
m150501_iid=3748029_0	GTAAAAGCCGCTAATATAATAAATAAACTAATAATAAATAAATAATTATCATCAATAAAA	5252
m150504_iid=2876272_0	GTAAAAGCCGCTAATATAATAAATAAACTAATAATAAATAAATAATTATCATCAATAAAA	7116
NC_042820.1	GTAAAAGCCGCTAATATAATAAATAAACTAATAATAAATAAATAATTATCATCAATAAAA	7359
m141102_iid=1228825_0	GTAAAAGCCGCTAATATAATAAATAAACTAATAATAAATAAATAATTATCATCAATAAAA	7185
m150504_iid=2876274_0	GTAAAAGCCGCTAATATAATAAATAAACTAATAATAAATAAATAATTATCATCAATAAAA	7188
m141030_iid=80036_0	GTAAAAGCCGCTAATATAATAAATAAACTAATAATAAATAAATAATTATCATCAATAAAA	6949
m150506_iid=2596494_0	GTAAAAGCCGCTAATATAATAAATAAACTAATAATAAATAAATAATTATCATCAATAAAA	7354
m150503_iid=2526862_0	GTAAAAGCCGCTAATATAATAAATAAACTAATAATAAATAAATAATTATCATCAATAAAA	7037
m150506_iid=2567000_0	GTAAAAGCCGCTAATATAATAAATAAACTAATAATAAATAAATAATTATCATCAATAAAA	4987
m150507_iid=3073271_0	GTAAAAGCCGCTAATATAATAAATAAACTAATAATAAATAAATAATTATCATCAATAAAA	6291
m150504_iid=3156087_0	GTAAAAGCCGCTAATATAATAAATAAACTAATAATAAATAAATAATTATCATCAATAAAA	6114
m150505_iid=2498502_0	GTAAAAGCCGCTAATATAATAAATAAACTAATAATAAATAAATAATTATCATCAATAAAA	3764
m141102_iid=754227_0	GTAAAAGCCGCTAATATAATAAATAAACTAATAATAAATAAATAATTATCATCAATAAAA	6326

```
m150507_iid=3469514_0      GTAAAAGCCGCTAATATAATAAATAAACTAATAATAAATAAATAATTATCATCAATAAAA   6884
Rdo_MitoGenome             GTAAAAGCCGCTAATATAATAAATAAACTAATAATAAATAAATAATTATCATCAATAAAA   7359
m141101_iid=614933_0       GTAAAAGCCGCTAATATAATAAATAAACTAATAATAAATAAATAATTATCATCAATAAAA   5910
```

nad5

```
m141031_iid=347546_0       AATACATAAAATAAATAATTTCACCTACCATAATTTAATATTCAAGCAATTCTTATTAAC   2141
m150506_iid=3127414_0      AATACATAAAATAAATAATTTCACCTACCATAATTTAATATTCAAGCAATTCTTATTAAC   3875
m141102_iid=1245517_0      AATACATAAAATAAATAATTTCACCTACCATAATTTAATATTCAAGCAATTCTTATTAAC   5525
m141107_iid=1405727_0      AATACATAAAATAAATAATTTCACCTACCATAATTTAATATTCAAGCAATTCTTATTAAC   2706
m141102_iid=987614_0       AATACATAAAATAAATAATTTCACCTACCATAATTTAATATTCAAGCAATTCTTATTAAC   5693
m141108_iid=1701429_0      AATACATAAAATAAATAATTTCACCTACCATAATTTAATATTCAAGCAATTCTTATTAAC   2430
m141102_iid=987286_0       AATACATAAAATAAATAATTTCACCTACCATAATTTAATATTCAAGCAATTCTTATTAAC   3645
m150430_iid=3272125_0      AATACATAAAATAAATAATTTCACCTACCATAATTTAATATTCAAGCAATTCTTATTAAC   5166
m141102_iid=754225_0       AATACATAAAATAAATAATTTCACCTACCATAATTTAATATTCAAGCAATTCTTATTAAC   6331
m141107_iid=1409934_0      AATACATAAAATAAATAATTTCACCTACCATAATTTAATATTCAAGCAATTCTTATTAAC   6876
m150503_iid=3416920_0      AATACATAAAATAAATAATTTCACCTACCATAATTTAATATTCAAGCAATTCTTATTAAC   5594
m141108_iid=1800121_0      AATACATAAAATAAATAATTTCACCTACCATAATTTAATATTCAAGCAATTCTTATTAAC   5406
m141031_iid=147540_0       AATACATAAAATAAATAATTTCACCTACCATAATTTAATATTCAAGCAATTCTTATTAAC   5751
m150507_iid=3674924_0      AATACATAAAATAAATAATTTCACCTACCATAATTTAATATTCAAGCAATTCTTATTAAC   5900
m150503_iid=2923751_0      AATACATAAAATAAATAATTTCACCTACCATAATTTAATATTCAAGCAATTCTTATTAAC   5738
m141031_iid=115118_0       AATACATAAAATAAATAATTTCACCTACCATAATTTAATATTCAAGCAATTCTTATTAAC   4595
m150430_iid=2722448_0      AATACATAAAATAAATAATTTCACCTACCATAATTTAATATTCAAGCAATTCTTATTAAC   4531
m150507_iid=3102647_0      AATACATAAAATAAATAATTTCACCTACCATAATTTAATATTCAAGCAATTCTTATTAAC   2835
m141108_iid=1811866_0      AATACATAAAATAAATAATTTCACCTACCATAATTTAATATTCAAGCAATTCTTATTAAC   4713
m150501_iid=2948495_0      AATACATAAAATAAATAATTTCACCTACCATAATTTAATATTCAAGCAATTCTTATTAAC   4135
m150504_iid=3238195_0      AATACATAAAATAAATAATTTCACCTACCATAATTTAATATTCAAGCAATTCTTATTAAC   6468
m150503_iid=3388511_0      AATACATAAAATAAATAATTTCACCTACCATAATTTAATATTCAAGCAATTCTTATTAAC   4166
m150505_iid=3777611_0      AATACATAAAATAAATAATTTCACCTACCATAATTTAATATTCAAGCAATTCTTATTAAC   5587
m150430_iid=2438833_0      AATACATAAAATAAATAATTTCACCTACCATAATTTAATATTCAAGCAATTCTTATTAAC   5925
m141108_iid=1698969_0      AATACATAAAATAAATAATTTCACCTACCATAATTTAATATTCAAGCAATTCTTATTAAC   7053
m150430_iid=3059190_0      AATACATAAAATAAATAATTTCACCTACCATAATTTAATATTCAAGCAATTCTTATTAAC   2774
m141107_iid=1383067_0      AATACATAAAATAAATAATTTCACCTACCATAATTTAATATTCAAGCAATTCTTATTAAC   2780
m141101_iid=597008_0       ------------------------------------------------------------   0
m150506_iid=2596496_0      AATACATAAAATAAATAATTTCACCTACCATAATTTAATATTCAAGCAATTCTTATTAAC   7411
m141102_iid=910051_0       AATACATAAAATAAATAATTTCACCTACCATAATTTAATATTCAAGCAATTCTTATTAAC   6901
m150505_iid=3784017_0      AATACATAAAATAAATAATTTCACCTACCATAATTTAATATTCAAGCAATTCTTATTAAC   5700
m141101_iid=682151_0       AATACATAAAATAAATAATTTCACCTACCATAATTTAATATTCAAGCAATTCTTATTAAC   4769
m150501_iid=3748029_0      AATACATAAAATAAATAATTTCACCTACCATAATTTAATATTCAAGCAATTCTTATTAAC   5312
m150504_iid=2876272_0      AATACATAAAATAAATAATTTCACCTACCATAATTTAATATTCAAGCAATTCTTATTAAC   7176
NC_042820.1                AATACATAAAATAAATAATTTCACCTACCATAATTTAATATTCAAGCAATCCTTATTAAC   7419
m141102_iid=1228825_0      AATACATAAAATAAATAATTTCACCTACCATAATTTAATATTCAAGCAATTCTTATTAAC   7245
m150504_iid=2876274_0      AATACATAAAATAAATAATTTCACCTACCATAATTTAATATTCAAGCAATTCTTATTAAC   7248
m141030_iid=80036_0        AATACATAAAATAAATAATTTCACCTACCATAATTTAATATTCAAGCAATTCTTATTAAC   7009
m150506_iid=2596494_0      AATACATAAAATAAATAATTTCACCTACCATAATTTAATATTCAAGCAATTCTTATTAAC   7414
m150503_iid=2526862_0      AATACATAAAATAAATAATTTCACCTACCATAATTTAATATTCAAGCAATTCTTATTAAC   7097
m150506_iid=2567000_0      AATACATAAAATAAATAATTTCACCTACCATAATTTAATATTCAAGCAATTCTTATTAAC   5047
m150507_iid=3073271_0      AATACATAAAATAAATAATTTCACCTACCATAATTTAATATTCAAGCAATTCTTATTAAC   6351
m150504_iid=3156087_0      AATACATAAAATAAATAATTTCACCTACCATAATTTAATATTCAAGCAATTCTTATTAAC   6174
m150505_iid=2498502_0      AATACATAAAATAAATAATTTCACCTACCATAATTTAATATTCAAGCAATTCTTATTAAC   3824
m141102_iid=754227_0       AATACATAAAATAAATAATTTCACCTACCATAATTTAATATTCAAGCAATTCTTATTAAC   6386
m150507_iid=3469514_0      AATACATAAAATAAATAATTTCACCTACCATAATTTAATATTCAAGCAATTCTTATTAAC   6944
Rdo_MitoGenome             AATACATAAAATAAATAATTTCACCTACCATAATTTAATATTCAAGCAATTCTTATTAAC   7419
m141101_iid=614933_0       AATACATAAAATAAATAATTTCACCTACCATAATTTAATATTCAAGCAATTCTTATTAAC   5970
```

nad5

```
m141031_iid=347546_0       AAAGCAACATCC-CAATCCGATTTCTTAAAGCAGTTAATATACCCGCACTAAAAGACTTA   2200
m150506_iid=3127414_0      AAAGCAACATCCCCAATCCGATTTCTTAAAGCAGTTAATATACCCGCACTAAAAGACTTA   3935
m141102_iid=1245517_0      AAAGCAACATCCCCAATCCGATTTCTTAAAGCAGTTAATATA-CCGCACTAAAAGACTTA   5584
m141107_iid=1405727_0      AAAGCAACATCCCCAATCCGATTTCTTAAAGCAGTTAATATACCCGCACTAAAAGACTTA   2766
m141102_iid=987614_0       AAAGCAACATCCCCAATCCGATTTCTTAAAGCAGTTAATATACCCGCACTAAAAGACTTA   5753
m141108_iid=1701429_0      AAAGCAACATCCCCAATCCGATTTCTTAAAGCAGTTAATATACCCGCACTAAAAGACTTA   2490
m141102_iid=987286_0       AAAGCAACATCCCCAATCCGATTTCTTAAAGCAGTTAATATACCCGCACTAAAAGACTTA   3705
m150430_iid=3272125_0      AAAGCAACATCCCCAATCCGATTTCTTAAAGCAGTTAA--TACCCGCACTAAAAGACTTA   5224
m141102_iid=754225_0       AAAGCAACATCCCCAATCCGATTTCTTAAAGCAGTTAATATACCCGCACTAAAAGACTTA   6391
m141107_iid=1409934_0      AAAGCAACATCCCCAATCCGATTTCTTAAAGCAGTTAATATACCCGCACTAAAAGACTTA   6936
m150503_iid=3416920_0      AAAGCAACATCCCCAATCCGATTTCTTAAAGCAGTTAATATACCCGCACTAAAAGACTTA   5654
m141108_iid=1800121_0      AAAGCAACATCCCCAATCCGATTTCTTAAAGCAGTTAATATACCCGCACTAAAAGACTTA   5466
m141031_iid=147540_0       AAAGCAACATCCCCAATCCGATTTCTTAAAGCAGTTAATATACCCGCACTAAAAGACTTA   5811
m150507_iid=3674924_0      AAAGCAACATCCCCAATCCGATTTCTTAAAGCAGTTAATATACCCGCACTAAAAGACTTA   5960
m150503_iid=2923751_0      AAAGCAACATCCCCAATCCGATTTCTTAAAGCAGTTAATATACCCGCACTAAAAGACTTA   5798
m141031_iid=115118_0       AAAGCAACATCCCCAATCCGATTTCTTAAAGCAGTTAATATACCCGCACTAAAAGACTTA   4655
```

```
m150430_iid=2722448_0      AAAGCAACATCCCCAATCCGATTTCTTAAAGCAGTTAATATACCCGCACTAAAAGACTTA   4591
m150507_iid=3102647_0      AAAGCAACATCCCCAATCCGATTTCTTAAAGCAGTTAATATACCCGCACTAAAAGACTTA   2895
m141108_iid=1811866_0      AAAGCAACATCCCCAATCCGATTTCTTAAAGCAGTTAATATACCCGCACTAAAAGACTTA   4773
m150501_iid=2948495_0      AAAGCAACATCCCCAATCCGATTTCTTAAAGCAGTTAATATACCCGCACTAAAAGACTTA   4195
m150504_iid=3238195_0      AAAGCAACATCCCCAATCCGATTTCTTAAAGCAGTTAATATACCCGCACTAAAAGACTTA   6528
m150503_iid=3388511_0      AAAGCAACATCCCCAATCCGATTTCTTAAAGCAGTTAATATACCCGCACTAAAAGACTTA   4226
m150505_iid=3777611_0      AAAGCAACATCCCCAATCCGATTTCTTAAAGCAGTTAATATACCCGCACTAAAAGACTTA   5647
m150430_iid=2438833_0      AAAGCAACATCCCCAATCCGATTTCTTAAAGCAGTTAATATACCCGCACTAAAAGACTTA   5985
m141108_iid=1698969_0      AAAGCAACATCCCCAATCCGATTTCTTAAAGCAGTTAATATACCCGCACTAAAAGACTTA   7113
m150430_iid=3059190_0      AAAGCAACATCCCCAATCCGATTTCTTAAAGCAGTTAATATACCCGCACTAAAAGACTTA   2834
m141107_iid=1383067_0      AAAGCAACATCCCCAATCCGATTTCTTAAAGCAGTTAATATACCCGCACTAAAAGACTTA   2840
m141101_iid=597008_0       ------------------------------------------------------------   0
m150506_iid=2596496_0      AAAGCAACATCCCCAATCCGATTTCTTAAAGCAGTTAATATACCCGCACTAAAAGACTTA   7471
m141102_iid=910051_0       AAAGCAACATCCCCAATCCGATTTCTTAAAGCAGTTAATATACCCGCACTAAA--ACTTA   6959
m150505_iid=3784017_0      AAAGCAACATCCCCAATCCGATTTCTTAAAGCAGTTAATATACCCGCACTAAAAGACTTA   5760
m141101_iid=682151_0       AAAGCAACATCCCCAATCCGATTTCTTAAAGCAGTTAATATACCCGCACTAAAAGACTTA   4829
m150501_iid=3748029_0      AAAGCAACATCCCCAATCCGATTTCTTAAAGCAGTTAATATACCCGCACTAAAAGACTTA   5372
m150504_iid=2876272_0      AAAGCAACATCCCCAATCCGATTTCTTAAAGCAGTTAATATACCCGCACTAAAAGACTTA   7236
NC_042820.1                AAAGCAACATCCCCAATCCGATTTCTTAAAGCAGTTAATATACCCGCACTAAAAGACTTA   7479
m141102_iid=1228825_0      AAAGCAACATCCCCAATCCGATTTCTTAAAGCAGTTAATATACCCGCACTAAAAGACTTA   7305
m150504_iid=2876274_0      AAAGCAACATCCCCAATCCGATTTCTTAAAGCAGTTAATATACCCGCACTAAAAGACTTA   7308
m141030_iid=80036_0        AAAGCAACATCCCCAATCCGATTTCTTAAAGCAGTTAATATACCCGCACTAAAAGACTTA   7069
m150506_iid=2596494_0      AAAGCAACATCCCCAATCCGATTTCTTAAAGCAGTTAATATACCCGCACTAAAAGACTTA   7474
m150503_iid=2526862_0      AAAGCAACATCCCCAATCCGATTTCTTAAAGCAGTTAATATACCCGCACTAAAAGACTTA   7157
m150506_iid=2567000_0      AAAGCAACATCCCCAATCCGATTTCTTAAAGCAGTTAATATACCCGCACTAAAAGACTTA   5107
m150507_iid=3073271_0      AAAGCAACATCCCCAATCCGATTTCTTAAAGCAGTTAATATACCCGCACTAAAAGACTTA   6411
m150504_iid=3156087_0      AAAGCAACATCCCCAATCCGATTTCTTAAAGCAGTTAATATACCCGCACTAAAAGACTTA   6234
m150505_iid=2498502_0      AAAGCAACATCCCCAATCCGATTTCTTAAAGCAGTTAATATACCCGCACTAAAAGACTTA   3884
m141102_iid=754227_0       AAAGCAACATCCCCAATCCGATTTCTTAAAGCAGTTAATATACCCGCACTAAAAGACTTA   6446
m150507_iid=3469514_0      AAAGCAACATCCCCAATCCGATTTCTTAAAGCAGTTAATATACCCGCACTAAAAGACTTA   7004
Rdo_MitoGenome             AAAGCAACATCCCCAATCCGATTTCTTAAAGCAGTTAATATACCCGCACTAAAAGACTTA   7479
m141101_iid=614933_0       AAAGCAACATCCCCAATCCGATTTCTTAAAGCAGTTAATATACCCGCACTAAAAGACTTA   6030
```

nad5

```
m141031_iid=347546_0       AAA-TTTTGATAATAAATAACTA-ACTATAAGAAACAAGA-CT-AAACCATCCCAACCTA   2256
m150506_iid=3127414_0      AAA-TTTTGATAATAAATAACTAAACTATAAGAAACAAGACCT-AAACCATCCCAACCTA   3993
m141102_iid=1245517_0      AAA-TTTTGATAATAAATAACTAAACTATAAGAAACAAGACCTA-AACCATCCCAACCTA   5642
m141107_iid=1405727_0      AAA-TTTTGATAATAAATAACTAAACTATA-GAAACAAGACCTAAAACCATCCCAACCTA   2824
m141102_iid=987614_0       AAA-TTTTGATAATAAATAACTAAACTATAAGAAACAAGACCT-AAACCATCCCAACCTA   5811
m141108_iid=1701429_0      AAA-TTTTGATAATAAATAACTAAACTATAAGAAACAAGACCT-AAACCATCCCAACCTA   2548
m141102_iid=987286_0       AAA-TTTTGATAATAAATAACTAAACTATAAGAAACAAGACCT-AAACCATCCCAACCTA   3763
m150430_iid=3272125_0      AAAATTTTGATAATAAATAACTAAACTATAAGAAACAAGACCT-AAACCATCCCAACCTA   5283
m141102_iid=754225_0       AAA-TTTTGATAATAAATAACTAAACTATAAGAAACAAGACCT-AAACCATCCCAACCTA   6449
m141107_iid=1409934_0      AAA-TTTTGATAATAAATAACTAAACTATAAGAAACAAGACCT-AAACCATCCCAACCTA   6994
m150503_iid=3416920_0      AAA-TTTTGATAATAAATAACTAAACTATAAGAAACAAGACCT-AAACCATCCCAACCTA   5712
m141108_iid=1800121_0      AAA-TTTTGATAATAAATAACTAAACTATAAGAAACAAGACCT-AAACCATCCCAACCTA   5524
m141031_iid=147540_0       AAA-TTTTGATAATAAATAACTAAACTATAAGAAACAAGACCT-AAACCATCCCAACCTA   5869
m150507_iid=3674924_0      AAA-TTTTGATAATAAATAACTAAACTATAAGAAACAAGACCT-AAACCATCCCAACCTA   6018
m150503_iid=2923751_0      AAA-TTTTGATAATAAATAACTAAACTATAAGAAACAAGACCT-AAACCATCCCAACCTA   5856
m141031_iid=115118_0       AAA-TTTTGATAATAAATAACTAAACTATAAGAAACAAGACCT-AAACCATCCCAACCTA   4713
m150430_iid=2722448_0      AAA-TTTTGATAATAAATAACTAAACTATAAGAAACAAGACCT-AAACCATCCCAACCTA   4649
m150507_iid=3102647_0      AAA-TTTTGATAATAAATAACTAAACTATAAGAAACAAGACCT-AAACCATCCCAACCTA   2953
m141108_iid=1811866_0      AAA-TTTTGATAATAAATAACTAAACTATAAGAAACAAGACCT-AAACCATCCCAACCTA   4831
m150501_iid=2948495_0      AAA-TTTTGATAATAAATAACTAAACTATAAGAAACAAGACCT-AAACCATCCCAACCTA   4253
m150504_iid=3238195_0      AAA-TTTTGATAATAAATAACTAAACTATAAGAAACAAGACCT-AAACCATCCCAACCTA   6586
m150503_iid=3388511_0      AAA-TTTTGATAATAAATAACTAAACTATAAGAAACAAGACCT-AAACCATCCCAACCTA   4284
m150505_iid=3777611_0      AAA-TTTTGATAATAAATAACTAAACTATAAGAAACAAGACCT-AAACCATCCCAACCTA   5705
m150430_iid=2438833_0      AAA-TTTTGATAATAAATAACTAAACTATAAGAAACAAGACCT-AAACCATCCCAACCTA   6043
m141108_iid=1698969_0      AAA-TTTTGATAATAAATAACTAAACTATAAGAAACAAGACCT-AAACCATCCCAACCTA   7171
m150430_iid=3059190_0      AAA-TTTTGATAATAAATAACTAAACTATAAGAAACAAGACCT-AAACCATCCCAACCTA   2892
m141107_iid=1383067_0      AAA-TTTTGATAATAAATAACTAAACTATAAGAAACAAGACCT-AAACCATCCCAACCTA   2898
m141101_iid=597008_0       ------------------------------------------------------------   0
m150506_iid=2596496_0      AAA-TTTTGATAATAAATAACTAAACTATAAGAAACAAGACCT-AAACCATCCCAACCTA   7529
m141102_iid=910051_0       AAA-TTTTGATAATAAATAACTAAACTATAAGAAACAAGACCT-AAACCATCCCAACCTA   7017
m150505_iid=3784017_0      AAA-TTTTGATAATAAATAACTAAACTATAAGAAACAAGACCT-AAACCATCCCAACCTA   5818
m141101_iid=682151_0       AAA-TTTTGATAATAAATAACTAAACTATAAGAAACAAGACCT-AAACCATCCCAACCTA   4887
m150501_iid=3748029_0      AAA-TTTTGATAATAAATAACTAAACTATAAGAAACAAGACCT-AAACCATCCCAACCTA   5430
m150504_iid=2876272_0      AAA-TTTTGATAATAAATAACTAAACTATAAGAAACAAGACCT-AAACCATCCCAACCTA   7294
NC_042820.1                AAA-TTTTGATAATAAATAACTAAACTATAAGAAACAAGACCT-AAACCATCCCAACCTA   7537
m141102_iid=1228825_0      AAA-TTTTGATAATAAATAACTAAACTATAAGAAACAAGACCT-AAACCATCCCAACCTA   7363
m150504_iid=2876274_0      AAA-TTTTGATAATAAATAACTAAACTATAAGAAACAAGACCT-AAACCATCCCAACCTA   7366
```

```
m141030_iid=80036_0        AAA-TTTTGATAATAAATAACTAAACTATAAGAAACAAGACCT-AAACCATCCCAACCTA   7127
m150506_iid=2596494_0      AAA-TTTTGATAATAAATAACTAAACTATAAGAAACAAGACCT-AAACCATCCCAACCTA   7532
m150503_iid=2526862_0      AAA-TTTTGATAATAAATAACTAAACTATAAGAAACAAGACCT-AAACCATCCCAACCTA   7215
m150506_iid=2567000_0      AAA-TTTTGATAATAAATAACTAAACTATAAGAAACAAGACCT-AAACCATCCCAACCTA   5165
m150507_iid=3073271_0      AAA-TTTTGATAATAAATAACTAAACTATAAGAAACAAGACCT-AAACCATCCCAACCTA   6469
m150504_iid=3156087_0      AAA-TTTTGATAATAAATAACTAAACTATAAGAAACAAGACCT-AAACCATCCCAACCTA   6292
m150505_iid=2498502_0      AAA-TTTTGATAATAAATAACTAAACTATAAGAAACAAGACCT-AAACCATCCCAACCTA   3942
m141102_iid=754227_0       AAA-TTTTGATAATAAATAACTAAACTATAAGAAACAAGACCT-AAACCATCCCAACCTA   6504
m150507_iid=3469514_0      AAA-TTTTGATAATAAATAACTAAACTATAAGAAACAAGACCT-AAACCATCCCAACCTA   7062
Rdo_MitoGenome             AAA-TTTTGATAATAAATAACTAAACTATAAGAAACAAGACCT-AAACCATCCCAACCTA   7537
m141101_iid=614933_0       AAA-TTTTGATAATAAATAACTAAACTATAAGAAACAAGACCT-AAACCATCCCAACCTA   6088
                                                     nad5
                           ------------------------------------------------------------>
m141031_iid=347546_0       AAAGAATTCTAAT-AATATTAGGAGATAAAATTATTATTATTATAGATAAA-AAAATAAA   2314
m150506_iid=3127414_0      AAAGAATTCTAAT-AATATTAGGAGATAAAATTATTATTATTATAGATAAAACAAATAAA   4052
m141102_iid=1245517_0      AAAGAATTCTAAT-AATATTAGGAGATAAAATTATTATTATTATAGATAAAAA--ATAAA   5699
m141107_iid=1405727_0      AAAGAATTCTAAATAATATTAGGAGATAAAATTATTATTATTATAGATAAAACAAATAAA   2884
m141102_iid=987614_0       AAAGAATTCTAAT-AATATTAGGAGATAAAATTATTATTATTATAGATAAAACAAATAAA   5870
m141108_iid=1701429_0      AAAGAATTCTAAT-AATATTAGGAGATAAAATTATTATTATTATAGATAAAACAAATAAA   2607
m141102_iid=987286_0       AAAGAATTCTAAT-AATATTAGGAGATAAAATTATTATTATTATAGATAAAACAAATAAA   3822
m150430_iid=3272125_0      AAAGAATTCTAAT-AATATTAGGAGATAAAATTATTATTATTATAGATAAAACAAATAAA   5342
m141102_iid=754225_0       AAAGAATTCTAAT-AATATTAGGAGATAAAATTATTATTATTATAGATAAAACAAATAAA   6508
m141107_iid=1409934_0      AAAGAATTCTAAT-AATATTAGGAGATAAAATTATTATTATTATAGATAAAACAAATAAA   7053
m150503_iid=3416920_0      AAAGAATTCTAAT-AATATTAGGAGATAAAATTATTAT--TTATAGATAAAACAAATAAA   5769
m141108_iid=1800121_0      AAAGAATTCTAAT-AAT-TTAGGAGATAAAATTATTATTATTATAGATAAAACAAATAAA   5582
m141031_iid=147540_0       AAAGAATTCTAAT-AATATTAGGAGATAAAATTATTATTATTATAGATAAAACAAATAAA   5928
m150507_iid=3674924_0      AAAGAATTCTAAT-AATATTAGGAGATAAAATTATTATTATTATAGATAAAACAAATAAA   6077
m150503_iid=2923751_0      AAAGAATTCTAAT-AATATTAGGAGATAAAATTATTATTATTATAGATAAAACAAATAAA   5915
m141031_iid=115118_0       AAAGAATTCTAAT-AATATTAGGAGATAAAATTATTATTATTATAGATAAAACAAATAAA   4772
m150430_iid=2722448_0      AAAGAATTCTAAT-AATATTAGGAGATAAAATTATTATTATTATAGATAAAACAAATAAA   4708
m150507_iid=3102647_0      AAAGAATTCTAAT-AATATTAGGAGATAAAATTATTATTATTATAGATAAAACAAATAAA   3012
m141108_iid=1811866_0      AAAGAATTCTAAT-AATATTAGGAGATAAAATTATTATTATTATAGATAAAACAAATAAA   4890
m150501_iid=2948495_0      AAAGAATTCTAAT-AATATTAGGAGATAAAATTATTATTATTATAGATAAAACAAATAAA   4312
m150504_iid=3238195_0      AAAGAATTCTAAT-AATATTAGGAGATAAAATTATTATTATTATAGATAAAACAAATAAA   6645
m150503_iid=3388511_0      AAAGAATTCTAAT-AATATTAGGAGATAAAATTATTATTATTATAGATAAAACAAATAAA   4343
m150505_iid=3777611_0      AAAGAATTCTAAT-AATATTAGGAGATAAAATTATTATTATTATAGATAAAACAAATAAA   5764
m150430_iid=2438833_0      AAAGAATTCTAAT-AATATTAGGAGATAAAATTATTATTATTATAGATAAAACAAATAAA   6102
m141108_iid=1698969_0      AAAGAATTCTAAT-AATATTAGGAGATAAAATTATTATTATTATAGATAAAACAAATAAA   7230
m150430_iid=3059190_0      AAAGAATTCTAAT-AATATTAGGAGATAAAATTATTATTATTATAGATAAAACAAATAAA   2951
m141107_iid=1383067_0      AAAGAATTCTAAT-AATATTAGGAGATAAAATTATTATTATTATAGATAAAACAAATAAA   2957
m141101_iid=597008_0       ------------------------------ATTATTATTATTATAGATAAAACAAATAAA   30
m150506_iid=2596496_0      AAAGAATTCTAAT-AATATTAGGAGATAAAATTATTATTATTATAGATAAAACAAA-AAA   7587
m141102_iid=910051_0       AAAGAATTCTAAT-AATATTAGGAGATAAAATTATTATTATTATAGATAAAACAAATAAA   7076
m150505_iid=3784017_0      AAAGAATTCTAAT-AATATTAGGAGATAAAATTATTATTATTATAGATAAAACAAATAAA   5877
m141101_iid=682151_0       AAAGAATTCTAAT-AATATTAGGAGATAAAATTATTATTATTATAGATAAAACAAATAAA   4946
m150501_iid=3748029_0      AAAGAATTCTAAT-AATATTAGGAGATAAAATTATTATTATTATAGATAAAACAAATAAA   5489
m150504_iid=2876272_0      AAAGAATTCTAAT-AATATTAGGAGATAAAATTATTATTATTATAGATAAAACAAATAAA   7353
NC_042820.1                AAAGAATTCTAAT-AATATTAGGAGATAAAATTATTATTATTATAGATAAAACAAATAAA   7596
m141102_iid=1228825_0      AAAGAATTCTAAT-AATATTAGGAGATAAAATTATTATTATTATAGATAAAACAAATAAA   7422
m150504_iid=2876274_0      AAAGAATTCTAAT-AATATTAGGAGATAAAATTATTATTATTATAGATAAAACAAATAAA   7425
m141030_iid=80036_0        AAAGAATTCTAAT-AATATTAGGAGATAAAATTATTATTATTATAGATAAAACAAATAAA   7186
m150506_iid=2596494_0      AAAGAATTCTAAT-AATATTAGGAGATAAAATTATTATTATTATAGATAAAACAAATAAA   7591
m150503_iid=2526862_0      AAAGAATTCTAAT-AATATTAGGAGATAAAATTATTATTATTATAGATAAAACAAATAAA   7274
m150506_iid=2567000_0      AAAGAATTCTAAT-AATATTAGGAGATAAAATTATTATTATTATAGATAAAACAAATAAA   5224
m150507_iid=3073271_0      AAAGAATTCTAAT-AATATTAGGAGATAAAATTATTATTATTATAGATAAAACAAATAAA   6528
m150504_iid=3156087_0      AAAGAATTCTAAT-AATATTAGGAGATAAAATTATTATTATTATAGATAAAACAAATAAA   6351
m150505_iid=2498502_0      AAAGAATTCTAAT-AATATTAGGAGATAAAATTATTATTATTATAGATAAAACAAATAAA   4001
m141102_iid=754227_0       AAAGAATTCTAAT-AATATTAGGAGATAAAATTATTATTATTATAGATAAAACAAATAAA   6563
m150507_iid=3469514_0      AAAGAATTCTAAT-AATATTAGGAGATAAAATTATTATTATTATAGATAAAACAAATAAA   7121
Rdo_MitoGenome             AAAGAATTCTAAT-AATATTAGGAGATAAAATTATTATTATTATAGATAAAACAAATAAA   7596
m141101_iid=614933_0       AAAGAATTCTAAT-AATATTAGGAGATAAAATTATTATTATTATAGATAAAACAAATAAA   6147
                                                             .****   ***********  .  * ***
                                                     nad5
                           ------------------------------------------------------------>
m141031_iid=347546_0       ACCACTAATAAAATAAAACGATTTAATATTAAATCCCCGTGTATATAATCCTCTCTATAA   2374
m150506_iid=3127414_0      ACCACTAATAAAATAAAACGATTTAATATTAAATCCCCGTGTATATAATCCTCTCTATAA   4112
m141102_iid=1245517_0      ACCAA-TCTAAAATAAAACGATTTAATATTAAATCCCCGTGTATATAATCCTCTCTATAA   5758
m141107_iid=1405727_0      ACCACTAATAAAATAAAACGATTTAATATTAAATCCCCGTGTATATAATCCTCTCTATAA   2944
m141102_iid=987614_0       ACCACTAATAAAATAAAACGATTTAATATTAAATCCCCGTGTATATAATCCTCTCTATAA   5930
m141108_iid=1701429_0      ACCACTAATAAAATAAAACGATTTAATATTAAATCCCCGTGTATATAATCCTCTCTATAA   2667
m141102_iid=987286_0       ACCACTAATAAAATAAAACGATTTAATATTAAATCCCCGTGTATATAATCCTCTCTATAA   3882
m150430_iid=3272125_0      ACCACTAATAAAATAAAACGATTTAATATTAAATCCCCGTGTATATAATCCTCTCTATAA   5402
```

```
m141102_iid=754225_0        ACCACTAATAAAATAAAACGATTTAATATTAAATCCCCGTGTATATAATCCTCTCTATAA   6568
m141107_iid=1409934_0       A---CTAATAAAATAAAACGATTTAATATTAAATCCCCGTGTATATAATCCTCTCTATAA   7110
m150503_iid=3416920_0       ACCACTAATAAAATAAAACGATTTAATATTAAATCCCCGTGTATATAATCCTCTCTATAA   5829
m141108_iid=1800121_0       ACCACTAATAAAATAAAACGATTTAATATTAAATCCCCGTGTATATAATCCTCTCTATAA   5642
m141031_iid=147540_0        ACCACTAATAAAATAAAACGATTTAATATTAAATCCCCGTGTATATAATCCTCTCTATAA   5988
m150507_iid=3674924_0       ACCACTAATAAAATAAAACGATTTAATATTAAATCCCCGTGTATATAATCCTCTCTATAA   6137
m150503_iid=2923751_0       ACCACTAATAAAATAAAACGATTTAATATTAA-ATCCCGTGTATATAATCCTCTCTATAA   5974
m141031_iid=115118_0        ACCACTAATAAAATAAAACGATTTAATATTAAATCCCCGTGTATATAATCCTCTCTATAA   4832
m150430_iid=2722448_0       ACCACTAATAAAATAAAACGATTTAATATTAAATCCCCGTGTATATAATCCTCTCTATAA   4768
m150507_iid=3102647_0       ACCACTAATAAAATAAAACGATTTAATATTAAATCCCCGTGTATATAATCCTCTCTATAA   3072
m141108_iid=1811866_0       ACCACTAATAAAATAAAACGATTTAATATTAAATCCCCGTGTATATAATCCTCTCTATAA   4950
m150501_iid=2948495_0       ACCACTAATAAAATAAAACGATTTAATATTAAATCCCCGTGTATATAATCCTCTCTATAA   4372
m150504_iid=3238195_0       ACCACTAATAAAATAAAACGATTTAATATTAAATCCCCGTGTATATAATCCTCTCTATAA   6705
m150503_iid=3388511_0       ACCACTAATAAAATAAAACGATTTAATATTAAAT-CCCGTGTATATAATCCTCTCTATAA   4402
m150505_iid=3777611_0       ACCACTAATAAAATAAAACGATTTAATATTAAATCCCCGTGTATATAATCCTCTCTATAA   5824
m150430_iid=2438833_0       ACCACTAATAAAATAAAACGATTTAATATTAAATCCCCGTGTATATAATCCTCTCTATAA   6162
m141108_iid=1698969_0       ACCACTAATAAAATAAAACGATTTAATATTAAATCCCCGTGTATATAATCCTCTCTATAA   7290
m150430_iid=3059190_0       ACCACTAATAAAATAAAACGATTTAATATTAAATCCCCGTGTATATAATCCTCTCTATAA   3011
m141107_iid=1383067_0       ACCACTAATAAAATAAAACGATTTAATATTAAATCCCCGTGTATATAATCCTCTCTATAA   3017
m141101_iid=597008_0        ACCACTAATAAAATAAAACGATTA-ATATTAAATCCCCGTGTATATAATCCTCTCTATAA   89
m150506_iid=2596496_0       ACCACTAATAAAATAAAACGATTTAATATTAAATCCCCGTGTATATAATCCTCTCTATAA   7647
m141102_iid=910051_0        ACCACTAATAAAATAAAACGATTTAATAT-TAATCCCCGTGTATATAATCCTCTCTATAA   7135
m150505_iid=3784017_0       ACCACTAATAAAATAAAACGATTTAATATTAAATCCCCGTGTATATAATCCTCTCTATAA   5937
m141101_iid=682151_0        ACCACTAATAAAATAAAACGATTTAATATTAAATCCCCGTGTATATAATCCTCTCTATAA   5006
m150501_iid=3748029_0       ACCACTAATAAAATAAAACGATTTAATATTAAATCCCCGTGTATATAATCCTCTCTATAA   5549
m150504_iid=2876272_0       ACCACTAATAAAATAAAACGATTTAATATTAAATCCCCGTGTATATAATCCTCTCTATAA   7413
NC_042820.1                 ACCACTAATAAAATAAAACGATTTAATATTAAATCCCCGTGTATATAATCCTCTCTATAA   7656
m141102_iid=1228825_0       ACCACTAATAAAATAAAACGATTTAATATTAAATCCCCGTGTATATAATCCTCTCTATAA   7482
m150504_iid=2876274_0       ACCACTAATAAAATAAAACGATTTAATATTAAATCCCCGTGTATATAATCCTCTCTATAA   7485
m141030_iid=80036_0         ACCACTAATAAAATAAAACGATTTAATATTAAATCCCCGTGTATATAATCCTCTCTATAA   7246
m150506_iid=2596494_0       ACCACTAATAAAATAAAACGATTTAATATTAAATCCCCGTGTATATAATCCTCTCTATAA   7651
m150503_iid=2526862_0       ACCACTAATAAAATAAAACGATTTAATATTAAATCCCCGTGTATATAATCCTCTCTATAA   7334
m150506_iid=2567000_0       ACCACTAATAAAATAAAACGATTTAATATTAAATCCCCGTGTATATAATCCTCTCTATAA   5284
m150507_iid=3073271_0       ACCACTAATAAAATAAAACGATTTAATATTAAATCCCCGTGTATATAATCCTCTCTATAA   6588
m150504_iid=3156087_0       ACCACTAATAAAATAAAACGATTTAATATTAAATCCCCGTGTATATAATCCTCTCTATAA   6411
m150505_iid=2498502_0       ACCACTAATAAAATAAAACGATTTAATATTAAATCCCCGTGTATATAATCCTCTCTATAA   4061
m141102_iid=754227_0        ACCACTAATAAAATAAAACGATTTAATATTAAATCCCCGTGTATATAATCCTCTCTATAA   6623
m150507_iid=3469514_0       ACCACTAATAAAATAAAACGATTTAATATTAAATCCCCGTGTATATAATCCTCTCTATAA   7181
Rdo_MitoGenome              ACCACTAATAAAATAAAACGATTTAATATTAAATCCCCGTGTATATAATCCTCTCTATAA   7656
m141101_iid=614933_0        ACCACTAATAAAATAAAACGATTTAATATTAAATCCCCGTGTATATAATCCTCTCTATAA   6207
                            *    . :.***************:   nad5      *************************

m141031_iid=347546_0        A-AAACTACTATAGAAGAAATAAA--AAAAACAAACCTTATAAAAATTAAACTTATAAAA   2431
m150506_iid=3127414_0       A-ACTATACTATAGAAGAAATAAA--AAAAACAAACCTTATAAAAATTAAACTTATAAAA   4169
m141102_iid=1245517_0       --AAACTACTATAGAAGAAATAAAA---AAACAA-ACTTATAAAAATTAAACTTATAAAA   5812
m141107_iid=1405727_0       AAAAACTACTATAGAAGAAATA----AAAAACAAACCTTATAAAAATTAAACTTATAAAA   3000
m141102_iid=987614_0        A-AAACTACTATAGAAGAAATAAA--AAAAACAAACCTTATAAAAATTAAACTTATAAAA   5987
m141108_iid=1701429_0       A-AAACTACTATAGAAGAAATAA---AAAAACAAACCTTATAAAAATTAAACTTATAAAA   2723
m141102_iid=987286_0        A-AAACTACTATAGAAGAAATAAA--AAAAACAAACCTTATAAAAATTAAACTTATAAAA   3939
m150430_iid=3272125_0       A-AAACTACTATAGAAGAAATAA---AAAAACAAACCTTATAAAAATTAAACTTATAAAA   5458
m141102_iid=754225_0        A-AAACTACTATAGAAGAAATAAA--AAAAACAAACCTTATAAAAATTAAACTTATAAAA   6625
m141107_iid=1409934_0       A-AAACTACTATAGAAGAAATAAA--AAAAACAAACCTTATAAAAATTAAACTTATAAAA   7167
m150503_iid=3416920_0       A-AAACTACTATAGAAGAAATAAA--AAAAACAAACCTTATAAAAATTAAACTTATAAAA   5886
m141108_iid=1800121_0       A-AAACTACTATAGAAGAAATAATAAAAAAACAAACCTTATAAAAATTAAACTTATAAAA   5701
m141031_iid=147540_0        A-AAACTACTATAGAAGAAATAAA--AAAAACAAACCTTATAAAAATTAAACTTATAAAA   6045
m150507_iid=3674924_0       A-AAACTACTATAGAAGAAATAAA--AAAAACAAACCTTATAAAAATTAAACTTATAAAA   6194
m150503_iid=2923751_0       A-AAACTACTATAGAAGAAATAA---AAAAACAAACCTTATAAAAATTAAACTTATAAAA   6030
m141031_iid=115118_0        A-AAACTACTATAGAAGAAATAAA--AAAAACAAACCTTATAAAAATTAAACTTATAAAA   4889
m150430_iid=2722448_0       A-AAACTACTATAGAAGAAATAAA--AAAAACAAACCTTATAAAAATTAAACTTATAAAA   4825
m150507_iid=3102647_0       A-AAACTACTATAGAAGAAATAAA--AAAAACAAACCTTATAAAAATTAAACTTATAAAA   3129
m141108_iid=1811866_0       A-AAACTACTATAGAAGAAATAAA--AAAAACAAACCTTATAAAAATTAAACTTATAAAA   5007
m150501_iid=2948495_0       A-AAACTACTATAGAAGAAATAAA--AAAAACAAACCTTATAAA-ATTAAACTTATAAAA   4428
m150504_iid=3238195_0       A-AAACTACTATAGAAGAAATAAA--AAAAACAAACCTTATAAAAATTAAACTTATAAAA   6762
m150503_iid=3388511_0       A-AAACTACTATAGAAGAAAT-AA--AAAAACAAACCTTATAAAAATTAAACTTATAAAA   4458
m150505_iid=3777611_0       A-AAACTACTATAGAAGAAATAAA--AAAAACAAACCTTATAAAAATTAAACTTATAAAA   5881
m150430_iid=2438833_0       A-AAACTACTATAGAAGAAATAAA--AAAAACAAACCTTATAAAAATTAAACTTATAAAA   6219
m141108_iid=1698969_0       A-AAACTACTATAGAAGAAATAAA--AAAAACAAACCTTATAAAAATTAAACTTATAAAA   7347
m150430_iid=3059190_0       A-AAACTACTATAGAAGAAATAAA--AAAAACAAACCTTATAAAAATTAAACTTATAAAA   3068
m141107_iid=1383067_0       A-AAACTACTATAGAAGAAATAAA--AAAAACAAACCTTATAAAAATTAAACTTATAAAA   3074
m141101_iid=597008_0        A-AAACTACTATAGAAGAAATAA----AAAACAAACCTTATAAAAATTAAACTTATAAAA   144
m150506_iid=2596496_0       A-AAACTACTATAGAAGAAATAAA--AAAAACAAACCTTATAAAAATTAAACTTATAAAA   7704
```

```
m141102_iid=910051_0       A-AAACTACTATAGAAGAAATAA---AAAAACAAACCTTATAAAAATTAAACTTATAAAA   7191
m150505_iid=3784017_0      A-AAACTACTATAGAAGAAATAAA--AAAAACAAACCTTATAAAAATTAAACTTATAAAA   5994
m141101_iid=682151_0       A-AAACTACTATAGAAGAAATAAA--AAAAACAAACCTTATAAAAATTAAACTTATAAAA   5063
m150501_iid=3748029_0      A-AAACTACTATAGAAGAAATAAA--AAAAACAAACCTTATAAAAATTAAACTTATAAAA   5606
m150504_iid=2876272_0      A-AAACTACTATAGAAGAAATAAA--AAAAACAAACCTTATAAAAATTAAACTTATAAAA   7470
NC_042820.1                A-AAACTACTATAGAAGAAATAAA--AAAAACAAACCTTATAAAAATTAAACTTATAAAA   7713
m141102_iid=1228825_0      A-AAACTACTATAGAAGAAATAAA--AAAAACAAACCTTATAAAAATTAAACTTATAAAA   7539
m150504_iid=2876274_0      A-AAACTACTATAGAAGAAAT-AA--AAAAACAAACCTTATAAAAATTAAACTTATAAAA   7541
m141030_iid=80036_0        A-AAACTACTATAGAAGAAATAAA--AAAAACAAACCTTATAAAAATTAAACTTATAAAA   7303
m150506_iid=2596494_0      A-AAACTACTATAGAAGAAATAAA--AAAAACAAACCTTATAAAAATTAAACTTATAAAA   7708
m150503_iid=2526862_0      A-AAACTACTATAGAAGAAATAAA--AAAAACAAACCTTATAAAAATTAAACTTATAAAA   7391
m150506_iid=2567000_0      A-AAACTACTATAGAAGAAATAAA--AAAAACAAACCTTATAAAAATTAAACTTATAAAA   5341
m150507_iid=3073271_0      A-AAACTACTATAGAAGAAATAAA--AAAAACAAACCTTATAAAAATTAAACTTATAAAA   6645
m150504_iid=3156087_0      A-AAACTACTATAGAAGAAATAAA--AAAAACAAACCTTATAAAAATTAAACTTATAAAA   6468
m150505_iid=2498502_0      A-AAACTACTATAGAAGAAATAAA--AAAAACAAACCTTATAAAAATTAAACTTATAAAA   4118
m141102_iid=754227_0       A-AAACTACTATAGAAGAAATAA---AAAAACAAACCTTATAAAAATTAAACTTATAAAA   6679
m150507_iid=3469514_0      A-AAACTACTATAGAAGAAATAA---AAAAACAAACCTTATAAAAATTAAACTTATAAAA   7237
Rdo_MitoGenome             A-AAACTACTATAGAAGAAATAAA--AAAAACAAACCTTATAAAAATTAAACTTATAAAA   7713
m141101_iid=614933_0       A-AAACTACTATAGAAGAAATAA---AAAAACAAACCTTATAAAAATTAAACTTATAAAA   6263
                             *.:.***************          .********* ***************
                                                     nad5

m141031_iid=347546_0       TCAAAATATAAAGAAAAT-TAATT-AAAGAAGAATTTAAAGAA--AAATTCATATTCAAC   2487
m150506_iid=3127414_0      TCAAAATATAAAGAAAAATTAATT-AAAGAAGAATTTAAAGAAAACAATTCATATTCAAC   4228
m141102_iid=1245517_0      TCAAAATATAAAGAAAAATTAATT-AAAGAAGAATTTAAAG----AAATTCATATTCAAC   5867
m141107_iid=1405727_0      TCAAAATATAAAGAAAAATTAATTAAAGAAGAATTTAAAGAAAACAATTCATATTCAAC    3060
m141102_iid=987614_0       TCAAAATATAAAGAAAAATTAATT-AAAGAAGAATTTAAAGAAAACAATTCATATTCAAC   6046
m141108_iid=1701429_0      TCAAAATATAAAGAAAAATTA-ATTAAAGAAGAATTTAAAGAAAACAATTCATATTCAAC   2782
m141102_iid=987286_0       TCAAAATATAAAGAAAAATTA-ATTAAAGAAGAATTTAAAGAAAACAATTCATATTCAAC   3998
m150430_iid=3272125_0      TCAAAATATAAAGAAA-ATTA-ATTAAAGAAGAATTTAAAGAAAACAATTCATATTCAAC   5516
m141102_iid=754225_0       TCAAAATATAAAGAAAAATTA-ATTAAAGAAGAATTTAAAGAAAACAATTCATATTCAAC   6684
m141107_iid=1409934_0      TCAAAATATAAAGAAAAATTA-ATTAAAGAAGAATTTAAAGAAAACAATTCATATTCAAC   7226
m150503_iid=3416920_0      TCAAAATATAAAGAAAAATTA-ATTAAAGAAGAATTTAAAGAAAACAATTCATATTCAAC   5945
m141108_iid=1800121_0      TCAAAATATAAAGAAAAATTAAT-TAAAGAAGAATTTAA-AGAAACAATTCATATTCAAC   5759
m141031_iid=147540_0       TCAAAATATAAAGAAAAATTAATT-AAAGAAGAATTTAAAGAAAACAATTCATATTCAAC   6104
m150507_iid=3674924_0      TCAAAATATAAAGAAAAATT-AATTAAAGAAGAATTTAAAGAAAACAATTCATATTCAAC   6253
m150503_iid=2923751_0      TCAAAATATAAAGAAAAT--TAATTAAAGAAGAATTTAAAGAAAACAATTCATATTCAAC   6088
m141031_iid=115118_0       TCAAAATATAAAGAAAAATTAAT-TAAAGAAGAATTTAAAGAAAACAATTCATATTCAAC   4948
m150430_iid=2722448_0      TCAAAATATAAAGAAAAATTAATTAAAAAGAGAATTTAAAGAAAACAATTCATATTCAAC   4885
m150507_iid=3102647_0      TCAAAATATAAAGAAAA-TTAATT-AAAGAAGAATTTAAA-GAAACAATTCATATTCAAC   3186
m141108_iid=1811866_0      TCAAAATATAAAGAAAAATTAATT-AAAGAAGAATTTAAAGAAAACAATTCATATTCAAC   5066
m150501_iid=2948495_0      TCAAAATATAAAGAAAAATTAATT-AAAGAAGAATTTAAAGAAAACAATTCATATTCAAC   4487
m150504_iid=3238195_0      TCAAAATATAAAGAAAAATTAATT-AAAGAAGAATTTAAAGAAAACAATTCATATTCAAC   6821
m150503_iid=3388511_0      TCAAAATATAAAGAAAAATTAATT-AAAGAAGAATTTAAAGAAAACAATTCATATTCAAC   4517
m150505_iid=3777611_0      TCAAAATATAAAGAAAAATTAATT-AAAGAAGAATTTAAAGAAAACAATTCATATTCAAC   5940
m150430_iid=2438833_0      TCAAAATATAAAGAAAAATTAATT-AAAGAAGAATTTAAAGAAAACAATTCATATTCAAC   6278
m141108_iid=1698969_0      TCAAAATATAAAGAAAAATTAATT-AAAGAAGAATTTAAAGAAAACAATTCATATTCAAC   7406
m150430_iid=3059190_0      TCAAAATATAAAGAAAAATTAATT-AAAGAAGAATTTAAAGAAAACAATTCATATTCAAC   3127
m141107_iid=1383067_0      TCAAAT-ATAAAGAAAAATTAATT-AAAGAAGAATTTAAAGAAAACAATTCATATTCAAC   3132
m141101_iid=597008_0       TCAAAATATAAAGAAAAATTAATT-AAAGAAGAATTTAAAG-AAACAATTCATATTCAAC   202
m150506_iid=2596496_0      TCAAAATATAAAGAAAAATTAATT-AAAGAAGAATTTAAAGAAAACAATTCATATTCAAC   7763
m141102_iid=910051_0       TCAAAATATAAAGAAAAATTAATT-AAAGAAGAATTTAAAGAAAACAATTCATATTCAAC   7250
m150505_iid=3784017_0      TCAAAATATAAAGAAAAATTAATT-AAAGAAGAATTTAAAGAAAACAATTCATATTCAAC   6053
m141101_iid=682151_0       TCAAAATATAAAGAAAAATTAATT-AAAGAAGAATTTAAAGAAAACAATTCATATTCAAC   5122
m150501_iid=3748029_0      TCAAAATATAAAGAAAAATTAATT-AAAGAAGAATTTAAAGAAAACAATTCATATTCAAC   5665
m150504_iid=2876272_0      TCAAAATATAAAGAAAAATTAATT-AAAGAAGAATTTAAAGAAAACAATTCATATTCAAC   7529
NC_042820.1                TCAAAATATAAAGAAAAATTAATT-AAAGAAGAATTTAAAGAAAACAATTCATATTCAAC   7772
m141102_iid=1228825_0      TCAAAATATAAAGAAAAATTAATT-AAAGAAGAATTTAAAGAAAACAATTCATATTCAAC   7598
m150504_iid=2876274_0      TCAAAATATAAAGAAAAATTAATT-AAAGAAGAATTTAAAGAAAACAATTCATATTCAAC   7600
m141030_iid=80036_0        TCAAAATATAAAGAAAAATTAATT-AAAGAAGAATTTAAAGAAAACAATTCATATTCAAC   7362
m150506_iid=2596494_0      T------AAAAAGAAAAATTAATT-AAAGAAGAATTTAAAGAAAACAATTCATATTCAAC   7761
m150503_iid=2526862_0      TCAAAATATAAAGAAAAATTAATT-AAAGAAGAATTTAAAGAAAACAATTCATATTCAAC   7450
m150506_iid=2567000_0      TCAAAATATAAAGAAAAATTAATT-AAAGAAGAATTTAAAGAAAACAATTCATATTCAAC   5400
m150507_iid=3073271_0      TCAAAATATAAAGAAAAATTAATT-AAAGAAGAATTTAAAGAAAACAATTCATATTCAAC   6704
m150504_iid=3156087_0      TCAAAATATAAAGAAAAATTAATT-AAAGAAGAATTTAAAGAAAACAATTCATATTCAAC   6527
m150505_iid=2498502_0      TCAAAATATAAAGAAAAATTAATT-AAAGAAGAATTTAAAGAAAACAATTCATATTCAAC   4177
m141102_iid=754227_0       TCAAAATATAAAGAAAAATTAATT-AAAGAAGAATTTAAAGAAAACAATTCATATTCAAC   6738
m150507_iid=3469514_0      TCAAAATATAAAGAAAAATTAATT-AAAGAAGAATTTAAAGAAAACAATTCATATTCAAC   7296
Rdo_MitoGenome             TCAAAATATAAAGAAAAATTAATT-AAAGAAGAATTTAAAGAAAACAATTCATATTCAAC   7772
m141101_iid=614933_0       TCAAAATATAAAGAAAAATTAATT-AAAGAAGAATTTAAAGAAAACAATTCATATTCAAC   6322
                           *      *:*******      :          *****     .**************
                                                     nad5
```

```
m141031_iid=347546_0      AAATAATGAAAAATCAAGGATTATAAAATAAAGAGATAAAAAG-AAAAAAAAAAATGAACA   2546
m150506_iid=3127414_0     AAATAATGAA-AATCAAGGATTATAAAATAAAGAGATAAAAAA-AA-AAAAAAATGAACA   4285
m141102_iid=1245517_0     AAATAATGAAAAATCAAGGATTATAAAAT-AAGAGATAAAAAGAAAAAAAAAAAATGAACA   5926
m141107_iid=1405727_0     AAATAATGAAAAATCAAGGTTAAAAATAA--AGAGAAAAAGAAAAAAAAAAAAAATGAACA   3118
m141102_iid=987614_0      AAATAATGAAAAATCAAGGATTATAAAATAAAGAGATAAAAAGAAAAAAAAAAAATGAACA   6106
m141108_iid=1701429_0     AAATAATGAAAAATCAAGGATTATAAAATAAAGAGATAAAAAGAAAAAAAAAAAATGAACA   2842
m141102_iid=987286_0      AAATAATGAAAAATCAAGGATTATAAAATAAAGAGATAAAAAGAAAAAAAAAAAATGAACA   4058
m150430_iid=3272125_0     AAATAATGAAAAATCAAGGATTATAAAATAAAGAGATAAAAAGAAAAAAAAAAAATGAACA   5576
m141102_iid=754225_0      AAATAATGAAAAATCAAGGATTATAAAATAAAGAGATAAAAAGAAAAAAAAAAAATGAACA   6744
m141107_iid=1409934_0     AAATAATGAAAAATCAAGGATTATAAAATAAAGAGATAAAAAGAAAAA-AAAAATGAACA   7285
m150503_iid=3416920_0     AAATAATGAAAAATCAAGGATTATAAAATAAAGAGATAAAAAGAAAAAAAAAAAATGAACA   6005
m141108_iid=1800121_0     AAATAATGAAAAATCAAGGATTATAAAATAAAGAGATAAAAAGAAAAAAAAAAAATGAACA   5819
m141031_iid=147540_0      AAATAATGAAAAATCAAGGATTATAAAATAAAGAGATAAAAAGAAAAAAAAAAAATGAACA   6164
m150507_iid=3674924_0     AAATAATGAAAAATCAAGGATTATAAAATAAAGAGATAAAAAGAAAAAAAAAAAATGAACA   6313
m150503_iid=2923751_0     AAATAATGAAAAATCAAGGATTATAAAATAAAGAGATAAAAAGAAAAA-AAAAATGAACA   6147
m141031_iid=115118_0      AAATAATGAAAAATCAAGGATTATAAAATAAAGAGATAAAAAGAAAAAAAAAAAATGAACA   5008
m150430_iid=2722448_0     AAATAATGAAAAATCAAGGATTATAAAAA-AAGAGATAAAAAGAAA-AAAAAAATGAACA   4943
m150507_iid=3102647_0     AAATAATGAAAAATCAAGGATTATAAAATAAAGAGATAAAAAGAAAAA-AAAAATGAACA   3245
m141108_iid=1811866_0     AAATAATGAAAAATCAAGGATTATAAAATAAAGAGATAAAAAGAAAAA-AAAAATGAACA   5125
m150501_iid=2948495_0     AAATAATGAAAAATCAAGGATTATAAAATAAAGAGATAAAA-GAAAAAAAAAAAATGAACA   4546
m150504_iid=3238195_0     AAATAATGAAAAATCAAGGATTATAAAATAAAGAGATAAAAAGAAAAAAAAAAAATGAACA   6881
m150503_iid=3388511_0     AAATAATGAAAAATCAAGGATTATAAAATAAAGAGATAAAAAG-AAAAAAAAAAATGAACA   4576
m150505_iid=3777611_0     AAATAATGAAAAATCAAGGATTATAAAATAAAGAGATAAAAAG-AAAAAAAAAAATGAACA   5999
m150430_iid=2438833_0     AAATAATGAAAAATCAAGGATTATAAAATAAAGAGATAAAAAG-AAAAAAAAAAATGAACA   6337
m141108_iid=1698969_0     AAATAATGAAAAATCAAGGATTATAAAATAAAGAGATAAAAAGAAAAAAAAAAAATGAACA   7466
m150430_iid=3059190_0     AAATAATGAAAAATCAAGGATTATAAAATAAAGAGATAAAAAGAAAAAAAAAAAATGAACA   3187
m141107_iid=1383067_0     AAATAATGAAAAATCAAGGATTATAAAATAAAGAGATAAAAAGAAAAA-AAAAATGAACA   3191
m141101_iid=597008_0      AAATAATGAAAAATCAAGGATTATAAAATAAAGAGATAAAAAGAAAAAAAAAAAATGAACA   262
m150506_iid=2596496_0     AAATAATGAAAAATCAAGGATTATAAAATAAAGAGATAAAAAGAAAA-AAAAAAATGAACA   7822
m141102_iid=910051_0      AAATAATGAAAAATCAAGGATATAA-AATAAAGAGATAAAAAGAA---AAAAAAATGAACA   7306
m150505_iid=3784017_0     AAATAATGAAAAATCAAGGATTATAAAATAAAGAGATAAAAAGAAAAAAAAAAAATGAACA   6113
m141101_iid=682151_0      AAATAATGAAAAATCAAGGATTATAAAATAAAGAGATAAAAAG-AAAAAAAAAAATGAACA   5181
m150501_iid=3748029_0     AAATAATGAAAAATCAAGGATTATAAAATAAAGAGATAAAAAGAAAAAAAAAAAATGAACA   5725
m150504_iid=2876272_0     AAATAATGAAAAATCAAGGATTATAAAATAAAGAGATAAAAAG-AAAAAAAAAAATGAACA   7588
NC_042820.1               AAATAATGAAAAATCAAGGATTATAAAATAAAGAGATAAAAAGAAAAAAAAAAAATGAACA   7832
m141102_iid=1228825_0     AAATAATGAAAAATCAAGGATTATAAAATAAAGAGATAAAAAG-AAAAAAAAAAATGAACA   7657
m150504_iid=2876274_0     AAATAATGAAAAATCAAGGATTATAAAATAAAGAGATAAAAAG-AAAAAAAAAAATGAACA   7659
m141030_iid=80036_0       AAATAATGAAAAATCAAGGATTATAAAATAAAGAGATAAAAAGAAAAAAAAAAAATGAACA   7422
m150506_iid=2596494_0     AAATAATGAAAAATCAAGGATTATAAAATAAAGAGATAAAAAGAAAAAAAAAAAATGAACA   7821
m150503_iid=2526862_0     AAATAATGAAAAATCAAGGATTATAAAATAAAGAGATAAAAAG-AAAAAAAAAAATGAACA   7509
m150506_iid=2567000_0     AAATAATGAAAAATCAAGGATTATAAAATAAAGAGATAAAAAGAAAAAAAAAAAATGAACA   5460
m150507_iid=3073271_0     AAATAATGAAAAATCAAGGATTATAAAATAAAGAGATAAAAAG-AAAAAAAAAAATGAACA   6763
m150504_iid=3156087_0     AAATAATGAAAAATCAAGGATTATAAAATAAAGAGATAAAAAGAAAAAAAAAAAATGAACA   6587
m150505_iid=2498502_0     AAATAATGAAAAATCAAGGATTATAAAATAAAGAGATAAAAAGAAAAAAAAAAAATGAACA   4237
m141102_iid=754227_0      AAATAATGAAAAATCAAGGATTATAAAATAAAGAGATAAAAAGAAAAAAAAAAAATGAACA   6798
m150507_iid=3469514_0     AAATAATGAAAAATCAAGGATTATAAAATAAAGAGATAAAAAGAAAAAAAAAAAATGAACA   7356
Rdo_MitoGenome            AAATAATGAAAAATCAAGGATTATAAAATAAAGAGATAAAAAGAAAAAAAAAAAATGAACA   7832
m141101_iid=614933_0      AAATAATGAAAAATCAAGGATTATAAAATAAAGAGATAAAAAGAAAAA-AAAAATGAACA   6381
                          ********** ********:*:::     **:***. . *    ***********
                                                   nad5

m141031_iid=347546_0      AAATAAAAATAATGAAAAA--AACAAAAGTTAAAGAAATAACTCAAGATCCATAGATATC   2604
m150506_iid=3127414_0     AATAAAAATAATGAAAAAA--TACAAAAGTTAAAGAAATAACTCAAGATCCATAGATATC   4343
m141102_iid=1245517_0     AAATAAAAATAATGAAAAA--AATCAAAGTTAAAGAAATAACTCAAGATCCATAGATATC   5984
m141107_iid=1405727_0     AAATA-AAATAATGAAAAAAAATACAAAAGTTAAAGAAATAACTCAAGATCCATAGATATC   3177
m141102_iid=987614_0      AAATAAAAATAATGAAAAAAAATACAAAAGTTAAAGAAATAACTCAAGATCCATAGATATC   6166
m141108_iid=1701429_0     AAATAAAAATAATGAAAAAAAATACAAAAGTTAAAGAAATAACTCAAGATCCATAGATATC   2902
m141102_iid=987286_0      AAATAAAAATAATGAAAAAAAATACAAAAGTTAAAGAAATAACTCAAGATCCATAGATATC   4118
m150430_iid=3272125_0     AAATAAAAATAATGAAAAAAAATACAAAAGTTAAAGAAATAACTCAAGATCCATAGATATC   5636
m141102_iid=754225_0      AAATAAAAATAATGAAAAAAAATACAAAAGTTAAAGAAATAACTCAAGATCCATAGATATC   6804
m141107_iid=1409934_0     AAATAAAAATAATGAAAAAAAATACAAAAGTTAAAGAAATAACTCAAGATCCATAGATATC   7345
m150503_iid=3416920_0     AAATAAAAATAATGAA-AAAATACAAAAGTTAAAGAAATAACTCAAGATCCATAGATATC   6064
m141108_iid=1800121_0     AAATAAAAATAATGAAAAAAAATACAAAAGTTAAAGAAATAACTCAAGATCCATAGATATC   5879
m141031_iid=147540_0      AAATAAAAATAATGAAAAAAAATACAAAAGTTAAAGAAATAACTCAAGATCCATAGATATC   6224
m150507_iid=3674924_0     AAATAAAAATAATGAAAAAAAATACAAAAGTTAAAGAAATAACTCAAGATCCATAGATATC   6373
m150503_iid=2923751_0     AAATAAAAATAATGAAAAAAAATACAAAAGTTAAAGAAATAACTCAAGATCCATAGATATC   6207
m141031_iid=115118_0      AAATAAAAATAATGAAAAAAAATACAAAAGTTAAAGAAATAACTCAAGATCCATAGATATC   5068
m150430_iid=2722448_0     AAATAAAAATAATGAAAAAAAATACAAAAGTTAAAGAAATAACTCAAGATCCATAGATATC   5003
m150507_iid=3102647_0     AAATAAAAATAATGA-AAAAATACAAAAGTTAAAGAAATAACTCAAGATCCATAGATATC   3304
m141108_iid=1811866_0     AAATAAAAATAATGAAAAAAAATACAAAAGTTAAAGAAATAACTCAAGATCCATAGATATC   5185
m150501_iid=2948495_0     AAATAAAAATAATGAAAAAAAATACAAAAGTTAAAGAAATAACTCAAGATCCATAGATATC   4606
m150504_iid=3238195_0     AAATAAAAATAATGAAAAAAAATACAAAAGTTAAAGAAATAACTCAAGATCCATAGATATC   6941
```

```
m150503_iid=3388511_0      AAATAAAAATAATGAAAAAAAATACAAAAGTTAAAGAAATAACTCAAGATCCATAGATATC   4636
m150505_iid=3777611_0      AAATAAAAATAATGAAAAAAAATACAAAAGTTAAAGAAATAACTCAAGATCCATAGATATC   6059
m150430_iid=2438833_0      AAATAAAAATAATGAAAAAAAATACAAAAGTTAAAGAAATAACTCAAGATCCATAGATATC   6397
m141108_iid=1698969_0      AAATAAAAATAATGAAAAAAAATACAAAAGTTAAAGAAATAACTCAAGATCCATAGATATC   7526
m150430_iid=3059190_0      AAATAAAAATAATGAAAAAAAATACAAAAGTTAAAGAAATAACTCAAGATCCATAGATATC   3247
m141107_iid=1383067_0      AAATAAAAATAATGAA-AAAATACAAAAGTTAAAGAAATAACTCAAGATCCATAGATATC   3250
m141101_iid=597008_0       AAATAAAAATAATGAA-AAAATACAAAAGTTAAAGAAATAACTCAAGATCT--AGATATC   319
m150506_iid=2596496_0      AAATAAAAATAATGAAAAAAAATACAAAAGTTAAAGAAATAACTCAAGATCCATAGATATC   7882
m141102_iid=910051_0       AAATAAAAATAATGAAAAAAAATACAAAAGTTAAAGAAATAACTCAAGATCCATAGATATC   7366
m150505_iid=3784017_0      AAATAAAAATAATGAAAAAAAATACAAAAGTTAAAGAAATAACTCAAGATCCATAGATATC   6173
m141101_iid=682151_0       AAATAAAAATAATGAAAAAAAATACAAAAGTTAAAGAAATAACTCAAGATCCATAGATATC   5241
m150501_iid=3748029_0      AAAT-AAAATAATGAAAAAAAATACAAAAGTTAAAGAAATAACTCAAGATCCATAGATATC   5784
m150504_iid=2876272_0      AAATAAAAATAATGAAAAAAAATACAAAAGTTAAAGAAATAACTCAAGATCCATAGATATC   7648
NC_042820.1                AAATAAAAATAATGAAAAAAAATACAAAAGTTAAAGAAATAACTCAAGATCCATAGATATC   7892
m141102_iid=1228825_0      AAATAAAAATAATGAAAAAAAATACAAAAGTTAAAGAAATAACTCAAGATCCATAGATATC   7717
m150504_iid=2876274_0      AAATAAAAATAATGAAAAAAAATACAAAAGTTAAAGAAATAACTCAAGATCCATAGATATC   7719
m141030_iid=80036_0        AAATAAAAATAATGAAAAAAAATACAAAAGTTAAAGAAATAACTCAAGATCCATAGATATC   7482
m150506_iid=2596494_0      AAATAAAAATAATGAAAAAAAATACAAAAGTTAAAGAAATAACTCAAGATCCATAGATATC   7881
m150503_iid=2526862_0      AAATAAAAATAATGAAAAAAAATACAAAAGTTAAAGAAATAACTCAAGATCCATAGATATC   7569
m150506_iid=2567000_0      AAATAAAAATAATGAAAAAAAATACAAAAGTTAAAGAAATAACTCAAGATCCATAGATATC   5520
m150507_iid=3073271_0      AAATAAAAATAATGAAAAAAAATACAAAAGTTAAAGAAATAACTCAAGATCCATAGATATC   6823
m150504_iid=3156087_0      AAATAAAAATAATGAAAAAAAATACAAAAGTTAAAGAAATAACTCAAGATCCATAGATATC   6647
m150505_iid=2498502_0      AAATAAAAATAATGAAAAAAAATACAAAAGTTAAAGAAATAACTCAAGATCCATAGATATC   4297
m141102_iid=754227_0       AAATAAAAATAATGAAAAAAAATACAAAAGTTAAAGAAATAACTCAAGATCCATAGATATC   6858
m150507_iid=3469514_0      AAATAAAAATAATGAAAAAAAATACAAAAGTTAAAGAAATAACTCAAGATCCATAGATATC   7416
Rdo_MitoGenome             AAATAAAAATAATGAAAAAAAATACAAAAGTTAAAGAAATAACTCAAGATCCATAGATATC   7892
m141101_iid=614933_0       AAATAAAAATAATGAAAAAAAATACAAAAGTTAAAGAAATAACTCAAGATCCATAGATATC   6441
                           **::  **::*: .*  **  :*           ****************   *******
                                                   nad5

m141031_iid=347546_0       T--TTTTACCACAAAACAAAATTTTTTATTAAAATACTTGAATATAAACTAAATAAATTA   2662
m150506_iid=3127414_0      T--TTGTTACCACAAACAAAATTTTTTATTAAAATACTTGAATATAAACTAAATAAATTA   4401
m141102_iid=1245517_0      TT-TGTTACCACAAAACAAAATTTTTTA---TTAAACTTGAATATAAACTAAATAAATTA   6040
m141107_iid=1405727_0      TT-TGTTACCACAAAAAACAATTTTTTATTAAAATACTTGAATATAAACTAAATAAATTA   3236
m141102_iid=987614_0       TT-TGTTACCACAAAAAAAATTTTTTTATATATATA---TAAAATATAAATTATATAAAT   6222
m141108_iid=1701429_0      TT-TGTTACCACAAAACAAAATTTTTTATTAAAATACTTGAATATAAACTAAATAAATTA   2961
m141102_iid=987286_0       TT-TGTTACCACAAAACAAAATTTTTTATTAAAATACTTGAATATAAACTAAATAAATTA   4177
m150430_iid=3272125_0      TT-TGTTACCACAAAACAAAATTTTTTATTAAAATACTTGAATATAAACTAAATAAATTA   5695
m141102_iid=754225_0       TT-TGTTACCACAAAACAAAATTTTTTATTAAAATACTTGAATATAAACTAAATAAATTA   6863
m141107_iid=1409934_0      TT-TGTTACCACAAAACAAAATTTTTTATTAAAATACTTGAATATAAACTAAATAAATTA   7404
m150503_iid=3416920_0      TT-TGTTACCACAAAACAAAATTTTTTATTAAAATACTTGAATATAAACTAAATAAATTA   6123
m141108_iid=1800121_0      TT-TGTTACCACAAAACAAAATTTTTTATTAAAATACTTGAATATAAACTAAATAAATTA   5938
m141031_iid=147540_0       TT-TGTTACCACAAAACAAAATTTTTTATTAAAATACTTGAATATAAACTAAATAAATTA   6283
m150507_iid=3674924_0      TT-TGTTACCACAAAACAAAATTTTTTATTAAAATACTTGAATATAAACTAAATAAATTA   6432
m150503_iid=2923751_0      TTTTGTTACCACAAAACAAAATTTTTTATTAAAATACTTGAATATAAACTAAATAAATTA   6267
m141031_iid=115118_0       TT-TGTTACCACAAAACAAAATTTTTTATTAAAATACTTGAATATAAACTAAATAAATTA   5127
m150430_iid=2722448_0      TT-TGTTACCACAAAACAAAATTTTTTATTAAAATACTTGAATATAAACTAAATAAATTA   5062
m150507_iid=3102647_0      TT-TGTTACCACAAAAAAAAT-TTTTTATTAAAATACTTGAATATAAACTAAATAAATTA   3362
m141108_iid=1811866_0      TT-TGTTACCACAAAACAAAATTTTTTATTAAAATACTTGAATATAAACTAAATAAATTA   5244
m150501_iid=2948495_0      TT-TGTTACCACAAAACAAAATTTT-TATTAAAATACTTGAATATAAACTAAATAAATTA   4664
m150504_iid=3238195_0      TT-TGTTACCACAAAACAAAATTTTTTATTAAAATACTTGAATATAAACTAAATAAATTA   7000
m150503_iid=3388511_0      TT-TGTTACCACAAAACAAAATTTTTTATTAAAATACTTGAATATAAACTAAATAAATTA   4695
m150505_iid=3777611_0      TT-TGTTACCACAAAACAAAATTTTTTATTAAAATACTTGAATATAAACTAAATAAATTA   6118
m150430_iid=2438833_0      TT-TGTTACCACAAAACAAAATTTTTTATTAAAATACTTGAATATAAACTAAATAAATTA   6456
m141108_iid=1698969_0      TT-TGTTACCACAAAACAAAATTTTTTATTAAAATACTTGAATATAAACTAAATAAATTA   7585
m150430_iid=3059190_0      TT-TGTTACCACAAAACAAAATTTTTTATTAAAATACTTGAATATAAACTAAATAAATTA   3306
m141107_iid=1383067_0      TT-TGTTACCACAAAACAAAATTTTTTATTAAAATACTTGAATATAAACTAAATAAATTA   3309
m141101_iid=597008_0       TT-TGTTACCACAAAACAAAATTTT-TATTAAAATACTTGAATATAAACTAAATAAATTA   377
m150506_iid=2596496_0      TT-TGTTACCACAAAACAAAATTTTTTATTAAAATACTTGAATATAAACTAAATAAATTA   7941
m141102_iid=910051_0       TT-TGTTACCACAAAACAAAATTTTTTATTAAAATACTTGAATATAAACTAAATAAATTA   7425
m150505_iid=3784017_0      TT-TGTTACCACAAAACAAAATTTTTTATTAAAATACTTGAATATAAACTAAATAAATTA   6232
m141101_iid=682151_0       TT-TGTTACCACAAAACAAAATTTTTTATTAAAATACTTGAATATAAACTAAATAAATTA   5300
m150501_iid=3748029_0      TT-TGTTACCACAAAACAAAATTTTTTATTAAAATACTTGAATATAAACTAAATAAATTA   5843
m150504_iid=2876272_0      TT-TGTTACCACAAAACAAAATTTTTTATTAAAATACTTGAATATAAACTAAATAAATTA   7707
NC_042820.1                TT-TGTTACCACAAAACAAAATTTTTTATTAAAATACTTGAATATAAACTAAATAAATTA   7951
m141102_iid=1228825_0      TT-TGTTACCACAAAACAAAATTTTTTATTAAAATACTTGAATATAAACTAAATAAATTA   7776
m150504_iid=2876274_0      TT-TGTTACCACAAAACAAAATTTTTTATTAAAATACTTGAATATAAACTAAATAAATTA   7778
m141030_iid=80036_0        TT-TGTTACCACAAAACAAAATTTTTTATTAAAATACTTGAATATAAACTAAATAAATTA   7541
m150506_iid=2596494_0      TT-TGTTACCACAAAACAAAATTTTTTATTAAAATACTTGAATATAAACTAAATAAATTA   7940
m150503_iid=2526862_0      TT-TGTTACCACAAAACAAAATTTTTTATTAAAATACTTGAATATAAACTAAATAAATTA   7628
m150506_iid=2567000_0      TT-TGTTACCACAAAACAAAATTTTTTATTAAAATACTTGAATATAAACTAAATAAATTA   5579
m150507_iid=3073271_0      TT-TGTTACCACAAAACAAAATTTTTTATTAAAATACTTGAATATAAACTAAATAAATTA   6882
```

```
m150504_iid=3156087_0      TT-TGTTACCACAAAACAAAATTTTTTATTAAAATACTTGAATATAAACTAAATAAATTA   6706
m150505_iid=2498502_0      TT-TGTTACCACAAAACAAAATTTTTTATTAAAATACTTGAATATAAACTAAATAAATTA   4356
m141102_iid=754227_0       TT-TGTTACCACAAAACAAAATTTTTTATTAAAATACTTGAATATAAACTAAATAAATTA   6917
m150507_iid=3469514_0      TT-TGTTACCACAAAACAAAATTTTTTATTAAAATACTTGAATATAAACTAAATAAATTA   7475
Rdo_MitoGenome             TT-TGTTACCACAAAACAAAATTTTTTATTAAAATACTTGAATATAAACTAAATAAATTA   7951
m141101_iid=614933_0       TT-TGTTACCACAAAACAAAATTTTTTATT[illegible]AATATAAACTAAATAAATTA   6500
                           *  *  *:.*...***.*.*: *** **   nad4    **:***:* .:::***:*::

m141031_iid=347546_0       TCTATAAATAAAAAAATTAAATTCAAAGGAATTCAATGAAAAAAAAGTAATAAATATTCA   2722
m150506_iid=3127414_0      TCTATAAATAAA-AAATTAAATTCAAAGGAATTCAATGAAAAAAAAGTAATAAATATTCA   4460
m141102_iid=1245517_0      TCTATAAATAAAAAAATTAAATTCAAAGGAATTCAATGAAAAAAAAGTAATAAATATTCA   6100
m141107_iid=1405727_0      TCTATAAAT-AAAAAATTAAATTCAAAGGAATTCAATGAAAA-AAAGTAATAAATATTCA   3294
m141102_iid=987614_0       AAAAAATTAAATAAAAAAAAAATTCAATGAAA---------AAAAAAAATAAATATATTCA   6273
m141108_iid=1701429_0      TCTATAAATAAAAAAATTAAATTCAAAGGAATTCAATGAAAAAAAAGTAATAAATATTCA   3021
m141102_iid=987286_0       TCTATAAATAAAAAAATTAAATTCAAAGGAATTCAATGAAAAAAAAGTAATAAATATTCA   4237
m150430_iid=3272125_0      TCTATAAATAAAAAAATTAAATTCAAAGGAATTCAATGAAAAAAAAGTAATAAATATTCA   5755
m141102_iid=754225_0       TCTATAAATAAAAAAATTAAATTCAAAGGAATTCAATGAAAAAAAAGTAATAAATATTCA   6923
m141107_iid=1409934_0      TCTATAAATAAAAAAATTAAATTCAAAGGAATTCAATGAAAAAAAAGTAATAAATATTCA   7464
m150503_iid=3416920_0      TCTATAAATAAAAAAATTAAATTCAAAGGAATTCAATGAAAAAAAAGTAATAAATATTCA   6183
m141108_iid=1800121_0      TCTATAAATAAAAAAATTAAATTCAAAGGAATTCAATGAAAA-AAAGTAATAAATATTCA   5997
m141031_iid=147540_0       TCTATAAATAAAAAAATTAAATTCAAAGGAATTCAATGAAAAAAAAGTAATAAATATTCA   6343
m150507_iid=3674924_0      TCTATAAATAAAAAAATTAAATTCAAAGGAATTCAATGAAAAAAAAGTAATAAATATTCA   6492
m150503_iid=2923751_0      TCTATAAATAAA-AAATTAAATTCAAAGGAATTCAATGAAAA-AAAGTAATAAATATTCA   6325
m141031_iid=115118_0       TCTATAAATAAAAAAATTAAATTCAAAGGAATTCAATGAAAAAAAAGTAATAAATATTCA   5187
m150430_iid=2722448_0      TCTATAAATAAAAAAATTAAATTCAAAGGAATTCAATGAAAAAAAAGTAATAAATATTCA   5122
m150507_iid=3102647_0      TCTATAAATAA-AAAATTAAATTCAAAGGAATTCAATGAAAAAAAAGTAATAAATATTCA   3421
m141108_iid=1811866_0      TCTATAAATAAAAAAATTAAATTCAAAGGAATTCAATGAAAA-AAAGTAATAAATATTCA   5303
m150501_iid=2948495_0      TCTATAAATAAAAAAATTAAATTCAAAGGAATTCAATGAAAA-AAAGTAATAAATATTCA   4723
m150504_iid=3238195_0      TCTATAAATAA-AAAATTAAATTCAAAGGAATTCAATGAAAAAAAAGTAATAAATATTCA   7059
m150503_iid=3388511_0      TCTATAAATAAAAAAATTAAATTCAAAGGAATTCAATGAAAAAAAAGTAATAAATATTCA   4755
m150505_iid=3777611_0      TCTATAAATAAAAAAATTAAATTCAAAGGAATTCAATGAAAAAAAAGTAATAAATATTCA   6178
m150430_iid=2438833_0      TCTATAAATAAAAAAATTAAATTCAAAGGAATTCAATGAAAAAAAAGTAATAAATATTCA   6516
m141108_iid=1698969_0      TCTATAAATAAAAAAATTAAATTCAAAGGAATTCAATGAAAAAAAAGTAATAAATATTCA   7645
m150430_iid=3059190_0      TCTATAAATAAAAAAATTAAATTCAAAGGAATTCAATGAAAAAAAAGTAATAAATATTCA   3366
m141107_iid=1383067_0      TCTATAAATAAAAAAATTAAATTCAAAGGAATTCAATGAAAAAAAAGTAATAAATATTCA   3369
m141101_iid=597008_0       TCTATAAATAAAAAAATTAAATTCAAAGGAATTCAATGAAAAAAAAGTAATAAATATTCA   437
m150506_iid=2596496_0      TCTATAAATAAAAAAATTAAATTCAAAGGAATTCAATGAAAA-AAAGTAATAAATATTCA   8000
m141102_iid=910051_0       TCT---------------------------------------------------------   7428
m150505_iid=3784017_0      TCTATAAATAAAAAAATTAAATTCAAAGGAATTCAATGAAAAAAAAGTAATAAATATTCA   6292
m141101_iid=682151_0       TCTATAAATAAAAAAATTAAATTCAAAGGAATTCAATGAAAAAAAAGTAATAAATATTCA   5360
m150501_iid=3748029_0      TCTATAAATAAAAAAATTAAATTCAAAGGAATTCAATGAAAAAAAAGTAATAAATATTCA   5903
m150504_iid=2876272_0      TCTATAAATAAAAAAATTAAATTCAAAGGAATTCAATGAAAAAAAAGTAATAAATATTCA   7767
NC_042820.1                TGTATAAATAAAAAAATTAAATTCAAAGGAATTCAATGACAAAAAAGTAATAAATATTCA   8011
m141102_iid=1228825_0      TCTATAAATAAAAAAATTAAATTCAAAGGAATTCAATGAAAAAAAAGTAATAAATATTCA   7836
m150504_iid=2876274_0      TCTATAAATAAAAAAATTAAATTCAAAGGAATTCAATGAAAAAAAAGTAATAAATATTCA   7838
m141030_iid=80036_0        TCTATAAATAAAAAAATTAAATTCAAAGGAATTCAATGAAAAAAAAGTAATAAATATTCA   7601
m150506_iid=2596494_0      TCTATAAATAAAAAAATTAAATTCAAAGGAATTCAATGAAAAAAAAGTAATAAATATTCA   8000
m150503_iid=2526862_0      TCTATAAATAAAAAAATTAAATTCAAAGGAATTCAATGAAAAAAAAGTAATAAATATTCA   7688
m150506_iid=2567000_0      TCTATAAATAAAAAAATTAAATTCAAAGGAATTCAATGAAAAAAAAGTAATAAATATTCA   5639
m150507_iid=3073271_0      TCTATAAATAAAAAAATTAAATTCAAAGGAATTCAATG-AAAAAAAGTAATAAATATTCA   6941
m150504_iid=3156087_0      TCTATAAATAAAAAAATTAAATTCAAAGGAATTCAATG-AAAAAAAGTAATAAATATTCA   6765
m150505_iid=2498502_0      TCTATAAATAAAAAAATTAAATTCAAAGGAATTCAATGAAAAAAAAGTAATAAATATTCA   4416
m141102_iid=754227_0       TCTATAAATAAAAAAATTAAATTCAAAGGAATTCAATGAAAAAAAAGTAATAAATATTCA   6977
m150507_iid=3469514_0      TCTATAAATAAAAAAATTAAATTCAAAGGAATTCAATGAAAAAAAAGTAATAAATATTCA   7535
Rdo_MitoGenome             TCTATAAATAAAAAAATTAAATTCAAAGGAATTCAATGAAAAAAAAGTAATAAATATTCA   8011
m141101_iid=614933_0       TCTATAAATAAAAAAATTAAATTCAAAGGAATTCAATGAAAAAAAAGTAATAAATATTCA   6560
                           : :
                                              nad4

m141031_iid=347546_0       CGAACATTAACAGATCAAAAACTAAATAAACCCCTACTTACCATGCCATGCTGAGTGAAA   2782
m150506_iid=3127414_0      CGAACTTA-ACAGATCAAAAAACTA-ATAAACCCTACTTACCATGCCATGCTGAGTGAAA   4518
m141102_iid=1245517_0      CGAAA-TTAACAGATCAAAAACTAAATAA-ACCCTACTTACCATGCCATGCTGAGTGAAA   6158
m141107_iid=1405727_0      CGAACATTAACAGATCAAAAACTAAATAAACCCCTACTTACCATGCCATGCTGAGTGAAA   3354
m141102_iid=987614_0       CGAACATTAACAGATCAAAAACTAAATAAACCCCTACTTACCATGCCATGCTGAGTGAAA   6333
m141108_iid=1701429_0      CGAACATTAACAGATCAAAAACTAAATAAACCCCTACTTACCATGCCATGCTGAGTGAAA   3081
m141102_iid=987286_0       CGAACATTAACAGATCAAAAACTAAATAAACCCCTACTTACCATGCCATGCTGAGTGAAA   4297
m150430_iid=3272125_0      CGAACATTAACAGATCAAAAACTAAATAAACCCCTACTTACCATGCCATGCTGAGTGAAA   5815
m141102_iid=754225_0       CGAACATTAACAGATCAAAAACTAAATAAACCCCTACTTACCATGCCATGCTGAGTGAAA   6983
m141107_iid=1409934_0      CGAACATTAACAGATCAAAAACTAAATAAACCCCTACTTACCATGCCATGCTGAGTGAAA   7524
m150503_iid=3416920_0      CGAACATTAACAGATCAAAAACTAAATAAA-CCCTACTTACCATGC-ATGCTGAGTGAAA   6241
m141108_iid=1800121_0      CGAACATTAACAGATCAAAAACTAAATAAACCCCTACTTACCATGCCATGCTGAGTGAAA   6057
m141031_iid=147540_0       CGAACATTAACAGATCAAAAACTAAATAAACCCCTACTTACCATGCCATGCTGAGTGAAA   6403
```

```
m150507_iid=3674924_0      CGAACATTAACAGATCAAAAACTAAATAAACCCCTACTTACCATGCCATGCTGAGTGAAA   6552
m150503_iid=2923751_0      CGAACATTAACAGATCAAAAACTAAATAA-ACCCTACTTACCATGCCATGCTGAGTGAAA   6384
m141031_iid=115118_0       CGAACATTAACAGATCAAAAACTAAATAAACCCCTACTTACCATGCCATGCTGAGTGAAA   5247
m150430_iid=2722448_0      CGAACATTAACAGATCAAAAACTAAATAAACCCCTACTTACCATGCCATGCTGAGTGAAA   5182
m150507_iid=3102647_0      CGAACATTAACAGATCAAAAACTAAATAAACCCCTACTTACCATGCCATGCTGAGTGAAA   3481
m141108_iid=1811866_0      CGAACATTAACAGATCAAAAACTAAATAAACCCCTACTTACCATGCCATGCTGAGTGAAA   5363
m150501_iid=2948495_0      CGAACATTAACAGATCAAAAACTAAATAAACCCCTACTTACCATGCCATGCTGAGTGAAA   4783
m150504_iid=3238195_0      CGAACATTAACAGATCAAAAACTAAATAAACCCCTACTTACCATGCCATGCTGAGTGAAA   7119
m150503_iid=3388511_0      CGAACATTAACAGATCAAAAACTAAATAAACCCCTACTTACCATGCCATGCTGAGTGAAA   4815
m150505_iid=3777611_0      CGAACATTAACAGATCAAAAACTAAATAAACCCCTACTTACCATGCCATGCTGAGTGAAA   6238
m150430_iid=2438833_0      CGAACATTAACAGATCAAAAACTAAATAAACCCCTACTTACCATGCCATGCTGAGTGAAA   6576
m141108_iid=1698969_0      CGAACATTAACAGATCAAAAACTAAATAAACCCCTACTTACCATGCCATGCTGAGTGAAA   7705
m150430_iid=3059190_0      CGAACATTAACAGATCAAAAACTAAATAAACCCCTACTTACCATGCCATGCTGAGTGAAA   3426
m141107_iid=1383067_0      CGAACATTAACAGATCAAAAACTAAATAAACCCCTACTTACCATGCCATGCTGAGTGAAA   3429
m141101_iid=597008_0       CGAACATTAACAGATCAAAAACTAAATAAACCCCTACTTACCATGCCATGCTGAGTGAAA   497
m150506_iid=2596496_0      CGAACATTAACAGATCAAAAACTAAATAAACCCCTACTTACCATGCCATGCTGAGTGAAA   8060
m141102_iid=910051_0       ------------------------------------------------------------   7428
m150505_iid=3784017_0      CGAACATTAACAGATCAAAAACTAAATAAACCCCTACTTACCATGCCATGCTGAGTGAAA   6352
m141101_iid=682151_0       CGAACATTAACAGATCAAAAACTAAATAAACCCCTACTTACCATGCCATGCTGAGTGAAA   5420
m150501_iid=3748029_0      CGAACATTAACAGATCAAAAACTAAATAAACCCCTACTTACCATGCCATGCTGAGTGAAA   5963
m150504_iid=2876272_0      CGAACATTAACAGATCAAAAACTAAATAAACCCCTACTTACCATGCCATGCTGAGTGAAA   7827
NC_042820.1                CGAACATTAACAGATCAAAAACTAAATAAACCCCTACTTACCATGCCATGCTGAGTGAAA   8071
m141102_iid=1228825_0      CGAACATTAACAGATCAAAAACTAAATAAACCCCTACTTACCATGCCATGCTGAGTGAAA   7896
m150504_iid=2876274_0      CGAACATTAACAGATCAAAAACTAAATAAACCCCTACTTACCATGCCATGCTGAGTGAAA   7898
m141030_iid=80036_0        CGAACATTAACAGATCAAAAACTAAATAAACCCCTACTTACCATGCCATGCTGAGTGAAA   7661
m150506_iid=2596494_0      CGAACATTAACAGATCAAAAACTAAATAAACCCCTACTTACCATGCCATGCTGAGTGAAA   8060
m150503_iid=2526862_0      CGAACATTAACAGATCAAAAACTAAATAAACCCCTACTTACCATGCCATGCTGAGTGAAA   7748
m150506_iid=2567000_0      CGAACATTAACAGATCAAAAACTAAATAAACCCCTACTTACCATGCCATGCTGAGTGAAA   5699
m150507_iid=3073271_0      CGAACATTAACAGATCAAAAACTAAATAAACCCCTACTTACCATGCCATGCTGAGTGAAA   7001
m150504_iid=3156087_0      CGAACATTAACAGATCAAAAACTAAATAAACCCCTACTTACCATGCCATGCTGAGTGAAA   6825
m150505_iid=2498502_0      CGAACATTAACAGATCAAAAACTAAATAAACCCCTACTTACCATGCCATGCTGAGTGAAA   4476
m141102_iid=754227_0       CGAACATTAACAGATCAAAAACTAAATAAACCCCTACTTACCATGCCATGCTGAGTGAAA   7037
m150507_iid=3469514_0      CGAACATTAACAGATCAAAAACTAAATAAACCCCTACTTACCATGCCATGCTGAGTGAAA   7595
Rdo_MitoGenome             CGAACATTAACAGATCAAAAACTAAATAAACCCCTACTTACCATGCCATGCTGAGTGAAA   8071
m141101_iid=614933_0       CGAACATTAACAGATCAAAAACTAAATAAACCCCTACTTACCATGCCATGCTGAGTGAAA   6620
```

nad4 →

```
m141031_iid=347546_0       GAATACAAAAATAATGAATAAGCAGCTCTAAGAAAAGAAAGAAAAGATAAAATTAAAATT   2842
m150506_iid=3127414_0      GAATACAAAAATAATGAATAAGCAGCTCTAAGAAAAGAAAGAAAGAT--AAATTAAAATT   4576
m141102_iid=1245517_0      GAATACAAA-ATAATGAATAAGCAGCTCTAAGAAAAGAAAGAAAAGATAAATTA-AAATT   6216
m141107_iid=1405727_0      GAATACAAAAATAATGAATAAGCAGCTCTAAGAAAAGAAAGAAAGATAAAATTAAAATTA   3414
m141102_iid=987614_0       GAATACAAAAATAATGAATAAGCAGCTCTAAGAAAAGAAAGAAAAGATAAAATTAAAATT   6393
m141108_iid=1701429_0      GAATACAAAAATAATGAATAAGCAGCTCTAAGAAA-GAAAGAAAAGATAAAATTAAAATT   3140
m141102_iid=987286_0       GAATACAAAAATAATGAATAAGCAGCTCTAAGAAAAGAAAGAAAAGATAAAATTAAAATT   4357
m150430_iid=3272125_0      GAATACAAAAATAATGAATAAGCAGCTCTAAGAAAAGAAAGAAAAGATAAAATTAAAATT   5875
m141102_iid=754225_0       GAATACAAAAATAATGAATAAGCAGCTCTAAGAAAAGAAAGAAAAGATAAAATTAAAATT   7043
m141107_iid=1409934_0      GAATACAAAAATAATGAATAAGCAGCTCTAAGAAAAGAAAGAAAAGATAAAATTAAAATT   7584
m150503_iid=3416920_0      GAATACAAAAATAATGAATAAGCAGCTCTAAGAAAAGAAAGAAAAGATAAAATTAAAATT   6301
m141108_iid=1800121_0      GAATACAAA-ATAATGAATAAGCAGCTCTAAGAAAAGAAAGAAAAGATAAAATTAAAATT   6116
m141031_iid=147540_0       GAATACAAAAATAATGAATAAGCAGCTCTAAGAAAAGAAAGAAAAGATAAAATTAAAATT   6463
m150507_iid=3674924_0      GAATACAAAAATAATGAATAAGCAGCTCTAAGAAAAGAAAGAAAAGATAAAATTAAAATT   6612
m150503_iid=2923751_0      GAATACAAAAATAATGAATAAGCAGCTCTAAGAAAAGAAAAGAAAGATAAAATTAAAATT   6444
m141031_iid=115118_0       GAATACAAAAATAATGAATAAGCAGCTCTAAGAAAAGAAAGAAAAGATAAAATTAAAATT   5307
m150430_iid=2722448_0      GAATACAAAAATAATGAATAAGCAGCTCTAAGAAAAGAAAGAAAAGATAAAATTAAAATT   5242
m150507_iid=3102647_0      GAATACAAAAATAATGAATAAGCAGCTCTAAGAAAAGAAAGAAAAGATAAAATTAAAATT   3541
m141108_iid=1811866_0      GAATACAAAAATAATGAATAAGCAGCTCTAAGAAAAGAAAGAAAAGATAAAATTAAAATT   5423
m150501_iid=2948495_0      GAATACAAAAATAATGAATAAGCAGCTCTAAGAAAAGAAAGAAAAGATAAAATTAAAATT   4843
m150504_iid=3238195_0      GAATACAAAAATAATGAATAAGCAGCTCTAAGAAAAGAAAGAAAAGATAAATTAAAAATT   7179
m150503_iid=3388511_0      GAATACAAAAATAATGAATAAGCAGCTCTAAGAAAAGAAAGAAAAGATAAAATTAAAATT   4875
m150505_iid=3777611_0      GAATACAAAAATAATGAATAAGCAGCTCTAAGAAAAGAAAGAAAAGATAAAATTAAAATT   6298
m150430_iid=2438833_0      GAATACAAAAATAATGAATAAGCAGCTCTAAGAAAAGAAAGAAAAGATAAAATTAAAATT   6636
m141108_iid=1698969_0      GAATACAAAAATAATGAATAAGCAGCTCTAAGAAAAGAAAGAAAAGATAAAATTAAAATT   7765
m150430_iid=3059190_0      GAATACAAAAATAATGAATAAGCAGCTCTAAGAAAAGAAAGAAAAGATAAAATTAAAATT   3486
m141107_iid=1383067_0      GAATACAAAAATAATGAATAAGCAGCTCTAAGAAAAGAAAGAAA-GATAAAATTAAAATT   3488
m141101_iid=597008_0       GAATACAAAAATAATGAATAAGCAGCTCTAAGAAAAGAAAGAAAAGATAAAATTAAAATT   557
m150506_iid=2596496_0      GAATACAAAAAT------AATGCAGCTCTAAGAAAAGAAAGAAAAGATAAAATTAAAATT   8114
m141102_iid=910051_0       ------------------------------------------------------------   7428
m150505_iid=3784017_0      GAATACAAAAATAATGAATAAGCAGCTCTAAGAAAAGAAAGAAAAGATAAAATTAAAATT   6412
m141101_iid=682151_0       GAATACAAAAATAATGAATAAGCAGCTCTAAGAAAAGAAAGAAAAGATAAAATTAAAATT   5480
m150501_iid=3748029_0      GAATACAAAAATAATGAATAAGCAGCTCTAAGAAAAGAAAGAAAAGATAAAATTAAAATT   6023
m150504_iid=2876272_0      GAATACAAAAATAATGAATAAGCAGCTCTAAGAAAAGAAAGAAAAGATAAAATTAAAATT   7887
```

```
NC_042820.1              GAATACAAAAATAATGAATAAGCAGCTCTAAGAAAAGAAAGAAAAGATAAAATTAAAATT   8131
m141102_iid=1228825_0    GAATACAAAAATAATGAATAAGCAGCTCTAAG-AAAGAAAGAAAAGATAAAATTAAAATT   7955
m150504_iid=2876274_0    GAATACAAAAATAATGAATAAGCAGCTCTAAGAAAAGAAAGAAAAGATAAAATTAAAATT   7958
m141030_iid=80036_0      GAATACAAAAATAATGAATAAGCAGCTCTAAGAAAAGAAAGAAAAGATAAAATTAAAATT   7721
m150506_iid=2596494_0    GAATACAAAAATAATGAATAAGCAGCTCTAAGAAAAGAAAGAAAAGATAAAATTAAAATT   8120
m150503_iid=2526862_0    GAATACAAAAATAATGAATAAGCAGCTCTAAGAAAAGAAAGAAAAGATAAAATTAAAATT   7808
m150506_iid=2567000_0    GAATACAAAAATAATGAATAAGCAGCTCTAAGAAAAGAAAGAAAAGATAAAATTAAAATT   5759
m150507_iid=3073271_0    GAATACAAAAATAATGAATAAGCAGCTCTAAGAAAAGAAAGAAAAGATAAAATTAAAATT   7061
m150504_iid=3156087_0    GAATACAAAAATAATGAATAAGCAGCTCTAAGAAAAGAAAGAAAAGATAAAATTAAAATT   6885
m150505_iid=2498502_0    GAATACAAAAATAATGAATAAGCAGCTCTAAGAAAAGAAAGAAAAGATAAAATTAAAATT   4536
m141102_iid=754227_0     GAATACAAAAATAATGAATAAGCAGCTCTAAGAAAAGAAAGAAAAGATAAAATTAAAATT   7097
m150507_iid=3469514_0    GAATACAAAAATAATGAATAAGCAGCTCTAAGAAAAGAAAGAAAAGATAAAATTAAAATT   7655
Rdo_MitoGenome           GAATACAAAAATAATGAATAAGCAGCTCTAAGAAAAGAAAGAAAAGATAAAATTAAAATT   8131
m141101_iid=614933_0     GAATACAAAAATAATGAATAAGCAGCTCTAAGAAAAGAAAGAAAAGATAAAATTAAAATT   6680
```

nad4

```
m141031_iid=347546_0     ATTAATCTTCTTCAACTAACTAAAGAACTTAAAATTAAAATTTCAGAAACAAGATTTAAA   2902
m150506_iid=3127414_0    ATTAATCTTCTTCAACTAACTAAAGAACTTAAAATTAAAATTTCAGAAACAAGATTTAAA   4636
m141102_iid=1245517_0    ATTAATCTTCTTC-ACTAACTAAAGAACTTAAAATTAAAATTTCAGAAACAAGATTTAAA   6275
m141107_iid=1405727_0    TTTAATCTTCTTCAACTAACTAAAGAACTTAAAATTAAAATTTCAGAAACAAGATTTAAA   3474
m141102_iid=987614_0     ATTAATCTTCTTCAACTAACTAAAGAACTTAAAATTAAAATTTCAGAAACAAGATTTAAA   6453
m141108_iid=1701429_0    ATTAATCTTCTTCAACTAACTAAAGAACTTAAAATTAAAATTTCAGAAACAAGATTTAAA   3200
m141102_iid=987286_0     ATTAATCTTCTTCAACTAACTAAAGAACTTAAAATTAAAATTTCAGAAACAAGATTTAAA   4417
m150430_iid=3272125_0    ATTAATCTTCTTCAACTAACTAAAGAACTTAAAATTAAAATTTCAGAAACAAGATTTAAA   5935
m141102_iid=754225_0     ATTAATCTTCTTCAACTAACTAAAGAACTTAAAATTAAAATTTCAGAAACAAGATTTAAA   7103
m141107_iid=1409934_0    ATTAATCTTCTTCAACTAACTAAAGAACTTAAAATTAAAATTTCAGAAACAAGATTTAAA   7644
m150503_iid=3416920_0    ATTAATCTTCTTCAACTAACTAAAGAACTTAAAATTAAAATTTCAGAAACAAGATTTAAA   6361
m141108_iid=1800121_0    ATTAATCTTCTTCAACTAACTAAAGAACTTAAAATTAAAATTTCAGAAACAAGATTTAAA   6176
m141031_iid=147540_0     ATTAATCTTCTTCAACTAACTAAAGAACTTAAAATTAAAATTTCAGAAACAAGATTTAAA   6523
m150507_iid=3674924_0    ATTAATCTTCTTCAACTAACTAAAGAACTTAAAATTAAAATTTCAGAAACAAGATTTAAA   6672
m150503_iid=2923751_0    ATTAATCTTCTTCAACTAACTAAAGAACTTAAAATTAAAATTTCAGAAACAAGATTTAAA   6504
m141031_iid=115118_0     ATTAATCTTCTTCAACTAACTAAAGAACTTAAAATTAAAATTTCAGAAACAAGATTTAAA   5367
m150430_iid=2722448_0    ATTAATCTTCTTCAACTAACTAAAGAACTTAAAATTAAAATTTCAGAAACAAGATTTAAA   5302
m150507_iid=3102647_0    ATTAATCTTCTTCAACTAACTAAAGAACTTAAAATTAAAATTTCAGAAACAAGATTTAAA   3601
m141108_iid=1811866_0    ATTAATCTTCTTCAACTAACTAAAGAACTTAAAATTAAAATTTCAGAAACAAGATTTAAA   5483
m150501_iid=2948495_0    ATTAATCTTCTTCAACTAACTAAAGAACTTAAAATTAAAATTTCAGAAACAAGATTTAAA   4903
m150504_iid=3238195_0    ATTAATCTTCTTCAACTAACTAAAGAACTTAAAATTAAAATTTCAGAAACAAGATTTAAA   7239
m150503_iid=3388511_0    ATTAATCTTCTTCAACTAACTAAAGAACTTAAAATTAAAATTTCAGAAACAAGATTTAAA   4935
m150505_iid=3777611_0    ATTAATCTTCTTCAACTAACTAAAGAACTTAAAATTAAAATTTCAGAAACAAGATTTAAA   6358
m150430_iid=2438833_0    ATTAATCTTCTTCAACTAACTAAAGAACTTAAAATTAAAATTTCAGAAACAAGATTTAAA   6696
m141108_iid=1698969_0    ATTAATCTTCTTCAACTAACTAAAGAACTTAAAATTAAAATTTCAGAAACAAGATTTAAA   7825
m150430_iid=3059190_0    ATTAATCTTCTTCAACTAACTAAAGAACTTAAAATTAAAATTTCAGAAACAAGATTTAAA   3546
m141107_iid=1383067_0    ATTAATCTTCTTCAACTAACTAAAGAACTTAAAATTAAAATTTCAGAAACAAGATTTAAA   3548
m141101_iid=597008_0     ATTAATCTTCTTCAACTAACTAAAGAACTTAAAATTAAAATTTCAGAAACAAGATTTAAA   617
m150506_iid=2596496_0    ATTAATCTTCTTCAACTAACTAAAGAACTTAAAATTAAAATTTCAGAAACAAGATTTAAA   8174
m141102_iid=910051_0     ------------------------------------------------------------   7428
m150505_iid=3784017_0    ATTAATCTTCTTCAACTAACTAAAGAACTTAAAATTAAAATTTCAGAAACAAGATTTAAA   6472
m141101_iid=682151_0     ATTAATCTTCTTCAACTAACTAAAGAACTTAAAATTAAAATTTCAGAAACAAGATTTAAA   5540
m150501_iid=3748029_0    ATTAATCTTCTTCAACTAACTAAAGAACTTAAAATTAAAATTTCAGAAACAAGATTTAAA   6083
m150504_iid=2876272_0    ATTAATCTTCTTCAACTAACTAAAGAACTTAAAATTAAAATTTCAGAAACAAGATTTAAA   7947
NC_042820.1              ATTAATCTTCTTCAACTAACTAAAGAACTTAAAATTAAAATTTCAGAAACAAGATTTAAA   8191
m141102_iid=1228825_0    ATTAATCTTCTTCAACTAACTAAAGAACTTAAAATTAAAATTTCAGAAACAAGATTTAAA   8015
m150504_iid=2876274_0    ATTAATCTTCTTCAACTAACTAAAGAACTTAAAATTAAAATTTCAGAAACAAGATTTAAA   8018
m141030_iid=80036_0      ATTAATCTTCTTCAACTAACTAAAGAACTTAAAATTAAAATTTCAGAAACAAGATTTAAA   7781
m150506_iid=2596494_0    ATTAATCTTCTTCAACTAACTAAAGAACTTAAAATTAAAATTTCAGAAACAAGATTTAAA   8180
m150503_iid=2526862_0    ATTAATCTTCTTCAACTAACTAAAGAACTTAAAATTAAAATTTCAGAAACAAGATTTAAA   7868
m150506_iid=2567000_0    ATTAATCTTCTTCAACTAACTAAAGAACTTAAAATTAAAATTTCAGAAACAAGATTTAAA   5819
m150507_iid=3073271_0    ATTAATCTTCTTCAACTAACTAAAGAACTTAAAATTAAAATTTCAGAAACAAGATTTAAA   7121
m150504_iid=3156087_0    ATTAATCTTCTTCAACTAACTAAAGAACTTAAAATTAAAATTTCAGAAACAAGATTTAAA   6945
m150505_iid=2498502_0    ATTAATCTTCTTCAACTAACTAAAGAACTTAAAATTAAAATTTCAGAAACAAGATTTAAA   4596
m141102_iid=754227_0     ATTAATCTTCTTCAACTAACTAAAGAACTTAAAATTAAAATTTCAGAAACAAGATTTAAA   7157
m150507_iid=3469514_0    ATTAATCTTCTTCAACTAACTAAAGAACTTAAAATTAAAATTTCAGAAACAAGATTTAAA   7715
Rdo_MitoGenome           ATTAATCTTCTTCAACTAACTAAAGAACTTAAAATTAAAATTTCAGAAACAAGATTTAAA   8191
m141101_iid=614933_0     ATTAATCTTCTTCAACTAACTAAAGAACTTAAAATTAAAATTTCAGAAACAAGATTTAAA   6740
```

nad4

```
m141031_iid=347546_0     GAAGGTGGAGCAGCTATATTTGATGAACATAAAAG--ATCACCATAAACTCAAGTTAGGT   2960
m150506_iid=3127414_0    GAAGGTGGAGCAGCTATATTTGATGAACATAAAAGAAATCACCATAAACTCAAGTTAGGT   4696
m141102_iid=1245517_0    GAAGGTGGAGCAGCTATATTTGATGAACATAAAAGAAATCACCATAAACTCAAGTTAGGT   6335
m141107_iid=1405727_0    GAAGGTGGAGCAGCTATATTTGATGAACATAAAAGAAATCACCAT-AACTCAAGTTAGGT   3533
m141102_iid=987614_0     GAAGGTGGAGCAGCTATATTTGATGAACATAAAAGAAATCACCATAAACTCAAGTTAGGT   6513
```

```
m141108_iid=1701429_0      GAAGGTGGAGCAGCTATATTTGATGAACATAAAAGAAATCACCATAAACTCAAGTTAGGT   3260
m141102_iid=987286_0       GAAGGTGGAGCAGCTATATTTGATGAACATAAAAGAAATCACCATAAACTCAAGTTAGGT   4477
m150430_iid=3272125_0      GAAGGTGGAGCAGCTATATTTGATGAACATAAAAGAAATCACCATAAACTCAAGTTAGGT   5995
m141102_iid=754225_0       GAAGGTGGAGCAGCTATATTTGATGAACATAAAAGAAATCACCATAAACTCAAGTTAGGT   7163
m141107_iid=1409934_0      GAAGGTGGAGCAGCTATATTTGATGAACATAAAAGAAATCACCATAAACTCAAGTTAGGT   7704
m150503_iid=3416920_0      GAAGGTGGAGCAGCTATATTTGATGAACATAAAAGAAATCACCATAAACTCAAGTTAGGT   6421
m141108_iid=1800121_0      GAAGGTGGAGCAGCTATATTTGATGAACATAAAAGAAATCACCATAAACTCAAGTTAGGT   6236
m141031_iid=147540_0       GAAGGTGGAGCAGCTATATTTGATGAACATAAAAGAAATCACCATAAACTCAAGTTAGGT   6583
m150507_iid=3674924_0      GAAGGTGGAGCAGCTATATTTGATGAACATAAAAGAAATCACCATAAACTCAAGTTAGGT   6732
m150503_iid=2923751_0      GAAGGTGGAGCAGCTATATTTGATGAACATAAAAGAAATCACCATAAACTCAAGTTAGGT   6564
m141031_iid=115118_0       GAAGGTGGAGCAGCTATATTTGATGAACATAAAAGAAATCACCATAAACTCAAGTTAGGT   5427
m150430_iid=2722448_0      GAAGGTGGAGCAGCTATATTTGATGAACATAAAAGAAATCACCATAAACTCAAGTTAGGT   5362
m150507_iid=3102647_0      GAAGGTGGAGCAGCTATATTTGATGAACATAAAAGAAATCACCATAAACTCAAGTTAGGT   3661
m141108_iid=1811866_0      GAAGGTGGAGCAGCTATATTTGATGAACATAAAAGAAATCACCATAAACTCAAGTTAGGT   5543
m150501_iid=2948495_0      GAAGGTGGAGCAGCTATATTTGATGAACATAAAAGAAATCACCATAAACTCAAGTTAGGT   4963
m150504_iid=3238195_0      GAAGGTGGAGCAGCTATATTTGATGAACATAAAAGAAATCACCATAAACTCAAGTTAGGT   7299
m150503_iid=3388511_0      GAAGGTGGAGCAGCTATATTTGATGAACATAAAAGAAATCACCATAAACTCAAGTTAGGT   4995
m150505_iid=3777611_0      GAAGGTGGAGCAGCTATATTTGATGAACATAAAAGAAATCACCATAAACTCAAGTTAGGT   6418
m150430_iid=2438833_0      GAAGGTGGAGCAGCTATATTTGATGAACATAAAAGAAATCACCATAAACTCAAGTTAGGT   6756
m141108_iid=1698969_0      GAAGGTGGAGCAGCTATATTTGATGAACATAAAAGAAATCACCATAAACTCAAGTTAGGT   7885
m150430_iid=3059190_0      GAAGGTGGAGCAGCTATATTTGATGAACATAAAAGAAATCACCATAAACTCAAGTTAGGT   3606
m141107_iid=1383067_0      GAAGGTGGAGCAGCTATATTTGATGAACATAAAAGAAATCACCATAAACTCAAGTTAGGT   3608
m141101_iid=597008_0       GAAGGTGGAGCAGCTATATTTGATGAACATAAAAGAAATCACCATAAACTCAAGTTAGGT   677
m150506_iid=2596496_0      GAAGGTGGAGCAGCTATATTTGATGAACATAAAAGAAATCACCATAAACTCAAGTTAGGT   8234
m141102_iid=910051_0       ------------------------------------------------------------   7428
m150505_iid=3784017_0      GAAGGTGGAGCAGCTATATTTGATGAACATAAAAGAAATCACCATAAACTCAAGTTAGGT   6532
m141101_iid=682151_0       GAAGGTGGAGCAGCTATATTTGATGAACATAAAAGAAATCACCATAAACTCAAGTTAGGT   5600
m150501_iid=3748029_0      GAAGGTGGAGCAGCTATATTTGATGAACATAAAAGAAATCACCATAAACTCAAGTTAGGT   6143
m150504_iid=2876272_0      GAAGGTGGAGCAGCTATATTTGATGAACATAAAAGAAATCACCATAAACTCAAGTTAGGT   8007
NC_042820.1                GAAGGTGGAGCAGCTATATTTGATGAACATAAAAGAAATCACCATAAACTCAAGTTAGGT   8251
m141102_iid=1228825_0      GAAGGTGGAGCAGCTATATTTGATGAACATAAAAGAAATCACCATAAACTCAAGTTAGGT   8075
m150504_iid=2876274_0      GAAGGTGGAGCAGCTATATTTGATGAACATAAAAGAAATCACCATAAACTCAAGTTAGGT   8078
m141030_iid=80036_0        GAAGGTGGAGCAGCTATATTTGATGAACATAAAAGAAATCACCATAAACTCAAGTTAGGT   7841
m150506_iid=2596494_0      GAAGGTGGAGCAGCTATATTTGATGAACATAAAAGAAATCACCATAAACTCAAGTTAGGT   8240
m150503_iid=2526862_0      GAAGGTGGAGCAGCTATATTTGATGAACATAAAAGAAATCACCATAAACTCAAGTTAGGT   7928
m150506_iid=2567000_0      GAAGGTGGAGCAGCTATATTTGATGAACATAAAAGAAATCACCATAAACTCAAGTTAGGT   5879
m150507_iid=3073271_0      GAAGGTGGAGCAGCTATATTTGATGAACATAAAAGAAATCACCATAAACTCAAGTTAGGT   7181
m150504_iid=3156087_0      GAAGGTGGAGCAGCTATATTTGATGAACATAAAAGAAATCACCATAAACTCAAGTTAGGT   7005
m150505_iid=2498502_0      GAAGGTGGAGCAGCTATATTTGATGAACATAAAAGAAATCACCATAAACTCAAGTTAGGT   4656
m141102_iid=754227_0       GAAGGTGGAGCAGCTATATTTGATGAACATAAAAGAAATCACCATAAACTCAAGTTAGGT   7217
m150507_iid=3469514_0      GAAGGTGGAGCAGCTATATTTGATGAACATAAAAGAAATCACCATAAACTCAAGTTAGGT   7775
Rdo_MitoGenome             GAAGGTGGAGCAGCTATATTTGATGAACATAAAAGAAATCACCATAAACTCAAGTTAGGT   8251
m141101_iid=614933_0       GAAGGTGGAGCAGCTATATTTGATGAACATAAAAGAAATCACCATAAACTCAAGTTAGGT   6800
```

nad4

```
m141031_iid=347546_0       AAAATATTAATATAACCTTTGTTTAAATACAAACTACGTCTCAAAGACCGTTCATAAGTA   3020
m150506_iid=3127414_0      AAAATATTAATATAACCTTTGTTTAAATACAAACTACGTCTCAAAGACCGTTCATAAGTA   4756
m141102_iid=1245517_0      AAAATATTAATATAACCTTTGTTTAAATACAAACTACGTCTCAAAGACCGTTCATAAGTA   6395
m141107_iid=1405727_0      AAAA--TTAATATAACCTTTGTTTAAATACAAACTACGTCTCAAAGA-CGTTCATAAGTA   3590
m141102_iid=987614_0       AAAATATTAATATAACCTTTGTTTAAATACAAACTACGTCTCAAAGACCGTTCATAAGTA   6573
m141108_iid=1701429_0      AAAATATTAATATAACCTTTGTTTAAATACAAACTACGTCTCAAAGACCGTTCATAAGTA   3320
m141102_iid=987286_0       AAAATATTAATATAACCTTTGTTTAAATACAAACTACGTCTCAAAGACCGTTCATAAGTA   4537
m150430_iid=3272125_0      AAAA--TTAATATAACCTTTGTTTAAATACAAACTACGTCTCAAAGACCGTTCATAAGTA   6053
m141102_iid=754225_0       AAAATATTAATATAACCTTTGTTTAAATACAAACTACGTCTCAAAGACCGTTCATAAGTA   7223
m141107_iid=1409934_0      AAAATATTAATATAACCTTTGTTTAAATACAAACTACGTCTCAAAGACCGTTCATAAGTA   7764
m150503_iid=3416920_0      AAAATATTAATATAACCTTTGTTTAAATACAAACTACGTCTCAAAGACCGTTCATAAGTA   6481
m141108_iid=1800121_0      AAAATATTAATATAACCTTTGTTTAAATACAAACTACGTCTCAAAGACCGTTCATAAGTA   6296
m141031_iid=147540_0       AAAATATTAATATAACCTTTGTTTAAATACAAACTACGTCTCAAAGACCGTTCATAAGTA   6643
m150507_iid=3674924_0      AAAATATTAATATAACCTTTGTTTAAATACAAACTACGTCTCAAAGACCGTTCATAAGTA   6792
m150503_iid=2923751_0      AAAATATTAATATAACCTTTGTTTAAATACAAACTACGTCTCAAAGACCGTTCATAAGTA   6624
m141031_iid=115118_0       AAAATATTAATATAACCTTTGTTTAAATACAAACTACGTCTCAAAGACCGTTCATAAGTA   5487
m150430_iid=2722448_0      AAAATATTAATATAACCTTTGTTTAAATACAAACTACGTCTCAAAGACCGTTCATAAGTA   5422
m150507_iid=3102647_0      AAAATATTAATATAACCTTTGTTTAAATACAAACTACGTCTCAAAGACCGTTCATAAGTA   3721
m141108_iid=1811866_0      AAAATATTAATATAACCTTTGTTTAAATACAAACTACGTCTCAAAGACCGTTCATAAGTA   5603
m150501_iid=2948495_0      AAAATATTAATATAACCTTTGTTTAAATACAAACTACGTCTCAAAGACCGTTCATAAGTA   5023
m150504_iid=3238195_0      AAAATATTAATATAACCTTTGTTTAAATACAAACTACGTCTCAAAGACCGTTCATAAGTA   7359
m150503_iid=3388511_0      AAAATATTAATATAACCTTTGTTTAAATACAAACTACGTCTCAAAGACCGTTCATAAGTA   5055
m150505_iid=3777611_0      AAAATATTAATATAACCTTTGTTTAAATACAAACTACGTCTCAAAGACCGTTCATAAGTA   6478
m150430_iid=2438833_0      AAAATATTAATATAACCTTTGTTTAAATACAAACTACGTCTCAAAGACCGTTCATAAGTA   6816
m141108_iid=1698969_0      AAAATATTAATATAACCTTTGTTTAAATACAAACTACGTCTCAAAGACCGTTCATAAGTA   7945
m150430_iid=3059190_0      AAAATATTAATATAACCTTTGTTTAAATACAAACTACGTCTCAAAGACCGTTCATAAGTA   3666
```

```
m141107_iid=1383067_0      AAAATATTAATATAACCTTTGTTTAAATACAAACTACGTCTCAAAGACCGTTCATAAGTA   3668
m141101_iid=597008_0       AAAATATTAATATAACCTTTGTTTAAATACAAACTACGTCTCAAAGACCGTTCATAAGTA   737
m150506_iid=2596496_0      AAAATATTAATATAACCTTTGTTTAAATACAAACTACGTCTCAAAGACCGTTCATAAGTA   8294
m141102_iid=910051_0       ------------------------------------------------------------   7428
m150505_iid=3784017_0      AAAATATTAATATAACCTTTGTTTAAATACAAACTACGTCTCAAAGACCGTTCATAAGTA   6592
m141101_iid=682151_0       AAAATATTAATATAACCTTTGTTTAAATACAAACTACGTCTCAAAGACCGTTCATAAGTA   5660
m150501_iid=3748029_0      AAAATATTAATATAACCTTTGTTTAAATACAAACTACGTCTCAAAGACCGTTCATAAGTA   6203
m150504_iid=2876272_0      AAAATATT-ATATAACCTTTGTTTAAATACAAACTACGTCTCAAAGACCGTTCATAAGTA   8066
NC_042820.1                AAAATATTAATATAACCTTTGTTTAAATACAAACTACGTCTCAAAGACCGTTCATAAGTA   8311
m141102_iid=1228825_0      AAAATATTAATATAACCTTTGTTTAAATACAAACTACGTCTCAAAGACCGTTCATAAGTA   8135
m150504_iid=2876274_0      AAAATATTAATATAACCTTTGTTTAAATACAAACTACGTCTCAAAGACCGTTCATAAGTA   8138
m141030_iid=80036_0        AAAATATTAATATAACCTTTGTTTAAATACAAACTACGTCTCAAAGACCGTTCATAAGTA   7901
m150506_iid=2596494_0      AAAATATTAATATAACCTTTGTTTAAATACAAACTACGTCTCAAAGACCGTTCATAAGTA   8300
m150503_iid=2526862_0      AAAATATTAATATAACCTTTGTTTAAATACAAACTACGTCTCAAAGACCGTTCATAAGTA   7988
m150506_iid=2567000_0      AAAATATTAATATAACCTTTGTTTAAATACAAACTACGTCTCAAAGACCGTTCATAAGTA   5939
m150507_iid=3073271_0      AAAATATTAATATAACCTTTGTTTAAATACAAACTACGTCTCAAAGACCGTTCATAAGTA   7241
m150504_iid=3156087_0      AAAATATTAATATAACCTTTGTTTAAATACAAACTACGTCTCAAAGACCGTTCATAAGTA   7065
m150505_iid=2498502_0      AAAATATTAATATAACCTTTGTTTAAATACAAACTACGTCTCAAAGACCGTTCATAAGTA   4716
m141102_iid=754227_0       AAAATATTAATATAACCTTTGTTTAAATACAAACTACGTCTCAAAGACCGTTCATAAGTA   7277
m150507_iid=3469514_0      AAAATATTAATATAACCTTTGTTTAAATACAAACTACGTCTCAAAGACCGTTCATAAGTA   7835
Rdo_MitoGenome             AAAATATTAATATAACCTTTGTTTAAATACAAACTACGTCTCAAAGACCGTTCATAAGTA   8311
m141101_iid=614933_0       AAAATATTAATATAACCTTTGTTTAAATACAAACTACGTCTCAAAGACCGTTCATAAGTA   6860
```

nad4

```
m141031_iid=347546_0       ATATTAGCCAGACAAAATAAACCAGAAGAACAAATACCATGAGAAATTATTATAATTAAA   3080
m150506_iid=3127414_0      ATATTAGCCAGACAAAATAAACCAGAAGAACAA-TACCATGAGAAATTATTATAATTAAA   4815
m141102_iid=1245517_0      ATATTAGCCAGACAAAATAAACCAGAAGAACAAATACCATGAGAAATTATTATAATTAAA   6455
m141107_iid=1405727_0      ATATTAGCCAGACAAA-TAAACCAGAAGAACAAAT---ATGAGAAATTATTATAATTAAA   3646
m141102_iid=987614_0       ATATTAGCCAGACAAAATAAACCAGAAGAACAAATACCATGAGAAATTATTATAATTAAA   6633
m141108_iid=1701429_0      ATATTAGCCAGACAAAATAAACCAGAAGAACAAATACCATGAGAAATTATTATAATTAAA   3380
m141102_iid=987286_0       ATATTAGCCAGACAAAATAAACCAGAAGAACAAATACCATGAGAAATTATTATAATTAAA   4597
m150430_iid=3272125_0      ATATTAGCCAGACAAAATAAACCAGAAGAACAAATACCATGAGAAATTATTATAATTAAA   6113
m141102_iid=754225_0       ATATTAGCCAGACAAAATAAACCAGAAGAACAAATACCATGAGAAATTATTATAATTAAA   7283
m141107_iid=1409934_0      ATATTAGCCAGACAAAATAAACCAGAAGAACAAATACCATGAGAAATTATTATAATTAAA   7824
m150503_iid=3416920_0      ATATTAGCCAGACAAAATAAACCAGAAGAACAAATACCATGAGAAATTATTATAATTAAA   6541
m141108_iid=1800121_0      ATATTAGCCAGACAAAATAAACCAGAAGAACAAATACCATGAGAAATTATTATAATTAAA   6356
m141031_iid=147540_0       ATATTAGCCAGACAAAATAAACCAGAAGAACAAATACCATGAGAAATTATTATAATTAAA   6703
m150507_iid=3674924_0      ATATTAGCCAGACAAAATAAACCAGAAGAACAAATACCATGAGAAATTATTATAATTAAA   6852
m150503_iid=2923751_0      ATATTAGCCAGACAAAATAAACCAGAAGAACAAATACCATGAGAAATTATTATAATTAAA   6684
m141031_iid=115118_0       ATATTAGCCAGACAAAATAAACCAGAAGAACAAATACCATGAGAAATTATTATAATTAAA   5547
m150430_iid=2722448_0      ATATTAGCCAGACAAAATAAACCAGAAGAACAA-TACCATGAGAAATTATTATAATTAAA   5481
m150507_iid=3102647_0      ATATTAGCCAGACAAAATAAACCAGAAGAACAAATACCATGAGAAATTATTATAATTAAA   3781
m141108_iid=1811866_0      ATATTAGCCAGACAAAATAAACCAGAAGAACAAATACCATGAGAAATTATTATAATTAAA   5663
m150501_iid=2948495_0      ATATTAGCCAGACAAAATAAACCAGAAGA-CAAATACCATGAGAAATTATTATAATTAAA   5082
m150504_iid=3238195_0      ATATTAGCCAGACAAAATAAACCAGAAGAACAAATACCATGAGAAATTATTATAATTAAA   7419
m150503_iid=3388511_0      ATATTAGCCAGACAAAATAAACCAGAAGAACAAATACCATGAGAAATTATTATAATTAAA   5115
m150505_iid=3777611_0      ATATTAGCCAGACAAAATAAACCAGAAGAACAAATACCATGAGAAATTATTATAATTAAA   6538
m150430_iid=2438833_0      ATATTAGCCAGACAAAATAAACCAGAAGAACAAATACCATGAGAAATTATTATAATTAAA   6876
m141108_iid=1698969_0      ATATTAGCCAGACAAAATAAACCAGAAGAACAAATACCATGAGAAATTATTATAATTAAA   8005
m150430_iid=3059190_0      ATATTAGCCAGACAAAATAAACCAGAAGAACAAATACCATGAGAAATTATTATAATTAAA   3726
m141107_iid=1383067_0      ATATTAGCCAGACAAAATAAACCAGAAGAACAAATACCATGAGAAATTATTATAATTAAA   3728
m141101_iid=597008_0       ATATTAGCCAGACAAAATAAACCAGAAGAACAAATACCATGAGAAATTATTATAATTAAA   797
m150506_iid=2596496_0      ATATTAGCCAGACAAAATAAACCAGAAGAACAAATACCATGAGAAATTATTATAATTAAA   8354
m141102_iid=910051_0       ------------------------------------------------------------   7428
m150505_iid=3784017_0      ATATTAGCCAGACAAAATAAACCAGAAGAACAAATACCATGAGAAATTATTATAATTAAA   6652
m141101_iid=682151_0       ATATTAGCCAGACAAAATAAACCAGAAGAACAAATACCATGAGAAATTATTATAATTAAA   5720
m150501_iid=3748029_0      ATATTAGCCAGACAAAATAAACCAGAAGAACAAATACCATGAGAAATTATTATAATTAAA   6263
m150504_iid=2876272_0      ATATTAGCCAGACAAAATAAACCAGAAGA--AAAAACCATGAGAAATTATTATAATTAAA   8124
NC_042820.1                ATATTAGCCAGACAAAATAAACCAGAAGAACAAATACCATGAGAAATTATTATAATTAAA   8371
m141102_iid=1228825_0      ATATTAGCCAGACAAAATAAACCAGAAGAACAAATACCATGAGAAATTATTATAATTAAA   8195
m150504_iid=2876274_0      ATATTAGCCAGACAAAATAAACCAGAAGAACAAATACCATGAGAAATTATTATAATTAAA   8198
m141030_iid=80036_0        ATATTAGCCAGACAAAATAAACCAGAAGAACAAATACCATGAGAAATTATTATAATTAAA   7961
m150506_iid=2596494_0      ATATTAGCCAGACAAAATAAACCAGAAGAACAAATACCATGAGAAATTATTATAATTAAA   8360
m150503_iid=2526862_0      ATATTAGCCAGACAAAATAAACCAGAAGAACAAATACCATGAGAAATTATTATAATTAAA   8048
m150506_iid=2567000_0      ATATTAGCCAGACAAAATAAACCAGAAGAACAAATACCATGAGAAATTATTATAATTAAA   5999
m150507_iid=3073271_0      ATATTAGCCAGACAAAATAAACCAGAAGAACAAATACCATGAGAAATTATTATAATTAAA   7301
m150504_iid=3156087_0      ATATTAGCCAGACAAAATAAACCAGAAGAACAAATACCATGAGAAATTATTATAATTAAA   7125
m150505_iid=2498502_0      ATATTAGCCAGACAAAATAAACCAGAAGAACAAATACCATGAGAAATTATTATAATTAAA   4776
m141102_iid=754227_0       ATATTAGCCAGACAAAATAAACCAGAAGAACAAATACCATGAGAAATTATTATAATTAAA   7337
m150507_iid=3469514_0      ATATTAGCCAGACAAAATAAACCAGAAGAACAAATACCATGAGAAATTATTATAATTAAA   7895
Rdo_MitoGenome             ATATTAGCCAGACAAAATAAACCAGAAGAACAAATACCATGAGAAATTATTATAATTAAA   8371
```

```
m141101_iid=614933_0       ATATTAGCCAGACAAAATAAACCAGAAGAACAAATACCATGAGAAATTATTATAATTAAA   6920
```

nad4

```
m141031_iid=347546_0       GATCCAATAA-CCCTCAAGTTGTTAAAGTAAAGCACCCAGAAATTACTATACTT-TATGA   3138
m150506_iid=3127414_0      GATCCAATAA-CCCTCAAGTTGTTAAAGTAAAGCACCCAGAAATTACTATACTTATATGA   4874
m141102_iid=1245517_0      GATCCAATAACCCCTCAAGTTGTTAAAGTAAAG--CCCAGAAATTACTATACTTATATGA   6513
m141107_iid=1405727_0      GATCCAATAACCCCTCAAGTTGTTAAAGTAAAGCACCCAGAAATTACTATACTTATATGA   3706
m141102_iid=987614_0       GATCCAATAACCCCTCAAGTTGT-----TAAAGCACCCAGAAATTACTATACTTATATGA   6688
m141108_iid=1701429_0      GATCCAATAACCCCTCAAGTTGTTAAAGTAAAGCACCCAGAAATTACTATACTTATATGA   3440
m141102_iid=987286_0       GATCCAATAACCCCTCAAGTTGTTAAAGTAAAGCACCCAGAAATTACTATACTTATATGA   4657
m150430_iid=3272125_0      GATCCAATAACCCC--AAGTTGTTAAAGTAAAGCACCCAGAAATTACTATACTTATATGA   6171
m141102_iid=754225_0       GATCCAATAACCCCTCAAGTTGTTAAAGTAAAGCACCCAGAAATTACTATACTTATATGA   7343
m141107_iid=1409934_0      GATCCAATAACCCCTCAAGTTGTTAAAGTAAAGCACCCAGAAATTACTATACTTATATGA   7884
m150503_iid=3416920_0      GATCCAATAACCCCTCAAGTTGTTAAAGTAAAGCACCCAGAA-TTACTATACTTATATGA   6600
m141108_iid=1800121_0      GATCCAATAA-CCCTCAAGTTGTTAAAGTAAAGCACCCAGAAATTACTATACTTATATGA   6415
m141031_iid=147540_0       GATCCAATAACCCCTCAAGTTGTTAAAGTAAAGCACCCAGAAATTACTATACTTATATGA   6763
m150507_iid=3674924_0      GATCCAATAACCCCTCAAGTTGTTAAAGTAAAGCACCCAGAAATTACTATACTTATATGA   6912
m150503_iid=2923751_0      GATCCAATAA-CCCTCAAGTTGTTAAAGTAAAGCACCCAGAAATTACTATACTTATATGA   6743
m141031_iid=115118_0       GATCCAATAACCCCTCAAGTTGTTAAAGTAAAGCACCCAGAAATTACTATACTTATATGA   5607
m150430_iid=2722448_0      GATCCAATAACCCCTCAAGTTGTTAAAGTAAAGCACCCAGAAATTACTATACTTATATGA   5541
m150507_iid=3102647_0      GATCCAATAACCCCTCAAGTTGTTAAAGTAAAGCACCCAGAAATTACTATACTTATATGA   3841
m141108_iid=1811866_0      GATCCAATAACCCCTCAAGTTGTTAAAGTAAAGCACCCAGAAATTACTATACTTATATGA   5723
m150501_iid=2948495_0      GATCCAATAA-CCCTCAAGTTGTTAAAGTAAAGCACCCAGAAATTACTATACTTATATGA   5141
m150504_iid=3238195_0      GATCCAATAACCCCTCAAGTTGTTAAAGTAAAGCACCCAGAAATTACTATACTTATATGA   7479
m150503_iid=3388511_0      GATCCAATAACCCCTCAAGTTGTTAAAGTAAAGCACCCAGAAATTACTATACTTATATGA   5175
m150505_iid=3777611_0      GATCCAATAACCCCTCAAGTTGTTAAAGTAAAGCACCCAGAAATTACTATACTTATATGA   6598
m150430_iid=2438833_0      GATCCAATAACCCCTCAAGTTGTTAAAGTAAAGCACCCAGAAATTACTATACTTATATGA   6936
m141108_iid=1698969_0      GATCCAATAACCCCTCAAGTTGTTAAAGTAAAGCACCCAGAAATTACTATACTTATATGA   8065
m150430_iid=3059190_0      GATCCAATAACCCCTCAAGTTGTTAAAGTAAAGCACCCAGAAATTACTATACTTATATGA   3786
m141107_iid=1383067_0      GATCCAATAACCCCTCAAGTTGTTAAAGTAAAGCACCCAGAAATTACTATACTTATATGA   3788
m141101_iid=597008_0       GATCCAATAACCCCTCAAGTTGTTAAAGTAAAGCACCCAGAAATTACTATACTTATATGA   857
m150506_iid=2596496_0      GATCCAATAACCCCTCAAGTTGTTAAAGTAAAGCACCCAGAAATTACTATACTTATATGA   8414
m141102_iid=910051_0       ------------------------------------------------------------   7428
m150505_iid=3784017_0      GATCCAATAACCCCTCAAGTTGTTAAAGTAAAGCACCCAGAAATTACTATACTTATATGA   6712
m141101_iid=682151_0       GATCCAATAACCCCTCAAGTTGTTAAAGTAAAGCACCCAGAAATTACTATACTTATATGA   5780
m150501_iid=3748029_0      GATCCAATAACCCCTCAAGTTGTTAAAGTAAAGCACCCAGAAATTACTATACTTATATGA   6323
m150504_iid=2876272_0      GATCCAATAACCCCTCAAGTTGTTAAAGTAAAGCACCCAGAAATTACTATACTTATATGA   8184
NC_042820.1                GATCCAATAACCCCTCAAGTTGTTAAAGTAAAGCACCCAGAAATTACTATACTTATATGA   8431
m141102_iid=1228825_0      GATCCAATAACCCCTCAAGTTGTTAAAGTAAAGCACCCAGAAATTACTATACTTATATGA   8255
m150504_iid=2876274_0      GATCCAATAACCCCTCAAGTTGTTAAAGTAAAGCACCCAGAAATTACTATACTTATATGA   8258
m141030_iid=80036_0        GATCCAATAACCCCTCAAGTTGTTAAAGTAAAGCACCCAGAAATTACTATACTTATATGA   8021
m150506_iid=2596494_0      GATCCAATAACCCCTCAAGTTGTTAAAGTAAAGCACCCAGAAATTACTATACTTATATGA   8420
m150503_iid=2526862_0      GATCCAATAACCCCTCAAGTTGTTAAAGTAAAGCACCCAGAAATTACTATACTTATATGA   8108
m150506_iid=2567000_0      GATCCAATAACCCCTCAAGTTGTTAAAGTAAAGCACCCAGAAATTACTATACTTATATGA   6059
m150507_iid=3073271_0      GATCCAATAACCCCTCAAGTTGTTAAAGTAAAGCACCCAGAAATTACTATACTTATATGA   7361
m150504_iid=3156087_0      GATCCAATAACCCCTCAAGTTGTTAAAGTAAAGCACCCAGAAATTACTATACTTATATGA   7185
m150505_iid=2498502_0      GATCCAATAACCCCTCAAGTTGTTAAAGTAAAGCACCCAGAAATTACTATACTTATATGA   4836
m141102_iid=754227_0       GATCCAATAACCCCTCAAGTTGTTAAAGTAAAGCACCCAGAAATTACTATACTTATATGA   7397
m150507_iid=3469514_0      GATCCAATAACCCCTCAAGTTGTTAAAGTAAAGCACCCAGAAATTACTATACTTATATGA   7955
Rdo_MitoGenome             GATCCAATAACCCCTCAAGTTGTTAAAGTAAAGCACCCAGAAATTACTATACTTATATGA   8431
m141101_iid=614933_0       GATCCAATAACCCCTCAAGTTGTTAAAGTAAAGCACCCAGAAATTACTATACTTATATGA   6980
```

nad4

```
m141031_iid=347546_0       GCTACTGAAGAATAAGCAATTAATGATTTAATATCTCTTTGACGTAAACAAATTAAAGA-   3197
m150506_iid=3127414_0      GCTACTGAAGAATAAGCAATTAATGATTTAATATCTCTTTGACGTAAAAAAATTAAAGA-   4933
m141102_iid=1245517_0      GCTACTGAAGAATAAGCAATTAATGATTTAATATCTCTTTGACGTAAACAAATTAAAGAA   6573
m141107_iid=1405727_0      GCTACTGAAGAATAAGCAATTAATGATTTAATATCTCTTTGACGTAAACAAATTAAAGA-   3765
m141102_iid=987614_0       GCTACTGAAGAATAAGCAATTAATGATTTAATATCTCTTTGACGTAAACAAATTAAAGA-   6747
m141108_iid=1701429_0      GCTACTGAAGAATAAGCAATTAATGATTTAATATCTCTTTGACGTAAACAAATTAAAGA-   3499
m141102_iid=987286_0       GCTACTGAAGAATAAGCAATTAATGATTTAATATCTCTTTGACGTAAACAAATTAAAGA-   4716
m150430_iid=3272125_0      GCTACTGAAGAATAAGCAATTAATGATTTAATATCTCTTTGACGTAAACAAATTAAAGA-   6230
m141102_iid=754225_0       GCTACTGAAGAATAAGCAATTAATGATTTAATATCTCTTTGACGTAAACAAATTAAAGA-   7402
m141107_iid=1409934_0      GCTACTGAAGAATAAGCAATTAATGATTTAATATCTCTTTGACGTAAACAAATTAAAGA-   7943
m150503_iid=3416920_0      GCTACTGAAGAATAAGCAATTAATGATTTAATATCTCTTTGACGTAAACAAATTAAAGA-   6659
m141108_iid=1800121_0      GCTACTGAAGAATAAGCAATTAATGATTTAATATCTCTTTGACGTAAACAAATTAAAGA-   6474
m141031_iid=147540_0       GCTACTGAAGAATAAGCAATTAATGATTTAATATCTCTTTGACGTAAACAAATTAAAGA-   6822
m150507_iid=3674924_0      GCTACTGAAGAATAAGCAATTAATGATTTAATATCTCTTTGACGTAAACAAATTAAAGA-   6971
m150503_iid=2923751_0      GCTACTGAAGAATAAGCAATTAATGATTTAATATCTCTTTGACGTAAACAAATTAAAGA-   6802
m141031_iid=115118_0       GCTACTGAAGAATAAGCAATTAATGATTTAATATCTCTTTGACGTAAACAAATTAAAGA-   5666
m150430_iid=2722448_0      GCTACTGAAGAATAAGCAATTAATGATTTAATATCTCTTTGACGTAAACAAATTAAAGA-   5600
m150507_iid=3102647_0      GCTACTGAAGAATAAGCAATTAATGATTTAATATCTCTTTGACGTAAACAAATTAAAGA-   3900
```

```
m141108_iid=1811866_0      GCTACTGAAGAATAAGCAATTAATGATTTAATATCTCTTTGACGTAAACAAATTAAAGA-   5782
m150501_iid=2948495_0      GCTACTGAAGAATAAGCAATTAATGATTTAATATCTCTTTGACGTAAACAAATTAAAGA-   5200
m150504_iid=3238195_0      GCTACTGAAGAATAAGCAATTAATGATTTAATATCTCTTTGACGTAAACAAATTAAAGA-   7538
m150503_iid=3388511_0      GCTACTGAAGAATAAGCAATTAATGATTTAATATCTCTTTGACGTAAACAAATTAAAGA-   5234
m150505_iid=3777611_0      GCTACTGAAGAATAAGCAATTAATGATTTAATATCTCTTTGACGTAAACAAATTAAAGA-   6657
m150430_iid=2438833_0      GCTACTGAAGAATAAGCAATTAATGATTTAATATCTCTTTGACGTAAACAAATTAAAGA-   6995
m141108_iid=1698969_0      GCTACTGAAGAATAAGCAATTAATGATTTAATATCTCTTTGACGTAAACAAATTAAAGA-   8124
m150430_iid=3059190_0      GCTACTGAAGAATAAGCAATTAATGATTTAATATCTCTTTGACGTAAACAAATTAAAGA-   3845
m141107_iid=1383067_0      GCTACTGAAGAATAAGCAATTAATGATTTAATATCTCTTTGACGTAAACAAATTAAAGA-   3847
m141101_iid=597008_0       GCTACTGAAGAATAAGCAATTAATGATTTAATATCTCTTTGACGTAAACAAATTAAAGA-   916
m150506_iid=2596496_0      GCTACTGAAGAATAAGCAATTAATGATTTAATATCTCTTTGACGTAAACAAATTAAAGA-   8473
m141102_iid=910051_0       ------------------------------------------------------------   7428
m150505_iid=3784017_0      GCTACTGAAGAATAAGCAATTAATGATTTAATATCTCTTTGACGTAAACAAATTAAAGA-   6771
m141101_iid=682151_0       GCTACTGAAGAATAAGCAATTAATGATTTAATATCTCTTTGACGTAAACAAATTAAAGA-   5839
m150501_iid=3748029_0      GCTACTGAAGAATAAGCAATTAATGATTTAATATCTCTTTGACGTAAACAAATTAAAGA-   6382
m150504_iid=2876272_0      GCTACTGAAGAATAAGCAATTAATGATTTAATATCTCTTTGACGTAAACAAATTAAAGA-   8243
NC_042820.1                GCTACTGAAGAATAAGCAATTAATGATTTAATATCTCTTTGACGTAAACAAATTAAAGA-   8490
m141102_iid=1228825_0      GCTACTGAAGAATAAGCAATTAATGATTTAATATCTCTTTGACGTAAACAAATTAAAGA-   8314
m150504_iid=2876274_0      GCTACTGAAGAATAAGCAATTAATGATTTAATATCTCTTTGACGTAAACAAATTAAAGA-   8317
m141030_iid=80036_0        GCTACTGAAGAATAAGCAATTAATGATTTAATATCTCTTTGACGTAAACAAATTAAAGA-   8080
m150506_iid=2596494_0      GCTACTGAAGAATAAGCAATTAATGATTTAATATCTCTTTGACGTAAACAAATTAAAGA-   8479
m150503_iid=2526862_0      GCTACTGAAGAATAAGCAATTAATGATTTAATATCTCTTTGACGTAAACAAATTAAAGA-   8167
m150506_iid=2567000_0      GCTACTGAAGAATAAGCAATTAATGATTTAATATCTCTTTGACGTAAACAAATTAAAGA-   6118
m150507_iid=3073271_0      GCTACTGAAGAATAAGCAATTAATGATTTAATATCTCTTTGACGTAAACAAATTAAAGA-   7420
m150504_iid=3156087_0      GCTACTGAAGAATAAGCAATTAATGATTTAATATCTCTTTGACGTAAACAAATTAAAGA-   7244
m150505_iid=2498502_0      GCTACTGAAGAATAAGCAATTAATGATTTAATATCTCTTTGACGTAAACAAATTAAAGA-   4895
m141102_iid=754227_0       GCTACTGAAGAATAAGCAATTAATGATTTAATATCTCTTTGACGTAAACAAATTAAAGA-   7456
m150507_iid=3469514_0      GCTACTGAAGAATAAGCAATTAATGATTTAATATCTCTTTGACGTAAACAAATTAAAGA-   8014
Rdo_MitoGenome             GCTACTGAAGAATAAGCAATTAATGATTTAATATCTCTTTGACGTAAACAAATTAAAGA-   8490
m141101_iid=614933_0       GCTACTGAAGAATAAGCAATTAATGATTTAATATCTCTTTGACGTAAACAAATTAAAGA-   7039
```

nad4

```
m141031_iid=347546_0       AACAATTAAACCACCAAAAACAGAAATTCTTACAAAAAAAGTTCTAAAAAGGA-AACCTC   3256
m150506_iid=3127414_0      AACAATTAAACCACCAAAAACAGAAATTCTTACAAAAAAGTTCTAAAAAAAGG-AACCTC   4992
m141102_iid=1245517_0      AACAATTAAACCACCAAAAACAGAAATTCTTACAAAAAAAGTTCTAAAAAAGGAAACCTC   6633
m141107_iid=1405727_0      AACAATTAAACCACCAAAAACAGAAATTCTTACAAAAAAAGTTCTAAAAAAGGAAACCTC   3825
m141102_iid=987614_0       AACAATTAAACCACCAAAAACAGAAATTCTTACAAAAAAAGTTCTAAAAAAGGAAACCTC   6807
m141108_iid=1701429_0      AACAATTAAACCACCAAAAACAGAAATTCTTACAAAAAAAGTTCTAAAAAAGGAAACCTC   3559
m141102_iid=987286_0       AACAATTAAACCACCAAAAACAGAAATTCTTACAAAAAAAGTTCTAAAAAAGGAAACCTC   4776
m150430_iid=3272125_0      AACAATTAAACCACCAAAAACAGAAATTCTTACAAAAAAAGTTCTAAAAAAGGAAACCTC   6290
m141102_iid=754225_0       AACAATTAAACCACCAAAAACAGAAATTCTTACAAAAAAAGTTCTAAAAAAGGAAACCTC   7462
m141107_iid=1409934_0      AACAATTAAACCACCAAAAACAGAAATTCTTACAAAAAAAGTTCTAAAAAAGGAAACCTC   8003
m150503_iid=3416920_0      AACAATTAAACCACCAAAAACAGAAATTCTTACAAAAAAAGTTCTAAAAAAGGAAACCTC   6719
m141108_iid=1800121_0      AACAATTAAACCACCAAAAACAGAAATTCTA-CAAAAAAAGTTCTA-AAAAGGAAACCTC   6532
m141031_iid=147540_0       AACAATTAAACCACCAAAAACAGAAATTCTTACAAAAAAAGTTCTAAAAAAGGAAACCTC   6882
m150507_iid=3674924_0      AACAATTAAACCACCAAAAACAGAAATTCTTACAAAAAAAGTTCTAAAAAAGGAAACCTC   7031
m150503_iid=2923751_0      AACAATTAAACCACCAAAAACAGAAATTCTTACAAAAAAAGTTCTAAAAAAGGAAACCTC   6862
m141031_iid=115118_0       AACAATTAAACCACCAAAAACAGAAATTCTTACAAAAAAAGTTCTAAAAAAGGAAACCTC   5726
m150430_iid=2722448_0      AACAATTAAACCACCAAAAACAGAAATTCTTACAAAAAAAGTTCTAAAAAAGGAAACCTC   5660
m150507_iid=3102647_0      AACAATTAAACCACCAAAAACAGAAATTCTTACAAA-AAAGTTCTAAAAAAGGAAACCTC   3959
m141108_iid=1811866_0      AACAATTAAACCACCAAAAACAGAAATTCTTACAAAAAAAGTTCTAAAAAAGGAAACCTC   5842
m150501_iid=2948495_0      AACAATTAAACCACCAAAAACAGAAATTCTTACAAAAAAAGTTCTAA-AAAGGAAACCTC   5259
m150504_iid=3238195_0      AACAATTAAACCACCAAAAACAGAAATTCTTACAAAAAAAGTTCTAAAAAAGGAAACCTC   7598
m150503_iid=3388511_0      AACAATTAAACCACCAAAAACAGAAATTCTTACAAAAAAAGTTCTAAAAAAGGAAACCTC   5294
m150505_iid=3777611_0      AACAATTAAACCACCAAAAACAGAAATTCTTACAAAAAAAGTTCTAAAAAAGGAAACCTC   6717
m150430_iid=2438833_0      AACAATTAAACCACCAAAAACAGAAATTCTTACAAAAAAAGTTCTAAAAAAGGAAACCTC   7055
m141108_iid=1698969_0      AACAATTAAACCACCAAAAACAGAAATTCTTACAAAAAAAGTTCTAAAAAAGGAAACCTC   8184
m150430_iid=3059190_0      AACAATTAAACCACCAAAAACAGAAATTCTTACAAAAAAAGTTCTAAAAAAGGAAACCTC   3905
m141107_iid=1383067_0      AACAATTAAACCACCAAAAACAGAAATTCTTACAAAAAAAGTTCTAAAAAAGGAAACCTC   3907
m141101_iid=597008_0       AACAATTAAACCACCAAAAACAGAAATTCTTACAAAAAAAGTTCTAA-AAAGGAAACCTC   975
m150506_iid=2596496_0      AACAATTAAACCACCAAAAACAGAAATTCTTACAAAAAAAGTTCTAAAAAAGGAAACCTC   8533
m141102_iid=910051_0       ------------------------------------------------------------   7428
m150505_iid=3784017_0      AACAATTAAACCACCAAAAACAGAAATTCTTACAAAAAAAGTTCTAAAAAAGGAAACCTC   6831
m141101_iid=682151_0       AACAATTAAACCACCAAAAACAGAAATTCTTACAAAAAAAGTTCTAAAAAAGGAAACCTC   5899
m150501_iid=3748029_0      AACAATTAAACCACCAAAAACAGAAATTCTTACAAAAAAAGTTCTAAAAAAGGAAACCTC   6442
m150504_iid=2876272_0      AACAATTAAACCACCAAAAACAGAAATTCTTACAAAAAAAGTTCTAAAAAAGGAAACCTC   8303
NC_042820.1                AACAATTAAACCACCAAAAACAGAAATTCTTACAAAAAAAGTTCTAAAAAAGGAAACCTC   8550
m141102_iid=1228825_0      AACAATTAAACCACCAAAAACAGAAATTCTTACAAAAAAAGTTCTAAAAAAGGAAACCTC   8374
m150504_iid=2876274_0      AACAATTAAACCACCAAAAACAGAAATTCTTACAAAAAAAGTTCTAAAAAAGGAAACCTC   8377
m141030_iid=80036_0        AACAATTAAACCACCAAAAACAGAAATTCTTACAAAAAAAGTTCTAAAAAAGGAAACCTC   8140
m150506_iid=2596494_0      AACAATTAAACCACCAAAAACAGAAATTCTTACAAAAAAAGTTCTAAAAAAGGAAACCTC   8539
```

```
m150503_iid=2526862_0      AACAATTAAACCACCAAAAACAGAAATTCTTACAAAAAAAGTTCTAAAAAAGGAAACCTC   8227
m150506_iid=2567000_0      AACAATTAAACCACCAAAAACAGAAATTCTTACAAAAAAAGTTCTAAAAAAGGAAACCTC   6178
m150507_iid=3073271_0      AACAATTAAACCACCAAAAACAGAAATTCTTACAAAAAAAGTTCTAAAAAAGGAAACCTC   7480
m150504_iid=3156087_0      AACAATTAAACCACCAAAAACAGAAATTCTTACAAAAAAAGTTCTAAAAAAGGAAACCTC   7304
m150505_iid=2498502_0      AACAATTAAACCACCAAAAACAGAAATTCTTACAAAAAAAGTTCTAAAAAAGGAAACCTC   4955
m141102_iid=754227_0       AACAATTAAACCACCAAAAACAGAAATTCTTACAAAAAAAGTTCTAAAAAAGGAAACCTC   7516
m150507_iid=3469514_0      AACAATTAAACCACCAAAAACAGAAATTCTTACAAAAAAAGTTCTAAAAAAGGAAACCTC   8074
Rdo_MitoGenome             AACAATTAAACCACCAAAAACAGAAATTCTTACAAAAAAAGTTCTAAAAAAGGAAACCTC   8550
m141101_iid=614933_0       AACAATTAAACCACCAAAAACAGAAATTCTTACAAAAAAAGTTCTAAAAAAGGAAACCTC   7099
```

nad4

```
m141031_iid=347546_0       -CGCCTTAAATAAAGGAAAC--ACGCAATAAACCATAACCTCCCAATTTTAATATAACTC   3313
m150506_iid=3127414_0      -CGCCTTAAATAAAGGAAACACACGCAATAAACCATAACCTCCCAATTTTAATATAACTC   5051
m141102_iid=1245517_0      -CGCCTTAAATAAAGGAAACACACGCAATAAACCATAACCTCCCAATTTTAATATAACTC   6692
m141107_iid=1405727_0      CCGCCTTAAATA-AAGGAAAACACGCAATAAACCATAACCTCCCAATTTTAATATAACTC   3884
m141102_iid=987614_0       C-GCCTTAAATAAAGGAAACACACGCAATAAACCATAACCTCCCAATTTTAATATAACTC   6866
m141108_iid=1701429_0      C-GCCTTAAATAAAGGAAACACACGCAATAAACCATAACCTCCCAATTTTAATATAACTC   3618
m141102_iid=987286_0       C-GCCTTAAATAAAGGAAACACACGCAATAAACCATAACCTCCCAATTTTAATATAACTC   4835
m150430_iid=3272125_0      C-GCCTTAAATAAAGGAAACACACGCAATAAACCATAACCTCCCAATTTTAATATAACTC   6349
m141102_iid=754225_0       C-GCCTTAAATAAAGGAAACACACGCAATAAACCATAACCTCCCAATTTTAATATAACTC   7521
m141107_iid=1409934_0      C-GCCTTAAATAAAGGAAACACACGCAATAAACCATAACCTCCCAATTTTAATATAACTC   8062
m150503_iid=3416920_0      C-GCCTTAAATAAAGGAAACACACGCAATAAACCATAA-CTCCCAATTTTAATATAACTC   6777
m141108_iid=1800121_0      C-GCCTTAAATAAAGGAAACACACGCAATAAACCATAACCTCCCAATTTTAATATAACTC   6591
m141031_iid=147540_0       C-GCCTTAAATAAAGGAAACACACGCAATAAACCATAACCTCCCAATTTTAATATAACTC   6941
m150507_iid=3674924_0      C-GCCTTAAATAAAGGAAACACACGCAATAAACCATAACCTCCCAATTTTAATATAACTC   7090
m150503_iid=2923751_0      C-GCCTTAAATAAAGGAAACACACGCAATAAACCATAACCTCCCAATTTTAATATAACTC   6921
m141031_iid=115118_0       C-GCCTTAAATAAAGGAAACACACGCAATAAACCATAACCTCCCAATTTTAATATAACTC   5785
m150430_iid=2722448_0      C-GCCTTAAATAAAGGAAACACACGCAATAAACCATAACCTCCCAATTTTAATATAACTC   5719
m150507_iid=3102647_0      C-GCCTTAAATAAAGGAAACACACGCAATAAACCATAACCTCCCAATTTTAATATAACTC   4018
m141108_iid=1811866_0      C-GCCTTAAATAAAGGAAACACACGCAATAAACCATAACCTCCCAATTTTAATATAACTC   5901
m150501_iid=2948495_0      C-GCCTTAAATAAAGGAAACACACGCAATAAACCATAACCTCCCAATTTTAATATAACTC   5318
m150504_iid=3238195_0      C-GCCTTAAATAAAGGAAACACACGCAATAAACCATAACCTCCCAATTTTAATATAACTC   7657
m150503_iid=3388511_0      C-GCCTTAAATAAAGGAAACACACGCAATAAACCATAACCTCCCAATTTTAATATAACTC   5353
m150505_iid=3777611_0      C-GCCTTAAATAAAGGAAACACACGCAATAAACCATAACCTCCCAATTTTAATATAACTC   6776
m150430_iid=2438833_0      C-GCCTTAAATAAAGGAAACACACGCAATAAACCATAACCTCCCAATTTTAATATAACTC   7114
m141108_iid=1698969_0      C-GCCTTAAATAAAGGAAACACACGCAATAAACCATAACCTCCCAATTTTAATATAACTC   8243
m150430_iid=3059190_0      C-GCCTTAAATAAAGGAAACACACGCAATAAACCATAACCTCCCAATTTTAATATAACTC   3964
m141107_iid=1383067_0      C-GCCTTAAATAAAGGAAACACACGCAATAAACCATAACCTCCCAATTTTAATATAACTC   3966
m141101_iid=597008_0       C-GCCTTAAATAAAGGAAACACACGCAATAAACCATAACCTCCCAATTTTAATATAACTC   1034
m150506_iid=2596496_0      C-GCCTTAAATAAAGGAAACACACGCAATAAACCATAACCTCCCAATTTTAATATAACTC   8592
m141102_iid=910051_0       ------------------------------------------------------------   7428
m150505_iid=3784017_0      C-GCCTTAAATAAAGGAAACACACGCAATAAACCATAACCTCCCAATTTTAATATAACTC   6890
m141101_iid=682151_0       C-GCCTTAAATAAAGGAAACACACGCAATAAACCATAACCTCCCAATTTTAATATAACTC   5958
m150501_iid=3748029_0      C-GCCTTAAATAAAGGAAACACACGCAATAAACCATAACCTCCCAATTTTAATATAACTC   6501
m150504_iid=2876272_0      C-GCCTTAAATAAAGGAAACACACGCAATAAACCATAACCTCCCAATTTTAATATAACTC   8362
NC_042820.1                C-GCCTTAAATAAAGGAAACACACGCAATAAACCATATCCTCCCAATTTTAATATAACTC   8609
m141102_iid=1228825_0      C-GCCTTAAATAAAGGAAACACACGCAATAAACCATAACCTCCCAATTTTAATATAACTC   8433
m150504_iid=2876274_0      C-GCCTTAAATAAAGGAAACACACGCAATAA---ATAACCTCCCAATTTTAATATAACTC   8433
m141030_iid=80036_0        C-GCCTTAAATAAAGGAAACACACGCAATAAACCATAACCTCCCAATTTTAATATAACTC   8199
m150506_iid=2596494_0      C-GCCTTAAATAAAGGAAACACACGCAATAAACCATAACCTCCCAATTTTAATATAACTC   8598
m150503_iid=2526862_0      C-GCCTTAAATAAAGGAAACACACGCAATAAACCATAACCTCCCAATTTTAATATAACTC   8286
m150506_iid=2567000_0      C-GCCTTAAATAAAGGAAACACACGCAATAAACCATAACCTCCCAATTTTAATATAACTC   6237
m150507_iid=3073271_0      C-GCCTTAAATAAAGGAAACACACGCAATAAACCATAACCTCCCAATTTTAATATAACTC   7539
m150504_iid=3156087_0      C-GCCTTAAATAAAGGAAACACACGCAATAAACCATAACCTCCCAATTTTAATATAACTC   7363
m150505_iid=2498502_0      C-GCCTTAAATAAAGGAAACACACGCAATAAACCATAACCTCCCAATTTTAATATAACTC   5014
m141102_iid=754227_0       C-GCCTTAAATAAAGGAAACACACGCAATAAACCATAACCTCCCAATTTTAATATAACTC   7575
m150507_iid=3469514_0      C-GCCTTAAATAAAGGAAACACACGCAATAAACCATAACCTCCCAATTTTAATATAACTC   8133
Rdo_MitoGenome             C-GCCTTAAATAAAGGAAACACACGCAATAAACCATAACCTCCCAATTTTAATATAACTC   8609
m141101_iid=614933_0       C-GCCTTAAATAAAGGAAACACACGCAATAAACCATAACCTCCCAATTTTAATATAACTC   7158
```

nad4

```
m141031_iid=347546_0       CAGCCAAAATTATAGAACCAGAAACAGGAGCCTCTACATGAGCCTTAGGTAATCACAAAT   3373
m150506_iid=3127414_0      CAGCCAAAATTATAGAACCAGAAACAGGAGCCTCTACATGAGCCTTAGGTAATCACAAAT   5111
m141102_iid=1245517_0      CAGCAAA-ATTATAGAACCAGAAAAGGGAG-CTCTACATGAGCCTTAGGTAATCACAAAT   6750
m141107_iid=1405727_0      CAGCCAAAATTATAGAACCAGAAACAGGAGCCTCTACATGAGCCTTAGGTAATCACAAAT   3944
m141102_iid=987614_0       CAGCCAAAATTATAGAACCAGAAACAGGAGCCTCTACATGAGCCTTAGGTAATCACAAAT   6926
m141108_iid=1701429_0      CAGCCAAAATTATAGAACCAGAAACAGGAGCCTCTACATGAGCCTTAGGTAATCACAAAT   3678
m141102_iid=987286_0       CAGCCAAAATTATAGAACCAGAAACAGGAGCCTCTACATGAGCCTTAGGTAATCACAAAT   4895
m150430_iid=3272125_0      CAGCCAAAAT-TTAGAACCAGAAACAGGAGCCTCTACATGAGCCTTAGGTAATCACAAAT   6408
m141102_iid=754225_0       CAGCCAAAATTATAGAACCAGAAACAGGAGCCTCTACATGAGCCTTAGGTAATCACAAAT   7581
m141107_iid=1409934_0      CAGCCAAAATTATAGAACCAGAAACAGGAGCCTCTACATGAGCCTTAGGTAATCACAAAT   8122
```

```
m150503_iid=3416920_0     CAGCCAAAATTATAGAACCAGA-ACAGGAGCCTCTACATGAGCCTTAGGTAATCACAAAT   6836
m141108_iid=1800121_0     CAGCCAAAATTATAGAACCAGAAACAGGAGCCTCTACATGAGCCTTAGGTAATCACAAAT   6651
m141031_iid=147540_0      CAGCCAAAATTATAGAACCAGAAACAGGAGCCTCTACATGAGCCTTAGGTAATCACAAAT   7001
m150507_iid=3674924_0     CAGCCAAAATTATAGAACCAGAAACAGGAGCCTCTACATGAGCCTTAGGTAATCACAAAT   7150
m150503_iid=2923751_0     CAGCCAAAATTATAGAACCAGAAACAGGAGCCTCTACATGAGCCTTAGGTAATCACAAAT   6981
m141031_iid=115118_0      CAGCCAAAATTATAGAACCAGAAACAGGAGCCTCTACATGAGCCTTAGGTAATCACAAAT   5845
m150430_iid=2722448_0     CAGCCAAAATTATAGAACCAGAAACAGGAGCCTCTACATGAGCCTTAGGTAATCACAAAT   5779
m150507_iid=3102647_0     CAGCCAAAATTATAGAACCAGAAACAGGAGCCTCTACATGAGCCTTAGGTAATCACAAAT   4078
m141108_iid=1811866_0     CAGCCAAAATTATAGAACCAGAAACAGGAGCCTCTACATGAGCCTTAGGTAATCACAAAT   5961
m150501_iid=2948495_0     CAGCCAAAATTATAGAACCAGAAACAGGAGCCTCTACATGAGCCTTAGGTAATCACAAAT   5378
m150504_iid=3238195_0     CAGCCAAAATTATAGAACCAGAAACAGGAGCCTCTACATGAGCCTTAGGTAATCACAAAT   7717
m150503_iid=3388511_0     CAGCCAAAATTATAGAACCAGAAACAGGAGCCTCTACATGAGCCTTAGGTAATCACAAAT   5413
m150505_iid=3777611_0     CAGCCAAAATTATAGAACCAGAAACAGGAGCCTCTACATGAGCCTTAGGTAATCACAAAT   6836
m150430_iid=2438833_0     CAGCCAAAATTATAGAACCAGAAACAGGAGCCTCTACATGAGCCTTAGGTAATCACAAAT   7174
m141108_iid=1698969_0     CAGCCAAAATTATAGAACCAGAAACAGGAGCCTCTACATGAGCCTTAGGTAATCACAAAT   8303
m150430_iid=3059190_0     CAGCCAAAATTATAGAACCAGAAACAGGAGCCTCTACATGAGCCTTAGGTAATCACAAAT   4024
m141107_iid=1383067_0     CAGCCAAAATTATAGAACCAGAAACAGGAGCCTCTACATGAGCCTTAGGTAATCACAAAT   4026
m141101_iid=597008_0      CAGCCAAAATTATAGAACCAGAAACAGGAGCCTCTACATGAGCCTTAGGTAATCACAAAT   1094
m150506_iid=2596496_0     CAGCCAAAATTATAGAACCAGAAACAGGAGCCTCTACATGAGCCTTAGGTAATCACAAAT   8652
m141102_iid=910051_0      ------------------------------------------------------------   7428
m150505_iid=3784017_0     CAGCCAAAATTATAGAACCAGAAACAGGAGCCTCTACATGAGCCTTAGGTAATCACAAAT   6950
m141101_iid=682151_0      CAGCCAAAATTATAGAACCAGAAACAGGAGCCTCTACATGAGCCTTAGGTAATCACAAAT   6018
m150501_iid=3748029_0     CAGCCAAAATTATAGAACCAGAAACAGGAGCCTCTACATGAGCCTTAGGTAATCACAAAT   6561
m150504_iid=2876272_0     CAGCCAAAATTATAGAACCAGAAACAGGAGCCTCTACATGAGCCTTAGGTAATCACAAAT   8422
NC_042820.1               CAGCCAAAATTATAGAACCAGAAACAGGAGCCTCTACATGAGCCTTAGGTAATCACAAAT   8669
m141102_iid=1228825_0     CAGCCAAAATTATAGAACCAGAAACAGGAGCCTCTACATGAGCCTTAGGTAATCACAAAT   8493
m150504_iid=2876274_0     CAGCCAAAATTATAGAACCAGAAACAGGAGCCTCTACATGAGCCTTAGGTAATCACAAAT   8493
m141030_iid=80036_0       CAGCCAAAATTATAGAACCAGAAACAGGAGCCTCTACATGAGCCTTAGGTAATCACAAAT   8259
m150506_iid=2596494_0     CAGCCAAAATTATAGAACCAGAAACAGGAGCCTCTACATGAGCCTTAGGTAATCACAAAT   8658
m150503_iid=2526862_0     CAGCCAAAATTATAGAACCAGAAACAGGAGCCTCTACATGAGCCTTAGGTAATCACAAAT   8346
m150506_iid=2567000_0     CAGCCAAAATTATAGAACCAGAAACAGGAGCCTCTACATGAGCCTTAGGTAATCACAAAT   6297
m150507_iid=3073271_0     CAGCCAAAATTATAGAACCAGAAACAGGAGCCTCTACATGAGCCTTAGGTAATCACAAAT   7599
m150504_iid=3156087_0     CAGCCAAAATTATAGAACCAGAAACAGGAGCCTCTACATGAGCCTTAGGTAATCACAAAT   7423
m150505_iid=2498502_0     CAGCCAAAATTATAGAACCAGAAACAGGAGCCTCTACATGAGCCTTAGGTAATCACAAAT   5074
m141102_iid=754227_0      CAGCCAAAATTATAGAACCAGAAACAGGAGCCTCTACATGAGCCTTAGGTAATCACAAAT   7635
m150507_iid=3469514_0     CAGCCAAAATTATAGAACCAGAAACAGGAGCCTCTACATGAGCCTTAGGTAATCACAAAT   8193
Rdo_MitoGenome            CAGCCAAAATTATAGAACCAGAAACAGGAGCCTCTACATGAGCCTTAGGTAATCACAAAT   8669
m141101_iid=614933_0      CAGCCAAAATTATAGAACCAGAAACAGGAGCCTCTACATGAGCCTTAGGTAATCACAAAT   7218
```

nad4

```
m141031_iid=347546_0      GAACAACAAATATAGGAATTTTTACCAAAAAAA-CAAAAATTATTATATT-AAAAAAATT   3431
m150506_iid=3127414_0     GAACAACAAATATAGGAATTTTTACCAAAAAAA-CAAAAATTATTATATT-AAAAAAATT   5169
m141102_iid=1245517_0     GAACAACAAATATAGGAATTTTTACCAAAAAAAACAAAAATTATTATATT-AAAAAAATT   6809
m141107_iid=1405727_0     GAACAACAAT-ATAGGAATTTTTACCAAAAAAA-CAAAATT-ATTATATTAAAAAAAATT   4001
m141102_iid=987614_0      GAACAACAAATATAGGAATTTTTACCAAAAAAA-CAAAAATTATTATATTAAAAAAAATT   6985
m141108_iid=1701429_0     GAACAACAAATATAGGAATTTTTACCAAAAAAA-CAAAAATTATTATATTAAAAAAAATT   3737
m141102_iid=987286_0      GAACAACAAATATAGGAATTTTTACCAAAAAAA-CAAAAATTATTAT-ATTAAAAAAATT   4953
m150430_iid=3272125_0     GAACAACAAATATAGGAATTTTTACCAAAAAAA-CAAAATTATTATA-TTAAAAAAAATT   6466
m141102_iid=754225_0      GAACAACAAATATAGGAATTTTTACCAAAAAAA-CAAAAATTATTATATTAAAAAAAATT   7640
m141107_iid=1409934_0     GAACAACAAATATAGGAATTTTTACCAAAAAAA-CAAAAATTATTATATTAAAAAAAATT   8181
m150503_iid=3416920_0     GAACAACAAATATAGGAATTTTTACCAAAAAAA-CAAAAATTATTATATTA-AAAAAATT   6894
m141108_iid=1800121_0     GAACAACAAATATAGGAATTTTTACCAAAAAAA-ACAAAATTATTATATTAAAAAAAATT   6710
m141031_iid=147540_0      GAACAACAAATATAGGAATTTTTACCAAAAAAA-CAAAAATTATTATATTAAAAAAAATT   7060
m150507_iid=3674924_0     GAACAACAAATATAGGAATTTTTACCAAAAAAA-CAAAAATTATTATATTAAAAAAAATT   7209
m150503_iid=2923751_0     GAACAACAAATATAGGAATTTTTACCAAAAAAA-CAAAAATTATTATATTAAAAAAAATT   7040
m141031_iid=115118_0      GAACAACAAATATAGGAATTTTTACCAAAAAAA-CAAAAATTATTATATTAAAAAAAATT   5904
m150430_iid=2722448_0     GAACAACAAATATAGGAATTTTTACCAAAAAAA-CAAAAATTATTATATTA-AAAAAATT   5837
m150507_iid=3102647_0     GAACAACAAATATAGGAATTTTTACCAAAAAAA-CAAAAATTATTATATTAAAAAAAATT   4137
m141108_iid=1811866_0     GAACAACAAATATAGGAATTTTTACCAAAAAAA-CAAAAATTATTATATTAAAAAAAATT   6020
m150501_iid=2948495_0     GAACAACAAATATAGGAATTTTTACCAAAAAAA-CAAAAATTATTATATTAAAAAAAATT   5437
m150504_iid=3238195_0     GAACAACAAATATAGGAATTTTTACCAAAAAAA-CAAAAATTATTATATTAAAAAAAATT   7776
m150503_iid=3388511_0     GAACAACAAATATAGGAATTTTTACCAAAAAAA-C-AAAATTATTATATTAAAAAAAATT   5471
m150505_iid=3777611_0     GAACAACAAATATAGGAATTTTTACCAAAAAAA-CAAAAATTATTATATTAAAAAAAATT   6895
m150430_iid=2438833_0     GAACAACAAATATAGGAATTTTTACCAAAAAA---AAAAATTATTATATTAAAAAAAATT   7231
m141108_iid=1698969_0     GAACAACAAATATAGGAATTTTTACCAAAAAAA-CAAAAATTATTATATTAAAAAAAATT   8362
m150430_iid=3059190_0     GAACAACAAATATAGGAATTTTTACCAAAAAAA-CAAAAATTATTATATTAAAAAAAATT   4083
m141107_iid=1383067_0     GAACAACAAATATAGGAATTTTTACCAAAAAAA-CAAAAATTATTATATTAAAAAAAATT   4085
m141101_iid=597008_0      GAACAACAAATATAGGAATTTTTACCAAAAAAA-CAAAAATTATTATATTAAAAAAAATT   1153
m150506_iid=2596496_0     GAACAACAAATATAGGAATTTTTACCAAAAAAA-AAAAAT-TATTAT-ATTAAAAAAATT   8709
m141102_iid=910051_0      ------------------------------------------------------------   7428
m150505_iid=3784017_0     GAACAACAAATATAGGAATTTTTACCAAAAAAA-CAAAAATTATTATATTAAAAAAAATT   7009
```

```
m141101_iid=682151_0        GAACAACAAATATAGGAATTTTTACCAAAAAAA-CAAAAATTATTATATTAAAAAAAATT   6077
m150501_iid=3748029_0       GAACAACAAATATAGGAATTTTTACCAAAAAAA-CAAAAATTATTATATTAAAAAAAATT   6620
m150504_iid=2876272_0       GAACAACAAATATAGGAATTTTTACCAAAAAAA-CAAAAATTATTATATTAAAAAAAATT   8481
NC_042820.1                 GAACAACAAATATAGGAATTTTTACCAAAAAAA-CAAAAATTATTATATTAAAAAAAATT   8728
m141102_iid=1228825_0       GAACAACAAATATAGGAATTTTTACCAAAAAAA-CAAAAATTATTATATTAAAAAAAATT   8552
m150504_iid=2876274_0       GAACAACAAATATAGGAATTTTTACCAAAAAAA-CAAAAA--ATTATATTAAAAAAAATT   8550
m141030_iid=80036_0         GAACAACAAATATAGGAATTTTTACCAAAAAAA-CAAAAATTATTATATTAAAAAAAATT   8318
m150506_iid=2596494_0       GAACAACAAATATAGGAATTTTTACCAAAAAAA-CAAAAATTATTATATTAAAAAAAATT   8717
m150503_iid=2526862_0       GAACAACAAATATAGGAATTTTTACCAAAAAAA-CAAAAATTATTATATTAAAAAAAATT   8405
m150506_iid=2567000_0       GAACAACAAATATAGGAATTTTTACCAAAAAAA-CAAAAATTATTATATTAAAAAAAATT   6356
m150507_iid=3073271_0       GAACAACAAATATAGGAATTTTTACCAAAAAAA-CAAAAATTATTATATTAAAAAAAATT   7658
m150504_iid=3156087_0       GAACAACAAATATAGGAATTTTTACCAAAAAAA-CAAAAATTATTATATTAAAAAAAATT   7482
m150505_iid=2498502_0       GAACAACAAATATAGGAATTTTTACCAAAAAAA-CAAAAATTATTATATTAAAAAAAATT   5133
m141102_iid=754227_0        GAACAACAAATATAGGAATTTTTACCAAAAAAA-CAAAAATTATTATATTAAAAAAAATT   7694
m150507_iid=3469514_0       GAACAACAAATATAGGAATTTTTACCAAAAAAA-CAAAAATTATTATATTAAAAAAAATT   8252
Rdo_MitoGenome              GAACAACAAATATAGGAATTTTTACCAAAAAAA-CAAAAATTATTATATTAAAAAAAATT   8728
m141101_iid=614933_0        GAACAACAAATATAGGAATTTTTACCAAAAAAA-CAAAAATTATTATATTAAAAAAAATT   7277
```

nad4

```
m141031_iid=347546_0        ATTCTGTTAACTT-GAAATATTAAAAATATAGATAAAGAACCGAAACTTAAATAATAATG   3490
m150506_iid=3127414_0       ATTCTGTTAACTT-GAAATATTAAAAATATAGATAAAGAACCGAAACTTAAATAATAATG   5228
m141102_iid=1245517_0       ATTCTGTTAC--TTGAAATATTAAAAATATAGATAAAGAACCGAAACTTAAATAATAATG   6867
m141107_iid=1405727_0       ATTCTGTTAACGTTGAAATATTAAAAATATAGATAAAGAACCGAAACTTAAATAATAATG   4061
m141102_iid=987614_0        ATTCTGTTAACTT-GAAATATTAAAAATATAGATAAAGAACCGAAACTTAAATAATAATG   7044
m141108_iid=1701429_0       ATTCTGTTAACTT-GAAATATTAAAAATATAGATAAAGAACCGAAACTTAAATAATAATG   3796
m141102_iid=987286_0        ATTCTGTTAACTT-GAAATATTAAAAATATAGATAAAGAACCGAAACTTAAATAATAATG   5012
m150430_iid=3272125_0       ATTCTGTTAACTT-GAAATATTAAAAATATAGATAAAGAACCGAAACTTAAATAATAATG   6525
m141102_iid=754225_0        ATTCTGTTAACTT-GAAATATTAAAAATATAGATAAAGAACCGAAACTTAAATAATAATG   7699
m141107_iid=1409934_0       ATTCTGTTAACTT-GAAATATTAAAAATATAGATAAAGAACCGAAACTTAAATAATAATG   8240
m150503_iid=3416920_0       ATTCTGTTAACTT-GAAATATTAAAAATATAGATAAAGAACCGAAACTTAAATAATAATG   6953
m141108_iid=1800121_0       ATTCTGTTAACTT-GAAATATTAAAAATATAGATAAAGAACCGAAACTTAAATAATAATG   6769
m141031_iid=147540_0        ATTCTGTTAACTT-GAAATATTAAAAATATAGATAAAGAACCGAAACTTAAATAATAATG   7119
m150507_iid=3674924_0       ATTCTGTTAACTT-GAAATATTAAAAATATAGATAAAGAACCGAAACTTAAATAATAATG   7268
m150503_iid=2923751_0       ATTCTGTTAACTT-GAAATATTAAAAATATAGATAAAGAACCGAAACTTAAATAATAATG   7099
m141031_iid=115118_0        ATTCTGTTAACTT-GAAATATTAAAAATATAGATAAAGAACCGAAACTTAAATAATAATG   5963
m150430_iid=2722448_0       ATTCTGTTAACTT-GAAATATTAAAAATATAGATAAAGAACCGAAACTTAAATAATAATG   5896
m150507_iid=3102647_0       ATTCTGTTAACTT-GAAATATTAAAAATATAGATAAAGAACCGAAACTTAAATAATAATG   4196
m141108_iid=1811866_0       ATTCTGTTAACTT-GAAATATTAAAAATATAGATAAAGAACCGAAACTTAAATAATAATG   6079
m150501_iid=2948495_0       ATTCTGTTAACTT-GAAATATTAAAAATATAGATAAAGAACCGAAACTTAAATAATAATG   5496
m150504_iid=3238195_0       ATTCTGTTAACTT-GAAATATTAAAAATATAGATAAAGAACCGAAACTTAAATAATAATG   7835
m150503_iid=3388511_0       ATTCTGTTAACTT-GAAATATTAAAAATATAGATAAAGAACCGAAACTTAAATAATAATG   5530
m150505_iid=3777611_0       ATTCTGTTAACTT-GAAATATTAAAAATATAGATAAAGAACCGAAACTTAAATAATAATG   6954
m150430_iid=2438833_0       ATTCTGTTAACTT-GAAATATTAAAAATATAGATAAAGAACCGAAACTTAAATAATAATG   7290
m141108_iid=1698969_0       ATTCTGTTAACTT-GAAATATTAAAAATATAGATAAAGAACCGAAACTTAAATAATAATG   8421
m150430_iid=3059190_0       ATTCTGTTAACTT-GAAATATTAAAAATATAGATAAAGAACCGAAACTTAAATAATAATG   4142
m141107_iid=1383067_0       ATTCTGTTAACTT-GAAATATTAAAAATATAGATAAAGAACCGAAACTTAAATAATAATG   4144
m141101_iid=597008_0        ATTCTGTTAACTT-GAAATATTAAAAATATAGATAAAGAACCGAAACTTAAATAATAATG   1212
m150506_iid=2596496_0       ATTCTGTTAACTT-GAAATATTAAAAATATAGATAAAGAACCGAAACTTAAATAATAATG   8768
m141102_iid=910051_0        ------------------------------------------------------------   7428
m150505_iid=3784017_0       ATTCTGTTAACTT-GAAATATTAAAAATATAGATAAAGAACCGAAACTTAAATAATAATG   7068
m141101_iid=682151_0        ATTCTGTTAACTT-GAAATATTAAAAATATAGATAAAGAACCGAAACTTAAATAATAATG   6136
m150501_iid=3748029_0       ATTCTGTTAACTT-GAAATATTAAAAATATAGATAAAGAACCGAAACTTAAATAATAATG   6679
m150504_iid=2876272_0       ATTCTGTTAACTT-GAAATATTAAAAATATAGATAAAGAACCGAAACTTAAATAATAATG   8540
NC_042820.1                 ATTCTGTTAACTT-GAAATATTAAAAATATAGATAAAGAACCGAAACTTAAATAATAATG   8787
m141102_iid=1228825_0       ATTCTGTTAACTT-GAAATATTAAAAATATAGATAAAGAACCGAAACTTAAATAATAATG   8611
m150504_iid=2876274_0       ATTCTGTTAACTT-GAAATATTAAAAATATAGATAAAGAACCGAAACTTAAATAATAATG   8609
m141030_iid=80036_0         ATTCTGTTAACTT-GAAATATTAAAAATATAGATAAAGAACCGAAACTTAAATAATAATG   8377
m150506_iid=2596494_0       ATTCTGTTAACTT-GAAATATTAAAAATATAGATAAAGAACCGAAACTTAAATAATAATG   8776
m150503_iid=2526862_0       ATTCTGTTAACTT-GAAATATTAAAAATATAGATAAAGAACCGAAACTTAAATAATAATG   8464
m150506_iid=2567000_0       ATTCTGTTAACTT-GAAATATTAAAAATATAGATAAAGAACCGAAACTTAAATAATAATG   6415
m150507_iid=3073271_0       ATTCTGTTAACTT-GAAATATTAAAAATATAGATAAAGAACCGAAACTTAAATAATAATG   7717
m150504_iid=3156087_0       ATTCTGTTAACTT-GAAATATTAAAAATATAGATAAAGAACCGAAACTTAAATAATAATG   7541
m150505_iid=2498502_0       ATTCTGTTAACTT-GAAATATTAAAAATATAGATAAAGAACCGAAACTTAAATAATAATG   5192
m141102_iid=754227_0        ATTCTGTTAACTT-GAAATATTAAAAATATAGATAAAGAACCGAAACTTAAATAATAATG   7753
m150507_iid=3469514_0       ATTCTGTTAACTT-GAAATATTAAAAATATAGATAAAGAACCGAAACTTAAATAATAATG   8311
Rdo_MitoGenome              ATTCTGTTAACTT-GAAATATTAAAAATATAGATAAAGAACCGAAACTTAAATAATAATG   8787
m141101_iid=614933_0        ATTCTGTTAACTT-GAAATATTAAAAATATAGATAAAGAACCGAAACTTAAATAATAATG   7336
```

nad4

```
m141031_iid=347546_0        TATTAACCCCAAAAATATAGGTAAAGAAGCAAAAAGAGTATAAAACAACAAATAAAAACC   3550
m150506_iid=3127414_0       TATTAACCCCAAAAATATAGGTAAAGAAGCAAAAAGAGTATAAAACA-CAAATAAAAACC   5287
```

```
m141102_iid=1245517_0      TATTAACCCCAA-AATATAGGTAAAGAAGCAAAAAGAGTATAAAACAACAAT-AAAAACC   6925
m141107_iid=1405727_0      TATTAACCCCAAAAATATAGGTAAAGAAGCAAAAAGAGTATAAAACAACAAATAAAAACC   4121
m141102_iid=987614_0       TATTAACCCCAAAAATATAGGTAAAGAAGCAAAAAGAGTATAAAACAACAAATAAAAACC   7104
m141108_iid=1701429_0      TATTAACCCCAAAAATATAGGTAAAGAAGCAAAAAGAGTATAAAACAACAAATAAAAACC   3856
m141102_iid=987286_0       TATTAACCCCAAAAATATAGGTAAAGAAGCAAAAAGAGTATAAA-AAACAAATAAAAACC   5071
m150430_iid=3272125_0      TATTAACCCCAAAAATATAGGTAAAGAAGCAAAAAGAGTATAAAACAACAAATAAAAACC   6585
m141102_iid=754225_0       TATTAACCCCAAAAATATAGGTAAAGAAGCAAAAAGAGTATAAAACAACAAATAAAAACC   7759
m141107_iid=1409934_0      TATTAACCCCAAAAATATAGGTAAAGAAGCAAAAAGAGTATAAAACAACAAATAAAAACC   8300
m150503_iid=3416920_0      TATTAACCCCAAAAATATAGGTAAAGAAGCAAAAAGAGTATAAAACAACAAATAAAAACC   7013
m141108_iid=1800121_0      TATTAACCCCAAAAATATAGGTAAAGAAGCAAAAAGAGTATAAAACAACAAATAAAAACC   6829
m141031_iid=147540_0       TATTAACCCCAAAAATATAGGTAAAGAAGCAAAAAGAGTATAAAACAACAAATAAAAACC   7179
m150507_iid=3674924_0      TATTAACCCCAAAAATATAGGTAAAGAAGCAAAAAGAGTATAAAACAACAAATAAAAACC   7328
m150503_iid=2923751_0      TATTAACC-CAAAAATATAGGTAAAGAAGCAAAAAGAGTATAAAACAACAAATAAAAACC   7158
m141031_iid=115118_0       TATTAACCCCAAAAATATAGGTAAAGAAGCAAAAAGAGTATAAAACAACAAATAAAAACC   6023
m150430_iid=2722448_0      TATTAACCCCAAAAATATAGGTAAAGAAGCAAAAAGAGTATAAAACAACAAAT--AAACA   5954
m150507_iid=3102647_0      TATTAACC-CAAAAATATAGGTAAAGAAGCAAAAAGAGTATAAAACAACAAATAAAAACC   4255
m141108_iid=1811866_0      TATTAACCCCAAAAATATAGGTAAAGAAGCAAAAAGAGTATAAAACAACAAATAAAAACC   6139
m150501_iid=2948495_0      TATTAACCCCAAAAATATAGGTAAAGAAGCAAA-AGAGTATAAAACAACAAATAAAAACC   5555
m150504_iid=3238195_0      TATTAACCCCAAAAATATAGGTAAAGAAGCAAAAAGAGTATAAAACAACAAATAAAAACC   7895
m150503_iid=3388511_0      TATTAACCCCAAAAATATAGGTAAAGAAGCAAAAAGAGTATAAAACAACAAATAAAAACC   5590
m150505_iid=3777611_0      TATTAACCCCAAAAATATAGGTAAAGAAGCAAAAAGAGTATAAAACAACAAATAAAAACC   7014
m150430_iid=2438833_0      TATTAACCCCAAAAATATAGGTAAAGAAGCAAAAAGAGTATAAAACAACAAATAAAAACC   7350
m141108_iid=1698969_0      TATTAACCCCAAAAATATAGGTAAAGAAGCAAAAAGAGTATAAAACAACAAATAAAAACC   8481
m150430_iid=3059190_0      TATTAACCCCAAAAATATAGGTAAAGAAGCAAAAAGAGTATAAAACAACAAATAAAAACC   4202
m141107_iid=1383067_0      TATTAACCCCAAAAATATAGGTAAAGAAGCAAAAAGAGTATAAAACAACAAATAAAAACC   4204
m141101_iid=597008_0       TATTAACCCCAAAAATATAGGTAAAGAAGCAAAAAGAGTATAAAACAACAAATAAAAACC   1272
m150506_iid=2596496_0      TATTAACCCCAAAAATATAGGTAAAGAAGCAAAAAGAGTATAAAACAACAAATAAAAACC   8828
m141102_iid=910051_0       ------------------------------------------------------------   7428
m150505_iid=3784017_0      TATTAACCCCAAAAATATAGGTAAAGAAGCAAAAAGAGTATAAAACAACAAATAAAAACC   7128
m141101_iid=682151_0       TATTAACCCCAAAAATATAGGTAAAGAAGCAAAAAGAGTATAAAACAACAAATAAAAACC   6196
m150501_iid=3748029_0      TATTAACCCCAAAAATATAGGTAAAGAAGCAAAAAGAGTATAAAACAACAAATAAAAACC   6739
m150504_iid=2876272_0      TATTAACCCCAAAAATATAGGTAAAGAAGCAAAAAGAGTATAAAACAACAAATAAAAA--   8598
NC_042820.1                TATTAACCCCAAAAATATAGGTAAAGAAGCAAAAAGAGTATAAAACAACAAATAAAAACC   8847
m141102_iid=1228825_0      TATTAACCCCAAAAATATAGGTAAAGAAGCAAAAAGAGTATAAAACAACAAATAAAAACC   8671
m150504_iid=2876274_0      TATTAACCCCAAAAATATAGGTAAAGAAGCAAAAAGAGTATAAAACAACAAATAAAAACC   8669
m141030_iid=80036_0        TATTAACCCCAAAAATATAGGTAAAGAAGCAAAAAGAGTATAAAACAACAAATAAAAACC   8437
m150506_iid=2596494_0      TATTAACCCCAAAAATATAGGTAAAGAAGCAAAAAGAGTATAAAACAACAAATAAAAACC   8836
m150503_iid=2526862_0      TATTAACCCCAAAAATATAGGTAAAGAAGCAAAAAGAGTATAAAACAACAAATAAAAACC   8524
m150506_iid=2567000_0      TATTAACCCCAAAAATATAGGTAAAGAAGCAAAAAGAGTATAAAACAACAAATAAAAACC   6475
m150507_iid=3073271_0      TATTAACCCCAAAAATATAGGTAAAGAAGCAAAAAGAGTATAAAACAACAAATAAAAACC   7777
m150504_iid=3156087_0      TATTAACCCCAAAAATATAGGTAAAGAAGCAAAAAGAGTATAAAACAACAAATAAAAACC   7601
m150505_iid=2498502_0      TATTAACCCCAAAAATATAGGTAAAGAAGCAAAAAGAGTATAAAACAACAAATAAAAACC   5252
m141102_iid=754227_0       TATTAACCCCAAAAATATAGGTAAAGAAGCAAAAAGAGTATAAAACAACAAATAAAAACC   7813
m150507_iid=3469514_0      TATTAACCCCAAAAATATAGGTAAAGAAGCAAAAAGAGTATAAAACAACAAATAAAAACC   8371
Rdo_MitoGenome             TATTAACCCCAAAAATATAGGTAAAGAAGCAAAAAGAGTATAAAACAACAAATAAAAACC   8847
m141101_iid=614933_0       TATTAACCCCAAAAATATAGGTAAAGAAGCAAAAAGAGTATAAAACAACAAATAAAAACC   7396

                                                       nad4

m141031_iid=347546_0       AGCCTGGATTCG-TTCTGGTTGATATCCTCAACCCAAAATTAA-AATAAAGTAGGAATTA   3608
m150506_iid=3127414_0      AGCCTGGATTCG-TTCTGGTTGATATCCTCAACCCAAAATTAAAAATAAAGTAGGAATTA   5346
m141102_iid=1245517_0      AGCCTGGATTCGT-TCTGGTTGATATCCTCAACCAAAA-TTAAAAATAAAGTAGGAATTA   6983
m141107_iid=1405727_0      AGCCTGGATTCGT-TCTGGTTGATATCCTCAACCCAAAATTAAAAATAAAGTAGGAATTA   4180
m141102_iid=987614_0       AGCCTGGATTCGTTTCTGGTTGATATCCTCAACCCAAAATTAAAAATAAAGTAGGAATTA   7164
m141108_iid=1701429_0      AGCCTGGATTCG-TTCTGGTTGATATCCTCAACCCAAAATTAAAAATAAAGTAGGAATTA   3915
m141102_iid=987286_0       AGCCTGGATTCG-TTCTGGTTGATATCCTCAACCCAAAATTAAAAATAAAGTAGGAATTA   5130
m150430_iid=3272125_0      AGCCTGGATTCG-TTCTGGTTGATATCCTCAACCCAAAATTAAAAATAAAGTAGGAATTA   6644
m141102_iid=754225_0       AGCCTGGATTCG-TTCTGGTTGATATCCTCAACCCAAAATTAAAAATAAAGTAGGAATTA   7818
m141107_iid=1409934_0      AGCCTGGATTCG-TTCTGGTTGATATCCTCAACCCAAAATTAAAAATAAAGTAGGAATTA   8359
m150503_iid=3416920_0      AGCCTGGATTCG-TTCTGGTTGATATCCTCAACCCAAAATTAAAAATAAAGTAGGAATTA   7072
m141108_iid=1800121_0      AGCCTGGATTCG-TTCTGGTTGATATCCTCAACCCAAAATTAAAAAAAAAGTAGGAATTA   6888
m141031_iid=147540_0       AGCCTGGATTCG-TTCTGGTTGATATCCTCAACCCAAAATTAAAAATAAAGTAGGAATTA   7238
m150507_iid=3674924_0      AGCCTGGATTCG-TTCTGGTTGATATCCTCAACCCAAAATTAAAAATAAAGTAGGAATTA   7387
m150503_iid=2923751_0      AGCCTGGATTCG-TTCTGGTTGATATCCTCAACCCAAAATTAAAAATAAAGTAGGAATTA   7217
m141031_iid=115118_0       AGCCTGGATTCG-TTCTGGTTGATATCCTCAACCCAAAATTAAAAATAAAGTAGGAATTA   6082
m150430_iid=2722448_0      AGCCTGGATTCG-TTCTGGTTGATATCCTCAACCCAAAATTAAAAATAAAGTAGGAATTA   6013
m150507_iid=3102647_0      AGCCTGGATTCG-TTCTGGTTGATATCCTCAACCCAAAATTAAAAATAAAGTAGGAATTA   4314
m141108_iid=1811866_0      AGCCTGGATTCG-TTCTGGTTGATATCCTCAACCCAAAATTAAAAATAAAGTAGGAATTA   6198
m150501_iid=2948495_0      AGCCTGGATTCG-TTCTGGTTGATATCCTCAACCCAAAATTAAAAATAAAGTAGGAATTA   5614
m150504_iid=3238195_0      AGCCTGGATTCG-TTCTGGTTGATATCCTCAACCCAAAATTAAAAATAAAGTAGGAATTA   7954
m150503_iid=3388511_0      AGCCTGGATTCG-TTCTGGTTGATATCCTCAACCCAAAATTAAAAATAAAGTAGGAATTA   5649
m150505_iid=3777611_0      AGCCTGGATTCG-TTCTGGTTGATATCCTCAACCCAAAATTAAAAATAAAGTAGGAATTA   7073
```

```
m150430_iid=2438833_0      AGCCTGGATTCG-TTCTGGTTGATATCCTCAACCCAAAATTAAAAATAAAGTAGGAATTA   7409
m141108_iid=1698969_0      AGCCTGGATTCG-TTCTGGTTGATATCCTCAACCCAAAATTAAAAATAAAGTAGGAATTA   8540
m150430_iid=3059190_0      AGCCTGGATTCG-TTCTGGTTGATATCCTCAACCCAAAATTAAAAATAAAGTAGGAATTA   4261
m141107_iid=1383067_0      AGCCTGGATTCG-TTCTGGTTGATATCCTCAACCCAAAATTAAAAATAAAGTAGGAATTA   4263
m141101_iid=597008_0       AGCCTGGATTCG-TTCTGGTTGATATCCTCAACCCAAAATTAAAAATAAAGTAGGAATTA   1331
m150506_iid=2596496_0      AGCCTGGATTCG-TTCTGGTTGATATCCTCAACCCAAAATTAAAAATAAAGTAGGAATTA   8887
m141102_iid=910051_0       ------------------------------------------------------------   7428
m150505_iid=3784017_0      AGCCTGGATTCG-TTCTGGTTGATATCCTCAACCCAAAATTAAAAATAAAGTAGGAATTA   7187
m141101_iid=682151_0       AGCCTGGATTCG-TTCTGGTTGATATCCTCAACCCAAAATTAAAAATAAAGTAGGAATTA   6255
m150501_iid=3748029_0      AGCCTGGATTCG-TTCTGGTTGATATCCTCAACCCAAAATTAAAAATAAAGTAGGAATTA   6798
m150504_iid=2876272_0      --CCTGGATTCG-TTCTGGTTGATATCCTCAACCCAAAATTAAAAATAAAGTAGGAATTA   8655
NC_042820.1                AGCCTGGATTCG-TTCTGGTTGATATCCTCAACCCAAAATTAAAAATAAAGTAGGAATTA   8906
m141102_iid=1228825_0      AGCCTGGATTCG-TTCTGGTTGATATCCTCAACCCAAAATTAAAAATAAAGTAGGAATTA   8730
m150504_iid=2876274_0      AGCCTGGATTCG-TTCTGGTTGATATCCTCAACCCAAAATTAAAAATAAAGTAGGAATTA   8728
m141030_iid=80036_0        AGCCTGGATTCG-TTCTGGTTGATATCCTCAACCCAAAATTAAAAATAAAGTAGGAATTA   8496
m150506_iid=2596494_0      AGCCTGGATTCG-TTCTGGTTGATATCCTCAACCCAAAATTAAAAATAAAGTAGGAATTA   8895
m150503_iid=2526862_0      AGCCTGGATTCG-TTCTGGTTGATATCCTCAACCCAAAATTAAAAATAAAGTAGGAATTA   8583
m150506_iid=2567000_0      AGCCTGGATTCG-TTCTGGTTGATATCCTCAACCCAAAATTAAAAATAAAGTAGGAATTA   6534
m150507_iid=3073271_0      AGCCTGGATTCG-TTCTGGTTGATATCCTCAACCCAAAATTAAAAATAAAGTAGGAATTA   7836
m150504_iid=3156087_0      AGCCTGGATTCG-TTCTGGTTGATATCCTCAACCCAAAATTAAAAATAAAGTAGGAATTA   7660
m150505_iid=2498502_0      AGCCTGGATTCG-TTCTGGTTGATATCCTCAACCCAAAATTAAAAATAAAGTAGGAATTA   5311
m141102_iid=754227_0       AGCCTGGATTCG-TTCTGGTTGATATCCTCAACCCAAAATTAAAAATAAAGTAGGAATTA   7872
m150507_iid=3469514_0      AGCCTGGATTCG-TTCTGGTTGATATCCTCAACCCAAAATTAAAAATAAAGTAGGAATTA   8430
Rdo_MitoGenome             AGCCTGGATTCG-TTCTGGTTGATATCCTCAACCCAAAATTAAAAATAAAGTAGGAATTA   8906
m141101_iid=614933_0       AGCCTGGATTCG-TTCTGGTTGATATCCTCAACCCAAAATTAAAAATAAAGTAGGAATTA   7455

                                                  nad4

m141031_iid=347546_0       AACTAGACTC-AAAAA-ACATAAAATAAAATAAAGTTTAAAGAACTAAAAGCGCAAATTA   3666
m150506_iid=3127414_0      AACTAGACTC-AAAAATACATAAAATAAAATAAAGTTTAAAGAACTAAAAGCGCAAATTA   5405
m141102_iid=1245517_0      AACTAGACTCAAAAAATACATAAAATAAAATAAAGTTTAAAGAACTAAAAGCGCAAAATT   7043
m141107_iid=1405727_0      AACTAGACTCAAAAAATACATAAAATAAAATAAAGTTTAAAGAACTAAAAGCGCAAATTA   4240
m141102_iid=987614_0       AACTAGACTCAAAAAATACATAAAATAAAATAAAGTTTAAAGAACTAAAAGCGCAAATTA   7224
m141108_iid=1701429_0      AACTAGACTCAAAAAATACATAAAATAAAATAAAGTTTAAAGAACTAAAAGCGCAAATTA   3975
m141102_iid=987286_0       AACTAGACTCAAAAAATACATAAAATAAAATAAAGTTTAAAGAACTAAAAGCGCAAATTA   5190
m150430_iid=3272125_0      AACTAGACTCAAAAAATACATAAAATAAAATAAAGTTTAAAGAACTAAAAGCGCAAATTA   6704
m141102_iid=754225_0       AACTAGACTCAAAAAATACATAAAATAAAATAAAGTTTAAAGAACTAAAAGCGCAAATTA   7878
m141107_iid=1409934_0      AACTAGACTCAAAAAATACATAAAATAAAATAAAGTTTAAAGAACTAAAAGCGCAAATTA   8419
m150503_iid=3416920_0      AACTAGACTCAAAAAATACATAAAATAAAATAAAGTTTAAAGAACTAAAAGCGCAAATTA   7132
m141108_iid=1800121_0      AACTAGACTCAAAAAATACATAAAATAAAATAAAGTTTAAAGAACTAAAAGCGCAAATTA   6948
m141031_iid=147540_0       AACTAGACTCAAAAAATACATAAAATAAAATAAAGTTTAAAGAACTAAAAGCGCAAATTA   7298
m150507_iid=3674924_0      AACTAGACTCAAAAAATACATAAAATAAAATAAAGTTTAAAGAACTAAAAGCGCAAATTA   7447
m150503_iid=2923751_0      AACTAGACTCAAAAATA-CATA--AAAAAATAAAGTTTAAAGAACTAAAAGCGCAAATTA   7274
m141031_iid=115118_0       AACTAGACTCAAAAAATACATAAAATAAAATAAAGTTTAAAGAACTAAAAGCGCAAATTA   6142
m150430_iid=2722448_0      AACTAGACTCAAAAAATACATAAAATAAAATAAAGTTTAAAGAACTAAAAGCGCAAATTA   6073
m150507_iid=3102647_0      AACTAGACTCAAAAAATACATAAAATAAAATAAAGTTTAAAGAACTAAAAGCGCAAATTA   4374
m141108_iid=1811866_0      AACTAGACTCAAAAAATACATAAAATAAAATAAAGTTTAAAGAACTAAAAGCGCAAATTA   6258
m150501_iid=2948495_0      AACTAGACTCAAAA-ATACATAAAATAAAATAAAGTTTAAAGAACTAAAAGCGCAAATTA   5673
m150504_iid=3238195_0      AACTAGACTCAAAAAATACATAAAATAAAATAAAGTTTAAAGAACTAAAAGCGCAAATTA   8014
m150503_iid=3388511_0      AACTAGACTCAAAAAATACATAAAATAAAATAAAGTTTAAAGAACTAAAAGCGCAAATT-   5708
m150505_iid=3777611_0      AACTAGACTC-AAAAATACATAAAATAAAATAAAGTTTAAAGAACTAAAAGCGCAAATTA   7132
m150430_iid=2438833_0      AACTAGACTCAAAAAATACATAAAATAAAATAAAGTTTAAAGAACTAAAAGCGCAAATTA   7469
m141108_iid=1698969_0      AACTAGACTCAAAAAATACATAAAATAAAATAAAGTTTAAAGAACTAAAAGCGCAAATTA   8600
m150430_iid=3059190_0      AACTAGACTCAAAAAATACATAAAATAAAATAAAGTTTAAAGAACTAAAAGCGCAAATTA   4321
m141107_iid=1383067_0      AACTAGACTCAAAAAATACATAAAATAAAATAAAGTTTAAAGAACTAAAAGCGCAAATTA   4323
m141101_iid=597008_0       AACTAGACTCAAAAAATACATAAAATAAAATAAAGTTTAAAGAACTAAAAGCGCAAATTA   1391
m150506_iid=2596496_0      AACTAGACTCAAAAAATACATAAAATAAAATAAAGTTTAAAGAACTAAAAGCGCAAATTA   8947
m141102_iid=910051_0       ------------------------------------------------------------   7428
m150505_iid=3784017_0      AACTAGACTCAAAAAATACATAAAATAAAATAAAGTTTAAAGAACTAAAAGCGCAAATTA   7247
m141101_iid=682151_0       AACTAGACTCAAAAAATACATAAAATAAAATAAAGTTTAAAGAACTAAAAGCGCAAATTA   6315
m150501_iid=3748029_0      AACTAGACTCAAAAAATACATAAAATAAAATAAAGTTTAAAGAACTAAAAGCGCAAATTA   6858
m150504_iid=2876272_0      AACTAGACTCAAAAAATACATAAAATAAAATAAAGTTTAAAGAACTAAAAGCGCAAATTA   8715
NC_042820.1                AACTAGACTCAAAAAATACATAAAATAAAATAAAGTTTAAAGAACTAAAAGCGCAAATTA   8966
m141102_iid=1228825_0      AACTAGACTCAAAAAATACATAAAATAAAATAAAGTTTAAAGAACTAAAAGCGCAAATTA   8790
m150504_iid=2876274_0      AACTAGACTCAAAAAATACATAAAATAAAATAAAGTTTAAAGAACTAAAAGCGCAAATTA   8788
m141030_iid=80036_0        AACTAGACTCAAAAAATACATAAAATAAAATAAAGTTTAAAGAACTAAAAGCGCAAATTA   8556
m150506_iid=2596494_0      AACTAGACTCAAAAAATACATAAAATAAAATAAAGTTTAAAGAACTAAAAGCGCAAATTA   8955
m150503_iid=2526862_0      AACTAGACTCAAAAAATACATAAAATAAAATAAAGTTTAAAGAACTAAAAGCGCAAATTA   8643
m150506_iid=2567000_0      AACTAGACTCAAAAAATACATAAAATAAAATAAAGTTTAAAGAACTAAAAGCGCAAATTA   6594
m150507_iid=3073271_0      AACTAGACTCAAAAAATACATAAAATAAAATAAAGTTTAAAGAACTAAAAGCGCAAATTA   7896
m150504_iid=3156087_0      AACTAGACTCAAAAAATACATAAAATAAAATAAAGTTTAAAGAACTAAAAGCGCAAATTA   7720
m150505_iid=2498502_0      AACTAGACTCAAAAAATACATAAAATAAAATAAAGTTTAAAGAACTAAAAGCGCAAATTA   5371
```

```
m141102_iid=754227_0      AACTAGACTCAAAAAATACATAAAATAAAATAAAGTTTAAAGAACTAAAAGCGCAAATTA   7932
m150507_iid=3469514_0     AACTAGACTCAAAAAATACATAAAATAAAATAAAGTTTAAAGAACTAAAAGCGCAAATTA   8490
Rdo_MitoGenome            AACTAGACTCAAAAAATACATAAAATAAAATAAAGTTTAAAGAACTAAAAGCGCAAATTA   8966
m141101_iid=614933_0      AACTAGACTCAAAAAATACATAAAATAAAATAAAGTTTAAAGAACTAAAAGCGCAAATTA   7515
```

nad4 →

```
m141031_iid=347546_0      AAAAAAACAAAAGTATAATAACATTTAAAGAAAA-ATAAAAATGAAAATTATTGTCCATA   3725
m150506_iid=3127414_0     AAAAAAACAAAAGTATAATAACATTTAAAGAAAAAATAAAAATGAAAATTATTGTCCATA   5465
m141102_iid=1245517_0     AAAAAAACAAAAGTATAATAACATTTAAAGAAAA-ATAAAAATGGAAATTATTGTCCATT   7102
m141107_iid=1405727_0     AAAAAAACAAAAGTATAATAACATTTAAAGAAAA-ATAAAAATGAAAATTATTGTCCATA   4299
m141102_iid=987614_0      AAAAAAACAAAAGTATAATAACATTTAAAGAAAA-ATAAAAATGAAAATTATTGTCCATA   7283
m141108_iid=1701429_0     AAAAA---AAAAGTATAATAACATTTAAAGAAAA-ATAAAAATGAAAATTATTGTCCATA   4031
m141102_iid=987286_0      AAAAAAACAAAAGTATAATAACATTTAAAGAAAA-ATAAAAATGAAAATTATTGTCCATA   5249
m150430_iid=3272125_0     AAAAAAACAAAAGTATAATAACATTTAAAGAAAA-ATAAAAATGAAAATTATTGTCCATA   6763
m141102_iid=754225_0      AAAAAAACAAAAGTATAATAACATTTAAAGAAAA-ATAAAAATGAAAATTATTGTCCATA   7937
m141107_iid=1409934_0     AAAAA-AAAAAAGTATAATAACATTTAAAGAAAA-ATAAAAATGAAAATTATTGTCCATA   8477
m150503_iid=3416920_0     AAAAAAAACAAAGTATAATAACATTTAAAGAAAA-ATAAAAATGAAAATTATTGTCCATA   7191
m141108_iid=1800121_0     AAAAAAACAAAAGTTATAAATAATTTAAAGAAAA--ATAAAATGAAAATTATTGTCCATA   7006
m141031_iid=147540_0      AAAAAAACAAAAGTATAATAACATTTAAAGAAAA-ATAAAAATGAAAATTATTGTCCATA   7357
m150507_iid=3674924_0     AAAAAAACAAAAGTATAATAACATTTAAAGAAAA-ATAAAAATGAAAATTATTGTCCATA   7506
m150503_iid=2923751_0     AA-AAAACAAAAGTATAATAACATTTAAAGAAAA-ATAAAAATGAAAATTATTGTCCATA   7332
m141031_iid=115118_0      AA-AAAACAAAAGTATAATAACATTTAAAGAAAA-ATAAAAATGAAAATTATTGTCCATA   6200
m150430_iid=2722448_0     AAAAAAACAAAAGTATAATAACATTTAAAGAAAA-ATAAAAATGAAAATTATTGTCCATA   6132
m150507_iid=3102647_0     AAAAA--AAAAAGTATAATAACATTTAAAGAAAA-ATAAAAATGAAAATTATTGTCCATA   4431
m141108_iid=1811866_0     AAAAAAACAAAAGTATAATAACATTTAAAGAAAA-ATAAAAATGAAAATTATTGTCCATA   6317
m150501_iid=2948495_0     AAAAAAACAAAAGTATAATAACATTTAAAGAAAA-ATAAAAATGAAAATTATTGTCCATA   5732
m150504_iid=3238195_0     AAAAAAACAAAAGTATAATAACATTTAAAAA-AA-ATAAAAATGAAAATTATTGTCCATA   8072
m150503_iid=3388511_0     AAAAAAACAAAAGTATAATAACATTTAAAGAAAA-ATAAAAATGAAAATTATTGTCCATA   5767
m150505_iid=3777611_0     AAAAAAACAAAAGTATAATAACATTTAAAGAAAA-ATAAAAATGAAAATTATTGTCCATA   7191
m150430_iid=2438833_0     AAAAAAACAAAAGTATAATAACATTTAAAGAAAA-ATAAAAATGAAAATTATTGTCCATA   7528
m141108_iid=1698969_0     AAAAAAACAAAAGTATAATAACATTTAAAGAAAA-ATAAAAATGAAAATTATTGTCCATA   8659
m150430_iid=3059190_0     AAAAAAACAAAAGTATAATAACATTTAAAGAAAA-ATAAAAATGAAAATTATTGTCCATA   4380
m141107_iid=1383067_0     AAAAAAACAAAAGTATAATAACATTTAAAGAAAA-ATAAAAATGAAAATTATTGTCCATA   4382
m141101_iid=597008_0      AAAAAAACAAAAGTATAATAACATTTAAAGAAAA-ATAAAAATGAAAATTATTGTCCATA   1450
m150506_iid=2596496_0     AAAAAAACAAAAGTATAATAACATTTAAAGAAAA-ATAAAAATGAAAATTATTGTCCATA   9006
m141102_iid=910051_0      ------------------------------------------------------------   7428
m150505_iid=3784017_0     AAAAAAACAAAAGTATAATAACATTTAAAGAAAA-ATAAAAATGAAAATTATTGTCCATA   7306
m141101_iid=682151_0      AAAAAAACAAAAGTATAATAACATTTAAAGAAAA-ATAAAAATGAAAATTATTGTCCATA   6374
m150501_iid=3748029_0     AAAAAAACAAAAGTATAATAACATTTAAAGAAAA-ATAAAAATGAAAATTATTGTCCATA   6917
m150504_iid=2876272_0     AAAAAAACAAAAGTATAATAACATTTAAAGAAAA-ATAAAAATGAAAATTATTGTCCATA   8774
NC_042820.1               AAAAAAACAAAAGTATAATAACATTTAAAGAAAA-ATAAAAATGAAAATTATTGTCCATA   9025
m141102_iid=1228825_0     AAAAAAACAAAAGTATAATAACATTTAAAGAAAA-ATAAAAATGAAAATTATTGTCCATA   8849
m150504_iid=2876274_0     AAAAAAACAAAAGTATAATAACATTTAAAGAAAA-ATAAAAATGAAAATTATTGTCCATA   8847
m141030_iid=80036_0       AAAAAAACAAAAGTATAATAACATTTAAAGAAAA-ATAAAAATGAAAATTATTGTCCATA   8615
m150506_iid=2596494_0     AAAAAAACAAAAGTATAATAACATTTAAAGAAAA-ATAAAAATGAAAATTATTGTCCATA   9014
m150503_iid=2526862_0     AAAAAAACAAAAGTATAATAACATTTAAAGAAAA-ATAAAAATGAAAATTATTGTCCATA   8702
m150506_iid=2567000_0     AAAAAAACAAAAGTATAATAACATTTAAAGAAAA-ATAAAAATGAAAATTATTGTCCATA   6653
m150507_iid=3073271_0     AAAAAAACAAAAGTATAATAACATTTAAAGAAAA-ATAAAAATGAAAATTATTGTCCATA   7955
m150504_iid=3156087_0     AAAAAAACAAAAGTATAATAACATTTAAAGAAAA-ATAAAAATGAAAATTATTGTCCATA   7779
m150505_iid=2498502_0     AAAAAAACAAAAGTATAATAACATTTAAAGAAAA-ATAAAAATGAAAATTATTGTCCATA   5430
m141102_iid=754227_0      AAAAAAACAAAAGTATAATAACATTTAAAGAAAA-ATAAAAATGAAAATTATTGTCCATA   7991
m150507_iid=3469514_0     AAAAAAACAAAAGTATAATAACATTTAAAGAAAA-ATAAAAATGAAAATTATTGTCCATA   8549
Rdo_MitoGenome            AAAAAAACAAAAGTATAATAACATTTAAAGAAAA-ATAAAAATGAAAATTATTGTCCATA   9025
m141101_iid=614933_0      AAAAAAACAAAAGTATAATAACATTTAAAGAAAA-ATAAAAATGAAAATTATTGTCCATA   7574
```

nad4 →

```
m141031_iid=347546_0      TAAA-TTTTAGACCTAGCAATAAATATCAAAAACAAATAAAAAATCTTAATAACAAAATA   3784
m150506_iid=3127414_0     TAAA-TTTTAGACCTAGCAATAAATATCAACAAACAAATAAAAAT---CTTAACAAAATA   5521
m141102_iid=1245517_0     ATAAATTTTAGACCTAGCAATAAATT-CAACA-ACAAATAAAAAAT--CTTAACAA-AAT   7157
m141107_iid=1405727_0     TAAATTTTTAGACCTAGCAATAAATATCAACAA-CAAATAAAAAAT--CTTAACAAAATA   4356
m141102_iid=987614_0      TAAA-TTTTAGACCTAGCAATAAATATCAACAAACAAATAAAAAAT--CTTAACAAAATA   7340
m141108_iid=1701429_0     TAAA-TTTTAGACCTAGCAATAAATATCAACAAACAAATAAAAAAT--CTTAACAAAATA   4088
m141102_iid=987286_0      TAAA-TTTTAGACCTAGCAATAAATATCAACAAACAAATAAAAAAT--CTTAACAAAATA   5306
m150430_iid=3272125_0     TAAA-TTTTAGACCTAGCAATAAATATCAACAAACAAATAA-AAAT--CTTAACAAAATA   6819
m141102_iid=754225_0      TAAA-TTTTAGACCTAGCAATAAATATCAACAAACAAATAAAAAAT--CTTAACAAAATA   7994
m141107_iid=1409934_0     TAAA-TTTTAGACCTAGCAATAAATATCAACAAACAAATAAAAAAT--CTTAACAAAATA   8534
m150503_iid=3416920_0     TAAA-TTTTAGACCTAGCAATAAATATCAACAAACAAAATAAAAAT--CTTAACAAAATA   7248
m141108_iid=1800121_0     TAAA-TTTTAGACCTAGCAATAAATATCAACAAACAAATAAAAAAT--CTTAACAAAATA   7063
m141031_iid=147540_0      TAAA-TTTTAGACCTAGCAATAAATATCAACAAACAAATAAAAAAT--CTTAACAAAATA   7414
m150507_iid=3674924_0     TAAA-TTTTAGACCTAGCAATAAATATCAACAAACAAATAAAAAAT--CTTAACAAAATA   7563
m150503_iid=2923751_0     TAAA-TTTTAGACCTAGCAATAAATATCAACAAACAAATAAAAAAT--CTTAACAAAATA   7389
```

```
m141031_iid=115118_0        TAAA-TTTTAGACCTAGCAATAAATATCAACAAACAAATAAAAAAT--CTTAACAAAATA   6257
m150430_iid=2722448_0       TAAA-TTTTAGACCTAGCAATAAATATCAACAAACAAATAA-AAAT--CTTAACAAAATA   6188
m150507_iid=3102647_0       TAAA-TTTTAGACCTAGCAATAAATATCAACAAACAAATAAAAAAT--CTTAACAAAATA   4488
m141108_iid=1811866_0       TAAA-TTTTAGACCTAGCAATAAATATCAACAAACAAATAAAAAAT--CTTAACAAAATA   6374
m150501_iid=2948495_0       TAAA-TTTTAGACCTAGCAATAAATATCAACAAACAAATAA-AAAT--CTTAACAAAATA   5788
m150504_iid=3238195_0       TAAA-TTTTAGACCTAGCAATAAATATCAACAAACAAATAAAAAAT--CTTAACAAAATA   8129
m150503_iid=3388511_0       TAAA-TTTTAGACCTAGCAATAAATATCAACAAACAAATAAAAAAT--CTTAACAAAATA   5824
m150505_iid=3777611_0       TAAA-TTTTAGACCTAGCAATAAATATCAACAAACAAATAAAAAAT--CTTAACAAAATA   7248
m150430_iid=2438833_0       TAAA-TTTTAGACCTAGCAATAAATATCAACAAACAAATAAAAAAT--CTTAACAAAATA   7585
m141108_iid=1698969_0       TAAA-TTTTAGACCTAGCAATAAATATCAACAAACAAATAAAAAAT--CTTAACAAAATA   8716
m150430_iid=3059190_0       TAAA-TTTTAGACCTAGCAATAAATATCAACAAACAAATAAAAAAT--CTTAACAAAATA   4437
m141107_iid=1383067_0       TAAA-TTTTAGACCTAGCAATAAATATCAACAAACAAATAAAAAAT--CTTAACAAAATA   4439
m141101_iid=597008_0        TAAA-TTTTAGACCTAGCAATAAATATCAACAAACAAATAAAAAAT--CTTAACAAAATA   1507
m150506_iid=2596496_0       TAAA-TTTTAGACCTAGCAATAAATATCAACAAACAAATAAAAAAT--CTTAACAAAATA   9063
m141102_iid=910051_0        ------------------------------------------------------------   7428
m150505_iid=3784017_0       TAAA-TTTTAGACCTAGCAATAAATATCAACAAACAAATAAAAAAT--CTTAACAAAATA   7363
m141101_iid=682151_0        TAAA-TTTTAGACCTAGCAATAAATATCAACAAACAAATAAAAAAT--CTTAACAAAATA   6431
m150501_iid=3748029_0       TAAA-TTTTAGACCTAGCAATAAATATCAACAAACAAATAAAAAAT--CTTAACAAAATA   6974
m150504_iid=2876272_0       TAAA-TTTTAGACCTAGCAATAAATATCAACAAACAAATAAAAAAT--CTTAACAAAATA   8831
NC_042820.1                 TAAA-TTTTAGACCTAGCAATAAATATCAACAAACAAATAAAAAAT--CTTAACAAAATA   9082
m141102_iid=1228825_0       TAAA-TTTTAGACCTAGCAATAAATATCAACAAACAAATAAAAAAT--CTTAACAAAATA   8906
m150504_iid=2876274_0       TAAA-TTTTAGACCTAGCAATAAATATCAACAAACAAATAAAAAAT--CTTAACAAAATA   8904
m141030_iid=80036_0         TAAA-TTTTAGACCTAGCAATAAATATCAACAAACAAATAAAAAAT--CTTAACAAAATA   8672
m150506_iid=2596494_0       TAAA-TTTTAGACCTAGCAATAAATATCAACAAACAAATAAAAAAT--CTTAACAAAATA   9071
m150503_iid=2526862_0       TAAA-TTTTAGACCTAGCAATAAATATCAACAAACAAATAAAAAAT--CTTAACAAAATA   8759
m150506_iid=2567000_0       TAAA-TTTTAGACCTAGCAATAAATATCAACAAACAAATAAAAAAT--CTTAACAAAATA   6710
m150507_iid=3073271_0       TAAA-TTTTAGACCTAGCAATAAATATCAACAAACAAATAAAAAAT--CTTAACAAAATA   8012
m150504_iid=3156087_0       TAAA-TTTTAGACCTAGCAATAAATATCAACAAACAAATAAAAAAT--CTTAACAAAATA   7836
m150505_iid=2498502_0       TAAA-TTTTAGACCTAGCAATAAATATCAACAAACAAATAAAAAAT--CTTAACAAAATA   5487
m141102_iid=754227_0        TAAA-TTTTAGACCTAGCAATAAATATCAACAAACAAATAAAAAAT--CTTAACAAAATA   8048
m150507_iid=3469514_0       TAAA-TTTTAGACCTAGCAATAAATATCAACAAACAAATAAAAAAT--CTTAACAAAATA   8606
Rdo_MitoGenome              TAAA-TTTTAGACCTAGCAATAAATATCAACAAACAAATAAAAAAT--CTTAACAAAATA   9082
m141101_iid=614933_0        TAAA-TTTTAGACCTAGCAATAAATATCAACAAACAAATAAAAAAT--CTTAACAAAATA   7631
```

nad4

```
m141031_iid=347546_0        AGAGGGCCAGAAA--AAATCACAACCGAAGCCTAAACCAAGAAGAATTGT-AGAGACAGG   3841
m150506_iid=3127414_0       AGAGGGCCAGAAAA-AAATCACAACCGAAGCCTAAACCAAGAAGAATTGT-AGAGACAGG   5579
m141102_iid=1245517_0       AGAGGGCCAGAAAACAAATCACAACCGAAGCCTAAACCAAGAAGAATTGTTAGAGACAGG   7217
m141107_iid=1405727_0       AGAGGGCCAGAAAACAAATCACAACCGAAGCCTAAACCAAGAAGAATTGT-AGAGACAGG   4415
m141102_iid=987614_0        AGAGGGCCAGAAAACAAATCACAACCGAAGCCTAAACCAAGAAGAATTGT-AGAGACAGG   7399
m141108_iid=1701429_0       AGAGGGCCAGAAAACAAATCACAACCGAAGCCTAAACCAAGAAGAATTGT-AGAGACAGG   4147
m141102_iid=987286_0        AGAGGGCCAGAAAACAAATCACAACCGAAGCCTAAACCAAGAAGAATTGT-AGAGACAGG   5365
m150430_iid=3272125_0       AGAGGGCCAGAAAACAAATCACAACCGAAGCCTAAACCAAGAAGAATTGT-AGAGACAGG   6878
m141102_iid=754225_0        AGAGGGCCAGAAAACAAATCACAACCGAAGCCTAAACCAAGAAGAATTGT-AGAGACAGG   8053
m141107_iid=1409934_0       AGAGGGCCAGAAAACAAATCACAACCGAAGCCTAAACCAAGAAGAATTGT-AGAGACAGG   8593
m150503_iid=3416920_0       AGAGGGCCAGAAAACAAATCACAACCGAAGCCTAAACCAAGAAGAATTGT-AGAGACAGG   7307
m141108_iid=1800121_0       AGAGGGCCAGAAAACAAATCACAACCGAAGCCTAAACCAAGAAGAATTGT-AGAGACAGG   7122
m141031_iid=147540_0        AGAGGGCCAGAAAACAAATCACAACCGAAGCCTAAACCAAGAAGAATTGT-AGAGACAGG   7473
m150507_iid=3674924_0       AGAGGGCCAGAAAACAAATCACAACCGAAGCCTAAACCAAGAAGAATTGT-AGAGACAGG   7622
m150503_iid=2923751_0       AGAGGGCCAGAAAACAAATCACAACCGAAGCCTAAACCAAGAAGAATTGT-AGAGACAGG   7448
m141031_iid=115118_0        AGAGGGCCAGAAAACAAATCACAACCGAAGCCTAAACCAAGAAGAATTGT-AGAGACAGG   6316
m150430_iid=2722448_0       AGAGGGCCAGAAAACAAATCACAACCGAAGCCTAAACCAAGAAGAATTGT-AGAGACAGG   6247
m150507_iid=3102647_0       AGAGGGCCAGAAAACAAATCACAACCGAAGCCTAAACCAAGAAGAATTGT-AGAGACAGG   4547
m141108_iid=1811866_0       AGAGGGCCAGAAAACAAATCACAACCGAAGCCTAAACCAAGAAGAATTGT-AGAGACAGG   6433
m150501_iid=2948495_0       AGAGGGCCAGAAAACAAATCACAACCGAAGCCTAAACCAAGAAGAATTGT-AGAGACAGG   5847
m150504_iid=3238195_0       AGAGGGCCAGAAAACAAATCACAACCGAAGCCTAAACCAAGAAGAATTGT-AGAGACAGG   8188
m150503_iid=3388511_0       AGAGGGCCAGAAAACAAATCACAACCGAAGCCTAAACCAAGAAGAATTGT-AGAGACAGG   5883
m150505_iid=3777611_0       AGAGGGCCAGAAAACAAATCACAACCGAAGCCTAAACCAAGAAGAATTGT-AGAGACAGG   7307
m150430_iid=2438833_0       AGAGGGCCAGAAAACAAATCACAACCGAAGCCTAAACCAAGAAGAATTGT-AGAGACAGG   7644
m141108_iid=1698969_0       AGAGGGCCAGAAAACAAATCACAACCGAAGCCTAAACCAAGAAGAATTGT-AGAGACAGG   8775
m150430_iid=3059190_0       AGAGGGCCAGAAAACAAATCACAACCGAAGCCTAAACCAAGAAGAATTGT-AGAGACAGG   4496
m141107_iid=1383067_0       AGAGGGCCAGAAAACAAATCACAACCGAAGCCTAAACCAAGAAGAATTGT-AGAGACAGG   4498
m141101_iid=597008_0        AGAGGGCCAGAAAACAAATCACAACCGAAGCCTAAACCAAGAAGAATTGT-AGAGACAGG   1566
m150506_iid=2596496_0       AGAGGGCCAGAAAACAAATCACAACCGAAGCCTAAACCAAGAAGAATTGT-AGAGACAGG   9122
m141102_iid=910051_0        ------------------------------------------------------------   7428
m150505_iid=3784017_0       AGAGGGCCAGAAAACAAATCACAACCGAAGCCTAAACCAAGAAGAATTGT-AGAGACAGG   7422
m141101_iid=682151_0        AGAGGGCCAGAAAACAAATCACAACCGAAGCCTAAACCAAGAAGAATTGT-AGAGACAGG   6490
m150501_iid=3748029_0       AGAGGGCCAGAAAACAAATCACAACCGAAGCCTAAACCAAGAAGAATTGT-AGAGACAGG   7033
m150504_iid=2876272_0       AGAGGGCCAGAAAACAAATCACAACCGAAGCCTAAACCAAGAAGAATTGT-AGAGACAGG   8890
NC_042820.1                 AGAGGGCCAGAAAACAAATCACAACCGAAGCCTAAACCAAGAAGAATTGT-AGAGACAGG   9141
m141102_iid=1228825_0       AGAGGGCCAGAAAACAAATCACAACCGAAGCCTAAACCAAGAAGAATTGT-AGAGACAGG   8965
```

```
m150504_iid=2876274_0     AGAGGGCCAGAAAACAAATCACAACCGAAGCCTAAACCAAGAAGAATTGT-AGAGACAGG   8963
m141030_iid=80036_0       AGAGGGCCAGAAAACAAATCACAACCGAAGCCTAAACCAAGAAGAATTGT-AGAGACAGG   8731
m150506_iid=2596494_0     AGAGGGCCAGAAAACAAATCACAACCGAAGCCTAAACCAAGAAGAATTGT-AGAGACAGG   9130
m150503_iid=2526862_0     AGAGGGCCAGAAAACAAATCACAACCGAAGCCTAAACCAAGAAGAATTGT-AGAGACAGG   8818
m150506_iid=2567000_0     AGAGGGCCAGAAAACAAATCACAACCGAAGCCTAAACCAAGAAGAATTGT-AGAGACAGG   6769
m150507_iid=3073271_0     AGAGGGCCAGAAAACAAATCACAACCGAAGCCTAAACCAAGAAGAATTGT-AGAGACAGG   8071
m150504_iid=3156087_0     AGAGGGCCAGAAAACAAATCACAACCGAAGCCTAAACCAAGAAGAATTGT-AGAGACAGG   7895
m150505_iid=2498502_0     AGAGGGCCAGAAAACAAATCACAACCGAAGCCTAAACCAAGAAGAATTGT-AGAGACAGG   5546
m141102_iid=754227_0      AGAGGGCCAGAAAACAAATCACAACCGAAGCCTAAACCAAGAAGAATTGT-AGAGACAGG   8107
m150507_iid=3469514_0     AGAGGGCCAGAAAACAAATCACAACCGAAGCCTAAACCAAGAAGAATTGT-AGAGACAGG   8665
Rdo_MitoGenome            AGAGGGCCAGAAAACAAATCACAACCGAAGCCTAAACCAAGAAGAATTGT-AGAGACAGG   9141
m141101_iid=614933_0      AGAGGGCCAGAAAACAAATCACAACCGAAGCCTAAACCAAGAAGAATTGT-AGAGACAGG   7690

                                                 nad4

m141031_iid=347546_0      AGCAATTTTCAAAAAAA-TAAATATTATTAAACCCAAAAATAAAAATACTAAATAAAAAT   3900
m150506_iid=3127414_0     AGCAATTTTCAAAAAAA-TAAATATTATTAAACCCAAAAATAAAAATACTAAATAAAAAT   5638
m141102_iid=1245517_0     AGCAATTTTCAAAAAAAATAA--TATTATTAAACCCAAAATAAATTA---ATATAATAAAT  7272
m141107_iid=1405727_0     AGCAATTTTCAAAAAAATAAAATATTATTAAACCCAAAAATAAAAATACTAAATAAAAAT   4475
m141102_iid=987614_0      AGCAATTTTCAAAAAAA-TAAATATTATTAAACCCAAAAATAAAAATACTAAATAAAAAT   7458
m141108_iid=1701429_0     AGCAATTTTCAAAAAAAT-AAATATTATTAAACCCAAAAATAAAAATACTAAATAAAAAT   4206
m141102_iid=987286_0      AGCAATTTTCAAAAAAAATA-AATATTATTAAACCCAAAAATAAAAATACTAAATAAAAAT  5424
m150430_iid=3272125_0     AGCAATTTTCAAAAAA-TA-AATATTATTAAACCCAAAAATAAAAATACTAAATAAAAAT   6936
m141102_iid=754225_0      AGCAATTTTCAAAAAAAATAA-ATATTATT-AAACCAAAAATAAAAATACTAAATAAAAAT  8111
m141107_iid=1409934_0     AGCAATTTTCAAAAAA--TAAATATTATTAAACCCAAAAATAAAAATACTAAATAAAAAT   8651
m150503_iid=3416920_0     AGCAATTTTCAAAAAAA-TAAATATTATTAAACCCAAAAATAAAAATACTAAATAAAAAT   7366
m141108_iid=1800121_0     AGCAATTTTCAAAAAAAATAAATATTATTAAACCCAAAAATAAAAATACTAAAT-AAAAT   7181
m141031_iid=147540_0      AGCAATTTTCAAAAAAA-TAAATATTATTAAACCCAAAAATAAAAATACTAAATAAAAAT   7532
m150507_iid=3674924_0     AGCAATTTTCAAAAAA--TAAATATTATTAAACCCAAAAATAAAAATACTAAATAAAAAT   7680
m150503_iid=2923751_0     AGCAATTTTCAAAAAA--TAAATATTATTAAACCCAAAAATAAAAATACTAAATAAAAAT   7506
m141031_iid=115118_0      AGCAATTTTCAAAAAAA-TAAATATTATTAAACCCAAAAATAAAAATACTAAATAAAAAT   6375
m150430_iid=2722448_0     AGCAATTTTCAAAAAAA-TAAATATTATTAAACCCAAAAAAA--AATACTAAATAAAAAT   6304
m150507_iid=3102647_0     AGCAATTTTCAAAAAAA-TAAATATTATTAAACCCAAAAATAAAAATACTAAATAAAAAT   4606
m141108_iid=1811866_0     AGCAATTTTCAAAAAAA-TAAATATTATTAAACCCAAAAATAAAAATACTAAATAAAAAT   6492
m150501_iid=2948495_0     AGCAATTTTCAAAAAAA-TAAATATTATTAAACCCAAAAATAAAAATACTAAATAAAAAT   5906
m150504_iid=3238195_0     AGCAATTTTCAAAAAAA-TAAATATTATTAAACCCAAAAATAAAAATACTAAATAAAAAT   8247
m150503_iid=3388511_0     AGCAATTTTCAAAAAAA-TAAATATTATTAAACCCAAAAATAAAAATACTAAATAAAAAT   5942
m150505_iid=3777611_0     AGCAATTTTCAAAAAAA-TAAATATTATTAAACCCAAAAATAAAAATACTAAATAAAAAT   7366
m150430_iid=2438833_0     AGCAATTTTCAAAAAAA-TAAATATTATTAAACCCAAAAATAAAAATACTAAATAAAAAT   7703
m141108_iid=1698969_0     AGCAATTTTCAAAAAAA-TAAATATTATTAAACCCAAAAATAAAAATACTAAATAAAAAT   8834
m150430_iid=3059190_0     AGCAATTTTCAAAAAAA-TAAATATTATTAAACCCAAAAATAAAAATACTAAATAAAAAT   4555
m141107_iid=1383067_0     AGCAATTTTCAAAAAAA-TAAATATTATTAAACCCAAAAATA--AAAACTAAATAAAAAT   4555
m141101_iid=597008_0      AGCAATTTTCAAAAAAA-TAAATATTATTAAACCCAAAAATAAAAATACTAAATAAAAAT   1625
m150506_iid=2596496_0     AGCAATTTTCAAAAAAA-TAAATATTATTAAACCCAAAAATAAAAATACTAAATAAAAAT   9181
m141102_iid=910051_0      ------------------------------------------------------------   7428
m150505_iid=3784017_0     AGCAATTTTCAAAAAAA-TAAATATTATTAAACCCAAAAATAAAAATACTAAATAAAAAT   7481
m141101_iid=682151_0      AGCAATTTTCAAAAAAA-TAAATATTATTAAACCCAAAAATAAAAATACTAAATAAAAAT   6549
m150501_iid=3748029_0     AGCAATTTTCAAAAAAA-TAAATATTATTAAACCCAAAAATAAAAATACTAAATAAAAAT   7092
m150504_iid=2876272_0     AGCAATTTTCAAAAAAA-TAAATATTATTAAACCCAAAAATAAAAATACTAAATAAAAAT   8949
NC_042820.1               AGCAATTTTCAAAAAAA-TAAATATTATTAAACCCAAAAATAAAAATACTAAATAAAAAT   9200
m141102_iid=1228825_0     AGCAATTTTCAAAAAAA-TAAATATTATTAAACCCAAAAATAAAAATACTAAATAAAAAT   9024
m150504_iid=2876274_0     AGCAATTTTCAAAAAAA-TAAATATTATTAAACCCAAAAATAAAAATACTAAATAAAAAT   9022
m141030_iid=80036_0       AGCAATTTTCAAAAAAA-TAAATATTATTAAACCCAAAAATAAAAATACTAAATAAAAAT   8790
m150506_iid=2596494_0     AGCAATTTTCAAAAAAA-TAAATATTATTAAACCCAAAAATAAAAATACTAAATAAAAAT   9189
m150503_iid=2526862_0     AGCAATTTTCAAAAAAA-TAAATATTATTAAACCCAAAAATAAAAATACTAAATAAAAAT   8877
m150506_iid=2567000_0     AGCAATTTTCAAAAAAA-TAAATATTATTAAACCCAAAAATAAAAATACTAAATAAAAAT   6828
m150507_iid=3073271_0     AGCAATTTTCAAAAAAA-TAAATATTATTAAACCCAAAAATAAAAATACTAAATAAAAAT   8130
m150504_iid=3156087_0     AGCAATTTTCAAAAAAA-TAAATATTATTAAACCCAAAAATAAAAATACTAAATAAAAAT   7954
m150505_iid=2498502_0     AGCAATTTTCAAAAAAA-A-AATATTATTAAACCCAAAAATAAAAATACTAAATAAAAAT   5604
m141102_iid=754227_0      AGCAATTTTCAAAAAAA-TAAATATTATTAAACCCAAAAATAAAAATACTAAATAAAAAT   8166
m150507_iid=3469514_0     AGCAATTTTCAAAAAAA-TAAATATTATTAAACCCAAAAATAAAAATACTAAATAAAAAT   8724
Rdo_MitoGenome            AGCAATTTTCAAAAAAA-TAAATATTATTAAACCCAAAAATAAAAATACTAAATAAAAAT   9200
m141101_iid=614933_0      AGCAATTTTCAAAAAAA-TAAATATTATTAAACCCAAAAATAAAAATACTAAATAAAAAT   7749

                                                 nad4

m141031_iid=347546_0      CTAAAAAACTAAGAGGGATCATAAAA-AATACAAACAAAATAAT-TTTATCATAAAACAT   3958
m150506_iid=3127414_0     CTAAAAAACTAAGAGGGATCATAAAA-AATACAAACAAAATAAATTTTATCATAAAACAT   5697
m141102_iid=1245517_0     CTAAAAAACTAAGAGGGATCATA-------------------------------------   7295
m141107_iid=1405727_0     CTA-AAAACTAAGAGGGATCATAAAAAAATACAAACAA--AAAAATTTTATCATAAAACAT  4532
m141102_iid=987614_0      CTAAAAAACTAAGAGGGATCATAAAAAAATACAAACAAAATATTTTATTATCATAAAACAT  7518
m141108_iid=1701429_0     CTAAAAAACTAAGAGGGATCATAAAA-AATACAAACAAAATAAATTTTATCATAAAACAT   4265
m141102_iid=987286_0      CTAAAAAACTAAGAGGGATCATAAAA-AATACAAACAAAATAAATTTTATCATAAAACAT   5483
```

```
m150430_iid=3272125_0      CTAAAAAACTAAGAGGGATCATAAAAAAATACAAACAAAATAAATTTTATCATAAAACAT   6996
m141102_iid=754225_0       CTAAAAAACTAAGAGGGATCATAAAA-AATACAAACAAAATAAATTTTATCATAAAACAT   8170
m141107_iid=1409934_0      CTAAAAAACTAAGAGGGATCATAAAA-AATACAAACAAAATAAATTTTATCATAAAACAT   8710
m150503_iid=3416920_0      CTAAAAAACTAAGAGGGATCATAAAA-AATACAAACAAAATAAATTTTATCATAAAACAT   7425
m141108_iid=1800121_0      CTAAAAAACTAAGAGGGATCATAAAA-AATACAAACAAAATAAATTTTATCATAAAACAT   7240
m141031_iid=147540_0       CTAAAAAACTAAGAGGGATCATAAAA-AATACAAACAAAATAAATTTTATCATAAAACAT   7591
m150507_iid=3674924_0      CTAAAAAACTAAGAGGGATCATAAAA-AATACAAACAAAATAAATTTTATCATAAAACAT   7739
m150503_iid=2923751_0      CTAAAAAACTAAGAGGGATCATAAAA-AATACAAACAAAATAAATTTTATCATAAAACAT   7565
m141031_iid=115118_0       CTAAAAAACTAAGAGGGATCATAAAA-AATACAAACAAAATAAATTTTATCATAAAACAT   6434
m150430_iid=2722448_0      CT-AAAAACTAAGAGGGATCATAAAA-AATACAAACAAAATAAATTTTATCATAAAACAT   6362
m150507_iid=3102647_0      CTAAAAAACTAAGAGGGATCATAAA--AATACAAACAAAATAAATTTTATCATAAAACAT   4664
m141108_iid=1811866_0      CTAAAAAACTAAGAGGGATCATAAAA-AATACAAACAAAATAAATTTTATCATAAAACAT   6551
m150501_iid=2948495_0      CTAAAAAACTAAGAGGGATCATAAAA-AATACAAACAAAATAAATTTTATCATAAAACAT   5965
m150504_iid=3238195_0      CTAAAAAACTAAGAGGGATCATAAAA-AATACAAACAAAATAAATTTTATCATAAAACAT   8306
m150503_iid=3388511_0      CTAAAAAACTAAGAGGGATCATAAAA-AATACAAACAAAATAAATTTTATCATAAAACAT   6001
m150505_iid=3777611_0      CTAAAAAACTAAGAGGGATCATAAAA-AATACAAACAAAATAAATTTTATCATAAAACAT   7425
m150430_iid=2438833_0      CT-AAAAACTAAGAGGGATCATAAAA-A--ACAAACAAAATAAATTTTATCATAAAACAT   7759
m141108_iid=1698969_0      CTAAAAAACTAAGAGGGATCATAAAA-AATACAAACAAAATAAATTTTATCATAAAACAT   8893
m150430_iid=3059190_0      CTAAAAAACTAAGAGGGATCATAAAA-AATACAAACAAAATAAATTTTATCATAAAACAT   4614
m141107_iid=1383067_0      CTAAAAAACTAAGAGGGATCATAAAA-AATACAAACAAAATAAATTTTATCATAAAACAT   4614
m141101_iid=597008_0       CTAAAAAACTAAGAGGGATCATAAAA-AATACAAACAAAATAAATTTTATCATAAAACAT   1684
m150506_iid=2596496_0      CTAAAAAACTAAGAGGGATCATAAAA-AATACAAACAAAATAAATTTTATCATAAAACAT   9240
m141102_iid=910051_0       ------------------------------------------------------------   7428
m150505_iid=3784017_0      CTAAAAAACTAAGAGGGATCATAAAA-AATACAAACAAAATAAATTTTATCATAAAACAT   7540
m141101_iid=682151_0       CTAAAAAACTAAGAGGGATCATAAAA-AATACAAACAAAATAAATTTTATCATAAAACAT   6608
m150501_iid=3748029_0      CTAAAAAACTAAGAGGGATCATAAAA-AATACAAACAAAATAAATTTTATCATAAAACAT   7151
m150504_iid=2876272_0      CTAAAAAACTAAGAGGGATCATAAAA-AATACAAACAAAATAAATTTTATCATAAAACAT   9008
NC_042820.1                CTAAAAAACTAAGAGGGATCATAAAA-AATACAAACAAAATAAATTTTATCATAAAACAT   9259
m141102_iid=1228825_0      CTAAAAAACTAAGAGGGATCATAAAA-AATACAAACAAAATAAATTTTATCATAAAACAT   9083
m150504_iid=2876274_0      CTAAAAAACTAAGAGGGATCATAAAA-AATACAAACAAAATAAATTTTATCATAAAACAT   9081
m141030_iid=80036_0        CTAAAAAACTAAGAGGGATCATAAAA-AATACAAACAAAATAAATTTTATCATAAAACAT   8849
m150506_iid=2596494_0      CTAAAAAACTAAGAGGGATCATAAAA-AATACAAACAAAATAAATTTTATCATAAAACAT   9248
m150503_iid=2526862_0      CTAAAAAACTAAGAGGGATCATAAAA-AATACAAACAAAATAAATTTTATCATAAAACAT   8936
m150506_iid=2567000_0      CTAAAAAACTAAGAGGGATCATAAAA-AATACAAACAAAATAAATTTTATCATAAAACAT   6887
m150507_iid=3073271_0      CTAAAAAACTAAGAGGGATCAT-AAA-AATACAAACAAAATAAATTTTATCATAAAACAT   8188
m150504_iid=3156087_0      CTAAAAAACTAAGAGGGATCATAAAA-AATACAAACAAAATAAATTTTATCATAAAACAT   8013
m150505_iid=2498502_0      CTAAAAAACTAAGAGGGATCATAAAA-AATACAAACAAAATAAATTTTATCATAAAACAT   5663
m141102_iid=754227_0       CTAAAAAACTAAGAGGGATCATAAAA-AATACAAACAAAATAAATTTTATCATAAAACAT   8225
m150507_iid=3469514_0      CTAAAAAACTAAGAGGGATCATAAAA-AATACAAACAAAATAAATTTTATCATAAAACAT   8783
Rdo_MitoGenome             CTAAAAAACTAAGAGGGATCATAAAA-AATACAAACAAAATAAATTTTATCATAAAACAT   9259
m141101_iid=614933_0       CTAAAAAACTAAGAGGGATCATAAAA-AATACAAACAAAATAAATTTTATCATAAAACAT   7808
                                                                               nad4L
                                                nad4
m141031_iid=347546_0       TTAAAAGTCTGAA-AAAAATCATTT[illegible]CGAATAATTAAAACCAATAA-ACTAA   4016
m150506_iid=3127414_0      T-AAAAGTCTGAAAAAAAAATCATTTCCACATGAACGAATAATTAAAACCAATAAACCTAA   5756
m141102_iid=1245517_0      ------------------------------------------------------------   7295
m141107_iid=1405727_0      T-AAAAGTCTGAA-A-AAATCATTTCCACATGAACGAATAATTAAAACCAATAAACCTAA   4589
m141102_iid=987614_0       T-AAAAGTCTGAA-AAAAATCATTTCCACATGAACGAATAATTAAAACCAATAAACCTAA   7576
m141108_iid=1701429_0      T-AAAAGTCTGAA-AAAAATCATTTCCACATGAACGAATAATTAAAACCAATAAACCTAA   4323
m141102_iid=987286_0       T-AAAAGTCTGAA-AAAAATCATTTCCACATGAACGAATAATTAAAACCAATAAACCTAA   5541
m150430_iid=3272125_0      T-AAAAGTCTGAA-AAAAATCATTTCCACATGAACGAATAATTAAAACCAATAAACCTAA   7054
m141102_iid=754225_0       T-AAAAGTCTGAA-AAAAATCATTTCCACATGAACGAATAATTAAAACCAATAAACCTAA   8228
m141107_iid=1409934_0      T-AAAAGTCTGAA-AAAAATCATTTCCACATGAACGAATAATTAAAACCAATAAACCTAA   8768
m150503_iid=3416920_0      T-AAAAGTCTGAA-A-AAATCATTTCCACATGAACGAATAATTAAAACCAATAAACCTAA   7482
m141108_iid=1800121_0      T-AAAAGTCTGAA-AAAAATCATTTCCACATGAACGAATAATTAAAACCAATAAACCTAA   7298
m141031_iid=147540_0       T-AAAAGTCTGAA-AAAAATCATTTCCACATGAACGAATAATTAAAACCAATAAA---AA   7646
m150507_iid=3674924_0      T-AAAAGTCTGAA-AAAAATCATTTCCACATGAACGAATAATTAAAACCAATAAACCTAA   7797
m150503_iid=2923751_0      T-AAAAGTCTGAA-AAAAATCATTTCCACATGAACGAATAATTAAAACCAATAAACCTAA   7623
m141031_iid=115118_0       T-AAAAGTCTGAA-AAAAATCATTTCCACATGAACGAATAATTAAAACCAATAAACCTAA   6492
m150430_iid=2722448_0      T-AAAAGTCTGAA-AAAAATCATTTCCACATGAACGAATAATTAAAACCAATAAACCTAA   6420
m150507_iid=3102647_0      T-AAAAGTCTGAA-AAAAATCATTTCCACATGAACGAATAATTAAAACCAATAAACCTAA   4722
m141108_iid=1811866_0      T-AAAAGTCTGAA-AAAAATCATTTCCACATGAACGAATAATTAAAACCAATAAACCTAA   6609
m150501_iid=2948495_0      T-AAAAGTCTGAA-AAAAATCATTTCCACATGAACGAATAATTAAAACCAATAAACCTAA   6023
m150504_iid=3238195_0      T-AAAAGTCTGAA-AAAAATCATTTCCACATGAACGAATAATTAAAACCAATAAACCTAA   8364
m150503_iid=3388511_0      T-AAAAGTCTGAA-AAAAATCATTTCCACATGAACGAATAATTAAAACCAATAAACCTAA   6059
m150505_iid=3777611_0      T-AAAAGTCTGAA-AAAAATCATTTCCACATGAACGAATAATTAAAACCAATAAACCTAA   7483
m150430_iid=2438833_0      T-AAAAGTCTGAA-AAAAATCATTTCCACATGAACGAATAATTAAAACCAATAAACCTAA   7817
m141108_iid=1698969_0      T-AAAAGTCTGAA-AAAAATCATTTCCACATGAACGAATAATTAAAACCAATAAACCTAA   8951
m150430_iid=3059190_0      T-AAAAGTCTGAA-AAAAATCATTTCCACATGAACGAATAATTAAAACCAATAAACCTAA   4672
m141107_iid=1383067_0      T-AAAAGTCTGAA-AAAAATCATTTCCACATGAACGAATAATTAAAACCAATAAACCTAA   4672
m141101_iid=597008_0       T-AAAAGTCTGAA-AAAAATCATTTCCACATGAACGAATAATTAAAACCAATAAACCTAA   1742
```

```
m150506_iid=2596496_0      T-AAAAGTCTGAA-AAAAATCATTTCCACATGAACGAATAATTAAAACCAATAAACCTAA   9298
m141102_iid=910051_0       ------------------------------------------------------------   7428
m150505_iid=3784017_0      T-AAAAGTCTGAA-AAAAATCATTTCCACATGAACGAATAATTAAAACCAATAAACCTAA   7598
m141101_iid=682151_0       T-AAAAGTCTGAA-AAAAATCATTTCCACATGAACGAATAATTAAAACCAATAAACCTAA   6666
m150501_iid=3748029_0      T-AAAAGTCTGAA-AAAAATCATTTCCACATGAACGAATAATTAAAACCAATAAACCTAA   7209
m150504_iid=2876272_0      T-AAAAGTCTGAA-AAAAATCATTTCCACATGAACGAATAATTAAAACCAATAAACCTAA   9066
NC_042820.1                T-AAAAGTCTGAA-AAAAATCATTTCCACATGAACGAATAATTAAAACCAATAAACCTAA   9317
m141102_iid=1228825_0      T-AAAAGTCTGAA-AAAAATCATTTCCACATGAACGAATAATTAAAACCAATAAACCTAA   9141
m150504_iid=2876274_0      T-AAAAGTCTGAA-AAAAATCATTTCCACATGAACGAATAATTAAAACCAATAAACCTAA   9139
m141030_iid=80036_0        T-AAAAGTCTGAA-AAAAATCATTTCCACATGAACGAATAATTAAAACCAATAAACCTAA   8907
m150506_iid=2596494_0      T-AAAAGTCTGAA-AAAAATCATTTCCACATGAACGAATAATTAAAACCAATAAACCTAA   9306
m150503_iid=2526862_0      T-AAAAGTCTGAA-AAAAATCATTTCCACATGAACGAATAATTAAAACCAATAAACCTAA   8994
m150506_iid=2567000_0      T-AAAAGTCTGAA-AAAAATCATTTCCACATGAACGAATAATTAAAACCAATAAACCTAA   6945
m150507_iid=3073271_0      T-AAAAGTCTGAA-AAAAATCATTTCCACATGAACGAATAATTAAAACCAATAAACCTAA   8246
m150504_iid=3156087_0      T-AAAAGTCTGAA-AAAAATCATTTCCACATGAACGAATAATTAAAACCAATAAACCTAA   8071
m150505_iid=2498502_0      T-AAAAGTCTGAA-AAAAATCATTTCCACATGAACGAATAATTAAAACCAATAAACCTAA   5721
m141102_iid=754227_0       T-AAAAGTCTGAA-AAAAATCATTTCCACATGAACGAATAATTAAAACCAATAAACCTAA   8283
m150507_iid=3469514_0      T-AAAAGTCTGAA-AAAAATCATTTCCACATGAACGAATAATTAAAACCAATAAACCTAA   8841
Rdo_MitoGenome             T-AAAAGTCTGAA-AAAAATCATTTCCACATGAACGAATAATTAAAACCAATAAACCTAA   9317
m141101_iid=614933_0       T-AAAAGTCTGAA-AAAAATCATTTCCACATGAACGAATAATTAAAACCAATAAACCTAA   7866
```

nad4L

```
m141031_iid=347546_0       ACCCAAAGCACCTTCACAGACCGAAAAACA--TAAAAAATTATTAAAAAAAAACCTTCAT   4074
m150506_iid=3127414_0      ACCCAAAGCACCTTCACAGACCGAAAAACA--AATAAAAAATTTAAAAAAAAACCTTCAT   5814
m141102_iid=1245517_0      ------------------------------------------------------------   7295
m141107_iid=1405727_0      ACCCAAAGCACCTTCACAGACCGAAAAACATAAAAAA-ATTATTAAAAAAAAACCTTCAT   4648
m141102_iid=987614_0       ACCCAAAGCACCTTCACAGACCGAAAAACATAAAAAAAATTATTAAAAAAAAACCTTCAT   7636
m141108_iid=1701429_0      ACCCAAAGCACCTTCACAGACCGAAAAACAT-AAAAAAATTATT-AAAAAAAACCTTCAT   4381
m141102_iid=987286_0       ACCCAAAGCACCTTCACAGACCGAAAAACAT-AAAAAAATTATTAAAAAAAAACCTTCAT   5600
m150430_iid=3272125_0      ACCCAAAGCACCTTCACAGACCGAAAAACAT-AAAAAAATTATT-AAAAAAAACCTTCAT   7112
m141102_iid=754225_0       ACCCAAAGCACCTTCACAGACCGAAAAACAT-AAAAAAATTATT-AAAAAAAACCTTCAT   8286
m141107_iid=1409934_0      ACCCAAAGCACCTTCACAGACCGAAAAACAT-AAAAAAATTATTAAAAAAAAACCTTCAT   8827
m150503_iid=3416920_0      ACCCAAAGCACCTTCACAGACCGAAAAACAT-AAAAAAATTAT-TAAAAAAAACCTTCAT   7540
m141108_iid=1800121_0      ACCCAAAGCACCTTCACAGACCGAAAACATA-A-AAAAATTA--TTAAAAAAACCTTCAT   7354
m141031_iid=147540_0       ACCCAAAGCACCTTCACAGACCGAAAAACAT-AAAAAAATTATT-AAAAAAAACCTTCAT   7704
m150507_iid=3674924_0      ACCCAAAGCACCTTCACAGACCGAAAAACAT-AAAAAAATTATTAAAAAAAAACCTTCAT   7856
m150503_iid=2923751_0      ACCCAAAGCACCTTCACAGACCGAAAAACAT-AAAAAAATTATTAAAAAAAAACCTTCAT   7682
m141031_iid=115118_0       ACCCAAAGCACCTTCACAGACCGAAAAACAT-AAAAAAATTATTAAAAAAAAACCTTCAT   6551
m150430_iid=2722448_0      ACCCAAAGCACCTTCACAGACCGAAAAACAT-AAAAAAATTATTAAAAAAAAACCTTCAT   6479
m150507_iid=3102647_0      ACCCAAAGCACCTTCACAGACCGAAAAACAT-AAAAAAATTATTAAAAAAAAACCTTCAT   4781
m141108_iid=1811866_0      ACCCAAAGCACCTTCACAGACCGAAAAACAT-AAAAAAATTATT-AAAAAAAACCTTCAT   6667
m150501_iid=2948495_0      ACCCAAAGCACCTTCACAGACCGAAAAACAT-AAAAAA-TTATTAAAAAAAAACCTTCAT   6081
m150504_iid=3238195_0      ACCCAAAGCACCTTCACAGACCGAAAAACAT-AAAAAAATTATTA-AAAAAAACCTTCAT   8422
m150503_iid=3388511_0      ACCCAAAGCACCTTCACAGACCGAAAAACAT-AAAAAAATTATTAAAAAAAAACCTTCAT   6118
m150505_iid=3777611_0      ACCCAAAGCACCTTCACAGACCGAAAAACAT-AAAAAAATTATTAAAAAAAAACCTTCAT   7542
m150430_iid=2438833_0      ACCCAAAGCACCTTCACAGACCGAAAAACAT-AAAAAAATTATT-AAAAAAAACCTTCAT   7875
m141108_iid=1698969_0      ACCCAAAGCACCTTCACAGACCGAAAAACAT-AAAAAAATTATT-AAAAAAAACCTTCAT   9009
m150430_iid=3059190_0      ACCCAAAGCACCTTCACAGACCGAAAAACAT-AAAAAAATTATTAAAAAAAAACCTTCAT   4731
m141107_iid=1383067_0      ACCCAAAGCACCTTCACAGACCGAAAAACAT-AAAAAAATTATT-AAAAAAAACCTTCAT   4730
m141101_iid=597008_0       ACCCAAAGCACCTTCACAGACCGAAAAACAT-AAAAAAATTATT-AAAAAAAACCTTCAT   1800
m150506_iid=2596496_0      ACCCAAAGCACCTTCACAGACCGAAAAACAT-AAAAAAATTATTAAAAAAAAACCTTCAT   9357
m141102_iid=910051_0       ------------------------------------------------------------   7428
m150505_iid=3784017_0      ACCCAAAGCACCTTCACAGACCGAAAAACAT-AAAAAAATTATTAAAAAAAAACCTTCAT   7657
m141101_iid=682151_0       ACCCAAAGCACCTTCACAGACCGAAAAACAT-AAAAAAATTATTAAAAAAAAACCTTCAT   6725
m150501_iid=3748029_0      ACCCAAAGCACCTTCACAGACCGAAAAACAT-AAAAAAATTATTAAAAAAAAACCTTCAT   7268
m150504_iid=2876272_0      ACCCAAAGCACCTTCACAGACCGAAAAACAT-AAAAAAATTATTAAAAAAAAACCTTCAT   9125
NC_042820.1                ACCCAAAGCACCTTCACAGACCGAAAAACAT-AAAAAAATTATTAAAAAAAAACCTTCAT   9376
m141102_iid=1228825_0      ACCCAAAGCACCTTCACAGACCGAAAAACAT-AAAAAAATTATTAAAAAAAAACCTTCAT   9200
m150504_iid=2876274_0      ACCCAAAGCACCTTCACAGACCGAAAAACAT-AAAAAAATTATTAAAAAAAAACCTTCAT   9198
m141030_iid=80036_0        ACCCAAAGCACCTTCACAGACCGAAAAACAT-AAAAAAATTATTAAAAAAAAACCTTCAT   8966
m150506_iid=2596494_0      ACCCAAAGCACCTTCACAGACCGAAAAACAT-AAAAAAATTATTAAAAAAAAACCTTCAT   9365
m150503_iid=2526862_0      ACCCAAAGCACCTTCACAGACCGAAAAACAT-AAAAAAATTATT-AAAAAAAACCTTCAT   9052
m150506_iid=2567000_0      ACCCAAAGCACCTTCACAGACCGAAAAACAT-AAAAAAATTATTAAAAAAAAACCTTCAT   7004
m150507_iid=3073271_0      ACCCAAAGCACCTTCACAGACCGAAAAACAT-AAAAAAATTATTAAAAAAAAACCTTCAT   8305
m150504_iid=3156087_0      ACCCAAAGCACCTTCACAGACCGAAAAACAT-AAAAAAATTATTAAAAAAAAACCTTCAT   8130
m150505_iid=2498502_0      ACCCAAAGCACCTTCACAGACCGAAAAACAT-AAAAAAATTATTAAAAAAAAACCTTCAT   5780
m141102_iid=754227_0       ACCCAAAGCACCTTCACAGACCGAAAAACAT-AAAAAAATTATTAAAAAAAAACCTTCAT   8342
m150507_iid=3469514_0      ACCCAAAGCACCTTCACAGACCGAAAAACAT-AAAAAAATTATTAAAAAAAAACCTTCAT   8900
Rdo_MitoGenome             ACCCAAAGCACCTTCACAGACCGAAAAACAT-AAAAAAATTATTAAAAAAAAACCTTCAT   9376
m141101_iid=614933_0       ACCCAAAGCACCTTCACAGACCGAAAAACAT-AAAAAAATTATTAAAAAAAAACCTTCAT   7925
```

nad4L

```
m141031_iid=347546_0      AATCAAAAAGAGAGACATAAT-AACAAGATTTAAATATAAAGATAAAACAATAAATTCCA  4133
m150506_iid=3127414_0     AATCAAAAAGAGAGACATAAT-AACAAGATTTAAATATAAAGATAAAACAATAAATTCCA  5873
m141102_iid=1245517_0     ------------------------------------------------------------  7295
m141107_iid=1405727_0     AATCAAAAAGAGAGACATAATAAACAAGATTTAAATATAAAGATAAAACAATAAATTCCA  4708
m141102_iid=987614_0      AATCAAAAAGAGAGACATAATAAACAAGATTTAAATATAAAGATAAAACAATAAATTCCA  7696
m141108_iid=1701429_0     AATCAAAAAGAGAGACATAATAAACAAGATTTAAATATAAAGATAAAACAATAAATTCCA  4441
m141102_iid=987286_0      AATCAAAAAGAGAGACATAATAAACAAGATTTAAATATAAAGATAAAACAATAAATTCCA  5660
m150430_iid=3272125_0     AATCAAAAAGAGAGACATAAA-AACAAGATTTAAATATAAAGAT-AAACAATAAATTCCA  7170
m141102_iid=754225_0      AATCAAAAAGAGAGACATAATAAACAAGATTTAAATATAAAGATAAAACAATAAATTCCA  8346
m141107_iid=1409934_0     AATCAAAAAGAGAGACATAATAAACAAGATTTAAATATAAAGATAAAACAATAAATTCCA  8887
m150503_iid=3416920_0     AATCAAAAAGAGAGACATAATAAACAAGATTTAAATATAAAGATAAAACAATAAATTCCA  7600
m141108_iid=1800121_0     AATCAA-AAGAGAGACATAATAAACAAGATTTAAATATAAAGATAAAACAATAAATTCCA  7413
m141031_iid=147540_0      AATCAAAAAGAGAGACATAATAAACAAGATTTAAATATAAAGATAAAACAATAAATTCCA  7764
m150507_iid=3674924_0     AATCAAAAAGAGAGACATAATAAACAAGATTTAAATATAAAGATAAAACAATAAATTCCA  7916
m150503_iid=2923751_0     AATCAAAAAGAGAGACATAATAAACAAGATTTAAATATAAAGATAAAACAATAAATTCCA  7742
m141031_iid=115118_0      AATCAAAAAGAGAGACATAATAAACAAGATTTAAATATAAAGATAAAACAATAAATTCCA  6611
m150430_iid=2722448_0     AATCAAAAAGAGAGACATAATAAACAAGATTTAAATATAAAGATAAAACAATAAATTCCA  6539
m150507_iid=3102647_0     AATCAAAAAGAGAGACATAATAAACAAGATTTAAATATAAAGATAAAACAATAAATTCCA  4841
m141108_iid=1811866_0     AATCAAAAAGAGAGACATAATAAACAAGATTTAAATATAAAGATAAAACAATAAATTCCA  6727
m150501_iid=2948495_0     AATCAAAAAGAGAGACATAATAAACAAGATTTAAATATAAAGATAAAACAATAAATTCCA  6141
m150504_iid=3238195_0     AATCAA-AAGAGAGACATAATAAACAAGATTTAAATATAAAGATAAAACAATAAATTCCA  8481
m150503_iid=3388511_0     AATCAAAA--AGAGACATAATAAACAAGATTTAAATATAAAGATAAAACAATAAATTCCA  6176
m150505_iid=3777611_0     AATCAAAAAGAGAGACATAATAAACAAGATTTAAATATAAAGATAAAACAATAAATTCCA  7602
m150430_iid=2438833_0     AATCAAAAAGAGAGACATAATAAACAAGATTTAAATATAAAGATAAAACAATAAATTCCA  7935
m141108_iid=1698969_0     AATCAAAAAGAGAGACATAATAAACAAGATTTAAATATAAAGATAAAACAATAAATTCCA  9069
m150430_iid=3059190_0     AATCAAAAAGAGAGACATAATAAACAAGATTTAAATATAAAGATAAAACAATAAATTCCA  4791
m141107_iid=1383067_0     AATCAAAAAGAGAGACATAATAAACAAGATTTAAATATAAAGATAAAACAATAAATTCCA  4790
m141101_iid=597008_0      AATCAAAAAGAGAGACATAATAAACAAGATTTAAATATAAAGATAAAACAATAAATTCCA  1860
m150506_iid=2596496_0     AATCAAAAAGAGAGACATAATAAACAAGATTTAAATATAAAGATAAAACAATAAATTCCA  9417
m141102_iid=910051_0      ------------------------------------------------------------  7428
m150505_iid=3784017_0     AATCAAAAAGAGAGACATAATAAACAAGATTTAAATATAAAGATAAAACAATAAATTCCA  7717
m141101_iid=682151_0      AATCAAAAAGAGAGACATAATAAACAAGATTTAAATATAAAGATAAAACAATAAATTCCA  6785
m150501_iid=3748029_0     AATCAAAAAGAGAGACATAATAAACAAGATTTAAATATAAAGATAAAACAATAAATTCCA  7328
m150504_iid=2876272_0     AATCAAAAAGAGAGACATAATAAACAAGATTTAAATATAAAGATAAAACAATAAATTCCA  9185
NC_042820.1               AATCAAAAAGAGAGACATAATAAACAAGATTTAAATATAAAGATAAAACAATAAATTCCA  9436
m141102_iid=1228825_0     AATCAAAAAGAGAGACATAATAAACAAGATTTAAATATAAAGATAAAACAATAAATTCCA  9260
m150504_iid=2876274_0     AATCAAAAAGAGAGACATAATAAACAAGATTTAAATATAAAGATAAAACAATAAATTCCA  9258
m141030_iid=80036_0       AATCAAAAAGAGAGACATAATAAACAAGATTTAAATATAAAGATAAAACAATAAATTCCA  9026
m150506_iid=2596494_0     AATCAAAAAGAGAGACATAATAAACAAGATTTAAATATAAAGATAAAACAATAAATTCCA  9425
m150503_iid=2526862_0     AATCAAAA--AGAGACATAATAAACAAGATTTAAATATAAAGATAAAACAATAAATTCCA  9110
m150506_iid=2567000_0     AATCAAAAAGAGAGACATAATAAACAAGATTTAAATATAAAGATAAAACAATAAATTCCA  7064
m150507_iid=3073271_0     AATCAAAAAGAGAGACATAATAAACAAGATTTAAATATAAAGATAAAACAATAAATTCCA  8365
m150504_iid=3156087_0     AATCAAAAAGAGAGACATAATAAACAAGATTTAAATATAAAGATAAAACAATAAATTCCA  8190
m150505_iid=2498502_0     AATCAAAAAGAGAGACATAATAAACAAGATTTAAATATAAAGATAAAACAATAAATTCCA  5840
m141102_iid=754227_0      AATCAAAAAGAGAGACATAATAAACAAGATTTAAATATAAAGATAAAACAATAAATTCCA  8402
m150507_iid=3469514_0     AATCAAAAAGAGAGACATAATAAACAAGATTTAAATATAAAGATAAAACAATAAATTCCA  8960
Rdo_MitoGenome            AATCAAAAAGAGAGACATAATAAACAAGATTTAAATATAAAGATAAAACAATAAATTCCA  9436
m141101_iid=614933_0      AATCAAAAAGAGAGACATAATAAACAAGATTTAAATATAAAGATAAAACAATAAATTCCA  7985
```

nad4L

```
m141031_iid=347546_0      AAGATAA--TAATAAAGAAAGTAAATGTTTACGAAAAAAGCTAAATACAGTTACACCACA  4191
m150506_iid=3127414_0     AAGATATAATAATAAAGAAAGTAAATGTTTACGAAAAAAGCTAAATACAGTTACACCACA  5933
m141102_iid=1245517_0     ------------------------------------------------------------  7295
m141107_iid=1405727_0     AAGATAA--TAATAAAGAAAGTAAATGTTTACGAAAAAAGCTAAATACAGTTACACCACA  4766
m141102_iid=987614_0      AAGATAA--TAATAAAGAAAGTAAATGTTTACGAAAAAAGCTAAATACAGTTACACCACA  7754
m141108_iid=1701429_0     AAGATAA--TAATAAAGAAAGTAAATGTTTACGAAAAAAGCTAAATACAGTTACACCACA  4499
m141102_iid=987286_0      AAGATAA--TAATAAAGAAAGTAAATGTTTACGAAAAAAGCTAAATACAGTTACACCACA  5718
m150430_iid=3272125_0     AAGATAA--TAATAAAGAAAGTAAATGTTTACGAAAAAAGCTAAATACAGTTACACCACA  7228
m141102_iid=754225_0      AAGATAA--TAATAAAGAAAGTAAATGTTTACGAAAAAAGCTAAATACAGTTACACCACA  8404
m141107_iid=1409934_0     AAGATAA--TAATAAAGAAAGTAAATGTTTACGAAAAAAGCTAAATACAGTTACACCACA  8945
m150503_iid=3416920_0     AAGATAA--TAATAAAGAAAGTAAATGTTTACGAAAAAAGCTAAATACAGTTACACCACA  7658
m141108_iid=1800121_0     AAGATAA--TAATAAAGAAAGTAAATGTTTACGAA-AAAGCTAAATACAGTTACACCACA  7470
m141031_iid=147540_0      AAGATAA--TAATAAAGAAAGTAAATGTTTACGAAAAAAGCTAAATACAGTTACACCACA  7822
m150507_iid=3674924_0     AAGATAA--TAATAAAGAAAGTAAATGTTTACGAAAAAAGCTAAATACAGTTACACCACA  7974
m150503_iid=2923751_0     AAGATAA--TAATAAAGAAAGTAAATGTTTACGAAAAAAGCTAAATACAGTTACACCACA  7800
m141031_iid=115118_0      AAGATAA--TAATAAAGAAAGTAAATGTTTACGAAAAAAGCTAAATACAGTTACACCACA  6669
m150430_iid=2722448_0     AAGATAA--TAATAAAGAAAGTAAATGTTTACGAAAAAAGCTAAATACAGTTACACCACA  6597
m150507_iid=3102647_0     AAGATAA--TAATAAAGAAAGTAAATGTTTACGAAAAAAGCTAAATACAGTTACACCACA  4899
m141108_iid=1811866_0     AAGATAA--TAATAAAGAAAGTAAATGTTTACGAAAAAAGCTAAATACAGTTACACCACA  6785
m150501_iid=2948495_0     AAGATAA--TAATAAAGAAAGTAAATGTTTACGAAAAAAGCTAAATACAGTTACACCACA  6199
```

```
m150504_iid=3238195_0      AAGATAA--TAATAAAGAAAGTAAATGTTTACGAAAAAAGCTAAATACAGTTACACCACA   8539
m150503_iid=3388511_0      AAGATAA--TAATAAAGAAAGTAAATGTTTACGAAAAAAGCTAAATACAGTTACACCACA   6234
m150505_iid=3777611_0      AAGATAA--TAATAAAGAAAGTAAATGTTTACGAAAAAAGCTAAATACAGTTACACCACA   7660
m150430_iid=2438833_0      AAGATAA--TAATAAAGAAAGTAAATGTTTACGAAAAAAGCTAAATACAGTTACACCACA   7993
m141108_iid=1698969_0      AAGATAA--TAATAAAGAAAGTAAATGTTTACGAAAAAAGCTAAATACAGTTACACCACA   9127
m150430_iid=3059190_0      AAGATAA--TAATAAAGAAAGTAAATGTTTACGAAAAAAGCTAAATACAGTTACACCACA   4849
m141107_iid=1383067_0      AAGATAA--TAATAAAGAAAGTAAATGTTTACGAAAAAAGCTAAATACAGTTACACCACA   4848
m141101_iid=597008_0       AAGATAA--TAATAAAGAAAGTAAATGTTTACGAAAAAAGCTAAATACAGTTACACCACA   1918
m150506_iid=2596496_0      AAGATAA--TAATAAAGAAAGTAAATGTTTACGAAAAAAGCTAAATACAGTTACACCACA   9475
m141102_iid=910051_0       ------------------------------------------------------------   7428
m150505_iid=3784017_0      AAGATAA--TAATAAAGAAAGTAAATGTTTACGAAAAAAGCTAAATACAGTTACACCACA   7775
m141101_iid=682151_0       AAGATAA--TAATAAAGAAAGTAAATGTTTACGAAAAAAGCTAAATACAGTTACACCACA   6843
m150501_iid=3748029_0      AAGATAA--TAATAAAGAAAGTAAATGTTTACGAAAAAAGCTAAATACAGTTACACCACA   7386
m150504_iid=2876272_0      AAGATAA--TAATAAAGAAAGTAAATGTTTACGAAAAAAGCTAAATACAGTTACACCACA   9243
NC_042820.1                AAGATAA--TAATAAAGAAAGTAAATGTTTACGAAAAAAGCTAAATACAGTTACACCACA   9494
m141102_iid=1228825_0      AAGATAA--TAATAAAGAAAGTAAATGTTTACGAAAAAAGCTAAATACAGTTACACCACA   9318
m150504_iid=2876274_0      AAGATAA--TAATAAAGAAAGTAAATGTTTACGAAAAAAGCTAAATACAGTTACACCACA   9316
m141030_iid=80036_0        AAGATAA--TAATAAAGAAAGTAAATGTTTACGAAAAAAGCTAAATACAGTTACACCACA   9084
m150506_iid=2596494_0      AAGATAA--TAATAAAGAAAGTAAATGTTTACGAAAAAAGCTAAATACAGTTACACCACA   9483
m150503_iid=2526862_0      AAGATAA--TAATAAAGAAAGTAAATGTTTACGAAAAAAGCTAAATACAGTTACACCACA   9168
m150506_iid=2567000_0      AAGATAA--TAATAAAGAAAGTAAATGTTTACGAAAAAAGCTAAATACAGTTACACCACA   7122
m150507_iid=3073271_0      AAGATAA--TAATAAAGAAAGTAAATGTTTACGAAAAAAGCTAAATACAGTTACACCACA   8423
m150504_iid=3156087_0      AAGATAA--TAATAAAGAAAGTAAATGTTTACGAAAAAAGCTAAATACAGTTACACCACA   8248
m150505_iid=2498502_0      AAGATAA--TAATAAAGAAAGTAAATGTTTACGAAAAAAGCTAAATACAGTTACACCACA   5898
m141102_iid=754227_0       AAGATAA--TAATAAAGAAAGTAAATGTTTACGAAAAAAGCTAAATACAGTTACACCACA   8460
m150507_iid=3469514_0      AAGATAA--TAATAAAGAAAGTAAATGTTTACGAAAAAAGCTAAATACAGTTACACCACA   9018
Rdo_MitoGenome             AAGATAA--TAATAAAGAAAGTAAATGTTTACGAAAAAAGCTAAATACAGTTACACCACA   9494
m141101_iid=614933_0       AAGATAA--TAATAAAGAAAGTAAATGTTTACGAAAAAAGCTAAATACAGTTACACCACA   8043
```

nad4L

```
m141031_iid=347546_0       TAAATAAACAAATAATATTTCCATTAGTTTTAATAGTTTA--AAAAAACATTGGTCTTGT   4249
m150506_iid=3127414_0      TAAATAAACAAAT-ATATTTCCATTAGTTTTAATAGTTTA--AAAAAACATTGGTCTTGT   5990
m141102_iid=1245517_0      ------------------------------------------------------------   7295
m141107_iid=1405727_0      TAAATAAACAAATAATATTTCCATTAGTTTTAATAGTTTAA-AAAAAACATTGGTCTTGT   4825
m141102_iid=987614_0       TAAATAAACAAAT-ATATTTCCATTAGTTTTAATAGTTTA--AAAAAACATTGGTCTTGT   7811
m141108_iid=1701429_0      TAAATAAACAAATAATATTTCCATTAGTTTTAATAGTTTAA-AAAAAACATTGGTCTTGT   4558
m141102_iid=987286_0       TAAATAAACAAATAATATTTCCATTAGTTTTAATAGTTTAA-AAAAAACATTGGTCTTGT   5777
m150430_iid=3272125_0      TAAATAAACAAATAATATTTCCATTAGTTTTAATAGTTTAAAAAAAAACATTGGTCTTGT   7288
m141102_iid=754225_0       TAAATAAACAAATAATATTTCCATTAGTTTTAATAGTTTAA--AAAAACATTGGTCTTGT   8462
m141107_iid=1409934_0      TAAATAAACAAATAATATTTCCATTAGTTTTAATAGTTTAA-AAAAAACATTGGTCTTGT   9004
m150503_iid=3416920_0      TAAATAAACAAATAATATTTCCATTAGTTTTAATAGTTTAA-AAAAAACATTGGTCTTGT   7717
m141108_iid=1800121_0      TAAATAAACAAATAATATTTCCATTAGTTTTAATAGTTTAA-AAAAAACATTGGTCTTGT   7529
m141031_iid=147540_0       TAAATAAACAAATAATATTTCCATTAGTTTTAATAGTTTAA-AAAAAACATTGGTCTTGT   7881
m150507_iid=3674924_0      TAAATAAACAAATAATATTTCCATTAGTTTTAATAGTTTAA-AAAAAACATTGGTCTTGT   8033
m150503_iid=2923751_0      TAAATAAACAAATAATATTTCCATTAGTTTTAATAGTTTAA-AAAAAACATTGGTCTTGT   7859
m141031_iid=115118_0       TAAATAAACAAATAATATTTCCATTAGTTTTAATAGTTTAA-AAAAAACATTGGTCTTGT   6728
m150430_iid=2722448_0      TAAATAAACAAATAATATTTCCATTAGTTTTAATAGTTTAA-AAAAAACATTGGTCTTGT   6656
m150507_iid=3102647_0      TAAATAAACAAATAATATTTCCATTAGTTTTAATAGTTTAA-AAAAAACATTGGTCTTGT   4958
m141108_iid=1811866_0      TAAATAAACAAATAATATTTCCATTAGTTTTAATAGTTTAA-AAAAAACATTGGTCTTGT   6844
m150501_iid=2948495_0      TAAATAAACAAATAATATTTCCATTAGTTTTAATAGTTTAA-AAAAAACATTGGTCTTGT   6258
m150504_iid=3238195_0      TAAATAAACAAATAATATTTCCATTAGTTTTAATAGTTTAA-AAAAAACATTGGTCTTGT   8598
m150503_iid=3388511_0      TAAATAAACAAATAATATTTCCATTAGTTTTAATAGTTTAA-AAAAAACATTGGTCTTGT   6293
m150505_iid=3777611_0      TAAATAAACAAATAATATTTCCATTAGTTTTAATAGTTTAA-AAAAAACATTGGTCTTGT   7719
m150430_iid=2438833_0      TAAATAAACAAATAATATTTCCATTAGTTTTAATAGTTTAA-AAAAAACATTGGTCTTGT   8052
m141108_iid=1698969_0      TAAATAAACAAATAATATTTCCATTAGTTTTAATAGTTTAA-AAAAAACATTGGTCTTGT   9186
m150430_iid=3059190_0      TAAATAAACAAATAATATTTCCATTAGTTTTAATAGTTTAA-AAAAAACATTGGTCTTGT   4908
m141107_iid=1383067_0      TAAATAAACAAATAATATTTCCATTAGTTTTAATAGTTTAA-AAAAAACATTGGTCTTGT   4907
m141101_iid=597008_0       TAAATAAACAAATAATATTTCCATTAGTTTTAATAGTTTAA-AAAAAACATTGGTCTTGT   1977
m150506_iid=2596496_0      TAAATAAACAAATAATATTTCCATTAGTTTTAATAGTTTAA-AAAAAACATTGGTCTTGT   9534
m141102_iid=910051_0       ------------------------------------------------------------   7428
m150505_iid=3784017_0      TAAATAAACAAATAATATTTCCATTAGTTTTAATAGTTTAA-AAAAAACATTGGTCTTGT   7834
m141101_iid=682151_0       TAAATAAACAAATAATATTTCCATTAGTTTTAATAGTTTAA-AAAAAACATTGGTCTTGT   6902
m150501_iid=3748029_0      TAAATAAACAAATAATATTTCCATTAGTTTTAATAGTTTAA-AAAAAACATTGGTCTTGT   7445
m150504_iid=2876272_0      TAAATAAACAAATAATATTTCCATTAGTTTTAATAGTTTAA-AAAAAACATTGGTCTTGT   9302
NC_042820.1                TAAATAAACAAATAATATTTCCATTAGTTTTAATAGTTTAA-AAAAAACATTGGTCTTGT   9553
m141102_iid=1228825_0      TAAATAAACAAATAATATTTCCATTAGTTTTAATAGTTTAA-AAAAAACATTGGTCTTGT   9377
m150504_iid=2876274_0      TAAATAAACAAATAATATTTCCATTAGTTTTAATAGTTTAA-AAAAAACATTGGTCTTGT   9375
m141030_iid=80036_0        TAAATAAACAAATAATATTTCCATTAGTTTTAATAGTTTAA-AAAAAACATTGGTCTTGT   9143
m150506_iid=2596494_0      TAAATAAACAAATAATATTTCCATTAGTTTTAATAGTTTAA-AAAAAACATTGGTCTTGT   9542
m150503_iid=2526862_0      TAAATAAACAAATAATATTTCCATTAGTTTTAATAGTTTAA-AAAAAACATTGGTCTTGT   9227
m150506_iid=2567000_0      TAAATAAACAAATAATATTTCCATTAGTTTTAATAGTTTAA-AAAAAACATTGGTCTTGT   7181
```

```
m150507_iid=3073271_0       TAAATAAACAAATAATATTTCCATTAGTTTTAATAGTTTAA-AAAAAA-ATTGGTCTTGT   8481
m150504_iid=3156087_0       TAAATAAACAAATAATATTTCCATTAGTTTTAATAGTTTAA-AAAAAACATTGGTCTTGT   8307
m150505_iid=2498502_0       TAAATAAACAAATAATATTTCCATTAGTTTTAATAGTTTAA-AAAAAACATTGGTCTTGT   5957
m141102_iid=754227_0        TAAATAAACAAATAATATTTCCATTAGTTTTAATAGTTTAA-AAAAAACATTGGTCTTGT   8519
m150507_iid=3469514_0       TAAATAAACAAATAATATTTCCATTAGTTTTAATAGTTTAA-AAAAAACATTGGTCTTGT   9077
Rdo_MitoGenome              TAAATAAACAAATAATATTTCCATTAGTTTTAATAGTTTAA-AAAAAACATTGGTCTTGT   9553
m141101_iid=614933_0        TAAATAAACAAATAATATTTCCATTAGTTTTAATAGTTTAA-AAAAAACATTGGTCTTGT   8102

                                     nad4L
m141031_iid=347546_0        AAACCAAAATTAATAATAATTTTAAAACTTCAGAGAAAAGAAAAACTTTTCATTAATCTC   4309
m150506_iid=3127414_0       AAACCAAAATTA-TAATAATTTTAAAACTTCAGAGAAAAGAAAAACTTTTCATTAATCTC   6049
m141102_iid=1245517_0       ------------------------------------------------------------   7295
m141107_iid=1405727_0       AAACCAAAATTAATAATAATTTTAAAAATTCAGAGAAAAGAAAAACTTTTCATTAATCTC   4885
m141102_iid=987614_0        AAACCAAAATTAATAATAATTTTAAAACTTCAGAGAAAAGAAAAACTTTTCATTAATCTC   7871
m141108_iid=1701429_0       AAACCAAAATTAATAATAATTTTAAAACTTCAGAGAAAAGAAAAACTTTTCATTAATCTC   4618
m141102_iid=987286_0        AAACCAAAATTAATAATAATTTTAAAACTTCAGAGAAAAGAAAAACTTTTCATTAATCTC   5837
m150430_iid=3272125_0       AAACCAAAATTAATAATAATTTTAAAACTTCAGAGAAAAGAAAAACTTTTCATTAATCTC   7348
m141102_iid=754225_0        AAACCAAAATTAATAATAATTTTAAAACTTCAGAGAAAAGAAAAACTTTTCATTAATCTC   8522
m141107_iid=1409934_0       AAACCAAAATTAATAATAATTTTAAAACTTCAGAGAAAAGAAAAACTTTTCATTAATCTC   9064
m150503_iid=3416920_0       AAACCAAAATTAATAATAATTTTAAAACTTCAGAGAA-AGAAAAACTTTTCATTAATCTC   7776
m141108_iid=1800121_0       AAACCAAAATTAATAATAATTTTAAAACTTCAGAGAAAAGAAAAACTTTTCATTAATCTC   7589
m141031_iid=147540_0        AAACCAAAATTAATAATAATTTTAAAACTTCAGAGAAAAGAAAAACTTTTCATTAATCTC   7941
m150507_iid=3674924_0       AAACCAAAATTAATAATAATTTTAAAACTTCAGAGAAAAGAAAAACTTTTCATTAATCTC   8093
m150503_iid=2923751_0       AAACCAAAATTAATAATAATTTTAAAACTTCAGAGAA-AGAAAAACTTTTCATTAATCTC   7918
m141031_iid=115118_0        AAACCAAAATTAATAATAATTTTAAAACTTCAGAGAAAAGAAAAACTTTTCATTAATCTC   6788
m150430_iid=2722448_0       AAACCAAAATTAATAATAATTTTAAAACTTCAGAGAAAAGAAAAACTTTTCATTAATCTC   6716
m150507_iid=3102647_0       AAACCAAAATTAATAATAATTTTAAAACTTCAGAGAAAAGAAAAACTTTTCATTAATCTC   5018
m141108_iid=1811866_0       AAACCAAAATTAATAATAATTTTAAAACTTCAGAGAAAAGAAAAACTTTTCATTAATCTC   6904
m150501_iid=2948495_0       AAACCAAAATTAATAATAATTTTAAAACTTCAGAGAAAAGAAAAACTTTTCATTAATCTC   6318
m150504_iid=3238195_0       AAACCAAAATTAATAATAATTTTAAAACTTCAGAGAAAAGAAAAACTTTTCATTAATCTC   8658
m150503_iid=3388511_0       AAACCAAAATTAATAATAATTTTAAAACTTCAGAGAAAAGAAAAACTTTTCATTAATCTC   6353
m150505_iid=3777611_0       AAACCAAAATTAATAATAATTTTAAAACTTCAGAGAAAAGAAAAACTTTTCATTAATCTC   7779
m150430_iid=2438833_0       AAACCAAAATTAATAATAATTTTAAAACTTCAGAGAAAAGAAAAACTTTTCATTAATCTC   8112
m141108_iid=1698969_0       AAACCAAAATTAATAATAATTTTAAAACTTCAGAGAAAAGAAAAACTTTTCATTAATCTC   9246
m150430_iid=3059190_0       AAACCAAAATTAATAATAATTTTAAAACTTCAGAGAAAAGAAAAACTTTTCATTAATCTC   4968
m141107_iid=1383067_0       AAACCAAAATTAATAATAATTTTAAAACTTCAGAGAAAAGAAAAACTTTTCATTAATCTC   4967
m141101_iid=597008_0        AAACCAAAATTAATAATAATTTTAAAACTTCAGAGAAAAGAAAAACTTTTCATTAATCTC   2037
m150506_iid=2596496_0       AAACCAAAATTAATAATAATTTTAAAACTTCAGAGAAAAGAAAAACTTTTCATTAATCTC   9594
m141102_iid=910051_0        ------------------------------------------------------------   7428
m150505_iid=3784017_0       AAACCAAAATTAATAATAATTTTAAAACTTCAGAGAAAAGAAAAACTTTTCATTAATCTC   7894
m141101_iid=682151_0        AAACCAAAATTAATAATAATTTTAAAACTTCAGAGAAAAGAAAAACTTTTCATTAATCTC   6962
m150501_iid=3748029_0       AAACCAAAATTAATAATAATTTTAAAACTTCAGAGAAAAGAAAAACTTTTCATTAATCTC   7505
m150504_iid=2876272_0       AAACCAAAATTAATAATAATTTTAAAACTTCAGAGAAAAGAAAAACTTTTCATTAATCTC   9362
NC_042820.1                 AAACCAAAATTAATAATAATTTTAAAACTTCAGAGAAAAGAAAAACTTTTCATTAATCTC   9613
m141102_iid=1228825_0       AAACCAAAATTAATAATAATTTTAAAACTTCAGAGAAAAGAAAAACTTTTCATTAATCTC   9437
m150504_iid=2876274_0       AAACCAAAATTAATAATAATTTTAAAACTTCAGAGAAAAGAAAAACTTTTCATTAATCTC   9435
m141030_iid=80036_0         AAACCAAAATTAATAATAATTTTAAAACTTCAGAGAAAAGAAAAACTTTTCATTAATCTC   9203
m150506_iid=2596494_0       AAACCAAAATTAATAATAATTTTAAAACTTCAGAGAAAAGAAAAACTTTTCATTAATCTC   9602
m150503_iid=2526862_0       AAACCAAAATTAATAATAATTTTAAAACTTCAGAGAAAAGAAAAACTTTTCATTAATCTC   9287
m150506_iid=2567000_0       AAACCAAAATTAATAATAATTTTAAAACTTCAGAGAAAAGAAAAACTTTTCATTAATCTC   7241
m150507_iid=3073271_0       AAACCAAAATTAATAATAATTTTAAAACTTCAGAGAAAAGAAAAACTTTTCATTAATCTC   8541
m150504_iid=3156087_0       AAACCAAAATTAATAATAATTTTAAAACTTCAGAGAAAAGAAAAACTTTTCATTAATCTC   8367
m150505_iid=2498502_0       AAACCAAAATTAATAATAATTTTAAAACTTCAGAGAAAAGAAAAACTTTTCATTAATCTC   6017
m141102_iid=754227_0        AAACCAAAATTAATAATAATTTTAAAACTTCAGAGAAAAGAAAAACTTTTCATTAATCTC   8579
m150507_iid=3469514_0       AAACCAAAATTAATAATAATTTTAAAACTTCAGAGAAAAGAAAAACTTTTCATTAATCTC   9137
Rdo_MitoGenome              AAACCAAAATTAATAATAATTTTAAAACTTCAGAGAAAAGAAAAACTTTTCATTAATCTC   9613
m141101_iid=614933_0        AAACCAAAATTAATAATAATTTTAAAACTTCAGAGAAAAGAAAAACTTTTCATTAATCTC   8162


m141031_iid=347546_0        CAAAATTAATAT-TTATTATAAACTATTCTCTGCATTAAAATCTTAACAACAATATTAAT   4368
m150506_iid=3127414_0       CAAAATTAATAT-TTTATATAAACTATTCTCTGCATTAAAATCTTAACAACAATATTAAT   6108
m141102_iid=1245517_0       ------------------------------------------------------------   7295
m141107_iid=1405727_0       CAAATTAATA--TTTTATATAAACTATTCTCTGCATTAA-ATCTTAACAACAATATTAAT   4942
m141102_iid=987614_0        CAAAATTAATAT-TTTATATAAACTATTCTCTGCATTAAAATCTTAACAACAATATTAAT   7930
m141108_iid=1701429_0       CAAAATTAATAT-TTTATATAAACTATTCTCTGCATTAAAATCTTAACAACAATATTAAT   4677
m141102_iid=987286_0        CAAAATTAATAT-TTTATATAAACTATTCTCTGCATTAAAATCTTAACAACAATATTAAT   5896
m150430_iid=3272125_0       CAAAATTAATAT-TTTATATAAACTATTCTCTGCATTAAAATCTTAACAACAATATTAAT   7407
m141102_iid=754225_0        CAAAATTAATAT-TTTATATAAACTATTCTCTGCATTAAAATCTTAACAACAATATTAAT   8581
m141107_iid=1409934_0       CAAAATTAATATTTTATATAAACTATTCTCTGCATTAAAATCTTAACAACAATATTAAT    9124
m150503_iid=3416920_0       CAAAATTAATAT-TTTATATAAACTATTCTCTGCATTAAAATCTTAACAACAATATTAAT   7835
m141108_iid=1800121_0       CAAAATTAATAT-TTTATATAAACTATTCTCTGCATTAAAATCTTAACAACAATATTAAT   7648
```

```
m141031_iid=147540_0        CAAAATTAATAT-TTTATATAAACTATTCTCTGCATTAAAATCTTAACAACAATATTAAT   8000
m150507_iid=3674924_0       CAAAATTAATAT-TTTATATAAACTATTCTCTGCATTAAAATCTTAACAACAATATTAAT   8152
m150503_iid=2923751_0       CAAAATTAATAT-TTTATATAAACTATTCTCTGCATTAAAATCTTAACAACAATATTAAT   7977
m141031_iid=115118_0        CAAAATTAATAT-TTTATATAAACTATTCTCTGCATTAAAATCTTAACAACAATATTAAT   6847
m150430_iid=2722448_0       CAAAATTAATAT-TTTATATAAACTATTCTCTGCATTAAAATCTTAACAACAATATTAAT   6775
m150507_iid=3102647_0       CAAAATTAATAT-TTTATATAAACTATTCTCTGCATTAAAATCTTAACAACAATATTAAT   5077
m141108_iid=1811866_0       CAAAATTAATAT-TTTATATAAACTATTCTCTGCATTAAAATCTTAACAACAATATTAAT   6963
m150501_iid=2948495_0       CAAAATTAATAT-TTTATATAAACTATTCTCTGCATTA-AATCTTAACAACAATATTAAT   6376
m150504_iid=3238195_0       CAAAATTAATAT-TTTATATAAACTATTCTCTGCATTAAAATCTTAACAACAATATTAAT   8717
m150503_iid=3388511_0       CAAAATTAATAT-TTTATATAAACTATTCTCTGCATTAAAATCTTAACAACAATATTAAT   6412
m150505_iid=3777611_0       CAAAATTAATAT-TTTATATAAACTATTCTCTGCATTAAAATCTTAACAACAATATTAAT   7838
m150430_iid=2438833_0       CAAAATTAATAT-TTTATATAAACTATTCTCTGCATTAAAATCTTAACAACAATATTAAT   8171
m141108_iid=1698969_0       CAAAATTAATAT-TTTATATAAACTATTCTCTGCATTAAAATCTTAACAACAATATTAAT   9305
m150430_iid=3059190_0       CAAAATTAATAT-TTTATATAAACTATTCTCTGCATTAAAATCTTAACAACAATATTAAT   5027
m141107_iid=1383067_0       CAAAATTAATAT-TTTATATAAACTATTCTCTGCATTAAAATCTTAACAACAATATTAAT   5026
m141101_iid=597008_0        CAAAATTAATAT-TTTATATAAACTATTCTCTGCATTAAAATCTTAACAACAATATTAAT   2096
m150506_iid=2596496_0       CAAAATTAATAT-TTTATATAAACTATTCTCTGCATTAAAATCTTAACAACAATATTAAT   9653
m141102_iid=910051_0        ------------------------------------------------------------   7428
m150505_iid=3784017_0       CAAAATTAATAT-TTTATATAAACTATTCTCTGCATTAAAATCTTAACAACAATATTAAT   7953
m141101_iid=682151_0        CAAAATTAATAT-TTTATATAAACTATTCTCTGCATTAAAATCTTAACAACAATATTAAT   7021
m150501_iid=3748029_0       CAAAATTAATAT-TTTATATAAACTATTCTCTGCATTAAAATCTTAACAACAATATTAAT   7564
m150504_iid=2876272_0       CAAAATTAATAT-TTTATATAAACTATTCTCTGCATTAAAATCTTAACAACAATATTAAT   9421
NC_042820.1                 CAAAATTAATAT-TTTATATAAACTATTCTCTGCATTAAAATCTTAACAACAATATTAAT   9672
m141102_iid=1228825_0       CAAAATTAATAT-TTTATATAAACTATTCTCTGCATTAAAATCTTAACAACAATATTAAT   9496
m150504_iid=2876274_0       CAAAATTAATAT-TTTATATAAACTATTCTCTGCATTAAAATCTTAACAACAATATTAAT   9494
m141030_iid=80036_0         CAAAATTAATAT-TTTATATAAACTATTCTCTGCATTAAAATCTTAACAACAATATTAAT   9262
m150506_iid=2596494_0       CAAAATTAATAT-TTTATATAAACTATTCTCTGCATTAAAATCTTAACAACAATATTAAT   9661
m150503_iid=2526862_0       CAAAATTAATAT-TTTATATAAACTATTCTCTGCATTAAAATCTTAACAACAATATTAAT   9346
m150506_iid=2567000_0       CAAAATTAATAT-TTTATATAAACTATTCTCTGCATTAAAATCTTAACAACAATATTAAT   7300
m150507_iid=3073271_0       CAAAATTAATAT-TTTATATAAACTATTCTCTGCATTAAAATCTTAACAACAATATTAAT   8600
m150504_iid=3156087_0       CAAAATTAATAT-TTTATATAAACTATTCTCTGCATTAAAATCTTAACAACAATATTAAT   8426
m150505_iid=2498502_0       CAAAATTAATAT-TTTATATAAACTATTCTCTGCATTAAAATCTTAACAACAATATTAAT   6076
m141102_iid=754227_0        CAAAATTAATAT-TTTATATAAACTATTCTCTGCATTAAAATCTTAACAACAATATTAAT   8638
m150507_iid=3469514_0       CAAAATTAATAT-TTTATATAAACTATTCTCTGCATTAAAATCTTAACAACAATATTAAT   9196
Rdo_MitoGenome              CAAAATTAATAT-TTTATATAAACTATTCTCTGCATTAAAATCTTAACAACAATATTAAT   9672
m141101_iid=614933_0        CAAAATTAATAT-TTTATATAAACTATTCTCTGCATTAAAATCTTAACAACAATATTAAT   8221

                                                          nad6

m141031_iid=347546_0        TATTTCAAGATCAACATTTATT-TTTATAATACATCCAATATCAATAACAATAAATTTAA   4427
m150506_iid=3127414_0       TATTTCAAGATCAACATTTTTATTTTATAATACATCCAATATCAATAACAATAAATTTAA   6168
m141102_iid=1245517_0       ------------------------------------------------------------   7295
m141107_iid=1405727_0       -TATTCAAGATCAACATTT----TTTATAATACATCCAATATCAATAACAATAAATTTAA   4997
m141102_iid=987614_0        TATTTCAAGATCAACATTTATTTTTTATAATACATCCAATATCAATAACAATAAATTTAA   7990
m141108_iid=1701429_0       TATTTCAAGATCAACATTTA-TTTTTATAATACATCCAATATCAATAACAATAAATTTAA   4736
m141102_iid=987286_0        TATTTCAAGATCAACATTTA-TTTTTATAATACATCCAATATCAATAACAATAAATTTAA   5955
m150430_iid=3272125_0       TATTTCAAGATCAACATTTA-TTTTTATAATACATCCAATATCAATAACAATAAATTTAA   7466
m141102_iid=754225_0        TATTTCAAGATCAACATTTA-TTTTTATAATACATCCAATATCAATAACAATAAATTTAA   8640
m141107_iid=1409934_0       TATTTCAAGATCAACATTTA-TTTTTATAATACATCCAATATCAATAACAATAAATTTAA   9183
m150503_iid=3416920_0       TATTTCAAGATCAACATTTA-TTTTTATAATACATCCAATATCAATAACAATAAATTTAA   7894
m141108_iid=1800121_0       TATTTCAAGATCAACATTTA-TTTTTATAATACATCCAATATCAATAACAATAAATTTAA   7707
m141031_iid=147540_0        TATTTCAAGATCAACATTTA-TTTTTATAATACATCCAATATCAATAACAATAAATTTAA   8059
m150507_iid=3674924_0       TATTTCAAGATCAACATTTA-TTTTTATAATACATCCAATATCAATAACAATAAATTTAA   8211
m150503_iid=2923751_0       TATTTCAAGATCAACATTTA-TTTTTATAATACATCCAATATCAATAACAATAAATTTAA   8036
m141031_iid=115118_0        TATTTCAAGATCAACATTTA-TTTTTATAATACATCCAATATCAATAACAATAAATTTAA   6906
m150430_iid=2722448_0       TATTTCAAGATCAACATTTA-TT-TTATAATACATCCAATATCAATAACAATAAATTTAA   6833
m150507_iid=3102647_0       TATTTCAAGATCAACATTTA-TTTTTATAATACATCCAATATCAATAACAATAAATTTAA   5136
m141108_iid=1811866_0       TATTTCAAGATCAACATTTA-TTTTTATAATACATCCAATATCAATAACAATAAATTTAA   7022
m150501_iid=2948495_0       TATTTCAAGATCAACATTTA-TTTTTATAATACATCCAATATCAATAACAATAAATTTAA   6435
m150504_iid=3238195_0       TATTTCAAGATCAACATTTA-TT-TTATAATACATCCAATATCAATAACAATAAATTTAA   8775
m150503_iid=3388511_0       TATTTCAAGATCAACATTTA-TTTTTATAATACATCCAATATCAATAACAATAAATTTAA   6471
m150505_iid=3777611_0       TATTTCAAGATCAACATTTA-TTTTTATAATACATCCAATATCAATAACAATAAATTTAA   7897
m150430_iid=2438833_0       TATTTCAAGATCAACATTTA-TTTTTATAATACATCCAATATCAATAACAATAAATTTAA   8230
m141108_iid=1698969_0       TATTTCAAGATCAACATTTA-TTTTTATAATACATCCAATATCAATAACAATAAATTTAA   9364
m150430_iid=3059190_0       TATTTCAAGATCAACATTTA-TTTTTATAATACATCCAATATCAATAACAATAAATTTAA   5086
m141107_iid=1383067_0       TATTTCAAGATCAACATTTA-TTTTTATAATACATCCAATATCAATAACAATAAATTTAA   5085
m141101_iid=597008_0        TATTTCAAGATCAACATTTA-TTTTTATAATACATCCAATATCAATAACAATAAATTTAA   2155
m150506_iid=2596496_0       TATTTCAAGATCAACATTTA-TTTTTATAATACATCCAATATCAATAACAATAAATTTAA   9712
m141102_iid=910051_0        ------------------------------------------------------------   7428
m150505_iid=3784017_0       TATTTCAAGATCAACATTTA-TTTTTATAATACATCCAATATCAATAACAATAAATTTAA   8012
m141101_iid=682151_0        TATTTCAAGATCAACATTTA-TTTTTATAATACATCCAATATCAATAACAATAAATTTAA   7080
m150501_iid=3748029_0       TATTTCAAGATCAACATTTA-TTTTTATAATACATCCAATATCAATAACAATAAATTTAA   7623
```

```
m150504_iid=2876272_0      TATTTCAAGATCAACATTTA-TTTTTATAATACATCCAATATCAATAACAATAAATTTAA   9480
NC_042820.1                TATTTCAAGATCAACATTTA-TTTTTATAATACATCCAATATCAATAACAATAAATTTAA   9731
m141102_iid=1228825_0      TATTTCAAGATCAACATTTA-TTTTTATAATACATCCAATATCAATAACAATAAATTTAA   9555
m150504_iid=2876274_0      TATTTCAAGATCAACATTTA--TTTTATAATACATCCAATATCAATAACAATAAATTTAA   9552
m141030_iid=80036_0        TATTTCAAGATCAACATTTA-TTTTTATAATACATCCAATATCAATAACAATAAATTTAA   9321
m150506_iid=2596494_0      TATTTCAAGATCAACATTTA-TTTTTATAATACATCCAATATCAATAACAATAAATTTAA   9720
m150503_iid=2526862_0      TATTTCAAGATCAACATTTA-TTTTTATAATACATCCAATATCAATAACAATAAATTTAA   9405
m150506_iid=2567000_0      TATTTCAAGATCAACATTTA-TTTTTATAATACATCCAATATCAATAACAATAAATTTAA   7359
m150507_iid=3073271_0      TATTTCAAGATCAACATTTA-TTTTTATAATACATCCAATATCAATAACAATAAATTTAA   8659
m150504_iid=3156087_0      TATTTCAAGATCAACATTTA-TTTTTATAATACATCCAATATCAATAACAATAAATTTAA   8485
m150505_iid=2498502_0      TATTTCAAGATCAACATTTA-TTTTTATAATACATCCAATATCAATAACAATAAATTTAA   6135
m141102_iid=754227_0       TATTTCAAGATCAACATTTA-TTTTTATAATACATCCAATATCAATAACAATAAATTTAA   8697
m150507_iid=3469514_0      TATTTCAAGATCAACATTTA-TTTTTATAATACATCCAATATCAATAACAATAAATTTAA   9255
Rdo_MitoGenome             TATTTCAAGATCAACATTTA-TTTTTATAATACATCCAATATCAATAACAATAAATTTAA   9731
m141101_iid=614933_0       TATTTCAAGATCAACATTTA-TTTTTATAATACATCCAATATCAATAACAATAAATTTAA   8280
```

nad6

```
m141031_iid=347546_0       TAATTCAAGCAATTATTATTGCAATCATAACAGGATCAATTCAAATTAATTACTGATATT   4487
m150506_iid=3127414_0      TAATTCAAGCAATTATTATTGCAATCATAACAGGATCAATTCAAATTAATTACTGATATT   6228
m141102_iid=1245517_0      ------------------------------------------------------------   7295
m141107_iid=1405727_0      TAATTCAAGCAATTATTATTGCAATCATAACAGGATCAATTCAAATTAATTACTGATATT   5057
m141102_iid=987614_0       TTTCAAGCAATTATTATATTGCAATCATAACAGGATCAATTCAAATTAATTACTGATATT   8050
m141108_iid=1701429_0      TAATTCAAGCAATTATTATTGCAATCATAACAGGATCAATTCAAATTAATTACTGATATT   4796
m141102_iid=987286_0       TAATTCAAGCAATTATTATTGCAATCATAACAGGATCAATTCAAATTAATTACTGATATT   6015
m150430_iid=3272125_0      TAATTCAAGCAATTATTATTGCAATCATAACAGGATCAATTCAAATTAATTACTGATATT   7526
m141102_iid=754225_0       TAATTCAAGCAATTATTATTGCAATCATAACAGGATCAATTCAAATTAATTACTGATATT   8700
m141107_iid=1409934_0      TAATTCAAGCAATTATTATTGCAATCATAACAGGATCAATTCAAATTAATTACTGATATT   9243
m150503_iid=3416920_0      TAATTCAAGCAATTATTATTGCAATCATAACAGGATCAATTCAAATTAATTACTGATATT   7954
m141108_iid=1800121_0      TAATTCAAGCAATTATTATTGCAATCATAACAGGATCAATTCAAATTAATTACTGATATT   7767
m141031_iid=147540_0       TAATTCAAGCAATTATTATTGCAATCATAACAGGATCAATTCAAATTAATTACTGATATT   8119
m150507_iid=3674924_0      TAATTCAAGCAATTATTATTGCAATCATAACAGGATCAATTCAAATTAATTACTGATATT   8271
m150503_iid=2923751_0      TAATTCAAGCAATTATTATTGCAATCATAACAGGATCAATTCAAATTAATTACTGATATT   8096
m141031_iid=115118_0       TAATTCAAGCAATTATTATTGCAATCATAACAGGATCAATTCAAATTAATTACTGATATT   6966
m150430_iid=2722448_0      TAATTCAAGCAATTATTATTGCAATCATAACAGGATCAATTCAAATTAATTACTGATATT   6893
m150507_iid=3102647_0      TAATTCAAGCAATTATTATTGCAATCATAACAGGATCAATTCAAATTAATTACTGATATT   5196
m141108_iid=1811866_0      TAATTCAAGCAATTATTATTGCAATCATAACAG-ATCAATTCAAATTAATTACTGATATT   7081
m150501_iid=2948495_0      TAATTCAAGCAATTATTATTGCAATCATAACAGGATCAATTCAAATTAATTACTGATATT   6495
m150504_iid=3238195_0      TAATTCAAGCAATTATTATTGCAATCATAACAGGATCAATTCAAATTAATTACTGATATT   8835
m150503_iid=3388511_0      TAATTCAAGCAATTATTATTGCAATCATAACAGGATCAATTCAAATTAATTACTGATATT   6531
m150505_iid=3777611_0      TAATTCAAGCAATTATTATTGCAATCATAACAGGATCAATTCAAATTAATTACTGATATT   7957
m150430_iid=2438833_0      TAATTCAAGCAATTATTATTGCAATCATAACAGGATCAATTCAAATTAATTACTGATATT   8290
m141108_iid=1698969_0      TAATTCAAGCAATTATTATTGCAATCATAACAGGATCAATTCAAATTAATTACTGATATT   9424
m150430_iid=3059190_0      TAATTCAAGCAATTATTATTGCAATCATAACAGGATCAATTCAAATTAATTACTGATATT   5146
m141107_iid=1383067_0      TAATTCAAGCAATTATTATTGCAATCATAACAGGATCAATTCAAATTAATTACTGATATT   5145
m141101_iid=597008_0       TAATTCAAGCAATTATTATTGCAATCATAACAGGATCAATTCAAATTAATTACTGATATT   2215
m150506_iid=2596496_0      TAATTCAAGCAATTATTATTGCAATCATAACAGGATCAATTCAAATTAATTACTGATATT   9772
m141102_iid=910051_0       ------------------------------------------------------------   7428
m150505_iid=3784017_0      TAATTCAAGCAATTATTATTGCAATCATAACAGGATCAATTCAAATTAATTACTGATATT   8072
m141101_iid=682151_0       TAATTCAAGCAATTATTATTGCAATCATAACAGGATCAATTCAAATTAATTACTGATATT   7140
m150501_iid=3748029_0      TAATTCAAGCAATTATTATTGCAATCATAACAGGATCAATTCAAATTAATTACTGATATT   7683
m150504_iid=2876272_0      TAATTCAAGCAATTATTATTGCAATCATAACAGGATCAATTCAAATTAATTACTGATATT   9540
NC_042820.1                TAATTCAAGCAATTATTATTGCAATCATAACAGGATCAATTCAAATTAATTACTGATATT   9791
m141102_iid=1228825_0      TAATTCAAGCAATTATTATTGCAATCATAACAGGATCAATTCAAATTAATTACTGATATT   9615
m150504_iid=2876274_0      TAATTCAAGCAATTATTATTGCAATCATAACAGGATCAATTCAAATTAATTACTGATATT   9612
m141030_iid=80036_0        TAATTCAAGCAATTATTATTGCAATCATAACAGGATCAATTCAAATTAATTACTGATATT   9381
m150506_iid=2596494_0      TAATTCAAGCAATTATTATTGCAATCATAACAGGATCAATTCAAATTAATTACTGATATT   9780
m150503_iid=2526862_0      TAATTCAAGCAATTATTATTGCAATCATAACAGGATCAATTCAAATTAATTACTGATATT   9465
m150506_iid=2567000_0      TAATTCAAGCAATTATTATTGCAATCATAACAGGATCAATTCAAATTAATTACTGATATT   7419
m150507_iid=3073271_0      TAATTCAAGCAATTATTATTGCAATCATAACAGGATCAATTCAAATTAATTACTGATATT   8719
m150504_iid=3156087_0      TAATTCAAGCAATTATTATTGCAATCATAACAGGATCAATTCAAATTAATTACTGATATT   8545
m150505_iid=2498502_0      TAATTCAAGCAATTATTATTGCAATCATAACAGGATCAATTCAAATTAATTACTGATATT   6195
m141102_iid=754227_0       TAATTCAAGCAATTATTATTGCAATCATAACAGGATCAATTCAAATTAATTACTGATATT   8757
m150507_iid=3469514_0      TAATTCAAGCAATTATTATTGCAATCATAACAGGATCAATTCAAATTAATTACTGATATT   9315
Rdo_MitoGenome             TAATTCAAGCAATTATTATTGCAATCATAACAGGATCAATTCAAATTAATTACTGATATT   9791
m141101_iid=614933_0       TAATTCAAGCAATTATTATTGCAATCATAACAGGATCAATTCAAATTAATTACTGATATT   8340
```

nad6

```
m141031_iid=347546_0       CTTACATTATATTTATAATCATAATTGGAGGAATAATAGTATTAATTATATATATA-ACA   4546
m150506_iid=3127414_0      CTTACATTATATTTATAATCATAATTGGAGGAATAATAGTATTAATTATATATATA-ACA   6287
m141102_iid=1245517_0      ------------------------------------------------------------   7295
m141107_iid=1405727_0      CTTACATTATATTTATAATCATAATTGGAGGAATAATAGTATTAATTAT-ATATATAACA   5116
```

```
m141102_iid=987614_0      CTTACATTATATTTATAATCATAATTGAGAGGAATAATAGTATTAATTATATATATATAACA   8110
m141108_iid=1701429_0     CTTACATTATATTTATAATCATAATTGGAGGAATAATAGTATTAATTATATATA-TAACA   4855
m141102_iid=987286_0      CTTACATTATATTTATAATCATAATTGGAGGAATAATAGTATTAATTATATATA-TAACA   6074
m150430_iid=3272125_0     CTTACATTATATTTATAATCATAATTGGAGGAATAATAGTATTAATTATATATA-TAACA   7585
m141102_iid=754225_0      CTTACATTATATTTATAATCATAATTGGAGGAATAATAGTATTAATTATATATA-TAACA   8759
m141107_iid=1409934_0     CTTACATTATATTTATAATCATAATTGGAGGAATAATAGTATTAATTATATATA-TAACA   9302
m150503_iid=3416920_0     CTTACATTATATTTATAATCATAATTGGAGGAATAATAGTATTAATTATATATA-TAACA   8013
m141108_iid=1800121_0     CTTACATTATATTTATAATCATAATTGGAGGAATAATAGTATTAATTATATATA-TAACA   7826
m141031_iid=147540_0      CTTACATTATATTTATAATCATAATTGGAGGAATAATAGTATTAATTATATATATA-ACA   8178
m150507_iid=3674924_0     CTTACATTATATTTATAATCATAATTGGAGGAATAATAGTATTAATTATATATA-TAACA   8330
m150503_iid=2923751_0     CTTACATTATATTTATAATCATAATTGGAGGAATAATAGTATTAATTATATATA-TAACA   8155
m141031_iid=115118_0      CTTACATTATATTTATAATCATAATTGGAGGAATAATAGTATTAATTATATATA-TAACA   7025
m150430_iid=2722448_0     CTTACATTATATTTATAATCATAATTGGAGGAATAATAGTATTAATA---TATTAACAAA   6950
m150507_iid=3102647_0     CTTACATTATATTTATAATCATAATTGGAGGAATAATAGTATTAATTATATATATATACA   5256
m141108_iid=1811866_0     CTTACATTATATTTATAATCATAATTGGAGGAATAATAGTATTAATTATATATA-TAACA   7140
m150501_iid=2948495_0     CTTACATTATATTTATAATCATAATTGGAGGAATAATAGTATTAATTATATATA-TAACA   6554
m150504_iid=3238195_0     CTTACATTATATTTATAATCATAATTGGAGGAATAATAGTATTAATTATATATA-TAACA   8894
m150503_iid=3388511_0     CTTACATTATATTTATAATCATAATTGGAGGAATAATAGTATTAATTATATATATA-ACA   6590
m150505_iid=3777611_0     CTTACATTATATTTATAATCATAATTGGAGGAATAATAGTATTAATTATATATATA-ACA   8016
m150430_iid=2438833_0     CTTACATTATATTTATAATCATAATTGGAGGAATAATAGTATTAATTATATATATA-ACA   8349
m141108_iid=1698969_0     CTTACATTATATTTATAATCATAATTGGAGGAATAATAGTATTAATTATATATATA-ACA   9483
m150430_iid=3059190_0     CTTACATTATATTTATAATCATAATTGGAGGAATAATAGTATTAATTATATATA-TAACA   5205
m141107_iid=1383067_0     CTTACATTATATTTATAATCATAATTGGAGGAATAATAGTATTAATTATATATA-TAACA   5204
m141101_iid=597008_0      CTTACATTATATTTATAATCATAATTGGAGGAATAATAGTATTAATTATATATA-TAACA   2274
m150506_iid=2596496_0     CTTACATTATATTTATAATCATAATTGGAGGAATAATAGTATTAATTATATATA-TAACA   9831
m141102_iid=910051_0      ------------------------------------------------------------   7428
m150505_iid=3784017_0     CTTACATTATATTTATAATCATAATTGGAGGAATAATAGTATTAATTATATATATA-ACA   8131
m141101_iid=682151_0      CTTACATTATATTTATAATCATAATTGGAGGAATAATAGTATTAATTATATATATA-ACA   7199
m150501_iid=3748029_0     CTTACATTATATTTATAATCATAATTGGAGGAATAATAGTATTAATTATATATATATACA   7743
m150504_iid=2876272_0     CTTACATTATATTTATAATCATAATTGGAGGAATAATAGTATTAATTATATATATA-ACA   9599
NC_042820.1               CTTACATTATATTTATAATCATAATTGGAGGAATAATAGTATTAATTATATATATA-ACA   9850
m141102_iid=1228825_0     CTTACATTATATTTATAATCATAATTGGAGGAATAATAGTATTAATTATATATATA-ACA   9674
m150504_iid=2876274_0     CTTACATTATATTTATAATCATAATTGGAGGAATAATAGTATTAATTATATATATA-ACA   9671
m141030_iid=80036_0       CTTACATTATATTTATAATCATAATTGGAGGAATAATAGTATTAATTATATATATA-ACA   9440
m150506_iid=2596494_0     CTTACATTATATTTATAATCATAATTGGAGGAATAATAGTATTAATTATATATATA-ACA   9839
m150503_iid=2526862_0     CTTACATTATATTTATAATCATAATTGGAGGAATAATAGTATTAATTATATATATA-ACA   9524
m150506_iid=2567000_0     CTTACATTATATTTATAATCATAATTGGAGGAATAATAGTATTAATTATATATATA-ACA   7478
m150507_iid=3073271_0     CTTACATTATATTTATAATCATAATTGGAGGAATAATAGTATTAATTATATATATA-ACA   8778
m150504_iid=3156087_0     CTTACATTATATTTATAATCATAATTGGAGGAATAATAGTATTAATTATATATATA-ACA   8604
m150505_iid=2498502_0     CTTACATTATATTTATAATCATAATTGGAGGAATAATAGTATTAATTATATATATA-ACA   6254
m141102_iid=754227_0      CTTACATTATATTTATAATCATAATTGGAGGAATAATAGTATTAATTATATATATA-ACA   8816
m150507_iid=3469514_0     CTTACATTATATTTATAATCATAATTGGAGGAATAATAGTATTAATTATATATATA-ACA   9374
Rdo_MitoGenome            CTTACATTATATTTATAATCATAATTGGAGGAATAATAGTATTAATTATATATATA-ACA   9850
m141101_iid=614933_0      CTTACATTATATTTATAATCATAATTGGAGGAATAATAGTATTAATTATATATATA-ACA   8399
```

nad6 →

```
m141031_iid=347546_0      AGAATTGCATCAAATGAAATATTCAAATTA-AAAAGAAAATACTTTTGATTCCAATCTTA   4605
m150506_iid=3127414_0     AGAATTGCATCAAATGAAATATTCAAATTAAAAAAGAAAATACTTTTGATTCCAATCTTA   6347
m141102_iid=1245517_0     ------------------------------------------------------------   7295
m141107_iid=1405727_0     AGAATTGCATCAAATGAAATATTCAAATTAAAAAAGAAAATACTTTTGATTCCAATCTTA   5176
m141102_iid=987614_0      AGAATTGCATCAAATGAAAATT---CAAATTAAAAAAAAAATACTTTTGATTCCAATCTTA   8167
m141108_iid=1701429_0     AGAATTGCATCAAATGAAATATTCAAATTAAAAAAGAAAATACTTTTGATTCCAATCTTA   4915
m141102_iid=987286_0      AGAATTGCATCAAATGAAATATTCAAATTAAAAAAGAAAATACTTTTGATTCCAATCTTA   6134
m150430_iid=3272125_0     AGAATTGCATCAAATGAAATATTCAAATTAAAAAAGAAAATACTTTTGATTCCAATCTTA   7645
m141102_iid=754225_0      AGAATTGCATCAAATGAAATATTCAAATTAAAAAAGAAAATACTTTTGATTCCAATCTTA   8819
m141107_iid=1409934_0     AGAATTGCATCAAATGAAATATTCAAATTAAAAAAGAAAATACTTTTGATTCCAATCTTA   9362
m150503_iid=3416920_0     AGAATTGCATCAAATGAAATATTCAAATTAAAAAAGAAAATACTTTTGATTCCAATCTTA   8073
m141108_iid=1800121_0     AGAATTGCATCAAATGAAATATTCAAATTAAAAAAGAAAATACTTTTGATTCCAATCTTA   7886
m141031_iid=147540_0      AGAATTGCATCAAATGAAATATTCAAATTAAAAAAGAAAATACTTTTGATTCCAATCTTA   8238
m150507_iid=3674924_0     AGAATTGCATCAAATGAAATATTCAAAAA--AAAAGAAAATACTTTTGATTCCAATCTTA   8388
m150503_iid=2923751_0     AGAATTGCATCAAATGAAATATTCAAATTAAAAAAGAAAATACTTTTGATTCCAATCTTA   8215
m141031_iid=115118_0      AGAATTGCATCAAATGAAATATTCAAATTAAAAAAGAAAATACTTTTGATTCCAATCTTA   7085
m150430_iid=2722448_0     AGAATTGCATCAAATGAAATATTCAAATTAAAAAAGAAAATACTTTTGATTCCAATCTTA   7010
m150507_iid=3102647_0     AGAATTGCATCAAATGAAATATTCAAATTAAAAAAGAAAATACTTTTGATTCCAATCTTA   5316
m141108_iid=1811866_0     AGAATTGCATCAAATGAAATATTCAAATTAAAAAAGAAAATACTTTTGATTCCAATCTTA   7200
m150501_iid=2948495_0     AGAATTGCATCAAATGAAATATTCAAATTAAAAAAGAAAATACTTTTGATTCCAATCTTA   6614
m150504_iid=3238195_0     AGAATTGCATCAAATGAAATATTCAAATTAAAAAAGAAAATACTTTTGATTCCAATCTTA   8954
m150503_iid=3388511_0     AGAATTGCATCAAATGAAATATTCAAATTAAAAAAGAAAATACTTTTGATTCCAATCTTA   6650
m150505_iid=3777611_0     AGAATTGCATCAAATGAAATATTCAAATTAAAAAAGAAAATACTTTTGATTCCAATCTTA   8076
m150430_iid=2438833_0     AGAATTGCATCAAATGAAATATTCAAATTAAAAAAGAAAATACTTTTGATTCCAATCTTA   8409
m141108_iid=1698969_0     AGAATTGCATCAAATGAAATATTCAAATTAAAAAAGAAAATACTTTTGATTCCAATCTTA   9543
```

```
m150430_iid=3059190_0      AGAATTGCATCAAATGAAATATTCAAATTAAAAAAGAAAATACTTTTGATTCCAATCTTA   5265
m141107_iid=1383067_0      AGAATTGCATCAAATGAAATATTCAAATTAAAAAAGAAAATACTTTTGATTCCAATCTTA   5264
m141101_iid=597008_0       AGAATTGCATCAAATGAAATATTCAAATTAAAAAAGAAAATACTTTTGATTCCAATCTTA   2334
m150506_iid=2596496_0      AGAATTGCATCAAATGAAATATTCAAATTAAAAAAGAAAATACTTTTGATTCCAATCTTA   9891
m141102_iid=910051_0       ------------------------------------------------------------   7428
m150505_iid=3784017_0      AGAATTGCATCAAATGAAATATTCAAATTAAAAAAGAAAATACTTTTGATTCCAATCTTA   8191
m141101_iid=682151_0       AGAATTGCATCAAATGAAATATTCAAATTAAAAAAGAAAATACTTTTGATTCCAATCTTA   7259
m150501_iid=3748029_0      AGAATTGCATCAAATGAAATATTCAAATTAAAAAAGAAAATACTTTTGATTCCAATCTTA   7803
m150504_iid=2876272_0      AGAATTGCATCAAATGAAATATTCAAATTAAAAAAGAAAATACTTTTGATTCCAATCTTA   9659
NC_042820.1                AGAATTGCATCAAATGAAATATTCAAATTAAAAAAGAAAATACTTTTGATTCCAATCTTA   9910
m141102_iid=1228825_0      AGAATTGCATCAAATGAAATATTCAAATTAAAAAA-AAAATACTTTTGATTCCAATCTTA   9733
m150504_iid=2876274_0      AGAATTGCATCAAATGAAATATTCAAATTAAAAAAGAAAATACTTTTGATTCCAATCTTA   9731
m141030_iid=80036_0        AGAATTGCATCAAATGAAATATTCAAATTAAAAAAGAAAATACTTTTGATTCCAATCTTA   9500
m150506_iid=2596494_0      AGAATTGCATCAAATGAAATATTCAAATTAAAAAAGAAAATACTTTTGATTCCAATCTTA   9899
m150503_iid=2526862_0      AGAATTGCATCAAATGAAATATTCAAATTAAAAAAGAAAATACTTTTGATTCCAATCTTA   9584
m150506_iid=2567000_0      AGAATTGCATCAAATGAAATATTCAAATTAAAAAAGAAAATACTTTTGATTCCAATCTTA   7538
m150507_iid=3073271_0      AGAATTGCATCAAATGAAATATTCAAATTAAAAAAGAAAATACTTTTGATTCCAATCTTA   8838
m150504_iid=3156087_0      AGAATTGCATCAAATGAAATATTCAAATTAAAAAAGAAAATACTTTTGATTCCAATCTTA   8664
m150505_iid=2498502_0      AGAATTGCATCAAATGAAATATTCAAATTAAAAAAGAAAATACTTTTGATTCCAATCTTA   6314
m141102_iid=754227_0       AGAATTGCATCAAATGAAATATTCAAATTAAAAAAGAAAATACTTTTGATTCCAATCTTA   8876
m150507_iid=3469514_0      AGAATTGCATCAAATGAAATATTCAAATTAAAAAAGAAAATACTTTTGATTCCAATCTTA   9434
Rdo_MitoGenome             AGAATTGCATCAAATGAAATATTCAAATTAAAAAAGAAAATACTTTTGATTCCAATCTTA   9910
m141101_iid=614933_0       AGAATTGCATCAAATGAAATATTCAAATTAAAAAAGAAAATACTTTTGATTCCAATCTTA   8459
```

nad6

```
m141031_iid=347546_0       AGAGCATTAA-TTTTATCAAACAATAAAATTAAAGAAATAAACCTCGTAGAAATAAGAAA   4664
m150506_iid=3127414_0      AGAGCATTAA-TTTTATCAAACAATAAAATTAAAGAAATAAACCTCGTAGAAATAAGAAA   6406
m141102_iid=1245517_0      ------------------------------------------------------------   7295
m141107_iid=1405727_0      AGAGCATTAAT-TTTATCAAACAATAAAATTAAAGAAATAAACCTCGTAGAAATAAGAAA   5235
m141102_iid=987614_0       AGAGCATTAATTTTATCAAACAATAAAATTAAAGAAATAAACCTCGTAGAAATAAGAAA   8227
m141108_iid=1701429_0      AGAGCATTAA-TTTTATCAAACAATAAAATTAAAGAAATAAACCTCGTAGAAAA-AGAAA   4973
m141102_iid=987286_0       AGAGCATTAA-TTTTATCAAACAATAAAATTAAAGAAATAAACCTCGTAGAAATAAGAAA   6193
m150430_iid=3272125_0      AGAGCATTAA-TTTTATCAAACAATAAAATTAAAGAAATAAACCTCGTAGAAATAAGAAA   7704
m141102_iid=754225_0       AGAGCATTAA-TTTTATCAAACAATAAAATTAAAGAAATAAACCTCGTAGAAAAAATAATA   8878
m141107_iid=1409934_0      AGAGCATTAA-TTTTATCAAACAATAAAATTAAAGAAATAAACCTCGTAGAAATAAGAAA   9421
m150503_iid=3416920_0      AGAGCATTAA-TTTTATCAAACAATAAAATTAAAGAAATAAACCTCGTAGAAATAAGAAA   8132
m141108_iid=1800121_0      AGAGCATTAA-TTTTATCAAACAATAAAATTAAAGAAATAAACCTCGTAGAAATAAGAAA   7945
m141031_iid=147540_0       AGAGCATTAA-TTTTATCAAACAATAAAATTAAAGAAATAAACCTCGTAGAAATAAGAAA   8297
m150507_iid=3674924_0      AGAGCATTAA-TTTTATCAAACAATAAAATTAAAGAAATAAACCTCGTAGAAATAAGAAA   8447
m150503_iid=2923751_0      AGAGCATTAA-TTTTATCAAACAATAAAATTAAAGAAATAAACCTCGTAGAAATAAGAAA   8274
m141031_iid=115118_0       AGAGCATTAA-TTTTATCAAACAATAAAATTAAAGAAATAAACCTCGTAGAAATAAGAAA   7144
m150430_iid=2722448_0      AGAGCATTAA-TTTTATCAAACAATAAAATTAAAGAAATAAACCTCGTAGAAATAAGAAA   7069
m150507_iid=3102647_0      AGAGCATTAA-TTTTATCAAACAATAAAATTAAAGAAATAAACCTCGTAGAAATAAGAAA   5375
m141108_iid=1811866_0      AGAGCATTAA-TTTTATCAAACAATAAAATTAAAGAAATAAACCTCGTAGAAATAAGAAA   7259
m150501_iid=2948495_0      AGAGCATTAA-TTTTATCAAACAATAAAATTAAAGAAATAAACCTCGTAGAAATAAGAAA   6673
m150504_iid=3238195_0      AGAGCATTAA-TTTTATCAAACAATAAAATTAAAGAAATAAACCTCGTAGAAAAAG-AAA   9012
m150503_iid=3388511_0      AGAGCATTAA-TTTTATCAAACAATAAAATTAAAGAAATAAACCTCGTAGAAATAAGAAA   6709
m150505_iid=3777611_0      AGAGCATTAA-TTTTATCAAACAATAAAATTAAAGAAATAAACCTCGTAGAAATAAGAAA   8135
m150430_iid=2438833_0      AGAGCATTAA-TTTTATCAAACAATAAAATTAAAGAAATAAACCTCGTAGAAATAAGAAA   8468
m141108_iid=1698969_0      AGAGCATTAA-TTTTATCAAACAATAAAATTAAAGAAATAAACCTCGTAGAAATAAGAAA   9602
m150430_iid=3059190_0      AGAGCATTAA-TTTTATCAAACAATAAAATTAAAGAAATAAACCTCGTAGAAATAAGAAA   5324
m141107_iid=1383067_0      AGAGCATTAA-TTTTATCAAACAATAAAATTAAAGAAATAAACCTCGTAGAAATAAGAAA   5323
m141101_iid=597008_0       AGAGCATTAA-TTTTATCAAACAATAAAATTAAAGAAATAAACCTCGTAGAAATAAGAAA   2393
m150506_iid=2596496_0      AGAGCATTAA-TTTTATCAAACAATAAAATTAAAGAAATAAACCTCGTAGAAATAAGAAA   9950
m141102_iid=910051_0       ------------------------------------------------------------   7428
m150505_iid=3784017_0      AGAGCATTAA-TTTTATCAAACAATAAAATTAAAGAAATAAACCTCGTAGAAATAAGAAA   8250
m141101_iid=682151_0       AGAGCATTAA-TTTTATCAAACAATAAAATTAAAGAAATAAACCTCGTAGAAATAAGAAA   7318
m150501_iid=3748029_0      AGAGCATTAA-TTTTATCAAACAATAAAATTAAAGAAATAAACCTCGTAGAAATAAGAAA   7862
m150504_iid=2876272_0      AGAGCATTAA-TTTTATCAAACAATAAAATTAAAGAAATAAACCTCGTAGAAATAAGAAA   9718
NC_042820.1                AGAGCATTAA-TTTTATCAAACAATAAAATTAAAGAAATAAACCTCGTAGAAATAAGAAA   9969
m141102_iid=1228825_0      AGAGCATTAA-TTTTATCAAACAATAAAATTAAAGAAATAAACCTCGTAGAAATAAGAAA   9792
m150504_iid=2876274_0      AGAGCATTAA-TTTTATCAAACAATAAAATTAAAGAAATAAACCTCGTAGAAATAAGAAA   9790
m141030_iid=80036_0        AGAGCATTAA-TTTTATCAAACAATAAAATTAAAGAAATAAACCTCGTAGAAATAAGAAA   9559
m150506_iid=2596494_0      AGAGCATTAA-TTTTATCAAACAATAAAATTAAAGAAATAAACCTCGTAGAAATAAGAAA   9958
m150503_iid=2526862_0      AGAGCATTAA-TTTTATCAAACAATAAAATTAAAGAAATAAACCTCGTAGAAATAAGAAA   9643
m150506_iid=2567000_0      AGAGCATTAA-TTTTATCAAACAATAAAATTAAAGAAATAAACCTCGTAGAAATAAGAAA   7597
m150507_iid=3073271_0      AGAGCATTAA-TTTTATCAAACAATAAAATTAAAGAAATAAACCTCGTAGAAATAAGAAA   8897
m150504_iid=3156087_0      AGAGCATTAA-TTTTATCAAACAATAAAATTAAAGAAATAAACCTCGTAGAAATAAGAAA   8723
m150505_iid=2498502_0      AGAGCATTAA-TTTTATCAAACAATAAAATTAAAGAAATAAACCTCGTAGAAATAAGAAA   6373
m141102_iid=754227_0       AGAGCATTAA-TTTTATCAAACAATAAAATTAAAGAAATAAACCTCGTAGAAATAAGAAA   8935
m150507_iid=3469514_0      AGAGCATTAA-TTTTATCAAACAATAAAATTAAAGAAATAAACCTCGTAGAAATAAGAAA   9493
```

```
Rdo_MitoGenome            AGAGCATTAA-TTTTATCAAACAATAAAATTAAAGAAATAAACCTCGTAGAAATAAGAAA   9969
m141101_iid=614933_0      AGAGCATTAA-TTTTATCAAACAATAAAATTAAAGAAATAAACCTCGTAGAAATAAGAAA   8518
```

nad6

```
m141031_iid=347546_0      TATAGAAAAAAAATTTAAACTTC-AAATTATCCATAACTAAATTCTTAAATG-AACCTATA   4722
m150506_iid=3127414_0     TATAGAAAAAAAATTTAAACTTC-AAATTATCCATAACTAAATTCTTAAATG-AACCTATA   6464
m141102_iid=1245517_0     ------------------------------------------------------------   7295
m141107_iid=1405727_0     TATAGAAAA--ATTTAAACTTCA-AATTATCCATAACTAAATTCTTAAATGA-ACCTATA   5291
m141102_iid=987614_0      TATAGAAAAAAAATTTAAACTTCAAATTATCCATAACTAAATTCTTAAATGAAACCTATA   8287
m141108_iid=1701429_0     TATAGAAAAAAAATTTAAACTTC-AAATTATCCATAACTAAATTCTTAAATG-AACCTATA   5031
m141102_iid=987286_0      TATAGAAAAAAAATTTAAACTTC-AAATTATCCATAACTAAATTCTTAAATG-AACCTATA   6251
m150430_iid=3272125_0     TATAGAAAAAAAATTTAAACTTC-AAATTATCCATAACTAAATTCTTAAATG-AACCTATA   7762
m141102_iid=754225_0      --TAGAAAAAAAATT-------T-AAACTTTCCATAACTAAATTCTTAAATG-AACCTATA   8927
m141107_iid=1409934_0     TATAGAAAAAAAATTTAAACTTC-AAATTATCCATAACTAAATTCTTAAATG-AACCTATA   9479
m150503_iid=3416920_0     TATAGAAAAAAAATTTAAACTTC-AAATTATCCATAACTAAATTCTTAAATG-AACCTATA   8190
m141108_iid=1800121_0     TATAGAAAA-AATTTAAACTTC-AAATTATCCATAACTAAATTCTTAAATG-AACCTATA   8002
m141031_iid=147540_0      TATAGAAAAAAAATTTAAACTTC-AAATTATCCATAACTAAATTCTTAAATG-AACCTATA   8355
m150507_iid=3674924_0     TATAGAAAAAAAATTTAAACTTC-AAATTATCCATAACTAAATTCTTAAATG-AACCTATA   8505
m150503_iid=2923751_0     TATAGAAAAAAAATTTAAACTTC-AAATTATCCATAACTAAATTCTTA-ATG-AACCTATA   8331
m141031_iid=115118_0      TATAGAAAAAAAATTTAAACTTC-AAATTATCCATAACTAAATTCTTAAATG-AACCTATA   7202
m150430_iid=2722448_0     TATAGAAAAAAAATTTAAACTTC-AAATTATCCATAACTAAATTCTTAAATG-AACCTATA   7127
m150507_iid=3102647_0     TATAGAAAAAAAATTTAAACTTC-AAATTATCCATAACTAAATTCTTAAATG-AACCTATA   5433
m141108_iid=1811866_0     TATAGAAAAAAAATTTAAACTTC-AAATTATCCATAACTAAATTCTTAAATG-AACCTATA   7317
m150501_iid=2948495_0     TATAGAAAAAAAATTTAAACTTC-AAATTATCCATAACTAAATTCTTAAATG-AACCTATA   6731
m150504_iid=3238195_0     TATAGA-AAAAAATTTAAACTTC-AAATTATCCATAACTAAATTCTTAAATG-AACCTATA   9069
m150503_iid=3388511_0     TATAGAAAAAAAATTTAAACTTC-AAATTATCCATAACTAAATTCTTAAATG-AACCTATA   6767
m150505_iid=3777611_0     TATAGAAAAAAAATTTAAACTTC-AAATTATCCATAACTAAATTCTTAAATG-AACCTATA   8193
m150430_iid=2438833_0     TATAGAAAAAAAATTTAAACTTC-AAATTATCCATAACTAAATTCTTAAATG-AACCTATA   8526
m141108_iid=1698969_0     TATAGAAAAAAAATTTAAACTTC-AAATTATCCATAACTAAATTCTTAAATG-AACCTATA   9660
m150430_iid=3059190_0     TATAGAAAAAAAATTTAAACTTC-AAATTATCCATAACTAAATTCTTAAATG-AACCTATA   5382
m141107_iid=1383067_0     TATAGAAAAAAAATTTAAACTTC-AAATTATCCATAACTAAATTCTTAAATG-AACCTATA   5381
m141101_iid=597008_0      TATAGAAAAAAAATTTAAACTTC-AAATTATCCATAACTAAATTCTTAAATG-AACCTATA   2451
m150506_iid=2596496_0     TATAGAAAAAAAATTTAAACTTC-AAATTATCCATAACTAAATTCTTAAATG-AACCTATA   10008
m141102_iid=910051_0      ------------------------------------------------------------   7428
m150505_iid=3784017_0     TATAGAAAAAAAATTTAAACTTC-AAATTATCCATAACTAAATTCTTAAATG-AACCTATA   8308
m141101_iid=682151_0      TATAGAAAAAAAATTTAAACTTC-AAATTATCCATAACTAAATTCTTAAATG-AACCTATA   7376
m150501_iid=3748029_0     TATAGAAAAAAAATTTAAACTTC-AAATTATCCATAACTAAATTCTTAAATG-AACCTATA   7920
m150504_iid=2876272_0     TATAGAAAAAAAATTTAAACTTC-AAATTATCCATAACTAAATTCTTAAATG-AACCTATA   9776
NC_042820.1               TATAGAAAAAAAATTTAAACTTC-AAATTATCCATAACTAAATTCTTAAATG-AACCTATA   10027
m141102_iid=1228825_0     TATAGAAAAAAAATTTAAACTTC-AAATTATCCATAACTAAATTCTTAAATG-AACCTATA   9850
m150504_iid=2876274_0     TATAGAAAAAAAATTTAAACTTC-AAATTATCCATAACTAAATTCTTAAATG-AACCTATA   9848
m141030_iid=80036_0       TATAGAAAAAAAATTTAAACTTC-AAATTATCCATAACTAAATTCTTAAATG-AACCTATA   9617
m150506_iid=2596494_0     TATAGAAAAAAAATTTAAACTTC-AAATTATCCATAACTAAATTCTTAAATG-AACCTATA   10016
m150503_iid=2526862_0     TATAGAAAAAAAATTTAAACTTC-AAATTATCCATAACTAAATTCTTAAATG-AACCTATA   9701
m150506_iid=2567000_0     TATAGAAAAAAAATTTAAACTTC-AAATTATCCATAACTAAATTCTTAAATG-AACCTATA   7655
m150507_iid=3073271_0     TATAGAAAAAAAATTTAAACTTC-AAATTATCCATAACTAAATTCTTAAATG-AACCTATA   8955
m150504_iid=3156087_0     TATAGAAAAAAAATTTAAACTTC-AAATTATCCATAACTAAATTCTTAAATG-AACCTATA   8781
m150505_iid=2498502_0     TATAGAAAAAAAATTTAAACTTC-AAATTATCCATAACTAAATTCTTAAATG-AACCTATA   6431
m141102_iid=754227_0      TATAGAAAAAAAATTTAAACTTC-AAATTATCCATAACTAAATTCTTAAATG-AACCTATA   8993
m150507_iid=3469514_0     TATAGAAAAAAAATTTAAACTTC-AAATTATCCATAACTAAATTCTTAAATG-AACCTATA   9551
Rdo_MitoGenome            TATAGAAAAAAAATTTAAACTTC-AAATTATCCATAACTAAATTCTTAAATG-AACCTATA   10027
m141101_iid=614933_0      TATAGAAAAAAAATTTAAACTTC-AAATTATCCATAACTAAATTCTTAAATG-AACCTATA   8576
```

nad6

```
m141031_iid=347546_0      ATAAA--TTTCATTAACAATTATAATTTACTTATTAA-TTACATTAATTGTAATTGT---   4776
m150506_iid=3127414_0     ATAAATTTTACATTAACAATTATAATTTACTTATTAA-TTACATTAATTGTAATTGT---   6520
m141102_iid=1245517_0     ------------------------------------------------------------   7295
m141107_iid=1405727_0     ATAAATTTTACATTAACAATTATAATTTACTTATTAA-TTACATTAATTGTAATTGT---   5347
m141102_iid=987614_0      ATAAATTTTACATTAACAATTATAATTTACTTATTAATTTACATTAATTGTAATTGTTTG   8347
m141108_iid=1701429_0     ATAAATTTTACATTAACAATTATAATTTACTTATTAA-TTACATTAATTGTAATTGT---   5087
m141102_iid=987286_0      ATAAATTTTACATTAACAATTATAATTTACTTATTAA-TTACATTAATTGTAATTGT---   6307
m150430_iid=3272125_0     ATAAATTTTACATTAACAATTATAA-TTACTTATTAA-TTACATTAATTGTAATTGT---   7817
m141102_iid=754225_0      ATAAATTTTACATTAACAATTATAATTTACTTATTAA-TTACATTAATTGTAATTGT---   8983
m141107_iid=1409934_0     ATAAATTTTACATTAACAATTATAATTTACTTATTAA-TTACATTAATTGTAATTGT---   9535
m150503_iid=3416920_0     ATAAATTTTACATTAACAATTATAATTTACTTATTAA-TTACATTAATTGTAATTGT---   8246
m141108_iid=1800121_0     ATAAATTTTACATTAACAATTATAATTTACTTATTAA-TTACATTAATTGTAATTGT---   8058
m141031_iid=147540_0      ATAAATTTTACATTAACAATTATAATTTACTTATTAA-TTACATTAATTGTAATTGT---   8411
m150507_iid=3674924_0     ATAAATTTTACATTAACAATTATAATTTACTTATTAA-TTACATTAATTGTAATTGT---   8561
m150503_iid=2923751_0     ATAAATTTTACATTAACAATTATAATTTACTTATTAA-TTACATTAATTGTAATTGT---   8387
m141031_iid=115118_0      ATAAATTTTACATTAACAATTATAATTTACTTATTAA-TTACATTAATTGTAATTGT---   7258
m150430_iid=2722448_0     ATAAATTTTACATTAACAATTATAATTTACTTATTAA-TTACATTAATTGTAATTGT---   7183
```

```
m150507_iid=3102647_0     ATAAATTTTACATTAACAATTATAATTTACTTATTAA-TTACATTAATTGTAATTGT---   5489
m141108_iid=1811866_0     ATAAATTTTACATTAACAATTATAATTTACTTATTAA-TTACATTAATTGTAATTGT---   7373
m150501_iid=2948495_0     ATAAATTTTACATTAACAATTATAATTTACTTATTAA-TTACATTAATTGTAATTGT---   6787
m150504_iid=3238195_0     ATAAATTTTACATTAACAATTATAATTTACTTATTAA-TTACATTAATTGTAATTGT---   9125
m150503_iid=3388511_0     ATAAATTTTACATTAACAATTATAATTTACTTATTAA-TTACATTAATTGTAATTGT---   6823
m150505_iid=3777611_0     ATAAATTTTACATTAACAATTATAATTTACTTATTAA-TTACATTAATTGTAATTGT---   8249
m150430_iid=2438833_0     ATAAATTTTACATTAACAATTATAATTTACTTATTAA-TTACATTAATTGTAATTGT---   8582
m141108_iid=1698969_0     ATAAATTTTACATTAACAATTATAATTTACTTATTAA-TTACATTAATTGTAATTGT---   9716
m150430_iid=3059190_0     ATAAATTTTACATTAACAATTATAATTTACTTATTAA-TTACATTAATTGTAATTGT---   5438
m141107_iid=1383067_0     ATAAATTTTACATTAACAATTATAATTTACTTATTAA-TTACATTAATTGTAATTGT---   5437
m141101_iid=597008_0      ATAAATTTTACATTAACAATTATAATTTACTTATTAA-TTACATTAATTGTAATTGT---   2507
m150506_iid=2596496_0     ATAAATTTTACATTAACAATTATAATTTACTTATTAA-TTACATTAATTGTAATTGT---   10064
m141102_iid=910051_0      ------------------------------------------------------------   7428
m150505_iid=3784017_0     ATAAATTTTACATTAACAATTATAATTTACTTATTAA-TTACATTAATTGTAATTGT---   8364
m141101_iid=682151_0      ATAAATTTTACATTAACAATTATAATTTACTTATTAA-TTACATTAATTGTAATTGT---   7432
m150501_iid=3748029_0     ATAAATTTTACATTAACAATTATAATTTACTTATTAA-TTACATTAATTGTAATTGT---   7976
m150504_iid=2876272_0     ATAAATTTTACATTAACAATTATAATTTACTTATTAA-TTACATTAATTGTAATTGT---   9832
NC_042820.1               ATAAATTTTACATTAACAATTATAATTTACTTATTAA-TTACATTAATTGTAATTGT---   10083
m141102_iid=1228825_0     ATAAATTTTACATTAACAATTATAATTTACTTATTAA-TTACATTAATTGTAATTGT---   9906
m150504_iid=2876274_0     ATAAATTTTACATTAACAATTATAATTTACTTATTAA-TTACATTAATTGTAATTGT---   9904
m141030_iid=80036_0       ATAAATTTTACATTAACAATTATAATTTACTTATTAA-TTACATTAATTGTAATTGT---   9673
m150506_iid=2596494_0     ATAAATTTTACATTAACAATTATAATTTACTTATTAA-TTACATTAATTGTAATTGT---   10072
m150503_iid=2526862_0     ATAAATTTTACATTAACAATTATAATTTACTTATTAA-TTACATTAATTGTAATTGT---   9757
m150506_iid=2567000_0     ATAAATTTTACATTAACAATTATAATTTACTTATTAA-TTACATTAATTGTAATTGT---   7711
m150507_iid=3073271_0     ATAAATTTTACATTAACAATTATAATTTACTTATTAA-TTACATTAATTGTAATTGT---   9011
m150504_iid=3156087_0     ATAAATTTTACATTAACAATTATAATTTACTTATTAA-TTACATTAATTGTAATTGT---   8837
m150505_iid=2498502_0     ATAAATTTTACATTAACAATTATAATTTACTTATTAA-TTACATTAATTGTAATTGT---   6487
m141102_iid=754227_0      ATAAATTTTACATTAACAATTATAATTTACTTATTAA-TTACATTAATTGTAATTGT---   9049
m150507_iid=3469514_0     ATAAATTTTACATTAACAATTATAATTTACTTATTAA-TTACATTAATTGTAATTGT---   9607
Rdo_MitoGenome            ATAAATTTTACATTAACAATTATAATTTACTTATTAA-TTACATTAATTGTAATTGT---   10083
m141101_iid=614933_0      ATAAATTTTACATTAACAATTATAATTTACTTATTAA-TTACATTAATTGTAATTGT---   8632
```

nad6

```
m141031_iid=347546_0      TAAAATCACCAACTTAAATGAAGGACCATTGCGGCAAAA-TTCTAATGAAAAAACAACTA   4835
m150506_iid=3127414_0     TAAAATCACCAACTTAAATGAAGGACCATTGCGGCAAAAATTCTAATGAAAAAACAACTA   6580
m141102_iid=1245517_0     ------------------------------------------------------------   7295
m141107_iid=1405727_0     TAAAATCACCAACTTAAATGAAGGACCATTGCGGCAAAAATTCTAATGAAAAAACAACTA   5407
m141102_iid=987614_0      TAAAATCACCAACTTAAATGAAGGACCATTGCGGCAAAAATTCTAATGAAAAAACAACTA   8407
m141108_iid=1701429_0     TAAAATCA------AACTTAAATGACCATTGCGGCAAAAATTCTAATGAAAAAAC---TA   5138
m141102_iid=987286_0      TAAAATCACCAACTTAAATGAAGGACCATTGCGGCAAAAT-TCTAATGA-AAAACAACTA   6365
m150430_iid=3272125_0     TAAAATCACCAACTTAAATGAAGGACCATTGCGGCAAAAATTCTAATGAAAAAACAACTA   7877
m141102_iid=754225_0      TAAAATCACCAACTTAAATGAAGGACCATTGCGGCAAAAATTCTAATGAAAAAACAACTA   9043
m141107_iid=1409934_0     TAAAATCACCAACTTAAATGAAGGACCATTGCGGCAAAAATTCTAATGAAAAAACAACTA   9595
m150503_iid=3416920_0     TAAAATCACCAACTTAAATGAAGGACCATTGCGGCAAAAATTCTAATGAAAAAACAACTA   8306
m141108_iid=1800121_0     TAAAATCACCAACTTAAATGAAGGACCATTGCGGCAAAA-TTCTAATGAAAA---AACTA   8114
m141031_iid=147540_0      TAAAATCACCAACTTAAATGAAGGACCATTGCGGCAAAAATTCTAATGAAAAAACAACTA   8471
m150507_iid=3674924_0     TAAAATCACCAACTTAAATGAAGGACCATTGCGGCAAAAATTCTAATGAAAAAACAACTA   8621
m150503_iid=2923751_0     TAAAATCACCAACTTAAATGAAGGACCATTGCGGCAAAAATTCTAATGAAAAAACAACTA   8447
m141031_iid=115118_0      TAAAATCACCAACTTAAATGAAGGACCATTGCGGCAAAAATTCTAATGAAAAAACAACTA   7318
m150430_iid=2722448_0     TAAAATCACCAACTTAAATGAA-GACCATTGCGGCAAAAATTCTAATGAAAAAACAACTA   7242
m150507_iid=3102647_0     TAAAATCACCAACTTAAATGAAGGACCATTGCGGCAAAAATTCTAATGAAAAAACAACTA   5549
m141108_iid=1811866_0     TAAAATCACCAACTTAAATGAAGGACCATTGCGGCAAAAATTCTAATGAAAAAACAACTA   7433
m150501_iid=2948495_0     TAAAATCACCAACTTAAATGAAGGACCATTGCGGCAAAAATTCTAATGAAAAAACAACTA   6847
m150504_iid=3238195_0     TAAAATCACCAACTTAAATGAA-GACCATTGCGGCAAAAATTCTAATGAAAAAACAACTA   9184
m150503_iid=3388511_0     TAAAATCACCAACTTAAATGAAGGACCATTGCGGCAAAAATTCTAATGAAAAAACAACTA   6883
m150505_iid=3777611_0     TAAAATCACCAACTTAAATGAAGGACCATTGCGGCAAAAATTCTAATGAAAAAACAACTA   8309
m150430_iid=2438833_0     TAAAATCACCAACTTAAATGAAGGACCATTGCGGCAAAAATTCTAATGAAAAAACAACTA   8642
m141108_iid=1698969_0     TAAAATCACCAACTTAAATGAAGGACCATTGCGGCAAAAATTCTAATGAAAAAACAACTA   9776
m150430_iid=3059190_0     TAAAATCACCAACTTAAATGAAGGACCATTGCGGCAAAAATTCTAATGAAAAAACAACTA   5498
m141107_iid=1383067_0     TAAAATCACCAACTTAAATGAAGGACCATTGCGGCAAAAATTCTAATGAAAAAACAACTA   5497
m141101_iid=597008_0      TAAAATCACCAACTTAAATGAAGGACCATTGCGGCAAAAATTCTAATGAAAAAACAACTA   2567
m150506_iid=2596496_0     TAAAATCACCAACTTAAATGAAGGACCATTGCGGCAAAAATTCTAATGAAAAAACAACTA   10124
m141102_iid=910051_0      ------------------------------------------------------------   7428
m150505_iid=3784017_0     TAAAATCACCAACTTAAATGAAGGACCATTGCGGCAAAAATTCTAATGAAAA---AACTA   8421
m141101_iid=682151_0      TAAAATCACCAACTTAAATGAAGGACCATTGCGGCAAAAATTCTAATGAAAAAACAACTA   7492
m150501_iid=3748029_0     TAAAATCACCAACTTAAATGAAGGACCATTGCGGCAAAAATTCTAATGAAAAAACAACTA   8036
m150504_iid=2876272_0     TAAAATCACCAACTTAAATGAAGGACCATTGCGGCAAAAATTCTAATGAAAAAACAACTA   9892
NC_042820.1               TAAAATCACCAACTTAAATGAAGGACCATTGCGGCAAAAATTCTAATGAAAAAACAACTA   10143
m141102_iid=1228825_0     TAAAATCACCAACTTAAATGAAGGACCATTGCGGCAAAAATTCTAATGAAAAAACAACTA   9966
m150504_iid=2876274_0     TAAAATCACCAACTTAAATGAAGGACCATTGCGGCAAAAATTCTAATGAAAAAACAACTA   9964
m141030_iid=80036_0       TAAAATCACCAACTTAAATGAAGGACCATTGCGGCAAAAATTCTAATGAAAAAACAACTA   9733
```

```
m150506_iid=2596494_0       TAAAATCACCAACTTAAATGAAGGACCATTGCGGCAAAAATTCTAATGAAAAAACAACTA   10132
m150503_iid=2526862_0       TAAAATCACCAACTTAAATGAAGGACCATTGCGGCAAAAATTCTAATGAAAAAACAACTA   9817
m150506_iid=2567000_0       TAAAATCACCAACTTAAATGAAGGACCATTGCGGCAAAAATTCTAATGAAAAAACAACTA   7771
m150507_iid=3073271_0       TAAAATCACCAACTTAAATGAAGGACCATTGCGGCAAAAATTCTAATGAAAAAACAACTA   9071
m150504_iid=3156087_0       TAAAATCACCAACTTAAATGAAGGACCATTGCGGCAAAAATTCTAATGAAAAAACAACTA   8897
m150505_iid=2498502_0       TAAAATCACCAACTTAAATGAAGGACCATTGCGGCAAAAATTCTAATGAAAAAACAACTA   6547
m141102_iid=754227_0        TAAAATCACCAACTTAAATGAAGGACCATTGCGGCAAAAATTCTAATGAAAAAACAACTA   9109
m150507_iid=3469514_0       TAAAATCACCAACTTAAATGAAGGACCATTGCGGCAAAAATTCTAATGAAAAAACAACTA   9667
Rdo_MitoGenome              TAAAATCACCAACTTAAATGAAGGACCATTGCGGCAAAAATTCTAATGAAAAAACAACTA   10143
m141101_iid=614933_0        TAAAATCACCAACTTAAATGAAGGACCATTGCGGCAAAAATTCTAATGAAAAAACAACTA   8692
```

nad6 *cytB*

```
m141031_iid=347546_0        ATAAAAATTTCACCTGTAACTAAAAATTATTAATAA-TGCATTAATTGATTTACCAACCC   4894
m150506_iid=3127414_0       ATA-AAATTTCACCTGTAACTAAA--TTATTAATAA-TGCATTAATTGATTTACCAACCC   6636
m141102_iid=1245517_0       ------------------------------------------------------------   7295
m141107_iid=1405727_0       ATAAAAATTTCACCTGTAACTAAAATTATTAAT--AATGCATTAATTGATTTACCAACCC   5465
m141102_iid=987614_0        ATAAAAATTTCACCTGTAACTAAAATTATTAATAA--TGCATTAATTGATTTACCAACCC   8465
m141108_iid=1701429_0       ATAAAAATTTCACCTGTAACTAAAATTATTAATAATATGCAT---TATATTTACCAACCC   5195
m141102_iid=987286_0        ATAAAAATTTCACCTGTAACTAAAATTATTAATAA--TGCATTAATTGATTTACCAACCC   6423
m150430_iid=3272125_0       ATAAAAATTTCACCTGTAACTAAAATTATTAATAA--TGCATTAATTGATTTACC-ACCC   7934
m141102_iid=754225_0        ATAAAAATTTCACCTGTAACTAAAATTATTAATAA--TGCATTAATTGATTTACCAACCC   9101
m141107_iid=1409934_0       ATAAAAATTTCACCTGTAACTAAAATTATTAATAA--TGCATTAATTGATTTACCAACCC   9653
m150503_iid=3416920_0       ATAA-AATTTCACCTGTAACTAAAATTATTAATAA--TGCATTAATTGATTTACCAACCC   8363
m141108_iid=1800121_0       ATAAAAATTTCACCTGTAACTAAAATTATTAATAA--TGCATTAATTGATTTACCAACCC   8172
m141031_iid=147540_0        ATAAAAATTTCACCTGTAACTAAAATTATTAATAA--TGCATTAATTGATTTACCAACCC   8529
m150507_iid=3674924_0       ATAAAAATTTCACCTGTAACTAAAATTATTAATAA--TGCATTAATTGATTTACCAACCC   8679
m150503_iid=2923751_0       ATAAAAATTTCACCTGTAACTAAAATTATTAATAA--TGCATTAATTGATTTACCAACCC   8505
m141031_iid=115118_0        ATAAAAATTTCACCTGTAACTAAAATTATTAATAA--TGCATTAATTGATTTACCAACCC   7376
m150430_iid=2722448_0       ATAAAAATTTCACCTGTAACTAAAATTATTAATAA--TGCATTAATTGATTTACCAACCC   7300
m150507_iid=3102647_0       ATAAAAATTTCACCTGTAACTAAAATTATTAATAA--TGCATTAATTGATTTACCAACCC   5607
m141108_iid=1811866_0       ATAAAAATTTCACCTGTAACTAAAATTATTAATAA--TGCATTAATTGATTTACCAACCC   7491
m150501_iid=2948495_0       ATAAAAATTTCACCTGTAACTAAAATTATTAATAA--TGCATTAATTGATTTACCAACCC   6905
m150504_iid=3238195_0       ATAAAAATTTCACCTGTAACTAAAATTATTAATAA--TGCATTAATTGATTTACCAACCC   9242
m150503_iid=3388511_0       ATAAAAATTTCACCTGTAACTAAAATTATTAATAA--TGCATTAATTGATTTACCAACCC   6941
m150505_iid=3777611_0       ATAAAAATTTCACCTGTAACTAAAATTATTAATAA--TGCATTAATTGATTTACCAACCC   8367
m150430_iid=2438833_0       ATAAAAATTTCACCTGTAACTAAAATTATTAATAA--TGCATTAATTGATTTACCAA-CC   8699
m141108_iid=1698969_0       ATAAAAATTTCACCTGTAACTAAAATTATTAATAA--TGCATTAATTGATTTACCAACCC   9834
m150430_iid=3059190_0       ATAAAAATTTCACCTGTAACTAAAATTATTAATAA--TGCATTAATTGATTTACCAACCC   5556
m141107_iid=1383067_0       ATAAAAATTTCACCTGTAACTAAAATTATTAATAA--TGCATTAATTGATTTACCAACCC   5555
m141101_iid=597008_0        ATAAAAATTTCACCTGTAACTAAAATTATTAATAA--TGCATTAATTGATTTACCAACCC   2625
m150506_iid=2596496_0       ATAAAAATTTCACCTGTAACTAAAATTATTAATAA--TGCATTAATTGATTTACCAACCC   10182
m141102_iid=910051_0        ------------------------------------------------------------   7428
m150505_iid=3784017_0       ATAAAAATTTCACCTGTAACTAAAATTATTAATAA--TGCATTAATTGATTTACCAACCC   8479
m141101_iid=682151_0        ATAAAAATTTCACCTGTAACTAAAATTATTAATAA--TGCATTAATTGATTTACCAACCC   7550
m150501_iid=3748029_0       ATAAAAATTTCACCTGTAACTAAAATTATTAATAA--TGCATTAATTGATTTACCAACCC   8094
m150504_iid=2876272_0       ATAAAAATTTCACCTGTAACTAAAATTATTAATAA--TGCATTAATTGATTTACCAACCC   9950
NC_042820.1                 ATAAAAATTTCACCTGTAACTAAAATTATTAATAA--TGCATTAATTGATTTACCAACCC   10201
m141102_iid=1228825_0       ATAAAAATTTCACCTGTAACTAAAATTATTAATAA--TGCATTAATTGATTTACCAACCC   10024
m150504_iid=2876274_0       ATAAAAATTTCACCTGTAACTAAAATTATTAATAA--TGCATTAATTGATTTACCAACCC   10022
m141030_iid=80036_0         ATAAAAATTTCACCTGTAACTAAAATTATTAATAA--TGCATTAATTGATTTACCAACCC   9791
m150506_iid=2596494_0       ATAAAAATTTCACCTGTAACTAAAATTATTAATAA--TGCATTAATTGATTTACCAACCC   10190
m150503_iid=2526862_0       ATAAAAATTTCACCTGTAACTAAAATTATTAATAA--TGCATTAATTGATTTACCAACCC   9875
m150506_iid=2567000_0       ATAAAAATTTCACCTGTAACTAAAATTATTAATAA--TGCATTAATTGATTTACCAACCC   7829
m150507_iid=3073271_0       ATAAAAATTTCACCTGTAACTAAAATTATTAATAA--TGCATTAATTGATTTACCAACCC   9129
m150504_iid=3156087_0       ATAAAAATTTCACCTGTAACTAAAATTATTAATAA--TGCATTAATTGATTTACCAACCC   8955
m150505_iid=2498502_0       ATAAAAATTTCACCTGTAACTAAAATTATTAATAA--TGCATTAATTGATTTACCAACCC   6605
m141102_iid=754227_0        ATAAAAATTTCACCTGTAACTAAAATTATTAATAA--TGCATTAATTGATTTACCAACCC   9167
m150507_iid=3469514_0       ATAAAAATTTCACCTGTAACTAAAATTATTAATAA--TGCATTAATTGATTTACCAACCC   9725
Rdo_MitoGenome              ATAAAAATTTCACCTGTAACTAAAATTATTAATAA--TGCATTAATTGATTTACCAACCC   10201
m141101_iid=614933_0        ATAAAAATTTCACCTGTAACTAAAATTATTAATAA--TGCATTAATTGATTTACCAACCC   8750
```

cytB

```
m141031_iid=347546_0        CATCAAACATCAGAATTTGATGAAATATAGGATCAATTCTTGGACTATGCTTAATT-ATT   4953
m150506_iid=3127414_0       CATCAAACATCAGAATTTGATGAAATATAGGATCAATTCTTGGACTATGCTTAATT-ATT   6695
m141102_iid=1245517_0       ------------------------------------------------------------   7295
m141107_iid=1405727_0       CATCAAACATCAGAATTTGATGAAATATAGGATCAATTCTTGGACTATGCTTAATT-ATT   5524
m141102_iid=987614_0        CATCAAACATCAGAATTTGATGAAATATAGGATCAATTCTTGGACTATGCTTAATT-ATT   8524
m141108_iid=1701429_0       CATCAAACATCAGAATTTGATGAAATATAGGATCAATTCTTGGACTATGCTTAATT-ATT   5254
m141102_iid=987286_0        CATCAAACATCAGAATTTGATGAAATATAGGATCAATTCTTGGACTATGCTTAATT-ATT   6482
m150430_iid=3272125_0       CATCAAACATCAGAATTTGATGAAATATAGGATCAATTCTTGGACTATGCTTAATT-ATT   7993
m141102_iid=754225_0        CATCAAA---CAGAATTTGATGAAATATAGGATCAATTCTTGGACTATGCTTAATT-ATT   9157
```

m141107_iid=1409934_0	CATCAAACATCAGAATTTGATGAAATATAGGATCAATTCTTGGACTATGCTTAATT-ATT	9712
m150503_iid=3416920_0	CATCAAACATCAGAATTTGATGAAATATAGGATCAATTCTTGGACTATGCTTAATTATTT	8423
m141108_iid=1800121_0	CATCAAACATCAGAATTTGATGAAATATAGGATCAATTCTTGGACTATGCTTAATT-ATT	8231
m141031_iid=147540_0	CATCAAACATCAGAATTTGATGAAATATAGGATCAATTCTTGGACTATGCTTAATT-ATT	8588
m150507_iid=3674924_0	CATCAAACATCAGAATTTGATGAAATATAGGATCAATTCTTGGACTATGCTTAATT-ATT	8738
m150503_iid=2923751_0	CATCAAACATCAGAATTTGATGAAATATAGGATCAATTCTTGGACTATGCTTAATT-ATT	8564
m141031_iid=115118_0	CATCAAACATCAGAATTTGATGAAATATAGGATCAATTCTTGGACTATGCTTAATT-ATT	7435
m150430_iid=2722448_0	CATCAAACATCAGAATTTGATGAAATATAGGATCAATTCTTGGACTATGCTTAATT-ATT	7359
m150507_iid=3102647_0	CATCAAACATCAGAATTTGATGAAATATAGGATCAATTCTTGGACTATGCTTAATT-ATT	5666
m141108_iid=1811866_0	CATCAAACATCAGAATTTGATGAAATATAGGATCAATTCTTGGACTATGCTTAATT-ATT	7550
m150501_iid=2948495_0	CATCAAACATCAGAATTTGATGAAATATAGGATCAATTCTTGGACTATGCTTAATT-ATT	6964
m150504_iid=3238195_0	CATCAAACATCAGAATTTGATGAAATATAGGATCAATTCTTGGACTATGCTTAATT-ATT	9301
m150503_iid=3388511_0	CATCAAACATCAGAATTTGATGAAATATAGGATCAATTCTTGGACTATGCTTAATT-ATT	7000
m150505_iid=3777611_0	CATCAAACATCAGAATTTGATGAAATATAGGATCAATTCTTGGACTATGCTTAATT-ATT	8426
m150430_iid=2438833_0	CATCAAACATCAGAATTTGATGAAATATAGGATCAATTCTTGGACTATGCTTAATT-ATT	8758
m141108_iid=1698969_0	CATCAAACATCAGAATTTGATGAAATATAGGATCAATTCTTGGACTATGCTTAATT-ATT	9893
m150430_iid=3059190_0	CATCAAACATCAGAATTTGATGAAATATAGGATCAATTCTTGGACTATGCTTAATT-ATT	5615
m141107_iid=1383067_0	CATCAAACATCAGAATTTGATGAAATATAGGATCAATTCTTGGACTATGCTTAATT-ATT	5614
m141101_iid=597008_0	CATCAAACATCAGAATTTGATGAAATATAGGATCAATTCTTGGACTATGCTTAATT-ATT	2684
m150506_iid=2596496_0	CATCAAACATCAGAATTTGATGAAATATAGGATCAATTCTTGGACTATGCTTAATT-ATT	10241
m141102_iid=910051_0	--	7428
m150505_iid=3784017_0	CATCAAACATCAGAATTTGATGAAATATAGGATCAATTCTTGGACTATGCTTAATT-ATT	8538
m141101_iid=682151_0	CATCAAACATCAGAATTTGATGAAATATAGGATCAATTCTTGGACTATGCTTAATT-ATT	7609
m150501_iid=3748029_0	CATCAAACATCAGAATTTGATGAAATATAGGATCAATTCTTGGACTATGCTTAATT-ATT	8153
m150504_iid=2876272_0	CATCAAACATCAGAATTTGATGAAATATAGGATCAATTCTTGGACTATGCTTAATT-ATT	10009
NC_042820.1	CATCAAACATCAGAATTTGATGAAATATAGGATCAATTCTTGGACTATGCTTAATT-ATT	10260
m141102_iid=1228825_0	CATCAAACATCAGAATTTGATGAAATATAGGATCAATTCTTGGACTATGCTTAATT-ATT	10083
m150504_iid=2876274_0	CATCAAACATCAGAATTTGATGAAATATAGGATCAATTCTTGGACTATGCTTAATT-ATT	10081
m141030_iid=80036_0	CATCAAACATCAGAATTTGATGAAATATAGGATCAATTCTTGGACTATGCTTAATT-ATT	9850
m150506_iid=2596494_0	CATCAAACATCAGAATTTGATGAAATATAGGATCAATTCTTGGACTATGCTTAATT-ATT	10249
m150503_iid=2526862_0	CATCAAACATCAGAATTTGATGAAATATAGGATCAATTCTTGGACTATGCTTAATT-ATT	9934
m150506_iid=2567000_0	CATCAAACATCAGAATTTGATGAAATATAGGATCAATTCTTGGACTATGCTTAATT-ATT	7888
m150507_iid=3073271_0	CATCAAACATCAGAATTTGATGAAATATAGGATCAATTCTTGGACTATGCTTAATT-ATT	9188
m150504_iid=3156087_0	CATCAAACATCAGAATTTGATGAAATATAGGATCAATTCTTGGACTATGCTTAATT-ATT	9014
m150505_iid=2498502_0	CATCAAACATCAGAATTTGATGAAATATAGGATCAATTCTTGGACTATGCTTAATT-ATT	6664
m141102_iid=754227_0	CATCAAACATCAGAATTTGATGAAATATAGGATCAATTCTTGGACTATGCTTAATT-ATT	9226
m150507_iid=3469514_0	CATCAAACATCAGAATTTGATGAAATATAGGATCAATTCTTGGACTATGCTTAATT-ATT	9784
Rdo_MitoGenome	CATCAAACATCAGAATTTGATGAAATATAGGATCAATTCTTGGACTATGCTTAATT-ATT	10260
m141101_iid=614933_0	CATCAAACATCAGAATTTGATGAAATATAGGATCAATTCTTGGACTATGCTTAATT-ATT	8809

cytB

m141031_iid=347546_0	CAAATCATTACCGGAATA-TTTCTAACAATACACTACACCCCAAATACAGAAATAGCCTT	5012
m150506_iid=3127414_0	CAAATCATTACCGGAATA-TTTCTAACAATACACTACACCCCAAATACAGAAATAGCCTT	6754
m141102_iid=1245517_0	--	7295
m141107_iid=1405727_0	CAAATCATTACCGGAATA-TTTCTAACAATACACTACACCC-AAATACAGAAATAGCCTT	5582
m141102_iid=987614_0	CAAATCATTACCGGAATATTTTCTAACAATACACTACACCCCAAATACAGAAATAG-CTT	8583
m141108_iid=1701429_0	CAAATCATTACCGGAATA-TTTCTAACAATACACTACACCCCAAATACAGAAATAGCCTT	5313
m141102_iid=987286_0	CAAATCATTACCGGAATA-TTTCTAACAATACACTACACCC-AAATACAGAAATAGCCTT	6540
m150430_iid=3272125_0	CAAATCATTACCGGAATA-TTTCTAACAATACACTACACCCCAAATACAGAAATAGCCTT	8052
m141102_iid=754225_0	CAAATCATTACCGGAATA-TTTCTAACAATACACTACACCCCAAATACAGAAATAGCCTT	9216
m141107_iid=1409934_0	CAAATCATTACCGGAATA-TTTCTAACAATACACTACACCCCAAATACAGAAATAGCCTT	9771
m150503_iid=3416920_0	CAAATCATTACCGGAATA-TTTCTAACAATACACTACACCCCAAATACAGAAATAGCCTT	8482
m141108_iid=1800121_0	CAAATCATTACCGGAATA-TTTCTAACAATACACTACACCC-AAATACAGAAATAGCCTT	8289
m141031_iid=147540_0	CAAATCATTACCGGAATA-TTTCTAACAATACACTACACCCCAAATACAGAAATAGCCTT	8647
m150507_iid=3674924_0	CAAATCATTACCGGAATA-TTTCTAACAATACACTACACCCCAAATACAGAAATAGCCTT	8797
m150503_iid=2923751_0	CAAATCATTACCGGAATA-TTTCTAACAATACACTACACCC-AAATACAGAAATAGCCTT	8622
m141031_iid=115118_0	CAAATCATTACCGGAATA-TTTCTAACAATACACTACACCCCAAATACAGAAATAGCCTT	7494
m150430_iid=2722448_0	CAAATCATTACCGGAATA-TTTCTAACAATACACTACACCCCAAATACAGAAATAGCCTT	7418
m150507_iid=3102647_0	CAAATCATTACCGGAATA-TTTCTAACAATACACTACACCCCAAATACAGAAATAGCCTT	5725
m141108_iid=1811866_0	CAAATCATTACCGGAATA-TTTCTAACAATACACTACACCCCAAATACAGAAATAGCCTT	7609
m150501_iid=2948495_0	CAAATCATTACCGGAATA-TTTCTAACAATACACTACACCCCAAATACAGAAATAGCCTT	7023
m150504_iid=3238195_0	CAAATCATTACCGGAATA-TTTCTAACAATACACTACACCCCAAATACAGAAATAGCCTT	9360
m150503_iid=3388511_0	CAAATCATTACCGGAATA-TTTCTAACAATACACTACACCCCAAATACAGAAATAGCCTT	7059
m150505_iid=3777611_0	CAAATCATTACCGGAATA-TTTCTAACAATACACTACACCCCAAATACAGAAATAGCCTT	8485
m150430_iid=2438833_0	CAAATCATTACCGGAATA-TTTCTAACAATACACTACACCCCAAATACAGAAATAGCCTT	8817
m141108_iid=1698969_0	CAAATCATTACCGGAATA-TTTCTAACAATACACTACACCCCAAATACAGAAATAGCCTT	9952
m150430_iid=3059190_0	CAAATCATTACCGGAATA-TTTCTAACAATACACTACACCCCAAATACAGAAATAGCCTT	5674
m141107_iid=1383067_0	CAAATCATTACCGGAATA-TTTCTAACAATACACTACACCCCAAATACAGAAATAGCCTT	5673
m141101_iid=597008_0	CAAATCATTACCGGAATA-TTTCTAACAATACACTACACCCCAAATACAGAAATAGCCTT	2743
m150506_iid=2596496_0	CAAATCATTACCGGAATA-TTTCTAACAATACACTACACCCCAAATACAGAAATAGCCTT	10300
m141102_iid=910051_0	--	7428

```
m150505_iid=3784017_0    CAAATCATTACCGGAATA-TTTCTAACAATACACTACACCCCAAATACAGAAATAGCCTT   8597
m141101_iid=682151_0     CAAATCATTACCGGAATA-TTTCTAACAATACACTACACCCCAAATACAGAAATAGCCTT   7668
m150501_iid=3748029_0    CAAATCATTACCGGAATA-TTTCTAACAATACACTACACCCCAAATACAGAAATAGCCTT   8212
m150504_iid=2876272_0    CAAATCATTACCGGAATA-TTTCTAACAATACACTACACCCCAAATACAGAAATAGCCTT   10068
NC_042820.1              CAAATCATTACCGGACTA-TTTCTAACAATACACTACACCCCAAATACAGAAATAGCCTT   10319
m141102_iid=1228825_0    CAAATCATTACCGGAATA-TTTCTAACAATACACTACACCCCAAATACAGAAATAGCCTT   10142
m150504_iid=2876274_0    CAAATCATTACCGGAATA-TTTCTAACAATACACTACACCCCAAATACAGAAATAGCCTT   10140
m141030_iid=80036_0      CAAATCATTACCGGAATA-TTTCTAACAATACACTACACCCCAAATACAGAAATAGCCTT   9909
m150506_iid=2596494_0    CAAATCATTACCGGAATA-TTTCTAACAATACACTACACCCCAAATACAGAAATAGCCTT   10308
m150503_iid=2526862_0    CAAATCATTACCGGAATA-TTTCTAACAATACACTACACCCCAAATACAGAAATAGCCTT   9993
m150506_iid=2567000_0    CAAATCATTACCGGAATA-TTTCTAACAATACACTACACCCCAAATACAGAAATAGCCTT   7947
m150507_iid=3073271_0    CAAATCATTACCGGAATA-TTTCTAACAATACACTACACCCCAAATACAGAAATAGCCTT   9247
m150504_iid=3156087_0    CAAATCATTACCGGAATA-TTTCTAACAATACACTACACCCCAAATACAGAAATAGCCTT   9073
m150505_iid=2498502_0    CAAATCATTACCGGAATA-TTTCTAACAATACACTACACCCCAAATACAGAAATAGCCTT   6723
m141102_iid=754227_0     CAAATCATTACCGGAATA-TTTCTAACAATACACTACACCCCAAATACAGAAATAGCCTT   9285
m150507_iid=3469514_0    CAAATCATTACCGGAATA-TTTCTAACAATACACTACACCCCAAATACAGAAATAGCCTT   9843
Rdo_MitoGenome           CAAATCATTACCGGAATA-TTTCTAACAATACACTACACCCCAAATACAGAAATAGCCTT   10319
m141101_iid=614933_0     CAAATCATTACCGGAATA-TTTCTAACAATACACTACACCCCAAATACAGAAATAGCCTT   8868
```

cytB

```
m141031_iid=347546_0     CAACAGAGTAGTTCACATTTG-CGAGACGTAAATTATGGATG-AATAATCCGAACTATTC   5070
m150506_iid=3127414_0    CAACAGAGTAGTTCACATTTGCCGAGACGTAAATTATGGATG-AATAATCCGAACTATTC   6813
m141102_iid=1245517_0    ------------------------------------------------------------   7295
m141107_iid=1405727_0    CAACAGAGTAGTTCACATTTGCCGAGACGTAAATTATGGATGA-ATAATCCGAACTATTC   5641
m141102_iid=987614_0     CAACAGAGTAGTTCACATTTGCCGAGACGTAAATTATGGATGAAATAATCCGAACTATTC   8643
m141108_iid=1701429_0    CAACAGAGTAGTTCACATTTGCCGAGACGTAAATTATGGATG-AATAATCCGAACTATTC   5372
m141102_iid=987286_0     CAACAGAGTAGTTCACATTTGCCGAGACGTAAATTATGGATG-AATAATCCGAACTATTC   6599
m150430_iid=3272125_0    CAACAGAGTAGTTCACATTTGCCGAGAT--AAATTATGGATG-AATAATCCGAACTATTC   8109
m141102_iid=754225_0     CAACAGAGTAGTTCACATTTGCCGAGACGTAAATTATGGATG-AATAATCCGAACTATTC   9275
m141107_iid=1409934_0    CAACAGAGTAGTTCACATTTGCCGAGACGTAAATTATGGATG-AATAATCCGAACTATTC   9830
m150503_iid=3416920_0    CAACAGAGTAGTTCACATTTGCCGAGACGTAAATTATGGATG-AATAATCCGAACTATTC   8541
m141108_iid=1800121_0    CAACAGAGTAGTTCACATTTGCC-AGACGTAAATTATGGATG-AATAATCCGAACTATTC   8347
m141031_iid=147540_0     CAACAGAGTAGTTCACATTTGCCGAGACGTAAATTATGGATG-AATAATCCGAACTATTC   8706
m150507_iid=3674924_0    CAACAGAGTAGTTCACATTTGCCGAGACGTAAATTATGGATG-AATAATCCGAACTATTC   8856
m150503_iid=2923751_0    CAACAGAGTAGTTCACATTTGCCGAGACGTAAATTATGGATG-AATAATCCGAACTATTC   8681
m141031_iid=115118_0     CAACAGAGTAGTTCACATTTGCCGAGACGTAAATTATGGATG-AATAATCCGAACTATTC   7553
m150430_iid=2722448_0    CAACAGAGTAGTTCACATTTGCCGAGACGTAAATTATGGATG-AATAATCCGAACTATTC   7477
m150507_iid=3102647_0    CAACAGAGTAGTTCACATTTGCCGAGACGTAAATTATGGATG-AATAATCCGAACTATTC   5784
m141108_iid=1811866_0    CAACAGAGTAGTTCACATTTGCCGAGACGTAAATTATGGATG-AATAATCCGAACTATTC   7668
m150501_iid=2948495_0    CAACAGAGTAGTTCACATTTGCCGAGACGTAAATTATGGATG-AATAATCCGAACTATTC   7082
m150504_iid=3238195_0    CAACAGAGTAGTTCACATTTGCCGAGACGTAAATTATGGATG-AATAATCCGAACTATTC   9419
m150503_iid=3388511_0    CAACAGAGTAGTTCACATTTGCCGAGACGTAAATTATGGATG-AATAATCCGAACTATTC   7118
m150505_iid=3777611_0    CAACAGAGTAGTTCACATTTGCCGAGACGTAAATTATGGATG-AATAATCCGAACTATTC   8544
m150430_iid=2438833_0    CAACAGAGTAGTTCACATTTGCCGAGACGTAAATTATGGATG-AATAATCCGAACTATTC   8876
m141108_iid=1698969_0    CAACAGAGTAGTTCACATTTGCCGAGACGTAAATTATGGATG-AATAATCCGAACTATTC   10011
m150430_iid=3059190_0    CAACAGAGTAGTTCACATTTGCCGAGACGTAAATTATGGATG-AATAATCCGAACTATTC   5733
m141107_iid=1383067_0    CAACAGAGTAGTTCACATTTGCCGAGACGTAAATTATGGATG-AATAATCCGAACTATTC   5732
m141101_iid=597008_0     CAACAGAGTAGTTCACATTTGCCGAGACGTAAATTATGGATG-AATAATCCGAACTATTC   2802
m150506_iid=2596496_0    CAACAGAGTAGTTCACATTTGCCGAGACGTAAATTATGGATG-AATAATCCGAACTATTC   10359
m141102_iid=910051_0     ------------------------------------------------------------   7428
m150505_iid=3784017_0    CAACAGAGTAGTTCACATTTGCCGAGACGTAAATTATGGATG-AATAATCCGAACTATTC   8656
m141101_iid=682151_0     CAACAGAGTAGTTCACATTTGCCGAGACGTAAATTATGGATG-AATAATCCGAACTATTC   7727
m150501_iid=3748029_0    CAACAGAGTAGTTCACATTTGCCGAGACGTAAATTATGGATG-AATAATCCGAACTATTC   8271
m150504_iid=2876272_0    CAACAGAGTAGTTCACATTTGCCGAGACGTAAATTATGGATG-AATAATCCGAACTATTC   10127
NC_042820.1              CAACAGAGTAGTTCACATTTGCCGAGACGTAAATTATGGATG-AATAATCCGAACTATTC   10378
m141102_iid=1228825_0    CAACAGAGTAGTTCACATTTGCCGAGACGTAAATTATGGATG-AATAATCCGAACTATTC   10201
m150504_iid=2876274_0    CAACAGAGTAGTTCACATTTGCCGAGACGTAAATTATGGATG-AATAATCCGAACTATTC   10199
m141030_iid=80036_0      CAACAGAGTAGTTCACATTTGCCGAGACGTAAATTATGGATG-AATAATCCGAACTATTC   9968
m150506_iid=2596494_0    CAACAGAGTAGTTCACATTTGCCGAGACGTAAATTATGGATG-AATAATCCGAACTATTC   10367
m150503_iid=2526862_0    CAACAGAGTAGTTCACATTTGCCGAGACGTAAATTATGGATG-AATAATCCGAACTATTC   10052
m150506_iid=2567000_0    CAACAGAGTAGTTCACATTTGCCGAGACGTAAATTATGGATG-AATAATCCGAACTATTC   8006
m150507_iid=3073271_0    CAACAGAGTAGTTCACATTTGCCGAGACGTAAATTATGGATG-AATAATCCGAACTATTC   9306
m150504_iid=3156087_0    CAACAGAGTAGTTCACATTTGCCGAGACGTAAATTATGGATG-AATAATCCGAACTATTC   9132
m150505_iid=2498502_0    CAACAGAGTAGTTCACATTTGCCGAGACGTAAATTATGGATG-AATAATCCGAACTATTC   6782
m141102_iid=754227_0     CAACAGAGTAGTTCACATTTGCCGAGACGTAAATTATGGATG-AATAATCCGAACTATTC   9344
m150507_iid=3469514_0    CAACAGAGTAGTTCACATTTGCCGAGACGTAAATTATGGATG-AATAATCCGAACTATTC   9902
Rdo_MitoGenome           CAACAGAGTAGTTCACATTTGCCGAGACGTAAATTATGGATG-AATAATCCGAACTATTC   10378
m141101_iid=614933_0     CAACAGAGTAGTTCACATTTGCCGAGACGTAAATTATGGATG-AATAATCCGAACTATTC   8927
```

cytB

```
m141031_iid=347546_0     ATGCTAATGGAGCATCAAT-ATTCTTTATTTGTATT-TATATACACATTGGACGAGGACT   5128
```

```
m150506_iid=3127414_0      ATGCTAATGGAGCATCAAT-ATTCTTTATTTGTATTTAATATACACATTGGACGAGGACT   6872
m141102_iid=1245517_0      ------------------------------------------------------------   7295
m141107_iid=1405727_0      ATGCTAATGGAGCATCAATA-TTCTTTATTTGTAT-TTATATACACATTGGACGAGGACT   5699
m141102_iid=987614_0       ATGCTAATGGAGCATCAAT-ATTCTTTATTTGTAT-TTATATACACATTGGACGAGGACT   8701
m141108_iid=1701429_0      ATGCTAATGGAGCATCAATAT-TCTTTATTTGTA-TTTATATACACATTGGACGAGGACT   5430
m141102_iid=987286_0       ATGCTAATGGAGCATCAATAT-TCTTTATTTGTA-TTTATATACACATTGGACGAGGACT   6657
m150430_iid=3272125_0      ATGCTAATGGAGCATCAATAT-TCTTTATTTGTA-TTTATATACACATTGGACGAGGACT   8167
m141102_iid=754225_0       ATGCTAATGGAGCATCAATAT-TCTTTATTTGTA-TTTATATACACATTGGACGAGGACT   9333
m141107_iid=1409934_0      ATGCTAATGGAGCATCAATAT-TCTTTATTTGTA-TTTATATACACATTGGACGAGGACT   9888
m150503_iid=3416920_0      ATGCTAATGGAGCATCAATAT-TCTTTATTTGTAT-TTATATACACATTGGACGAGGACT   8599
m141108_iid=1800121_0      ATGCTAATGGAGCATCAATAT-TCTTTATTTGTATTTATAATACACATTGGACGAGGACT   8406
m141031_iid=147540_0       ATGCTAATGGAGCATCAAT-ATTCTTTATTTGTAT-TTATATACACATTGGACGAGGACT   8764
m150507_iid=3674924_0      ATGCTAATGGAGCATCAATA-TTCTTTATTTGTAT-TTATATACACATTGGACGAGGACT   8914
m150503_iid=2923751_0      ATGCTAATGGAGCATCAAT--TTCTTTATTTGTAT-TTATATACACATTGGACGAGGACT   8738
m141031_iid=115118_0       ATGCTAATGGAGCATCAATA-TTCTTTATTTGTAT-TTATATACACATTGGACGAGGACT   7611
m150430_iid=2722448_0      ATGCTAATGGAGCATCAATA-TTCTTTATTTGTAT-TTATATACACATTGGACGAGGACT   7535
m150507_iid=3102647_0      ATGCTAATGGAGCATCAATA-TTCTTTATTTGTAT-TTATATACACATTGGACGAGGACT   5842
m141108_iid=1811866_0      ATGCTAATGGAGCATCAATAT-TCTTTATTTGTAT-TTATATACACATTGGACGAGGACT   7726
m150501_iid=2948495_0      ATGCTAATGGAGCATCAATATTC--TTATTTGTAT-TTATATACACATTGGACGAGGACT   7139
m150504_iid=3238195_0      ATGCTAATGGAGCATCAAT-ATTCTTTATTTGTAT-TTATATACACATTGGACGAGGACT   9477
m150503_iid=3388511_0      ATGCTAATGGAGCATCAAT-ATTCTTTATTTGTAT-TTATATACACATTGGACGAGGACT   7176
m150505_iid=3777611_0      ATGCTAATGGAGCATCAAT-ATTCTTTATTTGTAT-TTATATACACATTGGACGAGGACT   8602
m150430_iid=2438833_0      ATGCTAATGGAGCATCAAT-ATTCTTTATTTGTAT-TTATATACACATTGGACGAGGACT   8934
m141108_iid=1698969_0      ATGCTAATGGAGCATCAAT-ATTCTTTATTTGTAT-TTATATACACATTGGACGAGGACT   10069
m150430_iid=3059190_0      ATGCTAATGGAGCATCAAT-ATTCTTTATTTGTAT-TTATATACACATTGGACGAGGACT   5791
m141107_iid=1383067_0      ATGCTAATGGAGCATCAAT-ATTCTTTATTTGTAT-TTATATACACATTGGACGAGGACT   5790
m141101_iid=597008_0       ATGCTAATGGAGCATCAAT-ATTCTTTATTTGTAT-TTATATACACATTGGACGAGGACT   2860
m150506_iid=2596496_0      ATGCTAATGGAGCATCAAAAATTCTTTATTTGTAT-TTATATACACATTGGACGAGGACT   10418
m141102_iid=910051_0       ------------------------------------------------------------   7428
m150505_iid=3784017_0      ATGCTAATGGAGCATCAAT-ATTCTTTATTTGTAT-TTATATACACATTGGACGAGGACT   8714
m141101_iid=682151_0       ATGCTAATGGAGCATCAAT-ATTCTTTATTTGTAT-TTATATACACATTGGACGAGGACT   7785
m150501_iid=3748029_0      ATGCTAATGGAGCATCAAT-ATTCTTTATTTGTAT-TTATATACACATTGGACGAGGACT   8329
m150504_iid=2876272_0      ATGCTAATGGAGCATCAAT-ATTCTTTATTTGTAT-TTATATACACATTGGACGAGGACT   10185
NC_042820.1                ATGCTAATGGAGCATCAAT-ATTCTTTATTTGTAT-TTATATACACATTGGACGAGGACT   10436
m141102_iid=1228825_0      ATGCTAATGGAGCATCAAT-ATTCTTTATTTGTAT-TTATATACACATTGGACGAGGACT   10259
m150504_iid=2876274_0      ATGCTAATGGAGCATCAAT-ATTCTTTATTTGTAT-TTATATACACATTGGACGAGGACT   10257
m141030_iid=80036_0        ATGCTAATGGAGCATCAAT-ATTCTTTATTTGTAT-TTATATACACATTGGACGAGGACT   10026
m150506_iid=2596494_0      ATGCTAATGGAGCATCAAT-ATTCTTTATTTGTAT-TTATATACACATTGGACGAGGACT   10425
m150503_iid=2526862_0      ATGCTAATGGAGCATCAAT-ATTCTTTATTTGTAT-TTATATACACATTGGACGAGGACT   10110
m150506_iid=2567000_0      ATGCTAATGGAGCATCAAT-ATTCTTTATTTGTAT-TTATATACACATTGGACGAGGACT   8064
m150507_iid=3073271_0      ATGCTAATGGAGCATCAAT-ATTCTTTATTTGTAT-TTATATACACATTGGACGAGGACT   9364
m150504_iid=3156087_0      ATGCTAATGGAGCATCAAT-ATTCTTTATTTGTAT-TTATATACACATTGGACGAGGACT   9190
m150505_iid=2498502_0      ATGCTAATGGAGCATCAAT-ATTCTTTATTTGTAT-TTATATACACATTGGACGAGGACT   6840
m141102_iid=754227_0       ATGCTAATGGAGCATCAAT-ATTCTTTATTTGTAT-TTATATACACATTGGACGAGGACT   9402
m150507_iid=3469514_0      ATGCTAATGGAGCATCAAT-ATTCTTTATTTGTAT-TTATATACACATTGGACGAGGACT   9960
Rdo_MitoGenome             ATGCTAATGGAGCATCAAT-ATTCTTTATTTGTAT-TTATATACACATTGGACGAGGACT   10436
m141101_iid=614933_0       ATGCTAATGGAGCATCAAT-ATTCTTTATTTGTAT-TTATATACACATTGGACGAGGACT   8985
                                                     cytB
                           ----------------------------------------------------------->

m141031_iid=347546_0       ATATTATGGATCTTACAAAT-AATTCACACATGAATAGTAGGAGTAATCATTTTATTCTT   5187
m150506_iid=3127414_0      ATATTATGGATCTTACAAATTAATTCACACATGAATAGTAGGAGTAATCATTTTATTCTT   6932
m141102_iid=1245517_0      ------------------------------------------------------------   7295
m141107_iid=1405727_0      ATATTATGGATCTTACAAATTAATTCA-ACATGAATAGTAGGAGTAATCATTTTATTCTT   5758
m141102_iid=987614_0       ATATTATGGATCTTACAAATTAATTCACACATGAATAGTAGGAGTAATCATTTTATTCTT   8761
m141108_iid=1701429_0      ATATTATGGATCTTACAAATTAATTCACACATGAATAGTAGGAGTAATCATTTTATTCTT   5490
m141102_iid=987286_0       ATATTATGGATCTTACAAATTAATTCACACATGAATAGTAGGAGTAATCATTTTATTCTT   6717
m150430_iid=3272125_0      ATATTATGGATCTTACAAATTAATTCACACATGAATAGTAGGAGTAATCATTTTATTCTT   8227
m141102_iid=754225_0       ATATTATGGATCTTACAAATTAATTCACACATGAATAGTAGGAGTAATCATTTTATTCTT   9393
m141107_iid=1409934_0      ATATTATGGATCTTACAAATTAATTCACACATGAATAGTAGGAGTAATCATTTTATTCTT   9948
m150503_iid=3416920_0      ATATTATGGATCTTACAAATTAATTCACACATGAATAGTAGGAGTAATCATTTTATTCTT   8659
m141108_iid=1800121_0      ATATTATGGATCTTACAAATTAATTCACACATGAATAGTAGGAGTAATCATTTTATTCTT   8466
m141031_iid=147540_0       ATATTATGGATCTTACAAATTAATTCACACATGAATAGTAGGAGTAATCATTTTATTCTT   8824
m150507_iid=3674924_0      ATATTATGGATCTTACAAATTAATTCACACATGAATAGTAGGAGTAATCATTTTATTCTT   8974
m150503_iid=2923751_0      ATATTATGGATCTTACAAATTAATTCACACATGAATAGTAGGAGTAATCATTTTATTCTT   8798
m141031_iid=115118_0       ATATTATGGATCTTACAAATTAATTCACACATGAATAGTAGGAGTAATCATTTTATTCTT   7671
m150430_iid=2722448_0      ATATTATGGATCTTACAAATTAATTCACACATGAATAGTAGGAGTAATCATTTTATTCTT   7595
m150507_iid=3102647_0      ATATTATGGATCTTACAAATTAATTCACACATGAATAGTAGGAGTAATCATTTTATTCTT   5902
m141108_iid=1811866_0      ATATTATGGATCTTACAAATT--TTCACACATGAATAGTAGGAGTAATCATTTTATTCTT   7784
m150501_iid=2948495_0      ATATTATGGATCTTACAAATTAATTCACACATGAATAGTAGGAGTAATCATTTTATTCTT   7199
m150504_iid=3238195_0      ATATTATGGATCTTACAAATTAATTCACACATGAATAGTAGGAGTAATCATTTTATTCTT   9537
m150503_iid=3388511_0      ATATTATGGATCTTACAAATTAATTCACACATGAATAGTAGGAGTAATCATTTTATTCTT   7236
```

```
m150505_iid=3777611_0       ATATTATGGATCTTACAAATTAATTCACACATGAATAGTAGGAGTAATCATTTTATTCTT   8662
m150430_iid=2438833_0       ATATTATGGATCTTACAAATTAATTCACACATGAATAGTAGGAGTAATCATTTTATTCTT   8994
m141108_iid=1698969_0       ATATTATGGATCTTACAAATTAATTCACACATGAATAGTAGGAGTAATCATTTTATTCTT   10129
m150430_iid=3059190_0       ATATTATGGATCTTACAAATTAATTCACACATGAATAGTAGGAGTAATCATTTTATTCTT   5851
m141107_iid=1383067_0       ATATTATGGATCTTACAAATTAATTCACACATGAATAGTAGGAGTAATCATTTTATTCTT   5850
m141101_iid=597008_0        ATATTATGGATCTTACAAATTAATTCACACATGAATAGTAGGAGTAATCATTTTATTCTT   2920
m150506_iid=2596496_0       ATATTATGGATCTTACAAAT-TATTCACACATGAATAGTAGGAGTAATCATTTTATTCT-   10476
m141102_iid=910051_0        ------------------------------------------------------------   7428
m150505_iid=3784017_0       ATATTATGGATCTTACAAATTAATTCACACATGAATAGTAGGAGTAATCATTTTATTCTT   8774
m141101_iid=682151_0        ATATTATGGATCTTACAAATTAATTCACACATGAATAGTAGGAGTAATCATTTTATTCTT   7845
m150501_iid=3748029_0       ATATTATGGATCTTACAAATTAATTCACACATGAATAGTAGGAGTAATCATTTTATTCTT   8389
m150504_iid=2876272_0       ATATTATGGATCTTACAAATTAATTCACACATGAATAGTAGGAGTAATCATTTTATTCTT   10245
NC_042820.1                 ATATTATGGATCTTACAAATTAATTCACACATGAATAGTAGGAGTAATCATTTTATTCTT   10496
m141102_iid=1228825_0       ATATTATGGATCTTACAAATTAATTCACACATGAATAGTAGGAGTAATCATTTTATTCTT   10319
m150504_iid=2876274_0       ATATTATGGATCTTACAAATTAATTCACACATGAATAGTAGGAGTAATCATTTTATTCTT   10317
m141030_iid=80036_0         ATATTATGGATCTTACAAATTAATTCACACATGAATAGTAGGAGTAATCATTTTATTCTT   10086
m150506_iid=2596494_0       ATATTATGGATCTTACAAATTAATTCACACATGAATAGTAGGAGTAATCATTTTATTCTT   10485
m150503_iid=2526862_0       ATATTATGGATCTTACAAATTAATTCACACATGAATAGTAGGAGTAATCATTTTATTCTT   10170
m150506_iid=2567000_0       ATATTATGGATCTTACAAATTAATTCACACATGAATAGTAGGAGTAATCATTTTATTCTT   8124
m150507_iid=3073271_0       ATATTATGGATCTTACAAATTAATTCACACATGAATAGTAGGAGTAATCATTTTATTCTT   9424
m150504_iid=3156087_0       ATATTATGGATCTTACAAATTAATTCACACATGAATAGTAGGAGTAATCATTTTATTCTT   9250
m150505_iid=2498502_0       ATATTATGGATCTTACAAATTAATTCACACATGAATAGTAGGAGTAATCATTTTATTCTT   6900
m141102_iid=754227_0        ATATTATGGATCTTACAAATTAATTCACACATGAATAGTAGGAGTAATCATTTTATTCTT   9462
m150507_iid=3469514_0       ATATTATGGATCTTACAAATTAATTCACACATGAATAGTAGGAGTAATCATTTTATTCTT   10020
Rdo_MitoGenome              ATATTATGGATCTTACAAATTAATTCACACATGAATAGTAGGAGTAATCATTTTATTCTT   10496
m141101_iid=614933_0        ATATTATGGATCTTACAAATTAATTCACACATGAATAGTAGGAGTAATCATTTTATTCTT   9045
```

cytB →

```
m141031_iid=347546_0        AGTTATAGCAACAGCATTCCTGGGTTACGTTT-TACCATGAGGACAAATATCATTTTGAG   5246
m150506_iid=3127414_0       AGTTATAGCAACAGCATTCCTGGGTTACGTTTTTACCATGAGGACAAATATCATTTTGAG   6992
m141102_iid=1245517_0       ------------------------------------------------------------   7295
m141107_iid=1405727_0       AGTTATAGCAACAGCATTCCTGGGTTACGTTT-TACCATGAGGACAAATATCATTTTGAG   5817
m141102_iid=987614_0        AGTTATAGCAACAGCATTCCTGGGTTACGTTT-TACCATGAGGACAAATATCATTTTGAG   8820
m141108_iid=1701429_0       AGTTATAGCAACAGCATTCCTGGGTTACGTTT-TACCATGAGGACAAATATCATTTTGAG   5549
m141102_iid=987286_0        AGTTATAGCAACAGCATTCCTGGGTTACGTTT-TACCATGAGGACAAA-ATCATTTTGAG   6775
m150430_iid=3272125_0       AGTTATAGCAACAGCATTCCTGGGTTACGTTT-TACCATGAGGACAAATATCATTTTGAG   8286
m141102_iid=754225_0        AGTTATAGCAACAGCATTCCTGGGTTACGTTT-TACCATGAGGACAAATATCATTTTGAG   9452
m141107_iid=1409934_0       AGTTATAGCAACAGCATTCCTGGGTTACGTTT-TACCATGAGGACAAATATCATTTTGAG   10007
m150503_iid=3416920_0       AGTTATAGCAACAGCATTCCTGGGTTACGTTT-TACCATGAGGACAAATATCATTTTGAG   8718
m141108_iid=1800121_0       AGTTATAGCAACAGCATTCCTGGGTTACGTTT-TACCATGAGGACAAATATCATTTTGAG   8525
m141031_iid=147540_0        AGTTATAGCAACAGCATTCCTGGGTTACGTTT-TACCATGAGGACAAATATCATTTTGAG   8883
m150507_iid=3674924_0       AGTTATAGCAACAGCATTCCTGGGTTACGTTT-TACCATGAGGACAAATATCATTTTGAG   9033
m150503_iid=2923751_0       AGTTATAGCAACAGCATTCCTGGGTTACGTTT-TACCATGAGGACAAATATCATTTTGAG   8857
m141031_iid=115118_0        AGTTATAGCAACAGCATTCCTGGGTTACGTTT-TACCATGAGGACAAATATCATTTTGAG   7730
m150430_iid=2722448_0       AGTTATAGCAACAGCATTCCTGGGTTACGTTT-TACCATGAGGACAAATATCATTTTGAG   7654
m150507_iid=3102647_0       AGTTATAGCAACAGCATTCCTGGGTTACGTTT-TACCATGAGGACAAATATCATTTTGAG   5961
m141108_iid=1811866_0       AGTTATAGCAACAGCATTCCTGGGTTACGTTT-TACCATGAGGACAAATATCATTTTGAG   7843
m150501_iid=2948495_0       AGTTATAGCAACAGCATTCCTGGGTTACGTTT-TACCATGAGGACAAATATCATTTTGAG   7258
m150504_iid=3238195_0       AGTTATAGCAACAGCATTCCTGGGTTACGTTT-TACCATGAGGACAAATATCATTTTGAG   9596
m150503_iid=3388511_0       AGTTATAGCAACAGCATTCCTGGGTTACGTTT-TACCATGAGGACAAATATCATTTTGAG   7295
m150505_iid=3777611_0       AGTTATAGCAACAGCATTCCTGGGTTACGTTT-TACCATGAGGACAAATATCATTTTGAG   8721
m150430_iid=2438833_0       AGTTATAGCAACAGCATTCCTGGGTTACGTTT-TACCATGAGGACAAATATCATTTTGAG   9053
m141108_iid=1698969_0       AGTTATAGCAACAGCATTCCTGGGTTACGTTT-TACCATGAGGACAAATATCATTTTGAG   10188
m150430_iid=3059190_0       AGTTATAGCAACAGCATTCCTGGGTTACGTTT-TACCATGAGGACAAATATCATTTTGAG   5910
m141107_iid=1383067_0       AGTTATAGCAACAGCATTCCTGGGTTACGTTT-TACCATGAGGACAAATATCATTTTGAG   5909
m141101_iid=597008_0        AGTTATAGCAACAGCATTCCTGGGTTACGTTT-TACCATGAGGACAAATATCATTTTGAG   2979
m150506_iid=2596496_0       AGTTATAGCAACAGC---------------------------------------------   10491
m141102_iid=910051_0        ------------------------------------------------------------   7428
m150505_iid=3784017_0       AGTTATAGCAACAGCATTCCTGGGTTACGTTT-TACCATGAGGACAAATATCATTTTGAG   8833
m141101_iid=682151_0        AGTTATAGCAACAGCATTCCTGGGTTACGTTT-TACCATGAGGACAAATATCATTTTGAG   7904
m150501_iid=3748029_0       AGTTATAGCAACAGCATTCCTGGGTTACGTTT-TACCATGAGGACAAATATCATTTTGAG   8448
m150504_iid=2876272_0       AGTTATAGCAACAGCATTCCTGGGTTACGTTT-TACCATGAGGACAAATATCATTTTGAG   10304
NC_042820.1                 AATTATAGCAACAGCATTCCTGGGTTACGTTT-TACCATGAGGACAAATATCATTTTGAG   10555
m141102_iid=1228825_0       AGTTATAGCAACAGCATTCCTGGGTTACGTTT-TACCATGAGGACAAATATCATTTTGAG   10378
m150504_iid=2876274_0       AGTTATAGCAACAGCATTCCTGGGTTACGTTT-TACCATGAGGACAAATATCATTTTGAG   10376
m141030_iid=80036_0         AGTTATAGCAACAGCATTCCTGGGTTACGTTT-TACCATGAGGACAAATATCATTTTGAG   10145
m150506_iid=2596494_0       AGTTATAGCAACAGCATTCCTGGGTTACGTTT-TACCATGAGGACAAATATCATTTTGAG   10544
m150503_iid=2526862_0       AGTTATAGCAACAGCATTCCTGGGTTACGTTT-TACCATGAGGACAAATATCATTTTGAG   10229
m150506_iid=2567000_0       AGTTATAGCAACAGCATTCCTGGGTTACGTTT-TACCATGAGGACAAATATCATTTTGAG   8183
m150507_iid=3073271_0       AGTTATAGCAACAGCATTCCTGGGTTACGTTT-TACCATGAGGACAAATATCATTTTGAG   9483
m150504_iid=3156087_0       AGTTATAGCAACAGCATTCCTGGGTTACGTTT-TACCATGAGGACAAATATCATTTTGAG   9309
```

```
m150505_iid=2498502_0      AGTTATAGCAACAGCATTCCTGGGTTACGTTT-TACCATGAGGACAAATATCATTTTGAG   6959
m141102_iid=754227_0       AGTTATAGCAACAGCATTCCTGGGTTACGTTT-TACCATGAGGACAAATATCATTTTGAG   9521
m150507_iid=3469514_0      AGTTATAGCAACAGCATTCCTGGGTTACGTTT-TACCATGAGGACAAATATCATTTTGAG   10079
Rdo_MitoGenome             AGTTATAGCAACAGCATTCCTGGGTTACGTTT-TACCATGAGGACAAATATCATTTTGAG   10555
m141101_iid=614933_0       AGTTATAGCAACAGCATTCCTGGGTTACGTTT-TACCATGAGGACAAATATCATTTTGAG   9104
```

cytB

```
m141031_iid=347546_0       GAGCAACAGTTATCACACAAACCTTTTATACAGCTATTC-ATATGTAGGAA-AATAATTG   5304
m150506_iid=3127414_0      GAGCAACAGTTATCACA--AACCTTT-TATCAGCTATTCCATATGTAGGAAAAATAATTG   7049
m141102_iid=1245517_0      ------------------------------------------------------------   7295
m141107_iid=1405727_0      GAGCAACAGTTATCACA--AACCTTT-TATCAGCTATTCCATATGTAGGAAAAATAATTG   5874
m141102_iid=987614_0       GAGCAACAGTTATCACA--AACCTTT-TATCAGCTATTCCATATGTAGGAAAAATAATTG   8877
m141108_iid=1701429_0      GAGCAACAGTTATCACA--AACCTTT-TATCAGCTATTCCATATGTAGGAAAAATAATTG   5606
m141102_iid=987286_0       GAGCAACAGTTATCACA--AACCTTT-TATCAGCTATTCCATATGTAGGAAAAATAATTG   6832
m150430_iid=3272125_0      GAGCAACAGTTATCACA--AACCTTT-TATCTA-TATTCCATATGTAGGAAAAATAATTG   8342
m141102_iid=754225_0       GAGCAACAGTTATCACA--AACCTTT-TATCAGCTATTCCATATGTAGGAAAAATAATTG   9509
m141107_iid=1409934_0      GAGCAACAGTTATCACA--AACCTTT-TATCAGCTATTCCATATGTAGGAAAAATAATTG   10064
m150503_iid=3416920_0      GAGCAACAGTTATCACA--AACCTTT-TATCAGCTATTCCATATGTAGGAAAAATAATTG   8775
m141108_iid=1800121_0      GAGCAACAGTTATC-CA--AACCTTT-TATCAGCTATTCCATATGTAGGAAAAATAATTG   8581
m141031_iid=147540_0       GAGCAACAGTTATCACA--AACCTTT-TATCAGCTATTCCATATGTAGGAAAAATAATTG   8940
m150507_iid=3674924_0      GAGCAACAGTTATCACA--AACCTTT-TATCAGCTATTCCATATGTAGGAAAAATAATTG   9090
m150503_iid=2923751_0      GAGCAACAGTTATCACA--AACCTTT-TATCAGCTATTCCATATGTAGGAAAAATAATTG   8914
m141031_iid=115118_0       GAGCAACAGTTATCACA--AACCTTT-TATCAGCTATTCCATATGTAGGAAAAATAATTG   7787
m150430_iid=2722448_0      GAGCAACAGTTATCACA--AACCTTT-TATCAGCTATTCCATATGTAGGAAAAATAATTG   7711
m150507_iid=3102647_0      GAGCAACAGTTATCACA--AACCTTT-TATCAGCTATTCCATATGTAGGAAAAATAATTG   6018
m141108_iid=1811866_0      GAGCAACAGTTATCACA--AACCTTT-TATCAGCTATTCCATATGTAGGAAAAATAATTG   7900
m150501_iid=2948495_0      GAGCAACAGTTATCACA--AACCTTT-TATCAGCTATTCCATATGTAGGAAAAATAATTG   7315
m150504_iid=3238195_0      GAGCAACAGTTATCACA--AACCTTT-TATCAGCTATTCCATATGTAGGAAAAATAATTG   9653
m150503_iid=3388511_0      GAGCAACAGTTATCACA--AACCTTT-TATCAGCTATTCCATATGTAGGAAAAATAATTG   7352
m150505_iid=3777611_0      GAGCAACAGTTATCACA--AACCTTT-TATCAGCTATTCCATATGTAGGAAAAATAATTG   8778
m150430_iid=2438833_0      GAGCAACAGTTATCACA--AACCTTT-TATCAGCTATTCCATATGTAGGAAAAATAATTG   9110
m141108_iid=1698969_0      GAGCAACAGTTATCACA--AACCTTT-TATCAGCTATTCCATATGTAGGAAAAATAATTG   10245
m150430_iid=3059190_0      GAGCAACAGTTATCACA--AACCTTT-TATCAGCTATTCCATATGTAGGAAAAATAATTG   5967
m141107_iid=1383067_0      GAGCAACAGTTATCACA--AACCTTT-TATCAGCTATTCCATATGTAGGAAAAATAATTG   5966
m141101_iid=597008_0       GAGCAACAGTTATCACA--AACCTTT-TATCAGCTATTCCATATGTAGGAAAAATAATTG   3036
m150506_iid=2596496_0      ------------------------------------------------------------   10491
m141102_iid=910051_0       ------------------------------------------------------------   7428
m150505_iid=3784017_0      GAGCAACAGTTATCACA--AACCTTT-TATCAGCTATTCCATATGTAGGAAAAATAATTG   8890
m141101_iid=682151_0       GAGCAACAGTTATCACA--AACCTTT-TATCAGCTATTCCATATGTAGGAAAAATAATTG   7961
m150501_iid=3748029_0      GAGCAACAGTTATCACA--AACCTTT-TATCAGCTATTCCATATGTAGGAAAAATAATTG   8505
m150504_iid=2876272_0      GAGCAACAGTTATCACA--AACCTTT-TATCAGCTATTCCATATGTAGGAAAAATAATTG   10361
NC_042820.1                GAGCAACAGTTATCACA--AACCTTT-TATCAGCTATTCCATATGTAGGAAAAATAATTG   10612
m141102_iid=1228825_0      GAGCAACAGTTATCACA--AACCTTT-TATCAGCTATTCCATATGTAGGAAAAATAATTG   10435
m150504_iid=2876274_0      GAGCAACAGTTATCACA--AACCTTT-TATCAGCTATTCCATATGTAGGAAAAATAATTG   10433
m141030_iid=80036_0        GAGCAACAGTTATCACA--AACCTTT-TATCAGCTATTCCATATGTAGGAAAAATAATTG   10202
m150506_iid=2596494_0      GAGCAACAGTTATCACA--AACCTTT-TATCAGCTATTCCATATGTAGGAAAAATAATTG   10601
m150503_iid=2526862_0      GAGCAACAGTTATCACA--AACCTTT-TATCAGCTATTCCATATGTAGGAAAAATAATTG   10286
m150506_iid=2567000_0      GAGCAACAGTTATCACA--AACCTTT-TATCAGCTATTCCATATGTAGGAAAAATAATTG   8240
m150507_iid=3073271_0      GAGCAACAGTTATCACA--AACCTTT-TATCAGCTATTCCATATGTAGGAAAAATAATTG   9540
m150504_iid=3156087_0      GAGCAACAGTTATCACA--AACCTTT-TATCAGCTATTCCATATGTAGGAAAAATAATTG   9366
m150505_iid=2498502_0      GAGCAACAGTTATCACA--AACCTTT-TATCAGCTATTCCATATGTAGGAAAAATAATTG   7016
m141102_iid=754227_0       GAGCAACAGTTATCACA--AACCTTT-TATCAGCTATTCCATATGTAGGAAAAATAATTG   9578
m150507_iid=3469514_0      GAGCAACAGTTATCACA--AACCTTT-TATCAGCTATTCCATATGTAGGAAAAATAATTG   10136
Rdo_MitoGenome             GAGCAACAGTTATCACA--AACCTTT-TATCAGCTATTCCATATGTAGGAAAAATAATTG   10612
m141101_iid=614933_0       GAGCAACAGTTATCACA--AACCTTT-TATCAG---------------------------   9134
```

cytB

```
m141031_iid=347546_0       TAGAATGACTTTGAGGTGGATTTGCTGTAGATAACGCAACATTAAATCGATTCTTTGCAT   5364
m150506_iid=3127414_0      TAGAATGACTTTGAGGTGGATTTGCTGTAGATAACGCAACATTAAATCGATTCTTTGCAT   7109
m141102_iid=1245517_0      ------------------------------------------------------------   7295
m141107_iid=1405727_0      TAGAATGACTTTGAGGTGGATTTGCTGTAGATAACGCAACATTAAATCGATTCTTTGCAT   5934
m141102_iid=987614_0       TAGAATGACTTTGAGGTGGATTTGCTGTAGATAACGCAACATTAAATCGATTCTTTGCAT   8937
m141108_iid=1701429_0      TAGAATGACTTTGAGGTGGATTTGCTGTAGATAACGCAACATTAAATCGATTCTTTGCAT   5666
m141102_iid=987286_0       TAGAATGACTTTGAGGTGGATTTGCTGTAGATAACGCAACATTAAATCGATTCTTTGCAT   6892
m150430_iid=3272125_0      TAGAATGACTTTGAGGTGGATTTGCTGTAGATAACGCAACATTAAATCGATTCTTTGCAT   8402
m141102_iid=754225_0       TAGAATGACTTTGAGGTGGATTTGCTGTAGATAACGCAACATTAAATCGATTCTTTGCAT   9569
m141107_iid=1409934_0      TAGAATGACTTTGAGGTGGATTTGCTGTAGATAACGCAACATTAAATCGATTCTTTGCAT   10124
m150503_iid=3416920_0      TAGAATGACTTTGAGGTGGATTTGCTGTAGATAACGCAACATTAAATCGATTCTTTGCAT   8835
m141108_iid=1800121_0      TAGAATGACTTTGAGGTGGATTTGCTGTAGATAACGCAACATTAAATCGATTCTTTGCAT   8641
m141031_iid=147540_0       TAGAATGACTTTGAGGTGGATTTGCTGTAGATAACGCAACATTAAATCGATTCTTTGCAT   9000
m150507_iid=3674924_0      TAGAATGACTTTGAGGTGGATTTGCTGTAGATAACGCAACATTAAATCGATTCTTTGCAT   9150
```

```
m150503_iid=2923751_0      TAGAATGACTTTGAGGTGGATTTGCTGTAGATAACGCAACATTAAATCGATTCTTTGCAT   8974
m141031_iid=115118_0       TAGAATGACTTTGAGGTGGATTTGCTGTAGATAACGCAACATTAAATCGATTCTTTGCAT   7847
m150430_iid=2722448_0      TAGAATGACTTTGAGGTGGATTTGCTGTAGATAACGCAACATTAAATCGATTCTTTGCAT   7771
m150507_iid=3102647_0      TAGAATGACTTTGAGGTGGATTTGCTGTAGATAACGCAACATTAAATCGATTCTTTGCAT   6078
m141108_iid=1811866_0      TAGAATGACTTTGAGGTGGATTTGCTGTAGATAACGCAACATTAAATCGATTCTTTGCAT   7960
m150501_iid=2948495_0      TAGAATGACTTTGAGGTGGATTTGCTGTAGATAACGCAACATTAAATCGATTCTTTGCAT   7375
m150504_iid=3238195_0      TAGAATGACTTTGAGGTGGATTTGCTGTAGATAACGCAACATTAAATCGATTCTTTGCAT   9713
m150503_iid=3388511_0      TAGAATGACTTTGAGGTGGATTTGCTGTAGATAACGCAACATTAAATCGATTCTTTGCAT   7412
m150505_iid=3777611_0      TAGAATGACTTTGAGGTGGATTTGCTGTAGATAACGCAACATTAAATCGATTCTTTGCAT   8838
m150430_iid=2438833_0      TAGAATGACTTTGAGGTGGATTTGCTGTAGATAACGCAACATTAAATCGATTCTTTGCAT   9170
m141108_iid=1698969_0      TAGAATGACTTTGAGGTGGATTTGCTGTAGATAACGCAACATTAAATCGATTCTTTGCAT   10305
m150430_iid=3059190_0      TAGAATGACTTTGAGGTGGATTTGCTGTAGATAACGCAACATTAAATCGATTCTTTGCAT   6027
m141107_iid=1383067_0      TAGAATGACTTTGAGGTGGATTTGCTGTAGATAACGCAACATTAAATCGATTCTTTGCAT   6026
m141101_iid=597008_0       TAGAATGACTTTGAGGTGGATTTGCTGTAGATAACGCAACATTAAATCGATTCTTTGCAT   3096
m150506_iid=2596496_0      ------------------------------------------------------------   10491
m141102_iid=910051_0       ------------------------------------------------------------   7428
m150505_iid=3784017_0      TAGAATGACTTTGAGGTGGATTTGCTGTAGATAACGCAACATTAAATCGATTCTTTGCAT   8950
m141101_iid=682151_0       TAGAATGACTTTGAGGTGGATTTGCTGTAGATAACGCAACATTAAATCGATTCTTTGCAT   8021
m150501_iid=3748029_0      TAGAATGACTTTGAGGTGGATTTGCTGTAGATAACGCAACATTAAATCGATTCTTTGCAT   8565
m150504_iid=2876272_0      TAGAATGACTTTGAGGTGGATTTGCTGTAGATAACGCAACATTAAATCGATTCTTTGCAT   10421
NC_042820.1                TAGAATGACTTTGAGGTGGATTTGCTGTAGATAACGCAACATTAAATCGATTCTTTGCAT   10672
m141102_iid=1228825_0      TAGAATGACTTTGAGGTGGATTTGCTGTAGATAACGCAACATTAAATCGATTCTTTGCAT   10495
m150504_iid=2876274_0      TAGAATGACTTTGAGGTGGATTTGCTGTAGATAACGCAACATTAAATCGATTCTTTGCAT   10493
m141030_iid=80036_0        TAGAATGACTTTGAGGTGGATTTGCTGTAGATAACGCAACATTAAATCGATTCTTTGCAT   10262
m150506_iid=2596494_0      TAGAATGACTTTGAGGTGGATTTGCTGTAGATAACGCAACATTAAATCGATTCTTTGCAT   10661
m150503_iid=2526862_0      TAGAATGACTTTGAGGTGGATTTGCTGTAGATAACGCAACATTAAATCGATTCTTTGCAT   10346
m150506_iid=2567000_0      TAGAATGACTTTGAGGTGGATTTGCTGTAGATAACGCAACATTAAATCGATTCTTTGCAT   8300
m150507_iid=3073271_0      TAGAATGACTTTGAGGTGGATTTGCTGTAGATAACGCAACATTAAATCGATTCTTTGCAT   9600
m150504_iid=3156087_0      TAGAATGACTTTGAGGTGGATTTGCTGTAGATAACGCAACATTAAATCGATTCTTTGCAT   9426
m150505_iid=2498502_0      TAGAATGACTTTGAGGTGGATTTGCTGTAGATAACGCAACATTAAATCGATTCTTTGCAT   7076
m141102_iid=754227_0       TAGAATGACTTTGAGGTGGATTTGCTGTAGATAACGCAACATTAAATCGATTCTTTGCAT   9638
m150507_iid=3469514_0      TAGAATGACTTTGAGGTGGATTTGCTGTAGATAACGCAACATTAAATCGATTCTTTGCAT   10196
Rdo_MitoGenome             TAGAATGACTTTGAGGTGGATTTGCTGTAGATAACGCAACATTAAATCGATTCTTTGCAT   10672
m141101_iid=614933_0       ------------------------------------------------------------   9134
```

cytB

```
m141031_iid=347546_0       TCCACTTCCTTCTTTCCATTCATTGTAAGTGCAATAGTTATAATTCACTTATTATTCCTT   5424
m150506_iid=3127414_0      TCCACTTCCTTCTTCCCATTCATTGTAAGTGCAATAGTTATAATTCACTTATTAT-TCTT   7168
m141102_iid=1245517_0      ------------------------------------------------------------   7295
m141107_iid=1405727_0      TCCACTTCCTTCTT-CCATTCATTGTAAGTGCAATAGTTATAATTCACTTATTATTCCTT   5993
m141102_iid=987614_0       TCCACTTCCTTCTT-CCATTCATTGTAAGTGCAATAGTTATAATTCACTTATTATTCCTT   8996
m141108_iid=1701429_0      TCCACTTCCTTCTT-CCATTCATTGTAAGTGCAATAGTTATAATTCACTTATTATTCCTT   5725
m141102_iid=987286_0       TCCACTTC-TTCTT-CCATTCATTGTAAGTGCAATAGTTATAATTCACTTATTATTCCTT   6950
m150430_iid=3272125_0      TCCACTTCCTTCTT-CCATTCATTGTAAGTGCAATAGTTATAATTCACTTATTATTCCTT   8461
m141102_iid=754225_0       TCCACTTCCTTCTT-CCATTCATTGTAAGTGCAATAGTTATAATTCACTTATTATTCCTT   9628
m141107_iid=1409934_0      TCCACTTCCTTCTT-CCATTCATTGTAAGTGCAATAGTTATAATTCACTTATTATTCCTT   10183
m150503_iid=3416920_0      TCCACTTCCTTCTT-CCATTCATTGTAAGTGCAATAGTTATA-ATCACTTATTATTCCTT   8893
m141108_iid=1800121_0      TCCACTTCCTTCTT-CCATTCATTGTAAGTGCAATAGTTATAATTCACTTATTATTCCTT   8700
m141031_iid=147540_0       TCCACTTCCTTCTT-CCATTCATTGTAAGTGCAATAGTTATAATTCACTTATTATTCCTT   9059
m150507_iid=3674924_0      TCCACTTCCTTCTT-CCATTCATTGTAAGTGCAATAGTTATAATTCACTTATTATTCCTT   9209
m150503_iid=2923751_0      TCCACTTCCTTCTT-CCATTCATTGTAAGTGCAATAGTTATAATTCACTTATTATTCCTT   9033
m141031_iid=115118_0       TCCACTTCCTTCTT-CCATTCATTGTAAGTGCAATAGTTATAATTCACTTATTATTCCTT   7906
m150430_iid=2722448_0      TCCACTTCCTTCTT-CCATTCATTGTAAGTGCAATAGTTATAATTCACTTATTATTCCTT   7830
m150507_iid=3102647_0      TCCACTTCCTTCTT-CCATTCATTGTAAGTGCAATAGTTATAATTCACTTATTATTCCTT   6137
m141108_iid=1811866_0      TCCACTTCCTTCTT-CCATTCATTGTAAGTGCAATAGTTATAATTCACTTATTATTCCTT   8019
m150501_iid=2948495_0      TCCACTTCCTTCTT-CCATTCATTGTAAGTGCAATAGTTATAATTCACTTATTATTCCTT   7434
m150504_iid=3238195_0      TCCACTTCCTTCTT-CCATTCATTGTAAGTGCAATAGTTATAATTCACTTATTATTCCTT   9772
m150503_iid=3388511_0      TCCACTTCCTTCTT-CCATTCATTGTAAGTGCAATAGTTATAATTCACTTATTATTCCTT   7471
m150505_iid=3777611_0      TCCACTTCCTTCTT-CCATTCATTGTAAGTGCAATAGTTATAATTCACTTATTATTCCTT   8897
m150430_iid=2438833_0      TCCACTTCCTTCTT-CCATTCATTGTAAGTGCAATAGTTATAATTCACTTATTATTCCTT   9229
m141108_iid=1698969_0      TCCACTTCCTTCTT-CCATTCATTGTAAGTGCAATAGTTATAATTCACTTATTATTCCTT   10364
m150430_iid=3059190_0      TCCACTTCCTTCTT-CCATTCATTGTAAGTGCAATAGTTATAATTCACTTATTATTCCTT   6086
m141107_iid=1383067_0      TCCACTTCCTTCTT-CCATTCATTGTAAGTGCAATAGTTATAATTCACTA-TTATTCCTT   6084
m141101_iid=597008_0       TCCACTTCCTTCTT-CCATTCATTGTAAGTGCAATAGTTATAATTCACTTATTATTCCTT   3155
m150506_iid=2596496_0      ------------------------------------------------------------   10491
m141102_iid=910051_0       ------------------------------------------------------------   7428
m150505_iid=3784017_0      TCCACTTCCTTCTT-CCATTCATTGTAAGTGCAATAGTTATAATTCACTTATTATTCCTT   9009
m141101_iid=682151_0       TCCACTTCCTTCTT-CCATTCATTGTAAGTGCAATAGTTATAATTCACTTATTATTCCTT   8080
m150501_iid=3748029_0      TCCACTTCCTTCTT-CCATTCATTGTAAGTGCAATAGTTATAATTCACTTATTATTCCTT   8624
m150504_iid=2876272_0      TCCACTTCCTTCTT-CCATTCATTGTAAGTGCAATAGTTATAATTCACTTATTATTCCTT   10480
NC_042820.1                TCCACTTCCTTCTT-CCATTCATTGTAAGTGCAATAGTTATAATTCACTTATTATTCCTT   10731
```

```
m141102_iid=1228825_0      TCCACTTCCTTCTT-CCATTCATTGTAAGTGCAATAGTTATAATTCACTTATTATTCCTT   10554
m150504_iid=2876274_0      TCCACTTCCTTCTT-CCATTCATTGTAAGTGCAATAGTTATAATTCACTTATTATTCCTT   10552
m141030_iid=80036_0        TCCACTTCCTTCTT-CCATTCATTGTAAGTGCAATAGTTATAATTCACTTATTATTCCTT   10321
m150506_iid=2596494_0      TCCACTTCCTTCTT-CCATTCATTGTAAGTGCAATAGTTATAATTCACTTATTATTCCTT   10720
m150503_iid=2526862_0      TCCACTTCCTTCTT-CCATTCATTGTAAGTGCAATAGTTATAATTCACTTATTATTCCTT   10405
m150506_iid=2567000_0      TCCACTTCCTTCTT-CCATTCATTGTAAGTGCAATAGTTATAATTCACTTATTATTCCTT   8359
m150507_iid=3073271_0      TCCACTTCCTTCTT-CCATTCATTGTAAGTGCAATAGTTATAATTCACTTATTATTCCTT   9659
m150504_iid=3156087_0      TCCACTTCCTTCTT-CCATTCATTGTAAGTGCAATAGTTATAATTCACTTATTATTCCTT   9485
m150505_iid=2498502_0      TCCACTTCCTTCTT-CCATTCATTGTAAGTGCAATAGTTATAATTCACTTATTATTCCTT   7135
m141102_iid=754227_0       TCCACTTCCTTCTT-CCATTCATTGTAAGTGCAATAGTTATAATTCACTTATTATTCCTT   9697
m150507_iid=3469514_0      TCCACTTCCTTCTT-CCATTCATTGTAAGTGCAATAGTTATAATTC--------------   10241
Rdo_MitoGenome             TCCACTTCCTTCTT-CCATTCATTGTAAGTGCAATAGTTATAATTCACTTATTATTCCTT   10731
m141101_iid=614933_0       ------------------------------------------------------------   9134
```

cytB

```
m141031_iid=347546_0       CACCAAACAGGATCAAATAACCCTTTAGGAACAAATAGAAATATT---GATAAAATCCCA   5481
m150506_iid=3127414_0      CACCAAACAGGATCAAATAACCCTTTAGGAACAAATAGAAATATTGATGATAAAATCCCA   7228
m141102_iid=1245517_0      ------------------------------------------------------------   7295
m141107_iid=1405727_0      CACCAAACAGGATCAAATAACCCTTTAGGAACAAATAGAAATATT---GATAAAATCCCA   6050
m141102_iid=987614_0       CACCAAACAGGATCAAATAACCCTTTAGGAACAAATAGAAATATT---GATAAAATCCCA   9053
m141108_iid=1701429_0      CACCAAACAGGATCAAATAACCCTTTAGGAACAAATAGAAATATT---GATAAAATCCCA   5782
m141102_iid=987286_0       CACCAAACAGA-TCAAATAACCCTTTA-GAACAAATAGAAATATT---GATAAAA-TCCA   7004
m150430_iid=3272125_0      CACCAAACAGGATCAAATAACCCTTTAGGAACAAATAGAAATATT---GATAAAATCCCA   8518
m141102_iid=754225_0       CACCAAACAGGATCAAATAACCCTTTAGGAACAAATAGAAATATT---GATAAAATCCCA   9685
m141107_iid=1409934_0      CAC-AAACAGGATCAAATAACCCTTTAGGAACAAATAGAAATATT---GATAAAATCCCA   10239
m150503_iid=3416920_0      CACCAAACAGGATCAAATAACCCTTTAGGAACAAATAGAAATATT---GATAAAATCCCA   8950
m141108_iid=1800121_0      CACCAAACAGGATCAAATAACCCTTTAGGAACAAATAGAAATATT---GATAAAATCCCA   8757
m141031_iid=147540_0       CACCAAACAGGATCAAATAACCCTTTAGGAACAAATAGAAATATT---GATAAAATCCCA   9116
m150507_iid=3674924_0      CACCAAACAGGATCAAATAACCCTTTAGGAACAAATAGAAATATT---GATAAAATCCCA   9266
m150503_iid=2923751_0      CACCAAACAGGATCAAATAACCCTTTAGGAACAAATAGAAATATT---GATAAAATCCCA   9090
m141031_iid=115118_0       CACCAAACAGGATCAAATAACCCTTTAGGAACAAATAGAA-TATT---GATAAAATCCCA   7962
m150430_iid=2722448_0      CACCAAACAGGATCAAATAACCCTTTAGGAACAAATAGAAATATT---GATAAAATCCCA   7887
m150507_iid=3102647_0      CACCAAACAGGATCAAATAAC-CTTTAGGAACAAATAGAAATATT---GATAAAA-TCCA   6192
m141108_iid=1811866_0      CACCAAACAGGATCAA--AACCCTTTAGGAACAAATAGAAATATT---GATAAAATCCCA   8074
m150501_iid=2948495_0      CACCAAACAGGATCAAATAACCCTTTAGGAACAAATAGAAATATT---GATAAAATCCCA   7491
m150504_iid=3238195_0      CACCAAACAGGATCAAATAACCCTTTAGGAACAAATAGAAATATT---GATAAAATCCCA   9829
m150503_iid=3388511_0      CACCAAACAGGATCAAATAACCCTTTAGGAACAAATAGAAATATT---GATAAAATCCCA   7528
m150505_iid=3777611_0      CACCAAACAGGATCAAATAACCCTTTAGGAACAAATAGAAATATT---GATAAAATCCCA   8954
m150430_iid=2438833_0      CACCAAACAGGATCAAATAACCCTTTAGGAACAAATAGAAATATT---GATAAAATCCCA   9286
m141108_iid=1698969_0      CACCAAACAGGATCAAATAACCCTTTAGGAACAAATAGAAATATT---GATAAAATCCCA   10421
m150430_iid=3059190_0      CACCAAACAGGATCAAATAACCCTTTAGGAACAAATAGAAATATT---GATAAAATCCCA   6143
m141107_iid=1383067_0      CACCAAACAGGATCAAATAACCCTTTAGGAACAAATAGAAATATT---GATAAAATCCCA   6141
m141101_iid=597008_0       CACCAAACAGGATCAAATAACCCTTTAGGAACAAATAGAAATATT---GATAAAATCCCA   3212
m150506_iid=2596496_0      ------------------------------------------------------------   10491
m141102_iid=910051_0       ------------------------------------------------------------   7428
m150505_iid=3784017_0      CACCAAACAGGATCAAATAACCCTTTAGGAACAAATAGAAATATT---GATAAAATCCCA   9066
m141101_iid=682151_0       CACCAAACAGGATCAAATAACCCTTTAGGAACAAATAGAAATATT---GATAAAATCCCA   8137
m150501_iid=3748029_0      CACCAAACAGGATCAAATAACCCTTTAGGAACAAATAGAAATATT---GATAAAATCCCA   8681
m150504_iid=2876272_0      CACCAAACAGGATCAAATAACCCTTTAGGAACAAATAGAAATATT---GATAAAATCCCA   10537
NC_042820.1                CACCAAACAGGATCAAATAACCCTTTAGGAACAAATAGAAATATT---GATAAAATCCCA   10788
m141102_iid=1228825_0      CACCAAACAGGATCAAATAACCCTTTAGGAACAAATAGAAATATT---GATAAAATCCCA   10611
m150504_iid=2876274_0      CACCAAACAGGATCAAATAACCCTTTAGGAACAAATAGAAATATT---GATAAAATCCCA   10609
m141030_iid=80036_0        CACCAAACAGGATCAAATAACCCTTTAGGAACAAATAGAAATATT---GATAAAATCCCA   10378
m150506_iid=2596494_0      CACCAAACAGGATCAAATAACCCTTTAGGAACAAATAGAAATATT---GATAAAATCCCA   10777
m150503_iid=2526862_0      CACCAAACAGGATCAAATAACCCTTTAGGAACAAATAGAAATATT---GATAAAATCCCA   10462
m150506_iid=2567000_0      CACCAAACAGGATCAAATAACCCTTTAGGAACAAATAGAAATATT---GATAAAATCCCA   8416
m150507_iid=3073271_0      CACCAAACAGGATCAAATAACCCTTTAGGAACAAATAGAAATATT---GATAAAATCCCA   9716
m150504_iid=3156087_0      CACCAAACAGGATCAAATAACCCTTTAGGAACAAATAGAAATATT---GATAAAATCCCA   9542
m150505_iid=2498502_0      CACCAAACAGGATCAAATAACCCTTTAGGAACAAATAGAAATATT---GATAAAATCCCA   7192
m141102_iid=754227_0       CACCAAACAGGATCAAATAACCCTTTAGGAACAAATAGAAATATT---GATAAAATCCCA   9754
m150507_iid=3469514_0      ------------------------------------------------------------   10241
Rdo_MitoGenome             CACCAAACAGGATCAAATAACCCTTTAGGAACAAATAGAAATATT---GATAAAATCCCA   10788
m141101_iid=614933_0       ------------------------------------------------------------   9134
```

cytB

```
m141031_iid=347546_0       TTCCACCCTTACTTTTCAACAAAAGATATAATAGGATT--TATCGTTACAACCATAATAT   5539
m150506_iid=3127414_0      TTCCACCCTTACTTTTCAACAAAAGATATAATAGGATTTATATCGTTACAACCATAATAT   7288
m141102_iid=1245517_0      ------------------------------------------------------------   7295
m141107_iid=1405727_0      TTCCACCCTTACTTTTCAACAAAAGATATAATAGGATT--TATCGTTACAACCATAATAT   6108
m141102_iid=987614_0       TTCCACCCTTACTTTTCAACAAAAGATATAATAGGATT--TATCGTTACAACCATAATAT   9111
m141108_iid=1701429_0      TTCCACCCTTACTTTTCAACAAAAGATATAATAGGATT--TATCGTTACAACCATAATAT   5840
```

```
m141102_iid=987286_0     TTCA--CCTTACTTTTCAACAAAAGATATAATAGGATT--TATCGTTACAACCATAATAT   7060
m150430_iid=3272125_0    TTCC--CCTTACTTTTCAACAAAAGATATAATAGGATT--TATCGTTACAACCATAATAT   8574
m141102_iid=754225_0     TTCCACCCTTACTTTTCAACAAAAGATATAATAGGATT--TATCGTTACA-CCATAATAT   9742
m141107_iid=1409934_0    TTCCACCCTTACTTTTCAAAAAA-------------------------------------   10262
m150503_iid=3416920_0    TTCCACCCTTACTTTTCAACAAAAGATATAATAGGATT--TATCGTTACAACCATAATAT   9008
m141108_iid=1800121_0    TTCCACCCTTACTTTTCAACAAAAGATATAATAGGATT--TATCGTTACAACCATAATAT   8815
m141031_iid=147540_0     TTCCACCCTTACTTTTCAACAAAAGATATAATAGGATT--TATCGTTACAACCATAATAT   9174
m150507_iid=3674924_0    TTCCACCCTTACTTTTCAACAAAAGATATAATAGGATT--TATCGTTACAACCATAATAT   9324
m150503_iid=2923751_0    TTCCACCCTTACTTTTCAACAAAAGATATAATAGGATT--TATCGTTACAACCATAATAT   9148
m141031_iid=115118_0     TTCCACCCTTACTTTTCAA-----------------------------------------   7981
m150430_iid=2722448_0    TTCCACCCTTACTTTTCAACAAAAGATATAATAGGATT--TATCGTTACAACCATAATAT   7945
m150507_iid=3102647_0    TTCCACCCTTACTTTTCAACAAAAGATATAATAGGATT--TATCGTTACAACCATAATAT   6250
m141108_iid=1811866_0    TTCCACCCTTACTTTTCAACAAAAGATATAATAGGATT--TATCGTTACAACCATAATAT   8132
m150501_iid=2948495_0    TTCCACCCTTACTTTTCAACAAAAGATATAATAGGATT--TATCGTTACAACCATAATAT   7549
m150504_iid=3238195_0    TTCCACCCTTACTTTTCAACAAAAGATATAATAGGATT--TATCGTTACAACCATAATAT   9887
m150503_iid=3388511_0    TTCCACCCTTACTTTTCAACAAAAGATATAATAGGATT--TATCGTTACAACCATAATAT   7586
m150505_iid=3777611_0    TTCCACCCTTACTTTTCAACAAAAGATATAATAGGATT--TATCGTTACAACCATAATAT   9012
m150430_iid=2438833_0    TTCCACCCTTACTTTTCAACAAAAGATATAATAGGATT--TATCGTTACAACCATAATAT   9344
m141108_iid=1698969_0    TTCCACCCTTACTTTTCAACAAAAGATATAATAGGATT--TATCGTTACAACCATAATAT   10479
m150430_iid=3059190_0    TTCCAC-CTTACTTTTCAACAAAAGATATAATAGGATT--TATCGTTACAACCATAATAT   6200
m141107_iid=1383067_0    TTCCACCCTTACTTTTCAACAAAAGATATAATAGGATT--TATCGTTACAACCATAATAT   6199
m141101_iid=597008_0     TTCCACCCTTACTTTTCAACAAAAGATATAATAGGATT--TATCGTTACAACCATAATAT   3270
m150506_iid=2596496_0    ------------------------------------------------------------   10491
m141102_iid=910051_0     ------------------------------------------------------------   7428
m150505_iid=3784017_0    TTCCACCCTTACTTTTCAACAAAAGATATAATAGGATT--TATCGTTACAACCATAATAT   9124
m141101_iid=682151_0     TTCCACCCTTACTTTTCAACAAAAGATATAATAGGATT--TATCGTTACAACCATAATAT   8195
m150501_iid=3748029_0    TTCCACCCTTACTTTTCAACAAAAGATATAATAGGATT--TATCGTTACAACCATAATAT   8739
m150504_iid=2876272_0    TTCCACCCTTACTTTTCAACAAAAGATATAATAGGATT--TATCGTTACAACCATAATAT   10595
NC_042820.1              TTCCACCCTTACTTTTCAACAAAAGATATTATAGGATT--TATCGTTACAACCATAATAT   10846
m141102_iid=1228825_0    TTCCACCCTTACTTTTCAACAAAAGATATAATAGGATT--TATCGTTACAACCATAATAT   10669
m150504_iid=2876274_0    TTCCACCCTTACTTTTCAACAAAAGATATAATAGGATT--TATCGTTACAACCATAATAT   10667
m141030_iid=80036_0      TTCCACCCTTACTTTTCAACAAAAGATATAATAGGATT--TATCGTTACAACCATAATAT   10436
m150506_iid=2596494_0    TTCCAC-CTTACTTTTCAACAAAAGATATAATAGGATT--TATCGTTACAACCATAATAT   10834
m150503_iid=2526862_0    TTCCA-CCTTACTTTTCAACAAAAGATATAATAGGATT--TATCGTTACAACCATAATAT   10519
m150506_iid=2567000_0    TTCCACCCTTACTTTTCAACAAAAGATATAATAGGATT--TATCGTTACAACCATAATAT   8474
m150507_iid=3073271_0    TTCCACCCTTACTTTTCAACAAAAGATATAATAGGATT--TATCGTTACAACCATAATAT   9774
m150504_iid=3156087_0    TTCCACCCTTACTTTTCAACAAAAGATATAATAGGATT--TATCGTTACAACCATAATAT   9600
m150505_iid=2498502_0    TTCCACCCTTACTTTTCAACAAAAGATATAATAGGATT--TATCGTTACAACCATAATAT   7250
m141102_iid=754227_0     TTCCACCCTTACTTTTCAACAAAAGATATAATAGGATT--TATCGTTACAACCATAATAT   9812
m150507_iid=3469514_0    ------------------------------------------------------------   10241
Rdo_MitoGenome           TTCCACCCTTACTTTTCAACAAAAGATATAATAGGATT--TATCGTTACAACCATAATAT   10846
m141101_iid=614933_0     ------------------------------------------------------------   9134
```

cytB

```
m141031_iid=347546_0     TAATAACAATTTCACTATGAAATCCTTATTTATTAGGAGACCCAGACAATTTTATTCCAG   5599
m150506_iid=3127414_0    TAATAACAATTTCACTATGAAATCCTTATTTATTAGGAGACCCAGACAATTT--TTACAG   7346
m141102_iid=1245517_0    ------------------------------------------------------------   7295
m141107_iid=1405727_0    TAATAACAATTTCACTATGAAATCCTTATTTATTAGGAGACCCAGACAATTTTATTCCAG   6168
m141102_iid=987614_0     TAATAACAATTTCACTATGAAATCCTTATTTATTAGGAGACCCAGACAATTTTATTCCAG   9171
m141108_iid=1701429_0    TAATAACAATTTCACTATGAAATCCTTATTTATTAGGAGACCCAGACAATTTTATTCCAG   5900
m141102_iid=987286_0     TAATAACAATTTCACTATGAAATCCTTATTTATTAGGAGACCCAGACAATTTTATTCCAG   7120
m150430_iid=3272125_0    TAATAACAATTTCACTATGAAATCCTTATTTATTAGGAGACCCAGACAATTTTATTCCAG   8634
m141102_iid=754225_0     TAATAACAATTTCACTATGAAATCCTTATTTATTAGGAGACCCAGACAATTTTATTCCAG   9802
m141107_iid=1409934_0    ------------------------------------------------------------   10262
m150503_iid=3416920_0    TAATAACAATTTCACTATGAAATCCTTATTTATTAGGAGACCCAGACAATTTTATTCCAG   9068
m141108_iid=1800121_0    TAATAACAATTTCACTATGAAATCCTTATTTATTAGGAGACCCAGACAATTTTATTCCAG   8875
m141031_iid=147540_0     TAATAACAATTTCACTATGAAATCCTTATTTATTAGGAGACCCAGACAATTTTATTCCAG   9234
m150507_iid=3674924_0    TA-ATACAATTTCACTATGAAATCCTTATTTATTAGGAGACCCAGACAATTTTATTCCAG   9383
m150503_iid=2923751_0    TAATAACAATTTCACTATGAAATCCTTATTTATTAGGAGACCCAGACAATTTTATTCCAG   9208
m141031_iid=115118_0     ------------------------------------------------------------   7981
m150430_iid=2722448_0    TAATAACAATTTCACTATGAAATCCTTATTTATTAGGAGACCCAGACAATTTTATTCCAG   8005
m150507_iid=3102647_0    TAATAACAATTTCACTATGAAATCCTTATTTATTAGGAGACCCAGACAATTTTATTCCAG   6310
m141108_iid=1811866_0    TAATAACAATTTCACTATGAAATCCTTATTTATTAGGAGACCCAGACAATTTTATTCCAG   8192
m150501_iid=2948495_0    TAATAACAATTTCACTATGAAATCCTTATTTATTAGGAGACCCAGACAATTTTATTCCAG   7609
m150504_iid=3238195_0    TAATAACAATTTCACTATGAAATCCTTATTTATTAGGAGACCCAGACAATTTTATTCCAG   9947
m150503_iid=3388511_0    TAATAACAATTTCACTATGAAATCCTTATTTATTAGGAGACCCAGACAATTTTATTCCAG   7646
m150505_iid=3777611_0    TAATAACAATTTCACTATGAAATCCTTATTTATTAGGAGACCCAGACAATTTTATTCCAG   9072
m150430_iid=2438833_0    TAATAACAATTTCACTATGAAATCCTTATTTATTAGGAGACCCAGACAATTTTATTCCAG   9404
m141108_iid=1698969_0    TAATAACAATTTCACTATGAAATCCTTATTTATTAGGAGACCCAGACAATTTTATTCCAG   10539
m150430_iid=3059190_0    TAATAACAATTTCACTATGAAATCCTTATTTATTAGGAGACCCAGACAATTTTATTCCAG   6260
m141107_iid=1383067_0    TAATAACAATTTCACTATGAAATCCTTATTTATTAGGAGACCCAGACAATTTTATTCCAG   6259
```

```
m141101_iid=597008_0        TAATAACAATTTCACTATGAAATCCTTATTTATTAGGAGACCCAGACAATTTTATTCCAG   3330
m150506_iid=2596496_0       ------------------------------------------------------------   10491
m141102_iid=910051_0        ------------------------------------------------------------   7428
m150505_iid=3784017_0       TAATAACAATTTCACTATGAAATCCTTATTTATTAGGAGACCCAGACAATTTTATTCCAG   9184
m141101_iid=682151_0        TAATAACAATTTCACTATGAAATCCTTATTTATTAGGAGACCCAGACAATTTTATTCCAG   8255
m150501_iid=3748029_0       TAATAACAATTTCACTATGAAATCCTTATTTATTAGGAGACCCAGACAATTTTATTCCAG   8799
m150504_iid=2876272_0       TAATAACAATTTCACTATGAAATCCTTATTTATTAGGAGACCCAGACAATTTTATTCCAG   10655
NC_042820.1                 TAATAACAATTTCACTATGAAATCCTTATTTATTAGGAGACCCAGACAATTTTATTCCAG   10906
m141102_iid=1228825_0       TAATAACAATTTCACTATGAAATCCTTATTTATTAGGAGACCCAGACAATTTTATTCCAG   10729
m150504_iid=2876274_0       TAATAACAATTTCACTATGAAATCCTTATTTATTAGGAGACCCAGACAATTTTATTCCAG   10727
m141030_iid=80036_0         TAATAACAATTTCACTATGAAATCCTTATTTATTAGGAGACCCAGACAATTTTATTCCAG   10496
m150506_iid=2596494_0       TAATAACAATTTCACTATGAAATCCTTATTTATTAGGAGACCCAGACAATTTTATTCCAG   10894
m150503_iid=2526862_0       TAATAACAATTTCACTATGAAATCCTTATTTATTAGGAGACCCAGACAATTTTATTCCAG   10579
m150506_iid=2567000_0       TAATAACAATTTCACTATGAAATCCTTATTTATTAGGAGACCCAGACAATTTTATTCCAG   8534
m150507_iid=3073271_0       TAATAACAATTTCACTATGAAATCCTTATTTATTAGGAGACCCAGACAATTTTATTCCAG   9834
m150504_iid=3156087_0       TAATAACAATTTCACTATGAAATCCTTATTTATTAGGAGACCCAGACAATTTTATTCCAG   9660
m150505_iid=2498502_0       TAATAACAATTTCACTATGAAATCCTTATTTATTAGGAGACCCAGACAATTTTATTCCAG   7310
m141102_iid=754227_0        TAATAACAATTTCACTATGAAATCCTTATTTATTAGGAGACCCAGACAATTTTATTCCAG   9872
m150507_iid=3469514_0       ------------------------------------------------------------   10241
Rdo_MitoGenome              TAATAACAATTTCACTATGAAATCCTTATTTATTAGGAGACCCAGACAATTTTATTCCAG   10906
m141101_iid=614933_0        ------------------------------------------------------------   9134
```

cytB

```
m141031_iid=347546_0        CTAACCCCTAGT---TACACCAGTTCACATCCAACCTGAATGATATTTTCTATTCGCAT-   5655
m150506_iid=3127414_0       CTAACCCCTAGT---TACACCAGTTCACATCCAACCTGAATGATATTTTCTATTCGCAT-   7402
m141102_iid=1245517_0       ------------------------------------------------------------   7295
m141107_iid=1405727_0       CTAACCCCCTAG--TTACACCAGTTCACATCCAACCTGAATGATATTTTCTATTCGCAT-   6225
m141102_iid=987614_0        CTAACCCCTA---GTTACACCAGTTCACATCCAACCTGAATGATATTTTCTATTCGCAT-   9227
m141108_iid=1701429_0       CTAACCCCA----GTTACACCAGTTCACATCCAACCTGAATGATATTTTCTATTCGCAT-   5955
m141102_iid=987286_0        CTAAC-CCTA---GTTACACCAGTTCACATCCAACCTGAATGATATTTTCTATTCGCAT-   7175
m150430_iid=3272125_0       CTAACCCCTA---GTTACACCAGTTCACATCCAACCTGAATGATATTTTCTATTCGCAT-   8690
m141102_iid=754225_0        CTAACCCCCTAGT--TACACCAGTTCACATCCAACCTGAATGATATTTTCTATTCGCAT-   9859
m141107_iid=1409934_0       ------------------------------------------------------------   10262
m150503_iid=3416920_0       CTAACCCCTA---GTTACACCAGTTCACATCCAACCTGAATGATATTTTCTATTCGCAT-   9124
m141108_iid=1800121_0       CTAACCCCCTAGT--TACACCAGTTCACATCCAACCTGAATGATAT-TTCTATTCGCAT-   8931
m141031_iid=147540_0        CTAACCCCCTAGT--TACACCAGTTCACATCCAACCTGAATGATATTTTCTATTCGCAT-   9291
m150507_iid=3674924_0       CTAACCCCTA---GTTACACCAGTTCACATCCAACCTGAATGATATTTTCTATTCGCAT-   9439
m150503_iid=2923751_0       CTAACCCCCTAGT--TACACCAGTTCACATCCAACCTGAATGATATTTTCTATTCGCAT-   9265
m141031_iid=115118_0        ------------------------------------------------------------   7981
m150430_iid=2722448_0       CTAACCCCCTAGT--TACACCAGTTCACATCCAACCTGAATGATATTTTCTATTCGCAT-   8062
m150507_iid=3102647_0       CTAACCCCCTAGT--TACACCAGTTCACATCCAACCTGAATGATATTTTCTATTCGCAT-   6367
m141108_iid=1811866_0       CTAACCCCCTAGT--TACACCAGTTCACATCCAACCTGAATGATATTTTCTATTCGCAAA   8250
m150501_iid=2948495_0       CTAACCCCTAG---TTACACCAGTTCACATCCAACCTGAATGATATTTTCTATTCGCAT-   7665
m150504_iid=3238195_0       CTAACCCCT-AGTTACACACCAGTTCACATCCAACCTGAATGATATTTTCTATTCGCAT-   10005
m150503_iid=3388511_0       CTAA-CCCCTAGT--TACACCAGTTCACATCCAACCTGAATGATATTTTCTATTCGCAT-   7702
m150505_iid=3777611_0       CTAACCCCCTAGT--TACACCAGTTCACATCCAACCTGAATGATATTTTCTATTCGCAT-   9129
m150430_iid=2438833_0       CTAA-CCCCTAGT--TACACCAGTTCACATCCAACCTGAATGATATTTTCTATTCGCAT-   9460
m141108_iid=1698969_0       CTAACCCCCTAGT--TACACCAGTTCACATCCAACCTGAATGATATTTTCTATTCGCAT-   10596
m150430_iid=3059190_0       CTAACCCCCTAGT--TACACCAGTTCACATCCAACCTGAATGATATTTTCTATTCGCAT-   6317
m141107_iid=1383067_0       CTAACC-CCTAGT--TACACCAGTTCACATCCAACCTGAATGATATTTTCTATTCGCAT-   6315
m141101_iid=597008_0        CTAACCCCCTAGT--TACACCAGTTCACATCCAACCTGAATGATATTTTCTATTCGCAT-   3387
m150506_iid=2596496_0       ------------------------------------------------------------   10491
m141102_iid=910051_0        ------------------------------------------------------------   7428
m150505_iid=3784017_0       CTAA-CCCCTAGT--TACACCAGTTCACATCCAACCTGAATGATATTTTCTATTCGCAT-   9240
m141101_iid=682151_0        CTAACCCCCTAGT--TACACCAGTTCACATCCAACCTGAATGATATTTTCTATTCGCAT-   8312
m150501_iid=3748029_0       CTAACCCCCTAGT--TACACCAGTTCACATCCAACCTGAATGATATTTTCTATTCGCAT-   8856
m150504_iid=2876272_0       CTAACCCCCTAGT--TACACCAGTTCACATCCAACCTGAATGATATTTTCTATTCGCAT-   10712
NC_042820.1                 CTAACCCCCTAGT--TACACCAGTTCACATCCAACCTGAATGATATTTTCTATTCGCAT-   10963
m141102_iid=1228825_0       CTAACCCCCTAGT--TACACCAGTTCACATCCAACCTGAATGATATTTTCTATTCGCAT-   10786
m150504_iid=2876274_0       CTAACCCCCTAGT--TACACCAGTTCACATCCAACCTGAATGATATTTTCTATTCGCAT-   10784
m141030_iid=80036_0         CTAACCCCCTAGT--TACACCAGTTCACATCCAACCTGAATGATATTTTCTATTCGCAT-   10553
m150506_iid=2596494_0       CTAACC-CCTAGT--TACACCAGTTCACATCCAACCTGAATGATATTTTCTATTCGCAT-   10950
m150503_iid=2526862_0       CTAACCCCCTAGT--TACACCAGTTCACATCCAACCTGAATGATATTTTCTATTCGCAT-   10636
m150506_iid=2567000_0       CTAACCCCCTAGT--TACACCAGTTCACATCCAACCTGAATGATATTTTCTATTCGCAT-   8591
m150507_iid=3073271_0       CTAACCCCCTAGT--TACACCAGTTCACATCCAACCTGAATGATATTTTCTATTCGCAT-   9891
m150504_iid=3156087_0       CTAACCCCCTAGT--TACACCAGTTCACATCCAACCTGAATGATATTTTCTATTCGCAT-   9717
m150505_iid=2498502_0       CTAACCCCCTAGT--TACACCAGTTCACATCCAACCTGAATGATATTTTCTATTCGCAT-   7367
m141102_iid=754227_0        CTAACCCCCTAGT--TACACCAGTTCACATCCAACCTGAATGATATTTTCTATTCGCAT-   9929
m150507_iid=3469514_0       ------------------------------------------------------------   10241
Rdo_MitoGenome              CTAACCCCCTAGT--TACACCAGTTCACATCCAACCTGAATGATATTTTCTATTCGCAT-   10963
m141101_iid=614933_0        ------------------------------------------------------------   9134
```

cytB

```
m141031_iid=347546_0       -ATGCAATTCTTCGATCAATCCCTAATAAATTAGGAGGAGTAATTGCACTAATTATATCA   5714
m150506_iid=3127414_0      -ATGCAATTCTTCGATCAATCCCTAATAAATTAGGAGGAGTAATTGCACTAATTATATCA   7461
m141102_iid=1245517_0      ------------------------------------------------------------   7295
m141107_iid=1405727_0      -ATGCAATTCTTCGATCAATCCCTAATAAATTAGGAGGAGTAATTGCACTAATTATATCA   6284
m141102_iid=987614_0       -ATGCAATTCTTCGATCAATCCCTAATAAATTAGGAGGAGTAATTGCACTAATTATATCA   9286
m141108_iid=1701429_0      -ATGCAATTCTTCGATCAATCCCTAATAAATTAGGAGGAGTAATTGCACTAATTATATCA   6014
m141102_iid=987286_0       -ATGCAATTCTTCGATCAT-CCCTAATAAATTAGGAGGAGTAATTGCACTAATTATATCA   7233
m150430_iid=3272125_0      -ATGCAATTCTTCGATCAATCCCTAATAAATTAGGAGGAGTAATTGCACTAATTATATCA   8749
m141102_iid=754225_0       -ATGCAATTCTTCGATCAATCCCTAATAAATTAGGAGGAGTAATTGCACTAAT-TTATCA   9917
m141107_iid=1409934_0      ------------------------------------------------------------   10262
m150503_iid=3416920_0      -ATGCAATTCTTCGATCAATCCCTAATAAATTAGGAGGAGTAATTGCACTAATTATATCA   9183
m141108_iid=1800121_0      -ATGCAATTCTTCGATCAATCCCTAATAAATTAGGAGGAGTAATTGCACTAATTATATCA   8990
m141031_iid=147540_0       -ATGCAATTCTTCGATCAATCCCTAATAAATTAGGAGGAGTAATTGCACTAATTATATCA   9350
m150507_iid=3674924_0      -ATGCAATTCTTCGATCAATCCCTAATAAATTAGGAGGAGTAATTGCACTAATTATATCA   9498
m150503_iid=2923751_0      -ATGCAATTCTTCGATCAATCCCTAATAAATTAGGAGGAGTAATTGCACTAATTATATCA   9324
m141031_iid=115118_0       ------------------------------------------------------------   7981
m150430_iid=2722448_0      -ATGCAATTCTTCGATCAATCCCTAATAAATTAGGAGGAGTAATTGCACTAATTATATCA   8121
m150507_iid=3102647_0      -ATGCAATTCTTCGATCAATCCCTAATAAATTAGGAGGAGTAATTGCACTAATTATATCA   6426
m141108_iid=1811866_0      TATGCAATTCTTCGATCAATCCCTAATAAATTAGGAGGAGTAATTGCACTAATTATATCA   8310
m150501_iid=2948495_0      -ATGCAATTCTTCGATCAATCCCTAATAAATTAG---GAGTAATTGCACTAATTATATCA   7721
m150504_iid=3238195_0      -ATGCAATTCTTCGATCAATCCCTAATAAATTAGGAGGAGTAATTGCACTAATTATATCA   10064
m150503_iid=3388511_0      -ATGCAATTCTTCGATCAATCCCTAATAAATTAGGAGGAGTAATTGCACTAATTATATCA   7761
m150505_iid=3777611_0      -ATGCAATTCTTCGATCAATCCCTAATAAATTAGGAGGAGTAATTGCACTAATTATATCA   9188
m150430_iid=2438833_0      -ATGCAATTCTTCGATCAATCCCTAATAAATTAGGAGGAGTAATTGCACTAATTATATCA   9519
m141108_iid=1698969_0      -ATGCAATTCTTCGATCAATCCCTAATAAATTAGGAGGAGTAATTGCACTAATTATATCA   10655
m150430_iid=3059190_0      -ATGCAATTCTTCGATCAATCCCTAATAAATTAGGAGGAGTAATTGCACTAATTATATCA   6376
m141107_iid=1383067_0      -ATGCAATTCTTCGATCAATCC-TAATAAATTAGGAGGAGTAATTGCACTAATTATATCA   6373
m141101_iid=597008_0       -ATGCAATTCTTCGATCAATCCCTAATAAATTAGGAGGAGTAATTGCACTAATTATATCA   3446
m150506_iid=2596496_0      ------------------------------------------------------------   10491
m141102_iid=910051_0       ------------------------------------------------------------   7428
m150505_iid=3784017_0      -ATGCAATTCTTCGATCAATCCCTAATAAATTAGGAGGAGTAATTGCACTAATTATATCA   9299
m141101_iid=682151_0       -ATGCAATTCTTCGATCAATCCCTAATAAATTAGGAGGAGTAATTGCACTAATTATATCA   8371
m150501_iid=3748029_0      -ATGCAATTCTTCGATCAATCCCTAATAAATTAGGAGGAGTAATTGCACTAATTATATCA   8915
m150504_iid=2876272_0      -ATGCAATTCTTCGATCAATCCCTAATAAATTAGGAGGAGTAATTGCACTAATTATATCA   10771
NC_042820.1                -ATGCAATTCTTCGATCAATCCCTAATAAATTAGGAGGAGTAATTGCACTAATTATATCA   11022
m141102_iid=1228825_0      -ATGCAATTCTTCGATCAATCCCTAATAAATTAGGAGGAGTAATTGCACTAATTATATCA   10845
m150504_iid=2876274_0      -ATGCAATTCTTCGATCAATCCCTAATAAATTAGGAGGAGTAATTGCACTAATTATATCA   10843
m141030_iid=80036_0        -ATGCAATTCTTCGATCAATCCCTAATAAATTAGGAGGAGTAATTGCACTAATTATATCA   10612
m150506_iid=2596494_0      -ATGCAATTCTTCGATCAATCCCTAATAAATTAG--------------------------   10983
m150503_iid=2526862_0      -ATGCAATTCTTCGATCAATCCCTAATAAATTAGGAGGAGTAATTGCACTAATTATATCA   10695
m150506_iid=2567000_0      -ATGCAATTCTTCGATCAATCCCTAATAAATTAGGAGGAGTAATTGCACTAATTATATCA   8650
m150507_iid=3073271_0      -ATGCAATTCTTCGATCAATCCCTAATAAATTAGGAGGAGTAATTGCACTAATTATATCA   9950
m150504_iid=3156087_0      -ATGCAATTCTTCGATCAATCCCTAATAAATTAGGAGGAGTAATTGCACTAATTATATCA   9776
m150505_iid=2498502_0      -ATGCAATTCTTCGATCAATCCCTAATAAATTAGGAGGAGTAATTGCACTAATTATATCA   7426
m141102_iid=754227_0       -ATGCAATTCTTCGATCAATCC-TAATAAATTAGGAGGAGTAATTGCACTAATTATATCA   9987
m150507_iid=3469514_0      ------------------------------------------------------------   10241
Rdo_MitoGenome             -ATGCAATTCTTCGATCAATCCCTAATAAATTAGGAGGAGTAATTGCACTAATTATATCA   11022
m141101_iid=614933_0       ------------------------------------------------------------   9134
```

cytB

```
m141031_iid=347546_0       ATCGCAATTTTATTTATTATTCTCTTTAACTAACAAGAGAAAATTCCAAAGAATAAATTT   5774
m150506_iid=3127414_0      ATCGCAATTTTATTTATTATTCCT-TTAAC-TAAAAGAGAAAATTCCAAAGAATAAATTT   7519
m141102_iid=1245517_0      ------------------------------------------------------------   7295
m141107_iid=1405727_0      ATCGCAATTTTATTTATTATTCCT-TTAACTAACAAGAGAAAATTCCAAAGAATAAATTT   6343
m141102_iid=987614_0       ATCGCAATTTTATTTATTATTCCT-TTAACTAACAAGAGAAAATTCCAAAGAATAAA-TT   9344
m141108_iid=1701429_0      ATCGCAATTTTATTTATTATTCCT-TTAACTAACAAGAGAAAATTCCAAAGAATAAATTT   6073
m141102_iid=987286_0       ATCGCAATTTTATTTATTATTCCT-TTAAC----TAGAGAAAATTCCAAAGAATAAATTT   7288
m150430_iid=3272125_0      ATCGCAATTTTATTTATTATTCCT-TTAACTAACAAGAGAAAATTCCAAAGAATAAATTT   8808
m141102_iid=754225_0       ATCGCAATTTTATTTATTATTCCT-TTAACTAACAA------------------------   9952
m141107_iid=1409934_0      ------------------------------------------------------------   10262
m150503_iid=3416920_0      ATCGCAATTTTATTTATTATTCCT-TTAACTAACAAGA--AAATTCCAAAGAATAAATTT   9240
m141108_iid=1800121_0      ATCGCAATTTTATTTATTATTCCT-TTAACTAACAAGAGAAA-TTCCAAAGAATAAATTT   9048
m141031_iid=147540_0       ATCGCAATTTTATTTATTATTCCT-TTAACTAACAAGAGAAAATTCCAAAGAATAAATTT   9409
m150507_iid=3674924_0      ATCGCAATTTTATTTATTATTCCT-TTAACTAACAAGAGAAAATTCCAAAGAATAAATTT   9557
m150503_iid=2923751_0      ATCGCAATTTTATTTATTATTCCT-TTAACTAACAAGAGAAAATTCCAAAGAATAAATTT   9383
m141031_iid=115118_0       ------------------------------------------------------------   7981
m150430_iid=2722448_0      ATCGCAATTTTATTTATTATTCCT-TTAACTAACAAGAGAAAATTCCAAAGAATAAATTT   8180
m150507_iid=3102647_0      ATCGCAATTTTATTTATTATTCCT-TTAACTAACAAGAGAAAATTCCAAAGAATAAATTT   6485
m141108_iid=1811866_0      ATCGCAATTTATTA--TTA-TTCC-TTAACTAACAAGAGAAAATTCCAAAGAATAAATTT   8366
```

```
m150501_iid=2948495_0      ATCGCAATTTTATTTATTATTCCT-TTAACTAACAAGAGAAAATTCCAAAGAATAAATTT   7780
m150504_iid=3238195_0      ATCGCAATTTTATTTATTATTCCT-TTAACT-ACAAGAGAAAATTCCAAAGAATAAATTT   10122
m150503_iid=3388511_0      ATCGCAATTTTATTTATTATTCCT-TTAACTAACAAGAGAAAATTCCAAAGAATAAATTT   7820
m150505_iid=3777611_0      ATCGCAATTTTATTTATTATTCCT-TTAACTAACAAGAGAAAATTCCAAAGAATAAATTT   9247
m150430_iid=2438833_0      ATCGCAATTTTATTTATTATTCCT-TTAACTAACAAGAGAAAATTCCAAAGAATAAATTT   9578
m141108_iid=1698969_0      ATCGCAATTTTATTTATTATTCCT-TTAACTAACAAGAGAAAATTCCAAAGAATAAATTT   10714
m150430_iid=3059190_0      ATCGCAATTTTATTTATTATTCCT-TTAACTAACAAGAGAAAATTCCAAAGAATAAATTT   6435
m141107_iid=1383067_0      ATCGCAATTTTATTTATTATTCCT-TTAACTAACAAGAGAAAATTCCAAAGAATAAATTT   6432
m141101_iid=597008_0       ATCGCAATTTTATTTATTATTCCT-TTAACTAACAAGAGAAAATTCCAAAGAATAAATTT   3505
m150506_iid=2596496_0      ------------------------------------------------------------   10491
m141102_iid=910051_0       ------------------------------------------------------------   7428
m150505_iid=3784017_0      ATCGCAATTTTATTTATTATTCCT-TTAACTAACAAGAGAAAATTCCAAAGAATAAATTT   9358
m141101_iid=682151_0       ATCGCAATTTTATTTATTATTCCT-TTAACTAACAAGAGAAAATTCCAAAGAATAAATTT   8430
m150501_iid=3748029_0      ATCGCAATTTTATTTATTATTCCT-TTAACTAACAAGAGAAAATTCCAAAGAATAAATTT   8974
m150504_iid=2876272_0      ATCGCAATTTTATTTATTATTCCT-TTAACTAACAAGAGAAAATTCCAAAGAATAAATTT   10830
NC_042820.1                ATCGCAATTTTATTTATTATTCCT-TTAACTAACAAGAGAAAATTCCAAAGAATAAATTT   11081
m141102_iid=1228825_0      ATCGCAATTTTATTTATTATTCCT-TTAACTAACAAGAGAAAATTCCAAAGAATAAATTT   10904
m150504_iid=2876274_0      ATCGCAATTTTATTTATTATTCCT-TTAACTAACAAGAGAAAATTCCAAAGAATAAATTT   10902
m141030_iid=80036_0        ATCGCAATTTTATTTATTATTCCT-TTAACTAACAAGAGAAAATTCCAAAGAATAAATTT   10671
m150506_iid=2596494_0      ------------------------------------------------------------   10983
m150503_iid=2526862_0      ATCGCAATTTTATTTATTATTCCT-TTAACTAACAAGAGAAAATTCCAAAGAATAAATTT   10754
m150506_iid=2567000_0      ATCGCAATTTTATTTATTATTCCT-TTAACTAACAAGAGAAAATTCCAAAGAATAAATTT   8709
m150507_iid=3073271_0      ATCGCAATTTTATTTATTATTCCT-TTAACTAACAAGAGAAAATTCCAAAGAATAAATTT   10009
m150504_iid=3156087_0      ATCGCAATTTTATTTATTATTCCT-TTAACTAACAAGAGAAAATTCCAAAGAATAAATTT   9835
m150505_iid=2498502_0      ATCGCAATTTTATTTATTATTCCT-TTAACTAACAAGAGAAAATTCCAAAGAATAAATTT   7485
m141102_iid=754227_0       ATCGCAATTTTATTTATTATTCCT-TTAACTAACAAGAGAAAATTCCAAAGAATAAATTT   10046
m150507_iid=3469514_0      ------------------------------------------------------------   10241
Rdo_MitoGenome             ATCGCAATTTTATTTATTATTCCT-TTAACTAACAAGAGAAAATTCCAAAGAATAAATTT   11081
m141101_iid=614933_0       ------------------------------------------------------------   9134
```

cytB

```
m141031_iid=347546_0       TTACCCAATAAATAAATTCTTATTTTGATCAATAGTAAGAACAGTAATTTTATT--AAAT   5832
m150506_iid=3127414_0      TTACCCAATAAATAAATTCTT-TTTTGATCAATAGTAAGAACAGTAATTTTATTAAACAT   7578
m141102_iid=1245517_0      ------------------------------------------------------------   7295
m141107_iid=1405727_0      TTACCCAATAAATAAATTCTTATTTTGATCAATAGTAAGAACAGTAATTTTA-TTAACAT   6402
m141102_iid=987614_0       TTACCCAATAAATAAATTCTTATTTTGATCAATAGTAAGAACAGTAATTTTATTTAACAT   9404
m141108_iid=1701429_0      TTACCCAATAAATAAATTCTTATTTTGATCAATAGTAAGAACAGTAATTTTA-TTAACAT   6132
m141102_iid=987286_0       TA-C-CA-ATAAAAAATTCTTATTTTGATCAATAGTAAGAACAGTAATTTTA-TTAACAT   7344
m150430_iid=3272125_0      TTACCCAATAAATAAATTCTTATTTTGATCAATAGTAAGAACAGTAATTTTA-TTAACAT   8867
m141102_iid=754225_0       ------------------------------------------------------------   9952
m141107_iid=1409934_0      ------------------------------------------------------------   10262
m150503_iid=3416920_0      TTACCCAATAAATAAATTCTTATTTTGATCAATAGTAAGAACAGTAATTTTA-TTAACAT   9299
m141108_iid=1800121_0      TTACCCAATAAATAAATTCTTATTTTGATCAATAGTAAGAACAGTAATTTTA-TTAACAT   9107
m141031_iid=147540_0       TTACCCAATAAATAAATTCTTATTTTGATCAATAGTAAGAACAGTAATTTTA-TTAACAT   9468
m150507_iid=3674924_0      TTACCCAATAAATAAATTCTTATTTTGATCAATAGTAAGAACAGTAATTTTA-TTAACAT   9616
m150503_iid=2923751_0      TTACCCAATAAATAAATTCTTATTTTGATCAATAGTAAGAACAGTAATTTTA-TTAACAT   9442
m141031_iid=115118_0       ------------------------------------------------------------   7981
m150430_iid=2722448_0      TTACCCAATAAATAAATTCTTATTTTGATCAATAGTAAGAACAGTAATTTTA-TTAACAT   8239
m150507_iid=3102647_0      TTACCCAATAAATAAATTCTTATTTTGATCAATAGTAAGAACAGTAATTTTA-TTAACAT   6544
m141108_iid=1811866_0      TTACCCAATAAATAAATTCTTATTTTGATCAATAGTAAGAACAGTAATTTTA-TTAACAT   8425
m150501_iid=2948495_0      TTACCCAATAAATAAATTCTTATTTTGATCAATAGTAAGAACAGTAATTTTA-TTAACAT   7839
m150504_iid=3238195_0      TTACCCAATAAATAAATTCTTATTTTGATCAATAGTAAGAACAGTAATT-TA-TTAACAT   10180
m150503_iid=3388511_0      TTACCCAATAAATAAATTCTTATTTTGATCAATAGTAAGAACAGTAATTTTA-TTAACAT   7879
m150505_iid=3777611_0      TTACCCAATAAATAAATTCTTATTTTGATCAATAGTAAGAACAGTAATTTTA-TTAACAT   9306
m150430_iid=2438833_0      TTACCCAATAAATAAATTCTTATTTTGATCAATAGTAAGAACAGTAATTTTA-TTAACAT   9637
m141108_iid=1698969_0      TTACCCAATAAATAAATTCTTATTTTGATCAATAGTAAGAACAGTAATTTTA-TTAACAT   10773
m150430_iid=3059190_0      TTACCCAATAAATAAATTCTTATTTTGATCAATAGTAAGAACAGTAATTTTA-TTAACAT   6494
m141107_iid=1383067_0      TTACCCAATAAATAAATTCTTATTTTGATCAATAGTAAGAACAGTAATTTTA-TTAACAT   6491
m141101_iid=597008_0       TTACCCAATAAATAAATTCTTATTTTGATCAATAGTAAGAACAGTAATTTTA-TTAACAT   3564
m150506_iid=2596496_0      ------------------------------------------------------------   10491
m141102_iid=910051_0       ------------------------------------------------------------   7428
m150505_iid=3784017_0      TTACCCAATAAATAAATTCTTATTTTGATCAATAGTAAGAACAGTAATTTTA-TTAACAT   9417
m141101_iid=682151_0       TTACCCAATAAATAAATTCTTATTTTGATCAATAGTAAGAACAGTAATTTTA-TTAACAT   8489
m150501_iid=3748029_0      TTACCCAATAAATAAATTCTTATTTTGATCAATAGTAAGAACAGTAATTTTA-TTAACAT   9033
m150504_iid=2876272_0      TTACCCAATAAATAAATTCTTATTTTGATCAATAGTAAGAACAGTAATTTTA-TTAACAT   10889
NC_042820.1                TTACCCAGTAAATAAATTCTTATTTTGATCAATAGTAAGAACAGTAATTTTA-TTAACAT   11140
m141102_iid=1228825_0      TTACCCAATAAATAAATTCTTATTTTGATCAATAGTAAGAACAGTAATTTTA-TTAACAT   10963
m150504_iid=2876274_0      TTACCCAATAAATAAATTCTTATTTTGATCAATAGTAAGAACAGTAATTTTA-TTAACAT   10961
m141030_iid=80036_0        TTACCCAATAAATAAATTCTTATTTTGATCAATAGTAAGAACAGTAATTTTA-TTAACAT   10730
m150506_iid=2596494_0      ------------------------------------------------------------   10983
m150503_iid=2526862_0      TTACCCAATAAATAAATTCTTATTTTGATCAATAGTAAGAACAGTAATTTTA-TTAACAT   10813
```

```
m150506_iid=2567000_0      TTACCCAATAAATAAATTCTTATTTTGATCAATAGTAAGAACAGTAATTTTA-TTAACAT   8768
m150507_iid=3073271_0      TTACCCAATAAATAAATTCTTATTTTGATCAATAGTAAGAACAGTAATTTTA-TTAACAT   10068
m150504_iid=3156087_0      TTACCCAATAAATAAATTCTTATTTTGATCAATAGTA-----AGTAATTTTA-TTAACAT   9889
m150505_iid=2498502_0      TTACCCAATAAATAAATTCTTATTTTGATCAATAGTAAGAACAGTAATTTTA-TTAACAT   7544
m141102_iid=754227_0       TTACCCAATAAATAAATTCTTATTTTGATCAATAGTAA-AACAGTAATTTTA-TTA-ACT   10103
m150507_iid=3469514_0      ------------------------------------------------------------   10241
Rdo_MitoGenome             TTACCCAATAAATAAATTCTTATTTTGATCAATAGTAAGAACAGTAATTTTA-TTAACAT   11140
m141101_iid=614933_0       ------------------------------------------------------------   9134
```

cytB

```
m141031_iid=347546_0       GAATTGGAGCACGACCTGTTCAGGACCCTTACATTACAACGGGACAAGTATTAACAATTA   5892
m150506_iid=3127414_0      GAATTGGAGCACGACCTGTTCAGGACCCTTACATTACAACGGGACAAGTATTAACAATTA   7638
m141102_iid=1245517_0      ------------------------------------------------------------   7295
m141107_iid=1405727_0      GAATTGGAGCACGACCTGTTCAGGACCCTTACATTACAACGGGACAAGTATTAACAATTA   6462
m141102_iid=987614_0       GAATTGGAGCACGACCTGTTCAGGACCCTTACATTACAACGGGACAAGTATTAACAATTA   9464
m141108_iid=1701429_0      GAATTGGAGCACGACCTGTTCAGGACCCTTACATTACAACGGGACAAGTATTAACAATTA   6192
m141102_iid=987286_0       GAATTGGAGCACGACCTGTTCAGGACCCTTACATTACAACGG-ACAAGTATTAACAATA-   7402
m150430_iid=3272125_0      GAATTGGAGCACGACCTGTTCAGGACCCTTACATTACAACGGGACAAGTATTAACAATTA   8927
m141102_iid=754225_0       ------------------------------------------------------------   9952
m141107_iid=1409934_0      ------------------------------------------------------------   10262
m150503_iid=3416920_0      GAATTGGAGCACGACCTGTTCAGGACCCTTACATTACAACGGGACAAGTATTAACAATTA   9359
m141108_iid=1800121_0      GAATTGGAGCACGACCTGTTCAGGACCCTTACATTACAACGGGACAAGTATTAACAATTA   9167
m141031_iid=147540_0       GAATTGGAGCACGACCTGTTCAGGACCCTTACATTACAACGGGACAAGTATTAACAATTA   9528
m150507_iid=3674924_0      GAATTGGAGCACGACCTGTTCAGGACCCTTACATTACAACGGGACAAGTATTAA---TTA   9673
m150503_iid=2923751_0      GAATTGGAGCACGACCTGTTCAGGACCCTTACATTACAACGGGACAAGTATTAACAATTA   9502
m141031_iid=115118_0       ------------------------------------------------------------   7981
m150430_iid=2722448_0      GAATTGGAGCACGACCTGTTCAGGACCCTTACATTACAACGGGACAAGTATTAACAATTA   8299
m150507_iid=3102647_0      GAATTGGAGCACGACCTGTTCAGGACCCTTACATTACAACGGGACAAGTATTAACAATTA   6604
m141108_iid=1811866_0      GAATTGGAGCACGACCTGTTCAGGACCCTTACATTACAACGGGACAAGTATTAACAATTA   8485
m150501_iid=2948495_0      GAATTGGAGCACGACCTGTTCAGGACCCTTACATTACAACGGGACAAGTATTAACAATTA   7899
m150504_iid=3238195_0      GAATTGGAGCACGACCTGTTCAGGACCCTTACATTACAACGGGACAAGTATTAACAATTA   10240
m150503_iid=3388511_0      GAATTGGAGCACGACCTGTTCAGGACCCTTACATTACAACGGGACAAGTATTAACAATTA   7939
m150505_iid=3777611_0      GAATTGGAGCACGACCTGTTCAGGACCCTTACATTACAACGGGACAAGTATTAACAATTA   9366
m150430_iid=2438833_0      GAATTGGAGCACGACCTGTTCAGGACCCTTACATTACAACGGGACAAGTATTAACAATTA   9697
m141108_iid=1698969_0      GAATTGGAGCACGACCTGTTCAGGACCCTTACATTACAACGGGACAAGTATTAACAATTA   10833
m150430_iid=3059190_0      GAATTGGAGCACGACCTGTTCAGGACCCTTACATTACAACGGGACAAGTATTAACAATTA   6554
m141107_iid=1383067_0      GAATTGGAGCACGACCTGTTCAGGACCCTTACATTACAACGGGACAAGTATTAACAATTA   6551
m141101_iid=597008_0       GAATTGGAGCACGACCTGTTCAGGACCCTTACATTACAACGGGACAAGTATTAACAATTA   3624
m150506_iid=2596496_0      ------------------------------------------------------------   10491
m141102_iid=910051_0       ------------------------------------------------------------   7428
m150505_iid=3784017_0      GAATTGGAGCACGACCTGTTCAGGACCCTTACATTACAACGGGACAAGTATTAACAATTA   9477
m141101_iid=682151_0       GAATTGGAGCACGACCTGTTCAGGACCCTTACATTACAACGGGACAAGTATTAACAATTA   8549
m150501_iid=3748029_0      GAATTGGAGCACGACCTGTTCAGGACCCTTACATTACAACGGGACAAGTATTAACAATTA   9093
m150504_iid=2876272_0      GAATTGGAGCACGACCTGTTCAGGACCCTTACATTACAACGGGACAAGTATTAACAATTA   10949
NC_042820.1                GAATTGGAGCACGACCTGTTCAGGACCCTTACATTACAACGGGACAAGTATTAACAATTA   11200
m141102_iid=1228825_0      GAATTGGAGCACGACCTGTTCAGGACCCTTACATTACAACGGGACAAGTATTAACAATTA   11023
m150504_iid=2876274_0      GAATTGGAGCACGACCTGTTCAGGACCCTTACATTACAACGGGACAAGTATTAACAATTA   11021
m141030_iid=80036_0        GAATTGGAGCACGACCTGTTCAGGACCCTTACATTACAACGGGACAAGTATTAACAATTA   10790
m150506_iid=2596494_0      ------------------------------------------------------------   10983
m150503_iid=2526862_0      GAATTGGAGCACGACCTGTTCAGGACCCTTACATTACAACGGGACAAGTATTAACAATTA   10873
m150506_iid=2567000_0      GAATTGGAGCACGACCTGTTCAGGACCCTTACATTACAACGGGACAAGTATTAACAATTA   8828
m150507_iid=3073271_0      GAATTGGAGCACGACCTGTTCAGGACCCTTACATTACAACGGGACAAGTATTAACAATTA   10128
m150504_iid=3156087_0      GAATTGGAGCACGACCTGTTCAGGACCCTTACATTACAACGGGACAAGTATTAACAATTA   9949
m150505_iid=2498502_0      GAATTGGAGCACGACCTGTTCAGGACCCTTACATTACAACGGGACAAGTATTAACAATTA   7604
m141102_iid=754227_0       GAATTGGAGCACGACCTGTTCAGGACCCTTACATTACAACGGGACAAGTATAA-CAATTA   10162
m150507_iid=3469514_0      ------------------------------------------------------------   10241
Rdo_MitoGenome             GAATTGGAGCACGACCTGTTCAGGACCCTTACATTACAACGGGACAAGTATTAACAATTA   11200
m141101_iid=614933_0       ------------------------------------------------------------   9134
```

cytB

```
m141031_iid=347546_0       T-TTACTTTTTATATTATATAATTACACCACT-ACACTCAAAATTTGAGATAAAATCTTA   5950
m150506_iid=3127414_0      T-TTACTTTTTATATTATATAATTACACCACTAACACTCAAAATTTGAGATAAAATCTTA   7697
m141102_iid=1245517_0      ------------------------------------------------------------   7295
m141107_iid=1405727_0      TT-TACTTTTTATATTATATAATTAC-ACACTAACACTCAAAATTTGAGATAAAATCTTA   6520
m141102_iid=987614_0       T-TTACTTTTTATATTATATAATTACACCACTAACACTCAAAATTTGAGATAAAA---TA   9520
m141108_iid=1701429_0      T-TTACTTTTTATATTATATAATTACACCACTAACACTCAAAATTTGAGATAAAATCTTA   6251
m141102_iid=987286_0       TTATTCTTTTTATATTATATAATTACACCACTAACACTCAAA-TTTGAGATAAAATCTTA   7461
m150430_iid=3272125_0      TTTTACTTTTTATATTATATAATTACACCACTAACACTCAAAATTTGAGATAAAATCTTA   8987
m141102_iid=754225_0       ------------------------------------------------------------   9952
m141107_iid=1409934_0      ------------------------------------------------------------   10262
m150503_iid=3416920_0      T-TTACTTTTTATATTATATAATTACACCACTAACACTCAAAATTTGAGATAAAATCTTA   9418
```

```
m141108_iid=1800121_0      T-TTACTTTTTATATTATA-TATTACACCACTAACACTCAAAATTTGAGATAAAATCTTA   9225
m141031_iid=147540_0       T-TTACTTTTTATATTATATAATTACACCACTAACACTCAAAATTTGAGATAAAATCTTA   9587
m150507_iid=3674924_0      T-TTACTTTTTATATTATATAATTACACCACTAACACTCAAAATTTGAGATAAAATCTTA   9732
m150503_iid=2923751_0      T-TTACTTTTTATATTATATAATTACACCACTAACACTCAAAATTTGAGATAAAATCTTA   9561
m141031_iid=115118_0       ------------------------------------------------------------   7981
m150430_iid=2722448_0      T-TTACTTTTTATATTATATAATTACACCACTAACACTCAAAATTTGAGATAAAATCTTA   8358
m150507_iid=3102647_0      T-TTACTTTTTAT-TTATATAATTACACCACTAACACTCAAAATTTGAGATAAAATCTTA   6662
m141108_iid=1811866_0      T-TTACTTTTTATATTATA-TATTACACCACTAACACTCAAAATTTGAGATAAAATCTTA   8543
m150501_iid=2948495_0      T-TTACTTTTTATATTATATAATTACACCACTAACACTCAAAATTTGAGATAAAATCTTA   7958
m150504_iid=3238195_0      T-TTACTTTTTATATTATATAATTACACCACTAACACTCAAATTT-GAGAT--AAATCTA   10296
m150503_iid=3388511_0      T-TTACTTTTTATATTATATAATTACACCACTAACACTCAAAATTTGAGATAAAATCTTA   7998
m150505_iid=3777611_0      T-TTACTTTTTATATTATATAATTACACCACTAACACTCAAAATTTGAGATAAAATCTTA   9425
m150430_iid=2438833_0      T-TTACTTTTTATATTATATAATTACACCACTAACACTCAAAATTTGAGATAAAATCTTA   9756
m141108_iid=1698969_0      T-TTACTTTTTATATTATATAATTACACCACTAACACTCAAAATTTGAGATAAAA----A   10888
m150430_iid=3059190_0      T-TTACTTTTTATATTATATAATTACACCACTAACACTCAAAATTTGAGATAAAATCTTA   6613
m141107_iid=1383067_0      T-TTACTTTTTATATTATATAATTACACCACTAACACTCAAAATTTGAGATAAAATCTTA   6610
m141101_iid=597008_0       T-TTACTTTTTATATTATATAATTACACCACTAACACTCAAAATTTGAGATAAAATCTTA   3683
m150506_iid=2596496_0      ------------------------------------------------------------   10491
m141102_iid=910051_0       ------------------------------------------------------------   7428
m150505_iid=3784017_0      T-TTACTTTTTATATTATATAATTACACCACTAACACTCAAAATTTGAGATAAAATCTTA   9536
m141101_iid=682151_0       T-TTACTTTTTATA-TATATAATTACACCACTAACACTCAAAATTTGAGATAAAATCTTA   8607
m150501_iid=3748029_0      T-TTACTTTTTATATTATATAATTACACCACTAACACTCAAAATTTGAGATAAAATCTTA   9152
m150504_iid=2876272_0      T-TTACTTTTTATATTATATAATTACACCACTAACACTCAAAATTTGAGATAAAATCTTA   11008
NC_042820.1                T-TTACTTTTTATATTATATAATTACACCACTAACACTCAAAATTTGAGATAAAATCTTA   11259
m141102_iid=1228825_0      T-TTACTTTTTATATTATATAATTACACCACTAACACTCAAAATTTGAGATAAAATCTTA   11082
m150504_iid=2876274_0      T-TTACTTTTTATATTATATAATTACACCACTAACACTCAAAATTTGAGATAAAATCTTA   11080
m141030_iid=80036_0        T-TTACTTTTTATATTATATAATTACACCACTAACACTCAAAATTTGAGATAAAATCTTA   10849
m150506_iid=2596494_0      ------------------------------------------------------------   10983
m150503_iid=2526862_0      T-TTACTTTTTATATTATATAATTACACCACTAACACTCAAAATTTGAGATAAAATCTTA   10932
m150506_iid=2567000_0      T-TTACTTTTTATATTATATAATTACACCACTAACACTCAAAATTTGAGATAAAATCTTA   8887
m150507_iid=3073271_0      T-TTACTTTTTATATTATATAATTACACCACTAACACTCAAAATTTGAGATAAAATCTTA   10187
m150504_iid=3156087_0      T-TTACTTTTTATATTATATAATTACACCACTAACACTCAAAATTTGAGATAAAATCTTA   10008
m150505_iid=2498502_0      T-TTACTTTTTATATTATATAATTACACCACTAACACTCAAAATTTGAGATAAAATCTTA   7663
m141102_iid=754227_0       T-TTACTT-TTATATTATATAATTACACCACTAACACTCAAAATTTGAGATAAAATCTTA   10220
m150507_iid=3469514_0      ------------------------------------------------------------   10241
Rdo_MitoGenome             T-TTACTTTTTATATTATATAATTACACCACTAACACTCAAAATTTGAGATAAAATCTTA   11259
m141101_iid=614933_0       ------------------------------------------------------------   9134
```

cytB

```
m141031_iid=347546_0       ATCAAATAGCTAATGAACTTGAAAAGTATATATTTTGAAA-TATAAAATAGAAGTA-AAA   6008
m150506_iid=3127414_0      ATCAAATAGCTAATGAACTTGAAAAGTATATATTTTGAAAATATAAAATAGAAGTA-AAA   7756
m141102_iid=1245517_0      ------------------------------------------------------------   7295
m141107_iid=1405727_0      ATCAAATAGCTAATGAACTTGAAAAGTATATATTTTGAAAATATAAAATAGAAGTA-AAA   6579
m141102_iid=987614_0       ATCAAATAGCTAATGAACTTGAAAAGTATATATTTTGAAAATATAAAATAGAAGTA-AAA   9579
m141108_iid=1701429_0      ATCAAATAGCTAATGAACTTGAAAAGTATATATTTTGAAAATATAAAATAGAAGTA-AAA   6310
m141102_iid=987286_0       ATCAAATAGCTAATGAACTTGAAAAGTATATA----------------------------   7493
m150430_iid=3272125_0      ATCAAATAGCTAATGAACTTGAAAAGTATATATTTTGAAAATATAAAATAGAAGTA-AAA   9046
m141102_iid=754225_0       ------------------------------------------------------------   9952
m141107_iid=1409934_0      ------------------------------------------------------------   10262
m150503_iid=3416920_0      ATCAAATAGCTAATGAACTTGAAAAGTATATATTTTGAAAATATAAAATAGAAGTA-AAA   9477
m141108_iid=1800121_0      ATCAAATAGCTAATGAACTTGAAAAGTATATATTTTGAAAATATAAAATAGAAGTA-AAA   9284
m141031_iid=147540_0       ATCAAATAGCTAATGAACTTGAAAAGTATATATTTTGAAAATATAAAATAGAAGTA-AAA   9646
m150507_iid=3674924_0      ATCAAATAGCTAATGAACTTGAAAAGTATATATTTTGAAAATATAAAATAGAAGTA-AAA   9791
m150503_iid=2923751_0      ATCAAATAGCTAATGAACTTGAAAAGTATATATTTTGAAAATATAAAATAGAAGTA-AAA   9620
m141031_iid=115118_0       ------------------------------------------------------------   7981
m150430_iid=2722448_0      ATCAAATAGCTAATGAACTTGAAAAGTATATATTTTGAAATA-TAAAATAGAAGT-AAAA   8416
m150507_iid=3102647_0      ATCAAATAGCTAATGAACTTGAAAAGTATATATTTTGAAAATATAAAATAGAAGTA-AAA   6721
m141108_iid=1811866_0      ATCAAATAGCTAATGAACTTGAAAAGTATATATTTTGAAAATATAAAATAGAAGTAAAAT   8603
m150501_iid=2948495_0      ATCAAATAGCTAATGAACTTGAAAAGTATATATTTTGAAAATATAAAATAGAAGTA-AAA   8017
m150504_iid=3238195_0      ATCAAATAGCTAATGAACTTGAAAAGTATATATTTTGAAAATATAAAATAGAAGTA-AAA   10355
m150503_iid=3388511_0      ATCAAATAGCTAATGAACTTGAAAAGTATATATTTTGAAAATATAAAATAGAAGTA-AAA   8057
m150505_iid=3777611_0      ATCAAATAGCTAATGAACTTGAAAAGTATATATTTTGAAAATATAAAATAGAAGTA-AAA   9484
m150430_iid=2438833_0      ATCAAATAGCTAATGAACTTGAAAAGTATATATTTTGAAAATATAAAATAGAAGTA-AAA   9815
m141108_iid=1698969_0      ATCAAATAGCTAATGAACTTGAAAAGTATATATTTTGAAAATATAAAATAGAAGTA-AAA   10947
m150430_iid=3059190_0      ATCAAATAGCTAATGAACTTGAAAAGTATATATTTTGAAAATATAAAATAGAAGTA-AAA   6672
m141107_iid=1383067_0      ATCAAATAGCTAATGAACTTGAAAAGTATATATTTTGAAAATATAAAATAGAAGTA-AAA   6669
m141101_iid=597008_0       ATCAAATAGCTAATGAACTTGAAAAGTATATATTTTGAAAATATAAAATAGAAGTA-AAA   3742
m150506_iid=2596496_0      ------------------------------------------------------------   10491
m141102_iid=910051_0       ------------------------------------------------------------   7428
m150505_iid=3784017_0      ATCAAATAGCTAATGAACTTGAAAAGTATATATTTTGAAAATATAAAATAGAAGTA-AAA   9595
m141101_iid=682151_0       ATCAAATAGCTAATGAACTTGAAAAGTATATATTTTGAAAATATAAAATAGAAGTA-AAA   8666
```

```
m150501_iid=3748029_0      ATCAAATAGCTAATGAACTTGAAAAGTATATATTTTGAAAATATAAAATAGAAGTA-AAA   9211
m150504_iid=2876272_0      ATCAAATAGCTAATGAACTTGAAAAGTATATATTTTGAAAATATAAAATAGAAGTA-AAA   11067
NC_042820.1                ATCAAATAGCTAATGAACTTGAAAAGTATATATTTTGAAAATATAAAATAGAAGTA-AAA   11318
m141102_iid=1228825_0      ATCAAATAGCTAATGAACTTGAAAAGTATATATTTTGAAAATATAAAATAGAAGTA-AAA   11141
m150504_iid=2876274_0      ATCAAATAGCTAATGAACTTGAAAAGTATATATTTTGAAAATATAAAATAGAAGTA-AAA   11139
m141030_iid=80036_0        ATCAAATAGCTAATGAACTTGAAAAGTATATATTTTGAAAATATAAAATAGAAGTA-AAA   10908
m150506_iid=2596494_0      ------------------------------------------------------------   10983
m150503_iid=2526862_0      ATCAAATAGCTAATGAACTTGAAAAGTATATATTTTGAAAATATAAAATAGAAGTA-AAA   10991
m150506_iid=2567000_0      ATCAAATAGCTAATGAACTTGAAAAGTATATATTTTGAAAATATAAAATAGAAGTA-AAA   8946
m150507_iid=3073271_0      ATCAAATAGCTAATGAACTTGAAAAGTATATATTTTGAAAATATAAAATAGAAGTA-AAA   10246
m150504_iid=3156087_0      ATCAAATAGCTAATGAACTTGAAAAGTATATATTTTGAAAATATAAAATAGAAGTA-AAA   10067
m150505_iid=2498502_0      ATCAAATAGCTAATGAACTTGAAAAGTATATATTTTGAAAATATAAAATAGAAGTA-AAA   7722
m141102_iid=754227_0       ATCAAATAGCTAATGAACTTGAAAAGTATATATT--------------------------   10254
m150507_iid=3469514_0      ------------------------------------------------------------   10241
Rdo_MitoGenome             ATCAAATAGCTAATGAACTTGAAAAGTATATATTTTGAAAATATAAAATAGAAGTA-AAA   11318
m141101_iid=614933_0       ------------------------------------------------------------   9134
                           cytB

m141031_iid=347546_0       TCCTCTATTAGCTTATACTAAATTTTATT--AACTAAATAATAAAAACCAACAAAGAGTCT   6066
m150506_iid=3127414_0      TCCTCTATTAGCTTATACTAAATTTTATATAACTAAATAATAAAAC---CCAAAGAGTCT   7813
m141102_iid=1245517_0      ------------------------------------------------------------   7295
m141107_iid=1405727_0      TCCTCTATTAGCTTATACTAAATTTTATTAACTAAATAATAAA-AACCAACAAAGAGTCT   6638
m141102_iid=987614_0       TCCTCTATTAGCTTATACTAAATTTTATTAACTAAATAATAAAAACCCAACAAAGAGTCT   9639
m141108_iid=1701429_0      TCCTCTATTAGCTTATACTAAATTTTATTAACTAAATAATAAAAACCCAACAAAGAGTCT   6370
m141102_iid=987286_0       ------------------------------------------------------------   7493
m150430_iid=3272125_0      TCCTCTATTAGCTTATACTAAATTTTATTAACTAAAT---AATAACCCAACAAAGAGTCT   9103
m141102_iid=754225_0       ------------------------------------------------------------   9952
m141107_iid=1409934_0      ------------------------------------------------------------   10262
m150503_iid=3416920_0      TCCTCTATTAGCTTATACTAAATTTTATTAACTAAATAATAAAAAC-CCACAAAGAGTCT   9536
m141108_iid=1800121_0      TCCTCTATTAGCTTATACTAAATTTTATTAACTAAATAATAAAAACCCAACAAAGAGTCT   9344
m141031_iid=147540_0       TCCTCTATTAGCTTATACTAAATTTTATTAACTAAATAATAAAAACCCAACAAAGAGTCT   9706
m150507_iid=3674924_0      TCCTCTATTAGCTTATACTAAATTTTATTAACTAAATAAT-AAAACCCAACAAAGAGTCT   9850
m150503_iid=2923751_0      TCCTCTATTAGCTTATACTAAATTTTATTAACTAAATAATAAAAACCCAACAAAGAGTCT   9680
m141031_iid=115118_0       ------------------------------------------------------------   7981
m150430_iid=2722448_0      TCCTCTATTAGCTTATACTAAATTTTATTAACTAAATAAT-AAAACCCAACAAAGAGTCT   8475
m150507_iid=3102647_0      TCCTCTATTAGCTTATACTAAATTTTATTAACTAAATAATAAAAACCCAACAAAGAGTCT   6781
m141108_iid=1811866_0      TCCTCTATTAGCTTATACTAAATTTTATTAACTAAATAATAAAAACCCAACAAAGAGTCT   8663
m150501_iid=2948495_0      TCCTCTATTAGCTTATACTAAATTTTATTAACTAAATAATAAAAACCCAACAAAGAGTCT   8077
m150504_iid=3238195_0      TCCTCTATTAGCTTATACTAAATTTTATTAACTAAATAATAAAAACCCAACAAAGAGTCT   10415
m150503_iid=3388511_0      TCCTCTATTAGCTTATACTAAATTTTATTAACTAAATAATAAAAACCCAACAAAGAGTCT   8117
m150505_iid=3777611_0      TCCTCTATTAGCTTATACTAAATTTTATTAACTAAATAATAAAAACCCAACAAAGAGTCT   9544
m150430_iid=2438833_0      TCCTCTATTAGCTTATACTAAATTTTATTAACTAAATAAT-AAAACCCAACAAAGAGTCT   9874
m141108_iid=1698969_0      TCCTCTATTAGCTTATACTAAATTTTATTAACTAAATAATAAAAACCCAACAAAGAGTCT   11007
m150430_iid=3059190_0      TCCTCTATTAGCTTATACTAAATTTTATTAACTAAATAATAAAAACCCAACAAAGAGTCT   6732
m141107_iid=1383067_0      TCCTCTATTAGCTTATACTAAATTTTATTAACTAAATAATAAAAACCCAACAAAGAGTCT   6729
m141101_iid=597008_0       TCCTCTATTAGCTTATACTAAATTTTATTAACTAAATAATAAAAACCCAACAAAGAGTCT   3802
m150506_iid=2596496_0      ------------------------------------------------------------   10491
m141102_iid=910051_0       ------------------------------------------------------------   7428
m150505_iid=3784017_0      TCCTCTATTAGCTTATACTAAATTTTATTAACTAAATAATAAAAACCCAACAAAGAGTCT   9655
m141101_iid=682151_0       TCCTCTATTAGCTTATACTAAATTTTATTAACTAAATAATAAAAACCCAACAAAGAGTCT   8726
m150501_iid=3748029_0      TCCTCTATTAGCTTATACTAAATTTTATTAACTAAATAATAAAAACCCAACAAAGAGTCT   9271
m150504_iid=2876272_0      TCCTCTATTAGCTTATACTAAATTTTATTAACTAAATAATAAAAACCCAACAAAGAGTCT   11127
NC_042820.1                TCCTCTATTAGCTTATACTAAATTTTATTAACTAAATAATAAAAACCCAACAAAGAGTCT   11378
m141102_iid=1228825_0      TCCTCTATTAGCTTATACTAAATTTTATTAACTAAATAATAAAAACCCAACAAAGAGTCT   11201
m150504_iid=2876274_0      TCCTCTATTAGCTTATACTAAATTTTATTAACTAAATAATAAAAACCCAACAAAGAGTCT   11199
m141030_iid=80036_0        TCCTCTATTAGCTTATACTAAATTTTATTAACTAAATAATAAAAACCCAACAAAGAGTCT   10968
m150506_iid=2596494_0      ------------------------------------------------------------   10983
m150503_iid=2526862_0      TCCTCTATTAGCTTATACTAAATTTTATTAACTAAATAATAAAAACCCAACAAAGAGTCT   11051
m150506_iid=2567000_0      TCCTCTATTAGCTTATACTAAATTTTATTAACTAAATAATAAAAACCCAACAAAGAGTCT   9006
m150507_iid=3073271_0      TCCTCTATTAGCTTATACTAAATTTTATTAACTAAATAATAAAAACCCAACAAAGAGTCT   10306
m150504_iid=3156087_0      TCCTCTATTAGCTTATACTAAATTTTATTAACTA---AATAAAAACCCAACAAAGAGTCT   10124
m150505_iid=2498502_0      TCCTCTATTAGCTTATACTAAATTTTATTAACTAAATAATAAAAACCCAACAAAGAGTCT   7782
m141102_iid=754227_0       ------------------------------------------------------------   10254
m150507_iid=3469514_0      ------------------------------------------------------------   10241
Rdo_MitoGenome             TCCTCTATTAGCTTATACTAAATTTTATTAACTAAATAATAAAAACCCAACAAAGAGTCT   11378
m141101_iid=614933_0       ------------------------------------------------------------   9134
                                                                       nad1

m141031_iid=347546_0       TAATCCTAAATA-AAACAACAAAAAACATAAAGATACAGGTAAATAAATTTTTC--AAGC   6123
m150506_iid=3127414_0      TTAATCCTAAAT-AAACAAACAAAAACATAAAGATACAGGTAAATAAATTTTTC--AAGC   7870
m141102_iid=1245517_0      ------------------------------------------------------------   7295
```

```
m141107_iid=1405727_0      T--TTCCTAAATAAAACAACAAAAAACATAAAGATACAGGTAAATAAATTTTTC--AAGC   6694
m141102_iid=987614_0       TTAATCCTAAATAAAACAACAAAAAACATAAAGATACAGGTAAATAAATTTTC---AAGC   9696
m141108_iid=1701429_0      TTAATCCTAAATAAAACAACAAAAAACATAAAGATACAGGTAAATAAATTTTTC--AAGC   6428
m141102_iid=987286_0       ------------------------------------------------------------   7493
m150430_iid=3272125_0      TTAATCCTAAATAAAACAACAAAAAACATAAAGATACAGGTAAATAAATTTTTC--AAGC   9161
m141102_iid=754225_0       ------------------------------------------------------------   9952
m141107_iid=1409934_0      ------------------------------------------------------------   10262
m150503_iid=3416920_0      TTAATCCTAAATAAAACAAACAA-AAAATAAAGATACAGGTAAATAAAT--TTTTCAAGC   9593
m141108_iid=1800121_0      TTAATCCTAAATAAAACAACAAAAAT--AAAAGATACAGGTAAATAATT---TTTCAAGC   9399
m141031_iid=147540_0       TTAATCCTAAATAAAACAACAAAAAACATAAAGATACAGGTAAATAAATTTTTC--AAGC   9764
m150507_iid=3674924_0      TTAATCCTAAATAAAACAACAAAAAACATAAAGATACAGGTAAATAAATTTTTCA--AGC   9908
m150503_iid=2923751_0      TTAATCCTAAATAAAACAACAAAAAACATAAAGATACAGGTAAATAAATTTTTC--AAGC   9738
m141031_iid=115118_0       ------------------------------------------------------------   7981
m150430_iid=2722448_0      TTAATCCTAAATAAAACAACAAAAAAA-CATAAAGACAGGTAAATAAATTTTTC--AAGC   8532
m150507_iid=3102647_0      TTAATCCTAAATAAAACAACAAAAA--ATAAAGATACAGGTAAATAAATTTTTC--AAGC   6837
m141108_iid=1811866_0      TA-ATCCTAAATAAAACAACAAAAAACATAAAGATACAGGTAAATAAATTTTTC--AAGC   8720
m150501_iid=2948495_0      TTAATCCTAAATAAAACAACAA-AAACATAAAGATACAGGTAAATAAATTTTTC--AAGC   8134
m150504_iid=3238195_0      TTAATCCTAAATAAAACAAA--AAAACATAAAGATACAGGTAAATAAATTTTTC--AAGC   10471
m150503_iid=3388511_0      TTAATCCTAAATAAAACAACAAAAAACATAAAGATACAGGTAAATAAATTTTTC--AAGC   8175
m150505_iid=3777611_0      TTAATCCTAAATAAAACAACAAAAAACATAAAGATACAGGTAAATAAATTTTTC--AAGC   9602
m150430_iid=2438833_0      TTAATCCTAAATAAAACAACAAAAAACATAAAGATACAGGTAAATAAATTTTTC--AAGC   9932
m141108_iid=1698969_0      TTAATCCTAAATAAAACAAC-AAAAACATAAAGATACAGGTAAATAAATTTTTC--AAGC   11064
m150430_iid=3059190_0      TTAATCCTAAATAAAACAACAAAAAACATAAAGATACAGGTAAATAAATTTTTC--AAGC   6790
m141107_iid=1383067_0      TTAATCCTAAATAAAACAC-AAAAAACATAA-AGTACAGGTAAATAAATTTTTCAAAAGC   6787
m141101_iid=597008_0       TTAATCCTAAATAAAACAACAAAAAACATAAAGATACAGGTAAATAAATTTTTC--AAGC   3860
m150506_iid=2596496_0      ------------------------------------------------------------   10491
m141102_iid=910051_0       ------------------------------------------------------------   7428
m150505_iid=3784017_0      TTAATCCTAAATAAAACAACAAAAAACATAAAGATACAGGTAAATAAATTTTTC--AAGC   9713
m141101_iid=682151_0       TTAATCCTAAATAAAACAACAAAAAACATAAAGATACAGGTAAATAAATTTTTC--AAGC   8784
m150501_iid=3748029_0      TTAATCCTAAATAAAACAACAAAAAACATAAAGATACAGGTAAATAAATTTTTC--AAGC   9329
m150504_iid=2876272_0      TTAATCCTAAATAAAACAACAAAAAACATAAAGATACAGGTAAATAAATTTTTC--AAGC   11185
NC_042820.1                TTAATCCTAAATAAAACAACAAAAAACATAAAGATACAGGTAAATAAATTTTTC--AAGC   11436
m141102_iid=1228825_0      TTAATCCTAAATAAAACAACAAAAAACATAAAGATACAGGTAAATAAATTTTTC--AAGC   11259
m150504_iid=2876274_0      TTAATCCTAAATAAAACAACAAAAAACATAAAGATACAGGTAAATAAATTTTTC--AAGC   11257
m141030_iid=80036_0        TTAATCCTAAATAAAACAACAAAAAACATAAAGATACAGGTAAATAAATTTTTC--AAGC   11026
m150506_iid=2596494_0      ------------------------------------------------------------   10983
m150503_iid=2526862_0      TTAATCCTAAATAAAACAACAAAAAACATAAAGATACAGGTAAATAAATTTTTC--AAGC   11109
m150506_iid=2567000_0      TTAATCCTAAATAAAACAACAAAAAACATAAAGATACAGGTAAATAAATTTTTC--AAGC   9064
m150507_iid=3073271_0      TTAATCCTAAATAAAACAACAAAAAACATAAAGATACAGGTAAATAAATTTTTC--AAGC   10364
m150504_iid=3156087_0      TTAATCCTAAATAAAACAACAAAAAACATAAAGATACAGGTAAATAAA-TTTTC--AAGC   10181
m150505_iid=2498502_0      TTAATCCTAAATAAAAC-ACAAAAAACATAAAGATACAGGTAAATAAATTTTTC--AAGC   7839
m141102_iid=754227_0       ------------------------------------------------------------   10254
m150507_iid=3469514_0      ------------------------------------------------------------   10241
Rdo_MitoGenome             TTAATCCTAAATAAAACAACAAAAAACATAAAGATACAGGTAAATAAATTTTTC--AAGC   11436
m141101_iid=614933_0       ------------------------------------------------------------   9134

                                            nad1

m141031_iid=347546_0       TAAATATATCAATTTATCATAACGATAACGG-GAAGAGTACCTCGAACCCAAACCCATAA   6182
m150506_iid=3127414_0      TAAATATATCAATTTATCATAACGATAACGGGGAAGAGTACCTCGAACCCAAACCCATAA   7930
m141102_iid=1245517_0      ------------------------------------------------------------   7295
m141107_iid=1405727_0      TAAATATATCAATTTATCATAACGATAACGGGGAAGAGTACCTCGAACCAAA-CCCATAA   6753
m141102_iid=987614_0       TAAATATATCAATTTATCATAACGATAACGGGGAAGAGTACCTCGAACCCAAACCCATAA   9756
m141108_iid=1701429_0      TAAATATATCAATTTATCATAACGATAACGGGGAAGAGTACCTCGAACCCAAACCCATAA   6488
m141102_iid=987286_0       ------------------------------------------------------------   7493
m150430_iid=3272125_0      TAAATATATCAATTTATCATAACGATAACGGGGAAGAGTACCTCGAACCCAAACCCATAA   9221
m141102_iid=754225_0       ------------------------------------------------------------   9952
m141107_iid=1409934_0      ------------------------------------------------------------   10262
m150503_iid=3416920_0      TAAATATATCAATTTATCATAACGATAACGGGGAAGAGTACCTCGAACCCAAACCATAAA   9653
m141108_iid=1800121_0      TAAATATATCAATTTATCATAACGATAACGGGGAAGAGTACCTCGAACCCAAACCACATA   9459
m141031_iid=147540_0       TAAATATATCAATTTATCATAACGATAACGGGGAAGAGTACCTCGAACCCAAACCCATAA   9824
m150507_iid=3674924_0      T-AATATATCAATTTATCATAACGATAACGGGGAAGAGTACCTCGAACCCAAACCCATAA   9967
m150503_iid=2923751_0      TAAATATATCAATTTATCATAACGATAACGGGGAAGAGTACCTCGAACCCAAACCCATAA   9798
m141031_iid=115118_0       ------------------------------------------------------------   7981
m150430_iid=2722448_0      TAAATATATCAATTTATCATAACGATAACGGGGAAGAGTACCTCGAACCCAAACCCATAA   8592
m150507_iid=3102647_0      TAAATATATCAATTTATCATAACGATAACGGGGAAGAGTACCTCGAACCCAAACCCATAA   6897
m141108_iid=1811866_0      TAAATATATCAATTTATCATAACGATAACGGGGAAGAGTACCTCGAACCCAAACCCATAA   8780
m150501_iid=2948495_0      TAAATATATCAATTTATCATAACGATAACGGGGAAGAGTACCTCGAACCCAAACCCATAA   8194
m150504_iid=3238195_0      TAAATATATCAATTTATCATAACGATAACGGGGAAGAGTACCTCGAACCCAAACCCATAA   10531
m150503_iid=3388511_0      TAAATATATCAATTTATCATAACGATAACGGGGAAGAGTACCTCGAACCCAAACCCATAA   8235
m150505_iid=3777611_0      TAAATATATCAATTTATCATAACGATAACGGGGAAGAGTACCTCGAACCCAAACCCATAA   9662
m150430_iid=2438833_0      TAAATATATCAATTTATCATAACGATAACGGGGAAGAGTACCTCGAACCCAAACCCATAA   9992
```

```
m141108_iid=1698969_0      TAAATATATCAATTTATCATAACGATAACGGGGAAGAGTACCTCGAACCCAAACCCAT-A   11123
m150430_iid=3059190_0      TAAATATATCAATTTATCATAACGATAACGGGGAAGAGTACCTCGAACCCAAACCCATAA   6850
m141107_iid=1383067_0      TAAATATATCAATTTATCATAACGATAAAGGGGAAGAGTACCTCGAACCCAAACCCATAA   6847
m141101_iid=597008_0       TAAATATATCAATTTATCATAACGATAACGGGGAAGAGTACCTCGAACCCAAACCCATAA   3920
m150506_iid=2596496_0      ------------------------------------------------------------   10491
m141102_iid=910051_0       ------------------------------------------------------------   7428
m150505_iid=3784017_0      TAAATATATCAATTTATCATAACGATAACGGGGAAGAGTACCTCGAACCCAAACCCATAA   9773
m141101_iid=682151_0       TAAATATATCAATTTATCATAACGATAACGGGGAAGAGTACCTCGAACCCAAACCCATAA   8844
m150501_iid=3748029_0      TAAATATATCAATTTATCATAACGATAACGGGGAAGAGTACCTCGAACCCAAACCCATAA   9389
m150504_iid=2876272_0      TAAATATATCAATTTATCATAACGATAACGGGGAAGAGTACCTCGAACCCAAACCCATAA   11245
NC_042820.1                TAAATATATCAATTTATCATAACGATAACGGGGAAGAGTACCTCGAACCCAAACCCATAA   11496
m141102_iid=1228825_0      TAAATATATCAATTTATCATAACGATAACGGGGAAGAGTACCTCGAACCCAAACCCATAA   11319
m150504_iid=2876274_0      TAAATATATCAATTTATCATAACGATAACGGGGAAGAGTACCTCGAACCCAAACCCATAA   11317
m141030_iid=80036_0        TAAATATATCAATTTATCATAACGATAACGGGGAAGAGTACCTCGAACCCAAACCCATAA   11086
m150506_iid=2596494_0      ------------------------------------------------------------   10983
m150503_iid=2526862_0      TAAATATATCAATTTATCATAACGATAACGGGGAAGAGTACCTCGAACCCAAACCCATAA   11169
m150506_iid=2567000_0      TAAATATATCAATTTATCATAACGATAACGGGGAAGAGTACCTCGAACCCAAACCCATAA   9124
m150507_iid=3073271_0      TAAATATATCAATTTATCATAACGATAACGGGGAAGAGTACCTCGAACCCAAACCCATAA   10424
m150504_iid=3156087_0      TAAATATATCAATTTATCATAACGATAACGGGGAAGAGTACCTCGAACCCAAACCCATAA   10241
m150505_iid=2498502_0      TAAATATATCAATTTATCATAACGATAACGGGGAAGAGTACCTCGAACCCAAACCCATAA   7899
m141102_iid=754227_0       ------------------------------------------------------------   10254
m150507_iid=3469514_0      ------------------------------------------------------------   10241
Rdo_MitoGenome             TAAATATATCAATTTATCATAACGATAACGGGGAAGAGTACCTCGAACCCAAACCCATAA   11496
m141101_iid=614933_0       ------------------------------------------------------------   9134

                                                   nad1
                           ----------------------------------------------------------->

m141031_iid=347546_0       AAAGGAAAAAAA--ACAATTTTAATAAAAA---AAAATAAATTTATATATCA--CCACCA   6235
m150506_iid=3127414_0      AAAGGAAAAAAAAAACAATTTTAATAAAAA---AAATAAATTTATATAATCA--CCACCA   7985
m141102_iid=1245517_0      ------------------------------------------------------------   7295
m141107_iid=1405727_0      A-AAGGAAAAAAAAACAATTTTAATAAAAAAAAA----ATAAATTTATATAATCACCACCA   6808
m141102_iid=987614_0       AA-AGGAAAAAAAAACAATTTTAATAAAAAAATAAATT------TATATAATCACCACCA   9809
m141108_iid=1701429_0      AAAG-GAAAAAAAAACAATTTTAATAAAAAAAAA--TAAATT--TATATAATCACCACCA   6543
m141102_iid=987286_0       ------------------------------------------------------------   7493
m150430_iid=3272125_0      AAAG-GAAAAAAAAACAATTTTAATAAAAAAAAA--TAAATT--TATATAATCACCACCA   9276
m141102_iid=754225_0       ------------------------------------------------------------   9952
m141107_iid=1409934_0      ------------------------------------------------------------   10262
m150503_iid=3416920_0      A---GGAA-AAAAAACAATTTTAATAAAAA---AAATAAATTTATAT--AATCACCACCA   9704
m141108_iid=1800121_0      AAAAGGAAAAAAAAA-A-TTTTAATAAAAA--AAAATAAATTTATATAATCACCACACCA   9515
m141031_iid=147540_0       AA-AGGAAAAAAAAACAATTTTAATAAAAAA--AAATAAATTTATATAATCA--CCACCA   9879
m150507_iid=3674924_0      AA-AGGAAAAAAAAACAATTTTAATAAAAAA--AAATAAATTTATATAATCA--CCACCA   10022
m150503_iid=2923751_0      AA-AGGAAAAAAAAACAATTTTAATAAAAAA--AAATAAATTTATATAATCA--CCACCA   9853
m141031_iid=115118_0       ------------------------------------------------------------   7981
m150430_iid=2722448_0      AA-AGGAAAAAAAAACAATTTTAATAAAAAA--AAATAAATTTATATAATCA--CCACCA   8647
m150507_iid=3102647_0      AA-AGGAAAAAAA-ACAATTTTAATAAAAA--AAAATAAATTTATATAATCA--CCACCA   6951
m141108_iid=1811866_0      AA-AGAAAAAA---ACAATTTTAATAAAAA----AAATAAATTTATATATCA--CCACCA   8830
m150501_iid=2948495_0      AA-AGGAAAAAAAAACAATTTTAATAAAAAA--AAATAAATTTATATAATCA--CCACCA   8249
m150504_iid=3238195_0      AA-AGGAAAAAAAAACAATTTTAATAAAAAAAAAAAATAAATTTATATAATCA--CCACCA   10588
m150503_iid=3388511_0      AA-AGGAAAAAAAAACAATTTTAATAAAAAA--AAATAAATTTATATAATCA--CCACCA   8290
m150505_iid=3777611_0      AA-AGGAAAAAAAAACAATTTTAATAAAAAA--AAATAAATTTATATAATCA--CCACCA   9717
m150430_iid=2438833_0      AA-AGGAAAAAAAA--AATTTTAATAAAAAA--AAATAAATTTATATAATCA--CCACCA   10045
m141108_iid=1698969_0      AA-AGGAAAAAAAAACAATTTTAAT-----------------------------------   11147
m150430_iid=3059190_0      AA-AGGAAAAAAAAACAATTTTAATAAAAAA--AAATAAATTTATATAATCA--CCACCA   6905
m141107_iid=1383067_0      AA-AGGAAAAAAAAACAATTTTAATAAAAAA--AAATAAATTTATATAATCA--CCACCA   6902
m141101_iid=597008_0       AA-AGGAAAAAAAAACAATTTTAATAAAAAA--AAATAAATTTATATAATCA--CCACCA   3975
m150506_iid=2596496_0      ------------------------------------------------------------   10491
m141102_iid=910051_0       ------------------------------------------------------------   7428
m150505_iid=3784017_0      AA-AGGAAAAAAAAACAATTTTAATAAAAAA--AAATAAATTTATATAATCA--CCACCA   9828
m141101_iid=682151_0       AA-AGGAAAAAAAAACAATTTTAATAAAAAA--AAATAAATTTATATAATCA--CCACCA   8899
m150501_iid=3748029_0      AA-AGGAAAAAAAAACAATTTTAATAAAAAA--AAATAAATTTATATAATCA--CCACCA   9444
m150504_iid=2876272_0      AA-AGGAAAAAAAAACAATTTTAATAAAAAA--AATAAATTTATAT-AATCA--CCACCA   11299
NC_042820.1                AA-AGGAAAAAAAAACAATTTTAATAAAAAA--AATAAATTTATATAAATCA--CCACCA   11551
m141102_iid=1228825_0      AA-GGAAAAAAAAAACAATTTTAATAAAAAA--AAATAAAATT---TAATCA--CCACCA   11371
m150504_iid=2876274_0      AA-AGGAAAAAAAAACAATTTTAATAAAAAA--AAATAAATTTATATAATCA--CCACCA   11372
m141030_iid=80036_0        AA-AGGAAAAAAAAACAATTTTAATAAAAAA--AAATAAATTTATATAATCA--CCACCA   11141
m150506_iid=2596494_0      ------------------------------------------------------------   10983
m150503_iid=2526862_0      AA-AGGAAAAAAAAACAATTTTAATAAAAAA--AAAATAAATTATATAATCA--CCA--A   11222
m150506_iid=2567000_0      AA-AGGAAAAAAAAACAATTTTAATAAAAAA--AAATAAATTTATATAATCA--CCACCA   9179
m150507_iid=3073271_0      AA-AGGAAAAAAAAACAATTTTAATAAAAAA--AAATAAATTTATATAATCA--CCACCA   10479
m150504_iid=3156087_0      AA-AGGAAAAAAAAACAATTTTAATAAAAAA--AAATAAATTTATATAATCA--CCACCA   10296
m150505_iid=2498502_0      AA-AGGAAAAAAAAACAATTTTAATAAAAAA--AAATAAATTTATATAATCA--CCACCA   7954
m141102_iid=754227_0       ------------------------------------------------------------   10254
```

```
m150507_iid=3469514_0     ------------------------------------------------------------   10241
Rdo_MitoGenome            AA-AGGAAAAAAAAACAATTTTAATAAAAAA--AAATAAATTTATATAATCA--CCACCA   11551
m141101_iid=614933_0      ------------------------------------------------------------   9134
```

nad1

```
m141031_iid=347546_0      AAAAATATTAAAGAACAAATTATTCTTATAAATAAAATACTAGAATATTCAGCAAGAAAA   6295
m150506_iid=3127414_0     AAAAATATTAAAGAACAAATTATTCTTATAAATAAAATACTAGAATATTCAGCAAGAAAA   8045
m141102_iid=1245517_0     ------------------------------------------------------------   7295
m141107_iid=1405727_0     AAAAATATTAAAGAACAAATTATTCTTATAAATA-AAATCTAGAATATTCAGCAAGAAAA   6867
m141102_iid=987614_0      AAAAAT-TTAAAGAACAAATTATTCTTATAAATAAAATACTAGAATATTCAGCAAGAAAA   9868
m141108_iid=1701429_0     AAAAATATTAAAGAACAAATTATTCTTATAAATAAAATACTAGAATATTCAGCAAGAAAA   6603
m141102_iid=987286_0      ------------------------------------------------------------   7493
m150430_iid=3272125_0     AAAAATATTAAAGAACAAATTATTCTTATAAATAAAATACTAGA--ATTCAGCAAGAAAA   9334
m141102_iid=754225_0      ------------------------------------------------------------   9952
m141107_iid=1409934_0     ------------------------------------------------------------   10262
m150503_iid=3416920_0     AAAAATATTAAAGAACAAATTATTCTTATAAAT-AAATACTAGAATATTCAGCAAGAAAA   9763
m141108_iid=1800121_0     AAAAATATTAAAGAACAAATTATTCTTATAAATAAAATACTAGAATATTCAGCAAGAAAA   9575
m141031_iid=147540_0      AAAAATATTAAAGAACAAATTATTCTTATAAATAAAATACTAGAATATTCAGCAAGAAAA   9939
m150507_iid=3674924_0     AAAAATATTAAAGAACAAATTATTCTTATAA-TAAAATACTAGAATATTCAGCAAGAAAA   10081
m150503_iid=2923751_0     AAAAATATTAAAGAACAAATTATTCTTATAATAAAAATACTAGAATATTCAGCAAGAAAA   9913
m141031_iid=115118_0      ------------------------------------------------------------   7981
m150430_iid=2722448_0     AAAAATATTAAAGAACAAATTATTCTTATAAATAAAATACTAGAATATTCAGCAAGAAAA   8707
m150507_iid=3102647_0     AAAAATATTAAAGAACAAATTATTCTTATAAATAAAATACTAGAATATTCAGCAAGAAAA   7011
m141108_iid=1811866_0     AAA-ATATTAAAGAACAAATTATTCTTATAAATAAAATACTAGAATATTCAGCAAGAAAA   8889
m150501_iid=2948495_0     AAAAATATTAAAGAACAAATTATTCTTATAAATAAAATACTAGAATATTCAGCAAGAAAA   8309
m150504_iid=3238195_0     AAAAAA-TTAAAGAACAAATTAT-------------------------------------   10610
m150503_iid=3388511_0     AAAAATATTAAAGAACAAATTATTCTTATAAATAAAATACTAGAATATTCAGCAAGAAAA   8350
m150505_iid=3777611_0     AAAAATATTAAAGAACAAATTATTCTTATAAATAAAATACTAGAATATTCAGCAAGAAAA   9777
m150430_iid=2438833_0     AAAAATATTAAAGAACAAATTATTCTTATAAATAAAATACTAGAATATTCAGCAAGAAAA   10105
m141108_iid=1698969_0     ------------------------------------------------------------   11147
m150430_iid=3059190_0     AAAAATATTAAAGAACAAATTATTCTTATAAATAAAATACTAGAATATTCAGCAAGAAAA   6965
m141107_iid=1383067_0     AAAAATAT-TAAGAACAAATTATTCTTATAAA-TAAATACTAGAATATTCAGCAAGAAAA   6960
m141101_iid=597008_0      AAAAATATTAAAGAACAAATTATTCTTATAAATAAAATACTAGAATATTCAGCAAGAAAA   4035
m150506_iid=2596496_0     ------------------------------------------------------------   10491
m141102_iid=910051_0      ------------------------------------------------------------   7428
m150505_iid=3784017_0     AAAAATATTAAAGAACAAATTATTCTTATAAATAAAATACTAGAATATTCAGCAAGAAAA   9888
m141101_iid=682151_0      AAAAATATTAAAGAACAAATTATTCTTATAAATAAAATACTAGAATATTCAGCAAGAAAA   8959
m150501_iid=3748029_0     AAAAATATTAAAGAACAAATTATTCTTATAAATAAAATACTAGAATATTCAGCAAGAAAA   9504
m150504_iid=2876272_0     AAAAATATTAAAGAACAAATTATTCTTATAAATAAAATACTAGAATATTCAGCAAGAAAA   11359
NC_042820.1               AAAAATATTAAAGAACAAATTATTCTTATAAATAAAATACTAGAATATTCAGCAAGAAAA   11611
m141102_iid=1228825_0     AAAAATATTAAAGAACAAATTATTCTTATAAATAAAATACTAGAATATTCAGCAAGAAAA   11431
m150504_iid=2876274_0     AAAAATATTAAAAA--AAATTATTCTTATAAATAAAATACTAGAATATTCAGCAAGAAAA   11430
m141030_iid=80036_0       AAAAATATTAAAGAACAAATTATTCTTATAAATAAAATACTAGAATATTCAGCAAGAAAA   11201
m150506_iid=2596494_0     ------------------------------------------------------------   10983
m150503_iid=2526862_0     AAAAATATTAAAGAACAAATTATTCTTATAAATAAAATACTAGAATATTCAGCAAGAAAA   11282
m150506_iid=2567000_0     AAAAATATTAAAGAACAAATTATTCTTATAAATAAAATACTAGAATATTCAGCAAGAAAA   9239
m150507_iid=3073271_0     AAAAATATTAAAGAACAAATTATTCTTATAAATAAAATACTAGAATATTCAGCAAGAAAA   10539
m150504_iid=3156087_0     AAAAATATTAAAGAACAAATTATTCTTATAAATAAAATACTAGAATATTCAGCAAGAAAA   10356
m150505_iid=2498502_0     AAAAATATTAAAGAACAAATTATTCTTATAAATAAAATACTAGAATATTCAGCAAGAAAA   8014
m141102_iid=754227_0      ------------------------------------------------------------   10254
m150507_iid=3469514_0     ------------------------------------------------------------   10241
Rdo_MitoGenome            AAAAATATTAAAGAACAAATTATTCTTATAAATAAAATACTAGAATATTCAGCAAGAAAA   11611
m141101_iid=614933_0      ------------------------------------------------------------   9134
```

nad1

```
m141031_iid=347546_0      ATTAAAGCAAATCCACCTCTTCTATACTCAACATTAAAACCAGAGACCAATTCGGATTCA   6355
m150506_iid=3127414_0     ATTAAAGCAAATCCACCTCTTCTATACTCAACATTAAA--AAGAGACCAATTCGGATTCA   8103
m141102_iid=1245517_0     ------------------------------------------------------------   7295
m141107_iid=1405727_0     ATTAAAGCAAATCCACCTCTTCTATACTCAACATTAAAACCAGAGACCAATTCGGATTCA   6927
m141102_iid=987614_0      ATTAAAGCAAATCCACCTCTTCTATACTCAACATTAAAACCAGAGACCAATTCGGATTCA   9928
m141108_iid=1701429_0     ATTAAAGCAAATCCACCTCTTCTATACTCAACATTAAAACCAGAGACCAATTCGGATTCA   6663
m141102_iid=987286_0      ------------------------------------------------------------   7493
m150430_iid=3272125_0     ATTAAAGCAAATCCACCTCTTCTATACTCAACATTAAAACCAGAGACCAATTCGGATTCA   9394
m141102_iid=754225_0      ------------------------------------------------------------   9952
m141107_iid=1409934_0     ------------------------------------------------------------   10262
m150503_iid=3416920_0     ATTAAAGCAAATCCACCTCTTCTATACTCAACATTAAAACCAGAGACCAATTCGGATTCA   9823
m141108_iid=1800121_0     ATTAAAGCAAATCCACCTCTTCTATACTCAACATTAAAACCAGAGACCAATTCGGATTCA   9635
m141031_iid=147540_0      ATTAAAGCAAATCCACCTCTTCTATACTCAACATTAAAACCAGAGACCAATTCGGATTCA   9999
m150507_iid=3674924_0     ATTAAAGCAAATCCACCTCTTCTATACTCAACATTA-AACCAGAGACCAATTCGGATTCA   10140
m150503_iid=2923751_0     ATTAAAGCA-ATCCACCTCTTCTATACTCAACATTAAAACCAGAGACCAATTCGGATTCA   9972
m141031_iid=115118_0      ------------------------------------------------------------   7981
```

```
m150430_iid=2722448_0       ATTAAAGCAAATCCACCTCTTCTATACTCAACATTAAAACCAGAGACCAATTCGGATTCA   8767
m150507_iid=3102647_0       ATTAAAGCAAATCCACCTCTTCTATACTCAACATTAAAACCAGAGACCAATTCGGATTCA   7071
m141108_iid=1811866_0       ATTAAAGCAAATCCACCTCTTCTATACTCAACATTAAAACCAGAGACCAATTCGGATTCA   8949
m150501_iid=2948495_0       ATTAAAGCAAATCCACCTCTTCTATACTCAACATTAAAACCAGAGACCAATTCGGATTCA   8369
m150504_iid=3238195_0       ------------------------------------------------------------   10610
m150503_iid=3388511_0       ATTAAAGCAAATCCACCTCTTCTATACTCAACATTAAAACCAGAGACCAATTCGGATTCA   8410
m150505_iid=3777611_0       ATTAAAGCAAATCCACCTCTTCTATACTCAACATTAAAACCAGAGACCAATTCGGATTCA   9837
m150430_iid=2438833_0       ATTAAAGCAAATCCACCTCTTCTATACTCAACATTAAAACCAGAGACCAATTCGGATTCA   10165
m141108_iid=1698969_0       ------------------------------------------------------------   11147
m150430_iid=3059190_0       ATTAAAGCAAATCCACCTCTTCTATACTCAACATTAAAACCAGAGACCAATTCGGATTCA   7025
m141107_iid=1383067_0       ATTAAAGCAAATCCACCTCTTCTATACTCAACATTAAAAC--GAGACCAATTCGGATTCA   7018
m141101_iid=597008_0        ATTAAAGCAAATCCACCTCTTCTATACTCAACATTAAAACCAGAGACCAATTCGGATTCA   4095
m150506_iid=2596496_0       ------------------------------------------------------------   10491
m141102_iid=910051_0        ------------------------------------------------------------   7428
m150505_iid=3784017_0       ATTAAAGCAAATCCACCTCTTCTATACTCAACATTAAAACCAGAGACCAATTCGGATTCA   9948
m141101_iid=682151_0        ATTAAAGCAAATCCACCTCTTCTATACTCAACATTAAAACCAGAGACCAATTCGGATTCA   9019
m150501_iid=3748029_0       ATTAAAGCAAATCCACCTCTTCTATACTCAACATTAAAACCAGAGACCAATTCGGATTCA   9564
m150504_iid=2876272_0       ATTAAAGCAAATCCACCTCTTCTATACTCAACATTAAAACCAGAGACCAATTCGGATTCA   11419
NC_042820.1                 ATTAAAGCAAATCCACCTCTTCTATACTCAACATTAAAACCAGAGACCAATTCGGATTCA   11671
m141102_iid=1228825_0       ATTAAAGCAAATCCACCTCTTCTATACTCAACATTAAAACCAGAGACCAATTCGGATTCA   11491
m150504_iid=2876274_0       ATTAAAGCAAATCCACCTCTTCTATACTCAACATTAAAACCAGAGACCAATTCGGATTCA   11490
m141030_iid=80036_0         ATTAAAGCAAATCCACCTCTTCTATACTCAACATTAAAACCAGAGACCAATTCGGATTCA   11261
m150506_iid=2596494_0       ------------------------------------------------------------   10983
m150503_iid=2526862_0       ATTAAAGCAAATCCACCTCTTCTATACTCAACATTAAAACCAGAGACCAATTCGGATTCA   11342
m150506_iid=2567000_0       ATTAAAGCAAATCCACCTCTTCTATACTCAACATTAAAACCAGAGACCAATTCGGATTCA   9299
m150507_iid=3073271_0       ATTAAAGCAAATCCACCTCTTCTATACTCAACATTAAAACCAGAGACCAATTCGGATTCA   10599
m150504_iid=3156087_0       ATTAAAGCAAATCCACCTCTTCTATACTCAACATTAAAACCAGAGACCAATTCGGATTCA   10416
m150505_iid=2498502_0       ATTAAAGCAAATCCACCTCTTCTATACTCAACATTAAAACCAGAGACCAATTCGGATTCA   8074
m141102_iid=754227_0        ------------------------------------------------------------   10254
m150507_iid=3469514_0       ------------------------------------------------------------   10241
Rdo_MitoGenome              ATTAAAGCAAATCCACCTCTTCTATACTCAACATTAAAACCAGAGACCAATTCGGATTCA   11671
m141101_iid=614933_0        ------------------------------------------------------------   9134
```

nad1

```
m141031_iid=347546_0        CCCTCAGCAAAATCAAAAGGAGTACGATTAGTTTCTGCTAATATAGAA-ACAAATAAAAT   6414
m150506_iid=3127414_0       CCCTCAGCAAAATCAAAAGGAGTACGATTAGTTTCTGCTAATATAGAA-ACAAATAAAAT   8162
m141102_iid=1245517_0       ------------------------------------------------------------   7295
m141107_iid=1405727_0       CCCTCAGCAAAATCAAAAGGAGTACGATTAGTTTCTGCTAATATAGAA-ACAAATAAAAT   6986
m141102_iid=987614_0        CCCTCAGCAAAATCAAAAGGAGTACGATTAGTTTCTGCTAATATAGAA-ACAAATAAAAT   9987
m141108_iid=1701429_0       CCCTCAGCAAAATCAAAAGGAGTACGATTAGTTTCTGCTAATATAGAA-ACAAATAAAAT   6722
m141102_iid=987286_0        ------------------------------------------------------------   7493
m150430_iid=3272125_0       CCCTCAGCAAAATCAAAAGGAGTACGATTAGTTTCTGCTAATATAGAA-ACAAATAAAAT   9453
m141102_iid=754225_0        ------------------------------------------------------------   9952
m141107_iid=1409934_0       ------------------------------------------------------------   10262
m150503_iid=3416920_0       CCCTCAGCAAAATCA--AGGAGTACGATTAGTTTCTGCTAATATAGAA-ACAAATAAAAT   9880
m141108_iid=1800121_0       CCCTCAGCAAAATCAAAAGGAGTACGATTAGTTTCTGCTAATATAGAA-ACAAATAAAAT   9694
m141031_iid=147540_0        CCCTCAGCAAAATCAAAAGGAGTACGATTAGTTTCTGCTAATATAGAA-ACAAATAAAAT   10058
m150507_iid=3674924_0       CCCTCAGCAAAATCAAAAGGAGTACGATTAGTTTCTGCTAATATAGAA-ACAAATAAAAT   10199
m150503_iid=2923751_0       C-CTCAGCAAAATCAAAAGGAGTACGATTAGTTTCTGCTAATATAGAA-ACAAATAAAAT   10030
m141031_iid=115118_0        ------------------------------------------------------------   7981
m150430_iid=2722448_0       CCCTCAGCAAAATCAAAAGGAGTACGATTAGTTTCTGCTAATATAGAA-ACAAATAAAAT   8826
m150507_iid=3102647_0       CCCTCAGCAAAATCAAAAGGAGTACGATTAGTTTCTGCTAATATAGAA-ACAAATAAAAT   7130
m141108_iid=1811866_0       CCCTCAGCAAAATCAAAAGG-ATACGATTAGTTTCTGCTAATATAGAA-ACAAATAAAAT   9007
m150501_iid=2948495_0       CCCTCAGCAAAATCAAAAGGAGTACGATTAGTTTCTGCTAATATAGAA-ACAAATAAAAT   8428
m150504_iid=3238195_0       ------------------------------------------------------------   10610
m150503_iid=3388511_0       CCCTCAGCAAAATCAAAAGGAGTACGATTAGTTTCTGCTAATATAGAA-ACAAATAAAAT   8469
m150505_iid=3777611_0       CCCTCAGCAAAATCAAAAGGAGTACGATTAGTTTCTGCTAATATAGAA-ACAAATAAAAT   9896
m150430_iid=2438833_0       CCCTCAGCAAAATCAAAAGGAGTACGATTAGTTTCTGCTAATATAGAA-ACAAATAAAAT   10224
m141108_iid=1698969_0       ------------------------------------------------------------   11147
m150430_iid=3059190_0       CCCTCAGCAAAATCAAAAGGAGTACGATTAGTTTCTGCTAATATAGAAAACAAATAAAAT   7085
m141107_iid=1383067_0       CCCTCAGCAAAATCAAAAGGAGTACGATTAGTTTCTGCTAATA-----------------   7061
m141101_iid=597008_0        CCCTCAGCAAAATCAAAAGGAGTACGATTAGTTTCTGCTAATATAGAA-ACAAATAAAAT   4154
m150506_iid=2596496_0       ------------------------------------------------------------   10491
m141102_iid=910051_0        ------------------------------------------------------------   7428
m150505_iid=3784017_0       CCCTCAGCAAAATCAAAAGGAGTACGATTAGTTTCTGCTAATATAGAA-ACAAATAAAAT   10007
m141101_iid=682151_0        CCCTCAGCAAAATCAAAAGGAGTACGATTAGTTTCTGCTAATATAGAA-ACAAATAAAAT   9078
m150501_iid=3748029_0       CCCTCAGCAAAATCAAAAGGAGTACGATTAGTTTCTGCTAATATAGAA-ACAAATAAAAT   9623
m150504_iid=2876272_0       CCCTCAGCAAAATCAAAAGGAGTACGATTAGTTTCTGCTAATATAGAA-ACAAATAAAAT   11478
NC_042820.1                 CCCTCAGCAAAATCAAAAGGAGTACGATTAGTTTCTGCTAATATAGAA-ACAAATAAAAT   11730
m141102_iid=1228825_0       CCCTCAGCAAAATCAAAAGGAGTACGATTAGTTTCTGCTAATATAGAA-ACAAATAAAAT   11550
m150504_iid=2876274_0       CCCTCAGCAAAATCAAAAGGAGTACGATTAGTTTCTGCTAATATAGAA-ACAAATAAAAT   11549
```

```
m141030_iid=80036_0       CCCTCAGCAAAATCAAAAGGAGTACGATTAGTTTCTGCTAATATAGAA-ACAAATAAAAT  11320
m150506_iid=2596494_0     ------------------------------------------------------------  10983
m150503_iid=2526862_0     CCCTCAGCAAAATCAAAAGGAGTACGATTAGTTTCTGCTAATATAGAA-ACAAATAAAAT  11401
m150506_iid=2567000_0     CCCTCAGCAAAATCAAAAGGAGTACGATTAGTTTCTGCTAATATAGAA-ACAAATAAAAT  9358
m150507_iid=3073271_0     CCCTCAGCAAAATCAAAAGGAGTACGATTAGTTTCTGCTAATATAGAA-ACAAATAAAAT  10658
m150504_iid=3156087_0     CCCTCAGCAAAATCAAAAGGAGTACGATTAGTTTCTGCTAATATAGAA-ACAAATAAAAT  10475
m150505_iid=2498502_0     CCCTCAGCAAAATCAAAAGGAGTACGATTAGTTTCTGCTAATATAGAA-ACAAATAAAAT  8133
m141102_iid=754227_0      ------------------------------------------------------------  10254
m150507_iid=3469514_0     ------------------------------------------------------------  10241
Rdo_MitoGenome            CCCTCAGCAAAATCAAAAGGAGTACGATTAGTTTCTGCTAATATAGAA-ACAAATAAAAT  11730
m141101_iid=614933_0      ------------------------------------------------------------  9134
                                                   nad1

m141031_iid=347546_0      TAAACTTAAAGGAAACATCAAA--AAGACAAATCACAAATAT---TTCTGATACTTAAAA  6469
m150506_iid=3127414_0     TAAACTTAAAGGAAACATCAAAAAAAAGACAAATCACAAATATTTATACTTAAACTTAAAA  8222
m141102_iid=1245517_0     ------------------------------------------------------------  7295
m141107_iid=1405727_0     TAAACTTAAAGGAAACATCAAA--AAGACAAATCACAAATAT---TTCTGATACTTAAAA  7041
m141102_iid=987614_0      TAAACTTAAAGGAAACATCAAA--AAGACAAATCACAAATAT---TTCTGATACTTAAAA  10042
m141108_iid=1701429_0     TAAACTTAAAGGAAACATCAAA--AAGACAAATCACAAATAT---TTCTGATACTTAAAA  6777
m141102_iid=987286_0      ------------------------------------------------------------  7493
m150430_iid=3272125_0     TAAACTTAAAGGAAACATCAAA--AAGACAAATCACAAATAT---TTCTGATACTTAAAA  9508
m141102_iid=754225_0      ------------------------------------------------------------  9952
m141107_iid=1409934_0     ------------------------------------------------------------  10262
m150503_iid=3416920_0     TAAACTTAAAGGAAACATCAAA--AAGACAAATCACAAATAT---TTCTGATACTTAAAA  9935
m141108_iid=1800121_0     TAAACTTAAAGGAAACATCAAAA-AAGACAAATCACAAATAT---TTCTGATACTTAAAA  9750
m141031_iid=147540_0      TAAACTTAAAGGAAACATCAAA--AAGACAAATCACAAATAT---TTCTGATACTTAAAA  10113
m150507_iid=3674924_0     TAAACTTAAAGGAAACATCAAA--AAGACAAATCACAAATAT---TTCTGATACTTAAAA  10254
m150503_iid=2923751_0     TAAACTTAAAGGAAACATCAAA--AAGACAAATCACAAATAT---TTCTGATACTTAAAA  10085
m141031_iid=115118_0      ------------------------------------------------------------  7981
m150430_iid=2722448_0     TAAACTTAAAGGAAACATCAAA--AAGACAAATCACAAATAT---TTCTGATACTTAAAA  8881
m150507_iid=3102647_0     TAAACTTAAAGGAAACATCAAA--AAGACAAATCACAAATAT---TTCTGATACTTAAAA  7185
m141108_iid=1811866_0     TAAACTTAAAGGAAACATCAAA--AAGACAAAT---------------------------  9038
m150501_iid=2948495_0     TAAACTTAAAGGAAACATCAAA--AAGACAAATCACAAATAT---TTCTGATACTTAAAA  8483
m150504_iid=3238195_0     ------------------------------------------------------------  10610
m150503_iid=3388511_0     TAAACTTAAAGGAAACATCAAA--AAGACAAATCACAAATAT---TTCTGATACTTAAAA  8524
m150505_iid=3777611_0     TAAACTTAAAGGAAACATCAAA--AAGACAAATCACAAATAT---TTCTGATACTTAAAA  9951
m150430_iid=2438833_0     TAAACTTAAAGGAAACATCAAA--AAGACAAATCACAAATAT---TTCTGATACTT-AAA  10278
m141108_iid=1698969_0     ------------------------------------------------------------  11147
m150430_iid=3059190_0     TAAACTTAAAGGAAACATCAAA--AAGACAAATCACAAATAT---TTCTGATACTTAAAA  7140
m141107_iid=1383067_0     ------------------------------------------------------------  7061
m141101_iid=597008_0      TAAACTTAAAGGAAACATCAAA--AAGACAAATCACAAATAT---TTCTGATACTTAAAA  4209
m150506_iid=2596496_0     ------------------------------------------------------------  10491
m141102_iid=910051_0      ------------------------------------------------------------  7428
m150505_iid=3784017_0     TAAACTTAAAGGAAACATCAAA--AAGACAAATCACAAATAT---TTCTGATACTTAAAA  10062
m141101_iid=682151_0      TAAACTTAAAGGAAACATCAAA--AAGACAAATCACAAATAT---TTCTGATACTTAAAA  9133
m150501_iid=3748029_0     TAAACTTAAAGGAAACATCAAA--AAGACAAATCACAAATAT---TTCTGATACTTAAAA  9678
m150504_iid=2876272_0     TAAACTTAAAGGAAACATCAAA--AAGACAAATCACAAATAT---TTCTGATACTT-AAA  11532
NC_042820.1               TAAACTTAAAGGAAACATCAAA--AAGACAAATCACAAATAT---TTCTGATACTTAAAA  11785
m141102_iid=1228825_0     TAAACTTAAAGGAAACATCAAA--AAGACAAATCACAAATAT---TTCTGATACTT-AAA  11604
m150504_iid=2876274_0     TAAACTTAAAGGAAACATCAAA--AAGACAAATCACAAATAT---TTCTGATACTT-AAA  11603
m141030_iid=80036_0       TAAACTTAAAGGAAACATCAAA--AAGACAAATCACAAATAT---TTCTGATACTT-AAA  11374
m150506_iid=2596494_0     ------------------------------------------------------------  10983
m150503_iid=2526862_0     TAAACTTAAAGGAAACATCAAA--AAGACAAATCACAAATAT---TTCTGATACTTAAAA  11456
m150506_iid=2567000_0     TAAACTTAAAGGAAACATCAAA--AAGACAAATCACAAATAT---TTCTGATACTTAAAA  9413
m150507_iid=3073271_0     TAAACTTAAAGGAAACATCAAA--AAGACAAATCACAAATAT---TTCTGATACTTAAAA  10713
m150504_iid=3156087_0     TAAACTTAAAGGAAACATCAAA--AAGACAAATCACAAATAT---TTCTGATACTTAAAA  10530
m150505_iid=2498502_0     TAAACTTAAAGGAAACATCAAA--AAGACAAATCACAAATAT---TTCTGATACTTAAAA  8188
m141102_iid=754227_0      ------------------------------------------------------------  10254
m150507_iid=3469514_0     ------------------------------------------------------------  10241
Rdo_MitoGenome            TAAACTTAAAGGAAACATCAAA--AAGACAAATCACAAATAT---TTCTGATACTTAAAA  11785
m141101_iid=614933_0      ------------------------------------------------------------  9134
                                                   nad1

m141031_iid=347546_0      AAATCATA-AAAACATAAGCTAGAAG---TTATACTAAAAAAGAAATAATAATAATAACC  6525
m150506_iid=3127414_0     AAATCATA-AAAACATAAGCTAGAAG---TTATAACTAAAAAGAAATAATAATAATAAC-  8277
m141102_iid=1245517_0     ------------------------------------------------------------  7295
m141107_iid=1405727_0     AAATCATA-AAAACATAAGCTAGAAGTTA--TAACTAAAAAAGAAATAATAATAATAACC  7098
m141102_iid=987614_0      AAATCATA-AAAACATAAGCTAGAAGTTATATAACTAAAAAAGAAATAATAATAATAACC  10101
m141108_iid=1701429_0     AAATCATA-AAAACATAAGCTAGAAGT--TATAACTAAAAAAGAAATAATAATAATAACC  6834
m141102_iid=987286_0      ------------------------------------------------------------  7493
m150430_iid=3272125_0     AA-TCA-TAAAAACATAAGCTAGAAGT--TATAACTAAAAAAGAAATAATAATAATAACC  9564
```

```
m141102_iid=754225_0       ------------------------------------------------------------  9952
m141107_iid=1409934_0      ------------------------------------------------------------  10262
m150503_iid=3416920_0      AAATCA-TAAAAACATAAGCTAGAAGT--TATAACTAAAAAA--AATAATAATAATAACC  9990
m141108_iid=1800121_0      AAAATCATAAAAACATAAGCTAGAAGT--TATAACTAAAAAAGAAATAATAATAATAAC-  9807
m141031_iid=147540_0       AAATCATA-AAAACATAAGCTAGAAGT--TATAACTAAAA-AAGAAAAATAATAATAACC  10169
m150507_iid=3674924_0      AA-TCA-TAAAAACATAAGCTAGAAGT--TATAACTAAAA-AGAAATAATAATAATAACC  10309
m150503_iid=2923751_0      AAATCATAAAAAACATAAGCTAGAAGT--TATAACTAAAAAAGAAATAATA---------  10134
m141031_iid=115118_0       ------------------------------------------------------------  7981
m150430_iid=2722448_0      AAATCATA-AAAACATAAGCTAGAAGT--TATAACTAAAA-AGAAATAATAATAATAACC  8937
m150507_iid=3102647_0      AAATCATA-AAAACATAAGCTAGAAGT--TATAACTAAAAAAGAAATAATAATAATAACC  7242
m141108_iid=1811866_0      ------------------------------------------------------------  9038
m150501_iid=2948495_0      AAATCATA-AAAACATAAGCTAGAAGT--TATAACTAAAAA----ATAATAATAATAACC  8536
m150504_iid=3238195_0      ------------------------------------------------------------  10610
m150503_iid=3388511_0      AAATCATA-AAAACATAAGCTAGAAGT--TATAACTAAAAAAGAAATAATAATAATAACC  8581
m150505_iid=3777611_0      AAATCATA-AAAACATAAGCTAGAAGT--TATAACTAAAAAAGAAATAATAATAATAACC  10008
m150430_iid=2438833_0      AAATCATA-AAAACATAAGCTAGAAGT--TATAACTAAAAAAGAAATAATAATAATAACC  10335
m141108_iid=1698969_0      ------------------------------------------------------------  11147
m150430_iid=3059190_0      AAATCATA-AAAACATAAGCTAGAAGT--TATAACTAAAAAAGAAATAATAATAATAACC  7197
m141107_iid=1383067_0      ------------------------------------------------------------  7061
m141101_iid=597008_0       AAATCATA-AAAACATAAGCTAGAAGT--TATAACTAAAAAAGAAATAATAATAATAACC  4266
m150506_iid=2596496_0      ------------------------------------------------------------  10491
m141102_iid=910051_0       ------------------------------------------------------------  7428
m150505_iid=3784017_0      AAATCATA-AAAACATAAGCTAGAAGT--TATAACTAAAAAAGAAATAATAATAATAACC  10119
m141101_iid=682151_0       AAATCATA-AAAACATAAGCTAGAAGT--TATAACTAAAAAAGAAATAATAATAATAACC  9190
m150501_iid=3748029_0      AAATCATA-AAAACATAAGCTAGAAGT--TATAACTAAAAAAGAAATAATAATAATAACC  9735
m150504_iid=2876272_0      AAATCATA-AAAACATAAGCTAGAAGT--TATAACTAAAAAAGAAATAATAATAATAA--  11587
NC_042820.1                AAATCATA-AAAACATAAGCTAGAAGT--TATAACTAAAAAAGAAATAATAATAATAACC  11842
m141102_iid=1228825_0      AAATCATA-AAAACATAAGCTAGAAGT--TATAACTAAAAAAGAAATAATAATAATAACC  11661
m150504_iid=2876274_0      AAATCATA-AAAACATAAGCTAGAAGT--TATAACTAAAAAAGAAATAATAATAATAACC  11660
m141030_iid=80036_0        AAATCATA-AAAACATAAGCTAGAAGT--TATAACTAAAAAAGAAATAATAATAATAACC  11431
m150506_iid=2596494_0      ------------------------------------------------------------  10983
m150503_iid=2526862_0      AAATCATA-AAAACATAAGCTAGAAGT--TATAACTAAAAAAGAAATAATAATAATAACC  11513
m150506_iid=2567000_0      AAATCATA-AAAACATAAGCTAGAAGT--TATAACTAAAAAAGAAATAATAATAATAACC  9470
m150507_iid=3073271_0      AAATCATA-AAAACATAAGCTAGAAGT--TATAACTAAAAAAGAAATAATAATAATAACC  10770
m150504_iid=3156087_0      AAATCATA-AAAACATAAGCTAGAAGT--TATAACTAAAAAAG----AATAATAATAACC  10583
m150505_iid=2498502_0      AAATCATA-AAAACATAAGCTAGAAGT--TATAACTAAAAAAGAAATAATAATAATAACC  8245
m141102_iid=754227_0       ------------------------------------------------------------  10254
m150507_iid=3469514_0      ------------------------------------------------------------  10241
Rdo_MitoGenome             AAATCATA-AAAACATAAGCTAGAAGT--TATAACTAAAAAAGAAATAATAATAATAACC  11842
m141101_iid=614933_0       ------------------------------------------------------------  9134
```

nad1

```
m141031_iid=347546_0       AAACTAACCTCAT-AGGAAATAGTTTGAGCCATAGATCGCAA-AGAACA-ATTATTGAAT  6582
m150506_iid=3127414_0      CAACTAACCTCAT-AGGAAATAGTTTGAGCCATAGATCGCAA-AGAACCAATTATTGAAT  8335
m141102_iid=1245517_0      ------------------------------------------------------------  7295
m141107_iid=1405727_0      AAACTAACCTCAT-AGGAAATAGTTTGAGCCATAGATCGCAAAAGAACCAATTATTGAAT  7157
m141102_iid=987614_0       -AACTAACCTCAT-AGGAAATAGTTTGAGCCATAGATCGCAA-AGAACCAATTATTGAAT  10158
m141108_iid=1701429_0      AAACTAACCTCAT-AGGAAATAGTTTGAGCCATAGATCGCAA-AGAACCAATTATTGAAT  6892
m141102_iid=987286_0       ------------------------------------------------------------  7493
m150430_iid=3272125_0      AAACTAACCTCATAGGAAATAGTTTGAGCCATAGATCGCAA-AGAACCAATTATTGAAT  9623
m141102_iid=754225_0       ------------------------------------------------------------  9952
m141107_iid=1409934_0      ------------------------------------------------------------  10262
m150503_iid=3416920_0      AAACTAACCTCAT-AGGAAATAGTTTGAGCCATAGATCGCAA-AGAACCAATTATTGAAT  10048
m141108_iid=1800121_0      -AACTAACCTCATAAGGAAATAGTTTGAGCCATAGATCGCAA-AGAACAATTATTGAATA  9865
m141031_iid=147540_0       AAACTAACCTCAT-AGGAAATAGTTTGAGCCATAGATCGCAA-AGAACCAATTATTGAAT  10227
m150507_iid=3674924_0      AAACTAACCTCAT-AGGAAATAGTTTGAGCCATAGATCGCAA-AGAACCAATTATTGAAT  10367
m150503_iid=2923751_0      ------------------------------------------------------------  10134
m141031_iid=115118_0       ------------------------------------------------------------  7981
m150430_iid=2722448_0      AAACTAACCTCAT-AGGAAATAGTTTGAGCCATAGATCGCAA-AGAACCAAATTTTGAAT  8995
m150507_iid=3102647_0      AAACTAACCTCAT-AGGAAATAGTTTGAGCCATAGATCGCAA-AGAACCAATTATTGAAT  7300
m141108_iid=1811866_0      ------------------------------------------------------------  9038
m150501_iid=2948495_0      AAACTAACCTCAT-AGGAAATAGTTTGAGCCATAGATCGCAA-AGAACCAATTATTGAAT  8594
m150504_iid=3238195_0      ------------------------------------------------------------  10610
m150503_iid=3388511_0      AAACTAACCTCAT-AGGAAATAGTTTGAGCCATAGATCGCAA-AGAACCAATTATTGAAT  8639
m150505_iid=3777611_0      AAACTAACCTCAT-AGGAAATAGTTTGAGCCATAGATCGCAA-AGAACCAATTATTGAAT  10066
m150430_iid=2438833_0      AAACTAACCTCAT-AGGAAATAGTTTGAGCCATAGATCGCAA-AGAACCAATTATTGAAT  10393
m141108_iid=1698969_0      ------------------------------------------------------------  11147
m150430_iid=3059190_0      AAACTAACCTCAT-AGGAAATAGTTTGAGCCATAGATCGCAA-AGAACCAATTATTGAAT  7255
m141107_iid=1383067_0      ------------------------------------------------------------  7061
m141101_iid=597008_0       AAACTAACCTCAT-AGGAAATAGTTTGAGCCATAGATCGCAA-AGAACCAATTATTGAAT  4324
m150506_iid=2596496_0      ------------------------------------------------------------  10491
```

```
m141102_iid=910051_0        ------------------------------------------------------------   7428
m150505_iid=3784017_0       AAACTAACCTCAT-AGGAAATAGTTTGAGCCATAGATCGCAA-AGAACCAATTATTGAAT   10177
m141101_iid=682151_0        AAACTAACCTCAT-AGGAAATAGTTTGAGCCATAGATCGCAA-AGAACCAATTATTGAAT   9248
m150501_iid=3748029_0       AAACTAACCTCAT-AGGAAATAGTTTGAGCCATAGATCGCAA-AGAACCAATTATTGAAT   9793
m150504_iid=2876272_0       AAACTAACCTCAT-AGGAAATAGTTTGAGCCATAGATCGCAA-AGAACCAATTATTGAAT   11645
NC_042820.1                 AAACTAACCTCAT-AGGAAATAGTTTGAGCCATAGATCGCAA-AGAACCAATTATTGAAT   11900
m141102_iid=1228825_0       AAACTAACCTCAT-AGGAAATAGTTTGAGCCATAGATCGCAA-AGAACCAATTATTGAAT   11719
m150504_iid=2876274_0       AAACTAACCTCAT-AGGAAATAGTTTGAGCCATAGATCGCAA-AGAACCAATTATTGAAT   11718
m141030_iid=80036_0         AAACTAACCTCAT-AGGAAATAGTTTGAGCCATAGATCGCAA-AGAACCAATTATTGAAT   11489
m150506_iid=2596494_0       ------------------------------------------------------------   10983
m150503_iid=2526862_0       AAACTAACCTCAT-AGGAAATAGTTTGAGCCATAGATCGCAA-AGAACCAATTATTGAAT   11571
m150506_iid=2567000_0       AAACTAACCTCAT-AGGAAATAGTTTGAGCCATAGATCGCAA-AGAACCAATTATTGAAT   9528
m150507_iid=3073271_0       AAACTAACCTCAT-AGGAAATAGTTTGAGCCATAGATCGCAA-AGAACCAATTATTGAAT   10828
m150504_iid=3156087_0       AAACTAACCTCAT-AGGAAATAGTTTGAGCCATAGATCGCAA-AGAACCAATTATTGAAT   10641
m150505_iid=2498502_0       AAACTAACCTCAT-AGGAAATAGTTTGAGCCATAGATCGCAA-AGAACCAATTATTGAAT   8303
m141102_iid=754227_0        ------------------------------------------------------------   10254
m150507_iid=3469514_0       ------------------------------------------------------------   10241
Rdo_MitoGenome              AAACTAACCTCAT-AGGAAATAGTTTGAGCCATAGATCGCAA-AGAACCAATTATTGAAT   11900
m141101_iid=614933_0        ------------------------------------------------------------   9134
                                                       nad1
m141031_iid=347546_0        AATTAGAATTAGATGACCAACCAGAAATTATTACAGCATAAACTGAAAGACTTGAAAA--   6640
m150506_iid=3127414_0       AATTAGAATTAGATGACCAACCAGAAATTATTAAAGCATAAACTGAAAGACTTGAAAA--   8393
m141102_iid=1245517_0       ------------------------------------------------------------   7295
m141107_iid=1405727_0       AATTAGAATTAGATGACCAACCAGAAATTATTACAGCATAAACTGAAAGACTTGAAAAAC   7217
m141102_iid=987614_0        AATTAGAATTAGATGACCAACCAGAAATTATTACAGCATAAACTGAAAGACTTGAAAAAC   10218
m141108_iid=1701429_0       AATTAGAATTAGATGACCAACCAGAAATTATTACAGCATAAACTGAAAGACTTGAAAAAC   6952
m141102_iid=987286_0        ------------------------------------------------------------   7493
m150430_iid=3272125_0       AATTAGAATTAGATGACCAACCAGAAATTATTACAGCATAAACTGAAAGACTTGAAAAAA   9683
m141102_iid=754225_0        ------------------------------------------------------------   9952
m141107_iid=1409934_0       ------------------------------------------------------------   10262
m150503_iid=3416920_0       AATTAGAATTAGATGAC-AACCAGAAATTATTACAGCATAAACTGAAAGACTTGAAAA--   10105
m141108_iid=1800121_0       AATTAGAATTAGATGACCAACCAGAAATTATTACAGCATAAACTGAA-GACTTGAAA--A   9922
m141031_iid=147540_0        AATTAGAATTAGATGACCAACCAGAAATTATTACAGCATAAACTGAAAGACTTGAAAAA-   10286
m150507_iid=3674924_0       AATTAGAATTAGATGACCAACCAGAAATTATTACAGCATAAACTGAAAGACTTGAAAAAA   10427
m150503_iid=2923751_0       ------------------------------------------------------------   10134
m141031_iid=115118_0        ------------------------------------------------------------   7981
m150430_iid=2722448_0       AATTAGAATTAGATGACCAACCAGAAATTATTACAGCATAAACTGAAAGACTTGAAAAA-   9054
m150507_iid=3102647_0       AATTAGAATTAGATGACCAACCAGAAATTATTACAGCATAAACTGAAAGACTTGAAAAA-   7359
m141108_iid=1811866_0       ------------------------------------------------------------   9038
m150501_iid=2948495_0       AATTAGAATTAGATGACCAACCAGAAATTATTACAGCATAAACTGAAAGACTTGAAAAA-   8653
m150504_iid=3238195_0       ------------------------------------------------------------   10610
m150503_iid=3388511_0       AATTAGAATTAGATGACCAACCAGAAATTATTACAGCATAAACTGAAAGACTTGAAAAA-   8698
m150505_iid=3777611_0       AATTAGAATTAGATGACCAACCAGAAATTATTACAGCATAAACTGAAAGACTTGAAAAA-   10125
m150430_iid=2438833_0       AATTAGAATTAGATGACCAACCAGAAATTATTACAGCATAAACTGAAAGACTTGAAAAA-   10452
m141108_iid=1698969_0       ------------------------------------------------------------   11147
m150430_iid=3059190_0       AATTAGAATTAGATGACCAACCAGAAATTATTACAGCATAAACTGAAAGACTTGAAAAA-   7314
m141107_iid=1383067_0       ------------------------------------------------------------   7061
m141101_iid=597008_0        AATTAGAATTAGATGACCAACCAGAAATTATTACAGCATAAACTGAAAGACTTGAAAAA-   4383
m150506_iid=2596496_0       ------------------------------------------------------------   10491
m141102_iid=910051_0        ------------------------------------------------------------   7428
m150505_iid=3784017_0       AATTAGAATTAGATGACCAACCAGAAATTATTACAGCATAAACTGAAAGACTTGAAAAA-   10236
m141101_iid=682151_0        AATTAGAATTAGATGACCAACCAGAAATTATTACAGCATAAACTGAAAGACTTGAAAAA-   9307
m150501_iid=3748029_0       AATTAGAATTAGATGACCAACCAGAAATTATTACAGCATAAACTGAAAGACTTGAAAAA-   9852
m150504_iid=2876272_0       AATTAGAATTAGATGACCAACCAGAAATTATTACAGCATAAACTGAAAGACTTGAAAAA-   11704
NC_042820.1                 AATTAGAATTAGATGACCAACCAGAAATTATTACAGCATAAACTGAAAGACTTGAAAAA-   11959
m141102_iid=1228825_0       AATTAGAATTAGATGACCAACCAGAAATTATTACAGCATAAACTGAAAGACTTGAAAAA-   11778
m150504_iid=2876274_0       AATTAGAATTAGATGACCAACCAGAAATTATTACAGCATAAACTGAAAGACTTGAAAAA-   11777
m141030_iid=80036_0         AATTAGAATTAGATGACCAACCAGAAATTATTACAGCATAAACTGAAAGACTTGAAAAA-   11548
m150506_iid=2596494_0       ------------------------------------------------------------   10983
m150503_iid=2526862_0       AATTAGAATTAGATGACCAACCAGAAATTATTACAGCATAAACTGAAAGACTTGAAAAA-   11630
m150506_iid=2567000_0       AATTAGAATTAGATGACCAACCAGAAATTATTACAGCATAAACTGAAAGACTTGAAAAA-   9587
m150507_iid=3073271_0       AATTAGAATTAGATGACCAACCAGAAATTATTACAGCATAAACTGAAAGACTTGAAAAA-   10887
m150504_iid=3156087_0       AATTAGAATTAGATGA-CAACCAGAAATTATTACAGCATAAACTGAAAGACTTGAAAAA-   10699
m150505_iid=2498502_0       AATTAGAATTAGATGACCAACCAGAAATTATTACAGCATAAACTGAAAGACTTGAAAAA-   8362
m141102_iid=754227_0        ------------------------------------------------------------   10254
m150507_iid=3469514_0       ------------------------------------------------------------   10241
Rdo_MitoGenome              AATTAGAATTAGATGACCAACCAGAAATTATTACAGCATAAACTGAAAGACTTGAAAAA-   11959
m141101_iid=614933_0        ------------------------------------------------------------   9134
                                                       nad1
```

```
m141031_iid=347546_0       ACATAAAAAAATAATA-TACCTAAATTAAA-AGATAAAAAAAAG--AAAAAAATGGAAAT   6696
m150506_iid=3127414_0      CATAAAAAAAATAATA-TACCTAAATTAAA-AGATAAAAATAAG--A-AAAAATGGAAAT   8448
m141102_iid=1245517_0      ------------------------------------------------------------   7295
m141107_iid=1405727_0      ATAAAAAAA--ATAATATACCTAAATTAA-AAGATAAAAAATAA--GAAAAAATGGAAAT   7272
m141102_iid=987614_0       --ATAAAAAAAATAATATACTAAATTAAA--AGATAAAAAATAAGA--AAAAATGGAAAT   10272
m141108_iid=1701429_0      A-TAAAAAAAAT-AATATACCTAAATTAA--AGATAAAAAATAA-GAAAAAAATGGAAAT   7007
m141102_iid=987286_0       ------------------------------------------------------------   7493
m150430_iid=3272125_0      CATAAAAAAAATAATAATACCTAAATTAA-AAGATAAA---AATAAGAAAAAATGGAAAT   9739
m141102_iid=754225_0       ------------------------------------------------------------   9952
m141107_iid=1409934_0      ------------------------------------------------------------   10262
m150503_iid=3416920_0      CATAAAAAAAAATATA-TACCTAAATTAAAA-GA---TAAAAAGATAAAAAAATGGAAAT   10160
m141108_iid=1800121_0      CATAAAAAAAATAATA-TACCTAAATTAAA-AGATAAAAAATAAG-AAAAAAATGGAAAT   9979
m141031_iid=147540_0       CATAAAAAAAATAATA-TACCTAAATTAAA-AGATAAAAAATAAG-AAAAAAATGGAAAT   10343
m150507_iid=3674924_0      CATAAAAAAAATAATA-TACCTAAATTAAA-AGATAAAAAAT---AAGAAAAATGGAAAT   10482
m150503_iid=2923751_0      ------------------------------------------------------------   10134
m141031_iid=115118_0       ------------------------------------------------------------   7981
m150430_iid=2722448_0      CATAAAAAA-ATAATA-TACCTAAATTAAAAGATAAAAAATAA-GAAAAAAATGGAAAT   9111
m150507_iid=3102647_0      CATAAAAA-AATAATA-TACCTAAATTAAA-AGATAAAAAATAAGA-AAAAAATGGAAAT   7415
m141108_iid=1811866_0      ------------------------------------------------------------   9038
m150501_iid=2948495_0      CATAAAAA-AATAATA-TACCTAAATTAAA-AGATAAAAAATAAG--AAAAAATGGAAAT   8708
m150504_iid=3238195_0      ------------------------------------------------------------   10610
m150503_iid=3388511_0      CATAAAAAAAATAATA-TACCTAAATTAAA-AGATAAAAAATAAG-AAAAAAATGGAAAT   8755
m150505_iid=3777611_0      CAT-AAAAAAATAATA-TACCTAAATTAAA-AGATAAAAAATAAG-AAAAAAATGGAAAT   10181
m150430_iid=2438833_0      CATAAAAAAAATAATA-TACCTAAATTAAA-AGATAAAAAATAAG-AAAAAAATGGAAAT   10509
m141108_iid=1698969_0      ------------------------------------------------------------   11147
m150430_iid=3059190_0      CATAAAAAAAATAATA-TACCTAAATTAAA-AGATAAAAAATAAGA-AAAAAATGGAAAT   7371
m141107_iid=1383067_0      ------------------------------------------------------------   7061
m141101_iid=597008_0       CATAAAAAAAATAATA-TACCTAAATTAAA-AGATAAAAAATAAGA-AAAAAATGGAAAT   4440
m150506_iid=2596496_0      ------------------------------------------------------------   10491
m141102_iid=910051_0       ------------------------------------------------------------   7428
m150505_iid=3784017_0      CAT-AAAAAAATAATA-TACCTAAATTAAA-AGATAAAAAATAAGA--AAAAATGGAAAT   10291
m141101_iid=682151_0       CAT-AAAAAAATAATA-TACCTAAATTAAA-AGATAAAAAATAAG-AAAAAAATGGAAAT   9363
m150501_iid=3748029_0      CAT-AAAAAAATAATA-TACCTAAATTAAA-AGATAAAAAATAAG-AAAAAAATGGAAAT   9908
m150504_iid=2876272_0      CAT-AAAAAAATAATA-TACCTAAATTAAA-AGATAAAAAATAAG-AAAAAAATGGAAAT   11760
NC_042820.1                CATAAAAAAAATAATA-TACCTAAATTAAA-AGATAAAAAATAAG-AAAAAAATGGAAAT   12016
m141102_iid=1228825_0      CATAAAAAAAATAATA-TACCTAAATTAAA-AGATAAAAAATAAGAAAAAAAATGGAAAT   11836
m150504_iid=2876274_0      CATAAAAAAAATAATA-TACCTAAATTAAA-AGATAAAAAATAAG-AAAAAAATGGAAAT   11834
m141030_iid=80036_0        CATAAA------------------------------------------------------   11554
m150506_iid=2596494_0      ------------------------------------------------------------   10983
m150503_iid=2526862_0      CAT-AAAAAAATAATA-TACCTAAATTAAA-AGATAAAAAATAAG-AAAAAAATGGAAAT   11686
m150506_iid=2567000_0      CATAAAAAAAATAATA-TACCTAAATTAAA-AGATAAAAAATAAG-AAAAAAATGGAAAT   9644
m150507_iid=3073271_0      CATAAAAAAAATAATA-TACCTAAATTAAA-AGATAAAAAATAAG-AAAAAAATGGAAAT   10944
m150504_iid=3156087_0      CAT-AAAAAAATAATA-TACCTAAATTAAA-AGATAAAAAATAAG-AAAAAAATGGAAAT   10755
m150505_iid=2498502_0      CATAAAAAAAATAATA-TACCTAAATTAAA-AGATAAAAAATAAG-AAAAAAATGGAAAT   8419
m141102_iid=754227_0       ------------------------------------------------------------   10254
m150507_iid=3469514_0      ------------------------------------------------------------   10241
Rdo_MitoGenome             CATAAAAAAAATAATA-TACCTAAATTAAA-AGATAAAAAATAAG-AAAAAAATGGAAAT   12016
m141101_iid=614933_0       ------------------------------------------------------------   9134
                                                    nad1
                           ----------------------------------------------------------->

m141031_iid=347546_0       CTAAGTCACATTATTAAAGAAATAAAAAAATTTA-TTACAGGAGAAAAATAAT-AAAAAC   6754
m150506_iid=3127414_0      CTAAGTCACATTATTAAAGAAATAAA-AAATTTA-TTACAGGAGAAAAATAAT-AAAAAC   8505
m141102_iid=1245517_0      ------------------------------------------------------------   7295
m141107_iid=1405727_0      CTAAGTCACATTATTAAAGAAATAAAAAAAT-TTATTACAGGAG----------------   7315
m141102_iid=987614_0       CTAAGTCACATTATTAAAGAAATAAAAAAATTTA-TTACAGGAGAAAAATAATAAAAAAC   10331
m141108_iid=1701429_0      CTAAGTCACATTATTAAAGAAATAAAAAA-ATTTATTACAGGAGAAAAATAATAAAAAAC   7066
m141102_iid=987286_0       ------------------------------------------------------------   7493
m150430_iid=3272125_0      CTAAGTCACATTATTAAAGAAATAAAAAAATTTATTAACAGGAGAA-AATAATAAAAAAC   9798
m141102_iid=754225_0       ------------------------------------------------------------   9952
m141107_iid=1409934_0      ------------------------------------------------------------   10262
m150503_iid=3416920_0      CTAAGTCACATTATTAAAGAAATAAAAA--------------------------------   10188
m141108_iid=1800121_0      CTAAGTCACATTATTAAAGAAATAAAAAAATTTA-TTACAGGAGAAAAAA-ATAAAAAAC   10037
m141031_iid=147540_0       CTAAGTCACATTATTAAAGAAATAAAAAAATTTA-TTACAGGAGAAAAATAATAAAAAAC   10402
m150507_iid=3674924_0      CTAAGTCACATTATTAAAGAAATAAAAAAATTTA-TTACAGGAGAAAAATAAT-AAAAAC   10540
m150503_iid=2923751_0      ------------------------------------------------------------   10134
m141031_iid=115118_0       ------------------------------------------------------------   7981
m150430_iid=2722448_0      CTAAGTCACATTATTAAAGAAATAAAAAAATTTA-TTACAGGAGAAAAATAATAAAAAAC   9170
m150507_iid=3102647_0      CTAAGTCACATTATTAAAGAAATAAAAAAATTTA-TTACAGGAGAAAAATAATAAAAAAC   7474
m141108_iid=1811866_0      ------------------------------------------------------------   9038
m150501_iid=2948495_0      CTAAGTCACATTATTAAAGAAATAAA-AAATTTA-TTACAGGAGAAAAATAATAAAAAAC   8766
m150504_iid=3238195_0      ------------------------------------------------------------   10610
```

```
m150503_iid=3388511_0      CTAAGTCACATTATTAAAGAAATAAAAAAATTTA-TTACAGGAGAAAAATAATAAAAAAC   8814
m150505_iid=3777611_0      CTAAGTCACATTATTAAAGAAATAAAAAAATTTA-TTACAGGAGAAAAATAATAAAAAAC   10240
m150430_iid=2438833_0      CTAAGTCACATTATTAAAGAAATAAAAAAATTTA-TTACAGGAGAAAAATAATAAAAAAC   10568
m141108_iid=1698969_0      ------------------------------------------------------------   11147
m150430_iid=3059190_0      CTAAGTCACATTATTAAAGAAATAAAAAAATTTA-TTACAGGAGAAAAATAATAAAAAAC   7430
m141107_iid=1383067_0      ------------------------------------------------------------   7061
m141101_iid=597008_0       CTAAGTCACATTATTAAAGAAATAAAAAAATTTA-TTACAGGAGAAAAATAATAAAAAAC   4499
m150506_iid=2596496_0      ------------------------------------------------------------   10491
m141102_iid=910051_0       ------------------------------------------------------------   7428
m150505_iid=3784017_0      CTAAGTCACATTATTAAAGAAATAAAAAAATTTA-TTACAGGAGAAAAATAATAAAAAAC   10350
m141101_iid=682151_0       CTAAGTCACATTATTAAAGAAATAAAAAAATTTA-TTACAGGAGAAAAATAATAAAAAAC   9422
m150501_iid=3748029_0      CTAAGTCACATTATTAAAGAAATAAAAAAATTTA-TTACAGGAGAAAAATAATAAAAAAC   9967
m150504_iid=2876272_0      CTAAGTCACATTATTAAAGAAATAAAAAAATTTA-TTACAGGAGAAAAATAATAAAAAAC   11819
NC_042820.1                CTAAGTCACATTATTAAAGAAATAAAAAAATTTA-TTACAGGAGAAAAATAATAAAAAAC   12075
m141102_iid=1228825_0      CTAAGTCACATTATTAAAGAAATAAAAAAATTTA-TTACAGGAGAAAAATAAT-AAAAAC   11894
m150504_iid=2876274_0      CTAAGTCACATTATTAAAGAAATAAAAAAATTTA-TTACAGGAGAAAAATAATAAAAAAC   11893
m141030_iid=80036_0        ------------------------------------------------------------   11554
m150506_iid=2596494_0      ------------------------------------------------------------   10983
m150503_iid=2526862_0      CTAAGTCACATTATTAAAGAAATAAAAAAATTTA-TTACAGGAGAAAAATAATAAAAAAC   11745
m150506_iid=2567000_0      CTAAGTCACATTATTAAAGAAATAAAAAAATTTA-TTACAGGAGAAAAATAATAAAAAA-   9702
m150507_iid=3073271_0      CTAAGTCACATTATTAAAGAAATAAAAAAATTTA-TTACAGGAGAAAAATAATAAAAAAC   11003
m150504_iid=3156087_0      CTAAGTCACATTATTAAAGAAATAAAAAAATTTA-TTACAGGAGAAAAATAATAAAAAAC   10814
m150505_iid=2498502_0      CTAAGTCACATTATTAAAGAAATAAA-AAATTTA-TTACAGGAGAAAAATAATAAAAAAC   8477
m141102_iid=754227_0       ------------------------------------------------------------   10254
m150507_iid=3469514_0      ------------------------------------------------------------   10241
Rdo_MitoGenome             CTAAGTCACATTATTAAAGAAATAAAAAAATTTA-TTACAGGAGAAAAATAATAAAAAAC   12075
m141101_iid=614933_0       ------------------------------------------------------------   9134
```

nad1

```
m141031_iid=347546_0       ATAGTTAGAAACTAAAGGTAAACACTGCTCCTTAGAAAATAATT--TAATAGCATCTCTA   6812
m150506_iid=3127414_0      ATAGTTAGAAACTAAAGGTAAACACTGCTCCTTAGAAAATAATT--TAATAGCATCTCTA   8563
m141102_iid=1245517_0      ------------------------------------------------------------   7295
m141107_iid=1405727_0      ------------------------------------------------------------   7315
m141102_iid=987614_0       ATAGTTAGAAACTAAAGGTAAACACTGCTCCTTGAAAAATAATT--TAATAGCATCTCTA   10389
m141108_iid=1701429_0      ATAGTTAGAAACTAAAGGTAAACACTGCTCCTTAGAAAATAAT--TTAATAGCATCTCTA   7124
m141102_iid=987286_0       ------------------------------------------------------------   7493
m150430_iid=3272125_0      ATAGTTAGAAACTAAAGGTAAACACTGCTCCTTAGAAAATAAT--TTAATAGCATCTCTA   9856
m141102_iid=754225_0       ------------------------------------------------------------   9952
m141107_iid=1409934_0      ------------------------------------------------------------   10262
m150503_iid=3416920_0      ------------------------------------------------------------   10188
m141108_iid=1800121_0      ATAGTTAGAAACTAAAGGTA-AAACTGCTCCTTAGAAAATAATTTAAAATAGCATCTCTA   10096
m141031_iid=147540_0       ATAGTTAGAAACTAAAGGTAAACACTGCTCCTTAGAAAATAATT--TAATAGCATCTCTA   10460
m150507_iid=3674924_0      ATAGTTAGAAACTAAAGGTAAACACTGCTCCTTAGAAAATAATT--TAATAGCATCTCTA   10598
m150503_iid=2923751_0      ------------------------------------------------------------   10134
m141031_iid=115118_0       ------------------------------------------------------------   7981
m150430_iid=2722448_0      ATAGTTAGAAACTAAAGGTAAACACTGCTCCTTAGAAAAA-ATT--TAATAGCATCTCTA   9227
m150507_iid=3102647_0      ATAGTTAGAAACTAAAGGTAAACACTGCTCCTTAGAAAATAATT--TAATAGCATCTCTA   7532
m141108_iid=1811866_0      ------------------------------------------------------------   9038
m150501_iid=2948495_0      ATAGTTAGAAACTAAAGGTAAACACTGCTCCTTAGAAAATAATT--TAATAGCATCTCTA   8824
m150504_iid=3238195_0      ------------------------------------------------------------   10610
m150503_iid=3388511_0      ATAGTTAGAAACTAAAGGTAAACACTGCTCCTTAGAAAATAATT--TAATAGCATCTCTA   8872
m150505_iid=3777611_0      ATAGTTAGAAACTAAAGGTAAACACTGCTCCTTAGAAAATAATT--TAATAGCATCTCTA   10298
m150430_iid=2438833_0      ATAGTTAGAAACTAAAGGTAAACACTGCTCCTTAGAAAATAATT--TAATAGCATCTCTA   10626
m141108_iid=1698969_0      ------------------------------------------------------------   11147
m150430_iid=3059190_0      ATAGTTAGAAACTAAAGGTAAACACTGCTCCTTAGAAAATAATT--TAATAGCATCTCTA   7488
m141107_iid=1383067_0      ------------------------------------------------------------   7061
m141101_iid=597008_0       ATAGTTAGAAACTAAAGGTAAACACTGCTCCTTAGAAAATAATT--TAATAGCATCTCTA   4557
m150506_iid=2596496_0      ------------------------------------------------------------   10491
m141102_iid=910051_0       ------------------------------------------------------------   7428
m150505_iid=3784017_0      ATAGTTAGAAACTAAAGGTAAACACTGCTCCTTAGAAAATAATT--TAATAGCATCTCTA   10408
m141101_iid=682151_0       ATAGTTAGAAACTAAAGGTAAACACTGCTCCTTAGAAAATAATT--TAATAGCATCTCTA   9480
m150501_iid=3748029_0      ATAGTTAGAAACTAAAGGTAAACACTGCTCCTTAGAAAATAATT--TAATAGCATCTCTA   10025
m150504_iid=2876272_0      ATAGTTAGAAACTAAAGGTAAACACTGCTCCTTAGAAAATAATT--TAATAGCATCTCTA   11877
NC_042820.1                ATAGTTAGAAACTAAAGGTAAACACTGCTCCTTAGAAAATAATT--TAATAGCATCTCTA   12133
m141102_iid=1228825_0      ATAGTTAGAAACTAAAGGTAAACACTGCTCCTTAG-AAATAATT--TAATAGCATCTCTA   11951
m150504_iid=2876274_0      ATAGTTAGAAACTAAAGGTAAACACTGCTCCTTAGAAAATAATT--TAATAGCATCTCTA   11951
m141030_iid=80036_0        ------------------------------------------------------------   11554
m150506_iid=2596494_0      ------------------------------------------------------------   10983
m150503_iid=2526862_0      ATAGTTAGAAACTAAAGGTAAACACTGCTCCTTAGAAAATAATT--TAATAGCATCTCTA   11803
m150506_iid=2567000_0      ATAGTTAGAAACTAAAGGTAAACACTGCTCCTTAGAAAATAATT--TAATAGCATCTCTA   9760
m150507_iid=3073271_0      ATAGTTAGAAACTAAAGGTAAACACTGCTCCTTAGAAAATAATT--TAATAGCATCTCTA   11061
```

```
m150504_iid=3156087_0      ATAGTTAGAAACTAAAGGTAAACACTGCTCCTTAGAAAATAATT-------GCATCTCTA   10867
m150505_iid=2498502_0      ATAGTTAGAAACTAAAGGTAAACACTGCTCCTTAGAAAATAATT--TAATAGCATCTCTA   8535
m141102_iid=754227_0       ------------------------------------------------------------   10254
m150507_iid=3469514_0      ------------------------------------------------------------   10241
Rdo_MitoGenome             ATAGTTAGAAACTAAAGGTAAACACTGCTCCTTAGAAAATAATT--TAATAGCATCTCTA   12133
m141101_iid=614933_0       ------------------------         ---------------------------   9134
                                                 nad1

m141031_iid=347546_0       A-AGGTTGTAATAATCCTATAAAACCAAACCTTATTAGGACCT-TTTCGTAATTGA-ATA   6869
m150506_iid=3127414_0      AAAGGTTGTAATAATCCTATAAAACC-AACCTTATTAGGACCT-TTTCGTAATTGA-ATA   8620
m141102_iid=1245517_0      ------------------------------------------------------------   7295
m141107_iid=1405727_0      ------------------------------------------------------------   7315
m141102_iid=987614_0       AAAGGTTGTAATAATCCTAT-AAAACCAACCTTATTAGGACCT-TTTCGTAATTGA-ATA   10446
m141108_iid=1701429_0      AAAGGTTGTAATAATCCTATA-AAACCAACCTTATTAGGACCTT-TTCGTAATTGAA-TA   7181
m141102_iid=987286_0       ------------------------------------------------------------   7493
m150430_iid=3272125_0      AAAGGTTGTAATAATCCTAATAAAACCAACCTTATTAGGACCTT-TTCGTAATTGAA-TA   9914
m141102_iid=754225_0       ------------------------------------------------------------   9952
m141107_iid=1409934_0      ------------------------------------------------------------   10262
m150503_iid=3416920_0      ------------------------------------------------------------   10188
m141108_iid=1800121_0      AAAGGTTGTAATAATCCTAT-AAAACCAACCTTATTAGGACCTTT-TCGTAATTGAAATA   10154
m141031_iid=147540_0       AAAGGTTGTAATAATCCTAT-AAAACCAACCTTATTAGGACCT-TTTCGTAATTGA-ATA   10517
m150507_iid=3674924_0      AAAGGTTGTAATAATCCTTA--AAACCAACCTTATTAGGACCTTTTCGTAATTGA-ATA   10655
m150503_iid=2923751_0      ------------------------------------------------------------   10134
m141031_iid=115118_0       ------------------------------------------------------------   7981
m150430_iid=2722448_0      AAAGGTTGTAATAATCCTAT-AAAACCAACCTTATTAGGACCT-TTTCGTAATTGA-ATA   9284
m150507_iid=3102647_0      AAAGGTTGTAATAATCCTAT-AAAACCAACCTTATTAGGACCT-TTTCGTAATTGA-ATA   7589
m141108_iid=1811866_0      ------------------------------------------------------------   9038
m150501_iid=2948495_0      AAAGGTTGTAATAATCCTAT-AAAACCAACCTTATTAGGACCT-TTTCGTAATTGA-ATA   8881
m150504_iid=3238195_0      ------------------------------------------------------------   10610
m150503_iid=3388511_0      AAAGGTTGTAATAATCCTAT-AAAACCAACCTTATTAGGACCT-TTTCGTAATTGA-ATA   8929
m150505_iid=3777611_0      AAAGGTTGTAATAATCCTAT-AAAACCAACCTTATTAGGACCT-TTTCGTAATTGA-ATA   10355
m150430_iid=2438833_0      AAAGGTTGTAATAATCCTAT-AAAACCAACCTTATTAGGACCT-TTTCGTAATTGA-ATA   10683
m141108_iid=1698969_0      ------------------------------------------------------------   11147
m150430_iid=3059190_0      AAAGGTTGTAATAATCCTAT-AAAACCAACCTTATTAGGACCT-TTTCGTAATTGA-ATA   7545
m141107_iid=1383067_0      ------------------------------------------------------------   7061
m141101_iid=597008_0       AAAGGTTGTAATAATCCTAT-AAAACCAACCTTATTAGGACCT-TTTCGTAATTGA-ATA   4614
m150506_iid=2596496_0      ------------------------------------------------------------   10491
m141102_iid=910051_0       ------------------------------------------------------------   7428
m150505_iid=3784017_0      AAAGGTTGTAATAATCCTAT-AAAACCAACCTTATTAGGACCT-TTTCGTAATTGA-ATA   10465
m141101_iid=682151_0       AAAGGTTGTAATAATCCTAT-AAAACCAACCTTATTAGGACCT-TTTCGTAATTGA-ATA   9537
m150501_iid=3748029_0      AAAGGTTGTAATAATCCTAT-AAAACCAACCTTATTAGGACCT-TTTCGTAATTGA-ATA   10082
m150504_iid=2876272_0      AAAGGTTGTAATAATCCTAT-AAAACCAACCTTATTAGGACCT-TTTCGTAATTGA-ATA   11934
NC_042820.1                AAAGGTTGTAATAATCCTAT-AAAACCAACCTTATTAGGACCT-TTTCGTAATTGA-ATA   12190
m141102_iid=1228825_0      ------------------------------------------------------------   11951
m150504_iid=2876274_0      AAAGGTTGTAATAATCCTAT-AAAACCAACCTTATTAGGACCT-TTTCGTAATTGA-ATA   12008
m141030_iid=80036_0        ------------------------------------------------------------   11554
m150506_iid=2596494_0      ------------------------------------------------------------   10983
m150503_iid=2526862_0      AAAGGTTGTAATAATCCTAT-AAAACCAACCTTATTAGGACCT-TTTCGTAATTGA-ATA   11860
m150506_iid=2567000_0      AAAGGTTGTAATAATCCTAT-AAAACCAACCTTATTAGGACCT-TTTCGTAATTGA-ATA   9817
m150507_iid=3073271_0      AAAGGTTGTAATAATCCTAT-AAAACCAACCTTATTAGGACCT-TTTCGTAATTGA-ATA   11118
m150504_iid=3156087_0      AAAGGTTGTAATAATCCTAT-AAAACCAACCTTATTAGGACCT-TTTCGTAATTGA-ATA   10924
m150505_iid=2498502_0      AAAGGTTGTAATAATCCTAT-AAAACCAACCTTATTAGGACCT-TTTCGTAATTGA-ATA   8592
m141102_iid=754227_0       ------------------------------------------------------------   10254
m150507_iid=3469514_0      ------------------------------------------------------------   10241
Rdo_MitoGenome             AAAGGTTGTAATAATCCTAT-AAAACCAACCTTATTAGGACCT-TTTCGTAATTGA-ATA   12190
m141101_iid=614933_0       ------------------------------------------------------------   9134

                                                 nad1

m141031_iid=347546_0       TAACCAAGA-ACCTTACGC-TCCATCAAAGTTAAAAATGCAACACC--AACTAAAACCCC   6925
m150506_iid=3127414_0      TAACCAAGA-ACCTTACGC-TCCATCAAAGTTAAAAATGCAACACC--AACTAAAACCCC   8676
m141102_iid=1245517_0      ------------------------------------------------------------   7295
m141107_iid=1405727_0      ------------------------------------------------------------   7315
m141102_iid=987614_0       TAACCAAGA-ACCTTACGC-TCCATCAAAGTTAAAAATGCAACACC--AACTAAAACCCC   10502
m141108_iid=1701429_0      TAACCAAG-AACCTTACGC-TCCATCAAAGTTAAAAATGCAACACCA--ACTAAAACCCC   7237
m141102_iid=987286_0       ------------------------------------------------------------   7493
m150430_iid=3272125_0      TAACCAAG-AACCTTACGCT-CCATCAAAGTTA-AAATGCAACACC--AACTAAAACCCC   9969
m141102_iid=754225_0       ------------------------------------------------------------   9952
m141107_iid=1409934_0      ------------------------------------------------------------   10262
m150503_iid=3416920_0      ------------------------------------------------------------   10188
m141108_iid=1800121_0      TAACCAAGAAACCTTACGC-TCCATCAAAGTTAAAAATGCAACACCAACTA-AAACCGCC   10212
m141031_iid=147540_0       TAACCAAGA-ACCTTACGC-TCCATCAAAGTTAAAAATGCAACACC--AACTAAAACCCC   10573
```

```
m150507_iid=3674924_0      TAACCAAGA-ACCTTACGC-TCCATCAAAGTTAAAAATGCAACACACACACTAAAACCCC   10713
m150503_iid=2923751_0      ------------------------------------------------------------   10134
m141031_iid=115118_0       ------------------------------------------------------------   7981
m150430_iid=2722448_0      TAACCAAGA-ACCTTACGCCTCCATCAAAGTTAAAAATGCAACACC--AACTAAAACCCC   9341
m150507_iid=3102647_0      TAACCAAGA-ACCTTACGC-TCCATCAAAGTTAAAAATGCAACACC--AACTAAAACCCC   7645
m141108_iid=1811866_0      ------------------------------------------------------------   9038
m150501_iid=2948495_0      TAACCAAGA-ACCTTACGC-TCCATCAAAGTTAAAAATGCAACACC--AACTAAAACCCC   8937
m150504_iid=3238195_0      ------------------------------------------------------------   10610
m150503_iid=3388511_0      TAACCAAGA-ACCTTACGC-TCCATCAAAGTTAAAAATGCAACACC--AACTAAAACCCC   8985
m150505_iid=3777611_0      TAACCAAGA-ACCTTACGC-TCCATCAAAGTTAAAAATGCAACACC--AACTAAAACCCC   10411
m150430_iid=2438833_0      TAACCAAGA-ACCTTACGC-TCCATCAAAGTTAAAAATGCAACACC--AACTAAAACCCC   10739
m141108_iid=1698969_0      ------------------------------------------------------------   11147
m150430_iid=3059190_0      TAACCAAGA-ACCTTACGC-TCCATCAAAGTTAAAAATGCAACACC--AACTAAAACCCC   7601
m141107_iid=1383067_0      ------------------------------------------------------------   7061
m141101_iid=597008_0       TAACCAAGA-ACCTTACGC-TCCATCAAAGTTAAAAATGCAACACC--AACTAAAACCCC   4670
m150506_iid=2596496_0      ------------------------------------------------------------   10491
m141102_iid=910051_0       ------------------------------------------------------------   7428
m150505_iid=3784017_0      TAACCAAGA-ACCTTACGC-TCCATCAAAGTTAAAAATGCAACACC--AACTAAAACCCC   10521
m141101_iid=682151_0       TAACCAAGA-ACCTTACGC-TCCATCAAAGTTAAAAATGCAACACC--AACTAAAACCCC   9593
m150501_iid=3748029_0      TAACCAAGA-ACCTTACGC-TCCATCAAAGTTAAAAATGCAACACC--AACTAAAACCCC   10138
m150504_iid=2876272_0      TAACCAAGA-ACCTTACGC-TCCATCAAAGTTAAAAATGCAACACC--AACTAAAACCCC   11990
NC_042820.1                TAACCAAGA-ACCTTACGC-TCCATCAAAGTTAAAAATGCAACACC--AACTAAAACCCC   12246
m141102_iid=1228825_0      ------------------------------------------------------------   11951
m150504_iid=2876274_0      TAACCAAGA-ACCTTACGC-TCCATCAAAGTTAAAAATGCAACACC--AACTAAAACCCC   12064
m141030_iid=80036_0        ------------------------------------------------------------   11554
m150506_iid=2596494_0      ------------------------------------------------------------   10983
m150503_iid=2526862_0      TAACCAAGA-ACCTTACGC-TCCATCAAAGTTAAAAATGCAACACC--AACTAAAACCCC   11916
m150506_iid=2567000_0      TAACCAAGA-ACCTTACGC-TCCATCAAAGTTAAAAATGCAACACC--AACTAAAACCCC   9873
m150507_iid=3073271_0      TAACCAAGA-ACCTTACGC-TCCATCAAAGTTAAAAATGCAACACC--AACTAAAA-CCC   11173
m150504_iid=3156087_0      TAACCAAGA-ACCTTACGC-TCCATCAAAGTTAAAAATGCAACACC--AACTAAAACCCC   10980
m150505_iid=2498502_0      TAACCAAGA-ACCTTACGC-TCCATCAAAGTTAAAAATGCAACACC--AACTAAAACCCC   8648
m141102_iid=754227_0       ------------------------------------------------------------   10254
m150507_iid=3469514_0      ------------------------------------------------------------   10241
Rdo_MitoGenome             TAACCAAGA-ACCTTACGC-TCCATCAAAGTTAAAAATGCAACACC--AACTAAAACCCC   12246
m141101_iid=614933_0       ------------------------------------------------------------   9134
```

nad1

```
m141031_iid=347546_0       AATAATCAAGACTAATAAAG-AAAAAAAAACAAGAATAAAATCAAAAA-TAAACAATATT   6983
m150506_iid=3127414_0      AATAATCAAGACTAATAAAG-AAAA-AAAACAAGAATAAAATCAAAAA-TAAACAATATT   8733
m141102_iid=1245517_0      ------------------------------------------------------------   7295
m141107_iid=1405727_0      ------------------------------------------------------------   7315
m141102_iid=987614_0       AATAATCAAGACTAATAAAGAAAAAAAAAACAAGAATAAAATCAAAAATTAAACAATATT   10562
m141108_iid=1701429_0      AATAATCAAGACTAATAAAGAAA-AAAAAACAAGAATAAAATCAAAAA-TAAACAATATT   7295
m141102_iid=987286_0       ------------------------------------------------------------   7493
m150430_iid=3272125_0      AAATAACAAGACTAATAAAGAAAAAAAAAA--CAGAATAAAATCAAA-------------   10013
m141102_iid=754225_0       ------------------------------------------------------------   9952
m141107_iid=1409934_0      ------------------------------------------------------------   10262
m150503_iid=3416920_0      ------------------------------------------------------------   10188
m141108_iid=1800121_0      AATAATCAAGACTAATAAAGAAA-AAAAAACAAGAATAAAATCAAAAA-TAAACAATATT   10270
m141031_iid=147540_0       AATAATCAAGACTAATAAAG-AAAAAAAAACAAGAATAAAATCAAAAA-TAAACAATATT   10631
m150507_iid=3674924_0      AATAATCAAGACTAATAAAGAAAAAAAAAAAGA--AATAAAATCAAAA------------   10758
m150503_iid=2923751_0      ------------------------------------------------------------   10134
m141031_iid=115118_0       ------------------------------------------------------------   7981
m150430_iid=2722448_0      AATAATCAAGACTAATAAAGAAAAAAAAAACAAGAATAAAATCAAAAA-TAAACAATATT   9400
m150507_iid=3102647_0      AATAATCAAGACTAATAAAGAAA-AAAAAACAAGAATAAAATCAAAAA-TAAACAATATT   7703
m141108_iid=1811866_0      ------------------------------------------------------------   9038
m150501_iid=2948495_0      AATAATCAAGACTAATAAAGAAA--AAAAACAAGAATAAAATCAAAAA-TAAACAATATT   8994
m150504_iid=3238195_0      ------------------------------------------------------------   10610
m150503_iid=3388511_0      AATAATCAAGACTAATAAAG-AAAAAAAAACAAGAATAAAATCAAAAA-TAAACAATATT   9043
m150505_iid=3777611_0      AATAATCAAGACTAATAAAGAAAAAAAAAACAAGAATAAAATCAAAAA-TAAACAATATT   10470
m150430_iid=2438833_0      AATAATCAAGACTAATAAAG-AAAAAAAAACAAGAATAAAATCAAAAA-TAAACAATATT   10797
m141108_iid=1698969_0      ------------------------------------------------------------   11147
m150430_iid=3059190_0      AATAATCAAGACTAATAAAG-AAAAAAAAACAAGAATAAAATCAAAAA-TAAACAATATT   7659
m141107_iid=1383067_0      ------------------------------------------------------------   7061
m141101_iid=597008_0       AATAATCAAGACTAATAAAG-AAAA-AAAACAAGAATAAAATCAAAAA-TAAACAATATT   4727
m150506_iid=2596496_0      ------------------------------------------------------------   10491
m141102_iid=910051_0       ------------------------------------------------------------   7428
m150505_iid=3784017_0      AATAATCAAGACTAATAAAG-AAAAAAAAACAAG-ATAAAATCAAAAA-TAAACAATATT   10578
m141101_iid=682151_0       AATAATCAAGACTAATAAAG-AAAAAAAAACAAGAATAAAATCAAAAA-TAAACAATATT   9651
m150501_iid=3748029_0      AATAATCAAGACTAATAAAG-AAAAAAAAACAAGAATAAAATCAAAAA-TAAACAATATT   10196
m150504_iid=2876272_0      AATAATCAAGACTAATAAAG-AAAAAAAAACAAGAATAAAATCAAAAA-TAAACAATATT   12048
```

```
NC_042820.1                 AATAATCAAGACTAACAAAG-AAAAAAAAACAAGAATAAAATCAAAAA-TAAACAATATT   12304
m141102_iid=1228825_0       ------------------------------------------------------------   11951
m150504_iid=2876274_0       AATAATCAAGACTAATAAAG-AAAAAAAAA-AAGAATAAAATCAAAAA-TAAACAATATT   12121
m141030_iid=80036_0         ------------------------------------------------------------   11554
m150506_iid=2596494_0       ------------------------------------------------------------   10983
m150503_iid=2526862_0       AATAATCAAGACTAATAAAG-AAAAAAAAACAAGAATAAAATCAAAAA-TAAACAATATT   11974
m150506_iid=2567000_0       AATAATCAAGACTAATAAAG-AAAAAAAAACAAGAATAAAATCAAAAA-TAAACAATATT   9931
m150507_iid=3073271_0       AATAATCAAGACTAATAAAG-AAAAAAAAACAAGAATAAAATCAAAAA-TAAACAATATT   11231
m150504_iid=3156087_0       AATAATCAAGACTAATAAAG-AAAAAAAAACAAGAATAAAATCAAAAA-TAAACAATATT   11038
m150505_iid=2498502_0       AATAATCAAGACTAATAAAG-AAAA-AAAACAAGAATAAAATCAAAAA-TAAACAATATT   8705
m141102_iid=754227_0        ------------------------------------------------------------   10254
m150507_iid=3469514_0       ------------------------------------------------------------   10241
Rdo_MitoGenome              AATAATCAAGACTAATAAAG-AAAAAAAAACAAGAATAAAATCAAAAA-TAAACAATATT   12304
m141101_iid=614933_0        ------------------------------------------------------------   9134
```

nad1

```
m141031_iid=347546_0        ATTTGTATAAC-----ATATAAGA-TCCTAAATCTACCG---CACTAATCTGCCAATA--   7032
m150506_iid=3127414_0       ATTTGTATAAAATACATATAAAGA-TCCTAAATCTACCG---CACTAATCTGCCAAAA-T   8788
m141102_iid=1245517_0       ------------------------------------------------------------   7295
m141107_iid=1405727_0       ------------------------------------------------------------   7315
m141102_iid=987614_0        ATTTGTATAAAATACATATAAAGATTCCTAAATCTACCG---CACTAATCTGCAAAA--T   10617
m141108_iid=1701429_0       ATTTGTATAAAATACATATAAAGA-TCCTAAATCTACCG---CACTAATCTGCCAAAA-T   7350
m141102_iid=987286_0        ------------------------------------------------------------   7493
m150430_iid=3272125_0       ------------------------------------------------------------   10013
m141102_iid=754225_0        ------------------------------------------------------------   9952
m141107_iid=1409934_0       ------------------------------------------------------------   10262
m150503_iid=3416920_0       ------------------------------------------------------------   10188
m141108_iid=1800121_0       ATTTGTATAAAATACATATAAAGA-TCCTAAATCTACGCCAAAACTAATCTGCCAAAAAT   10329
m141031_iid=147540_0        ATTTGTATAAAATACATATAAAGA-TCCTAAATCTACCG---CACTAATCTGCCAAAA-T   10686
m150507_iid=3674924_0       ------------------------------------------------------------   10758
m150503_iid=2923751_0       ------------------------------------------------------------   10134
m141031_iid=115118_0        ------------------------------------------------------------   7981
m150430_iid=2722448_0       ATT---------------------------------------------------------   9403
m150507_iid=3102647_0       ATTTGTATAAAATACATATAAAGA-TCCTAAATCTACCG---CACTAATCTGCCAAAA-T   7758
m141108_iid=1811866_0       ------------------------------------------------------------   9038
m150501_iid=2948495_0       ATTTGTATAAAATACATATAAAGA-TCCTAAATCTACCG---CACTAATCTGCCAAAA-T   9049
m150504_iid=3238195_0       ------------------------------------------------------------   10610
m150503_iid=3388511_0       ATTTGTATAAAATACATATAAAGA-TCCTAAATCTACCG---CACTAATCTGCCAAAA-T   9098
m150505_iid=3777611_0       ATTTGTATAAAATACATATAAAGA-TCCTAAATCTACCG---CACTAATCTGCCAAAA-T   10525
m150430_iid=2438833_0       ATTTGTATAAAATACATATAAAGA-TCCTAAATCTACCG---CACTAATCTGCCAAAA-T   10852
m141108_iid=1698969_0       ------------------------------------------------------------   11147
m150430_iid=3059190_0       ATTTGTATAAAATACATATAAAGA-TCCTAAATCTACCG---CACTAATCTGCCAAAA-T   7714
m141107_iid=1383067_0       ------------------------------------------------------------   7061
m141101_iid=597008_0        ATTTGTATAAAATACATATAAAGA-TCCTAAATCTACCG---CACTAATCTGCCAAAA-T   4782
m150506_iid=2596496_0       ------------------------------------------------------------   10491
m141102_iid=910051_0        ------------------------------------------------------------   7428
m150505_iid=3784017_0       ATTTGTATAAAATACATATAAAGA-TCCTAAATCTACCG---CACTAATCTGCCAAAA-T   10633
m141101_iid=682151_0        ATTTGTATAAAATACATATAAAGA-TCCTAAATCTACCG---CACTAATCTGCCAAAA-T   9706
m150501_iid=3748029_0       ATTTGTATAAAATACATATAAAGA-TCCTAAATCTACCG---CACTAATCTGCCAAAA-T   10251
m150504_iid=2876272_0       ATTTGTATAAAATACATATAAAGA-TCCTAAATCTACCG---CACTAATCTGCCAAAA-T   12103
NC_042820.1                 ATTTGTATAAAATACATATAAAGA-TCCTAAATCTACCG---CACTAATCTGCCAAAA-T   12359
m141102_iid=1228825_0       ------------------------------------------------------------   11951
m150504_iid=2876274_0       ATTTGTATAAAATACATATAAAGA-TCCTAAATCTACCG---CACTAATCTGCCAAAA-T   12176
m141030_iid=80036_0         ------------------------------------------------------------   11554
m150506_iid=2596494_0       ------------------------------------------------------------   10983
m150503_iid=2526862_0       ATTTGTATAAAATACATATAAAGA-TCCTAAATCTACCG---CACTAATCTGCCAA----   12026
m150506_iid=2567000_0       ATTTGTATAAAATACATATAAAGA-TCCTAAATCTACCG---CACTAATCTGCCAAAA-T   9986
m150507_iid=3073271_0       ATTTGTATAAAATACATATAAAGA-TCCTAAATCTACCG---CACTAATCTGCCAAAA-T   11286
m150504_iid=3156087_0       ATTTGTATAAAATACATATAAAGA-TCCTAAATCTACCG---CACTAATCTGCCAAAA-T   11093
m150505_iid=2498502_0       ATTTGTATAAAATACATATAAAGA-TCCTAAATCTACCG---CACTAATCTGCCAAAA-T   8760
m141102_iid=754227_0        ------------------------------------------------------------   10254
m150507_iid=3469514_0       ------------------------------------------------------------   10241
Rdo_MitoGenome              ATTTGTATAAAATACATATAAAGA-TCCTAAATCTACCG---CACTAATCTGCCAAAA-T   12359
m141101_iid=614933_0        ------------------------------------------------------------   9134


m141031_iid=347546_0        -ATAAGAATACCCAAAAAATAAATAATATA--TTGAAATACTGGTCCTTTCGTACTAAGT   7089
m150506_iid=3127414_0       AATAAGAATACCCAAAAAATAAATAATATA--TTGAAATACTGGTCCTTTCGTACTAAGT   8846
m141102_iid=1245517_0       ------------------------------------------------------------   7295
m141107_iid=1405727_0       ------------------------------------------------------------   7315
m141102_iid=987614_0        AATAAGAATACCAAAAAATAAATAATATAT--TGAAATTACTGGTCCTTTCGTACTAAGT   10675
```

```
m141108_iid=1701429_0       AATAAGAATACCCAAAAAATAAATA--ATATATTGAAATACTGGTCCTTTCGTACTAAGT   7408
m141102_iid=987286_0        ------------------------------------------------------------   7493
m150430_iid=3272125_0       ------------------------------------------------------------   10013
m141102_iid=754225_0        ------------------------------------------------------------   9952
m141107_iid=1409934_0       ------------------------------------------------------------   10262
m150503_iid=3416920_0       ------------------------------------------------------------   10188
m141108_iid=1800121_0       AATAAGAATACCCAAAAAATAAATAATA-TAATTGAAATACTGGTCCTTTCGTACTAAGT   10388
m141031_iid=147540_0        AATAAGAATACCCAAAAAATAAATAATATA--TTGAAATACTGGTCCTTTCGTACTAAGT   10744
m150507_iid=3674924_0       ------------------------------------------------------------   10758
m150503_iid=2923751_0       ------------------------------------------------------------   10134
m141031_iid=115118_0        ------------------------------------------------------------   7981
m150430_iid=2722448_0       ------------------------------------------------------------   9403
m150507_iid=3102647_0       AATAAGAATACCCAAAAAATAAATAA--TATATTGAAATACTGGTCCTTTCGTACTAAGT   7816
m141108_iid=1811866_0       ------------------------------------------------------------   9038
m150501_iid=2948495_0       AATAAGAATACCCAAAAAATAAATAA--TATATTGAAATACTGGTCCTTTCGTACTAAGT   9107
m150504_iid=3238195_0       ------------------------------------------------------------   10610
m150503_iid=3388511_0       AATAAGAATACCCAAAAAATAAATAATATA--TTGAAATACTGGTCCTTTCGTACTAAGT   9156
m150505_iid=3777611_0       AATAAGAATACCCAAAAAAAAAATAATATA--TTGAAATACTGGTCCTTTCGTACTAAGT   10583
m150430_iid=2438833_0       AATAAGAATACCCAAAAAATAAATAATATA--TTGAAATACTGGTCCTTTCGTACTAAGT   10910
m141108_iid=1698969_0       ------------------------------------------------------------   11147
m150430_iid=3059190_0       AATAAGAATACCCAAAAAATAAATAA--TATATTGAAATACTGGTCCTTTCGTACTAAGT   7772
m141107_iid=1383067_0       ------------------------------------------------------------   7061
m141101_iid=597008_0        AATAAGAATACCCAAAAAATAAATAA--TATATTGAAATACTGGTCCTTTCGTACTAAGT   4840
m150506_iid=2596496_0       ------------------------------------------------------------   10491
m141102_iid=910051_0        ------------------------------------------------------------   7428
m150505_iid=3784017_0       AATAAGAATACCCAAAAAATAAATAATATA--TTGAAATACTGGTCCTTTCGTACTAAGT   10691
m141101_iid=682151_0        AATAAGAATACCCAAAAAATAAATAATATA--TTGAAATACTGGTCCTTTCGTACTAAGT   9764
m150501_iid=3748029_0       AATAAGAATACCCAAAAAATAAATAATATA--TTGAAATACTGGTCCTTTCGTACTAAGT   10309
m150504_iid=2876272_0       AATAAGAATACCCAAAAAATAAATAATATA--TTGAAATACTGGTCCTTTCGTACTAAGT   12161
NC_042820.1                 AATAAGAATACCCAAAAAATAAATAATATA--TTGAAATACTGGTCCTTTCGTACTAAGT   12417
m141102_iid=1228825_0       ------------------------------------------------------------   11951
m150504_iid=2876274_0       AATAAGAATACCCAAAAAATAAATAATATA--TTGAAATACTGGTCCTTTCGTACTAAGT   12234
m141030_iid=80036_0         ------------------------------------------------------------   11554
m150506_iid=2596494_0       ------------------------------------------------------------   10983
m150503_iid=2526862_0       AATAAGAATACCCAAAAAATAAATAATATA--TTGAAATACTGGTCCTTTCGTACTAAGT   12084
m150506_iid=2567000_0       AATAAGAATACCCAAAAAATAAATAATATA--TTGAAATACTGGTCCTTTCGTACTAAGT   10044
m150507_iid=3073271_0       AATAAGAATACCAAAAAATAAATAATATA---TTGAAATACTGGTCCTTTCGTACTAAGT   11343
m150504_iid=3156087_0       AATAAGAATACCCAAAAAATAAATAATATAATATGAAATACTGGTCCTTTCGTACTAAGT   11153
m150505_iid=2498502_0       AATAAGAATACCCAAAAAATAAATAATATA--TTGAAATACTGGTCCTTTCGTACTAAGT   8818
m141102_iid=754227_0        ------------------------------------------------------------   10254
m150507_iid=3469514_0       ------------------------------------------------------------   10241
Rdo_MitoGenome              AATAAGAATACCCAAAAAATAAATAATATA--TTGAAATACTGGTCCTTTCGTACTAAGT   12417
m141101_iid=614933_0        ------------------------------------------------------------   9134


m141031_iid=347546_0        AAATC-AAAAAAATTGAAGATAGAA-CCAACCTGTC------TGGCTCAAAACTCAGATC   7141
m150506_iid=3127414_0       AAATC-AAAAAAATTGAAGATAGAAACCAACCTGGCTCAAACCGGTTTTTAACTCAGATC   8905
m141102_iid=1245517_0       ------------------------------------------------------------   7295
m141107_iid=1405727_0       ------------------------------------------------------------   7315
m141102_iid=987614_0        AAATCAAAAAAAATTGAAGATAGAAACCAACCTGGCTCAAACCGG-TTTTAACTCAGATC   10734
m141108_iid=1701429_0       AAATCAAAAAAAATTGAAGATAGAAACCAACCTGGCTCAAACCGG-TTTTAACTCAGATC   7467
m141102_iid=987286_0        ------------------------------------------------------------   7493
m150430_iid=3272125_0       ------------------------------------------------------------   10013
m141102_iid=754225_0        ------------------------------------------------------------   9952
m141107_iid=1409934_0       ------------------------------------------------------------   10262
m150503_iid=3416920_0       ------------------------------------------------------------   10188
m141108_iid=1800121_0       AAATCAAAA-AAATTGAAGATAGAAACCAACCTGGCTCAAACCGG-TTTTAACTCAGATC   10446
m141031_iid=147540_0        AAATCAAAAAAAATTGAAGATAGAAACCAACCTGGCTCAAACCGG-TTTTAACTCAGATC   10803
m150507_iid=3674924_0       ------------------------------------------------------------   10758
m150503_iid=2923751_0       ------------------------------------------------------------   10134
m141031_iid=115118_0        ------------------------------------------------------------   7981
m150430_iid=2722448_0       ------------------------------------------------------------   9403
m150507_iid=3102647_0       AAATCAAAAAAAATTGAAGATAGAAACCAACCTGGCTCAAACCGG-TTTTAACTCAGATC   7875
m141108_iid=1811866_0       ------------------------------------------------------------   9038
m150501_iid=2948495_0       AAATCAAAAAAAATTGAAGATAGAAACCAACCTGGCTCAAACCGG-TTTTAACTCAGATC   9166
m150504_iid=3238195_0       ------------------------------------------------------------   10610
m150503_iid=3388511_0       AAATC-AAAAAAATTGAAGATAGAAACCAACCTGGCTCAAACCGG-TTTTAACTCAGATC   9214
m150505_iid=3777611_0       AAATCAAAAAAAATTGAAGATAGAAACCAACCTGGCTCAAACCGG-TTTTAACTCAGATC   10642
m150430_iid=2438833_0       AAATCAAAAAAAATTGAAGATAGAAACCAACCTGGCTCAAACCGG-TTTTAACTCAGATC   10969
m141108_iid=1698969_0       ------------------------------------------------------------   11147
m150430_iid=3059190_0       AAATCAAAAAAAATTGAAGATAGAAACCAACCTGGCTCAAACCGG-TTTTAACTCAGATC   7831
```

```
m141107_iid=1383067_0      ------------------------------------------------------------   7061
m141101_iid=597008_0       AAATCAAAAAAAATTGAAGATAGAAACCAACCTGGCTCAAACCGG-TTTTAACTCAGATC   4899
m150506_iid=2596496_0      ------------------------------------------------------------   10491
m141102_iid=910051_0       ------------------------------------------------------------   7428
m150505_iid=3784017_0      AAATCAAAAAAAATTGAAGATAGAAACCAACCTGGCTCAAACCGG-TTTTAACTCAGATC   10750
m141101_iid=682151_0       AAATCAAAAAAAATTGAAGATAGAAACCAACCTGGCTCAAACCGG-TTTTAACTCAGATC   9823
m150501_iid=3748029_0      AAATCAAAAAAAATTGAAGATAGAAACCAACCTGGCTCAAACCGG-TTTTAACTCAGATC   10368
m150504_iid=2876272_0      AAATCAAAAAAAATTGAAGATAGAAACCAACCTGGCTCAAACCGG-TTTTAACTCAGATC   12220
NC_042820.1                AAATC-AAAAAAATTGAAGATAGAAACCAACCTGGCTCAAACCGG-TTTTAACTCAGATC   12475
m141102_iid=1228825_0      ------------------------------------------------------------   11951
m150504_iid=2876274_0      AAATCAAAAAAAATTGAAGATAGAAACCAACCTGGCTCAAACCGG-TTTTAACTCAGATC   12293
m141030_iid=80036_0        ------------------------------------------------------------   11554
m150506_iid=2596494_0      ------------------------------------------------------------   10983
m150503_iid=2526862_0      AAATCAAAAAAAATTGAAGATAGAAACCAACCTGGCTCAAACCGG-TTTTAACTCAGATC   12143
m150506_iid=2567000_0      AAATCAAAAAAAATTGAAGATAGAAACCAACCTGGCTCAAACCGG-TTTTAACTCAGATC   10103
m150507_iid=3073271_0      AAATCAAAAAAAATTGAAGATAGAAACCAACCTGGCTCAAACCGG-TTTTAACTCAGATC   11402
m150504_iid=3156087_0      AAATCAAAAAAAATTGAAGATAGAAACCAACCTGGCTCAAACCGG-TTTTAACTCAGATC   11212
m150505_iid=2498502_0      AAATCAAAAAAAATTGAAGATAGAAACCAACCTGGCTCAAACCGG-TTTTAACTCAGATC   8877
m141102_iid=754227_0       ------------------------------------------------------------   10254
m150507_iid=3469514_0      ------------------------------------------------------------   10241
Rdo_MitoGenome             AAATCAAAAAAAATTGAAGATAGAAACCAACCTGGCTCAAACCGG-TTTTAACTCAGATC   12476
m141101_iid=614933_0       ------------------------------------------------------------   9134


m141031_iid=347546_0       ATGTAAAGTTTTAAAGGTCGAACAGACCTAACATTCAAGCTTTTGCACCAGAAGTTTACT   7201
m150506_iid=3127414_0      ATGTAAAGTTTTAAAGGTCGAACAGACCTAACATTCAAGCTTTTGCACCAGAAGTTTACT   8965
m141102_iid=1245517_0      ------------------------------------------------------------   7295
m141107_iid=1405727_0      ------------------------------------------------------------   7315
m141102_iid=987614_0       ATGTAAAGTTTTAAAGGTCGAACAGACCTAACATTCAAGCTTTTGCACCAGAAGTTTACT   10794
m141108_iid=1701429_0      ATGTAAAGTTTTAAAGGTCGAACAGACCTAACATTCAAGCTTTTGCACCAGAAGTTTACT   7527
m141102_iid=987286_0       ------------------------------------------------------------   7493
m150430_iid=3272125_0      ------------------------------------------------------------   10013
m141102_iid=754225_0       ------------------------------------------------------------   9952
m141107_iid=1409934_0      ------------------------------------------------------------   10262
m150503_iid=3416920_0      ------------------------------------------------------------   10188
m141108_iid=1800121_0      ATGTAAAGTTTTAAAGGTCGAACAGACCTAACATTCAAGCTT-TGCACCAGAAGTTTACT   10505
m141031_iid=147540_0       ATGTAAAGTTTTAAAGGTCGAACAGACCTAACATTCAAGCTTTTGCACCAGAAGTTTACT   10863
m150507_iid=3674924_0      ------------------------------------------------------------   10758
m150503_iid=2923751_0      ------------------------------------------------------------   10134
m141031_iid=115118_0       ------------------------------------------------------------   7981
m150430_iid=2722448_0      ------------------------------------------------------------   9403
m150507_iid=3102647_0      ATGTAAAGTTTTAAAGGTCGAACAGACCTAACATTCAAGCTTTTGCACCAGAAGTTTACT   7935
m141108_iid=1811866_0      ------------------------------------------------------------   9038
m150501_iid=2948495_0      ATGTAAAGTTTTAAAGGTCGAACAGACCTAACATTCAAGCTTTTGCACCAGAAGTTTACT   9226
m150504_iid=3238195_0      ------------------------------------------------------------   10610
m150503_iid=3388511_0      ATGTAAAGTTTTAAAGGTCGAACAGACCTAACATTCAAGCTTTTGCACCAGAAGTTTACT   9274
m150505_iid=3777611_0      ATGTAAAGTTTTAAAGGTCGAACAGACCTAACATTCAAGCTTTTGCACCAGAAGTTTACT   10702
m150430_iid=2438833_0      ATGTAAAGTTTTAAAGGTCGAACAGACCTAACATTCAAGCTTTTGCACCAGAAGTTTACT   11029
m141108_iid=1698969_0      ------------------------------------------------------------   11147
m150430_iid=3059190_0      ATGTAAAGTTTTAAAGGTCGAACAGACCTAACATTCAAGCTTTTGCACCAGAAGTTTACT   7891
m141107_iid=1383067_0      ------------------------------------------------------------   7061
m141101_iid=597008_0       ATGTAAAGTTTTAAAGGTCGAACAGACCTAACATTCAAGCTTTTGCACCAGAAGTTTACT   4959
m150506_iid=2596496_0      ------------------------------------------------------------   10491
m141102_iid=910051_0       ------------------------------------------------------------   7428
m150505_iid=3784017_0      ATGTAAAGTTTTAAAGGTCGAACAGACCTAACATTCAAGCTTTTGCACCAGAAGTTTACT   10810
m141101_iid=682151_0       ATGTAAAGTTTTAAAGGTCGAACAGACCTAACATTCAAGCTTTTGCACCAGAAGTTTACT   9883
m150501_iid=3748029_0      ATGTAAAGTTTTAAAGGTCGAACAGACCTAACATTCAAGCTTTTGCACCAGAAGTTTACT   10428
m150504_iid=2876272_0      ATGTAAAGTTTTAAAGGTCGAACAGACCTAACATTCAAGCTTTTGCACCAGAAGTTTACT   12280
NC_042820.1                ATGTAAAGTTTTAAAGGTCGAACAGACCTAACATTCAAGCTTTTGCACCAGAAGTTTACT   12535
m141102_iid=1228825_0      ------------------------------------------------------------   11951
m150504_iid=2876274_0      ATGTAAAGTTTTAAAGGTCGAACAGACCTAACATTCAAGCTTTTGCACCAGAAGTTTACT   12353
m141030_iid=80036_0        ------------------------------------------------------------   11554
m150506_iid=2596494_0      ------------------------------------------------------------   10983
m150503_iid=2526862_0      ATGTAAAGTTTTAAAGGTCGAACAGACCTAACATTCAAGCTTTTGCACCAGAAGTTTACT   12203
m150506_iid=2567000_0      ATGTAAAGTTTTAAAGGTCGAACAGACCTAACATTCAAGCTTTTGCACCAGAAGTTTACT   10163
m150507_iid=3073271_0      ATGTAAAGTTTTAAAGGTCGAACAGACCTAACATTCAAGCTTTTGCACCAGAAGTTTACT   11462
m150504_iid=3156087_0      ATGTAAAGTTTTAAAGGTCGAACAGACCTAACATTCAAGCTTTTGCACCAGAAGTTTACT   11272
m150505_iid=2498502_0      ATGTAAAGTTTTAAAGGTCGAACAGACCTAACATTCAAGCTTTTGCACCAGAAGTTTACT   8937
m141102_iid=754227_0       ------------------------------------------------------------   10254
m150507_iid=3469514_0      ------------------------------------------------------------   10241
Rdo_MitoGenome             ATGTAAAGTTTTAAAGGTCGAACAGACCTAACATTCAAGCTTTTGCACCAGAAGTTTACT   12536
```

```
m141101_iid=614933_0        ------------------------------------------------------------  9134


m141031_iid=347546_0        T-TAATCCAACATCGAGGTCGCAACCCCTTCCATCAAT-AAGAACTCTA-AAGAAAGATT  7258
m150506_iid=3127414_0       T-TAATCCAACATCGAGGTCGCAACCCCTTCCATCAAT-AAGAACTCTA-AAGAAAGATT  9022
m141102_iid=1245517_0       ------------------------------------------------------------  7295
m141107_iid=1405727_0       ------------------------------------------------------------  7315
m141102_iid=987614_0        T-TAATCCAACATCGAGGTCGCAACCCCTTCCATCAAT-AAGAACTCTA-AAGAAAGATT  10851
m141108_iid=1701429_0       T-TAATCCAACATCGAGGTCGCAACCCCTTCCATCAATA-AGAACTCTA-AAGAAAGATT  7584
m141102_iid=987286_0        ------------------------------------------------------------  7493
m150430_iid=3272125_0       ------------------------------------------------------------  10013
m141102_iid=754225_0        ------------------------------------------------------------  9952
m141107_iid=1409934_0       ------------------------------------------------------------  10262
m150503_iid=3416920_0       ------------------------------------------------------------  10188
m141108_iid=1800121_0       TTTAATCCAACATCGAGGTCGCAACCCCTTCCATCAAGTAAGAACTCTAAAAGAAAGATT  10565
m141031_iid=147540_0        T-TAATCCAACATCGAGGTCGCAACCCCTTCCATCAAT-AAGAACTCTA-AAGAAAGATT  10920
m150507_iid=3674924_0       ------------------------------------------------------------  10758
m150503_iid=2923751_0       ------------------------------------------------------------  10134
m141031_iid=115118_0        ------------------------------------------------------------  7981
m150430_iid=2722448_0       ------------------------------------------------------------  9403
m150507_iid=3102647_0       T-TAATCCAACATCGAGGTCGCAACC-CTTCCATCAAT-AAGAACTCTA-AAGAAAGATT  7991
m141108_iid=1811866_0       ------------------------------------------------------------  9038
m150501_iid=2948495_0       T-TAATCCAACATCGAGGTCGCAACCCCTTCCATCAAT-AAGAACTCTA-AAGAAAGATT  9283
m150504_iid=3238195_0       ------------------------------------------------------------  10610
m150503_iid=3388511_0       T-TAATCCAACATCGAGGTCGCAACCCCTTCCATCAAT-AAGAACTCTA-AAGAAAGATT  9331
m150505_iid=3777611_0       T-TAATCCAACATCGAGGTCGCAACCCCTTCCATCAAT-AAGAACTCTA-AAGAAAGATT  10759
m150430_iid=2438833_0       T-TAATCCAACATCGAGGTCGCAACCCCTTCCATCAAT-AAGAACTCTA-AAGAAAGATT  11086
m141108_iid=1698969_0       ------------------------------------------------------------  11147
m150430_iid=3059190_0       T-TAATCCAACATCGAGGTCGCAACCCCTTCCATCAAT-AAGAACTCTA-AAGAAAGATT  7948
m141107_iid=1383067_0       ------------------------------------------------------------  7061
m141101_iid=597008_0        T-TAATCCAACATCGAGGTCGCAACCCCTTCCATCAAT-AAGAACTCTA-AAGAAAGATT  5016
m150506_iid=2596496_0       ------------------------------------------------------------  10491
m141102_iid=910051_0        ------------------------------------------------------------  7428
m150505_iid=3784017_0       T-TAATCCAACATCGAGGTCGCAACCCCTTCCATCAAT-AAGAACTCTA-AAGAAAGATT  10867
m141101_iid=682151_0        T-TAATCCAACATCGAGGTCGCAACCCCTTCCATCAAT-AAGAACTCTA-AAGAAAGATT  9940
m150501_iid=3748029_0       T-TAATCCAACATCGAGGTCGCAACCCCTTCCATCAAT-AAGAACTCTA-AAGAAAGATT  10485
m150504_iid=2876272_0       T-TAATCCAACATCGAGGTCGCAACCCCTTCCATCAAT-AAGAACTCTA-AAGAAAGATT  12337
NC_042820.1                 T-TAATCCAACATCGAGGTCGCAACCCCTTCCATCAAT-AAGAACTCTA-AAGAAAGATT  12592
m141102_iid=1228825_0       ------------------------------------------------------------  11951
m150504_iid=2876274_0       T-TAATCCAACATCGAGGTCGCAACCCCTTCCATCAAT-AAGAACTCTA-AAGAAAGATT  12410
m141030_iid=80036_0         ------------------------------------------------------------  11554
m150506_iid=2596494_0       ------------------------------------------------------------  10983
m150503_iid=2526862_0       T-TAATCCAACATCGAGGTCGCAACCCCTTCCATCAAT-AAGAACTCTA-AAGAAAGATT  12260
m150506_iid=2567000_0       T-TAATCCAACATCGAGGTCGCAACCCCTTCCATCAAT-AAGAACTCTA-AAGAAAGATT  10220
m150507_iid=3073271_0       T-TAATCCAACATCGAGGTCGCAA-CCCTTCCATCAAT-AAGAACTCTA-AAGAAAGATT  11518
m150504_iid=3156087_0       T-TAATCCAACATCGAGGTCGCAACCCCTTCCATCAAT-AAGAACTCTA-AAGAAAGATT  11329
m150505_iid=2498502_0       T-TAATCCAACATCGAGGTCGCAACCCCTTCCATCAAT-AAGAACTCTA-AAGAAAGATT  8994
m141102_iid=754227_0        ------------------------------------------------------------  10254
m150507_iid=3469514_0       ------------------------------------------------------------  10241
Rdo_MitoGenome              T-TAATCCAACATCGAGGTCGCAACCCCTTCCATCAAT-AAGAACTCTA-AAGAAAGATT  12593
m141101_iid=614933_0        ------------------------------------------------------------  9134


m141031_iid=347546_0        ACGCTGTTATCCCTAAGGTAATTTTAACTTATAATCAAAAAAAATTGAATCAATAAAATA  7318
m150506_iid=3127414_0       ACGCTGTTATCC-TAAGGTAATTTTAACTTATAATCAAAAAAAATTGAATCAATAAAATA  9081
m141102_iid=1245517_0       ------------------------------------------------------------  7295
m141107_iid=1405727_0       ------------------------------------------------------------  7315
m141102_iid=987614_0        ACGCTGTTATCCTAAGGTAATTGTTAACTTATAATCAAAAAAAATTGAATCAATAAAATA  10911
m141108_iid=1701429_0       ACGCTGTTATCCCTAAGGTAATTTTAACTTATAATCAAAAAAAATTGAATCAATAAAATA  7644
m141102_iid=987286_0        ------------------------------------------------------------  7493
m150430_iid=3272125_0       ------------------------------------------------------------  10013
m141102_iid=754225_0        ------------------------------------------------------------  9952
m141107_iid=1409934_0       ------------------------------------------------------------  10262
m150503_iid=3416920_0       ------------------------------------------------------------  10188
m141108_iid=1800121_0       ACGCTGTTATCCCTAAGGTAATTTTAACTTATAATCAAAAAA-ATTGAATCAA-------  10617
m141031_iid=147540_0        ACGCTGTTATCCCTAAGGTAATTTTAACTTATAATCAAAAAAAATTGAATCAATAAAATA  10980
m150507_iid=3674924_0       ------------------------------------------------------------  10758
m150503_iid=2923751_0       ------------------------------------------------------------  10134
m141031_iid=115118_0        ------------------------------------------------------------  7981
m150430_iid=2722448_0       ------------------------------------------------------------  9403
m150507_iid=3102647_0       ACGCTGTTATCCCTAAGGTAATTTTAACTTATAATCAAAAA-AATTGAATCAATAAAATA  8050
```

```
m141108_iid=1811866_0      ------------------------------------------------------------   9038
m150501_iid=2948495_0      ACGCTGTTATCCCTAAGGTAATTTTAACTTATAATCAAAAAAAATTGAATCAATAAAATA   9343
m150504_iid=3238195_0      ------------------------------------------------------------   10610
m150503_iid=3388511_0      ACGCTGTTATCCCTAAGGTAATTTTAACTTATAATCAAAAAAAATTGAATCAATAAAATA   9391
m150505_iid=3777611_0      ACGCTGTTATCCCTAAGGTAATTTTAACTTATAATCAAAAAAAATTGAATCAATAAAATA   10819
m150430_iid=2438833_0      ACGCTGTTATCCCTAAGGTAATTTTAACTTATAATCAAAAAAAATTGAATCAATAAAATA   11146
m141108_iid=1698969_0      ------------------------------------------------------------   11147
m150430_iid=3059190_0      ACGCTGTTATCCCTAAGGTAATT-TAACTTATAATCAAAAAAAATTGAATCAATAAAATA   8007
m141107_iid=1383067_0      ------------------------------------------------------------   7061
m141101_iid=597008_0       ACGCTGTTATCCCTAAGGTAATTTTAACTTATAATCAAAAAAAATTGAATCAATAAAATA   5076
m150506_iid=2596496_0      ------------------------------------------------------------   10491
m141102_iid=910051_0       ------------------------------------------------------------   7428
m150505_iid=3784017_0      ACGCTGTTATCCCTAAGGTAATTTTAACTTATAATCAAAAAAAATTGAATCAATAAAATA   10927
m141101_iid=682151_0       ACGCTGTTATCCCTAAGGTAATTTTAACTTATAATCAAAAAAAATTGAATCAATAAAATA   10000
m150501_iid=3748029_0      ACGCTGTTATCCCTAAGGTAATTTTAACTTATAATCAAAAAAAATTGAATCAATAAAATA   10545
m150504_iid=2876272_0      ACGCTGTTATCCCTAAGGTAATTTTAACTTATAATCAAAAAAAATTGAATCAATAAAATA   12397
NC_042820.1                ACGCTGTTATCCCTAAGGTAATTTTAACTTATAATCAAAAAAAATTGAATCAATAAAATA   12652
m141102_iid=1228825_0      ------------------------------------------------------------   11951
m150504_iid=2876274_0      ACGCTGTTATCCCTAAGGTAATTTTAACTTATAATCAAAAAAAATTGAATCAATAAAATA   12470
m141030_iid=80036_0        ------------------------------------------------------------   11554
m150506_iid=2596494_0      ------------------------------------------------------------   10983
m150503_iid=2526862_0      ACGCTGTTATCCCTAAGGTAATTTTAACTTATAATCAAAAAAAATTGAATCAATAAAATA   12320
m150506_iid=2567000_0      ACGCTGTTATCCCTAAGGTAATTTTAACTTATAATCAAAAAAAATTGAATCAATAAAATA   10280
m150507_iid=3073271_0      ACGCTGTTATCCCTAAGGTAATTTTAACTTATAATCAAAAAAAATTGAATCAATAAAATA   11578
m150504_iid=3156087_0      ACGCTGTTAT-CCTAAGGTAATTTTAACTTATAATCAAAAAAAATTGA------------   11376
m150505_iid=2498502_0      ACGCTGTTATCCCTAAGGTAATTTTAACTTATAATCAAAAAAAATTGAATCAATAAAATA   9054
m141102_iid=754227_0       ------------------------------------------------------------   10254
m150507_iid=3469514_0      ------------------------------------------------------------   10241
Rdo_MitoGenome             ACGCTGTTATCCCTAAGGTAATTTTAACTTATAATCAAAAAAAATTGAATCAATAAAATA   12653
m141101_iid=614933_0       ------------------------------------------------------------   9134


m141031_iid=347546_0       TAAATAAATATAAAA--TAAAAGAAAGTTTGTCAA-TTATTCCCATCACCCC-AACAAAA   7374
m150506_iid=3127414_0      TAAATAAATATAATA---AAAGAAAAGTTTGTCAAATTATTCCCATCACACCCAACAAAA   9138
m141102_iid=1245517_0      ------------------------------------------------------------   7295
m141107_iid=1405727_0      ------------------------------------------------------------   7315
m141102_iid=987614_0       TAA--TAAATATAAAAATAAAGAGAAGTTTGTCAAATTATTCCCATCACCCC-AACAAAA   10968
m141108_iid=1701429_0      TAAATAAATATAAAAATAAAAGAAAAGTTTGTCAAATTATTCCCATCACCCC-AACAAAA   7703
m141102_iid=987286_0       ------------------------------------------------------------   7493
m150430_iid=3272125_0      ------------------------------------------------------------   10013
m141102_iid=754225_0       ------------------------------------------------------------   9952
m141107_iid=1409934_0      ------------------------------------------------------------   10262
m150503_iid=3416920_0      ------------------------------------------------------------   10188
m141108_iid=1800121_0      ------------------------------------------------------------   10617
m141031_iid=147540_0       TAAATAAATATAAAAATAAAAGAAAAGTTTGTCAAATTATTCCCATCACCCC-AACAAAA   11039
m150507_iid=3674924_0      ------------------------------------------------------------   10758
m150503_iid=2923751_0      ------------------------------------------------------------   10134
m141031_iid=115118_0       ------------------------------------------------------------   7981
m150430_iid=2722448_0      ------------------------------------------------------------   9403
m150507_iid=3102647_0      TAAATAAATATAAAAATAAAAGAAAAGTTTGTCAAATTATTCCCATCACCCC-AACAAAA   8109
m141108_iid=1811866_0      ------------------------------------------------------------   9038
m150501_iid=2948495_0      TAAATAAATATAAAAATAAAAGAAAAGTTTGTCAAATTATTCCCATCACCCC-AACAAAA   9402
m150504_iid=3238195_0      ------------------------------------------------------------   10610
m150503_iid=3388511_0      TAAATAAATATAAAAATAAAAGAAAAGTTTGTCAAATTATTCCCATCACCCC-AACAAAA   9450
m150505_iid=3777611_0      -AAATAAATATAAAAATAAAAGAAAAGTTTGTCAAATTATTCCCATCACCCC-AACAAAA   10877
m150430_iid=2438833_0      TAAATAAATATAAAAATAAAAGAAAAGTTTGTCAAATTATTCCCATCACCC---AAAAAA   11203
m141108_iid=1698969_0      ------------------------------------------------------------   11147
m150430_iid=3059190_0      TAAATAAATATAAAAATA-AAAGAAAGTTTGTCAAATTATTCCCATCACCCC-AACAAAA   8065
m141107_iid=1383067_0      ------------------------------------------------------------   7061
m141101_iid=597008_0       TAAATAAATATAAAAATAAAAGAAAAGTTTGTCAAATTATTCCCATCACCCC-AACAAAA   5135
m150506_iid=2596496_0      ------------------------------------------------------------   10491
m141102_iid=910051_0       ------------------------------------------------------------   7428
m150505_iid=3784017_0      TAAATAAATATAAAAATAAAAGAAAAGTTTGTCAAATTATTCCCATCACCCC-AACAAAA   10986
m141101_iid=682151_0       TAAATAAATATAAAAATAAAAGAAAAGTTTGTCAAATTATTCCCATCACCCC-AACAAAA   10059
m150501_iid=3748029_0      TAAATAAATATAAAAATAAAAGAAAAGTTTGTCAAATTATTCCCATCACCCC-AACAAAA   10604
m150504_iid=2876272_0      TAAATAAATATAAAAATAAAAGAAAAGTTTGTCAAATTATTCCCATCACCCC-AACAAAA   12456
NC_042820.1                TAAATAAATATAAAAATAAAAGAAAAGTTTGTCAAATTATTCCCATCACCCC-AACAAAA   12711
m141102_iid=1228825_0      ------------------------------------------------------------   11951
m150504_iid=2876274_0      TAAATAAATATAAAAATAAAAGAAAAGTTTGTCAAATTATTCCCATCACCCC-AACAAAA   12529
m141030_iid=80036_0        ------------------------------------------------------------   11554
m150506_iid=2596494_0      ------------------------------------------------------------   10983
```

```
m150503_iid=2526862_0      TAAATAAATATAAAAATAAAAGAAAAGTTTGTCAAATTATTCCCATCACCCC-AACAAAA   12379
m150506_iid=2567000_0      TAAATAAATATAAAAATAAAAGAAAAGTTTGTCAAATTATTCCCATCACCCC-AACAAAA   10339
m150507_iid=3073271_0      TAAAT-AATAAATAAATAAAAGAAAAGTTTGTCAAATTATT-CCATCACC----------   11626
m150504_iid=3156087_0      ------------------------------------------------------------   11376
m150505_iid=2498502_0      TAAATAAATATAAAAATAAAAGAAAAGTTTGTCAAATTATTCCCATCACCCC-AACAAAA   9113
m141102_iid=754227_0       ------------------------------------------------------------   10254
m150507_iid=3469514_0      ------------------------------------------------------------   10241
Rdo_MitoGenome             TAAATAAATATAAAAATAAAAGAAAAGTTTGTCAAATTATTCCCATCACCCC-AACAAAA   12712
m141101_iid=614933_0       ------------------------------------------------------------   9134


m141031_iid=347546_0       TAATTTTTAAT-TAATAAAGAAAAATCCTAAATAAATTATTAACC-AAAAAATATTAAAT   7432
m150506_iid=3127414_0      TAATTTTTAATTAATAAAAGAAAAATCCTAAATAAATTATTAACC-AAAAATATTAAACT   9197
m141102_iid=1245517_0      ------------------------------------------------------------   7295
m141107_iid=1405727_0      ------------------------------------------------------------   7315
m141102_iid=987614_0       TAATTTTTTAATTAATAAAAGAAAAATCCTAAATAAATTATTAACCAAAAATATTAAACT   11028
m141108_iid=1701429_0      TAATTTTTAATTAATAAAAGAAAAATCCTAAATAAATTATTAACCAAAAAATATTAAACT   7763
m141102_iid=987286_0       ------------------------------------------------------------   7493
m150430_iid=3272125_0      ------------------------------------------------------------   10013
m141102_iid=754225_0       ------------------------------------------------------------   9952
m141107_iid=1409934_0      ------------------------------------------------------------   10262
m150503_iid=3416920_0      ------------------------------------------------------------   10188
m141108_iid=1800121_0      ------------------------------------------------------------   10617
m141031_iid=147540_0       TAATTTTTAATTAATAAAAGAAAAATCCTAAATAAATTATTAACCAAAAAA-ATTAAACT   11098
m150507_iid=3674924_0      ------------------------------------------------------------   10758
m150503_iid=2923751_0      ------------------------------------------------------------   10134
m141031_iid=115118_0       ------------------------------------------------------------   7981
m150430_iid=2722448_0      ------------------------------------------------------------   9403
m150507_iid=3102647_0      TAATTTTTAATTAATAAAAGAAAAATCCTAAATAAATTATTAACCAAAAAATATTAAACT   8169
m141108_iid=1811866_0      ------------------------------------------------------------   9038
m150501_iid=2948495_0      TAATTTTTAATTAATAAAAGAAAAATCCTAAATAAATTATTAACCAAAAAATATTAAACT   9462
m150504_iid=3238195_0      ------------------------------------------------------------   10610
m150503_iid=3388511_0      TAATTTTTAATTAATAAAAGAAAAATCCTAAATAAATTATTAACC-AAAAAAATTAAACT   9509
m150505_iid=3777611_0      TAATTTTTAATTAATAAAAGAAAAATCCTAAATAAATTATTAACC-AAAAATATTAAACT   10936
m150430_iid=2438833_0      TAATTTTTAATTAATAAAAGAAAAATCCTAAATAAATTATTAACCAAAAAATATTAAACT   11263
m141108_iid=1698969_0      ------------------------------------------------------------   11147
m150430_iid=3059190_0      TAATTTTTAATTAATAAAAGAAAAATCCTAAATAAATTATTAACCAAAAAATATTAAACT   8125
m141107_iid=1383067_0      ------------------------------------------------------------   7061
m141101_iid=597008_0       TAATTTTTAATTAATAAAAGAAAAATCCTAAATAAATTATTAACCAAAAAATATTAAACT   5195
m150506_iid=2596496_0      ------------------------------------------------------------   10491
m141102_iid=910051_0       ------------------------------------------------------------   7428
m150505_iid=3784017_0      TAATTTTTAATTAATAAAAGAAAAATCCTAAATAAATTATTAACCAAAAAATATTAAACT   11046
m141101_iid=682151_0       TAATTTTTAATTAATAAAAGAAAAATCCTAAATAAATTATTAACCAAAAAATATTAAACT   10119
m150501_iid=3748029_0      TAATTTTTAATTAATAAAAGAAAAATCCTAAATAAATTATTAACCAAAAAATATT-AACT   10663
m150504_iid=2876272_0      TAATTTTTAATTAATAAAAGAAAAATCCTAAATAAATTATTAACCAAAAAATATTAAACT   12516
NC_042820.1                TAATTTTTAATTAATAAAAGAAAAATCCTAAATAAATTATTAACCAAAAAATATTAAACT   12771
m141102_iid=1228825_0      ------------------------------------------------------------   11951
m150504_iid=2876274_0      TAATTTTTAATTAATAAAAGAAAAATCCTAAATAAATTATTAACCAAAAAATATTAAACT   12589
m141030_iid=80036_0        ------------------------------------------------------------   11554
m150506_iid=2596494_0      ------------------------------------------------------------   10983
m150503_iid=2526862_0      TAATTTTTAATTAATAAAAGAAAAATCCTAAATAAATTATTAACCAAAAAATATTAAACT   12439
m150506_iid=2567000_0      TAATTTTTAATTAATAAAAGAAAAATCCTAAATAAATTATTAACCAAAAAATATTAAACT   10399
m150507_iid=3073271_0      ------------------------------------------------------------   11626
m150504_iid=3156087_0      ------------------------------------------------------------   11376
m150505_iid=2498502_0      TAATTTTTAATTAATAAAAGAAAAATCCTAAATAAATTATTAACCAAAAAATATTAAACT   9173
m141102_iid=754227_0       ------------------------------------------------------------   10254
m150507_iid=3469514_0      ------------------------------------------------------------   10241
Rdo_MitoGenome             TAATTTTTAATTAATAAAAGAAAAATCCTAAATAAATTATTAACCAAAAAATATTAAACT   12772
m141101_iid=614933_0       ------------------------------------------------------------   9134


m141031_iid=347546_0       CTATAGGGTCTTCTC--GTCTTTTCAAAAAAATTGAACTTTTTTAATTCAAAAATAAATT   7490
m150506_iid=3127414_0      CTATAGGGTCTTCTCTCGTCTT-TCAAAAAATTTGAACTTTTTAATTT-AAAATAAAATT   9255
m141102_iid=1245517_0      ------------------------------------------------------------   7295
m141107_iid=1405727_0      ------------------------------------------------------------   7315
m141102_iid=987614_0       CTATAGGGTCTTCTC--GTCTTTCAAAAAAATTTGAACTTTTTAATTCAAAAATAAAATT   11086
m141108_iid=1701429_0      CTATAGGGTCTTCTC--GTCTTTCAAAAAAATTTGAACTTTTTAATTCAAAAATAAAATT   7821
m141102_iid=987286_0       ------------------------------------------------------------   7493
m150430_iid=3272125_0      ------------------------------------------------------------   10013
m141102_iid=754225_0       ------------------------------------------------------------   9952
m141107_iid=1409934_0      ------------------------------------------------------------   10262
```

```
m150503_iid=3416920_0      ------------------------------------------------------------  10188
m141108_iid=1800121_0      ------------------------------------------------------------  10617
m141031_iid=147540_0       CTATAGGGTCTTCTC--GTCTTTCAAAAAAATTTGAACTTTTTAATTCAAAAATAAAATT  11156
m150507_iid=3674924_0      ------------------------------------------------------------  10758
m150503_iid=2923751_0      ------------------------------------------------------------  10134
m141031_iid=115118_0       ------------------------------------------------------------  7981
m150430_iid=2722448_0      ------------------------------------------------------------  9403
m150507_iid=3102647_0      CTATAGGGTCTTCTC--GTCTTTCAAAAAAATTTGAACTTTTTAATTCAAAAATAAAATT  8227
m141108_iid=1811866_0      ------------------------------------------------------------  9038
m150501_iid=2948495_0      CTATAGGGTCTTCTC--GTCTTTCAAAAAAATTTGAACTTTTTAATTCAAAAATAAAATT  9520
m150504_iid=3238195_0      ------------------------------------------------------------  10610
m150503_iid=3388511_0      CTATAGGGTCTTCTC--GTCTTTC-AAAAAATTTGAACTTTTTAATTCAAAAATAAAATT  9566
m150505_iid=3777611_0      CTATAGGGTCTTCTC--GTCTTTCAAAAAAATTTGAACTTTTTAATTCAAAAATAAAATT  10994
m150430_iid=2438833_0      CTATAGGGTCTTCTC--GTCTTTCAAAAAAATTTGAACTTTTTAATTCAAAAATAAAATT  11321
m141108_iid=1698969_0      ------------------------------------------------------------  11147
m150430_iid=3059190_0      CTATAGGGTCTTCTC--GTCTTTCAAAAAAATTTGAACTTTTTAATTCAAAATAAAACA-  8182
m141107_iid=1383067_0      ------------------------------------------------------------  7061
m141101_iid=597008_0       CTATAGGGTCTTCTC--GTCTTTCAAAAAAATTTGAACTTTTTAATTCAAAAATAAAATT  5253
m150506_iid=2596496_0      ------------------------------------------------------------  10491
m141102_iid=910051_0       ------------------------------------------------------------  7428
m150505_iid=3784017_0      CTATAGGGTCTTCTC--GTCTTTCAAAAAAATTTGAACTTTTTAATTCAAAAATAAAATT  11104
m141101_iid=682151_0       CTATAGGGTCTTCTC--GTCTTTCAAAAAAATTTGAACTTTTTAATTCAAAAATAAAATT  10177
m150501_iid=3748029_0      CTATAGGGTCTTCTC--GTCTTTCAAAAAAATTTGAACTTTTTAATTCAAAAATAAAATT  10721
m150504_iid=2876272_0      CTATAGGGTCTTCTC--GTCTTTCAAAAAAA-TTGAACTTTTTAATTCAAAAATAAAATT  12573
NC_042820.1                CTATAGGGTCTTCTC--GTCTTTCAAAAAAATTTGAACTTTTTAATTCAAAAATAAAATT  12829
m141102_iid=1228825_0      ------------------------------------------------------------  11951
m150504_iid=2876274_0      CTATAGGGTCTTCTC--GTCTTTC-AAAAAATTTGAACTTTTTAATTCAAAAATAAAATT  12646
m141030_iid=80036_0        ------------------------------------------------------------  11554
m150506_iid=2596494_0      ------------------------------------------------------------  10983
m150503_iid=2526862_0      CTATAGGGTCTTCTC--GTCTTTC-AAAAAATTTGAACTTTTTAATTC-AAAATAAAATT  12495
m150506_iid=2567000_0      CTATAGGGTCTTCTC--GTCTTTCAAAAAAATTTGAACTTTTTAATTCAAAAATAAAATT  10457
m150507_iid=3073271_0      ------------------------------------------------------------  11626
m150504_iid=3156087_0      ------------------------------------------------------------  11376
m150505_iid=2498502_0      CTATAGGGTCTTCTC--GTCTTTCAAAAAAATTTGAACTTTTTAATTCAAAAATAAAATT  9231
m141102_iid=754227_0       ------------------------------------------------------------  10254
m150507_iid=3469514_0      ------------------------------------------------------------  10241
Rdo_MitoGenome             CTATAGGGTCTTCTC--GTCTTTCAAAAAAATTTGAACTTTTTAATTCAAAAATAAAATT  12830
m141101_iid=614933_0       ------------------------------------------------------------  9134


m141031_iid=347546_0       CAAAAAATAAAGAATGAGACA---------------------------------------  7511
m150506_iid=3127414_0      CAAAAAATAAAGAATGAGACAAAACT-CCCTTGTCAAACCATTTCATTCAGCTTTCATTA  9314
m141102_iid=1245517_0      ------------------------------------------------------------  7295
m141107_iid=1405727_0      ------------------------------------------------------------  7315
m141102_iid=987614_0       CAAAAAATAAAGAATGAGACAAAACTCCCCTTGTCAAACCATTCATTCCAGCTTTCAATT  11146
m141108_iid=1701429_0      CAAAAAATAAAGAATGAGACAAAACTCCCCTTGTCAAACCATTCATTCCAGCTTTCAATT  7881
m141102_iid=987286_0       ------------------------------------------------------------  7493
m150430_iid=3272125_0      ------------------------------------------------------------  10013
m141102_iid=754225_0       ------------------------------------------------------------  9952
m141107_iid=1409934_0      ------------------------------------------------------------  10262
m150503_iid=3416920_0      ------------------------------------------------------------  10188
m141108_iid=1800121_0      ------------------------------------------------------------  10617
m141031_iid=147540_0       CAAAAAATAAAGAATGAGACAAAACTCCCCTTGTCAAACCATTCATTCCAGCTTTCAATT  11216
m150507_iid=3674924_0      ------------------------------------------------------------  10758
m150503_iid=2923751_0      ------------------------------------------------------------  10134
m141031_iid=115118_0       ------------------------------------------------------------  7981
m150430_iid=2722448_0      ------------------------------------------------------------  9403
m150507_iid=3102647_0      CAAAAAATAAAGAATGAGACAAAACTCCCCTTGTCAAACCATTCATTCCAGCTTTCAATT  8287
m141108_iid=1811866_0      ------------------------------------------------------------  9038
m150501_iid=2948495_0      CAAAAAATAAAGAATGAGACAAAACTCCCCTTGTCAAACCATTCATTCCAGCTTTCAATT  9580
m150504_iid=3238195_0      ------------------------------------------------------------  10610
m150503_iid=3388511_0      CAAAAAATAAAGAATGAGACAAAACTCCCCTTGTCAAACCATTCATTCCAGCTTTCAATT  9626
m150505_iid=3777611_0      CAAAAAATAAAGAATGAGACAAAACTCCCCTTGTCAAACCATTCATTCCAGCTTTCAATT  11054
m150430_iid=2438833_0      CAAAAAATAAAGAATGAGACAAAACTCCCCTTGTCAAACCATTCATTCCAGCTTTCAATT  11381
m141108_iid=1698969_0      ------------------------------------------------------------  11147
m150430_iid=3059190_0      --A-AAATAAAGAA-TGAACAAAACTCCCCTTGTCAAACCATTCATTCCAGCTTTCAATT  8238
m141107_iid=1383067_0      ------------------------------------------------------------  7061
m141101_iid=597008_0       CAAAAAATAAAGAATGAGACAAAACTCCCCTTGTCAAACCATTCATTCCAGCTTTCAATT  5313
m150506_iid=2596496_0      ------------------------------------------------------------  10491
m141102_iid=910051_0       ------------------------------------------------------------  7428
m150505_iid=3784017_0      CAAAAAATAAAGAATGAGACAAAACTCCCCTTGTCAAACCATTCATTCCAGCTTTCAATT  11164
```

```
m141101_iid=682151_0       CAAAAAATAAAGAATGAGACAAAACTCCCCTTGTCAAACCATTCATTCCAGCTTTCAATT   10237
m150501_iid=3748029_0      CAAAAAATAAAGAATGAGACAAAACTCCCCTTGTCAAACCATTCATTCCAGCTTTCAATT   10781
m150504_iid=2876272_0      CAAAAAATAAAGAATGAGACAAAACTCCCCTTGTCAAACCATTCATTCCAGCTTTCAATT   12633
NC_042820.1                CAAAAAATAAAGAATGAGACAAAACTCCCCTTGTCAAACCATTCATTCCAGCTTTCAATT   12889
m141102_iid=1228825_0      ------------------------------------------------------------   11951
m150504_iid=2876274_0      CAAAAA--AAAGAATGAGACAAAACTCCCCTTGTCAAACCATTCATTCCAGCTTTCAATT   12704
m141030_iid=80036_0        ------------------------------------------------------------   11554
m150506_iid=2596494_0      ------------------------------------------------------------   10983
m150503_iid=2526862_0      CAAAAAATAAAGAATGAGACAAAACTCCCCTTGTCAAACCATTCATTCCAGCTTTCAATT   12555
m150506_iid=2567000_0      CAAAAAATAAAGAATGAGACAAAACTCCCCTTGTCAAACCATTCATTCCAGCTTTCAATT   10517
m150507_iid=3073271_0      ------------------------------------------------------------   11626
m150504_iid=3156087_0      ------------------------------------------------------------   11376
m150505_iid=2498502_0      CAAAAAATAAAGAATGAGACAAAACTCCCCTTGTCAAACCATTCATTCCAGCTTTCAATT   9291
m141102_iid=754227_0       ------------------------------------------------------------   10254
m150507_iid=3469514_0      ------------------------------------------------------------   10241
Rdo_MitoGenome             CAAAAAATAAAGAATGAGACAAAACTCCCCTTGTCAAACCATTCATTCCAGCTTTCAATT   12890
m141101_iid=614933_0       ------------------------------------------------------------   9134


m141031_iid=347546_0       ------------------------------------------------------------   7511
m150506_iid=3127414_0      ---AAAACTAATGATTATGCTACCTTTGCACGGTTAAAATACCGCGGCCATTTAATTAAT   9371
m141102_iid=1245517_0      ------------------------------------------------------------   7295
m141107_iid=1405727_0      ------------------------------------------------------------   7315
m141102_iid=987614_0       AA-AAAACTAATGATTATGCTACCTTTGCACGGTTAAAATACCGC-GCCATTTAATTAAT   11204
m141108_iid=1701429_0      AA-AAAACTAATGATTATGCTACCTTTGCACGGTTAAAATACCGCGGAAA-----TTAAT   7935
m141102_iid=987286_0       ------------------------------------------------------------   7493
m150430_iid=3272125_0      ------------------------------------------------------------   10013
m141102_iid=754225_0       ------------------------------------------------------------   9952
m141107_iid=1409934_0      ------------------------------------------------------------   10262
m150503_iid=3416920_0      ------------------------------------------------------------   10188
m141108_iid=1800121_0      ------------------------------------------------------------   10617
m141031_iid=147540_0       AA-AAAACTAATGATTATGCTACCTTTGCACGGTTAAAATACCGCGGCCATTTAATTAAT   11275
m150507_iid=3674924_0      ------------------------------------------------------------   10758
m150503_iid=2923751_0      ------------------------------------------------------------   10134
m141031_iid=115118_0       ------------------------------------------------------------   7981
m150430_iid=2722448_0      ------------------------------------------------------------   9403
m150507_iid=3102647_0      AA-AAAACTAATGATTATGCTACCTTTGCACGGTTAAAATACCGCGGCCATTTAATTAAT   8346
m141108_iid=1811866_0      ------------------------------------------------------------   9038
m150501_iid=2948495_0      AA-AAAACTAATGATTATGCTACCTTTGCACGGTTAAAATACCGCGGCCATTTAATTAAT   9639
m150504_iid=3238195_0      ------------------------------------------------------------   10610
m150503_iid=3388511_0      AA-AAAACTAATGATTATGCTACCTTTGCACGGTTAAAATACCGCGGCCATTTAATTAAT   9685
m150505_iid=3777611_0      AA-AAAACTAATGATTATGCTACCTTTGCACGGTTAAAATACCGCGGCCATTTAATTAAT   11113
m150430_iid=2438833_0      AA-AAAACTAATGATTATGCTACCTTTGCACGGTTAAAATACCGCGGCCATTTAATTAAT   11440
m141108_iid=1698969_0      ------------------------------------------------------------   11147
m150430_iid=3059190_0      AAAAAAACTAATGATTATGCTACCTTTGCACGGTTAAAATACCGCGGCCATTTAATTAAT   8298
m141107_iid=1383067_0      ------------------------------------------------------------   7061
m141101_iid=597008_0       AA-AAAACTAATGATTATGCTACCTTTGCACGGTTAAAATACCGCGGCCATTTAATTAAT   5372
m150506_iid=2596496_0      ------------------------------------------------------------   10491
m141102_iid=910051_0       ------------------------------------------------------------   7428
m150505_iid=3784017_0      AA-AAAACTAATGATTATGCTACCTTTGCACGGTTAAAATACCGCGGCCATTTAATTAAT   11223
m141101_iid=682151_0       AA-AAAACTAATGATTATGCTACCTTTGCACGGTTAAAATACCGCGGCCATTTAATTAAT   10296
m150501_iid=3748029_0      AA-AAAACTAATGATTATGCTACCTTTGCACGGTTAAAATACCGCGGCCATTTAATTAAT   10840
m150504_iid=2876272_0      AA-AAAACTAATGATTATGCTACCTTTGCACGGTTAAAATACCGCGGCCATTTAATTAAT   12692
NC_042820.1                AA-AAAACTAATGATTATGCTACCTTTGCACGGTTAAAATACCGCGGCCATTTAATTAAT   12948
m141102_iid=1228825_0      ------------------------------------------------------------   11951
m150504_iid=2876274_0      AA-AAAACTAATGATTATGCTACCTTTGCACGGTTAAAATACCGCGGCCATTTAATTAAT   12763
m141030_iid=80036_0        ------------------------------------------------------------   11554
m150506_iid=2596494_0      ------------------------------------------------------------   10983
m150503_iid=2526862_0      AA-AAAACTAATGATTATGCTACCTTTGCACGGTTAAAATACCGCGGCCATTTAATTAAT   12614
m150506_iid=2567000_0      AA-AAAACTAATGATTATGCTACCTTTGCACGGTTAAAATACCGCGGCCATTTAATTAAT   10576
m150507_iid=3073271_0      ------------------------------------------------------------   11626
m150504_iid=3156087_0      ------------------------------------------------------------   11376
m150505_iid=2498502_0      AA-AAAACTAATGATTATGCTACCTTTGCACGGTTAAAATACCGCGGCCATTTAATTAAT   9350
m141102_iid=754227_0       ------------------------------------------------------------   10254
m150507_iid=3469514_0      ------------------------------------------------------------   10241
Rdo_MitoGenome             AA-AAAACTAATGATTATGCTACCTTTGCACGGTTAAAATACCGCGGCCATTTAATTAAT   12949
m141101_iid=614933_0       ------------------------------------------------------------   9134


m141031_iid=347546_0       ------------------------------------------------------------   7511
m150506_iid=3127414_0      CATTGGGCAGGCCAGACTTTAAAATATAATCAAAAAGACATGTTTTTAATAAACAGGCAA   9431
```

```
m141102_iid=1245517_0      ------------------------------------------------------------  7295
m141107_iid=1405727_0      ------------------------------------------------------------  7315
m141102_iid=987614_0       CATTGGGCAGGCCAGACTTTAAAATATAATCAAAAAGACATGTTTTTAATAAACAGGC-A  11263
m141108_iid=1701429_0      CATTGGGCAGGCCAGACTTTAAAATATAATCAAAAAGACATGTTTT-AATAAACAGGCAA  7994
m141102_iid=987286_0       ------------------------------------------------------------  7493
m150430_iid=3272125_0      ------------------------------------------------------------  10013
m141102_iid=754225_0       ------------------------------------------------------------  9952
m141107_iid=1409934_0      ------------------------------------------------------------  10262
m150503_iid=3416920_0      ------------------------------------------------------------  10188
m141108_iid=1800121_0      ------------------------------------------------------------  10617
m141031_iid=147540_0       CATTGGGCAGGCCAGACTTTAAAATATAATCAAAAAGACATGTTTTTAATAAACAGGCAA  11335
m150507_iid=3674924_0      ------------------------------------------------------------  10758
m150503_iid=2923751_0      ------------------------------------------------------------  10134
m141031_iid=115118_0       ------------------------------------------------------------  7981
m150430_iid=2722448_0      ------------------------------------------------------------  9403
m150507_iid=3102647_0      CATTGGGCAGGCCAGACTTTAAAATATAATCAAAAAGACATGTTTTTAATAAACAGGCAA  8406
m141108_iid=1811866_0      ------------------------------------------------------------  9038
m150501_iid=2948495_0      CATTGGGCAGGCCAGACTTTAAAATATAATCAAAAAGACATGTTTTTAATAAACAGGCAA  9699
m150504_iid=3238195_0      ------------------------------------------------------------  10610
m150503_iid=3388511_0      CATTGGGCAGGCCAGACTTTAAAATATAATCAAAAAGACATGTTTTTAATAAACAGGCAA  9745
m150505_iid=3777611_0      CATTGGGCAGGCCAGACTTTAAAATATAATCAAAAAGACATGTTTTTAATAAACAGGCAA  11173
m150430_iid=2438833_0      CATTGGGCAGGCCAGACTTTAAAATATAATCAAAAAGACATGTTTTTAATAAACAGGCAA  11500
m141108_iid=1698969_0      ------------------------------------------------------------  11147
m150430_iid=3059190_0      CATTGGGCAGGCCAGACTTTAA-ATATAATCAAAAAGACATGTTTTTAATAAACAGGCAA  8357
m141107_iid=1383067_0      ------------------------------------------------------------  7061
m141101_iid=597008_0       CATTGGGCAGGCCAGACTTTAAAATATAATCAAAAAGACATGTTTTTAATAAACAGGCAA  5432
m150506_iid=2596496_0      ------------------------------------------------------------  10491
m141102_iid=910051_0       ------------------------------------------------------------  7428
m150505_iid=3784017_0      CATTGGGCAGGCCAGACTTTAAAATATAATCAAAAAGACATGTTTTTAATAAACAGGCAA  11283
m141101_iid=682151_0       CATTGGGCAGGCCAGACTTTAAAATATAATCAAAAAGACATGTTTTTAATAAACAGGCAA  10356
m150501_iid=3748029_0      CATTGGGCAGGCCAGACTTTAAAATATAATCAAAAAGACATGTTTTTAATAAACAGGCAA  10900
m150504_iid=2876272_0      CATTGGGCAGGCCAGACTTTAAAATATAATCAAAAAGACATGTTTTTAATAAACAGGCAA  12752
NC_042820.1                CATTGGGCAGGCCAGACTTTAAAATATAATCAAAAAGACATGTTTTTAATAAACAGGCAA  13008
m141102_iid=1228825_0      ------------------------------------------------------------  11951
m150504_iid=2876274_0      CATTGGGCAGGCCAGACTTTAAAATATAATCAAAAAGACATGTTTTTAATAAACAGGCAA  12823
m141030_iid=80036_0        ------------------------------------------------------------  11554
m150506_iid=2596494_0      ------------------------------------------------------------  10983
m150503_iid=2526862_0      CATTGGGCAGGCCAGACTTTAAAATATAATCAAAAAGACATGTTTTTAATAAACAGGCAA  12674
m150506_iid=2567000_0      CATTGGGCAGGCCAGACTTTAAAATATAATCAAAAAGACATGTTTTTAATAAACAGGCAA  10636
m150507_iid=3073271_0      ------------------------------------------------------------  11626
m150504_iid=3156087_0      ------------------------------------------------------------  11376
m150505_iid=2498502_0      CATT-GGCAGGCCAGACTTTAAAATATAATCAAAAAGACATGTTTTTAATAAACAGGCAA  9409
m141102_iid=754227_0       ------------------------------------------------------------  10254
m150507_iid=3469514_0      ------------------------------------------------------------  10241
Rdo_MitoGenome             CATTGGGCAGGCCAGACTTTAAAATATAATCAAAAAGACATGTTTTTAATAAACAGGCAA  13009
m141101_iid=614933_0       ------------------------------------------------------------  9134


m141031_iid=347546_0       ------------------------------------------------------------  7511
m150506_iid=3127414_0      GAGAGAAAGTCGAGTTCCTTA-AACTCTTCTGAAAATAAACAAAAAATACAAAAACCATT  9490
m141102_iid=1245517_0      ------------------------------------------------------------  7295
m141107_iid=1405727_0      ------------------------------------------------------------  7315
m141102_iid=987614_0       GAGAGAAAGTCGAGTTCCTTA-AACTCTTCTGAAAATAAACAAAAAATACAAAAACCATT  11322
m141108_iid=1701429_0      GAGAGAAAGTCGAGTTCCTTA-AACTCTTCTGAAAATAAACAAAAAATACAAAAACCATT  8053
m141102_iid=987286_0       ------------------------------------------------------------  7493
m150430_iid=3272125_0      ------------------------------------------------------------  10013
m141102_iid=754225_0       ------------------------------------------------------------  9952
m141107_iid=1409934_0      ------------------------------------------------------------  10262
m150503_iid=3416920_0      ------------------------------------------------------------  10188
m141108_iid=1800121_0      ------------------------------------------------------------  10617
m141031_iid=147540_0       GAGAGAAAGTCGAGTTCCTTA-AACTCTTCTGAAAATAAACAAAAAATACAAAAACCATT  11394
m150507_iid=3674924_0      ------------------------------------------------------------  10758
m150503_iid=2923751_0      ------------------------------------------------------------  10134
m141031_iid=115118_0       ------------------------------------------------------------  7981
m150430_iid=2722448_0      ------------------------------------------------------------  9403
m150507_iid=3102647_0      GAGAGAAAGTCGAGTTCCTTA-AACTCTTCTGAAAATAAACAAAAAATACAAAAACCATT  8465
m141108_iid=1811866_0      ------------------------------------------------------------  9038
m150501_iid=2948495_0      GAGAGAAAGTCGAGTTCCTTA-AACTCTTCTGAAAATAAACAAAAAATACAAAAACCATT  9758
m150504_iid=3238195_0      ------------------------------------------------------------  10610
m150503_iid=3388511_0      GAGAGAAAGTCGAGTTCCTTA-AACTCTTCTGAAAATAAACAAAAAA-ACAAAAACCATT  9803
m150505_iid=3777611_0      GAGAGAAAGTCGAGTTCCTTA-AACTCTTCTGAAAATAA--AAAAAATACAAAAACCATT  11230
```

```
m150430_iid=2438833_0      GAGAGAAAGTCGAGTTCCTTA-AACTCTTCTGAAAATAAACAAAAAATACAAAAACCATT   11559
m141108_iid=1698969_0      ------------------------------------------------------------   11147
m150430_iid=3059190_0      GAGAGAAAGTCGAGTTCCTTAAATCTCTTCTGAAAAATAAACAAAAATACAAAAACCATT   8417
m141107_iid=1383067_0      ------------------------------------------------------------   7061
m141101_iid=597008_0       GAGAGAAAGTCGAGTTCCTTA-AACTCTTCTGAAAATAAACAAAAAATACAAAAACCATT   5491
m150506_iid=2596496_0      ------------------------------------------------------------   10491
m141102_iid=910051_0       ------------------------------------------------------------   7428
m150505_iid=3784017_0      GAGAGAAAGTCGAGTTCCTTA-AACTCTTCTGAAAATAAACAAAAAATACAAAAACCATT   11342
m141101_iid=682151_0       GAGAGAAAGTCGAGTTCCTTA-AACTCTTCTGAAAATAAAC-AAAAATACAAAAACCATT   10414
m150501_iid=3748029_0      GAGAGAAAGTCGAGTTCCTTA-AACTCTTCTGAAAATAAACAAAAAATACAAAAACCATT   10959
m150504_iid=2876272_0      GAGAGAAAGTCGAGTTCCTTA-AACTCTTCTGAAAATAAACAAAAAATACAAAAACCATT   12811
NC_042820.1                GAGAGAAAGTCGAGTTCCTTA-AACTCTTCTGAAAATAAACAAAAAATACAAAAACCATT   13067
m141102_iid=1228825_0      ------------------------------------------------------------   11951
m150504_iid=2876274_0      GAGAGAAAGTCGAGTTCCTTA-AACTCTTCTGAAAATAAACAAAAAATACAAAAACCATT   12882
m141030_iid=80036_0        ------------------------------------------------------------   11554
m150506_iid=2596494_0      ------------------------------------------------------------   10983
m150503_iid=2526862_0      GAGAGAAAGTCGAGTTCCTTA-AACTCTTCTGAAAATAAACAAAAAATACAAAAACCATT   12733
m150506_iid=2567000_0      GAGAGAAAGTCGAGTTCCTTA-AACTCTTCTGAAAATAAACAAAAAATACAAAAACCATT   10695
m150507_iid=3073271_0      ------------------------------------------------------------   11626
m150504_iid=3156087_0      ------------------------------------------------------------   11376
m150505_iid=2498502_0      GAGAGAAAGTCGAGTTCCTTA-AACTCTTCTGAAAATAAACAAAAAATACAAAAACCATT   9468
m141102_iid=754227_0       ------------------------------------------------------------   10254
m150507_iid=3469514_0      ------------------------------------------------------------   10241
Rdo_MitoGenome             GAGAGAAAGTCGAGTTCCTTA-AACTCTTCTGAAAATAAACAAAAAATACAAAAACCATT   13068
m141101_iid=614933_0       ------------------------------------------------------------   9134


m141031_iid=347546_0       ------------------------------------------------------------   7511
m150506_iid=3127414_0      TACTAATTTAATCATAATAATAAA------AACATTATTAAATTAAATTTTAAATAAAAA   9544
m141102_iid=1245517_0      ------------------------------------------------------------   7295
m141107_iid=1405727_0      ------------------------------------------------------------   7315
m141102_iid=987614_0       TACTAATTTAATCATAATAAATAAAAAACAATTATTAAATTAAAATTTTAAATAAAAAAC   11382
m141108_iid=1701429_0      TACTAATTTAATCATAATAAAATAAAAA---TTATTAAATTAAAATTTTAAATAAAAAAC   8110
m141102_iid=987286_0       ------------------------------------------------------------   7493
m150430_iid=3272125_0      ------------------------------------------------------------   10013
m141102_iid=754225_0       ------------------------------------------------------------   9952
m141107_iid=1409934_0      ------------------------------------------------------------   10262
m150503_iid=3416920_0      ------------------------------------------------------------   10188
m141108_iid=1800121_0      ------------------------------------------------------------   10617
m141031_iid=147540_0       TACTAATTTAATCATAATAAAATAAAAAC-ATTATTAAATTAAAATTTTAAATAAAAAAC   11453
m150507_iid=3674924_0      ------------------------------------------------------------   10758
m150503_iid=2923751_0      ------------------------------------------------------------   10134
m141031_iid=115118_0       ------------------------------------------------------------   7981
m150430_iid=2722448_0      ------------------------------------------------------------   9403
m150507_iid=3102647_0      TACTAATTTAATCATAATAAAATAAAAAC-ATTATTAAATTAAAATTTTAAATAAAAAAC   8524
m141108_iid=1811866_0      ------------------------------------------------------------   9038
m150501_iid=2948495_0      TACTAATTTAATCATAATAAAATAAAAAC-ATTATTAAATTAAAATTTTAAATAAAAAAC   9817
m150504_iid=3238195_0      ------------------------------------------------------------   10610
m150503_iid=3388511_0      TACTAATTTAATCATAATAAAATAAAAAC-ATTATTAAATTAAAATTTTAAAT-AAAAAC   9861
m150505_iid=3777611_0      TACTAATTTAATCATAATAAAATAAAAAC-ATTATTAAATTAAAATTTTAAATAAAAAAC   11289
m150430_iid=2438833_0      TACTAATTTAATCATAATAAAATAAAAAC-ATTATTAAATTAAAATTTTAAATAAAAAAC   11618
m141108_iid=1698969_0      ------------------------------------------------------------   11147
m150430_iid=3059190_0      TACTAATTTAATCATAATAAAATAAAAAC-ATTATTAAATTAAAATTTTAAATAAAAAAC   8476
m141107_iid=1383067_0      ------------------------------------------------------------   7061
m141101_iid=597008_0       TACTAATTTAATCATAATAAAATAAAAAC-ATTATTAAATTAAAATTTTAAATAAAAAAC   5550
m150506_iid=2596496_0      ------------------------------------------------------------   10491
m141102_iid=910051_0       ------------------------------------------------------------   7428
m150505_iid=3784017_0      TACTAATTTAATCATAATAAAATAAAAAC-ATTATTAAATTAAAATTTTAAATAAAAAAC   11401
m141101_iid=682151_0       TACTAATTTAATCATAATAAAATAAAAAC-ATTATTAAATTAAAATTTTAAATAAAAAAC   10473
m150501_iid=3748029_0      TACTAATTTAATCATAATAAAATAAAAAC-ATTATTAAATTAAAATTTTAAATAAAAAAC   11018
m150504_iid=2876272_0      TACTAATTTAATCATAATAAAATAAAAAC-ATTATTAAATTAAAATTTTAAATAAAAAAC   12870
NC_042820.1                TACTAATTTAATCATAATAAAATAAAAAC-ATTATTAAATTAAAATTTTAAATAAAAAAC   13126
m141102_iid=1228825_0      ------------------------------------------------------------   11951
m150504_iid=2876274_0      TACTAATTTAATCATAATAAAATAAAAAC-ATTATTAAATTAAAATTTTAAATAAAAAAC   12941
m141030_iid=80036_0        ------------------------------------------------------------   11554
m150506_iid=2596494_0      ------------------------------------------------------------   10983
m150503_iid=2526862_0      TACTAATTTAATCATAATAAAATAAAAAC-ATTATTAAATTAAAATTTTAAAT-AAAAAC   12791
m150506_iid=2567000_0      TACTAATTTAATCATAATAAAATAAAAAC-ATTATTAAATTAAAATTTTAAATAAAAAAC   10754
m150507_iid=3073271_0      ------------------------------------------------------------   11626
m150504_iid=3156087_0      ------------------------------------------------------------   11376
m150505_iid=2498502_0      TACTAATTTAATCATAATAAAATAAAAAC-ATTATTAAATTAAAATTTTAAATAAAAAAC   9527
```

```
m141102_iid=754227_0        ------------------------------------------------------------  10254
m150507_iid=3469514_0       ------------------------------------------------------------  10241
Rdo_MitoGenome              TACTAATTTAATCATAATAAAATAAAAAC-ATTATTAAATTAAAATTTTAAATAAAAAAC  13127
m141101_iid=614933_0        ------------------------------------------------------------  9134


m141031_iid=347546_0        ------------------------------------------------------------  7511
m150506_iid=3127414_0       ACTTAATAAACAAAATATTATTAAAAAATAAGTAATTAAAACATAATAAAAATGAAAAT  9604
m141102_iid=1245517_0       ------------------------------------------------------------  7295
m141107_iid=1405727_0       ------------------------------------------------------------  7315
m141102_iid=987614_0        TTAAAATAAACAAAATATTTATTAAAAATAAGTAATTAAAACAT-ATAAAAATGAAAAT  11441
m141108_iid=1701429_0       TTAAAATAAACAAAATATTATTAAAAAATAAGTAATTAAAACATAATAAAAATGAAAAT  8170
m141102_iid=987286_0        ------------------------------------------------------------  7493
m150430_iid=3272125_0       ------------------------------------------------------------  10013
m141102_iid=754225_0        ------------------------------------------------------------  9952
m141107_iid=1409934_0       ------------------------------------------------------------  10262
m150503_iid=3416920_0       ------------------------------------------------------------  10188
m141108_iid=1800121_0       ------------------------------------------------------------  10617
m141031_iid=147540_0        TTAAAATAAACAAAATATTATTAAAAAATAAGTAATTAAAACATAATAAAAATGAAAAT  11513
m150507_iid=3674924_0       ------------------------------------------------------------  10758
m150503_iid=2923751_0       ------------------------------------------------------------  10134
m141031_iid=115118_0        ------------------------------------------------------------  7981
m150430_iid=2722448_0       ------------------------------------------------------------  9403
m150507_iid=3102647_0       TTAAAATAAACAAAATATTATTAAAAAATAAGTAATTAAA--ATAATAAAAATGAAAAT  8582
m141108_iid=1811866_0       ------------------------------------------------------------  9038
m150501_iid=2948495_0       TTAAAATAAACAAAATATTATTAAAAAATAAGTAATTAAAACATAATAAAAATGAAAAT  9877
m150504_iid=3238195_0       ------------------------------------------------------------  10610
m150503_iid=3388511_0       TTAAAATAAACAAAATATTATTAAAAAATAAGTAATTAAAACATAATAAAAATGAAAAT  9921
m150505_iid=3777611_0       TTAAAATAAACAAAATATTATTAAAAAATAAGTAATTAAAACATAATAAAAATGAAAAT  11349
m150430_iid=2438833_0       TTAAAATAAACAAAATATTATTAAAAAATAAGTAATTAAAACATAATAAAAATGAAAAT  11678
m141108_iid=1698969_0       ------------------------------------------------------------  11147
m150430_iid=3059190_0       TTAAAATAAACAAAATATTATTAAAAAATAAGTAATTAAAACATAATAAAAATGAAAAT  8536
m141107_iid=1383067_0       ------------------------------------------------------------  7061
m141101_iid=597008_0        TTAAAATAAACAAAATATTATTAAAAAATAAGTAATTAAAACATAATAAAAATGAAAAT  5610
m150506_iid=2596496_0       ------------------------------------------------------------  10491
m141102_iid=910051_0        ------------------------------------------------------------  7428
m150505_iid=3784017_0       TTAAAATAAACAAAATATTATTAAAAAATAAGTAATTAAAACATAATAAAAATGAAAAT  11461
m141101_iid=682151_0        TTAAAATAA--AAAATATTATTAAAAAATAAGTAATTAAAACATAATAAAAATGAAAAT  10531
m150501_iid=3748029_0       TTAAAATAAACAAAATATTATTAAAAAATAAGTAATTAAAACATAAT-AAAATGAAAAT  11077
m150504_iid=2876272_0       TTAAAA-AAACAAAATATTATTAAAAAATAAGTAATTAAAACATAAT-AAAATGAAAAT  12928
NC_042820.1                 TTAAAATAAACAAAATATTATTAAAAAATAAGTAATTAAAACATAATAAAAATGAAAAT  13186
m141102_iid=1228825_0       ------------------------------------------------------------  11951
m150504_iid=2876274_0       TTAAAATAAACAAAATATTATTAAAAAATAAGTAATTAAAACATAATAAAAATGAAAAT  13001
m141030_iid=80036_0         ------------------------------------------------------------  11554
m150506_iid=2596494_0       ------------------------------------------------------------  10983
m150503_iid=2526862_0       TTAAAATAAAC-AAATATTATTAAAAAATAAGTAATTAAAACATAAT-AAAATGAAAAT  12849
m150506_iid=2567000_0       TTAAAATAAACAAAATATTATTAAAAAATAAGTAATTAAAACATAATAAAAATGAAAAT  10814
m150507_iid=3073271_0       ------------------------------------------------------------  11626
m150504_iid=3156087_0       ------------------------------------------------------------  11376
m150505_iid=2498502_0       TTAAAATAAACAAAATATTATTAAAAAATAAGTAATTAAAACATAATAAAAATGAAAAT  9587
m141102_iid=754227_0        ------------------------------------------------------------  10254
m150507_iid=3469514_0       ------------------------------------------------------------  10241
Rdo_MitoGenome              TTAAAATAAACAAAATATTATTAAAAAATAAGTAATTAAAACATAATAAAAATGAAAAT  13187
m141101_iid=614933_0        ------------------------------------------------------------  9134


m141031_iid=347546_0        ------------------------------------------------------------  7511
m150506_iid=3127414_0       TTAATCC---TATTAAATTTTATATTAAAAAATTATAAATATATATTAAAAAGCTTAT-C  9660
m141102_iid=1245517_0       ------------------------------------------------------------  7295
m141107_iid=1405727_0       ------------------------------------------------------------  7315
m141102_iid=987614_0        TCTAATCCTATTAAATATTATAT----TAAAAAATTATAATTATATTAAAAAGCTTATCC  11497
m141108_iid=1701429_0       TCTAATCCTATTAAATATTTTATATTAAAAAATTATAAATATATATTAAAAAGCTTATCC  8230
m141102_iid=987286_0        ------------------------------------------------------------  7493
m150430_iid=3272125_0       ------------------------------------------------------------  10013
m141102_iid=754225_0        ------------------------------------------------------------  9952
m141107_iid=1409934_0       ------------------------------------------------------------  10262
m150503_iid=3416920_0       ------------------------------------------------------------  10188
m141108_iid=1800121_0       ------------------------------------------------------------  10617
m141031_iid=147540_0        TCTAATCCTATTAAATATTTTATATTAAAAAATTATAAATATATATTAAAAAGCTTATCC  11573
m150507_iid=3674924_0       ------------------------------------------------------------  10758
m150503_iid=2923751_0       ------------------------------------------------------------  10134
```

```
m141031_iid=115118_0       ------------------------------------------------------------  7981
m150430_iid=2722448_0      ------------------------------------------------------------  9403
m150507_iid=3102647_0      TCTAATCCTATTAAATATTTTATATTAA-AAATTATAAATATATATTAAAAAGCTTATCC  8641
m141108_iid=1811866_0      ------------------------------------------------------------  9038
m150501_iid=2948495_0      TCTAATCCTATTAAATATTTTATATTAAAAAATTATAAATATATA-TTAAAAGCTTATC-  9935
m150504_iid=3238195_0      ------------------------------------------------------------  10610
m150503_iid=3388511_0      TCTAATCCTATTAAA-TATTTTATATTAAAAATTATAAATATATATTAAAAAGCTTATCC  9980
m150505_iid=3777611_0      TCTAATCCTATTAA--AATTTTATATTAAAAATTATAAATATATATTAAAAAGCTTATCC  11407
m150430_iid=2438833_0      TCTAATCCTATTAAATATTTTATATTAAAAAATTATAAATATATATTAAAAAGCTTATCC  11738
m141108_iid=1698969_0      ------------------------------------------------------------  11147
m150430_iid=3059190_0      TCTAATCCTATTAAATATTTTATATTAAAAA---ATAAATATATATTAAAAAGCTTATCC  8593
m141107_iid=1383067_0      ------------------------------------------------------------  7061
m141101_iid=597008_0       TCTAATCCTATTAAATATTTTATATTAAAAAATTATAAATATATATTAAAAAGCTTATCC  5670
m150506_iid=2596496_0      ------------------------------------------------------------  10491
m141102_iid=910051_0       ------------------------------------------------------------  7428
m150505_iid=3784017_0      TCTAATCCTATTAAATATTTTATATTAAAAAATTATAAATATATATTAAAAAGCTTATCC  11521
m141101_iid=682151_0       TCTAATCCTATTAAATATTTTATATTAAAAAATTATAAATATATATTAAAAAGCTTATCC  10591
m150501_iid=3748029_0      TCTAATCCTATTAAATATTTTATATTAAAAAATTATAAATATATATTAAAAAGCTTATCC  11137
m150504_iid=2876272_0      TCTAATCCTATTAAATATTTTATATTAAAAAATTATAAATATATATTAAAAAGCTTATCC  12988
NC_042820.1                TCTAATCCTATTAAATATTTTATATTAAAAAATTATAAATATATATTAAAAAGCTTATCC  13246
m141102_iid=1228825_0      ------------------------------------------------------------  11951
m150504_iid=2876274_0      TCTAATCCTATTAAATATTTTATATTAAAAAATTATAAATATATATTAAAAAGCTTATCC  13061
m141030_iid=80036_0        ------------------------------------------------------------  11554
m150506_iid=2596494_0      ------------------------------------------------------------  10983
m150503_iid=2526862_0      TCTAATCCTATTAAATATTTTATATTAAAAAATTATAAATATATATTAAAAAGCTTATCC  12909
m150506_iid=2567000_0      TCTAATCCTATTAAATATTTTATATTAAAAAATTATAAATATATATTAAAAAGCTTATCC  10874
m150507_iid=3073271_0      ------------------------------------------------------------  11626
m150504_iid=3156087_0      ------------------------------------------------------------  11376
m150505_iid=2498502_0      TCTAATCCTATTAAATATTTTATATTAAAAAATTATAAATATATATTAAAAAGCTTATCC  9647
m141102_iid=754227_0       ------------------------------------------------------------  10254
m150507_iid=3469514_0      ------------------------------------------------------------  10241
Rdo_MitoGenome             TCTAATCCTATTAAATATTTTATATTAAAAAATTATAAATATATATTAAAAAGCTTATCC  13247
m141101_iid=614933_0       ------------------------------------------------------------  9134


m141031_iid=347546_0       ------------------------------------------------------------  7511
m150506_iid=3127414_0      CCTTTAATATTTAAGCTCATAAG--AAAAATTATCAATAAAAAATCTTTCCTAAAGAACA  9718
m141102_iid=1245517_0      ------------------------------------------------------------  7295
m141107_iid=1405727_0      ------------------------------------------------------------  7315
m141102_iid=987614_0       CCTTTAATATTTAAGCTCATAAG-AAAAAATTATCAATAAAAAATCTTTCCTAAAGAACA  11556
m141108_iid=1701429_0      CCTTTAATATTTAAGCTCATAAGAAAAAAATTATCAATAAAAAATCTTTCCTAAAGAACA  8290
m141102_iid=987286_0       ------------------------------------------------------------  7493
m150430_iid=3272125_0      ------------------------------------------------------------  10013
m141102_iid=754225_0       ------------------------------------------------------------  9952
m141107_iid=1409934_0      ------------------------------------------------------------  10262
m150503_iid=3416920_0      ------------------------------------------------------------  10188
m141108_iid=1800121_0      ------------------------------------------------------------  10617
m141031_iid=147540_0       CCTTTAATATTTAAGCTCATAAGAAAAAAATTATCAATAAAAAATCTTTCCTAAAGAACA  11633
m150507_iid=3674924_0      ------------------------------------------------------------  10758
m150503_iid=2923751_0      ------------------------------------------------------------  10134
m141031_iid=115118_0       ------------------------------------------------------------  7981
m150430_iid=2722448_0      ------------------------------------------------------------  9403
m150507_iid=3102647_0      CCTTTAATATTTAAGCTCATAAGAAAAAAATTATCAATAAAAAATCTTTCCTAAAGAACA  8701
m141108_iid=1811866_0      ------------------------------------------------------------  9038
m150501_iid=2948495_0      CCTTTAATATTTAAGCTCATAAGAAAAAAATTATCAATAAAAAATCTTTCCTAAAGAACA  9995
m150504_iid=3238195_0      ------------------------------------------------------------  10610
m150503_iid=3388511_0      CCTTTAATATTTAAGCTCATAAGAAAAAAATTATCAAT-AAAAATCTTTCCTAAAGAACA  10039
m150505_iid=3777611_0      CCTTTAATATTTAAGCTCATAAGAAAAAAATTATCAATAAAAAATCTTTCCTAAAGAACA  11467
m150430_iid=2438833_0      CCTTTAATATTTAAGCTCATAAGAAAAAAATTATCAATAAAAAATCTTTCCTAAAGAACA  11798
m141108_iid=1698969_0      ------------------------------------------------------------  11147
m150430_iid=3059190_0      CCTTTAATATTTAAGCTCATAAGAAAA-AATTATCAATAAAAAATCTTTCCTAAAGAACA  8652
m141107_iid=1383067_0      ------------------------------------------------------------  7061
m141101_iid=597008_0       CCTTTAATATTTAAGCTCATAAGAAAA-AATTATCAATAAAAAATCTTTCCTAAAGAACA  5729
m150506_iid=2596496_0      ------------------------------------------------------------  10491
m141102_iid=910051_0       ------------------------------------------------------------  7428
m150505_iid=3784017_0      CCTTTAATATTTAAGCTCATAAGAAAAAAATTATCAAT-AAAAATCTTTCCTAAAGAACA  11580
m141101_iid=682151_0       CCTTTAATATTTAAGCTCATAAGAAAAAAATTATCAAT-AAAAATCTTTCCTAAAGAACA  10650
m150501_iid=3748029_0      CCTTTAATATTTAAGCTCATAAGAAAAAAATTATCAATAAAAAATCTTTCCTAAAGAACA  11197
m150504_iid=2876272_0      CCTTTAATATTTAAGCTCATAAGAAAAAAATTATCAATAAAAAATCTTTCCTAAAGAACA  13048
NC_042820.1                CCTTTAATATTTAAGCTCATAAGAAAAAAATTATCAATAAAAAATCTTTCCTAAAGAACA  13306
m141102_iid=1228825_0      ------------------------------------------------------------  11951
```

```
m150504_iid=2876274_0      CCTTTAATATTTAAGCTCATAAGAAAAAAATTATCAATAAAAAATCTTTCCTAAAGAACA   13121
m141030_iid=80036_0        ------------------------------------------------------------   11554
m150506_iid=2596494_0      ------------------------------------------------------------   10983
m150503_iid=2526862_0      CCTTTAATATTTAAGCTCATAAGAAAAAAATTATCAATAAAAAATCTTTCCTAAAGAACA   12969
m150506_iid=2567000_0      CCTTTAATATTTAAGCTCATAAGAAAAAAATTATCAATAAAAAATCTTTCCTAAAGAACA   10934
m150507_iid=3073271_0      ------------------------------------------------------------   11626
m150504_iid=3156087_0      ------------------------------------------------------------   11376
m150505_iid=2498502_0      CCTTTAATATTTAAGCTCATAAGAAAAAAATTATCAATAAAAAATCTTTCCTAAAGAACA   9707
m141102_iid=754227_0       ------------------------------------------------------------   10254
m150507_iid=3469514_0      ------------------------------------------------------------   10241
Rdo_MitoGenome             CCTTTAATATTTAAGCTCATAAGAAAAAAATTATCAATAAAAAATCTTTCCTAAAGAACA   13307
m141101_iid=614933_0       ------------------------------------------------------------   9134


m141031_iid=347546_0       ------------------------------------------------------------   7511
m150506_iid=3127414_0      TAAAATTTAATTTTT---TATTTAAAACCAGATATATCAAA--ACGAATTACATTTCAAA   9773
m141102_iid=1245517_0      ------------------------------------------------------------   7295
m141107_iid=1405727_0      ------------------------------------------------------------   7315
m141102_iid=987614_0       TAAAATTTAATTT--TTTATTTAAAAACCAGATATATCAAAAGACGAATTACATTTCAAA   11614
m141108_iid=1701429_0      TAAAATTTAATTT--TTTATTTAAAAACCAGATATATCAAAAGACGAATTACATTTCAAA   8348
m141102_iid=987286_0       ------------------------------------------------------------   7493
m150430_iid=3272125_0      ------------------------------------------------------------   10013
m141102_iid=754225_0       ------------------------------------------------------------   9952
m141107_iid=1409934_0      ------------------------------------------------------------   10262
m150503_iid=3416920_0      ------------------------------------------------------------   10188
m141108_iid=1800121_0      ------------------------------------------------------------   10617
m141031_iid=147540_0       TAAAATTTAATTTTTTT-ATTTAAAAACCAGATATATCAAAAGACGAATTACATTTCAAA   11692
m150507_iid=3674924_0      ------------------------------------------------------------   10758
m150503_iid=2923751_0      ------------------------------------------------------------   10134
m141031_iid=115118_0       ------------------------------------------------------------   7981
m150430_iid=2722448_0      ------------------------------------------------------------   9403
m150507_iid=3102647_0      TAAAATTTAATTTT-TTTATTTAAAAACCAGATATATCAAAAGACGAATTACATTTCAAA   8760
m141108_iid=1811866_0      ------------------------------------------------------------   9038
m150501_iid=2948495_0      TAAAATTTAATTTT-TTTATTTAAAAACCAGATATATCAAAAGACGAATTACATTTCAAA   10054
m150504_iid=3238195_0      ------------------------------------------------------------   10610
m150503_iid=3388511_0      TAAAATTTAATTTTTTT-ATTTAAAAACCAGATATATCAAAAGACGAATTACATTTCAAA   10098
m150505_iid=3777611_0      TAAAATTTAATTTTTTT-ATTTAAAAACCAGATATATCAAAAGACGAATTACATTTCAAA   11526
m150430_iid=2438833_0      TAAAATTTAATTTTT---ATTTAAAAACCAGATATATCAAAAGACGAATTACATTTCAAA   11855
m141108_iid=1698969_0      ------------------------------------------------------------   11147
m150430_iid=3059190_0      TAAAATTTAATTTTTTTTATTTAAAAACCAGATATATCAAAAGACGAATTACATTTCAAA   8712
m141107_iid=1383067_0      ------------------------------------------------------------   7061
m141101_iid=597008_0       TAAAATTTAATTTTTTT-ATTTAAAAACCAGATATATCAAAAGACGAATTACATTTCAAA   5788
m150506_iid=2596496_0      ------------------------------------------------------------   10491
m141102_iid=910051_0       ------------------------------------------------------------   7428
m150505_iid=3784017_0      TAAAATTTAATTTTTTT-ATTTAAAAACCAGATATATCAAAAGACGAATTACATTTCAAA   11639
m141101_iid=682151_0       TAAAATTTAATTTTTTT-ATTTAAAAACCAGATATATCAAAAGACGAATTACATTTCAAA   10709
m150501_iid=3748029_0      TAAAATTTAATTTTTTT-ATTTAAAAACCAGATATATCAAAAGACGAATTACATTTCAAA   11256
m150504_iid=2876272_0      TAAAATTTAATTTTTTT-ATTTAAAAACCAGATATATCAAAAGACGAATTACATTTCAAA   13107
NC_042820.1                TAAAATTTAATTTTTTT-ATTTAAAAACCAGATATATCAAAAGACGAATTACATTTCAAA   13365
m141102_iid=1228825_0      ------------------------------------------------------------   11951
m150504_iid=2876274_0      TAAAATTTAATTTTTTT-ATTTAAAAACCAGATATATCAAAAGACGAATTACATTTCAAA   13180
m141030_iid=80036_0        ------------------------------------------------------------   11554
m150506_iid=2596494_0      ------------------------------------------------------------   10983
m150503_iid=2526862_0      TAAAATTTAATTTTTTT-ATTTAAAAACCAGATATATCAAAAGACGAATTACATTTCAAA   13028
m150506_iid=2567000_0      TAAAATTTAATTTTTTT-ATTTAAAAACCAGATATATCAAAAGACGAATTACATTTCAAA   10993
m150507_iid=3073271_0      ------------------------------------------------------------   11626
m150504_iid=3156087_0      ------------------------------------------------------------   11376
m150505_iid=2498502_0      TAAAATTTAATT-TTTT-ATTTAAAAACCAGATATATCAAAAGACGAATTACATTTCAAA   9765
m141102_iid=754227_0       ------------------------------------------------------------   10254
m150507_iid=3469514_0      ------------------------------------------------------------   10241
Rdo_MitoGenome             TAAAATTTAATTTTTTT-ATTTAAAAACCAGATATATCAAAAGACGAATTACATTTCAAA   13366
m141101_iid=614933_0       ------------------------------------------------------------   9134


m141031_iid=347546_0       ------------------------------------------------------------   7511
m150506_iid=3127414_0      TCAAAATTAATATTATAAATTATTA-TTCAACATAAAAATTTAT-AAAAATTAACTCCTT   9831
m141102_iid=1245517_0      ------------------------------------------------------------   7295
m141107_iid=1405727_0      ------------------------------------------------------------   7315
m141102_iid=987614_0       TCAAAATTAATATTATAAATTATTATTTCAACATAAAAATTTATAAAAAATTAACTCCTT   11674
m141108_iid=1701429_0      TCAAAATTAATATTATAAATTATTA-TTCAACATAAAAATTTAT-AAAAATTAACTCCTT   8406
m141102_iid=987286_0       ------------------------------------------------------------   7493
```

```
m150430_iid=3272125_0      ------------------------------------------------------------  10013
m141102_iid=754225_0       ------------------------------------------------------------  9952
m141107_iid=1409934_0      ------------------------------------------------------------  10262
m150503_iid=3416920_0      ------------------------------------------------------------  10188
m141108_iid=1800121_0      ------------------------------------------------------------  10617
m141031_iid=147540_0       TCAAAATTAATATTATAAATTATTA-TTCAACATAAAAATTTATAAAAAATTAACTCCTT  11751
m150507_iid=3674924_0      ------------------------------------------------------------  10758
m150503_iid=2923751_0      ------------------------------------------------------------  10134
m141031_iid=115118_0       ------------------------------------------------------------  7981
m150430_iid=2722448_0      ------------------------------------------------------------  9403
m150507_iid=3102647_0      TCAAAATTAATATTATAAATTATTA-TTCAACATAAAAATTTATAAAAAATTAACTCCTT  8819
m141108_iid=1811866_0      ------------------------------------------------------------  9038
m150501_iid=2948495_0      TCAAAATTAATATTATAAATTATTA-TTCAACATAAAAATTTATAAAAAATTAACTCCTT  10113
m150504_iid=3238195_0      ------------------------------------------------------------  10610
m150503_iid=3388511_0      TCAAAATTAATATTATAAATTATTA-TTCAACATAAAAATTTATAAAAAATTAACTCCTT  10157
m150505_iid=3777611_0      TCAAAATTAATATTATAAATTATTA-TTCAACATAAAAATTTATAAAAAATTAACTCCTT  11585
m150430_iid=2438833_0      TCAAAATTAATATTATAAATTATTA-TTCAACATAAAAATTTATAAAAAATTAACTCCTT  11914
m141108_iid=1698969_0      ------------------------------------------------------------  11147
m150430_iid=3059190_0      TCAAAATTAATATTATAAATTATTA-TTCAACATAAAAATTTATAAAAAATTAACTCCTT  8771
m141107_iid=1383067_0      ------------------------------------------------------------  7061
m141101_iid=597008_0       TCAAAATTAATATTATAAATTATTA-TTCAACATAAAAATTTATAAAAAATTAACTCCTT  5847
m150506_iid=2596496_0      ------------------------------------------------------------  10491
m141102_iid=910051_0       ------------------------------------------------------------  7428
m150505_iid=3784017_0      TCAAAATTAATATTATAAATTATTA-TTCAACATAAAAATTTATAAAAAATTAACTCCTT  11698
m141101_iid=682151_0       TCAAAATTAATATTATAAATTATTA-TTCAACATAAAAATTTATAAAAAATTAACTCCTT  10768
m150501_iid=3748029_0      TCAAAATTAATATTATAAATTATTA-TTCAACATAAAAATTTATAAAAAATTAACTCCTT  11315
m150504_iid=2876272_0      TCAAAATTAATATTATAAATTATTA-TTCAACATAAAAATTTATAAAAAATTAACTCCTT  13166
NC_042820.1                TCAAAATTAATATTATAAATTATTA-TTCAACATAAAAATTTATAAAAAATTAACTCCTT  13424
m141102_iid=1228825_0      ------------------------------------------------------------  11951
m150504_iid=2876274_0      TCAAAATTAATATTATAAATTATTA-TTCAACATAAAAATTTATAAAAAATTAACTCCTT  13239
m141030_iid=80036_0        ------------------------------------------------------------  11554
m150506_iid=2596494_0      ------------------------------------------------------------  10983
m150503_iid=2526862_0      TCAAAATTAATATTATAAATTATTA-TTCAACATAAAAATTTATAAAAAATTAACTCCTT  13087
m150506_iid=2567000_0      TCAAAATTAATATTATAAATTATTA-TTCAACATAAAAATTTATAAAAAATTAACTCCTT  11052
m150507_iid=3073271_0      ------------------------------------------------------------  11626
m150504_iid=3156087_0      ------------------------------------------------------------  11376
m150505_iid=2498502_0      TCAAAATTAATATTATAAATTATTA-TTCAACATAAAAATTTATAAAAAATTAACTCCTT  9824
m141102_iid=754227_0       ------------------------------------------------------------  10254
m150507_iid=3469514_0      ------------------------------------------------------------  10241
Rdo_MitoGenome             TCAAAATTAATATTATAAATTATTA-TTCAACATAAAAATTTATAAAAAATTAACTCCTT  13425
m141101_iid=614933_0       ------------------------------------------------------------  9134


m141031_iid=347546_0       ------------------------------------------------------------  7511
m150506_iid=3127414_0      TTGATTCGGGATTAATATATACATATAAAAAAATTAATCAACCCTGATACACAAGGTACA  9891
m141102_iid=1245517_0      ------------------------------------------------------------  7295
m141107_iid=1405727_0      ------------------------------------------------------------  7315
m141102_iid=987614_0       TTGATTCGGGATTAATATATACATAT-AAAAAATTAATCAACCCTGATACACAAGGTACA  11733
m141108_iid=1701429_0      TTGATTCGGGATTAATATATACATATAAAAAAATTAATCAACCCTGATACACAAGGTACA  8466
m141102_iid=987286_0       ------------------------------------------------------------  7493
m150430_iid=3272125_0      ------------------------------------------------------------  10013
m141102_iid=754225_0       ------------------------------------------------------------  9952
m141107_iid=1409934_0      ------------------------------------------------------------  10262
m150503_iid=3416920_0      ------------------------------------------------------------  10188
m141108_iid=1800121_0      ------------------------------------------------------------  10617
m141031_iid=147540_0       TTGATTCGGGATTAATATATACATATAAA-------------------------------  11780
m150507_iid=3674924_0      ------------------------------------------------------------  10758
m150503_iid=2923751_0      ------------------------------------------------------------  10134
m141031_iid=115118_0       ------------------------------------------------------------  7981
m150430_iid=2722448_0      ------------------------------------------------------------  9403
m150507_iid=3102647_0      TTGATTCGGGATTAATATATACATATAAAAAAATTAATCAACCCTGATACACAAGGTACA  8879
m141108_iid=1811866_0      ------------------------------------------------------------  9038
m150501_iid=2948495_0      TTGATTCGGGATTAATATATACATATAAAAAAATTAATCAACCCTGATACACAAGGTACA  10173
m150504_iid=3238195_0      ------------------------------------------------------------  10610
m150503_iid=3388511_0      TTGATTCGGGATTAATATATACATATAAAAAAATTAATCAACCCTGATACACAAGGTACA  10217
m150505_iid=3777611_0      TTGATTCGGGATTAATATATACATATAAAAAAATTAATCAACCCTGATACACAAGGTACA  11645
m150430_iid=2438833_0      TTGATTCGGGATTAATATATACATATAAAAAAATTAATCAACCCTGATACACAAGGTACA  11974
m141108_iid=1698969_0      ------------------------------------------------------------  11147
m150430_iid=3059190_0      TTGATTCGGGATTAATATATACATATAAAAAAATTAATCAACCCTGATACACAAGGTACA  8831
m141107_iid=1383067_0      ------------------------------------------------------------  7061
m141101_iid=597008_0       TTGATTCGGGATTAATATATACATATAAAAAAATTAATCAACCCTGATACACAAGGTACA  5907
```

```
m150506_iid=2596496_0      ------------------------------------------------------------  10491
m141102_iid=910051_0       ------------------------------------------------------------  7428
m150505_iid=3784017_0      TTGATTCGGGATTAATATATACATATAAAAAAATTAATCAACCCTGATACACAAGGTACA  11758
m141101_iid=682151_0       TTGATTCGGGATTAATATATACATATAAAAAAATTAATCAACCCTGATACACAAGGTACA  10828
m150501_iid=3748029_0      TTGATTCGGGATTAATATATACATATAAAAAAATTAATCAACCCTGATACACAAGGTACA  11375
m150504_iid=2876272_0      TTGATTCGGGATTAATATATACATATAAAAAAATTAATCAACCCTGATACACAAGGTACA  13226
NC_042820.1                TTGATTCGGGATTAATATATACATATAAAAAAATTAATCAACCCTGATACACAAGGTACA  13484
m141102_iid=1228825_0      ------------------------------------------------------------  11951
m150504_iid=2876274_0      TTGATTCGGGATTAATATATACATATAAAAAAATTAATCAACCCTGATACACAAGGTACA  13299
m141030_iid=80036_0        ------------------------------------------------------------  11554
m150506_iid=2596494_0      ------------------------------------------------------------  10983
m150503_iid=2526862_0      TTGATTCGGGATTAATATAT-CATATAAAAAAATTAATCAACCCTGATACACAAGGTACA  13146
m150506_iid=2567000_0      TTGATTCGGGATTAATATATACATATAAAAAAATTAATCAACCCTGATACACAAGGTACA  11112
m150507_iid=3073271_0      ------------------------------------------------------------  11626
m150504_iid=3156087_0      ------------------------------------------------------------  11376
m150505_iid=2498502_0      TTGATTCGGGATTAATATATACATATAAAAAAATTAATCAACCCTGATACACAAGGTACA  9884
m141102_iid=754227_0       ------------------------------------------------------------  10254
m150507_iid=3469514_0      ------------------------------------------------------------  10241
Rdo_MitoGenome             TTGATTCGGGATTAATATATACATATAAAAAAATTAATCAACCCTGATACACAAGGTACA  13485
m141101_iid=614933_0       ------------------------------------------------------------  9134


m141031_iid=347546_0       ------------------------------------------------------------  7511
m150506_iid=3127414_0      ACAAATAAAATTTTCTTTGTCAATAATATATAATAAACCTTCACAGTACAAAAAAATAAT  9951
m141102_iid=1245517_0      ------------------------------------------------------------  7295
m141107_iid=1405727_0      ------------------------------------------------------------  7315
m141102_iid=987614_0       AACAATAAAATTTTCTTTGTCAATAATATATAATAAACCTTCACAGTACAAAAAAATAAT  11793
m141108_iid=1701429_0      ACAAATAAAATTTTCTTTGTCAATAAA-TATAATAAACCTTCACAGTACAAAAAATAATT  8525
m141102_iid=987286_0       ------------------------------------------------------------  7493
m150430_iid=3272125_0      ------------------------------------------------------------  10013
m141102_iid=754225_0       ------------------------------------------------------------  9952
m141107_iid=1409934_0      ------------------------------------------------------------  10262
m150503_iid=3416920_0      ------------------------------------------------------------  10188
m141108_iid=1800121_0      ------------------------------------------------------------  10617
m141031_iid=147540_0       ------------------------------------------------------------  11780
m150507_iid=3674924_0      ------------------------------------------------------------  10758
m150503_iid=2923751_0      ------------------------------------------------------------  10134
m141031_iid=115118_0       ------------------------------------------------------------  7981
m150430_iid=2722448_0      ------------------------------------------------------------  9403
m150507_iid=3102647_0      ACAAATAAAATTTTCTTTGTCAATAATATATAATAAACCTTCACAGTACAAAAAAATAAT  8939
m141108_iid=1811866_0      ------------------------------------------------------------  9038
m150501_iid=2948495_0      ACAAATAAAATTTTCTTTGTCAATAATATATAATAAACCTTCACAGTACAAAAATAAT--  10231
m150504_iid=3238195_0      ------------------------------------------------------------  10610
m150503_iid=3388511_0      ACAAATAAAATTTTCTTTGTCAATAATATATAATAAACCTTCACAGTACAAAAAAATAAT  10277
m150505_iid=3777611_0      ACAAATAAAATTTTCTTTGTCAATAATATATAATAAACCTTCACAGTA-AAAAAAAAATA  11704
m150430_iid=2438833_0      ACAAATAAAATTTTCTTTGTCAATAATATATAATAAACCTTCACAGTACAAAAAAATAAT  12034
m141108_iid=1698969_0      ------------------------------------------------------------  11147
m150430_iid=3059190_0      ACAAATAAAATTTTCTTTGTCAATAATA--TAATAAACCTTCACAGTACAAAAAAATAAT  8889
m141107_iid=1383067_0      ------------------------------------------------------------  7061
m141101_iid=597008_0       ACAAATAAAATTTTCTTTGTCAATAATATATAATAAACCTTCACAGTACAAAAAAATAAT  5967
m150506_iid=2596496_0      ------------------------------------------------------------  10491
m141102_iid=910051_0       ------------------------------------------------------------  7428
m150505_iid=3784017_0      ACAAATAAAATTTTCTTTGTCAATAATATATAATAAACCTTCACAGTAC-AAAAAATAAT  11817
m141101_iid=682151_0       ACAAATAAAATTTTCTTTGTCAATAATATATAATAAACCTTCACAGTAC-AAAAAATAAT  10887
m150501_iid=3748029_0      ACAAATAAAATTTTCTTTGTCAATAATATATAATAAACCTTCACAGTACAAAAAAATAAT  11435
m150504_iid=2876272_0      ACAAATAAAATTTTCTTTGTCAATAATATATAATAAACCTTCACAGTACAAAAAAATAAT  13286
NC_042820.1                ACAAATAAAATTTTCTTTGTCAATAATATATAATAAACCTTCACAGTACAAAAAAATAAT  13544
m141102_iid=1228825_0      ------------------------------------------------------------  11951
m150504_iid=2876274_0      ACAAATAAAATTTTCTTTGTCAATAATATATAATAAACCTTCACAGTACAAAAAATAATT  13359
m141030_iid=80036_0        ------------------------------------------------------------  11554
m150506_iid=2596494_0      ------------------------------------------------------------  10983
m150503_iid=2526862_0      ACAAATAAAATTTTCTTTGTCAATAATATATAATAAACCTTCACAGTACAAAAAATAATT  13206
m150506_iid=2567000_0      ACAAATAAAATTTTCTTTGTCAATAATATATAATAAACCTTCACAGTACAAAAAAATAAT  11172
m150507_iid=3073271_0      ------------------------------------------------------------  11626
m150504_iid=3156087_0      ------------------------------------------------------------  11376
m150505_iid=2498502_0      ACAAATAAAATTTTCTTTGTCAATAATATATAATAAACCTTCACAGTACAAAAAAATAAT  9944
m141102_iid=754227_0       ------------------------------------------------------------  10254
m150507_iid=3469514_0      ------------------------------------------------------------  10241
Rdo_MitoGenome             ACAAATAAAATTTTCTTTGTCAATAATATATAATAAACCTTCACAGTACAAAAAAATAAT  13545
m141101_iid=614933_0       ------------------------------------------------------------  9134
```

```
m141031_iid=347546_0        ------------------------------------------------------------   7511
m150506_iid=3127414_0       TTTTTAAATAAA---------AACTAAAAAAAAATAATACTTTAAAAAAATAAATAAATAA   10002
m141102_iid=1245517_0       ------------------------------------------------------------   7295
m141107_iid=1405727_0       ------------------------------------------------------------   7315
m141102_iid=987614_0        TTT--TTTAAAATAAAAAACTAAAAAAAATAATACTTTAAAAAAATAATTTAAATAAAAA   11851
m141108_iid=1701429_0       TTT--AAAATAAAAACTAAAAAAAAATAATACTTTAAAAAAATAAATTTAAATAAAAAAA   8583
m141102_iid=987286_0        ------------------------------------------------------------   7493
m150430_iid=3272125_0       ------------------------------------------------------------   10013
m141102_iid=754225_0        ------------------------------------------------------------   9952
m141107_iid=1409934_0       ------------------------------------------------------------   10262
m150503_iid=3416920_0       ------------------------------------------------------------   10188
m141108_iid=1800121_0       ------------------------------------------------------------   10617
m141031_iid=147540_0        ------------------------------------------------------------   11780
m150507_iid=3674924_0       ------------------------------------------------------------   10758
m150503_iid=2923751_0       ------------------------------------------------------------   10134
m141031_iid=115118_0        ------------------------------------------------------------   7981
m150430_iid=2722448_0       ------------------------------------------------------------   9403
m150507_iid=3102647_0       TTTTTAAAATAAAAACTAAA-A--AATAATACTTTAAAAAAATAAATTTAAATAAAAAAA   8996
m141108_iid=1811866_0       ------------------------------------------------------------   9038
m150501_iid=2948495_0       TTTTTAAAATAAAAACTAAA-AAAAATAATACTTTAAAAAAATAAATTTAAATAAAAAAA   10290
m150504_iid=3238195_0       ------------------------------------------------------------   10610
m150503_iid=3388511_0       TTTTTAAAATAAAAACTAAA-AAAAATAATACTTTAAAAAAA-TAAATTTAAATAAAAAA   10335
m150505_iid=3777611_0       TTTTTAAAATAAAAACTAAA-AAAAATAATACTTTAAAAAA--TAAATTTAAATAAAAAA   11761
m150430_iid=2438833_0       TTTTAAAATAAAAAC--TAA-AAAAATAATACTTTAAAAAAATAAATTTAAATAAAAAAA   12091
m141108_iid=1698969_0       ------------------------------------------------------------   11147
m150430_iid=3059190_0       TTTTTAAAATAAAAACTAAA-AA--ATAATACTTTAAAAAAATAAATTTAAAT-AAAAAA   8945
m141107_iid=1383067_0       ------------------------------------------------------------   7061
m141101_iid=597008_0        TTTTTAAAATAAAAACTAAA-AAAAATAATACTTTAAAAAAATAAATTTAAATAAAAAAA   6026
m150506_iid=2596496_0       ------------------------------------------------------------   10491
m141102_iid=910051_0        ------------------------------------------------------------   7428
m150505_iid=3784017_0       TTTTTAAAATAAAAACTAAA-AAAAATAATACTTTAAAAAA--TAAATTTAAATAAAAAA   11874
m141101_iid=682151_0        TTTTTAAAATAAAAACTAAA-AAAAATAATACTTTAAAAAAATAAATTTAAATAAAAAAA   10946
m150501_iid=3748029_0       TTTTTAAAATAAAAACTAAA-AAAAATAATACTTTAAAAAAATAAATTTAAATAAAAAAA   11494
m150504_iid=2876272_0       TTTTTAAAATAAAAACT-AA-AAAAATAATACTTTAAAAAAATAAATTTAAATAAAAAAA   13344
NC_042820.1                 TTTTTAAAATAAAAACTAAA-AAAAATAATACTTTAAAAAAATAAATTTAAATAAAAAAA   13603
m141102_iid=1228825_0       ------------------------------------------------------------   11951
m150504_iid=2876274_0       TTTTAAAATAAAAAC--TAA-AAAAATAATACTTTAAAAAAATAAATTTAAATAAAAAAA   13416
m141030_iid=80036_0         ------------------------------------------------------------   11554
m150506_iid=2596494_0       ------------------------------------------------------------   10983
m150503_iid=2526862_0       TTTTAAAATAAAAC---TAA-AAAAATAATACTTTAAAAAAATAAATTTAAATAAAAAAA   13262
m150506_iid=2567000_0       TTTTTAAAATAAAAACTAAA-AAAAATAATACTTTAAAAAAAT-AATTTAAATAAAAAAA   11230
m150507_iid=3073271_0       ------------------------------------------------------------   11626
m150504_iid=3156087_0       ------------------------------------------------------------   11376
m150505_iid=2498502_0       TTTTTAAAATAAAAACTAAA-AAAAATAATACTTTAAAAAAATAAATTTAAATAAAAAAA   10003
m141102_iid=754227_0        ------------------------------------------------------------   10254
m150507_iid=3469514_0       ------------------------------------------------------------   10241
Rdo_MitoGenome              TTTTTAAAATAAAAACTAAA-AAAAATAATACTTTAAAAAAATAAATTTAAATAAAAAAA   13604
m141101_iid=614933_0        ------------------------------------------------------------   9134


m141031_iid=347546_0        ------------------------------------------------------------   7511
m150506_iid=3127414_0       AATAAATTATTTATCTAACTAAGTTAAAAACTATCCTTTTAGTGTAAATAAAATGCTTAT   10062
m141102_iid=1245517_0       ------------------------------------------------------------   7295
m141107_iid=1405727_0       ------------------------------------------------------------   7315
m141102_iid=987614_0        AATAAATATATTATCAAACTAAGTTTAAAAACTT--------------------------   11885
m141108_iid=1701429_0       ATAAAATATATTATCAAACTAAGTTAAAAACTATCCTTTTAGTGTAAATAAAATGCTTAT   8643
m141102_iid=987286_0        ------------------------------------------------------------   7493
m150430_iid=3272125_0       ------------------------------------------------------------   10013
m141102_iid=754225_0        ------------------------------------------------------------   9952
m141107_iid=1409934_0       ------------------------------------------------------------   10262
m150503_iid=3416920_0       ------------------------------------------------------------   10188
m141108_iid=1800121_0       ------------------------------------------------------------   10617
m141031_iid=147540_0        ------------------------------------------------------------   11780
m150507_iid=3674924_0       ------------------------------------------------------------   10758
m150503_iid=2923751_0       ------------------------------------------------------------   10134
m141031_iid=115118_0        ------------------------------------------------------------   7981
m150430_iid=2722448_0       ------------------------------------------------------------   9403
m150507_iid=3102647_0       ATAAAATATATTATCAAACTAAGTTAAAAACTATCCTTTTAGTGTAAATAAAATGCTTAT   9056
m141108_iid=1811866_0       ------------------------------------------------------------   9038
m150501_iid=2948495_0       ATAAAATATATTATCAAACTAAGTTA-AAACTATCCTTTTAGTGTAAATAAAATGCTTAT   10349
```

```
m150504_iid=3238195_0     ------------------------------------------------------------  10610
m150503_iid=3388511_0     AATAAAATATTTATCAAACTAAGTTAAAAACTATCCTTTTAGTGTAAATAAAATGCTTAT  10395
m150505_iid=3777611_0     ATAAAATATATTATCAAACTAAGTTAAAAACTATCCTTTTAGTGTAAATAAAATGCTTA-  11820
m150430_iid=2438833_0     ATAAAATATATTATCAAACTAAGTTAAAAACTATCCTTTTAGTGTAAATAAAATGCTTAT  12151
m141108_iid=1698969_0     ------------------------------------------------------------  11147
m150430_iid=3059190_0     ATAAAATATATTATCAAACTAAGTTAAAAACTATCCTTTTAGTGTAAATAAAATGCTTAT  9005
m141107_iid=1383067_0     ------------------------------------------------------------  7061
m141101_iid=597008_0      ATAAAATATATTATCAAACTAAGTTAAAAACTATCCTTTTAGTGTAAATAAAATGCTTAT  6086
m150506_iid=2596496_0     ------------------------------------------------------------  10491
m141102_iid=910051_0      ------------------------------------------------------------  7428
m150505_iid=3784017_0     ATAAAATATATTATCAAACTAAGTTAAAAACTATCCTTTTAGTGTAAATAAAATGCTTAT  11934
m141101_iid=682151_0      ATAAAATATATTATCAAACTAAGTTAAAAACTATCCTTTTAGTGTAAATAAAATGCTTAT  11006
m150501_iid=3748029_0     ATAAAATATATTATCAAACTAAGTTAAAAACTATCCTTTTAGTGTAAATAAAATGCTTAT  11554
m150504_iid=2876272_0     ATAAAATATATTATCAAACTAAGTTAAAAACTATCCTTTTAGTGTAAATAAAATGCTTAT  13404
NC_042820.1               ATAAAATATATTATCAAACTAAGTTAAAAACTATCCTTTTAGTGTAAATAAAATGCTTAT  13663
m141102_iid=1228825_0     ------------------------------------------------------------  11951
m150504_iid=2876274_0     ATAAAATATATTATCAAACTAAGTTAAAAACTATCCTTTTAGTGTAAATAAAATGCTTAT  13476
m141030_iid=80036_0       ------------------------------------------------------------  11554
m150506_iid=2596494_0     ------------------------------------------------------------  10983
m150503_iid=2526862_0     ATAAAATATATTATCAAACTAAGTTAAAAACTATCCTTTTAGTGTAAATAAAATGCTTAT  13322
m150506_iid=2567000_0     ATAAAATATATTATCAAACTAAGTTAAAAACTATCCTTTTAGTGTAAATAAAATGCTTAT  11290
m150507_iid=3073271_0     ------------------------------------------------------------  11626
m150504_iid=3156087_0     ------------------------------------------------------------  11376
m150505_iid=2498502_0     ATAAAATATATTATCAAACTAAGTTAAAAACTATCCTTTTAGTGTAAATAAAATGCTTAT  10063
m141102_iid=754227_0      ------------------------------------------------------------  10254
m150507_iid=3469514_0     ------------------------------------------------------------  10241
Rdo_MitoGenome            ATAAAATATATTATCAAACTAAGTTAAAAACTATCCTTTTAGTGTAAATAAAATGCTTAT  13664
m141101_iid=614933_0      ------------------------------------------------------------  9134


m141031_iid=347546_0      ------------------------------------------------------------  7511
m150506_iid=3127414_0     TCAAGCTCCAGTTTGATATTATCAGACACACTTTCCAGTACGCCTACTATGTT-CGACTT  10121
m141102_iid=1245517_0     ------------------------------------------------------------  7295
m141107_iid=1405727_0     ------------------------------------------------------------  7315
m141102_iid=987614_0      ------------------------------------------------------------  11885
m141108_iid=1701429_0     TCAAGCTCCAGTTTGATATATCCAGACACACTTTCCAGTACGCCTACTATGTTACGACTT  8703
m141102_iid=987286_0      ------------------------------------------------------------  7493
m150430_iid=3272125_0     ------------------------------------------------------------  10013
m141102_iid=754225_0      ------------------------------------------------------------  9952
m141107_iid=1409934_0     ------------------------------------------------------------  10262
m150503_iid=3416920_0     ------------------------------------------------------------  10188
m141108_iid=1800121_0     ------------------------------------------------------------  10617
m141031_iid=147540_0      ------------------------------------------------------------  11780
m150507_iid=3674924_0     ------------------------------------------------------------  10758
m150503_iid=2923751_0     ------------------------------------------------------------  10134
m141031_iid=115118_0      ------------------------------------------------------------  7981
m150430_iid=2722448_0     ------------------------------------------------------------  9403
m150507_iid=3102647_0     TCAAGCTCCAGTTTGATATATCCAGACACACTTTCCAGTACGCCTACTATGTTACGACTT  9116
m141108_iid=1811866_0     ------------------------------------------------------------  9038
m150501_iid=2948495_0     TCAAGCTCCAGTTTGATATATCCA------------------------------------  10373
m150504_iid=3238195_0     ------------------------------------------------------------  10610
m150503_iid=3388511_0     TCAAGCTCCAGTTTGATATATCCAGACACACTTTCCAGTACGCCTACTATGTTACGACTT  10455
m150505_iid=3777611_0     TCAAGCTCCA--------------------------------------------------  11830
m150430_iid=2438833_0     TCAAGCTCCAGTTTGATATATCCAGACACACTTTCCAGTA-GCCTACTATGTTACGACTT  12210
m141108_iid=1698969_0     ------------------------------------------------------------  11147
m150430_iid=3059190_0     TCAAGCTCCAGTTTGATATATCCAGACACACTTTCCAGTACGCCTACTATGTTACGACTT  9065
m141107_iid=1383067_0     ------------------------------------------------------------  7061
m141101_iid=597008_0      TCAAGCTCCAGTTTGATATATCCAGACACACTTTCCAGTACGCCTACTATGTTACGACTT  6146
m150506_iid=2596496_0     ------------------------------------------------------------  10491
m141102_iid=910051_0      ------------------------------------------------------------  7428
m150505_iid=3784017_0     TCAAGCTCCAGTTTGATATATCCAGACACACTTTCCAGTACGCCTACTATGTTACGACTT  11994
m141101_iid=682151_0      TCAAGCTCCAGTTTGATATATCCAGACACACTTTCCAGTACGCCTACTATGTTACGACTT  11066
m150501_iid=3748029_0     TCAAGCTCCAGTTTGATATATCCAGACACACTTTCCAGTACGCCTACTATGTTACGACTT  11614
m150504_iid=2876272_0     TCAAGCTCCAGTTTGATATATCCAGACACACTTTCCAGTACGCCTACTATGTTACGACTT  13464
NC_042820.1               TCAAGCTCCAGTTTGATATATCCAGACACACTTTCCAGTACGCCTACTATGTTACGACTT  13723
m141102_iid=1228825_0     ------------------------------------------------------------  11951
m150504_iid=2876274_0     TCAAGCTCCAGTTTGATATATCCAGACACACTTTCCAGTACGCCTACTATGTTACGACTT  13536
m141030_iid=80036_0       ------------------------------------------------------------  11554
m150506_iid=2596494_0     ------------------------------------------------------------  10983
m150503_iid=2526862_0     TCAAGCTCCAGTTTGATATATCC-GACACACTTTCCAGTACGCCTACTATGTTACGACTT  13381
m150506_iid=2567000_0     TCAAGCTCCAGTTTGATATATCCAGACACACTTTCCAGTACGCCTACTATGTTACGACTT  11350
```

```
m150507_iid=3073271_0      ------------------------------------------------------------  11626
m150504_iid=3156087_0      ------------------------------------------------------------  11376
m150505_iid=2498502_0      TCAAGCTCCAGTTTGATATATCCAGACACACTTTCCAGTACGCCTACTATGTTACGACTT  10123
m141102_iid=754227_0       ------------------------------------------------------------  10254
m150507_iid=3469514_0      ------------------------------------------------------------  10241
Rdo_MitoGenome             TCAAGCTCCAGTTTGATATATCCAGACACACTTTCCAGTACGCCTACTATGTTACGACTT  13724
m141101_iid=614933_0       ------------------------------------------------------------  9134


m141031_iid=347546_0       ------------------------------------------------------------  7511
m150506_iid=3127414_0      ATCT-AAATTAATTGAGAGTGACGGGCGATATGTACATATTTTAGAGCTTAATCATAAAA  10180
m141102_iid=1245517_0      ------------------------------------------------------------  7295
m141107_iid=1405727_0      ------------------------------------------------------------  7315
m141102_iid=987614_0       ------------------------------------------------------------  11885
m141108_iid=1701429_0      ATCTCAAATTAATTGAGAGTGACG-GCGATATGTACATATTTTAGAGCTTAATCATAAAA  8762
m141102_iid=987286_0       ------------------------------------------------------------  7493
m150430_iid=3272125_0      ------------------------------------------------------------  10013
m141102_iid=754225_0       ------------------------------------------------------------  9952
m141107_iid=1409934_0      ------------------------------------------------------------  10262
m150503_iid=3416920_0      ------------------------------------------------------------  10188
m141108_iid=1800121_0      ------------------------------------------------------------  10617
m141031_iid=147540_0       ------------------------------------------------------------  11780
m150507_iid=3674924_0      ------------------------------------------------------------  10758
m150503_iid=2923751_0      ------------------------------------------------------------  10134
m141031_iid=115118_0       ------------------------------------------------------------  7981
m150430_iid=2722448_0      ------------------------------------------------------------  9403
m150507_iid=3102647_0      ATCTCAAATTAATTGAGAGTGACGGGCGATATGTACATATTTTAGAGCTTAATCATAAAA  9176
m141108_iid=1811866_0      ------------------------------------------------------------  9038
m150501_iid=2948495_0      ------------------------------------------------------------  10373
m150504_iid=3238195_0      ------------------------------------------------------------  10610
m150503_iid=3388511_0      ATCTCAAATTAATTGAGAGTGACGGGCGATATGTACATATTTTAGAGCTTAATCATAAAA  10515
m150505_iid=3777611_0      ------------------------------------------------------------  11830
m150430_iid=2438833_0      ATCTCAAATTAATT----------------------------------------------  12224
m141108_iid=1698969_0      ------------------------------------------------------------  11147
m150430_iid=3059190_0      ATCTCAAATTAATTGAGAGTGACGGGCGATATGTACATATTTTAGAGCTTAATCATAAAA  9125
m141107_iid=1383067_0      ------------------------------------------------------------  7061
m141101_iid=597008_0       ATCTCAAATTAATTGAGAGTGACGGGCGATATGTACATATTTTAGAGCTTAATCATAAAA  6206
m150506_iid=2596496_0      ------------------------------------------------------------  10491
m141102_iid=910051_0       ------------------------------------------------------------  7428
m150505_iid=3784017_0      ATCTCAAATTAATTGAGAGTGACGGGCGATATGTACATATTTTAGAGCTTAATCATAAAA  12054
m141101_iid=682151_0       ATCTCAAATTAATTGAGAGTGACGGGCGATATGTACATATTTTAGAGCTTAATCATAAAA  11126
m150501_iid=3748029_0      ATCTCAAATTAATTGAGAGTGACGGGCGATATGTACATATTTTAGAGCTTAATCATAAAA  11674
m150504_iid=2876272_0      ATCTCAAATTAATTGAGAGTGACGGGCGATATGTACATATTTTAGAGCTTAATCATAAAA  13524
NC_042820.1                ATCTCAAATTAATTGAGAGTGACGGGCGATATGTACATATTTTAGAGCTTAATCATAAAA  13783
m141102_iid=1228825_0      ------------------------------------------------------------  11951
m150504_iid=2876274_0      ATCTCAAATTAATTGAGAGTGACGGGCGATATGTACATATTTTAGAGCTTAATCATAAAA  13596
m141030_iid=80036_0        ------------------------------------------------------------  11554
m150506_iid=2596494_0      ------------------------------------------------------------  10983
m150503_iid=2526862_0      ATCTCAAATTAATTGAGAGTGACGGGCGATATGTACATATTTTAGAGCTTAATCATAAAA  13441
m150506_iid=2567000_0      ATCTCAAATTAATTGAGAGTGACGGGCGATATGTACATATTTTAGAGCTTAATCATAAAA  11410
m150507_iid=3073271_0      ------------------------------------------------------------  11626
m150504_iid=3156087_0      ------------------------------------------------------------  11376
m150505_iid=2498502_0      ATCTCAAATTAATTG---------------------------------------------  10138
m141102_iid=754227_0       ------------------------------------------------------------  10254
m150507_iid=3469514_0      ------------------------------------------------------------  10241
Rdo_MitoGenome             ATCTCAAATTAATTGAGAGTGACGGGCGATATGTACATATTTTAGAGCTTAATCATAAAA  13784
m141101_iid=614933_0       ------------------------------------------------------------  9134


m141031_iid=347546_0       ------------------------------------------------------------  7511
m150506_iid=3127414_0      TCTAATAAAAATTATTACTTTCAAATCCACTTTATTAAAAATT------AAAAATTAAAC  10234
m141102_iid=1245517_0      ------------------------------------------------------------  7295
m141107_iid=1405727_0      ------------------------------------------------------------  7315
m141102_iid=987614_0       ------------------------------------------------------------  11885
m141108_iid=1701429_0      TCTAATAAAAATTATTACTTTCAAATCCACTTTATTAAA-AATATCTAAAA-ATTAAACC  8820
m141102_iid=987286_0       ------------------------------------------------------------  7493
m150430_iid=3272125_0      ------------------------------------------------------------  10013
m141102_iid=754225_0       ------------------------------------------------------------  9952
m141107_iid=1409934_0      ------------------------------------------------------------  10262
m150503_iid=3416920_0      ------------------------------------------------------------  10188
m141108_iid=1800121_0      ------------------------------------------------------------  10617
```

```
m141031_iid=147540_0        ------------------------------------------------------------  11780
m150507_iid=3674924_0       ------------------------------------------------------------  10758
m150503_iid=2923751_0       ------------------------------------------------------------  10134
m141031_iid=115118_0        ------------------------------------------------------------  7981
m150430_iid=2722448_0       ------------------------------------------------------------  9403
m150507_iid=3102647_0       TCTAATAAAAATTATTACTTTCAAATCCACTTTATT-AAAAATATCTAAAAAATTAAACC  9235
m141108_iid=1811866_0       ------------------------------------------------------------  9038
m150501_iid=2948495_0       ------------------------------------------------------------  10373
m150504_iid=3238195_0       ------------------------------------------------------------  10610
m150503_iid=3388511_0       TCTAATAAAAATTATTACTTTCAAATCCACTTT-ATTAAAAATATCTAAAAAATTAAACC  10574
m150505_iid=3777611_0       ------------------------------------------------------------  11830
m150430_iid=2438833_0       ------------------------------------------------------------  12224
m141108_iid=1698969_0       ------------------------------------------------------------  11147
m150430_iid=3059190_0       TCTAATAAAAATTATTACTTTCAAATCCACTTTATT-AAAAATATCTAAAAAATTAAACC  9184
m141107_iid=1383067_0       ------------------------------------------------------------  7061
m141101_iid=597008_0        TCTAATAAAAATTATTACTTTCAAATCCACTTTATT-AAAAATATCTAAAAAATTAAACC  6265
m150506_iid=2596496_0       ------------------------------------------------------------  10491
m141102_iid=910051_0        ------------------------------------------------------------  7428
m150505_iid=3784017_0       TCTAATAAAAATTATTACTTTCAAATCCACTT--TATTAAAATATCTAAAAAATTAAACC  12112
m141101_iid=682151_0        TCTAATAAAAATTATTACTTTCAAATCCACTTT-ATTAAAAATATCTAAAAAATTAAACC  11185
m150501_iid=3748029_0       TCTAATAAAAATTATTACTTTCAAATCCACTTT-ATTAAAAATATCTAAAAAATTAAACC  11733
m150504_iid=2876272_0       TCTAATAAAAATTATTACTTTCAAATCCACTTT-ATTAAAAATCTCTAAAAAATTAAACC  13583
NC_042820.1                 TCTAATAAAAATTATTACTTTCAAATCCACTTT-ATTAAAAATATCTAAAAAATTAAACC  13842
m141102_iid=1228825_0       ------------------------------------------------------------  11951
m150504_iid=2876274_0       TCTAATAAAAATTATTACTTTCAAATCCACTTT-ATTAAAAATATCTAAAAAATTAAACC  13655
m141030_iid=80036_0         ------------------------------------------------------------  11554
m150506_iid=2596494_0       ------------------------------------------------------------  10983
m150503_iid=2526862_0       TCTAATAAAAATTATTACTTTCAAATCCACTTTAATTAAAAATATCTAAAAAATTAAACC  13501
m150506_iid=2567000_0       TCTAATAAAAATTATTACTTTCAAATCCACTTT-ATTAAAAATATCTAAAAAATTAAACC  11469
m150507_iid=3073271_0       ------------------------------------------------------------  11626
m150504_iid=3156087_0       ------------------------------------------------------------  11376
m150505_iid=2498502_0       ------------------------------------------------------------  10138
m141102_iid=754227_0        ------------------------------------------------------------  10254
m150507_iid=3469514_0       ------------------------------------------------------------  10241
Rdo_MitoGenome              TCTAATAAAAATTATTACTTTCAAATCCACTTT-ATTAAAAATATCTAAAAAATTAAACC  13843
m141101_iid=614933_0        ------------------------------------------------------------  9134


m141031_iid=347546_0        ------------------------------------------------------------  7511
m150506_iid=3127414_0       CATATAAATAATTTATTGTAACCCACCTCAACATGAATATAAACTGCA-CTTGATCTGAT  10293
m141102_iid=1245517_0       ------------------------------------------------------------  7295
m141107_iid=1405727_0       ------------------------------------------------------------  7315
m141102_iid=987614_0        ------------------------------------------------------------  11885
m141108_iid=1701429_0       ATAT-AATAAATTTATTGTAACCCACCTCAACATGAATATAAACTGCACCTTGATCTGAT  8879
m141102_iid=987286_0        ------------------------------------------------------------  7493
m150430_iid=3272125_0       ------------------------------------------------------------  10013
m141102_iid=754225_0        ------------------------------------------------------------  9952
m141107_iid=1409934_0       ------------------------------------------------------------  10262
m150503_iid=3416920_0       ------------------------------------------------------------  10188
m141108_iid=1800121_0       ------------------------------------------------------------  10617
m141031_iid=147540_0        ------------------------------------------------------------  11780
m150507_iid=3674924_0       ------------------------------------------------------------  10758
m150503_iid=2923751_0       ------------------------------------------------------------  10134
m141031_iid=115118_0        ------------------------------------------------------------  7981
m150430_iid=2722448_0       ------------------------------------------------------------  9403
m150507_iid=3102647_0       ATATAAATAAATTTATTGTAACCCACCTCAACATGAATATAAACTGCACCTTGATCTGAT  9295
m141108_iid=1811866_0       ------------------------------------------------------------  9038
m150501_iid=2948495_0       ------------------------------------------------------------  10373
m150504_iid=3238195_0       ------------------------------------------------------------  10610
m150503_iid=3388511_0       ATATAAATAAATTTATTGTAACCCACCTCAACATGAATATAAACTGCACCTTGATCTGAT  10634
m150505_iid=3777611_0       ------------------------------------------------------------  11830
m150430_iid=2438833_0       ------------------------------------------------------------  12224
m141108_iid=1698969_0       ------------------------------------------------------------  11147
m150430_iid=3059190_0       ATATAAATAAATTTATTGTAACCCACCTCAACATGAATATAAACTGCACCTTGATCTGAT  9244
m141107_iid=1383067_0       ------------------------------------------------------------  7061
m141101_iid=597008_0        ATATAAATAAATTTATTGTAACCCACCTCAACATGAATATAAACTGCACCTTGATCTGAT  6325
m150506_iid=2596496_0       ------------------------------------------------------------  10491
m141102_iid=910051_0        ------------------------------------------------------------  7428
m150505_iid=3784017_0       ATATAAATAAATTTATTGTAACCCACCTCAACATGAATATAAACTGCACCTTGATCTGAT  12172
m141101_iid=682151_0        ATATAAATAAATTTATTGTAACCCACCTCAACATGAATATAAACTGCACCTTGATCTGAT  11245
m150501_iid=3748029_0       ATATAAATAAATTTATTGTAACCCACCTCAACATGAATATAAACTGCACCTTGATCTGAT  11793
```

```
m150504_iid=2876272_0       ATATAAATAAATTTATTGTAACCCACCTCAACATGAATATAAACTGCACCTTGATCTGAT   13643
NC_042820.1                 ATATAAATAAATTTATTGTAACCCACCTCAACATGAATATAAACTGCACCTTGATCTGAT   13902
m141102_iid=1228825_0       ------------------------------------------------------------   11951
m150504_iid=2876274_0       ATATAAATAAATTTATTGTAA-CCACCTCAACATGAATATAAACTGCACCTTGATCTGAT   13714
m141030_iid=80036_0         ------------------------------------------------------------   11554
m150506_iid=2596494_0       ------------------------------------------------------------   10983
m150503_iid=2526862_0       ATATAAATAAATTTATTGTAA-CCACCTCAACATGAATATAAACTG--CCTTGATCTGAT   13558
m150506_iid=2567000_0       ATATAAATAAATTTATTGTAACCCACCTCAACATGAATATAAACTGCACCTTGATCTGAT   11529
m150507_iid=3073271_0       ------------------------------------------------------------   11626
m150504_iid=3156087_0       ------------------------------------------------------------   11376
m150505_iid=2498502_0       ------------------------------------------------------------   10138
m141102_iid=754227_0        ------------------------------------------------------------   10254
m150507_iid=3469514_0       ------------------------------------------------------------   10241
Rdo_MitoGenome              ATATAAATAAATTTATTGTAACCCACCTCAACATGAATATAAACTGCACCTTGATCTGAT   13903
m141101_iid=614933_0        ------------------------------------------------------------   9134


m141031_iid=347546_0        ------------------------------------------------------------   7511
m150506_iid=3127414_0       GTTAACTTTAATATAGAAATAATGATCATTTAATCCTTAAAAA--TACCAACTACGACGA   10351
m141102_iid=1245517_0       ------------------------------------------------------------   7295
m141107_iid=1405727_0       ------------------------------------------------------------   7315
m141102_iid=987614_0        ------------------------------------------------------------   11885
m141108_iid=1701429_0       GTTAACTTTAATATA-AGAAAATGATCATTTAATCCTTAA-AAATAACCAACTACGACGA   8937
m141102_iid=987286_0        ------------------------------------------------------------   7493
m150430_iid=3272125_0       ------------------------------------------------------------   10013
m141102_iid=754225_0        ------------------------------------------------------------   9952
m141107_iid=1409934_0       ------------------------------------------------------------   10262
m150503_iid=3416920_0       ------------------------------------------------------------   10188
m141108_iid=1800121_0       ------------------------------------------------------------   10617
m141031_iid=147540_0        ------------------------------------------------------------   11780
m150507_iid=3674924_0       ------------------------------------------------------------   10758
m150503_iid=2923751_0       ------------------------------------------------------------   10134
m141031_iid=115118_0        ------------------------------------------------------------   7981
m150430_iid=2722448_0       ------------------------------------------------------------   9403
m150507_iid=3102647_0       GTTAACTTTAATATA-AGAAAATGATCATTTAATCCTTAAAAAATAACCAACTACGACGA   9354
m141108_iid=1811866_0       ------------------------------------------------------------   9038
m150501_iid=2948495_0       ------------------------------------------------------------   10373
m150504_iid=3238195_0       ------------------------------------------------------------   10610
m150503_iid=3388511_0       GTTAACTTTAATATA-AGAAAATGATCATTTAATCCTTAAAAAATAACCAACTACGACGA   10693
m150505_iid=3777611_0       ------------------------------------------------------------   11830
m150430_iid=2438833_0       ------------------------------------------------------------   12224
m141108_iid=1698969_0       ------------------------------------------------------------   11147
m150430_iid=3059190_0       GTTAACTTTAATATA-AGAAAATGATCATTTAATCCTTAAAAAATAACCAACTACGACGA   9303
m141107_iid=1383067_0       ------------------------------------------------------------   7061
m141101_iid=597008_0        GTTAACTTTAATATA-AGAAAATGATCATTTAATCCTTAAAAAATAACCAACTACGACGA   6384
m150506_iid=2596496_0       ------------------------------------------------------------   10491
m141102_iid=910051_0        ------------------------------------------------------------   7428
m150505_iid=3784017_0       GTTAACTTTAATATA-AGAAAATGATCATTTAATCCTTAAAAAATAACCAACTACGACGA   12231
m141101_iid=682151_0        GTTAACTTTAATATA-AGAAAATGATCATTTAATCCTTAAAAAATAACCAACTACGACGA   11304
m150501_iid=3748029_0       GTTAACTTTAATATA-AGAAAATGATCATTTAATCCTTAAAAAATAACCAACTACGACGA   11852
m150504_iid=2876272_0       GTTAACTTTAATATA-AGAAAATGATCATTTAATCCTTAAAAAATAACCAACTACGACGA   13702
NC_042820.1                 GTTAACTTTAATATA-AGAAAATGATCATTTAATCCTTAAAAAATAACCAACTACGACGA   13961
m141102_iid=1228825_0       ------------------------------------------------------------   11951
m150504_iid=2876274_0       GTTAACTTTAATATA-AG-AAATGATCATTTAATCCTTAAAAAATAACCAACTACGACGA   13772
m141030_iid=80036_0         ------------------------------------------------------------   11554
m150506_iid=2596494_0       ------------------------------------------------------------   10983
m150503_iid=2526862_0       GTTAACTTTAATATA-AGAAAATGATCATTTAATCCTTAAAAAATAACCAACTACGACGA   13617
m150506_iid=2567000_0       GTTAACTTTAATATA-AG-AAATGATCATTTAATCCTTAAAAAATAACCAACTACGACGA   11587
m150507_iid=3073271_0       ------------------------------------------------------------   11626
m150504_iid=3156087_0       ------------------------------------------------------------   11376
m150505_iid=2498502_0       ------------------------------------------------------------   10138
m141102_iid=754227_0        ------------------------------------------------------------   10254
m150507_iid=3469514_0       ------------------------------------------------------------   10241
Rdo_MitoGenome              GTTAACTTTAATATA-AGAAAATGATCATTTAATCCTTAAAAAATAACCAACTACGACGA   13962
m141101_iid=614933_0        ------------------------------------------------------------   9134


m141031_iid=347546_0        ------------------------------------------------------------   7511
m150506_iid=3127414_0       TATACAAATT--TTAAATCAAGTAACTAAATCGTGGATTATCGTTTACAGGACAGGTTTC   10409
m141102_iid=1245517_0       ------------------------------------------------------------   7295
m141107_iid=1405727_0       ------------------------------------------------------------   7315
```

```
m141102_iid=987614_0       ------------------------------------------------------------  11885
m141108_iid=1701429_0      TATACAAATTT-TTAAATCAAGTAACTAAATCG---------------------------  8969
m141102_iid=987286_0       ------------------------------------------------------------  7493
m150430_iid=3272125_0      ------------------------------------------------------------  10013
m141102_iid=754225_0       ------------------------------------------------------------  9952
m141107_iid=1409934_0      ------------------------------------------------------------  10262
m150503_iid=3416920_0      ------------------------------------------------------------  10188
m141108_iid=1800121_0      ------------------------------------------------------------  10617
m141031_iid=147540_0       ------------------------------------------------------------  11780
m150507_iid=3674924_0      ------------------------------------------------------------  10758
m150503_iid=2923751_0      ------------------------------------------------------------  10134
m141031_iid=115118_0       ------------------------------------------------------------  7981
m150430_iid=2722448_0      ------------------------------------------------------------  9403
m150507_iid=3102647_0      TATACAAATTT-TTAAATCAAGTAACTAAATCGTGGATTATCGTTTACAGGACAGGTTCC  9413
m141108_iid=1811866_0      ------------------------------------------------------------  9038
m150501_iid=2948495_0      ------------------------------------------------------------  10373
m150504_iid=3238195_0      ------------------------------------------------------------  10610
m150503_iid=3388511_0      TATACAAATTT-TTAAATCAAGTAACTAAATCGTGGATTATCGTTTACAGGACAGGTTCC  10752
m150505_iid=3777611_0      ------------------------------------------------------------  11830
m150430_iid=2438833_0      ------------------------------------------------------------  12224
m141108_iid=1698969_0      ------------------------------------------------------------  11147
m150430_iid=3059190_0      TATACAAATTTTTTAAATCAAGTAACTAAATCGTGGATTATCGTTTACAGGACAGGTTCC  9363
m141107_iid=1383067_0      ------------------------------------------------------------  7061
m141101_iid=597008_0       TATACAAATTT-TTAAATCAAGTAACTAAATCGTGGATTATCGTTTACAGGACAGGTTCC  6443
m150506_iid=2596496_0      ------------------------------------------------------------  10491
m141102_iid=910051_0       ------------------------------------------------------------  7428
m150505_iid=3784017_0      TATACAAATTT-TTAAATCAAGTAACTAAATCGT-GATTATCGTTTACAGGACAGGTTCC  12289
m141101_iid=682151_0       TATACAAATTT-TTAAATCAAGTAACTAAATCGTGGATTATCGTTTACAGGACAGGTTCC  11363
m150501_iid=3748029_0      TATACAAATTT-TTAAATCAAGTAACTAAATCGTGGATTATCGTTTACAGGACAGGTTCC  11911
m150504_iid=2876272_0      TATACAAATTT-TTAAATCAAGTAACTAAATCGTGGATTATCGTTTACAGGACAGGTTCC  13761
NC_042820.1                TATACAAATTT-TTAAATCAAGTAACTAAATCGTGGATTATCGTTTACAGGACAGGTTCC  14020
m141102_iid=1228825_0      ------------------------------------------------------------  11951
m150504_iid=2876274_0      TATACAAATTT-TTAAATCAAGTAACTAAATCGTGGATTATCGTTTACAGGACAGGTTCC  13831
m141030_iid=80036_0        ------------------------------------------------------------  11554
m150506_iid=2596494_0      ------------------------------------------------------------  10983
m150503_iid=2526862_0      TATACAAATTT-TTAAATCAAGTAACT-AATCGTGGATTATCGTTTACAGGACAGGTT--  13673
m150506_iid=2567000_0      TATACAAA-TT-TTAAATCAAGTAACTAAATCGTGGATTATCGTTTACAGGACAGGTTCC  11645
m150507_iid=3073271_0      ------------------------------------------------------------  11626
m150504_iid=3156087_0      ------------------------------------------------------------  11376
m150505_iid=2498502_0      ------------------------------------------------------------  10138
m141102_iid=754227_0       ------------------------------------------------------------  10254
m150507_iid=3469514_0      ------------------------------------------------------------  10241
Rdo_MitoGenome             TATACAAATTT-TTAAATCAAGTAACTAAATCGTGGATTATCGTTTACAGGACAGGTTCC  14021
m141101_iid=614933_0       ------------------------------------------------------------  9134


m141031_iid=347546_0       ------------------------------------------------------------  7511
m150506_iid=3127414_0      TCTG-AAAGACTAAAATACCGCCAAATCCTTTTACTTTCAAGAACTTAACTAATACTAAT  10468
m141102_iid=1245517_0      ------------------------------------------------------------  7295
m141107_iid=1405727_0      ------------------------------------------------------------  7315
m141102_iid=987614_0       ------------------------------------------------------------  11885
m141108_iid=1701429_0      ------------------------------------------------------------  8969
m141102_iid=987286_0       ------------------------------------------------------------  7493
m150430_iid=3272125_0      ------------------------------------------------------------  10013
m141102_iid=754225_0       ------------------------------------------------------------  9952
m141107_iid=1409934_0      ------------------------------------------------------------  10262
m150503_iid=3416920_0      ------------------------------------------------------------  10188
m141108_iid=1800121_0      ------------------------------------------------------------  10617
m141031_iid=147540_0       ------------------------------------------------------------  11780
m150507_iid=3674924_0      ------------------------------------------------------------  10758
m150503_iid=2923751_0      ------------------------------------------------------------  10134
m141031_iid=115118_0       ------------------------------------------------------------  7981
m150430_iid=2722448_0      ------------------------------------------------------------  9403
m150507_iid=3102647_0      TCTGAAAAGACTAAAATACCGCCAAATCCTTTTACTTTCAAGAACTTAACTAATACTAAT  9473
m141108_iid=1811866_0      ------------------------------------------------------------  9038
m150501_iid=2948495_0      ------------------------------------------------------------  10373
m150504_iid=3238195_0      ------------------------------------------------------------  10610
m150503_iid=3388511_0      TCTGAAAAGACTAAAATACCGCCAAATCCTTTTACTTTCAAGAACTTAACTAATACTAAT  10812
m150505_iid=3777611_0      ------------------------------------------------------------  11830
m150430_iid=2438833_0      ------------------------------------------------------------  12224
m141108_iid=1698969_0      ------------------------------------------------------------  11147
```

```
m150430_iid=3059190_0       TCTGAAAAGACTAAAATACCGCCAAATCCTTTTACTTTCAAGAACTTAACTAATACTAAT   9423
m141107_iid=1383067_0       ------------------------------------------------------------   7061
m141101_iid=597008_0        TCTGAAAAGACTAAAATACCGCCAAATCCTTTTACTTTCAAGAACTTAACTAATACTAAT   6503
m150506_iid=2596496_0       ------------------------------------------------------------   10491
m141102_iid=910051_0        ------------------------------------------------------------   7428
m150505_iid=3784017_0       TCTGAAAAGACTAAAATACCGCCAAATCCTTTTACTTTCAAGAACTTAACTAATACTAAT   12349
m141101_iid=682151_0        TCTGAAAAGACTAAAATACCGCCAAATCCTTTTACTTTCAAGAACTTAACTAATACTAAT   11423
m150501_iid=3748029_0       TCTGAAAAGACTAAAATACCGCCAAATCCTTTTACTTTCAAGAACTTAACTAATACTAAT   11971
m150504_iid=2876272_0       TCTGAAAAGACTAAAATACCGCCAAATCCTTTTACTTTCAAGAACTTAACTAATACTAAT   13821
NC_042820.1                 TCTGAAAAGACTAAAATACCGCCAAATCCTTTTACTTTCAAGAACTTAACTAATACTAAT   14080
m141102_iid=1228825_0       ------------------------------------------------------------   11951
m150504_iid=2876274_0       TCTGAAAAGACT-AAATACCGCCAAAT-CTTTACTTTCAAGAACTTAACTAATACTAAT   13889
m141030_iid=80036_0         ------------------------------------------------------------   11554
m150506_iid=2596494_0       ------------------------------------------------------------   10983
m150503_iid=2526862_0       TCTGAAAAGACTAAAATACCGCCAAATCCTTTTACTTT-AAGAACTTAACTAATACTAAT   13732
m150506_iid=2567000_0       TCTGAAAAGACTAAAATACCGCCAAATCCTTTTACTTTCAAGAACTTAACTAATACTAAT   11705
m150507_iid=3073271_0       ------------------------------------------------------------   11626
m150504_iid=3156087_0       ------------------------------------------------------------   11376
m150505_iid=2498502_0       ------------------------------------------------------------   10138
m141102_iid=754227_0        ------------------------------------------------------------   10254
m150507_iid=3469514_0       ------------------------------------------------------------   10241
Rdo_MitoGenome              TCTGAAAAGACTAAAATACCGCCAAATCCTTTTACTTTCAAGAACTTAACTAATACTAAT   14081
m141101_iid=614933_0        ------------------------------------------------------------   9134


m141031_iid=347546_0        ------------------------------------------------------------   7511
m150506_iid=3127414_0       -AAAATTTTACACTCAGAATAACGGGGTATCT-ATCCCGATTTCAAAAACAGAATTTAAA   10526
m141102_iid=1245517_0       ------------------------------------------------------------   7295
m141107_iid=1405727_0       ------------------------------------------------------------   7315
m141102_iid=987614_0        ------------------------------------------------------------   11885
m141108_iid=1701429_0       ------------------------------------------------------------   8969
m141102_iid=987286_0        ------------------------------------------------------------   7493
m150430_iid=3272125_0       ------------------------------------------------------------   10013
m141102_iid=754225_0        ------------------------------------------------------------   9952
m141107_iid=1409934_0       ------------------------------------------------------------   10262
m150503_iid=3416920_0       ------------------------------------------------------------   10188
m141108_iid=1800121_0       ------------------------------------------------------------   10617
m141031_iid=147540_0        ------------------------------------------------------------   11780
m150507_iid=3674924_0       ------------------------------------------------------------   10758
m150503_iid=2923751_0       ------------------------------------------------------------   10134
m141031_iid=115118_0        ------------------------------------------------------------   7981
m150430_iid=2722448_0       ------------------------------------------------------------   9403
m150507_iid=3102647_0       AA-AAATTTACACTCAGAATAACGG-GTATCTAATCCCGATTTCAAAAACAGAATTTAAT   9531
m141108_iid=1811866_0       ------------------------------------------------------------   9038
m150501_iid=2948495_0       ------------------------------------------------------------   10373
m150504_iid=3238195_0       ------------------------------------------------------------   10610
m150503_iid=3388511_0       AAAAAATTTACACTCAGAATAACGGGGTATCTAATCCCGATTTCAAAAACAGAATTTAAT   10872
m150505_iid=3777611_0       ------------------------------------------------------------   11830
m150430_iid=2438833_0       ------------------------------------------------------------   12224
m141108_iid=1698969_0       ------------------------------------------------------------   11147
m150430_iid=3059190_0       AA-AAATTTACACTCAGAATAACGGGGTATCTAATCCCGATTTCAAAAACAGAATTTAAT   9482
m141107_iid=1383067_0       ------------------------------------------------------------   7061
m141101_iid=597008_0        AAAAAATTTACACTCAGAATAACGGGGTATCTAATCCCGATTTCAAAAACAGAATTTAAT   6563
m150506_iid=2596496_0       ------------------------------------------------------------   10491
m141102_iid=910051_0        ------------------------------------------------------------   7428
m150505_iid=3784017_0       -AAAAATTTACACTCAGAATAACGGGGTATCTAATCCCGATTTCAAAAACAGAATTTAAT   12408
m141101_iid=682151_0        AAAAAATTTACACTCAGAATAACGGGGTATCTAATCCCGATTTCAAAAACAGAATTTAAT   11483
m150501_iid=3748029_0       -AAAAATTTACACTCAGAATAACGGGGTATCTAATCCCGATTTCAAAAACAGAATTTAAT   12030
m150504_iid=2876272_0       -AAAAATTTACACTCAGAATAACGGGGTATCTAATCCCGATTTCAAAAACAGAATTTAAT   13880
NC_042820.1                 AAAAAATTTACACTCAGAATAACGGGGTATCTAATCCCGATTTCAAAAACAGAATTTAAT   14140
m141102_iid=1228825_0       ------------------------------------------------------------   11951
m150504_iid=2876274_0       -AAAAATTTACACTCAGAATAACGGGGTATCTAATCCCGATTTCAAAAACAGAATTTAAT   13948
m141030_iid=80036_0         ------------------------------------------------------------   11554
m150506_iid=2596494_0       ------------------------------------------------------------   10983
m150503_iid=2526862_0       -AAAAATTTACACTCAGAATAACGGGGTATCTAATCCCGATTTCAAAAACAGAATTT-AT   13790
m150506_iid=2567000_0       AAAAAATTTACACTCAGAATAACGGGGTATCTAATCCCGATTTCAAAAACAGAATTTAAT   11765
m150507_iid=3073271_0       ------------------------------------------------------------   11626
m150504_iid=3156087_0       ------------------------------------------------------------   11376
m150505_iid=2498502_0       ------------------------------------------------------------   10138
m141102_iid=754227_0        ------------------------------------------------------------   10254
m150507_iid=3469514_0       ------------------------------------------------------------   10241
```

```
Rdo_MitoGenome            AAAAAATTTACACTCAGAATAACGGGGTATCTAATCCCGATTTCAAAAACAGAATTTAAT   14141
m141101_iid=614933_0      ------------------------------------------------------------   9134


m141031_iid=347546_0      ------------------------------------------------------------   7511
m150506_iid=3127414_0     TAAAACAGAAAATAAAAAAAAATTTAATAAAATTTCAC-CTAATATTAAAATTTAA---T   10582
m141102_iid=1245517_0     ------------------------------------------------------------   7295
m141107_iid=1405727_0     ------------------------------------------------------------   7315
m141102_iid=987614_0      ------------------------------------------------------------   11885
m141108_iid=1701429_0     ------------------------------------------------------------   8969
m141102_iid=987286_0      ------------------------------------------------------------   7493
m150430_iid=3272125_0     ------------------------------------------------------------   10013
m141102_iid=754225_0      ------------------------------------------------------------   9952
m141107_iid=1409934_0     ------------------------------------------------------------   10262
m150503_iid=3416920_0     ------------------------------------------------------------   10188
m141108_iid=1800121_0     ------------------------------------------------------------   10617
m141031_iid=147540_0      ------------------------------------------------------------   11780
m150507_iid=3674924_0     ------------------------------------------------------------   10758
m150503_iid=2923751_0     ------------------------------------------------------------   10134
m141031_iid=115118_0      ------------------------------------------------------------   7981
m150430_iid=2722448_0     ------------------------------------------------------------   9403
m150507_iid=3102647_0     AAAACAGAAAATAAAAAATAAATTTAATAAAATTTCAC-CTAATATTAAAAATTTAATCT   9590
m141108_iid=1811866_0     ------------------------------------------------------------   9038
m150501_iid=2948495_0     ------------------------------------------------------------   10373
m150504_iid=3238195_0     ------------------------------------------------------------   10610
m150503_iid=3388511_0     AAAACAGAAAATAAAAAATAAATTTAATAAAATTTCAC-CTAATATTAAAAATTTAATCT   10931
m150505_iid=3777611_0     ------------------------------------------------------------   11830
m150430_iid=2438833_0     ------------------------------------------------------------   12224
m141108_iid=1698969_0     ------------------------------------------------------------   11147
m150430_iid=3059190_0     AAAACAGAAAATAAAAAATAAATTTAATAAAATTTCACACTAATATTAAAAATTTAATCT   9542
m141107_iid=1383067_0     ------------------------------------------------------------   7061
m141101_iid=597008_0      AAAACAGAAAATAAAAAATAAATTTAATAAAATTTCAC-CTAATATTAAAAATTTAATCT   6622
m150506_iid=2596496_0     ------------------------------------------------------------   10491
m141102_iid=910051_0      ------------------------------------------------------------   7428
m150505_iid=3784017_0     AAAACAGAAAAT-AAAAATAAATTTAATAAAATTTCAC-CTAATATTAAAAATTTAATCT   12466
m141101_iid=682151_0      AAAACAGAAAATAAAAAATAAATTTAATAAAATTTCAC-CTAATATTAAAAATTTAATCT   11542
m150501_iid=3748029_0     AAAACAGAAAAT-AAAAATAAATTTAATAAAATTTCAC-CTAATATTAAAAATTTAATCT   12088
m150504_iid=2876272_0     AAAACAGAAAAT-AAAAATAAATTTAATAAAATTTCAC-CTAATATTAAAAATTTAATCT   13938
NC_042820.1               AAAACAGAAAATAAAAAATAAATTTAATAAAATTTCAC-CTAATATTAAAAATTTAATCT   14199
m141102_iid=1228825_0     ------------------------------------------------------------   11951
m150504_iid=2876274_0     AAAACAGAAAATAAAAA-------------------------------------------   13965
m141030_iid=80036_0       ------------------------------------------------------------   11554
m150506_iid=2596494_0     ------------------------------------------------------------   10983
m150503_iid=2526862_0     AAAACAGAAAAT-AAAAATAAATTTAATAAAATTTCAC-CTAATATTAAAAATTTAATCT   13848
m150506_iid=2567000_0     AAAACAGAAAATAAAAAATAAATTTAATAAAATTTCAC-CTAATATTAAAAATTTAATCT   11824
m150507_iid=3073271_0     ------------------------------------------------------------   11626
m150504_iid=3156087_0     ------------------------------------------------------------   11376
m150505_iid=2498502_0     ------------------------------------------------------------   10138
m141102_iid=754227_0      ------------------------------------------------------------   10254
m150507_iid=3469514_0     ------------------------------------------------------------   10241
Rdo_MitoGenome            AAAACAGAAAATAAAAAATAAATTTAATAAAATTTCAC-CTAATATTAAAAATTTAATCT   14200
m141101_iid=614933_0      ------------------------------------------------------------   9134


m141031_iid=347546_0      ------------------------------------------------------------   7511
m150506_iid=3127414_0     TTAATTTAATCCTAATTACCATTAAA--TAAATTTTATTTGCACTAATTGTATA-AACCG   10639
m141102_iid=1245517_0     ------------------------------------------------------------   7295
m141107_iid=1405727_0     ------------------------------------------------------------   7315
m141102_iid=987614_0      ------------------------------------------------------------   11885
m141108_iid=1701429_0     ------------------------------------------------------------   8969
m141102_iid=987286_0      ------------------------------------------------------------   7493
m150430_iid=3272125_0     ------------------------------------------------------------   10013
m141102_iid=754225_0      ------------------------------------------------------------   9952
m141107_iid=1409934_0     ------------------------------------------------------------   10262
m150503_iid=3416920_0     ------------------------------------------------------------   10188
m141108_iid=1800121_0     ------------------------------------------------------------   10617
m141031_iid=147540_0      ------------------------------------------------------------   11780
m150507_iid=3674924_0     ------------------------------------------------------------   10758
m150503_iid=2923751_0     ------------------------------------------------------------   10134
m141031_iid=115118_0      ------------------------------------------------------------   7981
m150430_iid=2722448_0     ------------------------------------------------------------   9403
```

```
m150507_iid=3102647_0      TAATTTTAATCCTAATTACCATTAAA--TAAATTTATATTGCACTAATTGTA--TAACCG   9646
m141108_iid=1811866_0      ------------------------------------------------------------   9038
m150501_iid=2948495_0      ------------------------------------------------------------   10373
m150504_iid=3238195_0      ------------------------------------------------------------   10610
m150503_iid=3388511_0      TAATTTTAATCCTAATTACCATTAAA--TAAATTTATATTGCACTAATTGTAT--AACCG   10987
m150505_iid=3777611_0      ------------------------------------------------------------   11830
m150430_iid=2438833_0      ------------------------------------------------------------   12224
m141108_iid=1698969_0      ------------------------------------------------------------   11147
m150430_iid=3059190_0      TAATTTTAATCCTAATTACCATTAAAAATAAATTTATATTGCACTAATTGTA--TAACCG   9600
m141107_iid=1383067_0      ------------------------------------------------------------   7061
m141101_iid=597008_0       TAATTTTAATCCTAATTACCATTAA-A-TAAATTTATATTGCACTAATTGTA--TAACCG   6678
m150506_iid=2596496_0      ------------------------------------------------------------   10491
m141102_iid=910051_0       ------------------------------------------------------------   7428
m150505_iid=3784017_0      TAATTTTA----------------------------------------------------   12474
m141101_iid=682151_0       TAATTTTAATCCTAATTACCA-TTAAA-TAAATTTATATTGCACTAATTGTAT--AACCG   11598
m150501_iid=3748029_0      TAATTTTAATCCTAATTACCA-TTAAA-TAAATTTATATTGCACTAATTGTAT--AACCG   12144
m150504_iid=2876272_0      TAATTTTAATCCTAATTACCATTTAAA-TAAATTTATATTGCACTAATTGTAT--AACCG   13995
NC_042820.1                TAATTTTAATCCTAATTACCA-TTAAA-TAAATTTATATTGCACTAATTGTAT--AACCG   14255
m141102_iid=1228825_0      ------------------------------------------------------------   11951
m150504_iid=2876274_0      ------------------------------------------------------------   13965
m141030_iid=80036_0        ------------------------------------------------------------   11554
m150506_iid=2596494_0      ------------------------------------------------------------   10983
m150503_iid=2526862_0      TAATTTTAATCCTAATTACCA-TTAAA-TAAA-TTATATTGCACTAATTGTAT--AACCG   13903
m150506_iid=2567000_0      TAATTTTAATCCT-ATTACCA-TTAAA-TAAATTTATATTGCACTAATTGTATTAAACCG   11881
m150507_iid=3073271_0      ------------------------------------------------------------   11626
m150504_iid=3156087_0      ------------------------------------------------------------   11376
m150505_iid=2498502_0      ------------------------------------------------------------   10138
m141102_iid=754227_0       ------------------------------------------------------------   10254
m150507_iid=3469514_0      ------------------------------------------------------------   10241
Rdo_MitoGenome             TAATTTTAATCCTAATTACCA-TTAAA-TAAATTTATATTGCACTAATTGTAT--AACCG   14256
m141101_iid=614933_0       ------------------------------------------------------------   9134


m141031_iid=347546_0       ------------------------------------------------------------   7511
m150506_iid=3127414_0      CAACTGCTGG-CAACAAATTAGTTAGAACT--AAAAATTTCTGAATCTAAGTAA---TTA   10693
m141102_iid=1245517_0      ------------------------------------------------------------   7295
m141107_iid=1405727_0      ------------------------------------------------------------   7315
m141102_iid=987614_0       ------------------------------------------------------------   11885
m141108_iid=1701429_0      ------------------------------------------------------------   8969
m141102_iid=987286_0       ------------------------------------------------------------   7493
m150430_iid=3272125_0      ------------------------------------------------------------   10013
m141102_iid=754225_0       ------------------------------------------------------------   9952
m141107_iid=1409934_0      ------------------------------------------------------------   10262
m150503_iid=3416920_0      ------------------------------------------------------------   10188
m141108_iid=1800121_0      ------------------------------------------------------------   10617
m141031_iid=147540_0       ------------------------------------------------------------   11780
m150507_iid=3674924_0      ------------------------------------------------------------   10758
m150503_iid=2923751_0      ------------------------------------------------------------   10134
m141031_iid=115118_0       ------------------------------------------------------------   7981
m150430_iid=2722448_0      ------------------------------------------------------------   9403
m150507_iid=3102647_0      CAACTGCTGG-CACAAAATTAGTTAGAACTAAAAAAATTTCTGATTCTAAGTAACCTTAA   9705
m141108_iid=1811866_0      ------------------------------------------------------------   9038
m150501_iid=2948495_0      ------------------------------------------------------------   10373
m150504_iid=3238195_0      ------------------------------------------------------------   10610
m150503_iid=3388511_0      CAACTGCTGG-CACAAAATTAGTTAGAACT-AAAAAATTTCTGATTCTAAGTAACCTTAA   11045
m150505_iid=3777611_0      ------------------------------------------------------------   11830
m150430_iid=2438833_0      ------------------------------------------------------------   12224
m141108_iid=1698969_0      ------------------------------------------------------------   11147
m150430_iid=3059190_0      CAACTGCTGG-CACAAAATTAGTTAGAACTAAAAAAATTTCTGATTCTAAGTAACCTTAA   9659
m141107_iid=1383067_0      ------------------------------------------------------------   7061
m141101_iid=597008_0       CAACTGCTGG-CACAAAATTAGTTAGAACTAAAAAAATTTCTGATTCTAAGTAACCTTAA   6737
m150506_iid=2596496_0      ------------------------------------------------------------   10491
m141102_iid=910051_0       ------------------------------------------------------------   7428
m150505_iid=3784017_0      ------------------------------------------------------------   12474
m141101_iid=682151_0       CAACTGCTGG-CACAAAATTAGTTAGAACTAAAAAAATTTCTGATTCTAAGTAACCTTAA   11657
m150501_iid=3748029_0      CAACTGCTGG-CACAAAATTAGTTAGAACTAAAAAAATTTCTGATTCTAAGTAACCTTAA   12203
m150504_iid=2876272_0      CAACTGCTGG-CACAAAATTAGTTAGAACTAAAAAAATTTCTG-----------------   14037
NC_042820.1                CAACTGCTGG-CACAAAATTAGTTAGAACTAAAAAAATTTCTGATTCTAAGTAACCTTAA   14314
m141102_iid=1228825_0      ------------------------------------------------------------   11951
m150504_iid=2876274_0      ------------------------------------------------------------   13965
m141030_iid=80036_0        ------------------------------------------------------------   11554
```

```
m150506_iid=2596494_0      ------------------------------------------------------------   10983
m150503_iid=2526862_0      CAACTGCTGG-CACAAAATTAGTTAGAACTAAAA--------------------------   13936
m150506_iid=2567000_0      CAACTGCTGGCCACAAAATTAGTTAGAA--------------------------------   11909
m150507_iid=3073271_0      ------------------------------------------------------------   11626
m150504_iid=3156087_0      ------------------------------------------------------------   11376
m150505_iid=2498502_0      ------------------------------------------------------------   10138
m141102_iid=754227_0       ------------------------------------------------------------   10254
m150507_iid=3469514_0      ------------------------------------------------------------   10241
Rdo_MitoGenome             CAACTGCTGG-CACAAAATTAGTTAGAACTAAAAAAATTTCTGATTCTAAGTAACCTTAA   14315
m141101_iid=614933_0       ------------------------------------------------------------   9134


m141031_iid=347546_0       ------------------------------------------------------------   7511
m150506_iid=3127414_0      ATAACAAATATAAATACTGCAAAAAGAAAACTCTAAAATA--CATTACATATATTTAAAT   10751
m141102_iid=1245517_0      ------------------------------------------------------------   7295
m141107_iid=1405727_0      ------------------------------------------------------------   7315
m141102_iid=987614_0       ------------------------------------------------------------   11885
m141108_iid=1701429_0      ------------------------------------------------------------   8969
m141102_iid=987286_0       ------------------------------------------------------------   7493
m150430_iid=3272125_0      ------------------------------------------------------------   10013
m141102_iid=754225_0       ------------------------------------------------------------   9952
m141107_iid=1409934_0      ------------------------------------------------------------   10262
m150503_iid=3416920_0      ------------------------------------------------------------   10188
m141108_iid=1800121_0      ------------------------------------------------------------   10617
m141031_iid=147540_0       ------------------------------------------------------------   11780
m150507_iid=3674924_0      ------------------------------------------------------------   10758
m150503_iid=2923751_0      ------------------------------------------------------------   10134
m141031_iid=115118_0       ------------------------------------------------------------   7981
m150430_iid=2722448_0      ------------------------------------------------------------   9403
m150507_iid=3102647_0      TAATCAAATATAAATACTGCAAAAAGAAAACTCTAA-ATAACACTTACATATATTTAAAT   9764
m141108_iid=1811866_0      ------------------------------------------------------------   9038
m150501_iid=2948495_0      ------------------------------------------------------------   10373
m150504_iid=3238195_0      ------------------------------------------------------------   10610
m150503_iid=3388511_0      TAATCAAATATAAATACTGCAAAAAG-AAACTCTAAAATAACACTTACATATATTTAAAT   11104
m150505_iid=3777611_0      ------------------------------------------------------------   11830
m150430_iid=2438833_0      ------------------------------------------------------------   12224
m141108_iid=1698969_0      ------------------------------------------------------------   11147
m150430_iid=3059190_0      TAATCAAATATAAATACTGCAAAAAGAAAACTCTAAAATAACACTTACATATATTTAAAT   9719
m141107_iid=1383067_0      ------------------------------------------------------------   7061
m141101_iid=597008_0       TAATCAAATATAAATACTGCAAAAAGAAAACTCTAAAATAACACTTACATATATTTAAAT   6797
m150506_iid=2596496_0      ------------------------------------------------------------   10491
m141102_iid=910051_0       ------------------------------------------------------------   7428
m150505_iid=3784017_0      ------------------------------------------------------------   12474
m141101_iid=682151_0       TAATCAAATATAAATACTGCAAAAAGAAAACTCTAAAATAACACTTACATATATTTAAAT   11717
m150501_iid=3748029_0      TAATCAAATATAAATACTGCAAAAAGAAAACTCTAAAATAACACTTACATATATTTAAAT   12263
m150504_iid=2876272_0      ------------------------------------------------------------   14037
NC_042820.1                TAATCAAATATAAATACTGCAAAAAGAAAACTCTAAAATAACACTTACATATATTTAAAT   14374
m141102_iid=1228825_0      ------------------------------------------------------------   11951
m150504_iid=2876274_0      ------------------------------------------------------------   13965
m141030_iid=80036_0        ------------------------------------------------------------   11554
m150506_iid=2596494_0      ------------------------------------------------------------   10983
m150503_iid=2526862_0      ------------------------------------------------------------   13936
m150506_iid=2567000_0      ------------------------------------------------------------   11909
m150507_iid=3073271_0      ------------------------------------------------------------   11626
m150504_iid=3156087_0      ------------------------------------------------------------   11376
m150505_iid=2498502_0      ------------------------------------------------------------   10138
m141102_iid=754227_0       ------------------------------------------------------------   10254
m150507_iid=3469514_0      ------------------------------------------------------------   10241
Rdo_MitoGenome             TAATCAAATATAAATACTGCAAAAAGAAAACTCTAAAATAACACTTACATATATTTAAAT   14375
m141101_iid=614933_0       ------------------------------------------------------------   9134


m141031_iid=347546_0       ------------------------------------------------------------   7511
m150506_iid=3127414_0      TTATTTATAAAATAAAAAGCCAAAATAAAACTTTCCAACATAAAAAATAAATTTTAACAC   10811
m141102_iid=1245517_0      ------------------------------------------------------------   7295
m141107_iid=1405727_0      ------------------------------------------------------------   7315
m141102_iid=987614_0       ------------------------------------------------------------   11885
m141108_iid=1701429_0      ------------------------------------------------------------   8969
m141102_iid=987286_0       ------------------------------------------------------------   7493
m150430_iid=3272125_0      ------------------------------------------------------------   10013
m141102_iid=754225_0       ------------------------------------------------------------   9952
```

```
m141107_iid=1409934_0      ------------------------------------------------------------  10262
m150503_iid=3416920_0      ------------------------------------------------------------  10188
m141108_iid=1800121_0      ------------------------------------------------------------  10617
m141031_iid=147540_0       ------------------------------------------------------------  11780
m150507_iid=3674924_0      ------------------------------------------------------------  10758
m150503_iid=2923751_0      ------------------------------------------------------------  10134
m141031_iid=115118_0       ------------------------------------------------------------  7981
m150430_iid=2722448_0      ------------------------------------------------------------  9403
m150507_iid=3102647_0      TTATTATATA-AATAAAAGCCAAAATAAAACTTTCCAACATAAAAAATAAATTTTAACAC  9823
m141108_iid=1811866_0      ------------------------------------------------------------  9038
m150501_iid=2948495_0      ------------------------------------------------------------  10373
m150504_iid=3238195_0      ------------------------------------------------------------  10610
m150503_iid=3388511_0      TTATTATATAAATAAAAAGCCAAAATAAAACTTTCCAACAT-AAAAATAAATTTTAACAC  11163
m150505_iid=3777611_0      ------------------------------------------------------------  11830
m150430_iid=2438833_0      ------------------------------------------------------------  12224
m141108_iid=1698969_0      ------------------------------------------------------------  11147
m150430_iid=3059190_0      TTATTATATAA-ATAAAAGCCAAAATAAAACTTTCCAACATAAAAATAAATTT-------  9771
m141107_iid=1383067_0      ------------------------------------------------------------  7061
m141101_iid=597008_0       TTATTATATAAATAAAAAGCCAAAATAAAACTTTCCAACATAAAAAATAAATTTTAACAC  6857
m150506_iid=2596496_0      ------------------------------------------------------------  10491
m141102_iid=910051_0       ------------------------------------------------------------  7428
m150505_iid=3784017_0      ------------------------------------------------------------  12474
m141101_iid=682151_0       TTATTATATAAATAAAAAGCCAAAATAAAACTTTCCAACATAAAAAATAAATTTTAACAC  11777
m150501_iid=3748029_0      TTAT--TATTATAAAAAAGCC---------------------------------------  12282
m150504_iid=2876272_0      ------------------------------------------------------------  14037
NC_042820.1                TTATTATATAAATAAAAAGCCAAAATAAAACTTTCCAACATAAAAAATAAATTTTAACAC  14434
m141102_iid=1228825_0      ------------------------------------------------------------  11951
m150504_iid=2876274_0      ------------------------------------------------------------  13965
m141030_iid=80036_0        ------------------------------------------------------------  11554
m150506_iid=2596494_0      ------------------------------------------------------------  10983
m150503_iid=2526862_0      ------------------------------------------------------------  13936
m150506_iid=2567000_0      ------------------------------------------------------------  11909
m150507_iid=3073271_0      ------------------------------------------------------------  11626
m150504_iid=3156087_0      ------------------------------------------------------------  11376
m150505_iid=2498502_0      ------------------------------------------------------------  10138
m141102_iid=754227_0       ------------------------------------------------------------  10254
m150507_iid=3469514_0      ------------------------------------------------------------  10241
Rdo_MitoGenome             TTATTATATAAATAAAAAGCCAAAATAAAACTTTCCAACATAAAAAATAAATTTTAACAC  14435
m141101_iid=614933_0       ------------------------------------------------------------  9134


m141031_iid=347546_0       ------------------------------------------------------------  7511
m150506_iid=3127414_0      TTTAAAAATTTTTTTCCACAAAATAAGTAAAATTGACCAACCTAACC-AAGCACCGAAAA  10870
m141102_iid=1245517_0      ------------------------------------------------------------  7295
m141107_iid=1405727_0      ------------------------------------------------------------  7315
m141102_iid=987614_0       ------------------------------------------------------------  11885
m141108_iid=1701429_0      ------------------------------------------------------------  8969
m141102_iid=987286_0       ------------------------------------------------------------  7493
m150430_iid=3272125_0      ------------------------------------------------------------  10013
m141102_iid=754225_0       ------------------------------------------------------------  9952
m141107_iid=1409934_0      ------------------------------------------------------------  10262
m150503_iid=3416920_0      ------------------------------------------------------------  10188
m141108_iid=1800121_0      ------------------------------------------------------------  10617
m141031_iid=147540_0       ------------------------------------------------------------  11780
m150507_iid=3674924_0      ------------------------------------------------------------  10758
m150503_iid=2923751_0      ------------------------------------------------------------  10134
m141031_iid=115118_0       ------------------------------------------------------------  7981
m150430_iid=2722448_0      ------------------------------------------------------------  9403
m150507_iid=3102647_0      TTTAAAAATTTTTTTCCACAAAATAAGTAAAATTGACCAACCTAAC-AAAGCACCGA---  9879
m141108_iid=1811866_0      ------------------------------------------------------------  9038
m150501_iid=2948495_0      ------------------------------------------------------------  10373
m150504_iid=3238195_0      ------------------------------------------------------------  10610
m150503_iid=3388511_0      TTTAAAAATTTTTTTCCACAAAATAAGTAAAATTGACCAACCTAACCAAAGCACCGAAAA  11223
m150505_iid=3777611_0      ------------------------------------------------------------  11830
m150430_iid=2438833_0      ------------------------------------------------------------  12224
m141108_iid=1698969_0      ------------------------------------------------------------  11147
m150430_iid=3059190_0      ------------------------------------------------------------  9771
m141107_iid=1383067_0      ------------------------------------------------------------  7061
m141101_iid=597008_0       TTTAAAAATTTTTTTCCACAAAATAAGTAAAATTGACCAACCTAACCAAAGCACCGAAAA  6917
m150506_iid=2596496_0      ------------------------------------------------------------  10491
m141102_iid=910051_0       ------------------------------------------------------------  7428
```

```
m150505_iid=3784017_0       ------------------------------------------------------------   12474
m141101_iid=682151_0        TTTAAAAATTTTTTTCCACAAAATAAGTAAAATTGACCAACCTAACCAAAGCACCGAAAA   11837
m150501_iid=3748029_0       ------------------------------------------------------------   12282
m150504_iid=2876272_0       ------------------------------------------------------------   14037
NC_042820.1                 TTTAAAAATTTTTTTCCACAAAATAAGTAAAATTGACCAACCTAACCAAAGCACCGAAAA   14494
m141102_iid=1228825_0       ------------------------------------------------------------   11951
m150504_iid=2876274_0       ------------------------------------------------------------   13965
m141030_iid=80036_0         ------------------------------------------------------------   11554
m150506_iid=2596494_0       ------------------------------------------------------------   10983
m150503_iid=2526862_0       ------------------------------------------------------------   13936
m150506_iid=2567000_0       ------------------------------------------------------------   11909
m150507_iid=3073271_0       ------------------------------------------------------------   11626
m150504_iid=3156087_0       ------------------------------------------------------------   11376
m150505_iid=2498502_0       ------------------------------------------------------------   10138
m141102_iid=754227_0        ------------------------------------------------------------   10254
m150507_iid=3469514_0       ------------------------------------------------------------   10241
Rdo_MitoGenome              TTTAAAAATTTTTTTCCACAAAATAAGTAAAATTGACCAACCTAACCAAAGCACCGAAAA   14495
m141101_iid=614933_0        ------------------------------------------------------------   9134


m141031_iid=347546_0        ------------------------------------------------------------   7511
m150506_iid=3127414_0       AGAACAGCTGGAACAGGAAA-CACGACGAAGAAGCGAAAACGAGGCAACGCCGCCCTCGG   10929
m141102_iid=1245517_0       ------------------------------------------------------------   7295
m141107_iid=1405727_0       ------------------------------------------------------------   7315
m141102_iid=987614_0        ------------------------------------------------------------   11885
m141108_iid=1701429_0       ------------------------------------------------------------   8969
m141102_iid=987286_0        ------------------------------------------------------------   7493
m150430_iid=3272125_0       ------------------------------------------------------------   10013
m141102_iid=754225_0        ------------------------------------------------------------   9952
m141107_iid=1409934_0       ------------------------------------------------------------   10262
m150503_iid=3416920_0       ------------------------------------------------------------   10188
m141108_iid=1800121_0       ------------------------------------------------------------   10617
m141031_iid=147540_0        ------------------------------------------------------------   11780
m150507_iid=3674924_0       ------------------------------------------------------------   10758
m150503_iid=2923751_0       ------------------------------------------------------------   10134
m141031_iid=115118_0        ------------------------------------------------------------   7981
m150430_iid=2722448_0       ------------------------------------------------------------   9403
m150507_iid=3102647_0       ------------------------------------------------------------   9879
m141108_iid=1811866_0       ------------------------------------------------------------   9038
m150501_iid=2948495_0       ------------------------------------------------------------   10373
m150504_iid=3238195_0       ------------------------------------------------------------   10610
m150503_iid=3388511_0       AGAACAGCTGGAACAGGAAACCACGACGAAGAAGCGAAAACGAGGCAACGCCGCCCTC-G   11282
m150505_iid=3777611_0       ------------------------------------------------------------   11830
m150430_iid=2438833_0       ------------------------------------------------------------   12224
m141108_iid=1698969_0       ------------------------------------------------------------   11147
m150430_iid=3059190_0       ------------------------------------------------------------   9771
m141107_iid=1383067_0       ------------------------------------------------------------   7061
m141101_iid=597008_0        AGAACAGCTGGAACAGGAAACCACGACGAAGAAGCGAAAACGAGGCAACGCCGCCCTCGG   6977
m150506_iid=2596496_0       ------------------------------------------------------------   10491
m141102_iid=910051_0        ------------------------------------------------------------   7428
m150505_iid=3784017_0       ------------------------------------------------------------   12474
m141101_iid=682151_0        AGAACAGCTGGAACAGGAAACCACGACGAAGAAGCGAAAACGAGGCAACGCCGCCCTCGG   11897
m150501_iid=3748029_0       ------------------------------------------------------------   12282
m150504_iid=2876272_0       ------------------------------------------------------------   14037
NC_042820.1                 AGAACAGCCGGAACAGGAAACCACGACGAAGAAGCGAAAACGAGGAAACGCCGCCCTCGG   14554
m141102_iid=1228825_0       ------------------------------------------------------------   11951
m150504_iid=2876274_0       ------------------------------------------------------------   13965
m141030_iid=80036_0         ------------------------------------------------------------   11554
m150506_iid=2596494_0       ------------------------------------------------------------   10983
m150503_iid=2526862_0       ------------------------------------------------------------   13936
m150506_iid=2567000_0       ------------------------------------------------------------   11909
m150507_iid=3073271_0       ------------------------------------------------------------   11626
m150504_iid=3156087_0       ------------------------------------------------------------   11376
m150505_iid=2498502_0       ------------------------------------------------------------   10138
m141102_iid=754227_0        ------------------------------------------------------------   10254
m150507_iid=3469514_0       ------------------------------------------------------------   10241
Rdo_MitoGenome              AGAACAGCTGGAACAGGAAACCACGACGAAGAAGCGAAAACGAGGCAACGCCGCCCTCGG   14555
m141101_iid=614933_0        ------------------------------------------------------------   9134


m141031_iid=347546_0        ------------------------------------------------------------   7511
```

```
m150506_iid=3127414_0      GGTAAGAACCAGGTTTAATCCACGG--ATAACAACGCCCTATTGCCAACACCATGAATAC   10987
m141102_iid=1245517_0      ------------------------------------------------------------   7295
m141107_iid=1405727_0      ------------------------------------------------------------   7315
m141102_iid=987614_0       ------------------------------------------------------------   11885
m141108_iid=1701429_0      ------------------------------------------------------------   8969
m141102_iid=987286_0       ------------------------------------------------------------   7493
m150430_iid=3272125_0      ------------------------------------------------------------   10013
m141102_iid=754225_0       ------------------------------------------------------------   9952
m141107_iid=1409934_0      ------------------------------------------------------------   10262
m150503_iid=3416920_0      ------------------------------------------------------------   10188
m141108_iid=1800121_0      ------------------------------------------------------------   10617
m141031_iid=147540_0       ------------------------------------------------------------   11780
m150507_iid=3674924_0      ------------------------------------------------------------   10758
m150503_iid=2923751_0      ------------------------------------------------------------   10134
m141031_iid=115118_0       ------------------------------------------------------------   7981
m150430_iid=2722448_0      ------------------------------------------------------------   9403
m150507_iid=3102647_0      ------------------------------------------------------------   9879
m141108_iid=1811866_0      ------------------------------------------------------------   9038
m150501_iid=2948495_0      ------------------------------------------------------------   10373
m150504_iid=3238195_0      ------------------------------------------------------------   10610
m150503_iid=3388511_0      GGTAAGAACCAGGTTTAATCCACGGGATAAGCAACGCCCTATTGCCAAC-CCATGAATAC   11341
m150505_iid=3777611_0      ------------------------------------------------------------   11830
m150430_iid=2438833_0      ------------------------------------------------------------   12224
m141108_iid=1698969_0      ------------------------------------------------------------   11147
m150430_iid=3059190_0      ------------------------------------------------------------   9771
m141107_iid=1383067_0      ------------------------------------------------------------   7061
m141101_iid=597008_0       G-TAAGAACCAGGTTTAATCCACGGGATAAGCAACGCCCTATTGCCAAC-CCATGAATAC   7035
m150506_iid=2596496_0      ------------------------------------------------------------   10491
m141102_iid=910051_0       ------------------------------------------------------------   7428
m150505_iid=3784017_0      ------------------------------------------------------------   12474
m141101_iid=682151_0       GGTAAGAACCAGGTTTAATCCACGGGATAAGCAACGCCCTATTGCCAAC-CCATGAATAC   11956
m150501_iid=3748029_0      ------------------------------------------------------------   12282
m150504_iid=2876272_0      ------------------------------------------------------------   14037
NC_042820.1                GGTACGAACCAGGTTTAACCCACGGGATAAGCAACGCCCTATTGCCAAC-CCATGAATAC   14613
m141102_iid=1228825_0      ------------------------------------------------------------   11951
m150504_iid=2876274_0      ------------------------------------------------------------   13965
m141030_iid=80036_0        ------------------------------------------------------------   11554
m150506_iid=2596494_0      ------------------------------------------------------------   10983
m150503_iid=2526862_0      ------------------------------------------------------------   13936
m150506_iid=2567000_0      ------------------------------------------------------------   11909
m150507_iid=3073271_0      ------------------------------------------------------------   11626
m150504_iid=3156087_0      ------------------------------------------------------------   11376
m150505_iid=2498502_0      ------------------------------------------------------------   10138
m141102_iid=754227_0       ------------------------------------------------------------   10254
m150507_iid=3469514_0      ------------------------------------------------------------   10241
Rdo_MitoGenome             GGTAAGAACCAGGTTTAATCCACGGGATAAGCAACGCCCTATTGCCAAC-CCATGAATAC   14614
m141101_iid=614933_0       ------------------------------------------------------------   9134


m141031_iid=347546_0       ------------------------------------------------------------   7511
m150506_iid=3127414_0      GA-CAAAGCACCG-AAAAGAACAGCAGGAACAGGAAACGT--ACGAAGAAGCGAAAACGA   11043
m141102_iid=1245517_0      ------------------------------------------------------------   7295
m141107_iid=1405727_0      ------------------------------------------------------------   7315
m141102_iid=987614_0       ------------------------------------------------------------   11885
m141108_iid=1701429_0      ------------------------------------------------------------   8969
m141102_iid=987286_0       ------------------------------------------------------------   7493
m150430_iid=3272125_0      ------------------------------------------------------------   10013
m141102_iid=754225_0       ------------------------------------------------------------   9952
m141107_iid=1409934_0      ------------------------------------------------------------   10262
m150503_iid=3416920_0      ------------------------------------------------------------   10188
m141108_iid=1800121_0      ------------------------------------------------------------   10617
m141031_iid=147540_0       ------------------------------------------------------------   11780
m150507_iid=3674924_0      ------------------------------------------------------------   10758
m150503_iid=2923751_0      ------------------------------------------------------------   10134
m141031_iid=115118_0       ------------------------------------------------------------   7981
m150430_iid=2722448_0      ------------------------------------------------------------   9403
m150507_iid=3102647_0      ------------------------------------------------------------   9879
m141108_iid=1811866_0      ------------------------------------------------------------   9038
m150501_iid=2948495_0      ------------------------------------------------------------   10373
m150504_iid=3238195_0      ------------------------------------------------------------   10610
m150503_iid=3388511_0      GACCAAAGCACCGAAAAAGAACAGCAGGAACAGGAAACCACGACGAAGAAGCGAAAACGA   11401
```

```
m150505_iid=3777611_0     ------------------------------------------------------------  11830
m150430_iid=2438833_0     ------------------------------------------------------------  12224
m141108_iid=1698969_0     ------------------------------------------------------------  11147
m150430_iid=3059190_0     ------------------------------------------------------------  9771
m141107_iid=1383067_0     ------------------------------------------------------------  7061
m141101_iid=597008_0      GACCAAAGCACCGAAAAAGAACAGCAGGAACAGGAAACCACGACGAAGAAGCGAAAACGA  7095
m150506_iid=2596496_0     ------------------------------------------------------------  10491
m141102_iid=910051_0      ------------------------------------------------------------  7428
m150505_iid=3784017_0     ------------------------------------------------------------  12474
m141101_iid=682151_0      GACCAAAGCACCGAAAAAGAACAGCAGGAACAGGAAACCACGACGAAGAAGCGAAAACGA  12016
m150501_iid=3748029_0     ------------------------------------------------------------  12282
m150504_iid=2876272_0     ------------------------------------------------------------  14037
NC_042820.1               GACCAAAGCACCGAAAAAGAACAGCTTGAACAGGAAACCACGACGAAGAAGCGAAAACGA  14673
m141102_iid=1228825_0     ------------------------------------------------------------  11951
m150504_iid=2876274_0     ------------------------------------------------------------  13965
m141030_iid=80036_0       ------------------------------------------------------------  11554
m150506_iid=2596494_0     ------------------------------------------------------------  10983
m150503_iid=2526862_0     ------------------------------------------------------------  13936
m150506_iid=2567000_0     ------------------------------------------------------------  11909
m150507_iid=3073271_0     ------------------------------------------------------------  11626
m150504_iid=3156087_0     ------------------------------------------------------------  11376
m150505_iid=2498502_0     ------------------------------------------------------------  10138
m141102_iid=754227_0      ------------------------------------------------------------  10254
m150507_iid=3469514_0     ------------------------------------------------------------  10241
Rdo_MitoGenome            GACCAAAGCACCGAAAAAGAACAGCTTGAACAGGAAACCACGACGAAGAAGCGAAAACGA  14674
m141101_iid=614933_0      ------------------------------------------------------------  9134


m141031_iid=347546_0      ------------------------------------------------------------  7511
m150506_iid=3127414_0     GGAAAACACGCCG-CCTCGGGGTACG-AACCAGGTTAAACCCACGGGATAAGCAACGCCC  11101
m141102_iid=1245517_0     ------------------------------------------------------------  7295
m141107_iid=1405727_0     ------------------------------------------------------------  7315
m141102_iid=987614_0      ------------------------------------------------------------  11885
m141108_iid=1701429_0     ------------------------------------------------------------  8969
m141102_iid=987286_0      ------------------------------------------------------------  7493
m150430_iid=3272125_0     ------------------------------------------------------------  10013
m141102_iid=754225_0      ------------------------------------------------------------  9952
m141107_iid=1409934_0     ------------------------------------------------------------  10262
m150503_iid=3416920_0     ------------------------------------------------------------  10188
m141108_iid=1800121_0     ------------------------------------------------------------  10617
m141031_iid=147540_0      ------------------------------------------------------------  11780
m150507_iid=3674924_0     ------------------------------------------------------------  10758
m150503_iid=2923751_0     ------------------------------------------------------------  10134
m141031_iid=115118_0      ------------------------------------------------------------  7981
m150430_iid=2722448_0     ------------------------------------------------------------  9403
m150507_iid=3102647_0     ------------------------------------------------------------  9879
m141108_iid=1811866_0     ------------------------------------------------------------  9038
m150501_iid=2948495_0     ------------------------------------------------------------  10373
m150504_iid=3238195_0     ------------------------------------------------------------  10610
m150503_iid=3388511_0     GGAA---ACGCCG-CCTCGGGGTACGAAACCAGGTTAAACCCACGGGATAAGCAACGCCC  11457
m150505_iid=3777611_0     ------------------------------------------------------------  11830
m150430_iid=2438833_0     ------------------------------------------------------------  12224
m141108_iid=1698969_0     ------------------------------------------------------------  11147
m150430_iid=3059190_0     ------------------------------------------------------------  9771
m141107_iid=1383067_0     ------------------------------------------------------------  7061
m141101_iid=597008_0      GGAA---ACGCCGCCCTCGGGGTACG-AACCAGGTTAAACCCACGGGATAAGCA------  7145
m150506_iid=2596496_0     ------------------------------------------------------------  10491
m141102_iid=910051_0      ------------------------------------------------------------  7428
m150505_iid=3784017_0     ------------------------------------------------------------  12474
m141101_iid=682151_0      GGAA---ACGCCGCCCTCGGGGTACG-AACCAGGTTAAACCCACGG--------------  12058
m150501_iid=3748029_0     ------------------------------------------------------------  12282
m150504_iid=2876272_0     ------------------------------------------------------------  14037
NC_042820.1               GGAA---ACGCCGCCCTCGGGGTACG-AACCAGGTTTAACCCACGGGATAAGCAACGCCC  14729
m141102_iid=1228825_0     ------------------------------------------------------------  11951
m150504_iid=2876274_0     ------------------------------------------------------------  13965
m141030_iid=80036_0       ------------------------------------------------------------  11554
m150506_iid=2596494_0     ------------------------------------------------------------  10983
m150503_iid=2526862_0     ------------------------------------------------------------  13936
m150506_iid=2567000_0     ------------------------------------------------------------  11909
m150507_iid=3073271_0     ------------------------------------------------------------  11626
m150504_iid=3156087_0     ------------------------------------------------------------  11376
```

```
m150505_iid=2498502_0      ------------------------------------------------------------  10138
m141102_iid=754227_0       ------------------------------------------------------------  10254
m150507_iid=3469514_0      ------------------------------------------------------------  10241
Rdo_MitoGenome             GGAA---ACGCCGCCCTCGGGGTAAG-AACCAGGTTTAACCCAC----------------  14714
m141101_iid=614933_0       ------------------------------------------------------------  9134


m141031_iid=347546_0       ------------------------------------------------------------  7511
m150506_iid=3127414_0      TATTGCCAACCCATGAATACGACCAAAGCACCGAAAAAGAACAGCAGGAACAGGAAACCA  11161
m141102_iid=1245517_0      ------------------------------------------------------------  7295
m141107_iid=1405727_0      ------------------------------------------------------------  7315
m141102_iid=987614_0       ------------------------------------------------------------  11885
m141108_iid=1701429_0      ------------------------------------------------------------  8969
m141102_iid=987286_0       ------------------------------------------------------------  7493
m150430_iid=3272125_0      ------------------------------------------------------------  10013
m141102_iid=754225_0       ------------------------------------------------------------  9952
m141107_iid=1409934_0      ------------------------------------------------------------  10262
m150503_iid=3416920_0      ------------------------------------------------------------  10188
m141108_iid=1800121_0      ------------------------------------------------------------  10617
m141031_iid=147540_0       ------------------------------------------------------------  11780
m150507_iid=3674924_0      ------------------------------------------------------------  10758
m150503_iid=2923751_0      ------------------------------------------------------------  10134
m141031_iid=115118_0       ------------------------------------------------------------  7981
m150430_iid=2722448_0      ------------------------------------------------------------  9403
m150507_iid=3102647_0      ------------------------------------------------------------  9879
m141108_iid=1811866_0      ------------------------------------------------------------  9038
m150501_iid=2948495_0      ------------------------------------------------------------  10373
m150504_iid=3238195_0      ------------------------------------------------------------  10610
m150503_iid=3388511_0      TATTGCCAA-CCATGAATACGACCAAAGCACCGAAAAAGAACAGCAGGAACAGGAAACCA  11516
m150505_iid=3777611_0      ------------------------------------------------------------  11830
m150430_iid=2438833_0      ------------------------------------------------------------  12224
m141108_iid=1698969_0      ------------------------------------------------------------  11147
m150430_iid=3059190_0      ------------------------------------------------------------  9771
m141107_iid=1383067_0      ------------------------------------------------------------  7061
m141101_iid=597008_0       ------------------------------------------------------------  7145
m150506_iid=2596496_0      ------------------------------------------------------------  10491
m141102_iid=910051_0       ------------------------------------------------------------  7428
m150505_iid=3784017_0      ------------------------------------------------------------  12474
m141101_iid=682151_0       ------------------------------------------------------------  12058
m150501_iid=3748029_0      ------------------------------------------------------------  12282
m150504_iid=2876272_0      ------------------------------------------------------------  14037
NC_042820.1                TATTGCCAACCCATGAATACGACCAAAGCACCGAAAAAGAACAGCTTGAACAGGAAACCA  14789
m141102_iid=1228825_0      ------------------------------------------------------------  11951
m150504_iid=2876274_0      ------------------------------------------------------------  13965
m141030_iid=80036_0        ------------------------------------------------------------  11554
m150506_iid=2596494_0      ------------------------------------------------------------  10983
m150503_iid=2526862_0      ------------------------------------------------------------  13936
m150506_iid=2567000_0      ------------------------------------------------------------  11909
m150507_iid=3073271_0      ------------------------------------------------------------  11626
m150504_iid=3156087_0      ------------------------------------------------------------  11376
m150505_iid=2498502_0      ------------------------------------------------------------  10138
m141102_iid=754227_0       ------------------------------------------------------------  10254
m150507_iid=3469514_0      ------------------------------------------------------------  10241
Rdo_MitoGenome             ------------------------------------------------------------  14714
m141101_iid=614933_0       ------------------------------------------------------------  9134


m141031_iid=347546_0       ------------------------------------------------------------  7511
m150506_iid=3127414_0      CGACGAAGAAGCGAAAACGAGGAAACGCCGCCTC--GGGGACGAACCAGGTTAAACCCA-  11218
m141102_iid=1245517_0      ------------------------------------------------------------  7295
m141107_iid=1405727_0      ------------------------------------------------------------  7315
m141102_iid=987614_0       ------------------------------------------------------------  11885
m141108_iid=1701429_0      ------------------------------------------------------------  8969
m141102_iid=987286_0       ------------------------------------------------------------  7493
m150430_iid=3272125_0      ------------------------------------------------------------  10013
m141102_iid=754225_0       ------------------------------------------------------------  9952
m141107_iid=1409934_0      ------------------------------------------------------------  10262
m150503_iid=3416920_0      ------------------------------------------------------------  10188
m141108_iid=1800121_0      ------------------------------------------------------------  10617
m141031_iid=147540_0       ------------------------------------------------------------  11780
m150507_iid=3674924_0      ------------------------------------------------------------  10758
```

```
m150503_iid=2923751_0       ------------------------------------------------------------   10134
m141031_iid=115118_0        ------------------------------------------------------------   7981
m150430_iid=2722448_0       ------------------------------------------------------------   9403
m150507_iid=3102647_0       ------------------------------------------------------------   9879
m141108_iid=1811866_0       ------------------------------------------------------------   9038
m150501_iid=2948495_0       ------------------------------------------------------------   10373
m150504_iid=3238195_0       ------------------------------------------------------------   10610
m150503_iid=3388511_0       CGACGAAGAAGCGAAAACGAGGAAACGCCGCCCTCGGGGTACGAAACGGTTAAAC-----   11571
m150505_iid=3777611_0       ------------------------------------------------------------   11830
m150430_iid=2438833_0       ------------------------------------------------------------   12224
m141108_iid=1698969_0       ------------------------------------------------------------   11147
m150430_iid=3059190_0       ------------------------------------------------------------   9771
m141107_iid=1383067_0       ------------------------------------------------------------   7061
m141101_iid=597008_0        ------------------------------------------------------------   7145
m150506_iid=2596496_0       ------------------------------------------------------------   10491
m141102_iid=910051_0        ------------------------------------------------------------   7428
m150505_iid=3784017_0       ------------------------------------------------------------   12474
m141101_iid=682151_0        ------------------------------------------------------------   12058
m150501_iid=3748029_0       ------------------------------------------------------------   12282
m150504_iid=2876272_0       ------------------------------------------------------------   14037
NC_042820.1                 CGACGAAGAAGCGGAAACGAGGAAACGCCACCCTCGGGGTAAGAACCAGGTTTAACCCAT   14849
m141102_iid=1228825_0       ------------------------------------------------------------   11951
m150504_iid=2876274_0       ------------------------------------------------------------   13965
m141030_iid=80036_0         ------------------------------------------------------------   11554
m150506_iid=2596494_0       ------------------------------------------------------------   10983
m150503_iid=2526862_0       ------------------------------------------------------------   13936
m150506_iid=2567000_0       ------------------------------------------------------------   11909
m150507_iid=3073271_0       ------------------------------------------------------------   11626
m150504_iid=3156087_0       ------------------------------------------------------------   11376
m150505_iid=2498502_0       ------------------------------------------------------------   10138
m141102_iid=754227_0        ------------------------------------------------------------   10254
m150507_iid=3469514_0       ------------------------------------------------------------   10241
Rdo_MitoGenome              ------------------------------------------------------------   14714
m141101_iid=614933_0        ------------------------------------------------------------   9134


m141031_iid=347546_0        ------------------------------------------------------------   7511
m150506_iid=3127414_0       AGGATCAACAACGTCCTATTGCCAAC-----CCATGAATACGACAAGCACCGAAAAAGAA   11273
m141102_iid=1245517_0       ------------------------------------------------------------   7295
m141107_iid=1405727_0       ------------------------------------------------------------   7315
m141102_iid=987614_0        ------------------------------------------------------------   11885
m141108_iid=1701429_0       ------------------------------------------------------------   8969
m141102_iid=987286_0        ------------------------------------------------------------   7493
m150430_iid=3272125_0       ------------------------------------------------------------   10013
m141102_iid=754225_0        ------------------------------------------------------------   9952
m141107_iid=1409934_0       ------------------------------------------------------------   10262
m150503_iid=3416920_0       ------------------------------------------------------------   10188
m141108_iid=1800121_0       ------------------------------------------------------------   10617
m141031_iid=147540_0        ------------------------------------------------------------   11780
m150507_iid=3674924_0       ------------------------------------------------------------   10758
m150503_iid=2923751_0       ------------------------------------------------------------   10134
m141031_iid=115118_0        ------------------------------------------------------------   7981
m150430_iid=2722448_0       ------------------------------------------------------------   9403
m150507_iid=3102647_0       ------------------------------------------------------------   9879
m141108_iid=1811866_0       ------------------------------------------------------------   9038
m150501_iid=2948495_0       ------------------------------------------------------------   10373
m150504_iid=3238195_0       ------------------------------------------------------------   10610
m150503_iid=3388511_0       ------------------------------------------------------------   11571
m150505_iid=3777611_0       ------------------------------------------------------------   11830
m150430_iid=2438833_0       ------------------------------------------------------------   12224
m141108_iid=1698969_0       ------------------------------------------------------------   11147
m150430_iid=3059190_0       ------------------------------------------------------------   9771
m141107_iid=1383067_0       ------------------------------------------------------------   7061
m141101_iid=597008_0        ------------------------------------------------------------   7145
m150506_iid=2596496_0       ------------------------------------------------------------   10491
m141102_iid=910051_0        ------------------------------------------------------------   7428
m150505_iid=3784017_0       ------------------------------------------------------------   12474
m141101_iid=682151_0        ------------------------------------------------------------   12058
m150501_iid=3748029_0       ------------------------------------------------------------   12282
m150504_iid=2876272_0       ------------------------------------------------------------   14037
NC_042820.1                 AGGATCAACAACGTCCTATCGCCAACAAAATCAATACATTAATTATAATTTTCTTTTTTA   14909
```

```
m141102_iid=1228825_0      ------------------------------------------------------------   11951
m150504_iid=2876274_0      ------------------------------------------------------------   13965
m141030_iid=80036_0        ------------------------------------------------------------   11554
m150506_iid=2596494_0      ------------------------------------------------------------   10983
m150503_iid=2526862_0      ------------------------------------------------------------   13936
m150506_iid=2567000_0      ------------------------------------------------------------   11909
m150507_iid=3073271_0      ------------------------------------------------------------   11626
m150504_iid=3156087_0      ------------------------------------------------------------   11376
m150505_iid=2498502_0      ------------------------------------------------------------   10138
m141102_iid=754227_0       ------------------------------------------------------------   10254
m150507_iid=3469514_0      ------------------------------------------------------------   10241
Rdo_MitoGenome             AGGATCAACAACGTCCTATTCCCAACAAAATCAATACATTAATTATAATTTTCTTTTTTA   14774
m141101_iid=614933_0       ------------------------------------------------------------   9134


m141031_iid=347546_0       ------------------------------------------------------------   7511
m150506_iid=3127414_0      CAGCAGGAACAGGAAACCACGACGAAGAAGCGAAAAGAGGCAACGCGCCGCCTCGGGGTA   11333
m141102_iid=1245517_0      ------------------------------------------------------------   7295
m141107_iid=1405727_0      ------------------------------------------------------------   7315
m141102_iid=987614_0       ------------------------------------------------------------   11885
m141108_iid=1701429_0      ------------------------------------------------------------   8969
m141102_iid=987286_0       ------------------------------------------------------------   7493
m150430_iid=3272125_0      ------------------------------------------------------------   10013
m141102_iid=754225_0       ------------------------------------------------------------   9952
m141107_iid=1409934_0      ------------------------------------------------------------   10262
m150503_iid=3416920_0      ------------------------------------------------------------   10188
m141108_iid=1800121_0      ------------------------------------------------------------   10617
m141031_iid=147540_0       ------------------------------------------------------------   11780
m150507_iid=3674924_0      ------------------------------------------------------------   10758
m150503_iid=2923751_0      ------------------------------------------------------------   10134
m141031_iid=115118_0       ------------------------------------------------------------   7981
m150430_iid=2722448_0      ------------------------------------------------------------   9403
m150507_iid=3102647_0      ------------------------------------------------------------   9879
m141108_iid=1811866_0      ------------------------------------------------------------   9038
m150501_iid=2948495_0      ------------------------------------------------------------   10373
m150504_iid=3238195_0      ------------------------------------------------------------   10610
m150503_iid=3388511_0      ------------------------------------------------------------   11571
m150505_iid=3777611_0      ------------------------------------------------------------   11830
m150430_iid=2438833_0      ------------------------------------------------------------   12224
m141108_iid=1698969_0      ------------------------------------------------------------   11147
m150430_iid=3059190_0      ------------------------------------------------------------   9771
m141107_iid=1383067_0      ------------------------------------------------------------   7061
m141101_iid=597008_0       ------------------------------------------------------------   7145
m150506_iid=2596496_0      ------------------------------------------------------------   10491
m141102_iid=910051_0       ------------------------------------------------------------   7428
m150505_iid=3784017_0      ------------------------------------------------------------   12474
m141101_iid=682151_0       ------------------------------------------------------------   12058
m150501_iid=3748029_0      ------------------------------------------------------------   12282
m150504_iid=2876272_0      ------------------------------------------------------------   14037
NC_042820.1                ACTTAAAAATTTAAACACTAGTAAATAAATATTTAATAAATTATGAACAAGACCTAAGTC   14969
m141102_iid=1228825_0      ------------------------------------------------------------   11951
m150504_iid=2876274_0      ------------------------------------------------------------   13965
m141030_iid=80036_0        ------------------------------------------------------------   11554
m150506_iid=2596494_0      ------------------------------------------------------------   10983
m150503_iid=2526862_0      ------------------------------------------------------------   13936
m150506_iid=2567000_0      ------------------------------------------------------------   11909
m150507_iid=3073271_0      ------------------------------------------------------------   11626
m150504_iid=3156087_0      ------------------------------------------------------------   11376
m150505_iid=2498502_0      ------------------------------------------------------------   10138
m141102_iid=754227_0       ------------------------------------------------------------   10254
m150507_iid=3469514_0      ------------------------------------------------------------   10241
Rdo_MitoGenome             ACTTAAAAATTTAAACACTAGTAAATAAATATTTAATAAATTATGAACAAAACCTAAGTC   14834
m141101_iid=614933_0       ------------------------------------------------------------   9134


m141031_iid=347546_0       ------------------------------------------------------------   7511
m150506_iid=3127414_0      CGAACCAGTTAACCCACGGGATAAGCAACGCCT---------------------------   11366
m141102_iid=1245517_0      ------------------------------------------------------------   7295
m141107_iid=1405727_0      ------------------------------------------------------------   7315
m141102_iid=987614_0       ------------------------------------------------------------   11885
m141108_iid=1701429_0      ------------------------------------------------------------   8969
```

```
m141102_iid=987286_0      ------------------------------------------------------------  7493
m150430_iid=3272125_0     ------------------------------------------------------------  10013
m141102_iid=754225_0      ------------------------------------------------------------  9952
m141107_iid=1409934_0     ------------------------------------------------------------  10262
m150503_iid=3416920_0     ------------------------------------------------------------  10188
m141108_iid=1800121_0     ------------------------------------------------------------  10617
m141031_iid=147540_0      ------------------------------------------------------------  11780
m150507_iid=3674924_0     ------------------------------------------------------------  10758
m150503_iid=2923751_0     ------------------------------------------------------------  10134
m141031_iid=115118_0      ------------------------------------------------------------  7981
m150430_iid=2722448_0     ------------------------------------------------------------  9403
m150507_iid=3102647_0     ------------------------------------------------------------  9879
m141108_iid=1811866_0     ------------------------------------------------------------  9038
m150501_iid=2948495_0     ------------------------------------------------------------  10373
m150504_iid=3238195_0     ------------------------------------------------------------  10610
m150503_iid=3388511_0     ------------------------------------------------------------  11571
m150505_iid=3777611_0     ------------------------------------------------------------  11830
m150430_iid=2438833_0     ------------------------------------------------------------  12224
m141108_iid=1698969_0     ------------------------------------------------------------  11147
m150430_iid=3059190_0     ------------------------------------------------------------  9771
m141107_iid=1383067_0     ------------------------------------------------------------  7061
m141101_iid=597008_0      ------------------------------------------------------------  7145
m150506_iid=2596496_0     ------------------------------------------------------------  10491
m141102_iid=910051_0      ------------------------------------------------------------  7428
m150505_iid=3784017_0     ------------------------------------------------------------  12474
m141101_iid=682151_0      ------------------------------------------------------------  12058
m150501_iid=3748029_0     ------------------------------------------------------------  12282
m150504_iid=2876272_0     ------------------------------------------------------------  14037
NC_042820.1               CTTAAATTTTAAAACTAAATTTCAATTTAGCTTAAAATTTATTAATAAAATTATTAACAT  15029
m141102_iid=1228825_0     ------------------------------------------------------------  11951
m150504_iid=2876274_0     ------------------------------------------------------------  13965
m141030_iid=80036_0       ------------------------------------------------------------  11554
m150506_iid=2596494_0     ------------------------------------------------------------  10983
m150503_iid=2526862_0     ------------------------------------------------------------  13936
m150506_iid=2567000_0     ------------------------------------------------------------  11909
m150507_iid=3073271_0     ------------------------------------------------------------  11626
m150504_iid=3156087_0     ------------------------------------------------------------  11376
m150505_iid=2498502_0     ------------------------------------------------------------  10138
m141102_iid=754227_0      ------------------------------------------------------------  10254
m150507_iid=3469514_0     ------------------------------------------------------------  10241
Rdo_MitoGenome            CTTAAATTTTAAAACTAAATTTTAATTTAGATTAAAATTTATTAATAAAATTATTAACAT  14894
m141101_iid=614933_0      ------------------------------------------------------------  9134


m141031_iid=347546_0      ------------------------------------------------------------  7511
m150506_iid=3127414_0     ------------------------------------------------------------  11366
m141102_iid=1245517_0     ------------------------------------------------------------  7295
m141107_iid=1405727_0     ------------------------------------------------------------  7315
m141102_iid=987614_0      ------------------------------------------------------------  11885
m141108_iid=1701429_0     ------------------------------------------------------------  8969
m141102_iid=987286_0      ------------------------------------------------------------  7493
m150430_iid=3272125_0     ------------------------------------------------------------  10013
m141102_iid=754225_0      ------------------------------------------------------------  9952
m141107_iid=1409934_0     ------------------------------------------------------------  10262
m150503_iid=3416920_0     ------------------------------------------------------------  10188
m141108_iid=1800121_0     ------------------------------------------------------------  10617
m141031_iid=147540_0      ------------------------------------------------------------  11780
m150507_iid=3674924_0     ------------------------------------------------------------  10758
m150503_iid=2923751_0     ------------------------------------------------------------  10134
m141031_iid=115118_0      ------------------------------------------------------------  7981
m150430_iid=2722448_0     ------------------------------------------------------------  9403
m150507_iid=3102647_0     ------------------------------------------------------------  9879
m141108_iid=1811866_0     ------------------------------------------------------------  9038
m150501_iid=2948495_0     ------------------------------------------------------------  10373
m150504_iid=3238195_0     ------------------------------------------------------------  10610
m150503_iid=3388511_0     ------------------------------------------------------------  11571
m150505_iid=3777611_0     ------------------------------------------------------------  11830
m150430_iid=2438833_0     ------------------------------------------------------------  12224
m141108_iid=1698969_0     ------------------------------------------------------------  11147
m150430_iid=3059190_0     ------------------------------------------------------------  9771
m141107_iid=1383067_0     ------------------------------------------------------------  7061
```

```
m141101_iid=597008_0        ------------------------------------------------------------  7145
m150506_iid=2596496_0       ------------------------------------------------------------  10491
m141102_iid=910051_0        ------------------------------------------------------------  7428
m150505_iid=3784017_0       ------------------------------------------------------------  12474
m141101_iid=682151_0        ------------------------------------------------------------  12058
m150501_iid=3748029_0       ------------------------------------------------------------  12282
m150504_iid=2876272_0       ------------------------------------------------------------  14037
NC_042820.1                 TTTATTAATAAATAATTTTATTAATAATAAATAAGGTTTTTTTTTTTTTTTTTTTTTCTA  15089
m141102_iid=1228825_0       ------------------------------------------------------------  11951
m150504_iid=2876274_0       ------------------------------------------------------------  13965
m141030_iid=80036_0         ------------------------------------------------------------  11554
m150506_iid=2596494_0       ------------------------------------------------------------  10983
m150503_iid=2526862_0       ------------------------------------------------------------  13936
m150506_iid=2567000_0       ------------------------------------------------------------  11909
m150507_iid=3073271_0       ------------------------------------------------------------  11626
m150504_iid=3156087_0       ------------------------------------------------------------  11376
m150505_iid=2498502_0       ------------------------------------------------------------  10138
m141102_iid=754227_0        ------------------------------------------------------------  10254
m150507_iid=3469514_0       ------------------------------------------------------------  10241
Rdo_MitoGenome              TTTATTAATAAATAATTTTATTAATAATAAATAAGGTTTTTTTTTTTTTTTTTTTTTHTA  14954
m141101_iid=614933_0        ------------------------------------------------------------  9134

m141031_iid=347546_0        ------------------------------------------------------------  7511
m150506_iid=3127414_0       ------------------------------------------------------------  11366
m141102_iid=1245517_0       ------------------------------------------------------------  7295
m141107_iid=1405727_0       ------------------------------------------------------------  7315
m141102_iid=987614_0        ------------------------------------------------------------  11885
m141108_iid=1701429_0       ------------------------------------------------------------  8969
m141102_iid=987286_0        ------------------------------------------------------------  7493
m150430_iid=3272125_0       ------------------------------------------------------------  10013
m141102_iid=754225_0        ------------------------------------------------------------  9952
m141107_iid=1409934_0       ------------------------------------------------------------  10262
m150503_iid=3416920_0       ------------------------------------------------------------  10188
m141108_iid=1800121_0       ------------------------------------------------------------  10617
m141031_iid=147540_0        ------------------------------------------------------------  11780
m150507_iid=3674924_0       ------------------------------------------------------------  10758
m150503_iid=2923751_0       ------------------------------------------------------------  10134
m141031_iid=115118_0        ------------------------------------------------------------  7981
m150430_iid=2722448_0       ------------------------------------------------------------  9403
m150507_iid=3102647_0       ------------------------------------------------------------  9879
m141108_iid=1811866_0       ------------------------------------------------------------  9038
m150501_iid=2948495_0       ------------------------------------------------------------  10373
m150504_iid=3238195_0       ------------------------------------------------------------  10610
m150503_iid=3388511_0       ------------------------------------------------------------  11571
m150505_iid=3777611_0       ------------------------------------------------------------  11830
m150430_iid=2438833_0       ------------------------------------------------------------  12224
m141108_iid=1698969_0       ------------------------------------------------------------  11147
m150430_iid=3059190_0       ------------------------------------------------------------  9771
m141107_iid=1383067_0       ------------------------------------------------------------  7061
m141101_iid=597008_0        ------------------------------------------------------------  7145
m150506_iid=2596496_0       ------------------------------------------------------------  10491
m141102_iid=910051_0        ------------------------------------------------------------  7428
m150505_iid=3784017_0       ------------------------------------------------------------  12474
m141101_iid=682151_0        ------------------------------------------------------------  12058
m150501_iid=3748029_0       ------------------------------------------------------------  12282
m150504_iid=2876272_0       ------------------------------------------------------------  14037
NC_042820.1                 TAATTTTTATATTCAAATAAATAATTATAATAAAATAATTTTAATTTAAAATTATAAAAA  15149
m141102_iid=1228825_0       ------------------------------------------------------------  11951
m150504_iid=2876274_0       ------------------------------------------------------------  13965
m141030_iid=80036_0         ------------------------------------------------------------  11554
m150506_iid=2596494_0       ------------------------------------------------------------  10983
m150503_iid=2526862_0       ------------------------------------------------------------  13936
m150506_iid=2567000_0       ------------------------------------------------------------  11909
m150507_iid=3073271_0       ------------------------------------------------------------  11626
m150504_iid=3156087_0       ------------------------------------------------------------  11376
m150505_iid=2498502_0       ------------------------------------------------------------  10138
m141102_iid=754227_0        ------------------------------------------------------------  10254
m150507_iid=3469514_0       ------------------------------------------------------------  10241
Rdo_MitoGenome              TAATTTTTATATTCAAATAAATAATTATAATAAAATAATTTTAATTTAAAATTATAAAAA  15014
m141101_iid=614933_0        ------------------------------------------------------------  9134
```

```
m141031_iid=347546_0      ------------------------------------------------------------  7511
m150506_iid=3127414_0     ------------------------------------------------------------  11366
m141102_iid=1245517_0     ------------------------------------------------------------  7295
m141107_iid=1405727_0     ------------------------------------------------------------  7315
m141102_iid=987614_0      ------------------------------------------------------------  11885
m141108_iid=1701429_0     ------------------------------------------------------------  8969
m141102_iid=987286_0      ------------------------------------------------------------  7493
m150430_iid=3272125_0     ------------------------------------------------------------  10013
m141102_iid=754225_0      ------------------------------------------------------------  9952
m141107_iid=1409934_0     ------------------------------------------------------------  10262
m150503_iid=3416920_0     ------------------------------------------------------------  10188
m141108_iid=1800121_0     ------------------------------------------------------------  10617
m141031_iid=147540_0      ------------------------------------------------------------  11780
m150507_iid=3674924_0     ------------------------------------------------------------  10758
m150503_iid=2923751_0     ------------------------------------------------------------  10134
m141031_iid=115118_0      ------------------------------------------------------------  7981
m150430_iid=2722448_0     ------------------------------------------------------------  9403
m150507_iid=3102647_0     ------------------------------------------------------------  9879
m141108_iid=1811866_0     ------------------------------------------------------------  9038
m150501_iid=2948495_0     ------------------------------------------------------------  10373
m150504_iid=3238195_0     ------------------------------------------------------------  10610
m150503_iid=3388511_0     ------------------------------------------------------------  11571
m150505_iid=3777611_0     ------------------------------------------------------------  11830
m150430_iid=2438833_0     ------------------------------------------------------------  12224
m141108_iid=1698969_0     ------------------------------------------------------------  11147
m150430_iid=3059190_0     ------------------------------------------------------------  9771
m141107_iid=1383067_0     ------------------------------------------------------------  7061
m141101_iid=597008_0      ------------------------------------------------------------  7145
m150506_iid=2596496_0     ------------------------------------------------------------  10491
m141102_iid=910051_0      ------------------------------------------------------------  7428
m150505_iid=3784017_0     ------------------------------------------------------------  12474
m141101_iid=682151_0      ------------------------------------------------------------  12058
m150501_iid=3748029_0     ------------------------------------------------------------  12282
m150504_iid=2876272_0     ------------------------------------------------------------  14037
NC_042820.1               ATTAATTTTTATTATAAAATTTTAATTATATAAGGGAAAAAATTATATAAAAATATTTAT  15209
m141102_iid=1228825_0     ------------------------------------------------------------  11951
m150504_iid=2876274_0     ------------------------------------------------------------  13965
m141030_iid=80036_0       ------------------------------------------------------------  11554
m150506_iid=2596494_0     ------------------------------------------------------------  10983
m150503_iid=2526862_0     ------------------------------------------------------------  13936
m150506_iid=2567000_0     ------------------------------------------------------------  11909
m150507_iid=3073271_0     ------------------------------------------------------------  11626
m150504_iid=3156087_0     ------------------------------------------------------------  11376
m150505_iid=2498502_0     ------------------------------------------------------------  10138
m141102_iid=754227_0      ------------------------------------------------------------  10254
m150507_iid=3469514_0     ------------------------------------------------------------  10241
Rdo_MitoGenome            ATTAATTTTTATTATAAAATTTTAATTATATAAGAGAAAAAATTATATAAAAATATTTAT  15074
m141101_iid=614933_0      ------------------------------------------------------------  9134


m141031_iid=347546_0      ------------------------------------------------------------  7511
m150506_iid=3127414_0     ------------------------------------------------------------  11366
m141102_iid=1245517_0     ------------------------------------------------------------  7295
m141107_iid=1405727_0     ------------------------------------------------------------  7315
m141102_iid=987614_0      ------------------------------------------------------------  11885
m141108_iid=1701429_0     ------------------------------------------------------------  8969
m141102_iid=987286_0      ------------------------------------------------------------  7493
m150430_iid=3272125_0     ------------------------------------------------------------  10013
m141102_iid=754225_0      ------------------------------------------------------------  9952
m141107_iid=1409934_0     ------------------------------------------------------------  10262
m150503_iid=3416920_0     ------------------------------------------------------------  10188
m141108_iid=1800121_0     ------------------------------------------------------------  10617
m141031_iid=147540_0      ------------------------------------------------------------  11780
m150507_iid=3674924_0     ------------------------------------------------------------  10758
m150503_iid=2923751_0     ------------------------------------------------------------  10134
m141031_iid=115118_0      ------------------------------------------------------------  7981
m150430_iid=2722448_0     ------------------------------------------------------------  9403
m150507_iid=3102647_0     ------------------------------------------------------------  9879
m141108_iid=1811866_0     ------------------------------------------------------------  9038
```

```
m150501_iid=2948495_0      ------------------------------------------------------------  10373
m150504_iid=3238195_0      ------------------------------------------------------------  10610
m150503_iid=3388511_0      ------------------------------------------------------------  11571
m150505_iid=3777611_0      ------------------------------------------------------------  11830
m150430_iid=2438833_0      ------------------------------------------------------------  12224
m141108_iid=1698969_0      ------------------------------------------------------------  11147
m150430_iid=3059190_0      ------------------------------------------------------------  9771
m141107_iid=1383067_0      ------------------------------------------------------------  7061
m141101_iid=597008_0       ------------------------------------------------------------  7145
m150506_iid=2596496_0      ------------------------------------------------------------  10491
m141102_iid=910051_0       ------------------------------------------------------------  7428
m150505_iid=3784017_0      ------------------------------------------------------------  12474
m141101_iid=682151_0       ------------------------------------------------------------  12058
m150501_iid=3748029_0      ------------------------------------------------------------  12282
m150504_iid=2876272_0      ------------------------------------------------------------  14037
NC_042820.1                TCATTAATTATATATTTATATATAAATAATTTATGTATAAATAAAATTAATATAATTTTA  15269
m141102_iid=1228825_0      ------------------------------------------------------------  11951
m150504_iid=2876274_0      ------------------------------------------------------------  13965
m141030_iid=80036_0        ------------------------------------------------------------  11554
m150506_iid=2596494_0      ------------------------------------------------------------  10983
m150503_iid=2526862_0      ------------------------------------------------------------  13936
m150506_iid=2567000_0      ------------------------------------------------------------  11909
m150507_iid=3073271_0      ------------------------------------------------------------  11626
m150504_iid=3156087_0      ------------------------------------------------------------  11376
m150505_iid=2498502_0      ------------------------------------------------------------  10138
m141102_iid=754227_0       ------------------------------------------------------------  10254
m150507_iid=3469514_0      ------------------------------------------------------------  10241
Rdo_MitoGenome             TCATTAATTATATATTTATATATAAATAATTTATGTATAAATAAAATTAATATAATTTTA  15134
m141101_iid=614933_0       ------------------------------------------------------------  9134

m141031_iid=347546_0       ------------------------------------------------------------  7511
m150506_iid=3127414_0      ------------------------------------------------------------  11366
m141102_iid=1245517_0      ------------------------------------------------------------  7295
m141107_iid=1405727_0      ------------------------------------------------------------  7315
m141102_iid=987614_0       ------------------------------------------------------------  11885
m141108_iid=1701429_0      ------------------------------------------------------------  8969
m141102_iid=987286_0       ------------------------------------------------------------  7493
m150430_iid=3272125_0      ------------------------------------------------------------  10013
m141102_iid=754225_0       ------------------------------------------------------------  9952
m141107_iid=1409934_0      ------------------------------------------------------------  10262
m150503_iid=3416920_0      ------------------------------------------------------------  10188
m141108_iid=1800121_0      ------------------------------------------------------------  10617
m141031_iid=147540_0       ------------------------------------------------------------  11780
m150507_iid=3674924_0      ------------------------------------------------------------  10758
m150503_iid=2923751_0      ------------------------------------------------------------  10134
m141031_iid=115118_0       ------------------------------------------------------------  7981
m150430_iid=2722448_0      ------------------------------------------------------------  9403
m150507_iid=3102647_0      ------------------------------------------------------------  9879
m141108_iid=1811866_0      ------------------------------------------------------------  9038
m150501_iid=2948495_0      ------------------------------------------------------------  10373
m150504_iid=3238195_0      ------------------------------------------------------------  10610
m150503_iid=3388511_0      ------------------------------------------------------------  11571
m150505_iid=3777611_0      ------------------------------------------------------------  11830
m150430_iid=2438833_0      ------------------------------------------------------------  12224
m141108_iid=1698969_0      ------------------------------------------------------------  11147
m150430_iid=3059190_0      ------------------------------------------------------------  9771
m141107_iid=1383067_0      ------------------------------------------------------------  7061
m141101_iid=597008_0       ------------------------------------------------------------  7145
m150506_iid=2596496_0      ------------------------------------------------------------  10491
m141102_iid=910051_0       ------------------------------------------------------------  7428
m150505_iid=3784017_0      ------------------------------------------------------------  12474
m141101_iid=682151_0       ------------------------------------------------------------  12058
m150501_iid=3748029_0      ------------------------------------------------------------  12282
m150504_iid=2876272_0      ------------------------------------------------------------  14037
NC_042820.1                TTGTAAATATATAATATATTTAAATATGTTATTATATATTAATAAATAATTATATATTAT  15329
m141102_iid=1228825_0      ------------------------------------------------------------  11951
m150504_iid=2876274_0      ------------------------------------------------------------  13965
m141030_iid=80036_0        ------------------------------------------------------------  11554
m150506_iid=2596494_0      ------------------------------------------------------------  10983
m150503_iid=2526862_0      ------------------------------------------------------------  13936
```

```
m150506_iid=2567000_0      ------------------------------------------------------------   11909
m150507_iid=3073271_0      ------------------------------------------------------------   11626
m150504_iid=3156087_0      ------------------------------------------------------------   11376
m150505_iid=2498502_0      ------------------------------------------------------------   10138
m141102_iid=754227_0       ------------------------------------------------------------   10254
m150507_iid=3469514_0      ------------------------------------------------------------   10241
Rdo_MitoGenome             TTGTAAATATATAATATATTCAAATATGTTATTATATATTAATAAATAATTATATATTAT   15194
m141101_iid=614933_0       ------------------------------------------------------------   9134


m141031_iid=347546_0       ------------------------------------------------------------   7511
m150506_iid=3127414_0      ------------------------------------------------------------   11366
m141102_iid=1245517_0      ------------------------------------------------------------   7295
m141107_iid=1405727_0      ------------------------------------------------------------   7315
m141102_iid=987614_0       ------------------------------------------------------------   11885
m141108_iid=1701429_0      ------------------------------------------------------------   8969
m141102_iid=987286_0       ------------------------------------------------------------   7493
m150430_iid=3272125_0      ------------------------------------------------------------   10013
m141102_iid=754225_0       ------------------------------------------------------------   9952
m141107_iid=1409934_0      ------------------------------------------------------------   10262
m150503_iid=3416920_0      ------------------------------------------------------------   10188
m141108_iid=1800121_0      ------------------------------------------------------------   10617
m141031_iid=147540_0       ------------------------------------------------------------   11780
m150507_iid=3674924_0      ------------------------------------------------------------   10758
m150503_iid=2923751_0      ------------------------------------------------------------   10134
m141031_iid=115118_0       ------------------------------------------------------------   7981
m150430_iid=2722448_0      ------------------------------------------------------------   9403
m150507_iid=3102647_0      ------------------------------------------------------------   9879
m141108_iid=1811866_0      ------------------------------------------------------------   9038
m150501_iid=2948495_0      ------------------------------------------------------------   10373
m150504_iid=3238195_0      ------------------------------------------------------------   10610
m150503_iid=3388511_0      ------------------------------------------------------------   11571
m150505_iid=3777611_0      ------------------------------------------------------------   11830
m150430_iid=2438833_0      ------------------------------------------------------------   12224
m141108_iid=1698969_0      ------------------------------------------------------------   11147
m150430_iid=3059190_0      ------------------------------------------------------------   9771
m141107_iid=1383067_0      ------------------------------------------------------------   7061
m141101_iid=597008_0       ------------------------------------------------------------   7145
m150506_iid=2596496_0      ------------------------------------------------------------   10491
m141102_iid=910051_0       ------------------------------------------------------------   7428
m150505_iid=3784017_0      ------------------------------------------------------------   12474
m141101_iid=682151_0       ------------------------------------------------------------   12058
m150501_iid=3748029_0      ------------------------------------------------------------   12282
m150504_iid=2876272_0      ------------------------------------------------------------   14037
NC_042820.1                ATATTTATATATAATTTATATATAAATATATATTTACATATTATATAAGTTCTTATTATT   15389
m141102_iid=1228825_0      ------------------------------------------------------------   11951
m150504_iid=2876274_0      ------------------------------------------------------------   13965
m141030_iid=80036_0        ------------------------------------------------------------   11554
m150506_iid=2596494_0      ------------------------------------------------------------   10983
m150503_iid=2526862_0      ------------------------------------------------------------   13936
m150506_iid=2567000_0      ------------------------------------------------------------   11909
m150507_iid=3073271_0      ------------------------------------------------------------   11626
m150504_iid=3156087_0      ------------------------------------------------------------   11376
m150505_iid=2498502_0      ------------------------------------------------------------   10138
m141102_iid=754227_0       ------------------------------------------------------------   10254
m150507_iid=3469514_0      ------------------------------------------------------------   10241
Rdo_MitoGenome             ATATTTATATATAATTTATATATAAATATATATTTACATATTATATAAGTTCTTATTATT   15254
m141101_iid=614933_0       ------------------------------------------------------------   9134


m141031_iid=347546_0       ------------------------------------------------------------   7511
m150506_iid=3127414_0      ------------------------------------------------------------   11366
m141102_iid=1245517_0      ------------------------------------------------------------   7295
m141107_iid=1405727_0      ------------------------------------------------------------   7315
m141102_iid=987614_0       ------------------------------------------------------------   11885
m141108_iid=1701429_0      ------------------------------------------------------------   8969
m141102_iid=987286_0       ------------------------------------------------------------   7493
m150430_iid=3272125_0      ------------------------------------------------------------   10013
m141102_iid=754225_0       ------------------------------------------------------------   9952
m141107_iid=1409934_0      ------------------------------------------------------------   10262
m150503_iid=3416920_0      ------------------------------------------------------------   10188
```

```
m141108_iid=1800121_0       ------------------------------------------------------------  10617
m141031_iid=147540_0        ------------------------------------------------------------  11780
m150507_iid=3674924_0       ------------------------------------------------------------  10758
m150503_iid=2923751_0       ------------------------------------------------------------  10134
m141031_iid=115118_0        ------------------------------------------------------------  7981
m150430_iid=2722448_0       ------------------------------------------------------------  9403
m150507_iid=3102647_0       ------------------------------------------------------------  9879
m141108_iid=1811866_0       ------------------------------------------------------------  9038
m150501_iid=2948495_0       ------------------------------------------------------------  10373
m150504_iid=3238195_0       ------------------------------------------------------------  10610
m150503_iid=3388511_0       ------------------------------------------------------------  11571
m150505_iid=3777611_0       ------------------------------------------------------------  11830
m150430_iid=2438833_0       ------------------------------------------------------------  12224
m141108_iid=1698969_0       ------------------------------------------------------------  11147
m150430_iid=3059190_0       ------------------------------------------------------------  9771
m141107_iid=1383067_0       ------------------------------------------------------------  7061
m141101_iid=597008_0        ------------------------------------------------------------  7145
m150506_iid=2596496_0       ------------------------------------------------------------  10491
m141102_iid=910051_0        ------------------------------------------------------------  7428
m150505_iid=3784017_0       ------------------------------------------------------------  12474
m141101_iid=682151_0        ------------------------------------------------------------  12058
m150501_iid=3748029_0       ------------------------------------------------------------  12282
m150504_iid=2876272_0       ------------------------------------------------------------  14037
NC_042820.1                 ATCCCAATACCTATTTCTAATAGGTTATTATATATTGATATATAATTATATATATATAAA  15449
m141102_iid=1228825_0       ------------------------------------------------------------  11951
m150504_iid=2876274_0       ------------------------------------------------------------  13965
m141030_iid=80036_0         ------------------------------------------------------------  11554
m150506_iid=2596494_0       ------------------------------------------------------------  10983
m150503_iid=2526862_0       ------------------------------------------------------------  13936
m150506_iid=2567000_0       ------------------------------------------------------------  11909
m150507_iid=3073271_0       ------------------------------------------------------------  11626
m150504_iid=3156087_0       ------------------------------------------------------------  11376
m150505_iid=2498502_0       ------------------------------------------------------------  10138
m141102_iid=754227_0        ------------------------------------------------------------  10254
m150507_iid=3469514_0       ------------------------------------------------------------  10241
Rdo_MitoGenome              ATCCCAATACCTATTTCTAATAGGTTATTATATATTAATATATAATTATATATATATAAA  15314
m141101_iid=614933_0        ------------------------------------------------------------  9134
```

```
m141031_iid=347546_0        ------------------------------------------------------------  7511
m150506_iid=3127414_0       ------------------------------------------------------------  11366
m141102_iid=1245517_0       ------------------------------------------------------------  7295
m141107_iid=1405727_0       ------------------------------------------------------------  7315
m141102_iid=987614_0        ------------------------------------------------------------  11885
m141108_iid=1701429_0       ------------------------------------------------------------  8969
m141102_iid=987286_0        ------------------------------------------------------------  7493
m150430_iid=3272125_0       ------------------------------------------------------------  10013
m141102_iid=754225_0        ------------------------------------------------------------  9952
m141107_iid=1409934_0       ------------------------------------------------------------  10262
m150503_iid=3416920_0       ------------------------------------------------------------  10188
m141108_iid=1800121_0       ------------------------------------------------------------  10617
m141031_iid=147540_0        ------------------------------------------------------------  11780
m150507_iid=3674924_0       ------------------------------------------------------------  10758
m150503_iid=2923751_0       ------------------------------------------------------------  10134
m141031_iid=115118_0        ------------------------------------------------------------  7981
m150430_iid=2722448_0       ------------------------------------------------------------  9403
m150507_iid=3102647_0       ------------------------------------------------------------  9879
m141108_iid=1811866_0       ------------------------------------------------------------  9038
m150501_iid=2948495_0       ------------------------------------------------------------  10373
m150504_iid=3238195_0       ------------------------------------------------------------  10610
m150503_iid=3388511_0       ------------------------------------------------------------  11571
m150505_iid=3777611_0       ------------------------------------------------------------  11830
m150430_iid=2438833_0       ------------------------------------------------------------  12224
m141108_iid=1698969_0       ------------------------------------------------------------  11147
m150430_iid=3059190_0       ------------------------------------------------------------  9771
m141107_iid=1383067_0       ------------------------------------------------------------  7061
m141101_iid=597008_0        ------------------------------------------------------------  7145
m150506_iid=2596496_0       ------------------------------------------------------------  10491
m141102_iid=910051_0        ------------------------------------------------------------  7428
m150505_iid=3784017_0       ------------------------------------------------------------  12474
m141101_iid=682151_0        ------------------------------------------------------------  12058
```

```
m150501_iid=3748029_0      ------------------------------------------------------------  12282
m150504_iid=2876272_0      ------------------------------------------------------------  14037
NC_042820.1                TCAAGATATATATTAATAAATATAAATTAACTTTAAATTTAATATTATATATAAATATGT  15509
m141102_iid=1228825_0      ------------------------------------------------------------  11951
m150504_iid=2876274_0      ------------------------------------------------------------  13965
m141030_iid=80036_0        ------------------------------------------------------------  11554
m150506_iid=2596494_0      ------------------------------------------------------------  10983
m150503_iid=2526862_0      ------------------------------------------------------------  13936
m150506_iid=2567000_0      ------------------------------------------------------------  11909
m150507_iid=3073271_0      ------------------------------------------------------------  11626
m150504_iid=3156087_0      ------------------------------------------------------------  11376
m150505_iid=2498502_0      ------------------------------------------------------------  10138
m141102_iid=754227_0       ------------------------------------------------------------  10254
m150507_iid=3469514_0      ------------------------------------------------------------  10241
Rdo_MitoGenome             TCAAGATATATATTAATAAATATAAATTAACTTTAAATTTAATATTATATATAAATATGT  15374
m141101_iid=614933_0       ------------------------------------------------------------  9134


m141031_iid=347546_0       ------------------------------------------------------------  7511
m150506_iid=3127414_0      ------------------------------------------------------------  11366
m141102_iid=1245517_0      ------------------------------------------------------------  7295
m141107_iid=1405727_0      ------------------------------------------------------------  7315
m141102_iid=987614_0       ------------------------------------------------------------  11885
m141108_iid=1701429_0      ------------------------------------------------------------  8969
m141102_iid=987286_0       ------------------------------------------------------------  7493
m150430_iid=3272125_0      ------------------------------------------------------------  10013
m141102_iid=754225_0       ------------------------------------------------------------  9952
m141107_iid=1409934_0      ------------------------------------------------------------  10262
m150503_iid=3416920_0      ------------------------------------------------------------  10188
m141108_iid=1800121_0      ------------------------------------------------------------  10617
m141031_iid=147540_0       ------------------------------------------------------------  11780
m150507_iid=3674924_0      ------------------------------------------------------------  10758
m150503_iid=2923751_0      ------------------------------------------------------------  10134
m141031_iid=115118_0       ------------------------------------------------------------  7981
m150430_iid=2722448_0      ------------------------------------------------------------  9403
m150507_iid=3102647_0      ------------------------------------------------------------  9879
m141108_iid=1811866_0      ------------------------------------------------------------  9038
m150501_iid=2948495_0      ------------------------------------------------------------  10373
m150504_iid=3238195_0      ------------------------------------------------------------  10610
m150503_iid=3388511_0      ------------------------------------------------------------  11571
m150505_iid=3777611_0      ------------------------------------------------------------  11830
m150430_iid=2438833_0      ------------------------------------------------------------  12224
m141108_iid=1698969_0      ------------------------------------------------------------  11147
m150430_iid=3059190_0      ------------------------------------------------------------  9771
m141107_iid=1383067_0      ------------------------------------------------------------  7061
m141101_iid=597008_0       ------------------------------------------------------------  7145
m150506_iid=2596496_0      ------------------------------------------------------------  10491
m141102_iid=910051_0       ------------------------------------------------------------  7428
m150505_iid=3784017_0      ------------------------------------------------------------  12474
m141101_iid=682151_0       ------------------------------------------------------------  12058
m150501_iid=3748029_0      ------------------------------------------------------------  12282
m150504_iid=2876272_0      ------------------------------------------------------------  14037
NC_042820.1                AAATATATATAAATATATAATTATATATGAATAACTTATTGTTATATATAAAATTATTTA  15569
m141102_iid=1228825_0      ------------------------------------------------------------  11951
m150504_iid=2876274_0      ------------------------------------------------------------  13965
m141030_iid=80036_0        ------------------------------------------------------------  11554
m150506_iid=2596494_0      ------------------------------------------------------------  10983
m150503_iid=2526862_0      ------------------------------------------------------------  13936
m150506_iid=2567000_0      ------------------------------------------------------------  11909
m150507_iid=3073271_0      ------------------------------------------------------------  11626
m150504_iid=3156087_0      ------------------------------------------------------------  11376
m150505_iid=2498502_0      ------------------------------------------------------------  10138
m141102_iid=754227_0       ------------------------------------------------------------  10254
m150507_iid=3469514_0      ------------------------------------------------------------  10241
Rdo_MitoGenome             AAATATATATAAATATATAATTATATATGAATAACTTATTGTTATATATAAAATTATTTA  15434
m141101_iid=614933_0       ------------------------------------------------------------  9134


m141031_iid=347546_0       ------------------------------------------------------------  7511
m150506_iid=3127414_0      ------------------------------------------------------------  11366
m141102_iid=1245517_0      ------------------------------------------------------------  7295
```

```
m141107_iid=1405727_0       ------------------------------------------------------------   7315
m141102_iid=987614_0        ------------------------------------------------------------   11885
m141108_iid=1701429_0       ------------------------------------------------------------   8969
m141102_iid=987286_0        ------------------------------------------------------------   7493
m150430_iid=3272125_0       ------------------------------------------------------------   10013
m141102_iid=754225_0        ------------------------------------------------------------   9952
m141107_iid=1409934_0       ------------------------------------------------------------   10262
m150503_iid=3416920_0       ------------------------------------------------------------   10188
m141108_iid=1800121_0       ------------------------------------------------------------   10617
m141031_iid=147540_0        ------------------------------------------------------------   11780
m150507_iid=3674924_0       ------------------------------------------------------------   10758
m150503_iid=2923751_0       ------------------------------------------------------------   10134
m141031_iid=115118_0        ------------------------------------------------------------   7981
m150430_iid=2722448_0       ------------------------------------------------------------   9403
m150507_iid=3102647_0       ------------------------------------------------------------   9879
m141108_iid=1811866_0       ------------------------------------------------------------   9038
m150501_iid=2948495_0       ------------------------------------------------------------   10373
m150504_iid=3238195_0       ------------------------------------------------------------   10610
m150503_iid=3388511_0       ------------------------------------------------------------   11571
m150505_iid=3777611_0       ------------------------------------------------------------   11830
m150430_iid=2438833_0       ------------------------------------------------------------   12224
m141108_iid=1698969_0       ------------------------------------------------------------   11147
m150430_iid=3059190_0       ------------------------------------------------------------   9771
m141107_iid=1383067_0       ------------------------------------------------------------   7061
m141101_iid=597008_0        ------------------------------------------------------------   7145
m150506_iid=2596496_0       ------------------------------------------------------------   10491
m141102_iid=910051_0        ------------------------------------------------------------   7428
m150505_iid=3784017_0       ------------------------------------------------------------   12474
m141101_iid=682151_0        ------------------------------------------------------------   12058
m150501_iid=3748029_0       ------------------------------------------------------------   12282
m150504_iid=2876272_0       ------------------------------------------------------------   14037
NC_042820.1                 TTAATTTAATAATTATATATAAATATATAGTTATATATATATATAAATATATATTTTATA   15629
m141102_iid=1228825_0       ------------------------------------------------------------   11951
m150504_iid=2876274_0       ------------------------------------------------------------   13965
m141030_iid=80036_0         ------------------------------------------------------------   11554
m150506_iid=2596494_0       ------------------------------------------------------------   10983
m150503_iid=2526862_0       ------------------------------------------------------------   13936
m150506_iid=2567000_0       ------------------------------------------------------------   11909
m150507_iid=3073271_0       ------------------------------------------------------------   11626
m150504_iid=3156087_0       ------------------------------------------------------------   11376
m150505_iid=2498502_0       ------------------------------------------------------------   10138
m141102_iid=754227_0        ------------------------------------------------------------   10254
m150507_iid=3469514_0       ------------------------------------------------------------   10241
Rdo_MitoGenome              TTAATTTAATAATTATATATAAATATATAGTTATATATATATATAAATATATATTTTATA   15494
m141101_iid=614933_0        ------------------------------------------------------------   9134


m141031_iid=347546_0        ------------------------------------------------------------   7511
m150506_iid=3127414_0       ------------------------------------------------------------   11366
m141102_iid=1245517_0       ------------------------------------------------------------   7295
m141107_iid=1405727_0       ------------------------------------------------------------   7315
m141102_iid=987614_0        ------------------------------------------------------------   11885
m141108_iid=1701429_0       ------------------------------------------------------------   8969
m141102_iid=987286_0        ------------------------------------------------------------   7493
m150430_iid=3272125_0       ------------------------------------------------------------   10013
m141102_iid=754225_0        ------------------------------------------------------------   9952
m141107_iid=1409934_0       ------------------------------------------------------------   10262
m150503_iid=3416920_0       ------------------------------------------------------------   10188
m141108_iid=1800121_0       ------------------------------------------------------------   10617
m141031_iid=147540_0        ------------------------------------------------------------   11780
m150507_iid=3674924_0       ------------------------------------------------------------   10758
m150503_iid=2923751_0       ------------------------------------------------------------   10134
m141031_iid=115118_0        ------------------------------------------------------------   7981
m150430_iid=2722448_0       ------------------------------------------------------------   9403
m150507_iid=3102647_0       ------------------------------------------------------------   9879
m141108_iid=1811866_0       ------------------------------------------------------------   9038
m150501_iid=2948495_0       ------------------------------------------------------------   10373
m150504_iid=3238195_0       ------------------------------------------------------------   10610
m150503_iid=3388511_0       ------------------------------------------------------------   11571
m150505_iid=3777611_0       ------------------------------------------------------------   11830
m150430_iid=2438833_0       ------------------------------------------------------------   12224
```

```
m141108_iid=1698969_0      ------------------------------------------------------------  11147
m150430_iid=3059190_0      ------------------------------------------------------------  9771
m141107_iid=1383067_0      ------------------------------------------------------------  7061
m141101_iid=597008_0       ------------------------------------------------------------  7145
m150506_iid=2596496_0      ------------------------------------------------------------  10491
m141102_iid=910051_0       ------------------------------------------------------------  7428
m150505_iid=3784017_0      ------------------------------------------------------------  12474
m141101_iid=682151_0       ------------------------------------------------------------  12058
m150501_iid=3748029_0      ------------------------------------------------------------  12282
m150504_iid=2876272_0      ------------------------------------------------------------  14037
NC_042820.1                TAGTAAAAAAAAAGTTAGCGCAAAAACACGTTTTGAATGAAAATCCATAAATACATGGTT  15689
m141102_iid=1228825_0      ------------------------------------------------------------  11951
m150504_iid=2876274_0      ------------------------------------------------------------  13965
m141030_iid=80036_0        ------------------------------------------------------------  11554
m150506_iid=2596494_0      ------------------------------------------------------------  10983
m150503_iid=2526862_0      ------------------------------------------------------------  13936
m150506_iid=2567000_0      ------------------------------------------------------------  11909
m150507_iid=3073271_0      ------------------------------------------------------------  11626
m150504_iid=3156087_0      ------------------------------------------------------------  11376
m150505_iid=2498502_0      ------------------------------------------------------------  10138
m141102_iid=754227_0       ------------------------------------------------------------  10254
m150507_iid=3469514_0      ------------------------------------------------------------  10241
Rdo_MitoGenome             TAGTAAAAAAAAAGTTAGCGCAAAAACACGTTTTGAATGAAAATCCATAAATACATGGTT  15554
m141101_iid=614933_0       ------------------------------------------------------------  9134


m141031_iid=347546_0       ------------------------------------------------------------  7511
m150506_iid=3127414_0      ------------------------------------------------------------  11366
m141102_iid=1245517_0      ------------------------------------------------------------  7295
m141107_iid=1405727_0      ------------------------------------------------------------  7315
m141102_iid=987614_0       ------------------------------------------------------------  11885
m141108_iid=1701429_0      ------------------------------------------------------------  8969
m141102_iid=987286_0       ------------------------------------------------------------  7493
m150430_iid=3272125_0      ------------------------------------------------------------  10013
m141102_iid=754225_0       ------------------------------------------------------------  9952
m141107_iid=1409934_0      ------------------------------------------------------------  10262
m150503_iid=3416920_0      ------------------------------------------------------------  10188
m141108_iid=1800121_0      ------------------------------------------------------------  10617
m141031_iid=147540_0       ------------------------------------------------------------  11780
m150507_iid=3674924_0      ------------------------------------------------------------  10758
m150503_iid=2923751_0      ------------------------------------------------------------  10134
m141031_iid=115118_0       ------------------------------------------------------------  7981
m150430_iid=2722448_0      ------------------------------------------------------------  9403
m150507_iid=3102647_0      ------------------------------------------------------------  9879
m141108_iid=1811866_0      ------------------------------------------------------------  9038
m150501_iid=2948495_0      ------------------------------------------------------------  10373
m150504_iid=3238195_0      ------------------------------------------------------------  10610
m150503_iid=3388511_0      ------------------------------------------------------------  11571
m150505_iid=3777611_0      ------------------------------------------------------------  11830
m150430_iid=2438833_0      ------------------------------------------------------------  12224
m141108_iid=1698969_0      ------------------------------------------------------------  11147
m150430_iid=3059190_0      ------------------------------------------------------------  9771
m141107_iid=1383067_0      ------------------------------------------------------------  7061
m141101_iid=597008_0       ------------------------------------------------------------  7145
m150506_iid=2596496_0      ------------------------------------------------------------  10491
m141102_iid=910051_0       ------------------------------------------------------------  7428
m150505_iid=3784017_0      ------------------------------------------------------------  12474
m141101_iid=682151_0       ------------------------------------------------------------  12058
m150501_iid=3748029_0      ------------------------------------------------------------  12282
m150504_iid=2876272_0      ------------------------------------------------------------  14037
NC_042820.1                TTGCACAACCAATAATTTATTAACTAAACCAAAGCCCATAAAAAGAACAAAAATCGCCTA  15749
m141102_iid=1228825_0      ------------------------------------------------------------  11951
m150504_iid=2876274_0      ------------------------------------------------------------  13965
m141030_iid=80036_0        ------------------------------------------------------------  11554
m150506_iid=2596494_0      ------------------------------------------------------------  10983
m150503_iid=2526862_0      ------------------------------------------------------------  13936
m150506_iid=2567000_0      ------------------------------------------------------------  11909
m150507_iid=3073271_0      ------------------------------------------------------------  11626
m150504_iid=3156087_0      ------------------------------------------------------------  11376
m150505_iid=2498502_0      ------------------------------------------------------------  10138
m141102_iid=754227_0       ------------------------------------------------------------  10254
```

```
m150507_iid=3469514_0       ------------------------------------------------------------   10241
Rdo_MitoGenome              TTGCACAACCAATAATTTATTAACTAAACCAAAACCCATAAAAAGAAAAAAAATCGCCTA   15614
m141101_iid=614933_0        ------------------------------------------------------------   9134


m141031_iid=347546_0        ------------------------------------------------------------   7511
m150506_iid=3127414_0       ------------------------------------------------------------   11366
m141102_iid=1245517_0       ------------------------------------------------------------   7295
m141107_iid=1405727_0       ------------------------------------------------------------   7315
m141102_iid=987614_0        ------------------------------------------------------------   11885
m141108_iid=1701429_0       ------------------------------------------------------------   8969
m141102_iid=987286_0        ------------------------------------------------------------   7493
m150430_iid=3272125_0       ------------------------------------------------------------   10013
m141102_iid=754225_0        ------------------------------------------------------------   9952
m141107_iid=1409934_0       ------------------------------------------------------------   10262
m150503_iid=3416920_0       ------------------------------------------------------------   10188
m141108_iid=1800121_0       ------------------------------------------------------------   10617
m141031_iid=147540_0        ------------------------------------------------------------   11780
m150507_iid=3674924_0       ------------------------------------------------------------   10758
m150503_iid=2923751_0       ------------------------------------------------------------   10134
m141031_iid=115118_0        ------------------------------------------------------------   7981
m150430_iid=2722448_0       ------------------------------------------------------------   9403
m150507_iid=3102647_0       ------------------------------------------------------------   9879
m141108_iid=1811866_0       ------------------------------------------------------------   9038
m150501_iid=2948495_0       ------------------------------------------------------------   10373
m150504_iid=3238195_0       ------------------------------------------------------------   10610
m150503_iid=3388511_0       ------------------------------------------------------------   11571
m150505_iid=3777611_0       ------------------------------------------------------------   11830
m150430_iid=2438833_0       ------------------------------------------------------------   12224
m141108_iid=1698969_0       ------------------------------------------------------------   11147
m150430_iid=3059190_0       ------------------------------------------------------------   9771
m141107_iid=1383067_0       ------------------------------------------------------------   7061
m141101_iid=597008_0        ------------------------------------------------------------   7145
m150506_iid=2596496_0       ------------------------------------------------------------   10491
m141102_iid=910051_0        ------------------------------------------------------------   7428
m150505_iid=3784017_0       ------------------------------------------------------------   12474
m141101_iid=682151_0        ------------------------------------------------------------   12058
m150501_iid=3748029_0       ------------------------------------------------------------   12282
m150504_iid=2876272_0       ------------------------------------------------------------   14037
NC_042820.1                 CGTTTTAAAACAAAAAATGGTGAGGCATTACTATGAACATAAACATTAATAAAAAAATAT   15809
m141102_iid=1228825_0       ------------------------------------------------------------   11951
m150504_iid=2876274_0       ------------------------------------------------------------   13965
m141030_iid=80036_0         ------------------------------------------------------------   11554
m150506_iid=2596494_0       ------------------------------------------------------------   10983
m150503_iid=2526862_0       ------------------------------------------------------------   13936
m150506_iid=2567000_0       ------------------------------------------------------------   11909
m150507_iid=3073271_0       ------------------------------------------------------------   11626
m150504_iid=3156087_0       ------------------------------------------------------------   11376
m150505_iid=2498502_0       ------------------------------------------------------------   10138
m141102_iid=754227_0        ------------------------------------------------------------   10254
m150507_iid=3469514_0       ------------------------------------------------------------   10241
Rdo_MitoGenome              CGTTTTAAAACAAAAAATGGTGAGGCACTACTATGAACATAAACATTAATAAAAAAATAT   15674
m141101_iid=614933_0        ------------------------------------------------------------   9134


m141031_iid=347546_0        --------------------------------------------------     7511
m150506_iid=3127414_0       --------------------------------------------------     11366
m141102_iid=1245517_0       --------------------------------------------------     7295
m141107_iid=1405727_0       --------------------------------------------------     7315
m141102_iid=987614_0        --------------------------------------------------     11885
m141108_iid=1701429_0       --------------------------------------------------     8969
m141102_iid=987286_0        --------------------------------------------------     7493
m150430_iid=3272125_0       --------------------------------------------------     10013
m141102_iid=754225_0        --------------------------------------------------     9952
m141107_iid=1409934_0       --------------------------------------------------     10262
m150503_iid=3416920_0       --------------------------------------------------     10188
m141108_iid=1800121_0       --------------------------------------------------     10617
m141031_iid=147540_0        --------------------------------------------------     11780
m150507_iid=3674924_0       --------------------------------------------------     10758
m150503_iid=2923751_0       --------------------------------------------------     10134
m141031_iid=115118_0        --------------------------------------------------     7981
```

m150430_iid=2722448_0	--	9403
m150507_iid=3102647_0	--	9879
m141108_iid=1811866_0	--	9038
m150501_iid=2948495_0	--	10373
m150504_iid=3238195_0	--	10610
m150503_iid=3388511_0	--	11571
m150505_iid=3777611_0	--	11830
m150430_iid=2438833_0	--	12224
m141108_iid=1698969_0	--	11147
m150430_iid=3059190_0	--	9771
m141107_iid=1383067_0	--	7061
m141101_iid=597008_0	--	7145
m150506_iid=2596496_0	--	10491
m141102_iid=910051_0	--	7428
m150505_iid=3784017_0	--	12474
m141101_iid=682151_0	--	12058
m150501_iid=3748029_0	--	12282
m150504_iid=2876272_0	--	14037
NC_042820.1	AAAAAAATTTAAAAGAACAGATACAAATTTTTGTTTACAAGTAAAGTTT-	15858
m141102_iid=1228825_0	--	11951
m150504_iid=2876274_0	--	13965
m141030_iid=80036_0	--	11554
m150506_iid=2596494_0	--	10983
m150503_iid=2526862_0	--	13936
m150506_iid=2567000_0	--	11909
m150507_iid=3073271_0	--	11626
m150504_iid=3156087_0	--	11376
m150505_iid=2498502_0	--	10138
m141102_iid=754227_0	--	10254
m150507_iid=3469514_0	--	10241
Rdo_MitoGenome	AAAAAAATTTAAAAGAACAGATACAAATTTTTGTTTACAAGTAAAGTTTT	15724
m141101_iid=614933_0	--	9134
