# Supplementary material for: Variants in the Mitochondrial Genome Sequence of Rhyzopertha dominica (Fabricius) (Coleoptera: Bostrycidae)
Source: Insects. 2021 Apr 27;12(5):387. doi: 10.3390/insects12050387 (PMC8146127; doi:10.3390/insects12050387)

Figure S6. Plot of GC content (%) versus nt in the predicted sequence (accession #MW020612) of the *Rhyzopertha dominica* mitochondrial genome.

Webgenetics.com

Plot GC content of DNA sequences Wed Sep 23 11:40:48 2020

Rdo_MitoGenome: 15724 bps, window size 25 bps, everage gc 25.30%, t 4761, c 2410, a 6984, g 1568

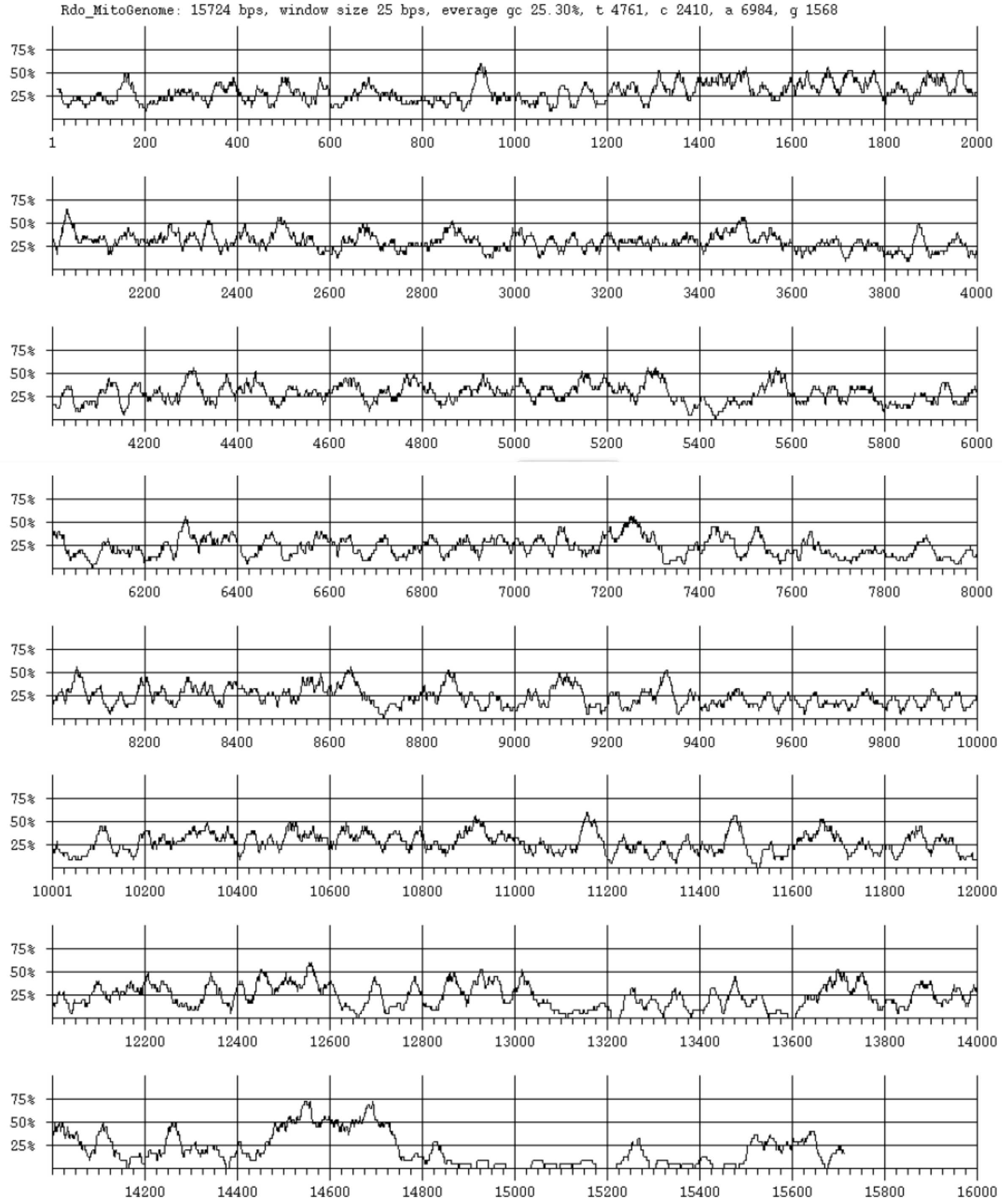

Supplement: Supplementary file 1 [file insects-12-00387-s001.zip › insects-1191702-s/Figure S6.pdf]
