# Supplementary material for: Variants in the Mitochondrial Genome Sequence of Rhyzopertha dominica (Fabricius) (Coleoptera: Bostrycidae)
Source: Insects. 2021 Apr 27;12(5):387. doi: 10.3390/insects12050387 (PMC8146127; doi:10.3390/insects12050387)
Supplement: Supplementary file 1 [file insects-12-00387-s001.zip › insects-1191702-s/insects-1191702-S1.docx]

**Figure S1.** Alignment of the predicted sequence (accession #MW020612; top sequence with Rdo_MitoGenome) of the *Rhyzopertha dominica* mitochondrial genome and that predicted by Ouyang et al. [15] (accession #NC_042820). Shaded areas are predicted coding sequences; notation above in black is the identification of the protein product; notation in green and red are the predicted start and stop codons, respectively, with the orientation in parentheses; SNVs are notated with turquoise shading.

**End trnI (-)**

**Start trnI (-)**

**trnI (-)**

Rdo_MitoGenome AATGAAGTGCCTGAAAAAAAGGATTATTTTGATAGAATAAATTATGTAGAAATACCTTCA 60

NC_042820.1 AATGAAGTGCCTGAAAAAAAGGATTATTTTGATAGAATAAATTATGTAGAAATACCTTCA 60

************************************************************

**Start trnM (+)**

**trnM (+)**

Rdo_MitoGenome TTATATGAAATAGAATTAAACTAAAACTTTAGATTCCAAAAATCTAAGTACATCTTATAC 120

NC_042820.1 TTATATGAAATAGAATTAAACTAAAACTTTAGATTCCAAAAATCTAAGTACATCTTATAC 120

************************************************************

**End trnQ (-)**

**trnQ (-)**

**End trnM(-)**

Rdo_MitoGenome TATCATATAAAAAGATAAGCTAATAAAGCTATTGGGTTCATACCCCAACCATAAAGGTTA 180

NC_042820.1 TATCATATAAAAAGATAAGCTAATAAAGCTATTGGGTTCATACCCCGACCATAAAGGTTA 180

********************************************** *************

**Start Nad2 (+)**

**Start trnQ (-)**

Rdo_MitoGenome TAATCCTTTTCTTTTTAATAATCAAATTATCAAAAATCTCATTTACAATTACAATAATAA 240

NC_042820.1 TAATCCTTTTCTTTTTAATAATCAAATTATCAAAAATCTCATTTACAATTACAATAATAA 240

************************************************************

Rdo_MitoGenome TCGGATCAATAATTACAATTTCATCGTATACATGAATAGGAATATGAATAGGCTTAGAAA 300

NC_042820.1 TCGGATCAATAATTACAATTTCATCGTATACATGAATAGGAATATGAATAGGCTTAGAAA 300

************************************************************

Rdo_MitoGenome TTAACCTTCTTTCAATTATTCCACTATTAACAACTAAAAAAAACATTTATTCAACCGAAG 360

NC_042820.1 TTAACCTTCTTTCAATTATTCCACTATTAACAACTAAAAAAAACATTTATTCAACCGAAG 360

************************************************************

Rdo_MitoGenome CAGCAATCAAATACTTCATTACACAAGCATTGGCATCATCAATCCTATTAATATCAATCC 420

NC_042820.1 CAGCAATCAAATACTTCATTACACAAGCATTGACATCATCAATCCTATTAATATCAATCC 420

******************************** ***************************

Rdo_MitoGenome TAATTTTATCAACAAATCTAATTAACCAAAAGTCCGCAATAATAATTCTTTATTCATCTT 480

NC_042820.1 TAATTTTATCAACAAATCTAATTAACCAAAAGTCCGCAATAATAATTCTTTATTCATCTT 480

************************************************************

Rdo_MitoGenome TAATAACAAAAATAGGAACAGCCCCATTCCATTTTTGATTTCCTGAAATCATTGAAGGAT 540

NC_042820.1 TAATAACAAAAATAGGAACAGCCCCATTCCATTTTTGATTTCCTGAAATCATTGAAGGAT 540

************************************************************

**nad2 (+)**

Rdo_MitoGenome TAGATTGAATAAATTGCTTAATTATAATAACCTGACAAAAAATTGCCCCTGCAATAATTC 600

NC_042820.1 TAGATTGAATAAATTGCTTAATTATAATAACCTGACAAAAAATTGCCCCTGCAATAATTC 600

************************************************************

Rdo_MitoGenome TAATATCATCAATAAATATAAACACATTTATTTACATTACAATTACGTCATCAATATTAA 660

NC_042820.1 TAATATCATCAATAAATATAAACACATTTATTTACATTACAATTACGTCATCAATATTAA 660

************************************************************

Rdo_MitoGenome TCAGAGGAATTATAGGAGTAAACCAGACAAGACTACGAAAAATCATAGCATATTCATCTA 720

NC_042820.1 TCAGAGGAATTATAGGAGTAAACCAGACAAGACTACGAAAAATCATAGCATATTCATCTA 720

************************************************************

Rdo_MitoGenome TTAACCATATAGCATGAATAATAGCATCAATAAACAATGAAAAAATCTTTTACATTTACT 780

NC_042820.1 TTAACCATATAGCATGAATAATAGCATCAATAAACAATGAAAAAATCTTTTACATTTACT 780

************************************************************

Rdo_MitoGenome TATCAATCTATATTACAATATCATCAATCTTCATTTTTACATTAAAAAGAAATAACTCCC 840

NC_042820.1 TATCAATCTATATTACAATATCATCAATCTTCATTTTTACATTAAAAAGAAATAACTCCC 840

************************************************************

Rdo_MitoGenome TATATATTTATCAAATAATAACAAGAAATCAAAGCCCAATTATTAAAAAAACATTTATTA 900

NC_042820.1 TATATATTTATCAAATAATAACAAGAAATCAAAGCCCAATTATTAAAAAAACATTTATTA 900

************************************************************

Rdo_MitoGenome TCAATCTACTAAGAATAGGAGGATTGCCCCCTCTCCTAGGATTTTTTCCCAAATGAATAG 960

NC_042820.1 TCAATCTACTAAGAATAGGAGGATTGCCCCCTCTCCTAGGATTTTTTCCCAAATGAATAG 960

************************************************************

Rdo_MitoGenome TAATTAACAGAATAATCCAAAGAAACAATCTAATTTTAAGAACCTTCATAGTAATAACAA 1020

NC_042820.1 TAATTAACAGAATAATCCAAAGAAACGATCTGATTTTAAGAACCTTCATAGTAATAACAA 1020

************************** **** ****************************

Rdo_MitoGenome CATTATTAACACTATACTTTTATATACAAATTTCAATACCAATCCTTACAATGTATAAAA 1080

NC_042820.1 CATTATTAACACTATACTTCTATATACAAATTTCAATACCAATCCTTACAATGTATAAAA 1080

******************* ****************************************

Rdo_MitoGenome TAAAAATAAAATGAAGGAAAAAAAACCCCCAACAATTCTTCATAAATAAAATAAACATAA 1140

NC_042820.1 TAAAAATAAAATGAAGGAAAAAAAACCCCCAACAATTCTTCATAAATAAAATAAACATAA 1140

************************************************************

**End Nad2 (+)**

**Start trnW (+)**

Rdo_MitoGenome TCAATCTGCTAAGAATTCCTGTGTGTACAATTTGATTTAACTTTTATTAAGGATTTAAGT 1200

NC_042820.1 TCAATCTGCTAAGAATTCCTGTATGTACAATTTGATTTAACTTTTATTAAGGATTTAAGT 1200

********************** *************************************

**End trnC (-)**

**End trnW (+)**

**trnW (+)**

Rdo_MitoGenome TAAATCAAACTAACAGCCTTCAAAGCTGTAAATAAGCAAATGTTTAAGCCTTAGAGCTTA 1260

NC_042820.1 TAAATCAAACTAACAGCCTTCAAAGCTGTAAATAAGCAAATGTTTAAGCCTTAGAGCTTA 1260

**Start trnC (-)**

************************************************************

**End trnY (-)**

**trnC (-)**

Rdo_MitoGenome ACTCACCTTTAAATTTGCAATTTAAAATCATTTTGAATATAAGACCGATGAGAAGGAAAA 1320

NC_042820.1 ACTCACCTTTAAATTTGCAATTTAAAATCATTTTGAATATAAGACCGATGAGAAGGAAAA 1320

************************************************************

**Start cox1 (+)**

**Start trnY (-)**

**trnY (-)**

Rdo_MitoGenome CCCCGTAAATAGATTTACAATCTAACGCTTAAACTCAGCCACCTCAACGAATAAATGATT 1380

NC_042820.1 CCCCGTAAATAGATTTACAATCTAACGCTTAAACTCAGCCACCTCAACGAATAAATGATT 1380

************************************************************

Rdo_MitoGenome ATATTCAACAAACCACAAAGACATCGGAACACTATACTTCATCTTCGGAATCTGATCAGG 1440

NC_042820.1 ATATTCAACAAACCACAAAGACATCGGAACACTATACTTCATCTTCGGAATCTGATCAGG 1440

************************************************************

Rdo_MitoGenome AATAGTAGGAACAGCCCTAAGAATACTAATCCGATCCGAATTAGGAAATCCAGGAGCCCT 1500

NC_042820.1 AATAGTAGGAACAGCCCTAAGAATACTAATCCGATCCGAATTAGGAAATCCAGGAGCCCT 1500

************************************************************

Rdo_MitoGenome AATTGGAGATGACCAAATTTATAACGTAATTGTAACAGCACATGCATTCATCATAATTTT 1560

NC_042820.1 AATTGGAGATGACCAAATTTATAACGTAATTGTAACAGCACATGCATTCATCATAATTTT 1560

************************************************************

Rdo_MitoGenome CTTCATAGTTATACCAATAATAATTGGAGGATTCGGAAATTGACTAGTTCCACTAATAAT 1620

NC_042820.1 CTTCATAGTTATACCAATAATAATTGGAGGATTCGGAAATTGACTAGTTCCACTAATAAT 1620

************************************************************

Rdo_MitoGenome TGGAGCACCAGATATAGCATTCCCTCGAATAAACAACATAAGATTTTGGCTTCTTCCACC 1680

NC_042820.1 TGGAGCACCAGATATAGCATTCCCTCGAATAAACAACATAAGATTTTGGCTTCTTCCACC 1680

************************************************************

Rdo_MitoGenome CTCCTTAACCCTTTTACTAACAAGAAGAATTGTAGAGACAGGAGCAGGAACAGGATGAAC 1740

NC_042820.1 CTCCTTAACCCTTTTACTAACAAGAAGAATTGTAGAGACAGGAGCAGGAACAGGATGAAC 1740

************************************************************

Rdo_MitoGenome AGTTTATCCACCTCTATCTAATAATACAGCCCATAGAGGAGCTTCTGTTGATTTAGCAAT 1800

NC_042820.1 AGTTTATCCACCTCTATCTAATAATACAGCCCATAGAGGAGCTTCTGTTGATTTAGCAAT 1800

************************************************************

**cox1 (+)**

Rdo_MitoGenome TTTTAGATTACATTTAGCAGGAATTTCATCAATTCTTGGAGCAGTAAACTTTATTACTAC 1860

NC_042820.1 TTTTAGATTACATTTAGCAGGAATTTCATCAATTCTTGGAGCAGTAAACTTTATTACTAC 1860

************************************************************

Rdo_MitoGenome AATCATTAATATACGACCAAAAGGAATAACACCAGAACGAATCCCCCTATTTGTATGATC 1920

NC_042820.1 AATCATTAATATACGACCAAAAGGAATAACACCAGAACGAATCCCCCTATTTGTATGATC 1920

************************************************************

Rdo_MitoGenome AGTAGGGATCACAGCTTTGCTTTTATTATTATCTCTTCCGGTTCTAGCTGGAGCTATCAC 1980

NC_042820.1 AGTAGGAATCACAGCTTTGCTTTTATTATTATCTCTTCCGGTTCTAGCTGGAGCTATCAC 1980

****** *****************************************************

Rdo_MitoGenome TATATTATTAACAGACCGAAACTTAAATACTTCATTTTTTGATCCAGCAGGAGGAGGGGA 2040

NC_042820.1 TATATTATTAACAGACCGAAACTTAAATACTTCATTTTTTGATCCAGCAGGAGGAGGGGA 2040

************************************************************

Rdo_MitoGenome CCCTATTTTGTATCAACACCTATTCTGATTTTTTGGACATCCAGAAGTTTACATTTTAAT 2100

NC_042820.1 CCCTATTTTGTATCAACACCTATTCTGATTTTTTGGACATCCAGAAGTTTACATTTTAAT 2100

************************************************************

Rdo_MitoGenome CCTACCAGGATTTGGTATAATTTCTCATATTATTAGACATGAAAGAGGAAAAAAGGAAAC 2160

NC_042820.1 CCTACCAGGATTTGGTATAATTTCTCATATTATTAGACATGAAAGAGGAAAAAAGGAAAC 2160

************************************************************

Rdo_MitoGenome CTTTGGTTCTCTAGGGATAATTTACGCAATAATAGCAATTGGACTATTAGGATTTATTGT 2220

NC_042820.1 CTTTGGTTCTCTAGGGATAATTTACGCAATAATAGCAATTGGACTATTAGGATTTATTGT 2220

************************************************************

Rdo_MitoGenome ATGAGCACATCACATATTTACTGTAGGAATAGACGTAGATACCCGAGCATATTTTACTTC 2280

NC_042820.1 ATGAGCACATCACATATTTACTGTAGGAATAGACGTAGATACCCGAGCATATTTTACTTC 2280

************************************************************

Rdo_MitoGenome AGCAACAATAATTATTGCAGTTCCAACCGGAATTAAAGTATTTAGATGATTAGCTACCCT 2340

NC_042820.1 AGCAACAATAATTATTGCAGTTCCAACCGGAATTAAAGTATTTAGATGATTAGCTACCCT 2340

************************************************************

Rdo_MitoGenome CCACGGAACACAAATAAATTATTCACCTTCAATAATATGATCATTAGGATTTGTCTTTCT 2400

NC_042820.1 CCACGGAACACAAATAAATTATTCACCTTCAATAATATGATCATTAGGATTTGTCTTTCT 2400

************************************************************

Rdo_MitoGenome ATTTACCGTAGGAGGATTAACAGGAGTAGTATTAGCAAATTCATCCATTGATATTATTCT 2460

NC_042820.1 ATTTACCGTAGGAGGATTAACAGGAGTAGTATTAGCAAATTCATCCATTGATATTATTCT 2460

************************************************************

Rdo_MitoGenome ACACGATACATATTATGTAGTAGCCCACTTCCACTACGTGCTATCCATAGGAGCAGTATT 2520

NC_042820.1 ACACGATACATATTATGTAGTAGCCCACTTCCACTACGTGCTATCCATAGGAGCAGTATT 2520

************************************************************

Rdo_MitoGenome TGCAATTATAGCAGGAATTATTCAATGATTCCCACTATTTACAGGATTAACACTTAATGA 2580

NC_042820.1 TGCAATTATAGCAGGAATTATTCAATGATTCCCACTATTTACAGGATTAACACTTAATGA 2580

************************************************************

Rdo_MitoGenome AAAAATACTTAAAATCCAATTCCTTATTATATTTATAGGAGTAAATATTACATTCTTCCC 2640

NC_042820.1 AAAAATACTTAAAATCCAATTCCTTATTATATTTATAGGAGTAAATATTACATTCTTCCC 2640

************************************************************

Rdo_MitoGenome TCAACATTTTTTAGGATTAAGAGGTATACCACGACGATACTCAGATTATCCAGATGCATA 2700

NC_042820.1 TCAACATTTTTTAGGATTAAGAGGTATACCACGACGATACTCAGATTATCCAGATGCATA 2700

************************************************************

Rdo_MitoGenome TCTCACATGAAATATTATTTCATCAATCGGATCAATAATAAGAACAGTAAGAATTATTTT 2760

NC_042820.1 TCTCACATGAAATATTATTTCATCAATCGGATCAATAATAAGAACAGTAAGAATTATTTT 2760

************************************************************

Rdo_MitoGenome TATGTCATTCATCATATGAGAATCATTATCATCTAAACGAAAGAATATCAGAAATAACCA 2820

NC_042820.1 TATGTCATTCATCATATGAGAATCATTATCATCTAAACGAAAGAATATCAGAAATAACCA 2820

************************************************************

Rdo_MitoGenome ATTATCATCAGCAATTGAATGACTTCAAAATTCACCCCCAGAAGAACACAGATACTCAGA 2880

NC_042820.1 ATTATCATCAGCAATTGAATGACTTCAAAATTCACCCCCAGCAGAACACAGATACTCAGA 2880

***************************************** ******************

**End cox1 (+)**

**Start trnL2 (+)**

**trnL2 (+)**

Rdo_MitoGenome ACTTCCTATCCTAGCTAAATTCTAATATGGCAGAATAGTGCAATGAATTTAAGATTCATA 2940

NC_042820.1 ACTTCCTATCCTAGCTAAATTCTAATATGGCAGAATAGTGCAATGAATTTAAGATTCATA 2940

************************************************************

**Start cox2 (+)**

**End trnL2 (+)**

Rdo_MitoGenome TATAAGTTAAACTTTTTTAGAAATAGCAACATGAAAAATAACATCAATGAGAAATAGAAA 3000

NC_042820.1 TATAAGTTAAACTTTTTTAGAAATAGCAACATGAAAAATAACATCAATGAGAAATAGAAA 3000

************************************************************

Rdo_MitoGenome TTCCCCGGCAATAGAACAATTAACCTTCTTTCATGACCACACAATAACAGTTTTAATTAT 3060

NC_042820.1 TTCCCCGGCAATAGAACAATTAACCTTCTTTCATGACCACACAATAACAGTTTTAATTAT 3060

************************************************************

Rdo_MitoGenome AATTACAATCCTAGTAGGATACCTAATATCAACACTATTTACAAATAAGTTAACATATCG 3120

NC_042820.1 AATTACAATCCTAGTAGGATACCTAATATCAACACTATTTACAAATAAGTTAACATATCG 3120

************************************************************

Rdo_MitoGenome GTTCCTATTAGAAGGACAAACAATTGAAATAATTTGAACTATTCTACCAGCAATTACATT 3180

NC_042820.1 GTTCCTATTAGAAGGACAAACAATTGAAATAATTTGAACTATTCTACCAGCAATTACATT 3180

************************************************************

Rdo_MitoGenome AATTTTTATTGCCTTACCCTCACTTCAAATTCTTTACACACTAGATGAAATTATTAACCC 3240

NC_042820.1 AATTTTTATTGCCTTACCCTCACTTCAGATTCTTTACACACTAGATGAAATTATTAACCC 3240

*************************** ********************************

Rdo_MitoGenome ATCCATATCAGTTAAATCTATTGGACATCAATGATACTGAAGATATGAATATTCAGACTT 3300

NC_042820.1 ATCCATATCAGTTAAATCTATTGGACATCAATGATACTGAAGATATGAATATTCAGACTT 3300

************************************************************

**cox2 (+)**

Rdo_MitoGenome CAAAAAAACCGAATTCGATTCATATATAAAATCACCAAACGAAATCAAAAATCAAGAATT 3360

NC_042820.1 CAAAAAAACCGAATTCGATTCATATATAAAATCACCAAACGAAATCAAAAATCAAGAATT 3360

************************************************************

Rdo_MitoGenome CCGATTACTAGATGTAGACAATCGAATAACCTTACCAATAAAGACAAAAATTCGATTATT 3420

NC_042820.1 CCGATTACTAGATGTAGACAATCGAATAACCTTACCAATAAAGACAAAAATTCGATTATT 3420

************************************************************

Rdo_MitoGenome AGTTTCGTCAACAGACGTAATTCACTCATGAACAATTCCATCCCTTGGAGTAAAGATTGA 3480

NC_042820.1 AGTTTCGTCAACAGACGTAATTCACTCATGAACAATTCCATCCCTTGGAGTAAAGATTGA 3480

************************************************************

Rdo_MitoGenome TGCAACACCAGGGCGTCTAAACCAAGCAAGAATATTCATCATTAATCCAGGACTAATATA 3540

NC_042820.1 TGCAACACCAGGGCGTCTAAACCAAGCAAGAATATTCATCATTAATCCAGGACTAATATA 3540

************************************************************

Rdo_MitoGenome CGGACAATGTTCAGAAATCTGTGGAGCAAATCATAGATTTATACCAATTGTTGTAGAAAG 3600

NC_042820.1 CGGACAATGTTCAGAAATCTGTGGAGCAAATCATAGATTTATACCAATTGTTGTAGAAAG 3600

************************************************************

**Start trnK (+)**

**End cox2 (+)**

Rdo_MitoGenome AATTACTCCTAATAAATTTATTGAGTGAATTAAAAACTCATTAGATAACTAAAAGTTAGT 3660

NC_042820.1 AATTACTCCTAATAAATTTATTGAGTGAATTAAAAACTCATTAGATAACTAAAAGTTAGT 3660

************************************************************

**Start trnD (+)**

**End trnK (+)**

**trnK (+)**

Rdo_MitoGenome AATGGTCTCTTAAACCAAAAAATAGTAGAGTAACGACTACTTCTAATGAAAAATTTAGTT 3720

NC_042820.1 AATGGTCTCTTAAACCAAAAAATAGTAGAGTAACGACTACTTCTAATGAAAAATTTAGTT 3720

************************************************************

**Start atp8 (+)**

**End trnD (+)**

**trnD (+)**

Rdo_MitoGenome AAAAAATAACATCAACTTGTCAAGTTGAAATTACGATTTCGTAATTTTTGATTCCACAAA 3780

NC_042820.1 AAAAAATAACATCAACTTGTCAAGTTGAAATTACGATTTCGTAATTTTTGATTCCACAAA 3780

************************************************************

Rdo_MitoGenome TATCTCCATTAAATTGAATAACCTTATTTATTTTCTTTACAATTACTTTAGTAATCATAT 3840

NC_042820.1 TATCTCCATTAAATTGAACAACCTTATTTATTTTCTTTACAATTACTTTAGTAATCATAT 3840

****************** *****************************************

**atp8 (+)**

Rdo_MitoGenome CAACAATAAATTTTTATAATTACAAACCTGAACCCCTCAAAGGAGAAAAAACAATTTCTA 3900

NC_042820.1 CAACAATAAATTTTTATAATTACAAACCTGAACCCCTCAAAGGAGAAAAAACAATTTCTA 3900

************************************************************

**Start atp6 (+)**

**End atp8 (+)**

Rdo_MitoGenome TAAGAAAAAAAAACTGAAAATGATAAGAAATCTATTTTCATCATTTGATCCATCTACTCA 3960

NC_042820.1 TAAGAAAAAAAAACTGAAAATGATAAGAAATCTATTTTCATCATTTGATCCATCTACTCA 3960

************************************************************

Rdo_MitoGenome AATTCTCTCCTTAAATTGATTAAGATCATTAATTATCCTTATAATTATACCAATAAATTT 4020

NC_042820.1 AATTCTCTCCTTAAATTGATTAAGATCATTAATTATCCTTATAATTATACCAATAAATTT 4020

************************************************************

Rdo_MitoGenome AATTCTCTCCTTAAATTGATTAAGATCATTAATTATCCTTATAATTATACCAATAAATTT 4080

NC_042820.1 TTGATTAATCCCGTCACGTATATCTTTATTATGAATAAAAATTTCAAAAAAACTTCATAA 4080

************************************************************

Rdo_MitoGenome CGAATTTAAAATCTTAATTGGAAAAAACAACGGATCATCATTAATGTTCACCTCCCTACT 4140

NC_042820.1 CGAATTTAAAATCTTAATTGGAAAAAACAACGGATCATCATTAATGTTCACCTCCCTACT 4140

************************************************************

Rdo_MitoGenome ACTTCTAATTATAATTAATAATTTTATAGGACTTTTCCCGTACATCTTTACAAGAACAAG 4200

NC_042820.1 ACTTCTAATTATAATTAATAATTTTATAGGACTTTTCCCGTACATCTTTACAAGAACAAG 4200

************************************************************

Rdo_MitoGenome ACATTTAACAATAACTTTAACCTTAGCTTTACCATTATGAACAAGATTTATAATTTACGG 4260

NC_042820.1 ACATTTAACAATAACTTTAACCTTAGCTTTACCATTATGAACAAGATTTATAATTTACGG 4260

************************************************************

**atp6 (+)**

Rdo_MitoGenome ATGATTTAATAATACAATCTCTATACTCGCACACTTAGTCCCTCAAGGAACACCTCCCAT 4320

NC_042820.1 ATGATTTAATAATACAATCTCTATACTCGCACACTTAGTCCCTCAAGGAACACCTCCCAT 4320

************************************************************

Rdo_MitoGenome TTTAATACCTTTTATAGTATGTATTGAAACAATTAGAAATATTATTCGACCAGGCACATT 4380

NC_042820.1 TTTAATACCTTTTATAGTATGTATTGAAACAATTAGAAATATTATTCGACCAGGCGCATT 4380

******************************************************* ****

Rdo_MitoGenome AGCAATTCGTTTAACAGCAAATATAATTGCAGGACACTTACTTTTAACCCTTCTAGGTAA 4440

NC_042820.1 AGCAATTCGTTTAACAGCAAATATAATTGCAGGACACTTACTTTTAACCCTTCTAGGTAA 4440

************************************************************

Rdo_MitoGenome TACAGGAGCAGGAATTTCATCAATTCTTGTAAGAATCTTAATTTTAACTCAAATTTTACT 4500

NC_042820.1 TACAGGAGCAGGAATTTCATCAATTCTTGTAAGAATCTTAATTTTAACTCAAATTTTACT 4500

************************************************************

Rdo_MitoGenome TTTAGTTCTAGAATCAGCAGTAGCAATTATTCAATCATACGTATTTGCAATTTTAAGAAC 4560

NC_042820.1 TTTAGTTCTAGAATCAGCAGTAGCAATTATTCAATCATACGTATTTGCAATTTTAAGAAC 4560

************************************************************

**End atp6 (+)**

**Start cox3 (+)**

Rdo_MitoGenome CCTTTATTCAAGAGAAGTAAACTAATGACAAAAAAAAATCACCCTTTTCACTTAGTAGAT 4620

NC_042820.1 CCTTTATTCAAGAGAAGTAAACTAATGACAAAAAAAAATCACCCTTTTCACTTAGTAGAT 4620

************************************************************

Rdo_MitoGenome GTAAGACCATGACCAATTCTTGGAGCATTAAGAGCCATATCCACAATAGTAGGATTAATT 4680

NC_042820.1 GTAAGACCATGACCAATTCTTGGAGCATTAAGAGCCATATCCACAATAGTAGGATTAATT 4680

************************************************************

Rdo_MitoGenome AAATGATTTCATATATATCAAGTAAACCTTTTTTTGGTAGGTTTACTTTCTACATCTCTT 4740

NC_042820.1 AAATGATTTCATATATATCAAGTAAACCTTTTTTTGGTAGGTTTACTTTCTACATCTCTT 4740

************************************************************

Rdo_MitoGenome ATTATATATCAATGATGACGAGATATCACACGTGAAGGTTCATTTCAAGGACACCACACA 4800

NC_042820.1 ATTATATATCAATGATGACGAGATATCACACGTGAAGGTTCATTTCAAGGACACCACACA 4800

************************************************************

Rdo_MitoGenome TTTATCGTTACTATAGGATTACGATGAGGAATAATTTTATTTATTACATCAGAAGTATTT 4860

NC_042820.1 TTTATCGTTACTATAGGATTACGATGAGGAATAATTTTATTTATTACATCAGAAGTATTT 4860

************************************************************

**cox3 (+)**

Rdo_MitoGenome TTCTTCATTTCATTTTTTTGAGGATTTTTCCACAGAAGATTATCTCCATCAATTGAAATT 4920

NC_042820.1 TTCTTCATTTCATTTTTTTGAGGATTTTTCCACAGAAGATTATCTCCATCAATTGAAATT 4920

************************************************************

Rdo_MitoGenome GGGATAAACTGACCACCTTTGGGAATTTTAACCTTCAATCCTTTAAGAATTCCTCTTCTT 4980

NC_042820.1 GGGATAAACTGACCACCTTTGGGAATTTTAACCTTCAATCCTTTAAGAATTCCTCTTCTT 4980

************************************************************

Rdo_MitoGenome AATACACTAATTCTTCTTACTAGAGGACTTACAGTAACATGAGCACATCATAGATTAATA 5040

NC_042820.1 AATACACTAATTCTTCTTACTAGAGGACTTACAGTAACATGAGCACATCATAGATTAATA 5040

************************************************************

Rdo_MitoGenome GAAAATAACTGAAAACAAGCAAATCAAGGACTAACACTAACAATCATTCTTGGTTTATAT 5100

NC_042820.1 GAAAATAACTGAAAACAAGCAAATCAAGGACTAACACTAACAATCATTCTTGGTTTATAT 5100

************************************************************

Rdo_MitoGenome TTTACTATTCTTCAAGCTTACGAATATATTGAAGCACCATTCACTATCTCCGATTCCGTT 5160

NC_042820.1 TTTACTATTCTTCAAGCTTACGAATATATTGAAGCACCATTCACTATCTCCGATTCCGTT 5160

************************************************************

Rdo_MitoGenome TACGGATCAAGTTTCTTTATAGCAACAGGATTCCATGGACTACATGTGATTATTGGAACA 5220

NC_042820.1 TACGGATCAAGTTTCTTTATAGCAACAGGATTCCATGGACTACATGTGATTATTGGAACA 5220

************************************************************

Rdo_MitoGenome ACATTTCTTAGAGTTTGCCTTTTACGTCATCTAATAAATCATTTCTCAATAATTCACCAC 5280

NC_042820.1 ACATTTCTTAGAGTTTGCCTTTTACGTCATCTAATAAATCATTTCTCAATAATTCACCAC 5280

************************************************************

Rdo_MitoGenome TTCGGATTTGAAGCAGCAGCCTGATACTGACACTTCGTAGACGTAGTTTGGCTATTCTTA 5340

NC_042820.1 TTCGGATTTGAAGCAGCAGCCTGATACTGACACTTCGTAGACGTAGTTTGGCTATTCTTA 5340

************************************************************

**Start trnG (+)**

**End cox3 (+)**

**trnG (+)**

Rdo_MitoGenome TATTTATCAATTTACTGATGAGGTAAATATTTATATAGTATAAAAATTATTTTTAACTTC 5400

NC_042820.1 TATTTATCAATTTACTGATGAGGTAGATATTTATATAGTATAAAAATTATTTTTAACTTC 5400

************************* **********************************

**End trnG (+)**

**Start nad3 (+)**

Rdo_MitoGenome CAATTAAAAGATCTAGAAATAGTATAAATAATAAAAATTATTTTAATTTCAAGAATAATT 5460

NC_042820.1 CAATTAAAAGATCTAGAAATAGTATAAATAATAAAAATTATTTTAATTTCAAGAATAATT 5460

************************************************************

Rdo_MitoGenome ATCTTTTCAATCAGATTAATCTTAATGATAATAAATCAAGTAATCTCAAAAAAAACATTT 5520

NC_042820.1 ATCTTTTCAATCAGATTAATCTTAATGATAATAAATCAAGTAATCTCAAAAAAAACATTT 5520

************************************************************

Rdo_MitoGenome AAAGACCGAGAAAAAATATCCCCATATGAATGTGGGTTTGACCCAAAATCACACGCCCGT 5580

NC_042820.1 AAAGACCGAGAAAAAATATCCCCATATGAATGTGGGTTTGACCCAAAATCACACGCCCGT 5580

************************************************************

**nad3 (+)**

Rdo_MitoGenome ATTCCATTATCAATCCGATTTTTTCTTATCACAATAATCTTTCTAATTTTTGATGTAGAA 5640

NC_042820.1 ATTCCATTATCAATCCGATTTTTTCTTATTACAGTAATCTTTCTAATTTTTGATGTAGAA 5640

***************************** *** **************************

Rdo_MitoGenome ATTACTCTTCTTCTTCCAGCAATTAATAACCTTAAAACTACAAATCCACTGGAATTCCTA 5700

NC_042820.1 ATTACTCTTCTTCTTCCGGCAATTAATAACCTTAAAACTACAAATCCACTGGAATTCCTA 5700

***************** ******************************************

Rdo_MitoGenome ATTACATTTATTTTCTTCATCTCAATTTTAACGTTAGGGACAATTCATGAATGAAAACAA 5760

NC_042820.1 ATTACATTTATTTTCTTCATCTCAATTTTAACGTTAGGGACAATTCATGAATGAAAACAA 5760

************************************************************

**End nad3 (+)**

**Start trnA (+)**

**trnA (+)**

Rdo_MitoGenome GGAGCATTGAATTGAAAAAACTAGGATAATAGTTTAAAAAACATTTAATTTGCATTTAAA 5820

NC_042820.1 GGAGCATTGAATTGAAAAAACTAGGATAATAGTTTAAAAAACATTTAATTTGCATTTAAA 5820

************************************************************

**Start trnR (+)**

**End trnA (+)**

**trnR (+)**

Rdo_MitoGenome AGAAGTTAAAATTAACTTATCTTAAATAAGAAACAATAAATTGTATTTAGTTTCGACCTA 5880

NC_042820.1 AGAAGTTAAAATTAACTTATCTTAAATAAGAAACAATAAATTGTATTTAGTTTCGACCTA 5880

************************************************************

**Start trnN (+)**

**End trnR (+)**

Rdo_MitoGenome AAATTTTGATGATAAACATCCTTATTTTTAATTGAAACCAAAATAGAGGTGTACCACTGT 5940

NC_042820.1 AAATTTTGATGATAAACATCCTTATTTTTAATTGAAACCAAAATAGAGGTGTACCACTGT 5940

************************************************************

**Start trnS (+)**

**End trnN (+)**

**trnN (+)**

Rdo_MitoGenome TAATGGTAAAATTGATTTAAATCCAATTAAGAAATATGATAATCAAGAAGAAGCTTCTAA 6000

NC_042820.1 TAATGGTAAAATTGATTTAAATCCAATTAAGAAATATGATAATCAAGAAGAAGCTTCTAA 6000

************************************************************

**Start trnE (+)**

**End trnS (+)**

**trnS (+)**

Rdo_MitoGenome CTTCAATCTTTAGCGGTGAAAATCCGTTAATATTTCTATTTATATAGTTTAAGAAAAACA 6060

NC_042820.1 CTTCAATCTTTAGCGGTGAAAATCCGTTAATATTTCTATTTATATAGTTTAAGAAAAACA 6060

************************************************************

**End of trnF (-)**

**End of trnE (+)**

**trnE (+)**

Rdo_MitoGenome TTACATTTTCAATGTAAAATTAATATTAATATTTATAAATACTTAAAAACACAAGTGTTA 6120

NC_042820.1 TTACATTTTCAATGTAAAATTAATATTAATATTTATAAATACTTAAAAACACAAGTGTTA 6120

************************************************************

**End nad5 (-)**

**Start trnF (-)**

**trnF (-)**

Rdo_MitoGenome CCTTAATATCTTCAATATTAAACTCTTTTTAAGCTATTTAAGTAAAACAAAATAAGAAAA 6180

NC_042820.1 CCTTAATATCTTCAATATTAAACTCTTTTTAAGCTATTTAAGTAAAACAAAATAAGAAAA 6180

************************************************************

Rdo_MitoGenome GAAACCCAAAAAATTAACATAATTAAAAATAACTTTAATTGATTAGAAAAAATTAAATGT 6240

NC_042820.1 GAAACCCAAAAAATTAACATAATTAAAAATAACTTTAATTGATTAGAAAAAATTAAATGT 6240

************************************************************

Rdo_MitoGenome AAACTTCGAGTTAGCTTTAAAATATTTATTATAAAACCCTGGGCCCCTAAATGTTCAAGT 6300

NC_042820.1 AAACTTCGAGTTAGCTTTAAAATATTTATTATAAAACCCTGGGCCCCTAAATGTTCAAGT 6300

************************************************************

Rdo_MitoGenome CAACCTTGATCAAAAGAAGAAATAAATAATCGGCCAAGTGTTAAAGGAAAAATTCTTATC 6360

NC_042820.1 CAACCTTGATCAAAAGAAGAAATAAATAATCGGCCAAGTGTTAAAGGAAAAATTCTTATC 6360

************************************************************

Rdo_MitoGenome CCTAAAGAAGAAATTAAAGGCAAGCCTCATATTAACGAAGAAAACCAAGAAAAATTTATA 6420

NC_042820.1 CCTAAAGAAGAAATTAAAGGCAAGCCTCATATTAACGAAGAAAACCAAGAAAAATTTATA 6420

************************************************************

Rdo_MitoGenome AAATTTAAAGATTTAGAATGATAATTTAAAGAAAAACGTGAAACTTCTACACCAATTAAA 6480

NC_042820.1 AAATTTAAAGATTTAGAATGATAATTTAAAGAAAAACGTGAAACTTCTACACCAATTAAA 6480

************************************************************

Rdo_MitoGenome AGGCCAGAAAAAATTACAAATAAAGTTATAAATTTTATATACAAAGGTAAACAAATATAA 6540

NC_042820.1 AGGCCAGAAAAAATTACAAATAAAGTTATAAATTTTATATACAAAGGTAAACAAATATAA 6540

************************************************************

Rdo_MitoGenome TAAGGAGTAGAAAATATTAACCAAGATAAAACTGAACCAGCAACAATTACAGGAAAAATC 6600

NC_042820.1 TAAGGAGTAGAAAATATTAACCAAGATAAAACTGAACCAGCAACAATTACAGGAAAAATC 6600

************************************************************

Rdo_MitoGenome AAGCCTAATATACCAAAAAGTATAAAATTTCCACCCTCAGAAATTAAGAATAATGGAGAA 6660

NC_042820.1 AAGCCTAATATACCAAAAAGTATAAAATTTCCACCCTCAGAAATTAAGAATAATGGAGAA 6660

************************************************************

Rdo_MitoGenome AAATTTAAATAACCAAAAAATAAATAATATATTAAACGTACAGTATAAGAAACTGTTAAA 6720

NC_042820.1 AAATTTAAATAACCAAAAAATAAATAATATATTAAACGTACAGTATAAGAAACTGTTAAA 6720

************************************************************

Rdo_MitoGenome CCTGTAGAGACAAAAAATAAAATATAAGAAAACATATTAACAAATTTTATTGAATAAAAC 6780

NC_042820.1 CCTGTAGAGACAAAAAATAAAATATAAGAAAACATATTAACAAATTTTATTGAATAAAAC 6780

************************************************************

Rdo_MitoGenome TCCAAAATTAAATCCTTTGAATAAAAACCTGAAAGAAAAGGTAAACCACAAAGAGAAAAA 6840

NC_042820.1 TCCGAAATTAAATCCTTTGAATAAAAACCTGAAAGAAAAGGTAAACCACAAAGAGAAAAA 6840

*** ********************************************************

**nad5 (-)**

Rdo_MitoGenome TTACAAATAATAAAAAAACAAGAAGTTAAAGGTATAAAGTTTAAAGCACTTCCTATAAAA 6900

NC_042820.1 TTACAAATAATAAAAAAACAAGAAGTTAAAGGTATAAAGTTTAAAGCACTTCCTATAAAA 6900

************************************************************

Rdo_MitoGenome CGAATATCCTGAAAATTTATAAATCTATGAATAAATGAACCAGCACATATAAACAATAAA 6960

NC_042820.1 CGAATATCCTGAAAATTTATAAATCTATGAATAAATGAACCAGCACATATAAACAATAAA 6960

************************************************************

Rdo_MitoGenome GCCTTGAAAAGAGCATGAGATAAAAGATGAAAAAAAGCTAAATATTCACCACCTCTAAAT 7020

NC_042820.1 GCCTTGAAAAGAGCATGAGATAAAAGATGAAAAAAAGCTAAATATTCACCACCTCTAAAT 7020

************************************************************

Rdo_MitoGenome AAAATTCTTATTATCAATCCTAATTGACTAAGAGTAGAAAGAGCAATGATTTTCTTTAAA 7080

NC_042820.1 AAAATTCTTATTATCAATCCTAATTGACTAAGAGTAGAAAGAGCAATGATTTTCTTTAAA 7080

************************************************************

Rdo_MitoGenome TCAAATTCAAAATTAGCTCCAAGCCCAGCTATAAATATAGTTAAACAAGAAAGCAATAAT 7140

NC_042820.1 TCAAATTCAAAATTAGCTCCAAGCCCAGCTATAAATATAGTTAAACAAGAAAGCAATAAT 7140

************************************************************

Rdo_MitoGenome CCTAAAATTAAAACTTTATCACAAAAACAATAACTAAAACGAATTATTAAGTAAACCCCA 7200

NC_042820.1 CCTAAAATTAAAACTTTATCACAAAAACAATAACTAAAACGAATTATTAAGTAAACCCCA 7200

************************************************************

Rdo_MitoGenome GCTGTTACTAAAGTAGAAGAATGAACCAATGAAGATACAGGTGTAGGAGCTGCTATAGCA 7260

NC_042820.1 GCTGTTACTAAAGTAGAAGAATGAACCAATGAAGATACAGGTGTAGGAGCTGCTATAGCA 7260

************************************************************

Rdo_MitoGenome GCAGGTAACCAAGCAGAAAAAGGAATTTGAGCTCTTTTAGTAAAAGCCGCTAATATAATA 7320

NC_042820.1 GCAGGTAACCAAGCAGAAAAAGGAATTTGAGCTCTTTTAGTAAAAGCCGCTAATATAATA 7320

************************************************************

Rdo_MitoGenome AATAAACTAATAATAAATAAATAATTATCATCAATAAAAAATACATAAAATAAATAATTT 7380

NC_042820.1 AATAAACTAATAATAAATAAATAATTATCATCAATAAAAAATACATAAAATAAATAATTT 7380

************************************************************

Rdo_MitoGenome CACCTACCATAATTTAATATTCAAGCAATTCTTATTAACAAAGCAACATCCCCAATCCGA 7440

NC_042820.1 CACCTACCATAATTTAATATTCAAGCAATCCTTATTAACAAAGCAACATCCCCAATCCGA 7440

***************************** ******************************

Rdo_MitoGenome TTTCTTAAAGCAGTTAATATACCCGCACTAAAAGACTTAAAATTTTGATAATAAATAACT 7500

NC_042820.1 TTTCTTAAAGCAGTTAATATACCCGCACTAAAAGACTTAAAATTTTGATAATAAATAACT 7500

************************************************************

Rdo_MitoGenome AAACTATAAGAAACAAGACCTAAACCATCCCAACCTAAAAGAATTCTAATAATATTAGGA 7560

NC_042820.1 AAACTATAAGAAACAAGACCTAAACCATCCCAACCTAAAAGAATTCTAATAATATTAGGA 7560

************************************************************

Rdo_MitoGenome GATAAAATTATTATTATTATAGATAAAACAAATAAAACCACTAATAAAATAAAACGATTT 7620

NC_042820.1 GATAAAATTATTATTATTATAGATAAAACAAATAAAACCACTAATAAAATAAAACGATTT 7620

************************************************************

Rdo_MitoGenome AATATTAAATCCCCGTGTATATAATCCTCTCTATAAAAAACTACTATAGAAGAAATAAAA 7680

NC_042820.1 AATATTAAATCCCCGTGTATATAATCCTCTCTATAAAAAACTACTATAGAAGAAATAAAA 7680

************************************************************

Rdo_MitoGenome AAAACAAACCTTATAAAAATTAAACTTATAAAATCAAAATATAAAGAAAAATTAATTAAA 7740

NC_042820.1 AAAACAAACCTTATAAAAATTAAACTTATAAAATCAAAATATAAAGAAAAATTAATTAAA 7740

************************************************************

Rdo_MitoGenome GAAGAATTTAAAGAAAACAATTCATATTCAACAAATAATGAAAAATCAAGGATTATAAAA 7800

NC_042820.1 GAAGAATTTAAAGAAAACAATTCATATTCAACAAATAATGAAAAATCAAGGATTATAAAA 7800

************************************************************

Rdo_MitoGenome TAAAGAGATAAAAAGAAAAAAAAAAATGAACAAAATAAAAATAATGAAAAAAATACAAAA 7860

NC_042820.1 TAAAGAGATAAAAAGAAAAAAAAAAATGAACAAAATAAAAATAATGAAAAAAATACAAAA 7860

**End trnH (-)**

************************************************************

**Start nad5 (-)**

**trnH (-)**

Rdo_MitoGenome GTTAAAGAAATAACTCAAGATCCATAGATATCTTTGTTACCACAAAACAAAATTTTTTAT 7920

NC_042820.1 GTTAAAGAAATAACTCAAGATCCATAGATATCTTTGTTACCACAAAACAAAATTTTTTAT 7920

************************************************************

**End Nad4 (-)**

**Start trnH (-)**

Rdo_MitoGenome TAAAATACTTGAATATAAACTAAATAAATTATCTATAAATAAAAAAATTAAATTCAAAGG 7980

NC_042820.1 TAAAATACTTGAATATAAACTAAATAAATTATGTATAAATAAAAAAATTAAATTCAAAGG 7980

******************************** ***************************

Rdo_MitoGenome AATTCAATGAAAAAAAAGTAATAAATATTCACGAACATTAACAGATCAAAAACTAAATAA 8040

NC_042820.1 AATTCAATGACAAAAAAGTAATAAATATTCACGAACATTAACAGATCAAAAACTAAATAA 8040

********** *************************************************

Rdo_MitoGenome ACCCCTACTTACCATGCCATGCTGAGTGAAAGAATACAAAAATAATGAATAAGCAGCTCT 8100

NC_042820.1 ACCCCTACTTACCATGCCATGCTGAGTGAAAGAATACAAAAATAATGAATAAGCAGCTCT 8100

************************************************************

Rdo_MitoGenome AAGAAAAGAAAGAAAAGATAAAATTAAAATTATTAATCTTCTTCAACTAACTAAAGAACT 8160

NC_042820.1 AAGAAAAGAAAGAAAAGATAAAATTAAAATTATTAATCTTCTTCAACTAACTAAAGAACT 8160

************************************************************

Rdo_MitoGenome TAAAATTAAAATTTCAGAAACAAGATTTAAAGAAGGTGGAGCAGCTATATTTGATGAACA 8220

NC_042820.1 TAAAATTAAAATTTCAGAAACAAGATTTAAAGAAGGTGGAGCAGCTATATTTGATGAACA 8220

************************************************************

Rdo_MitoGenome TAAAAGAAATCACCATAAACTCAAGTTAGGTAAAATATTAATATAACCTTTGTTTAAATA 8280

NC_042820.1 TAAAAGAAATCACCATAAACTCAAGTTAGGTAAAATATTAATATAACCTTTGTTTAAATA 8280

************************************************************

Rdo_MitoGenome CAAACTACGTCTCAAAGACCGTTCATAAGTAATATTAGCCAGACAAAATAAACCAGAAGA 8340

NC_042820.1 CAAACTACGTCTCAAAGACCGTTCATAAGTAATATTAGCCAGACAAAATAAACCAGAAGA 8340

************************************************************

Rdo_MitoGenome ACAAATACCATGAGAAATTATTATAATTAAAGATCCAATAACCCCTCAAGTTGTTAAAGT 8400

NC_042820.1 ACAAATACCATGAGAAATTATTATAATTAAAGATCCAATAACCCCTCAAGTTGTTAAAGT 8400

************************************************************

Rdo_MitoGenome AAAGCACCCAGAAATTACTATACTTATATGAGCTACTGAAGAATAAGCAATTAATGATTT 8460

NC_042820.1 AAAGCACCCAGAAATTACTATACTTATATGAGCTACTGAAGAATAAGCAATTAATGATTT 8460

************************************************************

Rdo_MitoGenome AATATCTCTTTGACGTAAACAAATTAAAGAAACAATTAAACCACCAAAAACAGAAATTCT 8520

NC_042820.1 AATATCTCTTTGACGTAAACAAATTAAAGAAACAATTAAACCACCAAAAACAGAAATTCT 8520

************************************************************

Rdo_MitoGenome TACAAAAAAAGTTCTAAAAAAGGAAACCTCCGCCTTAAATAAAGGAAACACACGCAATAA 8580

NC_042820.1 TACAAAAAAAGTTCTAAAAAAGGAAACCTCCGCCTTAAATAAAGGAAACACACGCAATAA 8580

************************************************************

Rdo_MitoGenome ACCATAACCTCCCAATTTTAATATAACTCCAGCCAAAATTATAGAACCAGAAACAGGAGC 8640

NC_042820.1 ACCATATCCTCCCAATTTTAATATAACTCCAGCCAAAATTATAGAACCAGAAACAGGAGC 8640

****** *****************************************************

Rdo_MitoGenome CTCTACATGAGCCTTAGGTAATCACAAATGAACAACAAATATAGGAATTTTTACCAAAAA 8700

NC_042820.1 CTCTACATGAGCCTTAGGTAATCACAAATGAACAACAAATATAGGAATTTTTACCAAAAA 8700

************************************************************

Rdo_MitoGenome AACAAAAATTATTATATTAAAAAAAATTATTCTGTTAACTTGAAATATTAAAAATATAGA 8760

NC_042820.1 AACAAAAATTATTATATTAAAAAAAATTATTCTGTTAACTTGAAATATTAAAAATATAGA 8760

************************************************************

**nad4 (-)**

Rdo_MitoGenome TAAAGAACCGAAACTTAAATAATAATGTATTAACCCCAAAAATATAGGTAAAGAAGCAAA 8820

NC_042820.1 TAAAGAACCGAAACTTAAATAATAATGTATTAACCCCAAAAATATAGGTAAAGAAGCAAA 8820

************************************************************

Rdo_MitoGenome AAGAGTATAAAACAACAAATAAAAACCAGCCTGGATTCGTTCTGGTTGATATCCTCAACC 8880

NC_042820.1 AAGAGTATAAAACAACAAATAAAAACCAGCCTGGATTCGTTCTGGTTGATATCCTCAACC 8880

************************************************************

Rdo_MitoGenome CAAAATTAAAAATAAAGTAGGAATTAAACTAGACTCAAAAAATACATAAAATAAAATAAA 8940

NC_042820.1 CAAAATTAAAAATAAAGTAGGAATTAAACTAGACTCAAAAAATACATAAAATAAAATAAA 8940

************************************************************

Rdo_MitoGenome GTTTAAAGAACTAAAAGCGCAAATTAAAAAAAACAAAAGTATAATAACATTTAAAGAAAA 9000

NC_042820.1 GTTTAAAGAACTAAAAGCGCAAATTAAAAAAAACAAAAGTATAATAACATTTAAAGAAAA 9000

************************************************************

Rdo_MitoGenome ATAAAAATGAAAATTATTGTCCATATAAATTTTAGACCTAGCAATAAATATCAACAAACA 9060

NC_042820.1 ATAAAAATGAAAATTATTGTCCATATAAATTTTAGACCTAGCAATAAATATCAACAAACA 9060

************************************************************

Rdo_MitoGenome AATAAAAAATCTTAACAAAATAAGAGGGCCAGAAAACAAATCACAACCGAAGCCTAAACC 9120

NC_042820.1 AATAAAAAATCTTAACAAAATAAGAGGGCCAGAAAACAAATCACAACCGAAGCCTAAACC 9120

************************************************************

Rdo_MitoGenome AAGAAGAATTGTAGAGACAGGAGCAATTTTCAAAAAAATAAATATTATTAAACCCAAAAA 9180

NC_042820.1 AAGAAGAATTGTAGAGACAGGAGCAATTTTCAAAAAAATAAATATTATTAAACCCAAAAA 9180

************************************************************

Rdo_MitoGenome TAAAAATACTAAATAAAAATCTAAAAAACTAAGAGGGATCATAAAAAATACAAACAAAAT 9240

NC_042820.1 TAAAAATACTAAATAAAAATCTAAAAAACTAAGAGGGATCATAAAAAATACAAACAAAAT 9240

************************************************************

**Start nad4(-)**

**End nad4L(-)**

Rdo_MitoGenome AAATTTTATCATAAAACATTAAAAGTCTGAAAAAAATCATTTCCACATGAACGAATAATT 9300

NC_042820.1 AAATTTTATCATAAAACATTAAAAGTCTGAAAAAAATCATTTCCACATGAACGAATAATT 9300

************************************************************

Rdo_MitoGenome AAAACCAATAAACCTAAACCCAAAGCACCTTCACAGACCGAAAAACATAAAAAAATTATT 9360

NC_042820.1 AAAACCAATAAACCTAAACCCAAAGCACCTTCACAGACCGAAAAACATAAAAAAATTATT 9360

************************************************************

**nad4L (-)**

Rdo_MitoGenome AAAAAAAAACCTTCATAATCAAAAAGAGAGACATAATAAACAAGATTTAAATATAAAGAT 9420

NC_042820.1 AAAAAAAAACCTTCATAATCAAAAAGAGAGACATAATAAACAAGATTTAAATATAAAGAT 9420

************************************************************

Rdo_MitoGenome AAAACAATAAATTCCAAAGATAATAATAAAGAAAGTAAATGTTTACGAAAAAAGCTAAAT 9480

NC_042820.1 AAAACAATAAATTCCAAAGATAATAATAAAGAAAGTAAATGTTTACGAAAAAAGCTAAAT 9480

************************************************************

**Start trnT(+)**

**Start nad4L(-)**

Rdo_MitoGenome ACAGTTACACCACATAAATAAACAAATAATATTTCCATTAGTTTTAATAGTTTAAAAAAA 9540

NC_042820.1 ACAGTTACACCACATAAATAAACAAATAATATTTCCATTAGTTTTAATAGTTTAAAAAAA 9540

************************************************************

**End trnP(-)**

**End trnT(+)**

**trnT (+)**

Rdo_MitoGenome ACATTGGTCTTGTAAACCAAAATTAATAATAATTTTAAAACTTCAGAGAAAAGAAAAACT 9600

NC_042820.1 ACATTGGTCTTGTAAACCAAAATTAATAATAATTTTAAAACTTCAGAGAAAAGAAAAACT 9600

************************************************************

**Start nad6 (+)**

**Start trnP(-)**

**trnP (-)**

Rdo_MitoGenome TTTCATTAATCTCCAAAATTAATATTTTATATAAACTATTCTCTGCATTAAAATCTTAAC 9660

NC_042820.1 TTTCATTAATCTCCAAAATTAATATTTTATATAAACTATTCTCTGCATTAAAATCTTAAC 9660

************************************************************

Rdo_MitoGenome AACAATATTAATTATTTCAAGATCAACATTTATTTTTATAATACATCCAATATCAATAAC 9720

NC_042820.1 AACAATATTAATTATTTCAAGATCAACATTTATTTTTATAATACATCCAATATCAATAAC 9720

************************************************************

Rdo_MitoGenome AATAAATTTAATAATTCAAGCAATTATTATTGCAATCATAACAGGATCAATTCAAATTAA 9780

NC_042820.1 AATAAATTTAATAATTCAAGCAATTATTATTGCAATCATAACAGGATCAATTCAAATTAA 9780

************************************************************

Rdo_MitoGenome TTACTGATATTCTTACATTATATTTATAATCATAATTGGAGGAATAATAGTATTAATTAT 9840

NC_042820.1 TTACTGATATTCTTACATTATATTTATAATCATAATTGGAGGAATAATAGTATTAATTAT 9840

************************************************************

**nad6-0 (+)**

Rdo_MitoGenome ATATATAACAAGAATTGCATCAAATGAAATATTCAAATTAAAAAAGAAAATACTTTTGAT 9900

NC_042820.1 ATATATAACAAGAATTGCATCAAATGAAATATTCAAATTAAAAAAGAAAATACTTTTGAT 9900

************************************************************

Rdo_MitoGenome TCCAATCTTAAGAGCATTAATTTTATCAAACAATAAAATTAAAGAAATAAACCTCGTAGA 9960

NC_042820.1 TCCAATCTTAAGAGCATTAATTTTATCAAACAATAAAATTAAAGAAATAAACCTCGTAGA 9960

************************************************************

Rdo_MitoGenome AATAAGAAATATAGAAAAAAATTTAAACTTCAAATTATCCATAACTAAATTCTTAAATGA 10020

NC_042820.1 AATAAGAAATATAGAAAAAAATTTAAACTTCAAATTATCCATAACTAAATTCTTAAATGA 10020

************************************************************

Rdo_MitoGenome ACCTATAATAAATTTTACATTAACAATTATAATTTACTTATTAATTACATTAATTGTAAT 10080

NC_042820.1 ACCTATAATAAATTTTACATTAACAATTATAATTTACTTATTAATTACATTAATTGTAAT 10080

************************************************************

**End nad6 (+)**

**Start cytB (+)**

Rdo_MitoGenome TGTTAAAATCACCAACTTAAATGAAGGACCATTGCGGCAAAAATTCTAATGAAAAAACAA 10140

NC_042820.1 TGTTAAAATCACCAACTTAAATGAAGGACCATTGCGGCAAAAATTCTAATGAAAAAACAA 10140

************************************************************

Rdo_MitoGenome CTAATAAAAATTTCACCTGTAACTAAAATTATTAATAATGCATTAATTGATTTACCAACC 10200

NC_042820.1 CTAATAAAAATTTCACCTGTAACTAAAATTATTAATAATGCATTAATTGATTTACCAACC 10200

************************************************************

Rdo_MitoGenome CCATCAAACATCAGAATTTGATGAAATATAGGATCAATTCTTGGACTATGCTTAATTATT 10260

NC_042820.1 CCATCAAACATCAGAATTTGATGAAATATAGGATCAATTCTTGGACTATGCTTAATTATT 10260

************************************************************

Rdo_MitoGenome CAAATCATTACCGGAATATTTCTAACAATACACTACACCCCAAATACAGAAATAGCCTTC 10320

NC_042820.1 CAAATCATTACCGGACTATTTCTAACAATACACTACACCCCAAATACAGAAATAGCCTTC 10320

*************** ********************************************

Rdo_MitoGenome AACAGAGTAGTTCACATTTGCCGAGACGTAAATTATGGATGAATAATCCGAACTATTCAT 10380

NC_042820.1 AACAGAGTAGTTCACATTTGCCGAGACGTAAATTATGGATGAATAATCCGAACTATTCAT 10380

************************************************************

Rdo_MitoGenome GCTAATGGAGCATCAATATTCTTTATTTGTATTTATATACACATTGGACGAGGACTATAT 10440

NC_042820.1 GCTAATGGAGCATCAATATTCTTTATTTGTATTTATATACACATTGGACGAGGACTATAT 10440

************************************************************

Rdo_MitoGenome TATGGATCTTACAAATTAATTCACACATGAATAGTAGGAGTAATCATTTTATTCTTAGTT 10500

NC_042820.1 TATGGATCTTACAAATTAATTCACACATGAATAGTAGGAGTAATCATTTTATTCTTAATT 10500

********************************************************* **

Rdo_MitoGenome ATAGCAACAGCATTCCTGGGTTACGTTTTACCATGAGGACAAATATCATTTTGAGGAGCA 10560

NC_042820.1 ATAGCAACAGCATTCCTGGGTTACGTTTTACCATGAGGACAAATATCATTTTGAGGAGCA 10560

************************************************************

Rdo_MitoGenome ACAGTTATCACAAACCTTTTATCAGCTATTCCATATGTAGGAAAAATAATTGTAGAATGA 10620

NC_042820.1 ACAGTTATCACAAACCTTTTATCAGCTATTCCATATGTAGGAAAAATAATTGTAGAATGA 10620

************************************************************

Rdo_MitoGenome CTTTGAGGTGGATTTGCTGTAGATAACGCAACATTAAATCGATTCTTTGCATTCCACTTC 10680

NC_042820.1 CTTTGAGGTGGATTTGCTGTAGATAACGCAACATTAAATCGATTCTTTGCATTCCACTTC 10680

************************************************************

Rdo_MitoGenome CTTCTTCCATTCATTGTAAGTGCAATAGTTATAATTCACTTATTATTCCTTCACCAAACA 10740

NC_042820.1 CTTCTTCCATTCATTGTAAGTGCAATAGTTATAATTCACTTATTATTCCTTCACCAAACA 10740

************************************************************

Rdo_MitoGenome GGATCAAATAACCCTTTAGGAACAAATAGAAATATTGATAAAATCCCATTCCACCCTTAC 10800

NC_042820.1 GGATCAAATAACCCTTTAGGAACAAATAGAAATATTGATAAAATCCCATTCCACCCTTAC 10800

************************************************************

Rdo_MitoGenome TTTTCAACAAAAGATATAATAGGATTTATCGTTACAACCATAATATTAATAACAATTTCA 10860

NC_042820.1 TTTTCAACAAAAGATATTATAGGATTTATCGTTACAACCATAATATTAATAACAATTTCA 10860

***************** ******************************************

**cytB (+)**

Rdo_MitoGenome CTATGAAATCCTTATTTATTAGGAGACCCAGACAATTTTATTCCAGCTAACCCCCTAGTT 10920

NC_042820.1 CTATGAAATCCTTATTTATTAGGAGACCCAGACAATTTTATTCCAGCTAACCCCCTAGTT 10920

************************************************************

Rdo_MitoGenome ACACCAGTTCACATCCAACCTGAATGATATTTTCTATTCGCATATGCAATTCTTCGATCA 10980

NC_042820.1 ACACCAGTTCACATCCAACCTGAATGATATTTTCTATTCGCATATGCAATTCTTCGATCA 10980

************************************************************

Rdo_MitoGenome ATCCCTAATAAATTAGGAGGAGTAATTGCACTAATTATATCAATCGCAATTTTATTTATT 11040

NC_042820.1 ATCCCTAATAAATTAGGAGGAGTAATTGCACTAATTATATCAATCGCAATTTTATTTATT 11040

************************************************************

Rdo_MitoGenome ATTCCTTTAACTAACAAGAGAAAATTCCAAAGAATAAATTTTTACCCAATAAATAAATTC 11100

NC_042820.1 ATTCCTTTAACTAACAAGAGAAAATTCCAAAGAATAAATTTTTACCCAGTAAATAAATTC 11100

************************************************ ***********

Rdo_MitoGenome TTATTTTGATCAATAGTAAGAACAGTAATTTTATTAACATGAATTGGAGCACGACCTGTT 11160

NC_042820.1 TTATTTTGATCAATAGTAAGAACAGTAATTTTATTAACATGAATTGGAGCACGACCTGTT 11160

************************************************************

Rdo_MitoGenome CAGGACCCTTACATTACAACGGGACAAGTATTAACAATTATTTACTTTTTATATTATATA 11220

NC_042820.1 CAGGACCCTTACATTACAACGGGACAAGTATTAACAATTATTTACTTTTTATATTATATA 11220

************************************************************

**Start trnS2 (+)**

**End cytB (+)**

Rdo_MitoGenome ATTACACCACTAACACTCAAAATTTGAGATAAAATCTTAATCAAATAGCTAATGAACTTG 11280

NC_042820.1 ATTACACCACTAACACTCAAAATTTGAGATAAAATCTTAATCAAATAGCTAATGAACTTG 11280

************************************************************

**End trnS2 (+)**

**trnS2 (+)**

Rdo_MitoGenome AAAAGTATATATTTTGAAAATATAAAATAGAAGTAAAATCCTCTATTAGCTTATACTAAA 11340

NC_042820.1 AAAAGTATATATTTTGAAAATATAAAATAGAAGTAAAATCCTCTATTAGCTTATACTAAA 11340

************************************************************

**End nad1 (-)**

Rdo_MitoGenome TTTTATTAACTAAATAATAAAAACCCAACAAAGAGTCTTTAATCCTAAATAAAACAACAA 11400

NC_042820.1 TTTTATTAACTAAATAATAAAAACCCAACAAAGAGTCTTTAATCCTAAATAAAACAACAA 11400

************************************************************

Rdo_MitoGenome AAAACATAAAGATACAGGTAAATAAATTTTTCAAGCTAAATATATCAATTTATCATAACG 11460

NC_042820.1 AAAACATAAAGATACAGGTAAATAAATTTTTCAAGCTAAATATATCAATTTATCATAACG 11460

************************************************************

Rdo_MitoGenome ATAACGGGGAAGAGTACCTCGAACCCAAACCCATAAAAAGGAAAAAAAAACAATTTTAAT 11520

NC_042820.1 ATAACGGGGAAGAGTACCTCGAACCCAAACCCATAAAAAGGAAAAAAAAACAATTTTAAT 11520

************************************************************

Rdo_MitoGenome AAAAAAAAATAAATTTATATAATCACCACCAAAAAATATTAAAGAACAAATTATTCTTAT 11580

NC_042820.1 AAAAAAAATAAATTTATATAAATCACCACCAAAAAATATTAAAGAACAAATTATTCTTAT 11580

******** ** ** ****************************************

Rdo_MitoGenome AAATAAAATACTAGAATATTCAGCAAGAAAAATTAAAGCAAATCCACCTCTTCTATACTC 11640

NC_042820.1 AAATAAAATACTAGAATATTCAGCAAGAAAAATTAAAGCAAATCCACCTCTTCTATACTC 11640

************************************************************

Rdo_MitoGenome AACATTAAAACCAGAGACCAATTCGGATTCACCCTCAGCAAAATCAAAAGGAGTACGATT 11700

NC_042820.1 AACATTAAAACCAGAGACCAATTCGGATTCACCCTCAGCAAAATCAAAAGGAGTACGATT 11700

************************************************************

Rdo_MitoGenome AGTTTCTGCTAATATAGAAACAAATAAAATTAAACTTAAAGGAAACATCAAAAAGACAAA 11760

NC_042820.1 AGTTTCTGCTAATATAGAAACAAATAAAATTAAACTTAAAGGAAACATCAAAAAGACAAA 11760

************************************************************

Rdo_MitoGenome TCACAAATATTTCTGATACTTAAAAAAATCATAAAAACATAAGCTAGAAGTTATAACTAA 11820

NC_042820.1 TCACAAATATTTCTGATACTTAAAAAAATCATAAAAACATAAGCTAGAAGTTATAACTAA 11820

************************************************************

**nad1 (-)**

Rdo_MitoGenome AAAAGAAATAATAATAATAACCAAACTAACCTCATAGGAAATAGTTTGAGCCATAGATCG 11880

NC_042820.1 AAAAGAAATAATAATAATAACCAAACTAACCTCATAGGAAATAGTTTGAGCCATAGATCG 11880

************************************************************

Rdo_MitoGenome CAAAGAACCAATTATTGAATAATTAGAATTAGATGACCAACCAGAAATTATTACAGCATA 11940

NC_042820.1 CAAAGAACCAATTATTGAATAATTAGAATTAGATGACCAACCAGAAATTATTACAGCATA 11940

************************************************************

Rdo_MitoGenome AACTGAAAGACTTGAAAAACATAAAAAAAATAATATACCTAAATTAAAAGATAAAAAATA 12000

NC_042820.1 AACTGAAAGACTTGAAAAACATAAAAAAAATAATATACCTAAATTAAAAGATAAAAAATA 12000

************************************************************

Rdo_MitoGenome AGAAAAAAATGGAAATCTAAGTCACATTATTAAAGAAATAAAAAAATTTATTACAGGAGA 12060

NC_042820.1 AGAAAAAAATGGAAATCTAAGTCACATTATTAAAGAAATAAAAAAATTTATTACAGGAGA 12060

************************************************************

Rdo_MitoGenome AAAATAATAAAAAACATAGTTAGAAACTAAAGGTAAACACTGCTCCTTAGAAAATAATTT 12120

NC_042820.1 AAAATAATAAAAAACATAGTTAGAAACTAAAGGTAAACACTGCTCCTTAGAAAATAATTT 12120

************************************************************

Rdo_MitoGenome AATAGCATCTCTAAAAGGTTGTAATAATCCTATAAAACCAACCTTATTAGGACCTTTTCG 12180

NC_042820.1 AATAGCATCTCTAAAAGGTTGTAATAATCCTATAAAACCAACCTTATTAGGACCTTTTCG 12180

************************************************************

Rdo_MitoGenome TAATTGAATATAACCAAGAACCTTACGCTCCATCAAAGTTAAAAATGCAACACCAACTAA 12240

NC_042820.1 TAATTGAATATAACCAAGAACCTTACGCTCCATCAAAGTTAAAAATGCAACACCAACTAA 12240

************************************************************

Rdo_MitoGenome AACCCCAATAATCAAGACTAATAAAGAAAAAAAAACAAGAATAAAATCAAAAATAAACAA 12300

NC_042820.1 AACCCCAATAATCAAGACTAACAAAGAAAAAAAAACAAGAATAAAATCAAAAATAAACAA 12300

********************* **************************************

**End trnL1 (-)**

**Start nad1 (-)**

**trnL1 (-)**

Rdo_MitoGenome TATTATTTGTATAAAATACATATAAAGATCCTAAATCTACCGCACTAATCTGCCAAAATA 12360

NC_042820.1 TATTATTTGTATAAAATACATATAAAGATCCTAAATCTACCGCACTAATCTGCCAAAATA 12360

************************************************************

**Start trnL1 (-)**

**End rrnL (-)**

Rdo_MitoGenome ATAAGAATACCCAAAAAATAAATAATATATTGAAATACTGGTCCTTTCGTACTAAGTAAA 12420

NC_042820.1 ATAAGAATACCCAAAAAATAAATAATATATTGAAATACTGGTCCTTTCGTACTAAGTAAA 12420

************************************************************

Rdo_MitoGenome TCAAAAAAAATTGAAGATAGAAACCAACCTGGCTCAAACCGGTTTTAACTCAGATCATGT 12480

NC_042820.1 TC-AAAAAAATTGAAGATAGAAACCAACCTGGCTCAAACCGGTTTTAACTCAGATCATGT 12479

** *********************************************************

Rdo_MitoGenome AAAGTTTTAAAGGTCGAACAGACCTAACATTCAAGCTTTTGCACCAGAAGTTTACTTTAA 12540

NC_042820.1 AAAGTTTTAAAGGTCGAACAGACCTAACATTCAAGCTTTTGCACCAGAAGTTTACTTTAA 12539

************************************************************

Rdo_MitoGenome TCCAACATCGAGGTCGCAACCCCTTCCATCAATAAGAACTCTAAAGAAAGATTACGCTGT 12600

NC_042820.1 TCCAACATCGAGGTCGCAACCCCTTCCATCAATAAGAACTCTAAAGAAAGATTACGCTGT 12599

************************************************************

Rdo_MitoGenome TATCCCTAAGGTAATTTTAACTTATAATCAAAAAAAATTGAATCAATAAAATATAAATAA 12660

NC_042820.1 TATCCCTAAGGTAATTTTAACTTATAATCAAAAAAAATTGAATCAATAAAATATAAATAA 12659

************************************************************

**rrnL (-)**

Rdo_MitoGenome ATATAAAAATAAAAGAAAAGTTTGTCAAATTATTCCCATCACCCCAACAAAATAATTTTT 12720

NC_042820.1 ATATAAAAATAAAAGAAAAGTTTGTCAAATTATTCCCATCACCCCAACAAAATAATTTTT 12719

************************************************************

Rdo_MitoGenome AATTAATAAAAGAAAAATCCTAAATAAATTATTAACCAAAAAATATTAAACTCTATAGGG 12780

NC_042820.1 AATTAATAAAAGAAAAATCCTAAATAAATTATTAACCAAAAAATATTAAACTCTATAGGG 12779

************************************************************

Rdo_MitoGenome TCTTCTCGTCTTTCAAAAAAATTTGAACTTTTTAATTCAAAAATAAAATTCAAAAAATAA 12840

NC_042820.1 TCTTCTCGTCTTTCAAAAAAATTTGAACTTTTTAATTCAAAAATAAAATTCAAAAAATAA 12839

************************************************************

Rdo_MitoGenome AGAATGAGACAAAACTCCCCTTGTCAAACCATTCATTCCAGCTTTCAATTAAAAAACTAA 12900

NC_042820.1 AGAATGAGACAAAACTCCCCTTGTCAAACCATTCATTCCAGCTTTCAATTAAAAAACTAA 12899

************************************************************

Rdo_MitoGenome TGATTATGCTACCTTTGCACGGTTAAAATACCGCGGCCATTTAATTAATCATTGGGCAGG 12960

NC_042820.1 TGATTATGCTACCTTTGCACGGTTAAAATACCGCGGCCATTTAATTAATCATTGGGCAGG 12959

************************************************************

Rdo_MitoGenome CCAGACTTTAAAATATAATCAAAAAGACATGTTTTTAATAAACAGGCAAGAGAGAAAGTC 13020

NC_042820.1 CCAGACTTTAAAATATAATCAAAAAGACATGTTTTTAATAAACAGGCAAGAGAGAAAGTC 13019

************************************************************

Rdo_MitoGenome GAGTTCCTTAAACTCTTCTGAAAATAAACAAAAAATACAAAAACCATTTACTAATTTAAT 13080

NC_042820.1 GAGTTCCTTAAACTCTTCTGAAAATAAACAAAAAATACAAAAACCATTTACTAATTTAAT 13079

************************************************************

Rdo_MitoGenome CATAATAAAATAAAAACATTATTAAATTAAAATTTTAAATAAAAAACTTAAAATAAACAA 13140

NC_042820.1 CATAATAAAATAAAAACATTATTAAATTAAAATTTTAAATAAAAAACTTAAAATAAACAA 13139

************************************************************

Rdo_MitoGenome AATATTATTAAAAAATAAGTAATTAAAACATAATAAAAATGAAAAATTCTAATCCTATTA 13200

NC_042820.1 AATATTATTAAAAAATAAGTAATTAAAACATAATAAAAATGAAAAATTCTAATCCTATTA 13199

************************************************************

Rdo_MitoGenome AATATTTTATATTAAAAAATTATAAATATATATTAAAAAGCTTATCCCCTTTAATATTTA 13260

NC_042820.1 AATATTTTATATTAAAAAATTATAAATATATATTAAAAAGCTTATCCCCTTTAATATTTA 13259

************************************************************

Rdo_MitoGenome AGCTCATAAGAAAAAAATTATCAATAAAAAATCTTTCCTAAAGAACATAAAATTTAATTT 13320

NC_042820.1 AGCTCATAAGAAAAAAATTATCAATAAAAAATCTTTCCTAAAGAACATAAAATTTAATTT 13319

************************************************************

Rdo_MitoGenome TTTTATTTAAAAACCAGATATATCAAAAGACGAATTACATTTCAAATCAAAATTAATATT 13380

NC_042820.1 TTTTATTTAAAAACCAGATATATCAAAAGACGAATTACATTTCAAATCAAAATTAATATT 13379

************************************************************

Rdo_MitoGenome ATAAATTATTATTCAACATAAAAATTTATAAAAAATTAACTCCTTTTGATTCGGGATTAA 13440

NC_042820.1 ATAAATTATTATTCAACATAAAAATTTATAAAAAATTAACTCCTTTTGATTCGGGATTAA 13439

************************************************************

Rdo_MitoGenome TATATACATATAAAAAAATTAATCAACCCTGATACACAAGGTACAACAAATAAAATTTTC 13500

NC_042820.1 TATATACATATAAAAAAATTAATCAACCCTGATACACAAGGTACAACAAATAAAATTTTC 13499

************************************************************

Rdo_MitoGenome TTTGTCAATAATATATAATAAACCTTCACAGTACAAAAAAATAATTTTTTAAAATAAAAA 13560

NC_042820.1 TTTGTCAATAATATATAATAAACCTTCACAGTACAAAAAAATAATTTTTTAAAATAAAAA 13559

************************************************************

**Start rrnL (-)**

**End trnV (-)**

Rdo_MitoGenome CTAAAAAAAATAATACTTTAAAAAAATAAATTTAAATAAAAAAAATAAAATATATTATCA 13620

NC_042820.1 CTAAAAAAAATAATACTTTAAAAAAATAAATTTAAATAAAAAAAATAAAATATATTATCA 13619

************************************************************

**Start trnV (-)**

**End rrnS (-)**

**trnV (-)**

Rdo_MitoGenome AACTAAGTTAAAAACTATCCTTTTAGTGTAAATAAAATGCTTATTCAAGCTCCAGTTTGA 13680

NC_042820.1 AACTAAGTTAAAAACTATCCTTTTAGTGTAAATAAAATGCTTATTCAAGCTCCAGTTTGA 13679

************************************************************

Rdo_MitoGenome TATATCCAGACACACTTTCCAGTACGCCTACTATGTTACGACTTATCTCAAATTAATTGA 13740

NC_042820.1 TATATCCAGACACACTTTCCAGTACGCCTACTATGTTACGACTTATCTCAAATTAATTGA 13739

************************************************************

Rdo_MitoGenome GAGTGACGGGCGATATGTACATATTTTAGAGCTTAATCATAAAATCTAATAAAAATTATT 13800

NC_042820.1 GAGTGACGGGCGATATGTACATATTTTAGAGCTTAATCATAAAATCTAATAAAAATTATT 13799

************************************************************

Rdo_MitoGenome ACTTTCAAATCCACTTTATTAAAAATATCTAAAAAATTAAACCATATAAATAAATTTATT 13860

NC_042820.1 ACTTTCAAATCCACTTTATTAAAAATATCTAAAAAATTAAACCATATAAATAAATTTATT 13859

************************************************************

Rdo_MitoGenome GTAACCCACCTCAACATGAATATAAACTGCACCTTGATCTGATGTTAACTTTAATATAAG 13920

NC_042820.1 GTAACCCACCTCAACATGAATATAAACTGCACCTTGATCTGATGTTAACTTTAATATAAG 13919

************************************************************

Rdo_MitoGenome AAAATGATCATTTAATCCTTAAAAAATAACCAACTACGACGATATACAAATTTTTAAATC 13980

NC_042820.1 AAAATGATCATTTAATCCTTAAAAAATAACCAACTACGACGATATACAAATTTTTAAATC 13979

************************************************************

Rdo_MitoGenome AAGTAACTAAATCGTGGATTATCGTTTACAGGACAGGTTCCTCTGAAAAGACTAAAATAC 14040

NC_042820.1 AAGTAACTAAATCGTGGATTATCGTTTACAGGACAGGTTCCTCTGAAAAGACTAAAATAC 14039

************************************************************

**rrnS (-)**

Rdo_MitoGenome CGCCAAATCCTTTTACTTTCAAGAACTTAACTAATACTAATAAAAAATTTACACTCAGAA 14100

NC_042820.1 CGCCAAATCCTTTTACTTTCAAGAACTTAACTAATACTAATAAAAAATTTACACTCAGAA 14099

************************************************************

Rdo_MitoGenome TAACGGGGTATCTAATCCCGATTTCAAAAACAGAATTTAATAAAACAGAAAATAAAAAAT 14160

NC_042820.1 TAACGGGGTATCTAATCCCGATTTCAAAAACAGAATTTAATAAAACAGAAAATAAAAAAT 14159

************************************************************

Rdo_MitoGenome AAATTTAATAAAATTTCACCTAATATTAAAAATTTAATCTTAATTTTAATCCTAATTACC 14220

NC_042820.1 AAATTTAATAAAATTTCACCTAATATTAAAAATTTAATCTTAATTTTAATCCTAATTACC 14219

************************************************************

Rdo_MitoGenome ATTAAATAAATTTATATTGCACTAATTGTATAACCGCAACTGCTGGCACAAAATTAGTTA 14280

NC_042820.1 ATTAAATAAATTTATATTGCACTAATTGTATAACCGCAACTGCTGGCACAAAATTAGTTA 14279

************************************************************

Rdo_MitoGenome GAACTAAAAAAATTTCTGATTCTAAGTAACCTTAATAATCAAATATAAATACTGCAAAAA 14340

NC_042820.1 GAACTAAAAAAATTTCTGATTCTAAGTAACCTTAATAATCAAATATAAATACTGCAAAAA 14339

************************************************************

Rdo_MitoGenome GAAAACTCTAAAATAACACTTACATATATTTAAATTTATTATATAAATAAAAAGCCAAAA 14400

NC_042820.1 GAAAACTCTAAAATAACACTTACATATATTTAAATTTATTATATAAATAAAAAGCCAAAA 14399

************************************************************

**Start rrnS (-)**

Rdo_MitoGenome TAAAACTTTCCAACATAAAAAATAAATTTTAACACTTTAAAAATTTTTTTCCACAAAATA 14460

NC_042820.1 TAAAACTTTCCAACATAAAAAATAAATTTTAACACTTTAAAAATTTTTTTCCACAAAATA 14459

************************************************************

Rdo_MitoGenome AGTAAAATTGACCAACCTAACCAAAGCACCGAAAAAGAACAGCTGGAACAGGAAACCACG 14520

NC_042820.1 AGTAAAATTGACCAACCTAACCAAAGCACCGAAAAAGAACAGCCGGAACAGGAAACCACG 14519

******************************************* ****************

Rdo_MitoGenome ACGAAGAAGCGAAAACGAGGCAACGCCGCCCTCGGGGTAAGAACCAGGTTTAATCCACGG 14580

NC_042820.1 ACGAAGAAGCGAAAACGAGGAAACGCCGCCCTCGGGGTACGAACCAGGTTTAACCCACGG 14579

******************** ****************** ************* ******

Rdo_MitoGenome GATAAGCAACGCCCTATTGCCAACCCATGAATACGACCAAAGCACCGAAAAAGAACAGCT 14640

NC_042820.1 GATAAGCAACGCCCTATTGCCAACCCATGAATACGACCAAAGCACCGAAAAAGAACAGCT 14639

************************************************************

Rdo_MitoGenome TGAACAGGAAACCACGACGAAGAAGCGAAAACGAGGAAACGCCGCCCTCGGGGTAAGAAC 14700

NC_042820.1 TGAACAGGAAACCACGACGAAGAAGCGAAAACGAGGAAACGCCGCCCTCGGGGTACGAAC 14699

******************************************************* ****

Rdo_MitoGenome CAGGTTTAACCCAC---------------------------------------------- 14714

NC_042820.1 CAGGTTTAACCCACGGGATAAGCAACGCCCTATTGCCAACCCATGAATACGACCAAAGCA 14759

**************

Rdo_MitoGenome ------------------------------------------------------------ 14714

NC_042820.1 CCGAAAAAGAACAGCTTGAACAGGAAACCACGACGAAGAAGCGGAAACGAGGAAACGCCA 14819

Rdo_MitoGenome ------------------------------AGGATCAACAACGTCCTATTCCCAACAAAA 14744

NC_042820.1 CCCTCGGGGTAAGAACCAGGTTTAACCCATAGGATCAACAACGTCCTATCGCCAACAAAA 14879

******************* *********

Rdo_MitoGenome TCAATACATTAATTATAATTTTCTTTTTTAACTTAAAAATTTAAACACTAGTAAATAAAT 14804

NC_042820.1 TCAATACATTAATTATAATTTTCTTTTTTAACTTAAAAATTTAAACACTAGTAAATAAAT 14939

************************************************************

Rdo_MitoGenome ATTTAATAAATTATGAACAAAACCTAAGTCCTTAAATTTTAAAACTAAATTTTAATTTAG 14864

NC_042820.1 ATTTAATAAATTATGAACAAGACCTAAGTCCTTAAATTTTAAAACTAAATTTCAATTTAG 14999

******************** ******************************* *******

Rdo_MitoGenome ATTAAAATTTATTAATAAAATTATTAACATTTTATTAATAAATAATTTTATTAATAATAA 14924

NC_042820.1 CTTAAAATTTATTAATAAAATTATTAACATTTTATTAATAAATAATTTTATTAATAATAA 15059

***********************************************************

Rdo_MitoGenome ATAAGGTTTTTTTTTTTTTTTTTTTTTHTATAATTTTTATATTCAAATAAATAATTATAA 14984

NC_042820.1 ATAAGGTTTTTTTTTTTTTTTTTTTTTCTATAATTTTTATATTCAAATAAATAATTATAA 15119

*************************** ********************************

Rdo_MitoGenome TAAAATAATTTTAATTTAAAATTATAAAAAATTAATTTTTATTATAAAATTTTAATTATA 15044

NC_042820.1 TAAAATAATTTTAATTTAAAATTATAAAAAATTAATTTTTATTATAAAATTTTAATTATA 15179

************************************************************

Rdo_MitoGenome TAAGAGAAAAAATTATATAAAAATATTTATTCATTAATTATATATTTATATATAAATAAT 15104

NC_042820.1 TAAGGGAAAAAATTATATAAAAATATTTATTCATTAATTATATATTTATATATAAATAAT 15239

**** *******************************************************

Rdo_MitoGenome TTATGTATAAATAAAATTAATATAATTTTATTGTAAATATATAATATATTCAAATATGTT 15164

NC_042820.1 TTATGTATAAATAAAATTAATATAATTTTATTGTAAATATATAATATATTTAAATATGTT 15299

************************************************** *********

Rdo_MitoGenome ATTATATATTAATAAATAATTATATATTATATATTTATATATAATTTATATATAAATATA 15224

NC_042820.1 ATTATATATTAATAAATAATTATATATTATATATTTATATATAATTTATATATAAATATA 15359

************************************************************

Rdo_MitoGenome TATTTACATATTATATAAGTTCTTATTATTATCCCAATACCTATTTCTAATAGGTTATTA 15284

NC_042820.1 TATTTACATATTATATAAGTTCTTATTATTATCCCAATACCTATTTCTAATAGGTTATTA 15419

************************************************************

Rdo_MitoGenome TATATTAATATATAATTATATATATATAAATCAAGATATATATTAATAAATATAAATTAA 15344

NC_042820.1 TATATTGATATATAATTATATATATATAAATCAAGATATATATTAATAAATATAAATTAA 15479

****** *****************************************************

Rdo_MitoGenome CTTTAAATTTAATATTATATATAAATATGTAAATATATATAAATATATAATTATATATGA 15404

NC_042820.1 CTTTAAATTTAATATTATATATAAATATGTAAATATATATAAATATATAATTATATATGA 15539

************************************************************

Rdo_MitoGenome ATAACTTATTGTTATATATAAAATTATTTATTAATTTAATAATTATATATAAATATATAG 15464

NC_042820.1 ATAACTTATTGTTATATATAAAATTATTTATTAATTTAATAATTATATATAAATATATAG 15599

************************************************************

Rdo_MitoGenome TTATATATATATATAAATATATATTTTATATAGTAAAAAAAAAGTTAGCGCAAAAACACG 15524

NC_042820.1 TTATATATATATATAAATATATATTTTATATAGTAAAAAAAAAGTTAGCGCAAAAACACG 15659

************************************************************

Rdo_MitoGenome TTTTGAATGAAAATCCATAAATACATGGTTTTGCACAACCAATAATTTATTAACTAAACC 15584

NC_042820.1 TTTTGAATGAAAATCCATAAATACATGGTTTTGCACAACCAATAATTTATTAACTAAACC 15719

************************************************************

Rdo_MitoGenome AAAACCCATAAAAAGAAAAAAAATCGCCTACGTTTTAAAACAAAAAATGGTGAGGCACTA 15644

NC_042820.1 AAAGCCCATAAAAAGAACAAAAATCGCCTACGTTTTAAAACAAAAAATGGTGAGGCATTA 15779

*** ************* *************************************** **

Rdo_MitoGenome CTATGAACATAAACATTAATAAAAAAATATAAAAAAATTTAAAAGAACAGATACAAATTT 15704

NC_042820.1 CTATGAACATAAACATTAATAAAAAAATATAAAAAAATTTAAAAGAACAGATACAAATTT 15839

************************************************************

Rdo_MitoGenome TTGTTTACAAGTAAAGTTTT 15724

NC_042820.1 TTGTTTACAAGTAAAGTTTT 15859

********************
